# A Deluge of Complex Repeats: The Solanum Genome

**DOI:** 10.1371/journal.pone.0133962

**Published:** 2015-08-04

**Authors:** Mrigaya Mehra, Indu Gangwar, Ravi Shankar

**Affiliations:** 1 Studio of Computational Biology & Bioinformatics, Biotechnology Division, CSIR-Institute of Himalayan Bioresource Technology (CSIR-IHBT), Palampur, 176061, HP, India; 2 Academy of Scientific & Innovative Research, Chennai, India; Leibniz-Institute of Plant Genetics and Crop Plant Research (IPK), GERMANY

## Abstract

Repetitive elements have lately emerged as key components of genome, performing varieties of roles. It has now become necessary to have an account of repeats for every genome to understand its dynamics and state. Recently, genomes of two major *Solanaceae* species, *Solanum tuberosum* and *Solanum lycopersicum*, were sequenced. These species are important crops having high commercial significance as well as value as model species. However, there is a reasonable gap in information about repetitive elements and their possible roles in genome regulation for these species. The present study was aimed at detailed identification and characterization of complex repetitive elements in these genomes, along with study of their possible functional associations as well as to assess possible transcriptionally active repetitive elements. In this study, it was found that ~50–60% of genomes of *S*. *tuberosum* and *S*. *lycopersicum* were composed of repetitive elements. It was also found that complex repetitive elements were associated with >95% of genes in both species. These two genomes are mostly composed of LTR retrotransposons. Two novel repeat families very similar to LTR/ERV1 and LINE/RTE-BovB have been reported for the first time. Active existence of complex repeats was estimated by measuring their transcriptional abundance using Next Generation Sequencing read data and Microarray platforms. A reasonable amount of regulatory components like transcription factor binding sites and miRNAs appear to be under the influence of these complex repetitive elements in these species, while several genes appeared to possess exonized repeats.

## Introduction

Very recently two important *Solanaceae* species, *S*. *tuberosum* and *S*. *lycopersicum*, genomes have been sequenced, reporting 810.6 Mb and 781.6 Mb of genome size for *S*. *tuberosum* and *S*. *lycopersicum*, respectively [1,2]. These genomes have been annotated for various genomic elements including repetitive elements, giving a total of 404,861 repetitive elements for *S*. *tuberosum* and 719,453 repetitive elements for *S*. *lycopersicum*. Initial studies with repetitive elements focused upon understanding their structure and functional aspects [3,4]. In one of the first attempts to study repeat content of *Solanaceae* genomes, Ganal *et al*. [5] had identified four different repeat families accounting for 0.15% of the genome of *S*. *lycopersicum*. Osborne *et al*. [4] studied the transposition behavior of *Ac* elements in *S*. *lycopersicum* by employing cloning and IPCR method to evaluate the transposition site preferences of these elements. Later, the importance of these elements in transposon tagging was discussed by Belzile & Yoder [6], who also studied the transposition behavior of maize *Ac* elements introduced in recombinant lines. Similar kind of study was performed by Stadler and colleagues [7] where they demonstrated the utility of repetitive elements in somatic hybridization technique and also identified novel repetitive elements having variable species specificity in different *Solanum* genomes. These studies were shown to be important for selection of different agronomically important traits in hybrid genomes.

A novel *in-silico* approach to identify the repetitive elements in different *Solanaceae* genomes was attempted by Oosumi *et al*. [8] where the authors searched the GenBank nucleic acid database for the presence of inverted repeats. The authors made observations that several genes possessed repetitive elements either in their 5' or 3' flanking regions or in introns, proposing functional aspects of repetitive elements in plants and their associations with gene-coding regions of the genome. Later studies on *Solanaceae* were focused on different satellite repetitive elements which could be developed as markers. Many genetic markers were developed for distinguishing different cultivars of *S*. *tuberosum*, *S*. *lycopersicum* and other *Solanaceae* species [9–12]. Tandem repeated DNA sequences were studied comprehensively and many new tandem repetitive elements were discovered in *Solanaceae* [13–18]. A novel method for estimation of repeat-content of a genome was proposed by Zhu *et al*. [19] where they estimated the genomic repeat-content by studying ~10% of a genome sequence. The authors studied 89.9 Mb of the genomic sequence of *S*. *tuberosum* in the form of BAC sequences and identified that repetitive content of the *S*. *tuberosum* BAC sequences was ~34% while for *S*. *lycopersicum* BAC sequences repetitive content was ~46% on the basis of homology. In both species, majority of the identified repetitive elements were not characterized and the authors observed prevalence of LTR retrotransposons, specifically LTR/Gypsy [19]. Thus, the authors provided a way to estimate the repetitive content of a genome even before the availability of the complete genome sequence. Studying the distribution of repetitive elements provides a view of the genomic localization as well as the abundance of different elements in a genome. Such a study was undertaken in the *S*. *lycopersicum* genome, FISH and fibre-FISH technique were employed to study the distribution of microsatellites as well as complex repeats [20]. Functional influence of a repetitive element on protein-coding genes was studied in *S*. *lycopersicum*, where the fruit shape gene was observed to be under the influence of a retrotransposon named *Rider* [21]. Kuang et al. [22] identified 22 MITE families in *Solanaceae* out of which fifteen were reported as novel repeat families. The authors also studied the functional roles of these MITE families and identified different exonized genes in their study [22] as well as active families and associated them with the biogenesis of different siRNA sequences. Active MITE elements were also reported in *S*. *tuberosum* genome, where the active nature of these elements produced phenotypic changes in *S*. *tuberosum* plants [23]. tRNA derived SINE elements were identified in different plant families including *Solanaceae* by Wenke *et al*. [24] and the chromosomal distribution of SINE elements was later assessed by using FISH. The authors also developed a tool to identify these elements *in-silico* and reported many novel SINE elements [24]. Another *in-silico* analysis was performed for the identification and study of MULE elements in different plant genomes including *S*. *tuberosum* and *S*. *lycopersicum* [25]. Identification of different LTR elements was also performed by Yadav and Singh [26] by using the tool LTR Finder on the EST sequences which were further validated by matching their prediction with the RepeatMasker output. Another most widely studied type of repetitive elements were the elements residing in the telomeric and centromeric regions. Centromeres are important structural components of a chromosome which aid the correct segregation of chromosomes during cell division. These repeats and the evolution of centromeres has been studied a lot. It was reported that centromeres are composed of specific histone proteins and long arrays of satellite repeats and retrotransposons [27]. These repeats were proposed to evolve rapidly and show divergence even in closely related species, thus the utility of these elements to identify different molecular characteristics of transgenic plants was studied [28–30]. Tang *et al*. [31] also studied the repeat-content of *S*. *tuberosum* using FISH and identified three repeat families. Thus as observed, most of the studies performed on the repetitive elements of *Solanaceae* genomes focused on derivation of agronomically important markers while limited studies were performed on the complex repetitive fraction of plant genomes.

From commercial point of view, many major crop species like potato (*Solanum tuberosum*), tomato (*Solanum lycopersicum*), pepper (*Capsicum annum*) and other *Capsicum* species, many ornamental plants, and biological model systems like *Nicotiana* spp and *Datura* spp belong to this family. *S*. *tuberosum* and *S*. *lycopersicum*, are two closely related species of *Solanaceae* family [32,33] which diverged very recently (~8 Mya) [34]. To reveal the genomes and provide a single stop molecular information about different *Solanaceae* species Sol Genomics Network (SGN) was established as a clade-oriented database [35,36]. Initially, SGN was developed to store the data like EST sequences and data of genetic mapping. The main focus of SGN was the identification of protein coding genes important for the development of different plant species and to understand the genetic basis of plant diversity [37]. SGN provides free access to all the information about the different *Solanaceae* families from a single web portal and has emerged as an important comprehensive resource for *Solanaceae* and other closely related families. It houses information about genomic (BAC sequences and genome sequences), transcriptomic (EST, unigene sequences, high throughput sequencing data and microarray data), proteomic, genetic and phenotypic (physical maps and markers), taxonomic and functional annotation of the different *Solanaceae* genomes [36,37]. SGN has been sequencing several species of *Solanaceae* genomes simultaneously. Currently in SGN, the complete as well as draft genome sequences are available for fourteen *Solanaceae* species. SGN initiated the tomato genome sequencing in 2004 using BAC-by-BAC sequencing methodology and later added the whole genome shotgun sequencing approach to the sequencing methodology [36], while the complete genome sequence of *S*. *lycopersicum*, was reported in 2012 [2]. The genome sequence of the *S*. *tuberosum* was released in 2011 [1] which is still being updated [38]. Although, SGN provided annotations and information for different repetitive elements of *S*. *tuberosum* and *S*. *lycopersicum*, there appears an enormous scope to carry out dedicated study with respect to the detailing of the repetitive elements in these species mainly but due to limited characterization of *de-novo* and species-specific repetitive elements where homology based methods have been applied predominantly. Also as mentioned above, most of the initial studies on *Solanaceae* repetitive elements were focused on tandem repetitive elements like satellite repeats while studies of complex repetitive element were mostly performed on isolated groups of repetitive elements. The functional impacts of these elements on the genome dynamics in *Solanaceae* genomes were seldom studied in detail. This all has been the motivation to carry out the present study on a genome-wide scale and identify the potential impacts of these elements on the genome dynamics of these two species in detailed comparative manner. In this study, an attempt has been made to identify and characterize the known as well as novel complex repetitive elements in the two most commercially valuable *Solanaceae* species of *S*. *tuberosum* and *S*. *lycopersicum* whose genomes have been reported recently [1,2]. The genomes of these two species appear to hold more complex repetitive elements than previously appreciated. Several of these repetitive elements appeared transcriptionally active while several were found associated with potential to carry out some regulatory impact. It was discovered that the repetitive elements had a huge influence over the host protein coding genes as >95% of the genes in the two *Solanaceae* genomes overlapped with repetitive elements, suggesting a major role being played by repetitive elements in gene formation and transcriptionally active elements. The impacts of repetitive elements with respect to regulatory elements was also studied. The present study is mainly focused around the complex repeats and has excluded analysis over simple and tandem repeats.

## Materials and Methods

### Sequence information

The genome sequences, co-ordinates of various genomic elements, protein and transcript sequences of *S*. *tuberosum* (*S*. *tuberosum* group phureja doubled monoploid clone) and *S*. *lycopersicum* (*S*. *lycopersicum* cv. Heinz 1706) were downloaded from Ensembl plants (http://plants.ensembl.org/index.html). Co-ordinates of introns and upstream regions with respect to gene start sites were extracted using an in-house PERL script. Syntenic regions between *S*. *tuberosum* and *S*. *lycopersicum* genomes were identified using Symap (version 42) [39]. The gene co-ordinates along-with sequences were submitted as an input in the form of GTF file to Symap for both species. BedTools [40] was used to merge the overlapping genomic co-ordinates of various genomic elements. Pre-miRNA sequences for *S*. *tuberosum* and *S*. *lycopersicum* were downloaded from miRBase (version 20) [41]. The non-coding RNA sequences were downloaded from Rfam database version 11 [42]. Orthologous genes in *S*. *tuberosum* and *S*. *lycopersicum* were identified by matching the respective protein sequences using BLASTP [43].

### Repetitive element identification

RepeatModeler (http://www.repeatmasker.org/RepeatModeler.html) is a *de-novo* repeat identification tool, which also provides annotation to identified sequences utilizing three different repeat identification algorithms namely RECON [44], RepeatScout [45] and TRF [46]. For identification of known (based on homology) as well as novel repetitive sequences, a database of the genome sequences of *S*. *tuberosum* and *S*. *lycopersicum* was generated using BuildDatabase command, on which RepeatModeler was executed. RepeatModeler (http://www.repeatmasker.org/RepeatModeler.html) generated the consensus sequences of identified repeat families, which was used by RepeatMasker (http://www.repeatmasker.org/RMDownload.html) to annotate the repeats in the genomes of *S*. *tuberosum* and *S*. *lycopersicum*. Repeat family sequences identified using RepeatModeler were matched to library sequences of RepeatMasker/Repbase to identify the already known repeats among the novel repeats.

To verify the annotation provided by RepeatModeler for the identified repetitive elements, two different approaches were followed. Firstly, the repetitive library sequences were provided as an input to RepeatMasker which identifies repeats based on homology search against Repbase annotations. Secondly, RepeatProteinMasker (http://www.repeatmasker.org/cgi-bin/RepeatProteinMaskRequest), a tool which annotates repeats based on their amino acids domains over their translated frames, was executed on the repetitive sequence library to identify the conserved protein domains in the library sequences. Annotation of library sequences was done by mapping the annotations provided by the mentioned approaches. If a library sequence had same annotation in all methods, the sequence was annotated with highest confidence. However, if the annotations provided by the mentioned approaches were not found converging, annotation provided by the RepeatProteinMasker was assigned. If RepeatProteinMasker could not identify any protein domain in any given sequence, then, the annotation of the sequence was determined based on RepeatMasker and RepeatModeler. Besides this, sequences were also subjected to manual characterization processes based on the defining features like sequence, domain, internal elements, and target site duplications properties of various families.

All those repetitive families which could not be assigned any annotation were specified as “Unknown”. Unknown families of *S*. *tuberosum* and *S*. *lycopersicum* were matched against each other to identify similar families in both species. For further annotation of the “Unknown” repeat families identified by RepeatModeler, the consensus repeat family sequences were matched to non-coding RNA sequences downloaded from Rfam database (version 11) [42]. Unknown repeat family sequences were matched to the non-coding RNA sequences using BLASTN and best hits were used to characterize the unknown families. The remaining unknown repeat family sequences were scanned against the NCBI NT database using TBLASTX to identify any multi-copy genes/pseudo-genes. Remaining “Unknown” repetitive element families were characterized by searching for repeat family specific properties mentioned above. The co-ordinates of the repetitive elements identified in this study were also matched with the co-ordinates of the repetitive elements provided in the PGSC_DM_v4.03 (http://solanaceae.plantbiology.msu.edu/pgsc_download.shtml) for *S*. *tuberosum* and the ITAG v2.3 [2] for *S*. *lycopersicum*. This was performed in order to identify the commonly reported repeats by various approaches, as well as the novel repeats reported in this study.

Multiple sequence alignments of some families was performed using ClustalW [47], while phylogenetic trees were created using the Neighbor Joining method with a bootstrap value of 1000.

### Genomic Analysis of Repetitive elements

To identify the distribution pattern of repetitive elements across the genomes of *S*. *tuberosum* and *S*. *lycopersicum*, different analyses were performed. Percentage of a chromosome's length occupied by repetitive elements was calculated as the total number of base-pairs of repetitive element coverage for a given chromosome with respect to the total length of the respective chromosome. For this analysis, the co-ordinates of repetitive elements provided by RepeatMasker using consensus library sequences were analyzed while merging the overlapping regions using BedTools [40]. The count of base-pairs of repetitive elements was then used to calculate the percentage of chromosome occupied by repeats. To assess the most common repeat families for a particular chromosome, percentage coverage of every repeat family for each chromosome was also calculated.

To identify the overall distribution of repetitive sequences in terms of proximity to gene rich regions, the percentage of repetitive sequences within a gene or in its 5kb upstream region was calculated. For this study, repeat sequences having overlap with the coding sequence and 5kb upstream region were identified. Repetitive sequences found overlapping with the aforementioned regions were considered as those preferring gene rich regions. Percentage of repeat family sequences found in either upstream region of the gene or in the coding regions were also calculated using the above mentioned relation. All other repetitive sequences which were not found in the vicinity of genes (coding region + 5kb upstream), were considered as repetitive sequences preferring intergenic regions. To test whether there is any significant enrichment of genes around repetitive regions, binomial test was applied on the count of genes found to overlapping with repetitive elements. The null hypothesis stated was: “There is no significant enrichment of genes near repetitive elements”. The test was implemented in R.

A protein coding gene usually consists of exons, introns and UTRs. It was previously studied that the repetitive elements which were found residing within a coding region also showed differential preferences with respect to exonic or intronic regions [48]. Therefore, in order to identify such differential preference, repetitive elements found overlapping exclusively with either exons or introns were identified. For this analysis, the repetitive sequences within the co-ordinates of exons or introns were considered. UTRs were not considered in this study as UTRs have not yet been identified in both species.

To further assess the relationship between genomic location of repetitive elements and coding regions (exons) of the genome, Pearson Correlation Coefficient (PCC) was calculated between percentage coverage of repetitive elements and percentage coverage of coding region (exons) for every chromosome. The correlation between gene coverage and repeat coverage was then statistically validated by calculating the p-value of PCC via implementing *t*-test. This analysis was performed to identify whether gene density has any association with the accumulation of repetitive elements. Post ENCODE the scenario has changed a lot in terms of functionality of genomes and perceptions about so called junk elements. Many previous works have showcased how these elements have emerged as regulatory engines of genes, and their general influences over genes have been increasingly revealed in many recent reports [49–54]. This provided motivation to understand the distribution of genes with respect to repetitive elements in a genome.

### Analysis of Exonization

Exonization is the process of insertion of a non-protein coding region in the coding region of a gene, where this region starts functioning as a part of exon [48,53,55,56]. This occurs due to the presence of pseudosplice or splice donor sites within repetitive elements which lead to generation of a new gene sequence [55]. Along-with protein coding genes, certain long non-coding RNAs were also reported to be generated in a similar manner [57]. However, the fraction of such exonized transcripts in the transcriptome is low [58,59]. If any exonized gene provides improved functionality or a novel function, then such events may become fixed in the genome. To study the presence of such exonized transcripts in the two genomes, the sequence and structure of orthologous genes were analyzed. The orthologous gene-pairs were subjected to global alignment using EMBOSS Stretcher program to measure the sequence differences. The identified indels and substitutions were mapped against the identified repetitive elements.

The positions of indels and substitutions were mapped to the corresponding amino acid sequence to identify its impact over the protein structure. This was performed by comparing the six frames translated transcript sequences for respective amino acid sequence for both species. Amino acid sequences of *S*. *tuberosum* and *S*. *lycopersicum* were also subjected to global alignment using EMBOSS Stretcher. The indels and substitutions in the protein sequences were transformed to the nucleotide sequences. The changes in the amino acid sequences which were found within the repeat-overlapping region in the nucleotide sequences were recorded. To identify effects of these changes on protein structures, the secondary structure of protein sequences was studied using PsiPred [60]. Orthologous proteins showing changes in the secondary structure were then subjected to three dimensional (3D) structure prediction via threading using RaptorX [61]. Due to unavailability of suitable template (having sequence identity ≥ 30%) for these proteins in Protein Data Bank, homology protein models could not be built. Therefore, threading based structure prediction was performed [62,63]. This method realizes upon unique protein folds present in several resolved protein 3D structures [64]. Thus, in the absence of suitable protein structure template it was the best way to search for a protein fold present in target protein sequence against the fold library of resolved 3D structures. The 3D structures obtained were validated using web-server of RAMPAGE (http://mordred.bioc.cam.ac.uk/~rapper/rampage.php). Structures formed represented only part of the complete sequence due to threading based tertiary structure modeling. Therefore, residues showing changes in orthologous proteins were matched with the structures. 3D structures were then used as input in LigPlot+ [65] to visualize residues targeted hydrogen bonding and hydrophobic contacts showing changes in orthologous proteins due to repetitive elements.

### Transcription Factor Analysis

Many transcription factor binding sites (TFBS) were previously reported to be exapted from repetitive elements and regulate the downstream genes [51,66,67]. Thus, the TFBS within repeat overlapping regions in 2kb upstream sequences were identified and significant gain/loss of TFBS in the repeat overlapping region of every pair of orthologous genes was studied.

To analyze the possible TFBS hosted by repetitive elements, TFBS on the 2kb upstream region with respect to the start co-ordinate of the genes were identified using pPromotif [50,68]. pPromotif is a tool to identify TFBS on plant genomic sequences based on probabilistic models for various TFBS derived using large amount of experimental and high throughput data. So far, pPromotif has matrices modeled for 57 transcription factor families. pPromotif was executed using the default parameters. Count of every TFBS on 2kb upstream region of every orthologous gene pair was calculated and difference between the total number of sites was calculated. Gain/loss of TFBS for orthologous genes was also estimated. Binomial test was applied to statistically test the significance of the observed gain/loss of TFBS.

### Role of Repetitive elements in miRNA evolution

To analyze the influence of repetitive regions over miRNAs, miRNAs reported so far from both species were identified for their overlap with repeats. Pre-miRNA sequences were downloaded from miRBase (version 20) [41] and mapped to both the genomes. Using the identified co-ordinates, overlaps between miRNAs and repetitive sequences were calculated. Orthologous miRNAs were identified in *S*. *tuberosum* and *S*. *lycopersicum* by comparing the pre-miRNAs with each other, while considering their annotations. The repetitive content across and around them was analyzed to find out any potential association of repetitive elements with the evolution of miRNAs in the two species. Binomial test was also applied to identify any possible influence of repetitive elements on the accumulation of miRNAs with the null hypothesis that repetitive elements are not associated with miRNAs and thus would not be enriched in miRNA sequences. The orthologous miRNAs were also studied for the presence of conserved motifs and their positional arrangements in 2kb upstream and downstream regions around the pre-miRNA sequence, considering any possibility of detecting the eroded repetitive regions hosting the miRNAs. Using WATER tool from the EMBOSS package [69], local alignment was performed between these sequences and conserved motifs between these orthologous miRNAs were extracted. Motifs at least 5 bp in length and having overlap with a repetitive element in at least one of the orthologous miRNAs were considered. Moreover, multiple motifs present in the same arrangement for both orthologous miRNAs were considered as strong candidates for being footprints of some repetitive elements which might have been eroded during evolution [70,71].

### Abundance analysis of repetitive elements

Transcriptional activity and abundance of repetitive elements identified in this study was calculated using data from two different platforms namely, Next Generation Sequencing and Microarrays. For *S*. *tuberosum*, RNA-Seq data was taken from NCBI SRA (SRP005965) [1] having a total of 40 different experimental conditions, while for *S*. *lycopersicum* RNA-seq data was taken from three different experiments namely SRP019504 [72], SRP007969 [73] and SRP026374 [74] having a total of 55 experimental conditions. Normalized microarray data and the probe sequences were downloaded from Array Express with the accessions E-MTAB-629 and E-MTAB-634 for *S*. *tuberosum* [75] for 42 conditions. For *S*. *lycopersicum*, microarray data was downloaded from GEO with the accession ID GSE22300 [76], making a total of 10 conditions.

For abundance calculation of repetitive elements and genes of both species, RPKM was calculated using SeqMap and R-seq with default commands. From the RPKM value, the average expression of every repeat family was then calculated by using the following equation:

$$\begin{array}{l} \text{Average abundance of an expressing repeat family}=\\ \frac{\mathrm{Sum of abundance of all members of a Repeat Family}}{\mathrm{Total number of members of a repeat family}\times \mathrm{Total number of experimental conditions}} \end{array}$$

For calculating expression from microarray platform, methodology as proposed by Reichmann *et al*. [77] was used. Probe sequences were searched against the genomes using BLAST. From BLAST result, the best match for every probe was identified and the co-ordinates of the probes were recorded. Co-ordinates of the probes were matched with the co-ordinates of the genes. If a probe overlapped with a gene with more than 90% of its length, expression values of the probe was assigned to the respective gene. The probes which could not be matched to genes were further matched with co-ordinates of repeats and expression of the probe whose length coverage was more than 90% with repeats was assigned to the corresponding repeat. Average expression of repeat family was then calculated using the above mentioned equation.

Although RPKM and microarray based abundance estimations were done for repetitive elements, small RNA sequencing data was also used, considering their reported association with small RNA biogenesis. For *S*. *tuberosum*, sRNA read data was downloaded from NCBI SRA under accession ID SRP033230 [78]. For *S*. *lycopersicum*, sRNA read data was downloaded from GEO (GSE18110) [79]. These sRNA reads were processed by removing adapter sequences and only those sRNA reads were selected which were at least 17 bp long. These processed sRNA reads were first mapped to ncRNA sequences downloaded from Rfam (version 11) and to the transcript sequences to remove any read sequence which could be a degradation product. The remaining reads were mapped to the repetitive elements using Bowtie [80] with maximum of one mismatch.

All metadata associated with this work has been made freely available at: http://scbb.ihbt.res.in/SCBB_dept/solanum_metadata.php and https://github.com/mrigayamehrajha/Solanum-Repeats-Metadata.

## Results and Discussion

**Fig 1:**
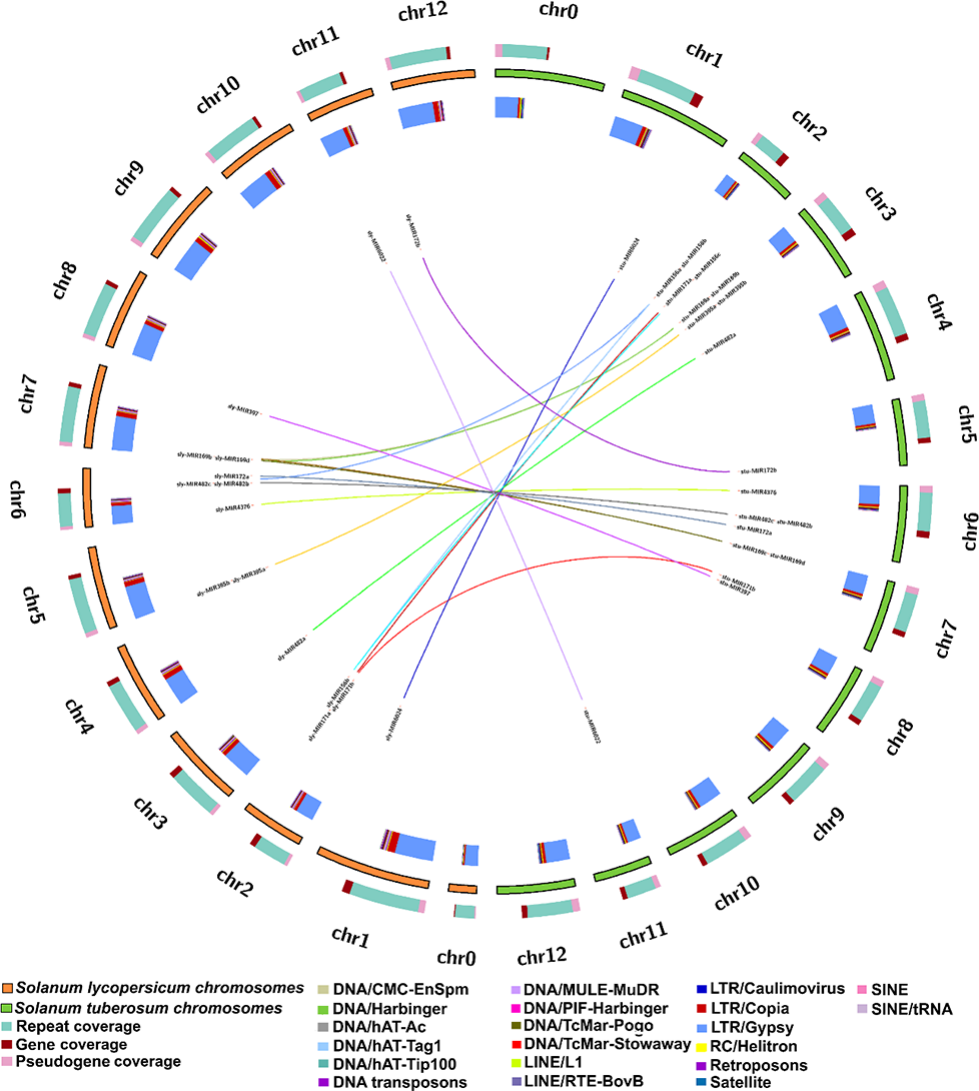
Distribution of repetitive elements, genes, pseudo-genes and positions of orthologous miRNAs having footprints of old repetitive elements in (A) *Solanum tuberosum* (B) *Solanum lycopersicum*.

The total genome size of *S*. *tuberosum* is 810.6 MB, ~85% of the genome is sequenced and total number of yet to be sequenced regions contributes towards 15.78% (127,958,425 bp) of the genome. For *S*. *lycopersicum*, the total genome size is 781.6 MB with ~5% (44,030,063 bp) of the genome as yet not sequenced. Thus, the sequences of these two genomes were sufficiently complete for this study. The repetitive elements have been reported to occupy a significant percentage of different genomes ranging up to ~80% of the genome sequence in many plant species like wheat and *Capsicum* spp [81,82]. *S*. *tuberosum* and *S*. *lycopersicum*, both have moderately large genomes with significant portion of their genome being represented as complex repetitive elements. Detailed identification and characterization of repetitive elements in these genomes provided a more complete map of repetitive elements of these two genomes and their impact over the associated genomes, several of which were not reported earlier. It was identified that repetitive elements occupy ~49% and ~60% of the genomic sequence of *S*. *tuberosum* and *S*. *lycopersicum*, respectively, with chromosome 12 as the most repeat rich chromosome in both *S*. *tuberosum* and *S*. *lycopersicum* (Fig 1, Table 1). The repeat families identified in both species included DNA transposons and retrotransposons, some of which could not be assigned to any particular DNA transposon or retrotransposon order. Repetitive elements are multifaceted which are increasingly being associated with several key functional aspects of the genome. Repetitive elements identified in this study were investigated with respect to their distribution across the genomes and it was found that these elements were remarkably abundant in the gene-coding regions. Since the presence of repetitive elements around genes could have a plethora of outcomes, the functional relevance of these elements was studied by analyzing their impacts on the associated genes via *cis*-regulation, exonization and miRNA evolution. It was found that ~4% (41,966) of repetitive elements in *S*. *tuberosum* and ~0.13% (1,358) repetitive elements in *S*. *lycopersicum* overlap with regions of the genome which are still not sequenced. Therefore, it is unlikely to see any major change in the findings made here even after sequencing of these regions.

**Table 1:** Chromosome wise coverage of repetitive elements. Coverage of different repeat super-families was calculated as the percentage of nucleotides represented by repetitive elements out of total nucleotides of every chromosome.

***Solanum tuberosum***			
**Chromosome**	**Total number of base pairs occupied by repeats**	**Total number of base pairs in the chromosome**	**Percentage of chromosome occupied by repeats**
chr0	33,415,365	85,736,662	38.97
chr1	43,197,010	88,663,952	48.72
chr2	19,955,974	48,614,681	41.05
chr3	28,514,945	62,190,286	45.85
chr4	36,487,020	72,208,621	50.53
chr5	27,570,804	52,070,158	52.95
chr6	28,789,650	59,532,096	48.36
chr7	28,734,258	56,760,843	50.62
chr8	28,479,417	56,938,457	50.02
chr9	32,022,384	61,540,751	52.03
chr10	32,804,959	59,756,223	54.90
chr11	21,872,185	45,475,667	48.10
chr12	33,669,946	61,165,649	55.05
Total	395,513,917	810,654,046	48.79
***Solanum lycopersicum***			
**Chromosome**	**Total number of base pairs occupied by repeats**	**Total number of base pairs in the chromosome**	**Percentage of chromosome occupied by repeats**
chr0	14,457,253	21,805,821	66.30
chr1	52,060,945	90,304,244	57.65
chr2	24,994,446	49,918,294	50.07
chr3	36,771,573	64,840,714	56.71
chr4	37,594,132	64,064,312	58.68
chr5	41,130,811	65,021,438	63.26
chr6	24,862,196	46,041,636	54.00
chr7	41,046,411	65,268,621	62.89
chr8	39,637,992	63,032,657	62.88
chr9	43,430,035	67,662,091	64.19
chr10	40,148,283	64,834,305	61.92
chr11	31,877,585	53,386,025	59.71
chr12	42,301,100	65,486,253	64.60
Total	470,312,762	781,666,411	60.17

### Repetitive elements identified in *S*. *tuberosum* and *S*. *lycopersicum*

The initial identification of repetitive elements in *S*. *tuberosum* genome was performed on 66,245 super-scaffolds [1] using similarity based approaches like RepeatMasker and RepeatProteinMask. 62.2% of the genome of *S*. *tuberosum* was reported to be represented by repetitive elements where ~25% of these repeats were not characterized. The super-scaffolds were distributed into twelve chromosomes and those which could not be assembled and assigned to any chromosome were merged to create a thirteenth chromosome, Chromosome 0. Therefore, sections of many previously identified repetitive elements were divided into different chromosomes, while some were merged together. This might lead to the identification of many new repetitive elements as well as removal of some previously identified repetitive elements across the super-scaffolds. Similarly, in *S*. *lycopersicum*, though the LTRs were found using LTR_STRUC program, most of the repetitive elements were identified using RepeatMasker [2]. Initially, the total repetitive content of these two genomes were reported as 57.6% and 62.2% in *S*. *lycopersicum* and *S*. *tuberosum* (at scaffold level), respectively. However, the observed repetitive content has reduced once the contigs and scaffolds were merged and distributed across the chromosomes with values lower than expected repetitive content (19.49% for *S*. *tuberosum*). As already mentioned, the previous repeat annotations for both the genomes had considered similarity based approaches predominantly to report the repeats, leaving ample scope for novel elements' discovery using combination of similarity and *de novo* based approaches. The similarity based approaches usually miss out sparsely similar and divergent members as well as species specific repeats. The present work applied a combination of similarity based, *de novo* based and manual analysis to identify the repetitive elements in the two species.

**Fig 2:**
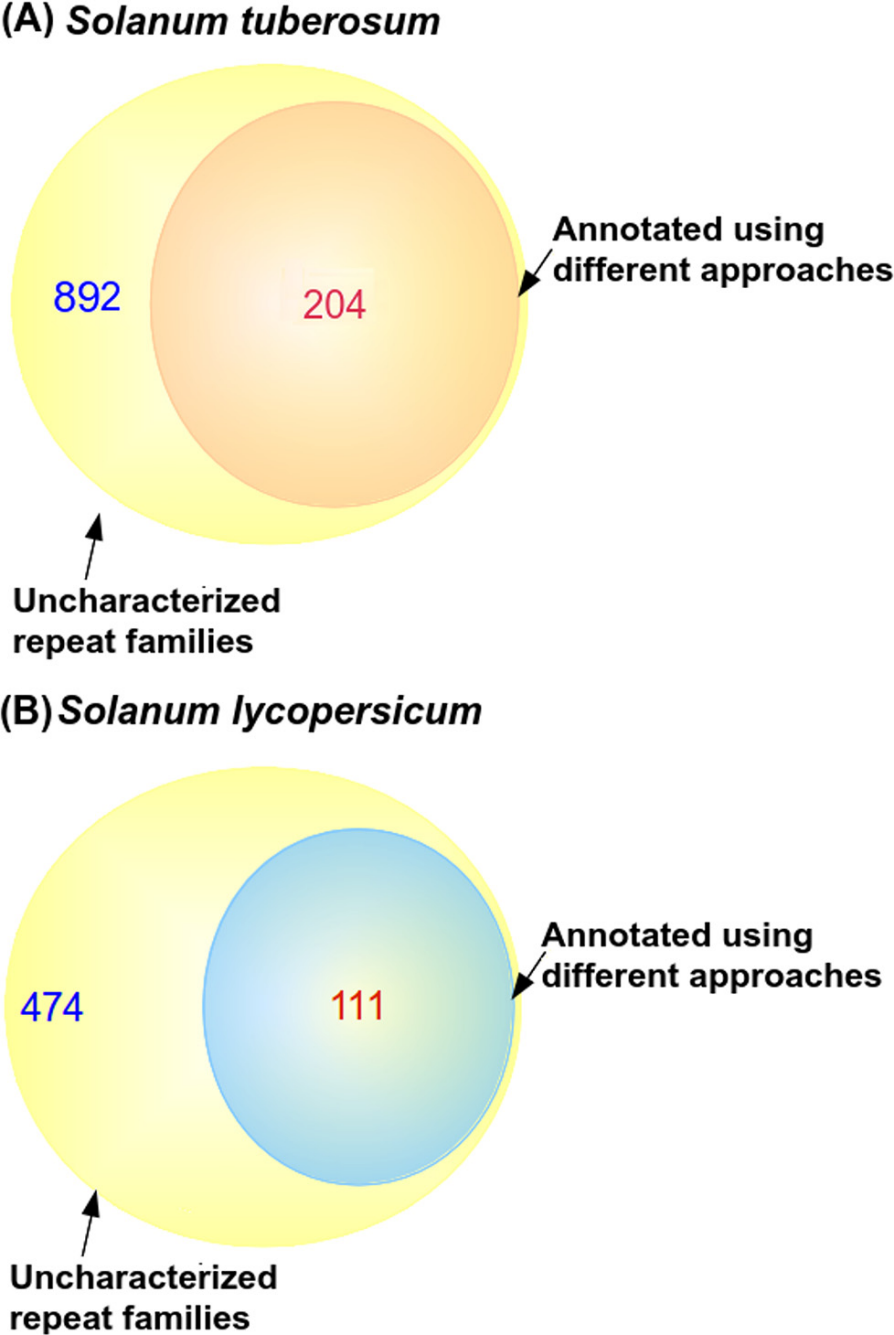
Venn diagram showing annotation provided to the non-characterized repeat families using different approaches (A) *Solanum tuberosum* (B) *Solanum lycopersicum*. The inner circle represents the repeat consensus sequences assigned annotations using the different approaches. 204 (out of 892) non-characterized repeat families were characterized in *S*. *tuberosum* and 111 (out of 474) non-characterized repeat families were annotated in *S*. *lycopersicum*.

RepeatModeler identified 1,921 and 1,438 consensus repeat family sequences in *S*. *tuberosum* and *S*. *lycopersicum* respectively (Table 2, S1 Table). Of these identified repeat families, 892 repeat family consensus sequences in *S*. *tuberosum* and 474 repeat family consensus sequences in *S*. *lycopersicum* were labeled as “Unknown” as no significant similarity with known repeat families could be detected for these families. For verifying the annotation of all repeat consensus families and to provide annotations to the non-characterized repeat consensus family sequences, using RepeatProteinMasker and RepeatMasker, the annotations were searched against the the annotated library sequences as mentioned in the methods section. Thus, annotation was verified and provided for 1,233 out of 1,921 and 1,075 out of 1,438 consensus repeat families for *S*. *tuberosum* and *S*. *lycopersicum*, respectively. A total of 204 “Unknown” repeat families in *S*. *tuberosum* and 111 “Unknown” repeat family consensus sequences in *S*. *lycopersicum* were annotated (Fig 2). A total of 1,061,377 and 793,890 repetitive sequences were identified in *S*. *tuberosum* and *S*. *lycopersicum* genomes, respectively. After removing the sequences annotated as rRNA, snRNA, tRNA, simple repeats, low complexity and unannotated elements, 629,713 and 589,561 repetitive elements belonging to different complex repeat families were obtained for *S*. *tuberosum* and *S*. *lycopersicum*, respectively (Table 2). DNA transposons identified in this study included hAT elements like hAT-Ac, hAT-Tag1, hAT-Tip100, Harbinger/PIF-Harbinger, CMC-EnSpm, MULE-MuDR, TcMar-Pogo/TcMar-Stowaway and Helitrons. Retrotransposons identified included non-LTR elements like LINE L1 and RTE-BovB, SINE elements, LTR elements including Gypsy, Copia, Caulimovirus and ERVs (Table 2).

**Table 2:** Distribution of identified repeat families in *S*. *tuberosum* and *S*. *lycopersicum*. Total number of families, super-families and elements identified in both species are presented.

Repeat Family	Repeat Super-family	Total number of families	Total number of elements
***Solanum tuberosum***			
DNA transposons	DNA/CMC-EnSpm	43	17,198
	DNA/CMC-EnSpm?	4	2,801
	DNA/Harbinger	18	14,341
	DNA/hAT-Ac	22	10,951
	DNA/hAT-Tag1	3	1,987
	DNA/hAT-Tag1?	2	1,201
	DNA/hAT-Tip100	21	12,689
	DNA/MULE-MuDR	16	8,318
	DNA/PIF-Harbinger	9	9,469
	DNA transposons	35	29,354
	DNA/TcMar-Pogo	6	1,967
	DNA/TcMar-Stowaway	13	27,720
	RC/Helitron	5	1,957
Retrotransposons	LINE/L1	63	23,521
	LINE/RTE-BovB	17	27,091
	LTR/Caulimovirus	23	5,858
	LTR/Copia	172	72,026
	LTR/Gypsy	558	334,474
	Retroposon	11	9,926
	SINE	2	3,853
	SINE/tRNA	6	10,567
Uncategorized	Unknown	688	329,727
snRNA		2	487
rRNA		5	1,845
Satellite		4	2,444
***Solanum lycopersicum***			
DNA transposons	DNA/CMC-EnSpm	28	21,007
	DNA/CMC-EnSpm?	2	2,442
	DNA/Harbinger	22	13,390
	DNA/hAT-Ac	30	15,164
	DNA/hAT-Tag1	8	3,655
	DNA/hAT-Tag1?	2	1,021
	DNA/hAT-Tip100	16	11,757
	DNA/MULE-MuDR	34	16,344
	DNA/PIF-Harbinger	9	6,118
	DNA transposons	38	34,059
	DNA/TcMar-Pogo	4	5,132
	DNA/TcMar-Stowaway	14	22,343
	RC/Helitron	2	2,398
Retrotransposons	LINE/L1	28	13,653
	LINE/RTE-BovB	14	21,154
	LTR/Caulimovirus	4	1,223
	LTR/Copia	165	71,179
	LTR/ERV1	1	56
	LTR/Gypsy	525	306,511
	Retroposon	11	9,062
	SINE	4	3,772
	SINE/tRNA	6	7,614
Uncategorized	Unknown	363	145,776
snRNA		1	157
rRNA		1	272
tRNA		3	838
Satellite		1	507

The remaining non-characterized consensus repeat families amounted to a total of 688 repetitive element families in *S*. *tuberosum* and 363 repetitive element families in *S*. *lycopersicum* (Table 2). The non-characterized families might account for some novel repeat families or pseudo-genes for which further characterization of these unknown repeat families was performed. These sequences were searched against the non-coding RNA sequences downloaded from the Rfam database. Of the 688 unknown repeat family consensus sequences in *S*. *tuberosum*, only nine repeat family consensus sequences matched the annotated non-coding RNA sequences which included introns, miRNA genes, 5S rRNA and SRP while the remaining 679 repeat family sequences did not match any non-coding RNAs (S2 Table). Similarly, out of 363 unknown repeat family sequences of *S*. *lycopersicum* only two repeat family consensus sequences matched the non-coding RNA sequences in Rfam (S2 Table) which included introns and miRNA genes. The remaining 679 repeat family consensus sequences of *S*. *tuberosum* and 361 repeat family consensus sequences of *S*. *lycopersicum* were searched against the NCBI nucleotide (NT) database using TBLASTX (S2 Table). In this analysis, 574 repeat family consensus sequences of *S*. *tuberosum* and 326 repeat family consensus sequences of *S*. *lycopersicum* matched with known nucleotide sequences as pseudo-genes (S2 Table). The remaining 105 repeat family consensus sequences of *S*. *tuberosum* and 35 repeat family consensus sequences of *S*. *lycopersicum* were then searched for conserved known motifs which would enable their classification. Motifs for internal Pol III promoters, A-Box and B-box, which are usually found within the SINE elements were searched in the remaining consensus sequences. Same was done for 5' and 3' conserved motifs for *B*. *oleraceae* SINE elements [83,84]. 105 repeat family consensus sequences in *S*. *tuberosum* and 34 repeat family consensus sequences in *S*. *lycopersicum* showed the presence of at least one of these motifs (S2 Table), suggesting their possible association with SINE elements. These non-characterized repetitive families of *S*. *tuberosum* and *S*. *lycopersicum* were also compared with each other to identify the common non-characterized families. In this analysis, it was found that 41 out of 688 non-characterized families in *S*. *tuberosum* matched with 44 out of 363 non-characterized families of *S*. *lycopersicum*, where some repeat family consensus sequences of *S*. *lycopersicum* exhibited multi-homologs for same repeat families in *S*. *tuberosum*.

The repeat families identified here were compared with the repeat families identified in the 4.03 version of the *S*. *tuberosum* genome released by the Potato genome sequencing consortium (PGSC_DM_v4.03) (http://solanaceae.plantbiology.msu.edu/pgsc_download.shtml) and the initially identified repetitive elements in *S*. *lycopersicum* by Tomato genome consortium (ITAG 2.3). 681 (35.45%) families of *S*. *tuberosum* identified in this study belonging to 7 repeat super-families matched with 7 repeat super-families (DNA transposons, DNA/Harbinger, DNA/hAT, LTR/Copia, LTR/Gypsy, RC/Helitron and SINE) identified by the potato genome sequencing consortium (PGSC_DM_v4.03), while 732 (50.90%) families of *S*. *lycopersicum* identified in this study belonging to 5 repeat super-families matched with 5 repeat super-families (DNA transposons, DNA/hAT, LTR/Copia, LTR/Gypsy and SINE) identified by the ITAG2.3 in *S*. *lycopersicum* genome. Although, a total of 699,160 (88.07%) elements identified in this study matched with 673,145 (93.56%) elements identified by the ITAG2.3 in *S*. *lycopersicum* genome and a total of 346,179 (32.62%) elements identified in this study matched with 331,009 (81.76%) elements identified by the PGSC_DM_v4.03 in *S*. *tuberosum*. Some annotations, however, differed from the previously done annotations at family level. Details are provided in S3 Table.

### Distribution of repetitive elements across the genomes

It has been observed that in mammalian species, non-LTR elements (LINEs and SINEs) are more abundant while in plants LTR elements are more prevalent [85]. Such differential accumulation of repetitive elements has been proposed to be either due to some species-specific amplifications or deletions of specific elements [85]. Even within a species the distribution of repetitive elements is highly dependent upon the family of complex repeats [85]. Some repeats have been found enriched in the regulatory regions upstream the protein coding genes, while some are found within introns where some get exonized and domesticated [86]. Thus, to understand the genomic hot spots for association of repetitive elements and their overall spread, the distribution patterns of these elements in the two genomes were studied.

**Fig 3:**
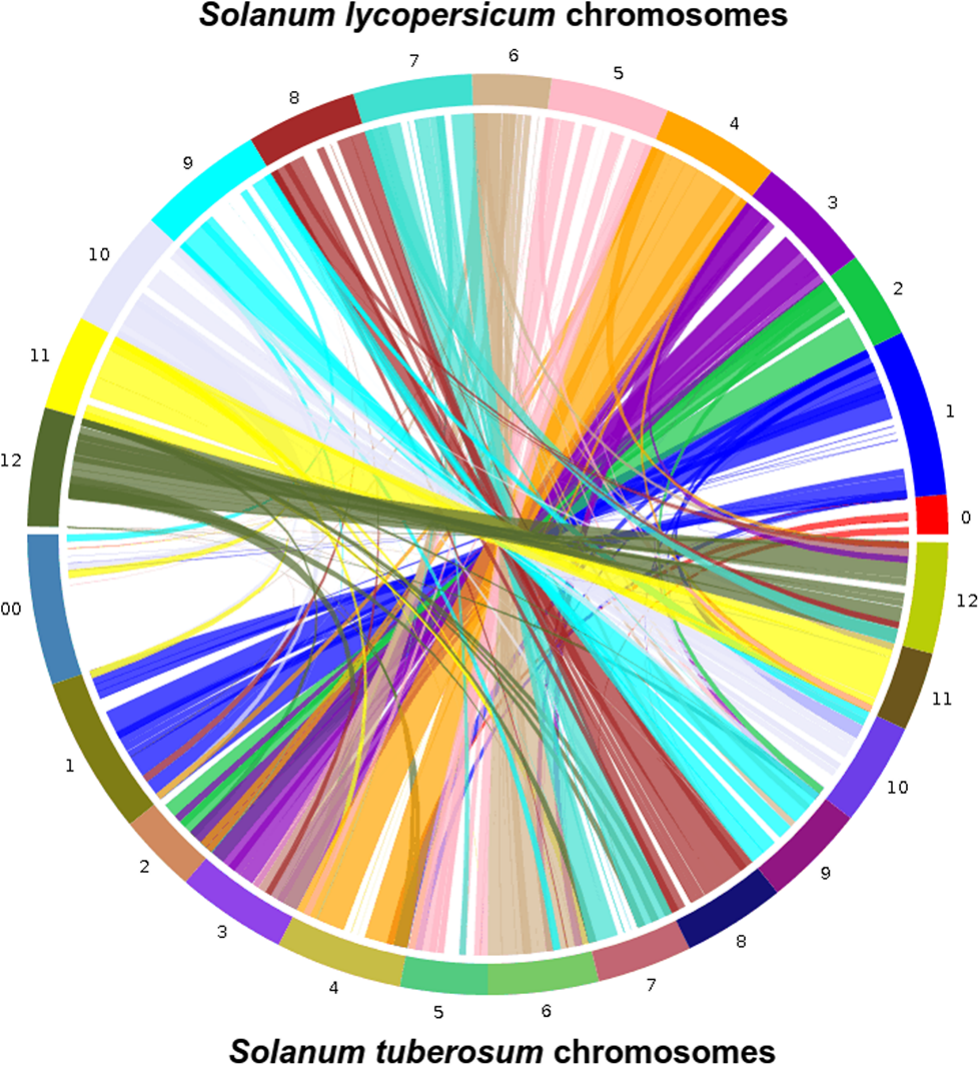
Syntenic regions across *S*. *tuberosum* and *S*. *lycopersicum*. All the 12 chromosomes in *S*. *tuberosum* and *S*. *lycopersicum* were observed to show high syntenic relationship, showing similar distribution of genes in both species.

The repeat families most prevalent in the genomes of *S*. *tuberosum* and *S*. *lycopersicum* were analyzed in two ways: 1) the total number of copies of every repeat family, and 2) genome-wide distribution of repeat families were calculated. The super-family having maximum copies in both species was LTR/Gypsy (Table 2). The total number of repetitive elements belonging to LTR Gypsy were 334,474 and 306,511 in *S*. *tuberosum* and *S*. *lycopersicum*, respectively. Similarly, coverage of each repeat super-family on every chromosome was also calculated which revealed LTR Gypsy occupying the largest number of bases on every chromosome in both the genomes (Fig 1, S4 Table). Two other repeat super-families which occupied a significant percentage of the genome sequence were LTR Copia and LINE elements L1 (S4 Table). This trend is in sync with earlier observations which report LTR Gypsy as the most prevalent element in plant genomes and presence of LTR elements higher than any other complex repetitive element in overall. Pearson Correlation Coefficient (PCC) was calculated between the gene coverage and coverage of repeat super-family for every chromosome (Table 3) to identify the association between gene coverage and accumulation of repetitive elements. It was found that coverages of DNA transposons and TcMar-Stowaway were significantly correlated with the gene coverage, supported by significant p-values (Table 3). When the correlation of gene coverage and coverage of LTR/Gypsy elements was calculated while considering chromosome 0, it was significant only in *S*. *lycopersicum*. When correlation was estimated excluding chromosome 0, correlation coefficient was significant in both species. Chromosome 0 symbolizes the yet incomplete and unassigned parts of the genome. This chromosome is ~2.5 times larger in *S*. *tuberosum* than in *S*. *lycopersicum*. Similar density of repetitive elements on the chromosomes of *S*. *tuberosum* and *S*. *lycopersicum* thus encouraged the identification of syntenic regions in the two genomes. It was found that all the twelve chromosomes of *S*. *tuberosum* and *S*. *lycopersicum* were highly syntenic to each other (Fig 3), sharing high similarity for genes and repeats distribution in overall.

**Table 3:** Correlation between coverage of repeat family and gene coverage on every chromosome.

***Solanum tuberosum***			
**Repeat Type**	**PCC**	**T-statistic**	**P-value**
DNA/TcMar-Stowaway	0.9620917122	11.7000058689	1.51E-007
DNA	0.9596712079	11.3219072983	2.11E-007
DNA/Harbinger	0.9347881483	8.7282979872	2.83E-006
DNA/PIF-Harbinger	0.9334391543	8.6299382074	3.16E-006
DNA/TcMar-Pogo	0.8493600038	5.3371237214	2.38E-004
DNA/MULE-MuDR	0.8462555467	5.2680784776	2.65E-004
DNA/hAT-Tag1	0.7845382608	4.1962606691	1.49E-003
DNA/hAT-Tip100	0.7474276283	3.7314099879	3.32E-003
SINE/tRNA	0.7375728291	3.622641389	4.01E-003
DNA/hAT-Ac	0.6500795482	2.8374338255	1.62E-002
SINE	0.6215187549	2.6312820978	2.34E-002
LINE/RTE-BovB	0.6028804797	2.5062037609	2.92E-002
LTR/Caulimovirus	-0.5729613412	2.3186171403	4.07E-002
LTR/Copia	0.4799071858	1.8142449766	9.70E-002
Satellite	-0.4216051498	1.542057425	1.51E-001
Retroposon	0.3105955013	1.0837275421	3.02E-001
RC/Helitron	-0.2474331189	0.8469796697	4.15E-001
LTR/Gypsy	-0.2390211119	0.8164074281	4.32E-001
LINE/L1	0.1577892178	0.5299666206	6.07E-001
DNA/CMC-EnSpm	-0.0200565794	0.0665335318	9.48E-001
***Solanum lycopersicum***			
**Repeat Type**	**PCC**	**T-statistic**	**P-value**
DNA/Harbinger	0.9544544177	10.6100060709	4.08E-007
DNA/TcMar-Stowaway	0.9455427665	9.6345035426	1.07E-006
LTR/Gypsy	-0.9252673099	8.0902647726	5.87E-006
DNA	0.8976427794	6.7551253297	3.14E-005
DNA/PIF-Harbinger	0.8687875924	5.818921915	1.16E-004
DNA/hAT-Tip100	0.7270065462	3.511652681	4.87E-003
DNA/TcMar-Pogo	0.7137034022	3.379389004	6.15E-003
DNA/MULE-MuDR	0.612600281	2.5705764119	2.60E-002
SINE/tRNA	0.6050936111	2.5207028186	2.84E-002
DNA/hAT-Ac	0.6041348528	2.5144103905	2.88E-002
DNA/hAT-Tag1	0.5995442809	2.4845184335	3.03E-002
SINE	0.5610626717	2.2479999158	4.61E-002
LINE/RTE-BovB	0.3807148849	1.3655225463	1.99E-001
LINE/L1	-0.3745541236	1.3397850768	2.07E-001
LTR/ERV1	0.3642191812	1.2970698665	2.21E-001
LTR/Copia	-0.3101460642	1.0819923379	3.02E-001
RC/Helitron	0.3012442711	1.0477870658	3.17E-001
DNA/CMC-EnSpm	0.2752760876	0.9496781064	3.63E-001
LTR/Caulimovirus	-0.2157779953	0.7329204594	4.79E-001
Retroposon	-0.0845703336	0.2814965229	7.84E-001
Satellite	0.0297194853	0.0986119407	9.23E-001

The co-ordinates of the repetitive elements and genic regions (5kb upstream + coding region) were studied to find any possible overlaps between the genic and repetitive regions. It was found that in *S*. *tuberosum*, 99.29% (38,740 genes out of 39,021 genes) genes had repeats overlapping either with their coding sequence or with the 5kb upstream region, whereas in *S*. *lycopersicum* 98.92% (34,303 genes out of 34,675 genes) genes had repeats overlapping with their genic regions and/or with the 5kb upstream region. This suggests that majority of protein coding genes in both species might be under the influence of repetitive sequences. This was also supported by a binomial test performed in both species suggesting a significant association between coding genes and repetitive elements (p-value <2.2E-16). When similar analysis was performed for the total repetitive elements identified and their genomic preferences, it was found that in *S*. *tuberosum* 33.72% of repetitive sequences (357,893 out of 1,061,377 repetitive sequences) were found overlapping with the genic regions while in *S*. *lycopersicum*, 30.37% of the repetitive sequences (283,295 out of 932,559 repetitive sequences) were found overlapping with the genic regions, suggesting the repetitive elements were more enriched in the intergenic regions rather than the genic regions. This distribution pattern was also studied for every repeat family on the basis of the count of the repetitive elements found in the genic or intergenic regions. It was identified that most of the repeat families had more number of elements in the intergenic regions than the genic regions. This pattern is quite understandable as compared to the intergenic regions the amount of genic region is very small. When the relative distribution of repeats was compared for the genic regions with the same for the intergenic regions, the association with intergenic region was found significantly higher. A *t-test* was also performed to validate significant enrichment of repetitive elements in the intergenic regions which gave a highly significant p-value for the enrichment of repetitive elements in the intergenic regions (p-value = 0.0001957 in *S*. *tuberosum* and p-value = 6.018e-11 in *S*. *lycopersicum*).

**Fig 4:**
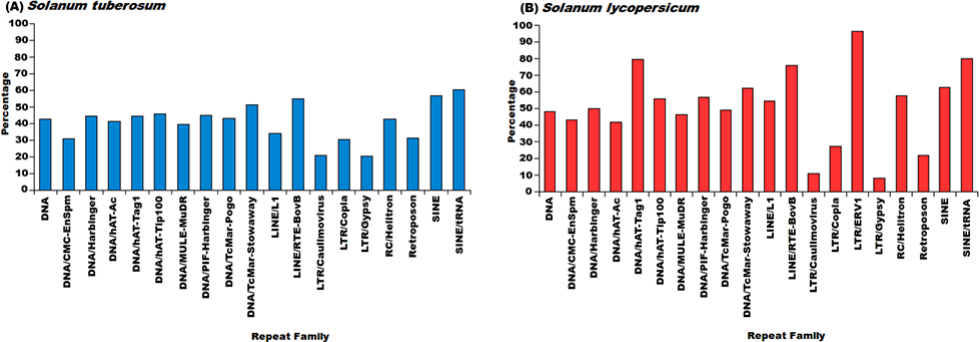
Percentage of repetitive elements found in the genic and intergenic regions in (A) *Solanum tuberosum* and (B) *Solanum lycopersicum*. Percentage calculated from the total elements identified for every repeat super-family. Most of the repeat super-families in both species prefer intergenic regions as their insertion sites.

**Fig 5:**
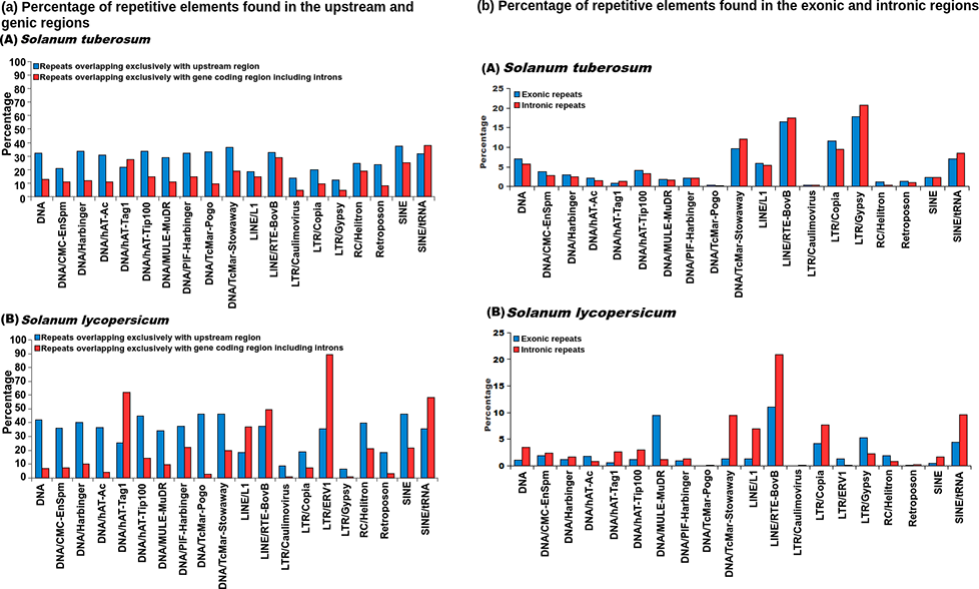
(a): Percentage of repetitive elements found in the upstream and genic regions in (A) *Solanum tuberosum* and (B) *Solanum lycopersicum*. In comparison to genic regions, repeat super-families prefer to be inserted within the upstream regions harboring regulatory elements. (b): Percentage of repetitive elements found in the exonic and intronic regions in (A) *Solanum tuberosum* and (B) *Solanum lycopersicum*. Distribution of repeat super-families did not show any preferential enrichment in exonic and intronic region in *S*. *tuberosum*, while in *S*. *lycopersicum*, different repeat super-families show differential enrichment in intronic and exonic region.

When analyzed for different repeat families, for LINE elements RTE-BovB, SINE elements and DNA transposon Stowaway, the larger proportions of the repetitive elements were found within the genic regions (Fig 4) in *S*. *tuberosum*. In *S*. *lycopersicum*, the DNA transposons hAT-Tag1, hAT-Tip100, PIF-Harbinger, RC Helitron and SINEs showed preferential abundance in the genic regions while the genic region preference of LTR/ERV1 repeat family was found very pronounced. The other repeat super-families were found within the intergenic regions are shown in Fig 4. The presence of repetitive elements within the coding regions points towards possible exonization event, while the presence of repetitive elements within the upstream region of gene might provide evidence for the possible exaptation of *cis*-regulatory elements. To study the repetitive elements' contribution towards such events, an analysis was performed and abundance of repetitive elements within the boundaries of gene coding regions or upstream regions was analyzed. It was found that most of the repeat families were more prevalent in the upstream regions of the genes (Fig 5(A)). Complex repetitive elements like LTR elements possess promoter elements which may provide regulatory elements for the downstream gene and in turn influence the gene expression in a tissue or stage-specific manner. A large number of previous studies highlighted the importance of accumulation of repetitive elements near genes where these elements served as sources of variation [87,88]. Presence of a repetitive elements in introns has been shown to influence the spatio-temporal expression of genes, creation of cryptic splices sites and other effects, whereas insertion of repetitive elements has been considered to be more devastating and associated with many disease conditions [89–94]. Therefore, identification of the different insertion spots of repetitive elements would provide insights into the possible mechanisms through which repetitive elements might be influencing genes and their products. To identify the preferential insertion of these elements in exonic or intronic regions, percentage count of repetitive elements overlapping with the exonic or intronic regions was calculated. It was found that in *S*. *tuberosum*, the distribution appeared uniform for both the regions. However, for *S*. *lycopersicum*, DNA transposon MULE/MuDR and LTR/ERV1 showed preferential accumulation within the exonic regions, while DNA transposons, TcMar-Stowaway, LINE elements RTE-BovB and SINE elements displayed a preferential association with the intronic regions (Fig 5(B)).

### Impact of exonized repeats on protein coding gene's structure

As mentioned by Jacob [95], “to create is to recombine”, thus there is a high probability that applying various permutations and combinations to existing genomic materials, evolution shapes a genome. In this context, it is presumable that exonization is a favored mechanism of evolution as creating new combinations by incorporating segments of repetitive elements seems much easier than *de novo* generation of functional elements. Exonization occurs due to the presence of splice-sites within the repetitive elements which are found overlapping with genes. There are many possible outcomes of exonization, most of which lead towards beneficial inclusion and fixation [96,97]. Exonization of repetitive elements and their impact on shaping the transcriptome of various species has been studied widely [48,55,98–100]. SINE elements, specifically Alu elements, have been associated with many exonized transcripts having consequential effects [48,49,101,102]. Although, the protein coding potential of exonized elements has been under speculation, the contributions of repetitive elements to provide exons to transcript sequences is undeniable [56,59]. In *A*. *thaliana*, ~2000 loci have been reported to be derived from the segments of repetitive elements [93]. In rice, exonization of Ds elements has been studied for *epsps* gene [96]. The increasing examples being uncovered with regard to exonization of different repetitive elements might be viewed as a widely opted mechanism by genome to create new genes. However, this process can also have negative impacts on the fitness of the genome [101]. The exonized genes might become causative agents for different diseases and subject to selective screening [101]. However, to minimize the possibility of negative effects of exonization, most of these events occur in the duplicated genes where the possibility of interference with the gene function is lowered as well as ways to evolve new genic isoforms are opened. To study any possible impact of exonization of repetitive elements over the structures of the genes and possible variations, the orthologous sequences were studied and their secondary and tertiary structures were compared with respect to exonized repeats and possible variations. A comparative analysis for homologous amino acids sequences of these two species revealed that there were 27,923 orthologous gene pairs (S5 Table). Global alignment was performed for each orthologous pair to identify the changes in the sequences in the form of indels and substitutions. However, only 2,968 gene-pairs had indels associated with repeat-inhabited genic regions. Similar analysis for substitution positions was performed and it was found that 27,923 genes had substitutions in their alignments, whereas only 4,723 gene-pairs had substitutions which were found within the repeat-overlapping region of the genes. These changes (indels and substitutions) in nucleotide sequences were translated at the level of amino acids and it was identified that only 120 gene-pairs had changes in amino acid sequences corresponding to the changes in repeat-inhabited regions of the genes. It was further identified that, 61 gene pairs had changes in their secondary structure for the corresponding amino acid change (indels and substitution) positions. The amino acid sequences of these 61 gene-pairs were then subjected to 3D structure modeling using threading as the homology between these sequences and known protein structures in PDB was extremely poor (identity < 30%). After the prediction of the 3D structures, residue positions which were identified to have undergone changes in the secondary structures due to the presence of repetitive elements were mapped to 3D structures of their respective proteins. This was performed in order to look for the variations in orthologous proteins due to these changes. It was identified that many of these residues were at locations for which 3D structures were not available. Therefore, after removing such residues only 23 orthologous genes remained available for analysis. These remaining residues were studied using LigPlot+ and changes in the local environments of these residues (S6 Table) were observed. For the sake of clarity, these changes were categorized into three parts: substitutions, insertions in *S*. *tuberosum* genes and insertion in *S*. *lycopersicum* genes. Overall protein stability and conformation is determined by hydrogen bonds, van der Waals' forces and hydrophobic contacts formed among amino acid residues. As given in Table 4, barring a few cases, most of the residues which were substituted or inserted either in *S*. *tuberosum* or *S*. *lycopersicum*, had a comparatively more hydrophobic local environment. These changes might have consequential effects on the functioning of proteins and their binding specificity. Due to substitution and insertions at corresponding positions, the local environments having hydrophobic contacts and hydrogen bonding patterns varied leading to changed conformation of orthologous proteins. Hydrogen bonds contribute little to overall protein stability, but they align molecular groups in a specific orientation giving proteins a defined structure. When different non polar residues come closer, the extent of solvation decreases due to availability of less surface to water resulting in increase in entropy and thereby providing more stability to the protein structure. Thus, changes in the hydrophobic or H-bonding patterns may lead to alterations in the activity of orthologous proteins. The current analysis underlined the role of repetitive elements in bringing structural changes in proteins through exonization, however, for a limited number of genes. Most of these changes did not influence the protein structure and the critical regions significantly, an observation very similar to previous findings [51]. A comparison of the exonized repetitive regions of genes with the non repetitive coding regions of the genes for partition of changes between these two regions also suggested no significant difference, corroborating that repeats had no significant impact over the structure of the genes and their protein products. All these findings are in concordance with the previous studies which reported least structural changes by the repeats and maintenance of neutral evolution [51,59].

**Table 4:** Local environment of inserted or substituted residues in orthologous proteins. The count of total H-bonds and hydrophobic contacts as represented in LigPlot+ results for orthologous genes of *S*. *tuberosum* and *S*. *lycopersicum*.

Types of changes	Orthologous Proteins	Hydrophobic contacts	Hydrogen bonds
**Substitutions**	PGSC0003DMT400026140/Solyc02g050240.2.1	7/3	3/2
	PGSC0003DMT400034983/ Solyc03g095680.1.1	3/0	0/0
	PGSC0003DMT400049939/ Solyc06g010200.2.1	2/2	2/4
	PGSC0003DMT400053786/ Solyc01g057550.1.1	6/6	2/0
	PGSC0003DMT400070945/ Solyc06g006040.1.1	4/6	2/2
**Insertion in *S*. *tuberosum***	PGSC0003DMT400000956	10	0
	PGSC0003DMT400021686	5	2
	PGSC0003DMT400021824	2	0
	PGSC0003DMT400039198	2	1
	PGSC0003DMT400039552	2	1
	PGSC0003DMT400049812	3	2
	PGSC0003DMT400053083	6	1
	PGSC0003DMT400053786	6	0
	PGSC0003DMT400056487	3	0
	PGSC0003DMT400064637	2	1
	PGSC0003DMT400074273	3	1
	PGSC0003DMT400074709	2	3
	PGSC0003DMT400078131	3	0
	PGSC0003DMT400078833	0	1
	PGSC0003DMT400077130	1	0
**Insertion in *S*. *lycopersicum***	Solyc00g025650.1.1	5	1
	Solyc09g074850.2.1	2	2
	Solyc09g074850.2.1	3	0

### Repetitive regions influence the distribution of regulatory spots across the genome

**Fig 6:**
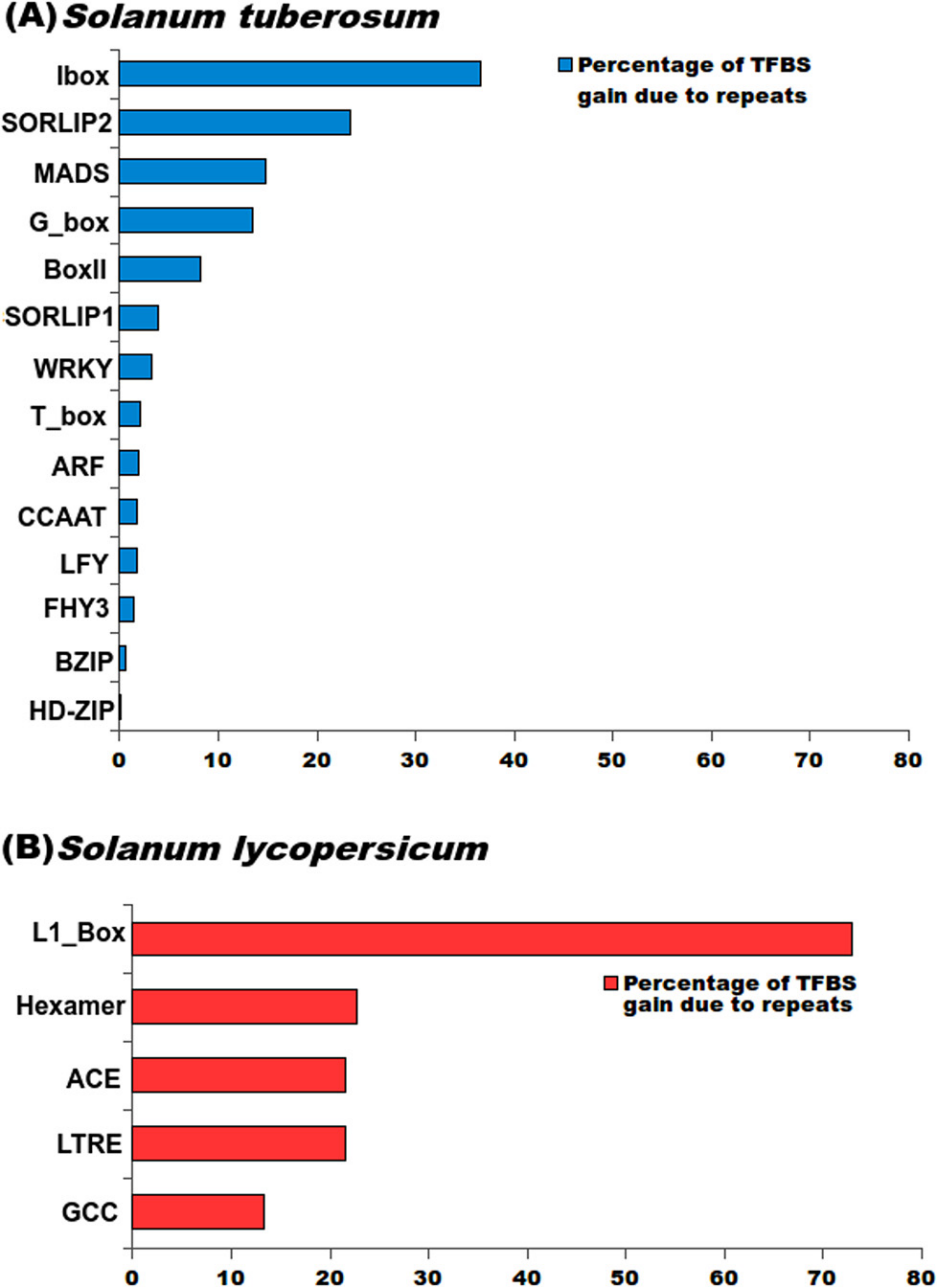
Gain/loss of Transcription factor binding sites (TFBS) in the upstream regions of orthologous genes in *S*. *tuberosum* and *S*. *lycopersicum*. Plot showing the percentage of TFBS gained in orthologous genes contributed by repetitive elements.

Compared to the influence of repetitive elements over structural variations in genes, some previous studies have given enough reasons for speciation through regulatory variability caused by the repetitive elements. The contribution of repetitive elements has been acknowledged widely for gene regulations by exaptation of various *cis*-regulatory elements, enhancers and silencers [103,104]. Evolution of *Brassica* species has been associated with regulatory evolution carried out through TEs like MITE elements [105]. Similarly, the evolution of sunflower has also been attributed to transposable elements like LTR elements [103]. In *P*. *abies*, the large genome size has been attributed to slow accumulation transposable elements [106], while in olive genome, accumulation of tandem repeats has influenced its genome size [107]. In mammalian and primate genomes, several studies have reported about some major roles being played by the repetitive elements in the distribution and evolution of regulatory sites [50,51,108,109]. Therefore, it becomes imperative to assess the possible regulatory impacts of the repetitive elements over the *Solanum* genomes, especially when it is found that >95% genes of these two *Solanum* species are associated with the repetitive elements. The TFBS gained/lost in the 2kb upstream regions of orthologous genes and present within the repetitive elements were identified. Probability of gain/loss of TFBS for every TF in every orthologous gene pair was elucidated using binomial test. In this analysis, null hypotheses assumed was that there was no significant gain/loss of TFBS in the 2kb upstream region of orthologous genes due to repetitive element. From this analysis, only those TFBS which showed significant p-value (≤ 0.05) for gain/loss of TFBS while being present within repeat sequences were retained, rejecting the null hypothesis. In *S*. *tuberosum*, it was found that of the total binding sites of I-box gained/lost in the 2kb upstream region of the genes, ~36% were found overlapping with repetitive elements (Fig 6). The I-box promoter motif has been found to be present in the upstream region of genes involved in light based responses. I-box has been found associated with tomato genes and classified as a member of Myb-group of transcription factors. Similarly, another transcription factor (TF), SORLIP2 (Sequences Over-Represented in Light-Induced Promoters (SORLIPs)), was found to have a significant gain of their TFBS in the orthologous genes of *S*. *tuberosum* with ~23% of the gained sites occurring within the repetitive regions (Fig 6). This transcription factor has been associated with light-induced genes in cotyledon and roots of plants including *A*. *thaliana*. Another TF G-Box, which has been found involved in the regulation of expression of genes in response to light, anaerobic stress, abscissic acid and other metabolites. It was identified that 13% of the gained sites of G-box were within the repeat overlapping regions. Also, MADS family of transcription factors had ~14% of the TFBS gained in *S*. *tuberosum*. MADS TF possesses the MADS domain and these transcription factors have been associated critically with all sorts of development processes in plants, including flower development and gametophyte, embryo and seed development.

In *S*. *lycopersicum*, gain of sites for five transcription factor families (L1-box, LTRE, Hexamer, GCC box and ACE) occurred in the repeat overlapping regions (Fig 6). GCC box binding factors have been shown to play significant roles in response to different secondary metabolites like jasmonate, which is involved in the activation of several pathogen responsive genes. GCC box also acts as an ethylene responsive element, which regulates some defense responsive genes. GCC box has also been found to be the binding site for PTi transcription factor which regulates the expression of defense related genes. In this study, ~12% of the gained sites of GCC in *S*. *lycopersicum* have been found within repeat-overlapping regions, showing that many defense responses are under indirect regulations by repetitive elements. Another transcription factor, whose binding sites were found overlapping with repetitive element was ACE (ACGT containing element). It was shown to have about 21% of the gained sites within the repeat-overlapping regions. ACE motifs are also associated with the light-responsive genes and anthocyanin biosynthetic genes. This shows another important plant specific functions being indirectly regulated by repetitive elements. LTRE is a low temperature response element found specifically in genes responsive to low temperature in plant species including *A*. *thaliana* and Barley, and it also had a significant number of gained sites overlapping with repetitive elements (~21%). Hexamer promoter element also seems to have been distributed by repetitive elements as ~22% of the gained hexamer sites were found within the repeat-overlapping regions. Hexamer promoter elements are reported to regulate histone H3 and H4 in different plant species including *A*. *thaliana* and Maize, where they were found regulating the expression of genes in meristems. The hexamer motif was seen to function in alliance with nonamer motifs. The case of L1 box response element requires exclusive mentioning, as the majority of gain of these sites could be attributed to the repetitive elements (~70%) (Fig 6). L1 box promoters are involved in L1-layer specific expression of genes, and it contains a L1-binding homeodomain and Myb binding motif. L1 layer corresponds to the outermost layer in a shoot apical meristem and is responsible for its growth. L1 box was identified to be 8 bp long *cis*-regulatory element essential for the expression of L1-layer specific genes. Other transcription factors whose TFBS have been gained in *S*. *tuberosum* and *S*. *lycopersicum* are described in Table 5 and S7 Table. All the transcription factors have been discussed elsewhere in good details [110].

**Table 5:** Gain of transcription factor binding sites due to repetitive elements in the promoter regions of orthologous genes.

***Solanum tuberosum***			
**TF name**	**Gain in repeat overlapping region**	**Gain in Total 2kb region**	**Percentage of TFBS gain due to repeats**
ARF	11	574	1.92
BoxII	102	1,245	8.19
BZIP	1,369	265,990	0.51
CCAAT	437,839	25,186,369	1.74
FHY3	45,132	3,328,200	1.36
G_box	12	89	13.48
HD-ZIP	1,519	3,453,104	0.04
Ibox	49	134	36.57
LFY	386	22,590	1.71
MADS	2,952	19,959	14.79
SORLIP1	6	154	3.90
SORLIP2	580	2,489	23.30
T_box	153	7,237	2.11
WRKY	458	14,375	3.19
***Solanum lycopersicum***			
**TF name**	**Gain in Repeat overlapping region**	**Gain in Total 2kb region**	**Percentage of TFBS gain due to repeats**
ACE	45	217	20.74
GCC	25	195	12.82
Hexamer	484	2,221	21.79
L1_box	35,998	51,380	70.06
LTRE	194	936	20.73

All transcription factors mentioned above were found involved in major metabolic pathways, defense response, regulating specifically the genes showing response to light-induced stimuli and normal plant growth and development. This displays the extent to which many repetitive elements have been domesticated by the plants for their own survival purpose, contradicting the tags like “genomic parasites” or “junks” given to the repetitive elements initially.

### Repetitive elements in miRNA genesis

**Fig 7:**
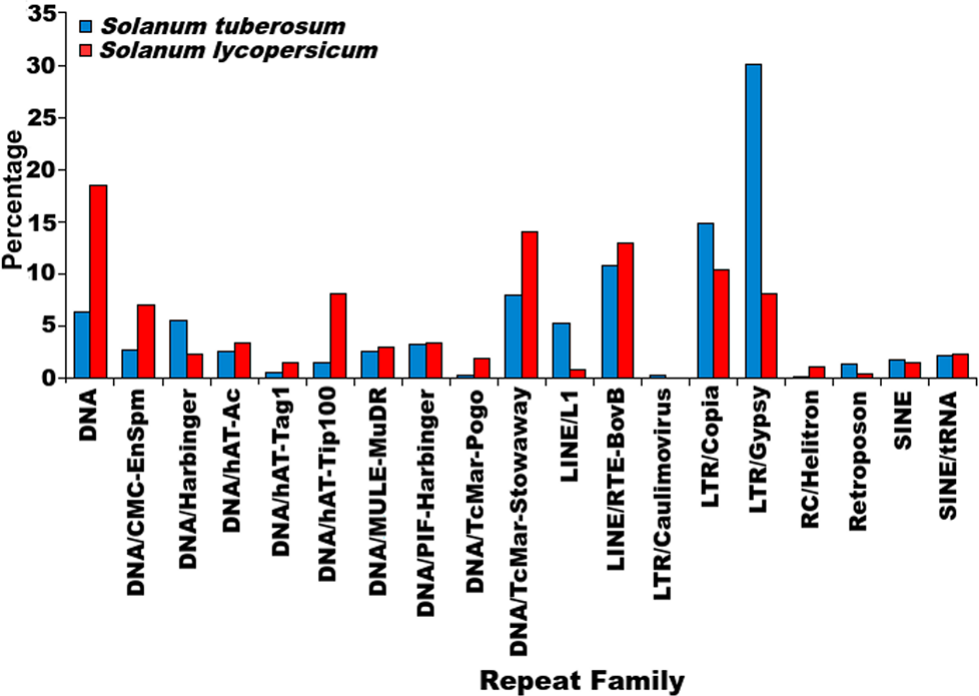
Repeat families most prevalent in miRNAs in *S*. *tuberosum* and *S*. *lycopersicum*. DNA transposons, LTR elements and LINE elements were observed as the most common in the miRNAs of both species.

miRNAs are a class of small non-coding RNAs with ~21–25 nucleotides in length [111–113]. These small RNA species have received enormous attention due to the regulatory roles played by them through post transcriptional gene silencing as well as RNA directed DNA methylation (RdDM) [68,114–118]. miRNAs have been shown to regulate ~60–70% of genes in an organism while displaying broad range of target interactions as well the modes of their biogenesis [112,114,119,120]. Due to such important implications posed by miRNAs in different biological processes and disease conditions, miRNAs have gained considerable importance. Many new miRNAs and their expressions have been studied with regard to many different diseases, where their roles in regulating these processes have been strengthened. miRNA sequences identified in different organisms have grown exponentially over the years [121]. However, the process of miRNA evolution and biogenesis is still intriguing and suggestive of multiple sources. In recent times, some dedicated studies by certain groups has helped a lot to identify the repetitive elements origin of miRNAs, more so with plants [122–125]. The biogenesis of miRNAs from transposable elements was first proposed by Smallheiser and Torvik in 2005 [123], but this did not get due attention until it was also observed by many other authors [124]. Since then multiple hypotheses have been proposed for the evolution of miRNAs from transposable elements [124]. Approximately half of the human genome and ~80% of several plant genomes are composed of transposable elements, making the origin of miRNAs from such elements more likely. Although many models for the origin of miRNAs from repetitive elements have been proposed, the one proposed by Smalheiser and Torvik remains the most highlighted one [126]. Plant miRNAs have been reported to be derived from different families of transposable elements. miRNAs like TamiR1123 was shown to be derived from MITE elements in wheat [70], which regulates the expression of a vernalization gene by influencing its promoter element. Similarly, many miRNAs in *O*. *sativa* and *A*. *thaliana* were also reported to be derived from different transposable elements [122]. The most common transposable elements have been associated with many conserved miRNAs include MITE (Miniature Inverted Transposable Elements) [70,122], LINE elements [123] and SINEs [125]. In the present study also, a close association between miRNAs and repetitive elements was observed. Thus, for identification of such transposable element-derived miRNAs, overlap between miRNAs and identified complex repetitive elements was assessed for the entire genome of both species. In *S*. *tuberosum*, 224 miRNA sequences were found across its genome. A total of 30 pre-miRNA sequences were found originating from multiple loci in the genome of *S*. *tuberosum* (S8 Table), displaying repetitiveness and suggesting a repetitive origin associated with them. All the multiple loci of these miRNAs were studied for the presence of repetitive elements and footprints in the 2kb upstream and downstream regions. Most of these multiple loci were observed to be overlapping with different repetitive elements. In *S*. *lycopersicum*, 77 pre-miRNA were found and all of them were found originating from single locus. The identified co-ordinates of pre-miRNA sequences were used to extract the 2kb upstream and downstream sequences in both species. For this range of 2kb upstream and downstream regions around the pre-miRNA sequences (~4kb), overlapping repeats were identified in both species. Considering all the multiple loci of miRNAs, 242 loci of miRNAs in *S*. *tuberosum* were identified to be overlapping with repetitive elements. Similarly, in *S*. *lycopersicum* 77 loci of miRNAs were identified as overlapping with repetitive elements. It was also identified that LTR/Gypsy was the most prevalent repeat family in *S*. *tuberosum* miRNAs while in *S*. *lycopersicum* DNA transposons were more prevalent (Fig 7). It was further found that same members of the multi-loci miRNAs were overlapping with different repeat families. This analysis led support to the previous reports that transposable elements might serve as precursors to enrich the miRNA repertoire in many plant species [122–126]. The probability of enrichment of miRNAs around repetitive elements was elucidated using binomial test. Significant p-values were obtained for *S*. *tuberosum* (4.136e-10) and *S*. *lycopersicum* (1.819e-12), suggesting that miRNAs were enriched around repetitive elements.

**Fig 8:**
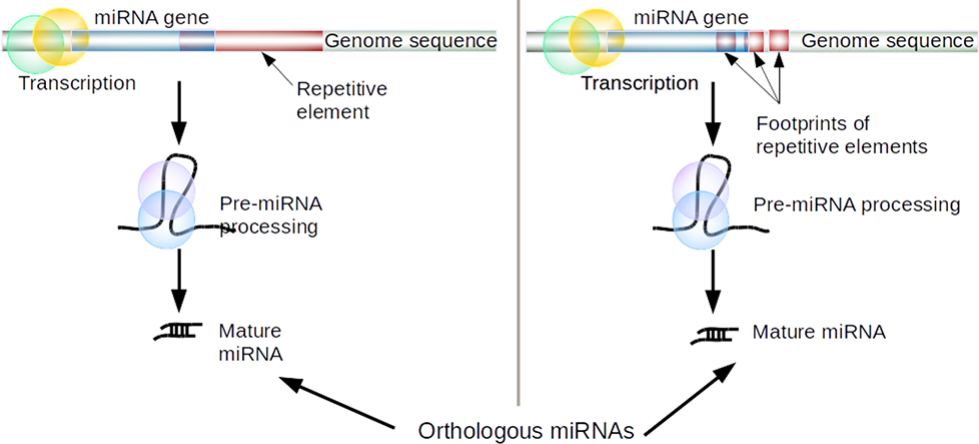
Evolution of miRNAs from repetitive elements. To search for candidate miRNAs which evolved from repetitive elements, footprints of repetitive elements around miRNA genes were identified.

During evolutionary course of an organism, repetitive elements may become unrecognizable as repeats due to different mutational events, sometimes leaving behind their footprints [70,71,127]. The above mentioned findings indicate that miRNA sequences might have taken birth from repetitive elements. Therefore, there could be a fare possibility that several miRNAs could have been contributed by complex repeats which gradually eroded with time and became unrecognizable into the genome. An attempt to discover such phenomenon through hunt for some sequential signatures in the flanking regions could support such view to some extent. The 2kb upstream and downstream regions including pre-miRNA sequences in both species were extracted from the genome. Orthologous pre-miRNAs were identified and local alignment between orthologous pre-miRNA pairs was performed. Common motifs in the orthologous sequences present in the same orientation and with same arrangement was found for some miRNAs, suggesting a repetitive origin for them despite of no clear presence of any full length or substantially long repetitive element around it. From the alignment, it was identified that in 14 orthologous miRNA pairs the selected motifs were present within repeat overlapping regions in both species. While in seven orthologous pairs, the motifs were found within the repeat overlapping region in *S*. *tuberosum* only, and in two orthologous miRNA pairs the motifs were found within the repeat overlapping region in *S*. *lycopersicum* only. Thus, these seven orthologous miRNAs in *S*. *lycopersicum* and two orthologous miRNAs in *S*. *tuberosum* might be have been generated through some transposable elements (Fig 8). In this study, it was also identified that the motifs which were found in the resulting orthologous genes, were originating from complex repetitive elements including LTR elements Copia and Gypsy, SINE/tRNA, DNA transposons TcMar-Stowaway, RTE-BovB, hAT-AC and CMC/EnSpm. However, the contribution of DNA transposons was observed to be more than retrotransposons.

### Transcriptional activity of repetitive elements

**Fig 9:**
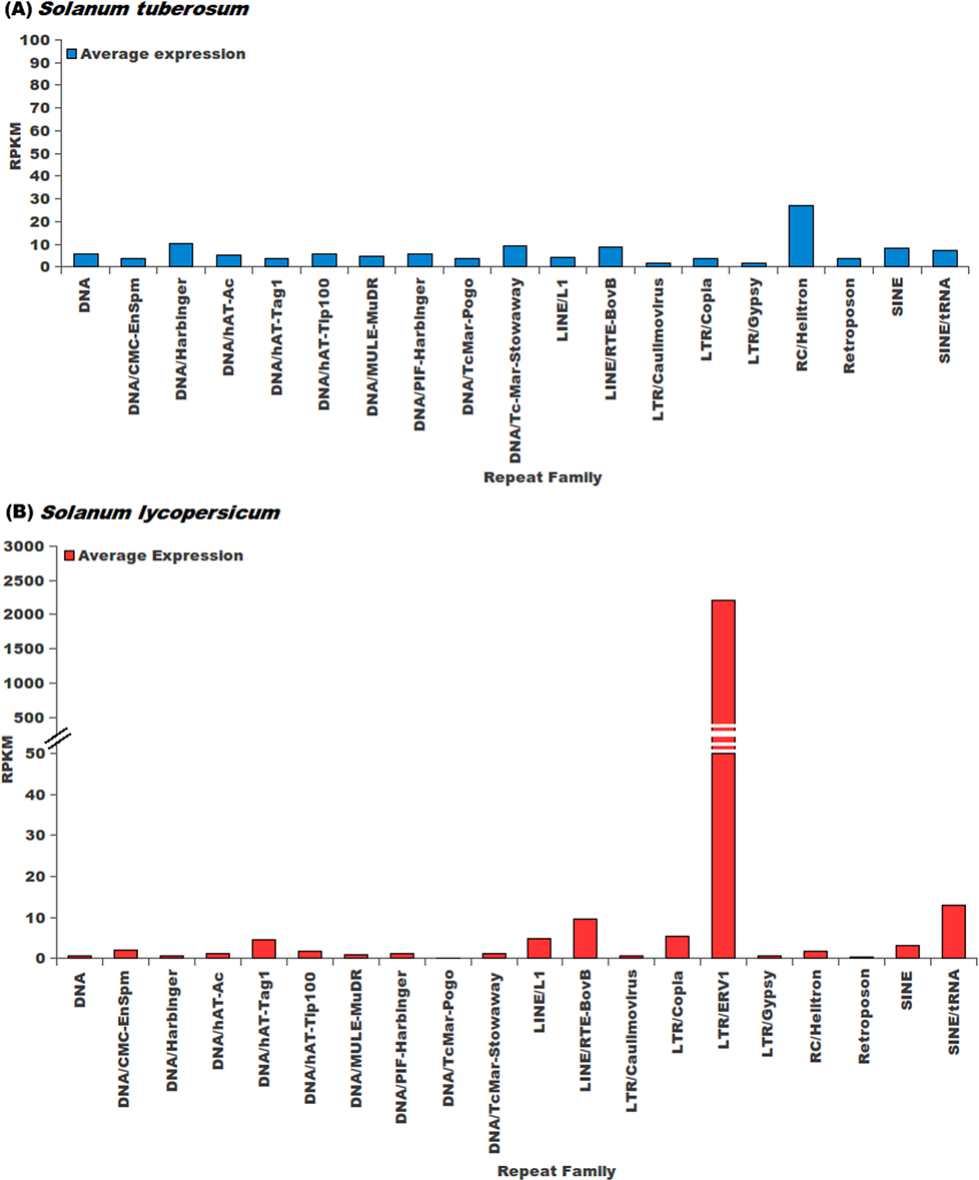
Transcriptionally most active repeat families on the basis of average RPKM expression. RC/Helitron and LTR/ERV1 were the transcriptionally most active repeat super-families in *S*. *tuberosum* and *S*. *lycopersicum*, respectively.

Repetitive elements are generally under high constraints and characterized by high DNA methylation making them silent components of the genome. Although transcriptional activity of repetitive elements has been observed under stress conditions, pathogen attack and tissue culture conditions [128,129]. Also a low level of activity for different repetitive elements has been reported in normal conditions which is one of the reasons for their amplifications in a genome. Many plant species, specially flowering plants, have been shown to possess active repetitive elements belonging to both classes of transposable elements [130,131]. Active nature of repetitive elements has been associated with the generation of small non-coding RNA (siRNAs) which through post transcriptional gene silencing mechanisms (PTGS) create a feed-back loop silencing the repetitive elements themselves [132]. Other than providing control to repetitive elements, transcriptional activity of repetitive elements may also provide tissue specific expression of certain genes [23]. These elements upon transcription can also alter the expression of certain genes by RNA interference or through antisense RNA as well as through different epigenetic modifications. Therefore, identification of active repetitive elements in these two genomes would help in defining the functional boundaries generated by these elements with regard to their host genes' expression patterns. Abundance of transcript sequences and repetitive elements was calculated using digital expression data from two different platforms. Using sequence read count based RPKM abundance measure, it was found that RC/Helitron was transcriptionally most active repeat super-family in *S*. *tuberosum* (Fig 9). Helitrons were initially discovered in *A*. *thaliana*, *C*. *elegans* and *O*. *sativa* using different *in-silico* methods [133,134] and since then, they have been identified in numerous eukaryotes. Helitrons transpose by rolling circle transposition rather than by traditional “cut and paste” mechanism as is followed by other DNA transposons [134,135]. Although, helitrons make only a small portion of genomes of eukaryotes, they have been known to contribute significantly to the evolution of genes by capturing exons as has been demonstrated in maize [135]. The other transcriptionally active repeat super-families in *S*. *tuberosum* on the basis of RPKM abundances include DNA transposon Harbinger and TcMar-Stowaway (Fig 9). TcMar-Stowaway was also transcriptionally most active repeat super-families identified using microarray data. DNA transposon Harbinger was the first super-family of DNA transposons to be identified in *A*. *thaliana* using *in-silico* analysis which was identified as the most transcriptionally active repetitive element on the basis of RPKM and microarray expression data (S1 Fig) [136]. A few of Harbinger elements have been reported to be active members of their respective genomes [137]. DNA transposons TcMar-Stowaway were first discovered in *S*. *bicolor* as the elements inserted within *Tourist* elements [89]. Stowaway elements can form a hairpin shaped structure [89] and have shown to be able to generate miRNAs [138]. DNA transposon TcMar-Pogo was another repeat super-family which was identified as highly transcriptionally active in *S*. *tuberosum* on the basis of microarray data (S1 Fig). Pogo super-family of repeats was first identified in *Drosophila* and since then has been identified in many other species [139,140]. Pogo elements have been associated with exaptation of the CENP-B gene in mammals [141] and of some MITE elements in *A*. *thaliana* [142]. SINE elements activity was also evident through *S*. *tuberosum* microarray data. However, in *S*. *lycopersicum*, a repeat super-family very similar to LTR/ERV1 repeat super-family was found as the transcriptionally most active repeat family in the RPKM based expression estimates (Fig 9). The other transcriptionally active repeat super-families according to NGS expression measures observed in *S*. *lycopersicum* belonged to SINEs and LINE element RTE-BovB, which was also observed to be transcriptionally active in microarray data (Fig 9). Other highly transcriptionally active repeat super-families in the NGS expression measures included DNA transposons CMC-EnSpm. Information regarding the annotation of repeat super-families and the method of annotation is provided in S9 Table.

**Fig 10:**
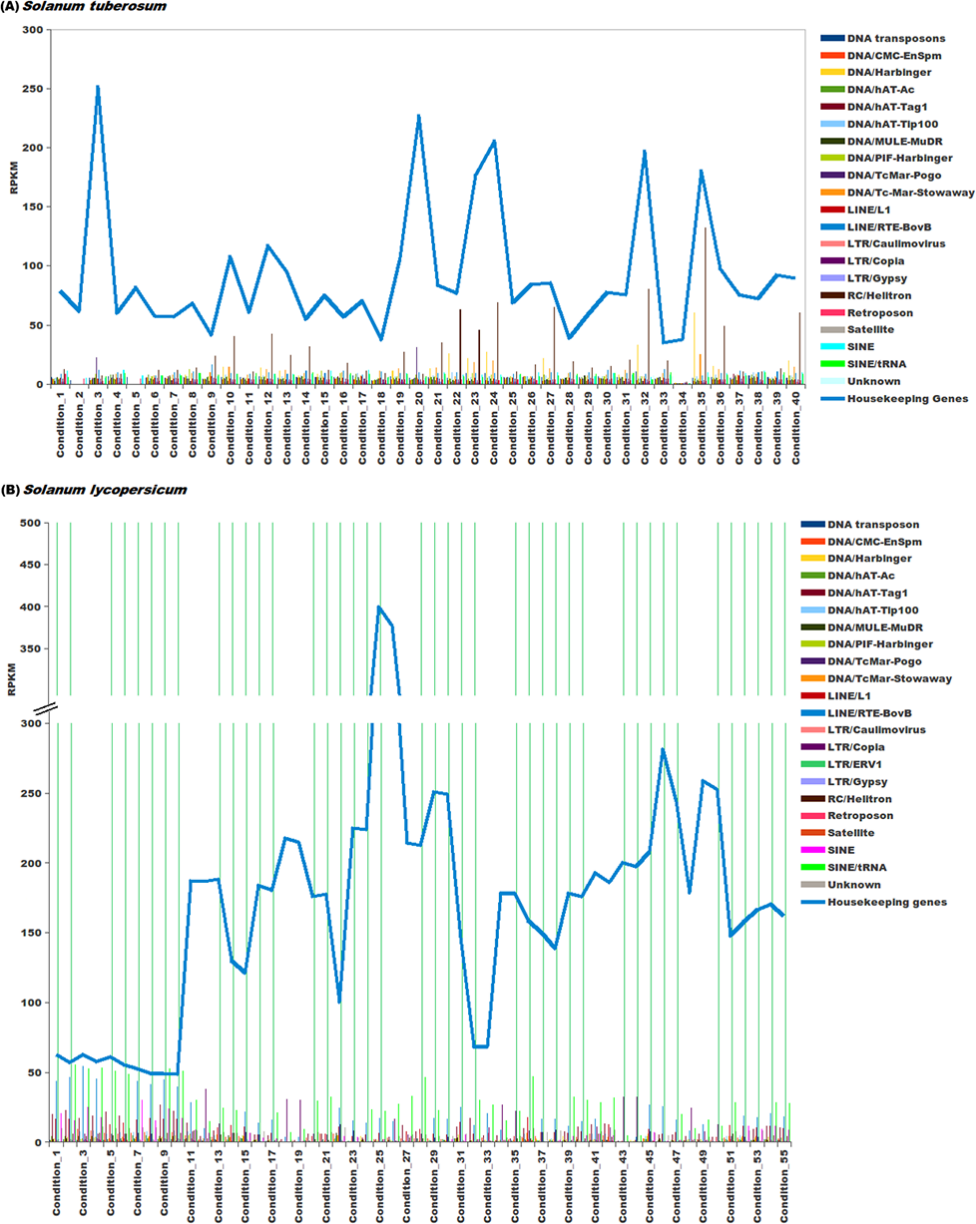
Comparison of average RPKM expression of repeat families and housekeeping genes. The details about experimental conditions is given in S11 Table.

Further, expression of repetitive elements was also compared with the expression of housekeeping genes to obtain the view about their relative abundance. It was observed that though several repetitive elements were active in the system and expressing themselves, their relative abundance with respect to the housekeeping genes was found much lower, with exception of LTR/ERV1 (Fig 10). Being an exonic part of transcribing genes makes such repeats to be detected easily as an expressing element. Abundance of repetitive element transcripts found within exons and introns was also calculated. This analysis showed that the repetitive elements found within exonic regions were having higher abundance in both the species (S2 Fig).

An interesting finding of this study has been observation for two novel repeat super-families, LTR/ERV1 and LINE/RTE-BovB, reported first time for any plant species, and therefore, deserve special mention. RTE-BovB was first discovered in reptiles and it was shown to be horizontally transmitted from reptiles to ruminants and marsupials [143,144]. Super-families very similar to RTE-BovB were identified in *S*. *tuberosum* and *S*. *lycopersicum*, although RTE-BovB has not been reported in plants so far. Interestingly, ERV1 was also not reported in any plant species, previously. LTR/ERV1 are the endogenous retroviral elements and their active nature has been observed previously in mouse [77,145] and they have also been found enriched in human linc RNAs [146]. The members for this repeat family were identified here primarily on the basis of the identification of conserved protein domains, sparse sequence similarity and conservation of certain signature spots. Most of the LINE/RTE-BovB elements exhibited >90% length coverage with the consensus given in Repbase. The information regarding the species with known LTR/ERV1 and LINE/RTE-BovB families is provided in S10 Table. Further, multiple sequence alignment (MSA) of the consensus sequences of identified LINE/RTE-BovB elements and LTR/ERV1 elements was performed with known LINE/RTE-BovB and LTR/ERV1 elements for various species. Phylogenetic trees were drawn using Neighbor Joining method with a bootstrap value of 1000. It was observed that in both the species, the identified families of LINE/RTE-BovB emerged as an outgroup compared to the rest of species, an expected result (S3 Fig LINE/RTE-BovB in *S*. *tuberosum* and *S*. *lycopersicum* and S4 Fig for LTR/ERV1 in *S*. *lycopersicum*). In the MSA, the central regions in the alignments of LINE/RTE-BovB and LTR/ERV1 sequences were observed as characteristically the most conserved ones. These regions are known to harbor the important genes specially the ORFs encoding endonuclease and reverse transcriptase genes in LINE/RTE-BovB elements, while *gag*, *pol* and *env* for LTR/ERV1 elements. It would be difficult to annotate such elements just based on traditional sequence similarity search against Repbase alone, as the overall similarity varied a lot (46% to 85% for LINE/RTE-BovB and 6% to 62% for LTR/ERV1 when considering indels while when considering only substitutions, similarity ranged from ~50% to 92% for LINE/RTE-BovB and ~33% to 90% for LTR/ERV1). For the LTR/ERV1 family identified in this study, the similarity was observed within range for known species (~21% when considering indels, and 71.97% when only substitutions were considered). The LINE/RTE-BovB showed similarity ranges upto from 40–98% (~82–98% similarity when substitution was considered only) for *S*. *tuberosum* while 71–95% (71–94% approximately, when substitution was considered only) similarity range for *S*. *lycopersicum* when compared with the consensus. However, as apparent from the MSA and structural domains study, certain spots and regions of this family exhibited high conservation across all families in a very characteristic manner (S5 Fig for LINE/RTE-BovB elements in *S*. *tuberosum* and *S*. *lycopersicum* and S6 Fig for LTR/ERV1 in *S*. *lycopersicum*).

**Fig 11:**
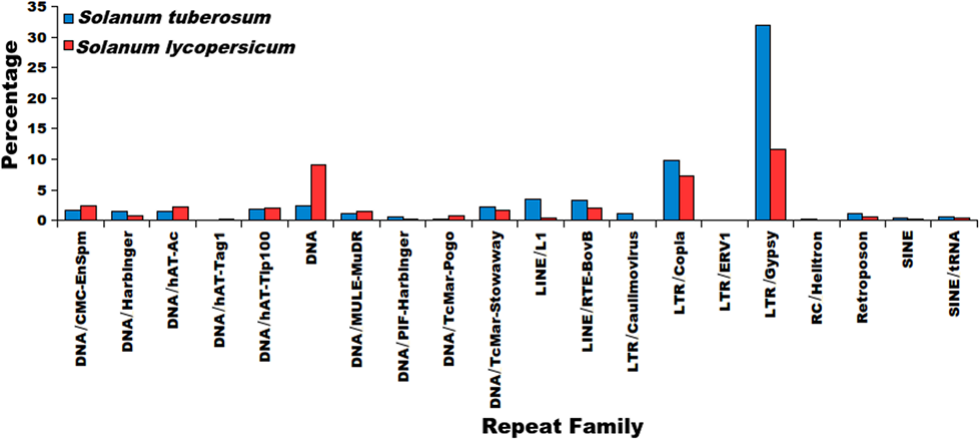
Percentage of sRNA reads mapping to different repeat families. It was observed that most of the sRNAs were originating from LTR elements Gypsy and Copia in both species.

**Fig 12:**
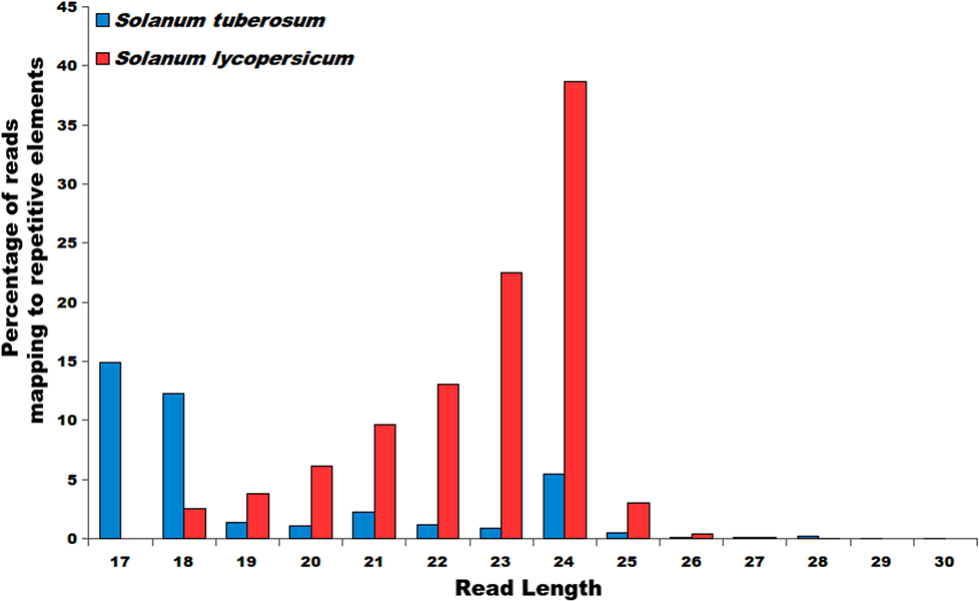
Length distribution plot of the sRNA reads mapping to repetitive elements. sRNA reads of length 24 bp were observed to be most enriched in *S*. *lycopersicum*, while in *S*. *tuberosum*, 17 bp long sRNAs were more prevalent.

**Fig 13:**
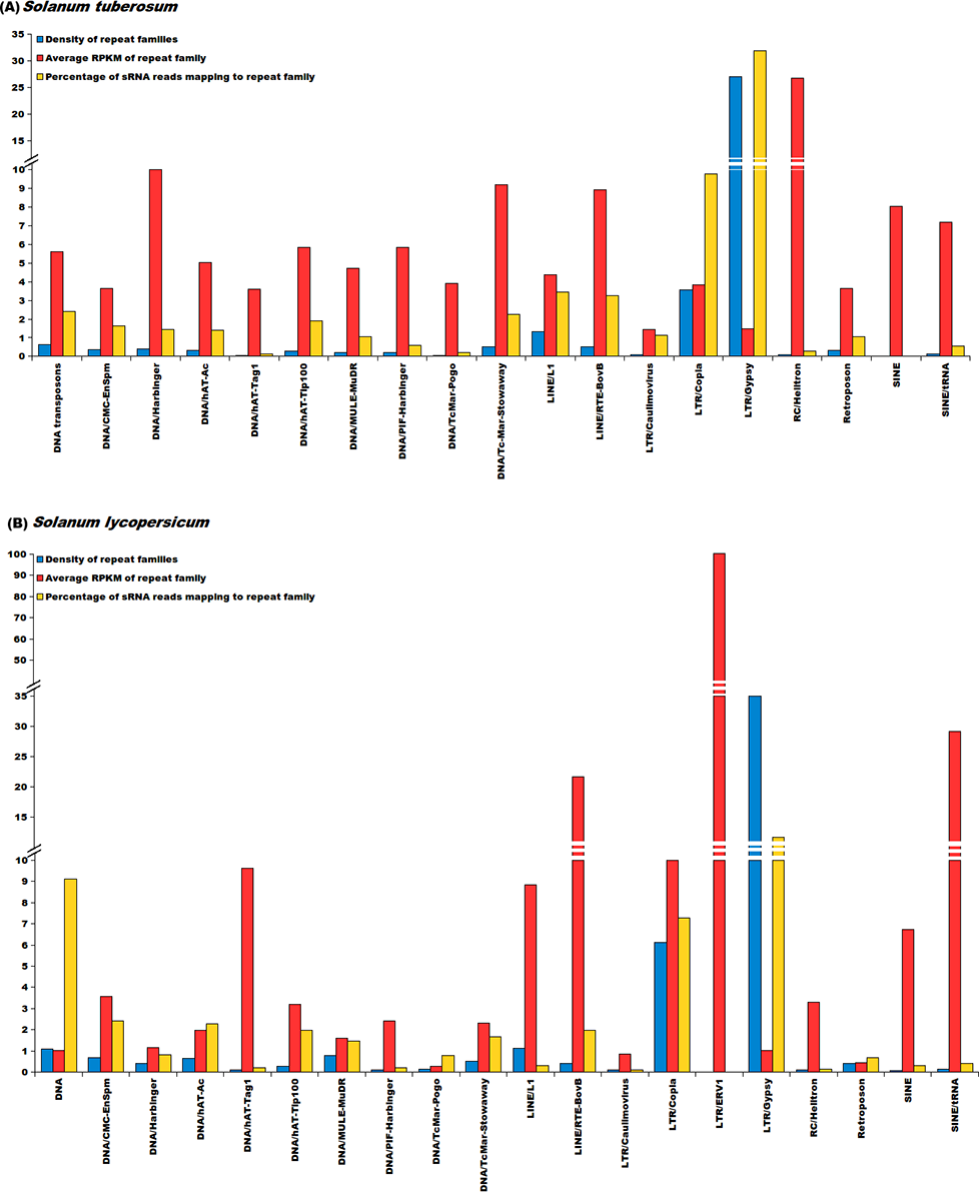
Comparative plots showing coverage of repeat families, average expression of repeat families and percentage of small RNA reads mapping to repeat families in *S*. *tuberosum* and *S*. *lycopersicum*. The plot shows that density of repetitive elements is not determining the abundance of repeat super-families and the generation of sRNAs from repeat super-families.

A large number of expressing repetitive elements have been reported to be involved in small RNAs/siRNA biogenesis [147,148]. Several of such sRNAs regulate the genes in either *cis* or *trans* manner. While some sRNAs are involved in post transcriptional gene silencing [148], a good number of such small RNAs are involved in *de novo* DNA methylation in plant genome [147,149–151]. Measuring the abundance of such small RNAs could also mirror the expression of repetitive elements which could have regulatory roles in the system. The small RNA sequencing reads were mapped to the repetitive elements as mentioned in the Methods section. Out of 35,992,757 unique small RNA reads in *S*. *tuberosum* and 9,620,265 unique reads in *S*. *lycopersicum*, ~0.23% (85,013) and ~33.41% (3,214,301) unique reads mapped to different repetitive elements, respectively. It was also found that most of the sRNA reads mapped to LTR/Gypsy elements in both species (Fig 11). To further verify the nature of these small RNAs, length distribution plot of the small RNA reads was made and it was identified that a high percentage of sRNA reads in *S*. *tuberosum* and *S*. *lycopersicum* were around 24bp, a length most prevalent with small regulatory RNAs like endogenous siRNAs and miRNAs (Fig 12). The overall comparison between the coverage of repeat family, the average abundance of repeat families and the percentage of sRNA reads mapped has been illustrated in Fig 13. It appears that expression of a repeat family is not correlated to its genomic prevalence.

The sRNAs were found closely associated with repetitive elements of these species, concordant with some recent reports [152–154] that there is a big stake of repetitive elements in sRNA biogenesis, which in turn are now considered core members of post transcriptional as well as RdDM based transcriptional gene regulatory processes. It opens a door for further studies with repetitive elements and their impact over *Solanum* gene regulatory system.

## Supporting Information

S1 Fig Transcriptionally most active repeat families on the basis of average microarray expression. (TIFF)

S2 Fig Transcriptionally most active repeat families on the basis of average RPKM for exonic and intronic repetitive elements. (TIFF)

S3 Fig Phylogenetic tree for the consensus repeat family sequences of LINE/RTE-BovB identified in *S*. *tuberosum*, *S*. *lycopersicum* and known LINE/RTE-BovB families. The LINE/RTE-BovB super-families identified in *S*. *tuberosum* are mentioned with the prefix “Stu”, while those identified in *S*. *lycopersicum* are mentioned with the prefix “Sly”.
(TIFF)

S4 Fig Phylogenetic tree drawn for the consensus repeat family sequences of LTR/ERV1 identified in *S*. *lycopersicum*, *S*. *tuberosum* and known LTR/ERV1 families. The consensus sequence of LTR/ERV1 identified in this study was matched with known consensus sequences of LTR/ERV1 and phylogenetic tree was created using Neighbor joining method with a bootstrap value of 1000. The LTR/ERV1 family identified in this study was named as “rnd-6_family-7426-LTR-ERV1”.
(TIFF)

S5 Fig Multiple sequence alignments for sequences of LINE/RTE-BovB repeats identified in *S*. *tuberosum*, *S*. *lycopersicum* and known LINE/RTE-BovB families. The LINE/RTE-BovB super-families identified in *S*. *tuberosum* are mentioned with the prefix “Stu”, while those identified in *S*. *lycopersicum* are mentioned with the prefix “Sly”.
(PDF)

S6 Fig Multiple sequence alignments for repeat family sequences of LTR/ERV1 families identified in *S*. *lycopersicum* and known LTR/ERV1 families. The consensus sequence of LTR/ERV1 identified in this study was matched with known consensus sequences of LTR/ERV1 and all the sequences matching with LTR/ERV1 were used for MSA.
(PDF)

S1 Table Consensus repeat family sequences identified. (DOC)

S2 Table Characterization of the remaining “Uncharacterized” consensus repeat family sequences. Characterization of uncharacterized elements in ncRNA, pseudo-genes and SINE elements identified by matching A-box motif, B-box motif, 5' and 3' conserved motifs of *B*. *oleraceae* SINE elements.
(XLS)

S3 Table Similar repetitive elements identified in *S*. *tuberosum* and *S*. *lycopersicum* reported in the presented study and those reported by PGSC_DM_v4.03 and ITAG2.3, respectively. (XLS)

S4 Table Chromosome wise coverage of all repeat families. Coverage of different repeat super-families was calculated as the percentage of nucleotides represented by repetitive elements out of total nucleotides for the given chromosome.
(DOC)

S5 Table List of orthologous genes of *S*. *tuberosum* and *S*. *lycopersicum*, identified using BLASTP. The best matches in BLAST result for every protein were considered as orthologs.
(XLS)

S6 Table Amino acid residues of orthologous genes showing changes in secondary and tertiary structures due to repetitive elements. (XLS)

S7 Table Transcription factor binding sites gained / lost in orthologous genes due to presence of repetitive elements in the genic regions. (XLS)

S8 Table *S*. *tuberosum* miRNAs mapping to multiple positions in the genome. (XLS)

S9 Table Annotation of repeat families. (XLS)

S10 Table Description of the different species which harbor LTR/ERV1 and LINE/RTE-BovB elements in their genomes. The list of species was prepared using RepBase.
(XLS)

S11 Table Description of the different experimental conditions displayed in Fig 10. (XLS)
