# Supplementary figures and images for: A Deluge of Complex Repeats: The Solanum Genome
Source: PLoS One. 2015 Aug 4;10(8):e0133962. doi: 10.1371/journal.pone.0133962 (PMC4524691; doi:10.1371/journal.pone.0133962)

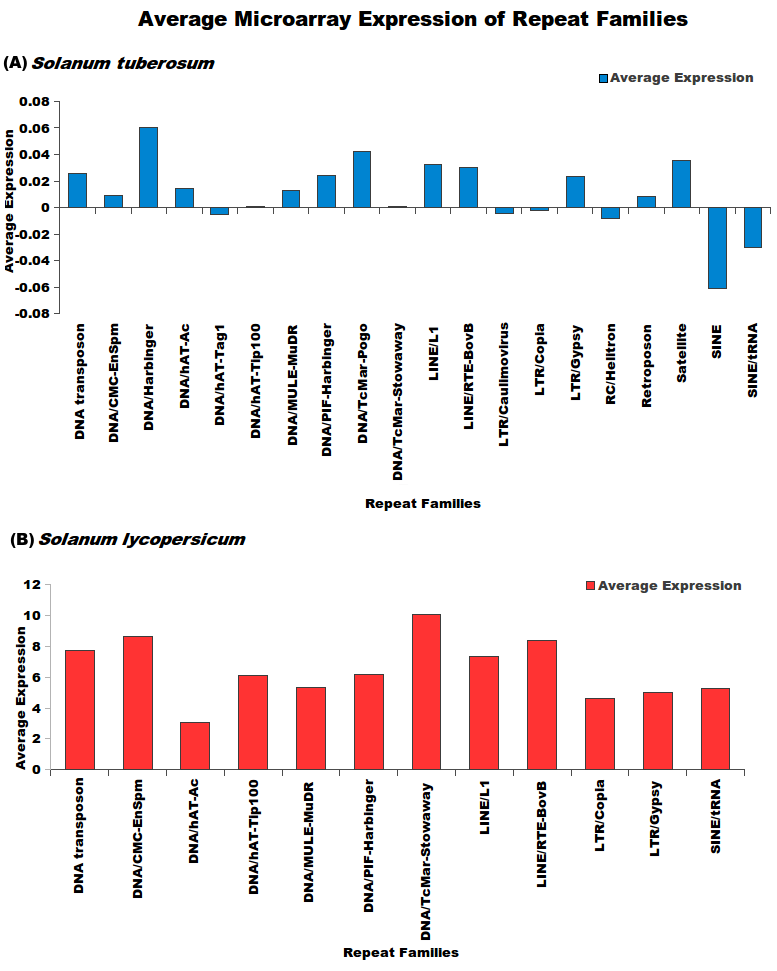

Supplement: S1 Fig — (TIFF) [file pone.0133962.s001.tiff]

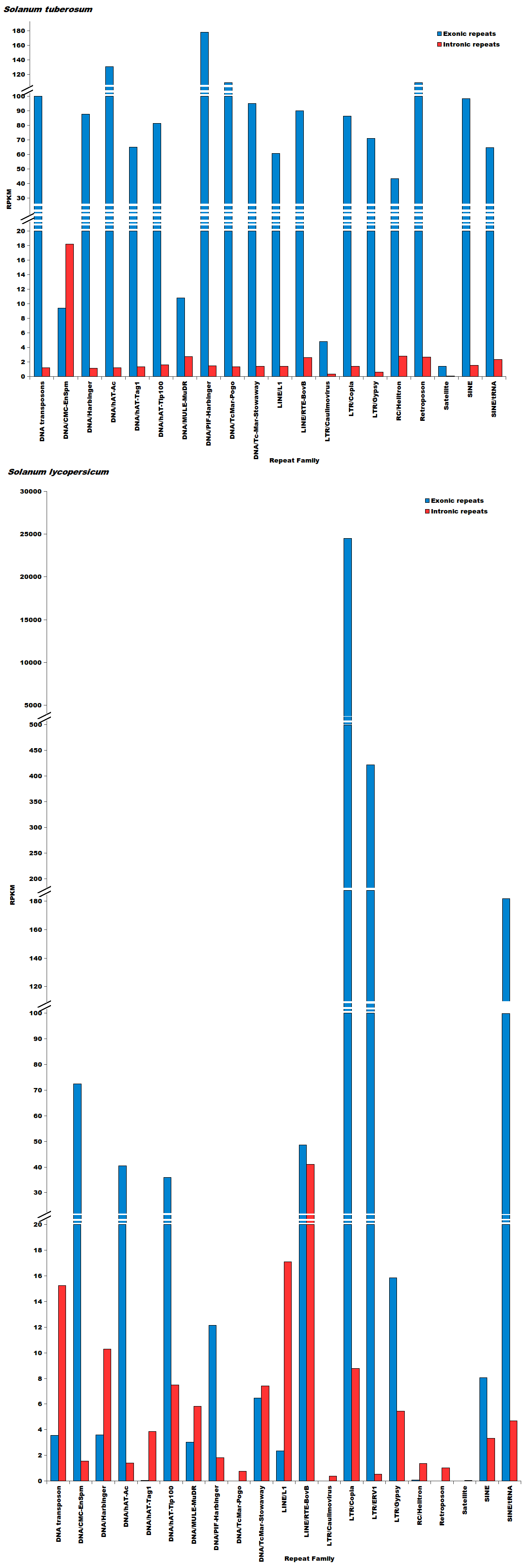

Supplement: S2 Fig — (TIFF) [file pone.0133962.s002.tiff]

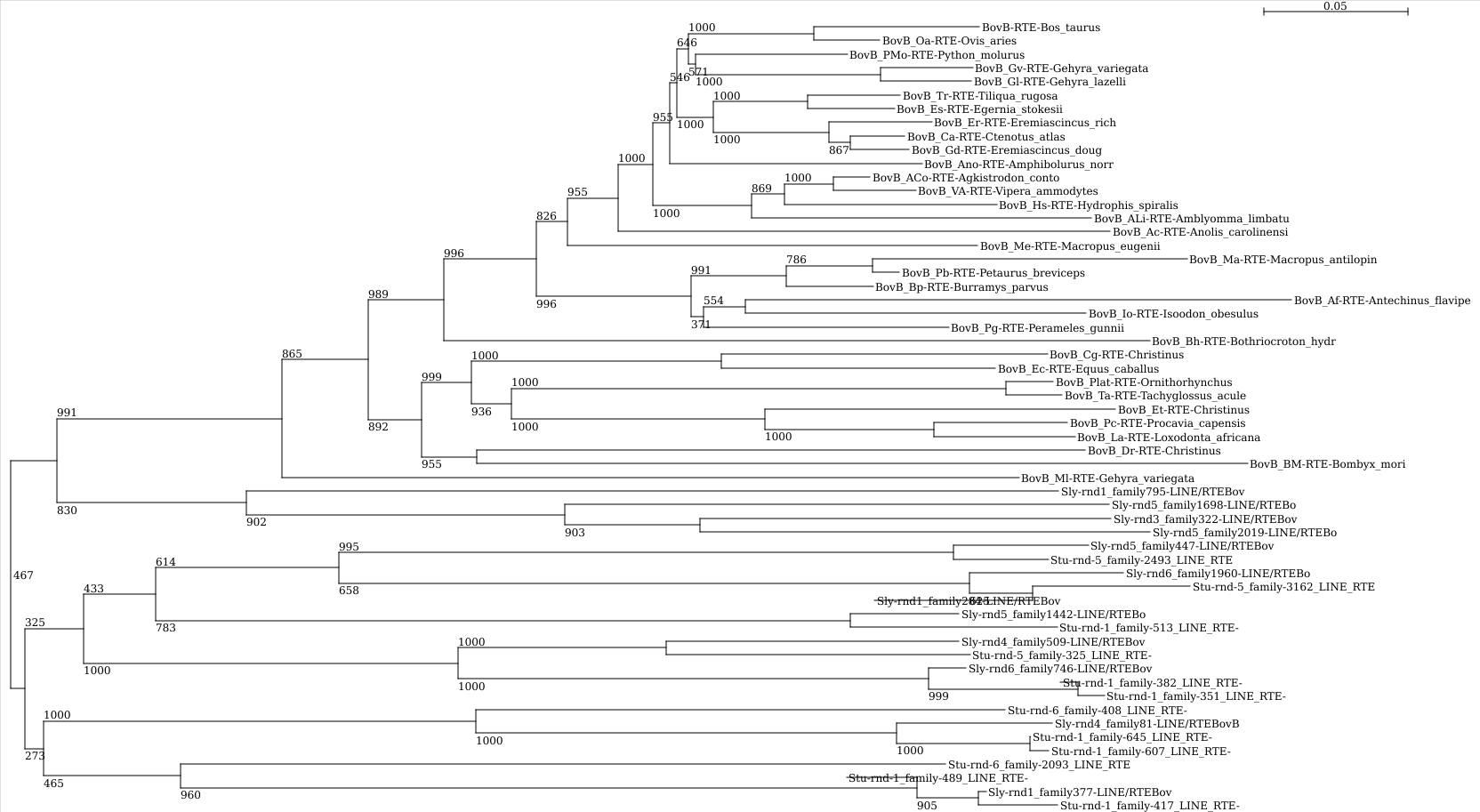

Supplement: S3 Fig — The LINE/RTE-BovB super-families identified in S. tuberosum are mentioned with the prefix “Stu”, while those identified in S. lycopersicum are mentioned with the prefix “Sly”. (TIFF) [file pone.0133962.s003.tiff]

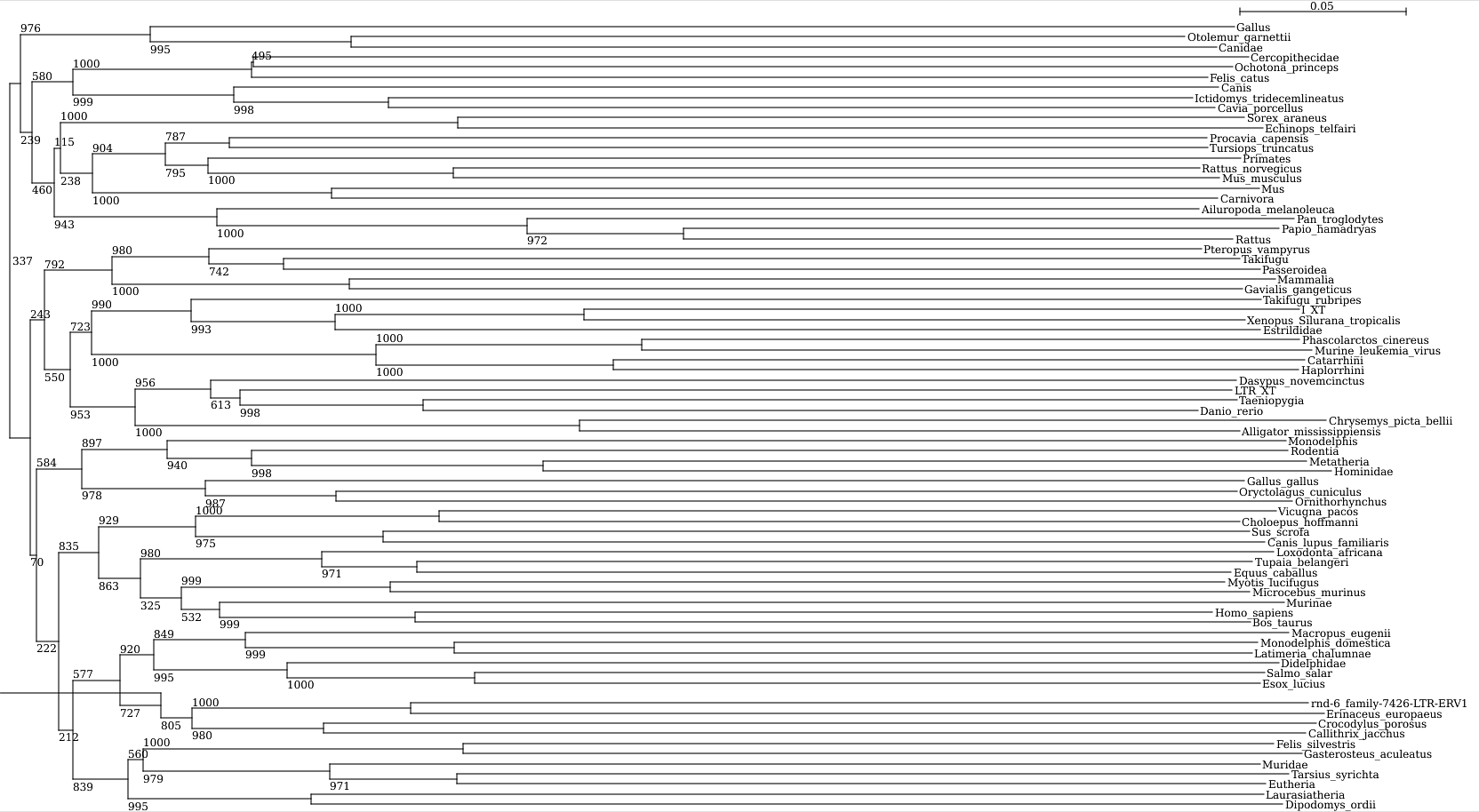

Supplement: S4 Fig — The consensus sequence of LTR/ERV1 identified in this study was matched with known consensus sequences of LTR/ERV1 and phylogenetic tree was created using Neighbor joining method with a bootstrap value of 1000. The LTR/ERV1 family identified in this study was named as “rnd-6_family-7426-LTR-ERV1”. (TIFF) [file pone.0133962.s004.tiff]
