# Supplementary material for: A Deluge of Complex Repeats: The Solanum Genome
Source: PLoS One. 2015 Aug 4;10(8):e0133962. doi: 10.1371/journal.pone.0133962 (PMC4524691; doi:10.1371/journal.pone.0133962)
Supplement: S5 Fig — The LINE/RTE-BovB super-families identified in S. tuberosum are mentioned with the prefix “Stu”, while those identified in S. lycopersicum are mentioned with the prefix “Sly”. (PDF) [file pone.0133962.s005.pdf]

	20 40 60	
Stu-rnd-1_family-417_LINE_RTE-	---------- ---------- ---------- ---------- ---------- ---------- ----------	-
Sly-rnd1_family377-LINE/RTEBov	---------- ---------- ---------- ---------- ---------- ---------- ----------	-
Stu-rnd-1_family-489_LINE_RTE-	---------- ---------- ---------- ---------- ---------- ---------- ----------	-
Stu-rnd-1_family-607_LINE_RTE-	---------- ---------- ---------- ---------- ---------- ---------- ----------	-
Stu-rnd-1_family-645_LINE_RTE-	---------- ---------- ---------- ---------- ---------- ---------- ----------	-
Sly-rnd4_family81-LINE/RTEBovB	---------- ---------- ---------- ---------- ---------- ---------- ----------	-
Stu-rnd-6_family-408_LINE_RTE-	---------- ---------- ---------- ---------- ---------- ---------- ----------	-
Stu-rnd-1_family-351_LINE_RTE-	---------- ---------- ---------- ---------- ---------- ---------- ----------	-
Stu-rnd-1_family-382_LINE_RTE-	---------- ---------- ---------- ---------- ---------- ---------- ----------	-
Sly-rnd6_family746-LINE/RTEBov	---------- ---------- ---------- ---------- ---------- ---------- ----------	-
Stu-rnd-5_family-325_LINE_RTE-	---------- ---------- ---------- ---------- ---------- ---------- ----------	-
Sly-rnd4_family509-LINE/RTEBov	---------- ---------- ---------- ---------- ---------- ---------- ----------	-
Stu-rnd-1_family-513_LINE_RTE-	---------- ---------- ---------- ---------- ---------- ---------- ----------	-
Sly-rnd5_family1442-LINE/RTEBo	---------- ---------- ---------- ---------- ---------- ---------- ----------	-
BovB_Gl-RTE-Gehyra_lazelli	---------- ---------- ---------- ---------- ---------- ---------- ----------	-
BovB_Gv-RTE-Gehyra_variegata	---------- ---------- ---------- ---------- ---------- ---------- ----------	-
BovB_PMo-RTE-Python_molurus	---------- ---------- ---------- ---------- ---------- ---------- ----------	-
BovB_Oa-RTE-Ovis_aries	---------- ---------- ---------- ---------- ---------- ---------- ----------	-
BovB-RTE-Bos_taurus	GCGCAGGCGG CTGCGACGGG CGCGCTAAGC GCGGCCGAGA GGAGCCACCC CACGTCCGAG GTCAGGGGCA	70
BovB_Me-RTE-Macropus_eugenii	---------- ---------- ---------- ---------- ---------- ---------- ----------	-
BovB_Ano-RTE-Amphibolurus_norr	---------- ---------- ---------- ---------- ---------- ---------- ----------	-
BovB_Gd-RTE-Eremiascincus_doug	---------- ---------- ---------- ---------- ---------- ---------- ----------	-
BovB_Er-RTE-Eremiascincus_rich	---------- ---------- ---------- ---------- ---------- ---------- ----------	-
BovB_Ca-RTE-Ctenotus_atlas	---------- ---------- ---------- ---------- ---------- ---------- ----------	-
BovB_Es-RTE-Egernia_stokesii	---------- ---------- ---------- ---------- ---------- ---------- ----------	-
BovB_Tr-RTE-Tiliqua_rugosa	---------- ---------- ---------- ---------- ---------- ---------- ----------	-
BovB_ALi-RTE-Amblyomma_limbatu	---------- ---------- ---------- ---------- ---------- ---------- ----------	-
BovB_Hs-RTE-Hydrophis_spiralis	---------- ---------- ---------- ---------- ---------- ---------- ----------	-
BovB_VA-RTE-Vipera_ammodytes	---------- ---------- ---------- ---------- ---------- ---------- ----------	-
BovB_ACo-RTE-Agkistrodon_conto	---------- ---------- ---------- ---------- ---------- ---------- ----------	-
BovB_Pg-RTE-Perameles_gunnii	---------- ---------- ---------- ---------- ---------- ---------- ----------	-
BovB_Io-RTE-Isoodon_obesulus	---------- ---------- ---------- ---------- ---------- ---------- ----------	-
BovB_Bp-RTE-Burramys_parvus	---------- ---------- ---------- ---------- ---------- ---------- ----------	-
BovB_Pb-RTE-Petaurus_breviceps	---------- ---------- ---------- ---------- ---------- ---------- ----------	-
BovB_Ma-RTE-Macropus_antilopin	---------- ---------- ---------- ---------- ---------- ---------- ----------	-
BovB_Af-RTE-Antechinus_flavipe	---------- ---------- ---------- ---------- ---------- ---------- ----------	-
BovB_Ac-RTE-Anolis_carolinensi	---------- ---------- ---------- ---------- ---------- ---------- ----------	-
BovB_BM-RTE-Bombyx_mori	---------- ---------- ---------- ---------- ---------- ---------- ----------	-
BovB_Dr-RTE-Christinus	---------- ---------- ---------- ---------- ---------- ---------- ----------	-
BovB_La-RTE-Loxodonta_africana	---------- ---------- ---------- ---------- ---------- ---------- ----------	-
BovB_Pc-RTE-Procavia_capensis	---------- ---------- ---------- ---------- ---------- ---------- ----------	-
BovB_Et-RTE-Christinus	---------- ---------- ---------- ---------- ---------- ---------- ----------	-
BovB_Ta-RTE-Tachyglossus_acule	---------- ---------- ---------- ---------- ---------- ---------- ----------	-
BovB_Plat-RTE-Ornithorhynchus	---------- ---------- ---------- ---------- ---------- ---------- ----------	-
BovB_Ec-RTE-Equus_caballus	---------- ---------- ---------- ---------- ---------- ---------- ----------	-
BovB_Cg-RTE-Christinus	---------- ---------- ---------- ---------- ---------- ---------- ----------	-
BovB_Bh-RTE-Bothriocroton_hydr	---------- ---------- ---------- ---------- ---------- ---------- ----------	-
BovB_Ml-RTE-Gehyra_variegata	---------- ---------- ---------- ---------- ---------- ---------- ----------	-
Sly-rnd1_family284-LINE/RTEBov	---------- ---------- ---------- ---------- ---------- ---------- ----------	-
Sly-rnd5_family2019-LINE/RTEBo	---------- ---------- ---------- ---------- ---------- ---------- ----------	-
Sly-rnd3_family322-LINE/RTEBov	---------- ---------- ---------- ---------- ---------- ---------- ----------	-
Sly-rnd5_family1698-LINE/RTEBo	---------- ---------- ---------- ---------- ---------- ---------- ----------	-
Sly-rnd1_family795-LINE/RTEBov	---------- ---------- ---------- ---------- ---------- ---------- ----------	-
Stu-rnd-5_family-3162_LINE_RTE	---------- ---------- ---------- ---------- ---------- ---------- ----------	-
Sly-rnd6_family1960-LINE/RTEBo	---------- ---------- ---------- ---------- ---------- ---------- ----------	-
Stu-rnd-5_family-2493_LINE_RTE	---------- ---------- ---------- ---------- ---------- ---------- ----------	-
Sly-rnd5_family447-LINE/RTEBov	---------- ---------- ---------- ---------- ---------- ---------- ----------	-
Stu-rnd-6_family-2093_LINE_RTE	---------- ---------- ---------- ---------- ---------- ---------- ----------	-
Consensus	---------- ---------- ---------- ---------- ---------- ---------- ----------	
Conservation (100% / 0%)		

```
                                          80                                    100                                    120                                    140
                                          |                                     |                                      |                                      |
Stu-rnd-1_family-417_LINE_RTE-      ---------- ---------- ---------- ---------- ---------- ---------- ---------- -
Sly-rnd1_family377-LINE/RTEBov      ---------- ---------- ---------- ---------- ---------- ---------- ---------- -
Stu-rnd-1_family-489_LINE_RTE-      ---------- ---------- ---------- ---------- ---------- ---------- ---------- -
Stu-rnd-1_family-607_LINE_RTE-      ---------- ---------- ---------- ---------- ---------- ---------- ---------- -
Stu-rnd-1_family-645_LINE_RTE-      ---------- ---------- ---------- ---------- ---------- ---------- ---------- -
Sly-rnd4_family81-LINE/RTEBovB      ---------- ---------- ---------- ---------- ---------- ---------- ---------- -
Stu-rnd-6_family-408_LINE_RTE-      ---------- ---------- ---------- ---------- ---------- ---------- ---------- -
Stu-rnd-1_family-351_LINE_RTE-      ---------- ---------- ---------- ---------- ---------- ---------- ---------- -
Stu-rnd-1_family-382_LINE_RTE-      ---------- ---------- ---------- ---------- ---------- ---------- ---------- -
Sly-rnd6_family746-LINE/RTEBov      ---------- ---------- ---------- ---------- ---------- ---------- ---------- -
Stu-rnd-5_family-325_LINE_RTE-      ---------- ---------- ---------- ---------- ---------- ---------- ---------- -
Sly-rnd4_family509-LINE/RTEBov      ---------- ---------- ---------- ---------- ---------- ---------- ---------- -
Stu-rnd-1_family-513_LINE_RTE-      ---------- ---------- ---------- ---------- ---------- ---------- ---------- -
Sly-rnd5_family1442-LINE/RTEBo      ---------- ---------- ---------- ---------- ---------- ---------- ---------- -
BovB_Gl-RTE-Gehyra_lazelli          ---------- ---------- ---------- ---------- ---------- ---------- ---------- -
BovB_Gv-RTE-Gehyra_variegata        ---------- ---------- ---------- ---------- ---------- ---------- ---------- -
BovB_PMo-RTE-Python_molurus         ---------- ---------- ---------- ---------- ---------- ---------- ---------- -
BovB_Oa-RTE-Ovis_aries              ---------- ---------- ---------- ---------- ---------- ---------- ---------- -
BovB-RTE-Bos_taurus                 GAAGCCGGGA GGACCCCATG CCCGAAGGGC GGCGGCCAAG AGGAGTTACC CCACGTCCGA GGTCAGGGGC 140
BovB_Me-RTE-Macropus_eugenii        ---------- ---------- ---------- ---------- ---------- ---------- ---------- -
BovB_Ano-RTE-Amphibolurus_norr      ---------- ---------- ---------- ---------- ---------- ---------- ---------- -
BovB_Gd-RTE-Eremiascincus_doug      ---------- ---------- ---------- ---------- ---------- ---------- ---------- -
BovB_Er-RTE-Eremiascincus_rich      ---------- ---------- ---------- ---------- ---------- ---------- ---------- -
BovB_Ca-RTE-Ctenotus_atlas          ---------- ---------- ---------- ---------- ---------- ---------- ---------- -
BovB_Es-RTE-Egernia_stokesii        ---------- ---------- ---------- ---------- ---------- ---------- ---------- -
BovB_Tr-RTE-Tiliqua_rugosa          ---------- ---------- ---------- ---------- ---------- ---------- ---------- -
BovB_ALi-RTE-Amblyomma_limbatu      ---------- ---------- ---------- ---------- ---------- ---------- ---------- -
BovB_Hs-RTE-Hydrophis_spiralis      ---------- ---------- ---------- ---------- ---------- ---------- ---------- -
BovB_VA-RTE-Vipera_ammodytes        ---------- ---------- ---------- ---------- ---------- ---------- ---------- -
BovB_ACo-RTE-Agkistrodon_conto      ---------- ---------- ---------- ---------- ---------- ---------- ---------- -
BovB_Pg-RTE-Perameles_gunnii        ---------- ---------- ---------- ---------- ---------- ---------- ---------- -
BovB_Io-RTE-Isoodon_obesulus        ---------- ---------- ---------- ---------- ---------- ---------- ---------- -
BovB_Bp-RTE-Burramys_parvus         ---------- ---------- ---------- ---------- ---------- ---------- ---------- -
BovB_Pb-RTE-Petaurus_breviceps      ---------- ---------- ---------- ---------- ---------- ---------- ---------- -
BovB_Ma-RTE-Macropus_antilopin      ---------- ---------- ---------- ---------- ---------- ---------- ---------- -
BovB_Af-RTE-Antechinus_flavipe      ---------- ---------- ---------- ---------- ---------- ---------- ---------- -
BovB_Ac-RTE-Anolis_carolinensi      ---------- ---------- ---------- ---------- ---------- ---------- ---------- -
BovB_BM-RTE-Bombyx_mori             ---------- ---------- ---------- ---------- ---------- ---------- ---------- -
BovB_Dr-RTE-Christinus              ---------- ---------- ---------- ---------- ---------- ---------- ---------- -
BovB_La-RTE-Loxodonta_africana      ---------- ---------- ---------- ---------- ---------- ---------- ---------- -
BovB_Pc-RTE-Procavia_capensis       ---------- ---------- ---------- ---------- ---------- ---------- ---------- -
BovB_Et-RTE-Christinus              ---------- ---------- ---------- ---------- ---------- ---------- ---------- -
BovB_Ta-RTE-Tachyglossus_acule      ---------- ---------- ---------- ---------- ---------- ---------- ---------- -
BovB_Plat-RTE-Ornithorhynchus       ---------- ---------- ---------- ---------- ---------- ---------- ---------- -
BovB_Ec-RTE-Equus_caballus          ---------- ---------- ---------- ---------- ---------- ---------- ---------- -
BovB_Cg-RTE-Christinus              ---------- ---------- ---------- ---------- ---------- ---------- ---------- -
BovB_Bh-RTE-Bothriocroton_hydr      ---------- ---------- ---------- ---------- ---------- ---------- ---------- -
BovB_Ml-RTE-Gehyra_variegata        ---------- ---------- ---------- ---------- ---------- ---------- ---------- -
Sly-rnd1_family284-LINE/RTEBov      ---------- ---------- ---------- ---------- ---------- ---------- ---------- -
Sly-rnd5_family2019-LINE/RTEBo      ---------- ---------- ---------- ---------- ---------- ---------- ---------- -
Sly-rnd3_family322-LINE/RTEBov      ---------- ---------- ---------- ---------- ---------- ---------- ---------- -
Sly-rnd5_family1698-LINE/RTEBo      ---------- ---------- ---------- ---------- ---------- ---------- ---------- -
Sly-rnd1_family795-LINE/RTEBov      ---------- ---------- ---------- ---------- ---------- ---------- ---------- -
Stu-rnd-5_family-3162_LINE_RTE      ---------- ---------- ---------- ---------- ---------- ---------- ---------- -
Sly-rnd6_family1960-LINE/RTEBo      ---------- ---------- ---------- ---------- ---------- ---------- ---------- -
Stu-rnd-5_family-2493_LINE_RTE      ---------- ---------- ---------- ---------- ---------- ---------- ---------- -
Sly-rnd5_family447-LINE/RTEBov      ---------- ---------- ---------- ---------- ---------- ---------- ---------- -
Stu-rnd-6_family-2093_LINE_RTE      ---------- ---------- ---------- ---------- ---------- ---------- ---------- -

Consensus                           ---------- ---------- ---------- ---------- ---------- ---------- ----------
                               100%
Conservation
                                 0%
```

	160 180 200	
Stu-rnd-1_family-417_LINE_RTE-	---------- ---------- ---------- ---------- ---------- ---------- ----------	-
Sly-rnd1_family377-LINE/RTEBov	---------- ---------- ---------- ---------- ---------- ---------- ----------	-
Stu-rnd-1_family-489_LINE_RTE-	---------- ---------- ---------- ---------- ---------- ---------- ----------	-
Stu-rnd-1_family-607_LINE_RTE-	---------- ---------- ---------- ---------- ---------- ---------- ----------	-
Stu-rnd-1_family-645_LINE_RTE-	---------- ---------- ---------- ---------- ---------- ---------- ----------	-
Sly-rnd4_family81-LINE/RTEBovB	---------- ---------- ---------- ---------- ---------- ---------- ----------	-
Stu-rnd-6_family-408_LINE_RTE-	---------- ---------- ---------- ---------- ---------- ---------- ----------	-
Stu-rnd-1_family-351_LINE_RTE-	---------- ---------- ---------- ---------- ---------- ---------- ----------	-
Stu-rnd-1_family-382_LINE_RTE-	---------- ---------- ---------- ---------- ---------- ---------- ----------	-
Sly-rnd6_family746-LINE/RTEBov	---------- ---------- ---------- ---------- ---------- ---------- ----------	-
Stu-rnd-5_family-325_LINE_RTE-	---------- ---------- ---------- ---------- ---------- ---------- ----------	-
Sly-rnd4_family509-LINE/RTEBov	---------- ---------- ---------- ---------- ---------- ---------- ----------	-
Stu-rnd-1_family-513_LINE_RTE-	---------- ---------- ---------- ---------- ---------- ---------- ----------	-
Sly-rnd5_family1442-LINE/RTEBo	---------- ---------- ---------- ---------- ---------- ---------- ----------	-
BovB_Gl-RTE-Gehyra_lazelli	---------- ---------- ---------- ---------- ---------- ---------- ----------	-
BovB_Gv-RTE-Gehyra_variegata	---------- ---------- ---------- ---------- ---------- ---------- ----------	-
BovB_PMo-RTE-Python_molurus	---------- ---------- ---------- ---------- ---------- ---------- ----------	-
BovB_Oa-RTE-Ovis_aries	---------- ---------- ---------- ---------- ---------- ---------- ----------	-
BovB-RTE-Bos_taurus	AGCGGCCGAG AGTACCAGAC TGCGACGGCG CAGGAACGGC CGAGAGGAGC TACCCCGCGT CCGAGGTCAG	210
BovB_Me-RTE-Macropus_eugenii	---------- ---------- ---------- ---------- ---------- ---------- ----------	-
BovB_Ano-RTE-Amphibolurus_norr	---------- ---------- ---------- ---------- ---------- ---------- ----------	-
BovB_Gd-RTE-Eremiascincus_doug	---------- ---------- ---------- ---------- ---------- ---------- ----------	-
BovB_Er-RTE-Eremiascincus_rich	---------- ---------- ---------- ---------- ---------- ---------- ----------	-
BovB_Ca-RTE-Ctenotus_atlas	---------- ---------- ---------- ---------- ---------- ---------- ----------	-
BovB_Es-RTE-Egernia_stokesii	---------- ---------- ---------- ---------- ---------- ---------- ----------	-
BovB_Tr-RTE-Tiliqua_rugosa	---------- ---------- ---------- ---------- ---------- ---------- ----------	-
BovB_ALi-RTE-Amblyomma_limbatu	---------- ---------- ---------- ---------- ---------- ---------- ----------	-
BovB_Hs-RTE-Hydrophis_spiralis	---------- ---------- ---------- ---------- ---------- ---------- ----------	-
BovB_VA-RTE-Vipera_ammodytes	---------- ---------- ---------- ---------- ---------- ---------- ----------	-
BovB_ACo-RTE-Agkistrodon_conto	---------- ---------- ---------- ---------- ---------- ---------- ----------	-
BovB_Pg-RTE-Perameles_gunnii	---------- ---------- ---------- ---------- ---------- ---------- ----------	-
BovB_Io-RTE-Isoodon_obesulus	---------- ---------- ---------- ---------- ---------- ---------- ----------	-
BovB_Bp-RTE-Burramys_parvus	---------- ---------- ---------- ---------- ---------- ---------- ----------	-
BovB_Pb-RTE-Petaurus_breviceps	---------- ---------- ---------- ---------- ---------- ---------- ----------	-
BovB_Ma-RTE-Macropus_antilopin	---------- ---------- ---------- ---------- ---------- ---------- ----------	-
BovB_Af-RTE-Antechinus_flavipe	---------- ---------- ---------- ---------- ---------- ---------- ----------	-
BovB_Ac-RTE-Anolis_carolinensi	---------- ---------- ---------- ---------- ---------- ---------- ----------	-
BovB_BM-RTE-Bombyx_mori	---------- ---------- ---------- ---------- ---------- ---------- ----------	-
BovB_Dr-RTE-Christinus	---------- ---------- ---------- ---------- ---------- ---------- ----------	-
BovB_La-RTE-Loxodonta_africana	---------- ---------- ---------- ---------- ---------- ---------- ----------	-
BovB_Pc-RTE-Procavia_capensis	---------- ---------- ---------- ---------- ---------- ---------- ----------	-
BovB_Et-RTE-Christinus	---------- ---------- ---------- ---------- ---------- ---------- ----------	-
BovB_Ta-RTE-Tachyglossus_acule	---------- ---------- ---------- ---------- ---------- ---------- ----------	-
BovB_Plat-RTE-Ornithorhynchus	---------- ---------- ---------- ---------- ---------- ---------- ----------	-
BovB_Ec-RTE-Equus_caballus	---------- ---------- ---------- ---------- ---------- ---------- ----------	-
BovB_Cg-RTE-Christinus	---------- ---------- ---------- ---------- ---------- ---------- ----------	-
BovB_Bh-RTE-Bothriocroton_hydr	---------- ---------- ---------- ---------- ---------- ---------- ----------	-
BovB_Ml-RTE-Gehyra_variegata	---------- ---------- ---------- ---------- ---------- ---------- ----------	-
Sly-rnd1_family284-LINE/RTEBov	---------- ---------- ---------- ---------- ---------- ---------- ----------	-
Sly-rnd5_family2019-LINE/RTEBo	---------- ---------- ---------- ---------- ---------- ---------- ----------	-
Sly-rnd3_family322-LINE/RTEBov	---------- ---------- ---------- ---------- ---------- ---------- ----------	-
Sly-rnd5_family1698-LINE/RTEBo	---------- ---------- ---------- ---------- ---------- ---------- ----------	-
Sly-rnd1_family795-LINE/RTEBov	---------- ---------- ---------- ---------- ---------- ---------- ----------	-
Stu-rnd-5_family-3162_LINE_RTE	---------- ---------- ---------- ---------- ---------- ---------- ----------	-
Sly-rnd6_family1960-LINE/RTEBo	---------- ---------- ---------- ---------- ---------- ---------- ----------	-
Stu-rnd-5_family-2493_LINE_RTE	---------- ---------- ---------- ---------- ---------- ---------- ----------	-
Sly-rnd5_family447-LINE/RTEBov	---------- ---------- ---------- ---------- ---------- ---------- ----------	-
Stu-rnd-6_family-2093_LINE_RTE	---------- ---------- ---------- ---------- ---------- ---------- ----------	-
Consensus	---------- ---------- ---------- ---------- ---------- ---------- ----------	
Conservation (100% / 0%)		

	220 240 260 280	
Stu-rnd-1_family-417_LINE_RTE-	---------- ---------- ---------- ---------- ---------- ---------- ----------	-
Sly-rnd1_family377-LINE/RTEBov	---------- ---------- ---------- ---------- ---------- ---------- ----------	-
Stu-rnd-1_family-489_LINE_RTE-	---------- ---------- ---------- ---------- ---------- ---------- ----------	-
Stu-rnd-1_family-607_LINE_RTE-	---------- ---------- ---------- ---------- ---------- ---------- ----------	-
Stu-rnd-1_family-645_LINE_RTE-	---------- ---------- ---------- ---------- ---------- ---------- ----------	-
Sly-rnd4_family81-LINE/RTEBovB	---------- ---------- ---------- ---------- ---------- ---------- ----------	-
Stu-rnd-6_family-408_LINE_RTE-	---------- ---------- ---------- ---------- ---------- ---------- ----------	-
Stu-rnd-1_family-351_LINE_RTE-	---------- ---------- ---------- ---------- ---------- ---------- ----------	-
Stu-rnd-1_family-382_LINE_RTE-	---------- ---------- ---------- ---------- ---------- ---------- ----------	-
Sly-rnd6_family746-LINE/RTEBov	---------- ---------- ---------- ---------- ---------- ---------- ----------	-
Stu-rnd-5_family-325_LINE_RTE-	---------- ---------- ---------- ---------- ---------- ---------- ----------	-
Sly-rnd4_family509-LINE/RTEBov	---------- ---------- ---------- ---------- ---------- ---------- ----------	-
Stu-rnd-1_family-513_LINE_RTE-	---------- ---------- ---------- ---------- ---------- ---------- ----------	-
Sly-rnd5_family1442-LINE/RTEBo	---------- ---------- ---------- ---------- ---------- ---------- ----------	-
BovB_Gl-RTE-Gehyra_lazelli	---------- ---------- ---------- ---------- ---------- ---------- ----------	-
BovB_Gv-RTE-Gehyra_variegata	---------- ---------- ---------- ---------- ---------- ---------- ----------	-
BovB_PMo-RTE-Python_molurus	---------- ---------- ---------- ---------- ---------- ---------- ----------	-
BovB_Oa-RTE-Ovis_aries	---------- ---------- ---------- ---------- ---------- ---------- ----------	-
BovB-RTE-Bos_taurus	GGGGGGCGGC CGAGAGGAGA TACCCAGCGT CCGAGGTCAG GGGCGGCGAC GAGAGGAGTT ACCCCGCGTC	280
BovB_Me-RTE-Macropus_eugenii	---------- ---------- ---------- ---------- ---------- ---------- ----------	-
BovB_Ano-RTE-Amphibolurus_norr	---------- ---------- ---------- ---------- ---------- ---------- ----------	-
BovB_Gd-RTE-Eremiascincus_doug	---------- ---------- ---------- ---------- ---------- ---------- ----------	-
BovB_Er-RTE-Eremiascincus_rich	---------- ---------- ---------- ---------- ---------- ---------- ----------	-
BovB_Ca-RTE-Ctenotus_atlas	---------- ---------- ---------- ---------- ---------- ---------- ----------	-
BovB_Es-RTE-Egernia_stokesii	---------- ---------- ---------- ---------- ---------- ---------- ----------	-
BovB_Tr-RTE-Tiliqua_rugosa	---------- ---------- ---------- ---------- ---------- ---------- ----------	-
BovB_ALi-RTE-Amblyomma_limbatu	---------- ---------- ---------- ---------- ---------- ---------- ----------	-
BovB_Hs-RTE-Hydrophis_spiralis	---------- ---------- ---------- ---------- ---------- ---------- ----------	-
BovB_VA-RTE-Vipera_ammodytes	---------- ---------- ---------- ---------- ---------- ---------- ----------	-
BovB_ACo-RTE-Agkistrodon_conto	---------- ---------- ---------- ---------- ---------- ---------- ----------	-
BovB_Pg-RTE-Perameles_gunnii	---------- ---------- ---------- ---------- ---------- ---------- ----------	-
BovB_Io-RTE-Isoodon_obesulus	---------- ---------- ---------- ---------- ---------- ---------- ----------	-
BovB_Bp-RTE-Burramys_parvus	---------- ---------- ---------- ---------- ---------- ---------- ----------	-
BovB_Pb-RTE-Petaurus_breviceps	---------- ---------- ---------- ---------- ---------- ---------- ----------	-
BovB_Ma-RTE-Macropus_antilopin	---------- ---------- ---------- ---------- ---------- ---------- ----------	-
BovB_Af-RTE-Antechinus_flavipe	---------- ---------- ---------- ---------- ---------- ---------- ----------	-
BovB_Ac-RTE-Anolis_carolinensi	---------- ---------- ---------- ---------- ---------- ---------- ----------	-
BovB_BM-RTE-Bombyx_mori	---------- ---------- ---------- ---------- ---------- ---------- ----------	-
BovB_Dr-RTE-Christinus	---------- ---------- ---------- ---------- ---------- ---------- ----------	-
BovB_La-RTE-Loxodonta_africana	---------- ---------- ---------- ---------- ---------- ---------- ----------	-
BovB_Pc-RTE-Procavia_capensis	---------- ---------- ---------- ---------- ---------- ---------- ----------	-
BovB_Et-RTE-Christinus	---------- ---------- ---------- ---------- ---------- ---------- ----------	-
BovB_Ta-RTE-Tachyglossus_acule	---------- ---------- ---------- ---------- ---------- ---------- ----------	-
BovB_Plat-RTE-Ornithorhynchus	---------- ---------- ---------- ---------- ---------- ---------- ----------	-
BovB_Ec-RTE-Equus_caballus	---------- ---------- ---------- ---------- ---------- ---------- ----------	-
BovB_Cg-RTE-Christinus	---------- ---------- ---------- ---------- ---------- ---------- ----------	-
BovB_Bh-RTE-Bothriocroton_hydr	---------- ---------- ---------- ---------- ---------- ---------- ----------	-
BovB_Ml-RTE-Gehyra_variegata	---------- ---------- ---------- ---------- ---------- ---------- ----------	-
Sly-rnd1_family284-LINE/RTEBov	---------- ---------- ---------- ---------- ---------- ---------- ----------	-
Sly-rnd5_family2019-LINE/RTEBo	---------- ---------- ---------- ---------- ---------- ---------- ----------	-
Sly-rnd3_family322-LINE/RTEBov	---------- ---------- ---------- ---------- ---------- ---------- ----------	-
Sly-rnd5_family1698-LINE/RTEBo	---------- ---------- ---------- ---------- ---------- ---------- ----------	-
Sly-rnd1_family795-LINE/RTEBov	---------- ---------- ---------- ---------- ---------- ---------- ----------	-
Stu-rnd-5_family-3162_LINE_RTE	---------- ---------- ---------- ---------- ---------- ---------- ----------	-
Sly-rnd6_family1960-LINE/RTEBo	---------- ---------- ---------- ---------- ---------- ---------- ----------	-
Stu-rnd-5_family-2493_LINE_RTE	---------- ---------- ---------- ---------- ---------- ---------- ----------	-
Sly-rnd5_family447-LINE/RTEBov	---------- ---------- ---------- ---------- ---------- ---------- ----------	-
Stu-rnd-6_family-2093_LINE_RTE	---------- ---------- ---------- ---------- ---------- ---------- ----------	-
Consensus	---------- ---------- ---------- ---------- ---------- ---------- ----------	
Conservation (100% / 0%)		

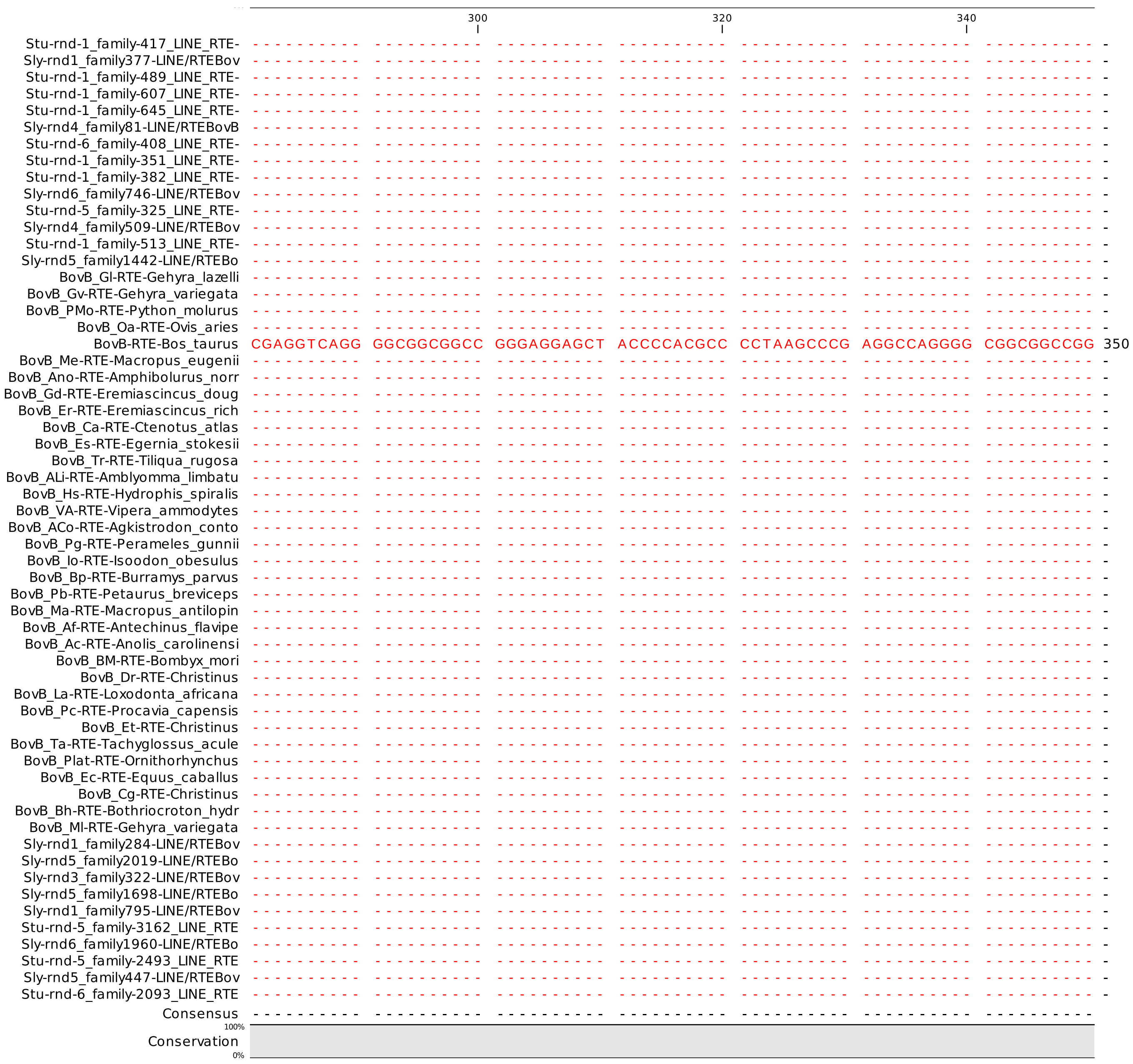
300
320
340
Stu-rnd-1_family-417_LINE_RTE-
Sly-rnd1_family377-LINE/RTEBov
Stu-rnd-1_family-489_LINE_RTE-
Stu-rnd-1_family-607_LINE_RTE-
Stu-rnd-1_family-645_LINE_RTE-
Sly-rnd4_family81-LINE/RTEBovB
Stu-rnd-6_family-408_LINE_RTE-
Stu-rnd-1_family-351_LINE_RTE-
Stu-rnd-1_family-382_LINE_RTE-
Sly-rnd6_family746-LINE/RTEBov
Stu-rnd-5_family-325_LINE_RTE-
Sly-rnd4_family509-LINE/RTEBov
Stu-rnd-1_family-513_LINE_RTE-
Sly-rnd5_family1442-LINE/RTEBo
BovB_Gl-RTE-Gehyra_lazelli
BovB_Gv-RTE-Gehyra_variegata
BovB_PMo-RTE-Python_molurus
BovB_Oa-RTE-Ovis_aries
BovB-RTE-Bos_taurus CGAGGTCAGG GGCGGCGGCC GGGAGGAGCT ACCCCACGCC CCTAAGCCCG AGGCCAGGGG CGGCGGCCGG 350
BovB_Me-RTE-Macropus_eugenii
BovB_Ano-RTE-Amphibolurus_norr
BovB_Gd-RTE-Eremiascincus_doug
BovB_Er-RTE-Eremiascincus_rich
BovB_Ca-RTE-Ctenotus_atlas
BovB_Es-RTE-Egernia_stokesii
BovB_Tr-RTE-Tiliqua_rugosa
BovB_ALi-RTE-Amblyomma_limbatu
BovB_Hs-RTE-Hydrophis_spiralis
BovB_VA-RTE-Vipera_ammodytes
BovB_ACo-RTE-Agkistrodon_conto
BovB_Pg-RTE-Perameles_gunnii
BovB_Io-RTE-Isoodon_obesulus
BovB_Bp-RTE-Burramys_parvus
BovB_Pb-RTE-Petaurus_breviceps
BovB_Ma-RTE-Macropus_antilopin
BovB_Af-RTE-Antechinus_flavipe
BovB_Ac-RTE-Anolis_carolinensi
BovB_BM-RTE-Bombyx_mori
BovB_Dr-RTE-Christinus
BovB_La-RTE-Loxodonta_africana
BovB_Pc-RTE-Procavia_capensis
BovB_Et-RTE-Christinus
BovB_Ta-RTE-Tachyglossus_acule
BovB_Plat-RTE-Ornithorhynchus
BovB_Ec-RTE-Equus_caballus
BovB_Cg-RTE-Christinus
BovB_Bh-RTE-Bothriocroton_hydr
BovB_Ml-RTE-Gehyra_variegata
Sly-rnd1_family284-LINE/RTEBov
Sly-rnd5_family2019-LINE/RTEBo
Sly-rnd3_family322-LINE/RTEBov
Sly-rnd5_family1698-LINE/RTEBo
Sly-rnd1_family795-LINE/RTEBov
Stu-rnd-5_family-3162_LINE_RTE
Sly-rnd6_family1960-LINE/RTEBo
Stu-rnd-5_family-2493_LINE_RTE
Sly-rnd5_family447-LINE/RTEBov
Stu-rnd-6_family-2093_LINE_RTE
Consensus
100%
Conservation
0%

	360		380		400		420	
Stu-rnd-1_family-417_LINE_RTE-	----------	----------	----------	----------	----------	----------	----------	-
Sly-rnd1_family377-LINE/RTEBov	----------	----------	----------	----------	----------	----------	----------	-
Stu-rnd-1_family-489_LINE_RTE-	----------	----------	----------	----------	----------	----------	----------	-
Stu-rnd-1_family-607_LINE_RTE-	----------	----------	----------	----------	----------	----------	----------	-
Stu-rnd-1_family-645_LINE_RTE-	----------	----------	----------	----------	----------	----------	----------	-
Sly-rnd4_family81-LINE/RTEBovB	----------	----------	----------	----------	----------	----------	----------	-
Stu-rnd-6_family-408_LINE_RTE-	----------	----------	----------	----------	----------	----------	----------	-
Stu-rnd-1_family-351_LINE_RTE-	----------	----------	----------	----------	----------	----------	----------	-
Stu-rnd-1_family-382_LINE_RTE-	----------	----------	----------	----------	----------	----------	----------	-
Sly-rnd6_family746-LINE/RTEBov	----------	----------	----------	----------	----------	----------	----------	-
Stu-rnd-5_family-325_LINE_RTE-	----------	----------	----------	----------	----------	----------	----------	-
Sly-rnd4_family509-LINE/RTEBov	----------	----------	----------	----------	----------	----------	----------	-
Stu-rnd-1_family-513_LINE_RTE-	----------	----------	----------	----------	----------	----------	----------	-
Sly-rnd5_family1442-LINE/RTEBo	----------	----------	----------	----------	----------	----------	----------	-
BovB_Gl-RTE-Gehyra_lazelli	----------	----------	----------	----------	----------	----------	----------	-
BovB_Gv-RTE-Gehyra_variegata	----------	----------	----------	----------	----------	----------	----------	-
BovB_PMo-RTE-Python_molurus	----------	----------	----------	----------	----------	----------	----------	-
BovB_Oa-RTE-Ovis_aries	----------	----------	----------	----------	----------	----------	----------	-
BovB-RTE-Bos_taurus	GAGGAGCAAC	CCCACGCCCG	AGGCCAGGGG	CGGCGGCCGG	GAGGACCAAC	CCCACGTCCA	AGGAGCCGTG	420
BovB_Me-RTE-Macropus_eugenii	----------	----------	----------	----------	----------	----------	----------	-
BovB_Ano-RTE-Amphibolurus_norr	----------	----------	----------	----------	----------	----------	----------	-
BovB_Gd-RTE-Eremiascincus_doug	----------	----------	----------	----------	----------	----------	----------	-
BovB_Er-RTE-Eremiascincus_rich	----------	----------	----------	----------	----------	----------	----------	-
BovB_Ca-RTE-Ctenotus_atlas	----------	----------	----------	----------	----------	----------	----------	-
BovB_Es-RTE-Egernia_stokesii	----------	----------	----------	----------	----------	----------	----------	-
BovB_Tr-RTE-Tiliqua_rugosa	----------	----------	----------	----------	----------	----------	----------	-
BovB_ALi-RTE-Amblyomma_limbatu	----------	----------	----------	----------	----------	----------	----------	-
BovB_Hs-RTE-Hydrophis_spiralis	----------	----------	----------	----------	----------	----------	----------	-
BovB_VA-RTE-Vipera_ammodytes	----------	----------	----------	----------	----------	----------	----------	-
BovB_ACo-RTE-Agkistrodon_conto	----------	----------	----------	----------	----------	----------	----------	-
BovB_Pg-RTE-Perameles_gunnii	----------	----------	----------	----------	----------	----------	----------	-
BovB_Io-RTE-Isoodon_obesulus	----------	----------	----------	----------	----------	----------	----------	-
BovB_Bp-RTE-Burramys_parvus	----------	----------	----------	----------	----------	----------	----------	-
BovB_Pb-RTE-Petaurus_breviceps	----------	----------	----------	----------	----------	----------	----------	-
BovB_Ma-RTE-Macropus_antilopin	----------	----------	----------	----------	----------	----------	----------	-
BovB_Af-RTE-Antechinus_flavipe	----------	----------	----------	----------	----------	----------	----------	-
BovB_Ac-RTE-Anolis_carolinensi	----------	----------	----------	----------	----------	----------	----------	-
BovB_BM-RTE-Bombyx_mori	----------	----------	----------	----------	----------	----------	----------	-
BovB_Dr-RTE-Christinus	----------	----------	----------	----------	----------	----------	----------	-
BovB_La-RTE-Loxodonta_africana	----------	----------	----------	----------	----------	----------	----------	-
BovB_Pc-RTE-Procavia_capensis	----------	----------	----------	----------	----------	----------	----------	-
BovB_Et-RTE-Christinus	----------	----------	----------	----------	----------	----------	----------	-
BovB_Ta-RTE-Tachyglossus_acule	----------	----------	----------	----------	----------	----------	----------	-
BovB_Plat-RTE-Ornithorhynchus	----------	----------	----------	----------	----------	----------	----------	-
BovB_Ec-RTE-Equus_caballus	----------	----------	----------	----------	----------	----------	----------	-
BovB_Cg-RTE-Christinus	----------	----------	----------	----------	----------	----------	----------	-
BovB_Bh-RTE-Bothriocroton_hydr	----------	----------	----------	----------	----------	----------	----------	-
BovB_Ml-RTE-Gehyra_variegata	----------	----------	----------	----------	----------	----------	----------	-
Sly-rnd1_family284-LINE/RTEBov	----------	----------	----------	----------	----------	----------	----------	-
Sly-rnd5_family2019-LINE/RTEBo	----------	----------	----------	----------	----------	----------	----------	-
Sly-rnd3_family322-LINE/RTEBov	----------	----------	----------	----------	----------	----------	----------	-
Sly-rnd5_family1698-LINE/RTEBo	----------	----------	----------	----------	----------	----------	----------	-
Sly-rnd1_family795-LINE/RTEBov	----------	----------	----------	----------	----------	----------	----------	-
Stu-rnd-5_family-3162_LINE_RTE	----------	----------	----------	----------	----------	----------	----------	-
Sly-rnd6_family1960-LINE/RTEBo	----------	----------	----------	----------	----------	----------	----------	-
Stu-rnd-5_family-2493_LINE_RTE	----------	----------	----------	----------	----------	----------	----------	-
Sly-rnd5_family447-LINE/RTEBov	----------	----------	----------	----------	----------	----------	----------	-
Stu-rnd-6_family-2093_LINE_RTE	----------	----------	----------	----------	----------	----------	----------	-
Consensus	----------	----------	----------	----------	----------	----------	----------	

Conservation (100% / 0%)

```
                                     440                 460                 480
Stu-rnd-1_family-417_LINE_RTE-       ---------- ---------- ---------- ---------- ---------- ---------- ---------- -
Sly-rnd1_family377-LINE/RTEBov       ---------- ---------- ---------- ---------- ---------- ---------- ---------- -
Stu-rnd-1_family-489_LINE_RTE-       ---------- ---------- ---------- ---------- ---------- ---------- ---------- -
Stu-rnd-1_family-607_LINE_RTE-       ---------- ---------- ---------- ---------- ---------- ---------- ---------- -
Stu-rnd-1_family-645_LINE_RTE-       ---------- ---------- ---------- ---------- ---------- ---------- ---------- -
Sly-rnd4_family81-LINE/RTEBovB       ---------- ---------- ---------- ---------- ---------- ---------- ---------- -
Stu-rnd-6_family-408_LINE_RTE-       ---------- ---------- ---------- ---------- ---------- ---------- ---------- -
Stu-rnd-1_family-351_LINE_RTE-       ---------- ---------- ---------- ---------- ---------- ---------- ---------- -
Stu-rnd-1_family-382_LINE_RTE-       ---------- ---------- ---------- ---------- ---------- ---------- ---------- -
Sly-rnd6_family746-LINE/RTEBov       ---------- ---------- ---------- ---------- ---------- ---------- ---------- -
Stu-rnd-5_family-325_LINE_RTE-       ---------- ---------- ---------- ---------- ---------- ---------- ---------- -
Sly-rnd4_family509-LINE/RTEBov       ---------- ---------- ---------- ---------- ---------- ---------- ---------- -
Stu-rnd-1_family-513_LINE_RTE-       ---------- ---------- ---------- ---------- ---------- ---------- ---------- -
Sly-rnd5_family1442-LINE/RTEBo       ---------- ---------- ---------- ---------- ---------- ---------- ---------- -
BovB_Gl-RTE-Gehyra_lazelli           ---------- ---------- ---------- ---------- ---------- ---------- ---------- -
BovB_Gv-RTE-Gehyra_variegata         ---------- ---------- ---------- ---------- ---------- ---------- ---------- -
BovB_PMo-RTE-Python_molurus          ---------- ---------- ---------- ---------- ---------- ---------- ---------- -
BovB_Oa-RTE-Ovis_aries               ---------- ---------- ---------- ---------- ---------- ---------- ---------- -
BovB-RTE-Bos_taurus                  GCTGCGCGGG CACAGGAGGG CCTAGAGGAG CTATCCCACG TTGAAGGTCA GGAAGGGCGG CGGTGAGGAG 490
BovB_Me-RTE-Macropus_eugenii         ---------- ---------- ---------- ---------- ---------- ---------- ---------- -
BovB_Ano-RTE-Amphibolurus_norr       ---------- ---------- ---------- ---------- ---------- ---------- ---------- -
BovB_Gd-RTE-Eremiascincus_doug       ---------- ---------- ---------- ---------- ---------- ---------- ---------- -
BovB_Er-RTE-Eremiascincus_rich       ---------- ---------- ---AAACTAA GATACGGGTA CTAAAGGATA GTGCGGGTGG GGCCGCGGTG 47
BovB_Ca-RTE-Ctenotus_atlas           ---------- ---------- ---------- --TGAAAGTA TCTGGGATCA GTTTTGCCAA AAGTGAGAGG 38
BovB_Es-RTE-Egernia_stokesii         ---------- ---------- ---------- ---------- ---------- ---------- ---------- -
BovB_Tr-RTE-Tiliqua_rugosa           ---------- ---------- ---------- ---------- ---------- ---------- ---------- -
BovB_ALi-RTE-Amblyomma_limbatu       ---------- ---------- ---------- ---------- ---------- ---------- ---------- -
BovB_Hs-RTE-Hydrophis_spiralis       ---------- ---------- ---------- ---------- ---------- ---------- ---------- -
BovB_VA-RTE-Vipera_ammodytes         ---------- ---------- ---------- ---------- ---------- ---------- ---------- -
BovB_ACo-RTE-Agkistrodon_conto       ---------- ---------- ---------- ---------- ---------- ---------- ---------- -
BovB_Pg-RTE-Perameles_gunnii         ---------- ---------- ---------- ---------- ---------- ---------- ---------- -
BovB_Io-RTE-Isoodon_obesulus         ---------- ---------- ---------- ---------- ---------- ---------- ---------- -
BovB_Bp-RTE-Burramys_parvus          ---------- ---------- ---------- ---------- ---------- ---------- ---------- -
BovB_Pb-RTE-Petaurus_breviceps       ---------- ---------- ---------- ---------- ---------- ---------- ---------- -
BovB_Ma-RTE-Macropus_antilopin       ---------- ---------- ---------- ---------- ---------- ---------- ---------- -
BovB_Af-RTE-Antechinus_flavipe       ---------- ---------- ---------- ---------- ---------- ---------- ---------- -
BovB_Ac-RTE-Anolis_carolinensi       ---------- ---------- ---------- ---------- ---------- ---------- ---------- -
BovB_BM-RTE-Bombyx_mori              ---------- ---------- ---------- ---------- ---------- ---------- ---------- -
BovB_Dr-RTE-Christinus               ---------- ---------- ---------- ---------- ---------- ---------- ---------- -
BovB_La-RTE-Loxodonta_africana       ---------- ---------- ---------- ---------- ---------- ---------- ---------- -
BovB_Pc-RTE-Procavia_capensis        ---------- ---------- ---------- ---------- ---------- ---------- ---------- -
BovB_Et-RTE-Christinus               ---------- ---------- ---------- ---------- ---------- ---------- ---------- -
BovB_Ta-RTE-Tachyglossus_acule       ---------- ---------- ---------- ---------- ---------- ---------- ---------- -
BovB_Plat-RTE-Ornithorhynchus        ---------- ---------- ---------- ---------- ---------- ---------- ---------- -
BovB_Ec-RTE-Equus_caballus           ---------- ---------- ---------- ---------- ---------- ---------- ---------- -
BovB_Cg-RTE-Christinus               ---------- ---------- ---------- ---------- ---------- ---------- ---------- -
BovB_Bh-RTE-Bothriocroton_hydr       ---------- ---------- ---------- ---------- ---------- ---------- ---------- -
BovB_Ml-RTE-Gehyra_variegata         ---------- ---------- ---------- ---------- ---------- ---------- ---------- -
Sly-rnd1_family284-LINE/RTEBov       ---------- ---------- ---------- ---------- ---------- ---------- ---------- -
Sly-rnd5_family2019-LINE/RTEBo       ---------- ---------- ---------- ---------- ---------- ---------- ---------- -
Sly-rnd3_family322-LINE/RTEBov       ---------- ---------- ---------- ---------- ---------- ---------- ---------- -
Sly-rnd5_family1698-LINE/RTEBo       ---------- ---------- ---------- ---------- ---------- ---------- ---------- -
Sly-rnd1_family795-LINE/RTEBov       ---------- ---------- ---------- ---------- ---------- ---------- ---------- -
Stu-rnd-5_family-3162_LINE_RTE       ---------- ---------- ---------- ---------- ---------- ---------- ---------- -
Sly-rnd6_family1960-LINE/RTEBo       ---------- ---------- ---------- ---------- ---------- ---------- ---------- -
Stu-rnd-5_family-2493_LINE_RTE       ---------- ---------- ---------- ---------- ---------- ---------- ---------- -
Sly-rnd5_family447-LINE/RTEBov       ---------- ---------- ---------- ---------- ---------- ---------- ---------- -
Stu-rnd-6_family-2093_LINE_RTE       ---------- ---------- ---------- ---------- ---------- ---------- ---------- -
Consensus                            ---------- ---------- ---------- ---------- ---------- ---------- ----------
```

Conservation

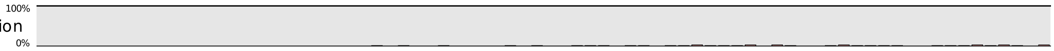

Ruler positions: 500, 520, 540, 560

Stu-rnd-1_family-417_LINE_RTE-	----------	----------	----------	----------	----------	----------	----------	-
Sly-rnd1_family377-LINE/RTEBov	----------	----------	----------	----------	----------	----------	----------	-
Stu-rnd-1_family-489_LINE_RTE-	----------	----------	----------	----------	----------	----------	----------	-
Stu-rnd-1_family-607_LINE_RTE-	----------	----------	----------	----------	----------	----------	----------	-
Stu-rnd-1_family-645_LINE_RTE-	----------	----------	----------	----------	----------	----------	----------	-
Sly-rnd4_family81-LINE/RTEBovB	----------	----------	----------	----------	----------	----------	----------	-
Stu-rnd-6_family-408_LINE_RTE-	----------	----------	----------	----------	----------	----------	----------	-
Stu-rnd-1_family-351_LINE_RTE-	----------	----------	----------	----------	----------	----------	----------	-
Stu-rnd-1_family-382_LINE_RTE-	----------	----------	----------	----------	----------	----------	----------	-
Sly-rnd6_family746-LINE/RTEBov	----------	----------	----------	----------	----------	----------	----------	-
Stu-rnd-5_family-325_LINE_RTE-	----------	----------	----------	----------	----------	----------	----------	-
Sly-rnd4_family509-LINE/RTEBov	----------	----------	----------	----------	----------	----------	----------	-
Stu-rnd-1_family-513_LINE_RTE-	----------	----------	----------	----------	----------	----------	----------	-
Sly-rnd5_family1442-LINE/RTEBo	----------	----------	----------	----------	----------	----------	----------	-
BovB_Gl-RTE-Gehyra_lazelli	----------	-------TTT	TCTTTTTCTT	TTTGAGGACA	TCGTCTTTAA	AAAACGTTTC	ATTGAAGGAA	53
BovB_Gv-RTE-Gehyra_variegata	----------	----------	----------	----------	----------	----------	----------	-
BovB_PMo-RTE-Python_molurus	----------	----------	----------	----------	----------	----------	----------	-
BovB_Oa-RTE-Ovis_aries	----------	----------	----------	----------	----------	----------	----------	-
BovB-RTE-Bos_taurus	ATACCCCTCG	TCCAAGGTAA	GGAGCAGCGG	CTGCGCTTTG	CTGGAGCAGC	CGTGAAGAGA	TACCCCACGC	560
BovB_Me-RTE-Macropus_eugenii	----------	----------	----------	----------	----------	----------	----------	-
BovB_Ano-RTE-Amphibolurus_norr	----------	----------	----------	----------	----------	----------	----------	-
BovB_Gd-RTE-Eremiascincus_doug	----------	----------	----------	----------	----------	----------	----------	-
BovB_Er-RTE-Eremiascincus_rich	GCACAATAGA	-GCAGGCAAG	ATCTCGTCTT	CGCTTCCCCG	TCGTCGCTTC	CCCGCTGTAA	TCGGAACAGC	116
BovB_Ca-RTE-Ctenotus_atlas	CCATCTTGAA	CATCTGCTAT	ATTTTGAATG	ATATATTTAA	ATCTGCTATA	TTTTGAGTGA	GGATCATTGC	108
BovB_Es-RTE-Egernia_stokesii	----------	----------	----------	----------	----------	----------	----------	-
BovB_Tr-RTE-Tiliqua_rugosa	----------	----------	----------	----------	----------	----------	----------	-
BovB_ALi-RTE-Amblyomma_limbatu	----------	----------	CCAAGCGTAC	CTTTGAGTGC	CCGATTTATT	TCAGACACAC	GCATCCCTAC	50
BovB_Hs-RTE-Hydrophis_spiralis	----------	----------	----------	----------	----------	----------	----------	-
BovB_VA-RTE-Vipera_ammodytes	----------	----------	----------	----------	----------	----------	----------	-
BovB_ACo-RTE-Agkistrodon_conto	----------	----------	----------	----------	----------	----------	----------	-
BovB_Pg-RTE-Perameles_gunnii	----------	----------	----------	----------	----------	----------	----------	-
BovB_Io-RTE-Isoodon_obesulus	----------	----------	----------	----------	----------	----------	----------	-
BovB_Bp-RTE-Burramys_parvus	----------	----------	----------	----------	----------	----------	----------	-
BovB_Pb-RTE-Petaurus_breviceps	----------	----------	----------	----------	----------	----------	----------	-
BovB_Ma-RTE-Macropus_antilopin	----------	----------	----------	----------	----------	----------	----------	-
BovB_Af-RTE-Antechinus_flavipe	----------	----------	----------	----------	----------	----------	----------	-
BovB_Ac-RTE-Anolis_carolinensi	----------	----------	----------	----------	----------	----------	----------	-
BovB_BM-RTE-Bombyx_mori	----------	----------	----------	----------	----------	----------	----------	-
BovB_Dr-RTE-Christinus	----------	----------	----------	----------	----------	----------	----------	-
BovB_La-RTE-Loxodonta_africana	----------	----------	----------	----------	----------	----------	----------	-
BovB_Pc-RTE-Procavia_capensis	----------	----------	----------	----------	----------	----------	----------	-
BovB_Et-RTE-Christinus	----------	----------	----------	----------	----------	----------	----------	-
BovB_Ta-RTE-Tachyglossus_acule	----------	----------	-------ATT	ATCCTGAAAC	AAAGTTCTCC	AACGAATCAA	TACCTTACCA	43
BovB_Plat-RTE-Ornithorhynchus	----------	----------	----------	----------	-ACTCTCTCC	TATGAATCAA	CGCCTTACCG	29
BovB_Ec-RTE-Equus_caballus	----------	----------	----------	----------	----------	----------	----------	-
BovB_Cg-RTE-Christinus	----------	----------	----------	----------	----------	----------	----------	-
BovB_Bh-RTE-Bothriocroton_hydr	----------	----------	----------	----------	----------	----------	----------	-
BovB_Ml-RTE-Gehyra_variegata	----------	----------	----------	----------	----------	----------	----------	-
Sly-rnd1_family284-LINE/RTEBov	----------	----------	----------	----------	----------	----------	----------	-
Sly-rnd5_family2019-LINE/RTEBo	----------	----------	----------	----------	----------	----------	----------	-
Sly-rnd3_family322-LINE/RTEBov	----------	----------	----------	----------	----------	----------	----------	-
Sly-rnd5_family1698-LINE/RTEBo	----------	----------	----------	----------	----------	----------	----------	-
Sly-rnd1_family795-LINE/RTEBov	----------	----------	----------	----------	----------	----------	----------	-
Stu-rnd-5_family-3162_LINE_RTE	----------	----------	----------	----------	----------	----------	----------	-
Sly-rnd6_family1960-LINE/RTEBo	----------	----------	----------	----------	----------	----------	----------	-
Stu-rnd-5_family-2493_LINE_RTE	----------	----------	----------	----------	----------	----------	----------	-
Sly-rnd5_family447-LINE/RTEBov	----------	----------	----------	----------	----------	----------	----------	-
Stu-rnd-6_family-2093_LINE_RTE	----------	----------	----------	----------	----------	----------	----------	-
Consensus	----------	----------	----------	----------	----------	----------	----------	

Conservation (100% – 0%)

```
                                        580                            600                            620
                                          |                              |                              |
Stu-rnd-1_family-417_LINE_RTE-  ---------- ---------- ---------- ---------- ---------- ---------- ---------- -
Sly-rnd1_family377-LINE/RTEBov  ---------- ---------- ---------- ---------- ---------- ---------- ---------- -
Stu-rnd-1_family-489_LINE_RTE-  ---------- ---------- ---------- ---------- ---------- ---------- ---------- -
Stu-rnd-1_family-607_LINE_RTE-  ---------- ---------- ---------- ---------- ---------- ---------- ---------- -
Stu-rnd-1_family-645_LINE_RTE-  ---------- ---------- ---------- ---------- ---------- ---------- ---------- -
Sly-rnd4_family81-LINE/RTEBovB  ---------- ---------- ---------- ---------- ---------- ---------- ---------- -
Stu-rnd-6_family-408_LINE_RTE-  ---------- ---------- ---------- ---------- ---------- ---------- ---------- -
Stu-rnd-1_family-351_LINE_RTE-  ---------- ---------- ---------- ---------- ---------- ---------- ---------- -
Stu-rnd-1_family-382_LINE_RTE-  ---------- ---------- ---------- ---------- ---------- ---------- ---------- -
Sly-rnd6_family746-LINE/RTEBov  ---------- ---------- ---------- ---------- ---------- ---------- ---------- -
Stu-rnd-5_family-325_LINE_RTE-  ---------- ---------- ---------- ---------- ---------- ---------- ---------- -
Sly-rnd4_family509-LINE/RTEBov  ---------- ---------- ---------- ---------- ---------- ---------- ---------- -
Stu-rnd-1_family-513_LINE_RTE-  ---------- ---------- ---------- ---------- ---------- ---------- ---------- -
Sly-rnd5_family1442-LINE/RTEBo  ---------- ---------- ---------- ---------- ---------- ---------- ---------- -
BovB_Gl-RTE-Gehyra_lazelli      AGTTCCTCAG CTCTTGGCAC ACATCTCCAG GATACCTTTA ATAAAGAAAA AATTTTTATC ATTTAATTAT 123
BovB_Gv-RTE-Gehyra_variegata    -----TTCAA CTTCCGAAGC AAAAGT--AG GTGATGATTA AGAATAAAAT AACAGTTGTC AT------GG 57
BovB_PMo-RTE-Python_molurus     ---------- ---------- ---------- ---------- ---------- ---------- ---------- -
BovB_Oa-RTE-Ovis_aries          ---------- ---------- ---------- ---------- ---------C AGAGGGCAGA CAGAATGAAA 21
BovB-RTE-Bos_taurus             CCAAGGTAAG AGAAACCCAA GTAAGACGGT AGGTGTTGCA AGAGGGCATC AGAGGGCAGA CACACTGAAA 630
BovB_Me-RTE-Macropus_eugenii    ---------- ---------- ---------- ---------- ---------- ---------- ---------- -
BovB_Ano-RTE-Amphibolurus_norr  ----TGAAAT ACAGAACTAG AAAGCAAAGT TTGTGTTAGT AATTACTCAT GGTAATATTT AAGTGGGATT 66
BovB_Gd-RTE-Eremiascincus_doug  ---------- ---------- ---------- ---------- ---------- ---------- ---------- -
BovB_Er-RTE-Eremiascincus_rich  TCAGAATGCC AACAACCCGT TTCCTAAGGG TGGTTTTGGG TCTGAGTAAG GTAACGTTTC AAACCCTCTA 186
BovB_Ca-RTE-Ctenotus_atlas      CTTGTCTTGT CTTGTAAAGA CACCTTAAAG CACCAAGAAG TGAAAGGCGC GATACAAATA AACATAAACA 178
BovB_Es-RTE-Egernia_stokesii    ---------- ---------- ---------- ---------- ---------- ---------- --------AT 2
BovB_Tr-RTE-Tiliqua_rugosa      ---------- ---------- ---------- ---------- -----ATGCT TCCTAAGACC CATTTGACTT 25
BovB_ALi-RTE-Amblyomma_limbatu  GGTGATAAAG GGCGAACGCT GTCCACGCGC GAGACCATCC TCTCACACGC AGAACATTAT TGGGTCAGTA 120
BovB_Hs-RTE-Hydrophis_spiralis  ---------- ---------- ---------- ---------- ---------- ---------- ---------- -
BovB_VA-RTE-Vipera_ammodytes    ---------- ---------- ---------- ---------- ---------- ---------- ---------- -
BovB_ACo-RTE-Agkistrodon_conto  ---------- ---------- ---------- ---------- ---------- ---------- ---------- -
BovB_Pg-RTE-Perameles_gunnii    ---------- ---------- ---------- ---------- ---------- ---------- ---------- -
BovB_Io-RTE-Isoodon_obesulus    ---------- ---------- ---------- ---------- ---------- ---------- ---------- -
BovB_Bp-RTE-Burramys_parvus     ---------- ---------- ---------- ---------- ---------- ---------- ---------- -
BovB_Pb-RTE-Petaurus_breviceps  ---------- ---------- ---------- ---------- ---------- ---------- ---------- -
BovB_Ma-RTE-Macropus_antilopin  ---------- ---------- ---------- ---------- ---------- ---------- ---------- -
BovB_Af-RTE-Antechinus_flavipe  ---------- ---------- ---------- ---------- ---------- ---------- ---------- -
BovB_Ac-RTE-Anolis_carolinensi  ---------- ---------- ---------- ---------- ---------- ---------- ---------- -
BovB_BM-RTE-Bombyx_mori         ---------- ---------- ---------- ---------- ---------- ---------- ---------- -
BovB_Dr-RTE-Christinus          ---------- ---------- ---------- ---------- ---------- ---------- ---------- -
BovB_La-RTE-Loxodonta_africana  ---------- ---------- ---------- ---------- ---------- ---------- ---------- -
BovB_Pc-RTE-Procavia_capensis   ---------- ---------- ---------- ---------- ---------- ---------- ---------- -
BovB_Et-RTE-Christinus          ---------- ---------- ---------- ---------- ---------- ---------- ---------- -
BovB_Ta-RTE-Tachyglossus_acule  GGGTCAGGAG AGTGAGCATA CGCCAATGAA ACTTAGAGCA ATGCCACTGA GAGATACTCT CTCACAAGGG 113
BovB_Plat-RTE-Ornithorhynchus   TGG-CAGGAG AGTTTGCGTA CGCCAATGAA GCTTAGAGCT ATGCCATTGA GAGATACTCT --CGCTAGGG 96
BovB_Ec-RTE-Equus_caballus      ---------- ---------- ---------- ---------- ---------- ---------- ---------- -
BovB_Cg-RTE-Christinus          ---------- ---------- ---------- ---------- ---------- ---------- ---------- -
BovB_Bh-RTE-Bothriocroton_hydr  ---------- ---------- ---------- ---------- ---------- ---------- ---------- -
BovB_Ml-RTE-Gehyra_variegata    ---------- ---------- ---------- ---------- ---------- ---------- ---------- -
Sly-rnd1_family284-LINE/RTEBov  ---------- ---------- ---------- ---------- ---------- ---------- ---------- -
Sly-rnd5_family2019-LINE/RTEBo  ---------- ---------- ---------- ---------- ---------- ---------- ---------- -
Sly-rnd3_family322-LINE/RTEBov  ---------- ---------- ---------- ---------- ---------- ---------- ---------- -
Sly-rnd5_family1698-LINE/RTEBo  ---------- ---------- ---------- ---------- ---------- ---------- ---------- -
Sly-rnd1_family795-LINE/RTEBov  ---------- ---------- ---------- ---------- ---------- ---------- ---------- -
Stu-rnd-5_family-3162_LINE_RTE  ---------- ---------- ---------- ---------- ---------- ---------- ---------- -
Sly-rnd6_family1960-LINE/RTEBo  ---------- ---------- ---------- ---------- ---------- ---------- ---------- -
Stu-rnd-5_family-2493_LINE_RTE  ---------- ---------- ---------- ---------- ---------- ---------- ---------- -
Sly-rnd5_family447-LINE/RTEBov  ---------- ---------- ---------- ---------- ---------- ---------- ---------- -
Stu-rnd-6_family-2093_LINE_RTE  ---------- ---------- ---------- ---------- ---------- ---------- ---------- -

Consensus                       ---------- ---------- ---------- ---------- ---------- ---------- ----------
                          100%
Conservation
                            0%
```

	640		660		680		700	
Stu-rnd-1_family-417_LINE_RTE-	----------	----------	----------	----------	----------	----------	----------	-
Sly-rnd1_family377-LINE/RTEBov	----------	----------	----------	----------	----------	----------	----------	-
Stu-rnd-1_family-489_LINE_RTE-	----------	----------	----------	----------	----------	----------	----------	-
Stu-rnd-1_family-607_LINE_RTE-	----------	----------	----------	----------	----------	----------	----------	-
Stu-rnd-1_family-645_LINE_RTE-	----------	----------	----------	----------	----------	----------	----------	-
Sly-rnd4_family81-LINE/RTEBovB	----------	----------	----------	----------	----------	----------	----------	-
Stu-rnd-6_family-408_LINE_RTE-	----------	----------	----------	----------	----------	----------	----------	-
Stu-rnd-1_family-351_LINE_RTE-	----------	----------	----------	----------	----------	----------	----------	-
Stu-rnd-1_family-382_LINE_RTE-	----------	----------	----------	----------	----------	----------	----------	-
Sly-rnd6_family746-LINE/RTEBov	----------	----------	----------	----------	----------	----------	----------	-
Stu-rnd-5_family-325_LINE_RTE-	----------	----------	----------	----------	----------	----------	----------	-
Sly-rnd4_family509-LINE/RTEBov	----------	----------	----------	----------	----------	----------	----------	-
Stu-rnd-1_family-513_LINE_RTE-	----------	----------	----------	----------	----------	----------	----------	-
Sly-rnd5_family1442-LINE/RTEBo	----------	----------	----------	----------	----------	----------	----------	-
BovB_Gl-RTE-Gehyra_lazelli	ATTTTTAATC	CCTCTCATGG	ATCA---TTG	CCTTGACGCG	GCGAGGGGG-	CTTGCGTGGT	TCGATGAGGC	189
BovB_Gv-RTE-Gehyra_variegata	ATCATTG--C	CCTCTCATGG	ATCA---TTG	CCTTGATGTG	GCGAGGGGG-	CTTGCGTGGT	TCGATGAGGC	121
BovB_PMo-RTE-Python_molurus	ACCCCCA--C	CCCTTCATGG	ATCA---CTG	CCTTGTCGTG	GCGAAGGGG-	CTTGCGTAAC	TCAGTGAAGC	64
BovB_Oa-RTE-Ovis_aries	ACCACAATCA	CCCTTCATGG	ATCA---CAG	CCTTGTCATG	GCAAACGGG-	CTTGTATAAC	TCAATGAAAC	87
BovB-RTE-Bos_taurus	-CCATACTCA	CAGAAAACTA	GTCAATCTAA	TCACACTAGG	ACCA-CAGC-	CTTGTCTAAC	TCAATGAAAC	697
BovB_Me-RTE-Macropus_eugenii	----------	----------	----------	----------	----------	----------	----------	-
BovB_Ano-RTE-Amphibolurus_norr	ATTTTACACC	CCCTTCATGG	ATTG---CTG	CCTTGTCGTG	GCGAAGGGG-	CTTGAGTAAC	TCAGAGAAGC	132
BovB_Gd-RTE-Eremiascincus_doug	----------	----------	----------	----------	----------	----------	----------	-
BovB_Er-RTE-Eremiascincus_rich	GTCGTCCCTC	CCTTTCATAC	AACA---AAC	CTCTCGTAGC	GAAAAGGGG-	CTTGAGTAAC	TCAATGAAGC	252
BovB_Ca-RTE-Ctenotus_atlas	AGTGTCCC-C	CCTTACAGAG	ATCA---CTG	CCTTGTTGTG	GCAAAGGGG-	CTTGAGTAGC	TCAGTGAAGC	243
BovB_Es-RTE-Egernia_stokesii	CAAGATTGGC	CCCTTCATGG	ATCA---CTG	CCTTGTCGTG	GCAAAGGGG-	CTTGAGTAAC	TCAATGAGGC	68
BovB_Tr-RTE-Tiliqua_rugosa	CATACCCCCC	CCCTTCATGG	ATCA---CTG	CCTTGTTGTG	GCGAAGGGG-	CTTGAGTAAC	TCAGTGAGGC	91
BovB_ALi-RTE-Amblyomma_limbatu	TATTCCGTCC	CTCTACATAG	ACTA---CTG	CCTTGTCGTG	GCGAAGGGGG	CTTGCGTAGC	TCACGGAAGC	187
BovB_Hs-RTE-Hydrophis_spiralis	----------	----------	----------	----------	----------	----------	----------	-
BovB_VA-RTE-Vipera_ammodytes	----------	-CTTCCATGG	ATTA---CTG	CCTTGTCGTG	GCGAAGGGG-	CTTGCATAAT	TCAATGAAGC	55
BovB_ACo-RTE-Agkistrodon_conto	---------A	CCCTTCATGG	ATTA---CTG	CCTTGTCGTG	GCGAAGGGG-	CTTGCGTAAC	TCAATGAAGC	57
BovB_Pg-RTE-Perameles_gunnii	----------	----------	----------	----------	----------	----------	----------	-
BovB_Io-RTE-Isoodon_obesulus	----------	----------	----------	----------	----------	----------	----------	-
BovB_Bp-RTE-Burramys_parvus	----------	----------	----------	----------	----------	----------	----------	-
BovB_Pb-RTE-Petaurus_breviceps	----------	----------	----------	----------	----------	----------	----------	-
BovB_Ma-RTE-Macropus_antilopin	---------T	CTGTAAATAT	AGGCATATGC	TACCATATGA	TGTCCTCCTT	CCTATGTATA	TGTATGCATA	61
BovB_Af-RTE-Antechinus_flavipe	----------	----------	----------	----------	----------	----------	----------	-
BovB_Ac-RTE-Anolis_carolinensi	----------	----------	----------	----------	----------	-TTGAGTGCC	TCGATGAAGC	19
BovB_BM-RTE-Bombyx_mori	----------	----------	----------	----------	----------	----------	----------	-
BovB_Dr-RTE-Christinus	----------	----------	----------	----------	----------	----------	----------	-
BovB_La-RTE-Loxodonta_africana	----------	----------	-----TTGTC	GTACTGTGGG	GGCTTGCGTG	TTGCTGTGAT	ACTGGAAGCT	45
BovB_Pc-RTE-Procavia_capensis	----------	----------	----CTTGTC	TGTCAGTGGA	GGCTTGCTTG	TTGCTGTGAT	GCAGGAAGCT	46
BovB_Et-RTE-Christinus	----------	----------	----------	----------	----------	----------	----------	-
BovB_Ta-RTE-Tachyglossus_acule	TTACCCATAA	CGGAAAAGTC	TAAGCCAAAG	AATCAGACAA	AGTTGATTCA	CCTGTGCTGG	GCAGCA-GCG	182
BovB_Plat-RTE-Ornithorhynchus	TTACCCATAA	TGGAAAGGTC	TAAGCGGAAG	CGTCAGACAA	AGTTGATTCG	CCTGTGCTGG	GCAGCA-GCG	165
BovB_Ec-RTE-Equus_caballus	----------	----------	----------	----------	----------	----------	----------	-
BovB_Cg-RTE-Christinus	----------	----------	----------	----------	----------	----------	----------	-
BovB_Bh-RTE-Bothriocroton_hydr	----------	----------	----------	----------	----------	----------	----------	-
BovB_Ml-RTE-Gehyra_variegata	----------	----------	----------	----------	----------	----------	----------	-
Sly-rnd1_family284-LINE/RTEBov	----------	----------	----------	----------	----------	----------	----------	-
Sly-rnd5_family2019-LINE/RTEBo	----------	----------	----------	----------	----------	----------	----------	-
Sly-rnd3_family322-LINE/RTEBov	----------	----------	----------	----------	----------	----------	----------	-
Sly-rnd5_family1698-LINE/RTEBo	----------	----------	----------	----------	----------	----------	----------	-
Sly-rnd1_family795-LINE/RTEBov	----------	----------	----------	----------	----------	----------	----------	-
Stu-rnd-5_family-3162_LINE_RTE	----------	----------	----------	----------	----------	----------	----------	-
Sly-rnd6_family1960-LINE/RTEBo	----------	----------	----------	----------	----------	----------	----------	-
Stu-rnd-5_family-2493_LINE_RTE	----------	----------	----------	----------	----------	----------	----------	-
Sly-rnd5_family447-LINE/RTEBov	----------	----------	----------	----------	----------	----------	----------	-
Stu-rnd-6_family-2093_LINE_RTE	----------	----------	----------	----------	----------	----------	----------	-
Consensus	----------	----------	----------	----------	----------	----------	----------	
Conservation (100% / 0%)								

```
                                       720                           740                           760
                                         |                             |                             |
Stu-rnd-1_family-417_LINE_RTE-    ---------- ---------- ---------- ---------- ---------- ---------- ---------- -
Sly-rnd1_family377-LINE/RTEBov    ---------- ---------- ---------- ---------- ---------- ---------- ---------- -
Stu-rnd-1_family-489_LINE_RTE-    ---------- ---------- ---------- ---------- ---------- ---------- ---------- -
Stu-rnd-1_family-607_LINE_RTE-    ---------- ---------- ---------- ---------- ---------- ---------- ---------- -
Stu-rnd-1_family-645_LINE_RTE-    ---------- ---------- ---------- ---------- ---------- ---------- ---------- -
Sly-rnd4_family81-LINE/RTEBovB    ---------- ---------- ---------- ---------- ---------- ---------- ---------- -
Stu-rnd-6_family-408_LINE_RTE-    ---------- ---------- ---------- ---------- ---------- ---------- ---------- -
Stu-rnd-1_family-351_LINE_RTE-    ---------- ---------- ---------- ---------- ---------- ---------- ---------- -
Stu-rnd-1_family-382_LINE_RTE-    ---------- ---------- ---------- ---------- ---------- ---------- ---------- -
Sly-rnd6_family746-LINE/RTEBov    ---------- ---------- ---------- ---------- ---------- ---------- ---------- -
Stu-rnd-5_family-325_LINE_RTE-    ---------- ---------- ---------- ---------- ---------- ---------- ---------- -
Sly-rnd4_family509-LINE/RTEBov    ---------- ---------- ---------- ---------- ---------- ---------- ---------- -
Stu-rnd-1_family-513_LINE_RTE-    ---------- ---------- ---------- ---------- ---------- ---------- ---------- -
Sly-rnd5_family1442-LINE/RTEBo    ---------- ---------- ---------- ---------- ---------- ---------- ---------- -
BovB_Gl-RTE-Gehyra_lazelli        TGTGGGCTAT GCCG--TGGA GGGCCACCCA GGA-CGGACA GGCCACAACA GAG--AACTC A-----GACA 249
BovB_Gv-RTE-Gehyra_variegata      TGTGGGCTAT GCCG--TGGA GGGCCACCCA GGA-CGGACA GGCCACAACA GAG--AACTC A-----GACA 181
BovB_PMo-RTE-Python_molurus       CATGAGCTAT ACCG--TGCA GGGCCACCCA AGA-CGGACA GGTCATGACA GAG--AGTTC T-----GACA 124
BovB_Oa-RTE-Ovis_aries            TATGAGCCAT GCCA--TGCA GGGCCACCCA AGA-TGGACG GGTCATAGTG GAG--AGTTC T-----GACA 147
BovB-RTE-Bos_taurus               TA--AGCCAT GCC---CGTG GGGCAACCCA AGA-TGGGCG GGTCATGGTG GAG--AGATC T-----GACA 754
BovB_Me-RTE-Macropus_eugenii      ---------- ---------- ---------- ---------- ---------- ---------- ---------- -
BovB_Ano-RTE-Amphibolurus_norr    TATGGGCTAT GCCG--TGCA GGGACACCCA AGA-CGGACA GGACATAGTG GAG--AGTTC C-----GACT 192
BovB_Gd-RTE-Eremiascincus_doug    ---------- ---------- ---------- ---------- ---------- ---------- ---------- -
BovB_Er-RTE-Eremiascincus_rich    AGTGAACTAT GCCATGTAGG G--TTACCCA AGA-TGGACA GGTCATAGTA GAG--AGTTC T-----GACT 312
BovB_Ca-RTE-Ctenotus_atlas        TGTGAACTAT GCCATGTAGA GATTTCCCCA AGA-TGGACA GGTCAAAGTA GAA--AGTTC T-----GACT 305
BovB_Es-RTE-Egernia_stokesii      TATGAGCTAT GCT--GTGTA GGGACACCCA AGA-TAGACA GGTCATAGCA GAGAAAGCTC TAAAGGGACT 135
BovB_Tr-RTE-Tiliqua_rugosa        TATGAGCTAT GCCA-GGGTG GGGACACCCA AGA-TGGACA GGTCATAGCA GAG--AGCTC T-----GACT 152
BovB_ALi-RTE-Amblyomma_limbatu    TATGAGCTAT GCCG--TGCA GGGTCACCCA AGA-CGGACA GGTCATAGCA GAG--AGCTC T-----TACA 247
BovB_Hs-RTE-Hydrophis_spiralis    ---------- ---------- ---------- ---------- ---------- ---------- ---------- -
BovB_VA-RTE-Vipera_ammodytes      TATGAGCTAT GCCG--TGCA GGGCCACCCA AGA-CGGAAA GGTCATAGCA GAG--AGTTC T-----GACA 115
BovB_ACo-RTE-Agkistrodon_conto    TATGAGCTAT GCCG--TGCA GGGCCACCCA AGATCGGACA GGTCATAGCA GAG--AGTTC T-----GACA 118
BovB_Pg-RTE-Perameles_gunnii      ---------- ---------- ---------- ---------- ---------- ---------- ---------- -
BovB_Io-RTE-Isoodon_obesulus      ---------- ---------- ---------- ---------- ---------- ---------- ---------- -
BovB_Bp-RTE-Burramys_parvus       ---------- ---------- ---------- ---------- ---------- ---------- ---------- -
BovB_Pb-RTE-Petaurus_breviceps    ---------- ---------- ---------- ---------- ---------- ---------- ---------- -
BovB_Ma-RTE-Macropus_antilopin    TATGTGTGCA TTCACAAGTG TGTATATATA TATCTATCCA TCTATATCTA TTTATCTCTC CTTCTAGATC 131
BovB_Af-RTE-Antechinus_flavipe    ---------- ---------- ---------- ---------- ---------- ---------- ---------- -
BovB_Ac-RTE-Anolis_carolinensi    CATGAGCTAC CG----TGCA GGGCCACCCA AGA-CGGGAA GGTCATGGCA GAG--AGGTC A-----GACT 77
BovB_BM-RTE-Bombyx_mori           ---------- ---------- ---------- ---------- ---------- ---------- ---------- -
BovB_Dr-RTE-Christinus            ---------- ---------- ---------- ---------- ---------- ---------- ---------- -
BovB_La-RTE-Loxodonta_africana    ATGCCACTGG TATTCAAATA CCAGCAGGGT CACCCATGGA AGACAGGTTT CAGCTGAGCT TCCAGACTAA 115
BovB_Pc-RTE-Procavia_capensis     ATGCCACTGG TATTTAGGTA CCAGCAGGGT CAACCATGGT AGACAAGTTT CAGCTGAGCT TCCAGACTAA 116
BovB_Et-RTE-Christinus            ---------- ---------- ---------- ---------- ---------- ---------- ---------- -
BovB_Ta-RTE-Tachyglossus_acule    GCATGGGAGA GAGTCAAAGG CAGATGAACA AATGATCAAA ATGTGGATCA AA-ACAACGG CAGAGACTCA 251
BovB_Plat-RTE-Ornithorhynchus     GCATGGGAAA GAGTCAAAGG CGGNTGATCA AATGATCAAA ATGTTGATCA AAGACAACGG CAGAGACTCA 235
BovB_Ec-RTE-Equus_caballus        ---------- ---------- ---------- ---------- ---------- ---------- ---CCATGCA 7
BovB_Cg-RTE-Christinus            ---------- ---------- ---------- ---------- ---------- ---------- ---------- -
BovB_Bh-RTE-Bothriocroton_hydr    ---------- ---------- ---------- ---------- ---------- ---------- ---------- -
BovB_Ml-RTE-Gehyra_variegata      ---------- ---------- ---------- ---------- ---------- ---------- ---------- -
Sly-rnd1_family284-LINE/RTEBov    ---------- ---------- ---------- ---------- ---------- ---------- ---------- -
Sly-rnd5_family2019-LINE/RTEBo    ---------- ---------- ---------- ---------- ---------- ---------- ---------- -
Sly-rnd3_family322-LINE/RTEBov    ---------- ---------- ---------- ---------- ---------- ---------- ---------- -
Sly-rnd5_family1698-LINE/RTEBo    ---------- ---------- ---------- ---------- ---------- ---------- ---------- -
Sly-rnd1_family795-LINE/RTEBov    ---------- ---------- ---------- ---------- ---------- ---------- ---------- -
Stu-rnd-5_family-3162_LINE_RTE    ---------- ---------- ---------- ---------- ---------- ---------- ---------- -
Sly-rnd6_family1960-LINE/RTEBo    ---------- ---------- ---------- ---------- ---------- ---------- ---------- -
Stu-rnd-5_family-2493_LINE_RTE    ---------- ---------- ---------- ---------- ---------- ---------- ---------- -
Sly-rnd5_family447-LINE/RTEBov    ---------- ---------- ---------- ---------- ---------- ---------- ---------- -
Stu-rnd-6_family-2093_LINE_RTE    ---------- ---------- ---------- ---------- ---------- ---------- ---------- -

Consensus                         ---------- ---------- ---------- ---------- ---------- ---------- ----------
```

Conservation

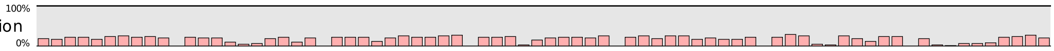

	780		800		820		840	
Stu-rnd-1_family-417_LINE_RTE-	----------	----------	----------	----------	----------	----------	----------	-
Sly-rnd1_family377-LINE/RTEBov	----------	----------	----------	----------	----------	----------	----------	-
Stu-rnd-1_family-489_LINE_RTE-	----------	----------	----------	----------	----------	----------	----------	-
Stu-rnd-1_family-607_LINE_RTE-	----------	----------	----------	----------	----------	----------	----------	-
Stu-rnd-1_family-645_LINE_RTE-	----------	----------	----------	----------	----------	----------	----------	-
Sly-rnd4_family81-LINE/RTEBovB	----------	----------	----------	----------	----------	----------	----------	-
Stu-rnd-6_family-408_LINE_RTE-	----------	----------	----------	----------	----------	----------	----------	-
Stu-rnd-1_family-351_LINE_RTE-	----------	----------	----------	----------	----------	----------	----------	-
Stu-rnd-1_family-382_LINE_RTE-	----------	----------	----------	----------	----------	----------	----------	-
Sly-rnd6_family746-LINE/RTEBov	----------	----------	----------	----------	----------	----------	----------	-
Stu-rnd-5_family-325_LINE_RTE-	----------	----------	----------	----------	----------	----------	----------	-
Sly-rnd4_family509-LINE/RTEBov	----------	----------	----------	----------	----------	----------	----------	-
Stu-rnd-1_family-513_LINE_RTE-	----------	----------	----------	----------	----------	----------	----------	-
Sly-rnd5_family1442-LINE/RTEBo	----------	----------	----------	----------	----------	----------	----------	-
BovB_Gl-RTE-Gehyra_lazelli	AAATGTGATC	CAC-TGGAGG	AGGAAATGGC	AA-CCCACTC	CAGTATCTTT	GCCAAGAAAA	CCCTATGGAC	317
BovB_Gv-RTE-Gehyra_variegata	AAATGTGATC	CAC-TGGAGG	AGGAAACGGC	AA-CCCACTC	CAGTATCTTT	GCCAAGAAAA	CCCTATGGAC	249
BovB_PMo-RTE-Python_molurus	AAATGTGGTC	CAC-TGGAGA	AGGAAATGGC	AA-CCCACTC	CAGTATCCTT	GCCAAGAAAA	CCCCATGGAC	192
BovB_Oa-RTE-Ovis_aries	AAATGTGGTC	CAC-TGGAGA	AGGGAATGGC	AA-ACCACTC	CAGTATTCTT	GCCTTGAGAA	CCCCATGAAC	215
BovB-RTE-Bos_taurus	GAATGTGGTC	CAC-TGGAGA	AGGGAATGGC	AA-ACCACTT	CAGTATTCTT	GCCTTGAGAA	CCCCATGAAC	822
BovB_Me-RTE-Macropus_eugenii	----------	----------	----------	----------	---TATCTTT	GCCA-GGAAA	GCCCATGGAC	26
BovB_Ano-RTE-Amphibolurus_norr	AAACGCAATC	CACCTGGAGT	AGGAACTGGC	AATGCCACTC	CAGTATCTTT	GCCAAGAAAA	CCCCATGATC	262
BovB_Gd-RTE-Eremiascincus_doug	----------	----------	----------	----------	----------	----------	----------	-
BovB_Er-RTE-Eremiascincus_rich	AAACGTGATC	CGC-TGGAGA	AGGAAATAGG	CAACCCACTC	CAGTACTCTT	GCCAAGAAAA	CCCCATGAAC	381
BovB_Ca-RTE-Ctenotus_atlas	AAACGTGATC	CGC-TGGAGA	AGGAAATGGG	CAACCCACTC	CAGTACTCTT	GCCAAGAAAA	CCCCATGGAT	374
BovB_Es-RTE-Egernia_stokesii	AAACGTGATC	CAC-TGAAGA	AGGAAAT-GA	CAACCCACTC	CAGCATCCTT	ACCAAGAAAA	CCCTATGAAC	203
BovB_Tr-RTE-Tiliqua_rugosa	AAACGTGATC	CAC-TGGAGA	AGGAAAT-GG	CAACCCACTC	CAGTATCCTT	GCCAAGAAAA	CCCTATGGAC	220
BovB_ALi-RTE-Amblyomma_limbatu	AAACATGATT	CAA-TAGAGA	AGGAAATGGC	AA-CCCACTC	CAATATCTCT	GCCATGAAAA	CTCCATCGAT	315
BovB_Hs-RTE-Hydrophis_spiralis	----------	----------	----------	----------	----------	----------	----------	-
BovB_VA-RTE-Vipera_ammodytes	AAACGTGATC	CAC-TGGAGA	AGGAAATGGC	AA-CCCACTC	CAGTATCTTT	GCCATGAAAA	CCCTATGGAC	183
BovB_ACo-RTE-Agkistrodon_conto	AAATGTGATC	CAC-TGGAGA	AGGAAATGGC	AA-CCCACTC	CAGTATCTTT	GCCATGAAAA	CCCCATGGAC	186
BovB_Pg-RTE-Perameles_gunnii	----------	----------	----------	----------	----------	----------	----------	-
BovB_Io-RTE-Isoodon_obesulus	----------	----------	----------	----------	----------	----------	----------	-
BovB_Bp-RTE-Burramys_parvus	----------	----------	----------	----------	----------	----------	----------	-
BovB_Pb-RTE-Petaurus_breviceps	----------	----------	----------	----------	----------	----------	----------	-
BovB_Ma-RTE-Macropus_antilopin	ACTGCCTCTC	CTTCTGAATC	ACTGCCTTGT	TGAGGTGGAG	GGGCTTGCAT	AGCTCAATGA	AGCTATGAGC	201
BovB_Af-RTE-Antechinus_flavipe	----------	----------	----------	----------	----------	----------	----------	-
BovB_Ac-RTE-Anolis_carolinensi	AAATACGATC	CCT--GGGGA	AGGTAATGGC	AA-CCCACCC	CAGTATTCTT	GCCATGAAAA	CTACATGGAT	144
BovB_BM-RTE-Bombyx_mori	----------	----------	----------	----------	----------	----------	---CCAAGAA	7
BovB_Dr-RTE-Christinus	----------	----TGAGGA	AAGCAATGGC	AAACTACCTC	ACAATTTTTG	ACAA--AAAA	CTTTATGGAT	54
BovB_La-RTE-Loxodonta_africana	GACAG--ACT	AGGAAGAAGG	ACCCGGAAGT	CTACTTCTGA	AAAGAATTAG	CCAGTGAAAA	CCTTATGAAT	183
BovB_Pc-RTE-Procavia_capensis	GACAG--ACT	AGGAAGAAGG	ACCTGGCAGT	CTACTTCTGA	AAAGAATTAG	CCAGGGAAAA	CCTTATGAAT	184
BovB_Et-RTE-Christinus	----------	----------	----------	----------	-AAGTCCTA-	TGAATGGAAA	CAACATG---	25
BovB_Ta-RTE-Tachyglossus_acule	GGTTTTCACT	GCGCGGAAGG	AGGCAATGGT	AAACCACTTC	TTTACTTTTA	CCAA-GAAAA	CTCTATGGAT	320
BovB_Plat-RTE-Ornithorhynchus	AGTTTTCACT	GCGCGGAAGG	AGGCAACGGT	AAACCGCTTC	CGTATTTTTA	CCAA-GAAAA	CTCTATGGAT	304
BovB_Ec-RTE-Equus_caballus	GCAGAAGGCA	ATGGTAAACC	ACTGCTGTAT	TTTCCTTTTT	TTTTTTTTTT	ACCTTGAAAA	CCCTATGATG	77
BovB_Cg-RTE-Christinus	----------	----------	----------	----------	----------	----------	----------	-
BovB_Bh-RTE-Bothriocroton_hydr	----------	----------	----------	----------	----------	----------	----------	-
BovB_Ml-RTE-Gehyra_variegata	----------	----------	----------	----------	----------	----------	----------	-
Sly-rnd1_family284-LINE/RTEBov	----------	----------	----------	----------	----------	----------	----------	-
Sly-rnd5_family2019-LINE/RTEBo	----------	----------	----------	----------	----------	----------	----------	-
Sly-rnd3_family322-LINE/RTEBov	----------	----------	----------	----------	----------	----------	----------	-
Sly-rnd5_family1698-LINE/RTEBo	----------	----------	----------	----------	----------	----------	----------	-
Sly-rnd1_family795-LINE/RTEBov	----------	----------	----------	----------	----------	----------	----------	-
Stu-rnd-5_family-3162_LINE_RTE	----------	----------	----------	----------	----------	----------	----------	-
Sly-rnd6_family1960-LINE/RTEBo	----------	----------	----------	----------	----------	----------	----------	-
Stu-rnd-5_family-2493_LINE_RTE	----------	----------	----------	----------	----------	----------	----------	-
Sly-rnd5_family447-LINE/RTEBov	----------	----------	----------	----------	----------	----------	----------	-
Stu-rnd-6_family-2093_LINE_RTE	----------	----------	----------	----------	----------	----------	----------	-
Consensus	----------	----------	----------	----------	----------	----------	----------	

Conservation

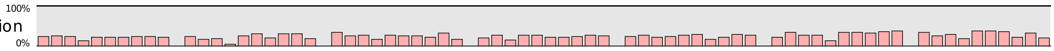

		860		880		900		
Stu-rnd-1_family-417_LINE_RTE-	----------	----------	----------	----------	----------	----------	----------	-
Sly-rnd1_family377-LINE/RTEBov	----------	----------	----------	----------	----------	----------	----------	-
Stu-rnd-1_family-489_LINE_RTE-	----------	----------	----------	----------	----------	----------	----------	-
Stu-rnd-1_family-607_LINE_RTE-	----------	----------	----------	----------	----------	----------	----------	-
Stu-rnd-1_family-645_LINE_RTE-	----------	----------	----------	----------	----------	----------	----------	-
Sly-rnd4_family81-LINE/RTEBovB	----------	----------	----------	----------	----------	----------	----------	-
Stu-rnd-6_family-408_LINE_RTE-	----------	----------	----------	----------	----------	----------	----------	-
Stu-rnd-1_family-351_LINE_RTE-	----------	----------	----------	----------	----------	----------	----------	-
Stu-rnd-1_family-382_LINE_RTE-	----------	----------	----------	----------	----------	----------	----------	-
Sly-rnd6_family746-LINE/RTEBov	----------	----------	----------	----------	----------	----------	----------	-
Stu-rnd-5_family-325_LINE_RTE-	----------	----------	----------	----------	----------	----------	----------	-
Sly-rnd4_family509-LINE/RTEBov	----------	----------	----------	----------	----------	----------	----------	-
Stu-rnd-1_family-513_LINE_RTE-	----------	----------	----------	----------	----------	----------	----------	-
Sly-rnd5_family1442-LINE/RTEBo	----------	----------	----------	----------	----------	----------	----------	-
BovB_Gl-RTE-Gehyra_lazelli	---AGAAACA	AAAGGCTAAA	AGATATGACG	CTGGAAGATG	AGCCCCTCAG	GTCGGAAGGT	GTCAG--ATA	382
BovB_Gv-RTE-Gehyra_variegata	---AGAAACA	AAAGGCTAAA	AGATATGACG	CCGGAAGATG	AGCCCCTCAG	GTCGGAAGGT	GTCAG--ATA	314
BovB_PMo-RTE-Python_molurus	---AATGTTA	AAAGGCTAAA	AGATATGACG	CCGGAAGATG	AGCCCCTCAG	GTCGGAAGGT	GTCCA--ATA	257
BovB_Oa-RTE-Ovis_aries	---AGTATGA	AAAGGCAAAA	AGATATGACA	CTGAAAGATG	AGCCCCCCAG	GTCAGAAGGT	GTCCA--ATA	280
BovB-RTE-Bos_taurus	---AGTATGA	AAAGGCAAAA	TGATAGGATA	CTGAAAGAGG	AACTCCCCAG	GTCAGTAGGT	GCCCA--ATA	887
BovB_Me-RTE-Macropus_eugenii	---AGTATGA	AAGTGATACA	ATATGTGATG	CTAGAAGCTG	AGCCCCTCAG	GTTGG-AAGT	GTCCA--ACA	90
BovB_Ano-RTE-Amphibolurus_norr	---AGAAACA	AAAGGCTAAA	AGATATGACG	CTGGAAGATG	GGCCCCTCAG	GTCGGAAGGC	GTCCA--ACA	327
BovB_Gd-RTE-Eremiascincus_doug	----------	----------	----------	----------	----------	----------	----------	-
BovB_Er-RTE-Eremiascincus_rich	TACAGTACTA	AAAGGCTAAA	CGATATGACA	CTGGAAGATG	AGCCCCTCAG	GTCGGAAGGT	GTCCA--TTA	449
BovB_Ca-RTE-Ctenotus_atlas	TGCAAAACTA	AAAGGCTAAA	CGATATGACA	CTGGAAGATG	AGACCCCCAG	GTCGGAAGGT	GTCCA--TTA	442
BovB_Es-RTE-Egernia_stokesii	TTAAGTATCA	AAAGGCTAAA	AGATATGATG	CAAGAAGATG	AGCCCCTCAG	GTCAGAAGGC	ATCCA--ATA	271
BovB_Tr-RTE-Tiliqua_rugosa	TTTTGTATCA	AAAGGCTAAA	AGATACGATG	CTGGAAGATG	AGCCCCTCAG	GTCAGAAGGC	ATCCA--ATA	288
BovB_ALi-RTE-Amblyomma_limbatu	---ACAACCA	AAAAGCAAAA	AGATATGACA	TAAAAAGATC	AGCCATTCAG	GTCTGAAGGT	GTCCA--ATA	380
BovB_Hs-RTE-Hydrophis_spiralis	----------	----------	----------	----------	----------	----------	----------	-
BovB_VA-RTE-Vipera_ammodytes	---AGTACCA	AAAGGCAATA	CGATATGACG	CTGGAAGATG	AGCCCCTCAG	GTCGGAAGGC	ATCCA--ATA	248
BovB_ACo-RTE-Agkistrodon_conto	---AGTACCA	AAAGGCAAAA	AGATATGACG	CTGGAAGATG	AGCCCCTCAG	GTCGGAAGGT	GTCCA--ATA	251
BovB_Pg-RTE-Perameles_gunnii	----------	----------	----------	----------	----------	----------	----------	-
BovB_Io-RTE-Isoodon_obesulus	----------	----------	----------	----------	----------	----------	----------	-
BovB_Bp-RTE-Burramys_parvus	----------	----------	----------	----------	----------	----------	----------	-
BovB_Pb-RTE-Petaurus_breviceps	----------	----------	----------	----------	----------	----------	----------	-
BovB_Ma-RTE-Macropus_antilopin	TATAGTACCC	AAGATGGACA	GGTCATAGTG	GAGAGTTCTG	ACACAAGGTG	ATCCACTTGA	GAAGG--AAA	269
BovB_Af-RTE-Antechinus_flavipe	----------	----------	----------	----------	----------	----------	----------	-
BovB_Ac-RTE-Anolis_carolinensi	CAGTACAACC	AGAGAAATGT	CGGTATACCA	TCAGAAGATA	GGACCCCCAG	GTCGGAAGAT	GACCA--AAA	212
BovB_BM-RTE-Bombyx_mori	A---ACCTCA	TGATGTCGAC	AGATATATTA	TTTGATGTTC	TCTCATGCGA	CCCGACTGAC	CACCGGCGCC	74
BovB_Dr-RTE-Christinus	C---ACAACT	CAATGCTTTC	AAATATGGTG	CTGGAAGGTA	GGCCCCCTGG	GCTGGAAGGC	ATCT--CACG	119
BovB_La-RTE-Loxodonta_africana	A---GCAGCG	GAACATTGTC	TGATATAGTG	CTGGAAGATG	AGCCCCCCAG	GTTGGAAGGC	ACTCA--AAA	248
BovB_Pc-RTE-Procavia_capensis	A---GCAGCT	GAACATTGTC	AGATATGGTG	CCGGAAAGTG	AGCCCCCCAG	GTTGGAAGGC	ACTCA--GAA	249
BovB_Et-RTE-Christinus	-----TTGTA	TATCACTGTG	AGATATATTG	TCAGAGGATG	GGACCCTTGG	GTCTGACAGC	ACTCA--AAA	88
BovB_Ta-RTE-Tachyglossus_acule	ACACATACCA	GAATGACTAC	AGATATAGTG	CTGGAAGATG	AGCCCCTCAG	GTTGGAAGGC	ACTCA--ACG	388
BovB_Plat-RTE-Ornithorhynchus	ACACATACCA	GAACGACTAC	AGATATAGTG	CTGGAAGATG	AGCCCCTCAG	GTCGGAAGGC	ACTCA--ACG	372
BovB_Ec-RTE-Equus_caballus	AGACAGTCCA	AAATGAAATA	TGAGATAGTG	CTGGAAGATG	AGACCCCCAG	GTCAGAAGGC	ACTCA--AAC	145
BovB_Cg-RTE-Christinus	----------	----------	----------	----------	----------	----------	----------	-
BovB_Bh-RTE-Bothriocroton_hydr	----------	----------	----------	----------	----------	----------	----------	-
BovB_Ml-RTE-Gehyra_variegata	----------	----------	----------	----------	----------	----------	----------	-
Sly-rnd1_family284-LINE/RTEBov	----------	----------	----------	----------	----------	----------	----------	-
Sly-rnd5_family2019-LINE/RTEBo	----------	----------	----------	----------	----------	----------	----------	-
Sly-rnd3_family322-LINE/RTEBov	----------	----------	----------	----------	----------	----------	----------	-
Sly-rnd5_family1698-LINE/RTEBo	----------	----------	----------	----------	----------	----------	----------	-
Sly-rnd1_family795-LINE/RTEBov	----------	----------	----------	----------	----------	----------	----------	-
Stu-rnd-5_family-3162_LINE_RTE	----------	----------	----------	----------	----------	----------	----------	-
Sly-rnd6_family1960-LINE/RTEBo	----------	----------	----------	----------	----------	----------	----------	-
Stu-rnd-5_family-2493_LINE_RTE	----------	----------	----------	----------	----------	----------	----------	-
Sly-rnd5_family447-LINE/RTEBov	----------	----------	----------	----------	----------	----------	----------	-
Stu-rnd-6_family-2093_LINE_RTE	----------	----------	----------	----------	----------	----------	----------	-
Consensus	----------	----------	----------	----------	----------	----------	----------	

Conservation

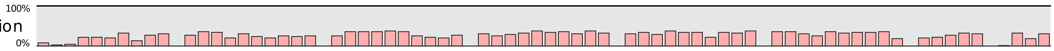

	920		940		960		980	
Stu-rnd-1_family-417_LINE_RTE-	----------	----------	----------	----------	----------	----------	----------	-
Sly-rnd1_family377-LINE/RTEBov	----------	----------	----------	----------	----------	----------	----------	-
Stu-rnd-1_family-489_LINE_RTE-	----------	----------	----------	----------	----------	----------	----------	-
Stu-rnd-1_family-607_LINE_RTE-	----------	----------	----------	----------	----------	----------	----------	-
Stu-rnd-1_family-645_LINE_RTE-	----------	----------	----------	----------	----------	----------	----------	-
Sly-rnd4_family81-LINE/RTEBovB	----------	----------	----------	----------	----------	----------	----------	-
Stu-rnd-6_family-408_LINE_RTE-	----------	----------	----------	----------	----------	----------	----------	-
Stu-rnd-1_family-351_LINE_RTE-	----------	----------	----------	----------	----------	----------	----------	-
Stu-rnd-1_family-382_LINE_RTE-	----------	----------	----------	----------	----------	----------	----------	-
Sly-rnd6_family746-LINE/RTEBov	----------	----------	----------	----------	----------	----------	----------	-
Stu-rnd-5_family-325_LINE_RTE-	----------	----------	----------	----------	----------	----------	----------	-
Sly-rnd4_family509-LINE/RTEBov	----------	----------	----------	----------	----------	----------	----------	-
Stu-rnd-1_family-513_LINE_RTE-	----------	----------	----------	----------	----------	----------	----------	-
Sly-rnd5_family1442-LINE/RTEBo	----------	----------	----------	----------	----------	----------	----------	-
BovB_Gl-RTE-Gehyra_lazelli	TGCTACTGGG	GAAGAGCAGA	GGGCAAGTAC	AAGTAACCCC	A--GAAAGAA	TGAAGCGGCT	GG-GCCAAAG	449
BovB_Gv-RTE-Gehyra_variegata	TGCTACTGGG	GAAGAGCAGA	GGGCAAGTAC	AAGTAACCCC	A--GAAAGAA	TGAAGCGGCT	GG-GCCAAAG	381
BovB_PMo-RTE-Python_molurus	TGCTACTGGG	GAAGAGCGGA	GGGCAATTCC	AAGTAGCTCC	A--GAAAGAG	TGAAGCGGCT	GG-GCCAAAG	324
BovB_Oa-RTE-Ovis_aries	TGCTACTGGA	GAAGAGTGGA	GAGCAATTAC	TAATAGCTCC	A--GAAAGAA	TGAAGAGGCT	GG-GCCAAAG	347
BovB-RTE-Bos_taurus	TGCTACTGGA	GATCAGTGGA	GA--------	-AATAACTCC	A--GAAAGAA	TGAAGGGATG	GA-GCCAAAG	945
BovB_Me-RTE-Macropus_eugenii	TGCTACTGGA	GAGAAG-AGA	GTACAACTCC	AAGTAACTTC	A--GTACTAA	TGAAGTGGAT	GG-GCTAAAG	156
BovB_Ano-RTE-Amphibolurus_norr	TGCTACTGAG	GAAGAGCGGA	GGACAAGTAC	AAGTAGCTCC	A--GAGCTAA	TGAAGTGGTT	GG-GCCAAAG	394
BovB_Gd-RTE-Eremiascincus_doug	----------	----------	----------	----------	----------	----------	----------	-
BovB_Er-RTE-Eremiascincus_rich	TGCTACTGGG	GAAGAGCAGA	ACACGAGTAC	TAGTAGCTCC	A--GAAAGAG	TGAAGTAGCT	GG-GCCAAAA	516
BovB_Ca-RTE-Ctenotus_atlas	TGCTACTGGG	GAAGAGCAGA	ACACGAGTAC	TAGTAGCTCC	A--GAAAGAG	TGAAGTAGCT	GG-GCCAAAC	509
BovB_Es-RTE-Egernia_stokesii	TGCTACTGGG	GAAGAGCAGA	GGACAATTAC	AAGTAGCCCC	A--GAAAGAA	TGAAGGAGCT	GG-GCCAAAA	338
BovB_Tr-RTE-Tiliqua_rugosa	TGCTACTGGG	GAAGAGTGGA	AGACAATTAC	AAGTAGCCCC	A--GAAAGAA	TGAAGGAGCT	GG-GCCAAAA	355
BovB_ALi-RTE-Amblyomma_limbatu	TGCTATTGGG	GAAGAGCGGA	GGACTAGTAC	TAGTAGCGCA	A--AAAAAAA	ATAAGCGACT	GC-GTCAAAG	447
BovB_Hs-RTE-Hydrophis_spiralis	----------	----------	----------	----------	----------	----------	----------	-
BovB_VA-RTE-Vipera_ammodytes	TGCTACTGGG	GAAGAGCAGA	GGGCTACTAC	TAGTAGCCCC	A--GAAAGAA	TGAAGCGACT	GG-GCCAAAG	315
BovB_ACo-RTE-Agkistrodon_conto	TGCTACTGGG	GAAGAGCAGA	GGGCTAGTAC	TAGTAGCGCC	A--GAAAGAG	TGAAGCGACT	GG-GCCAAAG	318
BovB_Pg-RTE-Perameles_gunnii	----------	----------	----------	----------	----------	----------	----------	-
BovB_Io-RTE-Isoodon_obesulus	----------	----------	----------	----------	----------	----------	----------	-
BovB_Bp-RTE-Burramys_parvus	----------	----------	----------	----------	----------	----------	----------	-
BovB_Pb-RTE-Petaurus_breviceps	----------	----------	----------	----------	----------	----------	----------	-
BovB_Ma-RTE-Macropus_antilopin	TGGCAAACCA	CTTCAGTATC	TTTGCCAAGA	AAAGTCCCAT	G--AACAGTC	TAAAAAGATA	GG-ACAACAG	336
BovB_Af-RTE-Antechinus_flavipe	----------	----------	----------	----------	----------	----------	----------	-
BovB_Ac-RTE-Anolis_carolinensi	TGCTACTGGG	GAGGAACAGA	GGACTAGTCC	AACTAACCCC	A--GATGTGA	TGACACAGTT	AA-CTCAAAG	279
BovB_BM-RTE-Bombyx_mori	ACCCGCTACC	CGGGGCGAG-	TGGTGTGAAA	TTGGCTACCG	AAGCTTCCCA	TGAAGTGACT	CG--TCAGG-	140
BovB_Dr-RTE-Christinus	ACCTACCAGG	GAAGAACAA-	GGGTGTTTTT	CAAGTAGCTC	GGGCATTATT	GACGCGGATT	GG--ACAAAG	186
BovB_La-RTE-Loxodonta_africana	GACAACTGGA	GAAGAGC---	TGCCTCCTCA	AAGTAGAGCC	A--CCTTAAT	CAACGTGGAT	GG-AGTAAAG	312
BovB_Pc-RTE-Procavia_capensis	GATGACTGGG	GAAGAGC---	TGCCTCCTCA	AAGTAGAGTC	G--ACCTTAA	TGACGTGGAT	GG-AGTAAGG	313
BovB_Et-RTE-Christinus	TGTGAGTGAA	AAAGA-----	TGCTTTCTCC	AAGTGGTGCT	GTGAGTTGGA	TAAAGCTTGT	GGGAATGGAG	153
BovB_Ta-RTE-Tachyglossus_acule	AGCTACTGGA	GATGAGCAGA	CGATGTCTCA	GAGTATCATG	T--GTGTTTA	TGACGTGGCC	AG-AATAAAG	455
BovB_Plat-RTE-Ornithorhynchus	AGCTACTGGG	GAAGAGCAGA	CGATGTCTCA	GAGTATCGTG	G--GTGTTTA	TGACGTGGCC	GG-AATAAAG	439
BovB_Ec-RTE-Equus_caballus	AGTTACTGGG	GAAGAGCAAA	GAACAAGTAC	GAATAGCACT	G--TTGCTAA	TGATGCAACT	AG-ACTAAAG	212
BovB_Cg-RTE-Christinus	----------	----------	----------	----------	----------	----------	----------	-
BovB_Bh-RTE-Bothriocroton_hydr	----------	----------	----------	----------	----------	----------	----------	-
BovB_Ml-RTE-Gehyra_variegata	----------	----------	----------	----------	----------	----------	----------	-
Sly-rnd1_family284-LINE/RTEBov	----------	----------	----------	----------	----------	----------	----------	-
Sly-rnd5_family2019-LINE/RTEBo	----------	----------	----------	----------	----------	----------	----------	-
Sly-rnd3_family322-LINE/RTEBov	----------	----------	----------	----------	----------	----------	----------	-
Sly-rnd5_family1698-LINE/RTEBo	----------	----------	----------	----------	----------	----------	----------	-
Sly-rnd1_family795-LINE/RTEBov	----------	----------	----------	----------	----------	----------	----------	-
Stu-rnd-5_family-3162_LINE_RTE	----------	----------	----------	----------	----------	----------	----------	-
Sly-rnd6_family1960-LINE/RTEBo	----------	----------	----------	----------	----------	----------	----------	-
Stu-rnd-5_family-2493_LINE_RTE	----------	----------	----------	----------	----------	----------	----------	-
Sly-rnd5_family447-LINE/RTEBov	----------	----------	----------	----------	----------	----------	----------	-
Stu-rnd-6_family-2093_LINE_RTE	----------	----------	----------	----------	----------	----------	----------	-
Consensus	----------	----------	----------	----------	----------	----------	----------	

Conservation

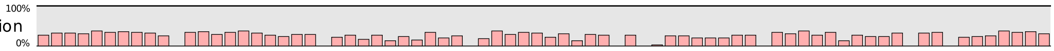

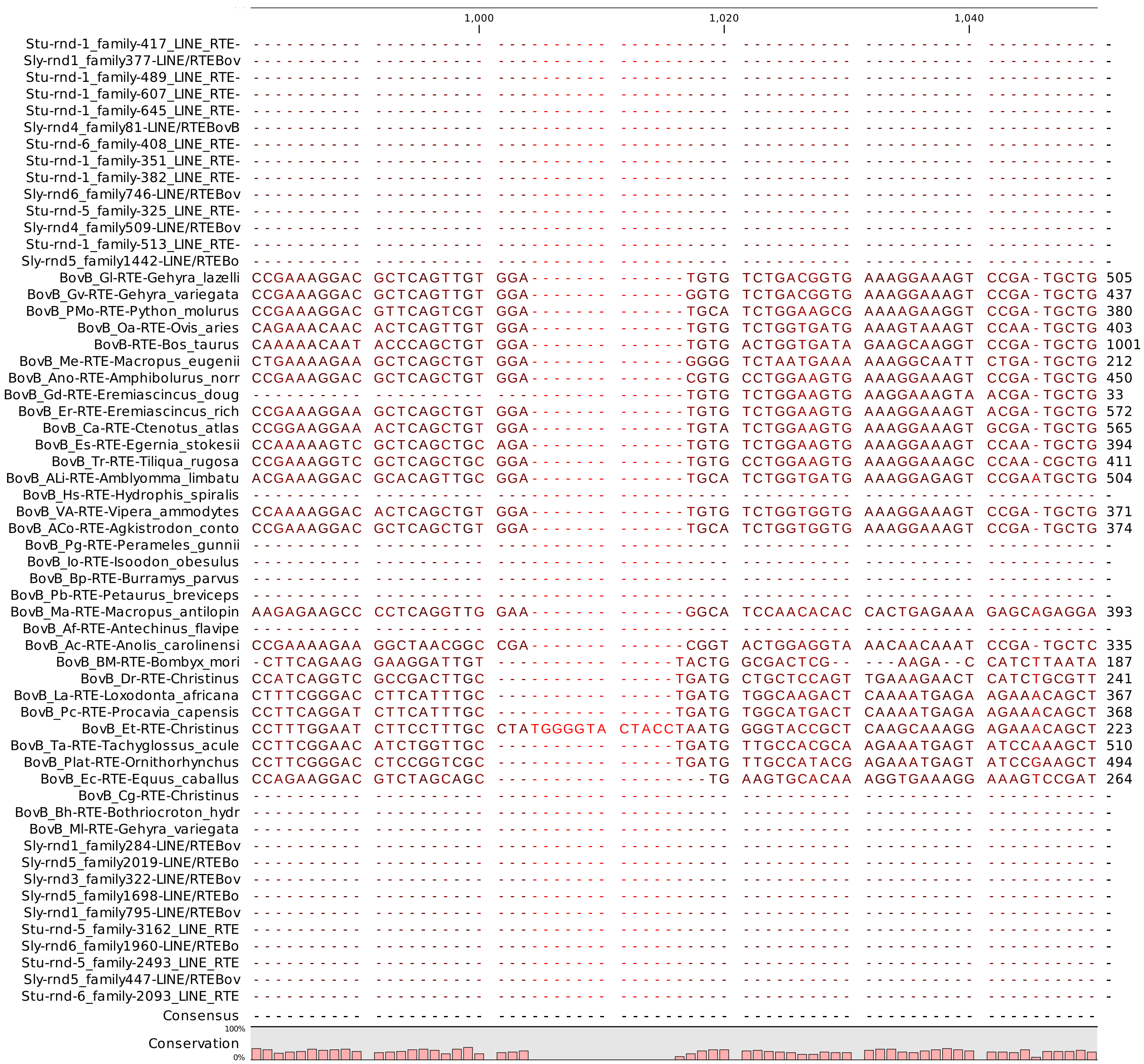
1,000 1,020 1,040
Stu-rnd-1_family-417_LINE_RTE- ---------- ---------- ---------- ---------- ---------- ---------- ---------- -
Sly-rnd1_family377-LINE/RTEBov ---------- ---------- ---------- ---------- ---------- ---------- ---------- -
Stu-rnd-1_family-489_LINE_RTE- ---------- ---------- ---------- ---------- ---------- ---------- ---------- -
Stu-rnd-1_family-607_LINE_RTE- ---------- ---------- ---------- ---------- ---------- ---------- ---------- -
Stu-rnd-1_family-645_LINE_RTE- ---------- ---------- ---------- ---------- ---------- ---------- ---------- -
Sly-rnd4_family81-LINE/RTEBovB ---------- ---------- ---------- ---------- ---------- ---------- ---------- -
Stu-rnd-6_family-408_LINE_RTE- ---------- ---------- ---------- ---------- ---------- ---------- ---------- -
Stu-rnd-1_family-351_LINE_RTE- ---------- ---------- ---------- ---------- ---------- ---------- ---------- -
Stu-rnd-1_family-382_LINE_RTE- ---------- ---------- ---------- ---------- ---------- ---------- ---------- -
Sly-rnd6_family746-LINE/RTEBov ---------- ---------- ---------- ---------- ---------- ---------- ---------- -
Stu-rnd-5_family-325_LINE_RTE- ---------- ---------- ---------- ---------- ---------- ---------- ---------- -
Sly-rnd4_family509-LINE/RTEBov ---------- ---------- ---------- ---------- ---------- ---------- ---------- -
Stu-rnd-1_family-513_LINE_RTE- ---------- ---------- ---------- ---------- ---------- ---------- ---------- -
Sly-rnd5_family1442-LINE/RTEBo ---------- ---------- ---------- ---------- ---------- ---------- ---------- -
BovB_Gl-RTE-Gehyra_lazelli CCGAAAGGAC GCTCAGTTGT GGA------- ------TGTG TCTGACGGTG AAAGGAAAGT CCGA-TGCTG 505
BovB_Gv-RTE-Gehyra_variegata CCGAAAGGAC GCTCAGTTGT GGA------- ------GGTG TCTGACGGTG AAAGGAAAGT CCGA-TGCTG 437
BovB_PMo-RTE-Python_molurus CCGAAAGGAC GTTCAGTCGT GGA------- ------TGCA TCTGGAAGCG AAAAGAAGGT CCGA-TGCTG 380
BovB_Oa-RTE-Ovis_aries CAGAAACAAC ACTCAGTTGT GGA------- ------TGTG TCTGGTGATG AAAGTAAAGT CCAA-TGCTG 403
BovB-RTE-Bos_taurus CAAAAACAAT ACCCAGCTGT GGA------- ------TGTG ACTGGTGATA GAAGCAAGGT CCGA-TGCTG 1001
BovB_Me-RTE-Macropus_eugenii CTGAAAAGAA GCTCAGCTGT GGA------- ------GGGG TCTAATGAAA AAAGGCAATT CTGA-TGCTG 212
BovB_Ano-RTE-Amphibolurus_norr CCGAAAGGAC GCTCAGCTGT GGA------- ------CGTG CCTGGAAGTG AAAGGAAAGT CCGA-TGCTG 450
BovB_Gd-RTE-Eremiascincus_doug ---------- ---------- ---------- ------TGTG TCTGGAAGTG AAGGAAAGTA ACGA-TGCTG 33
BovB_Er-RTE-Eremiascincus_rich CCGAAAGGAA GCTCAGCTGT GGA------- ------TGTG TCTGGAAGTG AAAGGAAAGT ACGA-TGCTG 572
BovB_Ca-RTE-Ctenotus_atlas CCGGAAGGAA ACTCAGCTGT GGA------- ------TGTA TCTGGAAGTG AAAGGAAAGT GCGA-TGCTG 565
BovB_Es-RTE-Egernia_stokesii CCAAAAAGTC GCTCAGCTGC AGA------- ------TGTG TCTGGAAGTG AAAGGAAAGT CCAA-TGCTG 394
BovB_Tr-RTE-Tiliqua_rugosa CCGAAAGGTC GCTCAGCTGC GGA------- ------TGTG CCTGGAAGTG AAAGGAAAGC CCAA-CGCTG 411
BovB_ALi-RTE-Amblyomma_limbatu ACGAAAGGAC GCACAGTTGC GGA------- ------TGCA TCTGGTGATG AAAGGAGAGT CCGAATGCTG 504
BovB_Hs-RTE-Hydrophis_spiralis ---------- ---------- ---------- ---------- ---------- ---------- ---------- -
BovB_VA-RTE-Vipera_ammodytes CCAAAAGGAC ACTCAGCTGT GGA------- ------TGTG TCTGGTGGTG AAAGGAAAGT CCGA-TGCTG 371
BovB_ACo-RTE-Agkistrodon_conto CCGAAAGGAC GCTCAGCTGT GGA------- ------TGCA TCTGGTGGTG AAAGGAAAGT CCGA-TGCTG 374
BovB_Pg-RTE-Perameles_gunnii ---------- ---------- ---------- ---------- ---------- ---------- ---------- -
BovB_Io-RTE-Isoodon_obesulus ---------- ---------- ---------- ---------- ---------- ---------- ---------- -
BovB_Bp-RTE-Burramys_parvus ---------- ---------- ---------- ---------- ---------- ---------- ---------- -
BovB_Pb-RTE-Petaurus_breviceps ---------- ---------- ---------- ---------- ---------- ---------- ---------- -
BovB_Ma-RTE-Macropus_antilopin AAGAGAAGCC CCTCAGGTTG GAA------- ------GGCA TCCAACACAC CACTGAGAAA GAGCAGAGGA 393
BovB_Af-RTE-Antechinus_flavipe ---------- ---------- ---------- ---------- ---------- ---------- ---------- -
BovB_Ac-RTE-Anolis_carolinensi CCGAAAAGAA GGCTAACGGC CGA------- ------CGGT ACTGGAGGTA AACAACAAAT CCGA-TGCTC 335
BovB_BM-RTE-Bombyx_mori -CTTCAGAAG GAAGGATTGT ---------- -----TACTG GCGACTCG-- ---AAGA--C CATCTTAATA 187
BovB_Dr-RTE-Christinus CCATCAGGTC GCCGACTTGC ---------- -----TGATG CTGCTCCAGT TGAAAGAACT CATCTGCGTT 241
BovB_La-RTE-Loxodonta_africana CTTTCGGGAC CTTCATTTGC ---------- -----TGATG TGGCAAGACT CAAAATGAGA AGAAACAGCT 367
BovB_Pc-RTE-Procavia_capensis CCTTCAGGAT CTTCATTTGC ---------- -----TGATG TGGCATGACT CAAAATGAGA AGAAACAGCT 368
BovB_Et-RTE-Christinus CCTTTGGAAT CTTCCTTTGC CTATGGGGTA CTACCTAATG GGGTACCGCT CAAGCAAAGG AGAAACAGCT 223
BovB_Ta-RTE-Tachyglossus_acule CCTTCGGAAC ATCTGGTTGC ---------- -----TGATG TTGCCACGCA AGAAATGAGT ATCCAAAGCT 510
BovB_Plat-RTE-Ornithorhynchus CCTTCGGGAC CTCCGGTCGC ---------- -----TGATG TTGCCATACG AGAAATGAGT ATCCGAAGCT 494
BovB_Ec-RTE-Equus_caballus CCAGAAGGAC GTCTAGCAGC ---------- --------TG AAGTGCACAA AGGTGAAAGG AAAGTCCGAT 264
BovB_Cg-RTE-Christinus ---------- ---------- ---------- ---------- ---------- ---------- ---------- -
BovB_Bh-RTE-Bothriocroton_hydr ---------- ---------- ---------- ---------- ---------- ---------- ---------- -
BovB_Ml-RTE-Gehyra_variegata ---------- ---------- ---------- ---------- ---------- ---------- ---------- -
Sly-rnd1_family284-LINE/RTEBov ---------- ---------- ---------- ---------- ---------- ---------- ---------- -
Sly-rnd5_family2019-LINE/RTEBo ---------- ---------- ---------- ---------- ---------- ---------- ---------- -
Sly-rnd3_family322-LINE/RTEBov ---------- ---------- ---------- ---------- ---------- ---------- ---------- -
Sly-rnd5_family1698-LINE/RTEBo ---------- ---------- ---------- ---------- ---------- ---------- ---------- -
Sly-rnd1_family795-LINE/RTEBov ---------- ---------- ---------- ---------- ---------- ---------- ---------- -
Stu-rnd-5_family-3162_LINE_RTE ---------- ---------- ---------- ---------- ---------- ---------- ---------- -
Sly-rnd6_family1960-LINE/RTEBo ---------- ---------- ---------- ---------- ---------- ---------- ---------- -
Stu-rnd-5_family-2493_LINE_RTE ---------- ---------- ---------- ---------- ---------- ---------- ---------- -
Sly-rnd5_family447-LINE/RTEBov ---------- ---------- ---------- ---------- ---------- ---------- ---------- -
Stu-rnd-6_family-2093_LINE_RTE ---------- ---------- ---------- ---------- ---------- ---------- ---------- -
Consensus ---------- ---------- ---------- ---------- ---------- ---------- ----------
100%
Conservation
0%

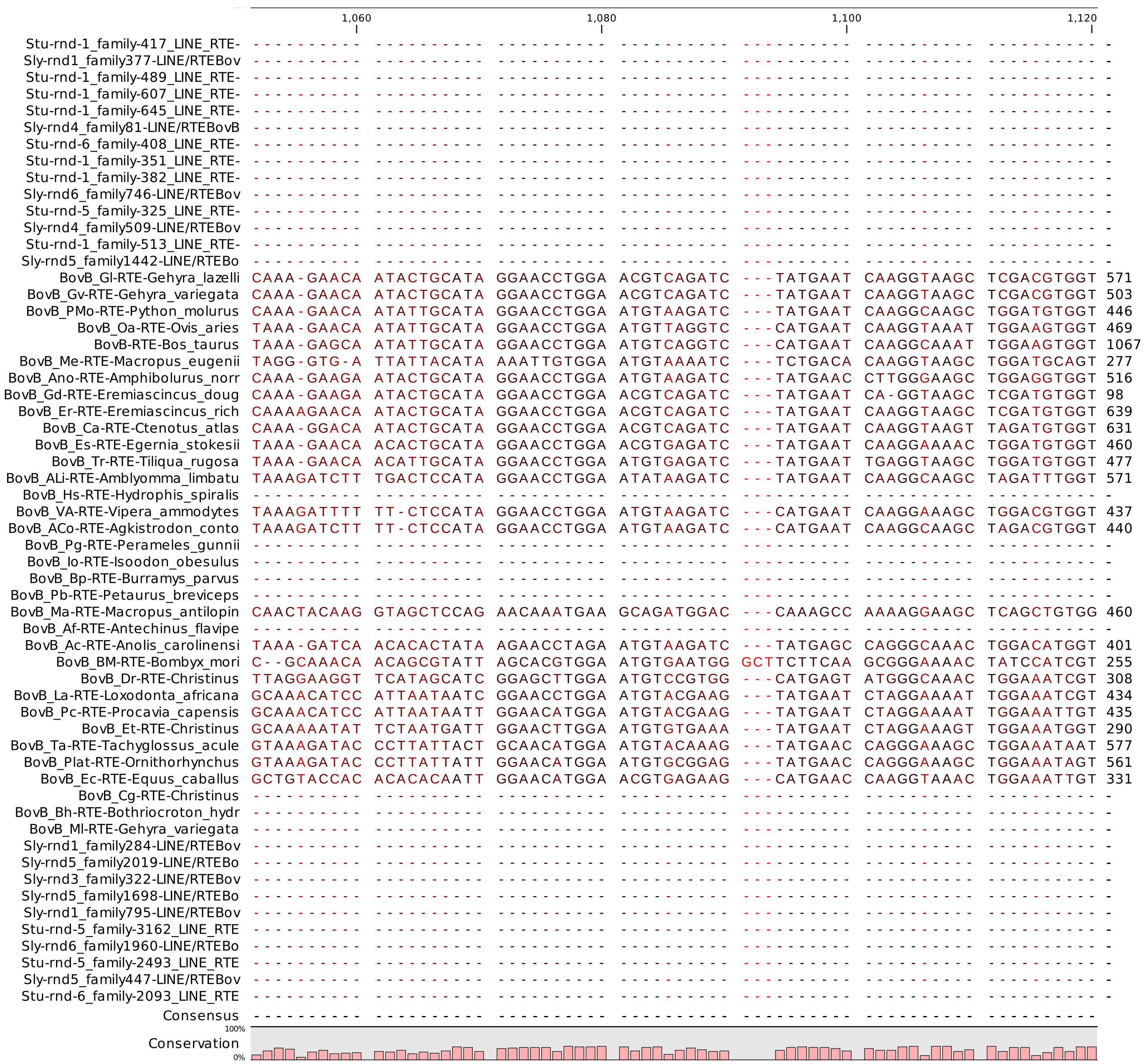

1,060
1,080
1,100
1,120
Stu-rnd-1_family-417_LINE_RTE- ---------- ---------- ---------- ---------- ---------- ---------- ---------- -
Sly-rnd1_family377-LINE/RTEBov ---------- ---------- ---------- ---------- ---------- ---------- ---------- -
Stu-rnd-1_family-489_LINE_RTE- ---------- ---------- ---------- ---------- ---------- ---------- ---------- -
Stu-rnd-1_family-607_LINE_RTE- ---------- ---------- ---------- ---------- ---------- ---------- ---------- -
Stu-rnd-1_family-645_LINE_RTE- ---------- ---------- ---------- ---------- ---------- ---------- ---------- -
Sly-rnd4_family81-LINE/RTEBovB ---------- ---------- ---------- ---------- ---------- ---------- ---------- -
Stu-rnd-6_family-408_LINE_RTE- ---------- ---------- ---------- ---------- ---------- ---------- ---------- -
Stu-rnd-1_family-351_LINE_RTE- ---------- ---------- ---------- ---------- ---------- ---------- ---------- -
Stu-rnd-1_family-382_LINE_RTE- ---------- ---------- ---------- ---------- ---------- ---------- ---------- -
Sly-rnd6_family746-LINE/RTEBov ---------- ---------- ---------- ---------- ---------- ---------- ---------- -
Stu-rnd-5_family-325_LINE_RTE- ---------- ---------- ---------- ---------- ---------- ---------- ---------- -
Sly-rnd4_family509-LINE/RTEBov ---------- ---------- ---------- ---------- ---------- ---------- ---------- -
Stu-rnd-1_family-513_LINE_RTE- ---------- ---------- ---------- ---------- ---------- ---------- ---------- -
Sly-rnd5_family1442-LINE/RTEBo ---------- ---------- ---------- ---------- ---------- ---------- ---------- -
BovB_Gl-RTE-Gehyra_lazelli CAAA-GAACA ATACTGCATA GGAACCTGGA ACGTCAGATC ---TATGAAT CAAGGTAAGC TCGACGTGGT 571
BovB_Gv-RTE-Gehyra_variegata CAAA-GAACA ATACTGCATA GGAACCTGGA ACGTCAGATC ---TATGAAT CAAGGTAAGC TCGACGTGGT 503
BovB_PMo-RTE-Python_molurus CAAA-GAACA ATATTGCATA GGAACCTGGA ATGTAAGATC ---TATGAAT CAAGGCAAGC TGGATGTGGT 446
BovB_Oa-RTE-Ovis_aries TAAA-GAACA ATATTGCATA GGAACCTGGA ATGTTAGGTC ---CATGAAT CAAGGTAAAT TGGAAGTGGT 469
BovB-RTE-Bos_taurus TAAA-GAGCA ATATTGCATA GGAACCTGGA ATGTCAGGTC ---CATGAAT CAAGGCAAAT TGGAAGTGGT 1067
BovB_Me-RTE-Macropus_eugenii TAGG-GTG-A TTATTACATA AAATTGTGGA ATGTAAAATC ---TCTGACA CAAGGTAAGC TGGATGCAGT 277
BovB_Ano-RTE-Amphibolurus_norr CAAA-GAAGA ATACTGCATA GGAACCTGGA ATGTAAGATC ---TATGAAC CTTGGGAAGC TGGAGGTGGT 516
BovB_Gd-RTE-Eremiascincus_doug CAAA-GAAGA ATACTGCATA GGAACCTGGA ACGTCAGATC ---TATGAAT CA-GGTAAGC TCGATGTGGT 98
BovB_Er-RTE-Eremiascincus_rich CAAAAGAACA ATACTGCATA GGAACCTGGA ACGTCAGATC ---TATGAAT CAAGGTAAGC TCGATGTGGT 639
BovB_Ca-RTE-Ctenotus_atlas CAAA-GGACA ATACTGCATA GGAACCTGGA ACGTCAGATC ---TATGAAT CAAGGTAAGT TAGATGTGGT 631
BovB_Es-RTE-Egernia_stokesii TAAA-GAACA ACACTGCATA GGAACCTGGA ACGTGAGATC ---TATGAAT CAAGGAAAAC TGGATGTGGT 460
BovB_Tr-RTE-Tiliqua_rugosa TAAA-GAACA ACATTGCATA GGAACCTGGA ATGTGAGATC ---TATGAAT TGAGGTAAGC TGGATGTGGT 477
BovB_ALi-RTE-Amblyomma_limbatu TAAAGATCTT TGACTCCATA GGAACCTGGA ATATAAGATC ---TATGAAT CAAGGCAAGC TAGATTTGGT 571
BovB_Hs-RTE-Hydrophis_spiralis ---------- ---------- ---------- ---------- ---------- ---------- ---------- -
BovB_VA-RTE-Vipera_ammodytes TAAAGATTTT TT-CTCCATA GGAACCTGGA ATGTAAGATC ---CATGAAT CAAGGAAAGC TGGACGTGGT 437
BovB_ACo-RTE-Agkistrodon_conto TAAAGATCTT TT-CTCCATA GGAACCTGGA ATGTAAGATC ---CATGAAT CAAGGCAAGC TAGACGTGGT 440
BovB_Pg-RTE-Perameles_gunnii ---------- ---------- ---------- ---------- ---------- ---------- ---------- -
BovB_Io-RTE-Isoodon_obesulus ---------- ---------- ---------- ---------- ---------- ---------- ---------- -
BovB_Bp-RTE-Burramys_parvus ---------- ---------- ---------- ---------- ---------- ---------- ---------- -
BovB_Pb-RTE-Petaurus_breviceps ---------- ---------- ---------- ---------- ---------- ---------- ---------- -
BovB_Ma-RTE-Macropus_antilopin CAACTACAAG GTAGCTCCAG AACAAATGAA GCAGATGGAC ---CAAAGCC AAAAGGAAGC TCAGCTGTGG 460
BovB_Af-RTE-Antechinus_flavipe ---------- ---------- ---------- ---------- ---------- ---------- ---------- -
BovB_Ac-RTE-Anolis_carolinensi TAAA-GATCA ACACACTATA AGAACCTAGA ATGTAAGATC ---TATGAGC CAGGGCAAAC TGGACATGGT 401
BovB_BM-RTE-Bombyx_mori C--GCAAACA ACAGCGTATT AGCACGTGGA ATGTGAATGG GCTTCTTCAA GCGGGAAAAC TATCCATCGT 255
BovB_Dr-RTE-Christinus TTAGGAAGGT TCATAGCATC GGAGCTTGGA ATGTCCGTGG ---CATGAGT ATGGGCAAAC TGGAAATCGT 308
BovB_La-RTE-Loxodonta_africana GCAAACATCC ATTAATAATC GGAACCTGGA ATGTACGAAG ---TATGAAT CTAGGAAAAT TGGAAATCGT 434
BovB_Pc-RTE-Procavia_capensis GCAAACATCC ATTAATAATT GGAACATGGA ATGTACGAAG ---TATGAAT CTAGGAAAAT TGGAAATTGT 435
BovB_Et-RTE-Christinus GCAAAAATAT TCTAATGATT GGAACTTGGA ATGTGTGAAA ---TATGAAT CTAGGAAAGT TGGAAATGGT 290
BovB_Ta-RTE-Tachyglossus_acule GTAAAGATAC CCTTATTACT GCAACATGGA ATGTACAAAG ---TATGAAC CAGGGAAAGC TGGAAATAAT 577
BovB_Plat-RTE-Ornithorhynchus GTAAAGATAC CCTTATTATT GGAACATGGA ATGTGCGGAG ---TATGAAC CAGGGAAAGC TGGAAATAGT 561
BovB_Ec-RTE-Equus_caballus GCTGTACCAC ACACACAATT GGAACATGGA ACGTGAGAAG ---CATGAAC CAAGGTAAAC TGGAAATTGT 331
BovB_Cg-RTE-Christinus ---------- ---------- ---------- ---------- ---------- ---------- ---------- -
BovB_Bh-RTE-Bothriocroton_hydr ---------- ---------- ---------- ---------- ---------- ---------- ---------- -
BovB_Ml-RTE-Gehyra_variegata ---------- ---------- ---------- ---------- ---------- ---------- ---------- -
Sly-rnd1_family284-LINE/RTEBov ---------- ---------- ---------- ---------- ---------- ---------- ---------- -
Sly-rnd5_family2019-LINE/RTEBo ---------- ---------- ---------- ---------- ---------- ---------- ---------- -
Sly-rnd3_family322-LINE/RTEBov ---------- ---------- ---------- ---------- ---------- ---------- ---------- -
Sly-rnd5_family1698-LINE/RTEBo ---------- ---------- ---------- ---------- ---------- ---------- ---------- -
Sly-rnd1_family795-LINE/RTEBov ---------- ---------- ---------- ---------- ---------- ---------- ---------- -
Stu-rnd-5_family-3162_LINE_RTE ---------- ---------- ---------- ---------- ---------- ---------- ---------- -
Sly-rnd6_family1960-LINE/RTEBo ---------- ---------- ---------- ---------- ---------- ---------- ---------- -
Stu-rnd-5_family-2493_LINE_RTE ---------- ---------- ---------- ---------- ---------- ---------- ---------- -
Sly-rnd5_family447-LINE/RTEBov ---------- ---------- ---------- ---------- ---------- ---------- ---------- -
Stu-rnd-6_family-2093_LINE_RTE ---------- ---------- ---------- ---------- ---------- ---------- ---------- -
Consensus ---------- ---------- ---------- ---------- ---------- ---------- ----------
100%
Conservation
0%

		1,140		1,160		1,180		
Stu-rnd-1_family-417_LINE_RTE-	----------	----------	----------	----------	----------	----------	----------	-
Sly-rnd1_family377-LINE/RTEBov	----------	----------	----------	----------	----------	----------	----------	-
Stu-rnd-1_family-489_LINE_RTE-	----------	----------	----------	----------	----------	----------	----------	-
Stu-rnd-1_family-607_LINE_RTE-	----------	----------	----------	----------	----------	----------	----------	-
Stu-rnd-1_family-645_LINE_RTE-	----------	----------	----------	----------	----------	----------	----------	-
Sly-rnd4_family81-LINE/RTEBovB	----------	----------	----------	----------	----------	----------	----------	-
Stu-rnd-6_family-408_LINE_RTE-	----------	----------	----------	----------	----------	----------	----------	-
Stu-rnd-1_family-351_LINE_RTE-	----------	----------	----------	----------	----------	----------	----------	-
Stu-rnd-1_family-382_LINE_RTE-	----------	----------	----------	----------	----------	----------	----------	-
Sly-rnd6_family746-LINE/RTEBov	----------	----------	----------	----------	----------	----------	----------	-
Stu-rnd-5_family-325_LINE_RTE-	----------	----------	----------	----------	----------	----------	----------	-
Sly-rnd4_family509-LINE/RTEBov	----------	----------	----------	----------	----------	----------	----------	-
Stu-rnd-1_family-513_LINE_RTE-	----------	----------	----------	----------	----------	----------	----------	-
Sly-rnd5_family1442-LINE/RTEBo	----------	----------	----------	----------	----------	----------	----------	-
BovB_Gl-RTE-Gehyra_lazelli	CAAAAATGAG	ATGGCAAGAC	TGAACATTGA	CATCTTGGGA	A-TCAGTGAA	C--TAAAGT-	GGACGGGAAT	637
BovB_Gv-RTE-Gehyra_variegata	CAAAAATGAG	ATGGTAAGAC	TGAACATTGA	CATCTTGGGA	A-TCAGTGAA	C--TAAAGT-	GGACGGGAAT	569
BovB_PMo-RTE-Python_molurus	CAAACATGAG	ATGACAAGAT	TGAACATCGA	CATCTTGGGA	A-TCAGCGAA	T--TGAAAT-	GGACGGGAAT	512
BovB_Oa-RTE-Ovis_aries	CAAACAGGAG	ATGGCAAGAG	TGAACATCGA	CATCTTAGGA	A-TCAGTGAA	C--TAAAAT-	GGACTGGAAT	535
BovB-RTE-Bos_taurus	CAAACAAGAG	ATGGCAAGAG	TGAATGTCGA	CATTCTAGGA	A-TCAGCGAA	C--TGAAAT-	GGACTGGAAT	1133
BovB_Me-RTE-Macropus_eugenii	CAAAT--GGG	ATGA-AAAAA	TAAACATCAG	CACCTAAGGT	G-TCAGTGAA	C--TCAAAT-	GCATGGGAAT	340
BovB_Ano-RTE-Amphibolurus_norr	CAAACAGGAG	ATGGCAAGAA	TAAACATCGA	CATCCTGGGC	A-TCAGTGAA	C--TAAAAT-	GGACAGGAAT	582
BovB_Gd-RTE-Eremiascincus_doug	CAAACGCGAG	ATGTCAAGAC	TGAACATCGA	CATCATGGGA	GGTCAGCGAA	C--TAAAATA	GGACAGGAAT	166
BovB_Er-RTE-Eremiascincus_rich	CAAACGCGAG	ATGTCAAGGC	TGAACATCGA	CATCATGGGA	G-TTAGTGAA	C--TAAAAT-	GGACAGGAAT	705
BovB_Ca-RTE-Ctenotus_atlas	CAAACGTGAG	ATGTCAAGGC	TGAACATAGA	CCTCATGGGA	G-TCAGTGAA	C--TAAAAT-	GGACAGGAAT	697
BovB_Es-RTE-Egernia_stokesii	CAAACATGAG	ATGGCAAGAT	TGAACACCGA	CATCTTCGGA	A-TTAGTGAA	CTTTAAAAT-	GGACAGAAAT	528
BovB_Tr-RTE-Tiliqua_rugosa	CCAACATGAG	ATGGCAAGAT	TGAACATTGA	CATCTTGGGA	A-TTAGTGAA	C--TAAAAT-	GGAAAGGAAT	543
BovB_ALi-RTE-Amblyomma_limbatu	CAAAGAAGAG	ATGACAAGAC	TGAACATCGA	CATCTTAGGA	A-TCAATGAA	C--TAAAATG	GATAGGAAGT	638
BovB_Hs-RTE-Hydrophis_spiralis	----------	----------	----------	----------	----------	----------	----------	-
BovB_VA-RTE-Vipera_ammodytes	CAAACAAGAG	ATGACAAGAT	TGAACATCGA	CATCTTAGGA	G-TCAGCGAA	C--TAAAATG	GACAGGAA-T	503
BovB_ACo-RTE-Agkistrodon_conto	CAAACAAGAG	ATGACAAGAC	TGAACATCGA	CATCTTAGGA	A-TCAGCGAA	C--TAAAATG	GACAGGAA-T	506
BovB_Pg-RTE-Perameles_gunnii	----------	----------	----------	----------	----------	----------	----------	-
BovB_Io-RTE-Isoodon_obesulus	----------	----------	----------	----------	----------	----------	----------	-
BovB_Bp-RTE-Burramys_parvus	----------	----------	----------	----------	----------	----------	----------	-
BovB_Pb-RTE-Petaurus_breviceps	----------	----------	----------	----------	----------	----------	----------	-
BovB_Ma-RTE-Macropus_antilopin	GTGGAAACGC	CGGTGAAAGG	AAAGACCAAC	ACCGTAAAGA	AAAACAGATC	ACAAGAACCA	GGAACCAAAG	530
BovB_Af-RTE-Antechinus_flavipe	----------	----------	----------	----------	----------	----------	----------	-
BovB_Ac-RTE-Anolis_carolinensi	TATTGGTGAA	ATGTCAAGAT	TAAAGATAGA	CATTCTGGGA	G-TCAGTGAA	C--TGAAAT-	GGACTAGAAT	467
BovB_BM-RTE-Bombyx_mori	CGAAAAAGAA	ATGGAAAATC	ACAACTTGTC	AATTCTGGGC	A-TCTGCGAG	A--CACATAT	GAGAAACAAT	322
BovB_Dr-RTE-Christinus	GAAATCTGAA	ATGGAACGTC	TCAACATCGA	CATTATCGCC	A-TTAGTGAG	C--TCCATTG	GACAGGCAAT	375
BovB_La-RTE-Loxodonta_africana	-AAAAATGAA	ATGGAACGCA	TAAACATCAA	TATCCTAGGC	A-TTAGTGAG	C--TGAAATG	GACTGGTATT	500
BovB_Pc-RTE-Procavia_capensis	CAAAAATGAA	ATGGAATGCA	TAAACATTGA	TATCCTAGGC	A-TTAGTGAG	C--TGAAATG	GACTGGTTTT	502
BovB_Et-RTE-Christinus	CAAACATGAA	ATGGAATGCA	TAATGATCAA	TATCACAGGC	A-TAAGTGAG	C--TGAAATT	GACTGATATT	357
BovB_Ta-RTE-Tachyglossus_acule	CAAGAATGAA	ATGGACTGAT	TGAAGATCGA	CATCCTAAGA	A-TAAGCAAA	A--TGAAATG	GACTGGTATT	644
BovB_Plat-RTE-Ornithorhynchus	CAAGAATGAA	ATGGACCGAT	TGAAGATCGA	CATCCTAGGA	A-TAAGCGAA	C--TGAAATG	GACTGGTATT	628
BovB_Ec-RTE-Equus_caballus	AAAGCAAGAA	ATGGAACATT	TAAATATTGC	AGTGCTTGGT	G-TTAGCGAA	C--TAAAATG	GACTGGAATG	398
BovB_Cg-RTE-Christinus	----------	------CTTT	TGTAGACTAA	CAGCAGTGCT	A-CT--TCAG	C--TAACCCT	GGTTAGTACT	49
BovB_Bh-RTE-Bothriocroton_hydr	----------	----------	----------	----------	----------	----------	----------	-
BovB_Ml-RTE-Gehyra_variegata	----------	----------	----------	----------	----------	----------	----------	-
Sly-rnd1_family284-LINE/RTEBov	----------	----------	----------	----------	----------	----------	----------	-
Sly-rnd5_family2019-LINE/RTEBo	----------	----------	----------	----------	----------	----------	----------	-
Sly-rnd3_family322-LINE/RTEBov	----------	----------	----------	----------	----------	----------	----------	-
Sly-rnd5_family1698-LINE/RTEBo	----------	----------	----------	----------	----------	----------	----------	-
Sly-rnd1_family795-LINE/RTEBov	----------	----------	----------	----------	----------	----------	----------	-
Stu-rnd-5_family-3162_LINE_RTE	----------	----------	----------	----------	----------	----------	----------	-
Sly-rnd6_family1960-LINE/RTEBo	----------	----------	----------	----------	----------	----------	----------	-
Stu-rnd-5_family-2493_LINE_RTE	----------	----------	----------	----------	----------	----------	----------	-
Sly-rnd5_family447-LINE/RTEBov	----------	----------	----------	----------	----------	----------	----------	-
Stu-rnd-6_family-2093_LINE_RTE	----------	----------	----------	----------	----------	----------	----------	-
Consensus	----------	----------	----------	----------	----------	----------	----------	

Conservation

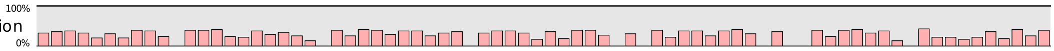

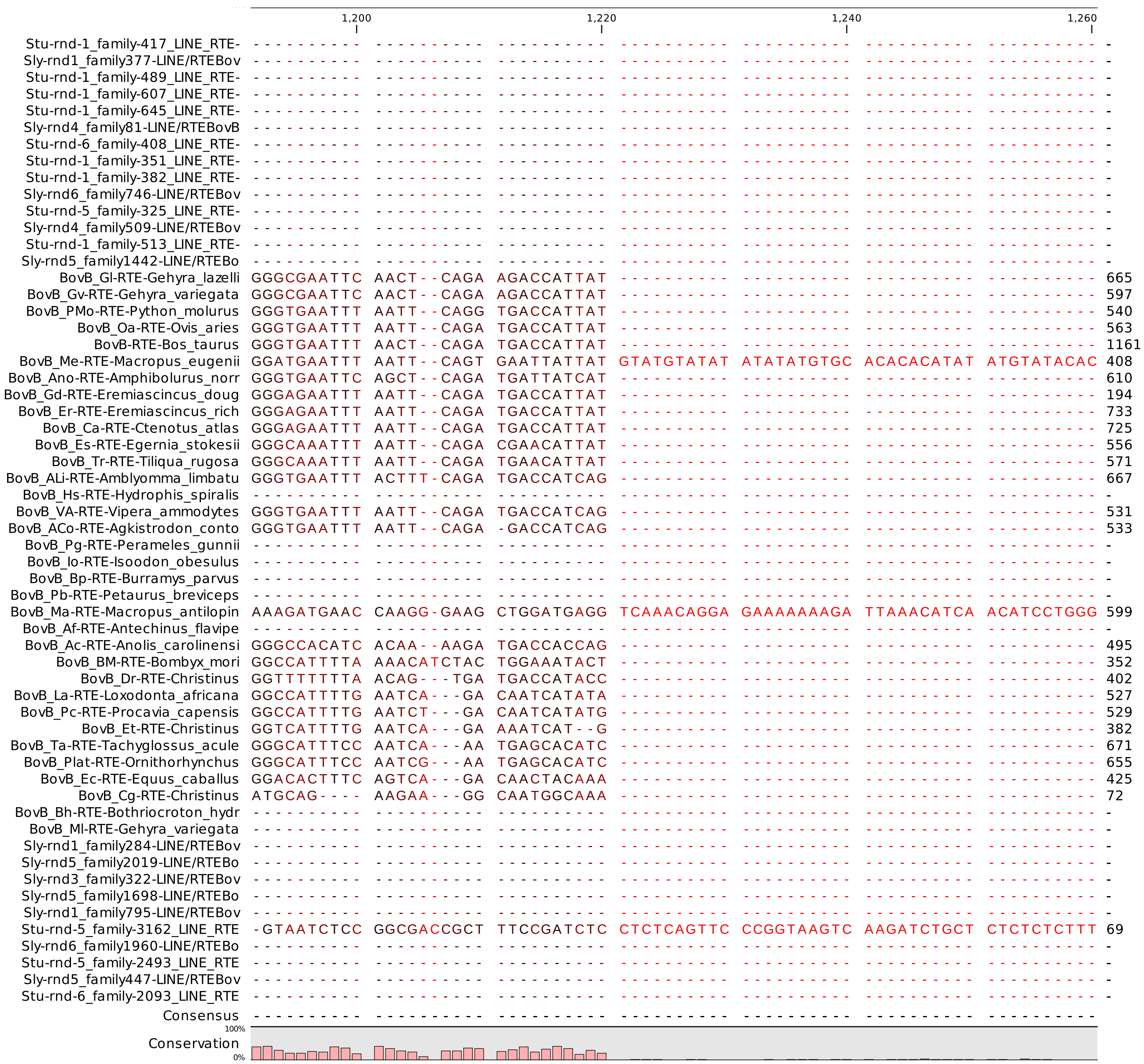

1,200 1,220 1,240 1,260
Stu-rnd-1_family-417_LINE_RTE- ---------- ---------- ---------- ---------- ---------- ---------- ---------- -
Sly-rnd1_family377-LINE/RTEBov ---------- ---------- ---------- ---------- ---------- ---------- ---------- -
Stu-rnd-1_family-489_LINE_RTE- ---------- ---------- ---------- ---------- ---------- ---------- ---------- -
Stu-rnd-1_family-607_LINE_RTE- ---------- ---------- ---------- ---------- ---------- ---------- ---------- -
Stu-rnd-1_family-645_LINE_RTE- ---------- ---------- ---------- ---------- ---------- ---------- ---------- -
Sly-rnd4_family81-LINE/RTEBovB ---------- ---------- ---------- ---------- ---------- ---------- ---------- -
Stu-rnd-6_family-408_LINE_RTE- ---------- ---------- ---------- ---------- ---------- ---------- ---------- -
Stu-rnd-1_family-351_LINE_RTE- ---------- ---------- ---------- ---------- ---------- ---------- ---------- -
Stu-rnd-1_family-382_LINE_RTE- ---------- ---------- ---------- ---------- ---------- ---------- ---------- -
Sly-rnd6_family746-LINE/RTEBov ---------- ---------- ---------- ---------- ---------- ---------- ---------- -
Stu-rnd-5_family-325_LINE_RTE- ---------- ---------- ---------- ---------- ---------- ---------- ---------- -
Sly-rnd4_family509-LINE/RTEBov ---------- ---------- ---------- ---------- ---------- ---------- ---------- -
Stu-rnd-1_family-513_LINE_RTE- ---------- ---------- ---------- ---------- ---------- ---------- ---------- -
Sly-rnd5_family1442-LINE/RTEBo ---------- ---------- ---------- ---------- ---------- ---------- ---------- -
BovB_Gl-RTE-Gehyra_lazelli GGGCGAATTC AACT--CAGA AGACCATTAT ---------- ---------- ---------- ---------- 665
BovB_Gv-RTE-Gehyra_variegata GGGCGAATTC AACT--CAGA AGACCATTAT ---------- ---------- ---------- ---------- 597
BovB_PMo-RTE-Python_molurus GGGTGAATTT AATT--CAGG TGACCATTAT ---------- ---------- ---------- ---------- 540
BovB_Oa-RTE-Ovis_aries GGGTGAATTT AATT--CAGA TGACCATTAT ---------- ---------- ---------- ---------- 563
BovB-RTE-Bos_taurus GGGTGAATTT AACT--CAGA TGACCATTAT ---------- ---------- ---------- ---------- 1161
BovB_Me-RTE-Macropus_eugenii GGATGAATTT AATT--CAGT GAATTATTAT GTATGTATAT ATATATGTGC ACACACATAT ATGTATACAC 408
BovB_Ano-RTE-Amphibolurus_norr GGGTGAATTC AGCT--CAGA TGATTATCAT ---------- ---------- ---------- ---------- 610
BovB_Gd-RTE-Eremiascincus_doug GGGAGAATTT AATT--CAGA TGACCATTAT ---------- ---------- ---------- ---------- 194
BovB_Er-RTE-Eremiascincus_rich GGGAGAATTT AATT--CAGA TGACCATTAT ---------- ---------- ---------- ---------- 733
BovB_Ca-RTE-Ctenotus_atlas GGGAGAATTT AATT--CAGA TGACCATTAT ---------- ---------- ---------- ---------- 725
BovB_Es-RTE-Egernia_stokesii GGGCAAATTT AATT--CAGA CGAACATTAT ---------- ---------- ---------- ---------- 556
BovB_Tr-RTE-Tiliqua_rugosa GGGCAAATTT AATT--CAGA TGAACATTAT ---------- ---------- ---------- ---------- 571
BovB_ALi-RTE-Amblyomma_limbatu GGGTGAATTT ACTTT-CAGA TGACCATCAG ---------- ---------- ---------- ---------- 667
BovB_Hs-RTE-Hydrophis_spiralis ---------- ---------- ---------- ---------- ---------- ---------- ---------- -
BovB_VA-RTE-Vipera_ammodytes GGGTGAATTT AATT--CAGA TGACCATCAG ---------- ---------- ---------- ---------- 531
BovB_ACo-RTE-Agkistrodon_conto GGGTGAATTT AATT--CAGA -GACCATCAG ---------- ---------- ---------- ---------- 533
BovB_Pg-RTE-Perameles_gunnii ---------- ---------- ---------- ---------- ---------- ---------- ---------- -
BovB_Io-RTE-Isoodon_obesulus ---------- ---------- ---------- ---------- ---------- ---------- ---------- -
BovB_Bp-RTE-Burramys_parvus ---------- ---------- ---------- ---------- ---------- ---------- ---------- -
BovB_Pb-RTE-Petaurus_breviceps ---------- ---------- ---------- ---------- ---------- ---------- ---------- -
BovB_Ma-RTE-Macropus_antilopin AAAGATGAAC CAAGG-GAAG CTGGATGAGG TCAAACAGGA GAAAAAAAGA TTAAACATCA ACATCCTGGG 599
BovB_Af-RTE-Antechinus_flavipe ---------- ---------- ---------- ---------- ---------- ---------- ---------- -
BovB_Ac-RTE-Anolis_carolinensi GGGCCACATC ACAA--AAGA TGACCACCAG ---------- ---------- ---------- ---------- 495
BovB_BM-RTE-Bombyx_mori GGCCATTTTA AAACATCTAC TGGAAATACT ---------- ---------- ---------- ---------- 352
BovB_Dr-RTE-Christinus GGTTTTTTTA ACAG---TGA TGACCATACC ---------- ---------- ---------- ---------- 402
BovB_La-RTE-Loxodonta_africana GGCCATTTTG AATCA---GA CAATCATATA ---------- ---------- ---------- ---------- 527
BovB_Pc-RTE-Procavia_capensis GGCCATTTTG AATCT---GA CAATCATATG ---------- ---------- ---------- ---------- 529
BovB_Et-RTE-Christinus GGTCATTTTG AATCA---GA AAATCAT--G ---------- ---------- ---------- ---------- 382
BovB_Ta-RTE-Tachyglossus_acule GGGCATTTCC AATCA---AA TGAGCACATC ---------- ---------- ---------- ---------- 671
BovB_Plat-RTE-Ornithorhynchus GGGCATTTCC AATCG---AA TGAGCACATC ---------- ---------- ---------- ---------- 655
BovB_Ec-RTE-Equus_caballus GGACACTTTC AGTCA---GA CAACTACAAA ---------- ---------- ---------- ---------- 425
BovB_Cg-RTE-Christinus ATGCAG---- AAGAA---GG CAATGGCAAA ---------- ---------- ---------- ---------- 72
BovB_Bh-RTE-Bothriocroton_hydr ---------- ---------- ---------- ---------- ---------- ---------- ---------- -
BovB_Ml-RTE-Gehyra_variegata ---------- ---------- ---------- ---------- ---------- ---------- ---------- -
Sly-rnd1_family284-LINE/RTEBov ---------- ---------- ---------- ---------- ---------- ---------- ---------- -
Sly-rnd5_family2019-LINE/RTEBo ---------- ---------- ---------- ---------- ---------- ---------- ---------- -
Sly-rnd3_family322-LINE/RTEBov ---------- ---------- ---------- ---------- ---------- ---------- ---------- -
Sly-rnd5_family1698-LINE/RTEBo ---------- ---------- ---------- ---------- ---------- ---------- ---------- -
Sly-rnd1_family795-LINE/RTEBov ---------- ---------- ---------- ---------- ---------- ---------- ---------- -
Stu-rnd-5_family-3162_LINE_RTE -GTAATCTCC GGCGACCGCT TTCCGATCTC CTCTCAGTTC CCGGTAAGTC AAGATCTGCT CTCTCTCTTT 69
Sly-rnd6_family1960-LINE/RTEBo ---------- ---------- ---------- ---------- ---------- ---------- ---------- -
Stu-rnd-5_family-2493_LINE_RTE ---------- ---------- ---------- ---------- ---------- ---------- ---------- -
Sly-rnd5_family447-LINE/RTEBov ---------- ---------- ---------- ---------- ---------- ---------- ---------- -
Stu-rnd-6_family-2093_LINE_RTE ---------- ---------- ---------- ---------- ---------- ---------- ---------- -
Consensus ---------- ---------- ---------- ---------- ---------- ---------- ----------
100%
Conservation
0%

```
                                                    1,280                         1,300                         1,320
Stu-rnd-1_family-417_LINE_RTE-      ---------- ---------- ---------- ---------- ---------- ---------- ---------- -
Sly-rnd1_family377-LINE/RTEBov      ---------- ---------- ---------- ---------- ---------- ---------- ---------- -
Stu-rnd-1_family-489_LINE_RTE-      ---------- ---------- ---------- ---------- ---------- ---------- ---------- -
Stu-rnd-1_family-607_LINE_RTE-      ---------- ---------- ---------- ---------- ---------- ---------- ---------- -
Stu-rnd-1_family-645_LINE_RTE-      ---------- ---------- ---------- ---------- ---------- ---------- ---------- -
Sly-rnd4_family81-LINE/RTEBovB      ---------- ---------- ---------- ---------- ---------- ---------- ---------- -
Stu-rnd-6_family-408_LINE_RTE-      ---------- ---------- ---------- ---------- ---------- ---------- ---------- -
Stu-rnd-1_family-351_LINE_RTE-      ---------- ---------- ---------- ---------- ---------- ---------- ---------- -
Stu-rnd-1_family-382_LINE_RTE-      ---------- ---------- ---------- ---------- ---------- ---------- ---------- -
Sly-rnd6_family746-LINE/RTEBov      ---------- ---------- ---------- ---------- ---------- ---------- ---------- -
Stu-rnd-5_family-325_LINE_RTE-      ---------- ---------- ---------- ---------- ---------- ---------- ---------- -
Sly-rnd4_family509-LINE/RTEBov      ---------- ---------- ---------- ---------- ---------- ---------- ---------- -
Stu-rnd-1_family-513_LINE_RTE-      ---------- ---------- ---------- ---------- ---------- ---------- ---------- -
Sly-rnd5_family1442-LINE/RTEBo      ---------- ---------- ---------- ---------- ---------- ---------- ---------- -
BovB_Gl-RTE-Gehyra_lazelli          --ATCTACTA CTGTGGCCAT GAATCCCGTA GAAAAAATGG TGTGGCCTTT ATAGTCAACA AGAGAGTGCA 733
BovB_Gv-RTE-Gehyra_variegata        --ATCTACTA CTGTGGCCAT GAATCCCGTA GAAAAAATGG TGTGGCCTTT ATAGTCAACA AGAGAGTGCA 665
BovB_PMo-RTE-Python_molurus         --ATCTACTA CTGTGGCAA- GAAACCCTTA GAAGAAATGG AGTTGCACTC ATAGTCAATA AGAGAGTGGG 607
BovB_Oa-RTE-Ovis_aries              --ATCTACTA CTGTGGGCAA GAATCCCTTA GAAGAAATGG AGTAGCCCTC ATAGTCAACA AAAGAGTCTG 631
BovB-RTE-Bos_taurus                 --ATCTACTA CTGCGGGCAG GAATCCCTCA GAAGAAATGG AGTGGCCATC ATGGTCAACA AAAGAGTCCG 1229
BovB_Me-RTE-Macropus_eugenii        ACATATGCCA CTG-GGGCAA GAAGTCTTTA GAAGAAGCAA AGTAGCCCTC ATAGTCAACA TAGC--TAAG 475
BovB_Ano-RTE-Amphibolurus_norr      --ATCTACTA TTGTGGGCAA GAATCCCGTA GAAGGAATGG AGTAGCCTTC ATAGTCAACA AAAGAGTGGG 678
BovB_Gd-RTE-Eremiascincus_doug      --ATCTGCTA CTGTGGGCAG GAATCCCTTA GAAGAAATGG AGTAGCCTTC ATCGTGAACA AGAGAGTGAG 262
BovB_Er-RTE-Eremiascincus_rich      --ATCTGCTA CTGTGGGCAG GAATCCCTTA GAAGAAATGG AGTAGCCTTC ATCGTGAACA AGAGAGTGAG 801
BovB_Ca-RTE-Ctenotus_atlas          --ATCTACTA CTGTGGGCAG GAATCCCTTA GAAGAAACGG CGTAGCCTTC ATAGTGAACA AGAGAATGAG 793
BovB_Es-RTE-Egernia_stokesii        --ATTTACTA CTGTGGACAA GAATCCCATA GAAGAAATGG AGTAGCCCTC ATAGTAAACA AAAGAATGGG 624
BovB_Tr-RTE-Tiliqua_rugosa          --ATTTACTA CTGTGGACAA GAATCCCATA GAAGAAATAG AGTAGCCCTC ATTGTGAACA AAAGAATGGG 639
BovB_ALi-RTE-Amblyomma_limbatu      --ATATAAAA CTGCGGGTAA GAATCCCTCA CAAGAAATTG AGTAGCCTTC ATATTCAATA AAAAATTAGG 735
BovB_Hs-RTE-Hydrophis_spiralis      ---------- ---------- ---------- ---------- ---------- ---------- ---------- -
BovB_VA-RTE-Vipera_ammodytes        --GTATACTA CTGCGGGCAG GAATCCCTCA GAAGAAATGG AGTAGCCTTC ACAGTCAATA AAAGAGTAGA 599
BovB_ACo-RTE-Agkistrodon_conto      --GTATACTA CTGCGGGCAA GAATCCCTCA GAAGAAATGG AGTAGCCTTC ATAGTCAATA AAAGAGTAGG 601
BovB_Pg-RTE-Perameles_gunnii        ---------- ---------- ---------- ---------- ---------- ---------- ---------- -
BovB_Io-RTE-Isoodon_obesulus        ---------- ---------- ---------- ---------- ---------- ---------- ---------- -
BovB_Bp-RTE-Burramys_parvus         ---------- ---------- ---------- ---------- ---------- ---------- ---------- -
BovB_Pb-RTE-Petaurus_breviceps      ---------- ---------- ---------- ---------- ---------- ---------- ---------- -
BovB_Ma-RTE-Macropus_antilopin      TGTCAGTGAA CTTAAATGAA TGAGAATGGG TAAATTTAAT TCAGGTGATC ACTACATATA CTACCGCGGA 669
BovB_Af-RTE-Antechinus_flavipe      ---------- ---------- ---------- ---------- ---------- ---------- ---------- -
BovB_Ac-RTE-Anolis_carolinensi      --ATCTACTA CAGCGGACAA GAGGAACACC GAAGAAATGG AGTAGCCTTC ATAATTAATA ACAAAGTTGC 563
BovB_BM-RTE-Bombyx_mori             --GTGTACTT CTCAGGTAAT GAAAATTCAA GCAGAAATGG AGTTGCAATT GTGCTGCCAC CCTACCTGAA 420
BovB_Dr-RTE-Christinus              --GTCTACTA CTCTGGGAAC AACAACATAA GACGCAATGG AGTAGCTGTC ATCTTAAACA AGAAAATTGC 470
BovB_La-RTE-Loxodonta_africana      --GTCTACTA TGCTGGGAAT GACAACTTGA AGAGGAATGG CATTGCATTC ATCGTCAAAA AGAACATTTC 595
BovB_Pc-RTE-Procavia_capensis       --GTCTACTA TGCTGGGAAT GAAAACTTGA AGAGGAATGG TGTTGCATTC ATCATCAAAA AGAACATTTC 597
BovB_Et-RTE-Christinus              --ATTTACTA TGTCAGGTAT AACA---CAA TCATGAATGG CATGACATTC ACTGTCAAAA AGGATATACC 447
BovB_Ta-RTE-Tachyglossus_acule      --ATCTACTA CTCTGCTCAT GCTGAAAAAA GAAGAAATGG CTTAGCGATA ATTGTGACAA AGAAAATTGC 739
BovB_Plat-RTE-Ornithorhynchus       --ATCTACTA CTCTGCCCAT GCTGAAAAAA GAAGAAATGG CGTAGCGGTA ATTGTGACAA AGAAAATTGC 723
BovB_Ec-RTE-Equus_caballus          --GTGTTCTA CTCTGGAAAT GACAAACTCA GAAGAAACAG AGTGGCTCTA ATACTGAGGC AAGATGTAGC 493
BovB_Cg-RTE-Christinus              -------CCA CTTC-----T GACTAACACA GAAGATACAG AGTTGTTCTA ATACTGAGGC AAGATTTAGC 130
BovB_Bh-RTE-Bothriocroton_hydr      ---------- ---------- ---------- ---------- ---------- ---------- ---------- -
BovB_Ml-RTE-Gehyra_variegata        ---------- ---------- ---------- ---------- ---------- ---------- ---------- -
Sly-rnd1_family284-LINE/RTEBov      ---------- ---------- ---------- ---------- ---------- ---------- ---------- -
Sly-rnd5_family2019-LINE/RTEBo      ---------- ---------- ---------- ---------- ---------- ---------- ---------- -
Sly-rnd3_family322-LINE/RTEBov      ---------- ---------- ---------- ---------- ---------- ---------- ---------- -
Sly-rnd5_family1698-LINE/RTEBo      ---------- ---------- ---------- ---------- ---------- ---------- ---------- -
Sly-rnd1_family795-LINE/RTEBov      ---------- ---------- ---------- ---------- ---------- ---------- ---------- -
Stu-rnd-5_family-3162_LINE_RTE      CTCTCTCTCC TCTAGTCTAC TAGCAGCTCA GGCGAAGCTG CGAACGGCGC CGGCCAGCGA ACAGCAGCGA 139
Sly-rnd6_family1960-LINE/RTEBo      ---------- ---------- ---------- ---------- ---------- ---------- ---------- -
Stu-rnd-5_family-2493_LINE_RTE      ---------- ---------- ---------- ---------- ---------- ---------- ---------- -
Sly-rnd5_family447-LINE/RTEBov      ---------- ---------- ---------- ---------- ---------- ---------- ---------- -
Stu-rnd-6_family-2093_LINE_RTE      ---------- ---------- ---------- ---------- ---------- ---------- ---------- -

Consensus                           ---------- ---------- ---------- ---------- ---------- ---------- ----------
```

Conservation

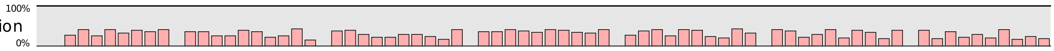

	1,340		1,360		1,380		1,400	
Stu-rnd-1_family-417_LINE_RTE-	----------	----------	----------	----------	----------	----------	----------	-
Sly-rnd1_family377-LINE/RTEBov	----------	----------	----------	----------	----------	----------	----------	-
Stu-rnd-1_family-489_LINE_RTE-	----------	----------	----------	----------	----------	----------	----------	-
Stu-rnd-1_family-607_LINE_RTE-	----------	----------	----------	----------	----------	----------	----------	-
Stu-rnd-1_family-645_LINE_RTE-	----------	----------	----------	----------	----------	----------	----------	-
Sly-rnd4_family81-LINE/RTEBovB	----------	----------	----------	----------	----------	----------	----------	-
Stu-rnd-6_family-408_LINE_RTE-	----------	----------	----------	----------	----------	----------	----------	-
Stu-rnd-1_family-351_LINE_RTE-	----------	----------	----------	----------	----------	----------	----------	-
Stu-rnd-1_family-382_LINE_RTE-	----------	----------	----------	----------	----------	----------	----------	-
Sly-rnd6_family746-LINE/RTEBov	----------	----------	----------	----------	----------	----------	----------	-
Stu-rnd-5_family-325_LINE_RTE-	----------	----------	----------	----------	----------	----------	----------	-
Sly-rnd4_family509-LINE/RTEBov	----------	----------	----------	----------	----------	----------	----------	-
Stu-rnd-1_family-513_LINE_RTE-	----------	----------	----------	----------	----------	----------	----------	-
Sly-rnd5_family1442-LINE/RTEBo	----------	----------	----------	----------	----------	----------	----------	-
BovB_Gl-RTE-Gehyra_lazelli	AAAGGCAGTA	ATGGGATACA	ATC-TCAAAA	ACG-ACAGAA	TGATCTCTGT	CCCGAATCCA	AGGCAAACCA	801
BovB_Gv-RTE-Gehyra_variegata	AAAGGCAGTA	ATGGGATACA	ATC-TCAAAA	ACG-ACAGAA	TGATCTCTAT	CC-GAATCCA	AGGCAAACCA	732
BovB_PMo-RTE-Python_molurus	AAAGGCAGTA	ATGGGGTACA	ACC-TCAAAA	ACG-ACAGAA	TGATCTCAGT	GC-GTATCCA	AGGCAAACCA	674
BovB_Oa-RTE-Ovis_aries	AAATGCAGTA	CTTGGGTGCA	ATC-TCAAAA	ATG-ACAGAA	TGATCTCTGT	TC-ATTTCCA	AGGCAAACCA	698
BovB-RTE-Bos_taurus	AAATGCAGTA	CTTGGATGCA	ATC-TCAAAA	ACG-ACAGAA	TGATCTCTGT	TC-GTTTCCA	AGGCAAACCA	1296
BovB_Me-RTE-Macropus_eugenii	AAAAGCAGTA	TGGGGGTATA	ATC-TCAAAA	AGG-ATAGAA	TCCTATCAGT	CT-GAATACA	AGACAAACTA	542
BovB_Ano-RTE-Amphibolurus_norr	AAAAGCTGTA	ATGGGATACA	ATC-TCAAAA	ATG-ATAGAA	TGATGTCAAT	AC-GAATCCA	AGGCAGACCT	745
BovB_Gd-RTE-Eremiascincus_doug	AACAGCAGTA	ATGGGGTATA	ACC-TCAAAA	ATG-ATAGAA	TGATTTCAAT	TC-GGATCCA	AGGCAAACCT	329
BovB_Er-RTE-Eremiascincus_rich	AACAGCAGTA	CTGGGGTATA	ACC-TCAAAA	ATG-ATAGAA	TGATTTCAAT	TC-GGATCCA	AGGCAAACCT	868
BovB_Ca-RTE-Ctenotus_atlas	AGCAGCAGTG	TTGGGGTATA	ACC-TCAAAA	ATG-ATAGAA	TGATTTCAAT	TC-GGATCCA	AGGCAAACCT	860
BovB_Es-RTE-Egernia_stokesii	AAAAACAGTA	GGGGGATACA	ATC-TCAAAA	ACG-ACAGAA	TGATTTCAGT	TC-AAATTCA	AGGCAAACCT	691
BovB_Tr-RTE-Tiliqua_rugosa	AAAAGCAGTA	CTGGGGTACA	ATC-TCAAAA	ACA-ACAGAA	TGATTTCAGT	TC-AAATTCA	AGGCAAACCT	706
BovB_ALi-RTE-Amblyomma_limbatu	AAGAATAATA	TTGTGATACA	ATC-TCCAAA	ATG-ACATAA	TGATATCAGT	TT-GAATTCA	ATGCAAACCA	802
BovB_Hs-RTE-Hydrophis_spiralis	----------	----------	----------	----------	----------	----------	----------	-
BovB_VA-RTE-Vipera_ammodytes	AAAAGCAATA	CTGGGATACA	ATC-CCCAAA	ATG-ACAGAA	TGATCTCAGT	TC-GAATCCA	AGGCAAACCA	666
BovB_ACo-RTE-Agkistrodon_conto	AAAAGCAATA	CTGGGATACA	ATC-CCCAAA	ATG-ACAGAA	TGATCTCAGT	GC-GAATCCA	AGGCAAACCA	668
BovB_Pg-RTE-Perameles_gunnii	----------	----------	----------	----------	----------	----------	----------	-
BovB_Io-RTE-Isoodon_obesulus	----------	----------	----------	----------	----------	----------	----------	-
BovB_Bp-RTE-Burramys_parvus	----------	----------	----------	----------	----------	----------	----------	-
BovB_Pb-RTE-Petaurus_breviceps	----------	----------	----------	----------	----------	----------	----------	-
BovB_Ma-RTE-Macropus_antilopin	AAAGAATCCC	TTAGAAGAAA	TGG-AACAGT	CAA-CACAGT	CAATAAAAGG	GTAAGAAAAG	CAATACCAGG	737
BovB_Af-RTE-Antechinus_flavipe	----------	----------	----------	----------	----------	----------	----------	-
BovB_Ac-RTE-Anolis_carolinensi	GAAAGCAGTG	ATTGGATACA	ACC-CAAAAA	ATG-ATAGAA	TGATCTCAAT	TC-GAGTTCA	AGGCAAGCCA	630
BovB_BM-RTE-Bombyx_mori	CCAATACGTC	CTCGGATACC	ATC-CCATAT	CTG-ATAGAA	TACTAACTTT	GAAACTCAAC	ACAAAACC-A	487
BovB_Dr-RTE-Christinus	TAAAGCTGTT	CAAAGCTTCA	ATG-CTATAA	ATG-GCCGAG	TCATATCCAT	CCGTTTCCAT	GGGAAGCC-T	537
BovB_La-RTE-Loxodonta_africana	AAGATCTATC	CTGAAGTACA	ACG-CTGTCA	GTG-ATAGGA	TAATATCCAT	ACGCCTACAA	GGAAGACC-A	662
BovB_Pc-RTE-Procavia_capensis	AAGATCTGTC	CTGAAGTACA	ATG-CTGTCA	GTG-ATAGGA	TAATATCCAT	ATGCCTACAA	GGAAGGCC-A	664
BovB_Et-RTE-Christinus	AAAATACATC	ATGAAATACA	ATG-CTATCT	AC--ATCAGA	TGATATCTAT	ACACTTTCAA	GGAAATCCCA	514
BovB_Ta-RTE-Tachyglossus_acule	TAAGACAGTT	ATCGGATATA	ATG-CTGTCA	ATG-ACCAAT	TGATATCTGT	TAGATTCAGA	GGAAAACC-A	806
BovB_Plat-RTE-Ornithorhynchus	TAAGACAGTT	ATCGGATATA	ATG-CTGTCA	ATG-ACCGAT	TGATATCTGT	TAGATTCAGA	GGAAAACC-A	790
BovB_Ec-RTE-Equus_caballus	ACAGGCAGTT	AGGGGCTCTA	ATG-CAAGGT	CTG-ACCAAA	TAATATCAAT	CAGACTTC-T	GGGAAAACCT	560
BovB_Cg-RTE-Christinus	ACAAGCAATC	AGGGACTATA	ATGACAAAGT	CTGGACCAAA	TAATTTCAAT	CAGACTTCAT	TGAAAACCCT	200
BovB_Bh-RTE-Bothriocroton_hydr	----------	----------	----------	----------	----------	----------	----------	-
BovB_Ml-RTE-Gehyra_variegata	----------	----------	----------	----------	----------	----------	----------	-
Sly-rnd1_family284-LINE/RTEBov	----------	----------	----------	----------	----------	----------	----------	-
Sly-rnd5_family2019-LINE/RTEBo	----------	----------	----------	----------	----------	----------	----------	-
Sly-rnd3_family322-LINE/RTEBov	----------	----------	----------	----------	----------	----------	----------	-
Sly-rnd5_family1698-LINE/RTEBo	----------	----------	----------	----------	----------	----------	----------	-
Sly-rnd1_family795-LINE/RTEBov	----------	----------	----------	----------	----------	----------	----------	-
Stu-rnd-5_family-3162_LINE_RTE	AAACCAGTCG	GCAACCGGAC	GTCGCCTCTG	ATCTGGTATT	CCCGGCCGAA	ATCGAAGGTT	GCGCCGGCCA	209
Sly-rnd6_family1960-LINE/RTEBo	----------	----------	----------	----------	----------	----------	----------	-
Stu-rnd-5_family-2493_LINE_RTE	----------	----------	----------	----------	----------	----------	----------	-
Sly-rnd5_family447-LINE/RTEBov	----------	----------	----------	----------	----------	----------	----------	-
Stu-rnd-6_family-2093_LINE_RTE	----------	----------	----------	----------	----------	----------	----------	-
Consensus	----------	----------	----------	----------	----------	----------	----------	

Conservation

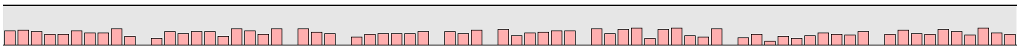

```
                                  1,420                1,440                1,460
                                      |                    |                    |
Stu-rnd-1_family-417_LINE_RTE-   ---------- ---------- ---------- ---------- ---------- ---------- ---------- -
Sly-rnd1_family377-LINE/RTEBov   ---------- ---------- ---------- ---------- ---------- ---------- ---------- -
Stu-rnd-1_family-489_LINE_RTE-   ---------- ---------- ---------- ---------- ---------- ---------- ---------- -
Stu-rnd-1_family-607_LINE_RTE-   ---------- ---------- ---------- ---------- ---------- ---------- ---------- -
Stu-rnd-1_family-645_LINE_RTE-   ---------- ---------- ---------- ---------- ---------- ---------- ---------- -
Sly-rnd4_family81-LINE/RTEBovB   ---------- ---------- ---------- ---------- ---------- ---------- ---------- -
Stu-rnd-6_family-408_LINE_RTE-   ---------- ---------- ---------- ---------- ---------- ---------- ---------- -
Stu-rnd-1_family-351_LINE_RTE-   ---------- ---------- ---------- ---------- ---------- ---------- ---------- -
Stu-rnd-1_family-382_LINE_RTE-   ---------- ---------- ---------- ---------- ---------- ---------- ---------- -
Sly-rnd6_family746-LINE/RTEBov   ---------- ---------- ---------- ---------- ---------- ---------- ---------- -
Stu-rnd-5_family-325_LINE_RTE-   ---------- ---------- ---------- ---------- ---------- ---------- ---------- -
Sly-rnd4_family509-LINE/RTEBov   ---------- ---------- ---------- ---------- ---------- ---------- ---------- -
Stu-rnd-1_family-513_LINE_RTE-   ---------- ---------- ---------- ---------- ---------- ---------- ---------- -
Sly-rnd5_family1442-LINE/RTEBo   ---------- ---------- ---------- ---------- ---------- ---------- ---------- -
BovB_Gl-RTE-Gehyra_lazelli       TTCAAACCAT CACAGTAAAT CCAAGTCTAT CGCCCC---- -------AAC CACTGACGCA GAAGAGGTTG 860
BovB_Gv-RTE-Gehyra_variegata     TTCAA--CAT CACAGTAA-T CCAAGTCTAT -GCCCC---- -------AAC CACTGACGCA GAAGAAGTTG 787
BovB_PMo-RTE-Python_molurus      TTCAA--CAT CACAGTCA-T CCAAGTCTAT -GCCCC---- -------AAC CACTGATGCT GAAGAAGCTG 729
BovB_Oa-RTE-Ovis_aries           TTCAA--CAT CACAGTAA-T CCAAGTCTAT -GCCCC---- -------AAC CACTAATGCT GAAGAAGCTG 753
BovB-RTE-Bos_taurus              TTCAA--TAT CACAGTAA-T CCAAGTCTAT -GCCCC---- -------AAC CAGTAACGCT GAAGAAGCTG 1351
BovB_Me-RTE-Macropus_eugenii     TTCAA--CAT CACAATAA-T AAAAATCTAT -GTTCCTGAT AGTCTATATT CATTGATGTA AAAGAGGTCA 608
BovB_Ano-RTE-Amphibolurus_norr   TTCAA--CAT CACAATAA-T CCAAGTTTAT -GCACC---- -------AAC CACCATTGCT GAGGAGACTG 800
BovB_Gd-RTE-Eremiascincus_doug   TTCAA--CAT CACAATAG-T CCAAGTCTAT -GCCCCC--- -------AAC CACTGGGGCT GAAGAGGAAG 385
BovB_Er-RTE-Eremiascincus_rich   TTCAA--CAT CACAATAG-T CCAAGTCTAT -GCCCC---- -------AAC CACTGGGGCT GAAGAGGAAG 923
BovB_Ca-RTE-Ctenotus_atlas       TTCAA--CAT CACAGTAA-T CCAAGTCTAT -GCCCC---- -------AAC CACTGATGCT GAAGAGGAAG 915
BovB_Es-RTE-Egernia_stokesii     TTCAA--CCT CACAGTAA-T TCAAATCTAT -GCTCC---- -------AAC CACTGACACT GAAGAGGATG 746
BovB_Tr-RTE-Tiliqua_rugosa       TTTAA--CCT CACAGTAA-T TCAAGTCTAT -GCTCC---- -------AAC CACTGACACT GAAGAGGATG 761
BovB_ALi-RTE-Amblyomma_limbatu   TTCAA--CAT ATCAATAG-T CCAAGTCTAT GACTCC---- -------AAC CACTGGTGCT GAAGAGGCTG 858
BovB_Hs-RTE-Hydrophis_spiralis   ---------- ---------- ---------- ---------- ---------- ---------- ---------- -
BovB_VA-RTE-Vipera_ammodytes     TTCAA--TAT CACAGTAG-T CCAAGTCTAT G-CCCC---- -------AAC CACCAGTGCT GAAGAGGATG 721
BovB_ACo-RTE-Agkistrodon_conto   TTCAA--TAT CACAGTAG-T CCAAGTCTAT G-CCCC---- -------AAC CACTGGTGCT GAAGAGGATG 723
BovB_Pg-RTE-Perameles_gunnii     ---------- ---------- ---------- ---------- ---------- ---------- ---------- -
BovB_Io-RTE-Isoodon_obesulus     ---------- ---------- ---------- ---------- ---------- ---------- ---------- -
BovB_Bp-RTE-Burramys_parvus      ---------- ---------- ---------- ---------- ---------- ---------- ---------- -
BovB_Pb-RTE-Petaurus_breviceps   ---------- ---------- ---------- ---------- ---------- ---------- ---------- -
BovB_Ma-RTE-Macropus_antilopin   ATATAATCTC AAAAAAGACA GAATGAGATC TGTTCAAATC CAAGGCAAAC CATTCAACAT CACAACAATA 807
BovB_Af-RTE-Antechinus_flavipe   ---------- ---------- ---------- ---------- ---------- ---------- ---GGGTAT 7
BovB_Ac-RTE-Anolis_carolinensi   TTTAA--CAT CACAGTGA-T CCAAATATAC -GCCCC---- -------AAC CACAGCTGCT GAAGAAGCAG 685
BovB_BM-RTE-Bombyx_mori          TTCATAAC-- -AAATGTCAT ACAAATATAT GCCCC----- -------AAC AGCCCAGGCC AAGTACGAGG 542
BovB_Dr-RTE-Christinus           CGCTCGCT-- -CACTTTCCT GCAGGTATAC GCGCC----- -------TAC TACTGATGCG AAAGTAGAGG 592
BovB_La-RTE-Loxodonta_africana   GTTAATAC-- -AACTATTAT TCAAATTTAC ACACC----- -------AAC CACTAAGGCC AAAGATGAAG 717
BovB_Pc-RTE-Procavia_capensis    GTTAATAC-- -GACTATTAT TCAAATTTAT GCACC----- -------AAC CACTAAGGCC AAAGATGAAG 719
BovB_Et-RTE-Christinus           ATCAATATTT GAACTATTAT TCAAATTTAT GTACC----- -------AAC CACAAAAGCT AGTGATGAAG 572
BovB_Ta-RTE-Tachyglossus_acule   TTCAGTAT-- -GACAATACT TCAGGTATAC GCCCC----- -------AAC AACAAATGCA GAGCAAGAA- 860
BovB_Plat-RTE-Ornithorhynchus    TTCAATAT-- -GACAATACT TCAGGTGTAC GCCCC----- -------AAC AACAGATGCA GAGCAAGAAG 845
BovB_Ec-RTE-Equus_caballus       ATTAACAT-- -AACCATCAT CCAAGTCTAT GCTCC----- -------AAC TACAGATGCT GAAGAAGATG 615
BovB_Cg-RTE-Christinus           ATCAACAT-- -AAAAATCAT TCAAGTTTAC ACCAC----- -------AAC TTCAGATGAT GAAGAGGAAG 255
BovB_Bh-RTE-Bothriocroton_hydr   ---------- ---------- ---------- ---------- ---------- ---------- ---------- -
BovB_Ml-RTE-Gehyra_variegata     ---------- ---------- ---------- ---------- ---------- ---------- ---------- -
Sly-rnd1_family284-LINE/RTEBov   ---------- ---------- ---------- ---------- ---------- ---------- ---------- -
Sly-rnd5_family2019-LINE/RTEBo   ---------- ---------- ---------- ---------- ---------- ---------- ---------- -
Sly-rnd3_family322-LINE/RTEBov   ---------- ---------- ---------- ---------- ---------- ---------- ---------- -
Sly-rnd5_family1698-LINE/RTEBo   ---------- ---------- ---------- ---------- ---------- ---------- ---------- -
Sly-rnd1_family795-LINE/RTEBov   ---------- ---------- ---------- ---------- ---------- ---------- ---------- -
Stu-rnd-5_family-3162_LINE_RTE   GAAGCAGCGC GAGGAACAGA CCCGACCGGC GAGCCTCTTT CTCTCTCTCC TCCTAAGCTT CAGTGAGGAC 279
Sly-rnd6_family1960-LINE/RTEBo   ---------- ---------- ---------- ---------- ---------- ---------- ---------- -
Stu-rnd-5_family-2493_LINE_RTE   ---------- ---------- ---------- ---------- ---------- ---------- ---------- -
Sly-rnd5_family447-LINE/RTEBov   ---------- ---------- ---------- ---------- ---------- ---------- ---------- -
Stu-rnd-6_family-2093_LINE_RTE   ---------- ---------- ---------- ---------- ---------- ---------- ---------- -
Consensus                        ---------- ---------- ---------- ---------- ---------- ---------- ----------
```

Conservation

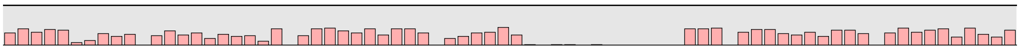

1,480 | 1,500 | 1,520 | 1,540

Stu-rnd-1_family-417_LINE_RTE-	----------	----------	----------	----------	----------	----------	----------	-
Sly-rnd1_family377-LINE/RTEBov	----------	----------	----------	----------	----------	----------	----------	-
Stu-rnd-1_family-489_LINE_RTE-	----------	----------	----------	----------	----------	----------	----------	-
Stu-rnd-1_family-607_LINE_RTE-	----------	----------	----------	----------	----------	----------	----------	-
Stu-rnd-1_family-645_LINE_RTE-	----------	----------	----------	----------	----------	----------	----------	-
Sly-rnd4_family81-LINE/RTEBovB	----------	----------	----------	----------	----------	----------	----------	-
Stu-rnd-6_family-408_LINE_RTE-	----------	----------	----------	----------	----------	----------	----------	-
Stu-rnd-1_family-351_LINE_RTE-	----------	----------	----------	----------	----------	----------	----------	-
Stu-rnd-1_family-382_LINE_RTE-	----------	----------	----------	----------	----------	----------	----------	-
Sly-rnd6_family746-LINE/RTEBov	----------	----------	----------	----------	----------	----------	----------	-
Stu-rnd-5_family-325_LINE_RTE-	----------	----------	----------	----------	----------	----------	----------	-
Sly-rnd4_family509-LINE/RTEBov	----------	----------	----------	----------	----------	----------	----------	-
Stu-rnd-1_family-513_LINE_RTE-	----------	----------	----------	----------	----------	----------	----------	-
Sly-rnd5_family1442-LINE/RTEBo	----------	----------	----------	----------	----------	----------	----------	-
BovB_Gl-RTE-Gehyra_lazelli	AA--------	GTGGACCA-G	TTCTATGAAG	ACCTACAACA	CCTCA-----	----------	--TAGAACTA	904
BovB_Gv-RTE-Gehyra_variegata	AA--------	GTGGACCA-G	TTCTATGAAG	ACTTACAACA	CCTCA-----	----------	--TAGAACTA	831
BovB_PMo-RTE-Python_molurus	AA--------	GTTGACCG-C	TTCTATGAAG	ACCTACAACA	CCTTC-----	----------	--TAGAACTA	773
BovB_Oa-RTE-Ovis_aries	AA--------	GTTGAATG-G	TTCTATGAAG	ACCTACAAGA	CCTTC-----	----------	--TAGAACTA	797
BovB-RTE-Bos_taurus	AA--------	GTTGAACG-G	TTCTATGAAG	ACCTACAAGA	CCTTT-----	----------	--TAGAACTA	1395
BovB_Me-RTE-Macropus_eugenii	GA--------	GTTGATCA-G	TTCTATGAAA	ACC---AATA	TCTTC-----	----------	--TAGAAATA	649
BovB_Ano-RTE-Amphibolurus_norr	AA--------	ATTGAACA-A	TTTTATGAAG	ATTTACAACA	CCTTC-----	----------	--TAGAACTG	844
BovB_Gd-RTE-Eremiascincus_doug	TA--------	GTTGACCG-T	TTCTATGAAG	ACCTACAAGA	CCTTC-----	----------	--TAGAAATA	429
BovB_Er-RTE-Eremiascincus_rich	TA--------	GTTGACCG-T	TTCTATGAAG	ACCTACAAGA	CCTTC-----	----------	--TAGAATTA	967
BovB_Ca-RTE-Ctenotus_atlas	AA--------	GTTGACCG-T	TTCTATGAAG	ACCTAGAACA	ACTTC-----	----------	--TAGAATTA	959
BovB_Es-RTE-Egernia_stokesii	AA--------	GTCAACCA-A	TTTTATGAAG	ACTTACAACA	CCTTC-----	----------	--TAGAACTA	790
BovB_Tr-RTE-Tiliqua_rugosa	AA--------	GTTAACCG-A	TTTTATGAAG	ACTTACAACA	CCTTC-----	----------	--TAGAAATA	805
BovB_ALi-RTE-Amblyomma_limbatu	AGGGGCTGAG	ATTGACAA-G	TTCTATGAAG	GCCTACAACA	CCTTCCAGAT	TGTGCCGCAT	TCTAGAATTA	927
BovB_Hs-RTE-Hydrophis_spiralis	----------	----------	----------	----------	----------	----------	----------	-
BovB_VA-RTE-Vipera_ammodytes	AA--------	ATTGACCG-G	TTCTGTGAAG	CCCTACAGCA	CCTTA-----	----------	--TAGAACTA	765
BovB_ACo-RTE-Agkistrodon_conto	AA--------	ATTGACCA-G	TTCTATGAAG	CCCTACAGCA	CCTTA-----	----------	--TAGAATTA	767
BovB_Pg-RTE-Perameles_gunnii	----------	----------	----------	----------	----------	----------	----------	-
BovB_Io-RTE-Isoodon_obesulus	----------	----------	----------	----------	----------	----------	----------	-
BovB_Bp-RTE-Burramys_parvus	----------	----------	----------	----------	----------	----------	----------	-
BovB_Pb-RTE-Petaurus_breviceps	----------	----------	----------	----------	----------	----------	----------	-
BovB_Ma-RTE-Macropus_antilopin	CAAGTCTATG	ATCCAAAC-A	CTAATGCCAA	AGAGGCCAAA	GTTGATCAGT	TCTATGAAGA	CCTACTAACA	876
BovB_Af-RTE-Antechinus_flavipe	AATCTACAAC	ATTAAGAA-A	TAATATCTAA	TTGAATCCAA	GCCAAACTAT	TCAGCATTAC	AATAATACAA	76
BovB_Ac-RTE-Anolis_carolinensi	AA--------	TTAAATAA-G	TTCTATGAGG	ATCTGCAGCA	CCTAC-----	----------	--TGGATAAC	729
BovB_BM-RTE-Bombyx_mori	AT--------	ATCGACGA-C	TTCTATCATT	CACTAGACGC	GACAC-----	----------	--TCGGATCA	586
BovB_Dr-RTE-Christinus	AT--------	ATCGAAAA-A	TTTTATGCGG	ATCTCCAACA	AGCAA-----	----------	--TTGATCAA	636
BovB_La-RTE-Loxodonta_africana	AA--------	ATAGAAGA-T	TTTTACCAAC	TG--GCAGTC	TGAAA-----	----------	--TTGATCGA	759
BovB_Pc-RTE-Procavia_capensis	AG--------	ATTGAAGA-T	TTTTACCAGC	TTCTGCAGTC	TGAAA-----	----------	--TTGATCGA	763
BovB_Et-RTE-Christinus	AA--------	ATAAAAGA-A	TTCCAACAAA	GACCTCAGGC	AGAAA-----	----------	--TTGATAAA	616
BovB_Ta-RTE-Tachyglossus_acule	----------	-TAAATAG-G	TTTTACGATG	AAGTACAGAG	AGAGA-----	----------	--TTGATAAA	901
BovB_Plat-RTE-Ornithorhynchus	AA--------	ATAGATAG-G	TTTTACGATG	AAGTGCAGAG	AGAGA-----	----------	--TTGATAAA	889
BovB_Ec-RTE-Equus_caballus	AA--------	ATTGAAAG-T	TTTTACGCAA	GTATCCAAGA	AGAAA-----	----------	--TTGATCAT	659
BovB_Cg-RTE-Christinus	AA--------	ATAGAAAGCT	TTTTATGCAA	GTATCCAAAA	AGAAA-----	----------	--TTGATCAC	300
BovB_Bh-RTE-Bothriocroton_hydr	----------	----------	----------	----------	----------	----------	----------	-
BovB_Ml-RTE-Gehyra_variegata	----------	----------	----------	----------	----------	----------	----------	-
Sly-rnd1_family284-LINE/RTEBov	----------	----------	----------	----------	----------	----------	----------	-
Sly-rnd5_family2019-LINE/RTEBo	----------	----------	----------	----------	----------	----------	----------	-
Sly-rnd3_family322-LINE/RTEBov	----------	----------	----------	----------	----------	----------	----------	-
Sly-rnd5_family1698-LINE/RTEBo	----------	----------	----------	----------	----------	----------	----------	-
Sly-rnd1_family795-LINE/RTEBov	----------	----------	----------	----------	----------	----------	----------	-
Stu-rnd-5_family-3162_LINE_RTE	GACAACGACG	AGTAGCAGCT	CCAGCGGCCC	GGACCAGCGA	GCGGCGAGCA	GCAAACTCCG	GCGAAGGCAA	349
Sly-rnd6_family1960-LINE/RTEBo	----------	----------	----------	----------	----------	----------	----------	-
Stu-rnd-5_family-2493_LINE_RTE	----------	----------	----------	----------	----------	----------	----------	-
Sly-rnd5_family447-LINE/RTEBov	----------	----------	----------	----------	----------	----------	----------	-
Stu-rnd-6_family-2093_LINE_RTE	----------	----------	----------	----------	----------	----------	----------	-
Consensus	----------	----------	----------	----------	----------	----------	----------	

Conservation

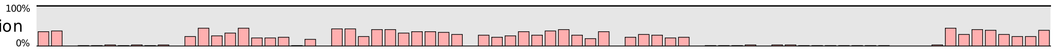

	1,560		1,580		1,600			
Stu-rnd-1_family-417_LINE_RTE-	----------	----------	----------	----------	----------	----------	----------	-
Sly-rnd1_family377-LINE/RTEBov	----------	----------	----------	----------	----------	----------	----------	-
Stu-rnd-1_family-489_LINE_RTE-	----------	----------	----------	----------	----------	----------	----------	-
Stu-rnd-1_family-607_LINE_RTE-	----------	----------	----------	----------	----------	----------	----------	-
Stu-rnd-1_family-645_LINE_RTE-	----------	----------	----------	----------	----------	----------	----------	-
Sly-rnd4_family81-LINE/RTEBovB	----------	----------	----------	----------	----------	----------	----------	-
Stu-rnd-6_family-408_LINE_RTE-	----------	----------	----------	----------	----------	----------	----------	-
Stu-rnd-1_family-351_LINE_RTE-	----------	----------	----------	----------	----------	----------	----------	-
Stu-rnd-1_family-382_LINE_RTE-	----------	----------	----------	----------	----------	----------	----------	-
Sly-rnd6_family746-LINE/RTEBov	----------	----------	----------	----------	----------	----------	----------	-
Stu-rnd-5_family-325_LINE_RTE-	----------	----------	----------	----------	----------	----------	----------	-
Sly-rnd4_family509-LINE/RTEBov	----------	----------	----------	----------	----------	----------	----------	-
Stu-rnd-1_family-513_LINE_RTE-	----------	----------	----------	----------	----------	----------	----------	-
Sly-rnd5_family1442-LINE/RTEBo	----------	----------	----------	----------	----------	----------	----------	-
BovB_Gl-RTE-Gehyra_lazelli	GCACCAAAAA	A-AGATGTTC	TCATCATCCT	AGGGGACTGG	AATGCT-AAA	GTAAGGTAGT	-CAAAAGGTA	971
BovB_Gv-RTE-Gehyra_variegata	GCACCAAAAA	A-AGATGTTC	TCATCATCCT	AGGGGACTGG	AATGCT-AAA	GTA-GGTAGT	-CAAAAGGTA	897
BovB_PMo-RTE-Python_molurus	GCACCAAAAA	A-AGATGTCC	TTTTCATCAT	AGGGGATTGG	AATGCT-AAA	GTA-GGAAGT	-CAAGGAGTA	839
BovB_Oa-RTE-Ovis_aries	ACACCAAAAA	A-AGATGTCC	TTTTCATCAT	AGGGGACTGG	AATGCA-AAA	GTA-GGAAGT	-CAAGAGATA	863
BovB-RTE-Bos_taurus	ACACCCAAAA	A-AGATGTCC	TTTTCATTAT	AGGGGACTGG	AATGCA-AAA	GTA-GGAAGT	-CAAGAAACA	1461
BovB_Me-RTE-Macropus_eugenii	ACACTATAAA	A-AAA-GTCA	CACTGTTTTT	AGGAGATTGG	AATGCT-AAA	CTA-GGAAAT	-AAAAAGATA	714
BovB_Ano-RTE-Amphibolurus_norr	ACACCAAAGA	A-AGATGTTC	TTCTCATTCT	AGGGGATTGG	AATGCT-AAA	GTA-GGGAGT	-CAAGAGATA	910
BovB_Gd-RTE-Eremiascincus_doug	ACACCAAAAA	ACGAATGTCC	TTTTCATCAT	AGGGGATTGG	AATGCT-AAA	GTA-GGCAGT	-CAAGAGGTA	496
BovB_Er-RTE-Eremiascincus_rich	ACACCAAAAA	A-AGATGTCC	TTCTCATCAT	AGGGGATTGG	AATGCT-AAA	GTA-GGCAGT	-CAAGAGGTA	1033
BovB_Ca-RTE-Ctenotus_atlas	ACACCCAAAA	A-AGATGTCC	TCCTCATCAT	AGGGGATTGG	AATGCT-AAA	GTA-GGCAGT	-CAAGAGGTA	1025
BovB_Es-RTE-Egernia_stokesii	ACACCAAAAA	A-AGACGTCC	TCCTTATTAC	AGGGGATGGG	AACACTTAAA	GTA-GGAAGT	-CAAGAGGTA	857
BovB_Tr-RTE-Tiliqua_rugosa	ACACCAAAAA	A-AGATGTCC	TCCTTATTAT	AGGGGATTGG	AATGCT-AAA	GTA-GGAAGT	-CAAGAGGTA	871
BovB_ALi-RTE-Amblyomma_limbatu	GCTCCCAAAA	A-TGATGTCC	TTATCATCAT	GGGGGATTAG	AATGCT-AAA	GTA-GGAAGC	-CAAAAGATA	993
BovB_Hs-RTE-Hydrophis_spiralis	----------	----------	----------	----------	----------	----------	----------	-
BovB_VA-RTE-Vipera_ammodytes	ACACCAAAAA	A-TGATGTCC	TTATCATCAT	GGGGGATTGG	AATGCT-AAA	GTA-GGAAGC	-CAAAAGATA	831
BovB_ACo-RTE-Agkistrodon_conto	ACACCAAAAA	A-TGATGTCC	TTATCATCAT	GGGGGATTGG	AATGCT-AAA	GTA-GGAAGC	-CAAAAGATA	833
BovB_Pg-RTE-Perameles_gunnii	----------	----------	----------	----------	----------	----------	----------	-
BovB_Io-RTE-Isoodon_obesulus	----------	----------	----------	----------	----------	----------	----------	-
BovB_Bp-RTE-Burramys_parvus	----------	----------	----------	----------	----------	----------	----------	-
BovB_Pb-RTE-Petaurus_breviceps	----------	----------	----------	----------	----------	----------	----------	-
BovB_Ma-RTE-Macropus_antilopin	TCTTCTAGAA	ATAACACAAA	AAAAAGATAT	CACATTCATC	ATAGAGGATT	GGA----ATG	-CTAAAGAAA	941
BovB_Af-RTE-Antechinus_flavipe	ATCTCCAGTC	ACTGAGGCCA	AAGAGGCTTA	AAATTGCTCA	GTTTTATGAA	GAC----CTA	-CAACAACTT	141
BovB_Ac-RTE-Anolis_carolinensi	ACACCAAAAA	G-AGACATTA	TTCTAATTAC	AGGAGATTGG	AATGCT-AAA	GTG-GGCAGT	-CAAATGACA	795
BovB_BM-RTE-Bombyx_mori	ATACCTAATC	G-AGAGATAA	CCTTTATTCC	TGGGGACTGG	AATGCAAAAG	TCGGTGAAAC	GACAGCGGAT	655
BovB_Dr-RTE-Christinus	GCACCAACAA	A-GGACACCA	TATATATTGG	TGGGGATTTC	AACGCAAAAG	TAGGCG----	--CTGATGAA	699
BovB_La-RTE-Loxodonta_africana	ACATGCAATC	A-AGATGCAC	TGATAATTAC	TGGCGATTGG	AATGCAAAAG	TTGGAAACA-	--AAAAAAAA	825
BovB_Pc-RTE-Procavia_capensis	ACATGCAATC	A-GGATGCAT	TGATAATTAC	TGGTGACTGG	AATGCAAAAG	TTGGAAACA-	--AAGAAGAA	829
BovB_Et-RTE-Christinus	AAATGCAATC	A-AGATGCAT	TGA---TTAT	TGGTAATTGG	AATGAAAAAG	TTG-AAACA-	--AAGAGGAA	678
BovB_Ta-RTE-Tachyglossus_acule	AGCTGCAAGC	A-AGATGTGT	TGATTATCAC	TGGGGATTGG	AATGCAAAAG	TTGGTAATG-	--GAATGGAA	967
BovB_Plat-RTE-Ornithorhynchus	AGCTGCAAGC	A-AGATGTGT	TGATTATCAC	TGGGGATTGG	AATGCAAAAG	TTGGTAATG-	--GAATGGAA	955
BovB_Ec-RTE-Equus_caballus	ACACCAAAAC	A-AGACATGC	TGATAATTAT	AGGTGACTGG	AACGCAAAAG	TAGGAAATA-	--AAGTGGAA	725
BovB_Cg-RTE-Christinus	ACACCTAAAC	A-AGATGTGC	TGATAATCAT	AGTTGACTGG	AATGCAAAAG	TAGGAAATA-	--AAGTAGAA	366
BovB_Bh-RTE-Bothriocroton_hydr	----------	----------	----------	----------	----------	----------	----------	-
BovB_Ml-RTE-Gehyra_variegata	----------	----------	----------	----------	----------	----------	----------	-
Sly-rnd1_family284-LINE/RTEBov	----------	----------	----------	----------	----------	----------	----------	-
Sly-rnd5_family2019-LINE/RTEBo	----------	----------	----------	----------	----------	----------	----------	-
Sly-rnd3_family322-LINE/RTEBov	----------	----------	----------	----------	----------	----------	----------	-
Sly-rnd5_family1698-LINE/RTEBo	----------	----------	----------	----------	----------	----------	----------	-
Sly-rnd1_family795-LINE/RTEBov	----------	----------	----------	----------	----------	----------	----------	-
Stu-rnd-5_family-3162_LINE_RTE	GAGCGTTTTT	CAGCTTCTCC	GGCGAACTCA	TCTATCTTCT	CCGGCGACCA	GCCCAGAAGA	GAGACGGCAG	419
Sly-rnd6_family1960-LINE/RTEBo	----------	----------	----------	----------	----------	----------	----------	-
Stu-rnd-5_family-2493_LINE_RTE	----------	----------	----------	----------	----------	----------	----------	-
Sly-rnd5_family447-LINE/RTEBov	----------	----------	----------	----------	----------	----------	----------	-
Stu-rnd-6_family-2093_LINE_RTE	----------	----------	----------	----------	----------	----------	----------	-
Consensus	----------	----------	----------	----------	----------	----------	----------	

Conservation

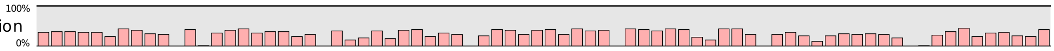

```
                                      1,620           1,640           1,660           1,680
Stu-rnd-1_family-417_LINE_RTE-    ---------- ---------- ---------- ---------- ---------- ---------- ---------- -
Sly-rnd1_family377-LINE/RTEBov    ---------- ---------- ---------- ---------- ---------- ---------- ---------- -
Stu-rnd-1_family-489_LINE_RTE-    ---------- ---------- ---------- ---------- ---------- ---------- ---------- -
Stu-rnd-1_family-607_LINE_RTE-    ---------- ---------- ---------- ---------- ---------- ---------- ---------- -
Stu-rnd-1_family-645_LINE_RTE-    ---------- ---------- ---------- ---------- ---------- ---------- ---------- -
Sly-rnd4_family81-LINE/RTEBovB    ---------- ---------- ---------- ---------- ---------- ---------- ---------- -
Stu-rnd-6_family-408_LINE_RTE-    ---------- ---------- ---------- ---------- ---------- ---------- ---------- -
Stu-rnd-1_family-351_LINE_RTE-    ---------- ---------- ---------- ---------- ---------- ---------- ---------- -
Stu-rnd-1_family-382_LINE_RTE-    ---------- ---------- ---------- ---------- ---------- ---------- ---------- -
Sly-rnd6_family746-LINE/RTEBov    ---------- ---------- ---------- ---------- ---------- ---------- ---------- -
Stu-rnd-5_family-325_LINE_RTE-    ---------- ---------- ---------- ---------- ---------- ---------- ---------- -
Sly-rnd4_family509-LINE/RTEBov    ---------- ---------- ---------- ---------- ---------- ---------- ---------- -
Stu-rnd-1_family-513_LINE_RTE-    ---------- ---------- ---------- ---------- ---------- ---------- ---------- -
Sly-rnd5_family1442-LINE/RTEBo    ---------- ---------- ---------- ---------- ---------- ---------- ---------- -
BovB_Gl-RTE-Gehyra_lazelli        ACTGGGATA- --------AC GGGCAAGTTT TGGCCTTGGA GAACAAAATG AAGCTGGGCA AAGGCTAACA 1032
BovB_Gv-RTE-Gehyra_variegata      GCTGGGATA- --------AC GGGCAAGTTT -GGCCTTGGA GAACAAAATG AAGCTGGGCA AAGGCTAACA 957
BovB_PMo-RTE-Python_molurus       ATCGGAATA- --------AC AGGCAAGTTT -GGCCTTGGA GTACAAAATG AAGCAGGGCA AAGGCTAATA 899
BovB_Oa-RTE-Ovis_aries            CCTGGAGTA- --------AC AGGCAAATTT -GGCCTTGGA GTACAAAATG AAGCAGGGCA AAGGCTAACA 923
BovB-RTE-Bos_taurus               CCTGGAGTA- --------AC AGGCAAATTT -GGCCTTGGA ATACGGAATG AAGCAGGGCA AAGACTAATA 1521
BovB_Me-RTE-Macropus_eugenii      ATTGGAATA- --------AC AGCCAAGTTT -GGCCTTGGA ATACAAAATG AAGCAAGG-A GAAGCCAATA 773
BovB_Ano-RTE-Amphibolurus_norr    AAAGGAATA- --------AC AGGGAAGTTT -GGCCTTGGA GTTCAAAACG AAGCAGGGCA AAGGCTAATA 970
BovB_Gd-RTE-Eremiascincus_doug    ATAGGAATA- --------AC AGGAAAGTTT -GGTCTTGGA ACACAAAATG AAGCAGGGCA AAGACTGATA 556
BovB_Er-RTE-Eremiascincus_rich    ATAGGAATA- --------AC AGGCAAGTTT -GGTCTTGGA GTACAAAATG AAGCAGGGCA AAGACTGATA 1093
BovB_Ca-RTE-Ctenotus_atlas        GTAGGAATT- --------AC AGGCAAGTTT -GGTCTTGGA GTACAAAATG AAGCAGGGCA AAGACTGATA 1085
BovB_Es-RTE-Egernia_stokesii      ACAAGAATA- --------AC AAGCAAGTTT -GACCTTGGA ACACAAAATG AAGCAGGACA AAGGCTAATA 917
BovB_Tr-RTE-Tiliqua_rugosa        ACAAGAATA- --------AC AGGCAAGTTT -GGCCTTGGA ATACAAAATG AAGCAGGGCA AAGGCTAATA 931
BovB_ALi-RTE-Amblyomma_limbatu    ACCGGAATA- --------AC AGGCAAGTTT -GGCCTTGGC GTACAAAATG AAGCAGGGCA CAGGCTGGTA 1053
BovB_Hs-RTE-Hydrophis_spiralis    ---------- ---------- ---------- ---------- ---------- ---------- ---------- -
BovB_VA-RTE-Vipera_ammodytes      ACCAGAATA- --------AC AGGCAAGTTT -GGCCTTGGA GTACAAAATG AAGCAGGGCA CAGGCTGATA 891
BovB_ACo-RTE-Agkistrodon_conto    AC-GGAATA- --------AC AGGCAAGTTT -GGCCTTGGA GTACAAAATG AAGCAGGGCA CAGGCTGATA 892
BovB_Pg-RTE-Perameles_gunnii      ---------- ---------- ---------- ---------- ---------- ---------- ---------- -
BovB_Io-RTE-Isoodon_obesulus      ---------- ---------- ---------- ---------- ---------- ---------- ---------- -
BovB_Bp-RTE-Burramys_parvus       ---------- ---------- ---------- ---------- ---------- ---------- ---------- -
BovB_Pb-RTE-Petaurus_breviceps    ---------- ---------- ---------- ---------- ---------- ---------- ---------- -
BovB_Ma-RTE-Macropus_antilopin    GAAACCAAA- --------AG ATAACTGGAA -TAACAGGCA AATTTGGCCT TGGAATAAAA AATGAAACAA 1001
BovB_Af-RTE-Antechinus_flavipe    TTAGAAATA- --------AC ACCAAAGAAA -AGATTTCAT ATTGATCATA GGGTATGGAA ATACTTAAGA 201
BovB_Ac-RTE-Anolis_carolinensi    ACCAGGATC- --------AC AGGCAAGCAT -GGTCTGGGA CAACAAAATG AAGCAGGACG TAGGCTGATA 855
BovB_BM-RTE-Bombyx_mori           GGACACATAA GACGCACTGT AGGAAATTTT -GGCTTGGGC AAACGAAATG ACCGTGGAGA ACGATTTTTA 724
BovB_Dr-RTE-Christinus            GAGCAAATG- -----ATCAT AGGCAATTTT -GGTTTAGGG GAACGGAACG AGGCTGGTGA CCGCTTGATC 762
BovB_La-RTE-Loxodonta_africana    GGATT-GTA- --------GT TGGAAAATAT -GGCCTTGGT GATAGAAACA ATGCCGGAGA TCAAATGATA 884
BovB_Pc-RTE-Procavia_capensis     GGATCAGTA- --------GT TGGAAAATAC -GGCCTTGGT GATAGAAATA ATGCCGGAGA TTGCATGATA 889
BovB_Et-RTE-Christinus            AGAACAATA- --------GT CAGA------ ----CTTGGT CACAGAAATA AAGCTGGAGA TCACATGACA 729
BovB_Ta-RTE-Tachyglossus_acule    ATGAAGGTT- --------GT TGGAAAATAT -GGCCTTGGA AATCAGAATC AAGCCAGGGA CCGACTTA-T 1026
BovB_Plat-RTE-Ornithorhynchus     ATGAAGGTT- --------GT TGGAAAATAT -GGCCTTGGA AATCGGAATC AAGCCGGGGA CCGACTTACT 1015
BovB_Ec-RTE-Equus_caballus        TCAAACATC- --------AT CGGAAAATTT -GGACTAGGG GTCAGAAACA AAGCAGGAGA CCGGCTCATG 785
BovB_Cg-RTE-Christinus            TCAAATATT- --------GT AGGAAAAAAT AGGAATATGA GCATGAAATG AAGCAGGAGA GCTACTCATA 427
BovB_Bh-RTE-Bothriocroton_hydr    ---------- ---------- ---------- ---------- ---------- ---------- ---------- -
BovB_Ml-RTE-Gehyra_variegata      ---------- ---------- ---------- ---------- ---------- ---------- ---------- -
Sly-rnd1_family284-LINE/RTEBov    ---------- ---------- ---------- ---------- ---------- ---------- ---------- -
Sly-rnd5_family2019-LINE/RTEBo    ---------- ---------- ---------- ---------- ---------- ---------- ---------- -
Sly-rnd3_family322-LINE/RTEBov    ---------- ---------- ---------- ---------- ---------- ---------- ---------- -
Sly-rnd5_family1698-LINE/RTEBo    ---------- ---------- ---------- ---------- ---------- ---------- ---------- -
Sly-rnd1_family795-LINE/RTEBov    ---------- ---------- ---------- ---------- ---------- ---------- ---------- -
Stu-rnd-5_family-3162_LINE_RTE    TGCTACTGTT TGCCGCCGGA GACCAACCAG ATCTGGCCGG AAATCANCCA GACCTGGCCG GAAAATTGCC 489
Sly-rnd6_family1960-LINE/RTEBo    ---------- ---------- ---------- ---------- ---------- ---------- ---------- -
Stu-rnd-5_family-2493_LINE_RTE    ---------- ---------- ---------- ---------- ---------- ---------- ---------- -
Sly-rnd5_family447-LINE/RTEBov    ---------- ---------- ---------- ---------- ---------- ---------- ---------- -
Stu-rnd-6_family-2093_LINE_RTE    ---------- ---------- ---------- ---------- ---------- ---------- ---------- -

Consensus                         ---------- ---------- ---------- ---------- ---------- ---------- ----------
```

Conservation

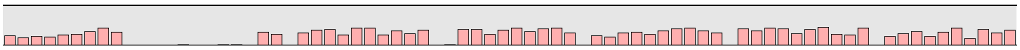

	1,700		1,720		1,740			
Stu-rnd-1_family-417_LINE_RTE-	----------	----------	----------	----------	----------	----------	----------	-
Sly-rnd1_family377-LINE/RTEBov	----------	----------	----------	----------	----------	----------	----------	-
Stu-rnd-1_family-489_LINE_RTE-	----------	----------	----------	----------	----------	----------	----------	-
Stu-rnd-1_family-607_LINE_RTE-	----------	----------	----------	----------	----------	----------	----------	-
Stu-rnd-1_family-645_LINE_RTE-	----------	----------	----------	----------	----------	----------	----------	-
Sly-rnd4_family81-LINE/RTEBovB	----------	----------	----------	----------	----------	----------	----------	-
Stu-rnd-6_family-408_LINE_RTE-	----------	----------	----------	----------	----------	----------	----------	-
Stu-rnd-1_family-351_LINE_RTE-	----------	----------	----------	----------	----------	----------	----------	-
Stu-rnd-1_family-382_LINE_RTE-	----------	----------	----------	----------	----------	----------	----------	-
Sly-rnd6_family746-LINE/RTEBov	----------	----------	----------	----------	----------	----------	----------	-
Stu-rnd-5_family-325_LINE_RTE-	----------	----------	----------	----------	----------	----------	----------	-
Sly-rnd4_family509-LINE/RTEBov	----------	----------	----------	----------	----------	----------	----------	-
Stu-rnd-1_family-513_LINE_RTE-	----------	----------	----------	----------	----------	----------	----------	-
Sly-rnd5_family1442-LINE/RTEBo	----------	----------	----------	----------	----------	----------	----------	-
BovB_Gl-RTE-Gehyra_lazelli	GAAGTTCTGT	CAAGAAAACA	TGCTGGTCAT	AGCGAA-CAC	CCTCTTTCAA	CAACCTAAGA	GGC-GACTCT	1100
BovB_Gv-RTE-Gehyra_variegata	GAA-TTCTGT	CAAGAAAACA	TGCTGGTCAT	AGCGAA-CAC	CCTCTTTCAA	CAACCTAAGA	GGC-GACTCT	1024
BovB_PMo-RTE-Python_molurus	GAG-TTCTGT	CAAGAGAACA	AGCTAGTCAT	AGCAAA-CAC	TCTTTTCCAA	CAACACAAGA	GAC-GACTCT	966
BovB_Oa-RTE-Ovis_aries	GAG-TTTTGC	CAAGAGAACG	CACTGGTCAT	AGCAAA-CAC	CCTCTTCCAA	CAACACAAGA	GAC-GACTCT	990
BovB-RTE-Bos_taurus	GAG-TTTTGC	CAAGAAAATG	CACTGGTCAT	AGCAAA-CAC	CCTCTTCCAA	CAACACAAGA	GAA-GACTCT	1588
BovB_Me-RTE-Macropus_eugenii	GAG-TTGTGT	CAAGAAAACT	TGCTGGTGAT	AACAAA-CAT	TCTTGTCCAA	CAACCGAAAA	AAGTGACTCT	841
BovB_Ano-RTE-Amphibolurus_norr	GAG-TTTTGT	CAAGAGAACA	AGCTGGTCAT	CACAAA-CAC	TCTTTTCCAA	CAACACAAGA	GGC-GACTCT	1037
BovB_Gd-RTE-Eremiascincus_doug	GAG-TTCTGT	CAGGAGAACA	CATTAGTCAT	AACAAA-CAC	CCTTTTCCAG	CAGCACAAAC	GGC-GACTGT	623
BovB_Er-RTE-Eremiascincus_rich	GAG-TTCTGT	CAGGAGAACA	CATTAGTCAT	AACAAA-CAC	CCTTTTCCAG	CAACACAAAC	GGC-GACTGT	1160
BovB_Ca-RTE-Ctenotus_atlas	GAG-TTTTGT	CAGGAGAACG	CATTAGTCAT	AGCAAA-CAC	CCTTTTCCAG	CAACACAAAC	GGC-GACTGT	1152
BovB_Es-RTE-Egernia_stokesii	GAA-TTTTGT	CAAGAGAACA	CACTGGTCAT	AGCAAA-CAC	CCTTTTCCTA	CAACCCAAGC	AGC-GACTCT	984
BovB_Tr-RTE-Tiliqua_rugosa	GAG-TTTTGT	CAAGAGAACA	CACTGGTCAT	AGCAAA-CAC	CCTTTTCCTA	CAACCCAAGC	GAC-GACTCT	998
BovB_ALi-RTE-Amblyomma_limbatu	GAA-TTTTGT	CAAGAGAATA	CGATGGTCAT	AGCTAA-CAC	TCTCTTTCAA	CAACCTAAGA	GAC-GACTCT	1120
BovB_Hs-RTE-Hydrophis_spiralis	----------	----------	----------	----------	----------	----------	----------	-
BovB_VA-RTE-Vipera_ammodytes	GAA-TTTTGT	CAAGAGAATA	CGATGGTCAT	AGCAAA-CAC	TCTTTTCCAA	CAACCCAAGA	GAC-GGCTGT	958
BovB_ACo-RTE-Agkistrodon_conto	GAA-TTTTGT	CAAGAGAATA	CGATGGTCAT	AGCAAA-CAC	TCTTTTCCAA	CAACCCAAGA	GAC-GGCTCT	959
BovB_Pg-RTE-Perameles_gunnii	----------	----------	----------	----------	----------	----------	----------	-
BovB_Io-RTE-Isoodon_obesulus	----------	----------	----------	----------	----------	----------	----------	-
BovB_Bp-RTE-Burramys_parvus	----------	----------	----------	----------	----------	----------	----------	-
BovB_Pb-RTE-Petaurus_breviceps	----------	----------	----------	----------	----------	----------	----------	-
BovB_Ma-RTE-Macropus_antilopin	GGCAGAGACT	GATAAAGTTT	TGTCAAGAAA	ACTCAC-TGG	TCAGAGCAAA	CACTCTTTTT	AAACAACCCA	1070
BovB_Af-RTE-Antechinus_flavipe	GTAGGAAATC	AAAACATAAT	TAGAATAAAA	AGCAAG-TTT	GGCTTTAGGA	TACAAAATGA	AGCAGGACAG	270
BovB_Ac-RTE-Anolis_carolinensi	GAA-TTTTGC	CAGGAAAACT	CACTGTGCAC	AACAAA-CAC	CCTCTTCCAA	CAACCTAAAA	GAACGGCTTT	923
BovB_BM-RTE-Bombyx_mori	GAA-TTTTGC	GTGGAAAAAG	ATCTCACGAT	TCTCAA-TAC	CTGCTTTCAA	CATCATGCGA	GGC-GACTGT	791
BovB_Dr-RTE-Christinus	CAG-TGCTGC	CAAGAAAATC	AATTAGTGAT	TGTAAA-CAC	CTGGTTTGAA	CAGCCGAAGC	GTC-GGTTAT	829
BovB_La-RTE-Loxodonta_africana	GAA-TTTTGC	AAGACCAACG	ACTTCTTCAT	TGCAAAATAC	CTTTTTTCAC	CAACATAAAC	GAC-GACTAT	952
BovB_Pc-RTE-Procavia_capensis	GAA-TTTTGC	AAGACTAATG	ACTTCTTCAT	TGCAAA-TAC	CTTTTTTAAC	CAACACAAAT	GGT-GACTGT	956
BovB_Et-RTE-Christinus	AAA-TTTTGC	AAAACCAACG	ACTTGCTCAT	AGAAAA-CAC	CTTTTTCAAA	-AACACAAAA	GGG-CACTAC	795
BovB_Ta-RTE-Tachyglossus_acule	GAT-TTCTGT	GAATCAAATG	ACTTTTTTAT	TGCCAA-CAC	CTTCTTTATC	CAACCTAAAC	GAC-GACTGT	1093
BovB_Plat-RTE-Ornithorhynchus	GAT-TTCTGT	GAATCAAATG	ACTTTTTTAT	TGCCAA-CAC	CTTCTTTATC	CAACCTAAAC	GAC-GACTGT	1082
BovB_Ec-RTE-Equus_caballus	GAT-TTCTGT	GAAGCCAACA	ATCTGTCCAT	CGCAAA-TAC	ATGCTTCAAG	CAACCGAACA	GAC-GACTGT	852
BovB_Cg-RTE-Christinus	GAA-TTCTGT	GAAGACGACT	----------	----------	----------	TAACCGTGAA	CAC-GATTGT	465
BovB_Bh-RTE-Bothriocroton_hydr	----------	----------	----------	----------	----------	----------	----------	-
BovB_Ml-RTE-Gehyra_variegata	----------	----------	----------	----------	----------	----------	----------	-
Sly-rnd1_family284-LINE/RTEBov	----------	----------	----------	----------	----------	----------	----------	-
Sly-rnd5_family2019-LINE/RTEBo	----------	----------	----------	----------	----------	----------	----------	-
Sly-rnd3_family322-LINE/RTEBov	----------	----------	----------	----------	----------	----------	----------	-
Sly-rnd5_family1698-LINE/RTEBo	----------	----------	----------	----------	----------	----------	----------	-
Sly-rnd1_family795-LINE/RTEBov	----------	----------	----------	----------	----------	----------	----------	-
Stu-rnd-5_family-3162_LINE_RTE	CATTGACCAC	CGAAGCCCTC	CATCTTCACC	GACTCAGGTT	CTATCTATCC	TTTTCTCTCT	CTTTTTCGCT	559
Sly-rnd6_family1960-LINE/RTEBo	----------	----------	----------	----------	----------	----------	----------	-
Stu-rnd-5_family-2493_LINE_RTE	----------	----------	----------	----------	----------	----------	----------	-
Sly-rnd5_family447-LINE/RTEBov	----------	----------	----------	----------	----------	----------	----------	-
Stu-rnd-6_family-2093_LINE_RTE	----------	----------	----------	----------	----------	----------	----------	-
Consensus	----------	----------	----------	----------	----------	----------	----------	

Conservation

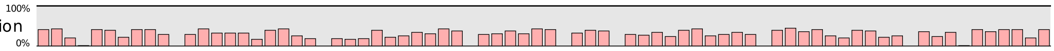

```
                                   1,760        1,780        1,800        1,820
Stu-rnd-1_family-417_LINE_RTE-     ---------- ---------- ---------- ---------- ---------- ---------- ---------- -
Sly-rnd1_family377-LINE/RTEBov     ---------- ---------- ---------- ---------- ---------- ---------- ---------- -
Stu-rnd-1_family-489_LINE_RTE-     ---------- ---------- ---------- ---------- ---------- ---------- ---------- -
Stu-rnd-1_family-607_LINE_RTE-     ---------- ---------- ---------- ---------- ---------- ---------- ---------- -
Stu-rnd-1_family-645_LINE_RTE-     ---------- ---------- ---------- ---------- ---------- ---------- ---------- -
Sly-rnd4_family81-LINE/RTEBovB     ---------- ---------- ---------- ---------- ---------- ---------- ---------- -
Stu-rnd-6_family-408_LINE_RTE-     ---------- ---------- ---------- ---------- ---------- ---------- ---------- -
Stu-rnd-1_family-351_LINE_RTE-     ---------- ---------- ---------- ---------- ---------- ---------- ---------- -
Stu-rnd-1_family-382_LINE_RTE-     ---------- ---------- ---------- ---------- ---------- ---------- ---------- -
Sly-rnd6_family746-LINE/RTEBov     ---------- ---------- ---------- ---------- ---------- ---------- ---------- -
Stu-rnd-5_family-325_LINE_RTE-     ---------- ---------- ---------- ---------- ---------- ---------- ---------- -
Sly-rnd4_family509-LINE/RTEBov     ---------- ---------- ---------- ---------- ---------- ---------- ---------- -
Stu-rnd-1_family-513_LINE_RTE-     ---------- ---------- ---------- ---------- ---------- ---------- ---------- -
Sly-rnd5_family1442-LINE/RTEBo     ---------- ---------- ---------- ---------- ---------- ---------- ---------- -
BovB_Gl-RTE-Gehyra_lazelli         ACACATGGAC ATCACCAGAT --GGGCAACA CCGAAATCAG ATTGATTATA TCATCTG-CA GTCAAAGATG 1167
BovB_Gv-RTE-Gehyra_variegata       ACACATGGAC ATCACCAGAT --GGGCAACA CCGAAATCAG ATTGATTATA TCATCTG-CA GTCAAAGATG 1091
BovB_PMo-RTE-Python_molurus        ACACATGGAC ATCACCAGAT --GGTCAATA CCGAAACCAG ATTGATTATA TACTTTG-CA GCCAAAGATG 1033
BovB_Oa-RTE-Ovis_aries             ACACATGGAC ATCACCAGAT --GGTCAATA CCGAAATCAG ATTGATTATA TTCTTTG-CA GCCAAAGATG 1057
BovB-RTE-Bos_taurus                ATACATGGAC ATCACCAGAT --GGTCAACA CCGAAATCAG ATTGATTATA TTCTTTG-CA GCCAAAGATG 1655
BovB_Me-RTE-Macropus_eugenii       ATACATGGAT GTCACCATAT AGGGTCAATA TTGAAATCAG ATTGAATATA TACTTTG-TA GTCAAAGGTG 910
BovB_Ano-RTE-Amphibolurus_norr     ATACATGGAA ATCACCAGAT --GGGCAATA CCGAAATCAG ATTGATTATA TTCTCTG-CA GCCAAAGATG 1104
BovB_Gd-RTE-Eremiascincus_doug     ACACATGGAC TTCACCAGAT --GGTCTACA TCGAAATCAG ATTGATTACA TTCTTTG-CA GCCAAAGGTG 690
BovB_Er-RTE-Eremiascincus_rich     ACACATGGAC TTCACCAGAT --GGTCTACA CCGAAATCAG ATTGATTACA TTCTTTG-CA GCCAAAGGTG 1227
BovB_Ca-RTE-Ctenotus_atlas         ACACATGGAC TTCACCAGAT --GGTCTACA TCGAAATCAG ATTGATTACA TCCTTTG-CA GCCAAAGGTG 1219
BovB_Es-RTE-Egernia_stokesii       ACACATGGAA ATCACCAGAT --GATCTACA CTGAAATCAG ACTGAATATG TTCTCTG-CA GCCAAAGATG 1051
BovB_Tr-RTE-Tiliqua_rugosa         ACACATGGAC ATCACCAGAT --AGTCTACA CTGAAATCAG ATTGATTATA TTCTCTG-CA GCCAAAGATG 1065
BovB_ALi-RTE-Amblyomma_limbatu     ACACATGGAC AGCACCTTAT --GGCCAACA CATAAATCAG ATTGACTATG TGATCTGACA GCCAAAGATG 1188
BovB_Hs-RTE-Hydrophis_spiralis     ---------- ---------- ---------- ---------- ---------- ---------- ---------- -
BovB_VA-RTE-Vipera_ammodytes       ACACATGGAC ATCACCAGAT --GGTCAATA CAGAAATCAG ATTGACTATG TGCTCTG-CA GCCAAAGATG 1025
BovB_ACo-RTE-Agkistrodon_conto     ACACATGGAC ATCACCAGAC --GGTCAACA CAGAAATCAG ATTGACTATG TGCTCTG-CA GCCAAAGATG 1026
BovB_Pg-RTE-Perameles_gunnii       ---------- ---------- ---------- ---------- ---------- ---------- ---------- -
BovB_Io-RTE-Isoodon_obesulus       ---------- ---------- ---------- ---------- ---------- ---------- ---------- -
BovB_Bp-RTE-Burramys_parvus        ---------- ---------- ---------- ---------- ---------- ---------- ---------- -
BovB_Pb-RTE-Petaurus_breviceps     ---------- ---------- ---------- ---------- ---------- ---------- ---------- -
BovB_Ma-RTE-Macropus_antilopin     AAAGGCAACT CTACACATGG ACATCACCAG ATAGTCAATA TCAAAATCAG ATTGATTATA CACTTTGCAG 1140
BovB_Af-RTE-Antechinus_flavipe     AGACAAACAG AGTTTTATAA CTCTATTTGC AACTTTCTGC TCATAGCAAA CATACTTCTT TTCAACAATC 340
BovB_Ac-RTE-Anolis_carolinensi     ACACATGGAC TTCACCAGAT --GGTCAACA CCGAAATCAA ATTGACTACA TACTCTG-CA GACAAAGGTG 990
BovB_BM-RTE-Bombyx_mori            ATACATGGAA ATCACCAGGT --GACAGATT CAGGAACCAG ATTAACTACA TATTGAT-CA ACTCTCGCTG 858
BovB_Dr-RTE-Christinus             ACACATGGAC GGCCCCAAAT --G---GATA TCGCAACCAG ATTGACTACA TCCTCTG-TC AAAAACGCTG 893
BovB_La-RTE-Loxodonta_africana     ACACATGGAC CTCACCAGAT --GGAACACA CAGAAATCAA ATCGACTACA TCTGTGG-AA AGAGACGATG 1019
BovB_Pc-RTE-Procavia_capensis      ACACATGGAC CTCACCAGAT --GGAATACA CAGGAACCAA ATTGACTACA TCTGTGG-AA AAAGATGATG 1023
BovB_Et-RTE-Christinus             ACACATGGAC TTCCCCAGAT --GAAATACA CAGAAATCAA ATTGACTACA TTTGTGG-GA AGTCAGGATG 862
BovB_Ta-RTE-Tachyglossus_acule     ACACATGGAC TTCACCAGAT --GGACTATA TAGAAATCAA ATAGACTATA TTATTTG-TA GAAAGAGATG 1160
BovB_Plat-RTE-Ornithorhynchus      ACACATGGAC TTCACCAGAT --GGACTATA TAGAAATCAA ATAGACTATA TTATTTG-TA GAAAGAGATG 1149
BovB_Ec-RTE-Equus_caballus         ATACATGGAC ATCACCAGAT --GGCCAATA TAGAAATCAA ATAGACTATG TAATTGG-AA GCAGGAGATG 919
BovB_Cg-RTE-Christinus             ATATGTGGAC ATCACTAGAT --GGCCAATA CAGAAATCAA ATAGATTACA TAATAGG-AA AAAAAAGATG 532
BovB_Bh-RTE-Bothriocroton_hydr     ---------- ---------- ---------- ---------- ---------- ---------- ---------- -
BovB_Ml-RTE-Gehyra_variegata       ---------- ---------- ---------- ---------- ---------- ---------- ---------- -
Sly-rnd1_family284-LINE/RTEBov     ---------- ---------- ---------- ---------- ---------- ---------- ---------- -
Sly-rnd5_family2019-LINE/RTEBo     ---------- ---------- ---------- ---------- ---------- ---------- ---------- -
Sly-rnd3_family322-LINE/RTEBov     ---------- ---------- ---------- ---------- ---------- ---------- ---------- -
Sly-rnd5_family1698-LINE/RTEBo     ---------- ---------- ---------- ---------- ---------- ---------- ---------- -
Sly-rnd1_family795-LINE/RTEBov     ---------- ---------- ---------- ---------- ---------- ---------- ---------- -
Stu-rnd-5_family-3162_LINE_RTE     GAACCTAGTT GCTGCTGGGC CCTGTCTTGC AGACTCCTTA TCCCGGAACA GTACTTCCCA GTTTTCTTAA 629
Sly-rnd6_family1960-LINE/RTEBo     ---------- ---------- ---------- ---------- ---------- ---------- ---------- -
Stu-rnd-5_family-2493_LINE_RTE     ---------- ---------- ---------- ---------- ---------- ---------- ---------- -
Sly-rnd5_family447-LINE/RTEBov     ---------- ---------- ---------- ---------- ---------- ---------- ---------- -
Stu-rnd-6_family-2093_LINE_RTE     ---------- ---------- ---------- ---------- ---------- ---------- ---------- -
Consensus                          ---------- ---------- ---------- ---------- ---------- ---------- ----------
```

Conservation (100% / 0%)

```
                                            1,840                1,860                1,880
Stu-rnd-1_family-417_LINE_RTE-       ---------- ---------- ---------- ---------- ---------- ---------- ---------- -
Sly-rnd1_family377-LINE/RTEBov       ---------- ---------- ---------- ---------- ---------- ---------- ---------- -
Stu-rnd-1_family-489_LINE_RTE-       ---------- ---------- ---------- ---------- ---------- ---------- ---------- -
Stu-rnd-1_family-607_LINE_RTE-       ---------- ---------- ---------- ---------- ---------- ---------- ---------- -
Stu-rnd-1_family-645_LINE_RTE-       ---------- ---------- ---------- ---------- ---------- ---------- ---------- -
Sly-rnd4_family81-LINE/RTEBovB       ---------- ---------- ---------- ---------- ---------- ---------- ---------- -
Stu-rnd-6_family-408_LINE_RTE-       ---------- ---------- ---------- ---------- ---------- ---------- ---------- -
Stu-rnd-1_family-351_LINE_RTE-       ---------- ---------- ---------- ---------- ---------- ---------- ---------- -
Stu-rnd-1_family-382_LINE_RTE-       ---------- ---------- ---------- ---------- ---------- ---------- ---------- -
Sly-rnd6_family746-LINE/RTEBov       ---------- ---------- ---------- ---------- ---------- ---------- ---------- -
Stu-rnd-5_family-325_LINE_RTE-       ---------- ---------- ---------- ---------- ---------- ---------- ---------- -
Sly-rnd4_family509-LINE/RTEBov       ---------- ---------- ---------- ---------- ---------- ---------- ---------- -
Stu-rnd-1_family-513_LINE_RTE-       ---------- ---------- ---------- ---------- ---------- ---------- ---------- -
Sly-rnd5_family1442-LINE/RTEBo       ---------- ---------- ---------- ---------- ---------- ---------- ---------- -
BovB_Gl-RTE-Gehyra_lazelli           GAGAAGCTCT A-TACAGTCA GCAAAAACAA G---ACCTGG AGCTGACTGT GGCTCAGATC ATGAGCTACT 1233
BovB_Gv-RTE-Gehyra_variegata         GAGAAGCTCT A-TACAGTCA GCAAAAACAA G---ACCTGG AGCTGACTGT GGCTCAGATC ATGAGCTACT 1157
BovB_PMo-RTE-Python_molurus          GAGAAGCTCT A-TACAGACA GCAAAAACAA G---ACCAGG AGCTGACTGT GGCTCAGATC ATGAGCTTCT 1099
BovB_Oa-RTE-Ovis_aries               GAGAAGCTCT A-TACAGTCA GCAAAAACAA G---ACCTGG AGCTGACTGT GGCTCAGATC ATGAACTCCT 1123
BovB-RTE-Bos_taurus                  GAGAAGCTCT A-TACAGTCA GCAAAAACAA G---ACCAGG AGCTGACTGT GGCTCAGACC ATGAACTCCT 1721
BovB_Me-RTE-Macropus_eugenii         GAAAAGCTCT A-TGCAGTCA GTTAAAAAAA AGATACCTGG AGCTGAGTGT GGCTCAGATC TTGAGTTTCT 979
BovB_Ano-RTE-Amphibolurus_norr       GAGAAGCTCT A-TACAGTCA GCAAAAACAA G---ACCTGG AGCTGATTGT GGCTCTGATC ATCAGCTTCT 1170
BovB_Gd-RTE-Eremiascincus_doug       GAGAAGCTCT A-TACAGGCA GTCAAAACAA G---ACCTGG AGCTGACTGT GGCTCAGATC ATGAGCTTCT 756
BovB_Er-RTE-Eremiascincus_rich       GAGAAGCTCT A-TACAGGCA GTCAAAACAA G---ACCTGG AGCTGACTGT GGCTCAGATC ATGAGCTTCT 1293
BovB_Ca-RTE-Ctenotus_atlas           GAGAAGCTCT A-TACAGGCA GTAAAAACAA G---ACCTGG AGCTGACTGT GGCTCAGATC ATGAGCTTCT 1285
BovB_Es-RTE-Egernia_stokesii         GAGAAGCTCT A-TACAGTCA GTAAAAACAA A---ACCTGG AGCTGACTGT GGATCAGATC ATGAGCTTCT 1117
BovB_Tr-RTE-Tiliqua_rugosa           GAGAAGCTCT A-CACAGTCA GTAAAAACAA G---ACCTGA AGCTGACTGC AGATCAGATC ATGAGCATCT 1131
BovB_ALi-RTE-Amblyomma_limbatu       GAGAAGTTCT AGTACAGTCA GTAAAAACTA G---ACCACG AACTGATTGT GGCTCAGATC ATCAGCTTCT 1255
BovB_Hs-RTE-Hydrophis_spiralis       ---------- ---------- ---------- ---------- ---------- ---------- ---------- -
BovB_VA-RTE-Vipera_ammodytes         GAGAAGCTCT A-TACAGTCA GTAAAAACAA G---ACCAGG AGCTGACTGT GGCTCAGACC ATGAGCTTCT 1091
BovB_ACo-RTE-Agkistrodon_conto       GAGAAGCTCT A-TACAGTCA GTAAAAACAA G---ACCAGG AGCTGACTGT GGCTCAGATC ATGAGCTTCT 1092
BovB_Pg-RTE-Perameles_gunnii         ---------- ---------- ---------- ---------- ---------- ---------- ---------- -
BovB_Io-RTE-Isoodon_obesulus         ---------- ---------- ---------- ---------- ---------- ---------- ---------- -
BovB_Bp-RTE-Burramys_parvus          ---------- ---------- ---------- ---------- ---------- ---------- ---------- -
BovB_Pb-RTE-Petaurus_breviceps       ---------- ---------- ---------- ---------- ---------- ---------- ---------- -
BovB_Ma-RTE-Macropus_antilopin       CCAAAGGTGA AGAAGCAATA TACAGTCAGT TAAAGCAAAA CCTGAAGCTG ACTGTGACTC AGATCATGAG 1210
BovB_Af-RTE-Antechinus_flavipe       CAAAAGATGA TTACATACGT AGACATCACC AGATAGATGG TCGATATAGA AATAAAATTG ATTATTTACT 410
BovB_Ac-RTE-Anolis_carolinensi       GCAGACATCC A-TCCAGTCG GCGAAAACAA G---ACCTGG AGCTGACTGT AGCTCAAATC ACAAACTTCT 1056
BovB_BM-RTE-Bombyx_mori              GAGGTCCACC G-TATCAAAT GTCAAGACCT A---TCCTGG GGCGGACTGT GGTTCAGACC ATGTGCTCCT 924
BovB_Dr-RTE-Christinus               GAAGAACTCA G-TACAGGTA GTCAAAACGC T---TCCTGG TGCAGACTGC GGCTCTGACC ACCAGCTACT 959
BovB_La-RTE-Loxodonta_africana       GAAAAGCTCA A-TATCATCA GTCAGAACAA G---GCCAGG GGCCGACTGT GGAACAGACC ATCAATTGCT 1085
BovB_Pc-RTE-Procavia_capensis        GAAAAGCTCC A-TATCAGCA GTCAGAACAA G---ACCAGG TGCTGACTGT GGCACAGATC ATCAATTGCT 1089
BovB_Et-RTE-Christinus               GAGAAGCTTG A-TATCAACC GCTAAAACCA G---ACCAGG AGCTGACTGT GGAACAGACC ATCAATGGCT 928
BovB_Ta-RTE-Tachyglossus_acule       GAGGAGTTCT A-TTTTGGCA GCAAAAACAT G---GCCTGG AGCTGATTGT GGAACAGATC ACGAGCTGTT 1226
BovB_Plat-RTE-Ornithorhynchus        GAGGAGTTCT A-TTTTGGCA GCAAAAACAT G---GCCTGG AGCTGACTGT GGAACAGATC ATGAGCTGTT 1215
BovB_Ec-RTE-Equus_caballus           GAGAAGCTGT A-TTCTCTCT GCCAAAACAA G---ACCAGG AGCAGATTGT GGTACTGACC ACGAACTGTT 985
BovB_Cg-RTE-Christinus               GAGAA-CAGT T-GACACACA GCCTTTACAA A---CTTGGG AA-AGATTTT G--------- ---------- 577
BovB_Bh-RTE-Bothriocroton_hydr       ---------- ---------- ---------- ---------- ---------- ---------- ---------- -
BovB_Ml-RTE-Gehyra_variegata         ---------- ---------- ---------- ---------- ---------- ---------- ---------- -
Sly-rnd1_family284-LINE/RTEBov       ---------- ---------- ---------- ---------- ---------- ---------- ---------- -
Sly-rnd5_family2019-LINE/RTEBo       ---------- ---------- ---------- ---------- ---------- ---------- ---------- -
Sly-rnd3_family322-LINE/RTEBov       ---------- ---------- ---------- ---------- ---------- ---------- ---------- -
Sly-rnd5_family1698-LINE/RTEBo       ---------- ---------- ---------- ---------- ---------- ---------- ---------- -
Sly-rnd1_family795-LINE/RTEBov       ---------- ---------- ---------- ---------- ---------- ---------- ---------- -
Stu-rnd-5_family-3162_LINE_RTE       TAAAGCTCTG ACCTGTGCTT CAGTCTTTAG TAGAGTGGGT GCTATCGTGG CCTCTGGATT ACACACTGTT 699
Sly-rnd6_family1960-LINE/RTEBo       ---------- ---------- ---------- ---------- ---------- ---------- ---------- -
Stu-rnd-5_family-2493_LINE_RTE       ---------- ---------- ---------- ---------- ---------- ---------- ---------- -
Sly-rnd5_family447-LINE/RTEBov       ---------- ---------- ---------- ---------- ---------- ---------- ---------- -
Stu-rnd-6_family-2093_LINE_RTE       ---------- ---------- ---------- ---------- ---------- ---------- ---------- -

Consensus                            ---------- ---------- ---------- ---------- ---------- ---------- ----------
                                100%
Conservation
                                  0%
```

1,900 | 1,920 | 1,940 | 1,960

Stu-rnd-1_family-417_LINE_RTE-	----------	----------	----------	----------	----------	----------	----------	-
Sly-rnd1_family377-LINE/RTEBov	----------	----------	----------	----------	----------	----------	----------	-
Stu-rnd-1_family-489_LINE_RTE-	----------	----------	----------	----------	----------	----------	----------	-
Stu-rnd-1_family-607_LINE_RTE-	----------	----------	----------	----------	----------	----------	----------	-
Stu-rnd-1_family-645_LINE_RTE-	----------	----------	----------	----------	----------	----------	----------	-
Sly-rnd4_family81-LINE/RTEBovB	----------	----------	----------	----------	----------	----------	----------	-
Stu-rnd-6_family-408_LINE_RTE-	----------	----------	----------	----------	----------	----------	----------	-
Stu-rnd-1_family-351_LINE_RTE-	----------	----------	----------	----------	----------	----------	----------	-
Stu-rnd-1_family-382_LINE_RTE-	----------	----------	----------	----------	----------	----------	----------	-
Sly-rnd6_family746-LINE/RTEBov	----------	----------	----------	----------	----------	----------	----------	-
Stu-rnd-5_family-325_LINE_RTE-	----------	----------	----------	----------	----------	----------	----------	-
Sly-rnd4_family509-LINE/RTEBov	----------	----------	----------	----------	----------	----------	----------	-
Stu-rnd-1_family-513_LINE_RTE-	----------	----------	----------	----------	----------	----------	----------	-
Sly-rnd5_family1442-LINE/RTEBo	----------	----------	----------	----------	----------	----------	----------	-
BovB_Gl-RTE-Gehyra_lazelli	CAT-TGCAAA	ATTTAGGCTT	AAACTGAAGA	AA-ATTGGGA	AAAC--CACT	AGGCC-TTTC	AGGTTTGACT	1298
BovB_Gv-RTE-Gehyra_variegata	CAT-TGCAAA	ATTTAGGCTT	AAACTGAAGA	AA-ATTGGGA	AAAC--CACT	AGGCCCTTTC	AGGTTTAACT	1223
BovB_PMo-RTE-Python_molurus	TAC-TGCAAA	ATTTAGGCTT	AAATTGAAGA	AT-GTAGGGA	AAAC--CACT	AGACC-ACTC	AGGTATGACT	1164
BovB_Oa-RTE-Ovis_aries	TAT-TGCAAA	ATTCAGACTT	AAATTGAAGA	AA-GTAGGGA	AAAC--CACT	AGACC-ATTC	AGGTATGACC	1188
BovB-RTE-Bos_taurus	TAT-TGCCAA	ATTCAGACTT	AAATTGAAGA	AA-GTAGGGA	AAAC--CACT	AGACC-ATTC	AGGTATGACC	1786
BovB_Me-RTE-Macropus_eugenii	TGC-TGAAAA	ATTCAGAC--	AAATTGAAGA	AA-GTAGGGA	AATC--AATC	ATACC-ATAT	AGGTATGACA	1042
BovB_Ano-RTE-Amphibolurus_norr	CAT-AGCAAA	ATTCAAGCTT	AGACTGAAGA	GA-GTAGGAA	AAAC--CACT	GGGCC-ACTC	AGGTATAATC	1235
BovB_Gd-RTE-Eremiascincus_doug	TATAGGCAAA	ATTCAGGCTT	AAACTTAAAA	AA-GTAGGGA	AAAC--CACT	AGGCC-CTCC	ACGTATGACC	822
BovB_Er-RTE-Eremiascincus_rich	TAT-GGCAAA	ATTCAGGCTT	AAACTGAAAA	AA-GTAGGGA	AAAC--CACT	AGGCC-CTCC	ACGTATGACC	1358
BovB_Ca-RTE-Ctenotus_atlas	TAT-TGCAAA	ATTCAGGCTT	AAACTGAAAA	AAAGTAGGGA	AAAC--CACT	AGGCC-CTCC	ACGTATGACC	1351
BovB_Es-RTE-Egernia_stokesii	TAT-TGCAAA	ATTCAGGCTT	AAACTGAAGA	AA-GTAGGGA	AAAAAACATT	AGGCC-ATTC	AGGTACGACC	1184
BovB_Tr-RTE-Tiliqua_rugosa	TAT-TACAAA	ATTCAGGCTT	AAACTGAAGA	AA-GTAGGGA	AAAA--CATT	AGGCC-AATC	AGGTATGACC	1196
BovB_ALi-RTE-Amblyomma_limbatu	TTT-TGCAAA	ATTTAGGCTT	AAGTTGAAGA	AA-GTAGGAA	AAAG--CACA	ATGCC-ACTC	AAGTATTACC	1320
BovB_Hs-RTE-Hydrophis_spiralis	----------	----------	----------	----------	----------	----------	----------	-
BovB_VA-RTE-Vipera_ammodytes	CGT-TGCAAA	GTTTAGGCTT	AAACTGAAGA	AA-GTAGGGA	AAAG--CACT	AGGCC-ACTC	AGGTATGAAT	1156
BovB_ACo-RTE-Agkistrodon_conto	CGT-TGCAAA	ATTTAGGCTT	AAATTGAAGA	AA-GTAGGGA	AAA---CACT	AGGCC-ACTC	AGGTATGAAC	1156
BovB_Pg-RTE-Perameles_gunnii	----------	----------	----------	----------	----------	----------	----------	-
BovB_Io-RTE-Isoodon_obesulus	----------	----------	----------	----------	----------	----------	----------	-
BovB_Bp-RTE-Burramys_parvus	----------	----------	----------	----------	----------	----------	----------	-
BovB_Pb-RTE-Petaurus_breviceps	----------	----------	----------	----------	----------	----------	----------	-
BovB_Ma-RTE-Macropus_antilopin	TTTCTTATTC	CAAAATTCAC	ACTTAAATGG	AAGAAAGTAG	GAAA--AACC	ATCAGAACAT	ATAGGTATGA	1278
BovB_Af-RTE-Antechinus_flavipe	TTGCCATGAA	AGGCAAAGTT	CTATATAGTC	AGTTAAAACG	CAAA--ATTC	AGACGAAAAT	TTGAAGAAAG	478
BovB_Ac-RTE-Anolis_carolinensi	TAT-TGCAAA	ATTCAGAATC	AAACTAAAGA	GA-ATGGGGA	AAAT--ACAC	AGACC-AATT	AGATATGACA	1121
BovB_BM-RTE-Bombyx_mori	GGT-TGCAAA	CTTCCGCCTG	CGCCTCAAAG	CG--CAAAAA	CGTA----AA	TGCAATTTAT	AGACCCGG-A	986
BovB_Dr-RTE-Christinus	CAT-TGCAAA	AGTCAAATTT	CGATTCAACA	CCATCAAAAG	GCTT----CC	TACAATGCGC	CGTTTTGACA	1024
BovB_La-RTE-Loxodonta_africana	CAT-ATGCAA	GTAAAAGC--	AAACTGAAGA	AAATCAGAGC	AAGT----CC	ATGAGAGCCA	AAGTACAACC	1148
BovB_Pc-RTE-Procavia_capensis	CAT-ATGCAA	ATTCAAATTG	AAACTGAAGA	AAATTAGAAC	AAGT----CC	ACGAGAGCCA	AAGTATGACC	1154
BovB_Et-RTE-Christinus	CCT-ATATAA	GTTTAGGATA	AAGCTGAAGA	AAATTAAAAT	AAGT----CC	ACAAGAGCCA	AAATATGACC	993
BovB_Ta-RTE-Tachyglossus_acule	AAT-GTGTAA	ACTCAGGCTA	AAACTTAGAA	AGATCAATCA	TGCC----TT	ACAGCTGCCC	AAATATGACC	1291
BovB_Plat-RTE-Ornithorhynchus	AAT-GTGTAA	ACTCAGGCTA	AAACTTAGAA	AGATCAATCA	TGCC----TT	ACAGCTGCCC	AAATATGACC	1280
BovB_Ec-RTE-Equus_caballus	AAT-ATCAAA	TATCAGAGTA	AAGCTGAAGA	AGAGCACTAA	AAGA----AT	CGTAGTGCCA	AAATATAACG	1050
BovB_Cg-RTE-Christinus	----ATCAGC	TACCGGGGAA	GAGCAGAAGA	AAAACACCCA	AACA----GT	CAAAGTACAA	AAATATAATA	639
BovB_Bh-RTE-Bothriocroton_hydr	----------	----------	----------	----------	----------	----------	----------	-
BovB_Ml-RTE-Gehyra_variegata	----------	----------	----------	----------	----------	----------	----------	-
Sly-rnd1_family284-LINE/RTEBov	----------	----------	----------	----------	----------	----------	----------	-
Sly-rnd5_family2019-LINE/RTEBo	----------	----------	----------	----------	----------	----------	----------	-
Sly-rnd3_family322-LINE/RTEBov	----------	----------	----------	----------	----------	----------	----------	-
Sly-rnd5_family1698-LINE/RTEBo	----------	----------	----------	----------	----------	----------	----------	-
Sly-rnd1_family795-LINE/RTEBov	----------	----------	----------	----------	----------	----------	----------	-
Stu-rnd-5_family-3162_LINE_RTE	CTGGACACTA	TAGTGTAGCT	GCTGCTATAC	TGTCCCTTAG	AGTGTACTAC	TGCTGCAGCT	TGTTTTTAAG	769
Sly-rnd6_family1960-LINE/RTEBo	----------	----------	----------	----------	----------	----------	----------	-
Stu-rnd-5_family-2493_LINE_RTE	----------	----------	----------	----------	----------	----------	----------	-
Sly-rnd5_family447-LINE/RTEBov	----------	----------	----------	----------	----------	----------	----------	-
Stu-rnd-6_family-2093_LINE_RTE	----------	----------	----------	----------	----------	----------	----------	-
Consensus	----------	----------	----------	----------	----------	----------	----------	

Conservation

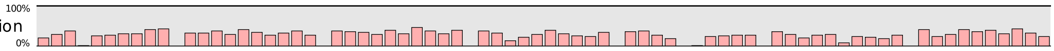

		1,980		2,000		2,020		
Stu-rnd-1_family-417_LINE_RTE-	----------	----------	----------	----------	----------	----------	----------	-
Sly-rnd1_family377-LINE/RTEBov	----------	----------	----------	----------	----------	----------	----------	-
Stu-rnd-1_family-489_LINE_RTE-	----------	----------	----------	----------	----------	----------	----------	-
Stu-rnd-1_family-607_LINE_RTE-	----------	----------	----------	----------	----------	----------	----------	-
Stu-rnd-1_family-645_LINE_RTE-	----------	----------	----------	----------	----------	----------	----------	-
Sly-rnd4_family81-LINE/RTEBovB	----------	----------	----------	----------	----------	----------	----------	-
Stu-rnd-6_family-408_LINE_RTE-	----------	----------	----------	----------	----------	----------	----------	-
Stu-rnd-1_family-351_LINE_RTE-	----------	----------	----------	----------	----------	----------	----------	-
Stu-rnd-1_family-382_LINE_RTE-	----------	----------	----------	----------	----------	----------	----------	-
Sly-rnd6_family746-LINE/RTEBov	----------	----------	----------	----------	----------	----------	----------	-
Stu-rnd-5_family-325_LINE_RTE-	----------	----------	----------	----------	----------	----------	----------	-
Sly-rnd4_family509-LINE/RTEBov	----------	----------	----------	----------	----------	----------	----------	-
Stu-rnd-1_family-513_LINE_RTE-	----------	----------	----------	----------	----------	----------	----------	-
Sly-rnd5_family1442-LINE/RTEBo	----------	----------	----------	----------	----------	----------	----------	-
BovB_Gl-RTE-Gehyra_lazelli	TAGATCACAT	TCCTTATGAA	TATACAGTGG	AGGT-----G	AAAAATAGGT	TTAAGGGACT	-AGATTTGAT	1362
BovB_Gv-RTE-Gehyra_variegata	TAGATCACAT	TCCTTATGAA	TATACAGTGG	AGGT-----G	AAAAATAGGT	TTAAGGGACT	-AGAGTTGAT	1287
BovB_PMo-RTE-Python_molurus	TAGATCATAT	TCCTTGTGAG	TACACAGTGA	AGGT-----G	ATGAATAGAT	TTAGAGGATT	-AGATTTGGT	1228
BovB_Oa-RTE-Ovis_aries	TAAATCAAAT	CCCTTATGAT	TATACAGTGG	AAGT-----G	ACAAATAGAT	TCAAGGGATT	-AGATCTGAT	1252
BovB-RTE-Bos_taurus	TAAATCAAAT	CCCTTATGAT	TATACAGTGG	AAGT-----G	AGAAATAGAT	TTAAGGGCCT	-AGATCTGAT	1850
BovB_Me-RTE-Macropus_eugenii	TATA--ATAT	CCCTTATGAA	TGTGAAGTGG	AAGT-----G	ATGAATAGAT	TTAAGGCTTT	-AGATATGGT	1104
BovB_Ano-RTE-Amphibolurus_norr	TAAACCAAAT	CCCTTATGAA	TACACAGTGG	AAGT-----G	AAGAACAGAT	TTAAGGAACT	-AGATTTGGT	1299
BovB_Gd-RTE-Eremiascincus_doug	TAAATCAGAT	CCCATATGAA	TATACAGTGG	AGGT-----G	ACCAATAGAT	TTAGGGGCTT	-AGATTTGGT	886
BovB_Er-RTE-Eremiascincus_rich	TAAATCAGAT	CCCATACGAA	TATACAGTGG	AGGT-----G	ACCAATAGAT	TTAGGGGCTT	-AGATTTGGT	1422
BovB_Ca-RTE-Ctenotus_atlas	TAAATCAGAT	CCCATACGAA	TATACAGTGG	AGGT-----G	ACTAATAGAT	TTAGGGGCTT	-AGATTTGGT	1415
BovB_Es-RTE-Egernia_stokesii	TAAATCATAT	TCCATATGAA	TACACAGTGG	AGGT-----G	ACAAATAGAT	TTAAAGGTTT	-AGATTTGGT	1248
BovB_Tr-RTE-Tiliqua_rugosa	TAAATCATAT	TCCACATGAA	TACACAGTGG	AGGT-----G	ACAAATAGAT	TTAAAAGTTT	-AGATTTGAG	1260
BovB_ALi-RTE-Amblyomma_limbatu	TAAATCATAT	CTCATATGAA	TATACAGTAA	AGGT-----G	ACAAATAGAT	TTAAGGAATT	-AGATCTGAT	1384
BovB_Hs-RTE-Hydrophis_spiralis	----------	----------	----------	----------	----------	----------	----------	-
BovB_VA-RTE-Vipera_ammodytes	TAAATCATAT	CCCTGTTGAA	TATACAGTAG	AGGT-----G	ACAAATAGAT	TTAAGGAATT	-AGATCTGAT	1220
BovB_ACo-RTE-Agkistrodon_conto	TAAATCATAT	CCCTGATGAA	TATACAGTAG	AGGT-----G	ACAAATAGAT	TTAAGGAATT	-AGATCTGAT	1220
BovB_Pg-RTE-Perameles_gunnii	----------	----------	----------	----------	----------	----------	----------	-
BovB_Io-RTE-Isoodon_obesulus	----------	----------	----------	----------	----------	----------	----------	-
BovB_Bp-RTE-Burramys_parvus	----------	----------	----------	----------	----------	----------	----------	-
BovB_Pb-RTE-Petaurus_breviceps	----------	----------	----------	----------	----------	----------	----------	-
BovB_Ma-RTE-Macropus_antilopin	CCTAAATAAC	ATCCCTTATG	AATATGAAGT	GGAA-----G	TGATGAATAG	ATTTAAGGGA	-TTAGATCTG	1342
BovB_Af-RTE-Antechinus_flavipe	TAGGGGAAAT	CATCAGATCA	TATAGGTATA	ATCT-----A	AACAACATCC	CTTATAATAT	-AAAGTGAAA	542
BovB_Ac-RTE-Anolis_carolinensi	TAACAAACAT	TCATAGTGAA	TATGCAGTGG	AGGT-----G	AAGAATAGAT	TTCAGGAACT	-AGATGTAAT	1185
BovB_BM-RTE-Bombyx_mori	CAAGCAAGAT	AGCTTC-CGC	AATGCTCTCG	AAA-------	---AACAGCT	TTTAGGTACA	ACACCACCAG	1045
BovB_Dr-RTE-Christinus	CCAGTAAGAT	CCCAACACAA	TATGCAGTCG	AGGT-----A	AATAATAGAT	TTGAGTCACT	-CGCTACAAC	1088
BovB_La-RTE-Loxodonta_africana	TTGAGTATAT	CCCACCTGAA	TTTAGAGACC	ATATGATGAC	AGCAAGAGAT	TTGACACATT	-GAACACTAA	1217
BovB_Pc-RTE-Procavia_capensis	TTGAGTATAT	CCCTCCTGAG	TTTAAAGACC	ATCT-----C	AAGAACAGAT	TTGATGTGTT	-GATCACTAA	1218
BovB_Et-RTE-Christinus	TTGAGTCTAT	CTCAGCTGAA	TTTTGAGAGC	ATCT-----T	AAGAGCAGAT	TTGATGCACT	-GAACAATAG	1057
BovB_Ta-RTE-Tachyglossus_acule	TGATGAATAT	TCCCCCTGTA	TTTAAGGATC	ATGT-----A	AAAAATAGAT	TTGAGGTGCT	-TAATCTCAC	1355
BovB_Plat-RTE-Ornithorhynchus	TGACGAATAT	TCCCCCTGTA	TTTAAGGATC	ATGT-----A	AAAAATAGAT	TTGAGGCGCT	-TAATCTCAC	1344
BovB_Ec-RTE-Equus_caballus	TAAACAACAT	TCCCAATGAA	TTTAAAGTCC	ACAT-----A	AAAAACAGAT	TTGCATTGTT	-AAACTTAAT	1114
BovB_Cg-RTE-Christinus	TAAACAACAT	TCTTAAAGAA	TTTGAAGAAC	ATAT-----A	AGGAACATAA	TAGCATTATT	-AAGTTTAAT	703
BovB_Bh-RTE-Bothriocroton_hydr	----------	----------	----------	----------	----------	----------	----------	-
BovB_Ml-RTE-Gehyra_variegata	----------	----------	----------	----------	----------	----------	----------	-
Sly-rnd1_family284-LINE/RTEBov	----------	----------	----------	----------	----------	----------	----------	-
Sly-rnd5_family2019-LINE/RTEBo	----------	----------	----------	----------	----------	----------	----------	-
Sly-rnd3_family322-LINE/RTEBov	----------	----------	----------	----------	----------	----------	----------	-
Sly-rnd5_family1698-LINE/RTEBo	----------	----------	----------	----------	----------	----------	----------	-
Sly-rnd1_family795-LINE/RTEBov	----------	----------	----------	----------	----------	----------	----------	-
Stu-rnd-5_family-3162_LINE_RTE	TGTTTGTCTG	TGTAGATCTT	TCATTAGTTT	CAGTATTTTC	TAGTGGATTT	GGTATCCGAT	GGTGGTACTA	839
Sly-rnd6_family1960-LINE/RTEBo	----------	----------	----------	----------	----TGATGT	AGTTTACGGT	CATG-TACTT	25
Stu-rnd-5_family-2493_LINE_RTE	----------	----------	----------	----------	----------	----------	----------	-
Sly-rnd5_family447-LINE/RTEBov	----------	----------	----------	----------	----------	----------	----------	-
Stu-rnd-6_family-2093_LINE_RTE	----------	----------	----------	----------	----------	----------	----------	-
Consensus	----------	----------	----------	----------	------A---	----------	----------	

Conservation

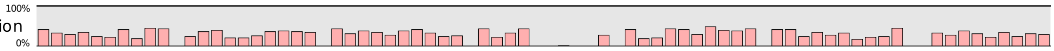

```
                                           2,040                            2,060                            2,080                            2,100
Stu-rnd-1_family-417_LINE_RTE-      ---------- ---------- ---------- ---------- ---------- ---------- ---------- -
Sly-rnd1_family377-LINE/RTEBov      ---------- ---------- ---------- ---------- ---------- ---------- ---------- -
Stu-rnd-1_family-489_LINE_RTE-      ---------- ---------- ---------- ---------- ---------- ---------- ---------- -
Stu-rnd-1_family-607_LINE_RTE-      ---------- ---------- ---------- ---------- ---------- ---------- ---------- -
Stu-rnd-1_family-645_LINE_RTE-      ---------- ---------- ---------- ---------- ---------- ---------- ---------- -
Sly-rnd4_family81-LINE/RTEBovB      ---------- ---------- ---------- ---------- ---------- ---------- ---------- -
Stu-rnd-6_family-408_LINE_RTE-      ---------- ---------- ---------- ---------- ---------- ---------- ---------- -
Stu-rnd-1_family-351_LINE_RTE-      ---------- ---------- ---------- ---------- ---------- ---------- ---------- -
Stu-rnd-1_family-382_LINE_RTE-      ---------- ---------- ---------- ---------- ---------- ---------- ---------- -
Sly-rnd6_family746-LINE/RTEBov      ---------- ---------- ---------- ---------- ---------- ---------- ---------- -
Stu-rnd-5_family-325_LINE_RTE-      ---------- ---------- ---------- ---------- ---------- ---------- ---------- -
Sly-rnd4_family509-LINE/RTEBov      ---------- ---------- ---------- ---------- ---------- ---------- ---------- -
Stu-rnd-1_family-513_LINE_RTE-      ---------- ---------- ---------- ---------- ---------- ---------- ---------- -
Sly-rnd5_family1442-LINE/RTEBo      ---------- ---------- ---------- ---------- ---------- ---------- ---------- -
BovB_Gl-RTE-Gehyra_lazelli          AGACAGAGCA C-CTGAAGAA CTATGGACGG AAGTTCGTGA CATTGTACAG GAGGCAGCAA TCAGCACCA- 1430
BovB_Gv-RTE-Gehyra_variegata        AGACAGAGCA C-CTGAAGAA CTATGGACGG AAGTTCGTGA CATTGTACAG GAGGCAGCAA TCAGCATCA- 1355
BovB_PMo-RTE-Python_molurus         AGACAGAGTG C-CTGAAGAA CTATGGACGG AGGTCCGTAA CATTGTACAG GAGGCAGCAA CTAAAACCA- 1296
BovB_Oa-RTE-Ovis_aries              AGACAGAGTG C-CTGAAGAA CTATGGACAG AGGTTCATAA CATTGTACAG GAGGCAGTGA TCAAAACCA- 1320
BovB-RTE-Bos_taurus                 AGATAGAGTG C-CTGATGAA CTATGGAATG AGGTTCGTGA CATTGTACAG GAGACAGGGA TCAAGACCA- 1918
BovB_Me-RTE-Macropus_eugenii        AGATAAAGTG C-CTGAAGAA CTATGGACAG AAGTTCTCAA TATTTTATAA GGAGCAGCAG A-AAAACCA- 1171
BovB_Ano-RTE-Amphibolurus_norr      GGACAGAGTG C-CTGAAGAA CTTTGGATAG AGGCTCGTAA CATTGTACAG GAGGCAGCAA CAAAAACCA- 1367
BovB_Gd-RTE-Eremiascincus_doug      AGACAGAGTG C-CTGAAGAA CTTTGGATGG AGGTCTGTAA CATTGTACAG GAGGCAGCAA CTAAAACCA- 954
BovB_Er-RTE-Eremiascincus_rich      AGACAGAGTG C-CTGAAGAA CTTTGGACGG AGGTCTGTAA CATTGTACAG GAGGCAGCAA CTAAAACCA- 1490
BovB_Ca-RTE-Ctenotus_atlas          AGACAGAGTG C-CTGAAGAA CTTTGGACGG AGGTCTGTAA CATAGTACAG GAAGCAGCAA CTAAAACCA- 1483
BovB_Es-RTE-Egernia_stokesii        AGACAGAGTA C-CTGAAGAA CTATGGATGG AGGCTTGCAA CATTGTTCAG GAGGTAGCAA CTAGAACTAG 1317
BovB_Tr-RTE-Tiliqua_rugosa          AGACAGAGTG C-CTGAAGAA CTGTGGACAG AGGCTCGCAA CTTTGTTCAG GAGGTGGCAA CTGGAACCA- 1328
BovB_ALi-RTE-Amblyomma_limbatu      AGACAGAGGG C-CTGAATAA CTATGAACGG AGGTTCGAAA CATTGTACAA GAGGCAGAAA CTAAAACTAT 1453
BovB_Hs-RTE-Hydrophis_spiralis      ---------- ---------- ---------- ---------- ---------- ---------- ---------- -
BovB_VA-RTE-Vipera_ammodytes        AGACAGAGTG C-CTGAAGAA CTATGGACAG AGGTTCGCAA CATTGTATAA GAGGTAGCAA CTAAAACCAT 1289
BovB_ACo-RTE-Agkistrodon_conto      AGACAGAGTG C-CTGAAGAA CTATGGACGG AGGTTCGCAA CATTGTACAA GAGGTAGCAA CTAAAACCAT 1289
BovB_Pg-RTE-Perameles_gunnii        ---------- ---------- ---------- ---------- ---------- ---------- ---------- -
BovB_Io-RTE-Isoodon_obesulus        ---------- ---------- ---------- ---------- ---------- ---------- ---------- -
BovB_Bp-RTE-Burramys_parvus         ---------- ---------- ---------- ---------- ---------- ---------- ---------- -
BovB_Pb-RTE-Petaurus_breviceps      ---------- ------CTAC TATCATCAAG AGGTATCTTA ATTCTTCACT TTCTGCCATC AAAGTGGTAT 54
BovB_Ma-RTE-Macropus_antilopin      GTAGAGAGAG T-GCCTGAAG AACTATGGAC AGAGGTTTCA CAATATTGCA CAGGAGACAG CAACAAAAAA 1411
BovB_Af-RTE-Antechinus_flavipe      GGTCAATGAA T-AGATTTAA GGGATTAGAT CTGGCAGGTA GAATATCTGG GAAATAAGGA CAGAGGTTTG 611
BovB_Ac-RTE-Anolis_carolinensi      AAATAGAATC C-CAGAAGAA CTATGGACAG AAGTCCACAA CATTGTTCAG GAGGCAGCAA CAAAGTACA- 1253
BovB_BM-RTE-Bombyx_mori             CCAGTGAGTC CGCCGAAGGG CTGTGGCAG- CGGCTGAAAT CATCTATTGC ATCGGCCGTC TCAGAAACA- 1113
BovB_Dr-RTE-Christinus              TGAAAGAGCT C-CAGATGAA CTATGGGAA- GAGATAAAAT TG---ATCAT AATGGATACA GCAGAAGGAT 1153
BovB_La-RTE-Loxodonta_africana      CGACCGAAGA C-CAGACAAG TTGTGGAAT- GACATCAAGG ACATCATACA TGAAGAAAGC CAAGAGACCA 1285
BovB_Pc-RTE-Procavia_capensis       TGACCGAAGA C-CAGATGAA TTGTGGAAT- GACATCAAAG ACATCATCCA TGAAGAGAGC -AAGAGGTCA 1285
BovB_Et-RTE-Christinus              TGACAGAAAG C-CTGATGAG CTGTGGGGGA GACATCAAGA CCATCATTCA AGAAGAAAGC TAA-AGGTCA 1125
BovB_Ta-RTE-Tachyglossus_acule      AGACAAGGAA C-CAGAGGAA ATGTGGACG- GAGATTAAAA ATGTCATCAA GGAAGAAAGT GACACAGTAT 1423
BovB_Plat-RTE-Ornithorhynchus       AGACAAGGAA C-CAGAGAAA ATGTGGACG- GAGATTAAAA ATGTCATCAA GGAAGAAAGC GACACGGTAT 1412
BovB_Ec-RTE-Equus_caballus          TGACCAAGAA C-CAGAAGAA CTATGGACT- GAAACCAGAA ATATTATTAA GGAAGAATGT GAAAAGACAA 1182
BovB_Cg-RTE-Christinus              TGAAAAAGAA C-TGGAAGAA CTATGGTTTT AAAACCAGAA AAATTATTAA AGAACAATGC ACTAAA--AA 770
BovB_Bh-RTE-Bothriocroton_hydr      ---------- ---------- ---------- ---------- ---------- ---------- ---------- -
BovB_Ml-RTE-Gehyra_variegata        ---------- ---------- ---------- ---------- ---------- ---------- ---------- -
Sly-rnd1_family284-LINE/RTEBov      ---------- ---------- ---------- ---------- ---------- ---------- ---------- -
Sly-rnd5_family2019-LINE/RTEBo      ---------- ---------- ---------- ---------- ---------- ---------- ---------- -
Sly-rnd3_family322-LINE/RTEBov      ---------- ---------- ---------- ---------- ---------- ---------- ---------- -
Sly-rnd5_family1698-LINE/RTEBo      ---------- ---------- ---------- ---------- ---------- ---------- ---------- -
Sly-rnd1_family795-LINE/RTEBov      ---------- ---------- ---------- ---------- ---------- ---------- ---------- -
Stu-rnd-5_family-3162_LINE_RTE      GGGTCATGTC NTCGGTTGAG GCCGAGGGCG AGGGGAAGTA GGGGTAAGTG GGTTACGGGT TCTCNTAAGN 909
Sly-rnd6_family1960-LINE/RTEBo      GATT--TGCA GCNCGNACGG GGCGGGGGCG AGGGTGGTAA GAGG-----G TACCAAGGGA GATCCTAGGT 88
Stu-rnd-5_family-2493_LINE_RTE      ---------- ---------- ---------- ---------- ---------- ---------- ---------- -
Sly-rnd5_family447-LINE/RTEBov      ---------- ---------- ---------- ---------- ---------- ---------- ---------- -
Stu-rnd-6_family-2093_LINE_RTE      ---------- ---------- ---------- ---------- ---------- ---------- ---------- -

Consensus                           ---------- --------A- ---------- ---------- ---------- ---------- ----------
```

Conservation

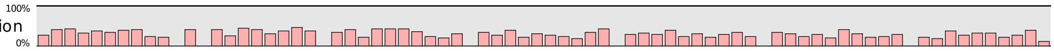

```
                                        2,120                   2,140                   2,160
                                          |                       |                       |
Stu-rnd-1_family-417_LINE_RTE-     ---------- ---------- ---------- ---------- ---------- ---------- ---------- -
Sly-rnd1_family377-LINE/RTEBov     ---------- ---------- ---------- ---------- ---------- ---------- ---------- -
Stu-rnd-1_family-489_LINE_RTE-     ---------- ---------- ---------- ---------- ---------- ---------- ---------- -
Stu-rnd-1_family-607_LINE_RTE-     ---------- ---------- ---------- ---------- ---------- ---------- ---------- -
Stu-rnd-1_family-645_LINE_RTE-     ---------- ---------- ---------- ---------- ---------- ---------- ---------- -
Sly-rnd4_family81-LINE/RTEBovB     ---------- ---------- ---------- ---------- ---------- ---------- ---------- -
Stu-rnd-6_family-408_LINE_RTE-     ---------- ---------- ---------- ---------- ---------- ---------- ---------- -
Stu-rnd-1_family-351_LINE_RTE-     ---------- ---------- ---------- ---------- ---------- ---------- ---------- -
Stu-rnd-1_family-382_LINE_RTE-     ---------- ---------- ---------- ---------- ---------- ---------- ---------- -
Sly-rnd6_family746-LINE/RTEBov     ---------- ---------- ---------- ---------- ---------- ---------- ---------- -
Stu-rnd-5_family-325_LINE_RTE-     ---------- ---------- ---------- ---------- ---------- ---------- ---------- -
Sly-rnd4_family509-LINE/RTEBov     ---------- ---------- ---------- ---------- ---------- ---------- ---------- -
Stu-rnd-1_family-513_LINE_RTE-     ---------- ---------- ---------- ---------- ---------- ---------- ---------- -
Sly-rnd5_family1442-LINE/RTEBo     ---------- ---------- ---------- ---------- ---------- ---------- ---------- -
BovB_Gl-RTE-Gehyra_lazelli         --TCCCAAAG AAACAGAAAC ACAAGAAAGC AAA-GTGGCT GTCTGAGGAG GACTTACAAA TAGCAGAGGA 1497
BovB_Gv-RTE-Gehyra_variegata       --TCCCAAAG AAACAGAAAC ACAAGAAAGC AAAAGTGGCT GTCTGAGGAG GCTTTACAAA TAGCAGAGGA 1423
BovB_PMo-RTE-Python_molurus        --TCCCAAAG AAAAAGAAAT GCAAGAAAGC AAA-ATGGCT GTCTGATGAG GCTTTACAAA TAGCTGTGGA 1363
BovB_Oa-RTE-Ovis_aries             --TCCCAAAG AAAAAGAAAT GCAAAAAGGC AAA-ATGGTT GTCTGAGGAG GCCTTACAAA TAGCTGAGAA 1387
BovB-RTE-Bos_taurus                --TCCCCATG GAAAAGAAAT GCAAAAAAGC AAA-ATGGCT GTCTGGGGAG GCCTTACAAA TAGCTGTGAA 1985
BovB_Me-RTE-Macropus_eugenii       --AACAAACC CCAAAAAGAT TCCAAAGAAA AAG-AAGAGC AAG-AATGAG GCCTTACAAA TAGCTGAGGA 1237
BovB_Ano-RTE-Amphibolurus_norr     --TCCCAAAG AAAAGGAAAG GCAAGAAAGC AAA-GTGGCT GTCCAATGAG GCCTTACAAA TAGCAGAGAA 1434
BovB_Gd-RTE-Eremiascincus_doug     --TCCCAAAG AGAAAGAAAT GCAAGAAGGC AAA-GTGGCT GTCTGACGAG GCTTTACAAA TAGCTGAGGA 1021
BovB_Er-RTE-Eremiascincus_rich     --TCCCAAAG AGAAAGAAAA GCAAGAAGGC AAA-GTGGCT GTCTGATGAG GCTTTACAAA TAGCTGAGGA 1557
BovB_Ca-RTE-Ctenotus_atlas         --TCCCCAAG AGAAAGAAAT GCAAGAAGGC AAA-GTGGCT ATCTGAGGAG GCTTTACAAA TAGCTGAGGA 1550
BovB_Es-RTE-Egernia_stokesii       AATCCCTAAG GAAAAGAAAT GCAAGAAGGC AAA-ATGGCT GTCTGAAGAA GCTGTAGGAA TTGCTGAGGA 1386
BovB_Tr-RTE-Tiliqua_rugosa         --TCCCTAAG GAGAAGAAGT GCAAGAAGGC AAA-ATGGCT GTCTGAAGAA GCTTTACAAA TTGCTGAGGA 1395
BovB_ALi-RTE-Amblyomma_limbatu     --ACCAAAAC AAAAAAAAAT GCAAAAAAGC AAA-ATGGCT GTCTGAGAAA GCTTTACAAA TAGCTGAGGA 1520
BovB_Hs-RTE-Hydrophis_spiralis     ---------- ---------- ---------- ---------- ---------- ---------- ---------- -
BovB_VA-RTE-Vipera_ammodytes       --CCCAAAG- AAAAAGAAAT GCAAGAAAGC AAA-ATGGCT GTCTGAGGAA GCTTTGCAAA TAGCTGACGA 1355
BovB_ACo-RTE-Agkistrodon_conto     --CCCAAAG- AAAAAGAAAT GCAAGAAAGC AAA-ATGGCT GTCTGAGGAA GCTTTGCAAA TAGCTGAGGA 1355
BovB_Pg-RTE-Perameles_gunnii       ---------- ---------- ---------- ---------- ---------- ---------- ---------- -
BovB_Io-RTE-Isoodon_obesulus       ---------- ---------- ---------- ---------- ---------- ---------- ---------- -
BovB_Bp-RTE-Burramys_parvus        ---------- ----AGAAGA GCAAGAAAGA AAATGATGAT TTGTTTTA-- GCTTTGCAAA TAGTGGAGGA 54
BovB_Pb-RTE-Petaurus_breviceps     CATCTCCAAA TCTGAGATTA CTGATACTTT TCCTGGCAAC CTTAATTACT GGGTTTAAAC TGAGGAAAGA 124
BovB_Ma-RTE-Macropus_antilopin     CATTCCAAAA AAAAAGAAAA GCAAGAAAGC AAAATGGCTG TCTGAAGAG- GCTTTACAAA TAGCTGAAGA 1480
BovB_Af-RTE-Antechinus_flavipe     CAATATTGTA CAGGAGGCAA CAAACAAAAA AACATTTCAA AGAAAAAAAA AGCAAGAAAG CAATAGCTGA 681
BovB_Ac-RTE-Anolis_carolinensi     --TCCCAAAG AAAAAGAAAA CCAAGAAGGC AAG-ATGGTT GTCTGCTGAG ACACTGGAAG TAGCCCAAGA 1320
BovB_BM-RTE-Bombyx_mori            GTGCCTAAAC AAGCAAAACA --AAAAAGAT CTATGTGGAT TTCAAACGAT ACATGGGAAT TGATTGAACG 1181
BovB_Dr-RTE-Christinus             GCATCCCGTA TAGGAAATCA GCAAAAAGAC CACCATGGCT CTCAGACTCA TCGATTGAAA TAGCCAATAA 1223
BovB_La-RTE-Loxodonta_africana     TTAAAAAGAC AGGAAAGAAA --GAAAAGAC CAAAATGGAT GTCAGAAGAG ACTCTGAAAC TTGCTCTTGA 1353
BovB_Pc-RTE-Procavia_capensis      TTAAAAAGAC AGGAAAGAAA --GAAAAGAC CAAATTGGAT GTCAGAAGAG ACTCTGAAAC TTGCTCTTGA 1353
BovB_Et-RTE-Christinus             TTAAAA-GAC AGGAAAGAAA --GAAAAGGT CAAAGCAGAT GTCAGAAAAG ACTTGGAG-- TTGCTCTTAA 1190
BovB_Ta-RTE-Tachyglossus_acule     TGCAAAAGGT CAGGAAGCA- --GAAAAAGC CAAAATGGAT GTCAAACATA ACTCTGGAAG TAGCCAAAAA 1490
BovB_Plat-RTE-Ornithorhynchus      TGCAAAAGGT CAGGAAGAA- --GAAAAAGC CAAAATGGAT GTCGAACATA ACTCTGGAAG TAGCCAGAAA 1479
BovB_Ec-RTE-Equus_caballus         TGCCTGAAGT AAAAAGAAAA ---GAAAAAT CAAGATGGAT GACAGAAGAA ACACTAAAAA TTGTTAAGGA 1249
BovB_Cg-RTE-Christinus             TTCCTGTAGT AAAAATAAAA ---GAAAAAA CAAGATGGAT GTCTGATGAA ACTATTAAAA TTGTTAAAGA 837
BovB_Bh-RTE-Bothriocroton_hydr     ---------- ---------- ---------- ---------- ---------- ---------- ---------- -
BovB_Ml-RTE-Gehyra_variegata       ---------- ---------- ---------- ---------- ---------- ---------- ---------- -
Sly-rnd1_family284-LINE/RTEBov     ---------- ---------- ---------- ---------- ---------- ---------- ---------- -
Sly-rnd5_family2019-LINE/RTEBo     ---------- ---------- ---------- ---------- ---------- ---------- ---------- -
Sly-rnd3_family322-LINE/RTEBov     ---------- ---------- ---------- ---------- ---------- ---------- ---------- -
Sly-rnd5_family1698-LINE/RTEBo     ---------- ---------- ---------- ---------- ---------- ---------- ---------- -
Sly-rnd1_family795-LINE/RTEBov     ---------- ---------- ---------- ---------- ---------- ---------- ---------- -
Stu-rnd-5_family-3162_LINE_RTE     TGAGGGTAGG GTCNTGGAAC ATAGGTTCGT TGACGGGGAA GTCCATAGAG CTAGTGAAGA TTCTCAAGAA 979
Sly-rnd6_family1960-LINE/RTEBo     TGAGAGTTGG GTCTTGAAAC ATAGGGNCGT TGACGAGGAA GTCCATAGAG TTAGTGAAGA TTCTTATGAA 158
Stu-rnd-5_family-2493_LINE_RTE     ---------- ---------- ---------- ---------- ---------- ---------- ---------- -
Sly-rnd5_family447-LINE/RTEBov     ---------- ---------- ---------- ---------- ---------- ---------- ---------- -
Stu-rnd-6_family-2093_LINE_RTE     ---------- ---------- ---------- ---------- ---------- ---------- ---------- -

Consensus                          ---------- ----A----- ---A-AA--- -A----GG-- -T-------- ----T--AA- T--------A
```

Conservation

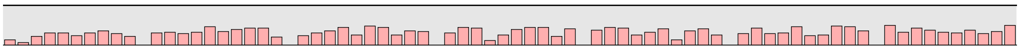

```
                                    2,180                          2,200                          2,220                          2,240
Stu-rnd-1_family-417_LINE_RTE-      ---------- ---------- ---------- ---------- ---------- ---------- ---------- -
Sly-rnd1_family377-LINE/RTEBov      ---------- ---------- ---------- ---------- ---------- ---------- ---------- -
Stu-rnd-1_family-489_LINE_RTE-      ---------- ---------- ---------- ---------- ---------- ---------- ---------- -
Stu-rnd-1_family-607_LINE_RTE-      ---------- ---------- ---------- ---------- ---------- ---------- ---------- -
Stu-rnd-1_family-645_LINE_RTE-      ---------- ---------- ---------- ---------- ---------- ---------- ---------- -
Sly-rnd4_family81-LINE/RTEBovB      ---------- ---------- ---------- ---------- ---------- ---------- ---------- -
Stu-rnd-6_family-408_LINE_RTE-      ---------- ---------- ---------- ---------- ---------- ---------- ---------- -
Stu-rnd-1_family-351_LINE_RTE-      ---------- ---------- ---------- ---------- ---------- ---------- ---------- -
Stu-rnd-1_family-382_LINE_RTE-      ---------- ---------- ---------- ---------- ---------- ---------- ---------- -
Sly-rnd6_family746-LINE/RTEBov      ---------- ---------- ---------- ---------- ---------- ---------- ---------- -
Stu-rnd-5_family-325_LINE_RTE-      ---------- ---------- ---------- ---------- ---------- ---------- ---------- -
Sly-rnd4_family509-LINE/RTEBov      ---------- ---------- ---------- ---------- ---------- ---------- ---------- -
Stu-rnd-1_family-513_LINE_RTE-      ---------- ---------- ---------- ---------- ---------- ---------- ---------- -
Sly-rnd5_family1442-LINE/RTEBo      ---------- ---------- ---------- ---------- ---------- ---------- ---------- -
BovB_Gl-RTE-Gehyra_lazelli          AAGAAAAAAA GCAAAA--GG CAAAGGTGAA AAGGAAAGAT TTAC--ACAA CTGAATG-CA GATTCCCAGA 1562
BovB_Gv-RTE-Gehyra_variegata        AAGAAGGAAA GCAAAAA-GG CAAAGGTGAA AAGGAAAGAT TTAC--CCAA CTGAATG-CA GATTTCCAGA 1489
BovB_PMo-RTE-Python_molurus         AAGAAGGAAT GCGAAA--AG CAAGGGTGAA AGGGAAAGAT ATAA--GCAA CTGAATG-CA GATTTCCAGA 1428
BovB_Oa-RTE-Ovis_aries              AAGAAGAGAA GCGAAA--GG CAAAGGAGAA AAGGAAAGAT ATAC--CCAT CTGAATG-CA GAGTTCCAAA 1452
BovB-RTE-Bos_taurus                 AAGAAGAGAA GCGAAA--AG CAAAGGAGAA AAGGAAAGAT ATAA--ACAT CTGAATG-CA GAGTTCCAAA 2050
BovB_Me-RTE-Macropus_eugenii        AAGAAGGAAA GGGAAA--GG GAAAGGAGAA AGGGAACAAT ATAC--CTAA CTGAATG-CA GAATTCCAGA 1302
BovB_Ano-RTE-Amphibolurus_norr      GAGAAGGGAA GCAAAA--TG CAAGGGAGAT AGGGAAAGTT ACAG--AAAA TTGAATG-CA GACTTCCAAA 1499
BovB_Gd-RTE-Eremiascincus_doug      AAGAAGGGAA GCCAAA--AG CAAGGGAGAA AGGGAAAGAT ATAG--CCAA CTGAATG-CA GATTTCCAGA 1086
BovB_Er-RTE-Eremiascincus_rich      AAGAAGGGAA GCCAAA--AG CAAGGGAGAA AGGGAAAGAT ATAG--CCAA CTGAATG-CA GATTTCCAGA 1622
BovB_Ca-RTE-Ctenotus_atlas          AAGAAGGGAA GCCAAA--AG CAAGGGAGAA AGGGAAAGAT ATAG--CCAA CTGAATG-CA GATTTCCAGA 1615
BovB_Es-RTE-Egernia_stokesii        AAGAAGGAAA GCAAAA--GG CAAGGGAGAA AGGGAAAGAT ATATACCCAA CTGAATG-CA GATTTCCAGA 1453
BovB_Tr-RTE-Tiliqua_rugosa          AAGAAGGAAA GCAAAA--GG CAAGGGAGAA AGGGAAAGAT ATAT--CCAA CTGAATG-CA GATTTCCAGA 1460
BovB_ALi-RTE-Amblyomma_limbatu      AAAAAAGTAA GCAAAAA-GG CAAGGAAGAA AAAGAAAGAT ACTAAACAAT TAGAATGACA GAATTCCACA 1589
BovB_Hs-RTE-Hydrophis_spiralis      ---------- ---------- ---------- ---------- ---------- ---------- ---------- -
BovB_VA-RTE-Vipera_ammodytes        AAGGAGGGAA GCGAAA--GG CAAGGGAGAA AAAGAAATTT AC--GCCCAA TTGAATG-CA GAATTCCAGA 1420
BovB_ACo-RTE-Agkistrodon_conto      AAGAAGGGAA GCGAAA--GG CAAAGGAGAA AGAGAAAGAT AC--ACCCAA TTGAATG-CA GAATTCCAGA 1420
BovB_Pg-RTE-Perameles_gunnii        ---------- ---------- ---------- ---------- ---------- ---------- ---------- -
BovB_Io-RTE-Isoodon_obesulus        ---------- ---------- -----GAAAA TATATACCCA ATTGAATTAC AGAATTC-CA GAAAATAGAC 44
BovB_Bp-RTE-Burramys_parvus         AAGAAGTAAC ATGAAAGGGA AAGGAGAAAG GGAAAAATAT GCCAGACCAA CTGAATG-CA AAATTCCAAA 123
BovB_Pb-RTE-Petaurus_breviceps      AGGAAAAGAC A-AAAGGGAA AAAAATAAAA GGAAAGATAT AC----CCAA GTGAATG-CA GAATTCTAGA 188
BovB_Ma-RTE-Macropus_antilopin      AACAAGGAAA AGAAAAGGTA AAAGAGAAAA GGAAAGATAA AC----CCAA CTGAATG-CA GAATTCCAGA 1545
BovB_Af-RTE-Antechinus_flavipe      AGTAACAATA GCTGAAGAAA AAAAGGAGAA AGGGAAAGTT AT--ATCTAA CTGAATG-CA GAATTCTAAA 748
BovB_Ac-RTE-Anolis_carolinensi      AAGAAGAAAG ACGAAAGGAA ACAGCAATGG GGAGGGGGAG ATATGCACAA TTAAATG-CA CAATACCAGA 1389
BovB_BM-RTE-Bombyx_mori             CAGAAAGAAC TTGCAAG-GA T-CAGATAAA ATTGAAAACT ACAA--TGCA CTCAGTA-AA GACATAAAAC 1246
BovB_Dr-RTE-Christinus              GAGACGGAAC AGCCAAA-GC CACTGGCAAG AGTTTTGAAT TCAG--ATCT CTAAATG-CA GAATTTCAAC 1289
BovB_La-RTE-Loxodonta_africana      ACG-TCGAGC AGCTAAA-GC AAAAGGAAGA AATGATGAAG TAAA--AGAA CTGAACA-GA AGATTTCAAA 1418
BovB_Pc-RTE-Procavia_capensis       ATG-TCGAGC AGCTAAA-GC AAAAGGAAGG AATGGCGAAG TAAA--AGAC CTAAACA-GA AGATTTCAAA 1418
BovB_Et-RTE-Christinus              TTG-TAGAGT TGCTAAA-GC AAATGGAAGA AACAATGAAG TCAA--GGAG CTGAACA-GA ACATTTCAAA 1255
BovB_Ta-RTE-Tachyglossus_acule      AAG-GAAGGA AGCAAAA-AT CAGAGGACAA AAGAACCTGG TTAA--AGAA CTGAGCC-GA GAATTCCAGC 1555
BovB_Plat-RTE-Ornithorhynchus       AAG-GAAGGA AGCAAAA-AT CAGAGGACAA AAGAACCTGG TTAA--AGAA CTGAGCC-GA GAATTCCAGC 1544
BovB_Ec-RTE-Equus_caballus          CAGGTAAGAA GCAAAAG-TA AAA-GGTGAC AAAAGCAGGG TTAG--AATC CTGAATG-CA GTTTTTCAGC 1314
BovB_Cg-RTE-Christinus              TAGACAAGAA GCAAAAG-TA AAAAGGTGAC AAAAACAGG- ------AAAT CTAAATG-AA GTATTTCAGC 898
BovB_Bh-RTE-Bothriocroton_hydr      ---------- ---------- ---------- ---------- ---------- ---------- ---------- -
BovB_Ml-RTE-Gehyra_variegata        ---------- ---------- ---------- ---------- ---------- ---------- ---------- -
Sly-rnd1_family284-LINE/RTEBov      ---------- ---------- ---------- ---------- ---------- ---------- ---------- -
Sly-rnd5_family2019-LINE/RTEBo      ---------- ---------- ---------- ---------- ---------- ---------- ---------- -
Sly-rnd3_family322-LINE/RTEBov      ---------- ---------- ---------- ---------- ---------- ---------- ---------- -
Sly-rnd5_family1698-LINE/RTEBo      ---------- ---------- ---------- ---------- ---------- ---------- ---------- -
Sly-rnd1_family795-LINE/RTEBov      ---------- ---------- ---------- ---------- ---------- ---------- ---------- -
Stu-rnd-5_family-3162_LINE_RTE      GAGGAAGATA AATATAGCTT GTGTCCAGGA GACCAGATGG GTAGGTACCA AGGCTCGGGA TGTNGA-CGG 1048
Sly-rnd6_family1960-LINE/RTEBo      GAGGAAGATC AATATAGCTT GTGTCCAGGA AACCAAATGG GTAGGAACCA AGGCCAGGGA TGTGGAATGG 228
Stu-rnd-5_family-2493_LINE_RTE      ---------- ---------- ---------- ---------- ---------- ---------- ---------- -
Sly-rnd5_family447-LINE/RTEBov      ---------- ---------- ---------- ---------- ---------- ---------- ---------- -
Stu-rnd-6_family-2093_LINE_RTE      ---------- ---------- ---------- ---------- ---------- ---------- ---------- -
Consensus                           --G------- ----AA---- -----G--A- A---A----- --------A- -TGAA----A ---TT--A--
```

Conservation

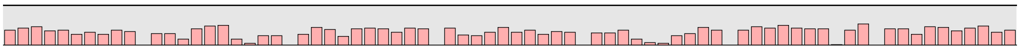

	2,260		2,280		2,300			
Stu-rnd-1_family-417_LINE_RTE-	----------	----------	----------	----------	----------	----------	----------	-
Sly-rnd1_family377-LINE/RTEBov	----------	----------	----------	----------	----------	----------	----------	-
Stu-rnd-1_family-489_LINE_RTE-	----------	----------	----------	----------	----------	----------	----------	-
Stu-rnd-1_family-607_LINE_RTE-	----------	----------	----------	----------	----------	----------	----------	-
Stu-rnd-1_family-645_LINE_RTE-	----------	----------	----------	----------	----------	----------	----------	-
Sly-rnd4_family81-LINE/RTEBovB	----------	----------	----------	----------	----------	----------	----------	-
Stu-rnd-6_family-408_LINE_RTE-	----------	----------	----------	----------	----------	----------	----------	-
Stu-rnd-1_family-351_LINE_RTE-	----------	----------	----------	----------	----------	----------	----------	-
Stu-rnd-1_family-382_LINE_RTE-	----------	----------	----------	----------	----------	----------	----------	-
Sly-rnd6_family746-LINE/RTEBov	----------	----------	----------	----------	----------	----------	----------	-
Stu-rnd-5_family-325_LINE_RTE-	----------	----------	----------	----------	----------	----------	----------	-
Sly-rnd4_family509-LINE/RTEBov	----------	----------	----------	----------	----------	----------	----------	-
Stu-rnd-1_family-513_LINE_RTE-	----------	----------	----------	----------	----------	----------	----------	-
Sly-rnd5_family1442-LINE/RTEBo	----------	----------	----------	----------	----------	----------	----------	-
BovB_Gl-RTE-Gehyra_lazelli	GAACAGCAAG	AAGAGATAAG	GAGGCATT--	CATGAACGAA	CAGTG---CA	AAGTAACAGA	GGAAAACAAT	1627
BovB_Gv-RTE-Gehyra_variegata	GAACAGCAAG	AAGAGATAAG	GAGGCATT--	CATGAACGAA	GAGTG---CA	AAGTAATAGA	GGAAAACAAT	1554
BovB_PMo-RTE-Python_molurus	GAATAGCAAG	GAGAGATAAG	AAGGCCTT--	CTTAAATGAA	CAATG---CA	AAGAAATCGA	GGAAAACAAT	1493
BovB_Oa-RTE-Ovis_aries	GAATAGCAAG	GAGAGATAAG	AAAGCCTT--	CCTCAGTGAA	CAATG---CA	AAGAAATAGA	GGAAAACAAT	1517
BovB-RTE-Bos_taurus	GAATAGCAAG	AAGAGATAAG	AAAGCCTT--	CTTCAGCGAT	CAATG---CA	AAGAAATAGA	GGAAAACAAC	2115
BovB_Me-RTE-Macropus_eugenii	GAATAGCAGG	AAGAGATAAG	AACATTTT--	CTTAAATAAA	CAATG---CG	ATGAAATAGA	AGAAAACAAT	1367
BovB_Ano-RTE-Amphibolurus_norr	GAATAGCAAG	GAGAGACAAG	AGGGCCTT--	CTTAAATGAA	CAATG---CA	AAGAAATAGA	GGAAAATAAC	1564
BovB_Gd-RTE-Eremiascincus_doug	GAACAGCAAG	GAGAGATAGG	AAGGCCTT--	CCTAAATGAG	CAATG---CA	AAGAAATAGA	GGAAAACAAT	1151
BovB_Er-RTE-Eremiascincus_rich	GAACAGCAAG	GAGAGATAGG	AAGGCCTT--	CCTAAATGAG	CAATG---CA	AAGAAATAGA	GGAAAACAAT	1687
BovB_Ca-RTE-Ctenotus_atlas	GAACAGCAAG	GAGAGATAGG	AAGGCTTT--	CCTAAATGAG	CAATG---CA	AAGAAATAGA	GGAAAACAAT	1680
BovB_Es-RTE-Egernia_stokesii	GAATAGCAGG	GAGAGATAGG	AATGCCTT--	CTTAAATGAG	CAATG---CA	GAGAAATAGA	GGCAAACAAT	1518
BovB_Tr-RTE-Tiliqua_rugosa	GAATAGCAAG	GAGAGATAGG	AATGCCTT--	CTTAAATGAG	CAATG---CA	GAGAAATAGA	GGAAAACAAT	1525
BovB_ALi-RTE-Amblyomma_limbatu	GAACAGCTAG	AAGAGATAAG	AATGTATTTA	CTTAAATGAA	CAGTAA--CA	AAAGAAATAG	AAGAAAATAT	1657
BovB_Hs-RTE-Hydrophis_spiralis	----------	----------	----------	----------	----------	----------	----------	-
BovB_VA-RTE-Vipera_ammodytes	CAATAGCTAG	AAGAGATAAG	AATGCCTT--	CTTAAATGAA	CAGTG---CA	AAGAAATAGA	AGAAAACAAT	1485
BovB_ACo-RTE-Agkistrodon_conto	GAATAGCTAG	AAGAGATAAG	AATGCCCT--	CTTAAATGAA	CAGTG---CA	AAGAAATAGA	AGAAAACAAT	1485
BovB_Pg-RTE-Perameles_gunnii	----------	----------	----------	----------	----------	----------	----------	-
BovB_Io-RTE-Isoodon_obesulus	AAGGAGAAGA	TTAAAAAAAA	AAAGATTT--	CTTAAATGAG	CAGTAGAGAA	AAA-AATAGA	AGAAAACAAT	111
BovB_Bp-RTE-Burramys_parvus	GAATAGAAAG	GAGAGATAAA	GAGGTTTT--	CTTAAATGAG	CAATGC--AA	AGA-AATAGA	AGAAAACAAG	188
BovB_Pb-RTE-Petaurus_breviceps	GAATAGCAAG	GAAAGATAA-	--GTTTTT--	CTTAAATAAG	CAATGC--AA	AGA-AATAGG	AGAAAATAA-	249
BovB_Ma-RTE-Macropus_antilopin	GAATAGCAAG	GAAAGATAAG	AAAGTTTT--	CTTAAATGAG	CAATGC--AA	AGA-AATAGA	AGAAAACAA-	1609
BovB_Af-RTE-Antechinus_flavipe	TAATAACAAA	AAGAAATAA-	--GGTTAT--	CCTACATGAG	CAATGCAAAG	AAATAGGAGG	GAAAAACCAA	813
BovB_Ac-RTE-Anolis_carolinensi	AGTTAGCCAG	AAGAGACAAG	GAACTATT--	TTTAAACAAG	CAATG---CA	TGGAACTGGA	AGAAGACAAC	1454
BovB_BM-RTE-Bombyx_mori	AACGCTGCCG	ACGTGACAAG	AATATTTT--	CATTGAAAAT	ATTTA---TA	AAGGAATTGA	GTCGCACTCC	1311
BovB_Dr-RTE-Christinus	GGGAAGCAAG	GCGGGACAAG	GAGCGCTA--	CTGGGATGAG	CGGTG---TT	CAAAACTGGA	AGCTGCTAGC	1354
BovB_La-RTE-Loxodonta_africana	GGGCAGCTCG	AGAAGACAAA	GTAAAGTA--	TTATAATGAC	ATGTA---CA	AAGACCTGGA	GATAGAAAAC	1483
BovB_Pc-RTE-Procavia_capensis	GAGCAGCTCG	AGAAGACAAA	AGAAAGTA--	TTATAATGAC	ATGTG---CA	AAGAGCTGGA	AATGGAAAAC	1483
BovB_Et-RTE-Christinus	----------	---AGGCAAA	GCCAAATA--	TTATAACGAA	ATGTT---CA	CAGACCTAGA	ATTAGA----	1303
BovB_Ta-RTE-Tachyglossus_acule	GCTCTGCCAG	GAAGGACAAA	CAGGAATA--	TTATAACAGC	ATATG---TA	AAGAAATGGA	AGTCAATAAC	1620
BovB_Plat-RTE-Ornithorhynchus	GCTCTGCCAG	GAAGGACAAA	CAGGAATA--	TTATAACAGC	ATATG---TA	AAGAAATGGA	AGTCAATAAC	1609
BovB_Ec-RTE-Equus_caballus	GACTGTCACG	TAGAGATAAA	GAGAACTA--	CTATAATGGC	CAATG---CA	AAGAGATAGA	AGAAAACAAC	1379
BovB_Cg-RTE-Christinus	CACTGGCATG	TAGAGACAAA	GAGAAATA--	ATATAAAAAC	CATTG---AA	AAGAAATAGA	AGAGAACAAC	963
BovB_Bh-RTE-Bothriocroton_hydr	----------	----------	----------	----------	----------	----------	----------	-
BovB_Ml-RTE-Gehyra_variegata	----------	----------	----------	CCAAGATGAT	TGATA-----	------AAAA	TTTAACTGTT	29
Sly-rnd1_family284-LINE/RTEBov	----------	----------	----------	----------	----------	----------	----------	-
Sly-rnd5_family2019-LINE/RTEBo	----------	----------	----------	----------	----------	----------	----------	-
Sly-rnd3_family322-LINE/RTEBov	----------	----------	----------	----------	----------	----------	----------	-
Sly-rnd5_family1698-LINE/RTEBo	----------	----------	----------	----------	----------	----------	----------	-
Sly-rnd1_family795-LINE/RTEBov	----------	----------	----------	----------	----------	----------	----------	-
Stu-rnd-5_family-3162_LINE_RTE	GTTTAAGTTG	TGGTACTCAG	GAGGNTCGAG	GGATAGAAAT	GGAGTGGGTA	TTNTAGTAGA	CGGGGATTTG	1118
Sly-rnd6_family1960-LINE/RTEBo	ATTTAAGTTG	TGGTACTCAG	GAGGATCGAG	GAATAGGAAT	GGAGTAGGCA	TTTTAGTAGA	CGGGGAACTT	298
Stu-rnd-5_family-2493_LINE_RTE	----------	----------	----------	----------	----------	----------	----------	-
Sly-rnd5_family447-LINE/RTEBov	----------	----------	----------	----------	----------	----------	----------	-
Stu-rnd-6_family-2093_LINE_RTE	----------	----------	----------	----------	----------	----------	----------	-
Consensus	---------G	---AGA-AA-	------T---	----AA--A-	---TG----A	----A-T-GA	-G-AAA-AA-	

Conservation

100%

0%

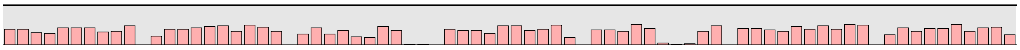

Sequence	2,320		2,340		2,360		2,380	
Stu-rnd-1_family-417_LINE_RTE-	----------	----------	----------	----------	----------	----------	----------	-
Sly-rnd1_family377-LINE/RTEBov	----------	----------	----------	----------	----------	----------	----------	-
Stu-rnd-1_family-489_LINE_RTE-	----------	----------	----------	----------	----------	----------	----------	-
Stu-rnd-1_family-607_LINE_RTE-	----------	----------	----------	----------	----------	----------	----------	-
Stu-rnd-1_family-645_LINE_RTE-	----------	----------	----------	----------	----------	----------	----------	-
Sly-rnd4_family81-LINE/RTEBovB	----------	----------	----------	----------	----------	----------	----------	-
Stu-rnd-6_family-408_LINE_RTE-	----------	----------	----------	----------	----------	----------	----------	-
Stu-rnd-1_family-351_LINE_RTE-	----------	----------	----------	----------	----------	----------	----------	-
Stu-rnd-1_family-382_LINE_RTE-	----------	----------	----------	----------	----------	----------	----------	-
Sly-rnd6_family746-LINE/RTEBov	----------	----------	----------	----------	----------	----------	----------	-
Stu-rnd-5_family-325_LINE_RTE-	----------	----------	----------	----------	----------	----------	----------	-
Sly-rnd4_family509-LINE/RTEBov	----------	----------	----------	----------	----------	----------	----------	-
Stu-rnd-1_family-513_LINE_RTE-	----------	----------	----------	----------	----------	----------	----------	-
Sly-rnd5_family1442-LINE/RTEBo	----------	----------	----------	----------	----------	----------	----------	-
BovB_Gl-RTE-Gehyra_lazelli	AGAA-TGGGA	AGGACTAGAG	AC-CTCTTCA	AGAAAAATCG	GAGAAATCAA	GGGAACGTTC	CAGGGCAA-G	1694
BovB_Gv-RTE-Gehyra_variegata	AGAAATGGGA	AGGACTAGAG	AC-CTTTTCA	AGAAAA-TCG	GAGAAATCAA	GGGAACGTTC	CAGG-CAA-G	1620
BovB_PMo-RTE-Python_molurus	AGAA-TGGGG	AAGACTAGAG	AT-CTCTTCA	AGAAAA-TTG	GAGATATCAA	AGGAACGTTT	-AGTGCAA-A	1558
BovB_Oa-RTE-Ovis_aries	AGAA-TGGGA	AAGACTAGAG	AT-CTCTTCA	AGAAAATTAG	A-GATACCAA	GGGAACATTT	CATGCAAA-G	1583
BovB-RTE-Bos_taurus	AGAA-TGGGA	AAGACTAGAG	AT-CTCTTCA	AGAAAATCAG	A-GATACCAA	GGGAACATTT	CATGCAAA-G	2181
BovB_Me-RTE-Macropus_eugenii	AGAA-TGGGA	AAGATAAGAG	AT-CTCTTCA	AGAAAATTAG	A-GATA---A	AGATATTTTT	CATGCAAA-A	1430
BovB_Ano-RTE-Amphibolurus_norr	AGAA-AAGGA	AAAACCAGAG	AT-CTGTACA	GGAAAATTGG	A-GATATTAA	AGGAACATTT	TGCGCAAA-G	1630
BovB_Gd-RTE-Eremiascincus_doug	AGAA-TGGGA	AAGACTAGAG	AT-CTTTTCA	AGAAAAATAG	AAGATGTCAA	AGGACAATTT	CATGCTAA-G	1218
BovB_Er-RTE-Eremiascincus_rich	AGAA-TGGGA	AAGACTAGAG	AT-CTTTTCA	AGAAAATTGG	A-GATGTCAA	AGGACAATTT	CATGCTAA-G	1753
BovB_Ca-RTE-Ctenotus_atlas	AGAA-TGGGA	AAGACTAGAG	AT-CTTTTCA	AGAAAATTGG	A-GATGTCAA	AGGACAATTT	CGTGCTAG-G	1746
BovB_Es-RTE-Egernia_stokesii	AGAA-GGGGG	AAGACTAGAG	AT-CTCTTCA	AGAAAATTGG	A-GATATCAC	AGGAACATTT	CATGTAAA-G	1584
BovB_Tr-RTE-Tiliqua_rugosa	AGAA-GGGGA	AAGACTAGAA	AT-CTCTTCA	AGAAAATTGG	A-GATATCAC	AGGAACATTC	CATGCAAA-G	1591
BovB_ALi-RTE-Amblyomma_limbatu	AGAA-TAGGG	AGGACCAGAG	ATACTCTTCA	AGAAAATTGG	A-GATATGAA	GGGAACATTT	CATCGAAA-G	1724
BovB_Hs-RTE-Hydrophis_spiralis	----------	----------	----------	----------	----------	----------	----------	-
BovB_VA-RTE-Vipera_ammodytes	AGAA-TAGGG	AGGACCAGAG	AT-CCTTTCA	AGAAAATTGG	A-GAGATGAA	GGGAACGTTT	CATGCAAA-A	1551
BovB_ACo-RTE-Agkistrodon_conto	AGAA-TAGGG	AGAACCAGAG	AT-CTCTTCA	AGAAAATTGG	A-GATATGAA	GGGAACGTTT	CATGCAAA-G	1551
BovB_Pg-RTE-Perameles_gunnii	----------	----------	----------	----------	----------	----------	----------	-
BovB_Io-RTE-Isoodon_obesulus	AGAATGAGGA	CAAGCAAGCT	ACTTCAAAAA	AAAAAAATTA	GAGATAGCAA	AGGAAACTTT	CATGCAAA-A	180
BovB_Bp-RTE-Burramys_parvus	TAGAATTGGA	AAAATGAGA-	-TCTCTTCAA	GAAAATAAGA	GATATCA---	--GAATATTT	CATGCAAA-A	250
BovB_Pb-RTE-Petaurus_breviceps	TAGAATGGGA	AAGACAAGAA	ATCTCTTCAA	GAAAATTAGA	GATATCACAG	GGGAACATTT	TATGCAAA-A	318
BovB_Ma-RTE-Macropus_antilopin	TAGAATGGGA	AAGAAAAGA-	-TCTCTTCAA	GAAAATTAGA	GATATCA--A	GGGAATGTTT	CACGCAAA-A	1674
BovB_Af-RTE-Antechinus_flavipe	CATAATGGGA	AAGACAAGAG	ATCTTAGCAA	GAAAATTAGA	AAGATTAT--	GAAAATGTTG	CATGCTGA-A	880
BovB_Ac-RTE-Anolis_carolinensi	AGAA-TAGGA	AGGACAAGAG	AC-CTCTTCC	AGAAAATCAG	A-AACATCGG	AGGCAAATTT	CAGGCAAA-A	1520
BovB_BM-RTE-Bombyx_mori	CTAAAGTT-A	CAATCCT-CT	GATTTATTTA	AGAAAGTTCG	T-CTGCTGTC	GAAAAAATAC	AAAGCA---A	1375
BovB_Dr-RTE-Christinus	AAGAAAGG-C	CACACCC-GT	GAGCTTTTTG	ACCATGTGAA	G-CAGGGGCG	GGCATCATTT	GCGCCCCGCA	1421
BovB_La-RTE-Loxodonta_africana	CAAAAGAA-A	AGAACAC-GC	TCAGCATTTC	TCAAGCTGAA	A-GAACTGAA	AAAAAAATTC	AAGCCTC---	1547
BovB_Pc-RTE-Procavia_capensis	CAGAAGGG-A	AGAACAC-GT	TCTGCATTTC	TCAAGCTGAA	A-GAACTGAA	GAAAAAATTC	AAGCCTC---	1547
BovB_Et-RTE-Christinus	-----GGG-A	AGAACGT-AC	-CAGCATACC	TGAAACGGAA	A-GACCCCAG	GAAAAAATTC	AAGCCTC---	1361
BovB_Ta-RTE-Tachyglossus_acule	AAATGTGG-G	CGGACAA-GA	TCACTATTTC	AGAAGATTAA	G-GAAATTAA	GGGAACATTT	CGTCCTT---	1684
BovB_Plat-RTE-Ornithorhynchus	AAATGTGG-G	CGGACAA-GA	TCACTATTTC	AGAAGATTAA	G-GAAATTAA	GGGAACATTT	CGTCCTC---	1673
BovB_Ec-RTE-Equus_caballus	AAAAAAGG-A	AGAACAA-GA	GGTCTCTTCC	AGAAGATTCA	A-GAAATCAA	AGGGAAATTT	AAACCTA---	1443
BovB_Cg-RTE-Christinus	AAAAAATGTA	AGAACAACAA	AATTGATTAC	ACAAAAATCA	A-GAAATCAT	AATGAAATTT	AAACCAT---	1029
BovB_Bh-RTE-Bothriocroton_hydr	----------	----------	----------	----------	----------	----------	----------	-
BovB_Ml-RTE-Gehyra_variegata	ATCATTTAGT	TTGATTATAT	ACTTTGCAGT	CAAAAATAGA	AAAGCACTGT	AAAGTCAGTT	AAAACTAG--	97
Sly-rnd1_family284-LINE/RTEBov	----------	----------	----------	----------	----------	----------	----------	-
Sly-rnd5_family2019-LINE/RTEBo	----------	----------	----------	----------	----------	----------	----------	-
Sly-rnd3_family322-LINE/RTEBov	----------	----------	----------	----------	----------	----------	----------	-
Sly-rnd5_family1698-LINE/RTEBo	----------	----------	----------	----------	----------	----------	----------	-
Sly-rnd1_family795-LINE/RTEBov	----------	----------	----------	----------	----------	----------	----------	-
Stu-rnd-5_family-3162_LINE_RTE	AGGGAGCGAG	TGGTGGAGGT	TAGGAGGATC	AATGATAGGN	TGATGACGAT	TAAGCTAGTC	ATTGGTAGGA	1188
Sly-rnd6_family1960-LINE/RTEBo	AGGGAGCAAG	CGGTAGAGGT	TAGGAGGGTN	AATGATAGGA	TGATGATNAT	TAAGCTAGTC	GTTGG-AGGG	367
Stu-rnd-5_family-2493_LINE_RTE	----------	----------	----------	----------	----------	----------	----------	-
Sly-rnd5_family447-LINE/RTEBov	----------	----------	----------	----------	----------	----------	----------	-
Stu-rnd-6_family-2093_LINE_RTE	----------	----------	----------	----------	----------	----------	----------	-
Consensus	---A---G--	A--AC-A---	------T---	--AAA-----	---A------	---AA--TT-	-A--C-----	

Conservation

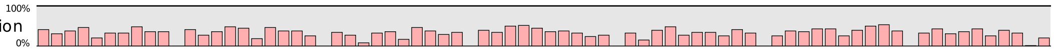

```
                                                      2,400                            2,420                            2,440
Stu-rnd-1_family-417_LINE_RTE-   ---------- ---------- ---------- ---------- ---------- ---------- ---------- -
Sly-rnd1_family377-LINE/RTEBov   ---------- ---------- ---------- ---------- ---------- ---------- ---------- -
Stu-rnd-1_family-489_LINE_RTE-   ---------- ---------- ---------- ---------- ---------- ---------- ---------- -
Stu-rnd-1_family-607_LINE_RTE-   ---------- ---------- ---------- ---------- ---------- ---------- ---------- -
Stu-rnd-1_family-645_LINE_RTE-   ---------- ---------- ---------- ---------- ---------- ---------- ---------- -
Sly-rnd4_family81-LINE/RTEBovB   ---------- ---------- ---------- ---------- ---------- ---------- ---------- -
Stu-rnd-6_family-408_LINE_RTE-   ---------- ---------- ---------- ---------- ---------- ---------- ---------- -
Stu-rnd-1_family-351_LINE_RTE-   ---------- ---------- ---------- ---------- ---------- ---------- ---------- -
Stu-rnd-1_family-382_LINE_RTE-   ---------- ---------- ---------- ---------- ---------- ---------- ---------- -
Sly-rnd6_family746-LINE/RTEBov   ---------- ---------- ---------- ---------- ---------- ---------- ---------- -
Stu-rnd-5_family-325_LINE_RTE-   ---------- ---------- ---------- ---------- ---------- ---------- ---------- -
Sly-rnd4_family509-LINE/RTEBov   ---------- ---------- ---------- ---------- ---------- ---------- ---------- -
Stu-rnd-1_family-513_LINE_RTE-   ---------- ---------- ---------- ---------- ---------- ---------- ---------- -
Sly-rnd5_family1442-LINE/RTEBo   ---------- ---------- ---------- ---------- ---------- ---------- ---------- -
BovB_Gl-RTE-Gehyra_lazelli       GCTGG-CCAC AATAAA-GGA CAAAAATGG- CAGGGACCTA ACAG--AAGC AGAAGAGATC AGGAAAAGAT 1759
BovB_Gv-RTE-Gehyra_variegata     GCTGGGCCAC AATAAAAGGA CAAAAATGGA CAGGGACCTA ACAGT-AAGC AGAAGAGATC AGGAAAAGAT 1689
BovB_PMo-RTE-Python_molurus      GATGG-GCAT GATAAA-GGA CAAAAAAGG- CAGGGACCTA ACAG--AAGC AGAAGAGATT AAGAAGAGGT 1623
BovB_Oa-RTE-Ovis_aries           A-TGG-GCAC AATAAA-GGA CAGAAATGG- TATGGACCTA ACAG--AAGC AGAAGAGATT AAGAAGAGGT 1647
BovB-RTE-Bos_taurus              A-TGG-GCTC GATAAA-GGA CAGAAATGG- TATGGACCTA ACAG--AAGC AGAAGATATT AAGAAGAGAT 2245
BovB_Me-RTE-Macropus_eugenii     AATGA-GCAT GGTAAA---A CAAAAATGG- TAGGGACTTA ACAG--AAG- ---------- ---------- 1472
BovB_Ano-RTE-Amphibolurus_norr   A-TGG-ACAT GATAAA-GGA CAAAAATGG- GAGGGACCTA ACAG--AAGC AGAAGACGTC AAGAAGAGGT 1694
BovB_Gd-RTE-Eremiascincus_doug   G-TGG-GCAC AATAAA-GGA CAAGAACGG- CAAGGACCTA ACAG--AAGC AGATGAGATT AAGAAGAGGT 1282
BovB_Er-RTE-Eremiascincus_rich   G-TGG-GCAC AATAAA-GGA CAAGAACGG- CAAGGACCTA ACAG--AAGC AGATGAGATT AAGAAGAGGT 1817
BovB_Ca-RTE-Ctenotus_atlas       G-TAG-GCAC AATAAA-GGA CAAGAACGG- CAAGGACCTA ACAG--AAGC AGATGAGATT AAGAAGAGGT 1810
BovB_Es-RTE-Egernia_stokesii     A-TGG-GCAT GATAAA-GAA CAAGAATGG- TAGGGACCTA ATGG--AAGC AGACAAAATT AAGAAGAGCT 1648
BovB_Tr-RTE-Tiliqua_rugosa       A-TGG-GCAT GATAAT-GGA CAAGAATAG- TAGGAACCTA ACGG--AAG- ----AAGATT AAGAAGAGAA 1650
BovB_ALi-RTE-Amblyomma_limbatu   ATGGG--CAT AATAAA-GGA CCAAAATGG- CAGGGACCTA ACAG--AGAC AGAAGAGATC AAGAAGAGGT 1788
BovB_Hs-RTE-Hydrophis_spiralis   ---------- ---------- ---------- --------GA ATGA--ACAC TCTGAGTCTG CCTCGTATCT 30
BovB_VA-RTE-Vipera_ammodytes     ATGGG--CAT GATAAA-GGA CCAAAATGG- CAGGGACCTA ACAG--AGGC AGAAGAGATT AAGAAGAGGT 1615
BovB_ACo-RTE-Agkistrodon_conto   ATGGG--CAT GATAAA-GGA CCAAAATGG- CAGGGACCTA ACAG--AGGC AGAAGAGATT AAGAAGAGGT 1615
BovB_Pg-RTE-Perameles_gunnii     ---------- ---------- ---------- ---------- ---------- ---------- ---------- -
BovB_Io-RTE-Isoodon_obesulus     ATCGT--CAT GATAAA-AGA CAAAATAGGG TA-------- --GA--CAAC AGAAGTGATT AA-AATAGAT 234
BovB_Bp-RTE-Burramys_parvus      ATGGG--CAT GATAAA-AGA CAAACAATGG TAGGGAATTA ACAG--AAGC AAAAGAGATT AAGAAGAGGT 315
BovB_Pb-RTE-Petaurus_breviceps   ATAGG--CAT GATAAA-AGA CAAA-AATGG TATGGACCTA ATAG--AAGC AGAAGAGATT AA---GAGGT 379
BovB_Ma-RTE-Macropus_antilopin   ATGGG--ATT AAAAAA-AAA AAAGATAGGA AAT------- -TAG--CAAC AGAAGAGAAT AAGAAGAGGT 1731
BovB_Af-RTE-Antechinus_flavipe   AGAGG--TAT AATAAA-AGT CAAA-AAGGA CAAAGTCTTG ACGG--AAGT AGAAGAGATT AAGATGAAGT 944
BovB_Ac-RTE-Anolis_carolinensi   AAGGG--CAT GATCAA-AAA CAAAGATGG- CAAGAACCTA ACAG--AAGC TGAAGAGATC AAGAAAAGGT 1584
BovB_BM-RTE-Bombyx_mori          AGACCTGGGT TATCGG-CGA CGAGGATGGC GACTCCATCA CTGA--CCTT CAGCTGA-TC ACAGAAAGAT 1441
BovB_Dr-RTE-Christinus           AAGCAGCAAA TGTCAA-AGA TCGGAATGGT AAAGT-ACTA CAGA--ATGA AGACCAAATC AAGAGAAGGT 1487
BovB_La-RTE-Loxodonta_africana   AAGTTGCAAT ACTGAA-GGA TTCCATGGGG AAAATATTAA ACGA---CGC AGAAAGCATC AAAAGAAGAT 1613
BovB_Pc-RTE-Procavia_capensis    GAGTTGCAAT AGTGAA-GGA TTCTACAGGG AAAATATTAA ATGA---CTC AGGGAGCATC AAAAGAAGAT 1613
BovB_Et-RTE-Christinus           AAACTTCAAT TCTGAA-AGA TTCTATAGGC AAAATATTGA ATGA---TGC AGGAAGCATC AAGAGAAGAT 1427
BovB_Ta-RTE-Tachyglossus_acule   GGATTGGCTT TGTCAA-AAG CAAAGATGGA CTGACGATAA ATGA---TGC CAGTGGAATC AAACAGAGAT 1750
BovB_Plat-RTE-Ornithorhynchus    GGATTGGCTT TGTCAA-AAG CAAAGATGGA CTGACGATAA ATGA---TGC CAGTGGAATC AAACAGAGAT 1739
BovB_Ec-RTE-Equus_caballus       GATTAGGAAT GCTGAA-CGA CCAACAGGGA AGCACACTAT CTGA---TCA GGAGAAAATA AAGGGAAGGT 1509
BovB_Cg-RTE-Christinus           GTTTAGGAAT GCTAGA-AGA ACAACATGGA AAATCATGAT CTGA---TCA AGAAAAAATT AAGAGAAGAT 1095
BovB_Bh-RTE-Bothriocroton_hydr   ---------- ---AATGAGA CAGAGTGGGA AACGCGCCCT CTCATCCTGC CCACTGACTC CCTCTATGGT 57
BovB_Ml-RTE-Gehyra_variegata     -ACCTAGAAC TGAATACAGA CTAGATTAT- GAGTTTTTTA TTAA---AAA ATTTTGACTT AAATTGAAGT 162
Sly-rnd1_family284-LINE/RTEBov   ---------- ---------- ---------- ---------- ---------- ---------- ---------- -
Sly-rnd5_family2019-LINE/RTEBo   ---------- ---------- ---------- ---------- ---------- ---------- ---------- -
Sly-rnd3_family322-LINE/RTEBov   ---------- ---------- ---------- ---------- ---------- ---------- ---------- -
Sly-rnd5_family1698-LINE/RTEBo   ---------- ---------- ---------- ---------- ---------- ---------- ---------- -
Sly-rnd1_family795-LINE/RTEBov   ---------- ---------- ---------- ---------- ---------- ---------- ---------- -
Stu-rnd-5_family-3162_LINE_RTE   TGTACTTTGA GTGTTATTAG TGCTTATGCG CCTCAAGTGG GCNTGGACGA GGAGGCCAAA AAGCTCTTTT 1258
Sly-rnd6_family1960-LINE/RTEBo   CTTACTTTGA ACATNATTAG TGCNTACGCG CCGCAAGCGG GCTTGGACGA GGAGGNCAAA AAGCTNTTTT 437
Stu-rnd-5_family-2493_LINE_RTE   ---------- ---------- ---------- ---------- ---------- ---------- ---------- -
Sly-rnd5_family447-LINE/RTEBov   ---------- ---------- ---------- ---------- ---------- ---------- ---------- -
Stu-rnd-6_family-2093_LINE_RTE   ---------- ---------- ---------- ---------- ---------- ---------- ---------- -

Consensus                        --------A- --T-AA--GA C-A--A-GG- -A-------A A-------GC -GA----AT- AA----AG-T
```

Conservation (100% / 0%)

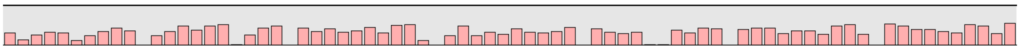

```
                                      2,460                              2,480                              2,500                              2,520
Stu-rnd-1_family-417_LINE_RTE-       ---------- ---------- ---------- ---------- ---------- ---------- ---------- -
Sly-rnd1_family377-LINE/RTEBov       ---------- ---------- ---------- ---------- ---------- ---------- ---------- -
Stu-rnd-1_family-489_LINE_RTE-       ---------- ---------- ---------- ---------- ---------- ---------- ---------- -
Stu-rnd-1_family-607_LINE_RTE-       ---------- ---------- ---------- ---------- ---------- ---------- ---------- -
Stu-rnd-1_family-645_LINE_RTE-       ---------- ---------- ---------- ---------- ---------- ---------- ---------- -
Sly-rnd4_family81-LINE/RTEBovB       ---------- ---------- ---------- ---------- ---------- ---------- ---------- -
Stu-rnd-6_family-408_LINE_RTE-       ---------- ---------- ---------- ---------- ---------- ---------- ---------- -
Stu-rnd-1_family-351_LINE_RTE-       ---------- ---------- ---------- ---------- ---------- ---------- ---------- -
Stu-rnd-1_family-382_LINE_RTE-       ---------- ---------- ---------- ---------- ---------- ---------- ---------- -
Sly-rnd6_family746-LINE/RTEBov       ---------- ---------- ---------- ---------- ---------- ---------- ---------- -
Stu-rnd-5_family-325_LINE_RTE-       ---------- ---------- ---------- ---------- ---------- ---------- ---------- -
Sly-rnd4_family509-LINE/RTEBov       ---------- ---------- ---------- ---------- ---------- ---------- ---------- -
Stu-rnd-1_family-513_LINE_RTE-       ---------- ---------- ---------- ---------- ---------- ---------- ---------- -
Sly-rnd5_family1442-LINE/RTEBo       ---------- ---------- ---------- ---------- ---------- ---------- ---------- -
BovB_Gl-RTE-Gehyra_lazelli           -GGCAAGAAT ACACAGAAGG ACTATACAAG AAAG------ ACCTCAATGT CCCT---GAC ACCCACGGCG 1819
BovB_Gv-RTE-Gehyra_variegata         -GGCAAGAAT ACACAGAAGG ATTGTACAAG AAAG------ ACCTCAATGT CCCT---GAC ACCCACGGTG 1749
BovB_PMo-RTE-Python_molurus          -GGCAAGAAT ACACAGAAGA ACTATACAAG AAAG------ ATCTTAATGT CCCT---GAT GTCCA--GCT 1681
BovB_Oa-RTE-Ovis_aries               -GGCAAGAAT ACACAGAAGA ACTATACAAA AAAG------ ATCTTAATGA CCCA---GAT AACCACGATG 1707
BovB-RTE-Bos_taurus                  -GGCAAGAAT ACACAGAAGA ACTGTACAAA AAAG------ ATCTTCACGA CCCA---GAT AATCACGATG 2305
BovB_Me-RTE-Macropus_eugenii         -------AAC ATACAT---- -CTATATATA TAAC------ ATGTATATAA C-------AT AA-------- 1509
BovB_Ano-RTE-Amphibolurus_norr       -GGCAAGAAT ACACAGAGGA ATTATATCAG AAAG------ ATTTGGATAT CCCG---GAC AACCCAGACA 1754
BovB_Gd-RTE-Eremiascincus_doug       -GGCAAGAAT ACACAGAGGA ACTCTATAAA GCTG------ ATCTAAATGT TCCT---GAT AACCACAATG 1342
BovB_Er-RTE-Eremiascincus_rich       -GGCAAGAAT ACACAGAGGA ACTATATAAG GCTG------ ATCTAAATGT TCCT---GAT AACCACAATG 1877
BovB_Ca-RTE-Ctenotus_atlas           -GGCAAGAAT ACACAGAGGA ACTATATAAG GCCG------ ATCTAAATGT TCCT---AAT AACCACAATG 1870
BovB_Es-RTE-Egernia_stokesii         -GGCAAGAAT ACACAGAGGA ACTCTACAAG AAAG------ ----TAGTGT CCCC---CAT AACCACGCTA 1704
BovB_Tr-RTE-Tiliqua_rugosa           -GGCAAGAAT ACACAGAGGA ACTCTACAAG AAAG------ ATCTTAATGT CCCT---GAT AACCAAGATG 1710
BovB_ALi-RTE-Amblyomma_limbatu       AGGCAAGATT ACACAGAAGA ACTATACAAG AAAGT----- AGCTTAACAT TCCA---TAA AACCACGAAG 1850
BovB_Hs-RTE-Hydrophis_spiralis       CCGCCGCTCT ACA-ACAATA ACTATATAAG AATG------ AGCTTAACAT CCCT---GAT AACCACGATG 90
BovB_VA-RTE-Vipera_ammodytes         -GGCAAAATT ACACAGAAGA ACTATACAAG AATG------ AGCTTAACGT CCCT---GAT AATCTCAATG 1675
BovB_ACo-RTE-Agkistrodon_conto       -GGCAAAATT ACACAGAAGA ACTATACAAG AACG------ AGCTTAACGT CCCT---GAT AACCACGATG 1675
BovB_Pg-RTE-Perameles_gunnii         ---------- ---------- ---------- ---------- ---------- ---------- ---------- -
BovB_Io-RTE-Isoodon_obesulus         -AGTAGAAGT ACATAAAATA ATTACACAAG A--------- ---TTTTCAC CTGT---GAT AACCACAACA 288
BovB_Bp-RTE-Burramys_parvus          -GGCAAGAAT ATACAAAAGA ACTATACAAG AAAG------ ATCTTAACAT CACT---GAT AACCATGATG 375
BovB_Pb-RTE-Petaurus_breviceps       -GGCAAGAAT ATACAAAAGA ACTATACAAG AAAG------ ATCTTAACAT CACC---AAT AACTACTATG 439
BovB_Ma-RTE-Macropus_antilopin       -GGCAAGAAT ACACAAAAGA ACTATCAAGA AAA------- -TCTTAACAT CACC---AAT AACCATGATG 1789
BovB_Af-RTE-Antechinus_flavipe       -AGCAAGAAT ATACAAAAGA TCTATACAAG AAAG------ ATTTTCACAT CACT---GAT AACCATAATG 1004
BovB_Ac-RTE-Anolis_carolinensi       -GGCGAGAAT ATACAGAAGA TCTGTATAGG AAGG------ ATAATAATAT AGAG---GAT AGCTTTGACG 1644
BovB_BM-RTE-Bombyx_mori              -GGCGTCGTT ATTGTGAAAG CCTTTACAAA AACCCCACTA ATGACACCAC TTGTGGTCCG GATTTTGTTT 1510
BovB_Dr-RTE-Christinus               -GGCGAGAGT ACACAGAAGA ATTATACACG GGC--AATCA ACTGAGTCAT TCA----GCC AATCAGGAAG 1550
BovB_La-RTE-Loxodonta_africana       -GGAAGGAAT ACACAGAGTC ACTATACCAA AAA------G AATTAGTCGA TATT---CAA CCAATTCAAG 1673
BovB_Pc-RTE-Procavia_capensis        -GGAAGGAAT ACACAGAGTC ATTATACCAG AAA------G AACTGGTCGA TGTT---CAG CCATTTGAAG 1673
BovB_Et-RTE-Christinus               -GGAAAGAAT AC-------- ---GTACCCC AAA------G AACTAATCAT TATT---CCA CCATTCTAGG 1476
BovB_Ta-RTE-Tachyglossus_acule       -GGAAGGAAT ACACACAGGA ACTATACCAG AAA------G ATAGCAATAT ACAA---GAA GTATTTGAAG 1810
BovB_Plat-RTE-Ornithorhynchus        -GGAAGGAAT ACACACAGGA ACTATACCAG AAA------G ATAGCAATAT ACAA---GAA GTATTTGAAG 1799
BovB_Ec-RTE-Equus_caballus           -GGAAACAGT ACACTGAAGA TCTATACAGA AGA------G ACAAAAGGAT GACA---GAT TCCTTTAAAG 1569
BovB_Cg-RTE-Christinus               ---------- ---------- ---ATGCAGA TGA------T ACAA------ ---------- ---------- 1110
BovB_Bh-RTE-Bothriocroton_hydr       -CGCGCGGAT GAGTCATTTT TCTATGTGCG TAGC------ ---ACGGTGC CTTA---CAT AGTGTGCTAT 114
BovB_Ml-RTE-Gehyra_variegata         TGAGAAAACT AT-CAGACAA AATAGCTAAG AACT------ -TACTAAAGT CCCTTA-TAA AGTGAAGATG 223
Sly-rnd1_family284-LINE/RTEBov       ---------- ---------- ---------- ---------- ---------- ---------- ---------- -
Sly-rnd5_family2019-LINE/RTEBo       ---------- ---------- ---------- ---------- ---------- ---------- ---------- -
Sly-rnd3_family322-LINE/RTEBov       ---------- ---------- ---------- ---------- ---------- ---------- ---------- -
Sly-rnd5_family1698-LINE/RTEBo       ---------- ---------- ---------- ---------- ---------- ---------- ---------- -
Sly-rnd1_family795-LINE/RTEBov       ---------- ---------- ---------- ---------- ---------- ---------- ---------- -
Stu-rnd-5_family-3162_LINE_RTE       AGGAGGATTT GGACGAGGTA GTNAGAGGTA TACCGAATAC CGAGAAGATT NTCATTGGTG GAGATTTTAA 1328
Sly-rnd6_family1960-LINE/RTEBo       GGGAGGAGTT GGACGAGGTN GTGAGAGGTA TACCGCACAC CGAGAAGCTA TTTNTAGGAG GAGATTTTAA 507
Stu-rnd-5_family-2493_LINE_RTE       ---------- ---------- ---------- ---------- ---------- ---------- ---------- -
Sly-rnd5_family447-LINE/RTEBov       ---------- ---------- ---------- ---------- ---------- ---------- ---------- -
Stu-rnd-6_family-2093_LINE_RTE       ---------- ---------- ---------- ---------- ---------- ---------- ---------- -

Consensus                            -GG-A--A-T ACACA-A--- -CTATACAAG AA-------- A----A---T --------A- A------A-G
```

Conservation

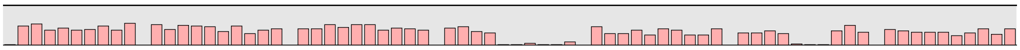

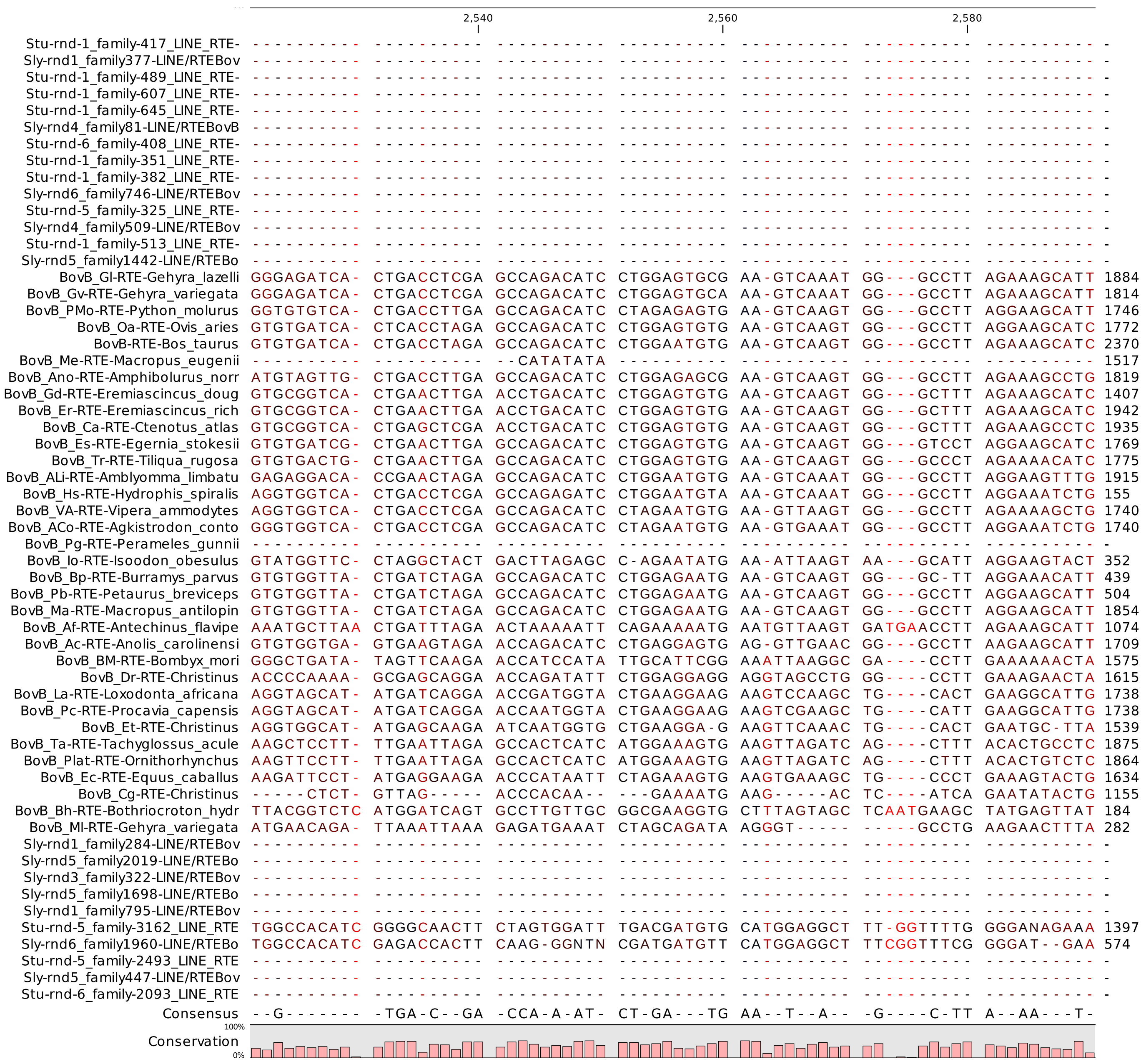
2,540
2,560
2,580
Stu-rnd-1_family-417_LINE_RTE- ---------- ---------- ---------- ---------- ---------- ---------- ---------- -
Sly-rnd1_family377-LINE/RTEBov ---------- ---------- ---------- ---------- ---------- ---------- ---------- -
Stu-rnd-1_family-489_LINE_RTE- ---------- ---------- ---------- ---------- ---------- ---------- ---------- -
Stu-rnd-1_family-607_LINE_RTE- ---------- ---------- ---------- ---------- ---------- ---------- ---------- -
Stu-rnd-1_family-645_LINE_RTE- ---------- ---------- ---------- ---------- ---------- ---------- ---------- -
Sly-rnd4_family81-LINE/RTEBovB ---------- ---------- ---------- ---------- ---------- ---------- ---------- -
Stu-rnd-6_family-408_LINE_RTE- ---------- ---------- ---------- ---------- ---------- ---------- ---------- -
Stu-rnd-1_family-351_LINE_RTE- ---------- ---------- ---------- ---------- ---------- ---------- ---------- -
Stu-rnd-1_family-382_LINE_RTE- ---------- ---------- ---------- ---------- ---------- ---------- ---------- -
Sly-rnd6_family746-LINE/RTEBov ---------- ---------- ---------- ---------- ---------- ---------- ---------- -
Stu-rnd-5_family-325_LINE_RTE- ---------- ---------- ---------- ---------- ---------- ---------- ---------- -
Sly-rnd4_family509-LINE/RTEBov ---------- ---------- ---------- ---------- ---------- ---------- ---------- -
Stu-rnd-1_family-513_LINE_RTE- ---------- ---------- ---------- ---------- ---------- ---------- ---------- -
Sly-rnd5_family1442-LINE/RTEBo ---------- ---------- ---------- ---------- ---------- ---------- ---------- -
BovB_Gl-RTE-Gehyra_lazelli GGGAGATCA- CTGACCTCGA GCCAGACATC CTGGAGTGCG AA-GTCAAAT GG---GCCTT AGAAAGCATT 1884
BovB_Gv-RTE-Gehyra_variegata GGGAGATCA- CTGACCTCGA GCCAGACATC CTGGAGTGCA AA-GTCAAAT GG---GCCTT AGAAAGCATT 1814
BovB_PMo-RTE-Python_molurus GGTGTGTCA- CTGACCTTGA GCCAGACATC CTAGAGAGTG AA-GTCAAGT GG---GCCTT AGGAAGCATT 1746
BovB_Oa-RTE-Ovis_aries GTGTGATCA- CTCACCTAGA GCCAGACATC CTGGAGTGTG AA-GTCAAGT GG---GCCTT AGGAAGCATC 1772
BovB-RTE-Bos_taurus GTGTGATCA- CTGACCTAGA GCCAGACATC CTGGAATGTG AA-GTCAAGT GG---GCCTT AGAAAGCATC 2370
BovB_Me-RTE-Macropus_eugenii ---------- ---------- --CATATATA ---------- ---------- ---------- ---------- 1517
BovB_Ano-RTE-Amphibolurus_norr ATGTAGTTG- CTGACCTTGA GCCAGACATC CTGGAGAGCG AA-GTCAAGT GG---GCCTT AGAAAGCCTG 1819
BovB_Gd-RTE-Eremiascincus_doug GTGCGGTCA- CTGAACTTGA ACCTGACATC CTGGAGTGTG AA-GTCAAGT GG---GCTTT AGAAAGCATC 1407
BovB_Er-RTE-Eremiascincus_rich GTGCGGTCA- CTGAACTTGA ACCTGACATC CTGGAGTGTG AA-GTCAAGT GG---GCTTT AGAAAGCATC 1942
BovB_Ca-RTE-Ctenotus_atlas GTGCGGTCA- CTGAGCTCGA ACCTGACATC CTGGAGTGTG AA-GTCAAGT GG---GCTTT AGAAAGCCTC 1935
BovB_Es-RTE-Egernia_stokesii GTGTGATCG- CTGAACTTGA GCCAGACATC CTGGAGTGTG AA-GTCAAGT GG---GTCCT AGGAAGCATC 1769
BovB_Tr-RTE-Tiliqua_rugosa GTGTGACTG- CTGAACTTGA GCCAGACATC CTGGAGTGTG AA-GTCAAGT GG---GCCCT AGAAAACATC 1775
BovB_ALi-RTE-Amblyomma_limbatu GAGAGGACA- CCGAACTAGA GCCAGACATC CTGGAATGTG AA-GTCAAAT GG---GCCTT AGGAAGTTTG 1915
BovB_Hs-RTE-Hydrophis_spiralis AGGTGGTCA- CTGACCTCGA GCCAGAGATC CTGGAATGTA AA-GTCAAAT GG---GCCTT AGGAAATCTG 155
BovB_VA-RTE-Vipera_ammodytes AGGTGGTCA- CTGACCTCGA GCCAGACATC CTAGAATGTG AA-GTTAAGT GG---GCCTT AGAAAAGCTG 1740
BovB_ACo-RTE-Agkistrodon_conto GGGTGGTCA- CTGACCTCGA GCCAGACATC CTAGAATGTG AA-GTGAAAT GG---GCCTT AGGAAATCTG 1740
BovB_Pg-RTE-Perameles_gunnii ---------- ---------- ---------- ---------- ---------- ---------- ---------- -
BovB_Io-RTE-Isoodon_obesulus GTATGGTTC- CTAGGCTACT GACTTAGAGC C-AGAATATG AA-ATTAAGT AA---GCATT AGGAAGTACT 352
BovB_Bp-RTE-Burramys_parvus GTGTGGTTA- CTGATCTAGA GCCAGACATC CTGGAGAATG AA-GTCAAGT GG---GC-TT AGGAAACATT 439
BovB_Pb-RTE-Petaurus_breviceps GTGTGGTTA- CTGATCTAGA GCCAGACATC CTGGAGAATG AA-GTCAAGT GG---GCCTT AGGAAGCATT 504
BovB_Ma-RTE-Macropus_antilopin GTGTGGTTA- CTGATCTAGA GCCAGACATC CTGGAGAATG AA-GTCAAGT GG---GCCTT AGGAAGCATT 1854
BovB_Af-RTE-Antechinus_flavipe AAATGCTTAA CTGATTTAGA ACTAAAAATT CAGAAAAATG AATGTTAAGT GATGAACCTT AGAAAGCATT 1074
BovB_Ac-RTE-Anolis_carolinensi GTGTGGTGA- GTGAAGTAGA ACCAGACATC CTGAGGAGTG AG-GTTGAAC GG---GCCTT AAGAAGCATT 1709
BovB_BM-RTE-Bombyx_mori GGGCTGATA- TAGTTCAAGA ACCATCCATA TTGCATTCGG AAATTAAGGC GA----CCTT GAAAAAACTA 1575
BovB_Dr-RTE-Christinus ACCCCAAAA- GCGAGCAGGA ACCAGATATT CTGGAGGAGG AGGTAGCCTG GG----CCTT GAAAGAACTA 1615
BovB_La-RTE-Loxodonta_africana AGGTAGCAT- ATGATCAGGA ACCGATGGTA CTGAAGGAAG AAGTCCAAGC TG----CACT GAAGGCATTG 1738
BovB_Pc-RTE-Procavia_capensis AGGTAGCAT- ATGATCAGGA ACCAATGGTA CTGAAGGAAG AAGTCGAAGC TG----CATT GAAGGCATTG 1738
BovB_Et-RTE-Christinus AGGTGGCAT- ATGAGCAAGA ATCAATGGTG CTGAAGGA-G AAGTTCAAAC TG----CACT GAATGC-TTA 1539
BovB_Ta-RTE-Tachyglossus_acule AAGCTCCTT- TTGAATTAGA GCCACTCATC ATGGAAAGTG AAGTTAGATC AG----CTTT ACACTGCCTC 1875
BovB_Plat-RTE-Ornithorhynchus AAGTTCCTT- TTGAATTAGA GCCACTCATC ATGGAAAGTG AAGTTAGATC AG----CTTT ACACTGTCTC 1864
BovB_Ec-RTE-Equus_caballus AAGATTCCT- ATGAGGAAGA ACCCATAATT CTAGAAAGTG AAGTGAAAGC TG----CCCT GAAAGTACTG 1634
BovB_Cg-RTE-Christinus -----CTCT- GTTAG----- ACCCACAA-- ---GAAAATG AAG-----AC TC----ATCA GAATATACTG 1155
BovB_Bh-RTE-Bothriocroton_hydr TTACGGTCTC ATGGATCAGT GCCTTGTTGC GGCGAAGGTG CTTTAGTAGC TCAATGAAGC TATGAGTTAT 184
BovB_Ml-RTE-Gehyra_variegata ATGAACAGA- TTAAATTAAA GAGATGAAAT CTAGCAGATA AGGGT----- -----GCCTG AAGAACTTTA 282
Sly-rnd1_family284-LINE/RTEBov ---------- ---------- ---------- ---------- ---------- ---------- ---------- -
Sly-rnd5_family2019-LINE/RTEBo ---------- ---------- ---------- ---------- ---------- ---------- ---------- -
Sly-rnd3_family322-LINE/RTEBov ---------- ---------- ---------- ---------- ---------- ---------- ---------- -
Sly-rnd5_family1698-LINE/RTEBo ---------- ---------- ---------- ---------- ---------- ---------- ---------- -
Sly-rnd1_family795-LINE/RTEBov ---------- ---------- ---------- ---------- ---------- ---------- ---------- -
Stu-rnd-5_family-3162_LINE_RTE TGGCCACATC GGGGCAACTT CTAGTGGATT TGACGATGTG CATGGAGGCT TT-GGTTTTG GGGANAGAAA 1397
Sly-rnd6_family1960-LINE/RTEBo TGGCCACATC GAGACCACTT CAAG-GGNTN CGATGATGTT CATGGAGGCT TTCGGTTTCG GGGAT--GAA 574
Stu-rnd-5_family-2493_LINE_RTE ---------- ---------- ---------- ---------- ---------- ---------- ---------- -
Sly-rnd5_family447-LINE/RTEBov ---------- ---------- ---------- ---------- ---------- ---------- ---------- -
Stu-rnd-6_family-2093_LINE_RTE ---------- ---------- ---------- ---------- ---------- ---------- ---------- -
Consensus --G------- -TGA-C--GA -CCA-A-AT- CT-GA---TG AA--T--A-- -G----C-TT A--AA---T-
100%
Conservation
0%

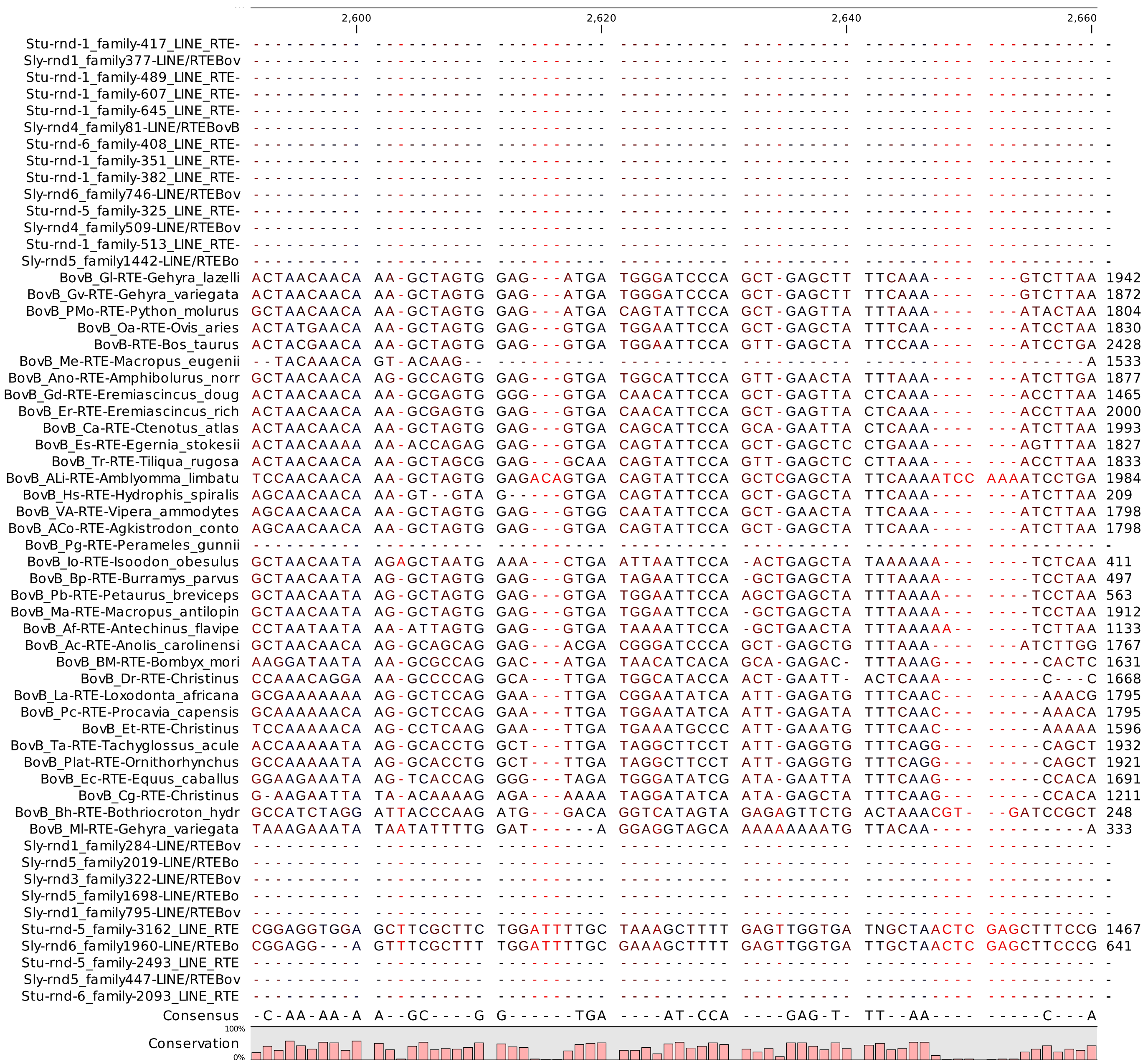

2,600 2,620 2,640 2,660
Stu-rnd-1_family-417_LINE_RTE- ---------- ---------- ---------- ---------- ---------- ---------- ---------- -
Sly-rnd1_family377-LINE/RTEBov ---------- ---------- ---------- ---------- ---------- ---------- ---------- -
Stu-rnd-1_family-489_LINE_RTE- ---------- ---------- ---------- ---------- ---------- ---------- ---------- -
Stu-rnd-1_family-607_LINE_RTE- ---------- ---------- ---------- ---------- ---------- ---------- ---------- -
Stu-rnd-1_family-645_LINE_RTE- ---------- ---------- ---------- ---------- ---------- ---------- ---------- -
Sly-rnd4_family81-LINE/RTEBovB ---------- ---------- ---------- ---------- ---------- ---------- ---------- -
Stu-rnd-6_family-408_LINE_RTE- ---------- ---------- ---------- ---------- ---------- ---------- ---------- -
Stu-rnd-1_family-351_LINE_RTE- ---------- ---------- ---------- ---------- ---------- ---------- ---------- -
Stu-rnd-1_family-382_LINE_RTE- ---------- ---------- ---------- ---------- ---------- ---------- ---------- -
Sly-rnd6_family746-LINE/RTEBov ---------- ---------- ---------- ---------- ---------- ---------- ---------- -
Stu-rnd-5_family-325_LINE_RTE- ---------- ---------- ---------- ---------- ---------- ---------- ---------- -
Sly-rnd4_family509-LINE/RTEBov ---------- ---------- ---------- ---------- ---------- ---------- ---------- -
Stu-rnd-1_family-513_LINE_RTE- ---------- ---------- ---------- ---------- ---------- ---------- ---------- -
Sly-rnd5_family1442-LINE/RTEBo ---------- ---------- ---------- ---------- ---------- ---------- ---------- -
BovB_Gl-RTE-Gehyra_lazelli ACTAACAACA AA-GCTAGTG GAG---ATGA TGGGATCCCA GCT-GAGCTT TTCAAA---- ---GTCTTAA 1942
BovB_Gv-RTE-Gehyra_variegata ACTAACAACA AA-GCTAGTG GAG---ATGA TGGGATCCCA GCT-GAGCTT TTCAAA---- ---GTCTTAA 1872
BovB_PMo-RTE-Python_molurus GCTAACAACA AA-GCTAGTG GAG---ATGA CAGTATTCCA GCT-GAGTTA TTTAAA---- ---ATACTAA 1804
BovB_Oa-RTE-Ovis_aries ACTATGAACA AA-GCTAGTG GAG---GTGA TGGAATTCCA GCT-GAGCTA TTTCAA---- ---ATCCTAA 1830
BovB-RTE-Bos_taurus ACTACGAACA AA-GCTAGTG GAG---GTGA TGGAATTCCA GTT-GAGCTA TTCCAA---- ---ATCCTGA 2428
BovB_Me-RTE-Macropus_eugenii --TACAAACA GT-ACAAG-- ---------- ---------- ---------- ---------- ---------A 1533
BovB_Ano-RTE-Amphibolurus_norr GCTAACAACA AG-GCCAGTG GAG---GTGA TGGCATTCCA GTT-GAACTA TTTAAA---- ---ATCTTGA 1877
BovB_Gd-RTE-Eremiascincus_doug ACTAACAACA AA-GCGAGTG GGG---GTGA CAACATTCCA GCT-GAGTTA CTCAAA---- ---ACCTTAA 1465
BovB_Er-RTE-Eremiascincus_rich ACTAACAACA AA-GCGAGTG GAG---GTGA CAACATTCCA GCT-GAGTTA CTCAAA---- ---ACCTTAA 2000
BovB_Ca-RTE-Ctenotus_atlas ACTAACAACA AA-GCTAGTG GAG---GTGA CAGCATTCCA GCA-GAATTA CTCAAA---- ---ATCTTAA 1993
BovB_Es-RTE-Egernia_stokesii ACTAACAAAA AA-ACCAGAG GAG---GTGA CAGTATTCCA GCT-GAGCTC CTGAAA---- ---AGTTTAA 1827
BovB_Tr-RTE-Tiliqua_rugosa ACTAACAACA AA-GCTAGCG GAG---GCAA CAGTATTCCA GTT-GAGCTC CTTAAA---- ---ACCTTAA 1833
BovB_ALi-RTE-Amblyomma_limbatu TCCAACAACA AA-GCTAGTG GAGACAGTGA CAGTATTCCA GCTCGAGCTA TTCAAAATCC AAAATCCTGA 1984
BovB_Hs-RTE-Hydrophis_spiralis AGCAACAACA AA-GT--GTA G-----GTGA CAGTATTCCA GCT-GAGCTA TTCAAA---- ---ATCTTAA 209
BovB_VA-RTE-Vipera_ammodytes AGCAACAACA AA-GCTAGTG GAG---GTGG CAATATTCCA GCT-GAACTA TTCAAA---- ---ATCTTAA 1798
BovB_ACo-RTE-Agkistrodon_conto AGCAACAACA AA-GCTAGTG GAG---GTGA CAGTATTCCA GCT-GAGCTA TTCAAA---- ---ATCTTAA 1798
BovB_Pg-RTE-Perameles_gunnii ---------- ---------- ---------- ---------- ---------- ---------- ---------- -
BovB_Io-RTE-Isoodon_obesulus GCTAACAATA AGAGCTAATG AAA---CTGA ATTAATTCCA -ACTGAGCTA TAAAAAA--- ----TCTCAA 411
BovB_Bp-RTE-Burramys_parvus GCTAACAATA AG-GCTAGTG GAG---GTGA TAGAATTCCA -GCTGAGCTA TTTAAAA--- ----TCCTAA 497
BovB_Pb-RTE-Petaurus_breviceps GCTAACAATA AG-GCTAGTG GAG---GTGA TGGAATTCCA AGCTGAGCTA TTTAAAA--- ----TCCTAA 563
BovB_Ma-RTE-Macropus_antilopin GCTAACAATA AG-GCTAGTG GAG---GTGA TGGAATTCCA -GCTGAGCTA TTTAAAA--- ----TCCTAA 1912
BovB_Af-RTE-Antechinus_flavipe CCTAATAATA AA-ATTAGTG GAG---GTGA TAAAATTCCA -GCTGAACTA TTTAAAAA-- ----TCTTAA 1133
BovB_Ac-RTE-Anolis_carolinensi GCTAACAACA AG-GCAGCAG GAG---ACGA CGGGATCCCA GCT-GAGCTG TTTAAA---- ---ATCTTGG 1767
BovB_BM-RTE-Bombyx_mori AAGGATAATA AA-GCGCCAG GAC---ATGA TAACATCACA GCA-GAGAC- TTTAAAG--- -----CACTC 1631
BovB_Dr-RTE-Christinus CCAAACAGGA AA-GCCCCAG GCA---TTGA TGGCATACCA ACT-GAATT- ACTCAAA--- -----C---C 1668
BovB_La-RTE-Loxodonta_africana GCGAAAAAAA AG-GCTCCAG GAA---TTGA CGGAATATCA ATT-GAGATG TTTCAAC--- -----AAACG 1795
BovB_Pc-RTE-Procavia_capensis GCAAAAAACA AG-GCTCCAG GAA---TTGA TGGAATATCA ATT-GAGATA TTTCAAC--- -----AAACA 1795
BovB_Et-RTE-Christinus TCCAAAAACA AG-CCTCAAG GAA---TTGA TGAAATGCCC ATT-GAAATG TTTCAAC--- -----AAAAA 1596
BovB_Ta-RTE-Tachyglossus_acule ACCAAAAATA AG-GCACCTG GCT---TTGA TAGGCTTCCT ATT-GAGGTG TTTCAGG--- -----CAGCT 1932
BovB_Plat-RTE-Ornithorhynchus GCCAAAAATA AG-GCACCTG GCT---TTGA TAGGCTTCCT ATT-GAGGTG TTTCAGG--- -----CAGCT 1921
BovB_Ec-RTE-Equus_caballus GGAAGAAATA AG-TCACCAG GGG---TAGA TGGGATATCG ATA-GAATTA TTTCAAG--- -----CCACA 1691
BovB_Cg-RTE-Christinus G-AAGAATTA TA-ACAAAAG AGA---AAAA TAGGATATCA ATA-GAGCTA TTTCAAG--- -----CCACA 1211
BovB_Bh-RTE-Bothriocroton_hydr GCCATCTAGG ATTACCCAAG ATG---GACA GGTCATAGTA GAGAGTTCTG ACTAAACGT- --GATCCGCT 248
BovB_Ml-RTE-Gehyra_variegata TAAAGAAATA TAATATTTTG GAT------A GGAGGTAGCA AAAAAAAATG TTACAA---- ---------A 333
Sly-rnd1_family284-LINE/RTEBov ---------- ---------- ---------- ---------- ---------- ---------- ---------- -
Sly-rnd5_family2019-LINE/RTEBo ---------- ---------- ---------- ---------- ---------- ---------- ---------- -
Sly-rnd3_family322-LINE/RTEBov ---------- ---------- ---------- ---------- ---------- ---------- ---------- -
Sly-rnd5_family1698-LINE/RTEBo ---------- ---------- ---------- ---------- ---------- ---------- ---------- -
Sly-rnd1_family795-LINE/RTEBov ---------- ---------- ---------- ---------- ---------- ---------- ---------- -
Stu-rnd-5_family-3162_LINE_RTE CGGAGGTGGA GCTTCGCTTC TGGATTTTGC TAAAGCTTTT GAGTTGGTGA TNGCTAACTC GAGCTTTCCG 1467
Sly-rnd6_family1960-LINE/RTEBo CGGAGG---A GTTTCGCTTT TGGATTTTGC GAAAGCTTTT GAGTTGGTGA TTGCTAACTC GAGCTTCCCG 641
Stu-rnd-5_family-2493_LINE_RTE ---------- ---------- ---------- ---------- ---------- ---------- ---------- -
Sly-rnd5_family447-LINE/RTEBov ---------- ---------- ---------- ---------- ---------- ---------- ---------- -
Stu-rnd-6_family-2093_LINE_RTE ---------- ---------- ---------- ---------- ---------- ---------- ---------- -
Consensus -C-AA-AA-A A--GC----G G------TGA ----AT-CCA ----GAG-T- TT--AA---- -----C---A
100%
Conservation
0%

```
                                   2,680                2,700                2,720
Stu-rnd-1_family-417_LINE_RTE-     ---------- ---------- ---------- ---------- ---------- ---------- ---------- -
Sly-rnd1_family377-LINE/RTEBov     ---------- ---------- ---------- ---------- ---------- ---------- ---------- -
Stu-rnd-1_family-489_LINE_RTE-     ---------- ---------- ---------- ---------- ---------- ---------- ---------- -
Stu-rnd-1_family-607_LINE_RTE-     ---------- ---------- ---------- ---------- ---------- ---------- ---------- -
Stu-rnd-1_family-645_LINE_RTE-     ---------- ---------- ---------- ---------- ---------- ---------- ---------- -
Sly-rnd4_family81-LINE/RTEBovB     ---------- ---------- ---------- ---------- ---------- ---------- ---------- -
Stu-rnd-6_family-408_LINE_RTE-     ---------- ---------- ---------- ---------- ---------- ---------- ---------- -
Stu-rnd-1_family-351_LINE_RTE-     ---------- ---------- ---------- ---------- ---------- ---------- ---------- -
Stu-rnd-1_family-382_LINE_RTE-     ---------- ---------- ---------- ---------- ---------- ---------- ---------- -
Sly-rnd6_family746-LINE/RTEBov     ---------- ---------- ---------- ---------- ---------- ---------- ---------- -
Stu-rnd-5_family-325_LINE_RTE-     ---------- ---------- ---------- ---------- ---------- ---------- ---------- -
Sly-rnd4_family509-LINE/RTEBov     ---------- ---------- ---------- ---------- ---------- ---------- ---------- -
Stu-rnd-1_family-513_LINE_RTE-     ---------- ---------- ---------- ---------- ---------- ---------- ---------- -
Sly-rnd5_family1442-LINE/RTEBo     ---------- ---------- ---------- ---------- ---------- ---------- ---------- -
BovB_Gl-RTE-Gehyra_lazelli         AAGA-GGACG CCGTTAAAGT GCTGCACTCA ATATGCCAGC AAATTTGGAA AACGCAACAG TGGCCACA-- 2009
BovB_Gv-RTE-Gehyra_variegata       AACA-TGATG CCGTTAAAGT GCTGCACTCA ATATGCCAGC AAATTTGGAA AACGCAACAG TGGCCACA-- 1939
BovB_PMo-RTE-Python_molurus        AAGA-TGATG CTGTTAAAGT GCTGCACTCA ATATGCCAGC AAGTTTGGAA AACGCAACAG TGGCCACG-- 1871
BovB_Oa-RTE-Ovis_aries             AAGA-TGATG CTGTGAAAGT GCTGCACTCA ATATGCCAGC AAATTTGGAA AACTCAGCAG TGGCCACA-- 1897
BovB-RTE-Bos_taurus                AAGA-TGATG CTGTGAAAGT GCTGCGCTCA ATATGCCAGC AAATTTGGAA AACTCAGCAG TGGCCACA-- 2495
BovB_Me-RTE-Macropus_eugenii       AAAA-T---- ---------- ------CTTA ACAT------ ---------- ----CACTGA TAATCACA-- 1560
BovB_Ano-RTE-Amphibolurus_norr     AAGA-TGATG CTGTTAAGGT GCTACATTCA ATATGCCAGC AAGTTTGGAA AACTCAACAG TGGCCAGA-- 1944
BovB_Gd-RTE-Eremiascincus_doug     AAGA-TGATG CTGTCAAAGT GCTGCACTCC ATATGCCAGC AAATTTGGAA AACCCAGCAG TGGCCACA-- 1532
BovB_Er-RTE-Eremiascincus_rich     AAGA-TGATG CTGTCAAAGT GCTGCACTCC ATATGCCAGC AAATTTGGAA AACCCAGCAG TGGCCACA-- 2067
BovB_Ca-RTE-Ctenotus_atlas         AAGA-TGATG CTGTCAAAGT GCTGCACTCC ATATGCCAGC AAATTTGGAA AACCCAACAG TGGCCGCA-- 2060
BovB_Es-RTE-Egernia_stokesii       AAGA-TGATG CTGTTAAAGT GCTGCACTCA ATCTGCCAGC AAATTGGGAA AACTCAACAG TGGCCTCA-- 1894
BovB_Tr-RTE-Tiliqua_rugosa         AAGA-TGAAG CTGTTAAAGT GCTGCACTCA ATCTGCCAGC AAAGTTGGAA AACTCAACAG TGGCCTCA-- 1900
BovB_ALi-RTE-Amblyomma_limbatu     AAGA-CGATG CAGTAAAAGT GCTACACTCA ATATGCCAGC AAATTTGGAA AACTCAACAG TTGCCACACG 2053
BovB_Hs-RTE-Hydrophis_spiralis     AACA-CGATG CAGTAAAAGT GCT-CACTCA ATTTGCCAGC AAATTTGGAA AACTCAACAG TGGCCACA-- 275
BovB_VA-RTE-Vipera_ammodytes       AAGA-CAATG CAGTAAAAGT GCTACACTCA ATTTGCCAGC AAATTTGGAA AACTCAACAG TGGCCACA-- 1865
BovB_ACo-RTE-Agkistrodon_conto     AAGA-CGATG CAGTAAAAGT GCTACACTCA ATTTGCCAGC AAATTTGGAA AACTCAACAG TGGCCACA-- 1865
BovB_Pg-RTE-Perameles_gunnii       ---------- ---------- --------AT GTT------C AAATTTGGAA AACTCAACAG TAGCCACT-- 34
BovB_Io-RTE-Isoodon_obesulus       AAGA-TGATA CTGTTAAAGT ATTGCACTTT AAA------C TAATTTGGAA AACTAAACAG TGGTCACT-- 472
BovB_Bp-RTE-Burramys_parvus        AAGA-TGATG CTGTTAAAGT GCTGTACTCA ATATGCCAGC AAATTTGGAA AACTCAACAG TGGCCACT-- 564
BovB_Pb-RTE-Petaurus_breviceps     AAGA-TGATG CTGTTAAAGT GCTGCACTCA ATATGCCAGC AAATTTGGAA AACTCAACAG TGACCACT-- 630
BovB_Ma-RTE-Macropus_antilopin     AAGA-TGATG CTGTTAAAGT GCTGCACTCA ATATGCCAGC AAATTTGGAA AACTCAACAG TGGCCACT-- 1979
BovB_Af-RTE-Antechinus_flavipe     AAGAATGATG CTGTCAAAGA GCTGCATTTA ATATGTTAAA AAATTTAGAA AACTTAACAG TGACCACT-- 1201
BovB_Ac-RTE-Anolis_carolinensi     AAGA-TCATG CTGTCAAGCT GATGCATGCA ATATGCCAGC AAATATGGAA AACACAAAAA TGGCCATC-- 1834
BovB_BM-RTE-Bombyx_mori            AGCGACGCAG GAATAGATGT ACTGCATAAA ATCTGCAATC ATATCTGGCA GAATGGTGAA TGGCCGGA-- 1699
BovB_Dr-RTE-Christinus             AATTCCG--- --ATTCATGT ATTAACAACG CTGTGTCGAC AGATATGGAA GACCAAATCA TGGCCTAA-- 1731
BovB_La-RTE-Loxodonta_africana     GATGCAGCGC TGG---AAGT GCTCACTCGT CTATGCCAAG AAATATGGAA GACAGCTACC TGGCCAAC-- 1860
BovB_Pc-RTE-Procavia_capensis      GATGCAGCAC TGG---AAGT GCTCACTCAT CTATGCCAAG AAATTTGGAG GACAGCTACC TGGCCAAC-- 1860
BovB_Et-RTE-Christinus             GA---AGCAC TGG---AAGC ACTCACTTGT CTATACCAGG AAATTTGGAA GACAGGCACT TGGCCAAG-- 1658
BovB_Ta-RTE-Tachyglossus_acule     GAAGAGGAAT CGGTTAAAGT CCTTACCAAA TTATGTCAAC AGGTATGGCT AACCACTCAA TGGCCATC-- 2000
BovB_Plat-RTE-Ornithorhynchus      GGAGAGGAAT CGGTTAAAGT CCTTACCAAA TTATGTCAAC AGGTATGGCT AACCACTCAA TGGCCATC-- 1989
BovB_Ec-RTE-Equus_caballus         GAGACTGAAT CTGTCAAAAT CCTAACAAGA ATATGCCAAC AAATATGGAA AACAAAACAA TGGCCTAC-- 1759
BovB_Cg-RTE-Christinus             GAAATAGAAT ATATCAAAAT CTTAACAAGA ATATGCCAAC AAAAATGGAA AACAAAACAA TGGCCTAT-- 1279
BovB_Bh-RTE-Bothriocroton_hydr     GGAGAAGGAA ATGGCAATCC ACTCCAGTAG TCCTGCCAA- ------GAAA ACCCGATGAA TTGCAGAACT 311
BovB_Ml-RTE-Gehyra_variegata       GAAA------ -----AGAGC AGGAACACAA ACTGGCTAAC TAAT---GAG GCTTTACAAA TAGCTGAG-- 387
Sly-rnd1_family284-LINE/RTEBov     ---------- ---------- ---------- ---------- ---------- ---------- ---------- -
Sly-rnd5_family2019-LINE/RTEBo     ---------- ---------- ---------- ---------- ---------- ---------- ---------- -
Sly-rnd3_family322-LINE/RTEBov     ---------- ---------- ---------- ---------- ---------- ---------- ---------- -
Sly-rnd5_family1698-LINE/RTEBo     ---------- ---------- ---------- ---------- ---------- ---------- ---------- -
Sly-rnd1_family795-LINE/RTEBov     ---------- ---------- ---------- ---------- ---------- ---------- ---------- -
Stu-rnd-5_family-3162_LINE_RTE     AAGAAGGAGG ACCACTTGGT NACCTTTCGT AGCNCGGTGG CTAAGACCCA GATAGATTAC TTACTCCTTA 1537
Sly-rnd6_family1960-LINE/RTEBo     A---AGGAGG ATCACTTGGT TACCTTCCGT AGCACGGTGG NTAAGACTCA NATAGATTAC TTACTCCTTA 708
Stu-rnd-5_family-2493_LINE_RTE     ---------- ---------- ---------- ---------- ---------- ---------- ---------- -
Sly-rnd5_family447-LINE/RTEBov     ---------- ---------- ---------- ---------- ---------- ---------- ---------- -
Stu-rnd-6_family-2093_LINE_RTE     ---------- ---------- ---------- ---------- ---------- ---------- ---------- -

Consensus                          AA-A--GA-- --G--AAAGT -CT----T-A ATATGCCA-C AAAT-TGGAA AAC--A-CA- TGGCCAC---
```

Conservation

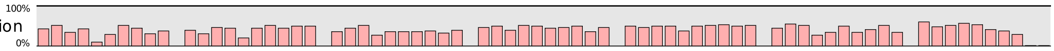

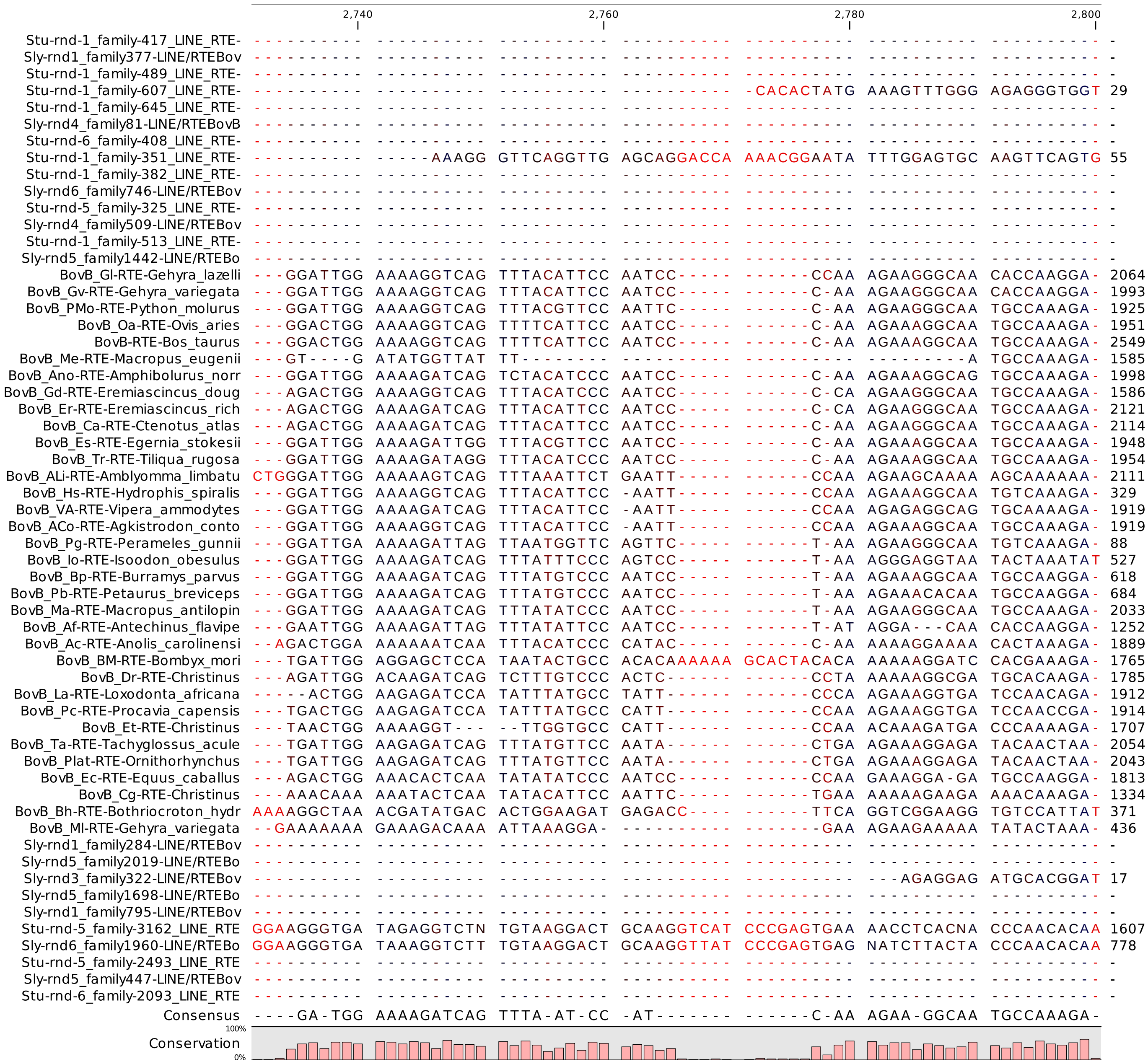
2,740
2,760
2,780
2,800
Stu-rnd-1_family-417_LINE_RTE- -
Sly-rnd1_family377-LINE/RTEBov -
Stu-rnd-1_family-489_LINE_RTE- -
Stu-rnd-1_family-607_LINE_RTE- 29
Stu-rnd-1_family-645_LINE_RTE- -
Sly-rnd4_family81-LINE/RTEBovB -
Stu-rnd-6_family-408_LINE_RTE- -
Stu-rnd-1_family-351_LINE_RTE- 55
Stu-rnd-1_family-382_LINE_RTE- -
Sly-rnd6_family746-LINE/RTEBov -
Stu-rnd-5_family-325_LINE_RTE- -
Sly-rnd4_family509-LINE/RTEBov -
Stu-rnd-1_family-513_LINE_RTE- -
Sly-rnd5_family1442-LINE/RTEBo -
BovB_Gl-RTE-Gehyra_lazelli 2064
BovB_Gv-RTE-Gehyra_variegata 1993
BovB_PMo-RTE-Python_molurus 1925
BovB_Oa-RTE-Ovis_aries 1951
BovB-RTE-Bos_taurus 2549
BovB_Me-RTE-Macropus_eugenii 1585
BovB_Ano-RTE-Amphibolurus_norr 1998
BovB_Gd-RTE-Eremiascincus_doug 1586
BovB_Er-RTE-Eremiascincus_rich 2121
BovB_Ca-RTE-Ctenotus_atlas 2114
BovB_Es-RTE-Egernia_stokesii 1948
BovB_Tr-RTE-Tiliqua_rugosa 1954
BovB_ALi-RTE-Amblyomma_limbatu 2111
BovB_Hs-RTE-Hydrophis_spiralis 329
BovB_VA-RTE-Vipera_ammodytes 1919
BovB_ACo-RTE-Agkistrodon_conto 1919
BovB_Pg-RTE-Perameles_gunnii 88
BovB_Io-RTE-Isoodon_obesulus 527
BovB_Bp-RTE-Burramys_parvus 618
BovB_Pb-RTE-Petaurus_breviceps 684
BovB_Ma-RTE-Macropus_antilopin 2033
BovB_Af-RTE-Antechinus_flavipe 1252
BovB_Ac-RTE-Anolis_carolinensi 1889
BovB_BM-RTE-Bombyx_mori 1765
BovB_Dr-RTE-Christinus 1785
BovB_La-RTE-Loxodonta_africana 1912
BovB_Pc-RTE-Procavia_capensis 1914
BovB_Et-RTE-Christinus 1707
BovB_Ta-RTE-Tachyglossus_acule 2054
BovB_Plat-RTE-Ornithorhynchus 2043
BovB_Ec-RTE-Equus_caballus 1813
BovB_Cg-RTE-Christinus 1334
BovB_Bh-RTE-Bothriocroton_hydr 371
BovB_Ml-RTE-Gehyra_variegata 436
Sly-rnd1_family284-LINE/RTEBov -
Sly-rnd5_family2019-LINE/RTEBo -
Sly-rnd3_family322-LINE/RTEBov 17
Sly-rnd5_family1698-LINE/RTEBo -
Sly-rnd1_family795-LINE/RTEBov -
Stu-rnd-5_family-3162_LINE_RTE 1607
Sly-rnd6_family1960-LINE/RTEBo 778
Stu-rnd-5_family-2493_LINE_RTE -
Sly-rnd5_family447-LINE/RTEBov -
Stu-rnd-6_family-2093_LINE_RTE -
Consensus ----GA-TGG AAAAGATCAG TTTA-AT-CC -AT------- ------C-AA AGAA-GGCAA TGCCAAAGA-
Conservation
100%
0%

	2,820		2,840		2,860			
Stu-rnd-1_family-417_LINE_RTE-	----------	----------	----------	----------	----------	----------	----------	-
Sly-rnd1_family377-LINE/RTEBov	----------	----------	----------	----------	----------	----------	----------	-
Stu-rnd-1_family-489_LINE_RTE-	----------	----------	----------	----------	----------	----------	----------	-
Stu-rnd-1_family-607_LINE_RTE-	GGAGATGAGG	GTGAGGAGAG	GGGTGTCCAT	TTCCGAGAAC	CAGTTCGGAT	TCATGTCGGG	GCGNTCGACT	99
Stu-rnd-1_family-645_LINE_RTE-	----------	----------	----------	----------	----------	----------	----------	-
Sly-rnd4_family81-LINE/RTEBovB	----------	----------	----------	----------	----------	----------	----------	-
Stu-rnd-6_family-408_LINE_RTE-	----------	----------	----------	----------	----------	----------	----------	-
Stu-rnd-1_family-351_LINE_RTE-	ACGCGACGCA	TGAGGCGGAC	GTGGAAGTGA	GGCTTGACGC	ACAGGTCATC	CCCAAGAGAG	AAAGTTTCAA	125
Stu-rnd-1_family-382_LINE_RTE-	----------	----------	----------	----------	----------	----------	----------	-
Sly-rnd6_family746-LINE/RTEBov	----------	----------	----------	----------	----------	----------	----------	-
Stu-rnd-5_family-325_LINE_RTE-	----------	----------	----------	----------	----------	----------	----------	-
Sly-rnd4_family509-LINE/RTEBov	----------	----------	----------	----------	----------	----------	----------	-
Stu-rnd-1_family-513_LINE_RTE-	----------	----------	----------	----------	----------	----------	----------	-
Sly-rnd5_family1442-LINE/RTEBo	----------	----------	----------	----------	----------	----------	----------	-
BovB_Gl-RTE-Gehyra_lazelli	----ATGTTC	CAACTATCGC	A-CAATTGCA	CTCATTTCAC	ACGCTAGCAA	GGTCATGCTC	AA---GAT-C	2125
BovB_Gv-RTE-Gehyra_variegata	----ATGTTC	CAACTATTGC	A-CAATTGCA	CTCATTTCAC	ATGCTAGCAA	GGTCATGCTC	AA---GAT-C	2054
BovB_PMo-RTE-Python_molurus	----ATGCTC	AAACTATCGC	A-CAATTGCA	CTCATTTCAC	ATGCTAGCAA	GGTTATGCTC	AA---AAT-C	1986
BovB_Oa-RTE-Ovis_aries	----ATGTTC	AAACTACCAC	A-CAATTGCA	CTCATCTCAC	ATGCTAGCAA	AGTAATGCTC	AA---AAT-C	2012
BovB-RTE-Bos_taurus	----ATGCTC	AAACTACCGC	A-CAATTGCA	CTCATCTCAC	ACGCTAGTAA	AGTAATGCTC	AA---AAT-T	2610
BovB_Me-RTE-Macropus_eugenii	----ATCTTC	CAGGTACAAA	AACAAAAACA	CTCACTTCAC	ATGCCAAGAA	AGCTATGCTT	AA---GAT-T	1647
BovB_Ano-RTE-Amphibolurus_norr	----ATGCTC	CAACTACCGT	A-CAATTGCA	CTCATTTCAC	ACGCTAGCAA	GGTTATGCTC	AA---AAT-C	2059
BovB_Gd-RTE-Eremiascincus_doug	----TTGTTC	CAACTACCGC	A-CCATTGCA	CTCATTTCAC	ATGCTAGCAA	GGTGATGCTC	AA---AAT-C	1647
BovB_Er-RTE-Eremiascincus_rich	----TTGTTC	CAACTACCGC	A-CCATTGCA	CTCATTTCAC	ATGCTAGCAA	GGTGATGCTC	AA---AAT-C	2182
BovB_Ca-RTE-Ctenotus_atlas	----TTGTTC	GAACTACCGC	A-CCATTGCA	CTCATTTCAC	ATGCTAGCAA	GGTGATGCTC	AA---AAT-C	2175
BovB_Es-RTE-Egernia_stokesii	----AAGTTC	AAACCACCGC	G-CAATTGCG	CTTATTTCAC	ATGCTAGCAA	GGTTATGCTC	AA---AAT-C	2009
BovB_Tr-RTE-Tiliqua_rugosa	----ATGTTC	AAACCACTGC	A-CAACTGCG	CTTATTTCAC	ATGCTAGCAA	AGTTATGCAC	AA---AATAC	2016
BovB_ALi-RTE-Amblyomma_limbatu	----TTGTTC	AAACTACCGG	A-CCATTACA	CTAATTTTAC	ATACTAGCAA	AGTAATGCTC	AACTGAAT-C	2175
BovB_Hs-RTE-Hydrophis_spiralis	----ATGTTC	AAATTATTGC	A-CCATTGCA	CTCATTTCAC	ATGCTAGAAA	AGTTGTGCTG	AA---AAT-C	390
BovB_VA-RTE-Vipera_ammodytes	----ATGTTC	AAACTATCGC	A-CCATTGCA	CTCATTTCTC	ATGCTAGTAA	AGTTATGCTT	AA---AAT-T	1980
BovB_ACo-RTE-Agkistrodon_conto	----ATGTTC	AAACTATCGC	A-CCATTGCA	CTCATTTCAC	ATGCTAGTAA	AGTTATGCTT	AA---AAT-C	1980
BovB_Pg-RTE-Perameles_gunnii	----ATGTTT	AAATTAATGA	A-CTATTG--	---------T	GCTC-----A	TTTCATGCTT	AA---GAT-T	133
BovB_Io-RTE-Isoodon_obesulus	AACTGTGCTT	ATTTCAAGAA	G-TTCATGC-	-----TTAAT	ACCTTAG-AA	GTTTATACT-	AA---GAT-A	584
BovB_Bp-RTE-Burramys_parvus	----ATGTTC	AAATTACTGA	A-CAATTACA	CTCATTTCAC	ATGCCAGCAA	GGTTATGCTT	AA---GAT-T	679
BovB_Pb-RTE-Petaurus_breviceps	----ATGCTC	AAATTACTAA	A-CAATTGTA	CTCATTTCAC	ATGCCAGCAA	GGTTATGCTT	AA---GAT-T	745
BovB_Ma-RTE-Macropus_antilopin	----ATGTTC	AAATTACTGA	A-CAATTGTA	CTCATTTCAC	ATGCCAGCAA	GGTTATGCTT	AA---GAT-T	2094
BovB_Af-RTE-Antechinus_flavipe	----ATGTTC	ACATTATCCA	A-CAATTGTG	CTCATTTCAC	ACATCAGCAA	AATTATATTT	AA---GAT-T	1313
BovB_Ac-RTE-Anolis_carolinensi	----ATGCTC	AAACTTCCGT	A-CAGTGGCA	CTTATTTCCC	ATGCCAGTAA	GGTAATGCTC	AA---GAT-C	1950
BovB_BM-RTE-Bombyx_mori	----CTGTAA	CAACTATCGG	A-CCCTTGCT	CTTATCTCAC	ATGCAAGTAA	AATCCTTCTG	CA---TAT-T	1826
BovB_Dr-RTE-Christinus	----ATGCTG	CAACTATCAG	A-CAATCGCG	CTCATACCAC	ACGCAAGCAA	AATCCTGCTG	AA---GATAT	1847
BovB_La-RTE-Loxodonta_africana	----ATGCGG	AAATTATAGA	A-CAATATCA	TTAATATCAC	ACGCAAGCAA	AATTTTGCTG	AA---GATCA	1974
BovB_Pc-RTE-Procavia_capensis	----ATGTGG	AAATTACCGA	A-CAATATCA	TTAATATCAC	ACGCAAGTAA	AATTTTGCTC	AA---GATCA	1976
BovB_Et-RTE-Christinus	----ATA---	--------GA	A-CAATATCC	TTA-TATCAC	ATGCAAGCAA	AATGTTGCTG	AA---GATCA	1757
BovB_Ta-RTE-Tachyglossus_acule	----ATGTGA	CAATTATCGT	A-CAATTTCC	TTGATCTCAC	ATGCCAGCAA	GGTATTGCTA	AA---AGTTA	2116
BovB_Plat-RTE-Ornithorhynchus	----ATGCGA	CAATTATCGT	A-CAGTTTCC	TTGATCTCAC	ATGCCAGCAA	GGTACTGCTA	AA---AGTTA	2105
BovB_Ec-RTE-Equus_caballus	----GTGCAG	TAACTATAGG	A-CCATTGCT	CTAATTTCCC	ATGCAAGTAA	AGTGATGCTC	AA---GGTGC	1875
BovB_Cg-RTE-Christinus	----CTGCAG	CAACTATCAG	A-CCATTGCA	TTAATTTCAC	ATGTAAGTAA	AGTGATGCTC	AA---AATCT	1396
BovB_Bh-RTE-Bothriocroton_hydr	GCTACTGGGG	GAAGAGCAGA	G-CACGAGTA	CTGA---CAC	ATCCAGAAAG	AGCGAAGTAG	GT--GGGCCA	435
BovB_Ml-RTE-Gehyra_variegata	----TTG---	--AATGTAGA	ATTAAGTTC-	TTAATATCAC	TGATAAGCAT	GATGGTGTGA	TT---ACTGA	493
Sly-rnd1_family284-LINE/RTEBov	----------	----------	----------	----------	----------	----------	----------	-
Sly-rnd5_family2019-LINE/RTEBo	----------	----------	----------	----------	----------	----------	----------	-
Sly-rnd3_family322-LINE/RTEBov	GCCCCAATGA	GGAGGTGCGA	GANGTTGGCT	ATGGATGGAT	ATCAGGAGAG	GTAGCGTAGC	GCNAGCGNCA	87
Sly-rnd5_family1698-LINE/RTEBo	----------	----------	----------	----------	----------	----------	----------	-
Sly-rnd1_family795-LINE/RTEBov	----------	----------	----------	----------	----------	----------	----------	-
Stu-rnd-5_family-3162_LINE_RTE	GCTTTTGGTG	ATGGACCTGG	AGATTAAGAG	GGAGAGGAGG	AAGAGGATTT	TATATGACCG	ATCGAGGATT	1677
Sly-rnd6_family1960-LINE/RTEBo	GCTCNTTGCG	ATGGATTTGG	AGATTAAGAG	GGAGATGAGG	ANGAGGACCA	TNTATGACCG	ACCGAGGATT	848
Stu-rnd-5_family-2493_LINE_RTE	----------	----------	----------	----------	----------	----------	----------	-
Sly-rnd5_family447-LINE/RTEBov	----------	----------	----------	----------	----------	----------	----------	-
Stu-rnd-6_family-2093_LINE_RTE	----------	----------	----------	----------	----------	----------	----------	-
Consensus	----ATGTTC	-AA-TA-CG-	A-CAATTGCA	CT-ATTTCAC	ATGC-AGCAA	-GT-ATGCT-	AA----AT--	

Conservation

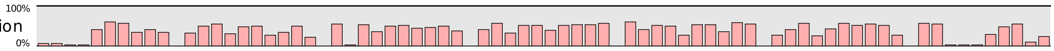

```
                                     2,880                     2,900                     2,920                     2,940
Stu-rnd-1_family-417_LINE_RTE-       ---------- ---------- ---------- ---------- ---------- ---------- ---------- -
Sly-rnd1_family377-LINE/RTEBov       ---------- ---------- ---------- ---------- ---------- ---------- ---------- -
Stu-rnd-1_family-489_LINE_RTE-       ---------- ---------- ---------- ---------- ---------- ---------- ---------- -
Stu-rnd-1_family-607_LINE_RTE-       ACAGAAGCCA TTCATCTCGT GAGGAGATTG GTGGAGAAAT ATAGAGANAG GAAGAGGGAC TTACATATGG 169
Stu-rnd-1_family-645_LINE_RTE-       ---------- ---------- ---------- ---------- ---------- ---------- ---------- -
Sly-rnd4_family81-LINE/RTEBovB       ---------- ---------- ---------- ---------- ---------- ----GGACGT TCAACTAATA 16
Stu-rnd-6_family-408_LINE_RTE-       ---------- ---------- ---------- ---------- ---------- ---------- ---------- -
Stu-rnd-1_family-351_LINE_RTE-       GTATCTTGGG TCTATAATCC AAGGAAATGG GGAGATCGAC GATGATGTCA CACATCGTAT TGGNGCGGCG 195
Stu-rnd-1_family-382_LINE_RTE-       ---------- ---------- ---------- ---------- ---------- ---------- ---------- -
Sly-rnd6_family746-LINE/RTEBov       ---------- ---------- ---------- ---------- ---------- ---------- ---------- -
Stu-rnd-5_family-325_LINE_RTE-       ---------- ---------- ---------- ---------- ---------- ---------- ---------- -
Sly-rnd4_family509-LINE/RTEBov       ---------- ---------- ---------- ---------- ---------- ---------- --AGATCGGA 8
Stu-rnd-1_family-513_LINE_RTE-       ---------- ---------- ---------- ---------- ---------- ---------- ---------- -
Sly-rnd5_family1442-LINE/RTEBo       ---------- ---------- ---------- ---------- ---------- ---------- ---------- -
BovB_Gl-RTE-Gehyra_lazelli           CTACAAGCTA GGCT-TCAGC AGTATGTGGA CAGA-GAATT GCCAGAAGTA CAAGCTGGGT TTCGAAGAGG 2193
BovB_Gv-RTE-Gehyra_variegata         CTACAAGCTA GGCT-TCAGC AGTACGTGGA CAGA-GAACT GCCAGAAGTA CAAGCTGGGT TTCGAAGAGG 2122
BovB_PMo-RTE-Python_molurus          CTACAAGCTA GGCT-TCAGC AGTACGTGAA CCGA-GAACT ACCGGATGTA CAAGCTGGGT TTCGAAGAGG 2054
BovB_Oa-RTE-Ovis_aries               CTCCAAGCTA GGCT-TCAAC AGTATGTGAA CCGA-GAACT TCCAGATGTT CAAGCTGGAT TTAGAAAAGG 2080
BovB-RTE-Bos_taurus                  CTCCAAGCCA GGCT-TCAGC AATACGTGAA CCGT-GAACT TCCTGATGTT CAAGCTGGTT TTAGAAAAGG 2678
BovB_Me-RTE-Macropus_eugenii         CTC-AAGCAA GGCT-TCAAC AATATGTAAA CTGC-GAATT AGCAGA---- -GAATTATCA GAAAAGCAGG 1709
BovB_Ano-RTE-Amphibolurus_norr       CTGCAAGGTA GGCT-TCAGC AGTATGTGGA CCGA-GAACT CCCAGAAGTA CAAGCTGGAT TCCGAAGGGG 2127
BovB_Gd-RTE-Eremiascincus_doug       CTACAAGCCA GGCT-TCAGC AGTATGTGAA TCGA-GAACT ACCAGATGTA CAAGCTGGTT TCCGAAGAGG 1715
BovB_Er-RTE-Eremiascincus_rich       CTACAAGCCA GGCT-TCAGC AGTATGTGAA TCGA-GAACT ACCAGATGTA CAAGCTGGTT TCCGAAGAGG 2250
BovB_Ca-RTE-Ctenotus_atlas           CTACAAGCTA GGCT-TCAGC AGTATGTCAA TCGA-GAACT ACCAGATGTA CAAGCTGGTT TCCGAAGAGG 2243
BovB_Es-RTE-Egernia_stokesii         CTACAAGCTA GGCT-TCAGC AGTATGTGAA CCGA-GAACT ACCAGAAGTA CAAGCTGGTT TTTGAAGAGG 2077
BovB_Tr-RTE-Tiliqua_rugosa           CTACAAGCCA GGCT-TCAGC AGTATGTGAA CAAA-GAACT ACCAGAAGTA AAAGATGGTT TCCGAAGAGG 2084
BovB_ALi-RTE-Amblyomma_limbatu       CTACAATCTA GACT-CCAGC AGTATATGGA TCAA-GAACT ATCTGAAGTT CAGGCATGAA TTCGAAAAGG 2243
BovB_Hs-RTE-Hydrophis_spiralis       CTACAAGCTA GGCT-CCAGC AGTATGTAGA TCAA-GAACT ACCAGAAGTA CAGGCAGGAT TTCAAAGAAG 458
BovB_VA-RTE-Vipera_ammodytes         CTACAAGCTA GACT-CCAGC AATATGTGGA TCGA-GAACT ACCAGAAGTA CAGGCAGGAT TTCGAAGAGG 2048
BovB_ACo-RTE-Agkistrodon_conto       CTACAAGCTA GACT-CCAGC AGTATGTGGA TCGA-GAACT ACCAGAAGTA CAGGCAGGAT TTCGAAGAGG 2048
BovB_Pg-RTE-Perameles_gunnii         CTGAAACCTA GGCT-TAAGC AATAT--AAA CCAA-GAATT ATCAGAAGAG CAGGGTGGTT TTTGAAGAGG 199
BovB_Io-RTE-Isoodon_obesulus         CTGTAAATTA ---T-TCAGT AATATCTGAA TTGA-GAATT ACCAGAAGAC CACTCTAGTT TTCAAAGGAG 649
BovB_Bp-RTE-Burramys_parvus          CTGCAAGCTA GGCT-TCAGC AATATGTGAA CTGA-GAATT ACCATAAGAG CAAGATGGTT TTCAAGG-GT 746
BovB_Pb-RTE-Petaurus_breviceps       CTGCAAGCTA GGCT-TCAGC AATATGTAAA ATGA-GAATT ACCAGAAGAG CAGGCTGGTT TTCAAAGAGG 813
BovB_Ma-RTE-Macropus_antilopin       CTGCAAGCTA GGCT-TCAGC AATATGTGAA CCGA-GAATT ACCAGAAGAG CAGGCTGGTT TTCAAAGAGG 2162
BovB_Af-RTE-Antechinus_flavipe       CTGCATGCTA GGCTCTCAAC AAT-----AA CTGA-AAATT ATCAGAAAAA AAGACTGATT TTAAAAGAAA 1377
BovB_Ac-RTE-Anolis_carolinensi       CTGCAAGAAA GACT-CCAGC AATACATGGA GCGA-GAGTT GCCAGATGTC CAAGCTGGGT TCAGAAAAGA 2018
BovB_BM-RTE-Bombyx_mori              ATCAATTCTA GAAT-CCGAC ACTTTCTGGA TTGG-CAAAT CCCTCAGGAA CAGGCTGGTT TTGTCAAAGG 1894
BovB_Dr-RTE-Christinus               TTCAACAACG CATG-GCCTC A-TACATCGA GAGG-GAGCT TCCTGACGTC CAAGCCGGTT TTCACAAAGG 1914
BovB_La-RTE-Loxodonta_africana       TTCAAAAAAC GGCT-GCAGC AGTATATC-A CAGG-GAACT GCCAGAAATT CAGGCCGGAT TCAGAAGAGG 2041
BovB_Pc-RTE-Procavia_capensis        TTCAAAAG-T GACT-GCAAC AGTATATTGA CAAG-GAACT GCCAGAAATT CAAGCTGGAT TCAGAAGAGG 2043
BovB_Et-RTE-Christinus               TCCAACAA-A GGTT-GTAGC AGTACATTAA CAGG-AAACT GCCAAAGGTT CAGGCCAGAT TCAGAAGA-- 1822
BovB_Ta-RTE-Tachyglossus_acule       TACAGCAT-C GAAT-GGAGA CCTACATGGA ACAT-GAATT GCCCAATGTT CAAGCAGGAT TCCAGAAAGG 2183
BovB_Plat-RTE-Ornithorhynchus        TACAGCGT-C GAAT-GGAGA CCTACATGGA ACGC-GAATT GCCCGATGTT CAAGCAGGAT TCCGGAAAGG 2172
BovB_Ec-RTE-Equus_caballus           TGCAACAA-A GGCT-TTTAC CTTATATGGA GCAA-GAAAT GCCTGATGTC CAAGCTGGAT TTCGAAAAGG 1942
BovB_Cg-RTE-Christinus               TACAACAA-A GACT-GTTAC CATATATGGA ATGA-GAAAT GCCAGATGTT CAAACTGGAT TCAGAAAAGG 1463
BovB_Bh-RTE-Bothriocroton_hydr       AAACCGGAAG GAAGCTCAGC TGTGGATGTG TAAG-GAAGT GAAAGGAAAC TACGATGCTC T---AAAGAG 501
BovB_Ml-RTE-Gehyra_variegata         TCTAGAGTCA TACCTCCTGG AGAATAAAGT CAAGTGGACT GTGGAAAGTA TTGATAAAAA T----AAGAG 559
Sly-rnd1_family284-LINE/RTEBov       ---------- ---------- ---------- ---------- ---------- ---------- ---------- -
Sly-rnd5_family2019-LINE/RTEBo       ---------- ---------- AAGTTGATTA GACAATATAT GGCGCANC-- -----TTCAG CTTACCGAGG 43
Sly-rnd3_family322-LINE/RTEBov       CGTTAAAAAA GAATTGGGAG ANGTGAGTAC GCCAANACAT TGCACATAGG GCTAGTGCAG CTTCCTGAGG 157
Sly-rnd5_family1698-LINE/RTEBo       ---------- ---------- ---------- ---------- ---------- ---------- ---------- -
Sly-rnd1_family795-LINE/RTEBov       TTGCTTAAAA CTTGATGGGG TTAATGTTTT TCAAGGACAT GGTGTGTTGG TAGGTGTAGG TCTATCATGG 70
Stu-rnd-5_family-3162_LINE_RTE       AAATGGGGTG GGTTGACTCC TGCCCTTTCT CGGGAGATGG GNGAGAAGTT GATGGCGATG GGGGCTTGGA 1747
Sly-rnd6_family1960-LINE/RTEBo       AAATGGGGTG GCTTGACCCC TACTCTTTCN CNGAAGATCG CGGAGAAGTT NATTGGTNTG GGGGCTGGGA 918
Stu-rnd-5_family-2493_LINE_RTE       ---------- ---------- ---------- ---------- ---------- ---------- ---------- -
Sly-rnd5_family447-LINE/RTEBov       ---------- ---------- -----GCATC AAAGACGAGG ANGGCAAAGT ATTGGTGGAG GAGGCACTCA 45
Stu-rnd-6_family-2093_LINE_RTE       ---------- ---------- ---------- ---------- ---------- ---------- ---------- -

Consensus                            CT-CAAGC-A GGCT-TCAGC AGTATGTGNA CCGA-GAACT ACCAGAAGT- CAAGCTGGAT TTCGAAGAGG
```

Conservation

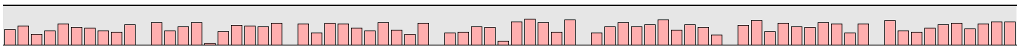

```
                                                 2,960                          2,980                          3,000
Stu-rnd-1_family-417_LINE_RTE-     ---------- ---------- ---------- ---------- ---------- ---------- ---------- -
Sly-rnd1_family377-LINE/RTEBov     ---------- ---------- ---------- ---------- ---------- ---------- ---------- -
Stu-rnd-1_family-489_LINE_RTE-     ---------- ---------- ---------- ---------- ---------- ---------- ---------- -
Stu-rnd-1_family-607_LINE_RTE-     TGTTCATTGA CCTTGAAAAG GCATACGACA AAGTCCCGAG GAATGTCCTN TGGAGATGTT TGGAGGCTAG 239
Stu-rnd-1_family-645_LINE_RTE-     ---------- ---------- ---------- ---------- ---------- ---------T TGGAGGCTAG 11
Sly-rnd4_family81-LINE/RTEBovB     AAGTCACTGA CCTTGAAAAA ACTTATGGTA AAGTCCCGAG GAAGNTTCTA TNTAGGTGCT TGGAGGCTAG 86
Stu-rnd-6_family-408_LINE_RTE-     ---------- ---------- ---------- ---------- ---------- ---------- ---------- -
Stu-rnd-1_family-351_LINE_RTE-     TGGATGAAAT GGAGGCTCGC ATCCGGAGTC TTGTGTGATA AGAAGGTGCC ACCAAGACTT AAAGGTAAGT 265
Stu-rnd-1_family-382_LINE_RTE-     ---------- ---------- ---------- ---------- ---------- ---------- ---------T 1
Sly-rnd6_family746-LINE/RTEBov     ---------- ---------- ---------- ---------- ---------- ---------- ---------- -
Stu-rnd-5_family-325_LINE_RTE-     ---------- ---------- ---------- ---------- ---------- ---------- ---------- -
Sly-rnd4_family509-LINE/RTEBov     CGGTTGAAAT GGAGAAGTNC TATCGGAGTG TTATGTGATA GAAGAATGCC TACCAAAGTG AAAGGTAAGT 78
Stu-rnd-1_family-513_LINE_RTE-     ---------- ---------- ---------- ---------- ---------- ---------- ---------- -
Sly-rnd5_family1442-LINE/RTEBo     ---------- ---------- ---------- ---------- ---------- ---------- ---------- -
BovB_Gl-RTE-Gehyra_lazelli         CAGAGGAACG A-GAGACCAA ATT---GCCA ACATTCGCTG G-ATTATGGA GAAA----GC AAGGGAGTAC 2254
BovB_Gv-RTE-Gehyra_variegata       CAGAGGAACG A-GAGACCAA ATT---GCCA ACATTCGCTG G-ATTATGGA GAAA----GC AAGGGAGTAC 2183
BovB_PMo-RTE-Python_molurus        CAGAGGAACT A-GAGATCAA ATT---GCCA ACATTCGCTG G-ATCATGGA GAAA----GC ACGAGAGTTC 2115
BovB_Oa-RTE-Ovis_aries             CAGAGGAACC A-GAGATCAA ATT---GCCA ACATCCATTG G-ATCATAGA AAAA----GC AAGAGAATTC 2141
BovB-RTE-Bos_taurus                CAGAGGAACC A-GAGATCAA ATT---GCCA ACATCCGCTG G-ATCATGGA AAAA----GC AAGAGAGTTC 2739
BovB_Me-RTE-Macropus_eugenii       CAGAGGAACA A-AAGACCAA ATT---GTCG ACATTGACTG G-ATTATGGA GAAA----GC AGGGGATTTC 1770
BovB_Ano-RTE-Amphibolurus_norr     CAGAGGAACT A-GAGACCAA ATT---GCTA ACATGCGCTG G-ATTATGGA GAAA----GC CAGAGAGTTC 2188
BovB_Gd-RTE-Eremiascincus_doug     CAGAGGAACT C-GAGATCAA ATT---GCCA ACCTTCGCTG G-ATCATGGA GAAA----GC AAGAGAGTTC 1776
BovB_Er-RTE-Eremiascincus_rich     CAGAGGAACT A-GAGATCAA ATT---GCCA ACCTTCGCTG G-ATCATGGA GAAA----GC AAGAGAGTTC 2311
BovB_Ca-RTE-Ctenotus_atlas         CAGAGGAACT A-GAGATCAA ATT---GCCA ACATTCGCTG G-ATCATGGA GAAA----GC AAGAGAGTTC 2304
BovB_Es-RTE-Egernia_stokesii       CAGAGGAACT A-GAGATCAA ATT---GCCA ACATTTGCTG G-ATTATGGA GAAA----GC AAGGGAGTTC 2138
BovB_Tr-RTE-Tiliqua_rugosa         CAGACGAACT A-GAGATCAA ATT---GCCA ACATTTGCTG GCAATATGGA GAAA----GC AAGCGAGTTC 2146
BovB_ALi-RTE-Amblyomma_limbatu     AAGAGGAACT AAGAGATTAA ATT---GTAA AAATACCCTG G-ATTATAAA GAAATTTAGC TAGGAAGTTC 2309
BovB_Hs-RTE-Hydrophis_spiralis     CAGAGAAACC A-GAGATCAA ATT---GCCA ACATACGCTG G-ATCATGGA GAAA----GC TAGGGAGTTC 519
BovB_VA-RTE-Vipera_ammodytes       CAGAGGAACT A-GAGATCAA ATT---GCCA ACATACGCTG G-CTCATGGA GAAA----GC TAGAGAGTTC 2109
BovB_ACo-RTE-Agkistrodon_conto     CAGAGGAACT A-GAGATCAA ATT---GCCA ACATACGCTG G-ATCATGGA GAAA----GC TAGGGAGTTC 2109
BovB_Pg-RTE-Perameles_gunnii       CAGAGGAACT A-GAGACCAA ATT---GCCA ACATTCACTG G-ATTATGGA GAAA----GC AAGGGAGTTC 260
BovB_Io-RTE-Isoodon_obesulus       CAGAGGAACC A-GAGATAAA ATT---GTCA AGGTTCACTG G-ATAATGAA GAAA----GC TGGAGAGTTG 710
BovB_Bp-RTE-Burramys_parvus        CAGAGGAACT A-GAGACCAA ATT---GCCA ACATTCACTG G-ATTATGGT GAAA----TC AAGGGAGATC 807
BovB_Pb-RTE-Petaurus_breviceps     CAGAGGAACT A-GAGACCAA ATT---GCCA ACATTTACTG G-ATTATGGA GAAA----GC AAGGGAGTTC 874
BovB_Ma-RTE-Macropus_antilopin     CAGAGGAACT A-GAGACCAA ATT---GCCA ACATTCACTG G-ATTATGGA GAAA----GC AAGGGAGTTC 2223
BovB_Af-RTE-Antechinus_flavipe     TAGAAGAATT A-GAGATCAA ATT---GCCA ACATTCACTA G-ATTATGAA TAAA----GC AAGAAAGTTC 1438
BovB_Ac-RTE-Anolis_carolinensi     CAGAGGAACG A-GAGACCAA ATTATCGCCA ATATCCACTG G-ATAATGGA GGAA---AGC CAGGGAGTTT 2083
BovB_BM-RTE-Bombyx_mori            CAGAGGTACT C-GCGAGCAG ATC---CTAA ACATCCGACA A-TTAATAGA AAGAT--GCC AC--GAAT-T 1954
BovB_Dr-RTE-Christinus             ACTTGGAACC A-GAGACCAG ATT---GCAA ACCTGCGTTG G-TTAATGGA GAAGA--CGC GT--GAATAT 1975
BovB_La-RTE-Loxodonta_africana     ACATGGAACC A-GGGATATC ATT---GCTG ATGTCAGATG G-ATCCTGGC TGAAA--GGC AC-AAAATAC 2103
BovB_Pc-RTE-Procavia_capensis      ACGTGGAACC A-GGGATATC ATT---GCTG ATGTCAGATG G-ATCTTGGC TGAAA--G-C AG-AGAATAC 2104
BovB_Et-RTE-Christinus             -TGTGAAACA A-AGGAGATC AAT---GCTA ATGTCAGATG A-ATCTTG-C TCAAA--G-C AA-AGAACAC 1881
BovB_Ta-RTE-Tachyglossus_acule     ACAAGGGACA T-GAGATATT ATT---GCTG ATGTCCGTTG G-ATAAT--T GAAAG--GAC AAGAGAATAT 2244
BovB_Plat-RTE-Ornithorhynchus      ACGAGGGACG C-GAGATATT ATT---GCTG ATGTCCGTTG G-ATAAT--T GAAAG--GAC AAGAGAATAT 2233
BovB_Ec-RTE-Equus_caballus         GAGAGGCACA T-GAGATCTA ATT---GTGA TTATTCATTG G-CTACTGGA GTGCT--CCA AA--GAATTT 2003
BovB_Cg-RTE-Christinus             AATAGGCACT A-GAGATCAA ATT---ATAA ATATATTTTG G-TTACTGGA GCATA--CGA AA--GAATGA 1524
BovB_Bh-RTE-Bothriocroton_hydr     CAATACTGCA TAGAACCTGG AAC---GTCA G-ATCTATGA A--TTAAGGT AAAC-----T AGATGTGGTC 560
BovB_Ml-RTE-Gehyra_variegata       TAAAGGAGCT G----ATGGA ATTTCAGGTA AGCTATTTTA A-ATCCTAAA AGATTC-TGT GGTTAAAGTA 623
Sly-rnd1_family284-LINE/RTEBov     ---------- ---------- ---------- ---------- ---------- ---------- ---------- -
Sly-rnd5_family2019-LINE/RTEBo     ACATGACCCT AGATAGGAGG GTG---TGGA GGTCGAG--- GATTAGGGTA GAAG------ -------GTT 94
Sly-rnd3_family322-LINE/RTEBov     AAATGGCCTT ATATAGTAAG GTG---TGTA GGACACG--- GATTAGGGTA GACGAAGGGC GAT---AGTT 218
Sly-rnd5_family1698-LINE/RTEBo     ---------- ---------- ---------- ---------- ---------- ---------- ---------- -
Sly-rnd1_family795-LINE/RTEBov     ATGTGTTTCA ATAGTGGCGG AAT---TAGG ATTTTCACTA GGTTGAAATA GGAATAATAA GTTTCTANGT 137
Stu-rnd-5_family-3162_LINE_RTE     GNAGTAG--- -CGGGGACGC GG-----ACA GCATGTGGG- ---ATAAGGC AGCTAGTTGC ATTAGGGAAG 1804
Sly-rnd6_family1960-LINE/RTEBo     GCGGTAGGTA GCGGGAAAAC GGGTACGNCA CCGCGCGGGT TAAATAAGAC AACTAGTTGC ATTGGGGAAG 988
Stu-rnd-5_family-2493_LINE_RTE     ---------- ---------- ---------- ---------- ---------- ---------- ---------- -
Sly-rnd5_family447-LINE/RTEBov     TTAGACGAGG ATGGCAGACG TACTTCCATA AACTCTTGAA CGAAGAGGGG ACGGAGNATC ACGTCCGGGT 115
Stu-rnd-6_family-2093_LINE_RTE     ---------- ---------- ---------- ---------- ---------- ---------- ---------- -
Consensus                          CAGAGGAACT A-GAGATCAA ATT---GCCA ACATTCGCTG G-ATTATGGA GAAA----GC AAGAGAGTTC
```

100%

Conservation

0%

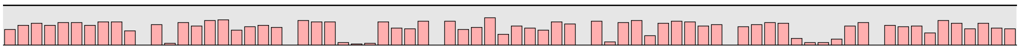

3,020 3,040 3,060 3,080

Stu-rnd-1_family-417_LINE_RTE-	----------	----------	----------	----------	----------	------GTGG	TGGACAAGAT	14
Sly-rnd1_family377-LINE/RTEBov	----------	----------	----------	----------	----------	----------	----------	-
Stu-rnd-1_family-489_LINE_RTE-	----------	----------	----------	----------	----------	----------	----------	-
Stu-rnd-1_family-607_LINE_RTE-	AGGTGTACCG	GTGGCTTACA	TTAGGGCGAT	NAAGGACATG	TACGATGGAG	-CCAAGACTC	GGGTNAGAAC	308
Stu-rnd-1_family-645_LINE_RTE-	AGGTGTACCG	GTGGCTTACA	TTAGGGCGAT	NAAGGACATG	TACGATGGAG	-CCAAGACTC	GGGTNAGAAC	80
Sly-rnd4_family81-LINE/RTEBovB	AGGTGTACTT	GTGGCNTACA	CTAGGGCGAT	TAAAGACATG	TATGATGGAG	-CCAAGACTC	GGGTAAGAAT	155
Stu-rnd-6_family-408_LINE_RTE-	----------	----------	----------	----------	-------AAG	-ACGTCACTA	GCGTGAGAAC	22
Stu-rnd-1_family-351_LINE_RTE-	TCTACAGAGT	GGTGGTTAGA	CCGACTTTGT	TGTATGGGGC	GGAGTGTTGG	CCAGTCAAGA	ACTCNCACGT	335
Stu-rnd-1_family-382_LINE_RTE-	TCTACAGAGT	GGTGGTTAGA	CCGACTTTGT	TGTATGGGGC	GGAGTGTTGG	CCAGTCAAGA	ACTCNCACGT	71
Sly-rnd6_family746-LINE/RTEBov	----------	----------	----------	----------	----------	----------	----------	-
Stu-rnd-5_family-325_LINE_RTE-	----------	----------	----------	----------	----------	----------	----------	-
Sly-rnd4_family509-LINE/RTEBov	TTTACGAAAC	AGTTGTAAGA	CCGACAATGT	TATATGAGAG	TGAATGTTGG	GCCGCTAAAG	TCCAACATAT	148
Stu-rnd-1_family-513_LINE_RTE-	----------	----------	----------	----------	----------	----------	----------	-
Sly-rnd5_family1442-LINE/RTEBo	----------	----------	----------	----------	----------	----------	----------	-
BovB_Gl-RTE-Gehyra_lazelli	CAGAAAAAC-	--------AT	CTACT-TCTG	CTTCATTGAC	TATGCTAAGG	-CCTTTGATT	GTGTGG----	2309
BovB_Gv-RTE-Gehyra_variegata	CAGAAAAAC-	--------AT	CTACT-TCTG	CTTCATTGAC	TATGCTAAGG	-CCTTTGATT	GTGTGG----	2238
BovB_PMo-RTE-Python_molurus	CAGAAAAAT-	--------AT	CTACT-TCTG	CTTCATTGAC	TATGCAAAAG	-CCTTTGATT	GTGTGG----	2170
BovB_Oa-RTE-Ovis_aries	CAGAAAAAC-	--------AT	CTACT-TCTG	CTTCATTGAC	TATGCCAAAG	-CCTTTGACT	GTGTGG----	2196
BovB-RTE-Bos_taurus	CAGAAAAAC-	--------AT	CTATT-TCTG	CTTTATTGAC	TATGCCAAAG	-CCTTTGACT	GTGTGG----	2794
BovB_Me-RTE-Macropus_eugenii	CAGGGAAAGC	-------AAG	ATACT-TCTG	CTTCATAGAC	TACACGAAAG	-CCTTTGACT	TTGCAG----	1827
BovB_Ano-RTE-Amphibolurus_norr	CAGAAAAAT-	--------AT	CTACT-TCTG	CTTCATTGAC	TATGCAAAAG	-CCTTTGACT	GTGTGG----	2243
BovB_Gd-RTE-Eremiascincus_doug	CAGAAAAA--	-------CAT	CTACC-TCTG	CTTCATTGAC	TATGCTAAAG	-CCTTTGATT	GTGTGG----	1831
BovB_Er-RTE-Eremiascincus_rich	CAGAAAAA--	-------CAT	CTACC-TCTG	CTTCATTGAC	TATGCTAAAG	-CCTTTGATT	GTGTGG----	2366
BovB_Ca-RTE-Ctenotus_atlas	CAGAAAAA--	-------CAT	CTACC-TCTG	CTTCATTGAC	TATGCTAAAG	-CCTTTGATT	GTGTGG----	2359
BovB_Es-RTE-Egernia_stokesii	CAGAAAAAA-	-------CAT	CTACT-TCTG	CTTCACTGAC	TACGCTAAAG	-CCTTTGATT	GTGTGG----	2194
BovB_Tr-RTE-Tiliqua_rugosa	CAGAAAAA--	-------CAT	CTACA-TCTG	CTTCATTGAC	TACACTAAAG	-CCTTTGATT	GTGTGG----	2201
BovB_ALi-RTE-Amblyomma_limbatu	TAGAAAAAA-	--------AT	CAATT-TTTA	CTTCTTTAAT	AAAATTAAAT	ACTTTTAATA	TTTTTATAAT	2369
BovB_Hs-RTE-Hydrophis_spiralis	CAGGAAAAC-	--------AT	CTACT-TCTG	CTTCATTGAC	TATGCTAAAC	-CTTTTAATT	TTTTTG----	574
BovB_VA-RTE-Vipera_ammodytes	CAGAAAAAC-	--------AT	CTACT-TCTG	CTTCATTGAC	TATGCTAAAG	-CCTTTGATT	GTGTGG----	2164
BovB_ACo-RTE-Agkistrodon_conto	CAGAAAAAC-	--------AT	CTACT-TCTG	CTTCATTGAC	TATGCTAAAG	-CCTTTGATT	GTGTGG----	2164
BovB_Pg-RTE-Perameles_gunnii	CAGAAAAATA	TCTTCTTCTA	CTACT-TCTG	CTTCATTGAC	TACACTAAAG	-CCTTTGATT	GTGTGTG---	325
BovB_Io-RTE-Isoodon_obesulus	TAGAAAAAAC	AAT-------	CTGCT-TTTG	CTACTTTTTC	TACATTAGAA	-CCTTTGACT	GTG-------	764
BovB_Bp-RTE-Burramys_parvus	CAGAAAAAA-	---------T	CTATT-TCTG	CTTCATTAAC	TACACTAAAG	-CCTTTGACT	GTATGA----	861
BovB_Pb-RTE-Petaurus_breviceps	CAGAAAAAAA	GCA------T	CTACT-TCTG	CTTCATTGAC	TACACTAAAG	-CCTTTGACT	GTGTG-----	931
BovB_Ma-RTE-Macropus_antilopin	CAGAAAAACA	---------T	CTACT-TCTG	CTTCATTGAC	TACACTAAAG	-CCTTTGACT	GTGTG-----	2277
BovB_Af-RTE-Antechinus_flavipe	CAGAAAAACA	---------T	CCACT-ATAG	C-ATGTCTGC	TTCATTTA--	--CTTTGACC	ATGTA-----	1488
BovB_Ac-RTE-Anolis_carolinensi	CAGAAAAAAC	--------AT	CTACT-TCTG	CTTTATTGAC	TATTCCAAAG	-CCTTCGACT	GCGTGG----	2139
BovB_BM-RTE-Bombyx_mori	CGATACACC-	--------TA	TTATCCTGTG	CTTCGTTGAC	TACAGCAAGG	-CGTTTGACT	GCGTTG----	2010
BovB_Dr-RTE-Christinus	CAAAAGAAC-	--------GT	GTATA-TGTG	CTTCATCGAC	TACAGCAAAG	-CTTTCGACT	GTGTCA----	2030
BovB_La-RTE-Loxodonta_africana	CAGAAAGAT-	--------GT	TTACC-TGTG	TTTTATTGAC	TATGCAAAGG	-CATTCGACT	GTGTGG----	2158
BovB_Pc-RTE-Procavia_capensis	CAGAAAGAT-	--------GT	TTACT-TGTG	TTTCATTGAC	TATGCAAAAG	-CATTTGACT	GTGTGG----	2159
BovB_Et-RTE-Christinus	CAGAAAGAT-	--------GT	TTACT-TCAG	TTTCACTGAC	TATGCAAAGG	-CATTCG--T	GGGTGG----	1934
BovB_Ta-RTE-Tachyglossus_acule	CAAAAGGAA-	--------GT	CAGCA-TGTG	CTTTATTGAT	TATAGTAAGG	-CCTTTGACT	GTGTGG----	2299
BovB_Plat-RTE-Ornithorhynchus	CAAAAGGAA-	--------GT	CAGCA-TGTG	CTTTATTGAT	TATAGTAAGG	-CCTTTGACT	GCGTGG----	2288
BovB_Ec-RTE-Equus_caballus	CAGAAGAAG-	--------GT	TAGTC-TATG	TTTTATAGAC	TACAGTAAAG	-CCTTTGACT	GCATGG----	2058
BovB_Cg-RTE-Christinus	CAGAAGAAG-	--------AT	CAGCT-TGTG	TTTTAAAGAA	TACAGTAAAG	-TTTTTGACA	ACATGG----	1579
BovB_Bh-RTE-Bothriocroton_hydr	AAACGCGAGA	-------TGT	CAAGACTGAA	C---ATCGAC	ATCATGGGAA	TTAGTCAACT	AAA-------	613
BovB_Ml-RTE-Gehyra_variegata	CAGGACCCA-	--------AT	GTGCCAGCAA	ATTTGGAAAA	CTCTACAGTG	ATCACTGGTT	TAGAAA----	680
Sly-rnd1_family284-LINE/RTEBov	----------	----------	----------	----------	----------	----------	----------	-
Sly-rnd5_family2019-LINE/RTEBo	AGTAGGTAGT	---------C	GAGTCTTGTC	TTGTTTTTCG	ACGGGTTA--	GTAGGTTAGT	TGGTATT---	150
Sly-rnd3_family322-LINE/RTEBov	NGTAGGACTN	---------A	GGGTCGCATC	CTGCNTTACN	ATTAGTCG-T	ACNANTGTAT	TAGTNTTG--	276
Sly-rnd5_family1698-LINE/RTEBo	----------	----------	----------	----------	----------	----------	----------	-
Sly-rnd1_family795-LINE/RTEBov	TTTGGATAAC	---------T	AAAACAACAC	TTATTTTACT	TGGATTTGAT	ACACTTGAGT	TGTTGGTC--	196
Stu-rnd-5_family-3162_LINE_RTE	TNGCTTCTAA	GGTGTTAGGG	GTNTC-GAGG	GGTAAAT---	-CCGGNGGAC	ATAGAGGAGA	TTG-GTGGTG	1868
Sly-rnd6_family1960-LINE/RTEBo	AAGCTACAAA	GGTGCAAAGG	GTATC-GAGG	GGTATGTACC	TCTAGTCGAG	ATCGAGGAGA	CTGAGTGGTG	1057
Stu-rnd-5_family-2493_LINE_RTE	----------	----------	----------	----------	----------	----------	----------	-
Sly-rnd5_family447-LINE/RTEBov	GATTTGGAGC	ACTCCGAGAG	ACGTC-GNGA	TTTCGGG---	--TANTGTAG	GTGTATAAAG	TTGAGGTGGA	179
Stu-rnd-6_family-2093_LINE_RTE	----------	----------	----------	----------	----------	----------	----------	-
Consensus	CAGAAAAA--	--------AT	CTACT-TCTG	CTTCATTGAC	TATGCTAAAG	-CCTTTGACT	GTGTGG----	

Conservation
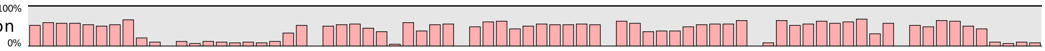

```
                                          3,100                    3,120                    3,140
Stu-rnd-1_family-417_LINE_RTE-   GAGGGAAGCG AGACTGAGAT GGTTCGGACA TGTGAAGAGG AGATGCGCAG ACGCCCCAGT GAGGAGGTGC 84
Sly-rnd1_family377-LINE/RTEBov   ---------- ---------- ---------- ---------- ---------- ---------- ---------- -
Stu-rnd-1_family-489_LINE_RTE-   ---------- ---------- ---------- ---------- ---------- ---------- ---------- -
Stu-rnd-1_family-607_LINE_RTE-   GGTGGGAGGA GACTCGGAGC ACTTCCCAGT TGTGATGGGG TTGCACCAGG GATCAACCCT TAGCCCGTTT 378
Stu-rnd-1_family-645_LINE_RTE-   GGTGGGAGGA GACTCGGAGC ACTTCCCAGT CGTGATGGGG TTGCACCAGG GATCAACCCT TAGCCCGTTT 150
Sly-rnd4_family81-LINE/RTEBovB   AGTGGGAGGA GACTCGGAGC ACTTTCCAGT TGNGATGGGG TTGCACCGAG GATCAGCNCT TAACCCGTTT 225
Stu-rnd-6_family-408_LINE_RTE-   AGTGGTAGGA GATACAAAGG AGTTTCCCAT AATCGTGCGT TTACACTAGG GATCGGCATT GATNNCNCTA 92
Stu-rnd-1_family-351_LINE_RTE-   TCAGAAGATG AAAGTTGCGG AAATGAGGAT GTTGAGATGG ATGTGTGGGC ATACTAGGAG AGATAAGATT 405
Stu-rnd-1_family-382_LINE_RTE-   TCAGAAGATG AAAGTTGCGG AAATGAGGAT GTTGAGATGG ATGTGTGGGC ATACTAGGAG AGATAAGATT 141
Sly-rnd6_family746-LINE/RTEBov   ---------- ---------- ---------- ---------- ---------- ---------- ---------- -
Stu-rnd-5_family-325_LINE_RTE-   ---------- ---------- ---------- ---------- --------TA TTACAAGATT AGACAAGATT 22
Sly-rnd4_family509-LINE/RTEBov   CCACAAGACG AGTGTCGTAG AAATGCGGAT GCTAAGATGG ATGTGCGGTC ATACAAGATT AGACAAGATT 218
Stu-rnd-1_family-513_LINE_RTE-   ---------- ---------- ---------- ---------- ---------- ---------- ---------- -
Sly-rnd5_family1442-LINE/RTEBo   ---------- ---------- ---------- ---------- ---------- ---------- ---------- -
BovB_Gl-RTE-Gehyra_lazelli       ----ATCACA ACCAACTATG GCAAGTCCTT AAAGAGATGG GAGTACCAGA CCACCTCAC- ACGTCTCTTG 2374
BovB_Gv-RTE-Gehyra_variegata     ----ATCACA ACCAACTATG GCAAGTCCTT AAAGAGATGG GAGTACCAGA CCACCTCAC- ACGTCTCTTG 2303
BovB_PMo-RTE-Python_molurus      ----ATCACA ACAAACTGTG GCAAATTCTT AAAGAGATGG GAGTACCAGA CCACCTTAC- CTGTCTCCTG 2235
BovB_Oa-RTE-Ovis_aries           ----ATCACA ACAAACTGTG GAAAATTCTT AAAGAGATGG GAATACCAGA CCACCTTAC- CTGCCTCCTG 2261
BovB-RTE-Bos_taurus              ----ATCACA ATAAACTGTG GAAAATTCTG AAAGAGATGG GAATACCAGA CCACCTGAC- CTGCCTCTTG 2859
BovB_Me-RTE-Macropus_eugenii     ----ATCACC ACAAAATATA GCAAGTGCTC AAAGAGATGG GAGCACTGCA TCACCTTAC- TTGCCT---G 1889
BovB_Ano-RTE-Amphibolurus_norr   ----ACCACA GCAAACTATG GCAAGTTCTT AAAGAAATGG GAGTGCCTGA CCACCTTAT- CTATCTCCTG 2308
BovB_Gd-RTE-Eremiascincus_doug   ----ATCACA ACAAACTGTG GCAAGTTCTG GAAGAGATGG GAGTACCAGA CCACCTGAT- CTGTCTTCTG 1896
BovB_Er-RTE-Eremiascincus_rich   ----ATCACA ACAAACTGTG GCAAGTTCTG GAAGAGATGG GAGTACCAGA CCACCTGAT- CTGTCTTCTG 2431
BovB_Ca-RTE-Ctenotus_atlas       ----ATCACA ACAAACTGTG GCAAGTTCTT GAAGAGATGG GAGTACCAGA CCACCTCAT- CTGTCTTCTG 2424
BovB_Es-RTE-Egernia_stokesii     ----ATCACA ACAAATTGTG GCAAGTTCTT AAAGAGATGG GAGTACCAAA CCACCTTAT- CTGTCTCCTG 2259
BovB_Tr-RTE-Tiliqua_rugosa       ----ATCACA ACAAATTGTG GCAAGTTCTT AAAGAGATGG GAGTACCAAA CCACCTTAT- CTGTCTCCTG 2266
BovB_ALi-RTE-Amblyomma_limbatu   ATCCAACACA AAAAATAGTA CCAAATTTTT AGAGAGATGG GAGCACCGGA CCATCTTAT- TTGTCTCTTG 2438
BovB_Hs-RTE-Hydrophis_spiralis   ----AACACA ACAAATTGTG GCAAGTTCTT AAAGAGATGG GAGTACCAGA CCATCTTAT- TTGTCTCTTA 639
BovB_VA-RTE-Vipera_ammodytes     ----ATCATA ACAAACTGTG GCAAGTTCTT AAAGAGATGG GCGTACCAGA CCATCTTAT- TTGTCTCTTG 2229
BovB_ACo-RTE-Agkistrodon_conto   ----ATCACA ACAAATTGTG GCAAGTTCTT AAAGAGATGG GAGTACCAGA CCATCTTAT- TTGTCTCTTG 2229
BovB_Pg-RTE-Perameles_gunnii     ---AATCACA ACAAAATGTG ACACGGCCTC AAAGAGATAG GAATACCAGA TCATCTTAC- TTGTCTCCTC 391
BovB_Io-RTE-Isoodon_obesulus     -----TTGCC ACACAATT-- GCAAGTCTCC AAATAAGTGA AAATATCAAA TCATCTTTCC TTGTCTCCTG 827
BovB_Bp-RTE-Burramys_parvus      ---GATCACA ACAAAATGTG GCAAGTCCTC AAAGAGATGG GAGTACCAGG TCATCTCAC- TTGTCTCCTG 927
BovB_Pb-RTE-Petaurus_breviceps   ---GATCACA ACAAAATGTG GCAAGTCCTC AAAAAGATGG GAGTACCAGA TCATCTTAC- TTGTCTCCTG 997
BovB_Ma-RTE-Macropus_antilopin   ---GATCACA ACAAAATGTG GCAAGTCCTC AAAGAGATGG GAGTACCAGA TCATCTTAC- TTGTCTCCTG 2343
BovB_Af-RTE-Antechinus_flavipe   ---ACTTATA ACATA-TGTG GGAAGTCCTC AAAGAGATG- ---TACTAGA CCACCCTAC- -TGCAGCCCC 1548
BovB_Ac-RTE-Anolis_carolinensi   ----ATCATA ATAAACTATG GCATGTTCTT GGTGGTATGG GGATACCAAG TCACCTTGT- CTGTCTCCTG 2204
BovB_BM-RTE-Bombyx_mori          ----GCTGGA ACTGCTTATG GCGGGTCCTT CAGGAACTCG GAGTACCCAT GCACCTTAA- -AGCTTTTCT 2074
BovB_Dr-RTE-Christinus           ----ATCATG GCGAGCTATG GAAATGTCTG AAGCAGATGG GCATCCCGCA GCATTTAAT- -AGATCTGAC 2094
BovB_La-RTE-Loxodonta_africana   ----ATCATA ACAAAT-ATG GATAACACTG CGAAGAATGG GAATTCCAGA ACACTTAAT- -TGTGCTCAT 2221
BovB_Pc-RTE-Procavia_capensis    ----ATCATA CTAAATTATG GCTAACATTG TGGAGAATGG GAATTCCAGA ACACCTAAT- -TGTGCTCTT 2223
BovB_Et-RTE-Christinus           ----ACCATA ACAAACTACG AAGAACACTC AAAGAGATGG GAATTCCACA ACACTTCAT- -TGTGCTAAT 1998
BovB_Ta-RTE-Tachyglossus_acule   ----ATCACG AGAAACTCTG GAGCACATTA AGGAAAATGG GCATGCTGGA CCATTTAAT- -TGCATTAAT 2363
BovB_Plat-RTE-Ornithorhynchus    ----ATCACG AGAAACTCTG GAGCACATTA AGGAAAATGG GCATGCCGGA CCATTTAAT- -TGCATTAAT 2352
BovB_Ec-RTE-Equus_caballus       ----ATCATG AAAAGCTATG GGCTGCTCTG AAAGAAATGG GCATGCCTCA GCACTTGAT- -TGTCCTGAT 2122
BovB_Cg-RTE-Christinus           ----ATCATG AAAAGTTATG GTTGGTTTTT AAAGAAATGG ATATGCCACA ACATCTGAT- -TGTTTTGAT 1643
BovB_Bh-RTE-Bothriocroton_hydr   ----AATAGG GCAGGA-ATG GGAGAATTTA TTTCAGATG- ----ACCATT ACATCTACT- -ACTATGGGC 671
BovB_Ml-RTE-Gehyra_variegata     -----TAATT GTTTTATGT- CCCAATCATA AAGAAGAGCA AAATGTTAAA TTACTGGAC- -AATTTCATT 742
Sly-rnd1_family284-LINE/RTEBov   ---------- ---------- ---------- ---------- ---------- ---------- ---------- -
Sly-rnd5_family2019-LINE/RTEBo   --CGGGTATC GTATTAGCC- ---TATT-CT CGTACGTATC TTGNTCCTTC GATTTCCGTT --ATCACCTG 211
Sly-rnd3_family322-LINE/RTEBov   --CTGTCATA CTAGTAGTCG TAGTATTACT CTTGTAGTTT CTTGTNCTTC CTTTTCTGTT --ACTATTTA 342
Sly-rnd5_family1698-LINE/RTEBo   ---------- ---------- ---------- --------TG TTGTTTATTG TGTTTCGATT --ATCGCACT 30
Sly-rnd1_family795-LINE/RTEBov   --TTTCGAAA ACAATCTCTT TTTCAATACA CAAGTAAACT AGGTAGTGTG TACTCTAACC GGACAGTTTC 264
Stu-rnd-5_family-3162_LINE_RTE   GAATGGGGAA GTCCAAGGCA AAGTGGAAGC GAAGAAGGCT GCGTACGCGA AGTTGGCGGA GTGCGTAGAC 1938
Sly-rnd6_family1960-LINE/RTEBo   GAATGGTGAA GTGNAAGGNA AAGTGGAAGC AAAGTAGGCG GCATATGCAA AGTTGGCGGA GTTCGCGGAT 1127
Stu-rnd-5_family-2493_LINE_RTE   GAGTAGGGGA AGAGCGACCG GACCGGATGA GATTCCGGTG GAATTTTGGA AGAGCGCAGA CAAGGCAGGT 70
Sly-rnd5_family447-LINE/RTEBov   GCGTAAGGGA AGAGCGACCG GGCCGGACGA GATCCNGATG GAATTTTGGA AGAGCGCGGG CAAGGCAGGT 249
Stu-rnd-6_family-2093_LINE_RTE   ---------- ---------- ---------- ---------- ---------- ---------- ---------- -
Consensus                        ----ATCACA ACAAACTGTG GCAAGTTCTT AAAGAGATGG GAGTACCAGA CCACCTTAT- -TGTCTCCTG
```

Conservation

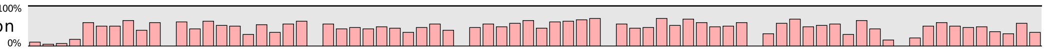

```
                                 3,160                  3,180                  3,200                  3,220
                                   |                      |                      |                      |
Stu-rnd-1_family-417_LINE_RTE-   GAGAGGTTGG CTATAGTGGG TNCGAGGAGA GGTAGAGGTA GGCCGAAGA- AGTATTGGGG AGAGGTGATT 153
Sly-rnd1_family377-LINE/RTEBov   ---------- ---------- ---------- ---------- -----AAGA- AGTATTGGGG AGAGGTGATT 24
Stu-rnd-1_family-489_LINE_RTE-   ---------- ---------- --TAAGGAGA GGTAGAGGTA GGCCGAAGA- AGTATTGGG- AGAGGTGATT 46
Stu-rnd-1_family-607_LINE_RTE-   TTATTTGCCT TGGTGATGGA TGAATTGACG CGACATATTC AAGANGAGG- TGTCGTGGTG TATGTTATTT 447
Stu-rnd-1_family-645_LINE_RTE-   TTATTTGCCT TGGTGATGGA TGAATTGACG CGACATATTC AAGANGAGG- TGTCATGGTG TATGTTATTT 219
Sly-rnd4_family81-LINE/RTEBovB   TTATTTGCCT TGGCGATGGA TGAATTGACG CGGCATATTC AAGGCGAGG- TGCCATGGTG TATGTTATTT 294
Stu-rnd-6_family-408_LINE_RTE-   TTGCGTTGCC CAATNATA-- ---------- ------ATAC AAGATGAGG- TCTCATGATG CATGCTATTT 143
Stu-rnd-1_family-351_LINE_RTE-   AGGAAT--GA GGATATCCGG GACAAGGTGG GAGTGGCCTC CGTGGTGGA- CAAGATGAGG GAAGCGAGAC 472
Stu-rnd-1_family-382_LINE_RTE-   AGGAAT--GA AGATATCCGG GACAAGGTGG GAGTGGCCTC CGTGGTGGA- CAAGATGAGG GAAGCGAGAC 208
Sly-rnd6_family746-LINE/RTEBov   ---------- ---------- ---------- ---------- ------AGA- CAAGATGAGG GAAGCGAGGC 23
Stu-rnd-5_family-325_LINE_RTE-   AGAAANATGA TCACATTCGN CAGAAGGTGC AAGTAGCACG CATNGAGGA- TAAAATGAGA GAAGGTCGCT 91
Sly-rnd4_family509-LINE/RTEBov   AGAAAC--GA CCACGTTCGC CAGAAGGTGC AAGTAGCACA CATCGAGGA- TAAAATGAGA GAATGTCGCT 285
Stu-rnd-1_family-513_LINE_RTE-   ---------- ---------- ---------- ---------- ---------- ---------- ---------- -
Sly-rnd5_family1442-LINE/RTEBo   ---------- ---------- ------ATCT ACCGATCATG CTTGCGAAA- TGAAATGGAC CCGAATAGAG 43
BovB_Gl-RTE-Gehyra_lazelli       AGAAA-CCTG T-ATAAG--G GTCAAGAAGC AACAGTCC-G AACCGGATA- TGGAAC-AAC TG-ATTGGTT 2436
BovB_Gv-RTE-Gehyra_variegata     AGAAA-CCTG T-ATAAG--G GTCAAGAAGC GACAGTCA-G AACCGGATA- TGGAAC-AAC TG-ATTGGTT 2365
BovB_PMo-RTE-Python_molurus      AGAAA-TCTG T-ATGCG--G GCCAAGAAGC AACAGTTA-G AACCGGTCA- TGGAAC-AAC CG-ATTGGTT 2297
BovB_Oa-RTE-Ovis_aries           AGAAA-CCTG T-ATGCA--G GTCAAGAAGC AACAGTTA-G AACCGGACA- TGGAAC-AAC AG-ACTGGTT 2323
BovB-RTE-Bos_taurus              AGAAA-TCTG T-ATGCA--G GTCAGGAAGC AACAGTTA-G AACTGGACA- TGGAAC-AAC AG-ACTGGTT 2921
BovB_Me-RTE-Macropus_eugenii     AGGAA-CCTA T-ATGTA--G ATCAAGAAGC AACAGTTA-G AACCAAACA- TGGAAT-AAC TG-ATTGGTT 1951
BovB_Ano-RTE-Amphibolurus_norr   AGAAA-CCTA T-ATGTG--G GACAGGAAGC AACAGTTA-G AACTGGATA- TGGAAC-AAC TG-ATTGGTT 2370
BovB_Gd-RTE-Eremiascincus_doug   AGAAA-CCTG T-ATGCG--G GTCAGGAAGC AACAGTTA-G AACTGGACA- TGGAAC-AAC CG-ACTGGTT 1958
BovB_Er-RTE-Eremiascincus_rich   AGAAA-CCTG T-ATGCG--G GTCAGGAAGC AACAGTTA-G AACTGGACA- TGGAAC-AAC CG-ACTGGTT 2493
BovB_Ca-RTE-Ctenotus_atlas       AGAAA-CCTG T-ATGTG--G GCCAGGAAGC AACAGTTA-G AACTGGACA- TGGAAC-AAC TG-ATTGGTT 2486
BovB_Es-RTE-Egernia_stokesii     AGAAA-TCTG T-ATGCG--G GTCAAGAAGC AACCGTTA-G AACCAGATA- TGGAAC-AAC TG-ATTGGTT 2321
BovB_Tr-RTE-Tiliqua_rugosa       AGAAA-TCTG C-ATGAA--A GTCAAGACGC AACAGTTA-G AACCAGATA- TGGAACCAAA TA-ATTGGTT 2329
BovB_ALi-RTE-Amblyomma_limbatu   AGAAA-CCTA T-ATGCA--G GTCAAGAGGC AACAGTGA-G AACTGGACA- TGGAAC-AAC TG-ATTGGTT 2500
BovB_Hs-RTE-Hydrophis_spiralis   AAAAAACCTA T-ATGCG--G GTCAAGAAGC AACAGTGA-C AACTAGACA- TGGAAC-CAC TG-ATTGGTT 702
BovB_VA-RTE-Vipera_ammodytes     AGAAA-CCTA T-ATGCG--G GCCAAGAAGC AACAGTGA-G AACTGGACA- CGGAAC-CAC TG-ATTGGTT 2291
BovB_ACo-RTE-Agkistrodon_conto   AGAAA-CCTA T-ATGCG--G GTCAAGAAGC AACAGTGA-G AACTGGACA- CGGAAC-CAC TG-ATTGGTT 2291
BovB_Pg-RTE-Perameles_gunnii     AGGAA-TCTG TTACATG--G ACCAAGAAAT AACAGTTA-A AATTGAACA- TGGAAC-AGC TG-ATAGATT 454
BovB_Io-RTE-Isoodon_obesulus     AGGAA-CTTG T-ATGTG--G GCCAAGAAGC AATAGTTA-A AACTGAATAA TGGAAC-AAC TA-ATTGGAT 890
BovB_Bp-RTE-Burramys_parvus      AGGAA-CCTG T-ATATGCCA GCCAAGAAGC AGCAGTTA-G AACCAAACA- TGGAAC-AAT TG-ATTGGTT 991
BovB_Pb-RTE-Petaurus_breviceps   AGGAA-CCTG T-ATGTG--- GGCAAGAAGC AACAGTTA-G AACCAAACA- TGGAAC-AAC TG-ATTGGTT 1058
BovB_Ma-RTE-Macropus_antilopin   AGGAA-CCTG T-ATGTG--G GCCAAGAAGC AACAGTTA-G AACTGAACA- TGGAAC-AAC TG-ATTGGTT 2405
BovB_Af-RTE-Antechinus_flavipe   AGGAA-CATG A-ATGTG--G ATCAAGAAGT AA-AGATA-C AACTAAACA- TGCAAC-AAC TA-ATTGCTT 1609
BovB_Ac-RTE-Anolis_carolinensi   AGAAA-TCTG T-ATAAA--G ACCAAGTAGC CACAATAA-G AACAGACCA- CGGAAC-AAC AG-ACTGGTT 2266
BovB_BM-RTE-Bombyx_mori          CCAATCCCTT T-ACTAT--G GCAGTCAGGG CACTGTAA-G GGTCGACTA- CACTAT-GTC AA-ACCGTTT 2137
BovB_Dr-RTE-Christinus           ACAATCACTC T-ATAAG--A ATCAAAAGGC CACGGTCA-G GACAGCATT- TGGCAA-CAC TG-ACTGGTT 2157
BovB_La-RTE-Loxodonta_africana   GAGGAACCTG T-ACATA--G ATCAAGAGGC AGTCGTTC-G AACAGAACA- AGGGGA-TAC TG-AGTGGTT 2284
BovB_Pc-RTE-Procavia_capensis    AAGGAATCTG T-ATATG--G ATCAAGAGGC AGTCGTGC-G AACAGAACA- AGGGGA-CAC TG-CATGGTT 2286
BovB_Et-RTE-Christinus           GCA-AACTTG T-ACATG--G ATCAAGAGAC AGTGGTGG-G AATAGAACA- AAGGAA-TAC TG-AACAGCT 2060
BovB_Ta-RTE-Tachyglossus_acule   ATGGAACCTG T-ATGCC--A AACAGGAGGC TACAATAA-G AACAGAATA- TGGAGA-AAC AG-ATTGGTT 2426
BovB_Plat-RTE-Ornithorhynchus    ACGGAACCTG T-ATGAC--G AACAGGAGGC TACAATAA-G AACAGAATA- TGGAGA-AAC AG-ATTGGTT 2415
BovB_Ec-RTE-Equus_caballus       GCGTAACCTG T-ATTGT--G GACAAGAAGC CACTGTCA-G GACAGAATA- TGGAGA-GAC AG-AATGGTT 2185
BovB_Cg-RTE-Christinus           GCACAACCTA T-ACTCT--G GACAAGAAGC TACTGTTA-G GACAGAATA- TGGAGA-AAC AG-AATGGTT 1706
BovB_Bh-RTE-Bothriocroton_hydr   AGGAATCCCT TAGAAGA--A ATGGAGTAGC CTTCATAGTG AACAAGAGAG TGAAAACAGC AGTACTGGCG 739
BovB_Ml-RTE-Gehyra_variegata     ------CATT TTACATC--- -CAGCAAGGT TATACTTG-A AATTTTACTT CAGCAA---- TATGTGAATT 797
Sly-rnd1_family284-LINE/RTEBov   ---------- ---------- ---------- ---------- ---------- ---------- ---------- -
Sly-rnd5_family2019-LINE/RTEBo   TTGTTCGCTT GTG-CTTC-G GTTATCGCAT TATT-TTGTT GTTGTTACTG TTCCTT--AA TTAGTTG-CT 275
Sly-rnd3_family322-LINE/RTEBov   TTGTAT-CTT GTA-GTTT-G ATTATTGTAT TATT-TCGTA GT-ATTGTTA TGCTAT--TA TTCGCTA-TA 404
Sly-rnd5_family1698-LINE/RTEBo   ATGTTT---- -TG-ATGT-T GTTATTGTTT CTTTCTCGTA GACTTTACAC TGCTTT--TC TTATTAACTA 91
Sly-rnd1_family795-LINE/RTEBov   GAGATTCCTT ACATATTG-T GTTCTTGTTT ATTTTCTACA GA-TTCTTGT TGTAGT--AG TCATTTGATG 330
Stu-rnd-5_family-3162_LINE_RTE   GAGGAGGAGA AGC---GGAC GNNTAAGGAA GTCTATAAGA CGGCGAAGAC GGAGGCGAAG TT-AGCGGTT 2004
Sly-rnd6_family1960-LINE/RTEBo   GAGGAAGCGA AGC---GGAC GGTTAAGGAA GTCTATATGG TGGCGAAAA- -GAGGCGAAG TT-AGCGGTT 1191
Stu-rnd-5_family-2493_LINE_RTE   ATGGAGTGGC TGACTGGGTT GTTTAATGTC ATTTTTAAGA CGGCGAAAAT --GCCCGAAG ---AATGG-- 133
Sly-rnd5_family447-LINE/RTEBov   ATGGAGTGGT TGACTGGGTT GTTTAACGTC ATTTTTAGGA CGGCAAAAAC --GCCCGAAG ---AATGG-- 312
Stu-rnd-6_family-2093_LINE_RTE   ---------- ---------- ---------- ---------- ---------- ---------- ---------- -

Consensus                        AGNAA-CCTG T-ATGTG--G GTCAAGAAGC AACAGTTA-G AACCGAACA- TGGAAC-AAC TG-ATTGGTT
```

Conservation

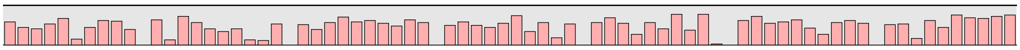

3,240 3,260 3,280

Stu-rnd-1_family-417_LINE_RTE-	AGACAGGACA	TGACGCANCT	TCA----GCT	TACCGAGGAC	ATGACCCTAG	ATAGGAAGGT	GTGGAGGTCG	219
Sly-rnd1_family377-LINE/RTEBov	AGACANGACA	TGACGCANCT	TCA----GCT	TACCGAGGAC	ATGACCCTAG	ATAGGAGGGT	GTGGAGGTCG	90
Stu-rnd-1_family-489_LINE_RTE-	AGACAGGACA	TGACGCANCT	TCA----GCT	TACCGAGGAC	ATGAC-----	----------	----------	87
Stu-rnd-1_family-607_LINE_RTE-	GCGGATGACA	TAGTACTGAT	TGACGAGACG	CGCGACGGAG	TTAACGCTAG	GTTGGAGGTT	TGGAGACAAA	517
Stu-rnd-1_family-645_LINE_RTE-	GCGGATGACA	TAGTACTGAT	TGACGAGACG	CGCGACGGAG	TTAACGCTAG	GTTGGAGGTT	TGGAGACAAA	289
Sly-rnd4_family81-LINE/RTEBovB	GCGAATGACA	TAGTTCTGAT	TGACGAGACT	CGCGACGGAG	TTAACGCTAG	GTCGGAGGTT	TGGAGACAAA	364
Stu-rnd-6_family-408_LINE_RTE-	GCAGATNATA	TTANGTTAAT	TGACGAAACT	AGCGAGGGAG	TCGACCAAAA	GCTCGAACTA	TGGAGAANCA	213
Stu-rnd-1_family-351_LINE_RTE-	TGAGATGGTT	CGGACATG--	TGAAGAGGAG	ATGCGC--GG	ACGCCCCAGT	GAGGAGGTGC	GAGAGGTTGG	538
Stu-rnd-1_family-382_LINE_RTE-	TGAGATGGTT	CGGACATG--	TGAAGAGGAG	ATGCGC--GG	ACGCCCCAGT	GAGGAGGTGC	GAGAGGTTGG	274
Sly-rnd6_family746-LINE/RTEBov	TGAGATGGTT	CGGACATG--	TGAAGAGGAG	ATGCGC--AG	ATGCCCCAGT	GAGGAGGTGT	GAGAGGTTGG	89
Stu-rnd-5_family-325_LINE_RTE-	TGAGATGGTT	TGGTCATG--	TCCTGCGTCG	ACCTCC--AG	ATGCACCGGT	CCGTAGGTGT	GAAACCATGG	157
Sly-rnd4_family509-LINE/RTEBov	TGAGATGGTT	TGGTCATG--	TCCTGCGTCG	ACCTCC--AG	ATGCACCGGT	CCGTAGGTGT	GAAACCATGG	351
Stu-rnd-1_family-513_LINE_RTE-	--AAATGGAA	TAGTGAGGAT	TCATATAGCC	GACCCC--AA	CTTGTTTGGG	ATTGAGGCGT	AGTTG-TTGT	65
Sly-rnd5_family1442-LINE/RTEBo	CGGAATGGA-	TACAGAGGAT	TCATATAGCC	GACCCC--AA	CTAATTTGGG	ATTGAGGTGT	AGTTGATTGA	110
BovB_Gl-RTE-Gehyra_lazelli	TAGCATAG-G	AAAAGGAGTA	CGACAAGGAT	GTATAT---T	GTCACCTTGC	TTATTTAATT	TATATGCAGA	2502
BovB_Gv-RTE-Gehyra_variegata	TAGCATAG-G	AAAAGGAGTA	CGACAAGGAT	GTATAT---T	GTCACCTTGC	TTATTTAATT	TATATGCAGA	2431
BovB_PMo-RTE-Python_molurus	CAAAATAG-G	GAAAGGAGTA	CGACAAGGCT	GTATAC---T	GTCACCCTGC	TTATTTAACC	TATATGCAGA	2363
BovB_Oa-RTE-Ovis_aries	CAAAATTG-G	GAAAGGAGTA	TGTCAAGGCT	GTATAT---T	GTCACCCTGC	TTATTTAACT	TATATGCAGA	2389
BovB-RTE-Bos_taurus	CCAAATAG-G	AAAAGGAGTA	CGTCAAGGCT	GTATAT---T	GTCACCCTGC	TTATTTAACT	TATATGCAGA	2987
BovB_Me-RTE-Macropus_eugenii	TAACAT----	----------	----------	----------	----------	-TATTTAACT	TATATGCCGA	1976
BovB_Ano-RTE-Amphibolurus_norr	CAAAATTG-G	GAAAGGAGTA	CGACAAGGCT	GTATAT---T	GTCCCCCAGC	TTATTTAACT	TATATGCAGA	2436
BovB_Gd-RTE-Eremiascincus_doug	TCAAATTG-G	GAAAGGAGTA	CGACAAGGCT	GTATAC---T	GTCACCCTGC	TTATTTAACT	TATATGCAGA	2024
BovB_Er-RTE-Eremiascincus_rich	TCAAATTG-G	GAAAGGAGTA	CGACAAGGCT	GTATAC---T	GTCACCCTGC	TTATTTAACT	TATATGCAGA	2559
BovB_Ca-RTE-Ctenotus_atlas	TCAAATTG-G	GAAAGGAGTA	CGACAAGGCT	GTATAC---T	GTCACCCTGC	TTATTTAACT	TATATGCAGA	2552
BovB_Es-RTE-Egernia_stokesii	TAAAATTG-G	GAAAGGAGTA	TGACAAGGCT	GTATAT---T	GTCACCCTGC	TTATTTAACT	TATATGCAGA	2387
BovB_Tr-RTE-Tiliqua_rugosa	TAAAATAG-G	GAAAGGAGTA	CAACAAGACT	GCAAAT---T	GTCACCCTGC	TTATTTAACT	TATATGCAGA	2395
BovB_ALi-RTE-Amblyomma_limbatu	CGAAATTG-C	GAAAGAAGTT	CGGCAAGGCT	GTATAC---T	GTCGCCCTTC	CTATTTAATT	TATATGCAGA	2566
BovB_Hs-RTE-Hydrophis_spiralis	CAAAATTG-G	GAAAGGAGTT	TGGCAAGGCT	GAATAC---T	GTCGCCCTGC	CTATTTAACT	TATATGCAGA	768
BovB_VA-RTE-Vipera_ammodytes	CAAAATTG-G	GAAAGGAGTC	CGGCAAGGCT	GTATAC---T	GTTGCCCTGC	CTATTTAACT	TATATGCAGA	2357
BovB_ACo-RTE-Agkistrodon_conto	CAAAATTG-G	GAAAGGAGTC	CGGCAAGGCT	GTATTC---T	GTCGCCCTGC	CTATTTAACT	TATATGCAGA	2357
BovB_Pg-RTE-Perameles_gunnii	TAAGATTG-G	AAAGGAAATA	TGCCAAGGCT	ATATAT---G	GTCATCTTAT	TTATTTAATT	TATATGCAAA	520
BovB_Io-RTE-Isoodon_obesulus	AAAGATTGTA	AAAAGGAGTA	TGACAAGGCT	GCATAT---T	ATCACATTAT	TTATTTAACT	TATATGCAGA	957
BovB_Bp-RTE-Burramys_parvus	TAAGATTG-G	AAAAGGAGTA	TGACAAGGCT	GTATAT---T	GTCACCTTAT	TTATTTAACT	TATATGCAGA	1057
BovB_Pb-RTE-Petaurus_breviceps	TAAGATTG-G	AAAAGGAGTA	CGACAAGGCT	GTATAT---T	GTCACCTTAT	TTATTTAACT	TATATGCAGA	1124
BovB_Ma-RTE-Macropus_antilopin	TAAGATTG-G	AAAAGGAGTA	TGACAAGGCT	GTATAT---T	GTCACCTTAT	TTATTTAACT	TATATGCAGA	2471
BovB_Af-RTE-Antechinus_flavipe	TCTGATTGGA	AAAAAGATTA	TGATCATATT	GTATGC---T	TTCTCTTTAT	TTAGTTAATT	TATATGTAGA	1676
BovB_Ac-RTE-Anolis_carolinensi	CAAGATTG-G	GAAAGGAGTA	CGGCAGGGCT	CCATACCTAT	TTCACCCTAC	CTATTCAACT	TGTACGCAGA	2335
BovB_BM-RTE-Bombyx_mori	TAATT-TCAG	AAGAGGAGTT	CGCCAGGGGT	GTATTC---T	GTCACCCATT	CTATTTAACA	TATACGGCGA	2203
BovB_Dr-RTE-Christinus	TGGGA-TTGA	GAAAGGTGTT	TGACAAGGAT	GCATTT---T	ATCGCCTACC	CTGTTCAATC	TGTATGCTGA	2223
BovB_La-RTE-Loxodonta_africana	TAAAG-TCAG	GAAAGGTGTG	CATCAGGGTT	GTATCC---T	TTCACCATAC	CTATCCAATC	TGTATGCTGA	2350
BovB_Pc-RTE-Procavia_capensis	TAAAG-TCAG	GAAAGGTGTG	TGTCAGGGTT	GTATCC---T	TTCACCATAC	CTTTTTAATC	TGTATGCCAA	2352
BovB_Et-RTE-Christinus	TAAAA-TCAG	AAAAGGTGGG	CGGCAGGGTT	GTATCC---T	ATCACCACAT	TTATTCAATA	AATAGGCAGA	2126
BovB_Ta-RTE-Tachyglossus_acule	TCCCA-TTAG	TAAGGGTGTA	AGACAAGGAT	GTATCT---T	ATCGCCTTAT	CTGTTCATCC	TTTACACCGA	2492
BovB_Plat-RTE-Ornithorhynchus	TCCCA-TTAG	TAAGGGTGTA	AGACAAGGAT	GTATCT---T	ATCGCCTTAT	CTGTTCATCC	TTTACACCGA	2481
BovB_Ec-RTE-Equus_caballus	TCCTA-TAGG	CAAAGGTGTC	AGACAAGGGT	GCATTT---T	ATCTCCCTAT	CTGTTTAATC	TGTACACAGA	2251
BovB_Cg-RTE-Christinus	TTCCAATTGG	CAAAGGTGTC	AGACAAGGAT	GTATTT---T	ATCTCCCTAT	CTCTTTAATC	TATATGCAGA	1773
BovB_Bh-RTE-Bothriocroton_hydr	TATAACCTCA	AAAAATGGCA	-GATTGATTT	CAATACAGGT	CCAAGGCAAA	CCCTTCAACG	GCCAC----A	804
BovB_Ml-RTE-Gehyra_variegata	CAGATTTACC	AAAA------	-GATAGGACT	GGTTTTCAAA	GTAGGCAGA-	-NAAACAAGA	GACCAAATTA	858
Sly-rnd1_family284-LINE/RTEBov	----------	----------	----------	----------	----------	----------	----------	-
Sly-rnd5_family2019-LINE/RTEBo	GTGTTTTCTT	---TACGTTG	TNTTCCCGTT	T--CGT--AG	CTGNTTCGAT	TTGTTTTACT	CGAGCCGAGG	338
Sly-rnd3_family322-LINE/RTEBov	TGTTGCTTCA	---TNTACTT	CTATTACTTT	T--CTT--NC	ATGATTGTTT	TCGTT---CT	TGAGCCGAGG	464
Sly-rnd5_family1698-LINE/RTEBo	CTATGCTTTC	---TTCATTG	TTTTC----T	T--CTT--CT	TTTACTTGAT	TTGATGCACT	TGAGCCGAAG	150
Sly-rnd1_family795-LINE/RTEBov	TCATGCCTCC	GGATGAACAA	AGACTACGTT	TACCTT--CT	CCTGATCGAC	TTAGCA-ACC	TTNCCATTCA	397
Stu-rnd-5_family-3162_LINE_RTE	ACGACGGCNA	AGACGGCAGC	TTTCGAACGC	TTGTATGTCG	AGCTGGANNN	NNNNNNNNNN	NNNNNNNNNN	2074
Sly-rnd6_family1960-LINE/RTEBo	ACGACGGCTA	AAACGACAGC	TTTTGAGCGC	TTGTACGAGG	AGCTAGGGAG	CAAAGGCGGG	GATANGAAGT	1261
Stu-rnd-5_family-2493_LINE_RTE	AGGTGGAGTA	CAATGA----	TTCCG-----	TTGTACAAGA	AC---AAGGG	TGACATCCAA	AATTGCAACA	191
Sly-rnd5_family447-LINE/RTEBov	AGGTGGAGTA	CAATGA----	TTCCG-----	TTGTACAAGA	AC---AAGGG	TGATATTCAG	AATTGCAACA	370
Stu-rnd-6_family-2093_LINE_RTE	----------	----------	----------	----------	GTGAGCTTGC	TCACATTGCC	GGTCTCAAGC	30
Consensus	TAAAATTG-G	GAAAGGAGTA	TGACAAGGCT	GTATAC---T	GTCACCCTAC	TTATTTAACT	TATATGCAGA	

Conservation 100% 0%

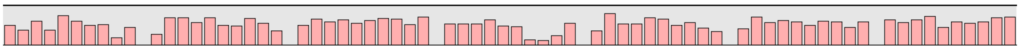

```
                                  3,300                          3,320                          3,340                          3,360
Stu-rnd-1_family-417_LINE_RTE-   CGNATTAGGG TAGAAGGTTA GTAGGTAGTC GAGTGTTGTC TTTCCTTGCG AGGGTATGGG TTGTTAGCGT 289
Sly-rnd1_family377-LINE/RTEBov   CGGATTAGGG TAGAAGGTTA GTAGGTAGTC GAGTGTTGTC TTGTT----- ---------- ---------- 135
Stu-rnd-1_family-489_LINE_RTE-   ---------- ---------- ---------- ---------- ---------- ---------- ---------- 87
Stu-rnd-1_family-607_LINE_RTE-   CCCTGGAGTC TAAAGGGTTC AGGTTGAGCA GGACCAAGAC AGAATATTTG GAGTGCAAGT TCAGTGACGC 587
Stu-rnd-1_family-645_LINE_RTE-   CCCTGGAGTC TAAAGGGTTC AGGTTGAGCA GGACCAAGAC GGAATATTTG GAGTGCAAGT TCAGTGACGC 359
Sly-rnd4_family81-LINE/RTEBovB   CTCTGGAGTC TAAAGGGTTC AGGTTGAGCA GGACCAAGAC AAAGTACTTG GAGTGTAAAT TCAGTGACGT 434
Stu-rnd-6_family-408_LINE_RTE-   CTCTAGAGAG TAAGGGTTTT AGGATAAGTA GAAGTAAGAC CGAATACATG CATTGCGAGT TTAGTCA-GC 282
Stu-rnd-1_family-351_LINE_RTE-   CTATAGTGGG TNCGAGGAGA GGTAGAGGTA GGCCGAAGAA GTATTGGGGA GAGGTGATTA --GACAGGAC 606
Stu-rnd-1_family-382_LINE_RTE-   CTATAGTGGG TNCGAGGAGA GGTAGAGGTA GGCCGAAGAA GTATTGGGGA GAGGTGATTA --GACAGGAC 342
Sly-rnd6_family746-LINE/RTEBov   CTATAGCGGG TNTNAGGAGA GGTAGAGGTA GAC------- ---------- ---------- ---------- 122
Stu-rnd-5_family-325_LINE_RTE-   TGAGTGAAGG TGTTAAAAGG G-ACGAGGTA GACCTAAAAT CACATGGAAG GAAGTTGTCT --CGAAAGAC 224
Sly-rnd4_family509-LINE/RTEBov   TGAGTGAAGG TGTTAAAAGG GGACGAGGTA GACCTAAAAT CACATGGAAG GAGGTTGTCT --CGAAAGAC 419
Stu-rnd-1_family-513_LINE_RTE-   ---------- ---------- ---------- ---------- ---------- ---------- ---------- 65
Sly-rnd5_family1442-LINE/RTEBo   TTGATACCT- ---------- ---------- ---------- ---------- ---------- ---------- 119
BovB_Gl-RTE-Gehyra_lazelli       GTACATCATG CGAAATGCCG GCCTGGACGA ATCACAAGCT GGAATT-AAG ATCGCTGGG- --AAAAACAT 2568
BovB_Gv-RTE-Gehyra_variegata     GTACATCATG CGAAATGCCG GCCTGGACGA ATCACAAGCT GGAATT-AAG ATCGCTGGG- --AAAAACAT 2497
BovB_PMo-RTE-Python_molurus      GTACATCATG AGAAATGCCA GGCTGGAAGA ATCACAAGCC GGAATT-AAG ATTGCAGGG- --AGAATCAT 2429
BovB_Oa-RTE-Ovis_aries           GTACATCATG AGAAATGCTG GGCTGGATGA AGCACAAGCT GGAATC-AAG ATTGCCAGG- --AGAAATAT 2455
BovB-RTE-Bos_taurus              GTACATCATG AGAAACGCTG GGCTGGAAGA AACACAAGCT GGAATC-AAG ATTGCCGGG- --AGAAATAT 3053
BovB_Me-RTE-Macropus_eugenii     GTATGTCATG TGAAATGCCA GACTGAGTGA ATCAAAAGCC AAAATT-AAG GTTGGTGGG- --AGAAATAT 2042
BovB_Ano-RTE-Amphibolurus_norr   ATACATCATG CGGAAGGCTG GACTGGAGGA ATCCCAAGCC GGAATT-AAG ATTGCCGGA- --AGAAATAT 2502
BovB_Gd-RTE-Eremiascincus_doug   GTACATCATG AGAAAGGCTG GGCTGGATGA TTCACAAGCT GGAATC-AAG ATTGCTGGG- --AGAAATAT 2090
BovB_Er-RTE-Eremiascincus_rich   GTACATCATG AGAAAGGCTG GGCTGGATGA TTCACAAGCT GGAATC-AAG ATTGCTGGG- --AGAAATAT 2625
BovB_Ca-RTE-Ctenotus_atlas       GTACATCATG AGAAAGGCTG GGCTGGATGA TTCACAAGCT GGAATC-AAG ATTGCTGGG- --AGAAATAT 2618
BovB_Es-RTE-Egernia_stokesii     GTACATCATG CGAAAGGCTG GGCTGGATGA ATCACAAGCT GGAGTT-AAG ATCGCCAGG- --AGAAATAT 2453
BovB_Tr-RTE-Tiliqua_rugosa       GTACATCATG CAAAAGGCTA GGCTGGATGA ATCACAAACT AGAATT-AAG ATAGCCAGG- --AGAAATAT 2461
BovB_ALi-RTE-Amblyomma_limbatu   ACACATCATG AGAAAGGCGG GGCTAGATGA ATCACCTGTC GGAATT-AAG ATCGATGGG- --AGAAATAT 2632
BovB_Hs-RTE-Hydrophis_spiralis   GCACATCATG AGAAAGGCGG GGCTAGATGA ATCAAAAGTT GGAATT-AAG ATTGCCGGG- --AGAAATAT 834
BovB_VA-RTE-Vipera_ammodytes     GCACATCATG AGAAAGGCAG GGCTAGATGA ATCAAAAGTT GGAATT-AAG ATTGCTGGA- --AGAAATAT 2423
BovB_ACo-RTE-Agkistrodon_conto   GCACATCATG AGAAAGGCAG GGCTAGATGA ATCAAAAGTT GGAATT-AAG ATTGCCGGG- --AGAAATAT 2423
BovB_Pg-RTE-Perameles_gunnii     A-ATATCATG TGAAATACCA GGCTGGACAA ATCAAAAGCT AGAAAT-AAG GTTGCCAGG- --AGAAAAAA 585
BovB_Io-RTE-Isoodon_obesulus     GTACATCATA TGAAATATCA GACTGGATGA ATCAAAAACT GGAATTTAAG GTTGACGGG- --AGAAATAT 1024
BovB_Bp-RTE-Burramys_parvus      GTACATCATG TGAAATGCCA GGCTGGATGA ATCAAAAGCC AGAATT-AAG GTTGCTAGT- --ACAAATAA 1123
BovB_Pb-RTE-Petaurus_breviceps   GTACATCATG TGAAATGCCA GGCTGGATGA ATCAAAAGCC AGAATT-AAG GTTGCCAGG- --AGAAATAT 1190
BovB_Ma-RTE-Macropus_antilopin   GTACATCATG TGAAATGCCA GGCTGGATGA ATCAAAAGCT GGAATT-AAG GTTGCCAGG- --AGAAATAT 2537
BovB_Af-RTE-Antechinus_flavipe   ACAAGTTATG AAAAATGACT GACTGGATGA AACAAAAAGC TGAAATCAAG TTTTTCAGG- --AG------ 1737
BovB_Ac-RTE-Anolis_carolinensi   ACACATCATG CGACATGCAG GGCTTGACGA ATCCAAGGCC AGAGTT-AAA ATTGCTGGA- --AGAAACAT 2401
BovB_BM-RTE-Bombyx_mori          ATACATCATG CGCAAGACC- --CTAGAAAA CTGG---GAT GGAGGC-ATA ACTATAGGT- --GACGAAGT 2263
BovB_Dr-RTE-Christinus           AGTGGTCATG AGGCTATCCA ATCTGGACGA GTCACCAATC GGAGTT-AAG ATCGGCGGG- --AGAAACAT 2289
BovB_La-RTE-Loxodonta_africana   GCAAATAATC CGAGAAGCTG GACTATATGA AGAAGAACGG GGCATC-AGG ATTGGAGGA- --AGACTCAT 2416
BovB_Pc-RTE-Procavia_capensis    GCAAATAATC CGAGAAGCTG GATTATATGA AGAAGAACGA GGCATC-AGG ATTGGAGGA- --TGACTCAT 2418
BovB_Et-RTE-Christinus           GCAAATCATC AGAAAAGCTG AATTACATAA AGAAGAAGAT GACATC-AGG ATTGGAGGA- --AGGCTTAT 2192
BovB_Ta-RTE-Tachyglossus_acule   ATACATAATG AGAGAAGCCG GATTGGATGA AGACAAACGG GGAGTA-AAG ATTGGAGGA- --AGGAATAT 2558
BovB_Plat-RTE-Ornithorhynchus    ATACCTAATG AGAGAAGCCG GATTGGATGA AGACGAACGG GGAGTA-AAG ATTGGAGGA- --AGGAATAT 2547
BovB_Ec-RTE-Equus_caballus       ACATATCATA CAAAAATCTG GGCTAGACTC AGATGAAGGA GGAGTG-AAA ATTGGTGGA- --AGAAATAT 2317
BovB_Cg-RTE-Christinus           ACATATCATA AGAAAAGCTG GATTAGATTT AGATGAAGGT GGAGTG-AAA ATTGGTGGAT --AGGAAAAT 1840
BovB_Bh-RTE-Bothriocroton_hydr   GTAGTCCAAG CCTACGCCCC AACCAC-TAA TGCTGAAGAC GAAG----AA GTTGGCGGG- ---------T 859
BovB_Ml-RTE-Gehyra_variegata     GTACATCATG AAAAATGCCA AGCT----GA ATCAAAAGCT ATTATT-AAA GTTGTTGGG- --AGAAATAT 920
Sly-rnd1_family284-LINE/RTEBov   ---------- ---------- ---------- ---------- ---------- ------TACA CTCTACCCTC 14
Sly-rnd5_family2019-LINE/RTEBo   GTCTATCGGA AACAGCCTCT CTA---CCTC CG--CGAGGT AGGGGTAAGG TCTGCGTACA CTCTACCCTC 403
Sly-rnd3_family322-LINE/RTEBov   GTCTATCAGA AACAACCTCT CTA---CCTT T---CAAGGT AGGGGTAAGG TCTGCGTACA CNCTACCCTC 528
Sly-rnd5_family1698-LINE/RTEBo   ATCTTTCGAA AATAATCTTT CTA---ATTC TACACGAGAT AATGATAAAA TTTGCGTACA TTTTATCTTC 217
Sly-rnd1_family795-LINE/RTEBov   TTTAATCGAT GACATTCTAA GTAGGTTGTC TTTTCGAGAT GTTGTAAGAA CTAGTACACT TTCGAAGGAC 467
Stu-rnd-5_family-3162_LINE_RTE   NNNNNNNNNN NNNNNNNNNN NNNNNNNNNN NNNNNNNNNN NNNNNNNNNN NNNNNNNNNN NNNNNNNNNN 2144
Sly-rnd6_family1960-LINE/RTEBo   TGTACAGGCT AGCCAAGGCG AGAGAGAGAG GGCTCGNGAC CTGGACCAAG TGAAG----- ---------- 1316
Stu-rnd-5_family-2493_LINE_RTE   ACTATAGGGG TATCAAGCTG CTAA------ ---------- ---------- ---------- ---------- 215
Sly-rnd5_family447-LINE/RTEBov   ACTATAGGGG TATCAAGCTG TTAAGTCACA CTATGAAAGT TTGGGAGAGG GTGGTGGAGA TGAGGGTGAG 440
Stu-rnd-6_family-2093_LINE_RTE   CCGGATAAAG GAGGAGGGTT GCGGTAGGCT GACAGCCANC GTAAAACTAG TCAATTCATG --ATGAATCC 98

Consensus                        GTACATCATG AGAAAGGCTG GGCTGGATGA ATCACAAGCT GGAATT-AAG ATTGCNGGG- --AGAAATAT
```

Conservation

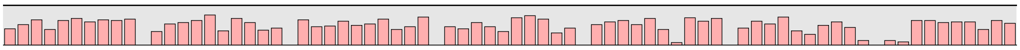

```
                                     3,380                          3,400                          3,420
Stu-rnd-1_family-417_LINE_RTE-     TTGTAGTAGT CCTAGCC--- ---------- ---------- ---------- ---------- ---------- 306
Sly-rnd1_family377-LINE/RTEBov     ---------- ---------- ---------- ---------- ---------- ---------- ---------- 135
Stu-rnd-1_family-489_LINE_RTE-     ---------- ---------- ---------- ---------- ---------- ---------- ---------- 87
Stu-rnd-1_family-607_LINE_RTE-     GACGCATGAG GCGGACGTGG AAGTGAGGCT TGACGCACAG GTCATCCCCA AGAGAGAAAG TTTCAAGTAT 657
Stu-rnd-1_family-645_LINE_RTE-     GACGCATGAG GCGGACGTGG AAGTGAGGCT TGACGCACAG GTCATCCCCA AGAGAGAAAG TTTCAAGTAT 429
Sly-rnd4_family81-LINE/RTEBovB     GACGTATGAA G---ACGTGG AAGTGAGGCT CGATACACAA GTCATCCCCA AGAGAGGAAG TTTCAAGTAT 501
Stu-rnd-6_family-408_LINE_RTE-     ATGAGAAGAA T--GAAGTTG AGGTGAGACT AGACGGGATT GCGGTGCCTA AATGCAAACA ATTCAGATAT 350
Stu-rnd-1_family-351_LINE_RTE-     ATG------- ---------- ---------- ---------- ---------- ---------- ---------- 609
Stu-rnd-1_family-382_LINE_RTE-     ATGGC----- ---------- ---------- ---------- ---------- ---------- ---------- 347
Sly-rnd6_family746-LINE/RTEBov     ---------- ---------- ---------- ---------- ---------- ---------- ---------- 122
Stu-rnd-5_family-325_LINE_RTE-     CTACAATCTN ---GGCACAA TGGAAGAAAA AGATCTATAT AGGCGATACC AATTAGTTGG GANTANGTTT 291
Sly-rnd4_family509-LINE/RTEBov     CTACAATCTC TT-GGAATCC ACGCAGACTT AGCGAAAGAT AGGGCATATG GA--AGAAAA AGATCTATAT 486
Stu-rnd-1_family-513_LINE_RTE-     ---------- ---------- ---------- ---------- ---------- ---------- ---------- 65
Sly-rnd5_family1442-LINE/RTEBo     ---------- ---------- ---------- ---------- ---------- ---------- ---------- 119
BovB_Gl-RTE-Gehyra_lazelli         CAACAATCT- ----CAGATA T-GCAGACGA TACCACTCTT ATGGCA-GAA AGTGAAGAGG ACC----TAA 2627
BovB_Gv-RTE-Gehyra_variegata       CAACAATCT- ----CAGATA T-GCAGACGA TACCACTCTT ATGGCA-GAA AGTGAAGAGG ACC----TAA 2556
BovB_PMo-RTE-Python_molurus        CAACAACCT- ----CAGATA T-GCAGACGA TACCACCCTA ATGGCA-GAA AGTGAAGAAG AAC----TAA 2488
BovB_Oa-RTE-Ovis_aries             CAACAACCT- ----CAGATA T-GCAGATGA CACCACCCTA ATGGCA-GAA AGTGAAGAGG AAC----TAA 2514
BovB-RTE-Bos_taurus                CAATAACCT- ----CAGATA T-GCAGATGA CACCACCCTT ATGGCA-GAA AGTGAAGAGG AAC----TAA 3112
BovB_Me-RTE-Macropus_eugenii       CAACAATTT- ----CACTTT T-ACAGATGA TACCATTCTG ATGGCA-GAA AGTGAAGAAA AAT----TAA 2101
BovB_Ano-RTE-Amphibolurus_norr     CAACAACCT- ----CAGATA T-GCAGATGA TACCACTCTG ATGGCA-GAA AGTGAGGAGG AAT----TAA 2561
BovB_Gd-RTE-Eremiascincus_doug     CAACAACCT- ----CAGATA T-GCAGACGA TACCACTCTA ATGGCA-GAA AGTGAGGAAG ACC----TAA 2149
BovB_Er-RTE-Eremiascincus_rich     CAACAACCT- ----CAGATA T-GCAGACGA TACCACTCTC ATGGCA-GAA AGTGAGGAAG ACC----TCA 2684
BovB_Ca-RTE-Ctenotus_atlas         CAACAACCT- ----CAGATA T-GCAGACGA TACCACTCTA ATGGCA-GAA AGTGAGGAAG ACC----TAA 2677
BovB_Es-RTE-Egernia_stokesii       CAATAACCT- ----CAGATA T-GCAGATGA TACCACTCTA ATGGCA-GAA AGTGAGGAGG ATC----TAA 2512
BovB_Tr-RTE-Tiliqua_rugosa         CAATAACCT- ----CAGATA T-GCAGATGA TACCACTCTA ATGGCA-GAA AATGAGGAGG ATCCTCATAA 2524
BovB_ALi-RTE-Amblyomma_limbatu     TAACAACCT- ----CACATA T-GCAGATGA TACCACGCTC ATGGCA-AAA AGTGAAGAGG AAC----TAA 2691
BovB_Hs-RTE-Hydrophis_spiralis     CAACAACCT- ----CAGATA T-GCAGATGA TACCACTCTA ATGGCAGAAA AGTGAAGAGG AAC----TAA 894
BovB_VA-RTE-Vipera_ammodytes       CAACAACCT- ----AAGATA T-GCAGATGA TACCACTCTA ATGGCA-GAA AGTGAAGAAG AAC----TAA 2482
BovB_ACo-RTE-Agkistrodon_conto     CAACAACCT- ----AAGATA T-GCAGATGA TACCACTCTA ATGGCA-GAA AGTGAAGAGG AAC----TAA 2482
BovB_Pg-RTE-Perameles_gunnii       AAATAATTT- ----TAGATA A-GCAGATAA TACCACTCTA ATGGCA-GAA AGTGAAGAAG AAT----TAA 644
BovB_Io-RTE-Isoodon_obesulus       TAACAATCTC ---ATAGATA T-GCAGATGA TACCACTCTG ATAGCA-GAA AGTGAAGAAG AAT----TAA 1085
BovB_Bp-RTE-Burramys_parvus        TAATAATTT- ----TAGATA T-TAATAAAA TAAAACTCTA ATGGCA-GAA AGTAAAGAAA AAT----TAA 1182
BovB_Pb-RTE-Petaurus_breviceps     CAACAATCT- ----CAGATA T-GCAGATGA TACCACTCTG ATGGCA-GAA AGTGAAGAAG AAT----TAA 1249
BovB_Ma-RTE-Macropus_antilopin     CAACAATCT- ----CAGATA T-GCAGATGA TACCACTCTG ATGGCA-GAA AGTGAAGAGG AAT----TAA 2596
BovB_Af-RTE-Antechinus_flavipe     ---TAAATT- ---------- --------GA TACCACTCTA ATGGCT-GAA CAGAAAGAAG AAT----TAA 1780
BovB_Ac-RTE-Anolis_carolinensi     TAACAACCT- ----TAGGTA T-GCAGACGA TACCACTCTG ATGGCT-GAA AGTGAGGAAG AGC----TGA 2460
BovB_BM-RTE-Bombyx_mori            GACGAACCT- ----GCGCTA C-GCAGATGA CACCACTCTG CTTGCC-ACA ATAGAAGCAG AGA----TGA 2322
BovB_Dr-RTE-Christinus             CAACAACCT- ----TAGTTA C-ACCGATGA TACGACCCTG ATTGCA-GAA AGTGAAAAGG ACC----TTG 2348
BovB_La-RTE-Loxodonta_africana     TAACAACCT- ----GCGATA T-GCAGATGA CACAACCTTG CTTGCT-GAA AGTGAAGAGG ACT----TGA 2475
BovB_Pc-RTE-Procavia_capensis      TAACAACCT- ----GCGTTA T-GCAGATGA CACCACCTTA CTTGCT-GGA AATGAAGAAG ATT----TGA 2477
BovB_Et-RTE-Christinus             TAACAACCC- ----GCGATA T-GCAGACGA CACAACTTTG CTTGCT-GAA AGTAAAGAAG ACT----TAA 2251
BovB_Ta-RTE-Tachyglossus_acule     AAACAACCT- ----TCGTTA T-GCTGATGA TACTACCCTA CTAGCA-GAA AGTGAAGAAG ATT----TGA 2617
BovB_Plat-RTE-Ornithorhynchus      AAACAACCT- ----TCGTTA T-GCTGATGA TACAACCCTA CTAGCA-GAA AGTGAAGAAG ATT----TGA 2606
BovB_Ec-RTE-Equus_caballus         CAACAATCT- ----AAGATA T-GCAGATGA CACCATCTTA CTGGCA-GAA AGCAGCAATG ACT----TGA 2376
BovB_Cg-RTE-Christinus             TAATAATTT- ----AAGATA T-GCAGATGA CACCACGTTA CTGGCA-GAA AACAGTGAAG ACT----TGA 1899
BovB_Bh-RTE-Bothriocroton_hydr     CAACAACCT- ----CAGATA T-GCAGACGA CACCACCCCA ATGGCA-GAA AGGAAGAAGA ACC----TAG 918
BovB_Ml-RTE-Gehyra_variegata       CAACAATTT- ----AAGATA TAGCAAATAA TACCACTTTA ATAATA-GAA AGTGAAAAAG AAT----TAA 980
Sly-rnd1_family284-LINE/RTEBov     CCCAGACCC- -----CACTT -GTGGGATTA CACTGGGTAT GTTGTTGTT- ---------- ---------- 56
Sly-rnd5_family2019-LINE/RTEBo     CCCAGACCC- -----CGCTT -GTGGGATTA CACTGGGTAT GTTGTTGTTA AATAGATGGC GTGATCTATT 466
Sly-rnd3_family322-LINE/RTEBov     CCCAGACCC- -----CACTT TGTGGGATTA CACTGGGTAT GTTGTT---- ---------- ---------- 568
Sly-rnd5_family1698-LINE/RTEBo     TCCAGACCT- -----CACTG ---------- ---------- ---------- ---------- ---------- 231
Sly-rnd1_family795-LINE/RTEBov     TGGCAATAT- -----ANTTG CCGNAGAATT CCACANGTGA AATTTGATCA AACGGTNTGG AAAACACCAG 531
Stu-rnd-5_family-3162_LINE_RTE     NNNNNNNNNN NNNNNNNNNN NNNNNNNNNN NNNNNNNNNN NNNNNNNNNN NNNNNNNNNN NNNNNNNNNN 2214
Sly-rnd6_family1960-LINE/RTEBo     ---------- ---------- ---------- ---------- ---------- ---------- ---------- 1316
Stu-rnd-5_family-2493_LINE_RTE     ---------- ---------- ---------- ---------- ---------- ---------- ---------- 215
Sly-rnd5_family447-LINE/RTEBov     GAGGTGTGTN TATTTCCGAG AACCAGTTCG GATTCATGCC GGGGCGATCG ACTACGGAAG CCATTCATCT 510
Stu-rnd-6_family-2093_LINE_RTE     TCACAAT--- ---------- ---------- ---------- ---------- ---------- ---------- 105
Consensus                          CAACAACCT- ----CAGATA T-GCAGATGA TACCACTCTA ATGGCA-GAA AGTGAAGAAG AAT----TAA
```

Conservation

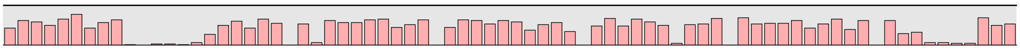

```
                                    3,440                  3,460                  3,480                  3,500
Stu-rnd-1_family-417_LINE_RTE-      ---------- ---------- ---------- ---------- ---------- ---------- ---------- 306
Sly-rnd1_family377-LINE/RTEBov      ---------- ---------- ---------- ---------- ---------- ---------- ---------- 135
Stu-rnd-1_family-489_LINE_RTE-      ---------- ---------- ---------- ---------- ---------- ---------- ---------- 87
Stu-rnd-1_family-607_LINE_RTE-      CTTGGG---T CTATAAT--- ---------- ---------- ---------- ---------- ---------- 671
Stu-rnd-1_family-645_LINE_RTE-      CTTGGG---T C--------- ---------- ---------- ---------- ---------- ---------- 437
Sly-rnd4_family81-LINE/RTEBovB      CTTGGG---T CTATGATCCA AGGAAATGGG GAGATCGACG ATGATGTCAC ACATCGTATT GGTGCGGGGT 568
Stu-rnd-6_family-408_LINE_RTE-      CTAGGC---T CGTTGTTTCA AGAGAATGGA ATGATAGATG AAGATG---- ---------- ---------- 393
Stu-rnd-1_family-351_LINE_RTE-      ---------- ---------- ---------- ---------- ---------- ---------- ---------- 609
Stu-rnd-1_family-382_LINE_RTE-      ---------- ---------- ---------- ---------- ---------- ---------- ---------- 347
Sly-rnd6_family746-LINE/RTEBov      ---------- ---------- ---------- ---------- ---------- ---------- ---------- 122
Stu-rnd-5_family-325_LINE_RTE-      TAGTCATGTT AGTACGTTTA CTTTAGGTCC TATGCTTTCT AGGAGTCTTT TAGTCCTGTC AGAGATTTNT 361
Sly-rnd4_family509-LINE/RTEBov      AGGCGATACC TACTAGTTGG GAATANGTTT TAGTCATGTT AGAGAACTTC TAATATTGTT ----ACTGTT 552
Stu-rnd-1_family-513_LINE_RTE-      ---------- ---------- ---------- ---------- ---------- ---------- ---------- 65
Sly-rnd5_family1442-LINE/RTEBo      ---------- ---------- ---------- ---------- ---------- ---------- ---------- 119
BovB_Gl-RTE-Gehyra_lazelli          AGGGC----C TCCTGCTGAG GGTGAAGG-A GGAGAGCGCA AAAGTTGG-C TT-GAAACTC AA-------- 2682
BovB_Gv-RTE-Gehyra_variegata        AGAGC----C TCCTGCTGAG GGTGAAGG-A GGAGAGCGCA AAAGTTGG-C TT-GAAACTC AA-------- 2611
BovB_PMo-RTE-Python_molurus         AGAGC----C TCTTGATGAG GGTGAAAG-A GGAGAGTGCA AAAGCTGG-C TT-GAAACTC AA-------- 2543
BovB_Oa-RTE-Ovis_aries              AGAGC----C TCTTGATGAA AGTGAAAG-A GGAGAGTGAA AAAGCTGG-C TT-AAAACTC AA-------- 2569
BovB-RTE-Bos_taurus                 AAAGC----C TCTTGATGAA AGTGAAAG-T GGAGAGTGAA AAAGTTGG-C TT-AAAGCTC AA-------- 3167
BovB_Me-RTE-Macropus_eugenii        GAAGC----C TCTTGA---- ------AG-C GG-------- AAAGCTGG-C TT-AAAGCTT AA-------- 2138
BovB_Ano-RTE-Amphibolurus_norr      AGAAC----C TTGTAATGAG GGTGAAAG-A GGAGAGTGCA AAAAACGG-T CT-GAAACTC AA-------- 2616
BovB_Gd-RTE-Eremiascincus_doug      AGAAC----C TTCTGATGAG GGTGAAAG-A GGAAAGTGCC AAAATTGGAC TT-GAAGCTC AA-------- 2205
BovB_Er-RTE-Eremiascincus_rich      AGAAC----C TTCTGATGAG GGTGAAAG-A GGAAAGTGCC AAAATTGG-C TT-GAAGCTC AA-------- 2739
BovB_Ca-RTE-Ctenotus_atlas          AGAAC----C TTCTGATGAG GGTGAAAG-A GGAAAGCGCC AAAATTGG-C TT-GAAGCTC AA-------- 2732
BovB_Es-RTE-Egernia_stokesii        AGAAC----C TCTTGATGAG GGTGAAGG-A GGAAAGTGCA AAATGTGG-C TT-GAAACTT AA-------- 2567
BovB_Tr-RTE-Tiliqua_rugosa          AGAAC----C TCTTGATGAG GGTGAAAG-A GGAAAGTGCA AAATGTGG-C TT-GAAACTT AA-------- 2579
BovB_ALi-RTE-Amblyomma_limbatu      AGAGC----C TCTTGATGCG GGTGAAGGGA GGAGAGTGCA GAAGTTGACC T--GAAACTC AA-------- 2747
BovB_Hs-RTE-Hydrophis_spiralis      AGAGC----C TCTTGATGAT GGTGAAGA-A GGAGAGTGAA AAAGTTGGCT TAAGAAACTC AA-------- 951
BovB_VA-RTE-Vipera_ammodytes        AGAGC----C TCTTGTTGCG GGTGAAGA-A GGAGAGTGCA AAACTTGGGT T--GAAACTT AA-------- 2537
BovB_ACo-RTE-Agkistrodon_conto      AGAGC----C TCTTGATGCG GGTGAAGA-A GGAGAGTGCA AAAGTTGGCT T--GAAACTC AA-------- 2537
BovB_Pg-RTE-Perameles_gunnii        GAAGT----C TCTTCATAAG AGTTAAAG-A AGAGAATTTA AAA--CATAC AT-TAAACTT AA---AAATT 703
BovB_Io-RTE-Isoodon_obesulus        GAAGT----C TCTTGATGAN GGTGAAAG-A GGAAAGTGTA AAAGTTGTAC TT-AAAGTTT AACATAAAAT 1149
BovB_Bp-RTE-Burramys_parvus         GAAGC----C TCTTGATGAA AGTGAAAG-A GGAGAGTGTA AAAGCTGGCT TG-AAGACTT AA-------- 1238
BovB_Pb-RTE-Petaurus_breviceps      GAAGC----C TCTTGATGAG GGTGAAAG-A GGAGAGTGTA AAAGCTGGCT TG-AAG-CTT AA-------- 1304
BovB_Ma-RTE-Macropus_antilopin      GAAGC----C TCTTGATGAG GGTGAAAG-A GGAGAGTGTA AAAGCTGGCT TG-AAG-CTT AA-------- 2651
BovB_Af-RTE-Antechinus_flavipe      AAAAAAAAAC TCCTGATGAG TGTGACAG-A GGAGAGTATA AAAA-TAATT TT-CAAGTTT AA-------- 1839
BovB_Ac-RTE-Anolis_carolinensi      GGAGC----C CTATCACCAA GGTGAAAG-A AGAAAGTGCA AAAGCCGGGC T--GCAGTTA AA-------- 2515
BovB_BM-RTE-Bombyx_mori             CAGAG----C TTCTGAACAG GATGGAGC-A CATAGGGTTG GAGATGGGAC TC--GCCCTC AA-------- 2377
BovB_Dr-RTE-Christinus              AATAT----C TGGTCCGAAG AGTAAAAG-A AGAAAGTGAA CACATGGGAC TG--TACCTC AA-------- 2403
BovB_La-RTE-Loxodonta_africana      AGCAC----T TACTGATGAA GATCAAAG-A CCACAGCCTT CAGTATGGAT TA--CACCTC AA-------- 2530
BovB_Pc-RTE-Procavia_capensis       AACAC----T TACTGATGAA GATCAAGG-A CCACAGCCTT CAGTATGGAT TG--CACCTC AA-------- 2532
BovB_Et-RTE-Christinus              AGAAC----T TGCTGATGAA GATCAAGG-A CTGCAGCCTT CAGTCTGGAT TA--CAAATC AA-------- 2306
BovB_Ta-RTE-Tachyglossus_acule      AGGGC----T TATTATTAAA AGTTAAGG-A ACAGAGCAAA AAGATGGGCC TA--TATTTG AA-------- 2672
BovB_Plat-RTE-Ornithorhynchus       AGGGC----T TATTATTAAA AGTTAAGG-A ACAGAGCAAA AAGATGGGCC TA--CATTTG AA-------- 2661
BovB_Ec-RTE-Equus_caballus          AACAA----C TTCTGATGAA AGTGAAAG-A AGAAAGTGCC AAAGCAGGAC TG--CATTTG AA-------- 2431
BovB_Cg-RTE-Christinus              AACAA----C TACTGATGAA GGTTAAAG-C AGAAAGTGCC AAAGCAGGAC TA--CATTTG AT-------- 1954
BovB_Bh-RTE-Bothriocroton_hydr      AGAAC----C TTATGATGAG GGTGAAAG-A GGAAAGGGCG AAAATTGGTT TG--AAGATC AA-------- 973
BovB_Ml-RTE-Gehyra_variegata        GAAGC----T TTTTAATAAG AATCAAAT-A ACACAGTGTA AAAATTGATT TA--AATTTT AATATAAAAC 1043
Sly-rnd1_family284-LINE/RTEBov      ---------- ---------- ---------- ---------- ---------- ---------- ---------- 56
Sly-rnd5_family2019-LINE/RTEBo      GTGGCATCTA G--------- ---------- ---------- ---------- ---------- ---------- 477
Sly-rnd3_family322-LINE/RTEBov      ---------- ---------- ---------- ---------- ---------- ---------- ---------- 568
Sly-rnd5_family1698-LINE/RTEBo      ---------- ---------- ---------- ---------- ---------- ---------- ---------- 231
Sly-rnd1_family795-LINE/RTEBov      AGGACTT--- ---------- ---------- ---------- ---------- ---------- ---------- 538
Stu-rnd-5_family-3162_LINE_RTE      NNNNNNNNNN NNNNNNNNNN NNNNNNNNNN NNNNNNNNNN NNNNNNNNNN NGGCATCACA ATATTAATGT 2284
Sly-rnd6_family1960-LINE/RTEBo      ---------- ---------- ---------- ---------- ---------- ---------- ---------- 1316
Stu-rnd-5_family-2493_LINE_RTE      ---------- ---------- ---------- ---------- ---------- ---------- ---------- 215
Sly-rnd5_family447-LINE/RTEBov      CGTAAGGAGA CTGGTGGAGN AGTATAGGGA GAGAAGAGGA ACTTACACAT GGTG------ ---------- 564
Stu-rnd-6_family-2093_LINE_RTE      ---------- ---------- ---------- ---------- ---------- ---------- ---------- 105

Consensus                           AGAGC----C TCTTGATGAG GGTGAAAG-A GGA-AGTG-A AAA--TGG-C T---AA-CT- AA--------
```

Conservation (100% / 0%)

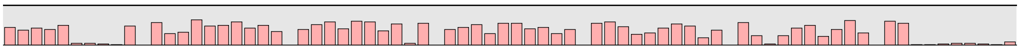

3,520 3,540 3,560

Stu-rnd-1_family-417_LINE_RTE-	---------- ---------- ---------- ---------- ---------- ---------- ----------	306
Sly-rnd1_family377-LINE/RTEBov	---------- ---------- ---------- ---------- ---------- ---------- ----------	135
Stu-rnd-1_family-489_LINE_RTE-	---------- ---------- ---------- ---------- ---------- ---------- ----------	87
Stu-rnd-1_family-607_LINE_RTE-	---------- ---------- ---------- ---------- ---------- ---------- ----------	671
Stu-rnd-1_family-645_LINE_RTE-	---------- ---------- ---------- ---------- ---------- ---------- ----------	437
Sly-rnd4_family81-LINE/RTEBovB	GGATGAAATG GAGGCTCGCA TCCGGCGTCT TGTGTGATAA GAAGGTGCCG CCGAAACTTA AAGGTAAGTT	638
Stu-rnd-6_family-408_LINE_RTE-	---------- ---------- ---------- ---------- ---------- ---------- ----------	393
Stu-rnd-1_family-351_LINE_RTE-	---------- ---------- ---------- ---------- ---------- ---------- ----------	609
Stu-rnd-1_family-382_LINE_RTE-	---------- ---------- ---------- ---------- ---------- ---------- ----------	347
Sly-rnd6_family746-LINE/RTEBov	---------- ---------- ---------- ---------- ---------- ---------- ----------	122
Stu-rnd-5_family-325_LINE_RTE-	ATGCCAGTAG AAAATATAGA GAACTTCTAG TAC-TTTTTA TTTTTATTTT GTATAATATT GGCCTGAGAT	430
Sly-rnd4_family509-LINE/RTEBov	ATNTTTTTAC A---CGTGTT GAACTTATTT TTCACTTTTA TTTTTATTTT GTATAATATT GGCNTGAGAT	619
Stu-rnd-1_family-513_LINE_RTE-	---------- ---------- ---------- ---------- ---------- ---------- ----------	65
Sly-rnd5_family1442-LINE/RTEBo	---------- ---------- ---------- ---------- ---------- ---------- ----------	119
BovB_Gl-RTE-Gehyra_lazelli	CATCA----- ------AAAA AACTAA---- --------GA TCATGGCATC CAGCCCCAT- TACTCCTTGG	2728
BovB_Gv-RTE-Gehyra_variegata	CATCA----- ------AAAA AACTAA---- --------GA TCATGGCATC CAGCCCCAT- TACTCCTTGG	2657
BovB_PMo-RTE-Python_molurus	CATTA----- ------AAAA AACTAA---- --------GA TCATGGCATC TGGACCCAT- CAATTCCTGG	2589
BovB_Oa-RTE-Ovis_aries	CATTAAAAAA -AAAACAAAA AACTAA---- --------GA TCATGGCATC TGGTCCCAT- CACTTCATGG	2625
BovB-RTE-Bos_taurus	CATT------ -----CAGAA AACGAA---- --------GA TCATGGCATC CGGTCCCAT- CACTTCATGG	3213
BovB_Me-RTE-Macropus_eugenii	CATTA----- -----AAAAA CCTTAA---- --------AA TCACAACAAC TGGTCCTAT- CATTTCCTGG	2185
BovB_Ano-RTE-Amphibolurus_norr	CATC------ -----AAAAA AACTAA---- --------GA TCATGGCCAC GGGTCCCAT- CACCTCCTGG	2662
BovB_Gd-RTE-Eremiascincus_doug	CATTA----- -----AAAAA AACTAA---- --------GA TCATGGCCAC CACTCCCAT- CACTTCCTGG	2252
BovB_Er-RTE-Eremiascincus_rich	CATTA----- -----AAAAA AACTAA---- --------GA TCATGGCAAC CACTCCCAT- CACTTCCTGG	2786
BovB_Ca-RTE-Ctenotus_atlas	CATTA----- -----AAAAA A-CTAA---- --------GA TCATGGCAAC CACTCCCAT- CACTTCCTGG	2778
BovB_Es-RTE-Egernia_stokesii	CATTA----- -----AAAAA A-CTAA---- --------GA TCATGGCATC CGGTCCCAT- CACCTCATGG	2613
BovB_Tr-RTE-Tiliqua_rugosa	CATTA----- -----AAAAA AACTAA---- --------GA TCATGGCATC CGGTCCCAT- CACCTCATGG	2626
BovB_ALi-RTE-Amblyomma_limbatu	CATTA----- ------AGAA AACTAA---- --------GA TCATGGCATC CAGTCCTCT- CACTTCCTGG	2793
BovB_Hs-RTE-Hydrophis_spiralis	CATTA----- ------AGAA AACTAA---- --------GA TCATGGCATC TGGCCCTCT- CAATTCCTGG	997
BovB_VA-RTE-Vipera_ammodytes	CATTA----- ------AAAA AACCAA---- --------GA TCATGGCATC CAACCCTCT- CAATTCCTGG	2583
BovB_ACo-RTE-Agkistrodon_conto	CATTA----- ------AGAA AACCAA---- --------GA TCATGGCATC CGGCCCTCT- CAATTCCTGG	2583
BovB_Pg-RTE-Perameles_gunnii	TAAAAAAACC TAAAATTAAA AACAAA---- --------AA TCTTGGCAAC TGGTCCCAT- CACTTCCTGG	760
BovB_Io-RTE-Isoodon_obesulus	TACAAAAACA -ACAATTACA AATAAACAAA TCCTAATAGA TCTTGGCAAC TGGTCTAATT CACTTCCTAG	1218
BovB_Bp-RTE-Burramys_parvus	CAAAAAAA-- -------AAC CACTAA---- --------GA TCTTGGCAAC TGGTCTCAT- CACTTCCTGG	1286
BovB_Pb-RTE-Petaurus_breviceps	CATCAAAA-- -------AAA -ACTAA---- --------GA TCATGGCAAC TGGTCCCAT- CACTTCCTGG	1351
BovB_Ma-RTE-Macropus_antilopin	CATAAAAA-- --------AA AACTAA---- --------GA TCTTGGCAAC TGGTCCCAT- CACTTCCTGG	2698
BovB_Af-RTE-Antechinus_flavipe	CATCAAAAC- -------AAA AACAAACTAA --------GA TCATAGCAAT TGATCCCAT- AATTTTCTGG	1892
BovB_Ac-RTE-Anolis_carolinensi	CATCA----- ------AGAA AACCAA---- --------GA CCATGGCAAC CACACCGAC- TGATAACTGG	2561
BovB_BM-RTE-Bombyx_mori	CAG-A----- -----TCCAA AACTAA---- --------AA TCATGGTCGT TGATCGCA-C AAAAAAGTTG	2423
BovB_Dr-RTE-Christinus	CATCA----- -----AAAAA AACGAA---- --------GG TAATGACAGT AGCATGCAGT GGTACAGTCC	2451
BovB_La-RTE-Loxodonta_africana	CAT-A----- -----AAGAA AACAAA---- --------AA TCCTCACAAC TGGACCAATA AGCAACATCA	2577
BovB_Pc-RTE-Procavia_capensis	CAT-C----- -----AAGAA AACAAA---- --------AA TCCTCACAAC TGGACCAGTA AGCAACATTG	2579
BovB_Et-RTE-Christinus	TGC-A----- -----AACAA GACCAA---- --------AA GCCTCACAAC TGGACCAATA GGTAACATCA	2353
BovB_Ta-RTE-Tachyglossus_acule	TGT-C----- -----AAGAA AACAAA---- --------GA TCATGACAAC TGGAAGTTTT AACACATTTG	2719
BovB_Plat-RTE-Ornithorhynchus	TGT-C----- -----AAGAA AACAAA---- --------GA TCATGACAAC TGGAAGTTTT AACACATTTG	2708
BovB_Ec-RTE-Equus_caballus	CAT-C----- -----AAGAA GACAAA---- --------AA TCATGACTAC AGAAGAACTA CACAACTTTA	2478
BovB_Cg-RTE-Christinus	CAT-C----- -----AAGAA GACAAA---- --------AG TCATGAATAC AGAAAAATTA CACAACTTAA	2001
BovB_Bh-RTE-Bothriocroton_hydr	CATAA----- ------AAAA AACTAA---- --------GA TCATGGCAAC CACTCCAGT- CACTTCCTGG	1019
BovB_Ml-RTE-Gehyra_variegata	AACAGCCCCC CCAAAAAAAA AATTAA---- --------GA TTATGTTAAC TAATTTTAT- CACTTTCTGA	1100
Sly-rnd1_family284-LINE/RTEBov	---------- ---------- ---------- ---------- ---------- ---------- ----------	56
Sly-rnd5_family2019-LINE/RTEBo	---------- ---------- ---------- ---------- ---------- ---------- ----------	477
Sly-rnd3_family322-LINE/RTEBov	---------- ---------- ---------- ---------- ---------- ---------- ----------	568
Sly-rnd5_family1698-LINE/RTEBo	---------- ---------- ---------- ---------- ---------- ---------- ----------	231
Sly-rnd1_family795-LINE/RTEBov	---------- ---------- ---------- ---------- ---------- ---------- ----------	538
Stu-rnd-5_family-3162_LINE_RTE	TGTGTATTGA GGTTGCGCCG AGGAGGTTAT ACGGGCTATT AGTAGGATGA GCAGGGGAAG AGCGACCGGA	2354
Sly-rnd6_family1960-LINE/RTEBo	---------- ---------- ---------- ---------- ---------- ---------- ----------	1316
Stu-rnd-5_family-2493_LINE_RTE	---------- ---------- ---------- ---------- ---------- ---------- ----------	215
Sly-rnd5_family447-LINE/RTEBov	---------- ---------- ---------- ---------- ---------- ---------- ----------	564
Stu-rnd-6_family-2093_LINE_RTE	---------- ---------- ---------- ---------- ---------- ---------- ----------	105
Consensus	CAT-A----- ------AAAA AAC-AA---- --------GA TCATGGCAAC -GGTCC-AT- CACTTCCTGG	

Conservation
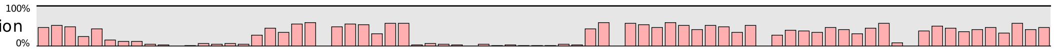

```
                                     3,580                  3,600                  3,620                  3,640
Stu-rnd-1_family-417_LINE_RTE-    ----------  ----------  ----------  ----------  ----------  ----------  ----------  306
Sly-rnd1_family377-LINE/RTEBov    ----------  ----------  ----------  ----------  ----------  ----------  ----------  135
Stu-rnd-1_family-489_LINE_RTE-    ----------  ----------  ----------  ----------  ----------  ----------  ----------  87
Stu-rnd-1_family-607_LINE_RTE-    ----------  ----------  ----------  ----------  ----------  ----------  ----------  671
Stu-rnd-1_family-645_LINE_RTE-    ----------  ----------  ----------  ----------  ----------  ----------  ----------  437
Sly-rnd4_family81-LINE/RTEBovB    CTATAGAGTG  GTGGTTAGAC  CGACTATGTT  GTATGGGGCG  GAGTGTTGGC  CAGTCAAGAA  CTCNCACGTT  708
Stu-rnd-6_family-408_LINE_RTE-    ----------  ----------  ----------  ----------  ----------  ----------  ----------  393
Stu-rnd-1_family-351_LINE_RTE-    ----------  ----------  ----------  ----------  ----------  ----------  ----------  609
Stu-rnd-1_family-382_LINE_RTE-    ----------  ----------  ----------  ----------  ----------  ----------  ----------  347
Sly-rnd6_family746-LINE/RTEBov    ----------  ----------  ----------  ----------  ----------  ----------  ----------  122
Stu-rnd-5_family-325_LINE_RTE-    GAGCTTAAAT  GGAGAA----  ----------  ----------  ----------  ----------  ----------  446
Sly-rnd4_family509-LINE/RTEBov    GAGCTTAAAT  GGAGCGACAT  GGTCAGTGAG  GATTCATATA  GCCGACCCCA  ACTTGTTTGG  GACTGAGGCG  689
Stu-rnd-1_family-513_LINE_RTE-    ----------  ----------  ----------  ----------  ----------  ----------  ----------  65
Sly-rnd5_family1442-LINE/RTEBo    ----------  ----------  ----------  ----------  ----------  ----------  ----------  119
BovB_Gl-RTE-Gehyra_lazelli        C-AAAT----  TGATGGGGAG  GTCATGGAAA  CCGTGACAGA  CTTCACGTT-  -CCTAGGATC  CAA-AATCAC  2790
BovB_Gv-RTE-Gehyra_variegata      C-AAAT----  TGACGGGGAG  GTCATGGAAA  CCGTGACAGA  CTTCACGTT-  -CCTAGGATC  CAA-AATCAC  2719
BovB_PMo-RTE-Python_molurus       C-AAAT----  AGAAGGGGTA  GAAATGGAAG  CAGTAACAGA  CTTCATTTA-  -TTTGGGCTC  CAA-GATCAC  2651
BovB_Oa-RTE-Ovis_aries            C-AAAT----  AGATGGGGAA  AAAATGGAAA  CAGTGACAGA  CTTTATTTT-  -CTTGGGCTC  CAA-AATCAC  2687
BovB-RTE-Bos_taurus               G-AAAT----  AGATGGGGAA  ACAGTGGAAA  CAGTGTCAGA  CTTTATTTT-  -TCTGGGCTC  CAA-AATCAC  3275
BovB_Me-RTE-Macropus_eugenii      C-AAAT----  AGAGGGAGAA  GAAATGGAAG  CAATGTCATA  TTTTGTATT-  -CTTGGGCTC  AAA-GATCAG  2247
BovB_Ano-RTE-Amphibolurus_norr    G-AAAT----  AGAAGGGGAA  GACATGGAGG  CAGTGACAGA  TTTTATTTT-  -CCTGGGCTC  CAT-GATCAC  2724
BovB_Gd-RTE-Eremiascincus_doug    C-AAAT----  AGAAGGGGAA  GAAATGGAAG  CAGTGACAGA  GTTTATTTT-  -CCTGGGCTC  CAA-GATCAC  2314
BovB_Er-RTE-Eremiascincus_rich    C-AAAT----  AGAAGGGGAA  GAAATGGAAG  CAGTGACAGA  GTTTATTTT-  -CCTGGGCTC  CAA-GATCAC  2848
BovB_Ca-RTE-Ctenotus_atlas        C-AAAT----  AGAAGGGGAA  GAAATGGAAG  CAGTGACAGA  CTTTATTTT-  -CCTGGGCTC  CAA-GATCAC  2840
BovB_Es-RTE-Egernia_stokesii      C-AAAT----  AGAAGGGGAA  GAAATGGAAG  CCGTGACAGA  GTTTATCTT-  -CTTGGGCTC  CAA-GATCAC  2675
BovB_Tr-RTE-Tiliqua_rugosa        C-AAAT----  AGAAGGGGAA  GAAATGGAAG  CTGTGACAGA  GTTTATCTT-  -CTTGGGCTC  CAA-GATCAC  2688
BovB_ALi-RTE-Amblyomma_limbatu    C-AAAT----  AGATGGGGAA  TAAATAGAGG  TAGTGACAGA  TTTTATTTTG  TCTTTGGCTC  CAA-GATCAT  2857
BovB_Hs-RTE-Hydrophis_spiralis    C-AAAT----  AGATGGGGAA  GAAATAGAGG  TAGTGACAGA  TTTTATTTT-  -CCTGGGCTC  CAA-GATCAC  1059
BovB_VA-RTE-Vipera_ammodytes      C-AAAT----  AGATGGGGAA  GAAATGGAGG  TAGTGACAGA  TTTTATTTT-  -TCTGGGCTC  CAA-GATCAC  2645
BovB_ACo-RTE-Agkistrodon_conto    C-AAAT----  AGATGGGGAA  GAAATGGAGG  TAGTGACAGA  TTTTATCTT-  -CCTGGGCTC  CAA-GATCAC  2645
BovB_Pg-RTE-Perameles_gunnii      A-AAAT----  TGAGAGAAAA  AACATGGAAA  CAGTGTCAGA  TTTTATA--T  TCTTAGGCTC  AAA-GATCAC  822
BovB_Io-RTE-Isoodon_obesulus      GCAAAT----  AGAAAGAGAA  GAAATGGAAG  CAGTGTCAGA  TTTTATAAAT  TCTTAGACTC  AAA-GATCAC  1283
BovB_Bp-RTE-Burramys_parvus       C-AAAT----  AGAGGGAGAA  GAAATGGAAA  CAGTGTCAGA  TTTTATA--T  TCTTGAGCTC  AAA-AATCAA  1348
BovB_Pb-RTE-Petaurus_breviceps    C-AAAT----  AGAGGGAGAA  GAAATGGAAG  CAGTGTCAGA  TTTTATA--T  TCTTGGATTC  AAA-GATCAC  1413
BovB_Ma-RTE-Macropus_antilopin    C-AAAT----  AGAGGGAGAA  GAAATGGAAG  CAGTGTCAGA  TTTTATA--T  TCTTGGGCTC  AAA-GATCAC  2760
BovB_Af-RTE-Antechinus_flavipe    C-AAAT----  AAAAGGAAAA  TAAATGTAAG  CAGTATCAGA  TTTTATA--T  TCTTGTGCTC  AAA-GATCAC  1954
BovB_Ac-RTE-Anolis_carolinensi    C-AAAT----  AGAGGAAGAA  AACGTGGAGG  CAGTGACAGA  CTTTATATT-  -TCTAGGCAC  AAA-GATCAC  2623
BovB_BM-RTE-Bombyx_mori           GAGCTTAGTG  GAACACTAA-  -ACCTTGAGC  TTGTTGACAA  CTTTATTTA-  -CCTAGGCTC  CAA-CATTAA  2488
BovB_Dr-RTE-Christinus            ACATAACAAT  TGACAATGAG  GAAATCGAAT  CAGTCCAAGA  CTTCATCTT-  -ACTTGGATC  CAA-GATCGA  2518
BovB_La-RTE-Loxodonta_africana    TGAT------  AAACGGAGAA  AAGATTGAAG  TTGTCAAGGA  TTTCATTTT-  -ACTTGGAGC  CAC-AATCAA  2638
BovB_Pc-RTE-Procavia_capensis     TCAT------  AAATGGAGAA  AGGATTGAGG  TTGTCAAGGA  TTTCATTTT-  -ACTTGGGTC  CAC-AATCAA  2640
BovB_Et-RTE-Christinus            CGAT------  AAATGGAGAA  AAGGTTGAAA  TGGTCAAGAA  TTTTGCCTT-  -GCTTAGATC  CAC-AATCAA  2414
BovB_Ta-RTE-Tachyglossus_acule    TAGT------  GGATGGAGAG  AAGATTGAAA  TAGTTGACAA  TTTTTCTCT-  -CCTGGGATC  GAT-AATCAA  2780
BovB_Plat-RTE-Ornithorhynchus     TAGT------  GGACGGAGAG  AAGATTGAAA  TAGTTGACAA  TTTTTCTCT-  -CCTGGGATC  GAT-AATCAA  2769
BovB_Ec-RTE-Equus_caballus        ACAT------  AGACAATGAA  GACATTGAAA  TTGTTAAAGA  TTTTGTTTA-  -CCTTGGTTC  AGT-CATCAA  2539
BovB_Cg-RTE-Christinus            ACAT------  AGATAAAGAA  GAAATTGAAA  TTGTTCAAAA  TTTTCTATA-  -CCTAGGCTC  CAC-TATCAA  2062
BovB_Bh-RTE-Bothriocroton_hydr    C-AAAT----  AGAATGGGAA  GAAATGGAAG  CAGTGACATA  ATTTAACTT-  -CTTGGGCTC  CAA-GATCAC  1081
BovB_Ml-RTE-Gehyra_variegata      TAAATA----  GAAGTAGAAG  AAGATAGGAG  AAATATAAAA  TTTTATATT-  -ATTAGGTTC  AAACAATCAC  1164
Sly-rnd1_family284-LINE/RTEBov    ----------  ----------  ----------  ----------  ----------  ----------  ----------  56
Sly-rnd5_family2019-LINE/RTEBo    ----------  ----------  ----------  ----------  ----------  ----------  ----------  477
Sly-rnd3_family322-LINE/RTEBov    ----------  ----------  ----------  ----------  ----------  ----------  ----------  568
Sly-rnd5_family1698-LINE/RTEBo    ----------  ----------  ----------  ----------  ----------  ----------  ----------  231
Sly-rnd1_family795-LINE/RTEBov    ----------  ----------  ----------  ----------  ----------  ----------  ----------  538
Stu-rnd-5_family-3162_LINE_RTE    CCCGACGAGA  TCCCGGTGGA  ATTTTGGAAG  AGCACGGACA  AGGCAGGTNT  GGAGTGGTTG  ACTGGGTTGT  2424
Sly-rnd6_family1960-LINE/RTEBo    ----------  ----------  ----------  ----------  ----------  ----------  ----------  1316
Stu-rnd-5_family-2493_LINE_RTE    ----------  ----------  ----------  ----------  ----------  ----------  ----------  215
Sly-rnd5_family447-LINE/RTEBov    ----------  ----------  ----------  ----------  ----------  ----------  ----------  564
Stu-rnd-6_family-2093_LINE_RTE    ----------  ----------  ----------  ----------  ----------  ----------  ----------  105

Consensus                         C-AAAT----  AGA-GG-GAA  GAAATGGAAG  CAGTGACAGA  -TTTAT-T--  -C-TGGGCTC  CAA-GATCAC
```

Conservation

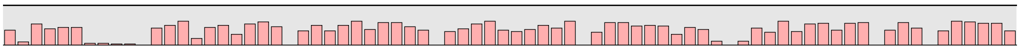

```
                                          3,660                           3,680                           3,700
Stu-rnd-1_family-417_LINE_RTE-    ---------- ---------- ---------- ---------- ---------- ---------- ---------- 306
Sly-rnd1_family377-LINE/RTEBov    ---------- ---------- ---------- ---------- ---------- ---------- ---------- 135
Stu-rnd-1_family-489_LINE_RTE-    ---------- ---------- ---------- ---------- ---------- ---------- ---------- 87
Stu-rnd-1_family-607_LINE_RTE-    ---------- ---------- ---------- ---------- ---------- ---------- ---------- 671
Stu-rnd-1_family-645_LINE_RTE-    ---------- ---------- ---------- ---------- ---------- ---------- ---------- 437
Sly-rnd4_family81-LINE/RTEBovB    CAGAAGATGA AAGTNGCGGA AATGAGGATG TTGAGATGGA TGTGTGGGCA TACTAGGAGA GATAAGATTA 778
Stu-rnd-6_family-408_LINE_RTE-    ---------- ---------- ---------- ---------- ---------- ---------- ---------- 393
Stu-rnd-1_family-351_LINE_RTE-    ---------- ---------- ---------- ---------- ---------- ---------- ---------- 609
Stu-rnd-1_family-382_LINE_RTE-    ---------- ---------- ---------- ---------- ---------- ---------- ---------- 347
Sly-rnd6_family746-LINE/RTEBov    ---------- ---------- ---------- ---------- ---------- ---------- ---------- 122
Stu-rnd-5_family-325_LINE_RTE-    ---------- ---------- ---------- ---------- ---------- ---------- ---------- 446
Sly-rnd4_family509-LINE/RTEBov    TAGTTGTTGT AGGCTAGAAG CAATGCTTGA CCTTAGAATG TGATAGAAGA GGAC------ ---------- 743
Stu-rnd-1_family-513_LINE_RTE-    ---------- ---------- ---------- ---------- ---------- ---------- ---------- 65
Sly-rnd5_family1442-LINE/RTEBo    ---------- ---------- ---------- ---------- ---------- ---------- ---------- 119
BovB_Gl-RTE-Gehyra_lazelli        TGCT-GATGG TGACTGCAGC C--------- ------TT-G AAATTAAAAG ACGCCTGCTC ------CTT- 2836
BovB_Gv-RTE-Gehyra_variegata      TGCT-GATGG TGACTGCAGC C--------- ------TT-G AAATTAAAAG ACGCCTGCTC ------CTT- 2765
BovB_PMo-RTE-Python_molurus       CGCA-GACGG AGACTGCAGC C--------- ------AT-G AAATTAAAAG ACGCTTGCTC ------CTT- 2697
BovB_Oa-RTE-Ovis_aries            TGCA-GATGG TGACTGCAGC C--------- ------AT-G AAATTAAAAG ACGCTTGCTC ------CTT- 2733
BovB-RTE-Bos_taurus               TGCA-GATGG TGACTGCAGC C--------- ------AT-G AAATTAAAAG ACGCTTACTC ------CTT- 3321
BovB_Me-RTE-Macropus_eugenii      TGGA-GGTAG TGACTGAAAT T--------- ------AT-G AAATTAAAAG ATACTTGCTC ------CTT- 2293
BovB_Ano-RTE-Amphibolurus_norr    TGCA-GATGG AGACAGCAGC C--------- ------AC-G AAATTAAAAG ACGCCTGCTT ------CTT- 2770
BovB_Gd-RTE-Eremiascincus_doug    CGCA-GATGG TGACTGCAGC C--------- ------AT-G AAATTAAAAG ACGTTTACTT ------CTT- 2360
BovB_Er-RTE-Eremiascincus_rich    CGCA-GATGG TGACTGCAGC C--------- ------AT-G AAATTAAAAG ACGTTTACTT ------CTT- 2894
BovB_Ca-RTE-Ctenotus_atlas        CGCA-GATGG TGACTGCAGC C--------- ------AT-G AAATTAAAAG ACGTTTACTT ------CTT- 2886
BovB_Es-RTE-Egernia_stokesii      TGCA-GATGG AGACTGCAGC C--------- ------AT-G AAATTAAAAG ACGCTTGCTT ------CTT- 2721
BovB_Tr-RTE-Tiliqua_rugosa        TGCA-GACGG AGATTGCAGC C--------- ------AT-G AAATTAAAAG ACGCTTGCTT ------CTT- 2734
BovB_ALi-RTE-Amblyomma_limbatu    CGCA-GATGG CGACTACAGC C--------- ------AA-G AAATTAAAAG ACGCTTGCTC ------CT-- 2902
BovB_Hs-RTE-Hydrophis_spiralis    TGCA-GATGG AGACTACAGC C--------- ------AA-G AAATTAAAAG ATGCTTGCTC ------CTTG 1106
BovB_VA-RTE-Vipera_ammodytes      TGCA-GATGG GGACTGTAGT C--------- ------AA-G AAATTAAAAG ACGCACGCTC ------CT-- 2690
BovB_ACo-RTE-Agkistrodon_conto    C-CA-GATGG GGACTGCAGC C--------- ------AA-G AAATTAAAAG ACGCTTGCTC ------CT-- 2689
BovB_Pg-RTE-Perameles_gunnii      TGCAAGATGG TGACTGTAGT C--------- ------AT-G AAATTAAAAG ACATTTGCTC ------CTT- 869
BovB_Io-RTE-Isoodon_obesulus      TGCA-GATGG TGACTGCAGC C--------- ------ATAG AAATTTAAAG ATGCTTGCTC ------CTT- 1330
BovB_Bp-RTE-Burramys_parvus       TGCA-GATAG TAACTACATC CTAAAATTGT TACCTAAT-G AAATTAAAAG ACACTTGTTT TCCTGGCTT- 1415
BovB_Pb-RTE-Petaurus_breviceps    TGCA-GATGG TGACTACAGC C--------- ------AT-G AAATTAAAAG ATGCTTGCTC ------CTT- 1459
BovB_Ma-RTE-Macropus_antilopin    TGCA-GATGG TGACTGCAGC C--------- ------AT-G AAATTAAAAG ATGCTTGCTC ------CTT- 2806
BovB_Af-RTE-Antechinus_flavipe    TGTA-GATGG TAACTATAGC C--------- ------AT-G AAATTAAAAC ATACTTATTA ------TTT- 2000
BovB_Ac-RTE-Anolis_carolinensi    TGCA-GATGC AGACTGCAGC C--------- ------AG-G AAATCAGAAG ACATTTACTT ------CTT- 2669
BovB_BM-RTE-Bombyx_mori           CAAT-ACC-- TGATCCAG-- ---------- ---------- ---------- ------CGAA -------CCT 2510
BovB_Dr-RTE-Christinus            CCAC-AATGG TGAGTCAGGG C--------- -------CGG AGATCAGACG AAGAATCGCA -------CTT 2564
BovB_La-RTE-Loxodonta_africana    CAGC-CATGG AAACAGCAGT C--------- -------AAG AAATCAAAAG ACGCATTGCA -------TTG 2684
BovB_Pc-RTE-Procavia_capensis     TGCC-CATGG AAGCAGCAGT C--------- -------AAG AAATCAAAAG ATGCATTGCA -------TTG 2686
BovB_Et-RTE-Christinus            TGCT-CATGG AAGCGGCAGT C--------- -------AAG AGATCAAA-G ACATGTTGCA -------TTA 2459
BovB_Ta-RTE-Tachyglossus_acule    TAAT-AAAGG AACTAGTAGC C--------- -------AAG AAATATGCCG AAGATTAATG -------TTA 2826
BovB_Plat-RTE-Ornithorhynchus     TAAT-AAAGG AACTAGTAGC C--------- -------AAG AAATACGCCG AAGATTAATG -------TTA 2815
BovB_Ec-RTE-Equus_caballus        TTTA-AATGG AGACTGCAGC C--------- -------AAG AAATCAAGAG AAGACTGAGA -------CTC 2585
BovB_Cg-RTE-Christinus            ACAA-AAGGG AGACTGCAAC C--------- -------GAG AAATCAGAAG GAGACTGAGA -------CTG 2108
BovB_Bh-RTE-Bothriocroton_hydr    CACA-GATGG TGACTTCAGC C--------- -------CTG AAATTAAAAG GCGTTTGCTT -------CTT 1127
BovB_Ml-RTE-Gehyra_variegata      TACA-AATAA TTACTACAAC C--------- ------ATGA AATTTTAAAA ATGTTTGTTA -------CAT 1211
Sly-rnd1_family284-LINE/RTEBov    ---------- ---------- ---------- ---------- ---------- ---------- ---------- 56
Sly-rnd5_family2019-LINE/RTEBo    ---------- ---------- ---------- ---------- ---------- ---------- ---------- 477
Sly-rnd3_family322-LINE/RTEBov    ---------- ---------- ---------- ---------- ---------- ---------- ---------- 568
Sly-rnd5_family1698-LINE/RTEBo    ---------- ---------- ---------- ---------- ---------- ---------- ---------- 231
Sly-rnd1_family795-LINE/RTEBov    ---------- ---------- ---------- ---------- ---------- ---------- ---------- 538
Stu-rnd-5_family-3162_LINE_RTE    TTAATGTCAT TTTTAAGACG GCNAAGATGC CCGAAGAATG GAGGTGGAGT ACAATGGTTC CGTTGTACAA 2494
Sly-rnd6_family1960-LINE/RTEBo    ---------- ---------- ---------- ---------- ---------- ---------- ---------- 1316
Stu-rnd-5_family-2493_LINE_RTE    ---------- ---------- ---------- ---------- ---------- ---------- ---------- 215
Sly-rnd5_family447-LINE/RTEBov    ---------- ---------- ---------- ---------- ---------- ---------- ---------- 564
Stu-rnd-6_family-2093_LINE_RTE    ---------- ---------- ---------- ---------- ---------- ---------- ---------- 105
Consensus                         TGCA-GATGG -GACTGCAGC C--------- ---------G AAATTAAAAG A-G-TTGCT- -------TT-
```

Conservation

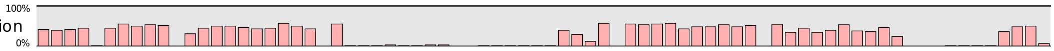

```
                                  3,720                3,740                3,760                3,780
Stu-rnd-1_family-417_LINE_RTE-    ---------- ---------- ---------- ---------- ---------- ---------- ---------- 306
Sly-rnd1_family377-LINE/RTEBov    ---------- ---------- ---------- ---------- ---------- ---------- ---------- 135
Stu-rnd-1_family-489_LINE_RTE-    ---------- ---------- ---------- ---------- ---------- ---------- ---------- 87
Stu-rnd-1_family-607_LINE_RTE-    ---------- ---------- ---------- ---------- ---------- ---------- ---------- 671
Stu-rnd-1_family-645_LINE_RTE-    ---------- ---------- ---------- ---------- ---------- ---------- ---------- 437
Sly-rnd4_family81-LINE/RTEBovB    GGAACGAAGA TATTCGGGAC AAGGTGGGAG TGGCCTCCGT GGCGGACAAG ATGAGGAAGC GAGGTTGAGA 848
Stu-rnd-6_family-408_LINE_RTE-    ---------- ---------- ---------- ---------- ---------- ---------- ---------- 393
Stu-rnd-1_family-351_LINE_RTE-    ---------- ---------- ---------- ---------- ---------- ---------- ---------- 609
Stu-rnd-1_family-382_LINE_RTE-    ---------- ---------- ---------- ---------- ---------- ---------- ---------- 347
Sly-rnd6_family746-LINE/RTEBov    ---------- ---------- ---------- ---------- ---------- ---------- ---------- 122
Stu-rnd-5_family-325_LINE_RTE-    ---------- ---------- ---------- ---------- ---------- ---------- ---------- 446
Sly-rnd4_family509-LINE/RTEBov    ---------- ---------- ---------- ---------- ---------- ---------- ---------- 743
Stu-rnd-1_family-513_LINE_RTE-    ---------- ---------- ---------- ---------- ---------- ---------- ---------- 65
Sly-rnd5_family1442-LINE/RTEBo    ---------- ---------- ---------- ---------- ---------- ---------- ---------- 119
BovB_Gl-RTE-Gehyra_lazelli        GGGAGGAAAA GCGATGGCAA AACCTCAACA GCATAATAAA -------AA- GTAGGGACAT C-ACCCTGCC 2897
BovB_Gv-RTE-Gehyra_variegata      GGGAGGAAA- GCGATGGCAA A-CCTCAACA GCATAATAAA -------AA- GTAGGGACAT C-ACCCTGCC 2824
BovB_PMo-RTE-Python_molurus       GGGAGGAAA- GCTATGGCAA A-CCTAGACA GCATATTAAA -------AA- GCAGAGACAT C-ACCTTGCC 2756
BovB_Oa-RTE-Ovis_aries            GGAAGAAAA- GCTATGACCA A-CCTAGACA GCATATTAAA -------AA- GCAGAGACAT T-ACTTTGCC 2792
BovB-RTE-Bos_taurus               GGAAGGAAA- GTTATGACCA A-CCTAGATA GCATATTCAA -------AA- GCAGAGACAT T-ACTTTGCC 3380
BovB_Me-RTE-Macropus_eugenii      GGAAGGAAG- --TATAGCAA A-TTTTGACA GCGTACTAAA -------AA- GCAGAGACGT C-ACCTTACT 2350
BovB_Ano-RTE-Amphibolurus_norr    GGGAGGAAA- GCGATGACAA A-TCTTGACA GCATCTTAAA -------AA- GCAGAGACAT C-ACCTTGCC 2829
BovB_Gd-RTE-Eremiascincus_doug    GGGAGGAAA- GCTATGGCGA A-TCTAGACA ACTTAATGAA -------AA- GCCGAGACAT C-ACCCTGCC 2419
BovB_Er-RTE-Eremiascincus_rich    GGGAGGAAA- GCTATGGCGA A-CCTAGACA ACTTAATAAA -------AA- GCCGAGACAT C-ACCCTGCC 2953
BovB_Ca-RTE-Ctenotus_atlas        GGGAGGAAA- GCTATGGCGA A-CCTAGACA ACTTAATAAA -------AA- GCCGAGACAT C-ACCCTGCC 2945
BovB_Es-RTE-Egernia_stokesii      GGGAGGAAA- GCTATGGTAA G-CCTAGACA ACTTAATTAA -------AA- GCCGAGACAT C-ACCTTGTC 2780
BovB_Tr-RTE-Tiliqua_rugosa        GGGAGGAAA- GCTATGGTAA G-CCTAGACA ACTTAATGAA -------AA- GCCGAGACAT C-ACCTTGTC 2793
BovB_ALi-RTE-Amblyomma_limbatu    GCGGAGGAAA TCTATGGCAA A-TTTAAATA GCATACTAAA -------AA- GCAGGGACAT C-ACCCTGCC 2962
BovB_Hs-RTE-Hydrophis_spiralis    GGGAGGAAAA GCTATGGCAA A-TCTAGACA GCATACTAAA -------AA- GCAGAGACAT CCACCCTGCC 1167
BovB_VA-RTE-Vipera_ammodytes      CAGGAGGAAA GCCATGGCAA A-TCTAGACA GCACACTAAA -------AA- GCAGAGACAT C-ACCCTGTC 2750
BovB_ACo-RTE-Agkistrodon_conto    GGGGAGGAAA GCTATGGCAA A-TCTAGACA GCATACTAAA -------AA- GCAGAGACAT C-ACCCTGCC 2749
BovB_Pg-RTE-Perameles_gunnii      GGAAGGAAA- ACTATGGCAA A-TCTGGACA GCATATCAAA -------AAA GCAGAGACAT C-ACCTTGCC 929
BovB_Io-RTE-Isoodon_obesulus      GGAAGGAAA- GCAATGGCAA A-TCTGGACA ACACACTAAA -------AAA GCAGAAACAT T-AACTTGCT 1390
BovB_Bp-RTE-Burramys_parvus       GGAAGGAAA- ACTATGGCAA A-TCAGGACA GCATACTAAA -------AA- GCAGAGACAT C-ACCTTGCT 1474
BovB_Pb-RTE-Petaurus_breviceps    GGAAGGAAA- GCTATGGCAA A-TCTGAACA GCATACTAAA -------AAT GCAGAGATAT A-ACCTTGCC 1519
BovB_Ma-RTE-Macropus_antilopin    GGAAGGAAA- GCTATGGCAA A-TCTGGACA GCATACTAAA -------AA- GCAGAGACAT C-ACCTTGCC 2865
BovB_Af-RTE-Antechinus_flavipe    AAAAGAAAA- GCTATGGCAA A-TATGAACA GCATACTAAA -------AA- GCAGAAACAT C-ATCTTTTT 2059
BovB_Ac-RTE-Anolis_carolinensi    GGGAAGAGA- GCAATGGCCA A-CCTCGACA AAATAGTGAA -------AA- GCAAAGACAT C-ACACTGGC 2728
BovB_BM-RTE-Bombyx_mori           GA----AGTA -CAGCTC--- -----GGTAA AA-TCTGGAA -------GG- ACCATAACAT T-ACCCAGAA 2557
BovB_Dr-RTE-Christinus            GGCCGCAGTG -CAATGCAAG G-AATGGTGA AGGTATGGAA -------AA- GCAAGGGCAT C-AGCATTGT 2623
BovB_La-RTE-Loxodonta_africana    GGCAAATCTG -CTGCAAAAG A-ACTCTTCA AAGTGTTGAA -------AA- GCAAAGACGT C-ACCTTGAA 2743
BovB_Pc-RTE-Procavia_capensis     GGAAAATCTG -CTGCAAAGG A-TCTCTTTA AAGTATTGAA -------AA- GTAAAGATGT C-ACCTTGAA 2745
BovB_Et-RTE-Christinus            GGTAAATCCA -CCACACACG G-TCTCGTTA GAGTACTGAA -------AA- G----GATCT T-ACTTCGAG 2514
BovB_Ta-RTE-Tachyglossus_acule     GGAAGACTTG -CTATGAAGA G-CCTGGAAA AAGTCATGAA -------AT- GTGCTGATGT A-ACAATTGC 2885
BovB_Plat-RTE-Ornithorhynchus     GGAAGACTTG -CTATGAAGA G-CCTGGAAA AAGTCATGAA -------AT- GTGCTGATGT A-ACAATTGC 2874
BovB_Ec-RTE-Equus_caballus        AGAAGGGCAG -CAATGAAAG A-ATTAGGAA AGATCATCAA -------GT- GTAAGGAAGT G-TCATTAGA 2644
BovB_Cg-RTE-Christinus            GGAAGGGCAG GCAATGAAAA A-CTTAGAAA AGATTCTGAA -------GT- GTAAGGATGT G-TCGCTGCA 2168
BovB_Bh-RTE-Bothriocroton_hydr    GGGAGAAAA- GCTATGGCGA A-CCTAGAAA ACTTAATAAA CTTAATAAAA GTCGAGACAT C-ACCCTCCC 1194
BovB_Ml-RTE-Gehyra_variegata      GGAAGGAAA- GCTATGGCAA A-TCTGGACA ATATATTAAC -------AA- GCAATGATAT C-ATCATGCT 1270
Sly-rnd1_family284-LINE/RTEBov    ---------- ---------- ---------- ---------- ---------- ---------- ---------- 56
Sly-rnd5_family2019-LINE/RTEBo    ---------- ---------- ---------- ---------- ---------- ---------- ---------- 477
Sly-rnd3_family322-LINE/RTEBov    ---------- ---------- ---------- ---------- ---------- ---------- ---------- 568
Sly-rnd5_family1698-LINE/RTEBo    ---------- ---------- ---------- ---------- ---------- ---------- ---------- 231
Sly-rnd1_family795-LINE/RTEBov    ---------- ---------- ---------- ---------- ---------- ---------- ---------- 538
Stu-rnd-5_family-3162_LINE_RTE    GAACAAGGGT GATATCCAAA ATTGCAACAA CTATAGGGGT ATCAAGTTGT TAA------- ---------- 2547
Sly-rnd6_family1960-LINE/RTEBo    ---------- ---------- ---------- ---------- ---------- ---------- ---------- 1316
Stu-rnd-5_family-2493_LINE_RTE    ---------- ---------- ---------- ---------- ---------- ---------- ---------- 215
Sly-rnd5_family447-LINE/RTEBov    ---------- ---------- ---------- ---------- ---------- ---------- ---------- 564
Stu-rnd-6_family-2093_LINE_RTE    ---------- ---------- ---------- ---------- ---------- ---------- ---------- 105
Consensus                         GG-AGGAAA- -CTATGGCAA A--CT-GACA -C-TA-T-AA -------AA- GCA--GACAT C-ACC-TG--
```

Conservation

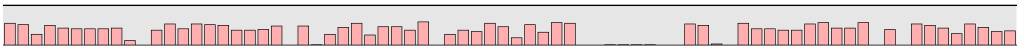

Sequence	3,800 · 3,820 · 3,840	
Stu-rnd-1_family-417_LINE_RTE-	---------- ---------- ---------- ---------- ---------- ---------- ----------	306
Sly-rnd1_family377-LINE/RTEBov	---------- ---------- ---------- ---------- ---------- ---------- ----------	135
Stu-rnd-1_family-489_LINE_RTE-	---------- ---------- ---------- ---------- ---------- ---------- ----------	87
Stu-rnd-1_family-607_LINE_RTE-	---------- ---------- ---------- ---------- ---------- ---------- ----------	671
Stu-rnd-1_family-645_LINE_RTE-	---------- ---------- ---------- ---------- ---------- ---------- ----------	437
Sly-rnd4_family81-LINE/RTEBovB	TGGTTCGGAC TCC------- ---------- ---------- ---------- ---------- ----------	861
Stu-rnd-6_family-408_LINE_RTE-	---------- ---------- ---------- ---------- ---------- ---------- ----------	393
Stu-rnd-1_family-351_LINE_RTE-	---------- ---------- ---------- ---------- ---------- ---------- ----------	609
Stu-rnd-1_family-382_LINE_RTE-	---------- ---------- ---------- ---------- ---------- ---------- ----------	347
Sly-rnd6_family746-LINE/RTEBov	---------- ---------- ---------- ---------- ---------- ---------- ----------	122
Stu-rnd-5_family-325_LINE_RTE-	---------- ---------- ---------- ---------- ---------- ---------- ----------	446
Sly-rnd4_family509-LINE/RTEBov	---------- ---------- ---------- ---------- ---------- ---------- ----------	743
Stu-rnd-1_family-513_LINE_RTE-	---------- ---------- ---------- ---------- ---------- ---------- ----------	65
Sly-rnd5_family1442-LINE/RTEBo	---------- ---------- ---------- ---------- ---------- ---------- ----------	119
BovB_Gl-RTE-Gehyra_lazelli	---AACAAAA GT----CCGT ATGGTCAAAG -CAATGGTC- ---------- ------TTCC CAGTAGTGAT	2942
BovB_Gv-RTE-Gehyra_variegata	---AACAAAA GT----TCGT ATGGTCAAAG -CAATGGTC- ---------- ------TTCC CAGTAGTGAT	2869
BovB_PMo-RTE-Python_molurus	---GACAAAG AT----CCGC ATAATCAAAG -CTATGGTT- ---------- ------TTTC CAGTAGTAAT	2801
BovB_Oa-RTE-Ovis_aries	---AACAAAG GT----CCAT ATAGTCAAAG -CTATGGTT- ---------- ------TTTC CAGTAGTCAT	2837
BovB-RTE-Bos_taurus	---AACAAAG GT----CCGT CTAGTCAAGG -CTATGGTT- ---------- ------TTTC CTGTGGTCAT	3425
BovB_Me-RTE-Macropus_eugenii	---AACAAAT GT----ACAT ATAGTTAAAG -TGATGATC- ---------- ------TTTC TAGTAGACAC	2395
BovB_Ano-RTE-Amphibolurus_norr	---AACAAAA GT----CCGA ATAGTCAAAG -CTATGGTT- ---------- ------TTTC CTGTAGTGAT	2874
BovB_Gd-RTE-Eremiascincus_doug	---AACAAAG GT----TCGC ATAGTCAAAG -CGATGGTT- ---------- ------TTCC CAGTAGCAAT	2464
BovB_Er-RTE-Eremiascincus_rich	---AACAAAG GT----TCGC ATAGTCAAAG -CTATGGTT- ---------- ------TTCC CAGTAGCAAT	2998
BovB_Ca-RTE-Ctenotus_atlas	---AACAAAG GT----TCGC ATAGTCAAAG -CTATGGTT- ---------- ------TTCC CAGTAGCAAT	2990
BovB_Es-RTE-Egernia_stokesii	---AACAAAG GT----CCGT ATAGTCAAAG -CTATGGTT- ---------- ------TTCC CAGTAGTAAT	2825
BovB_Tr-RTE-Tiliqua_rugosa	---AACAAAG GT----CCGT ATAGTCAAAG -CTATGGTT- ---------- ------TTCC CAGTAGTAAT	2838
BovB_ALi-RTE-Amblyomma_limbatu	---AAAAAAA AT----GCGT ATAGTTAAGG -ATATGGTT- ---------- ------TTCC AAGTTACAAT	3007
BovB_Hs-RTE-Hydrophis_spiralis	---AACAAAA GT----GTGT ATAGTCAAGG -CTATGGTT- ---------- ------TTCC CAGTTGCAAT	1212
BovB_VA-RTE-Vipera_ammodytes	---AACAAAA GT----GCGT ATAGTCAAGG -CTATGGTC- ---------- ------TTCC CAGTTGCAAT	2795
BovB_ACo-RTE-Agkistrodon_conto	---AACAAAA GT----ACGT ATAGTCAAGG -CCATGGTT- ---------- ------TTCC CAGTTGCAAT	2794
BovB_Pg-RTE-Perameles_gunnii	---AACAAAG GT----TCGT ATAGTCAAAG -CTATGGTT- ---------- ------TTTC TAGTAGCAAT	974
BovB_Io-RTE-Isoodon_obesulus	---GACAAAA GT----CTAT ATAGTTAAAA -TTATAGTT- ---------- ------TTTT CAGTAGCAAT	1435
BovB_Bp-RTE-Burramys_parvus	---GACAAAG GTA---CCAT ATAGTCAAAG TCTATGGTTG TTTGTTTGTT TGTTTGTTTC CAGTAGCAAT	1538
BovB_Pb-RTE-Petaurus_breviceps	---AACAAGG T-----CCAT ATAATAAAAA ACTATGGTT- ---------- ------TTTC CAGTAGCAAT	1564
BovB_Ma-RTE-Macropus_antilopin	---AACAAAG GT----CCAT ATAGTCAAAG -CTATGGTT- ---------- ------TTTC CAGTAGCAAT	2910
BovB_Af-RTE-Antechinus_flavipe	---AACAAAA GT----CTAT ATAGTCAAAG -CAGTATTTT ---------- ------TTTC TAATAATAAT	2105
BovB_Ac-RTE-Anolis_carolinensi	---AACAAAG GT----CCGC ATAGTCAAAG -CAATGGTA- ---------- ------TTCC CCATAATAAC	2773
BovB_BM-RTE-Bombyx_mori	---AACAAAG A----CAAAA CTGGTGCTCA -CTTTGGTC- ---------- -----TTTTC C-ATTTTTCT	2602
BovB_Dr-RTE-Christinus	---AACAAAG A----TCAGA ATAGTCAATG -CCATTGTT- ---------- -----TTCCC A-ATCTTTAT	2668
BovB_La-RTE-Loxodonta_africana	---GACTAAA GAAGGTGCAC CTGACCCAAG -CCATGGT-- ---------- -----TTTTC A-ATCACATC	2791
BovB_Pc-RTE-Procavia_capensis	---GACTAAA G----TGCGC CTGACCCAAG -CCATGGTA- ---------- -----TTTTC A-ATCGCATC	2790
BovB_Et-RTE-Christinus	---AAATAAG G----TGCAC CTGACCCACG -CCATGGCA- ---------- -----TTTTC A-ATTGCTTC	2559
BovB_Ta-RTE-Tachyglossus_acule	---CACAAAA A----TACAA ATTGTCAATT -CTATGGTG- ---------- -----TTTCC A-GTGACAAT	2930
BovB_Plat-RTE-Ornithorhynchus	---CACAAAA A----TACAA ATTGTCAACT -CTATGGTG- ---------- -----TTTCC A-GTGACAAT	2919
BovB_Ec-RTE-Equus_caballus	---GACTAAG G----CCAAG ATTATCCACA -CCCTCGTA- ---------- -----TTCCC A-ATTACTAT	2689
BovB_Cg-RTE-Christinus	TGCGACCAAG A----TCAAG ATAATTCATG -CCATAGTA- ---------- -----TTCCC CCATTACTAT	2217
BovB_Bh-RTE-Bothriocroton_hydr	---AACAAAG GT----TCGC ATAGTCAAAG -CTATGGTT- ---------- ------TTTC AAGTGGCAAT	1239
BovB_Ml-RTE-Gehyra_variegata	---GATAAAA AT----CTGT GAAATTAAAG -CTATGGTT- ---------- ------TTTC TAGTAGTAAA	1315
Sly-rnd1_family284-LINE/RTEBov	---------- ---------- ---------- ---------- ---------- ---------- ----------	56
Sly-rnd5_family2019-LINE/RTEBo	---------- ---------- ---------- ---------- ---------- ---------- ----------	477
Sly-rnd3_family322-LINE/RTEBov	---------- ---------- ---------- ---------- ---------- ---------- ----------	568
Sly-rnd5_family1698-LINE/RTEBo	---------- ---------- ---------- ---------- ---------- ---------- ----------	231
Sly-rnd1_family795-LINE/RTEBov	---------- ---------- ---------- ---------- ---------- ---------- ----------	538
Stu-rnd-5_family-3162_LINE_RTE	---------- ---------- ---------- ---------- ---------- ---------- ----------	2547
Sly-rnd6_family1960-LINE/RTEBo	---------- ---------- ---------- ---------- ---------- ---------- ----------	1316
Stu-rnd-5_family-2493_LINE_RTE	---------- ---------- ---------- ---------- ---------- ---------- ----------	215
Sly-rnd5_family447-LINE/RTEBov	---------- ---------- ---------- ---------- ---------- ---------- ----------	564
Stu-rnd-6_family-2093_LINE_RTE	---------- ---------- ---------- ---------- ---------- ---------- ----------	105
Consensus	---AACAAA- G------C-- ATAGTCAA-G -C-ATGGT-- ---------- ------TT-C --GT-G--AT	

Conservation

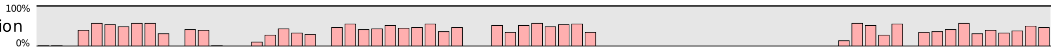

```
                                       3,860                3,880                3,900                3,920
Stu-rnd-1_family-417_LINE_RTE-      ---------- ---------- ---------- ---------- ---------- ---------- ---------- 306
Sly-rnd1_family377-LINE/RTEBov      ---------- ---------- ---------- ---------- ---------- ---------- ---------- 135
Stu-rnd-1_family-489_LINE_RTE-      ---------- ---------- ---------- ---------- ---------- ---------- ---------- 87
Stu-rnd-1_family-607_LINE_RTE-      ---------- ---------- ---------- ---------- ---------- ---------- ---------- 671
Stu-rnd-1_family-645_LINE_RTE-      ---------- ---------- ---------- ---------- ---------- ---------- ---------- 437
Sly-rnd4_family81-LINE/RTEBovB      ---------- ---------- ---------- ---------- ---------- ---------- ---------- 861
Stu-rnd-6_family-408_LINE_RTE-      ---------- ---------- ---------- ---------- ---------- ---------- ---------- 393
Stu-rnd-1_family-351_LINE_RTE-      ---------- ---------- ---------- ---------- ---------- ---------- ---------- 609
Stu-rnd-1_family-382_LINE_RTE-      ---------- ---------- ---------- ---------- ---------- ---------- ---------- 347
Sly-rnd6_family746-LINE/RTEBov      ---------- ---------- ---------- ---------- ---------- ---------- ---------- 122
Stu-rnd-5_family-325_LINE_RTE-      ---------- ---------- ---------- ---------- ---------- ---------- ---------- 446
Sly-rnd4_family509-LINE/RTEBov      ---------- ---------- ---------- ---------- ---------- ---------- ---------- 743
Stu-rnd-1_family-513_LINE_RTE-      ---------- ---------- ---------- ---------- ---------- ---------- ---------- 65
Sly-rnd5_family1442-LINE/RTEBo      ---------- ---------- ---------- ---------- ---------- ---------- ---------- 119
BovB_Gl-RTE-Gehyra_lazelli          --------GT ATGGCTGC-- ----GAGAGC TGGACTA-TA AGGAAGG--- ---CTGAGCG -CCGCAGAAT 2990
BovB_Gv-RTE-Gehyra_variegata        --------GT ATGGCTGC-- ----GAGAGC TGGACTA-TA AGGAAGG--- ---CTGAGCG -CCGCAGAAT 2917
BovB_PMo-RTE-Python_molurus         --------GT ATGGCTGT-- ----GAGAGC TGGACCA-TA AAGAAGG--- ---CTGAGCG -CCGAAGAAT 2849
BovB_Oa-RTE-Ovis_aries              --------GT ATGGATGT-- ----GAGAGT TGGACCA-TA AAGAAAG--- ---CTGAGCA -CCGAAGAAT 2885
BovB-RTE-Bos_taurus                 --------GT ATGGATGT-- ----GAGAGT TGGACTG-TG AAGAAGG--- ---CTGAGCG -CCGAAGAAT 3473
BovB_Me-RTE-Macropus_eugenii        --------AT ATGACCAT-- ----GAGAGC TAGACTA-TA AGGAAAC--- ---CTGAACA -CCATAGTAC 2443
BovB_Ano-RTE-Amphibolurus_norr      --------GT ATGGAAGT-- ----GAGAGC TGGACCA-TA AAGAAAG--- ---CAGACCG -CCGAAGAAT 2922
BovB_Gd-RTE-Eremiascincus_doug      --------GT ATGGCTGT-- ----GAGAGT TGGACCA-TA AGGAAGG--- ---CAGAGCG -CCGAAGAAT 2512
BovB_Er-RTE-Eremiascincus_rich      --------GT ATGGCTGT-- ----GAGAGC TGGACCA-TA AGGAAGG--- ---CAGAGCG -CCGAAGAAT 3046
BovB_Ca-RTE-Ctenotus_atlas          --------GT ATGGCTGT-- ----GAGAGC TGGACCA-TA AGAAAGG--- ---CAGAGCG -CCGAAGAAT 3038
BovB_Es-RTE-Egernia_stokesii        --------GT ATGGCTGT-- ----GAGAGC TGGACCA-TA AGGAGGG--- ---CTGAGTG -CCAAAGAAT 2873
BovB_Tr-RTE-Tiliqua_rugosa          --------GT ATGGCTGT-- ----GAGAGC TGGACCA-TA AGGAGGG--- ---CTGAGCG -CCGAAGAAT 2886
BovB_ALi-RTE-Amblyomma_limbatu      --------GT ATGGCTGTG- ----AAAGTT TAGACAA-AA AAAAAGC--- ---TCAAGCG -TAAAACTTT 3056
BovB_Hs-RTE-Hydrophis_spiralis      CCCCAGTTGT ATGGCTGTT- ----GAAAGT TGGACCA-TA AGGAAGG--- ---CTGAGCA -CCAAAGAAT 1269
BovB_VA-RTE-Vipera_ammodytes        --------GT ATGGCAGT-- ----GAAAGC TGGACCA-TA AAAAAGG--- ---CTGAGCG -CCAAAGAAT 2843
BovB_ACo-RTE-Agkistrodon_conto      --------GT ATGGCTGT-- ----GAAAGT TGGACCA-TA AGAAAGG--- ---CTGAGCG -CCAAAGAAT 2842
BovB_Pg-RTE-Perameles_gunnii        GT-------- ATAGCTGT-- ----GAGAGT TGGACTA-TA AGGAAAA--- ---CTGAATG -CTGCAGAAT 1022
BovB_Io-RTE-Isoodon_obesulus        GT-------- ATGACTAT-- ----GAGAAT TCAATTA-TA AGGAAAG--- ---CTGAGCA -CTGCAGAAT 1483
BovB_Bp-RTE-Burramys_parvus         GTC-----AA ATGGCTATAA GAAGGAGAGT TGGACTA-TA AGGAAAGAAA AGCCTGAGCA -TTACAGAAT 1601
BovB_Pb-RTE-Petaurus_breviceps      GT-------- ATGGTTGT-- ----GAGAGT TGGACTA-TA AGGAAAG--- ---CTGAGCA -CTCCAGAAT 1612
BovB_Ma-RTE-Macropus_antilopin      GT-------- ATGGCTGT-- ----GAGAGT TGGACTA-TA AGGAAAG--- ---CTGAGTG -CTGCAGAAT 2958
BovB_Af-RTE-Antechinus_flavipe      AT-------- ATAGCTGT-- ----GAAAAT TAGATTA-TA ATGAAAG--- ---CTAAAGA -A---AGAAT 2150
BovB_Ac-RTE-Anolis_carolinensi      C-------T  ATGGATGC-- ----GAGAGC TGGACCAATA AGGAAAG--- ---CTGAGCG -AAGGAAGAT 2822
BovB_BM-RTE-Bombyx_mori             --------AT ACG--GAG-- --CGGAGACC TGGACCCT-T AAAGCAG--- ---CTGATCG -CAAACGCGT 2650
BovB_Dr-RTE-Christinus              --------GT ATG--CAT-- --GTGAGAGC TGGACACT-C AAGAAGA--- ---AAGACAG -AAGGAAGAT 2716
BovB_La-RTE-Loxodonta_africana      --------AT ATG--CAT-- --GTGAAAGC TGGACAAT-G AATAAGG--- ---AAGACCA -AAGAAGAAT 2839
BovB_Pc-RTE-Procavia_capensis       --------AT ATG--CAT-- --GTGAAAGC TGGACAAT-G AACAAAG--- ---AAGACAG -AAGAAGAAT 2838
BovB_Et-RTE-Christinus              --------AT ATG--TG--- --GCGATGA- ---------- ---------- ---------- ---------- 2573
BovB_Ta-RTE-Tachyglossus_acule      --------GT ATG--GAT-- --CTGAAAGC TGGACAGT-G AAAAAAC--- ---AGGATAG -AAAGAACAT 2978
BovB_Plat-RTE-Ornithorhynchus       --------GT ATG--GAT-- --CCGAAAGC TGGACAGT-G AAAAAAC--- ---AGGATAG -AAAGAACAT 2967
BovB_Ec-RTE-Equus_caballus          --------GT ACA--GGT-- --GTGAAAGC TGGACAGT-G AAGAAGG--- ---CTGACAG GAAAAAAAAT 2738
BovB_Cg-RTE-Christinus              --------GT ATATGGGT-- --GTGAAAGC TGGACAGTTG AAGAAAG--- ---CTGACAG GAAAGAAAGT 2269
BovB_Bh-RTE-Bothriocroton_hydr      --------GT ATGGCTGT-- ----GAGAGC TGGACCA-TA AGAAAGG--- ---TAGAGTG -CCGAAGAAT 1287
BovB_Ml-RTE-Gehyra_variegata        T-------AT ATATTTGT-- ----GAGATT TGGACTA-AA AAGAAAC--- ---TTGAATA ------TAAC 1359
Sly-rnd1_family284-LINE/RTEBov      ---------- ---------- ---------- ---------- ---------- ---------- ---------- 56
Sly-rnd5_family2019-LINE/RTEBo      ---------- ---------- ---------- ---------- ---------- ---------- ---------- 477
Sly-rnd3_family322-LINE/RTEBov      ---------- ---------- ---------- ---------- ---------- ---------- ---------- 568
Sly-rnd5_family1698-LINE/RTEBo      ---------- ---------- ---------- ---------- ---------- ---------- ---------- 231
Sly-rnd1_family795-LINE/RTEBov      ---------- ---------- ---------- ---------- ---------- ---------- ---------- 538
Stu-rnd-5_family-3162_LINE_RTE      ---------- ---------- ---------- ---------- ---------- ---------- ---------- 2547
Sly-rnd6_family1960-LINE/RTEBo      ---------- ---------- ---------- ---------- ---------- ---------- ---------- 1316
Stu-rnd-5_family-2493_LINE_RTE      ---------- ---------- ---------- ---------- ---------- ---------- ---------- 215
Sly-rnd5_family447-LINE/RTEBov      ---------- ---------- ---------- ---------- ---------- ---------- ---------- 564
Stu-rnd-6_family-2093_LINE_RTE      ---------- ---------- ---------- ---------- ---------- ---------- ---------- 105

Consensus                           ---------- ATG---GT-- ----GA-AG- TGGAC-A--- A--AA-G--- ---C-GA--- -----AGAAT
```

Conservation

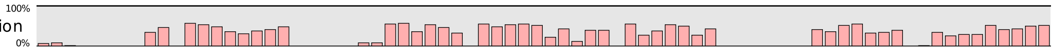

```
                                           3,940                         3,960                         3,980
Stu-rnd-1_family-417_LINE_RTE-    ---------- ---------- ---------- ---------- ---------- ---------- ---------- 306
Sly-rnd1_family377-LINE/RTEBov    ---------- ---------- ---------- ---------- ---------- ---------- ---------- 135
Stu-rnd-1_family-489_LINE_RTE-    ---------- ---------- ---------- ---------- ---------- ---------- ---------- 87
Stu-rnd-1_family-607_LINE_RTE-    ---------- ---------- ---------- ---------- ---------- ---------- ---------- 671
Stu-rnd-1_family-645_LINE_RTE-    ---------- ---------- ---------- ---------- ---------- ---------- ---------- 437
Sly-rnd4_family81-LINE/RTEBovB    ---------- ---------- ---------- ---------- ---------- ---------- ---------- 861
Stu-rnd-6_family-408_LINE_RTE-    ---------- ---------- ---------- ---------- ---------- ---------- ---------- 393
Stu-rnd-1_family-351_LINE_RTE-    ---------- ---------- ---------- ---------- ---------- ---------- ---------- 609
Stu-rnd-1_family-382_LINE_RTE-    ---------- ---------- ---------- ---------- ---------- ---------- ---------- 347
Sly-rnd6_family746-LINE/RTEBov    ---------- ---------- ---------- ---------- ---------- ---------- ---------- 122
Stu-rnd-5_family-325_LINE_RTE-    ---------- ---------- ---------- ---------- ---------- ---------- ---------- 446
Sly-rnd4_family509-LINE/RTEBov    ---------- ---------- ---------- ---------- ---------- ---------- ---------- 743
Stu-rnd-1_family-513_LINE_RTE-    ---------- ---------- ---------- ---------- ---------- ---------- ---------- 65
Sly-rnd5_family1442-LINE/RTEBo    ---------- ---------- ---------- ---------- ---------- ---------- ---------- 119
BovB_Gl-RTE-Gehyra_lazelli        AGAT-----T CTTTTGAATT ATGGTGC-TG GA-------G AAGACTCTT- GAGAG-TCCC TTGGAC---- 3041
BovB_Gv-RTE-Gehyra_variegata      AGAT-----T CTTTTGAATT ATGGTGC-TG GA-------G AAGACTCTT- GAGAG-TCCC TTGGAC---- 2968
BovB_PMo-RTE-Python_molurus       TGAT-----G CCTTTGAACT GTGGTGC-TG GA-------G AAGACTCTT- GAGAG-TCCC CTGGAC---- 2900
BovB_Oa-RTE-Ovis_aries            TGAT-----G CTTTTGAACT GTGGTGC-TG GA-------G AAGACTCTT- GAGAG-TCCC TTGGAC---- 2936
BovB-RTE-Bos_taurus               TGAT-----G CTTTTGAACT GTGGTGT-TG GA-------G AAGACTCTT- GAGAG-TCCC TTGGAC---- 3524
BovB_Me-RTE-Macropus_eugenii      TGAT-----G CTTTTGAATA TTGGTCT-TT GA-------G AAGACTTCT- GAGAG-TCCC TTGGAC---- 2494
BovB_Ano-RTE-Amphibolurus_norr    TGAT-----G CCTTTGAATT GTGGTGC-TG GA-------G GAGGCTCTT- GAGAG-TCCC CTGGAC---- 2973
BovB_Gd-RTE-Eremiascincus_doug    AGAT-----G CTTTCGAATT ATGGTGC-TG GA-------G AAGACTCTT- GAGAG-TCCC CTGGAC---- 2563
BovB_Er-RTE-Eremiascincus_rich    AGAT-----G CTTTCGAATT ATGGTGC-TG GA-------G AAGACTCTT- GAGAG-TCCC CTGGAC---- 3097
BovB_Ca-RTE-Ctenotus_atlas        AGAT-----G CTTTTGAATT ATGGTGT-TG GA-------G AAGACTCTT- GAGAG-TCCC CTGGAC---- 3089
BovB_Es-RTE-Egernia_stokesii      AGAT-----G CTTTTGAATT ATGGTGT-TG GA-------G AAGACTTTT- GAGAG-TCCC CTGGAC---- 2924
BovB_Tr-RTE-Tiliqua_rugosa        AGAT-----G CTTTCGAATT ATGGTGT-TG GA-------G AAGACTTTT- GAGAG-TCCC CTGGAC---- 2937
BovB_ALi-RTE-Amblyomma_limbatu    TGGATTCAGG CTTTTGAATT GTTGTGC-TC GG------AG GAGACTCCT- GGCGAGTCCC ATGGAC---- 3114
BovB_Hs-RTE-Hydrophis_spiralis    TGGA----GG CCTTTGAACT ATGGTGC-TG GAGGTGGAAG AACACTCCT- GCGAGTCCCC TTGGAC---- 1329
BovB_VA-RTE-Vipera_ammodytes      TGA-----GG CCTTTGAACT ATGGTGC-TG GA-------G AAGACTCCT- GCGAG-TCCC TTGGAC---- 2894
BovB_ACo-RTE-Agkistrodon_conto    TGA-----GG CCTTTGAACT ATGGTGC-TG GA-------G AAGACTCCT- GCGAG-TCCC CTGGAC---- 2893
BovB_Pg-RTE-Perameles_gunnii      CAAT----GC TTT-CAAATT GTGGTGCATG GA-------G AAGAATTTT- GAGAGTC-CC TTGGAC---- 1074
BovB_Io-RTE-Isoodon_obesulus      AGAT----GC AAT-CAAATT ATGGTGT-TG GA-------G AAGACTTTT- GAGAGTC-CC TTGGGCCACA 1538
BovB_Bp-RTE-Burramys_parvus       CAAT----AC TTTTCAAATT GTGGTGC-TG GA-------G AAGACTTTTT GAGAGTC-CT TTGGAC---- 1654
BovB_Pb-RTE-Petaurus_breviceps    TGAT----GC TTT-CAAATT GTGGTGC-TA AA-------G AAGACTTTTT GAGAGTC-CC TTGGAC---- 1664
BovB_Ma-RTE-Macropus_antilopin    CAAT----GC TTT-TGAATT GTGGTGC-TG GA-------G AAGACTTTT- GAGAGTC-CC TTGGAC---- 3009
BovB_Af-RTE-Antechinus_flavipe    CAAA----AA CTTTCAAATT GTGATGC-TA GA-------A AAGACTTTT- GAGAGTTACC TTGGAC---- 2203
BovB_Ac-RTE-Anolis_carolinensi    AGAC-----G CTTTTGAACT CTGGTGC-TG GA-------G GAAAATCCT- GAGAG-TGCC TTGGAC---- 2873
BovB_BM-RTE-Bombyx_mori           GGAT-----G CTTTTGAGAT GTGGTGC-TG GA-------G AAGAATGCT- CCGGA-TACC TTGGAC---- 2701
BovB_Dr-RTE-Christinus            CGAT-----G CTTTCGAGTT GTGGTGC-TG GA-------G GCGAATGAT- GCAC--TACC ATGGAC---- 2766
BovB_La-RTE-Loxodonta_africana    TGAC-----G CCTTTGAATT GTGGTGT-TG GC-------G AAGAATATT- GAATA-T-CC ATGGAC---- 2889
BovB_Pc-RTE-Procavia_capensis     TGAT-----G CCTTTGAATT GTGGTGT-TG GA-------G AAGAATGTT- GAATG-TACC ATGGAC---- 2889
BovB_Et-RTE-Christinus            ---------- ---------- ---------- ---------- ---------- ---------- ---------- 2573
BovB_Ta-RTE-Tachyglossus_acule    CGAT-----T CTTTTGAAAT GTGGTGT-TG GA-------G AAGGCTTTT- GTGAA-TACC ATGGAC---- 3029
BovB_Plat-RTE-Ornithorhynchus     CGAT-----T CTTTTGAAAT GTGGTGT-TG GA-------G AAGGCTTTT- GCGAA-TACC ATGGAC---- 3018
BovB_Ec-RTE-Equus_caballus        TGAT-----T CATTTGAAAT ATGGTGT-TG GA-------G GAGAGCTCT- ACGGA-TACC CTGGAC---- 2789
BovB_Cg-RTE-Christinus            AGAT-----T CTTTTGAAAT GTGGTGT-TG GA-------G GAGAGTTTTT ACGGA-TACC GTGGAC---- 2321
BovB_Bh-RTE-Bothriocroton_hydr    AGATTT--GT TTTTCGAATT ATGGTGT-TG GA-------G AAGACTTTT- GAGAG-TCCC ATGGAC---- 1341
BovB_Ml-RTE-Gehyra_variegata      TGAA------ --TTGAATCT ATTGGAC-TA GA-------G TAGGCTTTT- GAGAA-TCTA TTGGAT---- 1407
Sly-rnd1_family284-LINE/RTEBov    ---------- ---------- ---------- ---------- ---------- ---------- ---------- 56
Sly-rnd5_family2019-LINE/RTEBo    ---------- ---------- ---------- ---------- ---------- ---------- ---------- 477
Sly-rnd3_family322-LINE/RTEBov    ---------- ---------- ---------- ---------- ---------- ---------- ---------- 568
Sly-rnd5_family1698-LINE/RTEBo    ---------- ---------- ---------- ---------- ---------- ---------- ---------- 231
Sly-rnd1_family795-LINE/RTEBov    ---------- ---------- ---------- ---------- ---------- ---------- ---------- 538
Stu-rnd-5_family-3162_LINE_RTE    ---------- ---------- ---------- ---------- ---------- ---------- ---------- 2547
Sly-rnd6_family1960-LINE/RTEBo    ---------- ---------- ---------- ---------- ---------- ---------- ---------- 1316
Stu-rnd-5_family-2493_LINE_RTE    ---------- ---------- ---------- ---------- ---------- ---------- ---------- 215
Sly-rnd5_family447-LINE/RTEBov    ---------- ---------- ---------- ---------- ---------- ---------- ---------- 564
Stu-rnd-6_family-2093_LINE_RTE    ---------- ---------- ---------- ---------- ---------- ---------- ---------- 105
Consensus                         -GA------- C-TT-GAA-T -TGGTG--TG GA-------G AAGACT--T- G-GA--T-CC -TGGAC----
```

Conservation

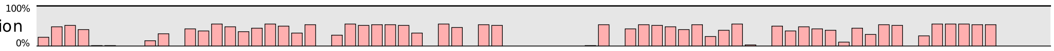

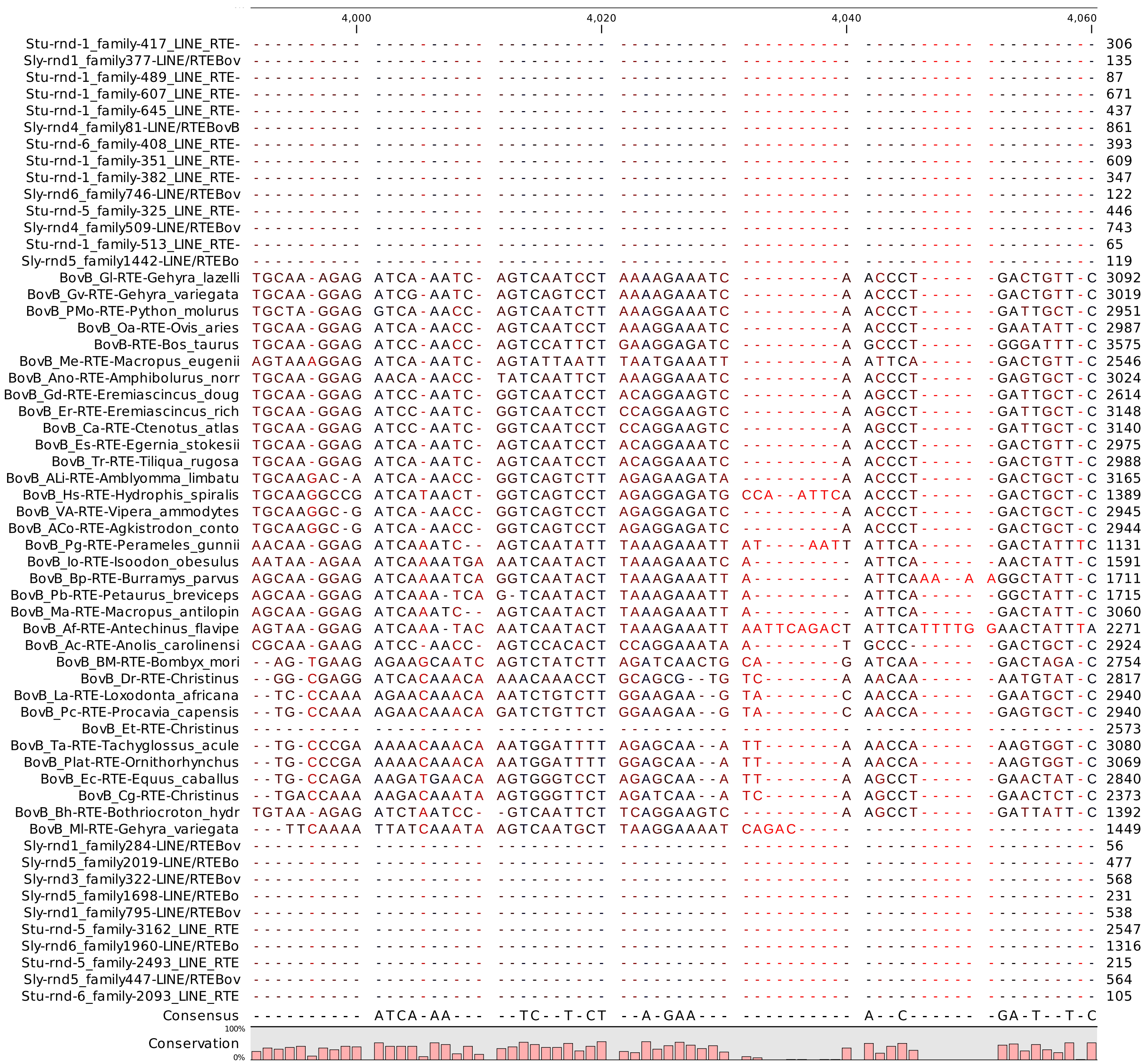
4,000 4,020 4,040 4,060
Stu-rnd-1_family-417_LINE_RTE- ---------- ---------- ---------- ---------- ---------- ---------- ---------- 306
Sly-rnd1_family377-LINE/RTEBov ---------- ---------- ---------- ---------- ---------- ---------- ---------- 135
Stu-rnd-1_family-489_LINE_RTE- ---------- ---------- ---------- ---------- ---------- ---------- ---------- 87
Stu-rnd-1_family-607_LINE_RTE- ---------- ---------- ---------- ---------- ---------- ---------- ---------- 671
Stu-rnd-1_family-645_LINE_RTE- ---------- ---------- ---------- ---------- ---------- ---------- ---------- 437
Sly-rnd4_family81-LINE/RTEBovB ---------- ---------- ---------- ---------- ---------- ---------- ---------- 861
Stu-rnd-6_family-408_LINE_RTE- ---------- ---------- ---------- ---------- ---------- ---------- ---------- 393
Stu-rnd-1_family-351_LINE_RTE- ---------- ---------- ---------- ---------- ---------- ---------- ---------- 609
Stu-rnd-1_family-382_LINE_RTE- ---------- ---------- ---------- ---------- ---------- ---------- ---------- 347
Sly-rnd6_family746-LINE/RTEBov ---------- ---------- ---------- ---------- ---------- ---------- ---------- 122
Stu-rnd-5_family-325_LINE_RTE- ---------- ---------- ---------- ---------- ---------- ---------- ---------- 446
Sly-rnd4_family509-LINE/RTEBov ---------- ---------- ---------- ---------- ---------- ---------- ---------- 743
Stu-rnd-1_family-513_LINE_RTE- ---------- ---------- ---------- ---------- ---------- ---------- ---------- 65
Sly-rnd5_family1442-LINE/RTEBo ---------- ---------- ---------- ---------- ---------- ---------- ---------- 119
BovB_Gl-RTE-Gehyra_lazelli TGCAA-AGAG ATCA-AATC- AGTCAATCCT AAAAGAAATC ---------A ACCCT----- -GACTGTT-C 3092
BovB_Gv-RTE-Gehyra_variegata TGCAA-GGAG ATCG-AATC- AGTCAGTCCT AAAAGAAATC ---------A ACCCT----- -GACTGTT-C 3019
BovB_PMo-RTE-Python_molurus TGCTA-GGAG GTCA-AACC- AGTCAATCTT AAAGGAAATC ---------A ACCCT----- -GATTGCT-C 2951
BovB_Oa-RTE-Ovis_aries TGCAA-GGAG ATCA-AACC- AGTCAATCCT AAAGGAAATC ---------A ACCCT----- -GAATATT-C 2987
BovB-RTE-Bos_taurus TGCAA-GGAG ATCC-AACC- AGTCCATTCT GAAGGAGATC ---------A GCCCT----- -GGGATTT-C 3575
BovB_Me-RTE-Macropus_eugenii AGTAAAGGAG ATCA-AATC- AGTATTAATT TAATGAAATT ---------A ATTCA----- -GACTGTT-C 2546
BovB_Ano-RTE-Amphibolurus_norr TGCAA-GGAG AACA-AACC- TATCAATTCT AAAGGAAATC ---------A ACCCT----- -GAGTGCT-C 3024
BovB_Gd-RTE-Eremiascincus_doug TGCAA-GGAG ATCC-AATC- GGTCAATCCT ACAGGAAGTC ---------A AGCCT----- -GATTGCT-C 2614
BovB_Er-RTE-Eremiascincus_rich TGCAA-GGAG ATCC-AATC- GGTCAATCCT CCAGGAAGTC ---------A AGCCT----- -GATTGCT-C 3148
BovB_Ca-RTE-Ctenotus_atlas TGCAA-GGAG ATCC-AATC- GGTCAATCCT CCAGGAAGTC ---------A AGCCT----- -GATTGCT-C 3140
BovB_Es-RTE-Egernia_stokesii TGCAA-GGAG ATCA-AATC- AGTCAATCCT ACAGGAAATC ---------A ACCCT----- -GACTGTT-C 2975
BovB_Tr-RTE-Tiliqua_rugosa TGCAA-GGAG ATCA-AATC- AGTCAATCCT ACAGGAAATC ---------A ACCCT----- -GACTGTT-C 2988
BovB_ALi-RTE-Amblyomma_limbatu TGCAAGAC-A ATCA-AACC- GGTCAGTCTT AGAGAAGATA ---------A ACCCT----- -GACTGCT-C 3165
BovB_Hs-RTE-Hydrophis_spiralis TGCAAGGCCG ATCATAACT- GGTCAGTCCT AGAGGAGATG CCA--ATTCA ACCCT----- -GACTGCT-C 1389
BovB_VA-RTE-Vipera_ammodytes TGCAAGGC-G ATCA-AACC- GGTCAGTCCT AGAGGAGATC ---------A ACCCT----- -GACTGCT-C 2945
BovB_ACo-RTE-Agkistrodon_conto TGCAAGGC-G ATCA-AACC- GGTCAGTCCT AGAGGAGATC ---------A ACCCT----- -GACTGCT-C 2944
BovB_Pg-RTE-Perameles_gunnii AACAA-GGAG ATCAAATC-- AGTCAATATT TAAAGAAATT AT----AATT ATTCA----- -GACTATTTC 1131
BovB_Io-RTE-Isoodon_obesulus AATAA-AGAA ATCAAAATGA AATCAATACT TAAAGAAATC A--------- ATTCA----- -AACTATT-C 1591
BovB_Bp-RTE-Burramys_parvus AGCAA-GGAG ATCAAAATCA GGTCAATACT TAAAGAAATT A--------- ATTCAAA--A AGGCTATT-C 1711
BovB_Pb-RTE-Petaurus_breviceps AGCAA-GGAG ATCAAA-TCA G-TCAATACT TAAAGAAATT A--------- ATTCA----- -GGCTATT-C 1715
BovB_Ma-RTE-Macropus_antilopin AGCAA-GGAG ATCAAATC-- AGTCAATACT TAAAGAAATT A--------- ATTCA----- -GACTATT-C 3060
BovB_Af-RTE-Antechinus_flavipe AGTAA-GGAG ATCAAA-TAC AATCAATACT TAAAGAAATT AATTCAGACT ATTCATTTTG GAACTATTTA 2271
BovB_Ac-RTE-Anolis_carolinensi CGCAA-GAAG ATCC-AACC- AGTCCACACT CCAGGAAATA A--------T GCCC------ -GACTGCT-C 2924
BovB_BM-RTE-Bombyx_mori --AG-TGAAG AGAAGCAATC AGTCTATCTT AGATCAACTG CA-------G ATCAA----- -GACTAGA-C 2754
BovB_Dr-RTE-Christinus --GG-CGAGG ATCACAAACA AAACAAACCT GCAGCG--TG TC-------A AACAA----- -AATGTAT-C 2817
BovB_La-RTE-Loxodonta_africana --TC-CCAAA AGAACAAACA AATCTGTCTT GGAAGAA--G TA-------C AACCA----- -GAATGCT-C 2940
BovB_Pc-RTE-Procavia_capensis --TG-CCAAA AGAACAAACA GATCTGTTCT GGAAGAA--G TA-------C AACCA----- -GAGTGCT-C 2940
BovB_Et-RTE-Christinus ---------- ---------- ---------- ---------- ---------- ---------- ---------- 2573
BovB_Ta-RTE-Tachyglossus_acule --TG-CCCGA AAAACAAACA AATGGATTTT AGAGCAA--A TT-------A AACCA----- -AAGTGGT-C 3080
BovB_Plat-RTE-Ornithorhynchus --TG-CCCGA AAAACAAACA AATGGATTTT GGAGCAA--A TT-------A AACCA----- -AAGTGGT-C 3069
BovB_Ec-RTE-Equus_caballus --TG-CCAGA AAGATGAACA AGTGGGTCCT AGAGCAA--A TT-------A AGCCT----- -GAACTAT-C 2840
BovB_Cg-RTE-Christinus --TGACCAAA AAGACAAATA AGTGGGTTCT AGATCAA--A TC-------A AGCCT----- -GAACTCT-C 2373
BovB_Bh-RTE-Bothriocroton_hydr TGTAA-AGAG ATCTAATCC- -GTCAATTCT TCAGGAAGTC ---------A AGCCT----- -GATTATT-C 1392
BovB_Ml-RTE-Gehyra_variegata ---TTCAAAA TTATCAAATA AGTCAATGCT TAAGGAAAAT CAGAC----- ---------- ---------- 1449
Sly-rnd1_family284-LINE/RTEBov ---------- ---------- ---------- ---------- ---------- ---------- ---------- 56
Sly-rnd5_family2019-LINE/RTEBo ---------- ---------- ---------- ---------- ---------- ---------- ---------- 477
Sly-rnd3_family322-LINE/RTEBov ---------- ---------- ---------- ---------- ---------- ---------- ---------- 568
Sly-rnd5_family1698-LINE/RTEBo ---------- ---------- ---------- ---------- ---------- ---------- ---------- 231
Sly-rnd1_family795-LINE/RTEBov ---------- ---------- ---------- ---------- ---------- ---------- ---------- 538
Stu-rnd-5_family-3162_LINE_RTE ---------- ---------- ---------- ---------- ---------- ---------- ---------- 2547
Sly-rnd6_family1960-LINE/RTEBo ---------- ---------- ---------- ---------- ---------- ---------- ---------- 1316
Stu-rnd-5_family-2493_LINE_RTE ---------- ---------- ---------- ---------- ---------- ---------- ---------- 215
Sly-rnd5_family447-LINE/RTEBov ---------- ---------- ---------- ---------- ---------- ---------- ---------- 564
Stu-rnd-6_family-2093_LINE_RTE ---------- ---------- ---------- ---------- ---------- ---------- ---------- 105
Consensus ---------- ATCA-AA--- --TC--T-CT --A-GAA--- ---------- A--C------ -GA-T--T-C
100%
Conservation
0%

		4,080		4,100		4,120		
Stu-rnd-1_family-417_LINE_RTE-	----------	----------	----------	----------	----------	----------	----------	306
Sly-rnd1_family377-LINE/RTEBov	----------	----------	----------	----------	----------	----------	----------	135
Stu-rnd-1_family-489_LINE_RTE-	----------	----------	----------	----------	----------	----------	----------	87
Stu-rnd-1_family-607_LINE_RTE-	----------	----------	----------	----------	----------	----------	----------	671
Stu-rnd-1_family-645_LINE_RTE-	----------	----------	----------	----------	----------	----------	----------	437
Sly-rnd4_family81-LINE/RTEBovB	----------	----------	----------	----------	----------	----------	----------	861
Stu-rnd-6_family-408_LINE_RTE-	----------	----------	----------	----------	----------	----------	----------	393
Stu-rnd-1_family-351_LINE_RTE-	----------	----------	----------	----------	----------	----------	----------	609
Stu-rnd-1_family-382_LINE_RTE-	----------	----------	----------	----------	----------	----------	----------	347
Sly-rnd6_family746-LINE/RTEBov	----------	----------	----------	----------	----------	----------	----------	122
Stu-rnd-5_family-325_LINE_RTE-	----------	----------	----------	----------	----------	----------	----------	446
Sly-rnd4_family509-LINE/RTEBov	----------	----------	----------	----------	----------	----------	----------	743
Stu-rnd-1_family-513_LINE_RTE-	----------	----------	----------	----------	----------	----------	----------	65
Sly-rnd5_family1442-LINE/RTEBo	----------	----------	----------	----------	----------	----------	----------	119
BovB_Gl-RTE-Gehyra_lazelli	TCTGG--AAG	GCCAGATACT	G-----AAGC	-TGAAGCTC-	AAATA-CTTT	GGCCACCAAA	TGAGAAGGGA	3152
BovB_Gv-RTE-Gehyra_variegata	TCTGG--AAG	GCCAGATACT	G-----AAGC	-TGAAGCTC-	AAATA-CTTT	GGCCACCAAA	TGAGAAGGGA	3079
BovB_PMo-RTE-Python_molurus	ATTGG--AAG	GAT-GATAAT	G-----AAGC	-TCAAGCTC-	AGATA-CTTT	GGTCACCTAA	TGCGAAGAGA	3010
BovB_Oa-RTE-Ovis_aries	ATTGG--AAG	GACTGATGCT	G-----AAGC	-TGAAGCTC-	CAATA-CTTT	GGCCACCTGA	TGTGAAGAGC	3047
BovB-RTE-Bos_taurus	TTTGG--AAG	GAATGATGCT	A-----AAGC	-TGAAACTC-	CAGTA-CTTT	GGCCACCTCA	TGCGAAGAGT	3635
BovB_Me-RTE-Macropus_eugenii	TCTGG--AAG	GTCAAATACT	----------	----------	----------	----------	----------	2564
BovB_Ano-RTE-Amphibolurus_norr	ACTGG--AAG	GACAGATCCT	G-----AAGC	-TGAGGCTC-	CAGTA-CTTT	GGCCATCTCA	TGAGAAGAAA	3084
BovB_Gd-RTE-Eremiascincus_doug	CCTGG--AAG	GACAAATCCT	G-----AAGC	-TGAAGCTC-	CAATA-CTTT	GGCCACCTCA	TGAGAAGAGA	2674
BovB_Er-RTE-Eremiascincus_rich	CCTGG--AAG	GACAAATCCT	G-----AAGC	-TGAAGCTC-	CAATA-CTTT	GGCCACCTCA	TGAGAAGAGA	3208
BovB_Ca-RTE-Ctenotus_atlas	CCTGG--AAG	GACAAATCCT	G-----AAGC	-TGAAGCTC-	CAATA-CTTT	GGCCACCTCA	TGAGAAGAGA	3200
BovB_Es-RTE-Egernia_stokesii	ATTGG--AAG	GACAGATGCT	G-----AAGC	-TGAAGCTT-	AAATA-CTTT	GGTCATCTCA	TGAGAAGGGA	3035
BovB_Tr-RTE-Tiliqua_rugosa	ATTGG--AAG	GACAGATGCT	G-----AAGC	-TGAAGCTT-	AAATA-CTTT	GGTCATCTCA	TGAGAAGGGA	3048
BovB_ALi-RTE-Amblyomma_limbatu	TTTTAG-AAG	GCCAGATAGT	C-----AAAG	ATGAAAACTT	AAATA-CTTT	GACCACCTCA	TGAGAAAGAA	3228
BovB_Hs-RTE-Hydrophis_spiralis	TTTAGA-AAG	GCCAGATCCT	G-----AAGA	GTGAAACTCA	AAATA-CTTT	GGCCACCTAA	TGAGAAGGAA	1452
BovB_VA-RTE-Vipera_ammodytes	TTTAG--AAG	GCCAGATCCT	G-----AAGC	-TAAAACTC-	AAATA-CTTT	GGCCACCTAA	TGAGAAGGAA	3005
BovB_ACo-RTE-Agkistrodon_conto	TTTAG--AAG	GCCAGATCCT	G-----AAGT	-TGAAACTC-	AAGTA-CTTT	GGCCACCTAA	TGAGAAGGAA	3004
BovB_Pg-RTE-Perameles_gunnii	ATTGGA--AG	GTCAAATACT	G-----AAGC	-TGAAGCTT-	AAATA-CTTT	GGTCATATAA	TGAGAAGACA	1191
BovB_Io-RTE-Isoodon_obesulus	ACTGAA--AG	GTTAAATATT	G-----AAAT	-TGAAGCTT-	AAATA-TTTT	GAACACATAA	TAAGAAAATA	1651
BovB_Bp-RTE-Burramys_parvus	ATTGGA--AG	GTCAAATACC	TTG---AAGC	-TGAAGCTT-	AAATATCTTT	GGCCACATAA	TGAGAAGACA	1774
BovB_Pb-RTE-Petaurus_breviceps	ACTGGA--AG	GTCAAATACT	GAAGCTAAGC	-TGAAGCTT-	AAATA-CTTT	GGCCACATAA	TGAGAAGACA	1780
BovB_Ma-RTE-Macropus_antilopin	ACTGGA--AG	GTCAAATACT	G-----AAGC	-TGAAGCTT-	AAATA-CTTT	GGCCACATAA	TGAGAAGACA	3120
BovB_Af-RTE-Antechinus_flavipe	AATGGAGAAG	ATCAAATACT	G-----AAGC	-TGAAACTT-	AAATA-CTTT	GGCCACATAA	TGAGAAGACA	2333
BovB_Ac-RTE-Anolis_carolinensi	ACTGG--AGG	AAAGGATATT	A-----GAGG	-CAAAGCTG-	AAGTA-TTTT	GGCCACATCA	TGAGAGACAG	2984
BovB_BM-RTE-Bombyx_mori	TGTCCA-CAC	T-CTGCCTTC	G-----TCGC	-GC--ACTCG	AGTACTTT--	GGTCATATTG	CTCGGAAAAC	2812
BovB_Dr-RTE-Christinus	GCTAGA-GGG	C-AAAATCAC	A-----AAGC	-AACGGCTCT	CGTTCTTT--	GGTCATATCA	TGCGAGCCAA	2877
BovB_La-RTE-Loxodonta_africana	CTTAGA-AGC	A-AGGATGGC	G-----AGAC	-TGCAGCGTA	CATACTTT--	GGACATGTTA	TCAGGAGGGA	3000
BovB_Pc-RTE-Procavia_capensis	CTTGGA-AGC	A-AAGATGGC	A-----AGAC	-TGCATCTTA	CATACTTT--	GGACATGTTG	TCAGGAAGGA	3000
BovB_Et-RTE-Christinus	----------	----------	----------	----------	----------	----------	----------	2573
BovB_Ta-RTE-Tachyglossus_acule	TTTGGA-AGG	C-CAAATGAC	T-----CGAC	-TTAGATTAG	CATATTTT--	GGACACATAA	TCAGGAGGAC	3140
BovB_Plat-RTE-Ornithorhynchus	TTTGGA-AGG	C-CAAATGAC	T-----CGAC	-TTAGATTAG	CATATTTT--	GGACACATAA	TCAGGAGGAC	3129
BovB_Ec-RTE-Equus_caballus	GCTGGA-GGC	A-AAAATGAT	A-----AAAC	-TGAGGCTGT	CCTACTTT--	GGGCACATCA	TGAGAAGGCA	2900
BovB_Cg-RTE-Christinus	CCTAGA-AGC	TTAAAATGAC	T-----AAAC	-TGAGGCTAT	CGTACTTTCC	GGTCACATTA	TGAGAAGACA	2436
BovB_Bh-RTE-Bothriocroton_hydr	CCTGAA--AA	GACAAATCCT	G-----AAGC	-AGAAGCTC-	AAATA-CTTT	GGCCACATCA	TGAGAACAGA	1452
BovB_Ml-RTE-Gehyra_variegata	----------	----------	----------	----------	----------	----------	----------	1449
Sly-rnd1_family284-LINE/RTEBov	----------	----------	----------	----------	----------	----------	----------	56
Sly-rnd5_family2019-LINE/RTEBo	----------	----------	----------	----------	----------	----------	----------	477
Sly-rnd3_family322-LINE/RTEBov	----------	----------	----------	----------	----------	----------	----------	568
Sly-rnd5_family1698-LINE/RTEBo	----------	----------	----------	----------	----------	----------	----------	231
Sly-rnd1_family795-LINE/RTEBov	----------	----------	----------	----------	----------	----------	----------	538
Stu-rnd-5_family-3162_LINE_RTE	----------	----------	----------	----------	----------	----------	----------	2547
Sly-rnd6_family1960-LINE/RTEBo	----------	----------	----------	----------	----------	----------	----------	1316
Stu-rnd-5_family-2493_LINE_RTE	----------	----------	----------	----------	----------	----------	----------	215
Sly-rnd5_family447-LINE/RTEBov	----------	----------	----------	----------	----------	----------	----------	564
Stu-rnd-6_family-2093_LINE_RTE	----------	----------	----------	----------	----------	----------	----------	105
Consensus	--T-G----G	-----AT---	------A---	-T----CT--	-------T--	GG-CA--T-A	T--G-A----	

Conservation

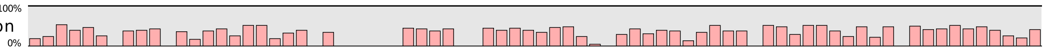

```
                                  4,140                 4,160                 4,180                 4,200
Stu-rnd-1_family-417_LINE_RTE-    ---------- ---------- ---------- ---------- ---------- ---------- ---------- 306
Sly-rnd1_family377-LINE/RTEBov    ---------- ---------- ---------- ---------- ---------- ---------- ---------- 135
Stu-rnd-1_family-489_LINE_RTE-    ---------- ---------- ---------- ---------- ---------- ---------- ---------- 87
Stu-rnd-1_family-607_LINE_RTE-    ---------- ---------- ---------- ---------- ---------- ---------- ---------- 671
Stu-rnd-1_family-645_LINE_RTE-    ---------- ---------- ---------- ---------- ---------- ---------- ---------- 437
Sly-rnd4_family81-LINE/RTEBovB    ---------- ---------- ---------- ---------- ---------- ---------- ---------- 861
Stu-rnd-6_family-408_LINE_RTE-    ---------- ---------- ---------- ---------- ---------- ---------- ---------- 393
Stu-rnd-1_family-351_LINE_RTE-    ---------- ---------- ---------- ---------- ---------- ---------- ---------- 609
Stu-rnd-1_family-382_LINE_RTE-    ---------- ---------- ---------- ---------- ---------- ---------- ---------- 347
Sly-rnd6_family746-LINE/RTEBov    ---------- ---------- ---------- ---------- ---------- ---------- ---------- 122
Stu-rnd-5_family-325_LINE_RTE-    ---------- ---------- ---------- ---------- ---------- ---------- ---------- 446
Sly-rnd4_family509-LINE/RTEBov    ---------- ---------- ---------- ---------- ---------- ---------- ---------- 743
Stu-rnd-1_family-513_LINE_RTE-    ---------- ---------- ---------- ---------- ---------- ---------- ---------- 65
Sly-rnd5_family1442-LINE/RTEBo    ---------- ---------- ---------- ---------- ---------- ---------- ---------- 119
BovB_Gl-RTE-Gehyra_lazelli        AGA------- --CTCACTGG --AGAAGACC CTGATGCTGG GAA-----AG ATT------G AAGGGAAAAG 3200
BovB_Gv-RTE-Gehyra_variegata      AGA------- --CTCACTGG --AGAAGACC CTGATGCTGG GAA-----AG ATT------G AAGGGAAAAG 3127
BovB_PMo-RTE-Python_molurus       GGA------- --CTCTTTGG --AAAAGACC CTGATGCTGG GAA-----AG ATT------G AAGGCAAAAG 3058
BovB_Oa-RTE-Ovis_aries            TGA------- --CTCATTGG --AAAAGACC CTGATGCTGG GAA-----AG ATT------G AAGGCAGGAG 3095
BovB-RTE-Bos_taurus               TGA------- --CTCATTGG --AAAAGACT CTGATGCTGG GAG-----GG ATT------G GGGGCAGGAG 3683
BovB_Me-RTE-Macropus_eugenii      ---------- ---------- ---------- ---------- ---------- ---------- ---------- 2564
BovB_Ano-RTE-Amphibolurus_norr    AGA------- --CTCCTTGG --AAAAAACC TTGATGTTAG GAA-----AG TGT------G ACGGCAAGAG 3132
BovB_Gd-RTE-Eremiascincus_doug    GGA------- --CTCTTTAG --AAAAGACC CTCATGCTGG GAA-----AG ATG------G AAGGCAAAAG 2722
BovB_Er-RTE-Eremiascincus_rich    GGA------- --CTCTTTAG --AAAAGACC CTCATGCTGG GAA-----AG ATT------G AAGGCAAAAG 3256
BovB_Ca-RTE-Ctenotus_atlas        GGA------- --CTCTTTAG --AAAAGACC CTCATGCTGG GAA-----AG ATT------G AAGGCAAAAG 3248
BovB_Es-RTE-Egernia_stokesii      GGA------- --CTCTTTAG --AAAAGACC CTGATGCTGG GAA-----AA ATT------G AAGGCAAAAG 3083
BovB_Tr-RTE-Tiliqua_rugosa        GGA------- --CTCTTTAG --AAAAGACC CTGATGCTGG GAA-----AA ATT------G AAGGCAAAAG 3096
BovB_ALi-RTE-Amblyomma_limbatu    GGA------- -CTTC-CTGA --AGAAGAAT ATAATCTTCA TATGCTTTAG AACACTA--G ATGGCAAAAA 3285
BovB_Hs-RTE-Hydrophis_spiralis    GGAAGGAAGG ACTCCACTGG --AGAAGAGC CTAATGCTGG GAA-----AG AATTTTGAGG GCAAAAGAAG 1515
BovB_VA-RTE-Vipera_ammodytes      GGA------- --CTCACTGG --AGAAGAGC CTAATGCTGG GAA-----AG ATT------G AGGGCAATAA 3053
BovB_ACo-RTE-Agkistrodon_conto    GGA------- --CTCACTGG --AGAAGAGC CTAATGCTGG GAA-----AG ATT------G AGGGCAATAG 3052
BovB_Pg-RTE-Perameles_gunnii      GGA------- -CT-CATTGG --AAAAGATT CTGATATTGG G-----AAAG ATT-----GA AAGACAAAAG 1240
BovB_Io-RTE-Isoodon_obesulus      GGA------- -CT-TAATGG GGAAAAAATT TTGATGTTGA GGGAA-AAAG ATT-----AA AAGTAAAAGA 1706
BovB_Bp-RTE-Burramys_parvus       AGA------- -CT-CATTGG --AAAAGACC CTGATGTTGG G-----AAAG ATT-----GA AGG-CAAAAG 1822
BovB_Pb-RTE-Petaurus_breviceps    GGA------- -CT-CATTGG --AAAAGACC CTGATGTTAG G-----AAAG ATT-----GA AGG-CAAAAG 1828
BovB_Ma-RTE-Macropus_antilopin    GGA------- -CT-CATTGG --AAAAGACC CTGATGTTGG G-----AAAG ATT-----GA AGG-CAAAAG 3168
BovB_Af-RTE-Antechinus_flavipe    GGA------- -CTTCATTGG --AAAAGATC CTGATATTGG G-----AAAG ATT-----GA AGG-AAAAAG 2382
BovB_Ac-RTE-Anolis_carolinensi    GAAA------ ----GCTTGG --AAAAGATC ATGATGCTGG GGA-----AA ATG------G AAGGAAAAAG 3031
BovB_BM-RTE-Bombyx_mori           ---------- --ACCGGATA G-CCT-AGAA ATACTATTCG TGA----CAG ----GGAAGA TCGAGGGAAA 2860
BovB_Dr-RTE-Christinus            ---------- --CTCTATGG A-AAC-AGCT TTAATGCTCG GTG----CAG --TCAGTGGA TCAAGAAGAA 2927
BovB_La-RTE-Loxodonta_africana    TCA------- --GTCCCTGG A-GAA-GGAC ATCATGCTTG GCA----AAG --TACAGAGT CAGCGGAAAA 3053
BovB_Pc-RTE-Procavia_capensis     TCA------- --GTCCCTGG A-GAA-GGAC ATCATGCTTG GTA----GAG --TGGAGGGT CAGTGAAAAA 3053
BovB_Et-RTE-Christinus            ---------- ---------- ---------- ---------- ---------- ---------- ---------- 2573
BovB_Ta-RTE-Tachyglossus_acule    TAA------- --TTCTCTGG A-GAA-GACA CTAATGCTAG GAA----AAG --TCCAGGGA AAACGTGGAA 3193
BovB_Plat-RTE-Ornithorhynchus     TAA------- --TTCTCTGG A-GAA-GACA CTAATGCTAG GAA----AAG --TCCAGGGA AAACGTGGAA 3182
BovB_Ec-RTE-Equus_caballus        GGA------- --TTCTTTGG A-AAA-GACA ATAATGCTGG GAA----AAG --TAGAAGGC AGCAGGAAAA 2953
BovB_Cg-RTE-Christinus            AGA------- --GTCACTGG A-AAAAGACA ATAATGCTAG GAA----AAA GTTGAAAGGC AGCAGGAAAA 2492
BovB_Bh-RTE-Bothriocroton_hydr    GGGA------ --CTCTTTAG --AAAAGACT TTCATGCTGG GAA-----AG ATC------G AAAACAAAAT 1501
BovB_Ml-RTE-Gehyra_variegata      ---------- ---------- ---------- ---------- ---------- ---------- ---------- 1449
Sly-rnd1_family284-LINE/RTEBov    ---------- ---------- ---------- ---------- ---------- ---------- ---------- 56
Sly-rnd5_family2019-LINE/RTEBo    ---------- ---------- ---------- ---------- ---------- ---------- ---------- 477
Sly-rnd3_family322-LINE/RTEBov    ---------- ---------- ---------- ---------- ---------- ---------- ---------- 568
Sly-rnd5_family1698-LINE/RTEBo    ---------- ---------- ---------- ---------- ---------- ---------- ---------- 231
Sly-rnd1_family795-LINE/RTEBov    ---------- ---------- ---------- ---------- ---------- ---------- ---------- 538
Stu-rnd-5_family-3162_LINE_RTE    ---------- ---------- ---------- ---------- ---------- ---------- ---------- 2547
Sly-rnd6_family1960-LINE/RTEBo    ---------- ---------- ---------- ---------- ---------- ---------- ---------- 1316
Stu-rnd-5_family-2493_LINE_RTE    ---------- ---------- ---------- ---------- ---------- ---------- ---------- 215
Sly-rnd5_family447-LINE/RTEBov    ---------- ---------- ---------- ---------- ---------- ---------- ---------- 564
Stu-rnd-6_family-2093_LINE_RTE    ---------- ---------- ---------- ---------- ---------- ---------- ---------- 105
Consensus                         ---------- ----C--T-G ----A-GA-- -T-ATG-T-G G-------AG ---------- --------A-
```

Conservation

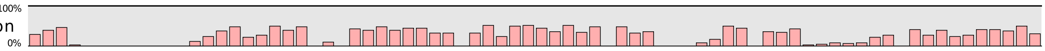

```
                                 4,220                 4,240                 4,260
Stu-rnd-1_family-417_LINE_RTE-   ---------- ---------- ---------- ---------- ---------- ---------- ---------- 306
Sly-rnd1_family377-LINE/RTEBov   ---------- ---------- ---------- ---------- ---------- ---------- ---------- 135
Stu-rnd-1_family-489_LINE_RTE-   ---------- ---------- ---------- ---------- ---------- ---------- ---------- 87
Stu-rnd-1_family-607_LINE_RTE-   ---------- ---------- ---------- ---------- ---------- ---------- ---------- 671
Stu-rnd-1_family-645_LINE_RTE-   ---------- ---------- ---------- ---------- ---------- ---------- ---------- 437
Sly-rnd4_family81-LINE/RTEBovB   ---------- ---------- ---------- ---------- ---------- ---------- ---------- 861
Stu-rnd-6_family-408_LINE_RTE-   ---------- ---------- ---------- ---------- ---------- ---------- ---------- 393
Stu-rnd-1_family-351_LINE_RTE-   ---------- ---------- ---------- ---------- ---------- ---------- ---------- 609
Stu-rnd-1_family-382_LINE_RTE-   ---------- ---------- ---------- ---------- ---------- ---------- ---------- 347
Sly-rnd6_family746-LINE/RTEBov   ---------- ---------- ---------- ---------- ---------- ---------- ---------- 122
Stu-rnd-5_family-325_LINE_RTE-   ---------- ---------- ---------- ---------- ---------- ---------- ---------- 446
Sly-rnd4_family509-LINE/RTEBov   ---------- ---------- ---------- ---------- ---------- ---------- ---------- 743
Stu-rnd-1_family-513_LINE_RTE-   ---------- ---------- ---------- ---------- ---------- ---------- ---------- 65
Sly-rnd5_family1442-LINE/RTEBo   ---------- ---------- ---------- ---------- ---------- ---------- ---------- 119
BovB_Gl-RTE-Gehyra_lazelli       AAGAAGGGGA CGACAAAGGA TGAGATGGTT AGACAGTGTC A--------- --TCGAGGCA ACA-----AA 3254
BovB_Gv-RTE-Gehyra_variegata     AAGAAGGGGA CGACAAAGGA TGAGATGGTT AGACAGTGTC A--------- --TCGAGGCA ACA-----AA 3181
BovB_PMo-RTE-Python_molurus      GAGAAGGGGA CGGCAGAGGA TGAGATGGTT AGATAGCATC A--------- --CTGAAGCA ATG-----AG 3112
BovB_Oa-RTE-Ovis_aries           GAGAAGGGGA TGACAGAGGA TGAGATGGTT GGATGGCATC A--------- --CCGACTCA ATG-----GA 3149
BovB-RTE-Bos_taurus              GAGAAGGGGA CGACAGAGGA TGAGATGGCT GGATGGCATC A--------- --CTGACTCG ATG-----GA 3737
BovB_Me-RTE-Macropus_eugenii     ---------- ---------- ---------- ---------- ---------- ---------- ---------- 2564
BovB_Ano-RTE-Amphibolurus_norr   GAGAAGGGGA CGACCGAGGA TGAGATGGCT AGACAGTGTC A--------- --TCGAAGCA ACC-----AA 3186
BovB_Gd-RTE-Eremiascincus_doug   GAGAAGGGGG TGGCAGAGGA TGAGATGGTT AGATAGTGTC A--------- --CCGAAGCA ACA-----AA 2776
BovB_Er-RTE-Eremiascincus_rich   GAGAAGGGGG CGGCAGAGGA TGAGATGGTT AGATAGTGTC A--------- --CCGAAGCA ATG-----AA 3310
BovB_Ca-RTE-Ctenotus_atlas       GAGAAGGGGG CGGCAGAGGA TGAGATGGTT AGATAGTGTC A--------- --CCGAAGCG ATG-----AA 3302
BovB_Es-RTE-Egernia_stokesii     GAGAAGGGGA CGGCAGAGGA TGAGATGGTT AGATAGTGTC A--------- --CGGAGGCA ATG-----AA 3137
BovB_Tr-RTE-Tiliqua_rugosa       GAGAAGGGGA CAGCAGAGGA TGAGATGGTT AGATAGTGTC A--------- --CAGAGGCA ATG-----AA 3150
BovB_ALi-RTE-Amblyomma_limbatu   AAGGAGGGGA CAGCATAGGA TGAAGTGGCT AGATTGAGTC A--------- --TAGAAGCA GTT-----AG 3339
BovB_Hs-RTE-Hydrophis_spiralis   AAGAAGGGGA CGGCAGAGAA TGAGGTGGCT GGATGGAGTC AGTAGGCATG AGCTGAAGCA GTCCAATGGG 1585
BovB_VA-RTE-Vipera_ammodytes     AAGAATGGGA CGACAGAGAA TGAGGGTGCT GGATGGAGTC A--------- --CTGAAGCA GTA-----GG 3107
BovB_ACo-RTE-Agkistrodon_conto   AAGAAGGGGA CGACAGAGAA TGAGGTGGCT GGATGGAGTC A--------- --CTGAAGCA GTA-----GG 3106
BovB_Pg-RTE-Perameles_gunnii     GAAAAGAGGA TGGCAGAAGA TGAGATGAAT AGATAGTAG- ---------T CATGGAAACA ATG-----AA 1295
BovB_Io-RTE-Isoodon_obesulus     AAAAAAAGGA TGACAGATCA TAAGATATAT AGAAAGT-G- ---------T CATAGAAACA ATG-----AA 1760
BovB_Bp-RTE-Burramys_parvus      GAGAAGGGGA TGGCAGAGGA TGAGATGGAT AGATAGTGGC T--------T CATGGAAACA ATG-----AA 1879
BovB_Pb-RTE-Petaurus_breviceps   GAAAAGGGGA TAGCAGAAGA TGAGATGGAT AGATAGTG-- ---------T CATGGAAACA ATG-----AA 1882
BovB_Ma-RTE-Macropus_antilopin   GAAAAGGGGA TGGCAGAGGA TGAGATGGAT AGATAGTG-- ---------T CATGGAAGCA ATG-----AA 3222
BovB_Af-RTE-Antechinus_flavipe   TAAAAAGGGA TGTCAGATAA TAAAATAAAT AAAAAGTA-- ---------T CATAGAAATA ATA-----AA 2436
BovB_Ac-RTE-Anolis_carolinensi   GAAGAGAGGC CGACCAAGGG CAAGATGGAT GGA-GGTATC C--------- --TTGAAGTG ACT-----GG 3084
BovB_BM-RTE-Bombyx_mori          GAG------- ---------- ---------- ---------- ---------- ---------- ---------- 2863
BovB_Dr-RTE-Christinus           GAGG--GCGT CAGAGAAC-- -ACGATGGCT GGACAA-TAT C--------- -AAGACGGAC TTG------A 2975
BovB_La-RTE-Loxodonta_africana   GAGG--AAGA CCCTCAAA-- -GAGATGGAT TGACAC-AGT G--------- -GCTGCAACA ATG------A 3101
BovB_Pc-RTE-Procavia_capensis    GAGG--AAGA CCCTCGAT-- -GAGATGGAT TGACAC-AGT G--------- -GCTGCAACA ATG------G 3101
BovB_Et-RTE-Christinus           ---------- ---------- ---------- ---------- ---------- ---------- ---------- 2573
BovB_Ta-RTE-Tachyglossus_acule   GAGG--CAGA CCAGCAGC-- -TAGATGGAT AGAGAC-CAT A--------- -ACAACAATA ATG------G 3241
BovB_Plat-RTE-Ornithorhynchus    GAGG--CAGA CCGGCAGC-- -TAGATGGAT AGAGAC-CAT A--------- -ACAACGATA ACG------G 3230
BovB_Ec-RTE-Equus_caballus       GAGG--AAGA CCAAATAT-- -GAGATGGAT TGACTC-CAT A--------- -AAGGAAGCC ATA------G 3001
BovB_Cg-RTE-Christinus           GAGGGAAAGA CCCAACATCA TGAGATGGAT TGACTCGTAT A--------- -AAGGAAGCC ATGAC---GG 2549
BovB_Bh-RTE-Bothriocroton_hydr   GAGAAACGGT CGGCAGAGGA TGAAATGGTT CGATATTGTC A--------- --CCGAAGCG ACA------A 1554
BovB_Ml-RTE-Gehyra_variegata     ---------- ---------- ---------- ---------- ---------- ---------- ---------- 1449
Sly-rnd1_family284-LINE/RTEBov   ---------- ---------- ---------- ---------- ---------- ---------- ---------- 56
Sly-rnd5_family2019-LINE/RTEBo   ---------- ---------- ---------- ---------- ---------- ---------- ---------- 477
Sly-rnd3_family322-LINE/RTEBov   ---------- ---------- ---------- ---------- ---------- ---------- ---------- 568
Sly-rnd5_family1698-LINE/RTEBo   ---------- ---------- ---------- ---------- ---------- ---------- ---------- 231
Sly-rnd1_family795-LINE/RTEBov   ---------- ---------- ---------- ---------- ---------- ---------- ---------- 538
Stu-rnd-5_family-3162_LINE_RTE   ---------- ---------- ---------- ---------- ---------- ---------- ---------- 2547
Sly-rnd6_family1960-LINE/RTEBo   ---------- ---------- ---------- ---------- ---------- ---------- ---------- 1316
Stu-rnd-5_family-2493_LINE_RTE   ---------- ---------- ---------- ---------- ---------- ---------- ---------- 215
Sly-rnd5_family447-LINE/RTEBov   ---------- ---------- ---------- ---------- ---------- ---------- ---------- 564
Stu-rnd-6_family-2093_LINE_RTE   ---------- ---------- ---------- ---------- ---------- ---------- ---------- 105
Consensus                        -A------G- ------A--- --A--T---T -GA------- ---------- ---------- ----------
```

Conservation

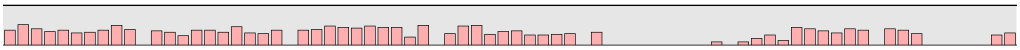

```
                                    4,280                    4,300                    4,320                    4,340
Stu-rnd-1_family-417_LINE_RTE-      ---------- ---------- ---------- ---------- ---------- ---------- ---------- 306
Sly-rnd1_family377-LINE/RTEBov      ---------- ---------- ---------- ---------- ---------- ---------- ---------- 135
Stu-rnd-1_family-489_LINE_RTE-      ---------- ---------- ---------- ---------- ---------- ---------- ---------- 87
Stu-rnd-1_family-607_LINE_RTE-      ---------- ---------- ---------- ---------- ---------- ---------- ---------- 671
Stu-rnd-1_family-645_LINE_RTE-      ---------- ---------- ---------- ---------- ---------- ---------- ---------- 437
Sly-rnd4_family81-LINE/RTEBovB      ---------- ---------- ---------- ---------- ---------- ---------- ---------- 861
Stu-rnd-6_family-408_LINE_RTE-      ---------- ---------- ---------- ---------- ---------- ---------- ---------- 393
Stu-rnd-1_family-351_LINE_RTE-      ---------- ---------- ---------- ---------- ---------- ---------- ---------- 609
Stu-rnd-1_family-382_LINE_RTE-      ---------- ---------- ---------- ---------- ---------- ---------- ---------- 347
Sly-rnd6_family746-LINE/RTEBov      ---------- ---------- ---------- ---------- ---------- ---------- ---------- 122
Stu-rnd-5_family-325_LINE_RTE-      ---------- ---------- ---------- ---------- ---------- ---------- ---------- 446
Sly-rnd4_family509-LINE/RTEBov      ---------- ---------- ---------- ---------- ---------- ---------- ---------- 743
Stu-rnd-1_family-513_LINE_RTE-      ---------- ---------- ---------- ---------- ---------- ---------- ---------- 65
Sly-rnd5_family1442-LINE/RTEBo      ---------- ---------- ---------- ---------- ---------- ---------- ---------- 119
BovB_Gl-RTE-Gehyra_lazelli          CA-TGAAATT --GAGCGAAC TCAG-----G AACATGACAG AAGACAGGAA -GGCCT-GGC GTGGTGTGGT 3314
BovB_Gv-RTE-Gehyra_variegata        CA-TGAAATT --GAGCGAAC TCAG-----G AGCATGACAG AAGACAGGAA -GGCCT-GGC GTGGTGTGGT 3241
BovB_PMo-RTE-Python_molurus         CA-TGAAACT --GAGCAAAC TCAG-----G GAGGCAGTGA AGGACAGGAA -GGCCT-GGC GCA-TATGGT 3171
BovB_Oa-RTE-Ovis_aries              CA-TGAGTTT --GAGCAAAC TCCG-----G GAGATGGTGA AGGACAGGGA -AGCCT-GGC GTGCTGCAGT 3209
BovB-RTE-Bos_taurus                 CG-TGAGTCT --GAGTGAAC TCCG-----G GAGTTGGTGA TGGACAGGGA -GGCCT-GGC GTGCTGCGAT 3797
BovB_Me-RTE-Macropus_eugenii        ---------- ---------- ---------- ---------- ---------- ---------- ---------- 2564
BovB_Ano-RTE-Amphibolurus_norr      CA-TGAATTT --GACACAAC TCCG-----G GAGGCAGTGG AAGATAGGAG -GGCCT-GGC GTGCTCTGGT 3246
BovB_Gd-RTE-Eremiascincus_doug      CA-TGAAATT --GGGCGAAC TCCG-----G AAGCTAGTGG AGGACAGGGA -GGCCT-GGC GTGCTGTGGT 2836
BovB_Er-RTE-Eremiascincus_rich      CA-TGAAATT --GGGCGAAC TCCG-----G AAGCTAGTGG AGGACAGGGA -GGCCT-GGC GTGCTGTGGT 3370
BovB_Ca-RTE-Ctenotus_atlas          CA-TGAAATT --GGGCGAAC TCCG-----G AAGCTAGTGG AGGACAGGGA -GGCCT-GGC GTGCTGTGGT 3362
BovB_Es-RTE-Egernia_stokesii        CA-TGAATTT --GGACAAAC TCCA-----G AAGTTAGTGG TAGACAGGGG -GGCCT-GGC GTGCTGTGGT 3197
BovB_Tr-RTE-Tiliqua_rugosa          CA-TGAATTT --GGACAAAC TCTG-----G AAGTTAGTGG TAGACAGGGG -GGCCT-GGC ATGCTGTGGT 3210
BovB_ALi-RTE-Amblyomma_limbatu      TG-CGAGCCT --AAGTGTAC ATCT-----G GAAATAGTGG AAGACAGGAA -GGCCTGGGA GGAGTGTTGT 3400
BovB_Hs-RTE-Hydrophis_spiralis      CA-TGAGCTT TCAAATGGAA AACTCTCCAG AAGATGGTAG AGGACAGGAA -GGCCTTGGA GGAACATTGT 1653
BovB_VA-RTE-Vipera_ammodytes        CG-TGAGCTT --AAATGGCC TCCA-----G AAGATGGTAG AGGACAGGAA -GGCCT-GGA GGAACATTGT 3167
BovB_ACo-RTE-Agkistrodon_conto      CG-TGAGCTT --AAATGGAC TCCA-----G AAGATGGTAG AGGACAGGAA -GGCCT-GGA GGAACGTTGT 3166
BovB_Pg-RTE-Perameles_gunnii        CAATGAACTT --GGACAGAC TTTGA----G AAGATGGAGG AGGATAGAA- -GGACCTGAC ATGCTATATT 1357
BovB_Io-RTE-Isoodon_obesulus        CA-TGAAATT --AAACAG-T CTTAA----A GATATAATAG AATATAAAA- -AAACCAGG- ATGCTATAAT 1819
BovB_Bp-RTE-Burramys_parvus         CA-TGAACTT --GGACAGAC TTTAG----A GAGATAGTGG AGGATAGAA- -GGACTTGGT GTACTAAGGT 1940
BovB_Pb-RTE-Petaurus_breviceps      CA-TGAACTT --GGACAGAC TT-GG----A GACATAGTGG AGGACAGAA- -GGGTCTGGC ATGCCATGGT 1942
BovB_Ma-RTE-Macropus_antilopin      CA-TGAACTT --GGACAGAC TTTG-----A GAGACAGTGG AAGATAGAAA AGGGGCAGGC ATGCTGTGGT 3284
BovB_Af-RTE-Antechinus_flavipe      AA-TGAGCTT --GTACATAC TTTA-----A GATACACTAG AGGATAAAA- -GAATCTAAT AATTTAAGGA 2496
BovB_Ac-RTE-Anolis_carolinensi      CT-TGACCTT --GAAGGAAC TGGG-----G GCGGTGACGG CCGACAGGGA --GCTCTGGC GTGGACTGGT 3144
BovB_BM-RTE-Bombyx_mori             ---------- ---------- ---------- ---------- ---------- ---------- ---------- 2863
BovB_Dr-RTE-Christinus              ACTTGAACAT --GGAACAAC TGAA-----A GAAGCGGTCA GAAATAGGAA --AGCATGGC AAATCATGAT 3036
BovB_La-RTE-Loxodonta_africana      GCTCAAGCAT --AACAACGA TTGT-----A AGCATGGCAC AGGACCAGGC --AGTGTTTC ATTCTGTTGT 3162
BovB_Pc-RTE-Procavia_capensis       GCTTAAGCAT --AAAAACGA TTGT-----G GACATGGCAC AAGACTGGGC --AGTGTTTC ATTCTGTTGT 3162
BovB_Et-RTE-Christinus              ---------- ---------- ---------- ---------- ---------- ---------- ---------- 2573
BovB_Ta-RTE-Tachyglossus_acule      AAGAACCATT --AGAAAGGT TGCG-----G ATTATGGCAG AGGACAGGAC --GTTCTGGA GAAAATATAT 3302
BovB_Plat-RTE-Ornithorhynchus       AAGAACCGTT --AGAAAGGT TGCG-----G ATTATGGCAG AGGACAGGAC --GTTCTGGA GAAAGTATAT 3291
BovB_Ec-RTE-Equus_caballus          GCATGAGTCT --ACAGAAGC TGAG-----T AGGGCTGTTG AGGACAGGAC --ATTGTGGA CATCACTCAT 3062
BovB_Cg-RTE-Christinus              CCCTCAGTTT --GCAAGACC TGAG-----C AAGGCTGTTA ACAATAGGAT --GTTTTGGA GGACATTAAT 2610
BovB_Bh-RTE-Bothriocroton_hydr      ACGTGAAATT --GGGCGAAC TCTC-----G AATCTAGTGA AGGACAGAGA -GGCCT--GC GTGCTGTGGC 1614
BovB_Ml-RTE-Gehyra_variegata        ---------- ---------- ---------- ---------- ---------- ---------- ---------- 1449
Sly-rnd1_family284-LINE/RTEBov      ---------- ---------- ---------- ---------- ---------- ---------- ---------- 56
Sly-rnd5_family2019-LINE/RTEBo      ---------- ---------- ---------- ---------- ---------- ---------- ---------- 477
Sly-rnd3_family322-LINE/RTEBov      ---------- ---------- ---------- ---------- ---------- ---------- ---------- 568
Sly-rnd5_family1698-LINE/RTEBo      ---------- ---------- ---------- ---------- ---------- ---------- ---------- 231
Sly-rnd1_family795-LINE/RTEBov      ---------- ---------- ---------- ---------- ---------- ---------- ---------- 538
Stu-rnd-5_family-3162_LINE_RTE      ---------- ---------- ---------- ---------- ---------- ---------- ---------- 2547
Sly-rnd6_family1960-LINE/RTEBo      ---------- ---------- ---------- ---------- ---------- ---------- ---------- 1316
Stu-rnd-5_family-2493_LINE_RTE      ---------- ---------- ---------- ---------- ---------- ---------- ---------- 215
Sly-rnd5_family447-LINE/RTEBov      ---------- ---------- ---------- ---------- ---------- ---------- ---------- 564
Stu-rnd-6_family-2093_LINE_RTE      ---------- ---------- ---------- ---------- ---------- ---------- ---------- 105

Consensus                           -----A---T ---------- ---------- ---------- ---A-A---- ---------- ---------T
```

Conservation

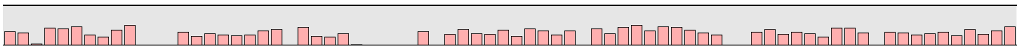

		4,360		4,380		4,400		
Stu-rnd-1_family-417_LINE_RTE-	----------	----------	----------	----------	----------	----------	----------	306
Sly-rnd1_family377-LINE/RTEBov	----------	----------	----------	----------	----------	----------	----------	135
Stu-rnd-1_family-489_LINE_RTE-	----------	----------	----------	----------	----------	----------	----------	87
Stu-rnd-1_family-607_LINE_RTE-	----------	----------	----------	----------	----------	----------	----------	671
Stu-rnd-1_family-645_LINE_RTE-	----------	----------	----------	----------	----------	----------	----------	437
Sly-rnd4_family81-LINE/RTEBovB	----------	----------	----------	----------	----------	----------	----------	861
Stu-rnd-6_family-408_LINE_RTE-	----------	----------	----------	----------	----------	----------	----------	393
Stu-rnd-1_family-351_LINE_RTE-	----------	----------	----------	----------	----------	----------	----------	609
Stu-rnd-1_family-382_LINE_RTE-	----------	----------	----------	----------	----------	----------	----------	347
Sly-rnd6_family746-LINE/RTEBov	----------	----------	----------	----------	----------	----------	----------	122
Stu-rnd-5_family-325_LINE_RTE-	----------	----------	----------	----------	----------	----------	----------	446
Sly-rnd4_family509-LINE/RTEBov	----------	----------	----------	----------	----------	----------	----------	743
Stu-rnd-1_family-513_LINE_RTE-	----------	----------	----------	----------	----------	----------	----------	65
Sly-rnd5_family1442-LINE/RTEBo	----------	----------	----------	----------	----------	----------	----------	119
BovB_Gl-RTE-Gehyra_lazelli	CCATGGGGTC	GCAAAGAGTC	--GGAGCCGA	CTGT--GCGA	CTGAACAACA	ACAACAAAA-	ACAACTAACA	3379
BovB_Gv-RTE-Gehyra_variegata	CCATGGGGTC	GCAAAGAGTC	--GGAGCCGA	CTGT--GCGA	CTGAACAACA	ACAACAGCTC	ATGATCTGAG	3307
BovB_PMo-RTE-Python_molurus	TCATGGGGTC	ACGAAGAGTC	--GGACACGA	CTTA--ACGA	CTGAACAACA	ACAACAATT-	CTATTCTATT	3236
BovB_Oa-RTE-Ovis_aries	CCATGGGGTC	ACAAAGAGTC	--AGACATGA	CTGA--GCGA	CTGAACAACA	ACAA------	----------	3259
BovB-RTE-Bos_taurus	TCATGGGGTC	GCAAAGAGTC	--GGACACGA	CTGA--GCGA	CTGAACTGAA	CTGA------	----------	3847
BovB_Me-RTE-Macropus_eugenii	----------	----------	----------	----------	----------	----------	----------	2564
BovB_Ano-RTE-Amphibolurus_norr	CCATGGGGTC	ACGAAGAGTC	--GGACACGA	CTAA--ACGA	CTAAACGAAC	GACGATGAAA	AAA-------	3305
BovB_Gd-RTE-Eremiascincus_doug	CCATGGGGTC	GCGAAGAGTC	--GGACACGA	CTTA--GCGA	CTGAACAACA	ACAATTACTA	GCAGCAGCCT	2902
BovB_Er-RTE-Eremiascincus_rich	CCATGGGGTC	GCGAAGAGTC	--GGACACGA	CTTA--GCGA	CTGAACAACA	ACAACAATTA	TTAAAGATTA	3436
BovB_Ca-RTE-Ctenotus_atlas	CCATGGGGTC	GCGAAGAGTC	--GGACACGA	CTTA--GCGA	CTGAACAACA	ACAACAACAA	A---------	3419
BovB_Es-RTE-Egernia_stokesii	CCATGGGGTC	ACGAAGAGTC	--GGACATGA	CTTA--ACGA	CTGAACAACA	ACAAAAACAA	CAAAACTGCA	3263
BovB_Tr-RTE-Tiliqua_rugosa	CCATGGGGTC	ACAAAGAGTC	--GGACATGA	CTTA--ACGA	CTGAACAACA	ACAA------	----------	3260
BovB_ALi-RTE-Amblyomma_limbatu	CCATCGGGTC	GCGATGGGTC	--GGACATGA	CTTTC-GCGA	CT-AACAACA	ATCAGTAAAA	AAAATAAGCA	3466
BovB_Hs-RTE-Hydrophis_spiralis	CCATGGGGTC	ATGATGGGTC	--AGACACGA	CTTT--GCAA	CT-AACAACA	AC------AA	CAAGTAAATA	1712
BovB_VA-RTE-Vipera_ammodytes	CCATAGGGTC	GCGATGGGTC	--GGACACGA	CTTC--GCAG	CT-AACAACA	AC------AA	CAATTCTATT	3226
BovB_ACo-RTE-Agkistrodon_conto	CCATGGGGTC	GCGATGGGTC	--GGACACGA	CTTC--GCAA	CT-AACAACA	AC------AA	CAATTCTATT	3225
BovB_Pg-RTE-Perameles_gunnii	GCAAA-----	-GGAAACAAA	--AAACTTAA	TTAA--ACAC	CAACAAAAAT	ATACCATAGA	TAAATAATAT	1417
BovB_Io-RTE-Isoodon_obesulus	TCAAAAACTC	AGGAAAAGTC	--AGACATGA	CTGA--ATGA	CTGAATAACA	ATCTCAT---	TCAATAGTAA	1882
BovB_Bp-RTE-Burramys_parvus	CCATGGAGTC	ATGAAAAGTC	--AGGAATGA	--AA--AAAA	CAAAAAAAAA	AAGAATTA--	-TGATACTCA	2001
BovB_Pb-RTE-Petaurus_breviceps	CCATGGACTC	ACAAAGACTG	--AGACGTGG	CTAA--ATGA	CTCAACAACA	ACTAGTTA--	-AAATAATAG	2005
BovB_Ma-RTE-Macropus_antilopin	CCATGGGGTC	ACAAAGAGTT	--GAACATAA	CGAA--AAAA	AACAACAACA	ACAACAAA--	-ACGTATAGC	3347
BovB_Af-RTE-Antechinus_flavipe	CCAGAAAGGC	CAAAAAAGGA	--AAAAAA--	--AA--ATAA	CTGAACAACA	ACAACAAT--	-AAGCAGAAA	2555
BovB_Ac-RTE-Anolis_carolinensi	CCATGAGGTC	ACGAAGAGTC	--GGAAACGA	CTAA--ACGA	----------	----------	----------	3180
BovB_BM-RTE-Bombyx_mori	----------	----------	----------	----------	----------	----------	----------	2863
BovB_Dr-RTE-Christinus	CCATAAAGTG	GCCGAAAGTC	--GGTGTCGA	CTGATTGGCT	AATCATCATC	ATAAT-----	----------	3089
BovB_La-RTE-Loxodonta_africana	ACATAGGGCC	ACTATGAGTC	--AGAACCGA	CTCGACGGCA	CCTAACAACA	A---------	----------	3211
BovB_Pc-RTE-Procavia_capensis	TCATAGGGTC	GCTATGAGTC	--AGAACCGA	CTCAATGGCA	CCTAACAACA	ACAACAACTC	T---------	3221
BovB_Et-RTE-Christinus	----------	----------	----------	----------	----------	----------	----------	2573
BovB_Ta-RTE-Tachyglossus_acule	CCATAGAGTC	GCTATGAATC	--GGAAATGA	CTCGATGGCA	CTTGATAATA	ATAATAATAA	TAACACTAAT	3370
BovB_Plat-RTE-Ornithorhynchus	CCATGGAGTC	GCTATGAATC	--GGAAACGA	CTCGACGGCA	CTTAATAATA	ATAATAATAA	----------	3349
BovB_Ec-RTE-Equus_caballus	TCATAGGGTC	ACCAGGAGTC	--AGAGCTGA	CTTGACGGCA	TGTAACA-AC	AA--------	----------	3111
BovB_Cg-RTE-Christinus	TCATAGGGTT	ACCATAAATC	CAGGAAGCAA	CTTGATGGCA	CTTAACACAC	ACACAAATAG	GCAAATATGA	2680
BovB_Bh-RTE-Bothriocroton_hydr	CCATGAGGTC	GCGAAGGGTC	--GGACACGA	ATTA--GCGA	ATGGACAACA	ACATATTGAT	ATCCGGCTTA	1680
BovB_Ml-RTE-Gehyra_variegata	----------	----------	----------	----------	----------	----------	----------	1449
Sly-rnd1_family284-LINE/RTEBov	----------	----------	----------	----------	----------	----------	----------	56
Sly-rnd5_family2019-LINE/RTEBo	----------	----------	----------	----------	----------	----------	----------	477
Sly-rnd3_family322-LINE/RTEBov	----------	----------	----------	----------	----------	----------	----------	568
Sly-rnd5_family1698-LINE/RTEBo	----------	----------	----------	----------	----------	----------	----------	231
Sly-rnd1_family795-LINE/RTEBov	----------	----------	----------	----------	----------	----------	----------	538
Stu-rnd-5_family-3162_LINE_RTE	----------	----------	----------	----------	----------	----------	----------	2547
Sly-rnd6_family1960-LINE/RTEBo	----------	----------	----------	----------	----------	----------	----------	1316
Stu-rnd-5_family-2493_LINE_RTE	----------	----------	----------	----------	----------	----------	----------	215
Sly-rnd5_family447-LINE/RTEBov	----------	----------	----------	----------	----------	----------	----------	564
Stu-rnd-6_family-2093_LINE_RTE	----------	----------	----------	----------	----------	----------	----------	105
Consensus	-CA-------	---A----T-	----A-----	----------	----A-----	----------	----------	

Conservation

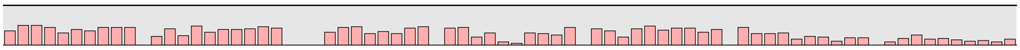

	4,420		4,440		4,460		4,480	
Stu-rnd-1_family-417_LINE_RTE-	----------	----------	----------	----------	----------	----------	----------	306
Sly-rnd1_family377-LINE/RTEBov	----------	----------	----------	----------	----------	----------	----------	135
Stu-rnd-1_family-489_LINE_RTE-	----------	----------	----------	----------	----------	----------	----------	87
Stu-rnd-1_family-607_LINE_RTE-	----------	----------	----------	----------	----------	----------	----------	671
Stu-rnd-1_family-645_LINE_RTE-	----------	----------	----------	----------	----------	----------	----------	437
Sly-rnd4_family81-LINE/RTEBovB	----------	----------	----------	----------	----------	----------	----------	861
Stu-rnd-6_family-408_LINE_RTE-	----------	----------	----------	----------	----------	----------	----------	393
Stu-rnd-1_family-351_LINE_RTE-	----------	----------	----------	----------	----------	----------	----------	609
Stu-rnd-1_family-382_LINE_RTE-	----------	----------	----------	----------	----------	----------	----------	347
Sly-rnd6_family746-LINE/RTEBov	----------	----------	----------	----------	----------	----------	----------	122
Stu-rnd-5_family-325_LINE_RTE-	----------	----------	----------	----------	----------	----------	----------	446
Sly-rnd4_family509-LINE/RTEBov	----------	----------	----------	----------	----------	----------	----------	743
Stu-rnd-1_family-513_LINE_RTE-	----------	----------	----------	----------	----------	----------	----------	65
Sly-rnd5_family1442-LINE/RTEBo	----------	----------	----------	----------	----------	----------	----------	119
BovB_Gl-RTE-Gehyra_lazelli	CTGCCGCATA	TCAACAATCC	ATGGCCCATA	AAATTTATAC	AAAAAAA-AA	TCAAAATACT	CTAATACAAT	3448
BovB_Gv-RTE-Gehyra_variegata	CCACAGT-CA	GCTCCAGGTC	TTGTTTTACT	GACTGTATAC	AGTATCACAA	TCTACAGACA	GTCACCAAAT	3376
BovB_PMo-RTE-Python_molurus	CTGTTCTATT	CTATTCTATT	CTATTCTATT	CTAT-TCTGT	TCTGTTCTGT	TCT-------	----------	3288
BovB_Oa-RTE-Ovis_aries	----------	----------	----------	----------	----------	----------	----------	3259
BovB-RTE-Bos_taurus	----------	----------	----------	----------	----------	----------	----------	3847
BovB_Me-RTE-Macropus_eugenii	----------	----------	----------	----------	----------	----------	----------	2564
BovB_Ano-RTE-Amphibolurus_norr	----------	----------	----------	----------	----------	----------	----------	3305
BovB_Gd-RTE-Eremiascincus_doug	GCCAA-----	----CAACAA	TGTACAATTT	ACG-TTGAAA	TACAGCATAT	AAATACTATT	AACTG-----	2957
BovB_Er-RTE-Eremiascincus_rich	AACAAATAAT	AATATGAAAA	TGTAAAATAT	CTAATTAACA	TTTCACTTGT	AACCCTAGTC	AATCCTAAAA	3506
BovB_Ca-RTE-Ctenotus_atlas	----------	----------	----------	----------	----------	----------	----------	3419
BovB_Es-RTE-Egernia_stokesii	AAAAAAATAA	TTCAAATAAC	AACAGAGAA-	----------	----------	----------	----------	3292
BovB_Tr-RTE-Tiliqua_rugosa	----------	----------	----------	----------	----------	----------	----------	3260
BovB_ALi-RTE-Amblyomma_limbatu	ATAAAAAAAT	AAATTTAATT	ATCAATATTT	ACTTCATATC	AAATTTACTT	ATAT--GACT	ATATTTCAAA	3534
BovB_Hs-RTE-Hydrophis_spiralis	CAAAGAAAAT	AA-CCCAACA	ATTA-TATTA	AATTGCAATT	ATTTACATCA	ATACCAAATT	AAACAAAGAA	1780
BovB_VA-RTE-Vipera_ammodytes	CTATTCTATT	CTATTCTATA	TTCTGGTTCT	GTTCTATATT	CTATTCTATT	CTATTCT---	----------	3283
BovB_ACo-RTE-Agkistrodon_conto	CTATTCTATT	CTATTCTATT	CTATTCTATT	CTATTCTATT	CTATTCTATT	CTATTCT---	----------	3282
BovB_Pg-RTE-Perameles_gunnii	AAT-TAATTT	CAAATAAATT	CT--TCATTC	AGAGAATGGT	CATTGAAAGA	GATTTCCCAG	GAAACAGTGT	1484
BovB_Io-RTE-Isoodon_obesulus	AAT-ATTATT	CAATTAAATT	CAAATAAACT	ATATTATCCT	TGCCTGTACA	TATTTTCCAA	----------	1941
BovB_Bp-RTE-Burramys_parvus	AAA-TCAATA	TTTTTATTTT	TTAGAAAACT	ATTTAACATA	AGGCAAAATC	TGCTGAACAC	AGAAAAT---	2067
BovB_Pb-RTE-Petaurus_breviceps	AAA---AGTG	GACCAG----	--AGGAAAAG	GAAGGAAGCA	AGGCAGGAAG	GTATGAAGAA	AGAAACCTGT	2066
BovB_Ma-RTE-Macropus_antilopin	ACT-ACGCTA	CAGTGGGACT	GT-GCTGGCT	TTAGTATCTA	AAAGACCT--	----------	----------	3393
BovB_Af-RTE-Antechinus_flavipe	ACTCTCAAGG	GATATACATT	C---------	----------	----------	----------	----------	2576
BovB_Ac-RTE-Anolis_carolinensi	----------	----------	----------	----------	----------	----------	----------	3180
BovB_BM-RTE-Bombyx_mori	----------	----------	----------	----------	----------	----------	----------	2863
BovB_Dr-RTE-Christinus	----------	----------	----------	----------	----------	----------	----------	3089
BovB_La-RTE-Loxodonta_africana	----------	----------	----------	----------	----------	----------	----------	3211
BovB_Pc-RTE-Procavia_capensis	----------	----------	----------	----------	----------	----------	----------	3221
BovB_Et-RTE-Christinus	----------	----------	----------	----------	----------	----------	----------	2573
BovB_Ta-RTE-Tachyglossus_acule	TGGAACACTG	TGTG------	----------	----------	----------	----------	----------	3384
BovB_Plat-RTE-Ornithorhynchus	----------	----------	----------	----------	----------	----------	----------	3349
BovB_Ec-RTE-Equus_caballus	----------	----------	----------	----------	----------	----------	----------	3111
BovB_Cg-RTE-Christinus	TCACATAAAA	TTTCCCTCTG	TCTGTACTCT	TCTCCCTGAA	AACAACTGTT	CTCTAGCTGA	TTCCCCTCCC	2750
BovB_Bh-RTE-Bothriocroton_hydr	TCTTACCAGT	ACTTACCTA-	----------	----------	----------	----------	----------	1699
BovB_Ml-RTE-Gehyra_variegata	----------	----------	----------	----------	----------	----------	----------	1449
Sly-rnd1_family284-LINE/RTEBov	----------	----------	----------	----------	----------	----------	----------	56
Sly-rnd5_family2019-LINE/RTEBo	----------	----------	----------	----------	----------	----------	----------	477
Sly-rnd3_family322-LINE/RTEBov	----------	----------	----------	----------	----------	----------	----------	568
Sly-rnd5_family1698-LINE/RTEBo	----------	----------	----------	----------	----------	----------	----------	231
Sly-rnd1_family795-LINE/RTEBov	----------	----------	----------	----------	----------	----------	----------	538
Stu-rnd-5_family-3162_LINE_RTE	----------	----------	----------	----------	----------	----------	----------	2547
Sly-rnd6_family1960-LINE/RTEBo	----------	----------	----------	----------	----------	----------	----------	1316
Stu-rnd-5_family-2493_LINE_RTE	----------	----------	----------	----------	----------	----------	----------	215
Sly-rnd5_family447-LINE/RTEBov	----------	----------	----------	----------	----------	----------	----------	564
Stu-rnd-6_family-2093_LINE_RTE	----------	----------	----------	----------	----------	----------	----------	105
Consensus	----------	----------	----------	----------	----------	----------	----------	

Conservation

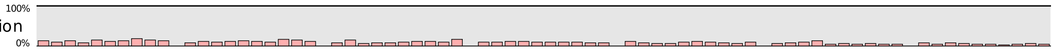

```
                                     4,500                          4,520                          4,540
                                       |                              |                              |
Stu-rnd-1_family-417_LINE_RTE-     ---------- ---------- ---------- ---------- ---------- ---------- ---------- 306
Sly-rnd1_family377-LINE/RTEBov     ---------- ---------- ---------- ---------- ---------- ---------- ---------- 135
Stu-rnd-1_family-489_LINE_RTE-     ---------- ---------- ---------- ---------- ---------- ---------- ---------- 87
Stu-rnd-1_family-607_LINE_RTE-     ---------- ---------- ---------- ---------- ---------- ---------- ---------- 671
Stu-rnd-1_family-645_LINE_RTE-     ---------- ---------- ---------- ---------- ---------- ---------- ---------- 437
Sly-rnd4_family81-LINE/RTEBovB     ---------- ---------- ---------- ---------- ---------- ---------- ---------- 861
Stu-rnd-6_family-408_LINE_RTE-     ---------- ---------- ---------- ---------- ---------- ---------- ---------- 393
Stu-rnd-1_family-351_LINE_RTE-     ---------- ---------- ---------- ---------- ---------- ---------- ---------- 609
Stu-rnd-1_family-382_LINE_RTE-     ---------- ---------- ---------- ---------- ---------- ---------- ---------- 347
Sly-rnd6_family746-LINE/RTEBov     ---------- ---------- ---------- ---------- ---------- ---------- ---------- 122
Stu-rnd-5_family-325_LINE_RTE-     ---------- ---------- ---------- ---------- ---------- ---------- ---------- 446
Sly-rnd4_family509-LINE/RTEBov     ---------- ---------- ---------- ---------- ---------- ---------- ---------- 743
Stu-rnd-1_family-513_LINE_RTE-     ---------- ---------- ---------- ---------- ---------- ---------- ---------- 65
Sly-rnd5_family1442-LINE/RTEBo     ---------- ---------- ---------- ---------- ---------- ---------- ---------- 119
BovB_Gl-RTE-Gehyra_lazelli         -------ATA TGGCTAAGAA AAAGAAAGAT CTACACACAT GT-CTCCTGC TCTAAAAAAA CAAA------ 3504
BovB_Gv-RTE-Gehyra_variegata       GATGTAGACA TTCCCATTAC AGACTAGACC CCTTAATAAT GTACTTTTGA AGAGCGGCAG CAAAGGTTAG 3446
BovB_PMo-RTE-Python_molurus        ---------- ---------- ---------- ---------- ---------- ---------- ---------- 3288
BovB_Oa-RTE-Ovis_aries             ---------- ---------- ---------- ---------- ---------- ---------- ---------- 3259
BovB-RTE-Bos_taurus                ---------- ---------- ---------- ---------- ---------- ---------- ---------- 3847
BovB_Me-RTE-Macropus_eugenii       ---------- ---------- ---------- ---------- ---------- ---------- ---------- 2564
BovB_Ano-RTE-Amphibolurus_norr     ---------- ---------- ---------- ---------- ---------- ---------- ---------- 3305
BovB_Gd-RTE-Eremiascincus_doug     --ATTGACTT TTTTAAAAAA GGAACAGACT TTCCCCCA-A ACACAAAACC -TCCCAG--C GTCCCAGCTC 3021
BovB_Er-RTE-Eremiascincus_rich     ATATTGCCAA AGTGAAAAAA GAAGCATATA TATTTTCAGA ACACCTAACT ATAATAGACC TATCAAACAC 3576
BovB_Ca-RTE-Ctenotus_atlas         ---------- ---------- ---------- ---------- ---------- ---------- ---------- 3419
BovB_Es-RTE-Egernia_stokesii       ---------- ---------- ---------- ---------- ---------- ---------- ---------- 3292
BovB_Tr-RTE-Tiliqua_rugosa         ---------- ---------- ---------- ---------- ---------- ---------- ---------- 3260
BovB_ALi-RTE-Amblyomma_limbatu     ATATTACAAA CAGTAGTATC GAAATATATA TTGTAGAAAC CATGAGCATT AAACAAGTAT ACTATTGATT 3604
BovB_Hs-RTE-Hydrophis_spiralis     GTACTN-AAA AGATAATATA CTAAAGCATA TTCTTGAAAT GATTAAAGCA AAATTGCTAA GACCGTTATT 1849
BovB_VA-RTE-Vipera_ammodytes       ---------- ---------- ---------- ---------- ---------- ---------- ---------- 3283
BovB_ACo-RTE-Agkistrodon_conto     ---------- ---------- ---------- ---------- ---------- ---------- ---------- 3282
BovB_Pg-RTE-Perameles_gunnii       ACTTGGAAC- TGGAATAATT GGGTTGATGT TGTGGACACT GATTGTGGCA AGAAAACTTC TGATCTAGAA 1553
BovB_Io-RTE-Isoodon_obesulus       ---------- ---------- ---------- ---------- ---------- ---------- ---------- 1941
BovB_Bp-RTE-Burramys_parvus        ---------- ---------- ---------- ---------- ---------- ---------- ---------- 2067
BovB_Pb-RTE-Petaurus_breviceps     CTATAACTTA TTAGATCATC TTTTTAAAGT TGCTGTGACA AAAA------ ---------- ---------- 2110
BovB_Ma-RTE-Macropus_antilopin     ---------- ---------- ---------- ---------- ---------- ---------- ---------- 3393
BovB_Af-RTE-Antechinus_flavipe     ---------- ---------- ---------- ---------- ---------- ---------- ---------- 2576
BovB_Ac-RTE-Anolis_carolinensi     ---------- ---------- ---------- ---------- ---------- ---------- ---------- 3180
BovB_BM-RTE-Bombyx_mori            ---------- ---------- ---------- ---------- ---------- ---------- ---------- 2863
BovB_Dr-RTE-Christinus             ---------- ---------- ---------- ---------- ---------- ---------- ---------- 3089
BovB_La-RTE-Loxodonta_africana     ---------- ---------- ---------- ---------- ---------- ---------- ---------- 3211
BovB_Pc-RTE-Procavia_capensis      ---------- ---------- ---------- ---------- ---------- ---------- ---------- 3221
BovB_Et-RTE-Christinus             ---------- ---------- ---------- ---------- ---------- ---------- ---------- 2573
BovB_Ta-RTE-Tachyglossus_acule     ---------- ---------- ---------- ---------- ---------- ---------- ---------- 3384
BovB_Plat-RTE-Ornithorhynchus      ---------- ---------- ---------- ---------- ---------- ---------- ---------- 3349
BovB_Ec-RTE-Equus_caballus         ---------- ---------- ---------- ---------- ---------- ---------- ---------- 3111
BovB_Cg-RTE-Christinus             CCCCCCCCAT TAGTGGAATT CCATTG---- ---------- ---------- ---------- ---------- 2776
BovB_Bh-RTE-Bothriocroton_hydr     ---------- ---------- ---------- ---------- ---------- ---------- ---------- 1699
BovB_Ml-RTE-Gehyra_variegata       ---------- ---------- ---------- ---------- ---------- ---------- ---------- 1449
Sly-rnd1_family284-LINE/RTEBov     ---------- ---------- ---------- ---------- ---------- ---------- ---------- 56
Sly-rnd5_family2019-LINE/RTEBo     ---------- ---------- ---------- ---------- ---------- ---------- ---------- 477
Sly-rnd3_family322-LINE/RTEBov     ---------- ---------- ---------- ---------- ---------- ---------- ---------- 568
Sly-rnd5_family1698-LINE/RTEBo     ---------- ---------- ---------- ---------- ---------- ---------- ---------- 231
Sly-rnd1_family795-LINE/RTEBov     ---------- ---------- ---------- ---------- ---------- ---------- ---------- 538
Stu-rnd-5_family-3162_LINE_RTE     ---------- ---------- ---------- ---------- ---------- ---------- ---------- 2547
Sly-rnd6_family1960-LINE/RTEBo     ---------- ---------- ---------- ---------- ---------- ---------- ---------- 1316
Stu-rnd-5_family-2493_LINE_RTE     ---------- ---------- ---------- ---------- ---------- ---------- ---------- 215
Sly-rnd5_family447-LINE/RTEBov     ---------- ---------- ---------- ---------- ---------- ---------- ---------- 564
Stu-rnd-6_family-2093_LINE_RTE     ---------- ---------- ---------- ---------- ---------- ---------- ---------- 105
Consensus                          ---------- ---------- ---------- ---------- ---------- ---------- ----------
```

Conservation

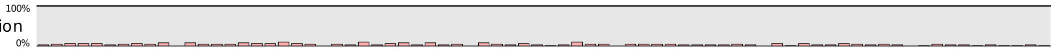

Sequence	4,560		4,580		4,600		4,620	
Stu-rnd-1_family-417_LINE_RTE-	----------	----------	----------	----------	----------	----------	----------	306
Sly-rnd1_family377-LINE/RTEBov	----------	----------	----------	----------	----------	----------	----------	135
Stu-rnd-1_family-489_LINE_RTE-	----------	----------	----------	----------	----------	----------	----------	87
Stu-rnd-1_family-607_LINE_RTE-	----------	----------	----------	----------	----------	----------	----------	671
Stu-rnd-1_family-645_LINE_RTE-	----------	----------	----------	----------	----------	----------	----------	437
Sly-rnd4_family81-LINE/RTEBovB	----------	----------	----------	----------	----------	----------	----------	861
Stu-rnd-6_family-408_LINE_RTE-	----------	----------	----------	----------	----------	----------	----------	393
Stu-rnd-1_family-351_LINE_RTE-	----------	----------	----------	----------	----------	----------	----------	609
Stu-rnd-1_family-382_LINE_RTE-	----------	----------	----------	----------	----------	----------	----------	347
Sly-rnd6_family746-LINE/RTEBov	----------	----------	----------	----------	----------	----------	----------	122
Stu-rnd-5_family-325_LINE_RTE-	----------	----------	----------	----------	----------	----------	----------	446
Sly-rnd4_family509-LINE/RTEBov	----------	----------	----------	----------	----------	----------	----------	743
Stu-rnd-1_family-513_LINE_RTE-	----------	----------	----------	----------	----------	----------	----------	65
Sly-rnd5_family1442-LINE/RTEBo	----------	----------	----------	----------	----------	----------	----------	119
BovB_Gl-RTE-Gehyra_lazelli	--------AA	GGCTCCACAC	CAATCCTGTT	CCTAATGAAC	A--------T	AGAAAATAAA	TCAATACTCG	3558
BovB_Gv-RTE-Gehyra_variegata	GTTGTTGAAA	GGTTGTTGAA	AGATGGTGTT	TGTTATGACC	GGTGTGTTTT	CATGCCTAGA	ACGCTGTTAG	3516
BovB_PMo-RTE-Python_molurus	----------	----------	----------	----------	----------	----------	----------	3288
BovB_Oa-RTE-Ovis_aries	----------	----------	----------	----------	----------	----------	----------	3259
BovB-RTE-Bos_taurus	----------	----------	----------	----------	----------	----------	----------	3847
BovB_Me-RTE-Macropus_eugenii	----------	----------	----------	----------	----------	----------	----------	2564
BovB_Ano-RTE-Amphibolurus_norr	----------	----------	----------	----------	----------	----------	----------	3305
BovB_Gd-RTE-Eremiascincus_doug	TGTGCACTCC	CA--GAACCC	TGCCCCCACT	CACCTGGTAG	AAGGCAGGCC	TGTGTCCTTC	AGGGAGCCAA	3089
BovB_Er-RTE-Eremiascincus_rich	CACCCGACTG	CAAGGAGATC	CAGTCGGGCA	ATCCTGCAGG	AAGTCAAGCC	TGATTGCTCC	CTGGAAGGAC	3646
BovB_Ca-RTE-Ctenotus_atlas	----------	----------	----------	----------	----------	----------	----------	3419
BovB_Es-RTE-Egernia_stokesii	----------	----------	----------	----------	----------	----------	----------	3292
BovB_Tr-RTE-Tiliqua_rugosa	----------	----------	----------	----------	----------	----------	----------	3260
BovB_ALi-RTE-Amblyomma_limbatu	TTTGACGCGG	ACTTTCTCAT	TGTTTCGCCT	CATGCTTCTA	T---------	----------	----------	3645
BovB_Hs-RTE-Hydrophis_spiralis	TTAATGACAG	TCTATAAAAC	AATGCTCACA	AATGGATTTA	TGCT------	----------	----------	1893
BovB_VA-RTE-Vipera_ammodytes	----------	----------	----------	----------	----------	----------	----------	3283
BovB_ACo-RTE-Agkistrodon_conto	----------	----------	----------	----------	----------	----------	----------	3282
BovB_Pg-RTE-Perameles_gunnii	AAG-------	----------	----------	----------	----------	----------	----------	1556
BovB_Io-RTE-Isoodon_obesulus	----------	----------	----------	----------	----------	----------	----------	1941
BovB_Bp-RTE-Burramys_parvus	----------	----------	----------	----------	----------	----------	----------	2067
BovB_Pb-RTE-Petaurus_breviceps	----------	----------	----------	----------	----------	----------	----------	2110
BovB_Ma-RTE-Macropus_antilopin	----------	----------	----------	----------	----------	----------	----------	3393
BovB_Af-RTE-Antechinus_flavipe	----------	----------	----------	----------	----------	----------	----------	2576
BovB_Ac-RTE-Anolis_carolinensi	----------	----------	----------	----------	----------	----------	----------	3180
BovB_BM-RTE-Bombyx_mori	----------	----------	----------	----------	----------	----------	----------	2863
BovB_Dr-RTE-Christinus	----------	----------	----------	----------	----------	----------	----------	3089
BovB_La-RTE-Loxodonta_africana	----------	----------	----------	----------	----------	----------	----------	3211
BovB_Pc-RTE-Procavia_capensis	----------	----------	----------	----------	----------	----------	----------	3221
BovB_Et-RTE-Christinus	----------	----------	----------	----------	----------	----------	----------	2573
BovB_Ta-RTE-Tachyglossus_acule	----------	----------	----------	----------	----------	----------	----------	3384
BovB_Plat-RTE-Ornithorhynchus	----------	----------	----------	----------	----------	----------	----------	3349
BovB_Ec-RTE-Equus_caballus	----------	----------	----------	----------	----------	----------	----------	3111
BovB_Cg-RTE-Christinus	----------	----------	----------	----------	----------	----------	----------	2776
BovB_Bh-RTE-Bothriocroton_hydr	----------	----------	----------	----------	----------	----------	----------	1699
BovB_Ml-RTE-Gehyra_variegata	----------	----------	----------	----------	----------	----------	----------	1449
Sly-rnd1_family284-LINE/RTEBov	----------	----------	----------	----------	----------	----------	----------	56
Sly-rnd5_family2019-LINE/RTEBo	----------	----------	----------	----------	----------	----------	----------	477
Sly-rnd3_family322-LINE/RTEBov	----------	----------	----------	----------	----------	----------	----------	568
Sly-rnd5_family1698-LINE/RTEBo	----------	----------	----------	----------	----------	----------	----------	231
Sly-rnd1_family795-LINE/RTEBov	----------	----------	----------	----------	----------	----------	----------	538
Stu-rnd-5_family-3162_LINE_RTE	----------	----------	----------	----------	----------	----------	----------	2547
Sly-rnd6_family1960-LINE/RTEBo	----------	----------	----------	----------	----------	----------	----------	1316
Stu-rnd-5_family-2493_LINE_RTE	----------	----------	----------	----------	----------	----------	----------	215
Sly-rnd5_family447-LINE/RTEBov	----------	----------	----------	----------	----------	----------	----------	564
Stu-rnd-6_family-2093_LINE_RTE	----------	----------	----------	----------	----------	----------	----------	105
Consensus	----------	----------	----------	----------	----------	----------	----------	

Conservation

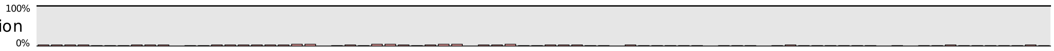

```
                                        4,640                         4,660                         4,680
Stu-rnd-1_family-417_LINE_RTE-      ---------- ---------- ---------- ---------- ---------- ---------- ---------- 306
Sly-rnd1_family377-LINE/RTEBov      ---------- ---------- ---------- ---------- ---------- ---------- ---------- 135
Stu-rnd-1_family-489_LINE_RTE-      ---------- ---------- ---------- ---------- ---------- ---------- ---------- 87
Stu-rnd-1_family-607_LINE_RTE-      ---------- ---------- ---------- ---------- ---------- ---------- ---------- 671
Stu-rnd-1_family-645_LINE_RTE-      ---------- ---------- ---------- ---------- ---------- ---------- ---------- 437
Sly-rnd4_family81-LINE/RTEBovB      ---------- ---------- ---------- ---------- ---------- ---------- ---------- 861
Stu-rnd-6_family-408_LINE_RTE-      ---------- ---------- ---------- ---------- ---------- ---------- ---------- 393
Stu-rnd-1_family-351_LINE_RTE-      ---------- ---------- ---------- ---------- ---------- ---------- ---------- 609
Stu-rnd-1_family-382_LINE_RTE-      ---------- ---------- ---------- ---------- ---------- ---------- ---------- 347
Sly-rnd6_family746-LINE/RTEBov      ---------- ---------- ---------- ---------- ---------- ---------- ---------- 122
Stu-rnd-5_family-325_LINE_RTE-      ---------- ---------- ---------- ---------- ---------- ---------- ---------- 446
Sly-rnd4_family509-LINE/RTEBov      ---------- ---------- ---------- ---------- ---------- ---------- ---------- 743
Stu-rnd-1_family-513_LINE_RTE-      ---------- ---------- ---------- ---------- ---------- ---------- ---------- 65
Sly-rnd5_family1442-LINE/RTEBo      ---------- ---------- ---------- ---------- ---------- ---------- ---------- 119
BovB_Gl-RTE-Gehyra_lazelli          TTGAAGCCAA ATAATATCAG TTCGAGTCAA AAGACCTAAT GCAGAAACT- ----TAAACC AAACTCCCCC 3623
BovB_Gv-RTE-Gehyra_variegata        CCTGTACCCA GTGGGAACAT AGCGGAG-AG GGATCCCAGG GCAAAAATTG TCCGTTATAC AAGGGACTCC 3585
BovB_PMo-RTE-Python_molurus         ---------- ---------- ---------- ---------- ---------- ---------- ---------- 3288
BovB_Oa-RTE-Ovis_aries              ---------- ---------- ---------- ---------- ---------- ---------- ---------- 3259
BovB-RTE-Bos_taurus                 ---------- ---------- ---------- ---------- ---------- ---------- ---------- 3847
BovB_Me-RTE-Macropus_eugenii        ---------- ---------- ---------- ---------- ---------- ---------- ---------- 2564
BovB_Ano-RTE-Amphibolurus_norr      ---------- ---------- ---------- ---------- ---------- ---------- ---------- 3305
BovB_Gd-RTE-Eremiascincus_doug      AGCCAACCTA GTAAAAGGGG CTGAACCCGG --TTCTTCC- ---------- ---------- ---------- 3126
BovB_Er-RTE-Eremiascincus_rich      AAATCCTGAA GCTGAAGCTC CAATACTTTG GCTACCTCCT GAGAAGAGAG GACTCTTTAG AAAAAACCCT 3716
BovB_Ca-RTE-Ctenotus_atlas          ---------- ---------- ---------- ---------- ---------- ---------- ---------- 3419
BovB_Es-RTE-Egernia_stokesii        ---------- ---------- ---------- ---------- ---------- ---------- ---------- 3292
BovB_Tr-RTE-Tiliqua_rugosa          ---------- ---------- ---------- ---------- ---------- ---------- ---------- 3260
BovB_ALi-RTE-Amblyomma_limbatu      ---------- ---------- ---------- ---------- ---------- ---------- ---------- 3645
BovB_Hs-RTE-Hydrophis_spiralis      ---------- ---------- ---------- ---------- ---------- ---------- ---------- 1893
BovB_VA-RTE-Vipera_ammodytes        ---------- ---------- ---------- ---------- ---------- ---------- ---------- 3283
BovB_ACo-RTE-Agkistrodon_conto      ---------- ---------- ---------- ---------- ---------- ---------- ---------- 3282
BovB_Pg-RTE-Perameles_gunnii        ---------- ---------- ---------- ---------- ---------- ---------- ---------- 1556
BovB_Io-RTE-Isoodon_obesulus        ---------- ---------- ---------- ---------- ---------- ---------- ---------- 1941
BovB_Bp-RTE-Burramys_parvus         ---------- ---------- ---------- ---------- ---------- ---------- ---------- 2067
BovB_Pb-RTE-Petaurus_breviceps      ---------- ---------- ---------- ---------- ---------- ---------- ---------- 2110
BovB_Ma-RTE-Macropus_antilopin      ---------- ---------- ---------- ---------- ---------- ---------- ---------- 3393
BovB_Af-RTE-Antechinus_flavipe      ---------- ---------- ---------- ---------- ---------- ---------- ---------- 2576
BovB_Ac-RTE-Anolis_carolinensi      ---------- ---------- ---------- ---------- ---------- ---------- ---------- 3180
BovB_BM-RTE-Bombyx_mori             ---------- ---------- ---------- ---------- ---------- ---------- ---------- 2863
BovB_Dr-RTE-Christinus              ---------- ---------- ---------- ---------- ---------- ---------- ---------- 3089
BovB_La-RTE-Loxodonta_africana      ---------- ---------- ---------- ---------- ---------- ---------- ---------- 3211
BovB_Pc-RTE-Procavia_capensis       ---------- ---------- ---------- ---------- ---------- ---------- ---------- 3221
BovB_Et-RTE-Christinus              ---------- ---------- ---------- ---------- ---------- ---------- ---------- 2573
BovB_Ta-RTE-Tachyglossus_acule      ---------- ---------- ---------- ---------- ---------- ---------- ---------- 3384
BovB_Plat-RTE-Ornithorhynchus       ---------- ---------- ---------- ---------- ---------- ---------- ---------- 3349
BovB_Ec-RTE-Equus_caballus          ---------- ---------- ---------- ---------- ---------- ---------- ---------- 3111
BovB_Cg-RTE-Christinus              ---------- ---------- ---------- ---------- ---------- ---------- ---------- 2776
BovB_Bh-RTE-Bothriocroton_hydr      ---------- ---------- ---------- ---------- ---------- ---------- ---------- 1699
BovB_Ml-RTE-Gehyra_variegata        ---------- ---------- ---------- ---------- ---------- ---------- ---------- 1449
Sly-rnd1_family284-LINE/RTEBov      ---------- ---------- ---------- ---------- ---------- ---------- ---------- 56
Sly-rnd5_family2019-LINE/RTEBo      ---------- ---------- ---------- ---------- ---------- ---------- ---------- 477
Sly-rnd3_family322-LINE/RTEBov      ---------- ---------- ---------- ---------- ---------- ---------- ---------- 568
Sly-rnd5_family1698-LINE/RTEBo      ---------- ---------- ---------- ---------- ---------- ---------- ---------- 231
Sly-rnd1_family795-LINE/RTEBov      ---------- ---------- ---------- ---------- ---------- ---------- ---------- 538
Stu-rnd-5_family-3162_LINE_RTE      ---------- ---------- ---------- ---------- ---------- ---------- ---------- 2547
Sly-rnd6_family1960-LINE/RTEBo      ---------- ---------- ---------- ---------- ---------- ---------- ---------- 1316
Stu-rnd-5_family-2493_LINE_RTE      ---------- ---------- ---------- ---------- ---------- ---------- ---------- 215
Sly-rnd5_family447-LINE/RTEBov      ---------- ---------- ---------- ---------- ---------- ---------- ---------- 564
Stu-rnd-6_family-2093_LINE_RTE      ---------- ---------- ---------- ---------- ---------- ---------- ---------- 105
Consensus                           ---------- ---------- ---------- ---------- ---------- ---------- ----------
```

Conservation

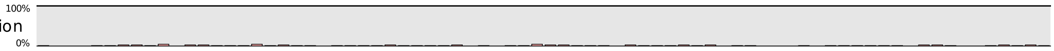

	4,700 · 4,720 · 4,740 · 4,760	
Stu-rnd-1_family-417_LINE_RTE-	---------- ---------- ---------- ---------- ---------- ---------- ----------	306
Sly-rnd1_family377-LINE/RTEBov	---------- ---------- ---------- ---------- ---------- ---------- ----------	135
Stu-rnd-1_family-489_LINE_RTE-	---------- ---------- ---------- ---------- ---------- ---------- ----------	87
Stu-rnd-1_family-607_LINE_RTE-	---------- ---------- ---------- ---------- ---------- ---------- ----------	671
Stu-rnd-1_family-645_LINE_RTE-	---------- ---------- ---------- ---------- ---------- ---------- ----------	437
Sly-rnd4_family81-LINE/RTEBovB	---------- ---------- ---------- ---------- ---------- ---------- ----------	861
Stu-rnd-6_family-408_LINE_RTE-	---------- ---------- ---------- ---------- ---------- ---------- ----------	393
Stu-rnd-1_family-351_LINE_RTE-	---------- ---------- ---------- ---------- ---------- ---------- ----------	609
Stu-rnd-1_family-382_LINE_RTE-	---------- ---------- ---------- ---------- ---------- ---------- ----------	347
Sly-rnd6_family746-LINE/RTEBov	---------- ---------- ---------- ---------- ---------- ---------- ----------	122
Stu-rnd-5_family-325_LINE_RTE-	---------- ---------- ---------- ---------- ---------- ---------- ----------	446
Sly-rnd4_family509-LINE/RTEBov	---------- ---------- ---------- ---------- ---------- ---------- ----------	743
Stu-rnd-1_family-513_LINE_RTE-	---------- ---------- ---------- ---------- ---------- ---------- ----------	65
Sly-rnd5_family1442-LINE/RTEBo	---------- ---------- ---------- ---------- ---------- ---------- ----------	119
BovB_Gl-RTE-Gehyra_lazelli	AAGAAGCCGA ACAGAACCAA AGCAACCG-- ---------- ---------- ---------- ----------	3651
BovB_Gv-RTE-Gehyra_variegata	TGCCTCCCGA GCAGCACGAA AGCCCATAAA AGCAACAA-- ---------- ---------- ----------	3623
BovB_PMo-RTE-Python_molurus	---------- ---------- ---------- ---------- ---------- ---------- ----------	3288
BovB_Oa-RTE-Ovis_aries	---------- ---------- ---------- ---------- ---------- ---------- ----------	3259
BovB-RTE-Bos_taurus	---------- ---------- ---------- ---------- ---------- ---------- ----------	3847
BovB_Me-RTE-Macropus_eugenii	---------- ---------- ---------- ---------- ---------- ---------- ----------	2564
BovB_Ano-RTE-Amphibolurus_norr	---------- ---------- ---------- ---------- ---------- ---------- ----------	3305
BovB_Gd-RTE-Eremiascincus_doug	---------- ---------- ---------- ---------- ---------- ---------- ----------	3126
BovB_Er-RTE-Eremiascincus_rich	CATGCTGGGA AAGATTGAAG GGAGAAGGAG AAGGGGGCGG CAGAGAAGGA CATCAATAGA GAATTTCTGT	3786
BovB_Ca-RTE-Ctenotus_atlas	---------- ---------- ---------- ---------- ---------- ---------- ----------	3419
BovB_Es-RTE-Egernia_stokesii	---------- ---------- ---------- ---------- ---------- ---------- ----------	3292
BovB_Tr-RTE-Tiliqua_rugosa	---------- ---------- ---------- ---------- ---------- ---------- ----------	3260
BovB_ALi-RTE-Amblyomma_limbatu	---------- ---------- ---------- ---------- ---------- ---------- ----------	3645
BovB_Hs-RTE-Hydrophis_spiralis	---------- ---------- ---------- ---------- ---------- ---------- ----------	1893
BovB_VA-RTE-Vipera_ammodytes	---------- ---------- ---------- ---------- ---------- ---------- ----------	3283
BovB_ACo-RTE-Agkistrodon_conto	---------- ---------- ---------- ---------- ---------- ---------- ----------	3282
BovB_Pg-RTE-Perameles_gunnii	---------- ---------- ---------- ---------- ---------- ---------- ----------	1556
BovB_Io-RTE-Isoodon_obesulus	---------- ---------- ---------- ---------- ---------- ---------- ----------	1941
BovB_Bp-RTE-Burramys_parvus	---------- ---------- ---------- ---------- ---------- ---------- ----------	2067
BovB_Pb-RTE-Petaurus_breviceps	---------- ---------- ---------- ---------- ---------- ---------- ----------	2110
BovB_Ma-RTE-Macropus_antilopin	---------- ---------- ---------- ---------- ---------- ---------- ----------	3393
BovB_Af-RTE-Antechinus_flavipe	---------- ---------- ---------- ---------- ---------- ---------- ----------	2576
BovB_Ac-RTE-Anolis_carolinensi	---------- ---------- ---------- ---------- ---------- ---------- ----------	3180
BovB_BM-RTE-Bombyx_mori	---------- ---------- ---------- ---------- ---------- ---------- ----------	2863
BovB_Dr-RTE-Christinus	---------- ---------- ---------- ---------- ---------- ---------- ----------	3089
BovB_La-RTE-Loxodonta_africana	---------- ---------- ---------- ---------- ---------- ---------- ----------	3211
BovB_Pc-RTE-Procavia_capensis	---------- ---------- ---------- ---------- ---------- ---------- ----------	3221
BovB_Et-RTE-Christinus	---------- ---------- ---------- ---------- ---------- ---------- ----------	2573
BovB_Ta-RTE-Tachyglossus_acule	---------- ---------- ---------- ---------- ---------- ---------- ----------	3384
BovB_Plat-RTE-Ornithorhynchus	---------- ---------- ---------- ---------- ---------- ---------- ----------	3349
BovB_Ec-RTE-Equus_caballus	---------- ---------- ---------- ---------- ---------- ---------- ----------	3111
BovB_Cg-RTE-Christinus	---------- ---------- ---------- ---------- ---------- ---------- ----------	2776
BovB_Bh-RTE-Bothriocroton_hydr	---------- ---------- ---------- ---------- ---------- ---------- ----------	1699
BovB_Ml-RTE-Gehyra_variegata	---------- ---------- ---------- ---------- ---------- ---------- ----------	1449
Sly-rnd1_family284-LINE/RTEBov	---------- ---------- ---------- ---------- ---------- ---------- ----------	56
Sly-rnd5_family2019-LINE/RTEBo	---------- ---------- ---------- ---------- ---------- ---------- ----------	477
Sly-rnd3_family322-LINE/RTEBov	---------- ---------- ---------- ---------- ---------- ---------- ----------	568
Sly-rnd5_family1698-LINE/RTEBo	---------- ---------- ---------- ---------- ---------- ---------- ----------	231
Sly-rnd1_family795-LINE/RTEBov	---------- ---------- ---------- ---------- ---------- ---------- ----------	538
Stu-rnd-5_family-3162_LINE_RTE	---------- ---------- ---------- ---------- ---------- ---------- ----------	2547
Sly-rnd6_family1960-LINE/RTEBo	---------- ---------- ---------- ---------- ---------- ---------- ----------	1316
Stu-rnd-5_family-2493_LINE_RTE	---------- ---------- ---------- ---------- ---------- ---------- ----------	215
Sly-rnd5_family447-LINE/RTEBov	---------- ---------- ---------- ---------- ---------- ---------- ----------	564
Stu-rnd-6_family-2093_LINE_RTE	---------- ---------- ---------- ---------- ---------- ---------- ----------	105
Consensus	---------- ---------- ---------- ---------- ---------- ---------- ----------	

Conservation

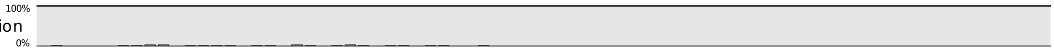

	4,780 4,800	
Stu-rnd-1_family-417_LINE_RTE-	---------- ---------- ---------- ---------- -----	306
Sly-rnd1_family377-LINE/RTEBov	---------- ---------- ---------- ---------- -----	135
Stu-rnd-1_family-489_LINE_RTE-	---------- ---------- ---------- ---------- -----	87
Stu-rnd-1_family-607_LINE_RTE-	---------- ---------- ---------- ---------- -----	671
Stu-rnd-1_family-645_LINE_RTE-	---------- ---------- ---------- ---------- -----	437
Sly-rnd4_family81-LINE/RTEBovB	---------- ---------- ---------- ---------- -----	861
Stu-rnd-6_family-408_LINE_RTE-	---------- ---------- ---------- ---------- -----	393
Stu-rnd-1_family-351_LINE_RTE-	---------- ---------- ---------- ---------- -----	609
Stu-rnd-1_family-382_LINE_RTE-	---------- ---------- ---------- ---------- -----	347
Sly-rnd6_family746-LINE/RTEBov	---------- ---------- ---------- ---------- -----	122
Stu-rnd-5_family-325_LINE_RTE-	---------- ---------- ---------- ---------- -----	446
Sly-rnd4_family509-LINE/RTEBov	---------- ---------- ---------- ---------- -----	743
Stu-rnd-1_family-513_LINE_RTE-	---------- ---------- ---------- ---------- -----	65
Sly-rnd5_family1442-LINE/RTEBo	---------- ---------- ---------- ---------- -----	119
BovB_Gl-RTE-Gehyra_lazelli	---------- ---------- ---------- ---------- -----	3651
BovB_Gv-RTE-Gehyra_variegata	---------- ---------- ---------- ---------- -----	3623
BovB_PMo-RTE-Python_molurus	---------- ---------- ---------- ---------- -----	3288
BovB_Oa-RTE-Ovis_aries	---------- ---------- ---------- ---------- -----	3259
BovB-RTE-Bos_taurus	---------- ---------- ---------- ---------- -----	3847
BovB_Me-RTE-Macropus_eugenii	---------- ---------- ---------- ---------- -----	2564
BovB_Ano-RTE-Amphibolurus_norr	---------- ---------- ---------- ---------- -----	3305
BovB_Gd-RTE-Eremiascincus_doug	---------- ---------- ---------- ---------- -----	3126
BovB_Er-RTE-Eremiascincus_rich	AGGTAAATTA TTAATGTGAA ATTCAGCTAA CTTACACGAG TCTAC	3831
BovB_Ca-RTE-Ctenotus_atlas	---------- ---------- ---------- ---------- -----	3419
BovB_Es-RTE-Egernia_stokesii	---------- ---------- ---------- ---------- -----	3292
BovB_Tr-RTE-Tiliqua_rugosa	---------- ---------- ---------- ---------- -----	3260
BovB_ALi-RTE-Amblyomma_limbatu	---------- ---------- ---------- ---------- -----	3645
BovB_Hs-RTE-Hydrophis_spiralis	---------- ---------- ---------- ---------- -----	1893
BovB_VA-RTE-Vipera_ammodytes	---------- ---------- ---------- ---------- -----	3283
BovB_ACo-RTE-Agkistrodon_conto	---------- ---------- ---------- ---------- -----	3282
BovB_Pg-RTE-Perameles_gunnii	---------- ---------- ---------- ---------- -----	1556
BovB_Io-RTE-Isoodon_obesulus	---------- ---------- ---------- ---------- -----	1941
BovB_Bp-RTE-Burramys_parvus	---------- ---------- ---------- ---------- -----	2067
BovB_Pb-RTE-Petaurus_breviceps	---------- ---------- ---------- ---------- -----	2110
BovB_Ma-RTE-Macropus_antilopin	---------- ---------- ---------- ---------- -----	3393
BovB_Af-RTE-Antechinus_flavipe	---------- ---------- ---------- ---------- -----	2576
BovB_Ac-RTE-Anolis_carolinensi	---------- ---------- ---------- ---------- -----	3180
BovB_BM-RTE-Bombyx_mori	---------- ---------- ---------- ---------- -----	2863
BovB_Dr-RTE-Christinus	---------- ---------- ---------- ---------- -----	3089
BovB_La-RTE-Loxodonta_africana	---------- ---------- ---------- ---------- -----	3211
BovB_Pc-RTE-Procavia_capensis	---------- ---------- ---------- ---------- -----	3221
BovB_Et-RTE-Christinus	---------- ---------- ---------- ---------- -----	2573
BovB_Ta-RTE-Tachyglossus_acule	---------- ---------- ---------- ---------- -----	3384
BovB_Plat-RTE-Ornithorhynchus	---------- ---------- ---------- ---------- -----	3349
BovB_Ec-RTE-Equus_caballus	---------- ---------- ---------- ---------- -----	3111
BovB_Cg-RTE-Christinus	---------- ---------- ---------- ---------- -----	2776
BovB_Bh-RTE-Bothriocroton_hydr	---------- ---------- ---------- ---------- -----	1699
BovB_Ml-RTE-Gehyra_variegata	---------- ---------- ---------- ---------- -----	1449
Sly-rnd1_family284-LINE/RTEBov	---------- ---------- ---------- ---------- -----	56
Sly-rnd5_family2019-LINE/RTEBo	---------- ---------- ---------- ---------- -----	477
Sly-rnd3_family322-LINE/RTEBov	---------- ---------- ---------- ---------- -----	568
Sly-rnd5_family1698-LINE/RTEBo	---------- ---------- ---------- ---------- -----	231
Sly-rnd1_family795-LINE/RTEBov	---------- ---------- ---------- ---------- -----	538
Stu-rnd-5_family-3162_LINE_RTE	---------- ---------- ---------- ---------- -----	2547
Sly-rnd6_family1960-LINE/RTEBo	---------- ---------- ---------- ---------- -----	1316
Stu-rnd-5_family-2493_LINE_RTE	---------- ---------- ---------- ---------- -----	215
Sly-rnd5_family447-LINE/RTEBov	---------- ---------- ---------- ---------- -----	564
Stu-rnd-6_family-2093_LINE_RTE	---------- ---------- ---------- ---------- -----	105
Consensus	---------- ---------- ---------- ---------- -----	
Conservation (100% / 0%)		
