# Supplementary material for: A Deluge of Complex Repeats: The Solanum Genome
Source: PLoS One. 2015 Aug 4;10(8):e0133962. doi: 10.1371/journal.pone.0133962 (PMC4524691; doi:10.1371/journal.pone.0133962)
Supplement: S6 Fig — The consensus sequence of LTR/ERV1 identified in this study was matched with known consensus sequences of LTR/ERV1 and all the sequences matching with LTR/ERV1 were used for MSA. (PDF) [file pone.0133962.s006.pdf]

	1–10	11–20	21–30	31–40	41–50	51–60	61–70	
		20		40		60		
Dipodomys_ordii	----------	----------	----------	----------	----------	----------	----------	-
Laurasiatheria	----------	----------	----------	----------	----------	----------	----------	-
Eutheria	----------	----------	----------	----------	----------	----------	----------	-
Tarsius_syrichta	----------	----------	----------	----------	----------	----------	----------	-
Muridae	----------	----------	----------	----------	----------	----------	----------	-
Gasterosteus_aculeatus	----------	----------	----------	----------	----------	----------	----------	-
Felis_silvestris	----------	----------	----------	----------	----------	----------	----------	-
Rattus	----------	----------	----------	----------	----------	----------	----------	-
Papio_hamadryas	----------	----------	----------	----------	----------	----------	----------	-
Pan_troglodytes	----------	----------	----------	----------	----------	----------	----------	-
Ailuropoda_melanoleuca	----------	----------	----------	----------	----------	----------	----------	-
Cavia_porcellus	----------	----------	----------	----------	----------	----------	----------	-
Ictidomys_tridecemlineatus	----------	----------	----------	----------	----------	----------	----------	-
Canis	----------	----------	----------	----------	----------	----------	----------	-
Felis_catus	----------	----------	----------	----------	----------	----------	----------	-
Ochotona_princeps	----------	----------	----------	----------	----------	----------	----------	-
Cercopithecidae	----------	----------	----------	----------	----------	----------	----------	-
Carnivora	----------	----------	----------	----------	----------	----------	----------	-
Mus	----------	----------	----------	----------	----------	----------	----------	-
Mus_musculus	----------	----------	----------	----------	----------	----------	----------	-
Rattus_norvegicus	----------	----------	----------	----------	----------	----------	----------	-
Tursiops_truncatus	----------	----------	----------	----------	----------	----------	----------	-
Procavia_capensis	----------	----------	----------	----------	----------	----------	----------	-
Primates	----------	----------	----------	----------	----------	----------	----------	-
Echinops_telfairi	----------	----------	----------	----------	----------	----------	----------	-
Sorex_araneus	----------	----------	----------	----------	----------	----------	----------	-
Canidae	----------	----------	----------	----------	----------	----------	----------	-
Otolemur_garnettii	----------	----------	----------	----------	----------	----------	----------	-
Gallus	----------	----------	----------	----------	----------	----------	----------	-
Callithrix_jacchus	----------	----------	----------	----------	----------	----------	----------	-
Crocodylus_porosus	----------	----------	----------	----------	----------	----------	----------	-
Erinaceus_europaeus	----------	----------	----------	----------	----------	----------	----------	-
rnd-6_family-7426-LTR-ERV1	----------	----------	----------	----------	----------	----------	----------	-
Esox_lucius	----------	----------	----------	----------	----------	----------	----------	-
Salmo_salar	----------	----------	----------	----------	----------	----------	----------	-
Didelphidae	----------	----------	----------	----------	----------	----------	----------	-
Latimeria_chalumnae	----------	----------	----------	----------	----------	----------	----------	-
Monodelphis_domestica	----------	----------	----------	----------	----------	----------	----------	-
Macropus_eugenii	----------	----------	----------	----------	----------	----------	----------	-
Alligator_mississippiensis	----------	----------	----------	----------	----------	----------	----------	-
Chrysemys_picta_bellii	----------	----------	----------	----------	----------	----------	----------	-
Danio_rerio	----------	----------	----------	----------	----------	----------	----------	-
Taeniopygia	----------	----------	----------	----------	----------	----------	----------	-
Dasypus_novemcinctus	----------	----------	----------	----------	----------	----------	----------	-
LTR_XT	----------	----------	----------	----------	----------	----------	----------	-
Haplorrhini	TTTCTGGGGG	CTCGTCCGGG	ATTGGAGATG	GCAGGTTTCT	GTCTCCTTTG	CCTGTGGGCT	AGAGCCCCAG	70
Catarrhini	----------	----------	----------	----------	----------	----------	----------	-
Anolis_carolinensis	----------	----------	----------	----------	----------	----------	----------	-
Murine_leukemia_virus	----------	----------	----------	----------	----------	----------	----------	-
Phascolarctos_cinereus	----------	----------	----------	----------	----------	----------	----------	-
Estrildidae	----------	----------	----------	----------	----------	----------	----------	-
Xenopus_Silurana_tropicalis	----------	----------	----------	----------	----------	----------	----------	-
I_XT	----------	----------	----------	----------	----------	----------	----------	-
Takifugu_rubripes	----------	----------	----------	----------	----------	----------	----------	-
Bos_taurus	----------	----------	----------	----------	----------	----------	----------	-
Homo_sapiens	----------	----------	----------	----------	----------	----------	----------	-
Microcebus_murinus	----------	----------	----------	----------	----------	----------	----------	-
Myotis_lucifugus	----------	----------	----------	----------	----------	----------	----------	-
Equus_caballus	----------	----------	----------	----------	----------	----------	----------	-
Tupaia_belangeri	----------	----------	----------	----------	----------	----------	----------	-
Loxodonta_africana	----------	----------	----------	----------	----------	----------	----------	-
Murinae	----------	----------	----------	----------	----------	----------	----------	-
Canis_lupus_familiaris	----------	----------	----------	----------	----------	----------	----------	-
Sus_scrofa	----------	----------	----------	----------	----------	----------	----------	-
Choloepus_hoffmanni	----------	----------	----------	----------	----------	----------	----------	-
Vicugna_pacos	----------	----------	----------	----------	----------	----------	----------	-
Gavialis_gangeticus	----------	----------	----------	----------	----------	----------	----------	-
Mammalia	----------	----------	----------	----------	----------	----------	----------	-
Passeroidea	----------	----------	----------	----------	----------	----------	----------	-
Takifugu	----------	----------	----------	----------	----------	----------	----------	-
Pteropus_vampyrus	----------	----------	----------	----------	----------	----------	----------	-
Ornithorhynchus	----------	----------	----------	----------	----------	----------	----------	-
Oryctolagus_cuniculus	----------	----------	----------	----------	----------	----------	----------	-
Gallus_gallus	----------	----------	----------	----------	----------	----------	----------	-
Hominidae	----------	----------	----------	----------	----------	----------	----------	-
Metatheria	----------	----------	----------	----------	----------	----------	----------	-
Rodentia	----------	----------	----------	----------	----------	----------	----------	-
Monodelphis	----------	----------	----------	----------	----------	----------	----------	-
Consensus	----------	----------	----------	----------	----------	----------	----------	

Conservation (100% – 0%)

	80 100 120 140	
Dipodomys_ordii	---------- ---------- ---------- ---------- ---------- ---------- ----------	-
Laurasiatheria	---------- ---------- ---------- ---------- ---------- ---------- ----------	-
Eutheria	---------- ---------- ---------- ---------- ---------- ---------- ----------	-
Tarsius_syrichta	---------- ---------- ---------- ---------- ---------- ---------- ----------	-
Muridae	---------- ---------- ---------- ---------- ---------- ---------- ----------	-
Gasterosteus_aculeatus	---------- ---------- ---------- ---------- ---------- ---------- ----------	-
Felis_silvestris	---------- ---------- ---------- ---------- ---------- ---------- ----------	-
Rattus	---------- ---------- ---------- ---------- ---------- ---------- ----------	-
Papio_hamadryas	---------- ---------- ---------- ---------- ---------- ---------- ----------	-
Pan_troglodytes	---------- ---------- ---------- ---------- ---------- ---------- ----------	-
Ailuropoda_melanoleuca	---------- ---------- ---------- ---------- ---------- ---------- ----------	-
Cavia_porcellus	---------- ---------- ---------- ---------- ---------- ---------- ----------	-
Ictidomys_tridecemlineatus	---------- ---------- ---------- ---------- ---------- ---------- ----------	-
Canis	---------- ---------- ---------- ---------- ---------- ---------- ----------	-
Felis_catus	---------- ---------- ---------- ---------- ---------- ---------- ----------	-
Ochotona_princeps	---------- ---------- ---------- ---------- ---------- ---------- ----------	-
Cercopithecidae	---------- ---------- ---------- ---------- ---------- ---------- ----------	-
Carnivora	---------- ---------- ---------- ---------- ---------- ---------- ----------	-
Mus	---------- ---------- ---------- ---------- ---------- ---------- ----------	-
Mus_musculus	---------- ---------- ---------- ---------- ---------- ---------- ----------	-
Rattus_norvegicus	---------- ---------- ---------- ---------- ---------- ---------- ----------	-
Tursiops_truncatus	---------- ---------- ---------- ---------- ---------- ---------- ----------	-
Procavia_capensis	---------- ---------- ---------- ---------- ---------- ---------- ----------	-
Primates	---------- ---------- ---------- ---------- ---------- ---------- ----------	-
Echinops_telfairi	---------- ---------- ---------- ---------- ---------- ---------- ----------	-
Sorex_araneus	---------- ---------- ---------- ---------- ---------- ---------- ----------	-
Canidae	---------- ---------- ---------- ---------- ---------- ---------- ----------	-
Otolemur_garnettii	---------- ---------- ---------- ---------- ---------- ---------- ----------	-
Gallus	---------- ---------- ---------- ---------- ---------- ---------- ----------	-
Callithrix_jacchus	---------- ---------- ---------- ---------- ---------- ---------- ----------	-
Crocodylus_porosus	---------- ---------- ---------- ---------- ---------- ---------- ----------	-
Erinaceus_europaeus	---------- ---------- ---------- ---------- ---------- ---------- ----------	-
rnd-6_family-7426-LTR-ERV1	---------- ---------- ---------- ---------- ---------- ---------- ----------	-
Esox_lucius	---------- ---------- ---------- ---------- ---------- ---------- ----------	-
Salmo_salar	---------- ---------- ---------- ---------- ---------- ---------- ----------	-
Didelphidae	---------- ---------- ---------- ---------- ---------- ---------- ----------	-
Latimeria_chalumnae	---------- ---------- ---------- ---------- ---------- ---------- ----------	-
Monodelphis_domestica	---------- ---------- ---------- ---------- ---------- ---------- ----------	-
Macropus_eugenii	---------- ---------- ---------- ---------- ---------- ---------- ----------	-
Alligator_mississippiensis	---------- ---------- ---------- ---------- ---------- ---------- ----------	-
Chrysemys_picta_bellii	---------- ---------- ---------- ---------- ---------- ---------- ----------	-
Danio_rerio	---------- ---------- ---------- ---------- ---------- ---------- ----------	-
Taeniopygia	---------- ---------- ---------- ---------- ---------- ---------- ----------	-
Dasypus_novemcinctus	---------- ---------- ---------- ---------- ---------- ---------- ----------	-
LTR_XT	---------- ---------- ---------- ---------- ---------- ---------- ----------	-
Haplorrhini	GACGCGGGAG ATTTGGGACC CTTGGCGCCA CTGGGTAAGA CTTAGCCCAG AAGGAGAACG GCTCTCCCGT	140
Catarrhini	---------- ---------- ---------- ---------- ---------- ---------- ----------	-
Anolis_carolinensis	---------- ---------- ---------- ---------- ---------- ---------- ----------	-
Murine_leukemia_virus	---------- ---------- ---------- ---------- ---------- ---------- ----------	-
Phascolarctos_cinereus	---------- ---------- ---------- ---------- ---------- ---------- ----------	-
Estrildidae	---------- ---------- ---------- ---------- ---------- ---------- ----------	-
Xenopus_Silurana_tropicalis	---------- ---------- ---------- ---------- ---------- ---------- ----------	-
I_XT	---------- ---------- ---------- ---------- ---------- ---------- ----------	-
Takifugu_rubripes	---------- ---------- ---------- ---------- ---------- ---------- ----------	-
Bos_taurus	---------- ---------- ---------- ---------- ---------- ---------- ----------	-
Homo_sapiens	---------- ---------- ---------- ---------- ---------- ---------- ----------	-
Microcebus_murinus	---------- ---------- ---------- ---------- ---------- ---------- ----------	-
Myotis_lucifugus	---------- ---------- ---------- ---------- ---------- ---------- ----------	-
Equus_caballus	---------- ---------- ---------- ---------- ---------- ---------- ----------	-
Tupaia_belangeri	---------- ---------- ---------- ---------- ---------- ---------- ----------	-
Loxodonta_africana	---------- ---------- ---------- ---------- ---------- ---------- ----------	-
Murinae	---------- ---------- ---------- ---------- ---------- ---------- ----------	-
Canis_lupus_familiaris	---------- ---------- ---------- ---------- ---------- ---------- ----------	-
Sus_scrofa	---------- ---------- ---------- ---------- ---------- ---------- ----------	-
Choloepus_hoffmanni	---------- ---------- ---------- ---------- ---------- ---------- ----------	-
Vicugna_pacos	---------- ---------- ---------- ---------- ---------- ---------- ----------	-
Gavialis_gangeticus	---------- ---------- ---------- ---------- ---------- ---------- ----------	-
Mammalia	---------- ---------- ---------- ---------- ---------- ---------- ----------	-
Passeroidea	---------- ---------- ---------- ---------- ---------- ---------- ----------	-
Takifugu	---------- ---------- ---------- ---------- ---------- ---------- ----------	-
Pteropus_vampyrus	---------- ---------- ---------- ---------- ---------- ---------- ----------	-
Ornithorhynchus	---------- ---------- ---------- ---------- ---------- ---------- ----------	-
Oryctolagus_cuniculus	---------- ---------- ---------- ---------- ---------- ---------- ----------	-
Gallus_gallus	---------- ---------- ---------- ---------- ---------- ---------- ----------	-
Hominidae	---------- ---------- ---------- ---------- ---------- ---------- ----------	-
Metatheria	---------- ---------- ---------- ---------- ---------- ---------- ----------	-
Rodentia	---------- ---------- ---------- ---------- ---------- ---------- ----------	-
Monodelphis	---------- ---------- ---------- ---------- ---------- ---------- ----------	-
Consensus	---------- ---------- ---------- ---------- ---------- ---------- ----------	
Conservation	100% 0%	

	160		180		200		
Dipodomys_ordii	---------- ----------	----------	----------	----------	----------	----------	-
Laurasiatheria	---------- ----------	----------	----------	----------	----------	----------	-
Eutheria	---------- ----------	----------	----------	----------	----------	----------	-
Tarsius_syrichta	---------- ----------	----------	----------	----------	----------	----------	-
Muridae	---------- ----------	----------	----------	----------	----------	----------	-
Gasterosteus_aculeatus	---------- ----------	----------	----------	----------	----------	----------	-
Felis_silvestris	---------- ----------	----------	----------	----------	----------	----------	-
Rattus	---------- ----------	----------	----------	----------	----------	----------	-
Papio_hamadryas	---------- ----------	----------	----------	----------	----------	----------	-
Pan_troglodytes	---------- ----------	----------	----------	----------	----------	----------	-
Ailuropoda_melanoleuca	---------- ----------	----------	----------	----------	----------	----------	-
Cavia_porcellus	---------- ----------	----------	----------	----------	----------	----------	-
Ictidomys_tridecemlineatus	---------- ----------	----------	----------	----------	----------	----------	-
Canis	---------- ----------	----------	----------	----------	----------	----------	-
Felis_catus	---------- ----------	----------	----------	----------	----------	----------	-
Ochotona_princeps	---------- ----------	----------	----------	----------	----------	----------	-
Cercopithecidae	---------- ----------	----------	----------	----------	----------	----------	-
Carnivora	---------- ----------	----------	----------	----------	----------	----------	-
Mus	---------- ----------	----------	----------	----------	----------	----------	-
Mus_musculus	---------- ----------	----------	----------	----------	----------	----------	-
Rattus_norvegicus	---------- ----------	----------	----------	----------	----------	----------	-
Tursiops_truncatus	---------- ----------	----------	----------	----------	----------	----------	-
Procavia_capensis	---------- ----------	----------	----------	----------	----------	----------	-
Primates	---------- ----------	----------	----------	----------	----------	----------	-
Echinops_telfairi	---------- ----------	----------	----------	----------	----------	----------	-
Sorex_araneus	---------- ----------	----------	----------	----------	----------	----------	-
Canidae	---------- ----------	----------	----------	----------	----------	----------	-
Otolemur_garnettii	---------- ----------	----------	----------	----------	----------	----------	-
Gallus	---------- ----------	----------	----------	----------	----------	----------	-
Callithrix_jacchus	---------- ----------	----------	----------	----------	----------	----------	-
Crocodylus_porosus	---------- ----------	----------	----------	----------	----------	----------	-
Erinaceus_europaeus	---------- ----------	----------	----------	----------	----------	----------	-
rnd-6_family-7426-LTR-ERV1	---------- ----------	----------	----------	----------	----------	----------	-
Esox_lucius	---------- ----------	----------	----------	----------	----------	----------	-
Salmo_salar	---------- ----------	----------	----------	----------	----------	----------	-
Didelphidae	---------- ----------	----------	----------	----------	----------	----------	-
Latimeria_chalumnae	---------- ----------	----------	----------	----------	----------	----------	-
Monodelphis_domestica	---------- ----------	----------	----------	----------	----------	----------	-
Macropus_eugenii	---------- ----------	----------	----------	----------	----------	----------	-
Alligator_mississippiensis	---------- ----------	----------	----------	----------	----------	----------	-
Chrysemys_picta_bellii	---------- ----------	----------	----------	----------	----------	----------	-
Danio_rerio	---------- ----------	----------	----------	----------	----------	----------	-
Taeniopygia	---------- ----------	----------	----------	----------	----------	----------	-
Dasypus_novemcinctus	---------- ----------	----------	----------	----------	----------	----------	-
LTR_XT	---------- ----------	----------	----------	----------	----------	----------	-
Haplorrhini	GTCTTGGAGC CTTCCCCTGA	CAGCGCAAAC	GGAACCGACT	CAGGAGTTGC	AGGACAGTCA	CAGGAGCAGC	210
Catarrhini	---------- ----------	----------	----------	----------	----------	----------	-
Anolis_carolinensis	---------- ----------	----------	----------	----------	----------	----------	-
Murine_leukemia_virus	---------- ----------	----------	----------	----------	----------	----------	-
Phascolarctos_cinereus	---------- ----------	----------	----------	----------	----------	----------	-
Estrildidae	---------- ----------	----------	----------	----------	----------	----------	-
Xenopus_Silurana_tropicalis	---------- ----------	----------	----------	----------	----------	----------	-
I_XT	---------- ----------	----------	----------	----------	----------	----------	-
Takifugu_rubripes	---------- ----------	----------	----------	----------	----------	----------	-
Bos_taurus	---------- ----------	----------	----------	----------	----------	----------	-
Homo_sapiens	---------- ----------	----------	----------	----------	----------	----------	-
Microcebus_murinus	---------- ----------	----------	----------	----------	----------	----------	-
Myotis_lucifugus	---------- ----------	----------	----------	----------	----------	----------	-
Equus_caballus	---------- ----------	----------	----------	----------	----------	----------	-
Tupaia_belangeri	---------- ----------	----------	----------	----------	----------	----------	-
Loxodonta_africana	---------- ----------	----------	----------	----------	----------	----------	-
Murinae	---------- ----------	----------	----------	----------	----------	----------	-
Canis_lupus_familiaris	---------- ----------	----------	----------	----------	----------	----------	-
Sus_scrofa	---------- ----------	----------	----------	----------	----------	----------	-
Choloepus_hoffmanni	---------- ----------	----------	----------	----------	----------	----------	-
Vicugna_pacos	---------- ----------	----------	----------	----------	----------	----------	-
Gavialis_gangeticus	---------- ----------	----------	----------	----------	----------	----------	-
Mammalia	---------- ----------	----------	----------	----------	----------	----------	-
Passeroidea	---------- ----------	----------	----------	----------	----------	----------	-
Takifugu	---------- ----------	----------	----------	----------	----------	----------	-
Pteropus_vampyrus	---------- ----------	----------	----------	----------	----------	----------	-
Ornithorhynchus	---------- ----------	----------	----------	----------	----------	----------	-
Oryctolagus_cuniculus	---------- ----------	----------	----------	----------	----------	----------	-
Gallus_gallus	---------- ----------	----------	----------	----------	----------	----------	-
Hominidae	---------- ----------	----------	----------	----------	----------	----------	-
Metatheria	---------- ----------	----------	----------	----------	----------	----------	-
Rodentia	---------- ----------	----------	----------	----------	----------	----------	-
Monodelphis	---------- ----------	----------	----------	----------	----------	----------	-
Consensus	---------- ----------	----------	----------	----------	----------	----------	

Conservation (100% – 0%)

	220 240 260 280	
Dipodomys_ordii	---------- ---------- ---------- ---------- ---------- ---------- ----------	-
Laurasiatheria	---------- ---------- ---------- ---------- ---------- ---------- ----------	-
Eutheria	---------- ---------- ---------- ---------- ---------- ---------- ----------	-
Tarsius_syrichta	---------- ---------- ---------- ---------- ---------- ---------- ----------	-
Muridae	---------- ---------- ---------- ---------- ---------- ---------- ----------	-
Gasterosteus_aculeatus	---------- ---------- ---------- ---------- ---------- ---------- ----------	-
Felis_silvestris	---------- ---------- ---------- ---------- ---------- ---------- ----------	-
Rattus	---------- ---------- ---------- ---------- ---------- ---------- ----------	-
Papio_hamadryas	---------- ---------- ---------- ---------- ---------- ---------- ----------	-
Pan_troglodytes	---------- ---------- ---------- ---------- ---------- ---------- ----------	-
Ailuropoda_melanoleuca	---------- ---------- ---------- ---------- ---------- ---------- ----------	-
Cavia_porcellus	---------- ---------- ---------- ---------- ---------- ---------- ----------	-
Ictidomys_tridecemlineatus	---------- ---------- ---------- ---------- ---------- ---------- ----------	-
Canis	---------- ---------- ---------- ---------- ---------- ---------- ----------	-
Felis_catus	---------- ---------- ---------- ---------- ---------- ---------- ----------	-
Ochotona_princeps	---------- ---------- ---------- ---------- ---------- ---------- ----------	-
Cercopithecidae	---------- ---------- ---------- ---------- ---------- ---------- ----------	-
Carnivora	---------- ---------- ---------- ---------- ---------- ---------- ----------	-
Mus	---------- ---------- ---------- ---------- ---------- ---------- ----------	-
Mus_musculus	---------- ---------- ---------- ---------- ---------- ---------- ----------	-
Rattus_norvegicus	---------- ---------- ---------- ---------- ---------- ---------- ----------	-
Tursiops_truncatus	---------- ---------- ---------- ---------- ---------- ---------- ----------	-
Procavia_capensis	---------- ---------- ---------- ---------- ---------- ---------- ----------	-
Primates	---------- ---------- ---------- ---------- ---------- ---------- ----------	-
Echinops_telfairi	---------- ---------- ---------- ---------- ---------- ---------- ----------	-
Sorex_araneus	---------- ---------- ---------- ---------- ---------- ---------- ----------	-
Canidae	---------- ---------- ---------- ---------- ---------- ---------- ----------	-
Otolemur_garnettii	---------- ---------- ---------- ---------- ---------- ---------- ----------	-
Gallus	---------- ---------- ---------- ---------- ---------- ---------- ----------	-
Callithrix_jacchus	---------- ---------- ---------- ---------- ---------- ---------- ----------	-
Crocodylus_porosus	---------- ---------- ---------- ---------- ---------- ---------- ----------	-
Erinaceus_europaeus	---------- ---------- ---------- ---------- ---------- ---------- ----------	-
rnd-6_family-7426-LTR-ERV1	---------- ---------- ---------- ---------- ---------- ---------- ----------	-
Esox_lucius	---------- ---------- ---------- ---------- ---------- ---------- ----------	-
Salmo_salar	---------- ---------- ---------- ---------- ---------- ---------- ----------	-
Didelphidae	---------- ---------- ---------- ---------- ---------- ---------- ----------	-
Latimeria_chalumnae	---------- ---------- ---------- ---------- ---------- ---------- ----------	-
Monodelphis_domestica	---------- ---------- ---------- ---------- ---------- ---------- ----------	-
Macropus_eugenii	---------- ---------- ---------- ---------- ---------- ---------- ----------	-
Alligator_mississippiensis	---------- ---------- ---------- ---------- ---------- ---------- ----------	-
Chrysemys_picta_bellii	---------- ---------- ---------- ---------- ---------- ---------- ----------	-
Danio_rerio	---------- ---------- ---------- ---------- ---------- ---------- ----------	-
Taeniopygia	---------- ---------- ---------- ---------- ---------- ---------- ----------	-
Dasypus_novemcinctus	---------- ---------- ---------- ---------- ---------- ---------- ----------	-
LTR_XT	---------- ---------- ---------- ---------- ---------- ---------- ----------	-
Haplorrhini	GCGCAGGCAG ACTACTGAAC CACGGTAAGG TTGGGCCCTG GGAAAGCCCG TCCCATAAGG ACAGAAGGGG	280
Catarrhini	---------- ---------- ---------- ---------- ---------- ---------- ---------T	1
Anolis_carolinensis	---------- ---------- ---------- ---------- ---------- ---------- ----------	-
Murine_leukemia_virus	---------- ---------- ---------- ---------- ---------- ---------- ----------	-
Phascolarctos_cinereus	---------- ---------- ---------- ---------- ---------- ---------- ----------	-
Estrildidae	---------- ---------- ---------- ---------- ---------- ---------- ----------	-
Xenopus_Silurana_tropicalis	---------- ---------- ---------- ---------- ---------- ---------- ----------	-
I_XT	---------- ---------- ---------- ---------- ---------- ---------- ----------	-
Takifugu_rubripes	---------- ---------- ---------- ---------- ---------- ---------- ----------	-
Bos_taurus	---------- ---------- ---------- ---------- ---------- ---------- ----------	-
Homo_sapiens	---------- ---------- ---------- ---------- ---------- ---------- ----------	-
Microcebus_murinus	---------- ---------- ---------- ---------- ---------- ---------- ----------	-
Myotis_lucifugus	---------- ---------- ---------- ---------- ---------- ---------- ----------	-
Equus_caballus	---------- ---------- ---------- ---------- ---------- ---------- ----------	-
Tupaia_belangeri	---------- ---------- ---------- ---------- ---------- ---------- ----------	-
Loxodonta_africana	---------- ---------- ---------- ---------- ---------- ---------- ----------	-
Murinae	---------- ---------- ---------- ---------- ---------- ---------- ----------	-
Canis_lupus_familiaris	---------- ---------- ---------- ---------- ---------- ---------- ----------	-
Sus_scrofa	---------- ---------- ---------- ---------- ---------- ---------- ----------	-
Choloepus_hoffmanni	---------- ---------- ---------- ---------- ---------- ---------- ----------	-
Vicugna_pacos	---------- ---------- ---------- ---------- ---------- ---------- ----------	-
Gavialis_gangeticus	---------- ---------- ---------- ---------- ---------- ---------- ----------	-
Mammalia	---------- ---------- ---------- ---------- ---------- ---------- ----------	-
Passeroidea	---------- ---------- ---------- ---------- ---------- ---------- ----------	-
Takifugu	---------- ---------- ---------- ---------- ---------- ---------- ----------	-
Pteropus_vampyrus	---------- ---------- ---------- ---------- ---------- ---------- ----------	-
Ornithorhynchus	---------- ---------- ---------- ---------- ---------- ---------- ----------	-
Oryctolagus_cuniculus	---------- ---------- ---------- ---------- ---------- ---------- ----------	-
Gallus_gallus	---------- ---------- ---------- ---------- ---------- ---------- ----------	-
Hominidae	---------- ---------- ---------- ---------- ---------- ---------- ----------	-
Metatheria	---------- ---------- ---------- ---------- ---------- ---------- ----------	-
Rodentia	---------- ---------- ---------- ---------- ---------- ---------- ----------	-
Monodelphis	---------- ---------- ---------- ---------- ---------- ---------- ----------	-
Consensus	---------- ---------- ---------- ---------- ---------- ---------- ----------	
Conservation	100% 0%	

		300		320		340		
Dipodomys_ordii	----------	----------	----------	----------	----------	----------	----------	-
Laurasiatheria	----------	----------	----------	----------	----------	----------	----------	-
Eutheria	----------	----------	----------	----------	----------	----------	----------	-
Tarsius_syrichta	----------	----------	----------	----------	----------	----------	----------	-
Muridae	----------	----------	----------	----------	----------	----------	----------	-
Gasterosteus_aculeatus	----------	----------	----------	----------	----------	----------	----------	-
Felis_silvestris	----------	----------	----------	----------	----------	----------	----------	-
Rattus	----------	----------	----------	----------	----------	----------	----------	-
Papio_hamadryas	----------	----------	----------	----------	----------	----------	----------	-
Pan_troglodytes	----------	----------	----------	----------	----------	----------	----------	-
Ailuropoda_melanoleuca	----------	----------	----------	----------	----------	----------	----------	-
Cavia_porcellus	----------	----------	----------	----------	----------	----------	----------	-
Ictidomys_tridecemlineatus	----------	----------	----------	----------	----------	----------	----------	-
Canis	----------	----------	----------	----------	----------	----------	----------	-
Felis_catus	----------	----------	----------	----------	----------	----------	----------	-
Ochotona_princeps	----------	----------	----------	----------	----------	----------	----------	-
Cercopithecidae	----------	----------	----------	----------	----------	----------	----------	-
Carnivora	----------	----------	----------	----------	----------	----------	----------	-
Mus	----------	----------	----------	----------	----------	----------	----------	-
Mus_musculus	----------	----------	----------	----------	----------	----------	----------	-
Rattus_norvegicus	----------	----------	----------	----------	----------	----------	----------	-
Tursiops_truncatus	----------	----------	----------	----------	----------	----------	----------	-
Procavia_capensis	----------	----------	----------	----------	----------	----------	----------	-
Primates	----------	----------	----------	----------	----------	----------	----------	-
Echinops_telfairi	----------	----------	----------	----------	----------	----------	----------	-
Sorex_araneus	----------	----------	----------	----------	----------	----------	----------	-
Canidae	----------	----------	----------	----------	----------	----------	----------	-
Otolemur_garnettii	----------	----------	----------	----------	----------	----------	----------	-
Gallus	----------	----------	----------	----------	----------	----------	----------	-
Callithrix_jacchus	----------	----------	----------	----------	----------	----------	----------	-
Crocodylus_porosus	----------	----------	----------	----------	----------	----------	----------	-
Erinaceus_europaeus	----------	----------	----------	----------	----------	----------	----------	-
rnd-6_family-7426-LTR-ERV1	----------	----------	----------	----------	----------	----------	----------	-
Esox_lucius	----------	----------	----------	----------	----------	----------	----------	-
Salmo_salar	----------	----------	----------	----------	----------	----------	----------	-
Didelphidae	----------	----------	----------	----------	----------	----------	----------	-
Latimeria_chalumnae	----------	----------	----------	----------	----------	----------	----------	-
Monodelphis_domestica	----------	----------	----------	----------	----------	----------	----------	-
Macropus_eugenii	----------	----------	----------	----------	----------	----------	----------	-
Alligator_mississippiensis	----------	----------	----------	----------	----------	----------	----------	-
Chrysemys_picta_bellii	----------	----------	----------	----------	----------	----------	----------	-
Danio_rerio	----------	----------	----------	----------	----------	----------	----------	-
Taeniopygia	----------	----------	----------	----------	----------	----------	----------	-
Dasypus_novemcinctus	----------	----------	----------	----------	----------	----------	----------	-
LTR_XT	----------	----------	----------	----------	----------	----------	----------	-
Haplorrhini	AGCTTGATCA	CCTCCCAGGG	AACGACCACT	TATCCAACCC	AGAGTGGCTG	GGGGCGGCAG	GAGTGGCCTG	350
Catarrhini	TTCTTGGTTC	CCTGACCGGG	AA--GCGAGG	TGATTAACGG	ACGGTCGA-G	GCAGCCCCTT	AGGCGGCTTA	68
Anolis_carolinensis	----------	----------	----------	----------	----------	----------	----------	-
Murine_leukemia_virus	----------	----------	----------	----------	----------	----------	----------	-
Phascolarctos_cinereus	----------	----------	----------	----------	----------	----------	----------	-
Estrildidae	----------	----------	----------	----------	----------	----------	----------	-
Xenopus_Silurana_tropicalis	----------	----------	----------	----------	----------	----------	----------	-
I_XT	----------	----------	----------	----------	----------	----------	----------	-
Takifugu_rubripes	----------	----------	----------	----------	----------	----------	----------	-
Bos_taurus	----------	----------	----------	----------	----------	----------	----------	-
Homo_sapiens	----------	----------	----------	----------	----------	----------	----------	-
Microcebus_murinus	----------	----------	----------	----------	----------	----------	----------	-
Myotis_lucifugus	----------	----------	----------	----------	----------	----------	----------	-
Equus_caballus	----------	----------	----------	----------	----------	----------	----------	-
Tupaia_belangeri	----------	----------	----------	----------	----------	----------	----------	-
Loxodonta_africana	----------	----------	----------	----------	----------	----------	----------	-
Murinae	----------	----------	----------	----------	----------	----------	----------	-
Canis_lupus_familiaris	----------	----------	----------	----------	----------	----------	----------	-
Sus_scrofa	----------	----------	----------	----------	----------	----------	----------	-
Choloepus_hoffmanni	----------	----------	----------	----------	----------	----------	----------	-
Vicugna_pacos	----------	----------	----------	----------	----------	----------	----------	-
Gavialis_gangeticus	----------	----------	----------	----------	----------	----------	----------	-
Mammalia	----------	----------	----------	----------	----------	----------	----------	-
Passeroidea	----------	----------	----------	----------	----------	----------	----------	-
Takifugu	----------	----------	----------	----------	----------	----------	----------	-
Pteropus_vampyrus	----------	----------	----------	----------	----------	----------	----------	-
Ornithorhynchus	----------	----------	----------	----------	----------	----------	----------	-
Oryctolagus_cuniculus	----------	----------	----------	----------	----------	----------	----------	-
Gallus_gallus	----------	----------	----------	----------	----------	----------	----------	-
Hominidae	----------	----------	----------	----------	----------	----------	----------	-
Metatheria	----------	----------	----------	----------	----------	----------	----------	-
Rodentia	----------	----------	----------	----------	----------	----------	----------	-
Monodelphis	----------	----------	----------	----------	----------	----------	----------	-
Consensus	----------	----------	----------	----------	----------	----------	----------	
Conservation (100% / 0%)								

	360 · 380 · 400 · 420	
Dipodomys_ordii	---------- ---------- ---------- ---------- ---------- ---------- ----------	-
Laurasiatheria	---------- ---------- ---------- ---------- ---------- ---------- ----------	-
Eutheria	---------- ---------- ---------- ---------- ---------- ---------- ----------	-
Tarsius_syrichta	---------- ---------- ---------- ---------- ---------- ---------- ----------	-
Muridae	---------- ---------- ---------- ---------- ---------- ---------- ----------	-
Gasterosteus_aculeatus	---------- ---------- ---------- ---------- ---------- ---------- ----------	-
Felis_silvestris	---------- ---------- ---------- ---------- ---------- ---------- ----------	-
Rattus	---------- ---------- ---------- ---------- ---------- ---------- ----------	-
Papio_hamadryas	---------- ---------- ---------- ---------- ---------- ---------- ----------	-
Pan_troglodytes	---------- ---------- ---------- ---------- ---------- ---------- ----------	-
Ailuropoda_melanoleuca	---------- ---------- ---------- ---------- ---------- ---------- ----------	-
Cavia_porcellus	---------- ---------- ---------- ---------- ---------- ---------- ----------	-
Ictidomys_tridecemlineatus	---------- ---------- ---------- ---------- ---------- ---------- ----------	-
Canis	---------- ---------- ---------- ---------- ---------- ---------- ----------	-
Felis_catus	---------- ---------- ---------- ---------- ---------- ---------- ----------	-
Ochotona_princeps	---------- ---------- ---------- ---------- ---------- ---------- ----------	-
Cercopithecidae	---------- ---------- ---------- ---------- ---------- ---------- ----------	-
Carnivora	---------- ---------- ---------- ---------- ---------- ---------- ----------	-
Mus	---------- ---------- ---------- ---------- ---------- ---------- ----------	-
Mus_musculus	---------- ---------- ---------- ---------- ---------- ---------- ----------	-
Rattus_norvegicus	---------- ---------- ---------- ---------- ---------- ---------- ----------	-
Tursiops_truncatus	---------- ---------- ---------- ---------- ---------- ---------- ----------	-
Procavia_capensis	---------- ---------- ---------- ---------- ---------- ---------- ----------	-
Primates	---------- ---------- ---------- ---------- ---------- ---------- ----------	-
Echinops_telfairi	---------- ---------- ---------- ---------- ---------- ---------- ----------	-
Sorex_araneus	---------- ---------- ---------- ---------- ---------- ---------- ----------	-
Canidae	---------- ---------- ---------- ---------- ---------- ---------- ----------	-
Otolemur_garnettii	---------- ---------- ---------- ---------- ---------- ---------- ----------	-
Gallus	---------- ---------- ---------- ---------- ---------- ---------- ----------	-
Callithrix_jacchus	---------- ---------- ---------- ---------- ---------- ---------- ----------	-
Crocodylus_porosus	---------- ---------- ---------- ---------- ---------- ---------- ----------	-
Erinaceus_europaeus	---------- ---------- ---------- ---------- ---------- ---------- ----------	-
rnd-6_family-7426-LTR-ERV1	---------- ---------- ---------- ---------- ---------- ---------- ----------	-
Esox_lucius	---------- ---------- ---------- ---------- ---------- ---------- ----------	-
Salmo_salar	---------- ---------- ---------- ---------- ---------- ---------- ----------	-
Didelphidae	---------- ---------- ---------- ---------- ---------- ---------- ----------	-
Latimeria_chalumnae	---------- ---------- ---------- ---------- ---------- ---------- ----------	-
Monodelphis_domestica	---------- ---------- ---------- ---------- ---------- ---------- ----------	-
Macropus_eugenii	---------- ---------- ---------- ---------- ---------- ---------- ----------	-
Alligator_mississippiensis	---------- ---------- ---------- ---------- ---------- ---------- ----------	-
Chrysemys_picta_bellii	---------- ---------- ---------- ---------- ---------- ---------- ----------	-
Danio_rerio	---------- ---------- ---------- ---------- ---------- ---------- ----------	-
Taeniopygia	---------- ---------- ---------- ---------- ---------- ---------- ----------	-
Dasypus_novemcinctus	---------- ---------- ---------- ---------- ---------- ---------- ----------	-
LTR_XT	---------- ---------- ---------- ---------- ---------- ---------- ----------	-
Haplorrhini	CCAATTTGGA TGAACCTCGT GTCCCCACTA CAAGCGAAAG TGGTTCACTG GATCTGGAGG CAGAAACTGG	420
Catarrhini	G------GCC TGCCCTGTGG AGCATCCCTG CGGGGGACTC CGGCCAGCTT GAGC--GACG CGGATCCTGA	130
Anolis_carolinensis	---------- ---------- ---------- ---------- ---------- ---------- ----------	-
Murine_leukemia_virus	---------- ----TTTGGG GGCTCGTCCG GGATCCGGAG ACCCCCGCCC AGGGACCACC GACCCACCGT	56
Phascolarctos_cinereus	---------- ----TTTGGG GGCTCGTCCG GGATCTG-AG ATTCCCACCC AAGGACCGCC GAACCACCGA	55
Estrildidae	---------- ---------- ---------- ---------- ---------- ---------- ----------	-
Xenopus_Silurana_tropicalis	---------- ---------- ---------- ---------- ---------- ---------- ----------	-
I_XT	---------- ---------- ---------- ---------- ---------- ---------- ----------	-
Takifugu_rubripes	---------- ---------- ---------- ---------- ---------- ---------- ----------	-
Bos_taurus	---------- ---------- ---------- ---------- ---------- ---------- ----------	-
Homo_sapiens	---------- ---------- ---------- ---------- ---------- ---------- ----------	-
Microcebus_murinus	---------- ---------- ---------- ---------- ---------- ---------- ----------	-
Myotis_lucifugus	---------- ---------- ---------- ---------- ---------- ---------- ----------	-
Equus_caballus	---------- ---------- ---------- ---------- ---------- ---------- ----------	-
Tupaia_belangeri	---------- ---------- ---------- ---------- ---------- ---------- ----------	-
Loxodonta_africana	---------- ---------- ---------- ---------- ---------- ---------- ----------	-
Murinae	---------- ---------- ---------- ---------- ---------- ---------- ----------	-
Canis_lupus_familiaris	---------- ---------- ---------- ---------- ---------- ---------- ----------	-
Sus_scrofa	---------- ---------- ---------- ---------- ---------- ---------- ----------	-
Choloepus_hoffmanni	---------- ---------- ---------- ---------- ---------- ---------- ----------	-
Vicugna_pacos	---------- ---------- ---------- ---------- ---------- ---------- ----------	-
Gavialis_gangeticus	---------- ---------- ---------- ---------- ---------- ---------- ----------	-
Mammalia	---------- ---------- ---------- ---------- ---------- ---------- ----------	-
Passeroidea	---------- ---------- ---------- ---------- ---------- ---------- ----------	-
Takifugu	---------- ---------- ---------- ---------- ---------- ---------- ----------	-
Pteropus_vampyrus	---------- ---------- ---------- ---------- ---------- ---------- ----------	-
Ornithorhynchus	---------- ---------- ---------- ---------- ---------- ---------- ----------	-
Oryctolagus_cuniculus	---------- ---------- ---------- ---------- ---------- ---------- ----------	-
Gallus_gallus	---------- ---------- ---------- ---------- ---------- ---------- ----------	-
Hominidae	---------- ---------- ---------- ---------- ---------- ---------- ----------	-
Metatheria	---------- ---------- ---------- ---------- ---------- ---------- ----------	-
Rodentia	---------- ---------- ---------- ---------- ---------- ---------- ----------	-
Monodelphis	---------- ---------- ---------- ---------- ---------- ---------- ----------	-
Consensus	---------- ---------- ---------- ---------- ---------- ---------- ----------	

Conservation
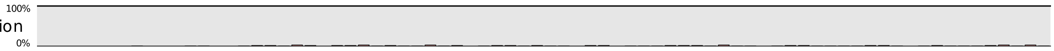

440 460 480

Dipodomys_ordii	----------	----------	----------	----------	----------	----------	----------	-
Laurasiatheria	----------	----------	----------	----------	----------	----------	----------	-
Eutheria	----------	----------	----------	----------	----------	----------	----------	-
Tarsius_syrichta	----------	----------	----------	----------	----------	----------	----------	-
Muridae	----------	----------	----------	----------	----------	----------	----------	-
Gasterosteus_aculeatus	----------	----------	----------	----------	----------	----------	----------	-
Felis_silvestris	----------	----------	----------	----------	----------	----------	----------	-
Rattus	----------	----------	----------	----------	----------	----------	----------	-
Papio_hamadryas	----------	----------	----------	----------	----------	----------	----------	-
Pan_troglodytes	----------	----------	----------	----------	----------	----------	----------	-
Ailuropoda_melanoleuca	----------	----------	----------	----------	----------	----------	----------	-
Cavia_porcellus	----------	----------	----------	----------	----------	----------	----------	-
Ictidomys_tridecemlineatus	----------	----------	----------	----------	----------	----------	----------	-
Canis	----------	----------	----------	----------	----------	----------	----------	-
Felis_catus	----------	----------	----------	----------	----------	----------	----------	-
Ochotona_princeps	----------	----------	----------	----------	----------	----------	----------	-
Cercopithecidae	----------	----------	----------	----------	----------	----------	----------	-
Carnivora	----------	----------	----------	----------	----------	----------	----------	-
Mus	----------	----------	----------	----------	----------	----------	----------	-
Mus_musculus	----------	----------	----------	----------	----------	----------	----------	-
Rattus_norvegicus	----------	----------	----------	----------	----------	----------	----------	-
Tursiops_truncatus	----------	----------	----------	----------	----------	----------	----------	-
Procavia_capensis	----------	----------	----------	----------	----------	----------	----------	-
Primates	----------	----------	----------	----------	----------	----------	----------	-
Echinops_telfairi	----------	----------	----------	----------	----------	----------	----------	-
Sorex_araneus	----------	----------	----------	----------	----------	----------	----------	-
Canidae	----------	----------	----------	----------	----------	----------	----------	-
Otolemur_garnettii	----------	----------	----------	----------	----------	----------	----------	-
Gallus	----------	----------	----------	----------	----------	----------	----------	-
Callithrix_jacchus	----------	----------	----------	----------	----------	----------	----------	-
Crocodylus_porosus	----------	----------	----------	----------	----------	----------	----------	-
Erinaceus_europaeus	----------	----------	----------	----------	----------	----------	----------	-
rnd-6_family-7426-LTR-ERV1	----------	----------	----------	----------	----------	----------	----------	-
Esox_lucius	----------	----------	----------	----------	----------	----------	----------	-
Salmo_salar	----------	----------	----------	----------	----------	----------	----------	-
Didelphidae	----------	----------	----------	----------	----------	----------	----------	-
Latimeria_chalumnae	----------	----------	----------	----------	----------	----------	----------	-
Monodelphis_domestica	----------	----------	----------	----------	----------	----------	----------	-
Macropus_eugenii	----------	----------	----------	----------	----------	----------	----------	-
Alligator_mississippiensis	----------	----------	----------	----------	----------	----------	----------	-
Chrysemys_picta_bellii	----------	----------	----------	----------	----------	----------	----------	-
Danio_rerio	----------	----------	----------	----------	----------	----------	----------	-
Taeniopygia	----------	----------	----------	----------	----------	----------	----------	-
Dasypus_novemcinctus	----------	----------	----------	----------	----------	----------	----------	-
LTR_XT	----------	----------	----------	----------	----------	----------	----------	-
Haplorrhini	GAGTGTGTGG	GTGCGTGTGA	ACCTACCCGG	GACACAAGGA	AGGCTTGTTT	CATCCGATGA	GGT-GGGGTA	489
Catarrhini	GAGCGCTCC-	-CGGGTAGGC	AATTGCCCCG	GTGGAACGCC	TCGCCAGAGC	AGCGCGTGGC	AGG-CCCCCG	197
Anolis_carolinensis	----------	----------	----------	----------	----------	----------	----------	-
Murine_leukemia_virus	CGGGAGGTAA	GCTGGCCAGC	GATCGTTTCG	TNNNNGNCNC	NNNCNTNNNG	CNTNTGNNNG	CNNGCNGNNN	126
Phascolarctos_cinereus	CGGGAGGTAA	GCTGGCCAGC	GATCGCTCTA	TGTTCTCTCT	GTGTCTGAC-	--TCCGAAAA	CTC-TGACTG	121
Estrildidae	----------	----------	----------	----------	----------	----------	----------	-
Xenopus_Silurana_tropicalis	----------	----------	----------	----------	----------	----------	----------	-
I_XT	----------	----------	----------	----------	----------	----------	----------	-
Takifugu_rubripes	----------	----------	----------	----------	----------	----------	----------	-
Bos_taurus	----------	----------	----------	----------	----------	----------	----------	-
Homo_sapiens	----------	----------	----------	----------	----------	----------	----------	-
Microcebus_murinus	----------	----------	----------	----------	----------	----------	----------	-
Myotis_lucifugus	----------	----------	----------	----------	----------	----------	----------	-
Equus_caballus	----------	----------	----------	----------	----------	----------	----------	-
Tupaia_belangeri	----------	----------	----------	----------	----------	----------	----------	-
Loxodonta_africana	----------	----------	----------	----------	----------	----------	----------	-
Murinae	----------	----------	----------	----------	----------	----------	----------	-
Canis_lupus_familiaris	----------	----------	----------	----------	----------	----------	----------	-
Sus_scrofa	----------	----------	----------	----------	----------	----------	----------	-
Choloepus_hoffmanni	----------	----------	----------	----------	----------	----------	----------	-
Vicugna_pacos	----------	----------	----------	----------	----------	----------	----------	-
Gavialis_gangeticus	----------	----------	----------	----------	----------	----------	----------	-
Mammalia	----------	----------	----------	----------	----------	----------	----------	-
Passeroidea	----------	----------	----------	----------	----------	----------	----------	-
Takifugu	----------	----------	----------	----------	----------	----------	----------	-
Pteropus_vampyrus	----------	----------	----------	----------	----------	----------	----------	-
Ornithorhynchus	----------	----------	----------	----------	----------	----------	----------	-
Oryctolagus_cuniculus	----------	----------	----------	----------	----------	----------	----------	-
Gallus_gallus	----------	----------	----------	----------	----------	----------	----------	-
Hominidae	----------	----------	----------	----------	----------	----------	----------	-
Metatheria	----------	----------	----------	----------	----------	----------	----------	-
Rodentia	----------	----------	----------	----------	----------	----------	----------	-
Monodelphis	----------	----------	----------	----------	----------	----------	----------	-
Consensus	----------	----------	----------	----------	----------	----------	----------	

Conservation 100% 0%

```
                                    500                           520                           540                         560
                                      |                             |                             |                           |
Dipodomys_ordii             ---------- ---------- ---------- ---------- ---------- ---------- ---------- -
Laurasiatheria              ---------- ---------- ---------- ---------- ---------- ---------- ---------- -
Eutheria                    ---------- ---------- ---------- ---------- ---------- ---------- ---------- -
Tarsius_syrichta            ---------- ---------- ---------- ---------- ---------- ---------- ---------- -
Muridae                     ---------- ---------- ---------- ---------- ---------- ---------- ---------- -
Gasterosteus_aculeatus      ---------- ---------- ---------- ---------- ---------- ---------- ---------- -
Felis_silvestris            ---------- ---------- ---------- ---------- ---------- ---------- ---------- -
Rattus                      ---------- ---------- ---------- ---------- ---------- ---------- ---------- -
Papio_hamadryas             ---------- ---------- ---------- ---------- ---------- ---------- ---------- -
Pan_troglodytes             ---------- ---------- ---------- ---------- ---------- ---------- ---------- -
Ailuropoda_melanoleuca      ---------- ---------- ---------- ---------- ---------- ---------- ---------- -
Cavia_porcellus             ---------- ---------- ---------- ---------- ---------- ---------- ---------- -
Ictidomys_tridecemlineatus  ---------- ---------- ---------- ---------- ---------- ---------- ---------- -
Canis                       ---------- ---------- ---------- ---------- ---------- ---------- ---------- -
Felis_catus                 ---------- ---------- ---------- ---------- ---------- ---------- ---------- -
Ochotona_princeps           ---------- ---------- ---------- ---------- ---------- ---------- ---------- -
Cercopithecidae             ---------- ---------- ---------- ---------- ---------- ---------- ---------- -
Carnivora                   ---------- ---------- ---------- ---------- ---------- ---------- ---------- -
Mus                         ---------- ---------- ---------- ---------- ---------- ---------- ---------- -
Mus_musculus                ---------- ---------- ---------- ---------- ---------- ---------- ---------- -
Rattus_norvegicus           ---------- ---------- ---------- ---------- ---------- ---------- ---------- -
Tursiops_truncatus          ---------- ---------- ---------- ---------- ---------- ---------- ---------- -
Procavia_capensis           ---------- ---------- ---------- ---------- ---------- ---------- ---------- -
Primates                    ---------- ---------- ---------- ---------- ---------- ---------- ---------- -
Echinops_telfairi           ---------- ---------- ---------- ---------- ---------- ---------- ---------- -
Sorex_araneus               ---------- ---------- ---------- ---------- ---------- ---------- ---------- -
Canidae                     ---------- ---------- ---------- ---------- ---------- ---------- ---------- -
Otolemur_garnettii          ---------- ---------- ---------- ---------- ---------- ---------- ---------- -
Gallus                      ---------- ---------- ---------- ---------- ---------- ---------- ---------- -
Callithrix_jacchus          ---------- ---------- ---------- ---------- ---------- ---------- ---------- -
Crocodylus_porosus          ---------- ---------- ---------- ---------- ---------- ---------- ---------- -
Erinaceus_europaeus         ---------- ---------- ---------- ---------- ---------- ---------- ---------- -
rnd-6_family-7426-LTR-ERV1  ---------- ---------- ---------- ---------- ---------- ---------- ---------- -
Esox_lucius                 ---------- ---------- ---------- ---------- ---------- ---------- ---------- -
Salmo_salar                 ---------- ---------- ---------- ---------- ---------- ---------- ---------- -
Didelphidae                 ---------- ---------- ---------- ---------- ---------- ---------- ---------- -
Latimeria_chalumnae         ---------- ---------- ---------- ---------- ---------- ---------- ---------- -
Monodelphis_domestica       ---------- ---------- ---------- ---------- ---------- ---------- ---------- -
Macropus_eugenii            ---------- ---------- ---------- ---------- ---------- ---------- ---------- -
Alligator_mississippiensis  ---------- ---------- ---------- ---------- ---------- ---------- ---------- -
Chrysemys_picta_bellii      ---------- ---------- ---------- ---------- ---------- ---------- ---------- -
Danio_rerio                 ---------- ---------- ---------- ---------- ---------- ---------- ---------- -
Taeniopygia                 ---------- ---------- ---------- ---------- ---------- ---------- ---------- -
Dasypus_novemcinctus        ---------- ---------- ---------- ---------- ---------- ---------- ---------- -
LTR_XT                      ---------- ---------- ---------- ---------- ---------- ---------- ---------- -
Haplorrhini                 GGAGTGGTGT GTGTATGTGT GTGAATGTGG GAGCCTAACT AG-GCTCACC CGGGACACGA GAGAGGCTCG 558
Catarrhini                  TGGAGGATCA ACGCA-GTGG CTGAACACCG GGAAGGAACT GGCACTTGGA GTCCGGACAN CTGAAACTTG 266
Anolis_carolinensis         ---------- ---------- ---------- ---------- ---------- ---------- ---------- -
Murine_leukemia_virus       TNNNNNCNTT NNGCNNGCGN NTGAANNNGT ACNAGTTAGC TN-ACTAGAT CTGTNNNNNN NNGTNNCNNG 195
Phascolarctos_cinereus      TCTATTCGGT GTGCGCGCAT TT---TTGGT TTATTCCGGG TT-A----AT CGATCTAAGA TCGAGGCGTG 183
Estrildidae                 ---------- ---------- ---------- ---------- ---------- ---------- ---------- -
Xenopus_Silurana_tropicalis ---------- ---------- ---------- ---------- ---------- ---------- ---------- -
I_XT                        ---------- ---------- ---------- -TTCGTCACC GGATGTTTAC GTTAAACAGA CCCCAATAAG 39
Takifugu_rubripes           ---------- ---------- ---------- ---------- ---------- ---------- ---------- -
Bos_taurus                  ---------- ---------- ---------- ---------- ---------- ---------- ---------- -
Homo_sapiens                ---------- ---------- ---------- ---------- ---------- ---------- ---------- -
Microcebus_murinus          ---------- ---------- ---------- ---------- ---------- ---------- ---------- -
Myotis_lucifugus            ---------- ---------- ---------- ---------- ---------- ---------- ---------- -
Equus_caballus              ---------- ---------- ---------- ---------- ---------- ---------- ---------- -
Tupaia_belangeri            ---------- ---------- ---------- ---------- ---------- ---------- ---------- -
Loxodonta_africana          ---------- ---------- ---------- ---------- ---------- ---------- ---------- -
Murinae                     ---------- ---------- ---------- ---------- ---------- ---------- ---------- -
Canis_lupus_familiaris      ---------- ---------- ---------- ---------- ---------- ---------- ---------- -
Sus_scrofa                  ---------- ---------- ---------- ---------- ---------- ---------- ---------- -
Choloepus_hoffmanni         ---------- ---------- ---------- ---------- ---------- ---------- ---------- -
Vicugna_pacos               ---------- ---------- ---------- ---------- ---------- ---------- ---------- -
Gavialis_gangeticus         ---------- ---------- ---------- ---------- ---------- ---------- ---------- -
Mammalia                    ---------- ---------- ---------- ---------- ---------- ---------- ---------- -
Passeroidea                 ---------- ---------- ---------- ---------- ---------- ---------- ---------- -
Takifugu                    ---------- ---------- ---------- ---------- ---------- ---------- ---------- -
Pteropus_vampyrus           ---------- ---------- ---------- ---------- ---------- ---------- ---------- -
Ornithorhynchus             ---------- ---------- ---------- ---------- ---------- ---------- ---------- -
Oryctolagus_cuniculus       ---------- ---------- ---------- ---------- ---------- ---------- ---------- -
Gallus_gallus               ---------- ---------- ---------- ---------- ---------- ---------- ---------- -
Hominidae                   ---------- ---------- ---------- ---------- ---------- ---------- ---------- -
Metatheria                  ---------- ---------- ---------- ---------- ---------- ---------- ---------- -
Rodentia                    ---------- ---------- ---------- ---------- ---------- ---------- ---------- -
Monodelphis                 ---------- ---------- ---------- ---------- ---------- ---------- ---------- -
Consensus                   ---------- ---------- ---------- ---------- ---------- ---------- ----------
                       100%
Conservation
                         0%
```

		580		600		620		
Dipodomys_ordii	----------	----------	----------	----------	----------	----------	----------	-
Laurasiatheria	----------	----------	----------	----------	----------	----------	----------	-
Eutheria	----------	----------	----------	----------	----------	----------	----------	-
Tarsius_syrichta	----------	----------	----------	----------	----------	----------	----------	-
Muridae	----------	----------	----------	----------	----------	----------	----------	-
Gasterosteus_aculeatus	----------	----------	----------	----------	----------	----------	----------	-
Felis_silvestris	----------	----------	----------	----------	----------	----------	----------	-
Rattus	----------	----------	----------	----------	----------	----------	----------	-
Papio_hamadryas	----------	----------	----------	----------	----------	----------	----------	-
Pan_troglodytes	----------	----------	----------	----------	----------	----------	----------	-
Ailuropoda_melanoleuca	----------	----------	----------	----------	----------	----------	----------	-
Cavia_porcellus	----------	----------	----------	----------	----------	----------	----------	-
Ictidomys_tridecemlineatus	----------	----------	----------	----------	----------	----------	----------	-
Canis	----------	----------	----------	----------	----------	----------	----------	-
Felis_catus	----------	----------	----------	----------	----------	----------	----------	-
Ochotona_princeps	----------	----------	----------	----------	----------	----------	----------	-
Cercopithecidae	----------	----------	----------	----------	----------	----------	----------	-
Carnivora	----------	----------	----------	----------	----------	----------	----------	-
Mus	----------	----------	----------	----------	----------	----------	----------	-
Mus_musculus	----------	----------	----------	----------	----------	----------	----------	-
Rattus_norvegicus	----------	----------	----------	----------	----------	----------	----------	-
Tursiops_truncatus	----------	----------	----------	----------	----------	----------	----------	-
Procavia_capensis	----------	----------	----------	----------	----------	----------	----------	-
Primates	----------	----------	----------	----------	----------	----------	----------	-
Echinops_telfairi	----------	----------	----------	----------	----------	----------	----------	-
Sorex_araneus	----------	----------	----------	----------	----------	----------	----------	-
Canidae	----------	----------	----------	----------	----------	----------	----------	-
Otolemur_garnettii	----------	----------	----------	----------	----------	----------	----------	-
Gallus	----------	----------	----------	----------	----------	----------	----------	-
Callithrix_jacchus	----------	----------	----------	----------	----------	----------	----------	-
Crocodylus_porosus	----------	----------	----------	----------	----------	----------	----------	-
Erinaceus_europaeus	----------	----------	----------	----------	----------	----------	----------	-
rnd-6_family-7426-LTR-ERV1	----------	----------	----------	----------	----------	----------	----------	-
Esox_lucius	----------	----------	----------	----------	----------	----------	----------	-
Salmo_salar	----------	----------	----------	----------	----------	----------	----------	-
Didelphidae	----------	----------	----------	----------	----------	----------	----------	-
Latimeria_chalumnae	----------	----------	----------	----------	----------	----------	----------	-
Monodelphis_domestica	----------	----------	----------	----------	----------	----------	----------	-
Macropus_eugenii	----------	----------	----------	----------	----------	----------	----------	-
Alligator_mississippiensis	----------	----------	----------	----------	----------	----------	----------	-
Chrysemys_picta_bellii	----------	----------	----------	----------	----------	----------	----------	-
Danio_rerio	----------	----------	----------	----------	----------	----------	----------	-
Taeniopygia	----------	----------	----------	----------	----------	----------	----------	-
Dasypus_novemcinctus	----------	----------	----------	----------	----------	----------	----------	-
LTR_XT	----------	----------	----------	----------	----------	----------	----------	-
Haplorrhini	TTTCATCTGA	TGAGGAGTCC	TGGGGCAGGG	GAGGTGTGTG	AAAGTGTGTG	AAAGAGATGG	TCTCGGGAGA	628
Catarrhini	GTAAGACTAG	TCTTTGGAAC	TTGCCCACTC	CATTTGAGTG	GAAGCGTG--	-GCCTGATCA	CC-CACGGCG	332
Anolis_carolinensis	----------	----------	----------	----------	----------	----------	----------	-
Murine_leukemia_virus	GAAGNANTNN	NNANNNNNTA	NNNCNGNNCN	NANCNNNGNN	AGNNGNCNNA	NAGGANATCN	GN-GGNCGAN	264
Phascolarctos_cinereus	--AGTAGCGG	ACAGACGT-G	TTCGGGGGCT	CACCGC--CC	GGCAATCCTG	GGAGACGTCC	CA-GGATCAG	247
Estrildidae	----------	----------	----------	----------	----------	----------	----------	-
Xenopus_Silurana_tropicalis	----------	----------	----------	----------	----------	----------	----------	-
I_XT	GCATATTTTG	AAAATAAATT	GATTAATCTT	ATATTGTCTT	ATGAACAATG	TATGAANCAA	NTATTANTNG	109
Takifugu_rubripes	----------	----------	----------	----------	----------	----------	----------	-
Bos_taurus	----------	----------	----------	----------	----------	----------	----------	-
Homo_sapiens	----------	----------	----------	----------	----------	----------	----------	-
Microcebus_murinus	----------	----------	----------	----------	----------	----------	----------	-
Myotis_lucifugus	----------	----------	----------	----------	----------	----------	----------	-
Equus_caballus	----------	----------	----------	----------	----------	----------	----------	-
Tupaia_belangeri	----------	----------	----------	----------	----------	----------	----------	-
Loxodonta_africana	----------	----------	----------	----------	----------	----------	----------	-
Murinae	----------	----------	----------	----------	----------	----------	----------	-
Canis_lupus_familiaris	----------	----------	----------	----------	----------	----------	----------	-
Sus_scrofa	----------	----------	----------	----------	----------	----------	----------	-
Choloepus_hoffmanni	----------	----------	----------	----------	----------	----------	----------	-
Vicugna_pacos	----------	----------	----------	----------	----------	----------	----------	-
Gavialis_gangeticus	----------	----------	----------	----------	----------	----------	----------	-
Mammalia	----------	----------	----------	----------	----------	----------	----------	-
Passeroidea	----------	----------	----------	----------	----------	----------	----------	-
Takifugu	----------	----------	----------	----------	----------	----------	----------	-
Pteropus_vampyrus	----------	----------	----------	----------	----------	----------	----------	-
Ornithorhynchus	----------	----------	----------	----------	----------	----------	----------	-
Oryctolagus_cuniculus	----------	----------	----------	----------	----------	----------	----------	-
Gallus_gallus	----------	----------	----------	----------	----------	----------	----------	-
Hominidae	----------	----------	----------	----------	----------	----------	----------	-
Metatheria	----------	----------	----------	----------	----------	----------	----------	-
Rodentia	----------	----------	----------	----------	----------	----------	----------	-
Monodelphis	----------	----------	----------	----------	----------	----------	----------	-
Consensus	----------	----------	----------	----------	----------	----------	----------	

Conservation (100% / 0%)

	640		660		680		700	
Dipodomys_ordii	----------	----------	----------	----------	----------	----------	----------	-
Laurasiatheria	----------	----------	----------	----------	----------	----------	----------	-
Eutheria	----------	----------	----------	----------	----------	----------	----------	-
Tarsius_syrichta	----------	----------	----------	----------	----------	----------	----------	-
Muridae	----------	----------	----------	----------	----------	----------	----------	-
Gasterosteus_aculeatus	----------	----------	----------	----------	----------	----------	----------	-
Felis_silvestris	----------	----------	----------	----------	----------	----------	----------	-
Rattus	----------	----------	----------	----------	----------	----------	----------	-
Papio_hamadryas	----------	----------	----------	----------	----------	----------	----------	-
Pan_troglodytes	----------	----------	----------	----------	----------	----------	----------	-
Ailuropoda_melanoleuca	----------	----------	----------	----------	----------	----------	----------	-
Cavia_porcellus	----------	----------	----------	----------	----------	----------	----------	-
Ictidomys_tridecemlineatus	----------	----------	----------	----------	----------	----------	----------	-
Canis	----------	----------	----------	----------	----------	----------	----------	-
Felis_catus	----------	----------	----------	----------	----------	----------	----------	-
Ochotona_princeps	----------	----------	----------	----------	----------	----------	----------	-
Cercopithecidae	----------	----------	----------	----------	----------	----------	----------	-
Carnivora	----------	----------	----------	----------	----------	----------	----------	-
Mus	----------	----------	----------	----------	----------	----------	----------	-
Mus_musculus	----------	----------	----------	----------	----------	----------	----------	-
Rattus_norvegicus	----------	----------	----------	----------	----------	----------	----------	-
Tursiops_truncatus	----------	----------	----------	----------	----------	----------	----------	-
Procavia_capensis	----------	----------	----------	----------	----------	----------	----------	-
Primates	----------	----------	----------	----------	----------	----------	----------	-
Echinops_telfairi	----------	----------	----------	----------	----------	----------	----------	-
Sorex_araneus	----------	----------	----------	----------	----------	----------	----------	-
Canidae	----------	----------	----------	----------	----------	----------	----------	-
Otolemur_garnettii	----------	----------	----------	----------	----------	----------	----------	-
Gallus	----------	----------	----------	----------	----------	----------	----------	-
Callithrix_jacchus	----------	----------	----------	----------	----------	----------	----------	-
Crocodylus_porosus	----------	----------	----------	----------	----------	----------	----------	-
Erinaceus_europaeus	----------	----------	----------	----------	----------	----------	----------	-
rnd-6_family-7426-LTR-ERV1	----------	----------	----------	----------	----------	----------	----------	-
Esox_lucius	----------	----------	----------	----------	----------	----------	----------	-
Salmo_salar	----------	----------	----------	----------	----------	----------	----------	-
Didelphidae	----------	----------	----------	----------	----------	----------	----------	-
Latimeria_chalumnae	----------	----------	----------	----------	----------	----------	----------	-
Monodelphis_domestica	----------	----------	----------	----------	----------	----------	----------	-
Macropus_eugenii	----------	----------	----------	----------	----------	----------	----------	-
Alligator_mississippiensis	----------	----------	----------	----------	----------	----------	----------	-
Chrysemys_picta_bellii	----------	----------	----------	----------	----------	----------	----------	-
Danio_rerio	----------	----------	----------	----------	----------	----------	----------	-
Taeniopygia	----------	----------	----------	----------	----------	----------	----------	-
Dasypus_novemcinctus	----------	----------	----------	----------	----------	----------	----------	-
LTR_XT	----------	----------	----------	----------	----------	----------	----------	-
Haplorrhini	GGCCAATGCG	GGGAGTGATG	TGGGGAGGTG	CAGATCTCTT	AGCGCAGACT	GTGTCCTCCG	AGGCGAGTGT	698
Catarrhini	TGCCTGTACC	GGCACT----	TTGGTTTTTG	TTTTCGACTT	GACTTGGATT	GCTTGATACT	T--TGGTTTT	396
Anolis_carolinensis	----------	----------	----------	----------	----------	----------	----------	-
Murine_leukemia_virus	NGCNTGGNCC	NNNNCNNANG	NCCNANCANN	NNANCNGTNN	NNGANNANNT	GGAGCCCCNN	CTNNGGNNNA	334
Phascolarctos_cinereus	GG-GAGGACC	AGGG---ACG	CCTGGTGGAT	CCCACGGCAA	GGGATAATT-	-------CTC	CTCTGATCT-	304
Estrildidae	----------	----------	----------	----------	----------	----------	----------	-
Xenopus_Silurana_tropicalis	----------	----------	----------	----------	----------	----------	----------	-
I_XT	NCTTAAAGTT	TGTGTNTGTA	AACANAAANA	NGTAANATTC	TGTNTATNTT	AAGATNGTAN	AGNNTTTTAA	179
Takifugu_rubripes	----------	----------	----------	----------	----------	----------	----------	-
Bos_taurus	----------	----------	----------	----------	----------	----------	----------	-
Homo_sapiens	----------	----------	----------	----------	----------	----------	----------	-
Microcebus_murinus	----------	----------	----------	----------	----------	----------	----------	-
Myotis_lucifugus	----------	----------	----------	----------	----------	----------	----------	-
Equus_caballus	----------	----------	----------	----------	----------	----------	----------	-
Tupaia_belangeri	----------	----------	----------	----------	----------	----------	----------	-
Loxodonta_africana	----------	----------	----------	----------	----------	----------	----------	-
Murinae	----------	----------	----------	----------	----------	----------	----------	-
Canis_lupus_familiaris	----------	----------	----------	----------	----------	----------	----------	-
Sus_scrofa	----------	----------	----------	----------	----------	----------	----------	-
Choloepus_hoffmanni	----------	----------	----------	----------	----------	----------	----------	-
Vicugna_pacos	----------	----------	----------	----------	----------	----------	----------	-
Gavialis_gangeticus	----------	----------	----------	----------	----------	----------	----------	-
Mammalia	----------	----------	----------	----------	----------	----------	----------	-
Passeroidea	----------	----------	----------	----------	----------	----------	----------	-
Takifugu	----------	----------	----------	----------	----------	----------	----------	-
Pteropus_vampyrus	----------	----------	----------	----------	----------	----------	----------	-
Ornithorhynchus	----------	----------	----------	----------	----------	----------	----------	-
Oryctolagus_cuniculus	----------	----------	----------	----------	----------	----------	----------	-
Gallus_gallus	----------	----------	----------	----------	----------	----------	----------	-
Hominidae	----------	----------	----------	----------	----------	----------	----------	-
Metatheria	----------	----------	----------	----------	----------	----------	----------	-
Rodentia	----------	----------	----------	----------	----------	----------	----------	-
Monodelphis	----------	----------	----------	----------	----------	----------	----------	-
Consensus	----------	----------	----------	----------	----------	----------	----------	

Conservation

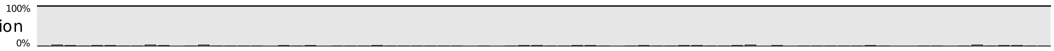

		720		740		760		
Dipodomys_ordii	----------	----------	----------	----------	----------	----------	----------	-
Laurasiatheria	----------	----------	----------	----------	----------	----------	----------	-
Eutheria	----------	----------	----------	----------	----------	----------	----------	-
Tarsius_syrichta	----------	----------	----------	----------	----------	----------	----------	-
Muridae	----------	----------	----------	----------	----------	----------	----------	-
Gasterosteus_aculeatus	----------	----------	----------	----------	----------	----------	----------	-
Felis_silvestris	----------	----------	----------	----------	----------	----------	----------	-
Rattus	----------	----------	----------	----------	----------	----------	----------	-
Papio_hamadryas	----------	----------	----------	----------	----------	----------	----------	-
Pan_troglodytes	----------	----------	----------	----------	----------	----------	----------	-
Ailuropoda_melanoleuca	----------	----------	----------	----------	----------	----------	----------	-
Cavia_porcellus	----------	----------	----------	----------	----------	----------	----------	-
Ictidomys_tridecemlineatus	----------	----------	----------	----------	----------	----------	----------	-
Canis	----------	----------	----------	----------	----------	----------	----------	-
Felis_catus	----------	----------	----------	----------	----------	----------	----------	-
Ochotona_princeps	----------	----------	----------	----------	----------	----------	----------	-
Cercopithecidae	----------	----------	----------	----------	----------	----------	----------	-
Carnivora	----------	----------	----------	----------	----------	----------	----------	-
Mus	----------	----------	----------	----------	----------	----------	----------	-
Mus_musculus	----------	----------	----------	----------	----------	----------	----------	-
Rattus_norvegicus	----------	----------	----------	----------	----------	----------	----------	-
Tursiops_truncatus	----------	----------	----------	----------	----------	----------	----------	-
Procavia_capensis	----------	----------	----------	----------	----------	----------	----------	-
Primates	----------	----------	----------	----------	----------	----------	----------	-
Echinops_telfairi	----------	----------	----------	----------	----------	----------	----------	-
Sorex_araneus	----------	----------	----------	----------	----------	----------	----------	-
Canidae	----------	----------	----------	----------	----------	----------	----------	-
Otolemur_garnettii	----------	----------	----------	----------	----------	----------	----------	-
Gallus	----------	----------	----------	----------	----------	----------	----------	-
Callithrix_jacchus	----------	----------	----------	----------	----------	----------	----------	-
Crocodylus_porosus	----------	----------	----------	----------	----------	----------	----------	-
Erinaceus_europaeus	----------	----------	----------	----------	----------	----------	----------	-
rnd-6_family-7426-LTR-ERV1	----------	----------	----------	----------	----------	----------	----------	-
Esox_lucius	----------	----------	----------	----------	----------	----------	----------	-
Salmo_salar	----------	----------	----------	----------	----------	----------	----------	-
Didelphidae	----------	----------	----------	----------	----------	----------	----------	-
Latimeria_chalumnae	----------	----------	----------	----------	----------	----------	----------	-
Monodelphis_domestica	----------	----------	----------	----------	----------	----------	----------	-
Macropus_eugenii	----------	----------	----------	----------	----------	----------	----------	-
Alligator_mississippiensis	----------	----------	----------	----------	----------	----------	----------	-
Chrysemys_picta_bellii	----------	----------	----------	----------	----------	----------	----------	-
Danio_rerio	----------	----------	----------	----------	----------	----------	----------	-
Taeniopygia	----------	----------	----------	----------	----------	----------	----------	-
Dasypus_novemcinctus	----------	----------	----------	----------	----------	----------	----------	-
LTR_XT	----------	----------	----------	----------	----------	----------	----------	-
Haplorrhini	GGGACGAGCC	AGACCTAGGT	CACTGCGTAA	GGCCGACAGG	ATTAGCTTCA	TAGCTTCACA	GCAGTAGTTG	768
Catarrhini	GGTTTTGACC	TGGCTTGGAT	TTCT-CGATA	CTCTGAT---	TTTGGTTTT-	--GATTC---	----TGGTTT	452
Anolis_carolinensis	----------	----------	----------	----------	----------	----------	----------	-
Murine_leukemia_virus	NGNNNANNTG	GTNCTCNNNN	NANGCGANNG	GCCCNAGCNN	NNGNNTCNNN	NANNTNNNNN	NTNNNTTTCN	404
Phascolarctos_cinereus	--CACAGTTG	--CACTGTCT	TGAGAGACAT	ACCCTACCTT	CTGACTCTTT	TTCCTGTCTT	TTTAATATAC	370
Estrildidae	----------	----------	----------	----------	----------	----------	----------	-
Xenopus_Silurana_tropicalis	----------	----------	----------	----------	----------	----------	----------	-
I_XT	NACTATGATN	NACANANANT	GTTTTNNNCA	GANTNNTTGN	AATCTNATNN	ACANAATAAA	GACACAGAAT	249
Takifugu_rubripes	----------	----------	----------	----------	----------	----------	----------	-
Bos_taurus	----------	----------	----------	----------	----------	----------	----------	-
Homo_sapiens	----------	----------	----------	----------	----------	----------	----------	-
Microcebus_murinus	----------	----------	----------	----------	----------	----------	----------	-
Myotis_lucifugus	----------	----------	----------	----------	----------	----------	----------	-
Equus_caballus	----------	----------	----------	----------	----------	----------	----------	-
Tupaia_belangeri	----------	----------	----------	----------	----------	----------	----------	-
Loxodonta_africana	----------	----------	----------	----------	----------	----------	----------	-
Murinae	----------	----------	----------	----------	----------	----------	----------	-
Canis_lupus_familiaris	----------	----------	----------	----------	----------	----------	----------	-
Sus_scrofa	----------	----------	----------	----------	----------	----------	----------	-
Choloepus_hoffmanni	----------	----------	----------	----------	----------	----------	----------	-
Vicugna_pacos	----------	----------	----------	----------	----------	----------	----------	-
Gavialis_gangeticus	----------	----------	----------	----------	----------	----------	----------	-
Mammalia	----------	----------	----------	----------	----------	----------	----------	-
Passeroidea	----------	----------	----------	----------	----------	----------	----------	-
Takifugu	----------	----------	----------	----------	----------	----------	----------	-
Pteropus_vampyrus	----------	----------	----------	----------	----------	----------	----------	-
Ornithorhynchus	----------	----------	----------	----------	----------	----------	----------	-
Oryctolagus_cuniculus	----------	----------	----------	----------	----------	----------	----------	-
Gallus_gallus	----------	----------	----------	----------	----------	----------	----------	-
Hominidae	----------	----------	----------	----------	----------	----------	----------	-
Metatheria	----------	----------	----------	----------	----------	----------	----------	-
Rodentia	----------	----------	----------	----------	----------	----------	----------	-
Monodelphis	----------	----------	----------	----------	----------	----------	----------	-
Consensus	----------	----------	----------	----------	----------	----------	----------	

Conservation 100% 0%

```
                                  780         800         820         840
                                    |           |           |           |
Dipodomys_ordii              ---------- ---------- ---------- ---------- ---------- ---------- ---------- -
Laurasiatheria               ---------- ---------- ---------- ---------- ---------- ---------- ---------- -
Eutheria                     ---------- ---------- ---------- ---------- ---------- ---------- ---------- -
Tarsius_syrichta             ---------- ---------- ---------- ---------- ---------- ---------- ---------- -
Muridae                      ---------- ---------- ---------- ---------- ---------- ---------- ---------- -
Gasterosteus_aculeatus       ---------- ---------- ---------- ---------- ---------- ---------- ---------- -
Felis_silvestris             ---------- ---------- ---------- ---------- ---------- ---------- ---------- -
Rattus                       ---------- ---------- ---------- ---------- ---------- ---------- ---------- -
Papio_hamadryas              ---------- ---------- ---------- ---------- ---------- ---------- ---------- -
Pan_troglodytes              ---------- ---------- ---------- ---------- ---------- ---------- ---------- -
Ailuropoda_melanoleuca       ---------- ---------- ---------- ---------- ---------- ---------- ---------- -
Cavia_porcellus              ---------- ---------- ---------- ---------- ---------- ---------- ---------- -
Ictidomys_tridecemlineatus   ---------- ---------- ---------- ---------- ---------- ---------- ---------- -
Canis                        ---------- ---------- ---------- ---------- ---------- ---------- ---------- -
Felis_catus                  ---------- ---------- ---------- ---------- ---------- ---------- ---------- -
Ochotona_princeps            ---------- ---------- ---------- ---------- ---------- ---------- ---------- -
Cercopithecidae              ---------- ---------- ---------- ---------- ---------- ---------- ---------- -
Carnivora                    ---------- ---------- ---------- ---------- ---------- ---------- ---------- -
Mus                          ---------- ---------- ---------- ---------- ---------- ---------- ---------- -
Mus_musculus                 ---------- ---------- ---------- ---------- ---------- ---------- ---------- -
Rattus_norvegicus            ---------- ---------- ---------- ---------- ---------- ---------- ---------- -
Tursiops_truncatus           ---------- ---------- ---------- ---------- ---------- ---------- ---------- -
Procavia_capensis            ---------- ---------- ---------- ---------- ---------- ---------- ---------- -
Primates                     ---------- ---------- ---------- ---------- ---------- ---------- ---------- -
Echinops_telfairi            ---------- ---------- ---------- ---------- ---------- ---------- ---------- -
Sorex_araneus                ---------- ---------- ---------- ---------- ---------- ---------- ---------- -
Canidae                      ---------- ---------- ---------- ---------- ---------- ---------- ---------- -
Otolemur_garnettii           ---------- ---------- ---------- ---------- ---------- ---------- ---------- -
Gallus                       ---------- ---------- ---------- ---------- ---------- ---------- ---------- -
Callithrix_jacchus           ---------- ---------- ---------- ---------- ---------- ---------- ---------- -
Crocodylus_porosus           ---------- ---------- ---------- ---------- ---------- ---------- ---------- -
Erinaceus_europaeus          ---------- ---------- ---------- ---------- ---------- ---------- ---------- -
rnd-6_family-7426-LTR-ERV1   ---------- ---------- ---------- ---------- ---------- ---------- ---------- -
Esox_lucius                  ---------- ---------- ---------- ---------- ---------- ---------- ---------- -
Salmo_salar                  ---------- ---------- ---------- ---------- ---------- ---------- ---------- -
Didelphidae                  ---------- ---------- ---------- ---------- ---------- ---------- ---------- -
Latimeria_chalumnae          ---------- ---------- ---------- ---------- ---------- ---------- ---------- -
Monodelphis_domestica        ---------- ---------- ---------- ---------- ---------- ---------- ---------- -
Macropus_eugenii             ---------- ---------- ---------- ---------- ---------- ---------- ---------- -
Alligator_mississippiensis   ---------- ---------- ---------- ---------- ---------- ---------- ---------- -
Chrysemys_picta_bellii       ---------- ---------- ---------- ---------- ---------- ---------- ---------- -
Danio_rerio                  ---------- ---------- ---------- ---------- ---------- ---------- ---------- -
Taeniopygia                  ---------- ---------- ---------- ---------- ---------- ---------- ---------- -
Dasypus_novemcinctus         ---------- ---------- ---------- ---------- ---------- ---------- ---------- -
LTR_XT                       ---------- ---------- ---------- ---------- ---------- ---------- ---------- -
Haplorrhini                  GCTGTGACCT GGCCAAGCAG CATCCGAACC TCCCGTAATA GGACCCGGTC TGGTGAATCC GAGAGTGAAA 838
Catarrhini                   GGTGTAAACT GTAAAAGT-G TGTGTGTGCC CTTTTTACCC GTTCTTTGTT TTGTGG-TGT GCGTGTGGT- 519
Anolis_carolinensis          ---------- ---------- ---------- ---------- ---------- ---------- ---------- -
Murine_leukemia_virus        GNTTNN-CNN NNANACCGNG NNNNNCNNCN NGNCTNNNTN ANTNNTNNNN NNTCANNTNN CTNNTCGNTN 473
Phascolarctos_cinereus       GTCTACGCCG CCATATAATT TTTTTCCTTT TTATTTCAAT AATAATCATT ATCTGGGTCG TAATTCCACT 440
Estrildidae                  ---------- ---------- ---------- ---------- ---------- ---------- ---------- -
Xenopus_Silurana_tropicalis  ---------- ---------- ---------- ---------- ---------- ---------- ---------- -
I_XT                         TANTGNAANT AATGNATNNC TNTNACTGTG ANATCNTAAT GAAACATATT NTAGNATNAG NGNNGCANAT 319
Takifugu_rubripes            ---------- ---------- ---------- ---------- ---------- ---------- ---------- -
Bos_taurus                   ---------- ---------- ---------- ---------- ---------- ---------- ---------- -
Homo_sapiens                 ---------- ---------- ---------- ---------- ---------- ---------- ---------- -
Microcebus_murinus           ---------- ---------- ---------- ---------- ---------- ---------- ---------- -
Myotis_lucifugus             ---------- ---------- ---------- ---------- ---------- ---------- ---------- -
Equus_caballus               ---------- ---------- ---------- ---------- ---------- ---------- ---------- -
Tupaia_belangeri             ---------- ---------- ---------- ---------- ---------- ---------- ---------- -
Loxodonta_africana           ---------- ---------- ---------- ---------- ---------- ---------- ---------- -
Murinae                      ---------- ---------- ---------- ---------- ---------- ---------- ---------- -
Canis_lupus_familiaris       ---------- ---------- ---------- ---------- ---------- ---------- ---------- -
Sus_scrofa                   ---------- ---------- ---------- ---------- ---------- ---------- ---------- -
Choloepus_hoffmanni          ---------- ---------- ---------- ---------- ---------- ---------- ---------- -
Vicugna_pacos                ---------- ---------- ---------- ---------- ---------- ---------- ---------- -
Gavialis_gangeticus          ---------- ---------- ---------- ---------- ---------- ---------- ---------- -
Mammalia                     ---------- ---------- ---------- ---------- ---------- ---------- ---------- -
Passeroidea                  ---------- ---------- ---------- ---------- ---------- ---------- ---------- -
Takifugu                     ---------- ---------- ---------- ---------- ---------- ---------- ---------- -
Pteropus_vampyrus            ---------- ---------- ---------- ---------- ---------- ---------- ---------- -
Ornithorhynchus              ---------- ---------- ---------- ---------- ---------- ---------- ---------- -
Oryctolagus_cuniculus        ---------- ---------- ---------- ---------- ---------- ---------- ---------- -
Gallus_gallus                ---------- ---------- ---------- ---------- ---------- ---------- ---------- -
Hominidae                    ---------- ---------- ---------- ---------- ---------- ---------- ---------- -
Metatheria                   ---------- ---------- ---------- ---------- ---------- ---------- ---------- -
Rodentia                     ---------- ---------- ---------- ---------- ---------- ---------- ---------- -
Monodelphis                  ---------- ---------- ---------- ---------- ---------- ---------- ---------- -
Consensus                    ---------- ---------- ---------- ---------- ---------- ---------- ----------
                   100%
Conservation
                     0%
```

	860		880		900			
Dipodomys_ordii	----------	----------	----------	----------	----------	----------	----------	-
Laurasiatheria	----------	----------	----------	----------	----------	----------	----------	-
Eutheria	----------	----------	----------	----------	----------	----------	----------	-
Tarsius_syrichta	----------	----------	----------	----------	----------	----------	----------	-
Muridae	----------	----------	----------	----------	----------	----------	----------	-
Gasterosteus_aculeatus	----------	----------	----------	----------	----------	----------	----------	-
Felis_silvestris	----------	----------	----------	----------	----------	----------	----------	-
Rattus	----------	----------	----------	----------	----------	----------	----------	-
Papio_hamadryas	----------	----------	----------	----------	----------	----------	----------	-
Pan_troglodytes	----------	----------	----------	----------	----------	----------	----------	-
Ailuropoda_melanoleuca	----------	----------	----------	----------	----------	----------	----------	-
Cavia_porcellus	----------	----------	----------	----------	----------	----------	----------	-
Ictidomys_tridecemlineatus	----------	----------	----------	----------	----------	----------	----------	-
Canis	----------	----------	----------	----------	----------	----------	----------	-
Felis_catus	----------	----------	----------	----------	----------	----------	----------	-
Ochotona_princeps	----------	----------	----------	----------	----------	----------	----------	-
Cercopithecidae	----------	----------	----------	----------	----------	----------	----------	-
Carnivora	----------	----------	----------	----------	----------	----------	----------	-
Mus	----------	----------	----------	----------	----------	----------	----------	-
Mus_musculus	----------	----------	----------	----------	----------	----------	----------	-
Rattus_norvegicus	----------	----------	----------	----------	----------	----------	----------	-
Tursiops_truncatus	----------	----------	----------	----------	----------	----------	----------	-
Procavia_capensis	----------	----------	----------	----------	----------	----------	----------	-
Primates	----------	----------	----------	----------	----------	----------	----------	-
Echinops_telfairi	----------	----------	----------	----------	----------	----------	----------	-
Sorex_araneus	----------	----------	----------	----------	----------	----------	----------	-
Canidae	----------	----------	----------	----------	----------	----------	----------	-
Otolemur_garnettii	----------	----------	----------	----------	----------	----------	----------	-
Gallus	----------	----------	----------	----------	----------	----------	----------	-
Callithrix_jacchus	----------	----------	----------	----------	----------	----------	----------	-
Crocodylus_porosus	----------	----------	----------	----------	----------	----------	----------	-
Erinaceus_europaeus	----------	----------	----------	----------	----------	----------	----------	-
rnd-6_family-7426-LTR-ERV1	----------	----------	----------	----------	----------	----------	----------	-
Esox_lucius	----------	----------	----------	----------	----------	----------	----------	-
Salmo_salar	----------	----------	----------	----------	----------	----------	----------	-
Didelphidae	----------	----------	----------	----------	----------	----------	----------	-
Latimeria_chalumnae	----------	----------	----------	----------	----------	----------	----------	-
Monodelphis_domestica	----------	----------	----------	----------	----------	----------	----------	-
Macropus_eugenii	----------	----------	----------	----------	----------	----------	----------	-
Alligator_mississippiensis	----------	----------	----------	----------	----------	----------	----------	-
Chrysemys_picta_bellii	----------	----------	----------	----------	----------	----------	----------	-
Danio_rerio	----------	----------	----------	----------	----------	----------	----------	-
Taeniopygia	----------	----------	----------	----------	----------	----------	----------	-
Dasypus_novemcinctus	----------	----------	----------	----------	----------	----------	----------	-
LTR_XT	----------	----------	----------	----------	----------	----------	----------	-
Haplorrhini	GTGAGAGTGA	AAGTGCGCCG	CGAGGGAGGA	AATGGGAGGA	AAAGCATCGA	AGCCAACTCC	ATTAGAATGT	908
Catarrhini	GTGAGCGTGG	TGTTTTGTCT	CGAGGAAACA	CGGGTCAGGC	ACAAAGT--A	AGCCCACCCC	ACTAGGAACT	587
Anolis_carolinensis	----------	----------	----------	----------	----------	----------	----------	-
Murine_leukemia_virus	NTGTNNNNGA	CCGNTTNTNN	NNNNNNNGNN	CNNNCCNTAT	ANNANNNNTN	NGANNNNNAC	NCTNGNACAC	543
Phascolarctos_cinereus	CT--TCGGGA	CCCCCAGAAT	GGGACAGGGT	GAGTC-----	--GACCCCTC	TCTCTCTTAC	ACTAGATCAC	501
Estrildidae	----------	----------	----------	----------	----------	----------	----------	-
Xenopus_Silurana_tropicalis	----------	----------	----------	----------	----------	----------	----------	-
I_XT	AGANGCNAGG	GTTANANTGA	TGTTAATGTT	AAAGATNNTN	TNTGGNGGCA	NAAAAGNTNT	CTCAGNNNTT	389
Takifugu_rubripes	----------	----------	----------	----------	----------	----------	----------	-
Bos_taurus	----------	----------	----------	----------	----------	----------	----------	-
Homo_sapiens	----------	----------	----------	----------	----------	----------	----------	-
Microcebus_murinus	----------	----------	----------	----------	----------	----------	----------	-
Myotis_lucifugus	----------	----------	----------	----------	----------	----------	----------	-
Equus_caballus	----------	----------	----------	----------	----------	----------	----------	-
Tupaia_belangeri	----------	----------	----------	----------	----------	----------	----------	-
Loxodonta_africana	----------	----------	----------	----------	----------	----------	----------	-
Murinae	----------	----------	----------	----------	----------	----------	----------	-
Canis_lupus_familiaris	----------	----------	----------	----------	----------	----------	----------	-
Sus_scrofa	----------	----------	----------	----------	----------	----------	----------	-
Choloepus_hoffmanni	----------	----------	----------	----------	----------	----------	----------	-
Vicugna_pacos	----------	----------	----------	----------	----------	----------	----------	-
Gavialis_gangeticus	----------	----------	----------	----------	----------	----------	----------	-
Mammalia	----------	----------	----------	----------	----------	----------	----------	-
Passeroidea	----------	----------	----------	----------	----------	----------	----------	-
Takifugu	----------	----------	----------	----------	----------	----------	----------	-
Pteropus_vampyrus	----------	----------	----------	----------	----------	----------	----------	-
Ornithorhynchus	----------	----------	----------	----------	----------	----------	----------	-
Oryctolagus_cuniculus	----------	----------	----------	----------	----------	----------	----------	-
Gallus_gallus	----------	----------	----------	----------	----------	----------	----------	-
Hominidae	----------	----------	----------	----------	----------	----------	----------	-
Metatheria	----------	----------	----------	----------	----------	----------	----------	-
Rodentia	----------	----------	----------	----------	----------	----------	----------	-
Monodelphis	----------	----------	----------	----------	----------	----------	----------	-
Consensus	----------	----------	----------	----------	----------	----------	----------	

Conservation 100% 0%

	920 / 940 / 960 / 980	
Dipodomys_ordii	---------- ---------- ---------- ---------- ---------- ---------- ----------	-
Laurasiatheria	---------- ---------- ---------- ---------- ---------- ---------- ----------	-
Eutheria	---------- ---------- ---------- ---------- ---------- ---------- ----------	-
Tarsius_syrichta	---------- ---------- ---------- ---------- ---------- ---------- ----------	-
Muridae	---------- ---------- ---------- ---------- ---------- ---------- ----------	-
Gasterosteus_aculeatus	---------- ---------- ---------- ---------- ---------- ---------- ----------	-
Felis_silvestris	---------- ---------- ---------- ---------- ---------- ---------- ----------	-
Rattus	---------- ---------- ---------- ---------- ---------- ---------- ----------	-
Papio_hamadryas	---------- ---------- ---------- ---------- ---------- ---------- ----------	-
Pan_troglodytes	---------- ---------- ---------- ---------- ---------- ---------- ----------	-
Ailuropoda_melanoleuca	---------- ---------- ---------- ---------- ---------- ---------- ----------	-
Cavia_porcellus	---------- ---------- ---------- ---------- ---------- ---------- ----------	-
Ictidomys_tridecemlineatus	---------- ---------- ---------- ---------- ---------- ---------- ----------	-
Canis	---------- ---------- ---------- ---------- ---------- ---------- ----------	-
Felis_catus	---------- ---------- ---------- ---------- ---------- ---------- ----------	-
Ochotona_princeps	---------- ---------- ---------- ---------- ---------- ---------- ----------	-
Cercopithecidae	---------- ---------- ---------- ---------- ---------- ---------- ----------	-
Carnivora	---------- ---------- ---------- ---------- ---------- ---------- ----------	-
Mus	---------- ---------- ---------- ---------- ---------- ---------- ----------	-
Mus_musculus	---------- ---------- ---------- ---------- ---------- ---------- ----------	-
Rattus_norvegicus	---------- ---------- ---------- ---------- ---------- ---------- ----------	-
Tursiops_truncatus	---------- ---------- ---------- ---------- ---------- ---------- ----------	-
Procavia_capensis	---------- ---------- ---------- ---------- ---------- ---------- ----------	-
Primates	---------- ---------- ---------- ---------- ---------- ---------- ----------	-
Echinops_telfairi	---------- ---------- ---------- ---------- ---------- ---------- ----------	-
Sorex_araneus	---------- ---------- ---------- ---------- ---------- ---------- ----------	-
Canidae	---------- ---------- ---------- ---------- ---------- ---------- ----------	-
Otolemur_garnettii	---------- ---------- ---------- ---------- ---------- ---------- ----------	-
Gallus	---------- ---------- ---------- ---------- ---------- ---------- ----------	-
Callithrix_jacchus	---------- ---------- ---------- ---------- ---------- ---------- ----------	-
Crocodylus_porosus	---------- ---------- ---------- ---------- ---------- ---------- ----------	-
Erinaceus_europaeus	---------- ---------- ---------- ---------- ---------- ---------- ----------	-
rnd-6_family-7426-LTR-ERV1	---------- ---------- ---------- ---------- ---------- ---------- ----------	-
Esox_lucius	---------- ---------- ---------- ---------- ---------- ---------- ----------	-
Salmo_salar	---------- ---------- ---------- ---------- ---------- ---------- ----------	-
Didelphidae	---------- ---------- ---------- ---------- ---------- ---------- ----------	-
Latimeria_chalumnae	---------- ---------- ---------- ---------- ---------- ---------- ----------	-
Monodelphis_domestica	---------- ---------- ---------- ---------- ---------- ---------- ----------	-
Macropus_eugenii	---------- ---------- ---------- ---------- ---------- ---------- ----------	-
Alligator_mississippiensis	---------- ---------- ---------- ---------- ---------- ---------- ----------	-
Chrysemys_picta_bellii	---------- ---------- ---------- ---------- ---------- ---------- ----------	-
Danio_rerio	---------- ---------- ---------- ---------- ---------- ---------- ----------	-
Taeniopygia	---------- ---------- ---------- ---------- ---------- ---------- ----------	-
Dasypus_novemcinctus	---------- ---------- ---------- ---------- ---------- ---------- ----------	-
LTR_XT	---------- ---------- ---------- ---------- ---------- ---------- ----------	-
Haplorrhini	ATGTTGAAGA ACTTTAAGAA AGGCTTTGAT GGTGATTATG GAATGAAGTT AACACCGCAG AGGCTAAGAA	978
Catarrhini	ATGTTGAAAA ATTTCAAAAA GGGATTTAAG GGAGACTATG GAGTCACCAT GACACCAGGA AAACTTAGAA	657
Anolis_carolinensis	---------- ---------- ---------- ---------- ---------- ---------- ----------	-
Murine_leukemia_virus	---NNGNNAG NCNTCCNNNN NANTGNGTCN NNCNNGTNCN NGNNNNNCNA NNANAGNCNN TNGCT-GTNN	609
Phascolarctos_cinereus	---TGGAAAG ACGTGAAGAC AAGGGCTCAC AATCTTTCCG TGGAGATAAG AAAGGGAAAG TGGC---AAA	565
Estrildidae	---------- ---------- ---------- ---------- ---------- ---------- ----------	-
Xenopus_Silurana_tropicalis	---------- ---------- ---------- ---------- ---------- ---------- ----------	-
I_XT	NAANGNATCA ANGNCNGGNT CAGNCAGANN GNGAAGANNN AGNACNNNNN GTNGANGANG AGTCNGATCA	459
Takifugu_rubripes	---------- ---------- ---------- ---------- ---------- ---------- ----------	-
Bos_taurus	---------- ---------- ---------- ---------- ---------- ---------- ----------	-
Homo_sapiens	---------- ---------- ---------- ---------- ---------- ---------- ----------	-
Microcebus_murinus	---------- ---------- ---------- ---------- ---------- ---------- ----------	-
Myotis_lucifugus	---------- ---------- ---------- ---------- ---------- ---------- ----------	-
Equus_caballus	---------- ---------- ---------- ---------- ---------- ---------- ----------	-
Tupaia_belangeri	---------- ---------- ---------- ---------- ---------- ---------- ----------	-
Loxodonta_africana	---------- ---------- ---------- ---------- ---------- ---------- ----------	-
Murinae	---------- ---------- ---------- ---------- ---------- ---------- ----------	-
Canis_lupus_familiaris	---------- ---------- ---------- ---------- ---------- ---------- ----------	-
Sus_scrofa	---------- ---------- ---------- ---------- ---------- ---------- ----------	-
Choloepus_hoffmanni	---------- ---------- ---------- ---------- ---------- ---------- ----------	-
Vicugna_pacos	---------- ---------- ---------- ---------- ---------- ---------- ----------	-
Gavialis_gangeticus	---------- ---------- ---------- ---------- ---------- ---------- ----------	-
Mammalia	---------- ---------- ---------- ---------- ---------- ---------- ----------	-
Passeroidea	---------- ---------- ---------- ---------- ---------- ---------- ----------	-
Takifugu	---------- ---------- ---------- ---------- ---------- ---------- ----------	-
Pteropus_vampyrus	---------- ---------- ---------- ---------- ---------- ---------- ----------	-
Ornithorhynchus	---------- ---------- ---------- ---------- ---------- ---------- ----------	-
Oryctolagus_cuniculus	---------- ---------- ---------- ---------- ---------- ---------- ----------	-
Gallus_gallus	---------- ---------- ---------- ---------- ---------- ---------- ----------	-
Hominidae	---------- ---------- ---------- ---------- ---------- ---------- ----------	-
Metatheria	---------- ---------- ---------- ---------- ---------- ---------- ----------	-
Rodentia	---------- ---------- ---------- ---------- ---------- ---------- ----------	-
Monodelphis	---------- ---------- ---------- ---------- ---------- ---------- ----------	-
Consensus	---------- ---------- ---------- ---------- ---------- ---------- ----------	

Conservation 100% 0%

		1,000		1,020		1,040		
Dipodomys_ordii	----------	----------	----------	----------	----------	----------	----------	-
Laurasiatheria	----------	----------	----------	----------	----------	----------	----------	-
Eutheria	----------	----------	----------	----------	----------	----------	----------	-
Tarsius_syrichta	----------	----------	----------	----------	----------	----------	----------	-
Muridae	----------	----------	----------	----------	----------	----------	----------	-
Gasterosteus_aculeatus	----------	----------	----------	----------	----------	----------	----------	-
Felis_silvestris	----------	----------	----------	----------	----------	----------	----------	-
Rattus	----------	----------	----------	----------	----------	----------	----------	-
Papio_hamadryas	----------	----------	----------	----------	----------	----------	----------	-
Pan_troglodytes	----------	----------	----------	----------	----------	----------	----------	-
Ailuropoda_melanoleuca	----------	----------	----------	----------	----------	----------	----------	-
Cavia_porcellus	----------	----------	----------	----------	----------	----------	----------	-
Ictidomys_tridecemlineatus	----------	----------	----------	----------	----------	----------	----------	-
Canis	----------	----------	----------	----------	----------	----------	----------	-
Felis_catus	----------	----------	----------	----------	----------	----------	----------	-
Ochotona_princeps	----------	----------	----------	----------	----------	----------	----------	-
Cercopithecidae	----------	----------	----------	----------	----------	----------	----------	-
Carnivora	----------	----------	----------	----------	----------	----------	----------	-
Mus	----------	----------	----------	----------	----------	----------	----------	-
Mus_musculus	----------	----------	----------	----------	----------	----------	----------	-
Rattus_norvegicus	----------	----------	----------	----------	----------	----------	----------	-
Tursiops_truncatus	----------	----------	----------	----------	----------	----------	----------	-
Procavia_capensis	----------	----------	----------	----------	----------	----------	----------	-
Primates	----------	----------	----------	----------	----------	----------	----------	-
Echinops_telfairi	----------	----------	----------	----------	----------	----------	----------	-
Sorex_araneus	----------	----------	----------	----------	----------	----------	----------	-
Canidae	----------	----------	----------	----------	----------	----------	----------	-
Otolemur_garnettii	----------	----------	----------	----------	----------	----------	----------	-
Gallus	----------	----------	----------	----------	----------	----------	----------	-
Callithrix_jacchus	----------	----------	----------	----------	----------	----------	----------	-
Crocodylus_porosus	----------	----------	----------	----------	----------	----------	----------	-
Erinaceus_europaeus	----------	----------	----------	----------	----------	----------	----------	-
rnd-6_family-7426-LTR-ERV1	----------	----------	----------	----------	----------	----------	----------	-
Esox_lucius	----------	----------	----------	----------	----------	----------	----------	-
Salmo_salar	----------	----------	----------	----------	----------	----------	----------	-
Didelphidae	----------	----------	----------	----------	----------	----------	----------	-
Latimeria_chalumnae	----------	----------	----------	----------	----------	----------	----------	-
Monodelphis_domestica	----------	----------	----------	----------	----------	----------	----------	-
Macropus_eugenii	----------	----------	----------	----------	----------	----------	----------	-
Alligator_mississippiensis	----------	----------	----------	----------	----------	----------	----------	-
Chrysemys_picta_bellii	----------	----------	----------	----------	----------	----------	----------	-
Danio_rerio	----------	----------	----------	----------	----------	----------	----------	-
Taeniopygia	----------	----------	----------	----------	----------	----------	----------	-
Dasypus_novemcinctus	----------	----------	----------	----------	----------	----------	----------	-
LTR_XT	----------	----------	----------	----------	----------	----------	----------	-
Haplorrhini	CNCTTTNTGA	AATTGACTGG	CCCTCCTTTA	ATGTAGGGTG	GNCGGCCGAG	GG--AACCAT	AGATAGGGAA	1046
Catarrhini	CTTTGTGTGA	GATAGACTGG	CCAGCATTAG	AGGTGGGTTG	GCCATCAGAA	GG--AAGCCT	GGACAGGTCC	725
Anolis_carolinensis	----------	----------	----------	----------	----------	----------	----------	-
Murine_leukemia_virus	NCNTCNGNNN	NGNNGNNTNG	CNNANNNTNG	ATGNNGGGTN	NCCNCANNNT	GNTTGACTNN	NNANNTNGNN	679
Phascolarctos_cinereus	CCTTCTGTTC	CTCCGAGTGG	CCCACGTTCG	AAGTGGGATG	GCCACCGGAG	GG--GACTTT	TAATCCTTCT	633
Estrildidae	----------	----------	----------	----------	----------	----------	----------	-
Xenopus_Silurana_tropicalis	----------	----------	----------	----------	----------	----------	----------	-
I_XT	GNANAATGGG	AATGNANAGT	TNTTTNACAA	ANCGCNAGGN	ANAANTNNAA	GATTCANGAT	CAACNGATGA	529
Takifugu_rubripes	----------	----------	----------	----------	----------	----------	----------	-
Bos_taurus	----------	----------	----------	----------	----------	----------	----------	-
Homo_sapiens	----------	----------	----------	----------	----------	----------	----------	-
Microcebus_murinus	----------	----------	----------	----------	----------	----------	----------	-
Myotis_lucifugus	----------	----------	----------	----------	----------	----------	----------	-
Equus_caballus	----------	----------	----------	----------	----------	----------	----------	-
Tupaia_belangeri	----------	----------	----------	----------	----------	----------	----------	-
Loxodonta_africana	----------	----------	----------	----------	----------	----------	----------	-
Murinae	----------	----------	----------	----------	----------	----------	----------	-
Canis_lupus_familiaris	----------	----------	----------	----------	----------	----------	----------	-
Sus_scrofa	----------	----------	----------	----------	----------	----------	----------	-
Choloepus_hoffmanni	----------	----------	----------	----------	----------	----------	----------	-
Vicugna_pacos	----------	----------	----------	----------	----------	----------	----------	-
Gavialis_gangeticus	----------	----------	----------	----------	----------	----------	----------	-
Mammalia	----------	----------	----------	----------	----------	----------	----------	-
Passeroidea	----------	----------	----------	----------	----------	----------	----------	-
Takifugu	----------	----------	----------	----------	----------	----------	----------	-
Pteropus_vampyrus	----------	----------	----------	----------	----------	----------	----------	-
Ornithorhynchus	----------	----------	----------	----------	----------	----------	----------	-
Oryctolagus_cuniculus	----------	----------	----------	----------	----------	----------	----------	-
Gallus_gallus	----------	----------	----------	----------	----------	----------	----------	-
Hominidae	----------	----------	----------	----------	----------	----------	----------	-
Metatheria	----------	----------	----------	----------	----------	----------	----------	-
Rodentia	----------	----------	----------	----------	----------	----------	----------	-
Monodelphis	----------	----------	----------	----------	----------	----------	----------	-
Consensus	----------	----------	----------	----------	----------	----------	----------	

Conservation (100% – 0%)

1,060 1,080 1,100 1,120

Dipodomys_ordii	----------	----------	----------	----------	----------	----------	----------	-
Laurasiatheria	----------	----------	----------	----------	----------	----------	----------	-
Eutheria	----------	----------	----------	----------	----------	----------	----------	-
Tarsius_syrichta	----------	----------	----------	----------	----------	----------	----------	-
Muridae	----------	----------	----------	----------	----------	----------	----------	-
Gasterosteus_aculeatus	----------	----------	----------	----------	----------	----------	----------	-
Felis_silvestris	----------	----------	----------	----------	----------	----------	----------	-
Rattus	----------	----------	----------	----------	----------	----------	----------	-
Papio_hamadryas	----------	----------	----------	----------	----------	----------	----------	-
Pan_troglodytes	----------	----------	----------	----------	----------	----------	----------	-
Ailuropoda_melanoleuca	----------	----------	----------	----------	----------	----------	----------	-
Cavia_porcellus	----------	----------	----------	----------	----------	----------	----------	-
Ictidomys_tridecemlineatus	----------	----------	----------	----------	----------	----------	----------	-
Canis	----------	----------	----------	----------	----------	----------	----------	-
Felis_catus	----------	----------	----------	----------	----------	----------	----------	-
Ochotona_princeps	----------	----------	----------	----------	----------	----------	----------	-
Cercopithecidae	----------	----------	----------	----------	----------	----------	----------	-
Carnivora	----------	----------	----------	----------	----------	----------	----------	-
Mus	----------	----------	----------	----------	----------	----------	----------	-
Mus_musculus	----------	----------	----------	----------	----------	----------	----------	-
Rattus_norvegicus	----------	----------	----------	----------	----------	----------	----------	-
Tursiops_truncatus	----------	----------	----------	----------	----------	----------	----------	-
Procavia_capensis	----------	----------	----------	----------	----------	----------	----------	-
Primates	----------	----------	----------	----------	----------	----------	----------	-
Echinops_telfairi	----------	----------	----------	----------	----------	----------	----------	-
Sorex_araneus	----------	----------	----------	----------	----------	----------	----------	-
Canidae	----------	----------	----------	----------	----------	----------	----------	-
Otolemur_garnettii	----------	----------	----------	----------	----------	----------	----------	-
Gallus	----------	----------	----------	----------	----------	----------	----------	-
Callithrix_jacchus	----------	----------	----------	----------	----------	----------	----------	-
Crocodylus_porosus	----------	----------	----------	----------	----------	----------	----------	-
Erinaceus_europaeus	----------	----------	----------	----------	----------	----------	----------	-
rnd-6_family-7426-LTR-ERV1	----------	----------	----------	----------	----------	----------	----------	-
Esox_lucius	----------	----------	----------	----------	----------	----------	----------	-
Salmo_salar	----------	----------	----------	----------	----------	----------	----------	-
Didelphidae	----------	----------	----------	----------	----------	----------	----------	-
Latimeria_chalumnae	----------	----------	----------	----------	----------	----------	----------	-
Monodelphis_domestica	----------	----------	----------	----------	----------	----------	----------	-
Macropus_eugenii	----------	----------	----------	----------	----------	----------	----------	-
Alligator_mississippiensis	----------	----------	----------	----------	----------	----------	----------	-
Chrysemys_picta_bellii	----------	----------	----------	----------	----------	----------	----------	-
Danio_rerio	----------	----------	----------	----------	----------	----------	----------	-
Taeniopygia	----------	----------	----------	----------	----------	----------	----------	-
Dasypus_novemcinctus	----------	----------	----------	----------	----------	----------	----------	-
LTR_XT	----------	----------	----------	----------	----------	----------	----------	-
Haplorrhini	ATAATTGGCC	GAGTGTTTCG	GGTGGTCACT	GGGGTCGGAG	AACAGCCTGG	GCACCCTGAT	CAGTTCCCAT	1116
Catarrhini	CTTGTCTCGA	AGGTATGGCA	CAGGGTAACC	TGTA------	---AGCCAGG	GCACCCGGAT	CAGTTCCCAT	786
Anolis_carolinensis	----------	----------	----------	----------	----------	----------	----------	-
Murine_leukemia_virus	NNTNTTTNAC	AGGTTAAATC	TAAGGTGTTC	TCTCCCGG--	-TCCCCACGG	ACACCCGGAT	CAGGTCCCAT	746
Phascolarctos_cinereus	ATTATTTCTG	CAGTCAAA--	-AGGATTGTC	TTCCAGGA--	-GACTGGAGG	ACACCCGGAC	CAGGTTCCCT	697
Estrildidae	----------	----------	----------	----------	----------	----------	----------	-
Xenopus_Silurana_tropicalis	----------	----------	----------	----------	----------	----------	----------	-
I_XT	AAATNGTGAA	AGCTNTGCCN	NNANATAACN	TTTNCNANAN	ACATTTGTTA	ATNTTNCCAT	NCNNGGAGCA	599
Takifugu_rubripes	----------	----------	----------	----------	----------	----------	----------	-
Bos_taurus	----------	----------	----------	----------	----------	----------	----------	-
Homo_sapiens	----------	----------	----------	----------	----------	----------	----------	-
Microcebus_murinus	----------	----------	----------	----------	----------	----------	----------	-
Myotis_lucifugus	----------	----------	----------	----------	----------	----------	----------	-
Equus_caballus	----------	----------	----------	----------	----------	----------	----------	-
Tupaia_belangeri	----------	----------	----------	----------	----------	----------	----------	-
Loxodonta_africana	----------	----------	----------	----------	----------	----------	----------	-
Murinae	----------	----------	----------	----------	----------	----------	----------	-
Canis_lupus_familiaris	----------	----------	----------	----------	----------	----------	----------	-
Sus_scrofa	----------	----------	----------	----------	----------	----------	----------	-
Choloepus_hoffmanni	----------	----------	----------	----------	----------	----------	----------	-
Vicugna_pacos	----------	----------	----------	----------	----------	----------	----------	-
Gavialis_gangeticus	----------	----------	----------	----------	----------	----------	----------	-
Mammalia	----------	----------	----------	----------	----------	----------	----------	-
Passeroidea	----------	----------	----------	----------	----------	----------	----------	-
Takifugu	----------	----------	----------	----------	----------	----------	----------	-
Pteropus_vampyrus	----------	----------	----------	----------	----------	----------	----------	-
Ornithorhynchus	----------	----------	----------	----------	----------	----------	----------	-
Oryctolagus_cuniculus	----------	----------	----------	----------	----------	----------	----------	-
Gallus_gallus	----------	----------	----------	----------	----------	----------	----------	-
Hominidae	----------	----------	----------	----------	----------	----------	----------	-
Metatheria	----------	----------	----------	----------	----------	----------	----------	-
Rodentia	----------	----------	----------	----------	----------	----------	----------	-
Monodelphis	----------	----------	----------	----------	----------	----------	----------	-
Consensus	----------	----------	----------	----------	----------	----------	----------	

Conservation 100% 0%

		1,140		1,160		1,180		
Dipodomys_ordii	----------	----------	----------	----------	----------	----------	----------	-
Laurasiatheria	----------	----------	----------	----------	----------	----------	----------	-
Eutheria	----------	----------	----------	----------	----------	----------	----------	-
Tarsius_syrichta	----------	----------	----------	----------	----------	----------	----------	-
Muridae	----------	----------	----------	----------	----------	----------	----------	-
Gasterosteus_aculeatus	----------	----------	----------	----------	----------	----------	----------	-
Felis_silvestris	----------	----------	----------	----------	----------	----------	----------	-
Rattus	----------	----------	----------	----------	----------	----------	----------	-
Papio_hamadryas	----------	----------	----------	----------	----------	----------	----------	-
Pan_troglodytes	----------	----------	----------	----------	----------	----------	----------	-
Ailuropoda_melanoleuca	----------	----------	----------	----------	----------	----------	----------	-
Cavia_porcellus	----------	----------	----------	----------	----------	----------	----------	-
Ictidomys_tridecemlineatus	----------	----------	----------	----------	----------	----------	----------	-
Canis	----------	----------	----------	----------	----------	----------	----------	-
Felis_catus	----------	----------	----------	----------	----------	----------	----------	-
Ochotona_princeps	----------	----------	----------	----------	----------	----------	----------	-
Cercopithecidae	----------	----------	----------	----------	----------	----------	----------	-
Carnivora	----------	----------	----------	----------	----------	----------	----------	-
Mus	----------	----------	----------	----------	----------	----------	----------	-
Mus_musculus	----------	----------	----------	----------	----------	----------	----------	-
Rattus_norvegicus	----------	----------	----------	----------	----------	----------	----------	-
Tursiops_truncatus	----------	----------	----------	----------	----------	----------	----------	-
Procavia_capensis	----------	----------	----------	----------	----------	----------	----------	-
Primates	----------	----------	----------	----------	----------	----------	----------	-
Echinops_telfairi	----------	----------	----------	----------	----------	----------	----------	-
Sorex_araneus	----------	----------	----------	----------	----------	----------	----------	-
Canidae	----------	----------	----------	----------	----------	----------	----------	-
Otolemur_garnettii	----------	----------	----------	----------	----------	----------	----------	-
Gallus	----------	----------	----------	----------	----------	----------	----------	-
Callithrix_jacchus	----------	----------	----------	----------	----------	----------	----------	-
Crocodylus_porosus	----------	----------	----------	----------	----------	----------	----------	-
Erinaceus_europaeus	----------	----------	----------	----------	----------	----------	----------	-
rnd-6_family-7426-LTR-ERV1	----------	----------	----------	----------	----------	----------	----------	-
Esox_lucius	----------	----------	----------	----------	----------	----------	----------	-
Salmo_salar	----------	----------	----------	----------	----------	----------	----------	-
Didelphidae	----------	----------	----------	----------	----------	----------	----------	-
Latimeria_chalumnae	----------	----------	----------	----------	----------	----------	----------	-
Monodelphis_domestica	----------	----------	----------	----------	----------	----------	----------	-
Macropus_eugenii	----------	----------	----------	----------	----------	----------	----------	-
Alligator_mississippiensis	----------	----------	----------	----------	----------	----------	----------	-
Chrysemys_picta_bellii	----------	----------	----------	----------	----------	----------	----------	-
Danio_rerio	----------	----------	----------	----------	----------	----------	----------	-
Taeniopygia	----------	----------	----------	----------	----------	----------	----------	-
Dasypus_novemcinctus	----------	----------	----------	----------	----------	----------	----------	-
LTR_XT	----------	----------	----------	----------	----------	----------	----------	-
Haplorrhini	ACATAGACTC	CTGGCTGAGC	GTGATTCAGA	CCCATCCGAA	ATGGCTACAG	GCCTGCTTTG	AAGATTATTG	1186
Catarrhini	ATATAGATTC	TTGGTTACAG	CTAGTTTTGG	ACCCCCCACA	GTGGTTAAGA	GG-------A	CAGGCAGCAG	849
Anolis_carolinensis	----------	----------	----------	----------	----------	----------	----------	-
Murine_leukemia_virus	ACATTGTCAC	CTGGGAGGCT	ATTGCCTATG	ANCCCCCTCC	GTGGGTCAAA	CC------TT	TTGTCTCTCC	810
Phascolarctos_cinereus	ACATCATAGT	TTGGCAGGAC	CTCTCCAACA	GCCCCCCCCC	ATGGGTGCCA	CC------CT	TAG------C	755
Estrildidae	----------	----------	----------	----------	----------	----------	----------	-
Xenopus_Silurana_tropicalis	----------	----------	----------	----------	----------	----------	----------	-
I_XT	AATNTNAACA	GATATNCTGN	GTCTGATCNA	CCAACCAATA	NGGTANTGAA	ANATNGACAC	CCCACNCNNN	669
Takifugu_rubripes	----------	----------	----------	----------	----------	----------	----------	-
Bos_taurus	----------	----------	----------	----------	----------	----------	----------	-
Homo_sapiens	----------	----------	----------	----------	----------	----------	----------	-
Microcebus_murinus	----------	----------	----------	----------	----------	----------	----------	-
Myotis_lucifugus	----------	----------	----------	----------	----------	----------	----------	-
Equus_caballus	----------	----------	----------	----------	----------	----------	----------	-
Tupaia_belangeri	----------	----------	----------	----------	----------	----------	----------	-
Loxodonta_africana	----------	----------	----------	----------	----------	----------	----------	-
Murinae	----------	----------	----------	----------	----------	----------	----------	-
Canis_lupus_familiaris	----------	----------	----------	----------	----------	----------	----------	-
Sus_scrofa	----------	----------	----------	----------	----------	----------	----------	-
Choloepus_hoffmanni	----------	----------	----------	----------	----------	----------	----------	-
Vicugna_pacos	----------	----------	----------	----------	----------	----------	----------	-
Gavialis_gangeticus	----------	----------	----------	----------	----------	----------	----------	-
Mammalia	----------	----------	----------	----------	----------	----------	----------	-
Passeroidea	----------	----------	----------	----------	----------	----------	----------	-
Takifugu	----------	----------	----------	----------	----------	----------	----------	-
Pteropus_vampyrus	----------	----------	----------	----------	----------	----------	----------	-
Ornithorhynchus	----------	----------	----------	----------	----------	----------	----------	-
Oryctolagus_cuniculus	----------	----------	----------	----------	----------	----------	----------	-
Gallus_gallus	----------	----------	----------	----------	----------	----------	----------	-
Hominidae	----------	----------	----------	----------	----------	----------	----------	-
Metatheria	----------	----------	----------	----------	----------	----------	----------	-
Rodentia	----------	----------	----------	----------	----------	----------	----------	-
Monodelphis	----------	----------	----------	----------	----------	----------	----------	-
Consensus	----------	----------	----------	----------	----------	----------	----------	

Conservation 100% 0%

	1,200		1,220		1,240		1,260	
Dipodomys_ordii	----------	----------	----------	----------	----------	----------	----------	-
Laurasiatheria	----------	----------	----------	----------	----------	----------	----------	-
Eutheria	----------	----------	----------	----------	----------	----------	----------	-
Tarsius_syrichta	----------	----------	----------	----------	----------	----------	----------	-
Muridae	----------	----------	----------	----------	----------	----------	----------	-
Gasterosteus_aculeatus	----------	----------	----------	----------	----------	----------	----------	-
Felis_silvestris	----------	----------	----------	----------	----------	----------	----------	-
Rattus	----------	----------	----------	----------	----------	----------	----------	-
Papio_hamadryas	----------	----------	----------	----------	----------	----------	----------	-
Pan_troglodytes	----------	----------	----------	----------	----------	----------	----------	-
Ailuropoda_melanoleuca	----------	----------	----------	----------	----------	----------	----------	-
Cavia_porcellus	----------	----------	----------	----------	----------	----------	----------	-
Ictidomys_tridecemlineatus	----------	----------	----------	----------	----------	----------	----------	-
Canis	----------	----------	----------	----------	----------	----------	----------	-
Felis_catus	----------	----------	----------	----------	----------	----------	----------	-
Ochotona_princeps	----------	----------	----------	----------	----------	----------	----------	-
Cercopithecidae	----------	----------	----------	----------	----------	----------	----------	-
Carnivora	----------	----------	----------	----------	----------	----------	----------	-
Mus	----------	----------	----------	----------	----------	----------	----------	-
Mus_musculus	----------	----------	----------	----------	----------	----------	----------	-
Rattus_norvegicus	----------	----------	----------	----------	----------	----------	----------	-
Tursiops_truncatus	----------	----------	----------	----------	----------	----------	----------	-
Procavia_capensis	----------	----------	----------	----------	----------	----------	----------	-
Primates	----------	----------	----------	----------	----------	----------	----------	-
Echinops_telfairi	----------	----------	----------	----------	----------	----------	----------	-
Sorex_araneus	----------	----------	----------	----------	----------	----------	----------	-
Canidae	----------	----------	----------	----------	----------	----------	----------	-
Otolemur_garnettii	----------	----------	----------	----------	----------	----------	----------	-
Gallus	----------	----------	----------	----------	----------	----------	----------	-
Callithrix_jacchus	----------	----------	----------	----------	----------	----------	----------	-
Crocodylus_porosus	----------	----------	----------	----------	----------	----------	----------	-
Erinaceus_europaeus	----------	----------	----------	----------	----------	----------	----------	-
rnd-6_family-7426-LTR-ERV1	----------	----------	----------	----------	----------	----------	----------	-
Esox_lucius	----------	----------	----------	----------	----------	----------	----------	-
Salmo_salar	----------	----------	----------	----------	----------	----------	----------	-
Didelphidae	----------	----------	----------	----------	----------	----------	----------	-
Latimeria_chalumnae	----------	----------	----------	----------	----------	----------	----------	-
Monodelphis_domestica	----------	----------	----------	----------	----------	----------	----------	-
Macropus_eugenii	----------	----------	----------	----------	----------	----------	----------	-
Alligator_mississippiensis	----------	----------	----------	----------	----------	----------	----------	-
Chrysemys_picta_bellii	----------	----------	----------	----------	----------	----------	----------	-
Danio_rerio	----------	----------	----------	----------	----------	----------	----------	-
Taeniopygia	----------	----------	----------	----------	----------	----------	----------	-
Dasypus_novemcinctus	----------	----------	----------	----------	----------	----------	----------	-
LTR_XT	----------	----------	----------	----------	----------	----------	----------	-
Haplorrhini	TAAGACTCTA	GTGGCTCGTA	CAAAACAAGG	AACCATAGAA	AAGACCCACA	AAGCGCAGNC	CCAGGAGAAA	1256
Catarrhini	CAGTACT--A	GTAGCA---A	AGGGACAGTT	AGT--TAAGG	AAGGTTCTCN	CTCCACCCGC	CGAGGGAAGT	912
Anolis_carolinensis	----------	----------	----------	----------	----------	----------	----------	-
Murine_leukemia_virus	CAAACTCTCC	CCCTCTCCGA	CCGCTCCCGT	CCTC-CCGTC	CGGTCCTTCG	ACCCAACCTC	CGCCCCGATC	879
Phascolarctos_cinereus	CAAGATCGCC	GTTGCCTC--	-TGGTCAAGA	TAAC-GGGCG	AAAGTCGGCG	GGGGGGAGGC	CGTCCGCTCC	821
Estrildidae	----------	----------	----------	----------	----------	----------	----------	-
Xenopus_Silurana_tropicalis	----------	----------	----------	----------	----------	----------	----------	-
I_XT	TGTNTAGANG	NCTAAGCNCT	CCNNTNAGTG	NGCNCANACN	NANTGGATTG	GGAGATGNNC	NACANGGTAC	739
Takifugu_rubripes	----------	----------	----------	----------	----------	----------	----------	-
Bos_taurus	----------	----------	----------	----------	----------	----------	----------	-
Homo_sapiens	----------	----------	----------	----------	----------	----------	----------	-
Microcebus_murinus	----------	----------	----------	----------	----------	----------	----------	-
Myotis_lucifugus	----------	----------	----------	----------	----------	----------	----------	-
Equus_caballus	----------	----------	----------	----------	----------	----------	----------	-
Tupaia_belangeri	----------	----------	----------	----------	----------	----------	----------	-
Loxodonta_africana	----------	----------	----------	----------	----------	----------	----------	-
Murinae	----------	----------	----------	----------	----------	----------	----------	-
Canis_lupus_familiaris	----------	----------	----------	----------	----------	----------	----------	-
Sus_scrofa	----------	----------	----------	----------	----------	----------	----------	-
Choloepus_hoffmanni	----------	----------	----------	----------	----------	----------	----------	-
Vicugna_pacos	----------	----------	----------	----------	----------	----------	----------	-
Gavialis_gangeticus	----------	----------	----------	----------	----------	----------	----------	-
Mammalia	----------	----------	----------	----------	----------	----------	----------	-
Passeroidea	----------	----------	----------	----------	----------	----------	----------	-
Takifugu	----------	----------	----------	----------	----------	----------	----------	-
Pteropus_vampyrus	----------	----------	----------	----------	----------	----------	----------	-
Ornithorhynchus	----------	----------	----------	----------	----------	----------	----------	-
Oryctolagus_cuniculus	----------	----------	----------	----------	----------	----------	----------	-
Gallus_gallus	----------	----------	----------	----------	----------	----------	----------	-
Hominidae	----------	----------	----------	----------	----------	----------	----------	-
Metatheria	----------	----------	----------	----------	----------	----------	----------	-
Rodentia	----------	----------	----------	----------	----------	----------	----------	-
Monodelphis	----------	----------	----------	----------	----------	----------	----------	-
Consensus	----------	----------	----------	----------	----------	----------	----------	

Conservation

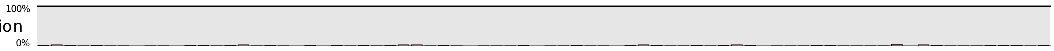

```
                                 1,280                        1,300                        1,320
Dipodomys_ordii                ---------- ---------- ---------- ---------- ---------- ---------- ---------- -
Laurasiatheria                 ---------- ---------- ---------- ---------- ---------- ---------- ---------- -
Eutheria                       ---------- ---------- ---------- ---------- ---------- ---------- ---------- -
Tarsius_syrichta               ---------- ---------- ---------- ---------- ---------- ---------- ---------- -
Muridae                        ---------- ---------- ---------- ---------- ---------- ---------- ---------- -
Gasterosteus_aculeatus         ---------- ---------- ---------- ---------- ---------- ---------- ---------- -
Felis_silvestris               ---------- ---------- ---------- ---------- ---------- ---------- ---------- -
Rattus                         ---------- ---------- ---------- ---------- ---------- ---------- ---------- -
Papio_hamadryas                ---------- ---------- ---------- ---------- ---------- ---------- ---------- -
Pan_troglodytes                ---------- ---------- ---------- ---------- ---------- ---------- ---------- -
Ailuropoda_melanoleuca         ---------- ---------- ---------- ---------- ---------- ---------- ---------- -
Cavia_porcellus                ---------- ---------- ---------- ---------- ---------- ---------- ---------- -
Ictidomys_tridecemlineatus     ---------- ---------- ---------- ---------- ---------- ---------- ---------- -
Canis                          ---------- ---------- ---------- ---------- ---------- ---------- ---------- -
Felis_catus                    ---------- ---------- ---------- ---------- ---------- ---------- ---------- -
Ochotona_princeps              ---------- ---------- ---------- ---------- ---------- ---------- ---------- -
Cercopithecidae                ---------- ---------- ---------- ---------- ---------- ---------- ---------- -
Carnivora                      ---------- ---------- ---------- ---------- ---------- ---------- ---------- -
Mus                            ---------- ---------- ---------- ---------- ---------- ---------- ---------- -
Mus_musculus                   ---------- ---------- ---------- ---------- ---------- ---------- ---------- -
Rattus_norvegicus              ---------- ---------- ---------- ---------- ---------- ---------- ---------- -
Tursiops_truncatus             ---------- ---------- ---------- ---------- ---------- ---------- ---------- -
Procavia_capensis              ---------- ---------- ---------- ---------- ---------- ---------- ---------- -
Primates                       ---------- ---------- ---------- ---------- ---------- ---------- ---------- -
Echinops_telfairi              ---------- ---------- ---------- ---------- ---------- ---------- ---------- -
Sorex_araneus                  ---------- ---------- ---------- ---------- ---------- ---------- ---------- -
Canidae                        ---------- ---------- ---------- ---------- ---------- ---------- ---------- -
Otolemur_garnettii             ---------- ---------- ---------- ---------- ---------- ---------- ---------- -
Gallus                         ---------- ---------- ---------- ---------- ---------- ---------- ---------- -
Callithrix_jacchus             ---------- ---------- ---------- ---------- ---------- ---------- ---------- -
Crocodylus_porosus             ---------- ---------- ---------- ---------- ---------- ---------- ---------- -
Erinaceus_europaeus            ---------- ---------- ---------- ---------- ---------- ---------- ---------- -
rnd-6_family-7426-LTR-ERV1     ---------- ---------- ---------- ---------- ---------- ---------- ---------- -
Esox_lucius                    ---------- ---------- ---------- ---------- ---------- ---------- ---------- -
Salmo_salar                    ---------- ---------- ---------- ---------- ---------- ---------- ---------- -
Didelphidae                    ---------- ---------- ---------- ---------- ---------- ---------- ---------- -
Latimeria_chalumnae            ---------- ---------- ---------- ---------- ---------- ---------- ---------- -
Monodelphis_domestica          ---------- ---------- ---------- ---------- ---------- ---------- ---------- -
Macropus_eugenii               ---------- ---------- ---------- ---------- ---------- ---------- ---------- -
Alligator_mississippiensis     ---------- ---------- ---------- ---------- ---------- ---------- ---------- -
Chrysemys_picta_bellii         ---------- ---------- ---------- ---------- ---------- ---------- ---------- -
Danio_rerio                    ---------- ---------- ---------- ---------- ---------- ---------- ---------- -
Taeniopygia                    ---------- ---------- ---------- ---------- ---------- ---------- ---------- -
Dasypus_novemcinctus           ---------- ---------- ---------- ---------- ---------- ---------- ---------- -
LTR_XT                         ---------- ---------- ---------- ---------- ---------- ---------- ---------- -
Haplorrhini                    GAGCAGCAAG GAAAACAGGA AAAACCTGTC CTACAGGCCC CGCCAGAAGA GCTAGAGACT CCACCCCCTT 1326
Catarrhini                     CGGCACCAA- ---------- AAGTCCTGTC CGACC--CAA CACCAGAAGA ATCATGG--- -CAGGAATTG 965
Anolis_carolinensis            ---------- ---------- ---------- ---------- ---------- ---------- ---------- -
Murine_leukemia_virus          TGCCCTTTA- ---------- ---CCCTGCT CTTAC--CCC CTCTATAAAA CCCAGAC--- ------CTTC 924
Phascolarctos_cinereus         TTCCCGGCT- ---------- ---CCCCA-T CTACC--CGG AGACGGACAG CCTGTTC--- ------CTCC 865
Estrildidae                    ---------- ---------- ---------- ---------- ---------- ---------- ---------- -
Xenopus_Silurana_tropicalis    ---------- ---------- ---------- ---------- ---------- ---------- ---------- -
I_XT                           CATTANNANT TNACTCCANC AGANNTTGCA TNTTAATNCT GGGGAAAAAN TNTNAGNTNN NGAGGTANTA 809
Takifugu_rubripes              ---------- ---------- ---------- ---------- ---------- ---------- ---------- -
Bos_taurus                     ---------- ---------- ---------- ---------- ---------- ---------- ---------- -
Homo_sapiens                   ---------- ---------- ---------- ---------- ---------- ---------- ---------- -
Microcebus_murinus             ---------- ---------- ---------- ---------- ---------- ---------- ---------- -
Myotis_lucifugus               ---------- ---------- ---------- ---------- ---------- ---------- ---------- -
Equus_caballus                 ---------- ---------- ---------- ---------- ---------- ---------- ---------- -
Tupaia_belangeri               ---------- ---------- ---------- ---------- ---------- ---------- ---------- -
Loxodonta_africana             ---------- ---------- ---------- ---------- ---------- ---------- ---------- -
Murinae                        ---------- ---------- ---------- ---------- ---------- ---------- ---------- -
Canis_lupus_familiaris         ---------- ---------- ---------- ---------- ---------- ---------- ---------- -
Sus_scrofa                     ---------- ---------- ---------- ---------- ---------- ---------- ---------- -
Choloepus_hoffmanni            ---------- ---------- ---------- ---------- ---------- ---------- ---------- -
Vicugna_pacos                  ---------- ---------- ---------- ---------- ---------- ---------- ---------- -
Gavialis_gangeticus            ---------- ---------- ---------- ---------- ---------- ---------- ---------- -
Mammalia                       ---------- ---------- ---------- ---------- ---------- ---------- ---------- -
Passeroidea                    ---------- ---------- ---------- ---------- ---------- ---------- ---------- -
Takifugu                       ---------- ---------- ---------- ---------- ---------- ---------- ---------- -
Pteropus_vampyrus              ---------- ---------- ---------- ---------- ---------- ---------- ---------- -
Ornithorhynchus                ---------- ---------- ---------- ---------- ---------- ---------- ---------- -
Oryctolagus_cuniculus          ---------- ---------- ---------- ---------- ---------- ---------- ---------- -
Gallus_gallus                  ---------- ---------- ---------- ---------- ---------- ---------- ---------- -
Hominidae                      ---------- ---------- ---------- ---------- ---------- ---------- ---------- -
Metatheria                     ---------- ---------- ---------- ---------- ---------- ---------- ---------- -
Rodentia                       ---------- ---------- ---------- ---------- ---------- ---------- ---------- -
Monodelphis                    ---------- ---------- ---------- ---------- ---------- ---------- ---------- -
Consensus                      ---------- ---------- ---------- ---------- ---------- ---------- ----------
```

Conservation

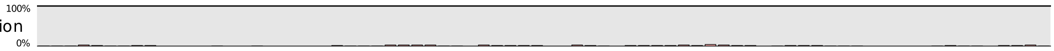

1,340 1,360 1,380 1,400

Dipodomys_ordii	----------	----------	----------	----------	----------	----------	----------	-
Laurasiatheria	----------	----------	----------	----------	----------	----------	----------	-
Eutheria	----------	----------	----------	----------	----------	----------	----------	-
Tarsius_syrichta	----------	----------	----------	----------	----------	----------	----------	-
Muridae	----------	----------	----------	----------	----------	----------	----------	-
Gasterosteus_aculeatus	----------	----------	----------	----------	----------	----------	----------	-
Felis_silvestris	----------	----------	----------	----------	----------	----------	----------	-
Rattus	----------	----------	----------	----------	----------	----------	----------	-
Papio_hamadryas	----------	----------	----------	----------	----------	----------	----------	-
Pan_troglodytes	----------	----------	----------	----------	----------	----------	----------	-
Ailuropoda_melanoleuca	----------	----------	----------	----------	----------	----------	----------	-
Cavia_porcellus	----------	----------	----------	----------	----------	----------	----------	-
Ictidomys_tridecemlineatus	----------	----------	----------	----------	----------	----------	----------	-
Canis	----------	----------	----------	----------	----------	----------	----------	-
Felis_catus	----------	----------	----------	----------	----------	----------	----------	-
Ochotona_princeps	----------	----------	----------	----------	----------	----------	----------	-
Cercopithecidae	----------	----------	----------	----------	----------	----------	----------	-
Carnivora	----------	----------	----------	----------	----------	----------	----------	-
Mus	----------	----------	----------	----------	----------	----------	----------	-
Mus_musculus	----------	----------	----------	----------	----------	----------	----------	-
Rattus_norvegicus	----------	----------	----------	----------	----------	----------	----------	-
Tursiops_truncatus	----------	----------	----------	----------	----------	----------	----------	-
Procavia_capensis	----------	----------	----------	----------	----------	----------	----------	-
Primates	----------	----------	----------	----------	----------	----------	----------	-
Echinops_telfairi	----------	----------	----------	----------	----------	----------	----------	-
Sorex_araneus	----------	----------	----------	----------	----------	----------	----------	-
Canidae	----------	----------	----------	----------	----------	----------	----------	-
Otolemur_garnettii	----------	----------	----------	----------	----------	----------	----------	-
Gallus	----------	----------	----------	----------	----------	----------	----------	-
Callithrix_jacchus	----------	----------	----------	----------	----------	----------	----------	-
Crocodylus_porosus	----------	----------	----------	----------	----------	----------	----------	-
Erinaceus_europaeus	----------	----------	----------	----------	----------	----------	----------	-
rnd-6_family-7426-LTR-ERV1	----------	----------	----------	----------	----------	----------	----------	-
Esox_lucius	----------	----------	----------	----------	----------	----------	----------	-
Salmo_salar	----------	----------	----------	----------	----------	----------	----------	-
Didelphidae	----------	----------	----------	----------	----------	----------	----------	-
Latimeria_chalumnae	----------	----------	----------	----------	----------	----------	----------	-
Monodelphis_domestica	----------	----------	----------	----------	----------	----------	----------	-
Macropus_eugenii	----------	----------	----------	----------	----------	----------	----------	-
Alligator_mississippiensis	----------	----------	----------	----------	----------	----------	----------	-
Chrysemys_picta_bellii	----------	----------	----------	----------	----------	----------	----------	-
Danio_rerio	----------	----------	----------	----------	----------	----------	----------	-
Taeniopygia	----------	----------	----------	----------	----------	----------	----------	-
Dasypus_novemcinctus	----------	----------	----------	----------	----------	----------	----------	-
LTR_XT	----------	----------	----------	----------	----------	----------	----------	-
Haplorrhini	ATGTTCCAAT	TTACCCATCT	CTGGCAAGGC	TTAGGCAGGA	AGCCACTCCA	GCAGCTGCCT	CCGGAGGGTC	1396
Catarrhini	GTACCAGCAG	TACCCCCTCC	TTATCGAGAG	GAAGG--GCT	CCCCACCCCT	GAGCCCACAG	CACCTACACC	1033
Anolis_carolinensis	----------	----------	----------	----------	----------	----------	----------	-
Murine_leukemia_virus	TAAGCCCCAG	GTTCTCCCCG	ATAGCGGC--	-------GGA	CCTCTCATTG	ACCTTCTCAC	AGAGGACCCC	985
Phascolarctos_cinereus	T----CTCAG	AACCCCCGCC	CTATCCA---	---------A	CGTCCCCTCC	ACCTCCCC-C	AG-----CCC	913
Estrildidae	----------	----------	----------	----------	----------	----------	----------	-
Xenopus_Silurana_tropicalis	----------	----------	----------	----------	----------	----------	----------	-
I_XT	AANACCNTGN	NAATCANNNC	ANGAANATCG	TTATNCNTNN	NTNNNTNTAN	TNGAACTTNC	TTNTAGAANG	879
Takifugu_rubripes	----------	----------	----------	----------	----------	----------	----------	-
Bos_taurus	----------	----------	----------	----------	----------	----------	----------	-
Homo_sapiens	----------	----------	----------	----------	----------	----------	----------	-
Microcebus_murinus	----------	----------	----------	----------	----------	----------	----------	-
Myotis_lucifugus	----------	----------	----------	----------	----------	----------	----------	-
Equus_caballus	----------	----------	----------	----------	----------	----------	----------	-
Tupaia_belangeri	----------	----------	----------	----------	----------	----------	----------	-
Loxodonta_africana	----------	----------	----------	----------	----------	----------	----------	-
Murinae	----------	----------	----------	----------	----------	----------	----------	-
Canis_lupus_familiaris	----------	----------	----------	----------	----------	----------	----------	-
Sus_scrofa	----------	----------	----------	----------	----------	----------	----------	-
Choloepus_hoffmanni	----------	----------	----------	----------	----------	----------	----------	-
Vicugna_pacos	----------	----------	----------	----------	----------	----------	----------	-
Gavialis_gangeticus	----------	----------	----------	----------	----------	----------	----------	-
Mammalia	----------	----------	----------	----------	----------	----------	----------	-
Passeroidea	----------	----------	----------	----------	----------	----------	----------	-
Takifugu	----------	----------	----------	----------	----------	----------	----------	-
Pteropus_vampyrus	----------	----------	----------	----------	----------	----------	----------	-
Ornithorhynchus	----------	----------	----------	----------	----------	----------	----------	-
Oryctolagus_cuniculus	----------	----------	----------	----------	----------	----------	----------	-
Gallus_gallus	----------	----------	----------	----------	----------	----------	----------	-
Hominidae	----------	----------	----------	----------	----------	----------	----------	-
Metatheria	----------	----------	----------	----------	----------	----------	----------	-
Rodentia	----------	----------	----------	----------	----------	----------	----------	-
Monodelphis	----------	----------	----------	----------	----------	----------	----------	-
Consensus	----------	----------	----------	----------	----------	----------	----------	

Conservation 100% 0%

```
                               1,420                1,440                1,460
Dipodomys_ordii             ---------- ---------- ---------- ---------- ---------- ---------- ---------- -
Laurasiatheria              ---------- ---------- ---------- ---------- ---------- ---------- ---------- -
Eutheria                    ---------- ---------- ---------- ---------- ---------- ---------- ---------- -
Tarsius_syrichta            ---------- ---------- ---------- ---------- ---------- ---------- ---------- -
Muridae                     ---------- ---------- ---------- ---------- ---------- ---------- ---------- -
Gasterosteus_aculeatus      ---------- ---------- ---------- ---------- ---------- ---------- ---------- -
Felis_silvestris            ---------- ---------- ---------- ---------- ---------- ---------- ---------- -
Rattus                      ---------- ---------- ---------- ---------- ---------- ---------- ---------- -
Papio_hamadryas             ---------- ---------- ---------- ---------- ---------- ---------- ---------- -
Pan_troglodytes             ---------- ---------- ---------- ---------- ---------- ---------- ---------- -
Ailuropoda_melanoleuca      ---------- ---------- ---------- ---------- ---------- ---------- ---------- -
Cavia_porcellus             ---------- ---------- ---------- ---------- ---------- ---------- ---------- -
Ictidomys_tridecemlineatus  ---------- ---------- ---------- ---------- ---------- ---------- ---------- -
Canis                       ---------- ---------- ---------- ---------- ---------- ---------- ---------- -
Felis_catus                 ---------- ---------- ---------- ---------- ---------- ---------- ---------- -
Ochotona_princeps           ---------- ---------- ---------- ---------- ---------- ---------- ---------- -
Cercopithecidae             ---------- ---------- ---------- ---------- ---------- ---------- ---------- -
Carnivora                   ---------- ---------- ---------- ---------- ---------- ---------- ---------- -
Mus                         ---------- ---------- ---------- ---------- ---------- ---------- ---------- -
Mus_musculus                ---------- ---------- ---------- ---------- ---------- ---------- ---------- -
Rattus_norvegicus           ---------- ---------- ---------- ---------- ---------- ---------- ---------- -
Tursiops_truncatus          ---------- ---------- ---------- ---------- ---------- ---------- ---------- -
Procavia_capensis           ---------- ---------- ---------- ---------- ---------- ---------- ---------- -
Primates                    ---------- ---------- ---------- ---------- ---------- ---------- ---------- -
Echinops_telfairi           ---------- ---------- ---------- ---------- ---------- ---------- ---------- -
Sorex_araneus               ---------- ---------- ---------- ---------- ---------- ---------- ---------- -
Canidae                     ---------- ---------- ---------- ---------- ---------- ---------- ---------- -
Otolemur_garnettii          ---------- ---------- ---------- ---------- ---------- ---------- ---------- -
Gallus                      ---------- ---------- ---------- ---------- ---------- ---------- ---------- -
Callithrix_jacchus          ---------- ---------- ---------- ---------- ---------- ---------- ---------- -
Crocodylus_porosus          ---------- ---------- ---------- ---------- ---------- ---------- ---------- -
Erinaceus_europaeus         ---------- ---------- ---------- ---------- ---------- ---------- ---------- -
rnd-6_family-7426-LTR-ERV1  ---------- ---------- ---------- ---------- ---------- ---------- ---------- -
Esox_lucius                 ---------- ---------- ---------- ---------- ---------- ---------- ---------- -
Salmo_salar                 ---------- ---------- ---------- ---------- ---------- ---------- ---------- -
Didelphidae                 ---------- ---------- ---------- ---------- ---------- ---------- ---------- -
Latimeria_chalumnae         ---------- ---------- ---------- ---------- ---------- ---------- ---------- -
Monodelphis_domestica       ---------- ---------- ---------- ---------- ---------- ---------- ---------- -
Macropus_eugenii            ---------- ---------- ---------- ---------- ---------- ---------- ---------- -
Alligator_mississippiensis  ---------- ---------- ---------- ---------- ---------- ---------- ---------- -
Chrysemys_picta_bellii      ---------- ---------- ---------- ---------- ---------- ---------- ---------- -
Danio_rerio                 ---------- ---------- ---------- ---------- ---------- ---------- ---------- -
Taeniopygia                 ---------- ---------- ---------- ---------- ---------- ---------- ---------- -
Dasypus_novemcinctus        ---------- ---------- ---------- ---------- ---------- ---------- ---------- -
LTR_XT                      ---------- ---------- ---------- ---------- ---------- ---------- ---------- -
Haplorrhini                 GGACTCAGAG GAGAGTACCC CCCAAACTTC ACCACGTAGG GAAGAGCCAG GGCCACTGCC CGATAAATCA 1466
Catarrhini                  TCCACCAGAT AACCACACCC CT-AGACCAC CCAGAGTAGA CAAAAG--AG GA--AGTG-- ----AAGCCG 1092
Anolis_carolinensis         ---------- ---------- ---------- ---------- ---------- ---------- ---------- -
Murine_leukemia_virus       CCGCCGTACG GAGNACAGGG ACCNTCCTCC T--CTGNCGG AGANGGCGAC GGAGAAGA-- -----GGCCG 1046
Phascolarctos_cinereus      CTCACGCGGC CAGACCAGCG CCAGGCCTAA TGGCTGAGGG ACTCGGCTCT GAGGGACC-- -----GGCCG 976
Estrildidae                 ---------- ---------- ---------- ---------- ---------- ---------- ---------- -
Xenopus_Silurana_tropicalis ---------- ---------- ---------- ---------- ---------- ---------- ---------- -
I_XT                        GTTANGNGAA NTCNNNATGN GNAAGNNCCN GNANNGATGN ATCTNATGNT NGTAATNGAA NTGNNANCTT 949
Takifugu_rubripes           ---------- ---------- ---------- ---------- ---------- ---------- ---------- -
Bos_taurus                  ---------- ---------- ---------- ---------- ---------- ---------- ---------- -
Homo_sapiens                ---------- ---------- ---------- ---------- ---------- ---------- ---------- -
Microcebus_murinus          ---------- ---------- ---------- ---------- ---------- ---------- ---------- -
Myotis_lucifugus            ---------- ---------- ---------- ---------- ---------- ---------- ---------- -
Equus_caballus              ---------- ---------- ---------- ---------- ---------- ---------- ---------- -
Tupaia_belangeri            ---------- ---------- ---------- ---------- ---------- ---------- ---------- -
Loxodonta_africana          ---------- ---------- ---------- ---------- ---------- ---------- ---------- -
Murinae                     ---------- ---------- ---------- ---------- ---------- ---------- ---------- -
Canis_lupus_familiaris      ---------- ---------- ---------- ---------- ---------- ---------- ---------- -
Sus_scrofa                  ---------- ---------- ---------- ---------- ---------- ---------- ---------- -
Choloepus_hoffmanni         ---------- ---------- ---------- ---------- ---------- ---------- ---------- -
Vicugna_pacos               ---------- ---------- ---------- ---------- ---------- ---------- ---------- -
Gavialis_gangeticus         ---------- ---------- ---------- ---------- ---------- ---------- ---------- -
Mammalia                    ---------- ---------- ---------- ---------- ---------- ---------- ---------- -
Passeroidea                 ---------- ---------- ---------- ---------- ---------- ---------- ---------- -
Takifugu                    ---------- ---------- ---------- ---------- ---------- ---------- ---------- -
Pteropus_vampyrus           ---------- ---------- ---------- ---------- ---------- ---------- ---------- -
Ornithorhynchus             ---------- ---------- ---------- ---------- ---------- ---------- ---------- -
Oryctolagus_cuniculus       ---------- ---------- ---------- ---------- ---------- ---------- ---------- -
Gallus_gallus               ---------- ---------- ---------- ---------- ---------- ---------- ---------- -
Hominidae                   ---------- ---------- ---------- ---------- ---------- ---------- ---------- -
Metatheria                  ---------- ---------- ---------- ---------- ---------- ---------- ---------- -
Rodentia                    ---------- ---------- ---------- ---------- ---------- ---------- ---------- -
Monodelphis                 ---------- ---------- ---------- ---------- ---------- ---------- ---------- -
Consensus                   ---------- ---------- ---------- ---------- ---------- ---------- ----------
```

Conservation

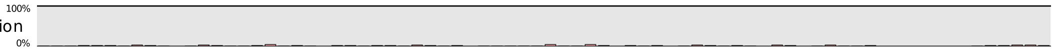

	1,480		1,500		1,520		1,540	
Dipodomys_ordii	----------	----------	----------	----------	----------	----------	----------	-
Laurasiatheria	----------	----------	----------	----------	----------	----------	----------	-
Eutheria	----------	----------	----------	----------	----------	----------	----------	-
Tarsius_syrichta	----------	----------	----------	----------	----------	----------	----------	-
Muridae	----------	----------	----------	----------	----------	----------	----------	-
Gasterosteus_aculeatus	----------	----------	----------	----------	----------	----------	----------	-
Felis_silvestris	----------	----------	----------	----------	----------	----------	----------	-
Rattus	----------	----------	----------	----------	----------	----------	----------	-
Papio_hamadryas	----------	----------	----------	----------	----------	----------	----------	-
Pan_troglodytes	----------	----------	----------	----------	----------	----------	----------	-
Ailuropoda_melanoleuca	----------	----------	----------	----------	----------	----------	----------	-
Cavia_porcellus	----------	----------	----------	----------	----------	----------	----------	-
Ictidomys_tridecemlineatus	----------	----------	----------	----------	----------	----------	----------	-
Canis	----------	----------	----------	----------	----------	----------	----------	-
Felis_catus	----------	----------	----------	----------	----------	----------	----------	-
Ochotona_princeps	----------	----------	----------	----------	----------	----------	----------	-
Cercopithecidae	----------	----------	----------	----------	----------	----------	----------	-
Carnivora	----------	----------	----------	----------	----------	----------	----------	-
Mus	----------	----------	----------	----------	----------	----------	----------	-
Mus_musculus	----------	----------	----------	----------	----------	----------	----------	-
Rattus_norvegicus	----------	----------	----------	----------	----------	----------	----------	-
Tursiops_truncatus	----------	----------	----------	----------	----------	----------	----------	-
Procavia_capensis	----------	----------	----------	----------	----------	----------	----------	-
Primates	----------	----------	----------	----------	----------	----------	----------	-
Echinops_telfairi	----------	----------	----------	----------	----------	----------	----------	-
Sorex_araneus	----------	----------	----------	----------	----------	----------	----------	-
Canidae	----------	----------	----------	----------	----------	----------	----------	-
Otolemur_garnettii	----------	----------	----------	----------	----------	----------	----------	-
Gallus	----------	----------	----------	----------	----------	----------	----------	-
Callithrix_jacchus	----------	----------	----------	----------	----------	----------	----------	-
Crocodylus_porosus	----------	----------	----------	----------	----------	----------	----------	-
Erinaceus_europaeus	----------	----------	----------	----------	----------	----------	----------	-
rnd-6_family-7426-LTR-ERV1	----------	----------	----------	----------	----------	----------	----------	-
Esox_lucius	----------	----------	----------	----------	----------	----------	----------	-
Salmo_salar	----------	----------	----------	----------	----------	----------	----------	-
Didelphidae	----------	----------	----------	----------	----------	----------	----------	-
Latimeria_chalumnae	----------	----------	----------	----------	----------	----------	----------	-
Monodelphis_domestica	----------	----------	----------	----------	----------	----------	----------	-
Macropus_eugenii	----------	----------	----------	----------	----------	----------	----------	-
Alligator_mississippiensis	----------	----------	----------	----------	----------	----------	----------	-
Chrysemys_picta_bellii	----------	----------	----------	----------	----------	----------	----------	-
Danio_rerio	----------	----------	----------	----------	----------	----------	----------	-
Taeniopygia	----------	----------	----------	----------	----------	----------	----------	-
Dasypus_novemcinctus	----------	----------	----------	----------	----------	----------	----------	-
LTR_XT	----------	----------	----------	----------	----------	----------	----------	-
Haplorrhini	AAGGAGGAAC	TCCAGGATGA	GGTTGGCCGT	CTCCGGTCAG	GACGCGCCCG	AGCCATGCAG	ATGCCCCTCA	1536
Catarrhini	CGGGAGAAAC	TCCTCCGTT-	GGCAGCTCGC	TTACGGCC--	--CGAGACTG	GA--ATCCAA	ATGCCCCTGA	1155
Anolis_carolinensis	----------	----------	----------	----------	----------	----------	----------	-
Murine_leukemia_virus	CCNCCACCTC	CGAGGTTCCC	GCCCCCTCTC	CCATGGTGTC	TCGNCTGCGG	GGNAGGAGGG	ACCCCCCCGC	1116
Phascolarctos_cinereus	CTGGAACTA-	-GGAGTCGCC	GTCCC-----	-CGCAGTCCT	ACGGGTG-AC	ACTGGTCCTG	ACTCCACTGT	1037
Estrildidae	----------	----------	----------	----------	----------	----------	----------	-
Xenopus_Silurana_tropicalis	----------	----------	----------	----------	----------	----------	----------	-
I_XT	TTGACNCTGN	NGNGGNTNTA	TTGNCGTNAA	ATNTAATNAN	GNTNGAGNTT	ANNGCANNTT	TCATNNANAN	1019
Takifugu_rubripes	----------	----------	----------	----------	----------	----------	----------	-
Bos_taurus	----------	----------	----------	----------	----------	----------	----------	-
Homo_sapiens	----------	----------	----------	----------	----------	----------	----------	-
Microcebus_murinus	----------	----------	----------	----------	----------	----------	----------	-
Myotis_lucifugus	----------	----------	----------	----------	----------	----------	----------	-
Equus_caballus	----------	----------	----------	----------	----------	----------	----------	-
Tupaia_belangeri	----------	----------	----------	----------	----------	----------	----------	-
Loxodonta_africana	----------	----------	----------	----------	----------	----------	----------	-
Murinae	----------	----------	----------	----------	----------	----------	----------	-
Canis_lupus_familiaris	----------	----------	----------	----------	----------	----------	----------	-
Sus_scrofa	----------	----------	----------	----------	----------	----------	----------	-
Choloepus_hoffmanni	----------	----------	----------	----------	----------	----------	----------	-
Vicugna_pacos	----------	----------	----------	----------	----------	----------	----------	-
Gavialis_gangeticus	----------	----------	----------	----------	----------	----------	----------	-
Mammalia	----------	----------	----------	----------	----------	----------	----------	-
Passeroidea	----------	----------	----------	----------	----------	----------	----------	-
Takifugu	----------	----------	----------	----------	----------	----------	----------	-
Pteropus_vampyrus	----------	----------	----------	----------	----------	----------	----------	-
Ornithorhynchus	----------	----------	----------	----------	----------	----------	----------	-
Oryctolagus_cuniculus	----------	----------	----------	----------	----------	----------	----------	-
Gallus_gallus	----------	----------	----------	----------	----------	----------	----------	-
Hominidae	----------	----------	----------	----------	----------	----------	----------	-
Metatheria	----------	----------	----------	----------	----------	----------	----------	-
Rodentia	----------	----------	----------	----------	----------	----------	----------	-
Monodelphis	----------	----------	----------	----------	----------	----------	----------	-
Consensus	----------	----------	----------	----------	----------	----------	----------	

Conservation

100%

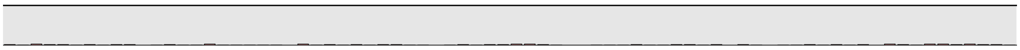

0%

```
                                                       1,560                1,580                1,600
Dipodomys_ordii              ---------- ---------- ---------- ---------- ---------- ---------- ---------- -
Laurasiatheria               ---------- ---------- ---------- ---------- ---------- ---------- ---------- -
Eutheria                     ---------- ---------- ---------- ---------- ---------- ---------- ---------- -
Tarsius_syrichta             ---------- ---------- ---------- ---------- ---------- ---------- ---------- -
Muridae                      ---------- ---------- ---------- ---------- ---------- ---------- ---------- -
Gasterosteus_aculeatus       ---------- ---------- ---------- ---------- ---------- ---------- ---------- -
Felis_silvestris             ---------- ---------- ---------- ---------- ---------- ---------- ---------- -
Rattus                       ---------- ---------- ---------- ---------- ---------- ---------- ---------- -
Papio_hamadryas              ---------- ---------- ---------- ---------- ---------- ---------- ---------- -
Pan_troglodytes              ---------- ---------- ---------- ---------- ---------- ---------- ---------- -
Ailuropoda_melanoleuca       ---------- ---------- ---------- ---------- ---------- ---------- ---------- -
Cavia_porcellus              ---------- ---------- ---------- ---------- ---------- ---------- ---------- -
Ictidomys_tridecemlineatus   ---------- ---------- ---------- ---------- ---------- ---------- ---------- -
Canis                        ---------- ---------- ---------- ---------- ---------- ---------- ---------- -
Felis_catus                  ---------- ---------- ---------- ---------- ---------- ---------- ---------- -
Ochotona_princeps            ---------- ---------- ---------- ---------- ---------- ---------- ---------- -
Cercopithecidae              ---------- ---------- ---------- ---------- ---------- ---------- ---------- -
Carnivora                    ---------- ---------- ---------- ---------- ---------- ---------- ---------- -
Mus                          ---------- ---------- ---------- ---------- ---------- ---------- ---------- -
Mus_musculus                 ---------- ---------- ---------- ---------- ---------- ---------- ---------- -
Rattus_norvegicus            ---------- ---------- ---------- ---------- ---------- ---------- ---------- -
Tursiops_truncatus           ---------- ---------- ---------- ---------- ---------- ---------- ---------- -
Procavia_capensis            ---------- ---------- ---------- ---------- ---------- ---------- ---------- -
Primates                     ---------- ---------- ---------- ---------- ---------- ---------- ---------- -
Echinops_telfairi            ---------- ---------- ---------- ---------- ---------- ---------- ---------- -
Sorex_araneus                ---------- ---------- ---------- ---------- ---------- ---------- ---------- -
Canidae                      ---------- ---------- ---------- ---------- ---------- ---------- ---------- -
Otolemur_garnettii           ---------- ---------- ---------- ---------- ---------- ---------- ---------- -
Gallus                       ---------- ---------- ---------- ---------- ---------- ---------- ---------- -
Callithrix_jacchus           ---------- ---------- ---------- ---------- ---------- ---------- ---------- -
Crocodylus_porosus           ---------- ---------- ---------- ---------- ---------- ---------- ---------- -
Erinaceus_europaeus          ---------- ---------- ---------- ---------- ---------- ---------- ---------- -
rnd-6_family-7426-LTR-ERV1   ---------- ---------- ---------- ---------- ---------- ---------- ---------- -
Esox_lucius                  ---------- ---------- ---------- ---------- ---------- ---------- ---------- -
Salmo_salar                  ---------- ---------- ---------- ---------- ---------- ---------- ---------- -
Didelphidae                  ---------- ---------- ---------- ---------- ---------- ---------- ---------- -
Latimeria_chalumnae          ---------- ---------- ---------- ---------- ---------- ---------- ---------- -
Monodelphis_domestica        ---------- ---------- ---------- ---------- ---------- ---------- ---------- -
Macropus_eugenii             ---------- ---------- ---------- ---------- ---------- ---------- ---------- -
Alligator_mississippiensis   ---------- ---------- ---------- ---------- ---------- ---------- ---------- -
Chrysemys_picta_bellii       ---------- ---------- ---------- ---------- ---------- ---------- ---------- -
Danio_rerio                  ---------- ---------- ---------- ---------- ---------- ---------- ---------- -
Taeniopygia                  ---------- ---------- ---------- ---------- ---------- ---------- ---------- -
Dasypus_novemcinctus         ---------- ---------- ---------- ---------- ---------- ---------- ---------- -
LTR_XT                       ---------- ---------- ---------- ---------- ---------- ---------- ---------- -
Haplorrhini                  GAGAAACTAG AGGACAGATC TATTTAGATG CACAAAATGA GGTTCAAGGA GGAGAA-CGG CTTTATGTTT 1605
Catarrhini                   GAG------- AGCAGCGATA TACTGGGGTA GATGAGGACG GACACATGGT GGAAAGGCGT GCCTTTGTGT 1218
Anolis_carolinensis          ---------- ---------- ---------- ---------- ---------- ---------- ---------- -
Murine_leukemia_virus        GGC-----GG ACTCCACCNC CTCCCGGGCN TTCCCACTCC GTNTGGGGGG NGATGGCCAG CTNCA---GT 1178
Phascolarctos_cinereus       GGC-----CC TGCCCCTCCG GGCCGTGGGA CCCCCGGCCG AACCAAACGG CTTAGTCCCC TTGCA---AT 1099
Estrildidae                  ---------- ---------- ---------- ---------- ---------- ---------- ---------- -
Xenopus_Silurana_tropicalis  ---------- ---------- ---------- ---------- ---------- ---------- ---------- -
I_XT                         ATNACAAGAC TANTAAATGT AAATGATANA GNTAANATAN ATGCANTTAC TAAATTANCA CNTAAANANA 1089
Takifugu_rubripes            ---------- ---------- ---------- ---------- ---------- ---------- ---------- -
Bos_taurus                   ---------- ---------- ---------- ---------- ---------- ---------- ---------- -
Homo_sapiens                 ---------- ---------- ---------- ---------- ---------- ---------- ---------- -
Microcebus_murinus           ---------- ---------- ---------- ---------- ---------- ---------- ---------- -
Myotis_lucifugus             ---------- ---------- ---------- ---------- ---------- ---------- ---------- -
Equus_caballus               ---------- ---------- ---------- ---------- ---------- ---------- ---------- -
Tupaia_belangeri             ---------- ---------- ---------- ---------- ---------- ---------- ---------- -
Loxodonta_africana           ---------- ---------- ---------- ---------- ---------- ---------- ---------- -
Murinae                      ---------- ---------- ---------- ---------- ---------- ---------- ---------- -
Canis_lupus_familiaris       ---------- ---------- ---------- ---------- ---------- ---------- ---------- -
Sus_scrofa                   ---------- ---------- ---------- ---------- ---------- ---------- ---------- -
Choloepus_hoffmanni          ---------- ---------- ---------- ---------- ---------- ---------- ---------- -
Vicugna_pacos                ---------- ---------- ---------- ---------- ---------- ---------- ---------- -
Gavialis_gangeticus          ---------- ---------- ---------- ---------- ---------- ---------- ---------- -
Mammalia                     ---------- ---------- ---------- ---------- ---------- ---------- ---------- -
Passeroidea                  ---------- ---------- ---------- ---------- ---------- ---------- ---------- -
Takifugu                     ---------- ---------- ---------- ---------- ---------- ---------- ---------- -
Pteropus_vampyrus            ---------- ---------- ---------- ---------- ---------- ---------- ---------- -
Ornithorhynchus              ---------- ---------- ---------- ---------- ---------- ---------- ---------- -
Oryctolagus_cuniculus        ---------- ---------- ---------- ---------- ---------- ---------- ---------- -
Gallus_gallus                ---------- ---------- ---------- ---------- ---------- ---------- ---------- -
Hominidae                    ---------- ---------- ---------- ---------- ---------- ---------- ---------- -
Metatheria                   ---------- ---------- ---------- ---------- ---------- ---------- ---------- -
Rodentia                     ---------- ---------- ---------- ---------- ---------- ---------- ---------- -
Monodelphis                  ---------- ---------- ---------- ---------- ---------- ---------- ---------- -
Consensus                    ---------- ---------- ---------- ---------- ---------- ---------- ----------
```

Conservation

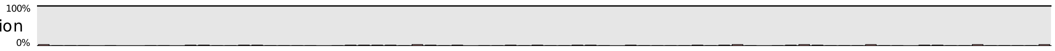

	1,620		1,640		1,660		1,680	
Dipodomys_ordii	----------	----------	----------	----------	----------	----------	----------	-
Laurasiatheria	----------	----------	----------	----------	----------	----------	----------	-
Eutheria	----------	----------	----------	----------	----------	----------	----------	-
Tarsius_syrichta	----------	----------	----------	----------	----------	----------	----------	-
Muridae	----------	----------	----------	----------	----------	----------	----------	-
Gasterosteus_aculeatus	----------	----------	----------	----------	----------	----------	----------	-
Felis_silvestris	----------	----------	----------	----------	----------	----------	----------	-
Rattus	----------	----------	----------	----------	----------	----------	----------	-
Papio_hamadryas	----------	----------	----------	----------	----------	----------	----------	-
Pan_troglodytes	----------	----------	----------	----------	----------	----------	----------	-
Ailuropoda_melanoleuca	----------	----------	----------	----------	----------	----------	----------	-
Cavia_porcellus	----------	----------	----------	----------	----------	----------	----------	-
Ictidomys_tridecemlineatus	----------	----------	----------	----------	----------	----------	----------	-
Canis	----------	----------	----------	----------	----------	----------	----------	-
Felis_catus	----------	----------	----------	----------	----------	----------	----------	-
Ochotona_princeps	----------	----------	----------	----------	----------	----------	----------	-
Cercopithecidae	----------	----------	----------	----------	----------	----------	----------	-
Carnivora	----------	----------	----------	----------	----------	----------	----------	-
Mus	----------	----------	----------	----------	----------	----------	----------	-
Mus_musculus	----------	----------	----------	----------	----------	----------	----------	-
Rattus_norvegicus	----------	----------	----------	----------	----------	----------	----------	-
Tursiops_truncatus	----------	----------	----------	----------	----------	----------	----------	-
Procavia_capensis	----------	----------	----------	----------	----------	----------	----------	-
Primates	----------	----------	----------	----------	----------	----------	----------	-
Echinops_telfairi	----------	----------	----------	----------	----------	----------	----------	-
Sorex_araneus	----------	----------	----------	----------	----------	----------	----------	-
Canidae	----------	----------	----------	----------	----------	----------	----------	-
Otolemur_garnettii	----------	----------	----------	----------	----------	----------	----------	-
Gallus	----------	----------	----------	----------	----------	----------	----------	-
Callithrix_jacchus	----------	----------	----------	----------	----------	----------	----------	-
Crocodylus_porosus	----------	----------	----------	----------	----------	----------	----------	-
Erinaceus_europaeus	----------	----------	----------	----------	----------	----------	----------	-
rnd-6_family-7426-LTR-ERV1	----------	----------	----------	----------	----------	----------	----------	-
Esox_lucius	----------	----------	----------	----------	----------	----------	----------	-
Salmo_salar	----------	----------	----------	----------	----------	----------	----------	-
Didelphidae	----------	----------	----------	----------	----------	----------	----------	-
Latimeria_chalumnae	----------	----------	----------	----------	----------	----------	----------	-
Monodelphis_domestica	----------	----------	----------	----------	----------	----------	----------	-
Macropus_eugenii	----------	----------	----------	----------	----------	----------	----------	-
Alligator_mississippiensis	----------	----------	----------	----------	----------	----------	----------	-
Chrysemys_picta_bellii	----------	----------	----------	----------	----------	----------	----------	-
Danio_rerio	----------	----------	----------	----------	----------	----------	----------	-
Taeniopygia	----------	----------	----------	----------	----------	----------	----------	-
Dasypus_novemcinctus	----------	----------	----------	----------	----------	----------	----------	-
LTR_XT	----------	----------	----------	----------	----------	----------	----------	-
Haplorrhini	ATCAGCCCTT	CTCTACTACA	GACATTTTCA	ACTGGAAGCA	GCATACTCCC	TCCTATACGG	AAAAACCCCA	1675
Catarrhini	ATCAACCTTT	CACCTCTGCT	GACCTCCTCA	ATTGGAAAAA	TAATACTCCA	TCTTACACTG	AAAAGCCTCA	1288
Anolis_carolinensis	----------	----------	----------	----------	----------	----------	----------	-
Murine_leukemia_virus	ACTGGCCGTT	TTCCTCCTCN	GACCTATACA	ACTGGAAAAA	TAATAACCCT	TCCTTTTCTG	AAGATCCAGG	1248
Phascolarctos_cinereus	ATTGGCCTTT	CTCCTCAGCA	GATCTCTATA	ATTGGAAATC	TAATCATCCT	TCTTTTTCCG	AGAATCCTAC	1169
Estrildidae	----------	----------	----------	----------	----------	----------	----------	-
Xenopus_Silurana_tropicalis	----------	----------	----------	----------	----------	----------	----------	-
I_XT	NNTATANNTA	ANCAGNTCAN	AATNAAAGAA	ATNNNAATNC	NNAAGACANG	NCNACTACAN	GNTTTCAAAG	1159
Takifugu_rubripes	----------	----------	----------	----------	----------	----------	----------	-
Bos_taurus	----------	----------	----------	----------	----------	----------	----------	-
Homo_sapiens	----------	----------	----------	----------	----------	----------	----------	-
Microcebus_murinus	----------	----------	----------	----------	----------	----------	----------	-
Myotis_lucifugus	----------	----------	----------	----------	----------	----------	----------	-
Equus_caballus	----------	----------	----------	----------	----------	----------	----------	-
Tupaia_belangeri	----------	----------	----------	----------	----------	----------	----------	-
Loxodonta_africana	----------	----------	----------	----------	----------	----------	----------	-
Murinae	----------	----------	----------	----------	----------	----------	----------	-
Canis_lupus_familiaris	----------	----------	----------	----------	----------	----------	----------	-
Sus_scrofa	----------	----------	----------	----------	----------	----------	----------	-
Choloepus_hoffmanni	----------	----------	----------	----------	----------	----------	----------	-
Vicugna_pacos	----------	----------	----------	----------	----------	----------	----------	-
Gavialis_gangeticus	----------	----------	----------	----------	----------	----------	----------	-
Mammalia	----------	----------	----------	----------	----------	----------	----------	-
Passeroidea	----------	----------	----------	----------	----------	----------	----------	-
Takifugu	----------	----------	----------	----------	----------	----------	----------	-
Pteropus_vampyrus	----------	----------	----------	----------	----------	----------	----------	-
Ornithorhynchus	----------	----------	----------	----------	----------	----------	----------	-
Oryctolagus_cuniculus	----------	----------	----------	----------	----------	----------	----------	-
Gallus_gallus	----------	----------	----------	----------	----------	----------	----------	-
Hominidae	----------	----------	----------	----------	----------	----------	----------	-
Metatheria	----------	----------	----------	----------	----------	----------	----------	-
Rodentia	----------	----------	----------	----------	----------	----------	----------	-
Monodelphis	----------	----------	----------	----------	----------	----------	----------	-
Consensus	----------	----------	----------	----------	----------	----------	----------	

Conservation (100% / 0%)

```
                                                1,700                         1,720                         1,740
Dipodomys_ordii                   ---------- ---------- ---------- ---------- ---------- ---------- ---------- -
Laurasiatheria                    ---------- ---------- ---------- ---------- ---------- ---------- ---------- -
Eutheria                          ---------- ---------- ---------- ---------- ---------- ---------- ---------- -
Tarsius_syrichta                  ---------- ---------- ---------- ---------- ---------- ---------- ---------- -
Muridae                           ---------- ---------- ---------- ---------- ---------- ---------- ---------- -
Gasterosteus_aculeatus            ---------- ---------- ---------- ---------- ---------- ---------- ---------- -
Felis_silvestris                  ---------- ---------- ---------- ---------- ---------- ---------- ---------- -
Rattus                            ---------- ---------- ---------- ---------- ---------- ---------- ---------- -
Papio_hamadryas                   ---------- ---------- ---------- ---------- ---------- ---------- ---------- -
Pan_troglodytes                   ---------- ---------- ---------- ---------- ---------- ---------- ---------- -
Ailuropoda_melanoleuca            ---------- ---------- ---------- ---------- ---------- ---------- ---------- -
Cavia_porcellus                   ---------- ---------- ---------- ---------- ---------- ---------- ---------- -
Ictidomys_tridecemlineatus        ---------- ---------- ---------- ---------- ---------- ---------- ---------- -
Canis                             ---------- ---------- ---------- ---------- ---------- ---------- ---------- -
Felis_catus                       ---------- ---------- ---------- ---------- ---------- ---------- ---------- -
Ochotona_princeps                 ---------- ---------- ---------- ---------- ---------- ---------- ---------- -
Cercopithecidae                   ---------- ---------- ---------- ---------- ---------- ---------- ---------- -
Carnivora                         ---------- ---------- ---------- ---------- ---------- ---------- ---------- -
Mus                               ---------- ---------- ---------- ---------- ---------- ---------- ---------- -
Mus_musculus                      ---------- ---------- ---------- ---------- ---------- ---------- ---------- -
Rattus_norvegicus                 ---------- ---------- ---------- ---------- ---------- ---------- ---------- -
Tursiops_truncatus                ---------- ---------- ---------- ---------- ---------- ---------- ---------- -
Procavia_capensis                 ---------- ---------- ---------- ---------- ---------- ---------- ---------- -
Primates                          ---------- ---------- ---------- ---------- ---------- ---------- ---------- -
Echinops_telfairi                 ---------- ---------- ---------- ---------- ---------- ---------- ---------- -
Sorex_araneus                     ---------- ---------- ---------- ---------- ---------- ---------- ---------- -
Canidae                           ---------- ---------- ---------- ---------- ---------- ---------- ---------- -
Otolemur_garnettii                ---------- ---------- ---------- ---------- ---------- ---------- ---------- -
Gallus                            ---------- ---------- ---------- ---------- ---------- ---------- ---------- -
Callithrix_jacchus                ---------- ---------- ---------- ---------- ---------- ---------- ---------- -
Crocodylus_porosus                ---------- ---------- ---------- ---------- ---------- ---------- ---------- -
Erinaceus_europaeus               ---------- ---------- ---------- ---------- ---------- ---------- ---------- -
rnd-6_family-7426-LTR-ERV1        ---------- ---------- ---------- ---------- ---------- ---------- ---------- -
Esox_lucius                       ---------- ---------- ---------- ---------- ---------- ---------- ---------- -
Salmo_salar                       ---------- ---------- ---------- ---------- ---------- ---------- ---------- -
Didelphidae                       ---------- ---------- ---------- ---------- ---------- ---------- ---------- -
Latimeria_chalumnae               ---------- ---------- ---------- ---------- ---------- ---------- ---------- -
Monodelphis_domestica             ---------- ---------- ---------- ---------- ---------- ---------- ---------- -
Macropus_eugenii                  ---------- ---------- ---------- ---------- ---------- ---------- ---------- -
Alligator_mississippiensis        ---------- ---------- ---------- ---------- ---------- ---------- ---------- -
Chrysemys_picta_bellii            ---------- ---------- ---------- ---------- ---------- ---------- ---------- -
Danio_rerio                       ---------- ---------- ---------- ---------- ---------- ---------- ---------- -
Taeniopygia                       ---------- ---------- ---------- ---------- ---------- ---------- ---------- -
Dasypus_novemcinctus              ---------- ---------- ---------- ---------- ---------- ---------- ---------- -
LTR_XT                            ---------- ---------- ---------- ---------- ---------- ---------- ---------- -
Haplorrhini                       GGCTCTTATC GACCTAATGC AGTCCATCTT CTTAACTCAC AACCCAACCT GGGCTGACTG CAAGCAGCTC 1745
Catarrhini                        AGCTTTAATT GACTTGCTCC AAACTATTAT ACAGACTCAT AATCCTACTT GGGCTGATTG CCACCAGCTG 1358
Anolis_carolinensis               ---------- ---------- ---------- ---------- ---------- ---------- ---------- -
Murine_leukemia_virus             TAAATTGACN GCCTTGATCG AGTCCGTCCT CATCACCCAC CAGCCCACCT GGGACGACTG TCAGCAGTTG 1318
Phascolarctos_cinereus            AGGACTTACG GGACTCCTTG AGTCCCTCAT GTTTTCCCAC CAGCCTACTT GGGACGATTG CCAGCAGCTC 1239
Estrildidae                       ---------- ---------- ---------- ---------- ---------- ---------- ---------- -
Xenopus_Silurana_tropicalis       ---------- ---------- ---------- ---------- ---------- ---------- ---------- -
I_XT                              NNNGANTTGT CAGGGGTNCC ATATAACGGG NCATCNGNAG GAGTNCNGCA TGCGNCAGGC AANGATANAC 1229
Takifugu_rubripes                 ---------- ---------- ---------- ---------- ---------- ---------- ---------- -
Bos_taurus                        ---------- ---------- ---------- ---------- ---------- ---------- ---------- -
Homo_sapiens                      ---------- ---------- ---------- ---------- ---------- ---------- ---------- -
Microcebus_murinus                ---------- ---------- ---------- ---------- ---------- ---------- ---------- -
Myotis_lucifugus                  ---------- ---------- ---------- ---------- ---------- ---------- ---------- -
Equus_caballus                    ---------- ---------- ---------- ---------- ---------- ---------- ---------- -
Tupaia_belangeri                  ---------- ---------- ---------- ---------- ---------- ---------- ---------- -
Loxodonta_africana                ---------- ---------- ---------- ---------- ---------- ---------- ---------- -
Murinae                           ---------- ---------- ---------- ---------- ---------- ---------- ---------- -
Canis_lupus_familiaris            ---------- ---------- ---------- ---------- ---------- ---------- ---------- -
Sus_scrofa                        ---------- ---------- ---------- ---------- ---------- ---------- ---------- -
Choloepus_hoffmanni               ---------- ---------- ---------- ---------- ---------- ---------- ---------- -
Vicugna_pacos                     ---------- ---------- ---------- ---------- ---------- ---------- ---------- -
Gavialis_gangeticus               ---------- ---------- ---------- ---------- ---------- ---------- ---------- -
Mammalia                          ---------- ---------- ---------- ---------- ---------- ---------- ---------- -
Passeroidea                       ---------- ---------- ---------- ---------- ---------- ---------- ---------- -
Takifugu                          ---------- ---------- ---------- ---------- ---------- ---------- ---------- -
Pteropus_vampyrus                 ---------- ---------- ---------- ---------- ---------- ---------- ---------- -
Ornithorhynchus                   ---------- ---------- ---------- ---------- ---------- ---------- ---------- -
Oryctolagus_cuniculus             ---------- ---------- ---------- ---------- ---------- ---------- ---------- -
Gallus_gallus                     ---------- ---------- ---------- ---------- ---------- ---------- ---------- -
Hominidae                         ---------- ---------- ---------- ---------- ---------- ---------- ---------- -
Metatheria                        ---------- ---------- ---------- ---------- ---------- ---------- ---------- -
Rodentia                          ---------- ---------- ---------- ---------- ---------- ---------- ---------- -
Monodelphis                       ---------- ---------- ---------- ---------- ---------- ---------- ---------- -
Consensus                         ---------- ---------- ---------- ---------- ---------- ---------- ----------
                              100%
Conservation
                                0%
```

1,760 1,780 1,800 1,820

Dipodomys_ordii	---------- ---------- ---------- ---------- ---------- ---------- ----------	-
Laurasiatheria	---------- ---------- ---------- ---------- ---------- ---------- ----------	-
Eutheria	---------- ---------- ---------- ---------- ---------- ---------- ----------	-
Tarsius_syrichta	---------- ---------- ---------- ---------- ---------- ---------- ----------	-
Muridae	---------- ---------- ---------- ---------- ---------- ---------- ----------	-
Gasterosteus_aculeatus	---------- ---------- ---------- ---------- ---------- ---------- ----------	-
Felis_silvestris	---------- ---------- ---------- ---------- ---------- ---------- ----------	-
Rattus	---------- ---------- ---------- ---------- ---------- ---------- ----------	-
Papio_hamadryas	---------- ---------- ---------- ---------- ---------- ---------- ----------	-
Pan_troglodytes	---------- ---------- ---------- ---------- ---------- ---------- ----------	-
Ailuropoda_melanoleuca	---------- ---------- ---------- ---------- ---------- ---------- ----------	-
Cavia_porcellus	---------- ---------- ---------- ---------- ---------- ---------- ----------	-
Ictidomys_tridecemlineatus	---------- ---------- ---------- ---------- ---------- ---------- ----------	-
Canis	---------- ---------- ---------- ---------- ---------- ---------- ----------	-
Felis_catus	---------- ---------- ---------- ---------- ---------- ---------- ----------	-
Ochotona_princeps	---------- ---------- ---------- ---------- ---------- ---------- ----------	-
Cercopithecidae	---------- ---------- ---------- ---------- ---------- ---------- ----------	-
Carnivora	---------- ---------- ---------- ---------- ---------- ---------- ----------	-
Mus	---------- ---------- ---------- ---------- ---------- ---------- ----------	-
Mus_musculus	---------- ---------- ---------- ---------- ---------- ---------- ----------	-
Rattus_norvegicus	---------- ---------- ---------- ---------- ---------- ---------- ----------	-
Tursiops_truncatus	---------- ---------- ---------- ---------- ---------- ---------- ----------	-
Procavia_capensis	---------- ---------- ---------- ---------- ---------- ---------- ----------	-
Primates	---------- ---------- ---------- ---------- ---------- ---------- ----------	-
Echinops_telfairi	---------- ---------- ---------- ---------- ---------- ---------- ----------	-
Sorex_araneus	---------- ---------- ---------- ---------- ---------- ---------- ----------	-
Canidae	---------- ---------- ---------- ---------- ---------- ---------- ----------	-
Otolemur_garnettii	---------- ---------- ---------- ---------- ---------- ---------- ----------	-
Gallus	---------- ---------- ---------- ---------- ---------- ---------- ----------	-
Callithrix_jacchus	---------- ---------- ---------- ---------- ---------- ---------- ----------	-
Crocodylus_porosus	---------- ---------- ---------- ---------- ---------- ---------- ----------	-
Erinaceus_europaeus	---------- ---------- ---------- ---------- ---------- ---------- ----------	-
rnd-6_family-7426-LTR-ERV1	---------- ---------- ---------- ---------- ---------- ---------- ----------	-
Esox_lucius	---------- ---------- ---------- ---------- ---------- ---------- ----------	-
Salmo_salar	---------- ---------- ---------- ---------- ---------- ---------- ----------	-
Didelphidae	---------- ---------- ---------- ---------- ---------- ---------- ----------	-
Latimeria_chalumnae	---------- ---------- ---------- ---------- ---------- ---------- ----------	-
Monodelphis_domestica	---------- ---------- ---------- ---------- ---------- ---------- ----------	-
Macropus_eugenii	---------- ---------- ---------- ---------- ---------- ---------- ----------	-
Alligator_mississippiensis	---------- ---------- ---------- ---------- ---------- ---------- ----------	-
Chrysemys_picta_bellii	---------- ---------- ---------- ---------- ---------- ---------- ----------	-
Danio_rerio	---------- ---------- ---------- ---------- ---------- ---------- ----------	-
Taeniopygia	---------- ---------- ---------- ---------- ---------- ---------- ----------	-
Dasypus_novemcinctus	---------- ---------- ---------- ---------- ---------- ---------- ----------	-
LTR_XT	---------- ---------- ---------- ---------- ---------- ---------- ----------	-
Haplorrhini	CTTCTGTCAC TGTTCAATAC AGAAGAACGC TGCAGAGTAA TACAAGCGGC TCATCAGTGG CTAGAAAGCA	1815
Catarrhini	CTCATGTACC TCTTTAATAC AGATGAAAGG CGAAGGGTGC TCCGGGTGGC AACTAAGTGG CTANAGGAGC	1428
Anolis_carolinensis	---------- ---------- ---------- ---------- ---------- ---------- ----------	-
Murine_leukemia_virus	TTGGGGACCC TGCTGACNGG AGAAGAAAAG CAGCGGGTGC TCCTAGAGGC TAGAAAGGCA GTCCGGGGCG	1388
Phascolarctos_cinereus	CTGCAAGTCC TCTTTACCAC CGAGGAAAGA GAAAGGATTC TTTTGGAGGC TCGCAAAAAC GTCCTTGGAG	1309
Estrildidae	---------- ---------- ---------- ---------- ---------- ---------- ----------	-
Xenopus_Silurana_tropicalis	---------- ---------- ---------- ---------- ---------- ---------- ----------	-
I_XT	NGCNNAAAGA NTNANNTAGA GANAAACNAN TTATGAGGNA AGCCACAGNG CCACCTGNNA ANATTCAAGC	1299
Takifugu_rubripes	---------- ---------- ---------- ---------- ---------- ---------- ----------	-
Bos_taurus	---------- ---------- ---------- ---------- ---------- ---------- ----------	-
Homo_sapiens	---------- ---------- ---------- ---------- ---------- ---------- ----------	-
Microcebus_murinus	---------- ---------- ---------- ---------- ---------- ---------- ----------	-
Myotis_lucifugus	---------- ---------- ---------- ---------- ---------- ---------- ----------	-
Equus_caballus	---------- ---------- ---------- ---------- ---------- ---------- ----------	-
Tupaia_belangeri	---------- ---------- ---------- ---------- ---------- ---------- ----------	-
Loxodonta_africana	---------- ---------- ---------- ---------- ---------- ---------- ----------	-
Murinae	---------- ---------- ---------- ---------- ---------- ---------- ----------	-
Canis_lupus_familiaris	---------- ---------- ---------- ---------- ---------- ---------- ----------	-
Sus_scrofa	---------- ---------- ---------- ---------- ---------- ---------- ----------	-
Choloepus_hoffmanni	---------- ---------- ---------- ---------- ---------- ---------- ----------	-
Vicugna_pacos	---------- ---------- ---------- ---------- ---------- ---------- ----------	-
Gavialis_gangeticus	---------- ---------- ---------- ---------- ---------- ---------- ----------	-
Mammalia	---------- ---------- ---------- ---------- ---------- ---------- ----------	-
Passeroidea	---------- ---------- ---------- ---------- ---------- ---------- ----------	-
Takifugu	---------- ---------- ---------- ---------- ---------- ---------- ----------	-
Pteropus_vampyrus	---------- ---------- ---------- ---------- ---------- ---------- ----------	-
Ornithorhynchus	---------- ---------- ---------- ---------- ---------- ---------- ----------	-
Oryctolagus_cuniculus	---------- ---------- ---------- ---------- ---------- ---------- ----------	-
Gallus_gallus	---------- ---------- ---------- ---------- ---------- ---------- ----------	-
Hominidae	---------- ---------- ---------- ---------- ---------- ---------- ----------	-
Metatheria	---------- ---------- ---------- ---------- ---------- ---------- ----------	-
Rodentia	---------- ---------- ---------- ---------- ---------- ---------- ----------	-
Monodelphis	---------- ---------- ---------- ---------- ---------- ---------- ----------	-
Consensus	---------- ---------- ---------- ---------- ---------- ---------- ----------	

Conservation 100% 0%

		1,840		1,860		1,880		
Dipodomys_ordii	----------	----------	----------	----------	----------	----------	----------	-
Laurasiatheria	----------	----------	----------	----------	----------	----------	----------	-
Eutheria	----------	----------	----------	----------	----------	----------	----------	-
Tarsius_syrichta	----------	----------	----------	----------	----------	----------	----------	-
Muridae	----------	----------	----------	----------	----------	----------	----------	-
Gasterosteus_aculeatus	----------	----------	----------	----------	----------	----------	----------	-
Felis_silvestris	----------	----------	----------	----------	----------	----------	----------	-
Rattus	----------	----------	----------	----------	----------	----------	----------	-
Papio_hamadryas	----------	----------	----------	----------	----------	----------	----------	-
Pan_troglodytes	----------	----------	----------	----------	----------	----------	----------	-
Ailuropoda_melanoleuca	----------	----------	----------	----------	----------	----------	----------	-
Cavia_porcellus	----------	----------	----------	----------	----------	----------	----------	-
Ictidomys_tridecemlineatus	----------	----------	----------	----------	----------	----------	----------	-
Canis	----------	----------	----------	----------	----------	----------	----------	-
Felis_catus	----------	----------	----------	----------	----------	----------	----------	-
Ochotona_princeps	----------	----------	----------	----------	----------	----------	----------	-
Cercopithecidae	----------	----------	----------	----------	----------	----------	----------	-
Carnivora	----------	----------	----------	----------	----------	----------	----------	-
Mus	----------	----------	----------	----------	----------	----------	----------	-
Mus_musculus	----------	----------	----------	----------	----------	----------	----------	-
Rattus_norvegicus	----------	----------	----------	----------	----------	----------	----------	-
Tursiops_truncatus	----------	----------	----------	----------	----------	----------	----------	-
Procavia_capensis	----------	----------	----------	----------	----------	----------	----------	-
Primates	----------	----------	----------	----------	----------	----------	----------	-
Echinops_telfairi	----------	----------	----------	----------	----------	----------	----------	-
Sorex_araneus	----------	----------	----------	----------	----------	----------	----------	-
Canidae	----------	----------	----------	----------	----------	----------	----------	-
Otolemur_garnettii	----------	----------	----------	----------	----------	----------	----------	-
Gallus	----------	----------	----------	----------	----------	----------	----------	-
Callithrix_jacchus	----------	----------	----------	----------	----------	----------	----------	-
Crocodylus_porosus	----------	----------	----------	----------	----------	----------	----------	-
Erinaceus_europaeus	----------	----------	----------	----------	----------	----------	----------	-
rnd-6_family-7426-LTR-ERV1	----------	----------	----------	----------	----------	----------	----------	-
Esox_lucius	----------	----------	----------	----------	----------	----------	----------	-
Salmo_salar	----------	----------	----------	----------	----------	----------	----------	-
Didelphidae	----------	----------	----------	----------	----------	----------	----------	-
Latimeria_chalumnae	----------	----------	----------	----------	----------	----------	----------	-
Monodelphis_domestica	----------	----------	----------	----------	----------	----------	----------	-
Macropus_eugenii	----------	----------	----------	----------	----------	----------	----------	-
Alligator_mississippiensis	----------	----------	----------	----------	----------	----------	----------	-
Chrysemys_picta_bellii	----------	----------	----------	----------	----------	----------	----------	-
Danio_rerio	----------	----------	----------	----------	----------	----------	----------	-
Taeniopygia	----------	----------	----------	----------	----------	----------	----------	-
Dasypus_novemcinctus	----------	----------	----------	----------	----------	----------	----------	-
LTR_XT	----------	----------	----------	----------	----------	----------	----------	-
Haplorrhini	ATGCCCCAGT	AGGCACAGGA	GATGTCAGAN	AGTATGCNCA	GCAGGCTTTG	CCAATAGAAA	CTGACCCAGG	1885
Catarrhini	ACGTCCCAGC	CGATTACCAA	AACCCCCAAG	AATATATAAG	A---ATTCAG	CTGCCAGGAA	CAGACCCCCA	1495
Anolis_carolinensis	----------	----------	----------	----------	----------	----------	----------	-
Murine_leukemia_virus	ACGATGGACG	CCCCACCCAG	----TTGCCT	AATGAAGTCG	ATGCCGCCTT	TCCCCTCGAA	C--GCCCCGA	1452
Phascolarctos_cinereus	TTAACGGGGC	CCCTACACAA	----CTCGAG	AACCTCATTA	ATGAGGCCTT	CCCCCTTAAT	C--GACCTCA	1373
Estrildidae	----------	----------	----------	----------	----------	----------	----------	-
Xenopus_Silurana_tropicalis	----------	----------	----------	----------	----------	----------	----------	-
I_XT	NACAAACAGN	AANCAGNTTA	TTNACCNCNT	CTNAGCCAAA	TTAGGGAATT	GCGNACAGTN	GNAGGNCANA	1369
Takifugu_rubripes	----------	----------	----------	----------	----------	----------	----------	-
Bos_taurus	----------	----------	----------	----------	----------	----------	----------	-
Homo_sapiens	----------	----------	----------	----------	----------	----------	----------	-
Microcebus_murinus	----------	----------	----------	----------	----------	----------	----------	-
Myotis_lucifugus	----------	----------	----------	----------	----------	----------	----------	-
Equus_caballus	----------	----------	----------	----------	----------	----------	----------	-
Tupaia_belangeri	----------	----------	----------	----------	----------	----------	----------	-
Loxodonta_africana	----------	----------	----------	----------	----------	----------	----------	-
Murinae	----------	----------	----------	----------	----------	----------	----------	-
Canis_lupus_familiaris	----------	----------	----------	----------	----------	----------	----------	-
Sus_scrofa	----------	----------	----------	----------	----------	----------	----------	-
Choloepus_hoffmanni	----------	----------	----------	----------	----------	----------	----------	-
Vicugna_pacos	----------	----------	----------	----------	----------	----------	----------	-
Gavialis_gangeticus	----------	----------	----------	----------	----------	----------	----------	-
Mammalia	----------	----------	----------	----------	----------	----------	----------	-
Passeroidea	----------	----------	----------	----------	----------	----------	----------	-
Takifugu	----------	----------	----------	----------	----------	----------	----------	-
Pteropus_vampyrus	----------	----------	----------	----------	----------	----------	----------	-
Ornithorhynchus	----------	----------	----------	----------	----------	----------	----------	-
Oryctolagus_cuniculus	----------	----------	----------	----------	----------	----------	----------	-
Gallus_gallus	----------	----------	----------	----------	----------	----------	----------	-
Hominidae	----------	----------	----------	----------	----------	----------	----------	-
Metatheria	----------	----------	----------	----------	----------	----------	----------	-
Rodentia	----------	----------	----------	----------	----------	----------	----------	-
Monodelphis	----------	----------	----------	----------	----------	----------	----------	-
Consensus	----------	----------	----------	----------	----------	----------	----------	

Conservation 100% 0%

```
                               1,900                1,920                1,940                1,960
Dipodomys_ordii                ---------- ---------- ---------- ---------- ---------- ---------- ---------- -
Laurasiatheria                 ---------- ---------- ---------- ---------- ---------- ---------- ---------- -
Eutheria                       ---------- ---------- ---------- ---------- ---------- ---------- ---------- -
Tarsius_syrichta               ---------- ---------- ---------- ---------- ---------- ---------- ---------- -
Muridae                        ---------- ---------- ---------- ---------- ---------- ---------- ---------- -
Gasterosteus_aculeatus         ---------- ---------- ---------- ---------- ---------- ---------- ---------- -
Felis_silvestris               ---------- ---------- ---------- ---------- ---------- ---------- ---------- -
Rattus                         ---------- ---------- ---------- ---------- ---------- ---------- ---------- -
Papio_hamadryas                ---------- ---------- ---------- ---------- ---------- ---------- ---------- -
Pan_troglodytes                ---------- ---------- ---------- ---------- ---------- ---------- ---------- -
Ailuropoda_melanoleuca         ---------- ---------- ---------- ---------- ---------- ---------- ---------- -
Cavia_porcellus                ---------- ---------- ---------- ---------- ---------- ---------- ---------- -
Ictidomys_tridecemlineatus     ---------- ---------- ---------- ---------- ---------- ---------- ---------- -
Canis                          ---------- ---------- ---------- ---------- ---------- ---------- ---------- -
Felis_catus                    ---------- ---------- ---------- ---------- ---------- ---------- ---------- -
Ochotona_princeps              ---------- ---------- ---------- ---------- ---------- ---------- ---------- -
Cercopithecidae                ---------- ---------- ---------- ---------- ---------- ---------- ---------- -
Carnivora                      ---------- ---------- ---------- ---------- ---------- ---------- ---------- -
Mus                            ---------- ---------- ---------- ---------- ---------- ---------- ---------- -
Mus_musculus                   ---------- ---------- ---------- ---------- ---------- ---------- ---------- -
Rattus_norvegicus              ---------- ---------- ---------- ---------- ---------- ---------- ---------- -
Tursiops_truncatus             ---------- ---------- ---------- ---------- ---------- ---------- ---------- -
Procavia_capensis              ---------- ---------- ---------- ---------- ---------- ---------- ---------- -
Primates                       ---------- ---------- ---------- ---------- ---------- ---------- ---------- -
Echinops_telfairi              ---------- ---------- ---------- ---------- ---------- ---------- ---------- -
Sorex_araneus                  ---------- ---------- ---------- ---------- ---------- ---------- ---------- -
Canidae                        ---------- ---------- ---------- ---------- ---------- ---------- ---------- -
Otolemur_garnettii             ---------- ---------- ---------- ---------- ---------- ---------- ---------- -
Gallus                         ---------- ---------- ---------- ---------- ---------- ---------- ---------- -
Callithrix_jacchus             ---------- ---------- ---------- ---------- ---------- ---------- ---------- -
Crocodylus_porosus             ---------- ---------- ---------- ---------- ---------- ---------- ---------- -
Erinaceus_europaeus            ---------- ---------- ---------- ---------- ---------- ---------- ---------- -
rnd-6_family-7426-LTR-ERV1     ---------- ---------- ---------- ---------- ---------- ---------- ---------- -
Esox_lucius                    ---------- ---------- ---------- ---------- ---------- ---------- ---------- -
Salmo_salar                    ---------- ---------- ---------- ---------- ---------- ---------- ---------- -
Didelphidae                    ---------- ---------- ---------- ---------- ---------- ---------- ---------- -
Latimeria_chalumnae            ---------- ---------- ---------- ---------- ---------- ---------- ---------- -
Monodelphis_domestica          ---------- ---------- ---------- ---------- ---------- ---------- ---------- -
Macropus_eugenii               ---------- ---------- ---------- ---------- ---------- ---------- ---------- -
Alligator_mississippiensis     ---------- ---------- ---------- ---------- ---------- ---------- ---------- -
Chrysemys_picta_bellii         ---------- ---------- ---------- ---------- ---------- ---------- ---------- -
Danio_rerio                    ---------- ---------- ---------- ---------- ---------- ---------- ---------- -
Taeniopygia                    ---------- ---------- ---------- ---------- ---------- ---------- ---------- -
Dasypus_novemcinctus           ---------- ---------- ---------- ---------- ---------- ---------- ---------- -
LTR_XT                         ---------- ---------- ---------- ---------- ---------- ---------- ---------- -
Haplorrhini                    CTGGGACCCA AATCAGGCCC AAGGGCTGCT GAACTTGCTG AGATACCGAG AGGCTCTGAT ACAAGGAATA 1955
Catarrhini                     GTGGGACCCG AACGAGGGAC CAGACATGGA GAGGCTAAGA CGGTACCGTG AGGCATTAAT AGAAGGTCTA 1565
Anolis_carolinensis            ---------- ---------- ---------- ---------- ---------- ---------- ---------- -
Murine_leukemia_virus          TTGGGATTAC ACCACCNCNG AAGGTAGGAA CCACCTAGTC CTCTATCGCC AGTTGCTCTT AGCGGGTCTC 1522
Phascolarctos_cinereus         GTGGGATCAC AACACGGCTG AAGGTAGGGA GCGTCTCCTG GTCTACCGCC GGACTCTAGT GGCGGGTCTC 1443
Estrildidae                    ---------- ---------- ---------- ---------- ---------- ---------- ---------- -
Xenopus_Silurana_tropicalis    ---------- ---------- ---------- ---------- ---------- ---------- ---------- -
I_XT                           GANCCGGAGN TATAANCTCA GANATNCCTT GNAAACNTAG CAAANNNCNA NTNATTNGAA NCGCCANTAC 1439
Takifugu_rubripes              ---------- ---------- ---------- ---------- ---------- ---------- ---------- -
Bos_taurus                     ---------- ---------- ---------- ---------- ---------- ---------- ---------- -
Homo_sapiens                   ---------- ---------- ---------- ---------- ---------- ---------- ---------- -
Microcebus_murinus             ---------- ---------- ---------- ---------- ---------- ---------- ---------- -
Myotis_lucifugus               ---------- ---------- ---------- ---------- ---------- ---------- ---------- -
Equus_caballus                 ---------- ---------- ---------- ---------- ---------- ---------- ---------- -
Tupaia_belangeri               ---------- ---------- ---------- ---------- ---------- ---------- ---------- -
Loxodonta_africana             ---------- ---------- ---------- ---------- ---------- ---------- ---------- -
Murinae                        ---------- ---------- ---------- ---------- ---------- ---------- ---------- -
Canis_lupus_familiaris         ---------- ---------- ---------- ---------- ---------- ---------- ---------- -
Sus_scrofa                     ---------- ---------- ---------- ---------- ---------- ---------- ---------- -
Choloepus_hoffmanni            ---------- ---------- ---------- ---------- ---------- ---------- ---------- -
Vicugna_pacos                  ---------- ---------- ---------- ---------- ---------- ---------- ---------- -
Gavialis_gangeticus            ---------- ---------- ---------- ---------- ---------- ---------- ---------- -
Mammalia                       ---------- ---------- ---------- ---------- ---------- ---------- ---------- -
Passeroidea                    ---------- ---------- ---------- ---------- ---------- ---------- ---------- -
Takifugu                       ---------- ---------- ---------- ---------- ---------- ---------- ---------- -
Pteropus_vampyrus              ---------- ---------- ---------- ---------- ---------- ---------- ---------- -
Ornithorhynchus                ---------- ---------- ---------- ---------- ---------- ---------- ---------- -
Oryctolagus_cuniculus          ---------- ---------- ---------- ---------- ---------- ---------- ---------- -
Gallus_gallus                  ---------- ---------- ---------- ---------- ---------- ---------- ---------- -
Hominidae                      ---------- ---------- ---------- ---------- ---------- ---------- ---------- -
Metatheria                     ---------- ---------- ---------- ---------- ---------- ---------- ---------- -
Rodentia                       ---------- ---------- ---------- ---------- ---------- ---------- ---------- -
Monodelphis                    ---------- ---------- ---------- ---------- ---------- ---------- ---------- -
Consensus                      ---------- ---------- ---------- ---------- ---------- ---------- ----------
```

Conservation

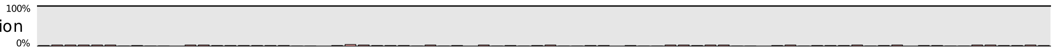

```
                                         1,980                          2,000                          2,020
Dipodomys_ordii                ---------- ---------- ---------- ---------- ---------- ---------- ---------- -
Laurasiatheria                 ---------- ---------- ---------- ---------- ---------- ---------- ---------- -
Eutheria                       ---------- ---------- ---------- ---------- ---------- ---------- ---------- -
Tarsius_syrichta               ---------- ---------- ---------- ---------- ---------- ---------- ---------- -
Muridae                        ---------- ---------- ---------- ---------- ---------- ---------- ---------- -
Gasterosteus_aculeatus         ---------- ---------- ---------- ---------- ---------- ---------- ---------- -
Felis_silvestris               ---------- ---------- ---------- ---------- ---------- ---------- ---------- -
Rattus                         ---------- ---------- ---------- ---------- ---------- ---------- ---------- -
Papio_hamadryas                ---------- ---------- ---------- ---------- ---------- ---------- ---------- -
Pan_troglodytes                ---------- ---------- ---------- ---------- ---------- ---------- ---------- -
Ailuropoda_melanoleuca         ---------- ---------- ---------- ---------- ---------- ---------- ---------- -
Cavia_porcellus                ---------- ---------- ---------- ---------- ---------- ---------- ---------- -
Ictidomys_tridecemlineatus     ---------- ---------- ---------- ---------- ---------- ---------- ---------- -
Canis                          ---------- ---------- ---------- ---------- ---------- ---------- ---------- -
Felis_catus                    ---------- ---------- ---------- ---------- ---------- ---------- ---------- -
Ochotona_princeps              ---------- ---------- ---------- ---------- ---------- ---------- ---------- -
Cercopithecidae                ---------- ---------- ---------- ---------- ---------- ---------- ---------- -
Carnivora                      ---------- ---------- ---------- ---------- ---------- ---------- ---------- -
Mus                            ---------- ---------- ---------- ---------- ---------- ---------- ---------- -
Mus_musculus                   ---------- ---------- ---------- ---------- ---------- ---------- ---------- -
Rattus_norvegicus              ---------- ---------- ---------- ---------- ---------- ---------- ---------- -
Tursiops_truncatus             ---------- ---------- ---------- ---------- ---------- ---------- ---------- -
Procavia_capensis              ---------- ---------- ---------- ---------- ---------- ---------- ---------- -
Primates                       ---------- ---------- ---------- ---------- ---------- ---------- ---------- -
Echinops_telfairi              ---------- ---------- ---------- ---------- ---------- ---------- ---------- -
Sorex_araneus                  ---------- ---------- ---------- ---------- ---------- ---------- ---------- -
Canidae                        ---------- ---------- ---------- ---------- ---------- ---------- ---------- -
Otolemur_garnettii             ---------- ---------- ---------- ---------- ---------- ---------- ---------- -
Gallus                         ---------- ---------- ---------- ---------- ---------- ---------- ---------- -
Callithrix_jacchus             ---------- ---------- ---------- ---------- ---------- ---------- ---------- -
Crocodylus_porosus             ---------- ---------- ---------- ---------- ---------- ---------- ---------- -
Erinaceus_europaeus            ---------- ---------- ---------- ---------- ---------- ---------- ---------- -
rnd-6_family-7426-LTR-ERV1     ---------- ---------- ---------- ---------- ---------- ---------- ---------- -
Esox_lucius                    ---------- ---------- ---------- ---------- ---------- ---------- ---------- -
Salmo_salar                    ---------- ---------- ---------- ---------- ---------- ---------- ---------- -
Didelphidae                    ---------- ---------- ---------- ---------- ---------- ---------- ---------- -
Latimeria_chalumnae            ---------- ---------- ---------- ---------- ---------- ---------- ---------- -
Monodelphis_domestica          ---------- ---------- ---------- ---------- ---------- ---------- ---------- -
Macropus_eugenii               ---------- ---------- ---------- ---------- ---------- ---------- ---------- -
Alligator_mississippiensis     ---------- ---------- ---------- ---------- ---------- ---------- ---------- -
Chrysemys_picta_bellii         ---------- ---------- ---------- ---------- ---------- ---------- ---------- -
Danio_rerio                    ---------- ---------- ---------- ---------- ---------- ---------- ---------- -
Taeniopygia                    ---------- ---------- ---------- ---------- ---------- ---------- ---------- -
Dasypus_novemcinctus           ---------- ---------- ---------- ---------- ---------- ---------- ---------- -
LTR_XT                         ---------- ---------- ---------- ---------- ---------- ---------- ---------- -
Haplorrhini                    AAGGCTGGAG GGAAAAAGGC AACAAACACT GGAAAGGTTT CAGAGGTCCA TCAGAAACCA GATGAAAGCC 2025
Catarrhini                     AAGAAAGGGG CTCAAAAGGC TACAAATGTA AATAAGGTCT CTGAGGTCAT CCAAGGAAAA GAGGAGAGTC 1635
Anolis_carolinensis            ---------- ---------- ---------- ---------- ---------- ---------- ---------- -
Murine_leukemia_virus          CAAAACGCGG GCAGAAGCCC CACCAATTTG GCCAAGGTAA AAGGGATAAC CCAGGGACCT AATGAGTCTC 1592
Phascolarctos_cinereus         AAAGGGGCAG CAAGGCGCCC CACCAATTTG GCCAAGGTAA GAGAAGTCTT GCAGGGACCA ACAGAACCCC 1513
Estrildidae                    ---------- ---------- ---------- ---------- ---------- ---------- ---------- -
Xenopus_Silurana_tropicalis    ---------- ---------- ---------- ---------- ---------- ---------- ---------- -
I_XT                           CACACCCCAT NNANNCNTTN NTNNTNNTNC ATCTTGACGN TTNGNGANGN CCTTAANTTA CTGCCNTTNT 1509
Takifugu_rubripes              ---------- ---------- ---------- ---------- ---------- ---------- ---------- -
Bos_taurus                     ---------- ---------- ---------- ---------- ---------- ---------- ---------- -
Homo_sapiens                   ---------- ---------- ---------- ---------- ---------- ---------- ---------- -
Microcebus_murinus             ---------- ---------- ---------- ---------- ---------- ---------- ---------- -
Myotis_lucifugus               ---------- ---------- ---------- ---------- ---------- ---------- ---------- -
Equus_caballus                 ---------- ---------- ---------- ---------- ---------- ---------- ---------- -
Tupaia_belangeri               ---------- ---------- ---------- ---------- ---------- ---------- ---------- -
Loxodonta_africana             ---------- ---------- ---------- ---------- ---------- ---------- ---------- -
Murinae                        ---------- ---------- ---------- ---------- ---------- ---------- ---------- -
Canis_lupus_familiaris         ---------- ---------- ---------- ---------- ---------- ---------- ---------- -
Sus_scrofa                     ---------- ---------- ---------- ---------- ---------- ---------- ---------- -
Choloepus_hoffmanni            ---------- ---------- ---------- ---------- ---------- ---------- ---------- -
Vicugna_pacos                  ---------- ---------- ---------- ---------- ---------- ---------- ---------- -
Gavialis_gangeticus            ---------- ---------- ---------- ---------- ---------- ---------- ---------- -
Mammalia                       ---------- ---------- ---------- ---------- ---------- ---------- ---------- -
Passeroidea                    ---------- ---------- ---------- ---------- ---------- ---------- ---------- -
Takifugu                       ---------- ---------- ---------- ---------- ---------- ---------- ---------- -
Pteropus_vampyrus              ---------- ---------- ---------- ---------- ---------- ---------- ---------- -
Ornithorhynchus                ---------- ---------- ---------- ---------- ---------- ---------- ---------- -
Oryctolagus_cuniculus          ---------- ---------- ---------- ---------- ---------- ---------- ---------- -
Gallus_gallus                  ---------- ---------- ---------- ---------- ---------- ---------- ---------- -
Hominidae                      ---------- ---------- ---------- ---------- ---------- ---------- ---------- -
Metatheria                     ---------- ---------- ---------- ---------- ---------- ---------- ---------- -
Rodentia                       ---------- ---------- ---------- ---------- ---------- ---------- ---------- -
Monodelphis                    ---------- ---------- ---------- ---------- ---------- ---------- ---------- -
Consensus                      ---------- ---------- ---------- ---------- ---------- ---------- ----------
```

Conservation

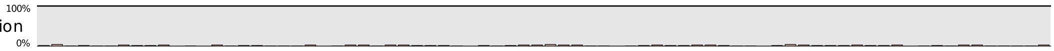

```
                               2,040                2,060                2,080                2,100
Dipodomys_ordii                ---------- ---------- ---------- ---------- ---------- ---------- ---------- -
Laurasiatheria                 ---------- ---------- ---------- ---------- ---------- ---------- ---------- -
Eutheria                       ---------- ---------- ---------- ---------- ---------- ---------- ---------- -
Tarsius_syrichta               ---------- ---------- ---------- ---------- ---------- ---------- ---------- -
Muridae                        ---------- ---------- ---------- ---------- ---------- ---------- ---------- -
Gasterosteus_aculeatus         ---------- ---------- ---------- ---------- ---------- ---------- ---------- -
Felis_silvestris               ---------- ---------- ---------- ---------- ---------- ---------- ---------- -
Rattus                         ---------- ---------- ---------- ---------- ---------- ---------- ---------- -
Papio_hamadryas                ---------- ---------- ---------- ---------- ---------- ---------- ---------- -
Pan_troglodytes                ---------- ---------- ---------- ---------- ---------- ---------- ---------- -
Ailuropoda_melanoleuca         ---------- ---------- ---------- ---------- ---------- ---------- ---------- -
Cavia_porcellus                ---------- ---------- ---------- ---------- ---------- ---------- ---------- -
Ictidomys_tridecemlineatus     ---------- ---------- ---------- ---------- ---------- ---------- ---------- -
Canis                          ---------- ---------- ---------- ---------- ---------- ---------- ---------- -
Felis_catus                    ---------- ---------- ---------- ---------- ---------- ---------- ---------- -
Ochotona_princeps              ---------- ---------- ---------- ---------- ---------- ---------- ---------- -
Cercopithecidae                ---------- ---------- ---------- ---------- ---------- ---------- ---------- -
Carnivora                      ---------- ---------- ---------- ---------- ---------- ---------- ---------- -
Mus                            ---------- ---------- ---------- ---------- ---------- ---------- ---------- -
Mus_musculus                   ---------- ---------- ---------- ---------- ---------- ---------- ---------- -
Rattus_norvegicus              ---------- ---------- ---------- ---------- ---------- ---------- ---------- -
Tursiops_truncatus             ---------- ---------- ---------- ---------- ---------- ---------- ---------- -
Procavia_capensis              ---------- ---------- ---------- ---------- ---------- ---------- ---------- -
Primates                       ---------- ---------- ---------- ---------- ---------- ---------- ---------- -
Echinops_telfairi              ---------- ---------- ---------- ---------- ---------- ---------- ---------- -
Sorex_araneus                  ---------- ---------- ---------- ---------- ---------- ---------- ---------- -
Canidae                        ---------- ---------- ---------- ---------- ---------- ---------- ---------- -
Otolemur_garnettii             ---------- ---------- ---------- ---------- ---------- ---------- ---------- -
Gallus                         ---------- ---------- ---------- ---------- ---------- ---------- ---------- -
Callithrix_jacchus             ---------- ---------- ---------- ---------- ---------- ---------- ---------- -
Crocodylus_porosus             ---------- ---------- ---------- ---------- ---------- ---------- ---------- -
Erinaceus_europaeus            ---------- ---------- ---------- ---------- ---------- ---------- ---------- -
rnd-6_family-7426-LTR-ERV1     ---------- ---------- ---------- ---------- ---------- ---------- ---------- -
Esox_lucius                    ---------- ---------- ---------- ---------- ---------- ---------- ---------- -
Salmo_salar                    ---------- ---------- ---------- ---------- ---------- ---------- ---------- -
Didelphidae                    ---------- ---------- ---------- ---------- ---------- ---------- ---------- -
Latimeria_chalumnae            ---------- ---------- ---------- ---------- ---------- ---------- ---------- -
Monodelphis_domestica          ---------- ---------- ---------- ---------- ---------- ---------- ---------- -
Macropus_eugenii               ---------- ---------- ---------- ---------- ---------- ---------- ---------- -
Alligator_mississippiensis     ---------- ---------- ---------- ---------- ---------- ---------- ---------- -
Chrysemys_picta_bellii         ---------- ---------- ---------- ---------- ---------- ---------- ---------- -
Danio_rerio                    ---------- ---------- ---------- ---------- ---------- ---------- ---------- -
Taeniopygia                    ---------- ---------- ---------- ---------- ---------- ---------- ---------- -
Dasypus_novemcinctus           ---------- ---------- ---------- ---------- ---------- ---------- ---------- -
LTR_XT                         ---------- ---------- ---------- ---------- ---------- ---------- ---------- -
Haplorrhini                    CCAGTGAGTT CTATGAGAGG CTTTGTGAGG CTTACCGGCT CTACACACCT TTTGACCCAG AAGCGGCAGG 2095
Catarrhini                     CAGCGCAATT CTATGAAAGA CTGTGTGAGG CTTACCGTAT GTACACTCCT TTTGATCCAG ATAGCCCTGA 1705
Anolis_carolinensis            ---------- ---------- ---------- ---------- ---------- ---------- ---------- -
Murine_leukemia_virus          CCTCGGCCTT TTTAGAGAGA CTCAAGGAGG CCTATCGCAG GTACACTCCT TATGACCCTG AGGACCCAGG 1662
Phascolarctos_cinereus         CGTCCGTTTT TCTAGAACGT CTAATGGAGG CTTATAGGAG ATATACCCCA TTTGATCCCT CTTCTGAGGG 1583
Estrildidae                    ---------- ---------- ---------- ---------- ---------- ---------- ---------- -
Xenopus_Silurana_tropicalis    ---------- ---------- ---------- ---------- ---------- ---------- ---------- -
I_XT                           TGNAGGAGCN CANNANGAAT TGTTAATTGA NACNGGGGCC NAATTTATGG TTATTNNTAA AAAANNTACT 1579
Takifugu_rubripes              ---------- ---------- ---------- ---------- ---------- ---------- ---------- -
Bos_taurus                     ---------- ---------- ---------- ---------- ---------- ---------- ---------- -
Homo_sapiens                   ---------- ---------- ---------- ---------- ---------- ---------- ---------- -
Microcebus_murinus             ---------- ---------- ---------- ---------- ---------- ---------- ---------- -
Myotis_lucifugus               ---------- ---------- ---------- ---------- ---------- ---------- ---------- -
Equus_caballus                 ---------- ---------- ---------- ---------- ---------- ---------- ---------- -
Tupaia_belangeri               ---------- ---------- ---------- ---------- ---------- ---------- ---------- -
Loxodonta_africana             ---------- ---------- ---------- ---------- ---------- ---------- ---------- -
Murinae                        ---------- ---------- ---------- ---------- ---------- ---------- ---------- -
Canis_lupus_familiaris         ---------- ---------- ---------- ---------- ---------- ---------- ---------- -
Sus_scrofa                     ---------- ---------- ---------- ---------- ---------- ---------- ---------- -
Choloepus_hoffmanni            ---------- ---------- ---------- ---------- ---------- ---------- ---------- -
Vicugna_pacos                  ---------- ---------- ---------- ---------- ---------- ---------- ---------- -
Gavialis_gangeticus            ---------- ---------- ---------- ---------- ---------- ---------- ---------- -
Mammalia                       ---------- ---------- ---------- ---------- ---------- ---------- ---------- -
Passeroidea                    ---------- ---------- ---------- ---------- ---------- ---------- ---------- -
Takifugu                       ---------- ---------- ---------- ---------- ---------- ---------- ---------- -
Pteropus_vampyrus              ---------- ---------- ---------- ---------- ---------- ---------- ---------- -
Ornithorhynchus                ---------- ---------- ---------- ---------- ---------- ---------- ---------- -
Oryctolagus_cuniculus          ---------- ---------- ---------- ---------- ---------- ---------- ---------- -
Gallus_gallus                  ---------- ---------- ---------- ---------- ---------- ---------- ---------- -
Hominidae                      ---------- ---------- ---------- ---------- ---------- ---------- ---------- -
Metatheria                     ---------- ---------- ---------- ---------- ---------- ---------- ---------- -
Rodentia                       ---------- ---------- ---------- ---------- ---------- ---------- ---------- -
Monodelphis                    ---------- ---------- ---------- ---------- ---------- ---------- ---------- -
Consensus                      ---------- ---------- ---------- ---------- ---------- ---------- ----------
Conservation                   100%
                               0%
```

```
                                                  2,120                          2,140                          2,160
                                                    |                              |                              |
Dipodomys_ordii                ---------- ---------- ---------- ---------- ---------- ---------- ---------- -
Laurasiatheria                 ---------- ---------- ---------- ---------- ---------- ---------- ---------- -
Eutheria                       ---------- ---------- ---------- ---------- ---------- ---------- ---------- -
Tarsius_syrichta               ---------- ---------- ---------- ---------- ---------- ---------- ---------- -
Muridae                        ---------- ---------- ---------- ---------- ---------- ---------- ---------- -
Gasterosteus_aculeatus         ---------- ---------- ---------- ---------- ---------- ---------- ---------- -
Felis_silvestris               ---------- ---------- ---------- ---------- ---------- ---------- ---------- -
Rattus                         ---------- ---------- ---------- ---------- ---------- ---------- ---------- -
Papio_hamadryas                ---------- ---------- ---------- ---------- ---------- ---------- ---------- -
Pan_troglodytes                ---------- ---------- ---------- ---------- ---------- ---------- ---------- -
Ailuropoda_melanoleuca         ---------- ---------- ---------- ---------- ---------- ---------- ---------- -
Cavia_porcellus                ---------- ---------- ---------- ---------- ---------- ---------- ---------- -
Ictidomys_tridecemlineatus     ---------- ---------- ---------- ---------- ---------- ---------- ---------- -
Canis                          ---------- ---------- ---------- ---------- ---------- ---------- ---------- -
Felis_catus                    ---------- ---------- ---------- ---------- ---------- ---------- ---------- -
Ochotona_princeps              ---------- ---------- ---------- ---------- ---------- ---------- ---------- -
Cercopithecidae                ---------- ---------- ---------- ---------- ---------- ---------- ---------- -
Carnivora                      ---------- ---------- ---------- ---------- ---------- ---------- ---------- -
Mus                            ---------- ---------- ---------- ---------- ---------- ---------- ---------- -
Mus_musculus                   ---------- ---------- ---------- ---------- ---------- ---------- ---------- -
Rattus_norvegicus              ---------- ---------- ---------- ---------- ---------- ---------- ---------- -
Tursiops_truncatus             ---------- ---------- ---------- ---------- ---------- ---------- ---------- -
Procavia_capensis              ---------- ---------- ---------- ---------- ---------- ---------- ---------- -
Primates                       ---------- ---------- ---------- ---------- ---------- ---------- ---------- -
Echinops_telfairi              ---------- ---------- ---------- ---------- ---------- ---------- ---------- -
Sorex_araneus                  ---------- ---------- ---------- ---------- ---------- ---------- ---------- -
Canidae                        ---------- ---------- ---------- ---------- ---------- ---------- ---------- -
Otolemur_garnettii             ---------- ---------- ---------- ---------- ---------- ---------- ---------- -
Gallus                         ---------- ---------- ---------- ---------- ---------- ---------- ---------- -
Callithrix_jacchus             ---------- ---------- ---------- ---------- ---------- ---------- ---------- -
Crocodylus_porosus             ---------- ---------- ---------- ---------- ---------- ---------- ---------- -
Erinaceus_europaeus            ---------- ---------- ---------- ---------- ---------- ---------- ---------- -
rnd-6_family-7426-LTR-ERV1     ---------- ---------- ---------- ---------- ---------- ---------- ---------- -
Esox_lucius                    ---------- ---------- ---------- ---------- ---------- ---------- ---------- -
Salmo_salar                    ---------- ---------- ---------- ---------- ---------- ---------- ---------- -
Didelphidae                    ---------- ---------- ---------- ---------- ---------- ---------- ---------- -
Latimeria_chalumnae            ---------- ---------- ---------- ---------- ---------- ---------- ---------- -
Monodelphis_domestica          ---------- ---------- ---------- ---------- ---------- ---------- ---------- -
Macropus_eugenii               ---------- ---------- ---------- ---------- ---------- ---------- ---------- -
Alligator_mississippiensis     ---------- ---------- ---------- ---------- ---------- ---------- ---------- -
Chrysemys_picta_bellii         ---------- ---------- ---------- ---------- ---------- ---------- ---------- -
Danio_rerio                    ---------- ---------- ---------- ---------- ---------- ---------- ---------- -
Taeniopygia                    ---------- ---------- ---------- ---------- ---------- ---------- ---------- -
Dasypus_novemcinctus           ---------- ---------- ---------- ---------- ---------- ---------- ---------- -
LTR_XT                         ---------- ---------- ---------- ---------- ---------- ---------- ---------- -
Haplorrhini                    GAATCAGTGC ATGGTTAATG CGGCATTCGT GAGTCAG-GC ACAAAATGAC ATTAAGCGAA AGTTGCAGAA 2164
Catarrhini                     AAATCAGCGC ATGATTAACA TGGCCTTAGT TAGTCAAAGC GCGGAA-GAT ATCAGGAGAA AATTGCAGAA 1774
Anolis_carolinensis            ---------- ---------- ---------- ---------- ---------- ---------- ---------- -
Murine_leukemia_virus          GCAAGAAACC AATGTGTCTA TGTCGTTCAT CTGGCAGTCT GCCCCG-GAT ATCGGGCGAA AGTTAGAGCG 1731
Phascolarctos_cinereus         ACAAAAGGCG GCGGTGGCCA TGTCCTTTAT CGGGCAGTCT GCCCCG-GAT ATTAAAAAGA AATTACAAAG 1652
Estrildidae                    ---------- ---------- ---------- ---------- ---------- ---------- ---------- -
Xenopus_Silurana_tropicalis    ---------- ---------- ---------- ---------- ---------- ---------- ---------- -
I_XT                           ATNTTAGCCG TNCCAGNAGN NCCNGCTNGT NCTNTTGTAG GNTTCAANAG AGNNAAANAC NNTGCCANAA 1649
Takifugu_rubripes              ---------- ---------- ---------- ---------- ---------- ---------- ---------- -
Bos_taurus                     ---------- ---------- ---------- ---------- ---------- ---------- ---------- -
Homo_sapiens                   ---------- ---------- ---------- ---------- ---------- ---------- ---------- -
Microcebus_murinus             ---------- ---------- ---------- ---------- ---------- ---------- ---------- -
Myotis_lucifugus               ---------- ---------- ---------- ---------- ---------- ---------- ---------- -
Equus_caballus                 ---------- ---------- ---------- ---------- ---------- ---------- ---------- -
Tupaia_belangeri               ---------- ---------- ---------- ---------- ---------- ---------- ---------- -
Loxodonta_africana             ---------- ---------- ---------- ---------- ---------- ---------- ---------- -
Murinae                        ---------- ---------- ---------- ---------- ---------- ---------- ---------- -
Canis_lupus_familiaris         ---------- ---------- ---------- ---------- ---------- ---------- ---------- -
Sus_scrofa                     ---------- ---------- ---------- ---------- ---------- ---------- ---------- -
Choloepus_hoffmanni            ---------- ---------- ---------- ---------- ---------- ---------- ---------- -
Vicugna_pacos                  ---------- ---------- ---------- ---------- ---------- ---------- ---------- -
Gavialis_gangeticus            ---------- ---------- ---------- ---------- ---------- ---------- ---------- -
Mammalia                       ---------- ---------- ---------- ---------- ---------- ---------- ---------- -
Passeroidea                    ---------- ---------- ---------- ---------- ---------- ---------- ---------- -
Takifugu                       ---------- ---------- ---------- ---------- ---------- ---------- ---------- -
Pteropus_vampyrus              ---------- ---------- ---------- ---------- ---------- ---------- ---------- -
Ornithorhynchus                ---------- ---------- ---------- ---------- ---------- ---------- ---------- -
Oryctolagus_cuniculus          ---------- ---------- ---------- ---------- ---------- ---------- ---------- -
Gallus_gallus                  ---------- ---------- ---------- ---------- ---------- ---------- ---------- -
Hominidae                      ---------- ---------- ---------- ---------- ---------- ---------- ---------- -
Metatheria                     ---------- ---------- ---------- ---------- ---------- ---------- ---------- -
Rodentia                       ---------- ---------- ---------- ---------- ---------- ---------- ---------- -
Monodelphis                    ---------- ---------- ---------- ---------- ---------- ---------- ---------- -

Consensus                      ---------- ---------- ---------- ---------- ---------- ---------- ----------
```

Conservation

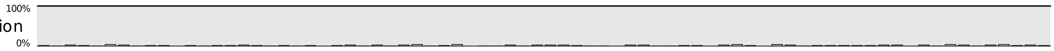

	2,180		2,200		2,220		2,240	
Dipodomys_ordii	----------	----------	----------	----------	----------	----------	----------	-
Laurasiatheria	----------	----------	----------	----------	----------	----------	----------	-
Eutheria	----------	----------	----------	----------	----------	----------	----------	-
Tarsius_syrichta	----------	----------	----------	----------	----------	----------	----------	-
Muridae	----------	----------	----------	----------	----------	----------	----------	-
Gasterosteus_aculeatus	----------	----------	----------	----------	----------	----------	----------	-
Felis_silvestris	----------	----------	----------	----------	----------	----------	----------	-
Rattus	----------	----------	----------	----------	----------	----------	----------	-
Papio_hamadryas	----------	----------	----------	----------	----------	----------	----------	-
Pan_troglodytes	----------	----------	----------	----------	----------	----------	----------	-
Ailuropoda_melanoleuca	----------	----------	----------	----------	----------	----------	----------	-
Cavia_porcellus	----------	----------	----------	----------	----------	----------	----------	-
Ictidomys_tridecemlineatus	----------	----------	----------	----------	----------	----------	----------	-
Canis	----------	----------	----------	----------	----------	----------	----------	-
Felis_catus	----------	----------	----------	----------	----------	----------	----------	-
Ochotona_princeps	----------	----------	----------	----------	----------	----------	----------	-
Cercopithecidae	----------	----------	----------	----------	----------	----------	----------	-
Carnivora	----------	----------	----------	----------	----------	----------	----------	-
Mus	----------	----------	----------	----------	----------	----------	----------	-
Mus_musculus	----------	----------	----------	----------	----------	----------	----------	-
Rattus_norvegicus	----------	----------	----------	----------	----------	----------	----------	-
Tursiops_truncatus	----------	----------	----------	----------	----------	----------	----------	-
Procavia_capensis	----------	----------	----------	----------	----------	----------	----------	-
Primates	----------	----------	----------	----------	----------	----------	----------	-
Echinops_telfairi	----------	----------	----------	----------	----------	----------	----------	-
Sorex_araneus	----------	----------	----------	----------	----------	----------	----------	-
Canidae	----------	----------	----------	----------	----------	----------	----------	-
Otolemur_garnettii	----------	----------	----------	----------	----------	----------	----------	-
Gallus	----------	----------	----------	----------	----------	----------	----------	-
Callithrix_jacchus	----------	----------	----------	----------	----------	----------	----------	-
Crocodylus_porosus	----------	----------	----------	----------	----------	----------	----------	-
Erinaceus_europaeus	----------	----------	----------	----------	----------	----------	----------	-
rnd-6_family-7426-LTR-ERV1	----------	----------	----------	----------	----------	----------	----------	-
Esox_lucius	----------	----------	----------	----------	----------	----------	----------	-
Salmo_salar	----------	----------	----------	----------	----------	----------	----------	-
Didelphidae	----------	----------	----------	----------	----------	----------	----------	-
Latimeria_chalumnae	----------	----------	----------	----------	----------	----------	----------	-
Monodelphis_domestica	----------	----------	----------	----------	----------	----------	----------	-
Macropus_eugenii	----------	----------	----------	----------	----------	----------	----------	-
Alligator_mississippiensis	----------	----------	----------	----------	----------	----------	----------	-
Chrysemys_picta_bellii	----------	----------	----------	----------	----------	----------	----------	-
Danio_rerio	----------	----------	----------	----------	----------	----------	----------	-
Taeniopygia	----------	----------	----------	----------	----------	----------	----------	-
Dasypus_novemcinctus	----------	----------	----------	----------	----------	----------	----------	-
LTR_XT	----------	----------	----------	----------	----------	----------	----------	-
Haplorrhini	GCTGGAGGGG	TTTGAAGGTA	TGAACATTTC	CCAGCTTATC	CAGGTGGCAA	CCAAAGTGTT	TGTAAATCGA	2234
Catarrhini	ACAGGCTGGG	TTTGCGGGTA	TGAATACCTC	ACGGTTACTG	GAAATAGCCA	ATCAAGTGTT	TGTGAATAGA	1844
Anolis_carolinensis	----------	----------	----------	----------	----------	----------	----------	-
Murine_leukemia_virus	GTTAGAAGAT	TTAAAGAGCA	AGACCTTAGG	AGACTTAGTG	AGGGAAGCTG	AAAAGATCTT	TAATAAGCGA	1801
Phascolarctos_cinereus	GCTGGAAGGA	CTCCAGGACC	ATTCCTTGCA	GGATCTCATA	AAGGAGGCAG	AGAAGGTATA	TCATAAGAGA	1722
Estrildidae	----------	----------	----------	----------	----------	----------	----------	-
Xenopus_Silurana_tropicalis	----------	----------	----------	----------	----------	----------	----------	-
I_XT	CACTGATNTC	NAACATTNCA	TGANTNATNC	CNGAGTTTCN	AANNACGGAG	NNNGGTTNCT	GANATNGTNA	1719
Takifugu_rubripes	----------	----------	----------	----------	----------	----------	----------	-
Bos_taurus	----------	----------	----------	----------	----------	----------	----------	-
Homo_sapiens	----------	----------	----------	----------	----------	----------	----------	-
Microcebus_murinus	----------	----------	----------	----------	----------	----------	----------	-
Myotis_lucifugus	----------	----------	----------	----------	----------	----------	----------	-
Equus_caballus	----------	----------	----------	----------	----------	----------	----------	-
Tupaia_belangeri	----------	----------	----------	----------	----------	----------	----------	-
Loxodonta_africana	----------	----------	----------	----------	----------	----------	----------	-
Murinae	----------	----------	----------	----------	----------	----------	----------	-
Canis_lupus_familiaris	----------	----------	----------	----------	----------	----------	----------	-
Sus_scrofa	----------	----------	----------	----------	----------	----------	----------	-
Choloepus_hoffmanni	----------	----------	----------	----------	----------	----------	----------	-
Vicugna_pacos	----------	----------	----------	----------	----------	----------	----------	-
Gavialis_gangeticus	----------	----------	----------	----------	----------	----------	----------	-
Mammalia	----------	----------	----------	----------	----------	----------	----------	-
Passeroidea	----------	----------	----------	----------	----------	----------	----------	-
Takifugu	----------	----------	----------	----------	----------	----------	----------	-
Pteropus_vampyrus	----------	----------	----------	----------	----------	----------	----------	-
Ornithorhynchus	----------	----------	----------	----------	----------	----------	----------	-
Oryctolagus_cuniculus	----------	----------	----------	----------	----------	----------	----------	-
Gallus_gallus	----------	----------	----------	----------	----------	----------	----------	-
Hominidae	----------	----------	----------	----------	----------	----------	----------	-
Metatheria	----------	----------	----------	----------	----------	----------	----------	-
Rodentia	----------	----------	----------	----------	----------	----------	----------	-
Monodelphis	----------	----------	----------	----------	----------	----------	----------	-
Consensus	----------	----------	----------	----------	----------	----------	----------	

Conservation 100% 0%

	2,260			2,280		2,300		
Dipodomys_ordii	----------	----------	----------	----------	----------	----------	----------	-
Laurasiatheria	----------	----------	----------	----------	----------	----------	----------	-
Eutheria	----------	----------	----------	----------	----------	----------	----------	-
Tarsius_syrichta	----------	----------	----------	----------	----------	----------	----------	-
Muridae	----------	----------	----------	----------	----------	----------	----------	-
Gasterosteus_aculeatus	----------	----------	----------	----------	----------	----------	----------	-
Felis_silvestris	----------	----------	----------	----------	----------	----------	----------	-
Rattus	----------	----------	----------	----------	----------	----------	----------	-
Papio_hamadryas	----------	----------	----------	----------	----------	----------	----------	-
Pan_troglodytes	----------	----------	----------	----------	----------	----------	----------	-
Ailuropoda_melanoleuca	----------	----------	----------	----------	----------	----------	----------	-
Cavia_porcellus	----------	----------	----------	----------	----------	----------	----------	-
Ictidomys_tridecemlineatus	----------	----------	----------	----------	----------	----------	----------	-
Canis	----------	----------	----------	----------	----------	----------	----------	-
Felis_catus	----------	----------	----------	----------	----------	----------	----------	-
Ochotona_princeps	----------	----------	----------	----------	----------	----------	----------	-
Cercopithecidae	----------	----------	----------	----------	----------	----------	----------	-
Carnivora	----------	----------	----------	----------	----------	----------	----------	-
Mus	----------	----------	----------	----------	----------	----------	----------	-
Mus_musculus	----------	----------	----------	----------	----------	----------	----------	-
Rattus_norvegicus	----------	----------	----------	----------	----------	----------	----------	-
Tursiops_truncatus	----------	----------	----------	----------	----------	----------	----------	-
Procavia_capensis	----------	----------	----------	----------	----------	----------	----------	-
Primates	----------	----------	----------	----------	----------	----------	----------	-
Echinops_telfairi	----------	----------	----------	----------	----------	----------	----------	-
Sorex_araneus	----------	----------	----------	----------	----------	----------	----------	-
Canidae	----------	----------	----------	----------	----------	----------	----------	-
Otolemur_garnettii	----------	----------	----------	----------	----------	----------	----------	-
Gallus	----------	----------	----------	----------	----------	----------	----------	-
Callithrix_jacchus	----------	----------	----------	----------	----------	----------	----------	-
Crocodylus_porosus	----------	----------	----------	----------	----------	----------	----------	-
Erinaceus_europaeus	----------	----------	----------	----------	----------	----------	----------	-
rnd-6_family-7426-LTR-ERV1	----------	----------	----------	----------	----------	----------	----------	-
Esox_lucius	----------	----------	----------	----------	----------	----------	----------	-
Salmo_salar	----------	----------	----------	----------	----------	----------	----------	-
Didelphidae	----------	----------	----------	----------	----------	----------	----------	-
Latimeria_chalumnae	----------	----------	----------	----------	----------	----------	----------	-
Monodelphis_domestica	----------	----------	----------	----------	----------	----------	----------	-
Macropus_eugenii	----------	----------	----------	----------	----------	----------	----------	-
Alligator_mississippiensis	----------	----------	----------	----------	----------	----------	----------	-
Chrysemys_picta_bellii	----------	----------	----------	----------	----------	----------	----------	-
Danio_rerio	----------	----------	----------	----------	----------	----------	----------	-
Taeniopygia	----------	----------	----------	----------	----------	----------	----------	-
Dasypus_novemcinctus	----------	----------	----------	----------	----------	----------	----------	-
LTR_XT	----------	----------	----------	----------	----------	----------	----------	-
Haplorrhini	GATGAAGAAG	CAAAGCGGGA	GGCCAAGCGT	AGAGTGAAGG	AAAAGGCAGA	GTTCTTGGCC	GCAGCCCTGG	2304
Catarrhini	GATGC---AA	CAAGCCGCAG	AGAAAGCCGT	AAGG--AAGG	CGAACGC---	----------	------CAGG	1890
Anolis_carolinensis	----------	----------	----------	----------	----------	----------	----------	-
Murine_leukemia_virus	GAAACCCCGG	AAGAAAGAGA	GGAACGTATC	AGGAGAGAAA	CAGAGG----	----------	---------A	1848
Phascolarctos_cinereus	GAGACAGAGG	AAGAAAAGCA	AGAAAGAGAG	AAAAAGGAGA	CAGAGG----	----------	---------A	1769
Estrildidae	----------	----------	----------	----------	----------	----------	----------	-
Xenopus_Silurana_tropicalis	----------	----------	----------	----------	----------	----------	----------	-
I_XT	GGGAGCNNAC	ACTNTACTAG	GGACNGATTN	NNTTGAAAAN	NGTGGATNGA	TAGTTGATCT	AGCANANAGG	1789
Takifugu_rubripes	----------	----------	----------	----------	----------	----------	----------	-
Bos_taurus	----------	----------	----------	----------	----------	----------	----------	-
Homo_sapiens	----------	----------	----------	----------	----------	----------	----------	-
Microcebus_murinus	----------	----------	----------	----------	----------	----------	----------	-
Myotis_lucifugus	----------	----------	----------	----------	----------	----------	----------	-
Equus_caballus	----------	----------	----------	----------	----------	----------	----------	-
Tupaia_belangeri	----------	----------	----------	----------	----------	----------	----------	-
Loxodonta_africana	----------	----------	----------	----------	----------	----------	----------	-
Murinae	----------	----------	----------	----------	----------	----------	----------	-
Canis_lupus_familiaris	----------	----------	----------	----------	----------	----------	----------	-
Sus_scrofa	----------	----------	----------	----------	----------	----------	----------	-
Choloepus_hoffmanni	----------	----------	----------	----------	----------	----------	----------	-
Vicugna_pacos	----------	----------	----------	----------	----------	----------	----------	-
Gavialis_gangeticus	----------	----------	----------	----------	----------	----------	----------	-
Mammalia	----------	----------	----------	----------	----------	----------	----------	-
Passeroidea	----------	----------	----------	----------	----------	----------	----------	-
Takifugu	----------	----------	----------	----------	----------	----------	----------	-
Pteropus_vampyrus	----------	----------	----------	----------	----------	----------	----------	-
Ornithorhynchus	----------	----------	----------	----------	----------	----------	----------	-
Oryctolagus_cuniculus	----------	----------	----------	----------	----------	----------	----------	-
Gallus_gallus	----------	----------	----------	----------	----------	----------	----------	-
Hominidae	----------	----------	----------	----------	----------	----------	----------	-
Metatheria	----------	----------	----------	----------	----------	----------	----------	-
Rodentia	----------	----------	----------	----------	----------	----------	----------	-
Monodelphis	----------	----------	----------	----------	----------	----------	----------	-
Consensus	----------	----------	----------	----------	----------	----------	----------	
Conservation (100% / 0%)								

	2,320		2,340		2,360		2,380	
Dipodomys_ordii	----------	----------	----------	----------	----------	----------	----------	-
Laurasiatheria	----------	----------	----------	----------	----------	----------	----------	-
Eutheria	----------	----------	----------	----------	----------	----------	----------	-
Tarsius_syrichta	----------	----------	----------	----------	----------	----------	----------	-
Muridae	----------	----------	----------	----------	----------	----------	----------	-
Gasterosteus_aculeatus	----------	----------	----------	----------	----------	----------	----------	-
Felis_silvestris	----------	----------	----------	----------	----------	----------	----------	-
Rattus	----------	----------	----------	----------	----------	----------	----------	-
Papio_hamadryas	----------	----------	----------	----------	----------	----------	----------	-
Pan_troglodytes	----------	----------	----------	----------	----------	----------	----------	-
Ailuropoda_melanoleuca	----------	----------	----------	----------	----------	----------	----------	-
Cavia_porcellus	----------	----------	----------	----------	----------	----------	----------	-
Ictidomys_tridecemlineatus	----------	----------	----------	----------	----------	----------	----------	-
Canis	----------	----------	----------	----------	----------	----------	----------	-
Felis_catus	----------	----------	----------	----------	----------	----------	----------	-
Ochotona_princeps	----------	----------	----------	----------	----------	----------	----------	-
Cercopithecidae	----------	----------	----------	----------	----------	----------	----------	-
Carnivora	----------	----------	----------	----------	----------	----------	----------	-
Mus	----------	----------	----------	----------	----------	----------	----------	-
Mus_musculus	----------	----------	----------	----------	----------	----------	----------	-
Rattus_norvegicus	----------	----------	----------	----------	----------	----------	----------	-
Tursiops_truncatus	----------	----------	----------	----------	----------	----------	----------	-
Procavia_capensis	----------	----------	----------	----------	----------	----------	----------	-
Primates	----------	----------	----------	----------	----------	----------	----------	-
Echinops_telfairi	----------	----------	----------	----------	----------	----------	----------	-
Sorex_araneus	----------	----------	----------	----------	----------	----------	----------	-
Canidae	----------	----------	----------	----------	----------	----------	----------	-
Otolemur_garnettii	----------	----------	----------	----------	----------	----------	----------	-
Gallus	----------	----------	----------	----------	----------	----------	----------	-
Callithrix_jacchus	----------	----------	----------	----------	----------	----------	----------	-
Crocodylus_porosus	----------	----------	----------	----------	----------	----------	----------	-
Erinaceus_europaeus	----------	----------	----------	----------	----------	----------	----------	-
rnd-6_family-7426-LTR-ERV1	----------	----------	----------	----------	----------	----------	----------	-
Esox_lucius	----------	----------	----------	----------	----------	----------	----------	-
Salmo_salar	----------	----------	----------	----------	----------	----------	----------	-
Didelphidae	----------	----------	----------	----------	----------	----------	----------	-
Latimeria_chalumnae	----------	----------	----------	----------	----------	----------	----------	-
Monodelphis_domestica	----------	----------	----------	----------	----------	----------	----------	-
Macropus_eugenii	----------	----------	----------	----------	----------	----------	----------	-
Alligator_mississippiensis	----------	----------	----------	----------	----------	----------	----------	-
Chrysemys_picta_bellii	----------	----------	----------	----------	----------	----------	----------	-
Danio_rerio	----------	----------	----------	----------	----------	----------	----------	-
Taeniopygia	----------	----------	----------	----------	----------	----------	----------	-
Dasypus_novemcinctus	----------	----------	----------	----------	----------	----------	----------	-
LTR_XT	----------	----------	----------	----------	----------	----------	----------	-
Haplorrhini	TTGAAAGAGA	GGCTGGATTT	GCAAGAGGAC	ATGGACGTGG	TCAT-GGATG	CGGTCACGGT	AGAGGACAAG	2373
Catarrhini	CTAGGCGAAA	CGCCG-ACTT	AC--------	-TGGCCGCGG	CCATTAGGGG	AATTCCCCCG	AAAGGAGAGG	1950
Anolis_carolinensis	----------	----------	----------	----------	----------	----------	----------	-
Murine_leukemia_virus	AAAAGAAGAA	CGCCG---TA	G--GGCAGAG	GATGAGCAGA	GAGAGAAAGA	AAGGGACCGC	AGAAGACATA	1913
Phascolarctos_cinereus	AAGAGAGAGA	CGGCG---TG	ACAGGCGCCA	GGAGAAAAAC	TTGACCAAAA	TCTTGGCCGC	GGTAGTAAGT	1836
Estrildidae	----------	----------	----------	----------	----------	----------	----------	-
Xenopus_Silurana_tropicalis	----------	----------	----------	----------	----------	----------	----------	-
I_XT	GAAATATGGA	ATNATGGCNT	AGGTAAANAT	CNGGTTGTTA	TTGATGCNNC	TGGGTTGACN	AAAAACTTTA	1859
Takifugu_rubripes	----------	----------	----------	----------	----------	----------	----------	-
Bos_taurus	----------	----------	----------	----------	----------	----------	----------	-
Homo_sapiens	----------	----------	----------	----------	----------	----------	----------	-
Microcebus_murinus	----------	----------	----------	----------	----------	----------	----------	-
Myotis_lucifugus	----------	----------	----------	----------	----------	----------	----------	-
Equus_caballus	----------	----------	----------	----------	----------	----------	----------	-
Tupaia_belangeri	----------	----------	----------	----------	----------	----------	----------	-
Loxodonta_africana	----------	----------	----------	----------	----------	----------	----------	-
Murinae	----------	----------	----------	----------	----------	----------	----------	-
Canis_lupus_familiaris	----------	----------	----------	----------	----------	----------	----------	-
Sus_scrofa	----------	----------	----------	----------	----------	----------	----------	-
Choloepus_hoffmanni	----------	----------	----------	----------	----------	----------	----------	-
Vicugna_pacos	----------	----------	----------	----------	----------	----------	----------	-
Gavialis_gangeticus	----------	----------	----------	----------	----------	----------	----------	-
Mammalia	----------	----------	----------	----------	----------	----------	----------	-
Passeroidea	----------	----------	----------	----------	----------	----------	----------	-
Takifugu	----------	----------	----------	----------	----------	----------	----------	-
Pteropus_vampyrus	----------	----------	----------	----------	----------	----------	----------	-
Ornithorhynchus	----------	----------	----------	----------	----------	----------	----------	-
Oryctolagus_cuniculus	----------	----------	----------	----------	----------	----------	----------	-
Gallus_gallus	----------	----------	----------	----------	----------	----------	----------	-
Hominidae	----------	----------	----------	----------	----------	----------	----------	-
Metatheria	----------	----------	----------	----------	----------	----------	----------	-
Rodentia	----------	----------	----------	----------	----------	----------	----------	-
Monodelphis	----------	----------	----------	----------	----------	----------	----------	-
Consensus	----------	----------	----------	----------	----------	----------	----------	

Conservation 100% 0%

	2,400		2,420			2,440		
Dipodomys_ordii	----------	----------	----------	----------	----------	----------	----------	-
Laurasiatheria	----------	----------	----------	----------	----------	----------	----------	-
Eutheria	----------	----------	----------	----------	----------	----------	----------	-
Tarsius_syrichta	----------	----------	----------	----------	----------	----------	----------	-
Muridae	----------	----------	----------	----------	----------	----------	----------	-
Gasterosteus_aculeatus	----------	----------	----------	----------	----------	----------	----------	-
Felis_silvestris	----------	----------	----------	----------	----------	----------	----------	-
Rattus	----------	----------	----------	----------	----------	----------	----------	-
Papio_hamadryas	----------	----------	----------	----------	----------	----------	----------	-
Pan_troglodytes	----------	----------	----------	----------	----------	----------	----------	-
Ailuropoda_melanoleuca	----------	----------	----------	----------	----------	----------	----------	-
Cavia_porcellus	----------	----------	----------	----------	----------	----------	----------	-
Ictidomys_tridecemlineatus	----------	----------	----------	----------	----------	----------	----------	-
Canis	----------	----------	----------	----------	----------	----------	----------	-
Felis_catus	----------	----------	----------	----------	----------	----------	----------	-
Ochotona_princeps	----------	----------	----------	----------	----------	----------	----------	-
Cercopithecidae	----------	----------	----------	----------	----------	----------	----------	-
Carnivora	----------	----------	----------	----------	----------	----------	----------	-
Mus	----------	----------	----------	----------	----------	----------	----------	-
Mus_musculus	----------	----------	----------	----------	----------	----------	----------	-
Rattus_norvegicus	----------	----------	----------	----------	----------	----------	----------	-
Tursiops_truncatus	----------	----------	----------	----------	----------	----------	----------	-
Procavia_capensis	----------	----------	----------	----------	----------	----------	----------	-
Primates	----------	----------	----------	----------	----------	----------	----------	-
Echinops_telfairi	----------	----------	----------	----------	----------	----------	----------	-
Sorex_araneus	----------	----------	----------	----------	----------	----------	----------	-
Canidae	----------	----------	----------	----------	----------	----------	----------	-
Otolemur_garnettii	----------	----------	----------	----------	----------	----------	----------	-
Gallus	----------	----------	----------	----------	----------	----------	----------	-
Callithrix_jacchus	----------	----------	----------	----------	----------	----------	----------	-
Crocodylus_porosus	----------	----------	----------	----------	----------	----------	----------	-
Erinaceus_europaeus	----------	----------	----------	----------	----------	----------	----------	-
rnd-6_family-7426-LTR-ERV1	----------	----------	----------	----------	----------	----------	----------	-
Esox_lucius	----------	----------	----------	----------	----------	----------	----------	-
Salmo_salar	----------	----------	----------	----------	----------	----------	----------	-
Didelphidae	----------	----------	----------	----------	----------	----------	----------	-
Latimeria_chalumnae	----------	----------	----------	----------	----------	----------	----------	-
Monodelphis_domestica	----------	----------	----------	----------	----------	----------	----------	-
Macropus_eugenii	----------	----------	----------	----------	----------	----------	----------	-
Alligator_mississippiensis	----------	----------	----------	----------	----------	----------	----------	-
Chrysemys_picta_bellii	----------	----------	----------	----------	----------	----------	----------	-
Danio_rerio	----------	----------	----------	----------	----------	----------	----------	-
Taeniopygia	----------	----------	----------	----------	----------	----------	----------	-
Dasypus_novemcinctus	----------	----------	----------	----------	----------	----------	----------	-
LTR_XT	----------	----------	----------	----------	----------	----------	----------	-
Haplorrhini	CTAGGCCAGG	TCAGGAGACC	AGGACAGGTC	AGGAAGGTCA	GCCTAGACTA	GAGAGAGATC	AATGTGCGAG	2443
Catarrhini	GAAAGGGGGG	TTCCGGGAAG	AATAC---CC	AGTCTAATCG	CCCACGCTTG	CAACGTAACC	AATGCGCCTA	2017
Anolis_carolinensis	----------	----------	----------	----------	----------	----------	----------	-
Murine_leukemia_virus	GAGAGATGAG	CAAGCTCTTG	GCCAC-----	-TGTAGTTAN	TGGTCAGAGA	CAGGATAGAC	AGGGGGGAGA	1977
Phascolarctos_cinereus	GAGAAAGGGT	TCAGAGGGAG	ACAG------	--GCAGGGAA	TC-TGAGCAA	CAGGGCAATG	AGGGCACCTA	1897
Estrildidae	----------	----------	----------	----------	----------	----------	----------	-
Xenopus_Silurana_tropicalis	----------	----------	----------	----------	----------	----------	----------	-
I_XT	NGNNNAACNG	ATATCTCNGC	ATNTGNNTTG	AAATGGCAAG	ACANTGNCGG	CTNTNNNGNA	CTTANANGNT	1929
Takifugu_rubripes	----------	----------	----------	----------	----------	----------	----------	-
Bos_taurus	----------	----------	----------	----------	----------	----------	----------	-
Homo_sapiens	----------	----------	----------	----------	----------	----------	----------	-
Microcebus_murinus	----------	----------	----------	----------	----------	----------	----------	-
Myotis_lucifugus	----------	----------	----------	----------	----------	----------	----------	-
Equus_caballus	----------	----------	----------	----------	----------	----------	----------	-
Tupaia_belangeri	----------	----------	----------	----------	----------	----------	----------	-
Loxodonta_africana	----------	----------	----------	----------	----------	----------	----------	-
Murinae	----------	----------	----------	----------	----------	----------	----------	-
Canis_lupus_familiaris	----------	----------	----------	----------	----------	----------	----------	-
Sus_scrofa	----------	----------	----------	----------	----------	----------	----------	-
Choloepus_hoffmanni	----------	----------	----------	----------	----------	----------	----------	-
Vicugna_pacos	----------	----------	----------	----------	----------	----------	----------	-
Gavialis_gangeticus	----------	----------	----------	----------	----------	----------	----------	-
Mammalia	----------	----------	----------	----------	----------	----------	----------	-
Passeroidea	----------	----------	----------	----------	----------	----------	----------	-
Takifugu	----------	----------	----------	----------	----------	----------	----------	-
Pteropus_vampyrus	----------	----------	----------	----------	----------	----------	----------	-
Ornithorhynchus	----------	----------	----------	----------	----------	----------	----------	-
Oryctolagus_cuniculus	----------	----------	----------	----------	----------	----------	----------	-
Gallus_gallus	----------	----------	----------	----------	----------	----------	----------	-
Hominidae	----------	----------	----------	----------	----------	----------	----------	-
Metatheria	----------	----------	----------	----------	----------	----------	----------	-
Rodentia	----------	----------	----------	----------	----------	----------	----------	-
Monodelphis	----------	----------	----------	----------	----------	----------	----------	-
Consensus	----------	----------	----------	----------	----------	----------	----------	

Conservation 100% 0%

	2,460		2,480		2,500		2,520	
Dipodomys_ordii	----------	----------	----------	----------	----------	----------	----------	-
Laurasiatheria	----------	----------	----------	----------	----------	----------	----------	-
Eutheria	----------	----------	----------	----------	----------	----------	----------	-
Tarsius_syrichta	----------	----------	----------	----------	----------	----------	----------	-
Muridae	----------	----------	----------	----------	----------	----------	----------	-
Gasterosteus_aculeatus	----------	----------	----------	----------	----------	----------	----------	-
Felis_silvestris	----------	----------	----------	----------	----------	----------	----------	-
Rattus	----------	----------	----------	----------	----------	----------	----------	-
Papio_hamadryas	----------	----------	----------	----------	----------	----------	----------	-
Pan_troglodytes	----------	----------	----------	----------	----------	----------	----------	-
Ailuropoda_melanoleuca	----------	----------	----------	----------	----------	----------	----------	-
Cavia_porcellus	----------	----------	----------	----------	----------	----------	----------	-
Ictidomys_tridecemlineatus	----------	----------	----------	----------	----------	----------	----------	-
Canis	----------	----------	----------	----------	----------	----------	----------	-
Felis_catus	----------	----------	----------	----------	----------	----------	----------	-
Ochotona_princeps	----------	----------	----------	----------	----------	----------	----------	-
Cercopithecidae	----------	----------	----------	----------	----------	----------	----------	-
Carnivora	----------	----------	----------	----------	----------	----------	----------	-
Mus	----------	----------	----------	----------	----------	----------	----------	-
Mus_musculus	----------	----------	----------	----------	----------	----------	----------	-
Rattus_norvegicus	----------	----------	----------	----------	----------	----------	----------	-
Tursiops_truncatus	----------	----------	----------	----------	----------	----------	----------	-
Procavia_capensis	----------	----------	----------	----------	----------	----------	----------	-
Primates	----------	----------	----------	----------	----------	----------	----------	-
Echinops_telfairi	----------	----------	----------	----------	----------	----------	----------	-
Sorex_araneus	----------	----------	----------	----------	----------	----------	----------	-
Canidae	----------	----------	----------	----------	----------	----------	----------	-
Otolemur_garnettii	----------	----------	----------	----------	----------	----------	----------	-
Gallus	----------	----------	----------	----------	----------	----------	----------	-
Callithrix_jacchus	----------	----------	----------	----------	----------	----------	----------	-
Crocodylus_porosus	----------	----------	----------	----------	----------	----------	----------	-
Erinaceus_europaeus	----------	----------	----------	----------	----------	----------	----------	-
rnd-6_family-7426-LTR-ERV1	----------	----------	----------	----------	----------	----------	----------	-
Esox_lucius	----------	----------	----------	----------	----------	----------	----------	-
Salmo_salar	----------	----------	----------	----------	----------	----------	----------	-
Didelphidae	----------	----------	----------	----------	----------	----------	----------	-
Latimeria_chalumnae	----------	----------	----------	----------	----------	----------	----------	-
Monodelphis_domestica	----------	----------	----------	----------	----------	----------	----------	-
Macropus_eugenii	----------	----------	----------	----------	----------	----------	----------	-
Alligator_mississippiensis	----------	----------	----------	----------	----------	----------	----------	-
Chrysemys_picta_bellii	----------	----------	----------	----------	----------	----------	----------	-
Danio_rerio	----------	----------	----------	----------	----------	----------	----------	-
Taeniopygia	----------	----------	----------	----------	----------	----------	----------	-
Dasypus_novemcinctus	----------	----------	----------	----------	----------	----------	----------	-
LTR_XT	----------	----------	----------	----------	----------	----------	----------	-
Haplorrhini	ATGCAAGCAA	AGAGGGCATT	GG-AAAGATG	AATGTCCAGA	GAAAGAAAAG	GATAAAGGCA	ACAACCAGGG	2512
Catarrhini	TTGTAAGGAA	ATAGGACATT	GG-AAAGATA	AGTGTCCC--	-CAACTGAAG	GAAAAGCAAG	GTGATTCGGA	2083
Anolis_carolinensis	----------	----------	----------	----------	----------	----------	----------	-
Murine_leukemia_virus	GCGAAGGAGG	CCC-CAACTT	GATAAGGACC	AATGCGCC--	-TACTGCAAA	GAAAAG---G	GACACTGGGC	2040
Phascolarctos_cinereus	GGGAAGGAAG	ACCGCCTCTG	GACAAAGATC	AGTGCGCA--	-TACTGTAAA	GAAAGG---G	GCCATTGGGC	1961
Estrildidae	----------	----------	----------	----------	----------	----------	----------	-
Xenopus_Silurana_tropicalis	----------	----------	----------	----------	----------	----------	----------	-
I_XT	TAGTNCANAN	GTNTACTGAT	GTATGGGNNC	AAAAAAAGNA	TGAATGTNGT	ATAATAGANG	NTGTGNNGGN	1999
Takifugu_rubripes	----------	----------	----------	----------	----------	----------	----------	-
Bos_taurus	----------	----------	----------	----------	----------	----------	----------	-
Homo_sapiens	----------	----------	----------	----------	----------	----------	----------	-
Microcebus_murinus	----------	----------	----------	----------	----------	----------	----------	-
Myotis_lucifugus	----------	----------	----------	----------	----------	----------	----------	-
Equus_caballus	----------	----------	----------	----------	----------	----------	----------	-
Tupaia_belangeri	----------	----------	----------	----------	----------	----------	----------	-
Loxodonta_africana	----------	----------	----------	----------	----------	----------	----------	-
Murinae	----------	----------	----------	----------	----------	----------	----------	-
Canis_lupus_familiaris	----------	----------	----------	----------	----------	----------	----------	-
Sus_scrofa	----------	----------	----------	----------	----------	----------	----------	-
Choloepus_hoffmanni	----------	----------	----------	----------	----------	----------	----------	-
Vicugna_pacos	----------	----------	----------	----------	----------	----------	----------	-
Gavialis_gangeticus	----------	----------	----------	----------	----------	----------	----------	-
Mammalia	----------	----------	----------	----------	----------	----------	----------	-
Passeroidea	----------	----------	----------	----------	----------	----------	----------	-
Takifugu	----------	----------	----------	----------	----------	----------	----------	-
Pteropus_vampyrus	----------	----------	----------	----------	----------	----------	----------	-
Ornithorhynchus	----------	----------	----------	----------	----------	----------	----------	-
Oryctolagus_cuniculus	----------	----------	----------	----------	----------	----------	----------	-
Gallus_gallus	----------	----------	----------	----------	----------	----------	----------	-
Hominidae	----------	----------	----------	----------	----------	----------	----------	-
Metatheria	----------	----------	----------	----------	----------	----------	----------	-
Rodentia	----------	----------	----------	----------	----------	----------	----------	-
Monodelphis	----------	----------	----------	----------	----------	----------	----------	-
Consensus	----------	----------	----------	----------	----------	----------	----------	

Conservation

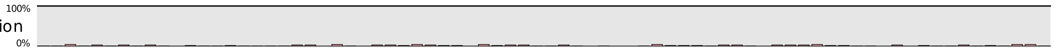

	2,540		2,560		2,580			
Dipodomys_ordii	----------	----------	----------	----------	----------	----------	----------	-
Laurasiatheria	----------	----------	----------	----------	----------	----------	----------	-
Eutheria	----------	----------	----------	----------	----------	----------	----------	-
Tarsius_syrichta	----------	----------	----------	----------	----------	----------	----------	-
Muridae	----------	----------	----------	----------	----------	----------	----------	-
Gasterosteus_aculeatus	----------	----------	----------	----------	----------	----------	----------	-
Felis_silvestris	----------	----------	----------	----------	----------	----------	----------	-
Rattus	----------	----------	----------	----------	----------	----------	----------	-
Papio_hamadryas	----------	----------	----------	----------	----------	----------	----------	-
Pan_troglodytes	----------	----------	----------	----------	----------	----------	----------	-
Ailuropoda_melanoleuca	----------	----------	----------	----------	----------	----------	----------	-
Cavia_porcellus	----------	----------	----------	----------	----------	----------	----------	-
Ictidomys_tridecemlineatus	----------	----------	----------	----------	----------	----------	----------	-
Canis	----------	----------	----------	----------	----------	----------	----------	-
Felis_catus	----------	----------	----------	----------	----------	----------	----------	-
Ochotona_princeps	----------	----------	----------	----------	----------	----------	----------	-
Cercopithecidae	----------	----------	----------	----------	----------	----------	----------	-
Carnivora	----------	----------	----------	----------	----------	----------	----------	-
Mus	----------	----------	----------	----------	----------	----------	----------	-
Mus_musculus	----------	----------	----------	----------	----------	----------	----------	-
Rattus_norvegicus	----------	----------	----------	----------	----------	----------	----------	-
Tursiops_truncatus	----------	----------	----------	----------	----------	----------	----------	-
Procavia_capensis	----------	----------	----------	----------	----------	----------	----------	-
Primates	----------	----------	----------	----------	----------	----------	----------	-
Echinops_telfairi	----------	----------	----------	----------	----------	----------	----------	-
Sorex_araneus	----------	----------	----------	----------	----------	----------	----------	-
Canidae	----------	----------	----------	----------	----------	----------	----------	-
Otolemur_garnettii	----------	----------	----------	----------	----------	----------	----------	-
Gallus	----------	----------	----------	----------	----------	----------	----------	-
Callithrix_jacchus	----------	----------	----------	----------	----------	----------	----------	-
Crocodylus_porosus	----------	----------	----------	----------	----------	----------	----------	-
Erinaceus_europaeus	----------	----------	----------	----------	----------	----------	----------	-
rnd-6_family-7426-LTR-ERV1	----------	----------	----------	----------	----------	----------	----------	-
Esox_lucius	----------	----------	----------	----------	----------	----------	----------	-
Salmo_salar	----------	----------	----------	----------	----------	----------	----------	-
Didelphidae	----------	----------	----------	----------	----------	----------	----------	-
Latimeria_chalumnae	----------	----------	----------	----------	----------	----------	----------	-
Monodelphis_domestica	----------	----------	----------	----------	----------	----------	----------	-
Macropus_eugenii	----------	----------	----------	----------	----------	----------	----------	-
Alligator_mississippiensis	----------	----------	----------	----------	----------	----------	----------	-
Chrysemys_picta_bellii	----------	----------	----------	----------	----------	----------	----------	-
Danio_rerio	----------	----------	----------	----------	----------	----------	----------	-
Taeniopygia	----------	----------	----------	----------	----------	----------	----------	-
Dasypus_novemcinctus	----------	----------	----------	----------	----------	----------	----------	-
LTR_XT	----------	----------	----------	----------	----------	----------	----------	-
Haplorrhini	ACAGAATGGC	TGGACAGGGC	CTCCTTCCGC	CGCTGGGCAG	GGCATAGTAG	GATCCGACGT	GGATCTAATT	2582
Catarrhini	ACAAAA----	-------GAC	CTC-------	----------	-----AGATA	AAGACGAGGG	AGCTTTGTTC	2120
Anolis_carolinensis	----------	----------	----------	----------	----------	----------	----------	-
Murine_leukemia_virus	TAAGGA----	------CTGC	CCAA------	----------	-----AGAAG	CCACGAGGGC	CCCGAGGACC	2079
Phascolarctos_cinereus	AAGGGA----	------GTGC	CCCC------	----------	-----GGAAG	--AAGAACGC	CAGGGAAACC	1998
Estrildidae	----------	----------	----------	----------	----------	----------	----------	-
Xenopus_Silurana_tropicalis	----------	----------	----------	----------	----------	----------	----------	-
I_XT	TGATANTAGG	AANAGATCCT	NACCCANAGA	GGCNGTACGA	CTNNANCCTA	ANGCANTGGN	ACCNTATGNT	2069
Takifugu_rubripes	----------	----------	----------	----------	----------	----------	----------	-
Bos_taurus	----------	----------	----------	----------	----------	----------	----------	-
Homo_sapiens	----------	----------	----------	----------	----------	----------	----------	-
Microcebus_murinus	----------	----------	----------	----------	----------	----------	----------	-
Myotis_lucifugus	----------	----------	----------	----------	----------	----------	----------	-
Equus_caballus	----------	----------	----------	----------	----------	----------	----------	-
Tupaia_belangeri	----------	----------	----------	----------	----------	----------	----------	-
Loxodonta_africana	----------	----------	----------	----------	----------	----------	----------	-
Murinae	----------	----------	----------	----------	----------	----------	----------	-
Canis_lupus_familiaris	----------	----------	----------	----------	----------	----------	----------	-
Sus_scrofa	----------	----------	----------	----------	----------	----------	----------	-
Choloepus_hoffmanni	----------	----------	----------	----------	----------	----------	----------	-
Vicugna_pacos	----------	----------	----------	----------	----------	----------	----------	-
Gavialis_gangeticus	----------	----------	----------	----------	----------	----------	----------	-
Mammalia	----------	----------	----------	----------	----------	----------	----------	-
Passeroidea	----------	----------	----------	----------	----------	----------	----------	-
Takifugu	----------	----------	----------	----------	----------	----------	----------	-
Pteropus_vampyrus	----------	----------	----------	----------	----------	----------	----------	-
Ornithorhynchus	----------	----------	----------	----------	----------	----------	----------	-
Oryctolagus_cuniculus	----------	----------	----------	----------	----------	----------	----------	-
Gallus_gallus	----------	----------	----------	----------	----------	----------	----------	-
Hominidae	----------	----------	----------	----------	----------	----------	----------	-
Metatheria	----------	----------	----------	----------	----------	----------	----------	-
Rodentia	----------	----------	----------	----------	----------	----------	----------	-
Monodelphis	----------	----------	----------	----------	----------	----------	----------	-
Consensus	----------	----------	----------	----------	----------	----------	----------	

Conservation

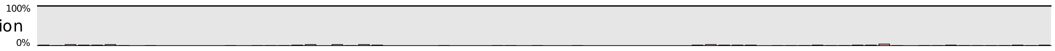

```
                                2,600                          2,620                          2,640                        2,660
              Dipodomys_ordii  ---------- ---------- ---------- ---------- ---------- ---------- ---------- -
                Laurasiatheria  ---------- ---------- ---------- ---------- ---------- ---------- ---------- -
                      Eutheria  ---------- ---------- ---------- ---------- ---------- ---------- ---------- -
               Tarsius_syrichta  ---------- ---------- ---------- ---------- ---------- ---------- ---------- -
                       Muridae  ---------- ---------- ---------- ---------- ---------- ---------- ---------- -
         Gasterosteus_aculeatus  ---------- ---------- ---------- ---------- ---------- ---------- ---------- -
               Felis_silvestris  ---------- ---------- ---------- ---------- ---------- ---------- ---------- -
                        Rattus  ---------- ---------- ---------- ---------- ---------- ---------- ---------- -
               Papio_hamadryas  ---------- ---------- ---------- ---------- ---------- ---------- ---------- -
               Pan_troglodytes  ---------- ---------- ---------- ---------- ---------- ---------- ---------- -
         Ailuropoda_melanoleuca  ---------- ---------- ---------- ---------- ---------- ---------- ---------- -
               Cavia_porcellus  ---------- ---------- ---------- ---------- ---------- ---------- ---------- -
    Ictidomys_tridecemlineatus  ---------- ---------- ---------- ---------- ---------- ---------- ---------- -
                         Canis  ---------- ---------- ---------- ---------- ---------- ---------- ---------- -
                    Felis_catus  ---------- ---------- ---------- ---------- ---------- ---------- ---------- -
              Ochotona_princeps  ---------- ---------- ---------- ---------- ---------- ---------- ---------- -
               Cercopithecidae  ---------- ---------- ---------- ---------- ---------- ---------- ---------- -
                     Carnivora  ---------- ---------- ---------- ---------- ---------- ---------- ---------- -
                           Mus  ---------- ---------- ---------- ---------- ---------- ---------- ---------- -
                   Mus_musculus  ---------- ---------- ---------- ---------- ---------- ---------- ---------- -
              Rattus_norvegicus  ---------- ---------- ---------- ---------- ---------- ---------- ---------- -
             Tursiops_truncatus  ---------- ---------- ---------- ---------- ---------- ---------- ---------- -
              Procavia_capensis  ---------- ---------- ---------- ---------- ---------- ---------- ---------- -
                      Primates  ---------- ---------- ---------- ---------- ---------- ---------- ---------- -
              Echinops_telfairi  ---------- ---------- ---------- ---------- ---------- ---------- ---------- -
                  Sorex_araneus  ---------- ---------- ---------- ---------- ---------- ---------- ---------- -
                       Canidae  ---------- ---------- ---------- ---------- ---------- ---------- ---------- -
              Otolemur_garnettii  ---------- ---------- ---------- ---------- ---------- ---------- ---------- -
                        Gallus  ---------- ---------- ---------- ---------- ---------- ---------- ---------- -
              Callithrix_jacchus  ---------- ---------- ---------- ---------- ---------- ---------- ---------- -
             Crocodylus_porosus  ---------- ---------- ---------- ---------- ---------- ---------- ---------- -
            Erinaceus_europaeus  ---------- ---------- ---------- ---------- ---------- ---------- ---------- -
     rnd-6_family-7426-LTR-ERV1  ---------- ---------- ---------- ---------- ---------- ---------- ---------- -
                    Esox_lucius  ---------- ---------- ---------- ---------- ---------- ---------- ---------- -
                    Salmo_salar  ---------- ---------- ---------- ---------- ---------- ---------- ---------- -
                    Didelphidae  ---------- ---------- ---------- ---------- ---------- ---------- ---------- -
            Latimeria_chalumnae  ---------- ---------- ---------- ---------- ---------- ---------- ---------- -
           Monodelphis_domestica  ---------- ---------- ---------- ---------- ---------- ---------- ---------- -
               Macropus_eugenii  ---------- ---------- ---------- ---------- ---------- ---------- ---------- -
      Alligator_mississippiensis  ---------- ---------- ---------- ---------- ---------- ---------- ---------- -
          Chrysemys_picta_bellii  ---------- ---------- ---------- ---------- ---------- ---------- ---------- -
                    Danio_rerio  ---------- ---------- ---------- ---------- ---------- ---------- ---------- -
                    Taeniopygia  ---------- ---------- ---------- ---------- ---------- ---------- ---------- -
            Dasypus_novemcinctus  ---------- ---------- ---------- ---------- ---------- ---------- ---------- -
                        LTR_XT  ---------- ---------- ---------- ---------- ---------- ---------- ---------- -
                    Haplorrhini  GGGCTGGCAG GAGCCAACGA CTACCTTGAA GACTGAGACA GACCGGGCTC CATCTCATTA GGCCCCGAGG 2652
                    Catarrhini  AATCTGGCTG AAG-----GG CTACT----G GACTGAAGGG GACCGGGCTC AAGCGC---- --CCCCAAGG 2175
              Anolis_carolinensis  ---------- ---------- ---------- ---------- ---------- ---------- ---------- -
           Murine_leukemia_virus  GAGGCCCCAG ACCTCCCTCC TGACCTTAGG TGACTAGGGA GGTCAGGGTC AGGAGCC--- --CCCCCCTG 2144
          Phascolarctos_cinereus  AATG------ -------TCC TGACCCTGGG TGACTAGGGG AGTCGGGGTT CGGACCC--- --CCTCCCCG 2050
                    Estrildidae  ---------- ---------- ---------- ---------- ---------- ---------- ---------- -
    Xenopus_Silurana_tropicalis  ---------- ---------- ---------- ---------- ---------- ---------- ---------- -
                          I_XT  ANAATTANTN NNGANCTNNA AACAGCAGGG TNAATNANTA AAANANAANC CANANNTN-A NNATCCTNNA 2138
              Takifugu_rubripes  ---------- ---------- ---------- ---------- ---------- ---------- ---------- -
                     Bos_taurus  ---------- ---------- ---------- ---------- ---------- ---------- ---------- -
                   Homo_sapiens  ---------- ---------- ---------- ---------- ---------- ---------- ---------- -
             Microcebus_murinus  ---------- ---------- ---------- ---------- ---------- ---------- ---------- -
              Myotis_lucifugus  ---------- ---------- ---------- ---------- ---------- ---------- ---------- -
                 Equus_caballus  ---------- ---------- ---------- ---------- ---------- ---------- ---------- -
               Tupaia_belangeri  ---------- ---------- ---------- ---------- ---------- ---------- ---------- -
              Loxodonta_africana  ---------- ---------- ---------- ---------- ---------- ---------- ---------- -
                       Murinae  ---------- ---------- ---------- ---------- ---------- ---------- ---------- -
          Canis_lupus_familiaris  ---------- ---------- ---------- ---------- ---------- ---------- ---------- -
                     Sus_scrofa  ---------- ---------- ---------- ---------- ---------- ---------- ---------- -
            Choloepus_hoffmanni  ---------- ---------- ---------- ---------- ---------- ---------- ---------- -
                   Vicugna_pacos  ---------- ---------- ---------- ---------- ---------- ---------- ---------- -
             Gavialis_gangeticus  ---------- ---------- ---------- ---------- ---------- ---------- ---------- -
                      Mammalia  ---------- ---------- ---------- ---------- ---------- ---------- ---------- -
                    Passeroidea  ---------- ---------- ---------- ---------- ---------- ---------- ---------- -
                      Takifugu  ---------- ---------- ---------- ---------- ---------- ---------- ---------- -
              Pteropus_vampyrus  ---------- ---------- ---------- ---------- ---------- ---------- ---------- -
                 Ornithorhynchus  ---------- ---------- ---------- ---------- ---------- ---------- ---------- -
          Oryctolagus_cuniculus  ---------- ---------- ---------- ---------- ---------- ---------- ---------- -
                   Gallus_gallus  ---------- ---------- ---------- ---------- ---------- ---------- ---------- -
                     Hominidae  ---------- ---------- ---------- ---------- ---------- ---------- ---------- -
                    Metatheria  ---------- ---------- ---------- ---------- ---------- ---------- ---------- -
                      Rodentia  ---------- ---------- ---------- ---------- ---------- ---------- ---------- -
                    Monodelphis  ---------- ---------- ---------- ---------- ---------- ---------- ---------- -
                     Consensus  ---------- ---------- ---------- ---------- ---------- ---------- ----------
```

Conservation

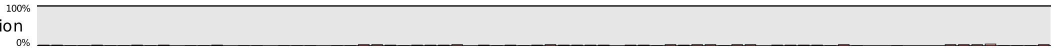

	2,680		2,700			2,720		
Dipodomys_ordii	----------	----------	----------	----------	----------	----------	----------	-
Laurasiatheria	----------	----------	----------	----------	----------	----------	----------	-
Eutheria	----------	----------	----------	----------	----------	----------	----------	-
Tarsius_syrichta	----------	----------	----------	----------	----------	----------	----------	-
Muridae	----------	----------	----------	----------	----------	----------	----------	-
Gasterosteus_aculeatus	----------	----------	----------	----------	----------	----------	----------	-
Felis_silvestris	----------	----------	----------	----------	----------	----------	----------	-
Rattus	----------	----------	----------	----------	----------	----------	----------	-
Papio_hamadryas	----------	----------	-------TGG	GGGCTCGTCC	GGGATTTGAG	AGGCGGCCAG	AGGACACCGG	43
Pan_troglodytes	----------	----------	----------	----------	----------	----------	----------	-
Ailuropoda_melanoleuca	----------	----------	----------	----------	----------	----------	----------	-
Cavia_porcellus	----------	----------	----------	----------	----------	----------	----------	-
Ictidomys_tridecemlineatus	----------	----------	----------	----------	----------	----------	----------	-
Canis	----------	----------	----------	----------	----------	----------	----------	-
Felis_catus	----------	----------	----------	----------	----------	----------	----------	-
Ochotona_princeps	----------	----------	----------	----------	----------	----------	----------	-
Cercopithecidae	----------	----------	----------	----------	----------	----------	----------	-
Carnivora	----------	----------	----------	----------	----------	----------	----------	-
Mus	----------	----------	----------	----------	----------	----------	----------	-
Mus_musculus	----------	----------	----------	----------	----------	----------	----------	-
Rattus_norvegicus	----------	----------	----------	----------	----------	----------	----------	-
Tursiops_truncatus	----------	----------	----------	----------	----------	----------	----------	-
Procavia_capensis	----------	----------	----------	----------	----------	----------	----------	-
Primates	----------	----------	----------	----------	----------	----------	----------	-
Echinops_telfairi	----------	----------	----------	----------	----------	----------	----------	-
Sorex_araneus	----------	----------	----------	----------	----------	----------	----------	-
Canidae	----------	----------	----------	----------	----------	----------	----------	-
Otolemur_garnettii	----------	----------	----------	----------	----------	----------	----------	-
Gallus	----------	----------	----------	----------	----------	----------	----------	-
Callithrix_jacchus	----------	----------	----------	----------	----------	----------	----------	-
Crocodylus_porosus	----------	----------	----------	----------	----------	----------	----------	-
Erinaceus_europaeus	----------	----------	----------	----------	----------	----------	----------	-
rnd-6_family-7426-LTR-ERV1	----------	----------	----------	----------	----------	----------	----------	-
Esox_lucius	----------	----------	----------	----------	----------	----------	----------	-
Salmo_salar	----------	----------	----------	----------	----------	----------	----------	-
Didelphidae	----------	----------	----------	----------	----------	----------	----------	-
Latimeria_chalumnae	----------	----------	----------	----------	----------	----------	----------	-
Monodelphis_domestica	----------	----------	----------	----------	----------	----------	----------	-
Macropus_eugenii	----------	----------	----------	----------	----------	----------	----------	-
Alligator_mississippiensis	----------	----------	----------	----------	----------	----------	----------	-
Chrysemys_picta_bellii	----------	----------	----------	----------	----------	----------	----------	-
Danio_rerio	----------	----------	----------	----------	----------	----------	----------	-
Taeniopygia	----------	----------	----------	----------	----------	----------	----------	-
Dasypus_novemcinctus	----------	----------	----------	----------	----------	----------	----------	-
LTR_XT	----------	----------	----------	----------	----------	----------	----------	-
Haplorrhini	AGCCCATGGT	CTCAATGGTA	GTAGGGGGCC	GAAAAACAGA	CTTTATGGTA	GACACAGGTG	CTGAACACTC	2722
Catarrhini	AGCCCACGGT	CAGGATTACA	ACTGGGGGCA	AGGACATTAA	GTTTTTGGTC	GATACTGGTG	CTGAACATTC	2245
Anolis_carolinensis	----------	----------	----------	----------	----------	----------	----------	-
Murine_leukemia_virus	AACCCAGGAT	AACCCTCAAA	GTCGGGGGGC	AACCCGTCAC	CTTCCTGGTA	GATACTGGGG	CCCAACACTC	2214
Phascolarctos_cinereus	AGCCCAGGGT	AACGTTAACT	GTGGAGGGGA	TCCCCACTGA	GTTTTTGGTT	GATACCGGGG	CCGAACATTC	2120
Estrildidae	----------	----------	----------	----------	----------	----------	----------	-
Xenopus_Silurana_tropicalis	----------	----------	----------	----------	----------	----------	----------	-
I_XT	TGGCCAGTAA	AAAANNNNGA	NGGTANATGG	NGANTNACNN	TTGNTNAAAG	GATGNTNAAT	NAAGNACCCC	2208
Takifugu_rubripes	----------	----------	----------	----------	----------	----------	----------	-
Bos_taurus	----------	----------	----------	----------	----------	----------	----------	-
Homo_sapiens	----------	----------	----------	----------	----------	----------	----------	-
Microcebus_murinus	----------	----------	----------	----------	----------	----------	----------	-
Myotis_lucifugus	----------	----------	----------	----------	----------	----------	----------	-
Equus_caballus	----------	----------	----------	----------	----------	----------	----------	-
Tupaia_belangeri	----------	----------	----------	----------	----------	----------	----------	-
Loxodonta_africana	----------	----------	----------	----------	----------	----------	----------	-
Murinae	----------	----------	----------	----------	----------	----------	----------	-
Canis_lupus_familiaris	----------	----------	----------	----------	----------	----------	----------	-
Sus_scrofa	----------	----------	----------	----------	----------	----------	----------	-
Choloepus_hoffmanni	----------	----------	----------	----------	----------	----------	----------	-
Vicugna_pacos	----------	----------	----------	----------	----------	----------	----------	-
Gavialis_gangeticus	----------	----------	----------	----------	----------	----------	----------	-
Mammalia	----------	----------	----------	----------	----------	----------	----------	-
Passeroidea	----------	----------	----------	----------	----------	----------	----------	-
Takifugu	----------	----------	----------	----------	----------	----------	----------	-
Pteropus_vampyrus	----------	----------	----------	----------	----------	----------	----------	-
Ornithorhynchus	----------	----------	----------	----------	----------	----------	----------	-
Oryctolagus_cuniculus	----------	----------	----------	----------	----------	----------	----------	-
Gallus_gallus	----------	----------	----------	----------	----------	----------	----------	-
Hominidae	----------	----------	----------	----------	----------	----------	----------	-
Metatheria	----------	----------	----------	----------	----------	----------	----------	-
Rodentia	----------	----------	----------	----------	----------	----------	----------	-
Monodelphis	----------	----------	----------	----------	----------	----------	----------	-
Consensus	----------	----------	----------	----------	----------	----------	----------	

Conservation 100% 0%

	2,740		2,760		2,780		2,800	
Dipodomys_ordii	----------	----------	----------	----------	----------	----------	----------	-
Laurasiatheria	----------	----------	----------	----------	----------	----------	----------	-
Eutheria	----------	----------	----------	----------	----------	----------	----------	-
Tarsius_syrichta	----------	----------	----------	----------	----------	----------	----------	-
Muridae	----------	----------	----------	----------	----------	----------	----------	-
Gasterosteus_aculeatus	----------	----------	----------	----------	----------	----------	----------	-
Felis_silvestris	----------	----------	----------	----------	----------	----------	----------	-
Rattus	----------	----------	----------	----------	----------	----------	----------	-
Papio_hamadryas	CCCCTTTTCC	TTTTCGGCAG	AAACCGCGCG	GCCCCGGCCA	CCGGTGGCGG	ACGGACGACG	GGACGACTCT	113
Pan_troglodytes	----------	----------	----------	----------	----------	----------	----------	-
Ailuropoda_melanoleuca	----------	----------	----------	----------	----------	----------	----------	-
Cavia_porcellus	----------	----------	----------	----------	----------	----------	----------	-
Ictidomys_tridecemlineatus	----------	----------	----------	----------	----------	----------	----------	-
Canis	----------	----------	----------	----------	----------	----------	----------	-
Felis_catus	----------	----------	----------	----------	----------	----------	----------	-
Ochotona_princeps	----------	----------	----------	----------	----------	----------	----------	-
Cercopithecidae	----------	----------	----------	----------	----------	----------	----------	-
Carnivora	----------	----------	----------	----------	----------	----------	----------	-
Mus	----------	----------	----------	----------	----------	----------	----------	-
Mus_musculus	----------	----------	----------	----------	----------	----------	----------	-
Rattus_norvegicus	----------	----------	----------	----------	----------	----------	----------	-
Tursiops_truncatus	----------	----------	----------	----------	----------	----------	----------	-
Procavia_capensis	----------	----------	----------	----------	----------	----------	----------	-
Primates	----------	----------	----------	----------	----------	----------	----------	-
Echinops_telfairi	----------	----------	----------	----------	----------	----------	----------	-
Sorex_araneus	----------	----------	----------	----------	----------	----------	----------	-
Canidae	----------	----------	----------	----------	----------	----------	----------	-
Otolemur_garnettii	----------	----------	----------	----------	----------	----------	----------	-
Gallus	----------	----------	----------	----------	----------	----------	----------	-
Callithrix_jacchus	----------	----------	----------	----------	----------	----------	----------	-
Crocodylus_porosus	----------	----------	----------	----------	----------	----------	----------	-
Erinaceus_europaeus	----------	----------	----------	----------	----------	----------	----------	-
rnd-6_family-7426-LTR-ERV1	----------	----------	----------	----------	----------	----------	----------	-
Esox_lucius	----------	----------	----------	----------	----------	----------	----------	-
Salmo_salar	----------	----------	----------	----------	----------	----------	----------	-
Didelphidae	----------	----------	----------	----------	----------	----------	----------	-
Latimeria_chalumnae	----------	----------	----------	----------	----------	----------	----------	-
Monodelphis_domestica	----------	----------	----------	----------	----------	----------	----------	-
Macropus_eugenii	----------	----------	----------	----------	----------	----------	----------	-
Alligator_mississippiensis	----------	----------	----------	----------	----------	----------	----------	-
Chrysemys_picta_bellii	----------	----------	----------	----------	----------	----------	----------	-
Danio_rerio	----------	----------	----------	----------	----------	----------	----------	-
Taeniopygia	----------	----------	----------	----------	----------	----------	----------	-
Dasypus_novemcinctus	----------	----------	----------	----------	----------	----------	----------	-
LTR_XT	----------	----------	----------	----------	----------	----------	----------	-
Haplorrhini	GGTTGTGACT	CAAACAATTG	GGCCGTTATC	AAAAGACTAT	GCTAATATTA	TCGGGGCCAC	AGGTATCACA	2792
Catarrhini	AGTAGTGACC	ACCCCGGTCG	CCCCCTTATC	CAAGAAAACC	ATTGATATAA	TCGGAGCAAC	AGGAGTTTCC	2315
Anolis_carolinensis	----------	----------	----------	----------	----------	----------	----------	-
Murine_leukemia_virus	CGTGCTGACC	CAAAATCCTG	GACCCCTAAG	TGACAAGTCT	GCCTGGGTCC	AAGGGGCTAC	TGGAG--GAA	2282
Phascolarctos_cinereus	AGTATTGACC	AAGCCTATGG	GAAAGATGGG	ATCCAAACGG	ACAGTCGTGG	CTGGGGCGAC	GGGCA--GCA	2188
Estrildidae	----------	----------	----------	----------	----------	----------	----------	-
Xenopus_Silurana_tropicalis	----------	----------	----------	----------	----------	----------	----------	-
I_XT	TNCTNNNGCN	CNACTTGTNG	CAGANACTNC	AGNNACANTN	TCAAANNTNA	CANCANNTGC	AAACTNTTAT	2278
Takifugu_rubripes	----------	----------	----------	----------	----------	----------	----------	-
Bos_taurus	----------	----------	----------	----------	----------	----------	----------	-
Homo_sapiens	----------	----------	----------	----------	----------	----------	----------	-
Microcebus_murinus	----------	----------	----------	----------	----------	----------	----------	-
Myotis_lucifugus	----------	----------	----------	----------	----------	----------	----------	-
Equus_caballus	----------	----------	----------	----------	----------	----------	----------	-
Tupaia_belangeri	----------	----------	----------	----------	----------	----------	----------	-
Loxodonta_africana	----------	----------	----------	----------	----------	----------	----------	-
Murinae	----------	----------	----------	----------	----------	----------	----------	-
Canis_lupus_familiaris	----------	----------	----------	----------	----------	----------	----------	-
Sus_scrofa	----------	----------	----------	----------	----------	----------	----------	-
Choloepus_hoffmanni	----------	----------	----------	----------	----------	----------	----------	-
Vicugna_pacos	----------	----------	----------	----------	----------	----------	----------	-
Gavialis_gangeticus	----------	----------	----------	----------	----------	----------	----------	-
Mammalia	----------	----------	----------	----------	----------	----------	----------	-
Passeroidea	----------	----------	----------	----------	----------	----------	----------	-
Takifugu	----------	----------	----------	----------	----------	----------	----------	-
Pteropus_vampyrus	----------	----------	----------	----------	----------	----------	----------	-
Ornithorhynchus	----------	----------	----------	----------	----------	----------	----------	-
Oryctolagus_cuniculus	----------	----------	----------	----------	----------	----------	----------	-
Gallus_gallus	----------	----------	----------	----------	----------	----------	----------	-
Hominidae	----------	----------	----------	----------	----------	----------	----------	-
Metatheria	----------	----------	----------	----------	----------	----------	----------	-
Rodentia	----------	----------	----------	----------	----------	----------	----------	-
Monodelphis	----------	----------	----------	----------	----------	----------	----------	-
Consensus	----------	----------	----------	----------	----------	----------	----------	

Conservation 100% 0%

```
                                           2,820                         2,840                         2,860
Dipodomys_ordii                ---------- ---------- ---------- ---------- ---------- ---------- ---------- -
Laurasiatheria                 ---------- ---------- ---------- ---------- ---------- ---------- ---------- -
Eutheria                       ---------- ---------- ---------- ---------- ---------- ---------- ---------- -
Tarsius_syrichta               ---------- ---------- ---------- ---------- ---------- ---------- ---------- -
Muridae                        ---------- ---------- ---------- ---------- ---------- ---------- ---------- -
Gasterosteus_aculeatus         ---------- ---------- ---------- ---------- ---------- ---------- ---------- -
Felis_silvestris               ---------- ---------- ---------- ---------- ---------- ---------- ---------- -
Rattus                         ---------- ---------- ---------- ---------- ---------- ---------- ---------- -
Papio_hamadryas                TGTGTGTAGT CAGGTACTTT ATTTTTGTTC TGTCTTTAAT CTCTGAGGTC GGCCAACCTT CGTAGGAGTG 183
Pan_troglodytes                ---------- ---------- ---------- ---------- ---------- ---------- ---------- -
Ailuropoda_melanoleuca         ---------- ---------- ---------- ---------- ---------- ---------- ---------- -
Cavia_porcellus                ---------- ---------- ---------- ---------- ---------- ---------- ---------- -
Ictidomys_tridecemlineatus     ---------- ---------- ---------- ---------- ---------- ---------- ---------- -
Canis                          ---------- ---------- ---------- ---------- ---------- ---------- ---------- -
Felis_catus                    ---------- ---------- ---------- ---------- ---------- ---------- ---------- -
Ochotona_princeps              ---------- ---------- ---------- ---------- ---------- ---------- ---------- -
Cercopithecidae                ---------- ---------- ---------- ---------- ---------- ---------- ---------- -
Carnivora                      ---------- ---------- ---------- ---------- ---------- ---------- ---------- -
Mus                            ---------- ---------- ---------- ---------- ---------- ---------- ---------- -
Mus_musculus                   ---------- ---------- ---------- ---------- ---------- ---------- ---------- -
Rattus_norvegicus              ---------- ---------- ---------- ---------- ---------- ---------- ---------- -
Tursiops_truncatus             ---------- ---------- ---------- ---------- ---------- ---------- ---------- -
Procavia_capensis              ---------- ---------- ---------- ---------- ---------- ---------- ---------- -
Primates                       ---------- ---------- ---------- ---------- ---------- ---------- ---------- -
Echinops_telfairi              ---------- ---------- ---------- ---------- ---------- ---------- ---------- -
Sorex_araneus                  ---------- ---------- ---------- ---------- ---------- ---------- ---------- -
Canidae                        ---------- ---------- ---------- ---------- ---------- ---------- ---------- -
Otolemur_garnettii             ---------- ---------- ---------- ---------- ---------- ---------- ---------- -
Gallus                         ---------- ---------- ---------- ---------- ---------- ---------- ---------- -
Callithrix_jacchus             ---------- ---------- ---------- ---------- ---------- ---------- ---------- -
Crocodylus_porosus             ---------- ---------- ---------- ---------- ---------- ---------- ---------- -
Erinaceus_europaeus            ---------- ---------- ---------- ---------- ---------- ---------- ---------- -
rnd-6_family-7426-LTR-ERV1     ---------- ---------- ---------- ---------- ---------- ---------- ---------- -
Esox_lucius                    ---------- ---------- ---------- ---------- ---------- ---------- ---------- -
Salmo_salar                    ---------- ---------- ---------- ---------- ---------- ---------- ---------- -
Didelphidae                    ---------- ---------- ---------- ---------- ---------- ---------- ---------- -
Latimeria_chalumnae            ---------- ---------- ---------- ---------- ---------- ---------- ---------- -
Monodelphis_domestica          ---------- ---------- ---------- ---------- ---------- ---------- ---------- -
Macropus_eugenii               ---------- ---------- ---------- ---------- ---------- ---------- ---------- -
Alligator_mississippiensis     ---------- ---------- ---------- ---------- ---------- ---------- ---------- -
Chrysemys_picta_bellii         ---------- ---------- ---------- ---------- ---------- ---------- ---------- -
Danio_rerio                    ---------- ---------- ---------- ---------- ---------- ---------- ---------- -
Taeniopygia                    ---------- ---------- ---------- ---------- ---------- ---------- ---------- -
Dasypus_novemcinctus           ---------- ---------- ---------- ---------- ---------- ---------- ---------- -
LTR_XT                         ---------- ---------- ---------- ---------- ---------- ---------- ---------- -
Haplorrhini                    GAAAAGACAC CTTTTTTCAA ATCAAAGAGA TGTATGATTG GAGACCAAGA AGTCCAACAC AAGTTCTTAT 2862
Catarrhini                     ACTAAGCAGG CTTTCTGTCT ACCACGGACC TGCTCGGTGG GGGGACGTGA GATAGTTCAC CAGTTCTTGT 2385
Anolis_carolinensis            ---------- ---------- ---------- ---------- ---------- ---------- ---------- -
Murine_leukemia_virus          AGCGGTATCG CTGGACCACG GATCGCAAAG T-ACATCTAG CTACCGGTAA GGTCACCCAC TCTTTCCTCC 2351
Phascolarctos_cinereus         AAGTTTACCC TTGGACCACC AAGAGACTTT TGAAAATTGG ACAAAAGCAA G-TGACCCAC TCATTCTTGG 2257
Estrildidae                    ---------- ---------- ---------- ---------- ---------- ---------- ---------- -
Xenopus_Silurana_tropicalis    ---------- ---------- ---------- ---------- ---------- ---------- ---------- -
I_XT                           TCAAANATTG ANATNGANAN NANNTNTTTT AGGNTAGNAG TGNNCNANGT ATGTCNATAT AAAANGGCAT 2348
Takifugu_rubripes              ---------- ---------- ---------- ---------- ---------- ---------- ---------- -
Bos_taurus                     ---------- ---------- ---------- ---------- ---------- ---------- ---------- -
Homo_sapiens                   ---------- ---------- ---------- ---------- ---------- ---------- ---------- -
Microcebus_murinus             ---------- ---------- ---------- ---------- ---------- ---------- ---------- -
Myotis_lucifugus               ---------- ---------- ---------- ---------- ---------- ---------- ---------- -
Equus_caballus                 ---------- ---------- ---------- ---------- ---------- ---------- ---------- -
Tupaia_belangeri               ---------- ---------- ---------- ---------- ---------- ---------- ---------- -
Loxodonta_africana             ---------- ---------- ---------- ---------- ---------- ---------- ---------- -
Murinae                        ---------- ---------- ---------- ---------- ---------- ---------- ---------- -
Canis_lupus_familiaris         ---------- ---------- ---------- ---------- ---------- ---------- ---------- -
Sus_scrofa                     ---------- ---------- ---------- ---------- ---------- ---------- ---------- -
Choloepus_hoffmanni            ---------- ---------- ---------- ---------- ---------- ---------- ---------- -
Vicugna_pacos                  ---------- ---------- ---------- ---------- ---------- ---------- ---------- -
Gavialis_gangeticus            ---------- ---------- ---------- ---------- ---------- ---------- ---------- -
Mammalia                       ---------- ---------- ---------- ---------- ---------- ---------- ---------- -
Passeroidea                    ---------- ---------- ---------- ---------- ---------- ---------- ---------- -
Takifugu                       ---------- ---------- ---------- ---------- ---------- ---------- ---------- -
Pteropus_vampyrus              ---------- ---------- ---------- ---------- ---------- ---------- ---------- -
Ornithorhynchus                ---------- ---------- ---------- ---------- ---------- ---------- ---------- -
Oryctolagus_cuniculus          ---------- ---------- ---------- ---------- ---------- ---------- ---------- -
Gallus_gallus                  ---------- ---------- ---------- ---------- ---------- ---------- ---------- -
Hominidae                      ---------- ---------- ---------- ---------- ---------- ---------- ---------- -
Metatheria                     ---------- ---------- ---------- ---------- ---------- ---------- ---------- -
Rodentia                       ---------- ---------- ---------- ---------- ---------- ---------- ---------- -
Monodelphis                    ---------- ---------- ---------- ---------- ---------- ---------- ---------- -
Consensus                      ---------- ---------- ---------- ---------- ---------- ---------- ----------
```

100%
Conservation
0%

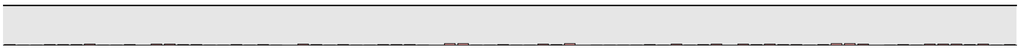

2,880 2,900 2,920 2,940

Dipodomys_ordii	----------	----------	----------	----------	----------	----------	----------	-
Laurasiatheria	----------	----------	----------	----------	----------	----------	----------	-
Eutheria	----------	----------	----------	----------	----------	----------	----------	-
Tarsius_syrichta	----------	----------	----------	----------	----------	----------	----------	-
Muridae	----------	----------	----------	----------	----------	----------	----------	-
Gasterosteus_aculeatus	----------	----------	----------	----------	----------	----------	----------	-
Felis_silvestris	----------	----------	----------	----------	----------	----------	----------	-
Rattus	----------	----------	----------	----------	----------	----------	----------	-
Papio_hamadryas	TAGAGGGAGG	ACAGACGTGT	CCTGGACCCT	CACACTCCGA	CCCCGGGGGA	CGCCCCGGCG	GTCGTCTGGA	253
Pan_troglodytes	----------	----------	----------	----------	----------	----------	----------	-
Ailuropoda_melanoleuca	----------	----------	----------	----------	----------	----------	----------	-
Cavia_porcellus	----------	----------	----------	----------	----------	----------	----------	-
Ictidomys_tridecemlineatus	----------	----------	----------	----------	----------	----------	----------	-
Canis	----------	----------	----------	----------	----------	----------	----------	-
Felis_catus	----------	----------	----------	----------	----------	----------	----------	-
Ochotona_princeps	----------	----------	----------	----------	----------	----------	----------	-
Cercopithecidae	----------	----------	----------	----------	----------	----------	----------	-
Carnivora	----------	----------	----------	----------	----------	----------	----------	-
Mus	----------	----------	----------	----------	----------	----------	----------	-
Mus_musculus	----------	----------	----------	----------	----------	----------	----------	-
Rattus_norvegicus	----------	----------	----------	----------	----------	----------	----------	-
Tursiops_truncatus	----------	----------	----------	----------	----------	----------	----------	-
Procavia_capensis	----------	----------	----------	----------	----------	----------	----------	-
Primates	----------	----------	----------	----------	----------	----------	----------	-
Echinops_telfairi	----------	----------	----------	----------	----------	----------	----------	-
Sorex_araneus	----------	----------	----------	----------	----------	----------	----------	-
Canidae	----------	----------	----------	----------	----------	----------	----------	-
Otolemur_garnettii	----------	----------	----------	----------	----------	----------	----------	-
Gallus	----------	----------	----------	----------	----------	----------	----------	-
Callithrix_jacchus	----------	----------	----------	----------	----------	----------	----------	-
Crocodylus_porosus	----------	----------	----------	----------	----------	----------	----------	-
Erinaceus_europaeus	----------	----------	----------	----------	----------	----------	----------	-
rnd-6_family-7426-LTR-ERV1	----------	----------	----------	----------	----------	----------	----------	-
Esox_lucius	----------	----------	----------	----------	----------	----------	----------	-
Salmo_salar	----------	----------	----------	----------	----------	----------	----------	-
Didelphidae	----------	----------	----------	----------	----------	----------	----------	-
Latimeria_chalumnae	----------	----------	----------	----------	----------	----------	----------	-
Monodelphis_domestica	----------	----------	----------	----------	----------	----------	----------	-
Macropus_eugenii	----------	----------	----------	----------	----------	----------	----------	-
Alligator_mississippiensis	----------	----------	----------	----------	----------	----------	----------	-
Chrysemys_picta_bellii	----------	----------	----------	----------	----------	----------	----------	-
Danio_rerio	----------	----------	----------	----------	----------	----------	----------	-
Taeniopygia	----------	----------	----------	----------	----------	----------	----------	-
Dasypus_novemcinctus	----------	----------	----------	----------	----------	----------	----------	-
LTR_XT	----------	----------	----------	----------	----------	----------	----------	-
Haplorrhini	ACTTGCCAAA	CTGCCCAGTG	CCGCTGTTGG	GAAGAGACTT	GCTGCAGAAG	CTGCAGGCTC	AGATCTCCTT	2932
Catarrhini	ACATGCCTGA	CTGTCCCTTG	CCCTTGCTGG	GAAGAGACTT	GCTTAGCAAG	CTGAGAGCCA	CCATCTCCTT	2455
Anolis_carolinensis	----------	----------	----------	----------	----------	----------	----------	-
Murine_leukemia_virus	ATGTACCAGA	CTGCCCCTAT	CCTCTGCTAG	GAAGAGACTT	GCTGACTAAA	CTAAAAGCCC	AGATCCACTT	2421
Phascolarctos_cinereus	TCATACCCGA	ATGCCCTGCT	CCCCTGTTAG	GCAGGGACCT	CCTCACCAAG	CTAAAAGCTC	AAATCCAGTT	2327
Estrildidae	----------	----------	----------	----------	----------	----------	----------	-
Xenopus_Silurana_tropicalis	----------	----------	----------	----------	----------	----------	----------	-
I_XT	TGACTAGNAT	CANCANTNTG	ACTNCTNANG	ACTACTCCAA	GGTTTGTACA	NNNCNNCCNC	GTTNTNTCAT	2418
Takifugu_rubripes	----------	----------	----------	----------	----------	----------	----------	-
Bos_taurus	----------	----------	----------	----------	----------	----------	----------	-
Homo_sapiens	----------	----------	----------	----------	----------	----------	----------	-
Microcebus_murinus	----------	----------	----------	----------	----------	----------	----------	-
Myotis_lucifugus	----------	----------	----------	----------	----------	----------	----------	-
Equus_caballus	----------	----------	----------	----------	----------	----------	----------	-
Tupaia_belangeri	----------	----------	----------	----------	----------	----------	----------	-
Loxodonta_africana	----------	----------	----------	----------	----------	----------	----------	-
Murinae	----------	----------	----------	----------	----------	----------	----------	-
Canis_lupus_familiaris	----------	----------	----------	----------	----------	----------	----------	-
Sus_scrofa	----------	----------	----------	----------	----------	----------	----------	-
Choloepus_hoffmanni	----------	----------	----------	----------	----------	----------	----------	-
Vicugna_pacos	----------	----------	----------	----------	----------	----------	----------	-
Gavialis_gangeticus	----------	----------	----------	----------	----------	----------	----------	-
Mammalia	----------	----------	----------	----------	----------	----------	----------	-
Passeroidea	----------	----------	----------	----------	----------	----------	----------	-
Takifugu	----------	----------	----------	----------	----------	----------	----------	-
Pteropus_vampyrus	----------	----------	----------	----------	----------	----------	----------	-
Ornithorhynchus	----------	----------	----------	----------	----------	----------	----------	-
Oryctolagus_cuniculus	----------	----------	----------	----------	----------	----------	----------	-
Gallus_gallus	----------	----------	----------	----------	----------	----------	----------	-
Hominidae	----------	----------	----------	----------	----------	----------	----------	-
Metatheria	----------	----------	----------	----------	----------	----------	----------	-
Rodentia	----------	----------	----------	----------	----------	----------	----------	-
Monodelphis	----------	----------	----------	----------	----------	----------	----------	-
Consensus	----------	----------	----------	----------	----------	----------	----------	

Conservation

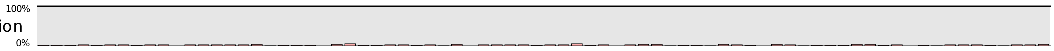

		2,960		2,980		3,000		
Dipodomys_ordii	----------	----------	----------	----------	----------	----------	----------	-
Laurasiatheria	----------	----------	----------	----------	----------	----------	----------	-
Eutheria	----------	----------	----------	----------	----------	----------	----------	-
Tarsius_syrichta	----------	----------	----------	----------	----------	----------	----------	-
Muridae	----------	----------	----------	----------	----------	----------	----------	-
Gasterosteus_aculeatus	----------	----------	----------	----------	----------	----------	----------	-
Felis_silvestris	----------	----------	----------	----------	----------	----------	----------	-
Rattus	----------	----------	----------	----------	----------	----------	----------	-
Papio_hamadryas	GGAAGGCTGA	TGACACCGTC	AGCCTCCTCA	AATCTGAAGG	CAGGTTCCCC	CTGCCATCTG	AATCACTTGT	323
Pan_troglodytes	----------	----------	----------	----------	----------	----------	----------	-
Ailuropoda_melanoleuca	----------	----------	----------	----------	----------	----------	----------	-
Cavia_porcellus	----------	----------	----------	----------	----------	----------	----------	-
Ictidomys_tridecemlineatus	----------	----------	----------	----------	----------	----------	----------	-
Canis	----------	----------	----------	----------	----------	----------	----------	-
Felis_catus	----------	----------	----------	----------	----------	----------	----------	-
Ochotona_princeps	----------	----------	----------	----------	----------	----------	----------	-
Cercopithecidae	----------	----------	----------	----------	----------	----------	----------	-
Carnivora	----------	----------	----------	----------	----------	----------	----------	-
Mus	----------	----------	----------	----------	----------	----------	----------	-
Mus_musculus	----------	----------	----------	----------	----------	----------	----------	-
Rattus_norvegicus	----------	----------	----------	----------	----------	----------	----------	-
Tursiops_truncatus	----------	----------	----------	----------	----------	----------	----------	-
Procavia_capensis	----------	----------	----------	----------	----------	----------	----------	-
Primates	----------	----------	----------	----------	----------	----------	----------	-
Echinops_telfairi	----------	----------	----------	----------	----------	----------	----------	-
Sorex_araneus	----------	----------	----------	----------	----------	----------	----------	-
Canidae	----------	----------	----------	----------	----------	----------	----------	-
Otolemur_garnettii	----------	----------	----------	----------	----------	----------	----------	-
Gallus	----------	----------	----------	----------	----------	----------	----------	-
Callithrix_jacchus	----------	----------	----------	----------	----------	----------	----------	-
Crocodylus_porosus	----------	----------	----------	----------	----------	----------	----------	-
Erinaceus_europaeus	----------	----------	----------	----------	----------	----------	----------	-
rnd-6_family-7426-LTR-ERV1	----------	----------	----------	----------	----------	----------	----------	-
Esox_lucius	----------	----------	----------	----------	----------	----------	----------	-
Salmo_salar	----------	----------	----------	----------	----------	----------	----------	-
Didelphidae	----------	----------	----------	----------	----------	----------	----------	-
Latimeria_chalumnae	----------	----------	----------	----------	----------	----------	----------	-
Monodelphis_domestica	----------	----------	----------	----------	----------	----------	----------	-
Macropus_eugenii	----------	----------	----------	----------	----------	----------	----------	-
Alligator_mississippiensis	----------	----------	----------	----------	----------	----------	----------	-
Chrysemys_picta_bellii	----------	----------	----------	----------	----------	----------	----------	-
Danio_rerio	----------	----------	----------	----------	----------	----------	----------	-
Taeniopygia	----------	----------	----------	----------	----------	----------	----------	-
Dasypus_novemcinctus	----------	----------	----------	----------	----------	----------	----------	-
LTR_XT	----------	----------	----------	----------	----------	----------	----------	-
Haplorrhini	CACACCGAGT	GGAGATATGA	CCTTGAGCCT	AGGGCAAAAA	AAGGCTATGG	TACTAACNCT	CACAGTNCCC	3002
Catarrhini	TACAAAACAG	GGCTCTTTAC	AGCTAAAGTT	ACCGGGAACA	GGAGTTATCA	TG---GCCCT	TACGGTCCCC	2522
Anolis_carolinensis	----------	----------	----------	----------	----------	----------	----------	-
Murine_leukemia_virus	CGAGGGATCG	GGAGCTCAGG	TTGTGGGACC	AATGGGACAG	CCCCTGCAAG	TGCTGA-CCC	TAAA-----C	2485
Phascolarctos_cinereus	TTCCACAGAG	GGCCCACAGG	TAACATGG--	--GAAGACCG	CCCCGCCATG	TGTTTAGTCC	TGAA-----C	2388
Estrildidae	----------	----------	----------	----------	----------	----------	----------	-
Xenopus_Silurana_tropicalis	----------	----------	----------	----------	----------	----------	----------	-
I_XT	ATCNCNCTGG	CAGANATNCT	AGNANNGTTN	TCAAGNCNAG	AATGCTTNCT	ACAATATGTN	GACGACNTNC	2488
Takifugu_rubripes	----------	----------	----------	----------	----------	----------	----------	-
Bos_taurus	----------	----------	----------	----------	----------	----------	----------	-
Homo_sapiens	----------	----------	----------	----------	----------	----------	----------	-
Microcebus_murinus	----------	----------	----------	----------	----------	----------	----------	-
Myotis_lucifugus	----------	----------	----------	----------	----------	----------	----------	-
Equus_caballus	----------	----------	----------	----------	----------	----------	----------	-
Tupaia_belangeri	----------	----------	----------	----------	----------	----------	----------	-
Loxodonta_africana	----------	----------	----------	----------	----------	----------	----------	-
Murinae	----------	----------	----------	----------	----------	----------	----------	-
Canis_lupus_familiaris	----------	----------	----------	----------	----------	----------	----------	-
Sus_scrofa	----------	----------	----------	----------	----------	----------	----------	-
Choloepus_hoffmanni	----------	----------	----------	----------	----------	----------	----------	-
Vicugna_pacos	----------	----------	----------	----------	----------	----------	----------	-
Gavialis_gangeticus	----------	----------	----------	----------	----------	----------	----------	-
Mammalia	----------	----------	----------	----------	----------	----------	----------	-
Passeroidea	----------	----------	----------	----------	----------	----------	----------	-
Takifugu	----------	----------	----------	----------	----------	----------	----------	-
Pteropus_vampyrus	----------	----------	----------	----------	----------	----------	----------	-
Ornithorhynchus	----------	----------	----------	----------	----------	----------	----------	-
Oryctolagus_cuniculus	----------	----------	----------	----------	----------	----------	----------	-
Gallus_gallus	----------	----------	----------	----------	----------	----------	----------	-
Hominidae	----------	----------	----------	----------	----------	----------	----------	-
Metatheria	----------	----------	----------	----------	----------	----------	----------	-
Rodentia	----------	----------	----------	----------	----------	----------	----------	-
Monodelphis	----------	----------	----------	----------	----------	----------	----------	-
Consensus	----------	----------	----------	----------	----------	----------	----------	
Conservation (100% – 0%)								

3,020 | 3,040 | 3,060 | 3,080

Dipodomys_ordii	---------- ---------- ---------- ---------- ---------- ---------- ----------	-
Laurasiatheria	---------- ---------- ---------- ---------- ---------- ---------- ----------	-
Eutheria	---------- ---------- ---------- ---------- ---------- ---------- ----------	-
Tarsius_syrichta	---------- ---------- ---------- ---------- ---------- ---------- ----------	-
Muridae	---------- ---------- ---------- ---------- ---------- ---------- ----------	-
Gasterosteus_aculeatus	---------- ---------- ---------- ---------- ---------- ---------- ----------	-
Felis_silvestris	---------- ---------- ---------- ---------- ---------- ---------- ----------	-
Rattus	---------- ---------- ---------- ---------- ---------- ---------- ----------	-
Papio_hamadryas	AGTACTTTGG CGCCATTCTC TGGCCGCGCG GCTCATCTGT TTTTGTCTGG TTTGTGTTGT TACTGTTGTC	393
Pan_troglodytes	---------- ---------- ---------- ---------- ---------- ---------- ----------	-
Ailuropoda_melanoleuca	---------- ---------- ---------- ---------- ---------- ---------- ----------	-
Cavia_porcellus	---------- ---------- ---------- ---------- ---------- ---------- ----------	-
Ictidomys_tridecemlineatus	---------- ---------- ---------- ---------- ---------- ---------- ----------	-
Canis	---------- ---------- ---------- ---------- ---------- ---------- ----------	-
Felis_catus	---------- ---------- ---------- ---------- ---------- ---------- ----------	-
Ochotona_princeps	---------- ---------- ---------- ---------- ---------- ---------- ----------	-
Cercopithecidae	---------- ---------- ---------- ---------- ---------- ---------- ----------	-
Carnivora	---------- ---------- ---------- ---------- ---------- ---------- ----------	-
Mus	---------- ---------- ---------- ---------- ---------- ---------- ----------	-
Mus_musculus	---------- ---------- ---------- ---------- ---------- ---------- ----------	-
Rattus_norvegicus	---------- ---------- ---------- ---------- ---------- ---------- ----------	-
Tursiops_truncatus	---------- ---------- ---------- ---------- ---------- ---------- ----------	-
Procavia_capensis	---------- ---------- ---------- ---------- ---------- ---------- ----------	-
Primates	---------- ---------- ---------- ---------- ---------- ---------- ----------	-
Echinops_telfairi	---------- ---------- ---------- ---------- ---------- ---------- ----------	-
Sorex_araneus	---------- ---------- ---------- ---------- ---------- ---------- ----------	-
Canidae	---------- ---------- ---------- ---------- ---------- ---------- ----------	-
Otolemur_garnettii	---------- ---------- ---------- ---------- ---------- ---------- ----------	-
Gallus	---------- ---------- ---------- ---------- ---------- ---------- ----------	-
Callithrix_jacchus	---------- ---------- ---------- ---------- ---------- ---------- ----------	-
Crocodylus_porosus	---------- ---------- ---------- ---------- ---------- ---------- ----------	-
Erinaceus_europaeus	---------- ---------- ---------- ---------- ---------- ---------- ----------	-
rnd-6_family-7426-LTR-ERV1	---------- ---------- ---------- ---------- ---------- ---------- ----------	-
Esox_lucius	---------- ---------- ---------- ---------- ---------- ---------- ----------	-
Salmo_salar	---------- ---------- ---------- ---------- ---------- ---------- ----------	-
Didelphidae	---------- ---------- ---------- ---------- ---------- ---------- ----------	-
Latimeria_chalumnae	---------- ---------- ---------- ---------- ---------- ---------- ----------	-
Monodelphis_domestica	---------- ---------- ---------- ---------- ---------- ---------- ----------	-
Macropus_eugenii	---------- ---------- ---------- ---------- ---------- ---------- ----------	-
Alligator_mississippiensis	---------- ---------- ---------- ---------- ---------- ---------- ----------	-
Chrysemys_picta_bellii	---------- ---------- ---------- ---------- ---------- ---------- ----------	-
Danio_rerio	---------- ---------- ---------- ---------- ---------- ---------- ----------	-
Taeniopygia	---------- ---------- ---------- ---------- ---------- ---------- ----------	-
Dasypus_novemcinctus	---------- ---------- ---------- ---------- ---------- ---------- ----------	-
LTR_XT	---------- ---------- ---------- ---------- ---------- ---------- ----------	-
Haplorrhini	AAAGCAGAGG AGTGGAGACT TTATGAAAGT AGTTGCCAGG AGTGTGNANN GGGNNNNANN GNANNTNANA	3072
Catarrhini	CGGGAAGAAG AATGGAGACT TT-------- ---------- --------TT CTAACCGAGC CAGGCCAAGA	2566
Anolis_carolinensis	---------- ---------- ---------- ---------- ---------- ---------- ----------	-
Murine_leukemia_virus	ATAGAGGATG AGTATCGGCT ACA------- ---------- --------TG AGACCTCAAC AGAGCCGGAT	2530
Phascolarctos_cinereus	TTGGAGGAGG AATACCGATT ACA------- ---------- --------CG AAA----AGC CGGTCCC--T	2427
Estrildidae	---------- ---------- ---------- ---------- ---------- ---------- ----------	-
Xenopus_Silurana_tropicalis	---------- ---------- ---------- ---------- ---------- ---------- ----------	-
I_XT	TGCTNCAAAC AGTAGTTNAA NAGGANCATT TGANTNTNTT AGAAGAANTN TTANATCTGA TATANAACNN	2558
Takifugu_rubripes	---------- ---------- ---------- ---------- ---------- ---------- ----------	-
Bos_taurus	---------- ---------- ---------- ---------- ---------- ---------- ----------	-
Homo_sapiens	---------- ---------- ---------- ---------- ---------- ---------- ----------	-
Microcebus_murinus	---------- ---------- ---------- ---------- ---------- ---------- ----------	-
Myotis_lucifugus	---------- ---------- ---------- ---------- ---------- ---------- ----------	-
Equus_caballus	---------- ---------- ---------- ---------- ---------- ---------- ----------	-
Tupaia_belangeri	---------- ---------- ---------- ---------- ---------- ---------- ----------	-
Loxodonta_africana	---------- ---------- ---------- ---------- ---------- ---------- ----------	-
Murinae	---------- ---------- ---------- ---------- ---------- ---------- ----------	-
Canis_lupus_familiaris	---------- ---------- ---------- ---------- ---------- ---------- ----------	-
Sus_scrofa	---------- ---------- ---------- ---------- ---------- ---------- ----------	-
Choloepus_hoffmanni	---------- ---------- ---------- ---------- ---------- ---------- ----------	-
Vicugna_pacos	---------- ---------- ---------- ---------- ---------- ---------- ----------	-
Gavialis_gangeticus	---------- ---------- ---------- ---------- ---------- ---------- ----------	-
Mammalia	---------- ---------- ---------- ---------- ---------- ---------- ----------	-
Passeroidea	---------- ---------- ---------- ---------- ---------- ---------- ----------	-
Takifugu	---------- ---------- ---------- ---------- ---------- ---------- ----------	-
Pteropus_vampyrus	---------- ---------- ---------- ---------- ---------- ---------- ----------	-
Ornithorhynchus	---------- ---------- ---------- ---------- ---------- ---------- ----------	-
Oryctolagus_cuniculus	---------- ---------- ---------- ---------- ---------- ---------- ----------	-
Gallus_gallus	---------- ---------- ---------- ---------- ---------- ---------- ----------	-
Hominidae	---------- ---------- ---------- ---------- ---------- ---------- ----------	-
Metatheria	---------- ---------- ---------- ---------- ---------- ---------- ----------	-
Rodentia	---------- ---------- ---------- ---------- ---------- ---------- ----------	-
Monodelphis	---------- ---------- ---------- ---------- ---------- ---------- ----------	-
Consensus	---------- ---------- ---------- ---------- ---------- ---------- ----------	

Conservation

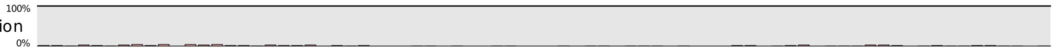

3,100 3,120 3,140

Dipodomys_ordii	----------	----------	----------	----------	----------	----------	----------	-
Laurasiatheria	----------	----------	----------	----------	----------	----------	----------	-
Eutheria	----------	----------	----------	----------	----------	----------	----------	-
Tarsius_syrichta	----------	----------	----------	----------	----------	----------	----------	-
Muridae	----------	----------	----------	----------	----------	----------	----------	-
Gasterosteus_aculeatus	----------	----------	----------	----------	----------	----------	----------	-
Felis_silvestris	----------	----------	----------	----------	----------	----------	----------	-
Rattus	----------	----------	----------	----------	----------	----------	----------	-
Papio_hamadryas	TTATTTATAT	GTGTGTATGA	GCCTAAGGAC	GGGACAATGG	GACAGACGCT	AACAACTCCT	CTATCTTTGA	463
Pan_troglodytes	----------	----------	----------	----------	----------	----------	----------	-
Ailuropoda_melanoleuca	----------	----------	----------	----------	----------	----------	----------	-
Cavia_porcellus	----------	----------	----------	----------	----------	----------	----------	-
Ictidomys_tridecemlineatus	----------	----------	----------	----------	----------	----------	----------	-
Canis	----------	----------	----------	----------	----------	----------	----------	-
Felis_catus	----------	----------	----------	----------	----------	----------	----------	-
Ochotona_princeps	----------	----------	----------	----------	----------	----------	----------	-
Cercopithecidae	----------	----------	----------	----------	----------	----------	----------	-
Carnivora	----------	----------	----------	----------	----------	----------	----------	-
Mus	----------	----------	----------	----------	----------	----------	----------	-
Mus_musculus	----------	----------	----------	----------	----------	----------	----------	-
Rattus_norvegicus	----------	----------	----------	----------	----------	----------	----------	-
Tursiops_truncatus	----------	----------	----------	----------	----------	----------	----------	-
Procavia_capensis	----------	----------	----------	----------	----------	----------	----------	-
Primates	----------	----------	----------	----------	----------	----------	----------	-
Echinops_telfairi	----------	----------	----------	----------	----------	----------	----------	-
Sorex_araneus	----------	----------	----------	----------	----------	----------	----------	-
Canidae	----------	----------	----------	----------	----------	----------	----------	-
Otolemur_garnettii	----------	----------	----------	----------	----------	----------	----------	-
Gallus	----------	----------	----------	----------	----------	----------	----------	-
Callithrix_jacchus	----------	----------	----------	----------	----------	----------	----------	-
Crocodylus_porosus	----------	----------	----------	----------	----------	----------	----------	-
Erinaceus_europaeus	----------	----------	----------	----------	----------	----------	----------	-
rnd-6_family-7426-LTR-ERV1	----------	----------	----------	----------	----------	----------	----------	-
Esox_lucius	----------	----------	----------	----------	----------	----------	----------	-
Salmo_salar	----------	----------	----------	----------	----------	----------	----------	-
Didelphidae	----------	----------	----------	----------	----------	----------	----------	-
Latimeria_chalumnae	----------	----------	----------	----------	----------	----------	----------	-
Monodelphis_domestica	----------	----------	----------	----------	----------	----------	----------	-
Macropus_eugenii	----------	----------	----------	----------	----------	----------	----------	-
Alligator_mississippiensis	----------	----------	----------	----------	----------	----------	----------	-
Chrysemys_picta_bellii	----------	----------	----------	----------	----------	----------	----------	-
Danio_rerio	----------	----------	----------	----------	----------	----------	----------	-
Taeniopygia	----------	----------	----------	----------	----------	----------	----------	-
Dasypus_novemcinctus	----------	----------	----------	----------	----------	----------	----------	-
LTR_XT	----------	----------	----------	----------	----------	----------	----------	-
Haplorrhini	GAGNANAANN	GTNNANANAN	TTANTCNTTA	AGTTNCNNGN	AGNNNGNNNG	NANNANANNC	CCCCAGGGCT	3142
Catarrhini	GATAAAACCA	GCTC---TAG	CTAAGCGATG	----GCCCCG	AGTATGGGCA	GAGGATAATC	CTCCGGGACT	2629
Anolis_carolinensis	----------	----------	----------	----------	----------	----------	----------	-
Murine_leukemia_virus	GTTTCTCTAG	GGTC--CACC	TGGCTTTCTG	ATTTTCCCCA	GGCCTGGGCG	GAAACCGGGG	GCATGGGACT	2598
Phascolarctos_cinereus	CCTTCTATCG	ACCC--GTCA	TGGCTCCAAC	TCTTTCCCAT	GGTTTGGGCC	GAGAAGGCAG	GTATGGGACT	2495
Estrildidae	----------	----------	----------	----------	----------	----------	----------	-
Xenopus_Silurana_tropicalis	----------	----------	----------	----------	----------	----------	----------	-
I_XT	AGGCTTTAAA	TANNCNANAA	ATGTNNNNTN	ANTTGAANNN	GNTGTCNAAT	TTCTTGNGGT	CAACATAGGN	2628
Takifugu_rubripes	----------	----------	----------	----------	----------	----------	----------	-
Bos_taurus	----------	----------	----------	----------	----------	----------	----------	-
Homo_sapiens	----------	----------	----------	----------	----------	----------	----------	-
Microcebus_murinus	----------	----------	----------	----------	----------	----------	----------	-
Myotis_lucifugus	----------	----------	----------	----------	----------	----------	----------	-
Equus_caballus	----------	----------	----------	----------	----------	----------	----------	-
Tupaia_belangeri	----------	----------	----------	----------	----------	----------	----------	-
Loxodonta_africana	----------	----------	----------	----------	----------	----------	----------	-
Murinae	----------	----------	----------	----------	----------	----------	----------	-
Canis_lupus_familiaris	----------	----------	----------	----------	----------	----------	----------	-
Sus_scrofa	----------	----------	----------	----------	----------	----------	----------	-
Choloepus_hoffmanni	----------	----------	----------	----------	----------	----------	----------	-
Vicugna_pacos	----------	----------	----------	----------	----------	----------	----------	-
Gavialis_gangeticus	----------	----------	----------	----------	----------	----------	----------	-
Mammalia	----------	----------	----------	----------	----------	----------	----------	-
Passeroidea	----------	----------	----------	----------	----------	----------	----------	-
Takifugu	----------	----------	----------	----------	----------	----------	----------	-
Pteropus_vampyrus	----------	----------	----------	----------	----------	----------	----------	-
Ornithorhynchus	----------	----------	----------	----------	----------	----------	----------	-
Oryctolagus_cuniculus	----------	----------	----------	----------	----------	----------	----------	-
Gallus_gallus	----------	----------	----------	----------	----------	----------	----------	-
Hominidae	----------	----------	----------	----------	----------	----------	----------	-
Metatheria	----------	----------	----------	----------	----------	----------	----------	-
Rodentia	----------	----------	----------	----------	----------	----------	----------	-
Monodelphis	----------	----------	----------	----------	----------	----------	----------	-
Consensus	----------	----------	----------	----------	----------	----------	----------	

Conservation

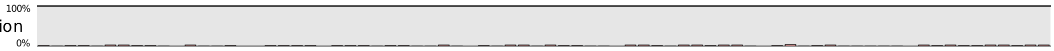

3,160 3,180 3,200 3,220

Dipodomys_ordii	----------	----------	----------	----------	----------	----------	----------	-
Laurasiatheria	----------	----------	----------	----------	----------	----------	----------	-
Eutheria	----------	----------	----------	----------	----------	----------	----------	-
Tarsius_syrichta	----------	----------	----------	----------	----------	----------	----------	-
Muridae	----------	----------	----------	----------	----------	----------	----------	-
Gasterosteus_aculeatus	----------	----------	----------	----------	----------	----------	----------	-
Felis_silvestris	----------	----------	----------	----------	----------	----------	----------	-
Rattus	----------	----------	----------	----------	----------	----------	----------	-
Papio_hamadryas	CTTTGACGCA	CTTTTCAGAC	GTCCGGGCCA	GAGCCCACAA	TCTTTCCGTA	GGAGTCCGAA	AAGGACGATG	533
Pan_troglodytes	----------	----------	----------	----------	----------	----------	----------	-
Ailuropoda_melanoleuca	----------	----------	----------	----------	----------	----------	----------	-
Cavia_porcellus	----------	----------	----------	----------	----------	----------	----------	-
Ictidomys_tridecemlineatus	----------	----------	----------	----------	----------	----------	----------	-
Canis	----------	----------	----------	----------	----------	----------	----------	-
Felis_catus	----------	----------	----------	----------	----------	----------	----------	-
Ochotona_princeps	----------	----------	----------	----------	----------	----------	----------	-
Cercopithecidae	----------	----------	----------	----------	----------	----------	----------	-
Carnivora	----------	----------	----------	----------	----------	----------	----------	-
Mus	----------	----------	----------	----------	----------	----------	----------	-
Mus_musculus	----------	----------	----------	----------	----------	----------	----------	-
Rattus_norvegicus	----------	----------	----------	----------	----------	----------	----------	-
Tursiops_truncatus	----------	----------	----------	----------	----------	----------	----------	-
Procavia_capensis	----------	----------	----------	----------	----------	----------	----------	-
Primates	----------	----------	----------	----------	----------	----------	----------	-
Echinops_telfairi	----------	----------	----------	----------	----------	----------	----------	-
Sorex_araneus	----------	----------	----------	----------	----------	----------	----------	-
Canidae	----------	----------	----------	----------	----------	----------	----------	-
Otolemur_garnettii	----------	----------	----------	----------	----------	----------	----------	-
Gallus	----------	----------	----------	----------	----------	----------	----------	-
Callithrix_jacchus	----------	----------	----------	----------	----------	----------	----------	-
Crocodylus_porosus	----------	----------	----------	----------	----------	----------	----------	-
Erinaceus_europaeus	----------	----------	----------	----------	----------	----------	----------	-
rnd-6_family-7426-LTR-ERV1	----------	----------	----------	----------	----------	----------	----------	-
Esox_lucius	----------	----------	----------	----------	----------	----------	----------	-
Salmo_salar	----------	----------	----------	----------	----------	----------	----------	-
Didelphidae	----------	----------	----------	----------	----------	----------	----------	-
Latimeria_chalumnae	----------	----------	----------	----------	----------	----------	----------	-
Monodelphis_domestica	----------	----------	----------	----------	----------	----------	----------	-
Macropus_eugenii	----------	----------	----------	----------	----------	----------	----------	-
Alligator_mississippiensis	----------	----------	----------	----------	----------	----------	----------	-
Chrysemys_picta_bellii	----------	----------	----------	----------	----------	----------	----------	-
Danio_rerio	----------	----------	----------	----------	----------	----------	----------	-
Taeniopygia	----------	----------	----------	----------	----------	----------	----------	-
Dasypus_novemcinctus	----------	----------	----------	----------	----------	----------	----------	-
LTR_XT	----------	----------	----------	----------	----------	----------	----------	-
Haplorrhini	AGCAGTGANN	CNNNNNCCTG	NNNTANNAGA	GCTACTACGA	GGAACCTANN	NNNTGCNNAT	NNNTCAGTAN	3212
Catarrhini	GGCGGTCAAC	CAAGCCCCCG	TACTCATAGA	AGTTAAGCCT	GGGGCCCAAC	CAATTAGACA	AAAGCAGTAT	2699
Anolis_carolinensis	----------	----------	----------	----------	----------	----------	----------	-
Murine_leukemia_virus	GGCAGTTCGC	CAAGCGCCTC	TGATTATACC	TCTGAAGGCA	ACCTCCACCC	CCGTGTCCAT	AAAACAATAC	2668
Phascolarctos_cinereus	GGCCAATCAA	GTCCCACCAG	TGGTAGTGGA	ACTGAAGTCA	GATGCCTCAC	CAGTGGCTGT	TCGACAGTAC	2565
Estrildidae	----------	----------	----------	----------	----------	----------	----------	-
Xenopus_Silurana_tropicalis	----------	----------	----------	----------	----------	----------	----------	-
I_XT	CCAGGTTNTA	GGATTCCNNT	ANANGACAGA	ANNTANNNTA	TNTCTAAANT	CNNAGTNCNA	ACNTCNCAAA	2698
Takifugu_rubripes	----------	----------	----------	----------	----------	----------	----------	-
Bos_taurus	----------	----------	----------	----------	----------	----------	----------	-
Homo_sapiens	----------	----------	----------	----------	----------	----------	----------	-
Microcebus_murinus	----------	----------	----------	----------	----------	----------	----------	-
Myotis_lucifugus	----------	----------	----------	----------	----------	----------	----------	-
Equus_caballus	----------	----------	----------	----------	----------	----------	----------	-
Tupaia_belangeri	----------	----------	----------	----------	----------	----------	----------	-
Loxodonta_africana	----------	----------	----------	----------	----------	----------	----------	-
Murinae	----------	----------	----------	----------	----------	----------	----------	-
Canis_lupus_familiaris	----------	----------	----------	----------	----------	----------	----------	-
Sus_scrofa	----------	----------	----------	----------	----------	----------	----------	-
Choloepus_hoffmanni	----------	----------	----------	----------	----------	----------	----------	-
Vicugna_pacos	----------	----------	----------	----------	----------	----------	----------	-
Gavialis_gangeticus	----------	----------	----------	----------	----------	----------	----------	-
Mammalia	----------	----------	----------	----------	----------	----------	----------	-
Passeroidea	----------	----------	----------	----------	----------	----------	----------	-
Takifugu	----------	----------	----------	----------	----------	----------	----------	-
Pteropus_vampyrus	----------	----------	----------	----------	----------	----------	----------	-
Ornithorhynchus	----------	----------	----------	----------	----------	----------	----------	-
Oryctolagus_cuniculus	----------	----------	----------	----------	----------	----------	----------	-
Gallus_gallus	----------	----------	----------	----------	----------	----------	----------	-
Hominidae	----------	----------	----------	----------	----------	----------	----------	-
Metatheria	----------	----------	----------	----------	----------	----------	----------	-
Rodentia	----------	----------	----------	----------	----------	----------	----------	-
Monodelphis	----------	----------	----------	----------	----------	----------	----------	-
Consensus	----------	----------	----------	----------	----------	----------	----------	

Conservation 100% 0%

		3,240		3,260		3,280		
Dipodomys_ordii	----------	----------	----------	----------	----------	----------	----------	-
Laurasiatheria	----------	----------	----------	----------	----------	----------	----------	-
Eutheria	----------	----------	----------	----------	----------	----------	----------	-
Tarsius_syrichta	----------	----------	----------	----------	----------	----------	----------	-
Muridae	----------	----------	----------	----------	----------	----------	----------	-
Gasterosteus_aculeatus	----------	----------	----------	----------	----------	----------	----------	-
Felis_silvestris	----------	----------	----------	----------	----------	----------	----------	-
Rattus	----------	----------	----------	----------	----------	----------	----------	-
Papio_hamadryas	GCAAACTTTC	TGCTCGTCCG	AGTGGCCCAC	CCTTCATGTC	GGGTGGCCCC	GGGACGGAAC	TTTTGACCTC	603
Pan_troglodytes	----------	----------	----------	----------	----------	----------	----------	-
Ailuropoda_melanoleuca	----------	----------	----------	----------	----------	----------	----------	-
Cavia_porcellus	----------	----------	----------	----------	----------	----------	----------	-
Ictidomys_tridecemlineatus	----------	----------	----------	----------	----------	----------	----------	-
Canis	----------	----------	----------	----------	----------	----------	----------	-
Felis_catus	----------	----------	----------	----------	----------	----------	----------	-
Ochotona_princeps	----------	----------	----------	----------	----------	----------	----------	-
Cercopithecidae	----------	----------	----------	----------	----------	----------	----------	-
Carnivora	----------	----------	----------	----------	----------	----------	----------	-
Mus	----------	----------	----------	----------	----------	----------	----------	-
Mus_musculus	----------	----------	----------	----------	----------	----------	----------	-
Rattus_norvegicus	----------	----------	----------	----------	----------	----------	----------	-
Tursiops_truncatus	----------	----------	----------	----------	----------	----------	----------	-
Procavia_capensis	----------	----------	----------	----------	----------	----------	----------	-
Primates	----------	----------	----------	----------	----------	----------	----------	-
Echinops_telfairi	----------	----------	----------	----------	----------	----------	----------	-
Sorex_araneus	----------	----------	----------	----------	----------	----------	----------	-
Canidae	----------	----------	----------	----------	----------	----------	----------	-
Otolemur_garnettii	----------	----------	----------	----------	----------	----------	----------	-
Gallus	----------	----------	----------	----------	----------	----------	----------	-
Callithrix_jacchus	----------	----------	----------	----------	----------	----------	----------	-
Crocodylus_porosus	----------	----------	----------	----------	----------	----------	----------	-
Erinaceus_europaeus	----------	----------	----------	----------	----------	----------	----------	-
rnd-6_family-7426-LTR-ERV1	----------	----------	----------	----------	----------	----------	----------	-
Esox_lucius	----------	----------	----------	----------	----------	----------	----------	-
Salmo_salar	----------	----------	----------	----------	----------	----------	----------	-
Didelphidae	----------	----------	----------	----------	----------	----------	----------	-
Latimeria_chalumnae	----------	----------	----------	----------	----------	----------	----------	-
Monodelphis_domestica	----------	----------	----------	----------	----------	----------	----------	-
Macropus_eugenii	----------	----------	----------	----------	----------	----------	----------	-
Alligator_mississippiensis	----------	----------	----------	----------	----------	----------	----------	-
Chrysemys_picta_bellii	----------	----------	----------	----------	----------	----------	----------	-
Danio_rerio	----------	----------	----------	----------	----------	----------	----------	-
Taeniopygia	----------	----------	----------	----------	----------	----------	----------	-
Dasypus_novemcinctus	----------	----------	----------	----------	----------	----------	----------	-
LTR_XT	----------	----------	----------	----------	----------	----------	----------	-
Haplorrhini	NCNATNCCCA	NAGAGGCTTA	NCNNNGGATT	NCAAANCNNT	TNANATGGCT	CCTTGANNNT	GGAATAANAG	3282
Catarrhini	CCGGTTCCCA	GAGAAGCTCT	CGAAGGAATC	CAGGTTCATC	TCAGGCGCTT	GAAAGCCTAT	GGAATTATAG	2769
Anolis_carolinensis	----------	----------	----------	----------	----------	----------	----------	-
Murine_leukemia_virus	CCCATGTCGC	ANGAAGCCAG	ACTGGGGATC	AAGCCCCACA	TACAGAGGCT	GTTGGACCAG	GGAATATTGG	2738
Phascolarctos_cinereus	CCAATGAGCA	AGGAAGCCCG	GGAGGGTATC	AGGCCCCACA	TCCAAAGATT	CTTGGATTTG	GGGATTCTGG	2635
Estrildidae	----------	----------	----------	----------	----------	----------	----------	-
Xenopus_Silurana_tropicalis	----------	----------	----------	----------	----------	----------	----------	-
I_XT	NGGCCCTAAG	ATCATTNNNG	GGANTAGNAA	ATTAATCTAG	NGAGTTNATA	AANTNCTTTG	NNGANAAAGC	2768
Takifugu_rubripes	----------	----------	----------	----------	----------	----------	----------	-
Bos_taurus	----------	----------	----------	----------	----------	----------	----------	-
Homo_sapiens	----------	----------	----------	----------	----------	----------	----------	-
Microcebus_murinus	----------	----------	----------	----------	----------	----------	----------	-
Myotis_lucifugus	----------	----------	----------	----------	----------	----------	----------	-
Equus_caballus	----------	----------	----------	----------	----------	----------	----------	-
Tupaia_belangeri	----------	----------	----------	----------	----------	----------	----------	-
Loxodonta_africana	----------	----------	----------	----------	----------	----------	----------	-
Murinae	----------	----------	----------	----------	----------	----------	----------	-
Canis_lupus_familiaris	----------	----------	----------	----------	----------	----------	----------	-
Sus_scrofa	----------	----------	----------	----------	----------	----------	----------	-
Choloepus_hoffmanni	----------	----------	----------	----------	----------	----------	----------	-
Vicugna_pacos	----------	----------	----------	----------	----------	----------	----------	-
Gavialis_gangeticus	----------	----------	----------	----------	----------	----------	----------	-
Mammalia	----------	----------	----------	----------	----------	----------	----------	-
Passeroidea	----------	----------	----------	----------	----------	----------	----------	-
Takifugu	----------	----------	----------	----------	----------	----------	----------	-
Pteropus_vampyrus	----------	----------	----------	----------	----------	----------	----------	-
Ornithorhynchus	----------	----------	----------	----------	----------	----------	----------	-
Oryctolagus_cuniculus	----------	----------	----------	----------	----------	----------	----------	-
Gallus_gallus	----------	----------	----------	----------	----------	----------	----------	-
Hominidae	----------	----------	----------	----------	----------	----------	----------	-
Metatheria	----------	----------	----------	----------	----------	----------	----------	-
Rodentia	----------	----------	----------	----------	----------	----------	----------	-
Monodelphis	----------	----------	----------	----------	----------	----------	----------	-
Consensus	----------	----------	----------	----------	----------	----------	----------	
Conservation (100% / 0%)								

3,300 3,320 3,340 3,360

Dipodomys_ordii	----------	----------	----------	----------	----------	----------	----------	-
Laurasiatheria	----------	----------	----------	----------	----------	----------	----------	-
Eutheria	----------	----------	----------	----------	----------	----------	----------	-
Tarsius_syrichta	----------	----------	----------	----------	----------	----------	----------	-
Muridae	----------	----------	----------	----------	----------	----------	----------	-
Gasterosteus_aculeatus	----------	----------	----------	----------	----------	----------	----------	-
Felis_silvestris	----------	----------	----------	----------	----------	----------	----------	-
Rattus	----------	----------	----------	----------	----------	----------	----------	-
Papio_hamadryas	TCCGTTATTT	TGCAGGTTAA	GACAAAGGTA	ATGGATCCTG	GGCCGCATGG	TCACCCGGAC	CAAGTGGCCT	673
Pan_troglodytes	----------	----------	----------	----------	----------	----------	----------	-
Ailuropoda_melanoleuca	----------	----------	----------	----------	----------	----------	----------	-
Cavia_porcellus	----------	----------	----------	----------	----------	----------	----------	-
Ictidomys_tridecemlineatus	----------	----------	----------	----------	----------	----------	----------	-
Canis	----------	----------	----------	----------	----------	----------	----------	-
Felis_catus	----------	----------	----------	----------	----------	----------	----------	-
Ochotona_princeps	----------	----------	----------	----------	----------	----------	----------	-
Cercopithecidae	----------	----------	----------	----------	----------	----------	----------	-
Carnivora	----------	----------	----------	----------	----------	----------	----------	-
Mus	----------	----------	----------	----------	----------	----------	----------	-
Mus_musculus	----------	----------	----------	----------	----------	----------	----------	-
Rattus_norvegicus	----------	----------	----------	----------	----------	----------	----------	-
Tursiops_truncatus	----------	----------	----------	----------	----------	----------	----------	-
Procavia_capensis	----------	----------	----------	----------	----------	----------	----------	-
Primates	----------	----------	----------	----------	----------	----------	----------	-
Echinops_telfairi	----------	----------	----------	----------	----------	----------	----------	-
Sorex_araneus	----------	----------	----------	----------	----------	----------	----------	-
Canidae	----------	----------	----------	----------	----------	----------	----------	-
Otolemur_garnettii	----------	----------	----------	----------	----------	----------	----------	-
Gallus	----------	----------	----------	----------	----------	----------	----------	-
Callithrix_jacchus	----------	----------	----------	----------	----------	----------	----------	-
Crocodylus_porosus	----------	----------	----------	----------	----------	----------	----------	-
Erinaceus_europaeus	----------	----------	----------	----------	----------	----------	----------	-
rnd-6_family-7426-LTR-ERV1	----------	----------	----------	----------	----------	----------	----------	-
Esox_lucius	----------	----------	----------	----------	----------	----------	----------	-
Salmo_salar	----------	----------	----------	----------	----------	----------	----------	-
Didelphidae	----------	----------	----------	----------	----------	----------	----------	-
Latimeria_chalumnae	----------	----------	----------	----------	----------	----------	----------	-
Monodelphis_domestica	----------	----------	----------	----------	----------	----------	----------	-
Macropus_eugenii	----------	----------	----------	----------	----------	----------	----------	-
Alligator_mississippiensis	----------	----------	----------	----------	----------	----------	----------	-
Chrysemys_picta_bellii	----------	----------	----------	----------	----------	----------	----------	-
Danio_rerio	----------	----------	----------	----------	----------	----------	----------	-
Taeniopygia	----------	----------	----------	----------	----------	----------	----------	-
Dasypus_novemcinctus	----------	----------	----------	----------	----------	----------	----------	-
LTR_XT	----------	----------	----------	----------	----------	----------	----------	-
Haplorrhini	NGAGNTNTNN	CTNCNCATGG	AATACTNCCC	TANNGCNGNT	GTNAANNCNN	NNNGNT---G	ANNANCANNN	3349
Catarrhini	TTCCTTGCCA	GTCTCCGCGG	AACACCCCCC	TCCTGCCTGT	CCCTAAGCCA	GGGACCAAGG	ACTACCGGCC	2839
Anolis_carolinensis	----------	----------	----------	----------	----------	----------	----------	-
Murine_leukemia_virus	TACCCTGCCA	GTCCCCCTGG	AACACGCCCC	TGCTGCCCGT	TAAGAAACCA	GGGACTAATG	ATTACAGGCC	2808
Phascolarctos_cinereus	TACCTTGCCA	GTCGCCCTGG	AACACCCCTT	TGTTACCTGT	AAAAAAACCT	GGAACCAATG	ACTATCGGCC	2705
Estrildidae	----------	----------	----------	----------	----------	----------	----------	-
Xenopus_Silurana_tropicalis	----------	----------	----------	----------	----------	----------	----------	-
I_XT	TAGACCTTTT	TATGNCCTNC	NANACNGNAG	TNAATACACC	TTTGGTCCCT	GNNGTCAAGN	ACACTANNNA	2838
Takifugu_rubripes	----------	----------	----------	----------	----------	----------	----------	-
Bos_taurus	----------	----------	----------	----------	----------	----------	----------	-
Homo_sapiens	----------	----------	----------	----------	----------	----------	----------	-
Microcebus_murinus	----------	----------	----------	----------	----------	----------	----------	-
Myotis_lucifugus	----------	----------	----------	----------	----------	----------	----------	-
Equus_caballus	----------	----------	----------	----------	----------	----------	----------	-
Tupaia_belangeri	----------	----------	----------	----------	----------	----------	----------	-
Loxodonta_africana	----------	----------	----------	----------	----------	----------	----------	-
Murinae	----------	----------	----------	----------	----------	----------	----------	-
Canis_lupus_familiaris	----------	----------	----------	----------	----------	----------	----------	-
Sus_scrofa	----------	----------	----------	----------	----------	----------	----------	-
Choloepus_hoffmanni	----------	----------	----------	----------	----------	----------	----------	-
Vicugna_pacos	----------	----------	----------	----------	----------	----------	----------	-
Gavialis_gangeticus	----------	----------	----------	----------	----------	----------	----------	-
Mammalia	----------	----------	----------	----------	----------	----------	----------	-
Passeroidea	----------	----------	----------	----------	----------	----------	----------	-
Takifugu	----------	----------	----------	----------	----------	----------	----------	-
Pteropus_vampyrus	----------	----------	----------	----------	----------	----------	----------	-
Ornithorhynchus	----------	----------	----------	----------	----------	----------	----------	-
Oryctolagus_cuniculus	----------	----------	----------	----------	----------	----------	----------	-
Gallus_gallus	----------	----------	----------	----------	----------	----------	----------	-
Hominidae	----------	----------	----------	----------	----------	----------	----------	-
Metatheria	----------	----------	----------	----------	----------	----------	----------	-
Rodentia	----------	----------	----------	----------	----------	----------	----------	-
Monodelphis	----------	----------	----------	----------	----------	----------	----------	-
Consensus	----------	----------	----------	----------	----------	----------	----------	

Conservation 100% 0%

3,380 3,400 3,420

Dipodomys_ordii	----------	----------	----------	----------	----------	----------	----------	-
Laurasiatheria	----------	----------	----------	----------	----------	----------	----------	-
Eutheria	----------	----------	----------	----------	----------	----------	----------	-
Tarsius_syrichta	----------	----------	----------	----------	----------	----------	----------	-
Muridae	----------	----------	----------	----------	----------	----------	----------	-
Gasterosteus_aculeatus	----------	----------	----------	----------	----------	----------	----------	-
Felis_silvestris	----------	----------	----------	----------	----------	----------	----------	-
Rattus	----------	----------	----------	----------	----------	----------	----------	-
Papio_hamadryas	ACATCATCAC	CTGGGAGGAT	CTCGTCCGAA	ATCCTCCGCC	TTGGGTGAAA	CCCTTTCTCC	ATACCCCTTC	743
Pan_troglodytes	----------	----------	----------	----------	----------	----------	----------	-
Ailuropoda_melanoleuca	----------	----------	----------	----------	----------	----------	----------	-
Cavia_porcellus	----------	----------	----------	----------	----------	----------	----------	-
Ictidomys_tridecemlineatus	----------	----------	----------	----------	----------	----------	----------	-
Canis	----------	----------	----------	----------	----------	----------	----------	-
Felis_catus	----------	----------	----------	----------	----------	----------	----------	-
Ochotona_princeps	----------	----------	----------	----------	----------	----------	----------	-
Cercopithecidae	----------	----------	----------	----------	----------	----------	----------	-
Carnivora	----------	----------	----------	----------	----------	----------	----------	-
Mus	----------	----------	----------	----------	----------	----------	----------	-
Mus_musculus	----------	----------	----------	----------	----------	----------	----------	-
Rattus_norvegicus	----------	----------	----------	----------	----------	----------	----------	-
Tursiops_truncatus	----------	----------	----------	----------	----------	----------	----------	-
Procavia_capensis	----------	----------	----------	----------	----------	----------	----------	-
Primates	----------	----------	----------	----------	----------	----------	----------	-
Echinops_telfairi	----------	----------	----------	----------	----------	----------	----------	-
Sorex_araneus	----------	----------	----------	----------	----------	----------	----------	-
Canidae	----------	----------	----------	----------	----------	----------	----------	-
Otolemur_garnettii	----------	----------	----------	----------	----------	----------	----------	-
Gallus	----------	----------	----------	----------	----------	----------	----------	-
Callithrix_jacchus	----------	----------	----------	----------	----------	----------	----------	-
Crocodylus_porosus	----------	----------	----------	----------	----------	----------	----------	-
Erinaceus_europaeus	----------	----------	----------	----------	----------	----------	----------	-
rnd-6_family-7426-LTR-ERV1	----------	----------	----------	----------	----------	----------	----------	-
Esox_lucius	----------	----------	----------	----------	----------	----------	----------	-
Salmo_salar	----------	----------	----------	----------	----------	----------	----------	-
Didelphidae	----------	----------	----------	----------	----------	----------	----------	-
Latimeria_chalumnae	----------	----------	----------	----------	----------	----------	----------	-
Monodelphis_domestica	----------	----------	----------	----------	----------	----------	----------	-
Macropus_eugenii	----------	----------	----------	----------	----------	----------	----------	-
Alligator_mississippiensis	----------	----------	----------	----------	----------	----------	----------	-
Chrysemys_picta_bellii	----------	----------	----------	----------	----------	----------	----------	-
Danio_rerio	----------	----------	----------	----------	----------	----------	----------	-
Taeniopygia	----------	----------	----------	----------	----------	----------	----------	-
Dasypus_novemcinctus	----------	----------	----------	----------	----------	----------	----------	-
LTR_XT	----------	----------	----------	----------	----------	----------	----------	-
Haplorrhini	NNTACAANAN	NTANGNNNNN	NCAANGANGT	NNNNNNTNNN	NTGNATNCNA	NTGTNNNNNA	NNNNTNNACT	3419
Catarrhini	AGTACAGGAC	TTGCGCTTGG	TCAACCAAGC	TACAGTGACT	CTGCACCCAA	CAGTTCCTAA	CCCTTACACA	2909
Anolis_carolinensis	----------	----------	----------	----------	----------	----------	----------	-
Murine_leukemia_virus	TGTCCAGGAT	CTGAGAGAAG	TCAACAAGCG	GGTGGAAGAC	ATCCACCCCA	CCGTGCCCAA	CCCTTACAAC	2878
Phascolarctos_cinereus	AGTCCAAGAT	TTGAGGGAGG	TCAACAAAAG	AGTACAGGAC	ATTCATCCCA	CAGTCCCGAA	CCCCTATAAC	2775
Estrildidae	----------	----------	----------	----------	----------	----------	----------	-
Xenopus_Silurana_tropicalis	----------	----------	----------	----------	----------	----------	----------	-
I_XT	GCNTTNGNAN	CNCTTAAAAN	ANGACTTNCN	NGCNGNANNN	GCNACAATNG	ACCCAANNGC	ACCNTTTGTN	2908
Takifugu_rubripes	----------	----------	----------	----------	----------	----------	----------	-
Bos_taurus	----------	----------	----------	----------	----------	----------	----------	-
Homo_sapiens	----------	----------	----------	----------	----------	----------	----------	-
Microcebus_murinus	----------	----------	----------	----------	----------	----------	----------	-
Myotis_lucifugus	----------	----------	----------	----------	----------	----------	----------	-
Equus_caballus	----------	----------	----------	----------	----------	----------	----------	-
Tupaia_belangeri	----------	----------	----------	----------	----------	----------	----------	-
Loxodonta_africana	----------	----------	----------	----------	----------	----------	----------	-
Murinae	----------	----------	----------	----------	----------	----------	----------	-
Canis_lupus_familiaris	----------	----------	----------	----------	----------	----------	----------	-
Sus_scrofa	----------	----------	----------	----------	----------	----------	----------	-
Choloepus_hoffmanni	----------	----------	----------	----------	----------	----------	----------	-
Vicugna_pacos	----------	----------	----------	----------	----------	----------	----------	-
Gavialis_gangeticus	----------	----------	----------	----------	----------	----------	----------	-
Mammalia	----------	----------	----------	----------	----------	----------	----------	-
Passeroidea	----------	----------	----------	----------	----------	----------	----------	-
Takifugu	----------	----------	----------	----------	----------	----------	----------	-
Pteropus_vampyrus	----------	----------	----------	----------	----------	----------	----------	-
Ornithorhynchus	----------	----------	----------	----------	----------	----------	----------	-
Oryctolagus_cuniculus	----------	----------	----------	----------	----------	----------	----------	-
Gallus_gallus	----------	----------	----------	----------	----------	----------	----------	-
Hominidae	----------	----------	----------	----------	----------	----------	----------	-
Metatheria	----------	----------	----------	----------	----------	----------	----------	-
Rodentia	----------	----------	----------	----------	----------	----------	----------	-
Monodelphis	----------	----------	----------	----------	----------	----------	----------	-
Consensus	----------	----------	----------	----------	----------	----------	----------	
Conservation (100% / 0%)								

3,440 3,460 3,480 3,500

Dipodomys_ordii	----------	----------	----------	----------	----------	----------	----------	-
Laurasiatheria	----------	----------	----------	----------	----------	----------	----------	-
Eutheria	----------	----------	----------	----------	----------	----------	----------	-
Tarsius_syrichta	----------	----------	----------	----------	----------	----------	----------	-
Muridae	----------	----------	----------	----------	----------	----------	----------	-
Gasterosteus_aculeatus	----------	----------	----------	----------	----------	----------	----------	-
Felis_silvestris	----------	----------	----------	----------	----------	----------	----------	-
Rattus	----------	----------	----------	----------	----------	----------	----------	-
Papio_hamadryas	TACATCCAAG	TCCACCCTCC	TTGCCCTAGA	AGTCCCAAAG	AACCGGACCC	TGGATCCGCC	TAAACCCGTA	813
Pan_troglodytes	----------	----------	----------	----------	----------	----------	----------	-
Ailuropoda_melanoleuca	----------	----------	----------	----------	----------	----------	----------	-
Cavia_porcellus	----------	----------	----------	----------	----------	----------	----------	-
Ictidomys_tridecemlineatus	----------	----------	----------	----------	----------	----------	----------	-
Canis	----------	----------	----------	----------	----------	----------	----------	-
Felis_catus	----------	----------	----------	----------	----------	----------	----------	-
Ochotona_princeps	----------	----------	----------	----------	----------	----------	----------	-
Cercopithecidae	----------	----------	----------	----------	----------	----------	----------	-
Carnivora	----------	----------	----------	----------	----------	----------	----------	-
Mus	----------	----------	----------	----------	----------	----------	----------	-
Mus_musculus	----------	----------	----------	----------	----------	----------	----------	-
Rattus_norvegicus	----------	----------	----------	----------	----------	----------	----------	-
Tursiops_truncatus	----------	----------	----------	----------	----------	----------	----------	-
Procavia_capensis	----------	----------	----------	----------	----------	----------	----------	-
Primates	----------	----------	----------	----------	----------	----------	----------	-
Echinops_telfairi	----------	----------	----------	----------	----------	----------	----------	-
Sorex_araneus	----------	----------	----------	----------	----------	----------	----------	-
Canidae	----------	----------	----------	----------	----------	----------	----------	-
Otolemur_garnettii	----------	----------	----------	----------	----------	----------	----------	-
Gallus	----------	----------	----------	----------	----------	----------	----------	-
Callithrix_jacchus	----------	----------	----------	----------	----------	----------	----------	-
Crocodylus_porosus	----------	----------	----------	----------	----------	----------	----------	-
Erinaceus_europaeus	----------	----------	----------	----------	----------	----------	----------	-
rnd-6_family-7426-LTR-ERV1	----------	----------	----------	----------	----------	----------	----------	-
Esox_lucius	----------	----------	----------	----------	----------	----------	----------	-
Salmo_salar	----------	----------	----------	----------	----------	----------	----------	-
Didelphidae	----------	----------	----------	----------	----------	----------	----------	-
Latimeria_chalumnae	----------	----------	----------	----------	----------	----------	----------	-
Monodelphis_domestica	----------	----------	----------	----------	----------	----------	----------	-
Macropus_eugenii	----------	----------	----------	----------	----------	----------	----------	-
Alligator_mississippiensis	----------	----------	----------	----------	----------	----------	----------	-
Chrysemys_picta_bellii	----------	----------	----------	----------	----------	----------	----------	-
Danio_rerio	----------	----------	----------	----------	----------	----------	----------	-
Taeniopygia	----------	----------	----------	----------	----------	----------	----------	-
Dasypus_novemcinctus	----------	----------	----------	----------	----------	----------	----------	-
LTR_XT	----------	----------	----------	----------	----------	----------	----------	-
Haplorrhini	NNNNTTGNNC	NNNNNNCTGC	NAGTNCTGCT	TNNTNCACNN	NCNTNGACNN	NNNNNANNNN	NTNNTNNNNA	3489
Catarrhini	TTGTTAGGGC	TGCTGCCGGC	TGAGGACAGC	TGGTTTACCT	GTCTGGACTT	AAAAGATGCC	TTCTTTAGCA	2979
Anolis_carolinensis	----------	----------	----------	----------	----------	----------	----------	-
Murine_leukemia_virus	CTCTTAAGCG	GGCTCCCNCC	GTCCCACCAG	TGGTACACTG	TGCTTGATTT	AAAGGATGCC	TTTTTCTGCC	2948
Phascolarctos_cinereus	CTACTCAGTT	CCCTCCCGCC	CAGCCACACC	TGGTATTCAG	TTTTAGACCT	CAAAGATGCT	TTCTTTTGCC	2845
Estrildidae	----------	----------	----------	----------	----------	----------	----------	-
Xenopus_Silurana_tropicalis	----------	----------	----------	----------	----------	----------	----------	-
I_XT	CTAGAGGTAC	ANACTANANN	TGCGACTATN	CCAGCNGTNG	TNNNNCAATT	CANGGNTGTT	NAAATNAGTG	2978
Takifugu_rubripes	----------	----------	----------	----------	----------	----------	----------	-
Bos_taurus	----------	----------	----------	----------	----------	----------	----------	-
Homo_sapiens	----------	----------	----------	----------	----------	----------	----------	-
Microcebus_murinus	----------	----------	----------	----------	----------	----------	----------	-
Myotis_lucifugus	----------	----------	----------	----------	----------	----------	----------	-
Equus_caballus	----------	----------	----------	----------	----------	----------	----------	-
Tupaia_belangeri	----------	----------	----------	----------	----------	----------	----------	-
Loxodonta_africana	----------	----------	----------	----------	----------	----------	----------	-
Murinae	----------	----------	----------	----------	----------	----------	----------	-
Canis_lupus_familiaris	----------	----------	----------	----------	----------	----------	----------	-
Sus_scrofa	----------	----------	----------	----------	----------	----------	----------	-
Choloepus_hoffmanni	----------	----------	----------	----------	----------	----------	----------	-
Vicugna_pacos	----------	----------	----------	----------	----------	----------	----------	-
Gavialis_gangeticus	----------	----------	----------	----------	----------	----------	----------	-
Mammalia	----------	----------	----------	----------	----------	----------	----------	-
Passeroidea	----------	----------	----------	----------	----------	----------	----------	-
Takifugu	----------	----------	----------	----------	----------	----------	----------	-
Pteropus_vampyrus	----------	----------	----------	----------	----------	----------	----------	-
Ornithorhynchus	----------	----------	----------	----------	----------	----------	----------	-
Oryctolagus_cuniculus	----------	----------	----------	----------	----------	----------	----------	-
Gallus_gallus	----------	----------	----------	----------	----------	----------	----------	-
Hominidae	----------	----------	----------	----------	----------	----------	----------	-
Metatheria	----------	----------	----------	----------	----------	----------	----------	-
Rodentia	----------	----------	----------	----------	----------	----------	----------	-
Monodelphis	----------	----------	----------	----------	----------	----------	----------	-
Consensus	----------	----------	----------	----------	----------	----------	----------	

Conservation 100% 0%

```
                                              3,520                         3,540                         3,560
Dipodomys_ordii                 ---------- ---------- ---------- ---------- ---------- ---------- ---------- -
Laurasiatheria                  ---------- ---------- ---------- ---------- ---------- ---------- ---------- -
Eutheria                        ---------- ---------- ---------- ---------- ---------- ---------- ---------- -
Tarsius_syrichta                ---------- ---------- ---------- ---------- ---------- ---------- ---------- -
Muridae                         ---------- ---------- ---------- ---------- ---------- ---------- ---------- -
Gasterosteus_aculeatus          ---------- ---------- ---------- ---------- ---------- ---------- ---------- -
Felis_silvestris                ---------- ---------- ---------- ---------- ---------- ---------- ---------- -
Rattus                          ---------- ---------- ---------- ---------- ---------- ---------- ---------- -
Papio_hamadryas                 CTCCCGGATG AGTCGCAGCA AGACCTCCTC TTCCAAGACC CTCTACCTCA TCCACCACAT AATCCCCTCC 883
Pan_troglodytes                 ---------- ---------- ---------- ---------- ---------- ---------- ---------- -
Ailuropoda_melanoleuca          ---------- ---------- ---------- ---------- ---------- ---------- ---------- -
Cavia_porcellus                 ---------- ---------- ---------- ---------- ---------- ---------- ---------- -
Ictidomys_tridecemlineatus      ---------- ---------- ---------- ---------- ---------- ---------- ---------- -
Canis                           ---------- ---------- ---------- ---------- ---------- ---------- ---------- -
Felis_catus                     ---------- ---------- ---------- ---------- ---------- ---------- ---------- -
Ochotona_princeps               ---------- ---------- ---------- ---------- ---------- ---------- ---------- -
Cercopithecidae                 ---------- ---------- ---------- ---------- ---------- ---------- ---------- -
Carnivora                       ---------- ---------- ---------- ---------- ---------- ---------- ---------- -
Mus                             ---------- ---------- ---------- ---------- ---------- ---------- ---------- -
Mus_musculus                    ---------- ---------- ---------- ---------- ---------- ---------- ---------- -
Rattus_norvegicus               ---------- ---------- ---------- ---------- ---------- ---------- ---------- -
Tursiops_truncatus              ---------- ---------- ---------- ---------- ---------- ---------- ---------- -
Procavia_capensis               ---------- ---------- ---------- ---------- ---------- ---------- ---------- -
Primates                        ---------- ---------- ---------- ---------- ---------- ---------- ---------- -
Echinops_telfairi               ---------- ---------- ---------- ---------- ---------- ---------- ---------- -
Sorex_araneus                   ---------- ---------- ---------- ---------- ---------- ---------- ---------- -
Canidae                         ---------- ---------- ---------- ---------- ---------- ---------- ---------- -
Otolemur_garnettii              ---------- ---------- ---------- ---------- ---------- ---------- ---------- -
Gallus                          ---------- ---------- ---------- ---------- ---------- ---------- ---------- -
Callithrix_jacchus              ---------- ---------- ---------- ---------- ---------- ---------- ---------- -
Crocodylus_porosus              ---------- ---------- ---------- ---------- ---------- ---------- ---------- -
Erinaceus_europaeus             ---------- ---------- ---------- ---------- ---------- ---------- ---------- -
rnd-6_family-7426-LTR-ERV1      ---------- ---------- ---------- ---------- ---------- ---------- ---------- -
Esox_lucius                     ---------- ---------- ---------- ---------- ---------- ---------- ---------- -
Salmo_salar                     ---------- ---------- ---------- ---------- ---------- ---------- ---------- -
Didelphidae                     ---------- ---------- ---------- ---------- ---------- ---------- ---------- -
Latimeria_chalumnae             ---------- ---------- ---------- ---------- ---------- ---------- ---------- -
Monodelphis_domestica           ---------- ---------- ---------- ---------- ---------- ---------- ---------- -
Macropus_eugenii                ---------- ---------- ---------- ---------- ---------- ---------- ---------- -
Alligator_mississippiensis      ---------- ---------- ---------- ---------- ---------- ---------- ---------- -
Chrysemys_picta_bellii          ---------- ---------- ---------- ---------- ---------- ---------- ---------- -
Danio_rerio                     ---------- ---------- ---------- ---------- ---------- ---------- ---------- -
Taeniopygia                     ---------- ---------- ---------- ---------- ---------- ---------- ---------- -
Dasypus_novemcinctus            ---------- ---------- ---------- ---------- ---------- ---------- ---------- -
LTR_XT                          ---------- ---------- ---------- ---------- ---------- ---------- ---------- -
Haplorrhini                     NNNGATTNNN CCCTNTNNNC NGNGACATNT NNGNCTTTGA NTGGNN---- ---------- ----CCNANN 3541
Catarrhini                      TCAGACTAGC TCCTGAGAGC CAGAAGCTGT TTGCCTTTCA GTGGGAAGAT CCGGAGTCAG GTGTCACTAC 3049
Anolis_carolinensis             ---------- ---------- ---------- ---------- ---------- ---------- ---------- -
Murine_leukemia_virus           TGAGACTCCA CCCCACCAGT CAGCCTCTCT TCGCCTTTGA GTGGAGAGAT CCAGGGATGG GAATCTCNGG 3018
Phascolarctos_cinereus          TCAAGCTACA CCCCAACAGC CAGCCATTGT TCGCGTTTGA ATGGAGAGAT CCAGAAAAAG GCAATACTGG 2915
Estrildidae                     ---------- ---------- ---------- ---------- ---------- ---------- ---------- -
Xenopus_Silurana_tropicalis     ---------- ---------- ---------- ---------- ---------- ---------- ---------- -
I_XT                            GATGCCTAGA NTTNTGTCGC CANTAGAGAN AAGATTCAAT GCTTGTTTGC GTTAACTTTT AGNNGTCANC 3048
Takifugu_rubripes               ---------- ---------- ---------- ---------- ---------- ---------- ---------- -
Bos_taurus                      ---------- ---------- ---------- ---------- ---------- ---------- ---------- -
Homo_sapiens                    ---------- ---------- ---------- ---------- ---------- ---------- ---------- -
Microcebus_murinus              ---------- ---------- ---------- ---------- ---------- ---------- ---------- -
Myotis_lucifugus                ---------- ---------- ---------- ---------- ---------- ---------- ---------- -
Equus_caballus                  ---------- ---------- ---------- ---------- ---------- ---------- ---------- -
Tupaia_belangeri                ---------- ---------- ---------- ---------- ---------- ---------- ---------- -
Loxodonta_africana              ---------- ---------- ---------- ---------- ---------- ---------- ---------- -
Murinae                         ---------- ---------- ---------- ---------- ---------- ---------- ---------- -
Canis_lupus_familiaris          ---------- ---------- ---------- ---------- ---------- ---------- ---------- -
Sus_scrofa                      ---------- ---------- ---------- ---------- ---------- ---------- ---------- -
Choloepus_hoffmanni             ---------- ---------- ---------- ---------- ---------- ---------- ---------- -
Vicugna_pacos                   ---------- ---------- ---------- ---------- ---------- ---------- ---------- -
Gavialis_gangeticus             ---------- ---------- ---------- ---------- ---------- ---------- ---------- -
Mammalia                        ---------- ---------- ---------- ---------- ---------- ---------- ---------- -
Passeroidea                     ---------- ---------- ---------- ---------- ---------- ---------- ---------- -
Takifugu                        ---------- ---------- ---------- ---------- ---------- ---------- ---------- -
Pteropus_vampyrus               ---------- ---------- ---------- ---------- ---------- ---------- ---------- -
Ornithorhynchus                 ---------- ---------- ---------- ---------- ---------- ---------- ---------- -
Oryctolagus_cuniculus           ---------- ---------- ---------- ---------- ---------- ---------- ---------- -
Gallus_gallus                   ---------- ---------- ---------- ---------- ---------- ---------- ---------- -
Hominidae                       ---------- ---------- ---------- ---------- ---------- ---------- ---------- -
Metatheria                      ---------- ---------- ---------- ---------- ---------- ---------- ---------- -
Rodentia                        ---------- ---------- ---------- ---------- ---------- ---------- ---------- -
Monodelphis                     ---------- ---------- ---------- ---------- ---------- ---------- ---------- -
Consensus                       ---------- ---------- ---------- ---------- ---------- ---------- ----------
                           100%
Conservation
                             0%
```

3,580 | 3,600 | 3,620 | 3,640

Dipodomys_ordii	----------	----------	----------	----------	----------	----------	----------	-
Laurasiatheria	----------	----------	----------	----------	----------	----------	----------	-
Eutheria	----------	----------	----------	----------	----------	----------	----------	-
Tarsius_syrichta	----------	----------	----------	----------	----------	----------	----------	-
Muridae	----------	----------	----------	----------	----------	----------	----------	-
Gasterosteus_aculeatus	----------	----------	----------	----------	----------	----------	----------	-
Felis_silvestris	----------	----------	----------	----------	----------	----------	----------	-
Rattus	----------	----------	----------	----------	----------	----------	----------	-
Papio_hamadryas	TGGAACCCCC	ACCCTACAAC	TCACCCTCGC	CCCCTGTCTT	GTCCCCCGTT	TCTCCTACCA	CCCCTTCTGC	953
Pan_troglodytes	----------	----------	----------	----------	----------	----------	----------	-
Ailuropoda_melanoleuca	----------	----------	----------	----------	----------	----------	----------	-
Cavia_porcellus	----------	----------	----------	----------	----------	----------	----------	-
Ictidomys_tridecemlineatus	----------	----------	----------	----------	----------	----------	----------	-
Canis	----------	----------	----------	----------	----------	----------	----------	-
Felis_catus	----------	----------	----------	----------	----------	----------	----------	-
Ochotona_princeps	----------	----------	----------	----------	----------	----------	----------	-
Cercopithecidae	----------	----------	----------	----------	----------	----------	----------	-
Carnivora	----------	----------	----------	----------	----------	----------	----------	-
Mus	----------	----------	----------	----------	----------	----------	----------	-
Mus_musculus	----------	----------	----------	----------	----------	----------	----------	-
Rattus_norvegicus	----------	----------	----------	----------	----------	----------	----------	-
Tursiops_truncatus	----------	----------	----------	----------	----------	----------	----------	-
Procavia_capensis	----------	----------	----------	----------	----------	----------	----------	-
Primates	----------	----------	----------	----------	----------	----------	----------	-
Echinops_telfairi	----------	----------	----------	----------	----------	----------	----------	-
Sorex_araneus	----------	----------	----------	----------	----------	----------	----------	-
Canidae	----------	----------	----------	----------	----------	----------	----------	-
Otolemur_garnettii	----------	----------	----------	----------	----------	----------	----------	-
Gallus	----------	----------	----------	----------	----------	----------	----------	-
Callithrix_jacchus	----------	----------	----------	----------	----------	----------	----------	-
Crocodylus_porosus	----------	----------	----------	----------	----------	----------	----------	-
Erinaceus_europaeus	----------	----------	----------	----------	----------	----------	----------	-
rnd-6_family-7426-LTR-ERV1	----------	----------	----------	----------	----------	----------	----------	-
Esox_lucius	----------	----------	----------	----------	----------	----------	----------	-
Salmo_salar	----------	----------	----------	----------	----------	----------	----------	-
Didelphidae	----------	----------	----------	----------	----------	----------	----------	-
Latimeria_chalumnae	----------	----------	----------	----------	----------	----------	----------	-
Monodelphis_domestica	----------	----------	----------	----------	----------	----------	----------	-
Macropus_eugenii	----------	----------	----------	----------	----------	----------	----------	-
Alligator_mississippiensis	----------	----------	----------	----------	----------	----------	----------	-
Chrysemys_picta_bellii	----------	----------	----------	----------	----------	----------	----------	-
Danio_rerio	----------	----------	----------	----------	----------	----------	----------	-
Taeniopygia	----------	----------	----------	----------	----------	----------	----------	-
Dasypus_novemcinctus	----------	----------	----------	----------	----------	----------	----------	-
LTR_XT	----------	----------	----------	----------	----------	----------	----------	-
Haplorrhini	NCNNNNNANC	NGGACTNGAN	NNCCNNAANN	ATNTAANNNC	TCNCCNNNNN	TCTNTNNNGN	NNCNCNNGNC	3611
Catarrhini	TCAGTACACT	TGGACCCGGC	TTCCCCAAGG	GTTCGAGAAC	TCCCCTACTA	TCTTCGGGGA	GGCCCTGGCT	3119
Anolis_carolinensis	----------	----------	----------	----------	----------	----------	----------	-
Murine_leukemia_virus	ACAATTGACC	TGGACCAGAC	TCCCACAGGG	TTTCAAAAAC	AGTCCCACCC	TGTTTGATGA	GGCACTGCAC	3088
Phascolarctos_cinereus	CCAGCTAACC	TGGACTCGGC	TACCGCAAGG	ATTCAAGAAC	TCTCCCACCC	TCTTCGATGA	GGCCCTCCAC	2985
Estrildidae	----------	----------	----------	----------	----------	----------	----------	-
Xenopus_Silurana_tropicalis	----------	----------	----------	----------	----------	----------	----------	-
I_XT	TTTNTACNTG	CGNTCGACTA	NNTCATGGGA	TTTANNAACN	CTCCCTGCAA	ACTCCACACA	CACCACTTAA	3118
Takifugu_rubripes	----------	----------	----------	----------	----------	----------	----------	-
Bos_taurus	----------	----------	----------	----------	----------	----------	----------	-
Homo_sapiens	----------	----------	----------	----------	----------	----------	----------	-
Microcebus_murinus	----------	----------	----------	----------	----------	----------	----------	-
Myotis_lucifugus	----------	----------	----------	----------	----------	----------	----------	-
Equus_caballus	----------	----------	----------	----------	----------	----------	----------	-
Tupaia_belangeri	----------	----------	----------	----------	----------	----------	----------	-
Loxodonta_africana	----------	----------	----------	----------	----------	----------	----------	-
Murinae	----------	----------	----------	----------	----------	----------	----------	-
Canis_lupus_familiaris	----------	----------	----------	----------	----------	----------	----------	-
Sus_scrofa	----------	----------	----------	----------	----------	----------	----------	-
Choloepus_hoffmanni	----------	----------	----------	----------	----------	----------	----------	-
Vicugna_pacos	----------	----------	----------	----------	----------	----------	----------	-
Gavialis_gangeticus	----------	----------	----------	----------	----------	----------	----------	-
Mammalia	----------	----------	----------	----------	----------	----------	----------	-
Passeroidea	----------	----------	----------	----------	----------	----------	----------	-
Takifugu	----------	----------	----------	----------	----------	----------	----------	-
Pteropus_vampyrus	----------	----------	----------	----------	----------	----------	----------	-
Ornithorhynchus	----------	----------	----------	----------	----------	----------	----------	-
Oryctolagus_cuniculus	----------	----------	----------	----------	----------	----------	----------	-
Gallus_gallus	----------	----------	----------	----------	----------	----------	----------	-
Hominidae	----------	----------	----------	----------	----------	----------	----------	-
Metatheria	----------	----------	----------	----------	----------	----------	----------	-
Rodentia	----------	----------	----------	----------	----------	----------	----------	-
Monodelphis	----------	----------	----------	----------	----------	----------	----------	-
Consensus	----------	----------	----------	----------	----------	----------	----------	

Conservation 100% 0%

		3,660		3,680		3,700		
Dipodomys_ordii	----------	----------	----------	----------	----------	----------	----------	-
Laurasiatheria	----------	----------	----------	----------	----------	----------	----------	-
Eutheria	----------	----------	----------	----------	----------	----------	----------	-
Tarsius_syrichta	----------	----------	----------	----------	----------	----------	----------	-
Muridae	----------	----------	----------	----------	----------	----------	----------	-
Gasterosteus_aculeatus	----------	----------	----------	----------	----------	----------	----------	-
Felis_silvestris	----------	----------	----------	----------	----------	----------	----------	-
Rattus	----------	----------	----------	----------	----------	----------	----------	-
Papio_hamadryas	CCCCACTCCT	TCCTCTCTTG	TCTCCTCGTC	GACCCCGCCT	TCCTCTCCAG	CCCCACCTGA	ACTCACCCCC	1023
Pan_troglodytes	----------	----------	----------	----------	----------	----------	----------	-
Ailuropoda_melanoleuca	----------	----------	----------	----------	----------	----------	----------	-
Cavia_porcellus	----------	----------	----------	----------	----------	----------	----------	-
Ictidomys_tridecemlineatus	----------	----------	----------	----------	----------	----------	----------	-
Canis	----------	----------	----------	----------	----------	----------	----------	-
Felis_catus	----------	----------	----------	----------	----------	----------	----------	-
Ochotona_princeps	----------	----------	----------	----------	----------	----------	----------	-
Cercopithecidae	----------	----------	----------	----------	----------	----------	----------	-
Carnivora	----------	----------	----------	----------	----------	----------	----------	-
Mus	----------	----------	----------	----------	----------	----------	----------	-
Mus_musculus	----------	----------	----------	----------	----------	----------	----------	-
Rattus_norvegicus	----------	----------	----------	----------	----------	----------	----------	-
Tursiops_truncatus	----------	----------	----------	----------	----------	----------	----------	-
Procavia_capensis	----------	----------	----------	----------	----------	----------	----------	-
Primates	----------	----------	----------	----------	----------	----------	----------	-
Echinops_telfairi	----------	----------	----------	----------	----------	----------	----------	-
Sorex_araneus	----------	----------	----------	----------	----------	----------	----------	-
Canidae	----------	----------	----------	----------	----------	----------	----------	-
Otolemur_garnettii	----------	----------	----------	----------	----------	----------	----------	-
Gallus	----------	----------	----------	----------	----------	----------	----------	-
Callithrix_jacchus	----------	----------	----------	----------	----------	----------	----------	-
Crocodylus_porosus	----------	----------	----------	----------	----------	----------	----------	-
Erinaceus_europaeus	----------	----------	----------	----------	----------	----------	----------	-
rnd-6_family-7426-LTR-ERV1	----------	----------	----------	----------	----------	----------	----------	-
Esox_lucius	----------	----------	----------	----------	----------	----------	----------	-
Salmo_salar	----------	----------	----------	----------	----------	----------	----------	-
Didelphidae	----------	----------	----------	----------	----------	----------	----------	-
Latimeria_chalumnae	----------	----------	----------	----------	----------	----------	----------	-
Monodelphis_domestica	----------	----------	----------	----------	----------	----------	----------	-
Macropus_eugenii	----------	----------	----------	----------	----------	----------	----------	-
Alligator_mississippiensis	----------	----------	----------	----------	----------	----------	----------	-
Chrysemys_picta_bellii	----------	----------	----------	----------	----------	----------	----------	-
Danio_rerio	----------	----------	----------	----------	----------	----------	----------	-
Taeniopygia	----------	----------	----------	----------	----------	----------	----------	-
Dasypus_novemcinctus	----------	----------	----------	----------	----------	----------	----------	-
LTR_XT	----------	----------	----------	----------	----------	----------	----------	-
Haplorrhini	TCNGANTTNA	AGGCTNNCNC	NNCACCNAGC	GACCGNTGTG	TCTTACTGCA	ATATATAGAT	GATCTGCTGT	3681
Catarrhini	CGAGACCTGC	AAAAGTTTCC	TGCTAAAGAC	CTAGGCTGCG	TCTTGCTCCT	GTACGTGGAC	GACCTTCTGC	3189
Anolis_carolinensis	----------	----------	----------	----------	----------	----------	----------	-
Murine_leukemia_virus	AGAGACCTAG	CAGACTTCCG	GATCCAGCAC	CCAGACTTGA	TCCTGCTACA	GTACGTGGAT	GACNTACTGC	3158
Phascolarctos_cinereus	CGGGATCTGG	CATCNNNNNG	NGNNNNNAAN	CNNNAGGNAG	NNATNNTNCN	NTNNNNTGAT	GNNNNCNNNN	3055
Estrildidae	----------	----------	----------	----------	----------	----------	----------	-
Xenopus_Silurana_tropicalis	----------	----------	----------	----------	----------	----------	----------	-
I_XT	GCTGCTGTTT	GANAGANTTG	CGNGNCTGTC	TANGGAACGA	TTGCNCNATN	GTATGTTGAN	GTCTTGCTNA	3188
Takifugu_rubripes	----------	----------	----------	----------	----------	----------	----------	-
Bos_taurus	----------	----------	----------	----------	----------	----------	----------	-
Homo_sapiens	----------	----------	----------	----------	----------	----------	----------	-
Microcebus_murinus	----------	----------	----------	----------	----------	----------	----------	-
Myotis_lucifugus	----------	----------	----------	----------	----------	----------	----------	-
Equus_caballus	----------	----------	----------	----------	----------	----------	----------	-
Tupaia_belangeri	----------	----------	----------	----------	----------	----------	----------	-
Loxodonta_africana	----------	----------	----------	----------	----------	----------	----------	-
Murinae	----------	----------	----------	----------	----------	----------	----------	-
Canis_lupus_familiaris	----------	----------	----------	----------	----------	----------	----------	-
Sus_scrofa	----------	----------	----------	----------	----------	----------	----------	-
Choloepus_hoffmanni	----------	----------	----------	----------	----------	----------	----------	-
Vicugna_pacos	----------	----------	----------	----------	----------	----------	----------	-
Gavialis_gangeticus	----------	----------	----------	----------	----------	----------	----------	-
Mammalia	----------	----------	----------	----------	----------	----------	----------	-
Passeroidea	----------	----------	----------	----------	----------	----------	----------	-
Takifugu	----------	----------	----------	----------	----------	----------	----------	-
Pteropus_vampyrus	----------	----------	----------	----------	----------	----------	----------	-
Ornithorhynchus	----------	----------	----------	----------	----------	----------	----------	-
Oryctolagus_cuniculus	----------	----------	----------	----------	----------	----------	----------	-
Gallus_gallus	----------	----------	----------	----------	----------	----------	----------	-
Hominidae	----------	----------	----------	----------	----------	----------	----------	-
Metatheria	----------	----------	----------	----------	----------	----------	----------	-
Rodentia	----------	----------	----------	----------	----------	----------	----------	-
Monodelphis	----------	----------	----------	----------	----------	----------	----------	-
Consensus	----------	----------	----------	----------	----------	----------	----------	

Conservation (100% – 0%)

	3,720		3,740		3,760		3,780	
Dipodomys_ordii	----------	----------	----------	----------	----------	----------	----------	-
Laurasiatheria	----------	----------	----------	----------	----------	----------	----------	-
Eutheria	----------	----------	----------	----------	----------	----------	----------	-
Tarsius_syrichta	----------	----------	----------	----------	----------	----------	----------	-
Muridae	----------	----------	----------	----------	----------	----------	----------	-
Gasterosteus_aculeatus	----------	----------	----------	----------	----------	----------	----------	-
Felis_silvestris	----------	----------	----------	----------	----------	----------	----------	-
Rattus	----------	----------	----------	----------	----------	----------	----------	-
Papio_hamadryas	AGGACCCCGC	CACAAACCCC	CCGTCTCCGC	CTCCGGCGGG	CCGAAGGTCA	GGATGGCCCT	TCCACCTGGC	1093
Pan_troglodytes	----------	----------	----------	----------	----------	----------	----------	-
Ailuropoda_melanoleuca	----------	----------	----------	----------	----------	----------	----------	-
Cavia_porcellus	----------	----------	----------	----------	----------	----------	----------	-
Ictidomys_tridecemlineatus	----------	----------	----------	----------	----------	----------	----------	-
Canis	----------	----------	----------	----------	----------	----------	----------	-
Felis_catus	----------	----------	----------	----------	----------	----------	----------	-
Ochotona_princeps	----------	----------	----------	----------	----------	----------	----------	-
Cercopithecidae	----------	----------	----------	----------	----------	----------	----------	-
Carnivora	----------	----------	----------	----------	----------	----------	----------	-
Mus	----------	----------	----------	----------	----------	----------	----------	-
Mus_musculus	----------	----------	----------	----------	----------	----------	----------	-
Rattus_norvegicus	----------	----------	----------	----------	----------	----------	----------	-
Tursiops_truncatus	----------	----------	----------	----------	----------	----------	----------	-
Procavia_capensis	----------	----------	----------	----------	----------	----------	----------	-
Primates	----------	----------	----------	----------	----------	----------	----------	-
Echinops_telfairi	----------	----------	----------	----------	----------	----------	----------	-
Sorex_araneus	----------	----------	----------	----------	----------	----------	----------	-
Canidae	----------	----------	----------	----------	----------	----------	----------	-
Otolemur_garnettii	----------	----------	----------	----------	----------	----------	----------	-
Gallus	----------	----------	----------	----------	----------	----------	----------	-
Callithrix_jacchus	----------	----------	----------	----------	----------	----------	----------	-
Crocodylus_porosus	----------	----------	----------	----------	----------	----------	----------	-
Erinaceus_europaeus	----------	----------	----------	----------	----------	----------	----------	-
rnd-6_family-7426-LTR-ERV1	----------	----------	----------	----------	----------	----------	----------	-
Esox_lucius	----------	----------	----------	----------	----------	----------	----------	-
Salmo_salar	----------	----------	----------	----------	----------	----------	----------	-
Didelphidae	----------	----------	----------	----------	----------	----------	----------	-
Latimeria_chalumnae	----------	----------	----------	----------	----------	----------	----------	-
Monodelphis_domestica	----------	----------	----------	----------	----------	----------	----------	-
Macropus_eugenii	----------	----------	----------	----------	----------	----------	----------	-
Alligator_mississippiensis	----------	----------	----------	----------	----------	----------	----------	-
Chrysemys_picta_bellii	----------	----------	----------	----------	----------	----------	----------	-
Danio_rerio	----------	----------	----------	----------	----------	----------	----------	-
Taeniopygia	----------	----------	----------	----------	----------	----------	----------	-
Dasypus_novemcinctus	----------	----------	----------	----------	----------	----------	----------	-
LTR_XT	----------	----------	----------	----------	----------	----------	----------	-
Haplorrhini	TAGCCGCACC	CACNAGAAAG	GAATGTATCC	AAGGAACAGA	GA-GTCTCCT	TCGAGTGCTG	TGGGAAGCTG	3750
Catarrhini	TGGGACACTC	CACGGCAGTC	GGGTGTGCAA	AAGGGATGGA	TG-CCCTGCT	TCGGCACCTG	GAGGACTGTG	3258
Anolis_carolinensis	----------	----------	----------	----------	----------	----------	----------	-
Murine_leukemia_virus	TGGCCGCCAC	TTCCGAGCTC	GACTGCCAAC	AAGGTACTCG	GG-CCCTNCT	ACAAACCCTA	GGGGACCTCG	3227
Phascolarctos_cinereus	NNGNGGNCCC	AANGTNNNGN	GACNGNNNNN	NNNNGNCACA	GNANNNNNTN	ANAGNAANNA	ANNNANNNNN	3125
Estrildidae	----------	----------	----------	----------	----------	----------	----------	-
Xenopus_Silurana_tropicalis	----------	----------	----------	----------	----------	----------	----------	-
I_XT	GNANNGCAAN	CTCGAGAAAC	AGGTAAANTA	CATNCTTCCA	CNTCTCTGCT	ATAATATCTA	CATGAATGTG	3258
Takifugu_rubripes	----------	----------	----------	----------	----------	----------	----------	-
Bos_taurus	----------	----------	----------	----------	----------	----------	----------	-
Homo_sapiens	----------	----------	----------	----------	----------	----------	----------	-
Microcebus_murinus	----------	----------	----------	----------	----------	----------	----------	-
Myotis_lucifugus	----------	----------	----------	----------	----------	----------	----------	-
Equus_caballus	----------	----------	----------	----------	----------	----------	----------	-
Tupaia_belangeri	----------	----------	----------	----------	----------	----------	----------	-
Loxodonta_africana	----------	----------	----------	----------	----------	----------	----------	-
Murinae	----------	----------	----------	----------	----------	----------	----------	-
Canis_lupus_familiaris	----------	----------	----------	----------	----------	----------	----------	-
Sus_scrofa	----------	----------	----------	----------	----------	----------	----------	-
Choloepus_hoffmanni	----------	----------	----------	----------	----------	----------	----------	-
Vicugna_pacos	----------	----------	----------	----------	----------	----------	----------	-
Gavialis_gangeticus	----------	----------	----------	----------	----------	----------	----------	-
Mammalia	----------	----------	----------	----------	----------	----------	----------	-
Passeroidea	----------	----------	----------	----------	----------	----------	----------	-
Takifugu	----------	----------	----------	----------	----------	----------	----------	-
Pteropus_vampyrus	----------	----------	----------	----------	----------	----------	----------	-
Ornithorhynchus	----------	----------	----------	----------	----------	----------	----------	-
Oryctolagus_cuniculus	----------	----------	----------	----------	----------	----------	----------	-
Gallus_gallus	----------	----------	----------	----------	----------	----------	----------	-
Hominidae	----------	----------	-----TTGGT	GCCAAAACCC	GGGAGGAGAC	CCCTCTCTGA	CCCAGGGTCG	45
Metatheria	----------	----------	----------	----------	----------	----------	----------	-
Rodentia	----------	----------	----------	----------	----------	----------	----------	-
Monodelphis	----------	----------	----------	----------	----------	----------	----------	-
Consensus	----------	----------	----------	----------	----------	----------	----------	

Conservation 100% 0%

		3,800		3,820		3,840		
Dipodomys_ordii	----------	----------	----------	----------	----------	----------	----------	-
Laurasiatheria	----------	----------	----------	----------	----------	----------	----------	-
Eutheria	----------	----------	----------	----------	----------	----------	----------	-
Tarsius_syrichta	----------	----------	----------	----------	----------	----------	----------	-
Muridae	----------	----------	----------	----------	----------	----------	----------	-
Gasterosteus_aculeatus	----------	----------	----------	----------	----------	----------	----------	-
Felis_silvestris	----------	----------	----------	----------	----------	----------	----------	-
Rattus	----------	----------	----------	----------	----------	----------	----------	-
Papio_hamadryas	AATCTTCCCT	TTTTCCCCTT	CGCACGGTCA	ACCGCACGAT	CCAGTACTGG	CCCTTTTCTG	CCTCGGACCT	1163
Pan_troglodytes	----------	----------	----------	----------	----------	----------	----------	-
Ailuropoda_melanoleuca	----------	----------	----------	----------	----------	----------	----------	-
Cavia_porcellus	----------	----------	----------	----------	----------	----------	----------	-
Ictidomys_tridecemlineatus	----------	----------	----------	----------	----------	----------	----------	-
Canis	----------	----------	----------	----------	----------	----------	----------	-
Felis_catus	----------	----------	----------	----------	----------	----------	----------	-
Ochotona_princeps	----------	----------	----------	----------	----------	----------	----------	-
Cercopithecidae	----------	----------	----------	----------	----------	----------	----------	-
Carnivora	----------	----------	----------	----------	----------	----------	----------	-
Mus	----------	----------	----------	----------	----------	----------	----------	-
Mus_musculus	----------	----------	----------	----------	----------	----------	----------	-
Rattus_norvegicus	----------	----------	----------	----------	----------	----------	----------	-
Tursiops_truncatus	----------	----------	----------	----------	----------	----------	----------	-
Procavia_capensis	----------	----------	----------	----------	----------	----------	----------	-
Primates	----------	----------	----------	----------	----------	----------	----------	-
Echinops_telfairi	----------	----------	----------	----------	----------	----------	----------	-
Sorex_araneus	----------	----------	----------	----------	----------	----------	----------	-
Canidae	----------	----------	----------	----------	----------	----------	----------	-
Otolemur_garnettii	----------	----------	----------	----------	----------	----------	----------	-
Gallus	----------	----------	----------	----------	----------	----------	----------	-
Callithrix_jacchus	----------	----------	----------	----------	----------	----------	----------	-
Crocodylus_porosus	----------	----------	----------	----------	----------	----------	----------	-
Erinaceus_europaeus	----------	----------	----------	----------	----------	----------	----------	-
rnd-6_family-7426-LTR-ERV1	----------	----------	----------	----------	----------	----------	----------	-
Esox_lucius	----------	----------	----------	----------	----------	----------	----------	-
Salmo_salar	----------	----------	----------	----------	----------	----------	----------	-
Didelphidae	----------	----------	----------	----------	----------	----------	----------	-
Latimeria_chalumnae	----------	----------	----------	----------	----------	----------	----------	-
Monodelphis_domestica	----------	----------	----------	----------	----------	----------	----------	-
Macropus_eugenii	----------	----------	----------	----------	----------	----------	----------	-
Alligator_mississippiensis	----------	----------	----------	----------	----------	----------	----------	-
Chrysemys_picta_bellii	----------	----------	----------	----------	----------	----------	----------	-
Danio_rerio	----------	----------	----------	----------	----------	----------	----------	-
Taeniopygia	----------	----------	----------	----------	----------	----------	----------	-
Dasypus_novemcinctus	----------	----------	----------	----------	----------	----------	----------	-
LTR_XT	----------	----------	----------	----------	----------	----------	----------	-
Haplorrhini	GCTATAAAGT	GTCTAAGGAA	AAGGCACAGA	TCTGTGGCCA	AGGAGTTCAG	TATCTTGGCT	TTTACGTCTC	3820
Catarrhini	GGTATAAGGT	GTCCAAGAAG	AAAGCTCAGA	TCTGCAGACA	GCAGGTACGC	TACCTGGGAT	TCACTATTCG	3328
Anolis_carolinensis	----------	----------	----------	----------	----------	----------	----------	-
Murine_leukemia_virus	GATACCGGGC	CTCGGCCAAG	AAAGCCCAAA	TCTGCCAGAA	ACAGGTCAAG	TACCTGGGGT	ACCTTCTGAG	3297
Phascolarctos_cinereus	GATNNNGAGT	GNCNNNCNNN	ANNNCNNNGN	TNNNNCNNNN	GNNAGTTNCN	NANCNCGGNN	NTNTGCTAAA	3195
Estrildidae	----------	----------	----------	----------	----------	----------	----------	-
Xenopus_Silurana_tropicalis	----------	----------	----------	----------	----------	----------	----------	-
I_XT	GATTTAATAN	ANCACCTNTC	AAATTGCATT	TCNGTCAGNG	GCAAGTACAN	TATATGGGAC	NGTGTATNTC	3328
Takifugu_rubripes	----------	----------	----------	----------	----------	----------	----------	-
Bos_taurus	----------	----------	----------	----------	----------	----------	----------	-
Homo_sapiens	----------	----------	----------	----------	----------	----------	----------	-
Microcebus_murinus	----------	----------	----------	----------	----------	----------	----------	-
Myotis_lucifugus	----------	----------	----------	----------	----------	----------	----------	-
Equus_caballus	----------	----------	----------	----------	----------	----------	----------	-
Tupaia_belangeri	----------	----------	----------	----------	----------	----------	----------	-
Loxodonta_africana	----------	----------	----------	----------	----------	----------	----------	-
Murinae	----------	----------	----------	----------	----------	----------	----------	-
Canis_lupus_familiaris	----------	----------	----------	----------	----------	----------	----------	-
Sus_scrofa	----------	----------	----------	----------	----------	----------	----------	-
Choloepus_hoffmanni	----------	----------	----------	----------	----------	----------	----------	-
Vicugna_pacos	----------	----------	----------	----------	----------	----------	----------	-
Gavialis_gangeticus	----------	----------	----------	----------	----------	----------	----------	-
Mammalia	----------	----------	----------	----------	----------	----------	----------	-
Passeroidea	----------	----------	----------	----------	----------	----------	----------	-
Takifugu	----------	----------	----------	----------	----------	----------	----------	-
Pteropus_vampyrus	----------	----------	----------	----------	----------	----------	----------	-
Ornithorhynchus	----------	----------	----------	----------	----------	----------	----------	-
Oryctolagus_cuniculus	----------	----------	----------	----------	----------	----------	----------	-
Gallus_gallus	----------	----------	----------	----------	----------	----------	----------	-
Hominidae	GGGAGCATCT	CCTCTCCCTA	CCTGCCAGGA	ACCAGACTCG	GGCCAGCGCA	TTCGGCCTTC	GCTATTGGGT	115
Metatheria	----------	----------	----------	----------	----------	----------	----------	-
Rodentia	----------	----------	----------	----------	----------	----------	----------	-
Monodelphis	----------	----------	----------	----------	----------	----------	----------	-
Consensus	----------	----------	----------	----------	----------	----------	----------	

Conservation (100% – 0%)

	3,860		3,880		3,900		3,920	
Dipodomys_ordii	----------	----------	----------	----------	----------	----------	----------	-
Laurasiatheria	----------	----------	----------	----------	----------	----------	----------	-
Eutheria	----------	----------	----------	----------	----------	----------	----------	-
Tarsius_syrichta	----------	----------	----------	----------	----------	----------	----------	-
Muridae	----------	----------	----------	----------	----------	----------	----------	-
Gasterosteus_aculeatus	----------	----------	----------	----------	----------	----------	----------	-
Felis_silvestris	----------	----------	----------	----------	----------	----------	----------	-
Rattus	----------	----------	----------	----------	----------	----------	----------	-
Papio_hamadryas	CTATAATTGG	AAAACCCATA	ACCCCTCCTT	TTCCCAAGAC	CCCCAGGCCT	TGACCTCGTT	GATAGAATCA	1233
Pan_troglodytes	----------	----------	----------	----------	----------	----------	----------	-
Ailuropoda_melanoleuca	----------	----------	----------	----------	----------	----------	----------	-
Cavia_porcellus	----------	----------	----------	----------	----------	----------	----------	-
Ictidomys_tridecemlineatus	----------	----------	----------	----------	----------	----------	----------	-
Canis	----------	----------	----------	----------	----------	----------	----------	-
Felis_catus	----------	----------	----------	----------	----------	----------	----------	-
Ochotona_princeps	----------	----------	----------	----------	----------	----------	----------	-
Cercopithecidae	----------	----------	----------	----------	----------	----------	----------	-
Carnivora	----------	----------	----------	----------	----------	----------	----------	-
Mus	----------	----------	----------	----------	----------	----------	----------	-
Mus_musculus	----------	----------	----------	----------	----------	----------	----------	-
Rattus_norvegicus	----------	----------	----------	----------	----------	----------	----------	-
Tursiops_truncatus	----------	----------	----------	----------	----------	----------	----------	-
Procavia_capensis	----------	----------	----------	----------	----------	----------	----------	-
Primates	----------	----------	----------	----------	----------	----------	----------	-
Echinops_telfairi	----------	----------	----------	----------	----------	----------	----------	-
Sorex_araneus	----------	----------	----------	----------	----------	----------	----------	-
Canidae	----------	----------	----------	----------	----------	----------	----------	-
Otolemur_garnettii	----------	----------	----------	----------	----------	----------	----------	-
Gallus	----------	----------	----------	----------	----------	----------	----------	-
Callithrix_jacchus	----------	----------	----------	----------	----------	----------	----------	-
Crocodylus_porosus	----------	----------	----------	----------	----------	----------	----------	-
Erinaceus_europaeus	----------	----------	----------	----------	----------	----------	----------	-
rnd-6_family-7426-LTR-ERV1	----------	----------	----------	----------	----------	----------	----------	-
Esox_lucius	----------	----------	----------	----------	----------	----------	----------	-
Salmo_salar	----------	----------	----------	----------	----------	----------	----------	-
Didelphidae	----------	----------	----------	----------	----------	----------	----------	-
Latimeria_chalumnae	----------	----------	----------	----------	----------	----------	----------	-
Monodelphis_domestica	----------	----------	----------	----------	----------	----------	----------	-
Macropus_eugenii	----------	----------	----------	----------	----------	----------	----------	-
Alligator_mississippiensis	----------	----------	----------	----------	----------	----------	----------	-
Chrysemys_picta_bellii	----------	----------	----------	----------	----------	----------	----------	-
Danio_rerio	----------	----------	----------	----------	----------	----------	----------	-
Taeniopygia	----------	----------	----------	----------	----------	----------	----------	-
Dasypus_novemcinctus	----------	----------	----------	----------	----------	----------	----------	-
LTR_XT	----------	----------	----------	----------	----------	----------	----------	-
Haplorrhini	CCAAGGGCGG	CGTGAGCTTG	GGCGGGAGCG	AAAAGAGACT	GTC-TGTAGC	ATTCCTCAGC	CAGACACAAG	3889
Catarrhini	GAAAGGGGAG	CGCAGCCTGG	GGTCAGAAAG	AAAGCAGGTC	ATC-TGCAGC	CTACCGGAAC	CTAGAACCAG	3397
Anolis_carolinensis	----------	----------	----------	----------	----------	----------	----------	-
Murine_leukemia_virus	GGAGGGTCAG	AGATGGCTGA	CTGAGGCCAG	AAAAGAGACT	GTGATGGGGC	AGCCC-GCTC	CGAAGACCCC	3366
Phascolarctos_cinereus	NNNNNNNNAG	ANATNGCTGA	NCNCANCCCG	AANNGNNNCT	NNNNTGNNNN	TCNNCAGNNN	CTANAACCCN	3265
Estrildidae	----------	----------	----------	----------	----------	----------	----------	-
Xenopus_Silurana_tropicalis	----------	----------	----------	----------	----------	----------	----------	-
I_XT	NATGGGTCNT	NNTTNTNTTA	ACACGGTAAN	ANTATACAGG	TTATNCCATT	NAGTATCNAA	ACNACAATGT	3398
Takifugu_rubripes	----------	----------	----------	----------	----------	----------	----------	-
Bos_taurus	----------	----------	----------	----------	----------	----------	----------	-
Homo_sapiens	----------	----------	----------	----------	----------	----------	----------	-
Microcebus_murinus	----------	----------	----------	----------	----------	----------	----------	-
Myotis_lucifugus	----------	----------	----------	----------	----------	----------	----------	-
Equus_caballus	----------	----------	----------	----------	----------	----------	----------	-
Tupaia_belangeri	----------	----------	----------	----------	----------	----------	----------	-
Loxodonta_africana	----------	----------	----------	----------	----------	----------	----------	-
Murinae	----------	----------	----------	----------	----------	----------	----------	-
Canis_lupus_familiaris	----------	----------	----------	----------	----------	----------	----------	-
Sus_scrofa	----------	----------	----------	----------	----------	----------	----------	-
Choloepus_hoffmanni	----------	----------	----------	----------	----------	----------	----------	-
Vicugna_pacos	----------	----------	----------	----------	----------	----------	----------	-
Gavialis_gangeticus	----------	----------	----------	----------	----------	----------	----------	-
Mammalia	----------	----------	----------	----------	----------	----------	----------	-
Passeroidea	----------	----------	----------	----------	----------	----------	----------	-
Takifugu	----------	----------	----------	----------	----------	----------	----------	-
Pteropus_vampyrus	----------	----------	----------	----------	----------	----------	----------	-
Ornithorhynchus	----------	----------	----------	----------	----------	----------	----------	-
Oryctolagus_cuniculus	----------	----------	----------	----------	----------	----------	----------	-
Gallus_gallus	----------	----------	----------	----------	----------	----------	----------	-
Hominidae	AAGTCTCCCC	TCCGTCCTGT	AGGCCCCGGG	AACCTCTGTC	CGTAATCGCG	GCCACGTCAG	AGTCTTCCCC	185
Metatheria	----------	----------	----------	----------	----------	----------	----------	-
Rodentia	----------	----------	----------	----------	----------	----------	----------	-
Monodelphis	----------	----------	----------	----------	----------	----------	----------	-
Consensus	----------	----------	----------	----------	----------	----------	----------	

Conservation

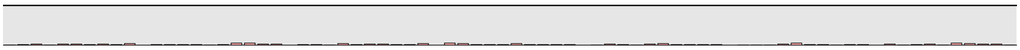

	3,940		3,960		3,980			
Dipodomys_ordii	----------	----------	----------	----------	----------	----------	----------	-
Laurasiatheria	----------	----------	----------	----------	----------	----------	----------	-
Eutheria	----------	----------	----------	----------	----------	----------	----------	-
Tarsius_syrichta	----------	----------	----------	----------	----------	----------	----------	-
Muridae	----------	----------	----------	----------	----------	----------	----------	-
Gasterosteus_aculeatus	----------	----------	----------	----------	----------	----------	----------	-
Felis_silvestris	----------	----------	----------	----------	----------	----------	----------	-
Rattus	----------	----------	----------	----------	----------	----------	----------	-
Papio_hamadryas	ATTCTCCTCA	CCCACCAGCC	TACCTGGGAT	GATTGTCAAC	AGCTTTTGCA	GGTCCTTCTA	ACCACCGAAG	1303
Pan_troglodytes	----------	----------	----------	----------	----------	----------	----------	-
Ailuropoda_melanoleuca	----------	----------	----------	----------	----------	----------	----------	-
Cavia_porcellus	----------	----------	----------	----------	----------	----------	----------	-
Ictidomys_tridecemlineatus	----------	----------	----------	----------	----------	----------	----------	-
Canis	----------	----------	----------	----------	----------	----------	----------	-
Felis_catus	----------	----------	----------	----------	----------	----------	----------	-
Ochotona_princeps	----------	----------	----------	----------	----------	----------	----------	-
Cercopithecidae	----------	----------	----------	----------	----------	----------	----------	-
Carnivora	----------	----------	----------	----------	----------	----------	----------	-
Mus	----------	----------	----------	----------	----------	----------	----------	-
Mus_musculus	----------	----------	----------	----------	----------	----------	----------	-
Rattus_norvegicus	----------	----------	----------	----------	----------	----------	----------	-
Tursiops_truncatus	----------	----------	----------	----------	----------	----------	----------	-
Procavia_capensis	----------	----------	----------	----------	----------	----------	----------	-
Primates	----------	----------	----------	----------	----------	----------	----------	-
Echinops_telfairi	----------	----------	----------	----------	----------	----------	----------	-
Sorex_araneus	----------	----------	----------	----------	----------	----------	----------	-
Canidae	----------	----------	----------	----------	----------	----------	----------	-
Otolemur_garnettii	----------	----------	----------	----------	----------	----------	----------	-
Gallus	----------	----------	----------	----------	----------	----------	----------	-
Callithrix_jacchus	----------	----------	----------	----------	----------	----------	----------	-
Crocodylus_porosus	----------	----------	----------	----------	----------	----------	----------	-
Erinaceus_europaeus	----------	----------	----------	----------	----------	----------	----------	-
rnd-6_family-7426-LTR-ERV1	----------	----------	----------	----------	----------	----------	----------	-
Esox_lucius	----------	----------	----------	----------	----------	----------	----------	-
Salmo_salar	----------	----------	----------	----------	----------	----------	----------	-
Didelphidae	----------	----------	----------	----------	----------	----------	----------	-
Latimeria_chalumnae	----------	----------	----------	----------	----------	----------	----------	-
Monodelphis_domestica	----------	----------	----------	----------	----------	----------	----------	-
Macropus_eugenii	----------	----------	----------	----------	----------	----------	----------	-
Alligator_mississippiensis	----------	----------	----------	----------	----------	----------	----------	-
Chrysemys_picta_bellii	----------	----------	----------	----------	----------	----------	----------	-
Danio_rerio	----------	----------	----------	----------	----------	----------	----------	-
Taeniopygia	----------	----------	----------	----------	----------	----------	----------	-
Dasypus_novemcinctus	----------	----------	----------	----------	----------	----------	----------	-
LTR_XT	----------	----------	----------	----------	----------	----------	----------	-
Haplorrhini	GCGGCAAGTG	CGGGAATTCC	TAGGGGCAGC	TGGTTTCTGC	CGTATATGGA	TCCCCAACTA	CTCGCTCCTA	3959
Catarrhini	AAGGCAAGTA	AGGGAATTCC	TAGGAGCTGT	GGGGTTTTGC	AGATTATGGA	TTCCAAACTT	TGCAGTACTA	3467
Anolis_carolinensis	----------	AAGGAATTCT	TC--AGTTTT	TGCGTTTAGT	TTTTTTTGTA	CTTTA----T	GAAGGTACAA	54
Murine_leukemia_virus	TCGACAACTA	AGGGAGTTCC	TAGGGACGGC	AGGCTTCTGC	CGCCTCTGGA	TCCCTGGGTT	TGCGGAAATG	3436
Phascolarctos_cinereus	CNNNNNAGTC	CGTGAGTTTT	TNNNCACTGC	TNNNTNNNNN	ANGNTCTNGN	TCCCTGGTTT	CGCTTCCNTN	3335
Estrildidae	----------	----------	----------	----------	----------	----------	----------	-
Xenopus_Silurana_tropicalis	----------	----------	----------	----------	----------	----------	----------	-
I_XT	TACANATAAA	NTCAGNACAT	TCCTCGGCAC	AGTGGGNAGT	TGTAGAGCAG	TNAGTTTTAN	AAAATGAAAA	3468
Takifugu_rubripes	----------	----------	----------	----------	----------	----------	----------	-
Bos_taurus	----------	----------	----------	----------	----------	----------	----------	-
Homo_sapiens	----------	----------	----------	----------	----------	----------	----------	-
Microcebus_murinus	----------	----------	----------	----------	----------	----------	----------	-
Myotis_lucifugus	----------	----------	----------	----------	----------	----------	----------	-
Equus_caballus	----------	----------	----------	----------	----------	----------	----------	-
Tupaia_belangeri	----------	----------	----------	----------	----------	----------	----------	-
Loxodonta_africana	----------	----------	----------	----------	----------	----------	----------	-
Murinae	----------	----------	----------	----------	----------	----------	----------	-
Canis_lupus_familiaris	----------	----------	----------	----------	----------	----------	----------	-
Sus_scrofa	----------	----------	----------	----------	----------	----------	----------	-
Choloepus_hoffmanni	----------	----------	----------	----------	----------	----------	----------	-
Vicugna_pacos	----------	----------	----------	----------	----------	----------	----------	-
Gavialis_gangeticus	----------	----------	----------	----------	----------	----------	----------	-
Mammalia	----------	----------	----------	----------	----------	----------	----------	-
Passeroidea	----------	----------	----------	----------	----------	----------	----------	-
Takifugu	----------	----------	----------	----------	----------	----------	----------	-
Pteropus_vampyrus	----------	----------	----------	----------	----------	----------	----------	-
Ornithorhynchus	----------	----------	----------	----------	----------	----------	----------	-
Oryctolagus_cuniculus	----------	----------	----------	----------	----------	----------	----------	-
Gallus_gallus	----------	----------	----------	----------	----------	----------	----------	-
Hominidae	CCATCGGTTT	CCGACTACGG	GACCGAGGAC	GCGGAGACGT	CCGTCCTCCT	CGGCCTCCGC	CATCCGCGCT	255
Metatheria	----------	----------	----------	----------	----------	----------	----------	-
Rodentia	----------	----------	----------	----------	----------	----------	----------	-
Monodelphis	----------	----------	----------	----------	----------	----------	----------	-
Consensus	----------	----------	----------	----------	----------	----------	----------	

Conservation

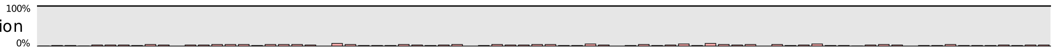

	4,000		4,020		4,040		4,060	
Dipodomys_ordii	----------	----------	----------	----------	----------	----------	----------	-
Laurasiatheria	----------	----------	----------	----------	----------	----------	----------	-
Eutheria	----------	----------	----------	----------	----------	----------	----------	-
Tarsius_syrichta	----------	----------	----------	----------	----------	----------	----------	-
Muridae	----------	----------	----------	----------	----------	----------	----------	-
Gasterosteus_aculeatus	----------	----------	----------	----------	----------	----------	----------	-
Felis_silvestris	----------	----------	----------	----------	----------	----------	----------	-
Rattus	----------	----------	----------	----------	----------	----------	----------	-
Papio_hamadryas	AAAGGCAGCG	AGTCCTCCTG	GAAGCCCGGA	AAAATGTGCC	GGGGCCTGGA	GGCCTTCCAA	CCCAGCTCCC	1373
Pan_troglodytes	----------	----------	----------	----------	----------	----------	----------	-
Ailuropoda_melanoleuca	----------	----------	----------	----------	----------	----------	----------	-
Cavia_porcellus	----------	----------	----------	----------	----------	----------	----------	-
Ictidomys_tridecemlineatus	----------	----------	----------	----------	----------	----------	----------	-
Canis	----------	----------	----------	----------	----------	----------	----------	-
Felis_catus	----------	----------	----------	----------	----------	----------	----------	-
Ochotona_princeps	----------	----------	----------	----------	----------	----------	----------	-
Cercopithecidae	----------	----------	----------	----------	----------	----------	----------	-
Carnivora	----------	----------	----------	----------	----------	----------	----------	-
Mus	----------	----------	----------	----------	----------	----------	----------	-
Mus_musculus	----------	----------	----------	----------	----------	----------	----------	-
Rattus_norvegicus	----------	----------	----------	----------	----------	----------	----------	-
Tursiops_truncatus	----------	----------	----------	----------	----------	----------	----------	-
Procavia_capensis	----------	----------	----------	----------	----------	----------	----------	-
Primates	----------	----------	----------	----------	----------	----------	----------	-
Echinops_telfairi	----------	----------	----------	----------	----------	----------	----------	-
Sorex_araneus	----------	----------	----------	----------	----------	----------	----------	-
Canidae	----------	----------	----------	----------	----------	----------	----------	-
Otolemur_garnettii	----------	----------	----------	----------	----------	----------	----------	-
Gallus	----------	----------	----------	----------	----------	----------	----------	-
Callithrix_jacchus	----------	----------	----------	----------	----------	----------	----------	-
Crocodylus_porosus	----------	----------	----------	----------	----------	----------	----------	-
Erinaceus_europaeus	----------	----------	----------	----------	----------	----------	----------	-
rnd-6_family-7426-LTR-ERV1	----------	----------	----------	----------	----------	----------	----------	-
Esox_lucius	----------	----------	----------	----------	----------	----------	----------	-
Salmo_salar	----------	----------	----------	----------	----------	----------	----------	-
Didelphidae	----------	----------	----------	----------	----------	----------	----------	-
Latimeria_chalumnae	----------	----------	----------	----------	----------	----------	----------	-
Monodelphis_domestica	----------	----------	----------	----------	----------	----------	----------	-
Macropus_eugenii	----------	----------	----------	----------	----------	----------	----------	-
Alligator_mississippiensis	----------	----------	----------	----------	----------	----------	----------	-
Chrysemys_picta_bellii	----------	----------	----------	----------	----------	----------	----------	-
Danio_rerio	----------	----------	----------	----------	----------	----------	----------	-
Taeniopygia	----------	----------	----------	----------	----------	----------	----------	-
Dasypus_novemcinctus	----------	----------	----------	----------	----------	----------	----------	-
LTR_XT	----------	----------	----------	----------	----------	----------	----------	-
Haplorrhini	GCAAAGCCTT	TATATGAGGC	GACCAAAGNG	GGGGAAAAGG	AGCCCCTCCT	TTGGGGAAAA	GAACAGGACA	4029
Catarrhini	GCCAAACCTT	TGTACGGGGT	TACAAAGGGG	GGCGACCGGG	AGCCTTTTGA	ATGGGGGCCT	CTACAACAGC	3537
Anolis_carolinensis	GTTAAAAGCT	CAGTTTTGGT	TTCTTCGGTG	GGAGAAAATT	GCTCCCCACG	GCATGGTTTG	GGGCGGTTGG	124
Murine_leukemia_virus	GCGGCCCCCT	TGTACCCTCT	CACCAAAACG	GG------GA	CTCTGTTTAA	TTGGGGCCCA	GACCAGCAGA	3500
Phascolarctos_cinereus	NCNGCNCNNN	TNTNCNNNNN	NANGAGANNA	AA------GN	NTCCCTTTAC	CTGGACTGAG	GCNCNCCAGN	3399
Estrildidae	----------	----------	----------	----------	----------	----------	----------	-
Xenopus_Silurana_tropicalis	----------	----------	----------	----------	----------	----------	----------	-
I_XT	CAANTNAAAA	CACCNCTTAT	GNCATNTATA	NTNANAGTTC	TGACATAGTA	ATNTCACNGA	CGAACNAANN	3538
Takifugu_rubripes	----------	----------	----------	----------	----------	----------	----------	-
Bos_taurus	----------	----------	----------	----------	----------	----------	----------	-
Homo_sapiens	----------	----------	----------	----------	----------	----------	----------	-
Microcebus_murinus	----------	----------	----------	----------	----------	----------	----------	-
Myotis_lucifugus	----------	----------	----------	----------	----------	----------	----------	-
Equus_caballus	----------	----------	----------	----------	----------	----------	----------	-
Tupaia_belangeri	----------	----------	----------	----------	----------	----------	----------	-
Loxodonta_africana	----------	----------	----------	----------	----------	----------	----------	-
Murinae	----------	----------	----------	----------	----------	----------	----------	-
Canis_lupus_familiaris	----------	----------	----------	----------	----------	----------	----------	-
Sus_scrofa	----------	----------	----------	----------	----------	----------	----------	-
Choloepus_hoffmanni	----------	----------	----------	----------	----------	----------	----------	-
Vicugna_pacos	----------	----------	----------	----------	----------	----------	----------	-
Gavialis_gangeticus	----------	----------	----------	----------	----------	----------	----------	-
Mammalia	----------	----------	----------	----------	----------	----------	----------	-
Passeroidea	----------	----------	----------	----------	----------	----------	----------	-
Takifugu	----------	----------	----------	----------	----------	----------	----------	-
Pteropus_vampyrus	----------	----------	----------	----------	----------	----------	----------	-
Ornithorhynchus	----------	----------	----------	----------	----------	----------	----------	-
Oryctolagus_cuniculus	----------	----------	----------	----------	----------	----------	----------	-
Gallus_gallus	----------	----------	----------	----------	----------	----------	----------	-
Hominidae	TCAAGGAAGG	TTGGGGGACG	CCCCTCCCTG	ACCTTGAATC	TCCCGCCTCA	GGACAATGGG	AGGTGCCCAA	325
Metatheria	----------	----------	----------	----------	----------	----------	----------	-
Rodentia	----------	----------	----------	----------	----------	----------	----------	-
Monodelphis	----------	----------	----------	----------	----------	----------	----------	-
Consensus	----------	----------	----------	----------	----------	----------	----------	

Conservation

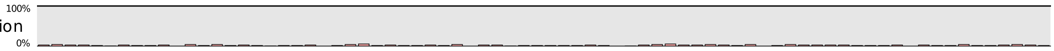

	4,080			4,100		4,120		
Dipodomys_ordii	----------	----------	----------	----------	----------	----------	----------	-
Laurasiatheria	----------	----------	----------	----------	----------	----------	----------	-
Eutheria	----------	----------	----------	----------	----------	----------	----------	-
Tarsius_syrichta	----------	----------	----------	----------	----------	----------	----------	-
Muridae	----------	----------	----------	----------	----------	----------	----------	-
Gasterosteus_aculeatus	----------	----------	----------	----------	----------	----------	----------	-
Felis_silvestris	----------	----------	----------	----------	----------	----------	----------	-
Rattus	----------	----------	----------	----------	----------	----------	----------	-
Papio_hamadryas	CAATGAAATA	GACGAGGGAT	TTCCCCTCAC	CCGCCCGGAC	TGGGATTATG	AGACAGCACC	GGGTAGGGAG	1443
Pan_troglodytes	----------	----------	----------	----------	----------	----------	----------	-
Ailuropoda_melanoleuca	----------	----------	----------	----------	----------	----------	----------	-
Cavia_porcellus	----------	----------	----------	----------	----------	----------	----------	-
Ictidomys_tridecemlineatus	----------	----------	----------	----------	----------	----------	----------	-
Canis	----------	----------	----------	----------	----------	----------	----------	-
Felis_catus	----------	----------	----------	----------	----------	----------	----------	-
Ochotona_princeps	----------	----------	----------	----------	----------	----------	----------	-
Cercopithecidae	----------	----------	----------	----------	----------	----------	----------	-
Carnivora	----------	----------	----------	----------	----------	----------	----------	-
Mus	----------	----------	----------	----------	----------	----------	----------	-
Mus_musculus	----------	----------	----------	----------	----------	----------	----------	-
Rattus_norvegicus	----------	----------	----------	----------	----------	----------	----------	-
Tursiops_truncatus	----------	----------	----------	----------	----------	----------	----------	-
Procavia_capensis	----------	----------	----------	----------	----------	----------	----------	-
Primates	----------	----------	----------	----------	----------	----------	----------	-
Echinops_telfairi	----------	----------	----------	----------	----------	----------	----------	-
Sorex_araneus	----------	----------	----------	----------	----------	----------	----------	-
Canidae	----------	----------	----------	----------	----------	----------	----------	-
Otolemur_garnettii	----------	----------	----------	----------	----------	----------	----------	-
Gallus	----------	----------	----------	----------	----------	----------	----------	-
Callithrix_jacchus	----------	----------	----------	----------	----------	----------	----------	-
Crocodylus_porosus	----------	----------	----------	----------	----------	----------	----------	-
Erinaceus_europaeus	----------	----------	----------	----------	----------	----------	----------	-
rnd-6_family-7426-LTR-ERV1	----------	----------	----------	----------	----------	----------	----------	-
Esox_lucius	----------	----------	----------	----------	----------	----------	----------	-
Salmo_salar	----------	----------	----------	----------	----------	----------	----------	-
Didelphidae	----------	----------	----------	----------	----------	----------	----------	-
Latimeria_chalumnae	----------	----------	----------	----------	----------	----------	----------	-
Monodelphis_domestica	----------	----------	----------	----------	----------	----------	----------	-
Macropus_eugenii	----------	----------	----------	----------	----------	----------	----------	-
Alligator_mississippiensis	----------	----------	----------	----------	----------	----------	----------	-
Chrysemys_picta_bellii	----------	----------	----------	----------	----------	----------	----------	-
Danio_rerio	----------	----------	----------	----------	----------	----------	----------	-
Taeniopygia	----------	----------	----------	----------	----------	----------	----------	-
Dasypus_novemcinctus	----------	----------	----------	----------	----------	----------	----------	-
LTR_XT	----------	----------	----------	----------	----------	----------	----------	-
Haplorrhini	TGGCTT--TC	AAGGAAATCA	AGAAAGCTTT	AATCCAGGCC	CC--AGCACT	AGGGCTGCCA	GACATGACAA	4095
Catarrhini	AAGCCT--TT	TGTAAGTTAA	AGGAAAAATT	TATGTCGGCC	CC--AGCCCT	AGGACTACCA	GATTTGACAA	3603
Anolis_carolinensis	TGGTCCAACT	TGTGCCCCTA	AGGCACAGGG	ATTGGCGAGC	TTTAAAAGCT	TTTCCCGCTT	GGGCCGANTT	194
Murine_leukemia_virus	GGGCCT--AT	CAAGAAATCA	AACAGGCCCT	CCTGACTGCC	CC--CGCCCT	GGGATTGCCA	GACTTGACCA	3566
Phascolarctos_cinereus	NAGCCT--NT	NGNNGNNTNA	NAGAGGCCCT	GCTATCGGCC	CN--CGCCCN	NGNNCTNNNN	NNNCTCANNA	3465
Estrildidae	----------	----------	----------	----------	----------	----------	----------	-
Xenopus_Silurana_tropicalis	----------	----------	----------	----------	----------	----------	----------	-
I_XT	NNTGNAGTNN	ACCCGTNNAA	GATTTGTAGC	TCNANNAACA	NTCTTACATN	AAATTNGACA	TTATANGCAA	3608
Takifugu_rubripes	----------	----------	----------	----------	----------	----------	----------	-
Bos_taurus	----------	----------	----------	----------	----------	----------	----------	-
Homo_sapiens	----------	----------	----------	----------	----------	----------	----------	-
Microcebus_murinus	----------	----------	----------	----------	----------	----------	----------	-
Myotis_lucifugus	----------	----------	----------	----------	----------	----------	----------	-
Equus_caballus	----------	----------	----------	----------	----------	----------	----------	-
Tupaia_belangeri	----------	----------	----------	----------	----------	----------	----------	-
Loxodonta_africana	----------	----------	----------	----------	----------	----------	----------	-
Murinae	----------	----------	----------	----------	----------	----------	----------	-
Canis_lupus_familiaris	----------	----------	----------	----------	----------	----------	----------	-
Sus_scrofa	----------	----------	----------	----------	----------	----------	----------	-
Choloepus_hoffmanni	----------	----------	----------	----------	----------	----------	----------	-
Vicugna_pacos	----------	----------	----------	----------	----------	----------	----------	-
Gavialis_gangeticus	----------	----------	----------	----------	----------	----------	----------	-
Mammalia	----------	----------	----------	----------	----------	----------	----------	-
Passeroidea	----------	----------	----------	----------	----------	----------	----------	-
Takifugu	----------	----------	----------	----------	----------	----------	----------	-
Pteropus_vampyrus	----------	----------	----------	----------	----------	----------	----------	-
Ornithorhynchus	----------	----------	----------	----------	----------	----------	----------	-
Oryctolagus_cuniculus	----------	----------	----------	----------	----------	----------	----------	-
Gallus_gallus	----------	----------	----------	----------	----------	----------	----------	-
Hominidae	TCCAAAACCT	CCCTGACCTT	GAATCGCCCG	CCTCAGGACA	ATGGGAGGTG	CCCAATCCAA	AATTGATCCT	395
Metatheria	----------	----------	----------	----------	----------	----------	----------	-
Rodentia	----------	----------	----------	----------	----------	----------	----------	-
Monodelphis	----------	----------	----------	----------	----------	----------	----------	-
Consensus	----------	----------	----------	----------	----------	----------	----------	

Conservation

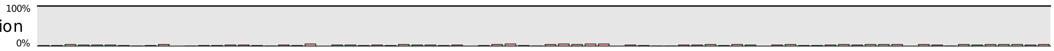

	4,140		4,160		4,180		4,200	
Dipodomys_ordii	----------	----------	----------	----------	----------	----------	----------	-
Laurasiatheria	----------	----------	----------	----------	----------	----------	----------	-
Eutheria	----------	----------	----------	----------	----------	----------	----------	-
Tarsius_syrichta	----------	----------	----------	----------	----------	----------	----------	-
Muridae	----------	----------	----------	----------	----------	----------	----------	-
Gasterosteus_aculeatus	----------	----------	----------	----------	----------	----------	----------	-
Felis_silvestris	----------	----------	----------	----------	----------	----------	----------	-
Rattus	----------	----------	----------	----------	----------	----------	----------	-
Papio_hamadryas	AGTCTCCGAA	TCTATCGCCA	GGCTCTGTTG	GCGGGTCTCA	AGGGGGCAGG	AAAACGCCCC	ACCAATTTGG	1513
Pan_troglodytes	----------	----------	----------	----------	----------	----------	----------	-
Ailuropoda_melanoleuca	----------	----------	----------	----------	----------	----------	----------	-
Cavia_porcellus	----------	----------	----------	----------	----------	----------	----------	-
Ictidomys_tridecemlineatus	----------	----------	----------	----------	----------	----------	----------	-
Canis	----------	----------	----------	----------	----------	----------	----------	-
Felis_catus	----------	----------	----------	----------	----------	----------	----------	-
Ochotona_princeps	----------	----------	----------	----------	----------	----------	----------	-
Cercopithecidae	----------	----------	----------	----------	----------	----------	----------	-
Carnivora	----------	----------	----------	----------	----------	----------	----------	-
Mus	----------	----------	----------	----------	----------	----------	----------	-
Mus_musculus	----------	----------	----------	----------	----------	----------	----------	-
Rattus_norvegicus	----------	----------	----------	----------	----------	----------	----------	-
Tursiops_truncatus	----------	----------	----------	----------	----------	----------	----------	-
Procavia_capensis	----------	----------	----------	----------	----------	----------	----------	-
Primates	----------	----------	----------	----------	----------	----------	----------	-
Echinops_telfairi	----------	----------	----------	----------	----------	----------	----------	-
Sorex_araneus	----------	----------	----------	----------	----------	----------	----------	-
Canidae	----------	----------	----------	----------	----------	----------	----------	-
Otolemur_garnettii	----------	----------	----------	----------	----------	----------	----------	-
Gallus	----------	----------	----------	----------	----------	----------	----------	-
Callithrix_jacchus	----------	----------	----------	----------	----------	----------	----------	-
Crocodylus_porosus	----------	----------	----------	----------	----------	----------	----------	-
Erinaceus_europaeus	----------	----------	----------	----------	----------	----------	----------	-
rnd-6_family-7426-LTR-ERV1	----------	----------	----------	----------	----------	----------	----------	-
Esox_lucius	----------	----------	----------	----------	----------	----------	----------	-
Salmo_salar	----------	----------	----------	----------	----------	----------	----------	-
Didelphidae	----------	----------	----------	----------	----------	----------	----------	-
Latimeria_chalumnae	----------	----------	----------	----------	----------	----------	----------	-
Monodelphis_domestica	----------	----------	----------	----------	----------	----------	----------	-
Macropus_eugenii	----------	----------	----------	----------	----------	----------	----------	-
Alligator_mississippiensis	----------	----------	----------	----------	----------	----------	----------	-
Chrysemys_picta_bellii	----------	----------	----------	----------	----------	----------	----------	-
Danio_rerio	----------	----------	----------	----------	----------	----------	----------	-
Taeniopygia	----------	----------	----------	----------	----------	----------	----------	-
Dasypus_novemcinctus	----------	----------	----------	----------	----------	----------	----------	-
LTR_XT	----------	----------	----------	----------	----------	----------	----------	-
Haplorrhini	AGCC--CTTT	TACCTGTATG	TTCATGAAAG	AAAAGAAATG	GCTACAGGAG	TCTTAGTGCA	AATGCTAGGG	4163
Catarrhini	AGCC--CTTT	ACACTCTATG	TGTCAGAAAG	AGAAAAAATG	GCAGTTGGAG	TTTTAACCCA	GACTGTGGGG	3671
Anolis_carolinensis	GGACAACTTC	TANNTGTATG	TNGGAGAACG	A-ANGGAGTG	GCAGTAGGAG	TATTGACTCA	G---------	254
Murine_leukemia_virus	AGCC--CTTT	GAACTCTTTG	TCGACGAGAA	GCAGGGCTAC	GCCAAAGGCG	TCCTAACGCA	GAAACTGGGA	3634
Phascolarctos_cinereus	ANCC--NNTT	GCTNTNTNCN	TNNNNNNNNN	NGNNNGNGTT	GNNCNNGGGN	NGNTTNCTCA	ANNNCNNNGN	3533
Estrildidae	----------	----------	----------	----------	----------	----------	----------	-
Xenopus_Silurana_tropicalis	----------	----------	----------	----------	----------	----------	----------	-
I_XT	ANTATGANTT	TGCGTTNTGT	NCANGAGAAG	GGTAAGCGAT	TGTCTNNAGT	CCNNGCACAN	AAGAAAGGNG	3678
Takifugu_rubripes	----------	----------	----------	----------	----------	----------	----------	-
Bos_taurus	----------	----------	----------	----------	----------	----------	----------	-
Homo_sapiens	----------	----------	----------	----------	----------	----------	----------	-
Microcebus_murinus	----------	----------	----------	----------	----------	----------	----------	-
Myotis_lucifugus	----------	----------	----------	----------	----------	----------	----------	-
Equus_caballus	----------	----------	----------	----------	----------	----------	----------	-
Tupaia_belangeri	----------	----------	----------	----------	----------	----------	----------	-
Loxodonta_africana	----------	----------	----------	----------	----------	----------	----------	-
Murinae	----------	----------	----------	----------	----------	----------	----------	-
Canis_lupus_familiaris	----------	----------	----------	----------	----------	----------	----------	-
Sus_scrofa	----------	----------	----------	----------	----------	----------	----------	-
Choloepus_hoffmanni	----------	----------	----------	----------	----------	----------	----------	-
Vicugna_pacos	----------	----------	----------	----------	----------	----------	----------	-
Gavialis_gangeticus	----------	----------	----------	----------	----------	----------	----------	-
Mammalia	----------	----------	----------	----------	----------	----------	----------	-
Passeroidea	----------	----------	----------	----------	----------	----------	----------	-
Takifugu	----------	----------	----------	----------	----------	----------	----------	-
Pteropus_vampyrus	----------	----------	----------	----------	----------	----------	----------	-
Ornithorhynchus	----------	----------	----------	----------	----------	----------	----------	-
Oryctolagus_cuniculus	----------	----------	----------	----------	----------	----------	----------	-
Gallus_gallus	----------	----------	----------	----------	----------	----------	----------	-
Hominidae	AAGACACCCC	TGGGGTGTCT	CCTAGCCAAC	TTTGAAGCTC	TAGGCCTCAG	TATGGACCTT	AAGCGGAAGC	465
Metatheria	----------	----------	----------	----------	----------	----------	----------	-
Rodentia	----------	----------	----------	----------	----------	----------	----------	-
Monodelphis	----------	----------	----------	----------	----------	----------	----------	-
Consensus	----------	----------	----------	----------	----------	----------	----------	

Conservation (100% / 0%)

		4,220		4,240		4,260		
Dipodomys_ordii	----------	----------	----------	----------	----------	----------	----------	-
Laurasiatheria	----------	----------	----------	----------	----------	----------	----------	-
Eutheria	----------	----------	----------	----------	----------	----------	----------	-
Tarsius_syrichta	----------	----------	----------	----------	----------	----------	----------	-
Muridae	----------	----------	----------	----------	----------	----------	----------	-
Gasterosteus_aculeatus	----------	----------	----------	----------	----------	----------	----------	-
Felis_silvestris	----------	----------	----------	----------	----------	----------	----------	-
Rattus	----------	----------	----------	----------	----------	----------	----------	-
Papio_hamadryas	CCAAGGTAAG	GACTATAACT	CAGGGAAAAG	ATGAAAGCCC	GGCAGCCTTT	ATGGAAAGAC	TTCTGGAAGG	1583
Pan_troglodytes	----------	----------	----------	----------	----------	----------	----------	-
Ailuropoda_melanoleuca	----------	----------	----------	----------	----------	----------	----------	-
Cavia_porcellus	----------	----------	----------	----------	----------	----------	----------	-
Ictidomys_tridecemlineatus	----------	----------	----------	----------	----------	----------	----------	-
Canis	----------	----------	----------	----------	----------	----------	----------	-
Felis_catus	----------	----------	----------	----------	----------	----------	----------	-
Ochotona_princeps	----------	----------	----------	----------	----------	----------	----------	-
Cercopithecidae	----------	----------	----------	----------	----------	----------	----------	-
Carnivora	----------	----------	----------	----------	----------	----------	----------	-
Mus	----------	----------	----------	----------	----------	----------	----------	-
Mus_musculus	----------	----------	----------	----------	----------	----------	----------	-
Rattus_norvegicus	----------	----------	----------	----------	----------	----------	----------	-
Tursiops_truncatus	----------	----------	----------	----------	----------	----------	----------	-
Procavia_capensis	----------	----------	----------	----------	----------	----------	----------	-
Primates	----------	----------	----------	----------	----------	----------	----------	-
Echinops_telfairi	----------	----------	----------	----------	----------	----------	----------	-
Sorex_araneus	----------	----------	----------	----------	----------	----------	----------	-
Canidae	----------	----------	----------	----------	----------	----------	----------	-
Otolemur_garnettii	----------	----------	----------	----------	----------	----------	----------	-
Gallus	----------	----------	----------	----------	----------	----------	----------	-
Callithrix_jacchus	----------	----------	----------	----------	----------	----------	----------	-
Crocodylus_porosus	----------	----------	----------	----------	----------	----------	----------	-
Erinaceus_europaeus	----------	----------	----------	----------	----------	----------	----------	-
rnd-6_family-7426-LTR-ERV1	----------	----------	----------	----------	----------	----------	----------	-
Esox_lucius	----------	----------	----------	----------	----------	----------	----------	-
Salmo_salar	----------	----------	----------	----------	----------	----------	----------	-
Didelphidae	----------	----------	----------	----------	----------	----------	----------	-
Latimeria_chalumnae	----------	----------	----------	----------	----------	----------	----------	-
Monodelphis_domestica	----------	----------	----------	----------	----------	----------	----------	-
Macropus_eugenii	----------	----------	----------	----------	----------	----------	----------	-
Alligator_mississippiensis	----------	----------	----------	----------	----------	----------	----------	-
Chrysemys_picta_bellii	----------	----------	----------	----------	----------	----------	----------	-
Danio_rerio	----------	----------	----------	----------	----------	----------	----------	-
Taeniopygia	----------	----------	----------	----------	----------	----------	----------	-
Dasypus_novemcinctus	----------	----------	----------	----------	----------	----------	----------	-
LTR_XT	----------	----------	----------	----------	----------	----------	----------	-
Haplorrhini	TCATGGTATC	GGCCTGTAGC	ATATTTGTCC	AAGCAACTGG	ACTTGGTGGC	TACGGGATGG	CCACCCTGTT	4233
Catarrhini	CCCTGGCCAA	GGCCAGTGGC	CTATCTCTCA	AAACAACTAG	ATGGGGTTTC	CAAAGGCTGG	CCACCATGTC	3741
Anolis_carolinensis	--CTAGGTGC	AGCCAGT-GC	TTACTTGTCT	AAGCAANTGG	ACAACGTGGC	CCGTGGATGG	CC-CCNTGCN	320
Murine_leukemia_virus	CCTTGGCGCC	GGCCTGTGGC	CTACCTGTCC	AAAAAGCTGG	ACCCAGTGGC	AGCCGGGTGG	CCCCCCTGCC	3704
Phascolarctos_cinereus	NCNNNNCGAN	NNNNGGNNNC	TTNCNTGTCA	AAAAAATTAG	ATCCGGTGGC	CAGTGGGTGG	CCAACTTGCC	3603
Estrildidae	----------	----------	----------	----------	----------	----------	----------	-
Xenopus_Silurana_tropicalis	----------	----------	----------	----------	----------	----------	----------	-
I_XT	ACAATCCCAN	NACNGTNGGA	TATTCCATNG	CGGAGTTAGN	NGCTATTGCN	GCAGCCCTAA	CTGGNTTGAG	3748
Takifugu_rubripes	----------	----------	----------	----------	----------	----------	----------	-
Bos_taurus	----------	----------	----------	----------	----------	----------	----------	-
Homo_sapiens	----------	----------	----------	----------	----------	----------	----------	-
Microcebus_murinus	----------	----------	----------	----------	----------	----------	----------	-
Myotis_lucifugus	----------	----------	----------	----------	----------	----------	----------	-
Equus_caballus	----------	----------	----------	----------	----------	----------	----------	-
Tupaia_belangeri	----------	----------	----------	----------	----------	----------	----------	-
Loxodonta_africana	----------	----------	----------	----------	----------	----------	----------	-
Murinae	----------	----------	----------	----------	----------	----------	----------	-
Canis_lupus_familiaris	----------	----------	----------	----------	----------	----------	----------	-
Sus_scrofa	----------	----------	----------	----------	----------	----------	----------	-
Choloepus_hoffmanni	----------	----------	----------	----------	----------	----------	----------	-
Vicugna_pacos	----------	----------	----------	----------	----------	----------	----------	-
Gavialis_gangeticus	----------	----------	----------	----------	----------	----------	----------	-
Mammalia	----------	----------	----------	----------	----------	----------	----------	-
Passeroidea	----------	----------	----------	----------	----------	----------	----------	-
Takifugu	----------	----------	----------	----------	----------	----------	----------	-
Pteropus_vampyrus	----------	----------	----------	----------	----------	----------	----------	-
Ornithorhynchus	----------	----------	----------	----------	----------	----------	----------	-
Oryctolagus_cuniculus	----------	----------	----------	----------	----------	----------	----------	-
Gallus_gallus	----------	----------	----------	----------	----------	----------	----------	-
Hominidae	GACTCATTTT	CTTTTGCTCG	GTCGCTTGGC	CGCAATACAA	ATTGGACAAC	CAATCTCGGT	GGCCGCCCGA	535
Metatheria	----------	----------	----------	----------	----------	----------	----------	-
Rodentia	----------	----------	----------	----------	----------	----------	----------	-
Monodelphis	----------	----------	----------	----------	----------	----------	----------	-
Consensus	----------	----------	----------	----------	----------	----------	----------	

Conservation

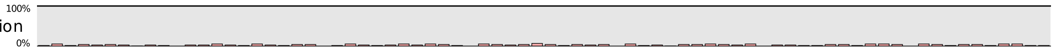

4,280 | 4,300 | 4,320 | 4,340

Dipodomys_ordii	----------	----------	----------	----------	----------	----------	----------	-
Laurasiatheria	----------	----------	----------	----------	----------	----------	----------	-
Eutheria	----------	----------	----------	----------	----------	----------	----------	-
Tarsius_syrichta	----------	----------	----------	----------	----------	----------	----------	-
Muridae	----------	----------	----------	----------	----------	----------	----------	-
Gasterosteus_aculeatus	----------	----------	----------	----------	----------	----------	----------	-
Felis_silvestris	----------	----------	----------	----------	----------	----------	----------	-
Rattus	----------	----------	----------	----------	----------	----------	----------	-
Papio_hamadryas	GTTTCGAATG	TATACTCCAT	TTGATCCAGA	AGCACCAGAA	CACAAGGCTA	CCGTGGCCAT	GTCGTTCATA	1653
Pan_troglodytes	----------	----------	----------	----------	----------	----------	----------	-
Ailuropoda_melanoleuca	----------	----------	----------	----------	----------	----------	----------	-
Cavia_porcellus	----------	----------	----------	----------	----------	----------	----------	-
Ictidomys_tridecemlineatus	----------	----------	----------	----------	----------	----------	----------	-
Canis	----------	----------	----------	----------	----------	----------	----------	-
Felis_catus	----------	----------	----------	----------	----------	----------	----------	-
Ochotona_princeps	----------	----------	----------	----------	----------	----------	----------	-
Cercopithecidae	----------	----------	----------	----------	----------	----------	----------	-
Carnivora	----------	----------	----------	----------	----------	----------	----------	-
Mus	----------	----------	----------	----------	----------	----------	----------	-
Mus_musculus	----------	----------	----------	----------	----------	----------	----------	-
Rattus_norvegicus	----------	----------	----------	----------	----------	----------	----------	-
Tursiops_truncatus	----------	----------	----------	----------	----------	----------	----------	-
Procavia_capensis	----------	----------	----------	----------	----------	----------	----------	-
Primates	----------	----------	----------	----------	----------	----------	----------	-
Echinops_telfairi	----------	----------	----------	----------	----------	----------	----------	-
Sorex_araneus	----------	----------	----------	----------	----------	----------	----------	-
Canidae	----------	----------	----------	----------	----------	----------	----------	-
Otolemur_garnettii	----------	----------	----------	----------	----------	----------	----------	-
Gallus	----------	----------	----------	----------	----------	----------	----------	-
Callithrix_jacchus	----------	----------	----------	----------	----------	----------	----------	-
Crocodylus_porosus	----------	----------	----------	----------	----------	----------	----------	-
Erinaceus_europaeus	----------	----------	----------	----------	----------	----------	----------	-
rnd-6_family-7426-LTR-ERV1	----------	----------	----------	----------	----------	----------	----------	-
Esox_lucius	----------	----------	----------	----------	----------	----------	----------	-
Salmo_salar	----------	----------	----------	----------	----------	----------	----------	-
Didelphidae	----------	----------	----------	----------	----------	----------	----------	-
Latimeria_chalumnae	----------	----------	----------	----------	----------	----------	----------	-
Monodelphis_domestica	----------	----------	----------	----------	----------	----------	----------	-
Macropus_eugenii	----------	----------	----------	----------	----------	----------	----------	-
Alligator_mississippiensis	----------	----------	----------	----------	----------	----------	----------	-
Chrysemys_picta_bellii	----------	----------	----------	----------	----------	----------	----------	-
Danio_rerio	----------	----------	----------	----------	----------	----------	----------	-
Taeniopygia	----------	----------	----------	----------	----------	----------	----------	-
Dasypus_novemcinctus	----------	----------	----------	----------	----------	----------	----------	-
LTR_XT	----------	----------	----------	----------	----------	----------	----------	-
Haplorrhini	TCAAGGCGCT	GGCCGCCACT	GCCTTGTTAG	CCGAGGATGC	TAACAAGCTC	ACATTTGGAC	AGAAGCTGAT	4303
Catarrhini	TAAGGGCCCT	GGCAGCAACA	GCCCTGTTAG	CACAAGAAGC	AGATAAACTA	ACCCTTGGGC	AAAACCTGAA	3811
Anolis_carolinensis	TGAGAGCTGT	GGCTGCT-CT	GCTGTTCTTG	T-GAAGANGC	AAANAA--TG	ACCTTAGGAC	AGC-CGTGAT	385
Murine_leukemia_virus	TACGGATGGT	AGCAGCCATT	GCCGTTCTGA	CAAAGGATGC	AGGCAAGCTA	ACTATGGGAC	AGCCGCTAGT	3774
Phascolarctos_cinereus	TAAAAGCAAT	TGCGGCCGTA	GCACTCCTTC	TCAAGGACGC	TGATAAGTTA	ACCCTAGGAC	AGAACGTGTT	3673
Estrildidae	----------	----------	----------	----------	----------	----------	----------	-
Xenopus_Silurana_tropicalis	----------	----------	----------	----------	----------	----------	----------	-
I_XT	AGAGTAGTAN	CAGCAGCAGC	CATAACTGTN	GAGNCTACTN	NNGANNCAGT	NNNACAATAT	AACCTNTTTG	3818
Takifugu_rubripes	----------	----------	----------	----------	----------	----------	----------	-
Bos_taurus	----------	----------	----------	----------	----------	----------	----------	-
Homo_sapiens	----------	----------	----------	----------	----------	----------	----------	-
Microcebus_murinus	----------	----------	----------	----------	----------	----------	----------	-
Myotis_lucifugus	----------	----------	----------	----------	----------	----------	----------	-
Equus_caballus	----------	----------	----------	----------	----------	----------	----------	-
Tupaia_belangeri	----------	----------	----------	----------	----------	----------	----------	-
Loxodonta_africana	----------	----------	----------	----------	----------	----------	----------	-
Murinae	----------	----------	----------	----------	----------	----------	----------	-
Canis_lupus_familiaris	----------	----------	----------	----------	----------	----------	----------	-
Sus_scrofa	----------	----------	----------	----------	----------	----------	----------	-
Choloepus_hoffmanni	----------	----------	----------	----------	----------	----------	----------	-
Vicugna_pacos	----------	----------	----------	----------	----------	----------	----------	-
Gavialis_gangeticus	----------	----------	----------	----------	----------	----------	----------	-
Mammalia	----------	----------	----------	----------	----------	----------	----------	-
Passeroidea	----------	----------	----------	----------	----------	----------	----------	-
Takifugu	----------	----------	----------	----------	----------	----------	----------	-
Pteropus_vampyrus	----------	----------	----------	----------	----------	----------	----------	-
Ornithorhynchus	----------	----------	----------	----------	----------	----------	----------	-
Oryctolagus_cuniculus	----------	----------	----------	----------	----------	----------	----------	-
Gallus_gallus	----------	----------	----------	----------	----------	----------	----------	-
Hominidae	AGGAACTTTC	GATTTCCAAA	TTTTACAGGA	CCTAGACAAC	CTTTGCAGAA	GACAAGGCAA	ATGGTCAGAG	605
Metatheria	----------	----------	----------	----------	----------	----------	----------	-
Rodentia	----------	----------	----------	----------	----------	----------	----------	-
Monodelphis	----------	----------	----------	----------	----------	----------	----------	-
Consensus	----------	----------	----------	----------	----------	----------	----------	

Conservation

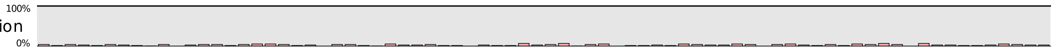

		4,360		4,380		4,400		
Dipodomys_ordii	----------	----------	----------	----------	----------	----------	----------	-
Laurasiatheria	----------	----------	----------	----------	----------	----------	----------	-
Eutheria	----------	----------	----------	----------	----------	----------	----------	-
Tarsius_syrichta	----------	----------	----------	----------	----------	----------	----------	-
Muridae	----------	----------	----------	----------	----------	----------	----------	-
Gasterosteus_aculeatus	----------	----------	----------	----------	----------	----------	----------	-
Felis_silvestris	----------	----------	----------	----------	----------	----------	----------	-
Rattus	----------	----------	----------	----------	----------	----------	----------	-
Papio_hamadryas	GATCAGGCAG	CACTAGACAT	AAAAGGAAAA	CTCCAAAGGC	TAGACGGGAT	CCAAACTCAT	GGGCTGCAGG	1723
Pan_troglodytes	----------	----------	----------	----------	----------	----------	----------	-
Ailuropoda_melanoleuca	----------	----------	----------	----------	----------	----------	----------	-
Cavia_porcellus	----------	----------	----------	----------	----------	----------	----------	-
Ictidomys_tridecemlineatus	----------	----------	----------	----------	----------	----------	----------	-
Canis	----------	----------	----------	----------	----------	----------	----------	-
Felis_catus	----------	----------	----------	----------	----------	----------	----------	-
Ochotona_princeps	----------	----------	----------	----------	----------	----------	----------	-
Cercopithecidae	----------	----------	----------	----------	----------	----------	----------	-
Carnivora	----------	----------	----------	----------	----------	----------	----------	-
Mus	----------	----------	----------	----------	----------	----------	----------	-
Mus_musculus	----------	----------	----------	----------	----------	----------	----------	-
Rattus_norvegicus	----------	----------	----------	----------	----------	----------	----------	-
Tursiops_truncatus	----------	----------	----------	----------	----------	----------	----------	-
Procavia_capensis	----------	----------	----------	----------	----------	----------	----------	-
Primates	----------	----------	----------	----------	----------	----------	----------	-
Echinops_telfairi	----------	----------	----------	----------	----------	----------	----------	-
Sorex_araneus	----------	----------	----------	----------	----------	----------	----------	-
Canidae	----------	----------	----------	----------	----------	----------	----------	-
Otolemur_garnettii	----------	----------	----------	----------	----------	----------	----------	-
Gallus	----------	----------	----------	----------	----------	----------	----------	-
Callithrix_jacchus	----------	----------	----------	----------	----------	----------	----------	-
Crocodylus_porosus	----------	----------	----------	----------	----------	----------	----------	-
Erinaceus_europaeus	----------	----------	----------	----------	----------	----------	----------	-
rnd-6_family-7426-LTR-ERV1	----------	----------	----------	----------	----------	----------	----------	-
Esox_lucius	----------	----------	----------	----------	----------	----------	----------	-
Salmo_salar	----------	----------	----------	----------	----------	----------	----------	-
Didelphidae	----------	----------	----------	----------	----------	----------	----------	-
Latimeria_chalumnae	----------	----------	----------	----------	----------	----------	----------	-
Monodelphis_domestica	----------	----------	----------	----------	----------	----------	----------	-
Macropus_eugenii	----------	----------	----------	----------	----------	----------	----------	-
Alligator_mississippiensis	----------	----------	----------	----------	----------	----------	----------	-
Chrysemys_picta_bellii	----------	----------	----------	----------	----------	----------	----------	-
Danio_rerio	----------	----------	----------	----------	----------	----------	----------	-
Taeniopygia	----------	----------	----------	----------	----------	----------	----------	-
Dasypus_novemcinctus	----------	----------	----------	----------	----------	----------	----------	-
LTR_XT	----------	----------	----------	----------	----------	----------	----------	-
Haplorrhini	AATTCAGGTG	CCCCACACAG	TTGTCACCCT	GATGGAACAG	AGAGGACATC	GTTGGCTCTC	TAACCCTAGG	4373
Catarrhini	TATAAAGGCC	CCCCATGCTG	TGGTAACTTT	GATGAATACC	AAAGGACATC	ATTGGCTAAC	AAATGCTAGA	3881
Anolis_carolinensis	TTTAAAGG--	CCCCATGCGG	TAGTG---CT	TATGGA--AT	AGNGGACACC	ACTGGCTCAC	CAATANNCGT	448
Murine_leukemia_virus	CATCCTGGCC	CCCCATGCGG	TAGAGGCACT	GGTCAAGCAA	CCCCCTGACC	GCTGGCTATC	CAACGCCCGC	3844
Phascolarctos_cinereus	GGTGATTGCA	CCTCACAACC	TGGAAAGCAT	TGTGAGACAG	CCCCCCGACC	GATGGATGAC	AAATGCCAGG	3743
Estrildidae	----------	----------	----------	----------	----------	----------	----------	-
Xenopus_Silurana_tropicalis	----------	----------	----------	----------	----------	----------	----------	-
I_XT	TAGATGGGCA	ANCAAACNNT	ACAANNTNTG	TTGATNNACN	GNTTGACANA	ACATTCCNNT	TTATTCGTGA	3888
Takifugu_rubripes	----------	----------	----------	----------	----------	----------	----------	-
Bos_taurus	----------	----------	----------	----------	----------	----------	----------	-
Homo_sapiens	----------	----------	----------	----------	----------	----------	----------	-
Microcebus_murinus	----------	----------	----------	----------	----------	----------	----------	-
Myotis_lucifugus	----------	----------	----------	----------	----------	----------	----------	-
Equus_caballus	----------	----------	----------	----------	----------	----------	----------	-
Tupaia_belangeri	----------	----------	----------	----------	----------	----------	----------	-
Loxodonta_africana	----------	----------	----------	----------	----------	----------	----------	-
Murinae	----------	----------	----------	----------	----------	----------	----------	-
Canis_lupus_familiaris	----------	----------	----------	----------	----------	----------	----------	-
Sus_scrofa	----------	----------	----------	----------	----------	----------	----------	-
Choloepus_hoffmanni	----------	----------	----------	----------	----------	----------	----------	-
Vicugna_pacos	----------	----------	----------	----------	----------	----------	----------	-
Gavialis_gangeticus	----------	----------	----------	----------	----------	----------	----------	-
Mammalia	----------	----------	----------	----------	----------	----------	----------	-
Passeroidea	----------	----------	----------	----------	----------	----------	----------	-
Takifugu	----------	----------	----------	----------	----------	----------	----------	-
Pteropus_vampyrus	----------	----------	----------	----------	----------	----------	----------	-
Ornithorhynchus	----------	----------	----------	----------	----------	----------	----------	-
Oryctolagus_cuniculus	----------	----------	----------	----------	----------	----------	----------	-
Gallus_gallus	----------	----------	----------	----------	----------	----------	----------	-
Hominidae	GTCCCTTATG	TACAAGCCTT	TTGGGACCTA	CGCTCTCGTC	CTGACCTATG	TGCCAAGTGT	TCCCTTGGAC	675
Metatheria	----------	----------	----------	----------	----------	----------	----------	-
Rodentia	----------	----------	----------	----------	----------	----------	----------	-
Monodelphis	----------	----------	----------	----------	----------	----------	----------	-
Consensus	----------	----------	----------	----------	----------	----------	----------	

Conservation

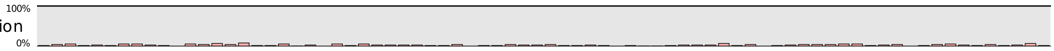

	4,420		4,440		4,460		4,480	
Dipodomys_ordii	----------	----------	----------	----------	----------	----------	----------	-
Laurasiatheria	----------	----------	----------	----------	----------	----------	----------	-
Eutheria	----------	----------	----------	----------	----------	----------	----------	-
Tarsius_syrichta	----------	----------	----------	----------	----------	----------	----------	-
Muridae	----------	----------	----------	----------	----------	----------	----------	-
Gasterosteus_aculeatus	----------	----------	----------	----------	----------	----------	----------	-
Felis_silvestris	----------	----------	----------	----------	----------	----------	----------	-
Rattus	----------	----------	----------	----------	----------	----------	----------	-
Papio_hamadryas	AATTAGTAAG	GGAGGCAGAA	AAGGTATACA	ATAAAAGGGA	AACCCCAGAA	GAAAGAGAAG	CTAGGCTTAT	1793
Pan_troglodytes	----------	----------	----------	----------	----------	----------	----------	-
Ailuropoda_melanoleuca	----------	----------	----------	----------	----------	----------	----------	-
Cavia_porcellus	----------	----------	----------	----------	----------	----------	----------	-
Ictidomys_tridecemlineatus	----------	----------	----------	----------	----------	----------	----------	-
Canis	----------	----------	----------	----------	----------	----------	----------	-
Felis_catus	----------	----------	----------	----------	----------	----------	----------	-
Ochotona_princeps	----------	----------	----------	----------	----------	----------	----------	-
Cercopithecidae	----------	----------	----------	----------	----------	----------	----------	-
Carnivora	----------	----------	----------	----------	----------	----------	----------	-
Mus	----------	----------	----------	----------	----------	----------	----------	-
Mus_musculus	----------	----------	----------	----------	----------	----------	----------	-
Rattus_norvegicus	----------	----------	----------	----------	----------	----------	----------	-
Tursiops_truncatus	----------	----------	----------	----------	----------	----------	----------	-
Procavia_capensis	----------	----------	----------	----------	----------	----------	----------	-
Primates	----------	----------	----------	----------	----------	----------	----------	-
Echinops_telfairi	----------	----------	----------	----------	----------	----------	----------	-
Sorex_araneus	----------	----------	----------	----------	----------	----------	----------	-
Canidae	----------	----------	----------	----------	----------	----------	----------	-
Otolemur_garnettii	----------	----------	----------	----------	----------	----------	----------	-
Gallus	----------	----------	----------	----------	----------	----------	----------	-
Callithrix_jacchus	----------	----------	----------	----------	----------	----------	----------	-
Crocodylus_porosus	----------	----------	----------	----------	----------	----------	----------	-
Erinaceus_europaeus	----------	----------	----------	----------	----------	----------	----------	-
rnd-6_family-7426-LTR-ERV1	----------	----------	----------	----------	----------	----------	----------	-
Esox_lucius	----------	----------	----------	----------	----------	----------	----------	-
Salmo_salar	----------	----------	----------	----------	----------	----------	----------	-
Didelphidae	----------	----------	----------	----------	----------	----------	----------	-
Latimeria_chalumnae	----------	----------	----------	----------	----------	----------	----------	-
Monodelphis_domestica	----------	----------	----------	----------	----------	----------	----------	-
Macropus_eugenii	----------	----------	----------	----------	----------	----------	----------	-
Alligator_mississippiensis	----------	----------	----------	----------	----------	----------	----------	-
Chrysemys_picta_bellii	----------	----------	----------	----------	----------	----------	----------	-
Danio_rerio	----------	----------	----------	----------	----------	----------	----------	-
Taeniopygia	----------	----------	----------	----------	----------	----------	----------	-
Dasypus_novemcinctus	----------	----------	----------	----------	----------	----------	----------	-
LTR_XT	----------	----------	----------	----------	----------	----------	----------	-
Haplorrhini	ATGCTAAGAT	ACCAAGGGCT	CCTATGTGAG	AATCCATACA	TCACC-TTAG	AGACTGTGAA	TACCCTAAAT	4442
Catarrhini	TTAACCAAGT	ACCAAAGCTT	GCTATGTGAA	AATCCCCGCA	TAACTATTGA	AGTCTGT-AA	CACCCTAAAT	3950
Anolis_carolinensis	NTGTTTAAGT	ACGAGGGCTT	GCTGNTTGAC	AATCCACAGG	TCAATCTGCC	ANTTTGN-AC	NACTTTAAAT	517
Murine_leukemia_virus	ATGACCCACT	ACCAGGCAAT	GCTCCTAGAC	ACTGACCGAG	TTCAG-TTCG	GACCGGTGGT	GGCCCTCAAT	3913
Phascolarctos_cinereus	ATGACCCATT	ACCAAAGTCT	GCTGCTGAA-	--TGAGAGGG	TGTCT-TTTG	CGCCCCCGGC	CATTCTGAAT	3809
Estrildidae	----------	----------	----------	----------	----------	----------	----------	-
Xenopus_Silurana_tropicalis	----------	----------	----------	----------	----------	----------	----------	-
I_XT	GAACCNAATA	TTATGTTANG	CNATATATNG	GCAGTTAANT	NCNGTTGTNC	CAGAACTGTN	ACANATTGNA	3958
Takifugu_rubripes	----------	----------	----------	----------	----------	----------	----------	-
Bos_taurus	----------	----------	----------	----------	----------	----------	----------	-
Homo_sapiens	----------	----------	----------	----------	----------	----------	----------	-
Microcebus_murinus	----------	----------	----------	----------	----------	----------	----------	-
Myotis_lucifugus	----------	----------	----------	----------	----------	----------	----------	-
Equus_caballus	----------	----------	----------	----------	----------	----------	----------	-
Tupaia_belangeri	----------	----------	----------	----------	----------	----------	----------	-
Loxodonta_africana	----------	----------	----------	----------	----------	----------	----------	-
Murinae	----------	----------	----------	----------	----------	----------	----------	-
Canis_lupus_familiaris	----------	----------	----------	----------	----------	----------	----------	-
Sus_scrofa	----------	----------	----------	----------	----------	----------	----------	-
Choloepus_hoffmanni	----------	----------	----------	----------	----------	----------	----------	-
Vicugna_pacos	----------	----------	----------	----------	----------	----------	----------	-
Gavialis_gangeticus	----------	----------	----------	----------	----------	----------	----------	-
Mammalia	----------	----------	----------	----------	----------	----------	----------	-
Passeroidea	----------	----------	----------	----------	----------	----------	----------	-
Takifugu	----------	----------	----------	----------	----------	----------	----------	-
Pteropus_vampyrus	----------	----------	----------	----------	----------	----------	----------	-
Ornithorhynchus	----------	----------	----------	----------	----------	----------	----------	-
Oryctolagus_cuniculus	----------	----------	----------	----------	----------	----------	----------	-
Gallus_gallus	----------	----------	----------	----------	----------	----------	----------	-
Hominidae	AGGTGTTACT	GGCTAAGGCA	TCCCCCTCTA	ACAAAGAACC	TGATTCCTCC	CCTCTCTCCG	AGCCTCCTGA	745
Metatheria	----------	----------	----------	----------	----------	----------	----------	-
Rodentia	----------	----------	----------	----------	----------	----------	----------	-
Monodelphis	----------	----------	----------	----------	----------	----------	----------	-
Consensus	----------	----------	----------	----------	----------	----------	----------	

Conservation

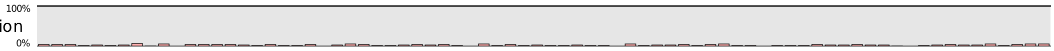

		4,500		4,520		4,540		
Dipodomys_ordii	----------	----------	----------	----------	----------	----------	----------	-
Laurasiatheria	----------	----------	----------	----------	----------	----------	----------	-
Eutheria	----------	----------	----------	----------	----------	----------	----------	-
Tarsius_syrichta	----------	----------	----------	----------	----------	----------	----------	-
Muridae	----------	----------	----------	----------	----------	----------	----------	-
Gasterosteus_aculeatus	----------	----------	----------	----------	----------	----------	----------	-
Felis_silvestris	----------	----------	----------	----------	----------	----------	----------	-
Rattus	----------	----------	----------	----------	----------	----------	----------	-
Papio_hamadryas	AAAAGAACAG	GAAGAACGGG	AAGATCGGAG	AGACAGAAAA	AGAGATAAGC	ATTTAACCAA	AATCCTGGCA	1863
Pan_troglodytes	----------	----------	----------	----------	----------	----------	----------	-
Ailuropoda_melanoleuca	----------	----------	----------	----------	----------	----------	----------	-
Cavia_porcellus	----------	----------	----------	----------	----------	----------	----------	-
Ictidomys_tridecemlineatus	----------	----------	----------	----------	----------	----------	----------	-
Canis	----------	----------	----------	----------	----------	----------	----------	-
Felis_catus	----------	----------	----------	----------	----------	----------	----------	-
Ochotona_princeps	----------	----------	----------	----------	----------	----------	----------	-
Cercopithecidae	----------	----------	----------	----------	----------	----------	----------	-
Carnivora	----------	----------	----------	----------	----------	----------	----------	-
Mus	----------	----------	----------	----------	----------	----------	----------	-
Mus_musculus	----------	----------	----------	----------	----------	----------	----------	-
Rattus_norvegicus	----------	----------	----------	----------	----------	----------	----------	-
Tursiops_truncatus	----------	----------	----------	----------	----------	----------	----------	-
Procavia_capensis	----------	----------	----------	----------	----------	----------	----------	-
Primates	----------	----------	----------	----------	----------	----------	----------	-
Echinops_telfairi	----------	----------	----------	----------	----------	----------	----------	-
Sorex_araneus	----------	----------	----------	----------	----------	----------	----------	-
Canidae	----------	----------	----------	----------	----------	----------	----------	-
Otolemur_garnettii	----------	----------	----------	----------	----------	----------	----------	-
Gallus	----------	----------	----------	----------	----------	----------	----------	-
Callithrix_jacchus	----------	----------	----------	----------	----------	----------	----------	-
Crocodylus_porosus	----------	----------	----------	----------	----------	----------	----------	-
Erinaceus_europaeus	----------	----------	----------	----------	----------	----------	----------	-
rnd-6_family-7426-LTR-ERV1	----------	----------	----------	----------	----------	----------	----------	-
Esox_lucius	----------	----------	----------	----------	----------	----------	----------	-
Salmo_salar	----------	----------	----------	----------	----------	----------	----------	-
Didelphidae	----------	----------	----------	----------	----------	----------	----------	-
Latimeria_chalumnae	----------	----------	----------	----------	----------	----------	----------	-
Monodelphis_domestica	----------	----------	----------	----------	----------	----------	----------	-
Macropus_eugenii	----------	----------	----------	----------	----------	----------	----------	-
Alligator_mississippiensis	----------	----------	----------	----------	----------	----------	----------	-
Chrysemys_picta_bellii	----------	----------	----------	----------	----------	----------	----------	-
Danio_rerio	----------	----------	----------	----------	----------	----------	----------	-
Taeniopygia	----------	----------	----------	----------	----------	----------	----------	-
Dasypus_novemcinctus	----------	----------	----------	----------	----------	----------	----------	-
LTR_XT	----------	----------	----------	----------	----------	----------	----------	-
Haplorrhini	CCGGCCACAC	TGTTACCAAT	AGAATGTGCA	GAACATGGGA	AGCCCCCGTT	ATGTGCCCCA	GGGTACCACT	4512
Catarrhini	CCCGCCACCC	TGCTCCCAGT	A---------	-----TCAGA	GAGCCCGGTC	GAG-------	------CATA	3993
Anolis_carolinensis	CCTGCCACNT	TGTTGCCTGT	NGAA------	-----GAAGN	AGAATNGATN	CNA-------	------CATG	563
Murine_leukemia_virus	CCTGCCACCT	TGCTCCCTCT	ACCG------	-----GAGAA	AGGAGCCCCC	----------	------CATG	3956
Phascolarctos_cinereus	CCTGCCACCC	TGCTCCCCGT	AGAG------	-----TCAGA	CGACACCCCA	ATA-------	------CACA	3855
Estrildidae	----------	----------	----------	----------	----------	----------	----------	-
Xenopus_Silurana_tropicalis	----------	----------	----------	----------	----------	----------	----------	-
I_XT	TNCAGAAAAT	CTANTCACNA	TTGGGNCACA	TAGGANAGAN	AAANCTAANC	AAGATTATGN	ANGACAACAT	4028
Takifugu_rubripes	----------	----------	----------	----------	----------	----------	----------	-
Bos_taurus	----------	----------	----------	----------	----------	----------	----------	-
Homo_sapiens	----------	----------	----------	----------	----------	----------	----------	-
Microcebus_murinus	----------	----------	----------	----------	----------	----------	----------	-
Myotis_lucifugus	----------	----------	----------	----------	----------	----------	----------	-
Equus_caballus	----------	----------	----------	----------	----------	----------	----------	-
Tupaia_belangeri	----------	----------	----------	----------	----------	----------	----------	-
Loxodonta_africana	----------	----------	----------	----------	----------	----------	----------	-
Murinae	----------	----------	----------	----------	----------	----------	----------	-
Canis_lupus_familiaris	----------	----------	----------	----------	----------	----------	----------	-
Sus_scrofa	----------	----------	----------	----------	----------	----------	----------	-
Choloepus_hoffmanni	----------	----------	----------	----------	----------	----------	----------	-
Vicugna_pacos	----------	----------	----------	----------	----------	----------	----------	-
Gavialis_gangeticus	----------	----------	----------	----------	----------	----------	----------	-
Mammalia	----------	----------	----------	----------	----------	----------	----------	-
Passeroidea	----------	----------	----------	----------	----------	----------	----------	-
Takifugu	----------	----------	----------	----------	----------	----------	----------	-
Pteropus_vampyrus	----------	----------	----------	----------	----------	----------	----------	-
Ornithorhynchus	----------	----------	----------	----------	----------	----------	----------	-
Oryctolagus_cuniculus	----------	----------	----------	----------	----------	----------	----------	-
Gallus_gallus	----------	----------	----------	----------	----------	----------	----------	-
Hominidae	AGCCCTCGCT	TTACCACCAT	TGCCAGCGGC	GCTCCCTCCT	CCCTATCCAG	GATCCTCTGG	CCCCACCCCA	815
Metatheria	----------	----------	----------	----------	----------	----------	----------	-
Rodentia	----------	----------	----------	----------	----------	----------	----------	-
Monodelphis	----------	----------	----------	----------	----------	----------	----------	-
Consensus	----------	----------	----------	----------	----------	----------	----------	

Conservation

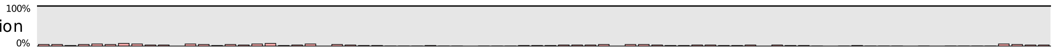

```
                               4,560                 4,580                 4,600                 4,620
Dipodomys_ordii                ----------  ----------  ----------  ----------  ----------  ----------  ----------  -
Laurasiatheria                 ----------  ----------  ----------  ----------  ----------  ----------  ----------  -
Eutheria                       ----------  ----------  ----------  ----------  ----------  ----------  ----------  -
Tarsius_syrichta               ----------  ----------  ----------  ----------  ----------  ----------  ----------  -
Muridae                        ----------  ----------  ----------  ----------  ----------  ----------  ----------  -
Gasterosteus_aculeatus         ----------  ----------  ----------  ----------  ----------  ----------  ----------  -
Felis_silvestris               ----------  ----------  ----------  ----------  ----------  ----------  ----------  -
Rattus                         ----------  ----------  ----------  ----------  ----------  ----------  ----------  -
Papio_hamadryas                GCCGTAGTGA  CTGAAAAAAG  GGCAGGAAAG  TCAGGGGAAA  CAAGAAGGCG  GCCTAAAGTA  GATAAGGACC  1933
Pan_troglodytes                ----------  ----------  ----------  ----------  ---------G  GCTCGTGCCC  CCGACCAGCG  21
Ailuropoda_melanoleuca         ----------  ----------  ----------  ----------  ----------  ----------  ----------  -
Cavia_porcellus                ----------  ----------  ----------  ----------  ----------  ----------  ----------  -
Ictidomys_tridecemlineatus     ----------  ----------  ----------  ----------  ----------  ----------  ----------  -
Canis                          ----------  ----------  ----------  ----------  ----------  ----------  ----------  -
Felis_catus                    ----------  ----------  ----------  ----------  ----------  ----------  ----------  -
Ochotona_princeps              ----------  ----------  ----------  ----------  ----------  ----------  ----------  -
Cercopithecidae                ----------  ----------  ----------  ----------  ----------  ----------  ----------  -
Carnivora                      ----------  ----------  ----------  ----------  ----------  ----------  ----------  -
Mus                            ----------  ----------  ----------  ----------  ----------  ----------  ----------  -
Mus_musculus                   ----------  ----------  ----------  ----------  ----------  ----------  ----------  -
Rattus_norvegicus              ----------  ----------  ----------  ----------  ----------  ----------  ----------  -
Tursiops_truncatus             ----------  ----------  ----------  ----------  ----------  ----------  ----------  -
Procavia_capensis              ----------  ----------  ----------  ----------  ----------  ----------  ----------  -
Primates                       ----------  ----------  ----------  ----------  ----------  ----------  ----------  -
Echinops_telfairi              ----------  ----------  ----------  ----------  ----------  ----------  ----------  -
Sorex_araneus                  ----------  ----------  ----------  ----------  ----------  ----------  ----------  -
Canidae                        ----------  ----------  ----------  ----------  ----------  ----------  ----------  -
Otolemur_garnettii             ----------  ----------  ----------  ----------  ----------  ----------  ----------  -
Gallus                         ----------  ----------  ----------  ----------  ----------  ----------  ----------  -
Callithrix_jacchus             ----------  ----------  ----------  ----------  ----------  ----------  ----------  -
Crocodylus_porosus             ----------  ----------  ----------  ----------  ----------  ----------  ----------  -
Erinaceus_europaeus            ----------  ----------  ----------  ----------  ----------  ----------  ----------  -
rnd-6_family-7426-LTR-ERV1     ----------  ----------  ----------  ----------  ----------  ----------  ----------  -
Esox_lucius                    ----------  ----------  ----------  ----------  ----------  ----------  ----------  -
Salmo_salar                    ----------  ----------  ----------  ----------  ----------  ----------  ----------  -
Didelphidae                    ----------  ----------  ----------  ----------  ----------  ----------  ----------  -
Latimeria_chalumnae            ----------  ----------  ----------  ----------  ----------  ----------  ----------  -
Monodelphis_domestica          ----------  ----------  ----------  ----------  ----------  ----------  ----------  -
Macropus_eugenii               ----------  ----------  ----------  ----------  ----------  ----------  ----------  -
Alligator_mississippiensis     ----------  ----------  ----------  ----------  ----------  ----------  ----------  -
Chrysemys_picta_bellii         ----------  ----------  ----------  ----------  ----------  ----------  ----------  -
Danio_rerio                    ----------  ----------  ----------  ----------  ----------  ----------  ----------  -
Taeniopygia                    ----------  ----------  ----------  ----------  ----------  ----------  ----------  -
Dasypus_novemcinctus           ----------  ----------  ----------  ----------  ----------  ----------  ----------  -
LTR_XT                         ----------  ----------  ----------  ----------  ----------  ----------  ----------  -
Haplorrhini                    GCTGTGTAGA  AACAGTGGAT  GAAGTCTTTT  CAAGCCGGGA  AGACTTAAAG  GATCAACCCT  TAAAAGACCC  4582
Catarrhini                     ACCGTGTAGA  GGTGTTGGAC  TCAGTCTATT  CTAGCAGACC  TGACCTTCGG  GACCAGCCAT  GGGCATCAGT  4063
Anolis_carolinensis            ATTGTTTAGA  ANTGATNGAT  GAAGTGTACT  CNAGTNGGCC  NGATCTGAAA  NATNTGCCAC  TTCCA-AGGC  632
Murine_leukemia_virus          ATTGCCTCGA  GATCTTGGCT  GAAACGCATG  GAACCAGACC  GGACCTCACC  GACCAGCCCA  TCCCAGACGC  4026
Phascolarctos_cinereus         TATGCTCAGA  AATCCTTGCC  GAGGAGACAG  GAACCCGACC  TGATCTGAGA  GATCAGCCGT  TGCCCGGGGT  3925
Estrildidae                    ----------  ----------  ----------  ----------  ----------  ----------  ----------  -
Xenopus_Silurana_tropicalis    ----------  ----------  ----------  ----------  ----------  ----------  ----------  -
I_XT                           GTNATGGAAN  ACAATNAAAG  NTACTGTNAA  NCAGGTATAN  GNTTANTGTT  TGACNTGTGC  NNAANTAAAT  4098
Takifugu_rubripes              ----------  ----------  ----------  ----------  ----------  ----------  ----------  -
Bos_taurus                     ----------  ----------  ----------  ----------  ----------  ----------  ----------  -
Homo_sapiens                   ----------  ----------  ----------  ----------  ----------  ----------  ----------  -
Microcebus_murinus             ----------  ----------  ----------  ----------  ----------  ----------  ----------  -
Myotis_lucifugus               ----------  ----------  ----------  ----------  ----------  ----------  ----------  -
Equus_caballus                 ----------  ----------  ----------  ----------  ----------  ----------  ----------  -
Tupaia_belangeri               ----------  ----------  ----------  ----------  ----------  ----------  ----------  -
Loxodonta_africana             ----------  ----------  ----------  ----------  ----------  ----------  ----------  -
Murinae                        ----------  ----------  ----------  ----------  ----------  ----------  ----------  -
Canis_lupus_familiaris         ----------  ----------  ----------  ----------  ----------  ----------  ----------  -
Sus_scrofa                     ----------  ----------  ----------  ----------  ----------  ----------  ----------  -
Choloepus_hoffmanni            ----------  ----------  ----------  ----------  ----------  ----------  ----------  -
Vicugna_pacos                  ----------  ----------  ----------  ----------  ----------  ----------  ----------  -
Gavialis_gangeticus            ----------  ----------  ----------  ----------  ----------  ----------  ----------  -
Mammalia                       ----------  ----------  ----------  ----------  ----------  ----------  ----------  -
Passeroidea                    ----------  ----------  ----------  ----------  ----------  ----------  ----------  -
Takifugu                       ----------  ----------  ----------  ----------  ----------  ----------  ----------  -
Pteropus_vampyrus              ----------  ----------  ----------  ----------  ----------  ----------  ----------  -
Ornithorhynchus                ----------  ----------  ----------  ----------  ----------  ----------  ----------  -
Oryctolagus_cuniculus          ----------  ----------  ----------  ----------  ----------  ----------  ----------  -
Gallus_gallus                  ----------  ----------  ----------  ----------  ----------  ----------  ----------  -
Hominidae                      ACGGCTCCTC  CGCTACCTCC  TACACCACCT  TCCTCTCCCG  CTAACCCTCC  CGCTTCTGCT  CTGCCACCGC  885
Metatheria                     ----------  ----------  ----------  ----------  ----------  ----------  ----------  -
Rodentia                       ----------  ----------  ----------  ----------  ----------  ----------  ----------  -
Monodelphis                    ----------  ----------  ----------  ----------  ----------  ----------  ----------  -
Consensus                      ----------  ----------  ----------  ----------  ----------  ----------  ----------
```

Conservation

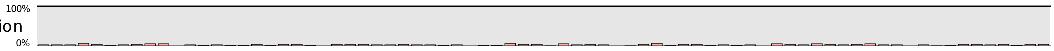

4,640 4,660 4,680

Dipodomys_ordii	----------	----------	----------	----------	----------	----------	----------	-
Laurasiatheria	----------	----------	----------	----------	----------	----------	----------	-
Eutheria	----------	----------	----------	----------	----------	----------	----------	-
Tarsius_syrichta	----------	----------	----------	----------	----------	----------	----------	-
Muridae	----------	----------	----------	----------	----------	----------	----------	-
Gasterosteus_aculeatus	----------	----------	----------	----------	----------	----------	----------	-
Felis_silvestris	----------	----------	----------	----------	----------	----------	----------	-
Rattus	----------	----------	----------	----------	----------	----------	----------	-
Papio_hamadryas	AGTGCGCCTA	CTGCAAAGAG	CGAGGGCATT	GGATCAAGGA	CTGCCCCAAG	CGTCCTAGAG	ACCAGAAGAA	2003
Pan_troglodytes	GCCGGCCGGG	CGGCGTAGAT	CTGGGGCGCT	CAGCCGCTGG	GTTGGGGAGG	CGCCCCGGTG	----GGGCAG	87
Ailuropoda_melanoleuca	----------	----------	----------	----------	----------	----------	----------	-
Cavia_porcellus	----------	----------	----------	----------	----------	----------	----------	-
Ictidomys_tridecemlineatus	----------	----------	----------	----------	----------	----------	----------	-
Canis	----------	----------	----------	----------	----------	----------	----------	-
Felis_catus	----------	----------	----------	----------	----------	----------	----------	-
Ochotona_princeps	----------	----------	----------	----------	----------	----------	----------	-
Cercopithecidae	----------	----------	----------	----------	----------	----------	----------	-
Carnivora	----------	----------	----------	----------	----------	----------	----------	-
Mus	----------	----------	----------	----------	----------	----------	----------	-
Mus_musculus	----------	----------	----------	----------	----------	----------	----------	-
Rattus_norvegicus	----------	----------	----------	----------	----------	----------	----------	-
Tursiops_truncatus	----------	----------	----------	----------	----------	----------	----------	-
Procavia_capensis	----------	----------	----------	----------	----------	----------	----------	-
Primates	----------	----------	----------	----------	----------	----------	----------	-
Echinops_telfairi	----------	----------	----------	----------	----------	----------	----------	-
Sorex_araneus	----------	----------	----------	----------	----------	----------	----------	-
Canidae	----------	----------	----------	----------	----------	----------	----------	-
Otolemur_garnettii	----------	----------	----------	----------	----------	----------	----------	-
Gallus	----------	----------	----------	----------	----------	----------	----------	-
Callithrix_jacchus	----------	----------	----------	----------	----------	----------	----------	-
Crocodylus_porosus	----------	----------	----------	----------	----------	----------	----------	-
Erinaceus_europaeus	----------	----------	----------	----------	----------	----------	----------	-
rnd-6_family-7426-LTR-ERV1	----------	----------	----------	----------	----------	----------	----------	-
Esox_lucius	----------	----------	----------	----------	----------	----------	----------	-
Salmo_salar	----------	----------	----------	----------	----------	----------	----------	-
Didelphidae	----------	----------	----------	----------	----------	----------	----------	-
Latimeria_chalumnae	----------	----------	----------	----------	----------	----------	----------	-
Monodelphis_domestica	----------	----------	----------	----------	----------	----------	----------	-
Macropus_eugenii	----------	----------	----------	----------	----------	----------	----------	-
Alligator_mississippiensis	----------	----------	----------	----------	----------	----------	----------	-
Chrysemys_picta_bellii	----------	----------	----------	----------	----------	----------	----------	-
Danio_rerio	----------	----------	----------	----------	----------	----------	----------	-
Taeniopygia	----------	----------	----------	----------	----------	----------	----------	-
Dasypus_novemcinctus	----------	----------	----------	----------	----------	----------	----------	-
LTR_XT	----------	----------	----------	----------	----------	----------	----------	-
Haplorrhini	AGATGTTGAA	TATTTTACTG	ATGGAAGCAG	CTTCAT--AT	CCG-AGGGTA	TCAGAAAGGC	CAGATATGCC	4649
Catarrhini	AGACTGGGAG	TTATACGTGG	ACGGGAGCAG	CTTCATCAAC	CCACAAGGAG	AAAGATGTGC	AGGATATGCG	4133
Anolis_carolinensis	AGACTGGNTN	TTTTTNACAG	ATGGNNCTAG	NNGTAT--GG	TNA-NTNGCG	AAAGGTAAGC	GGGGTATGCA	699
Murine_leukemia_virus	CGACCACACC	TGGTATACCG	ATGGGAGCAG	CTTTTT---G	CAAGAAGGAC	AGCGAAAAGC	TGGGGCAGCG	4093
Phascolarctos_cinereus	--ACC-CGCT	TGGTATACGG	ATGGCAGCAG	TTTCAT---C	ATGGATGGCA	GGCGACAAGC	AGGTGCTGCC	3989
Estrildidae	----------	----------	----------	----------	----------	----------	----------	-
Xenopus_Silurana_tropicalis	----------	----------	----------	----------	----------	----------	----------	-
I_XT	CTATGTGCNN	ANGGTNAAAG	AACTTTNCAA	CATCCTGCTC	CTGCAGACNG	ANTTTGGTNN	NCTNTNNNAA	4168
Takifugu_rubripes	----------	----------	----------	----------	----------	----------	----------	-
Bos_taurus	----------	----------	----------	----------	----------	----------	----------	-
Homo_sapiens	----------	----------	----------	----------	----------	----------	----------	-
Microcebus_murinus	----------	----------	----------	----------	----------	----------	----------	-
Myotis_lucifugus	----------	----------	----------	----------	----------	----------	----------	-
Equus_caballus	----------	----------	----------	----------	----------	----------	----------	-
Tupaia_belangeri	----------	----------	----------	----------	----------	----------	----------	-
Loxodonta_africana	----------	----------	----------	----------	----------	----------	----------	-
Murinae	----------	----------	----------	----------	----------	----------	----------	-
Canis_lupus_familiaris	----------	----------	----------	----------	----------	----------	----------	-
Sus_scrofa	----------	----------	----------	----------	----------	----------	----------	-
Choloepus_hoffmanni	----------	----------	----------	----------	----------	----------	----------	-
Vicugna_pacos	----------	----------	----------	----------	----------	----------	----------	-
Gavialis_gangeticus	----------	----------	----------	----------	----------	----------	----------	-
Mammalia	----------	----------	----------	----------	----------	----------	----------	-
Passeroidea	----------	----------	----------	----------	----------	----------	----------	-
Takifugu	----------	----------	----------	----------	----------	----------	----------	-
Pteropus_vampyrus	----------	----------	----------	----------	----------	----------	----------	-
Ornithorhynchus	----------	----------	----------	----------	----------	----------	----------	-
Oryctolagus_cuniculus	----------	----------	----------	----------	----------	----------	----------	-
Gallus_gallus	----------	----------	----------	----------	----------	----------	----------	-
Hominidae	CTTCCCCTGT	ATCTGCGCAC	ACTCGGTCGA	AGACGGACCT	CTTGTGTCCG	CTCCGTGAAG	TTGCCGGTGC	955
Metatheria	----------	----------	----------	----------	----------	----------	----------	-
Rodentia	----------	----------	----------	----------	----------	----------	----------	-
Monodelphis	----------	----------	----------	----------	----------	----------	----------	-
Consensus	----------	----------	----------	----------	----------	----------	----------	

Conservation
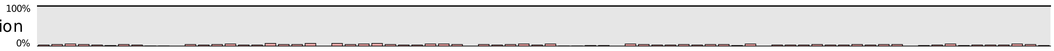

```
                                  4,700                4,720                4,740                4,760
Dipodomys_ordii                   ---------- ---------- ---------- ---------- ---------- ---------- ---------- -
Laurasiatheria                    ---------- ---------- ---------- ---------- ---------- ---------- ---------- -
Eutheria                          ---------- ---------- ---------- ---------- ---------- ---------- ---------- -
Tarsius_syrichta                  ---------- ---------- ---------- ---------- ---------- ---------- ---------- -
Muridae                           ---------- ---------- ---------- ---------- ---------- ---------- ---------- -
Gasterosteus_aculeatus            ---------- ---------- ---------- ---------- ---------- ---------- ---------- -
Felis_silvestris                  ---------- ---------- ---------- ---------- ---------- ---------- ---------- -
Rattus                            ---------- ---------- ---------- ---------- ---------- ---------- ---------- -
Papio_hamadryas                   ACCCGCCCCT GTCCTCACCT TAGGTGAGGA CAGCGAATAG GGGTGTCAGG GCTCTGGAGC CCCCCCCGAG 2073
Pan_troglodytes                   GCTTCTATCT GGCCGCCCTG ACGGTTCCGG GCTCGATCCG CTCCGGGGCA GCTGCCGA-- -----CAGGG 150
Ailuropoda_melanoleuca            ---------- ---------- ---------- ---------- ---------- ---------- ---------- -
Cavia_porcellus                   ---------- ---------- ---------- ---------- ---------- ---------- ---------- -
Ictidomys_tridecemlineatus        ---------- ---------- ---------- ---------- ---------- ---------- ---------- -
Canis                             ---------- ---------- ---------- ---------- ---------- ---------- ---------- -
Felis_catus                       ---------- ---------- ---------- ---------- ---------- ---------- ---------- -
Ochotona_princeps                 ---------- ---------- ---------- ---------- ---------- ---------- ---------- -
Cercopithecidae                   ---------- ---------- ---------- ---------- ---------- ---------- ---------- -
Carnivora                         ---------- ---------- ---------- ---------- ---------- ---------- ---------- -
Mus                               ---------- ---------- ---------- ---------- ---------- ---------- ---------- -
Mus_musculus                      ---------- ---------- ---------- ---------- ---------- ---------- ---------- -
Rattus_norvegicus                 ---------- ---------- ---------- ---------- ---------- ---------- ---------- -
Tursiops_truncatus                ---------- ---------- ---------- ---------- ---------- ---------- ---------- -
Procavia_capensis                 ---------- ---------- ---------- ---------- ---------- ---------- ---------- -
Primates                          ---------- ---------- ---------- ---------- ---------- ---------- ---------- -
Echinops_telfairi                 ---------- ---------- ---------- ---------- ---------- ---------- ---------- -
Sorex_araneus                     ---------- ---------- ---------- ---------- ---------- ---------- ---------- -
Canidae                           ---------- ---------- ---------- ---------- ---------- ---------- ---------- -
Otolemur_garnettii                ---------- ---------- ---------- ---------- ---------- ---------- ---------- -
Gallus                            ---------- ---------- ---------- ---------- ---------- ---------- ---------- -
Callithrix_jacchus                ---------- ---------- ---------- ---------- ---------- ---------- ---------- -
Crocodylus_porosus                ---------- ---------- ---------- ---------- ---------- ---------- ---------- -
Erinaceus_europaeus               ---------- ---------- ---------- ---------- ---------- ---------- ---------- -
rnd-6_family-7426-LTR-ERV1        ---------- ---------- ---------- ---------- ---------- ---------- ---------- -
Esox_lucius                       ---------- ---------- ---------- ---------- ---------- ---------- ---------- -
Salmo_salar                       ---------- ---------- ---------- ---------- ---------- ---------- ---------- -
Didelphidae                       ---------- ---------- ---------- ---------- ---------- ---------- ---------- -
Latimeria_chalumnae               ---------- ---------- ---------- ---------- ---------- ---------- ---------- -
Monodelphis_domestica             ---------- ---------- ---------- ---------- ---------- ---------- ---------- -
Macropus_eugenii                  ---------- ---------- ---------- ---------- ---------- ---------- ---------- -
Alligator_mississippiensis        ---------- ---------- ---------- ---------- ---------- ---------- ---------- -
Chrysemys_picta_bellii            ---------- ---------- ---------- ---------- ---------- ---------- ---------- -
Danio_rerio                       ---------- ---------- ---------- ---------- ---------- ---------- ---------- -
Taeniopygia                       ---------- ---------- ---------- ---------- ---------- ---------- ---------- -
Dasypus_novemcinctus              ---------- ---------- ---------- ---------- ---------- ---------- ---------- -
LTR_XT                            ---------- ---------- ---------- ---------- ---------- ---------- ---------- -
Haplorrhini                       GTAGTCACAT TAAGCTCGG- ---TGGCTGA GGCCCGCCCC CTACCGGTAG GAACCTCAGC GCAGAAGGCA 4715
Catarrhini                        GTGGTAACTT TGGACGCTG- ---TCATTGA AGCCAAACCG TTGCCACAGG GCACTTCAGC CCAGAAGGCT 4199
Anolis_carolinensis               GTGGT-GTTT AGAACTCGGG AGACCCTAGA GGCAAAGGGN CTGCCTCCGG GGACTTCCGC TCAAAANGCC 768
Murine_leukemia_virus             GTGACGACNG AGACCGAGG- ---TAATCTG GGCGAGGGCC CTGCCGGCTG GAACGTCAGC CCAGCGAGCC 4159
Phascolarctos_cinereus            ATTGTGGACA ACAAGCGGA- ---CGGTGTG GGCGAGCAAC CTGCCAGAGG GAACGTCAGC TCAGAAGGCC 4055
Estrildidae                       ---------- ---------- ---------- ---------- ---------- ---------- ---------- -
Xenopus_Silurana_tropicalis       ---------- ---------- ---------- ---------- ---------- ---------- ---------- -
I_XT                              TCAATTATAN AGGNNCANTA CAGTCAGGGA CTTATGGNCT TAAGTACANA CTNNTNNTAA TGNACNTATT 4238
Takifugu_rubripes                 ---------- ---------- ---------- ---------- ---------- ---------- ---------- -
Bos_taurus                        ---------- ---------- ---------- ---------- ---------- ---------- ---------- -
Homo_sapiens                      ---------- ---------- ---------- ---------- ---------- ---------- ---------- -
Microcebus_murinus                ---------- ---------- ---------- ---------- ---------- ---------- ---------- -
Myotis_lucifugus                  ---------- ---------- ---------- ---------- ---------- ---------- ---------- -
Equus_caballus                    ---------- ---------- ---------- ---------- ---------- ---------- ---------- -
Tupaia_belangeri                  ---------- ---------- ---------- ---------- ---------- ---------- ---------- -
Loxodonta_africana                ---------- ---------- ---------- ---------- ---------- ---------- ---------- -
Murinae                           ---------- ---------- ---------- ---------- ---------- ---------- ---------- -
Canis_lupus_familiaris            ---------- ---------- ---------- ---------- ---------- ---------- ---------- -
Sus_scrofa                        ---------- ---------- ---------- ---------- ---------- ---------- ---------- -
Choloepus_hoffmanni               ---------- ---------- ---------- ---------- ---------- ---------- ---------- -
Vicugna_pacos                     ---------- ---------- ---------- ---------- ---------- ---------- ---------- -
Gavialis_gangeticus               ---------- ---------- ---------- ---------- ---------- ---------- ---------- -
Mammalia                          ---------- ---------- ---------- ---------- ---------- ---------- ---------- -
Passeroidea                       ---------- ---------- ---------- ---------- ---------- ---------- ---------- -
Takifugu                          ---------- ---------- ---------- ---------- ---------- ---------- ---------- -
Pteropus_vampyrus                 ---------- ---------- ---------- ---------- ---------- ---------- ---------- -
Ornithorhynchus                   ---------- ---------- ---------- ---------- ---------- ---------- ---------- -
Oryctolagus_cuniculus             ---------- ---------- ---------- ---------- ---------- ---------- ---------- -
Gallus_gallus                     ---------- ---------- ---------- ---------- ---------- ---------- ---------- -
Hominidae                         GGAAGGCGTG GTCAGGGTCC ATGTTCCCTT TTCTCTTACT GACTTATCTA AAATAGAGAA GCGGCCTGGG 1025
Metatheria                        ---------- ---------- ---------- ---------- ---------- ---------- ---------- -
Rodentia                          ---------- ---------- ---------- ---------- ---------- ---------- ---------- -
Monodelphis                       ---------- ---------- ---------- ---------- ---------- ---------- ---------- -

Consensus                         ---------- ---------- ---------- ---------- ---------- ---------- ----------
```

Conservation

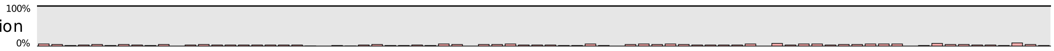

4,780 4,800 4,820

Dipodomys_ordii	----------	----------	----------	----------	----------	----------	----------	-
Laurasiatheria	----------	----------	----------	----------	----------	----------	----------	-
Eutheria	----------	----------	----------	----------	----------	----------	----------	-
Tarsius_syrichta	----------	----------	----------	----------	----------	----------	----------	-
Muridae	----------	----------	----------	----------	----------	----------	----------	-
Gasterosteus_aculeatus	----------	----------	----------	----------	----------	----------	----------	-
Felis_silvestris	----------	----------	----------	----------	----------	----------	----------	-
Rattus	----------	----------	----------	----------	----------	----------	----------	-
Papio_hamadryas	CCCCGGCTAA	CTCTATCTGT	AGGGGGGCAT	CCCACCACCT	TCTTGGTGGA	CACAGGCGCC	CAACACTCGG	2143
Pan_troglodytes	CTGCAGGCTG	GATTCTTAGT	AATCTTTTTC	GTTCACATTT	AGGAAGAAAA	TATAGGAGAC	CCGAACTCCT	220
Ailuropoda_melanoleuca	----------	----------	----------	----------	----------	----------	----------	-
Cavia_porcellus	----------	----------	----------	----------	----------	----------	----------	-
Ictidomys_tridecemlineatus	----------	----------	----------	----------	----------	----------	----------	-
Canis	----------	----------	----------	----------	----------	----------	----------	-
Felis_catus	----------	----------	----------	----------	----------	----------	----------	-
Ochotona_princeps	----------	----------	----------	----------	----------	----------	----------	-
Cercopithecidae	----------	----------	----------	----------	----------	----------	----------	-
Carnivora	----------	----------	----------	----------	----------	----------	----------	-
Mus	----------	----------	----------	----------	----------	----------	----------	-
Mus_musculus	----------	----------	----------	----------	----------	----------	----------	-
Rattus_norvegicus	----------	----------	----------	----------	----------	----------	----------	-
Tursiops_truncatus	----------	----------	----------	----------	----------	----------	----------	-
Procavia_capensis	----------	----------	----------	----------	----------	----------	----------	-
Primates	----------	----------	----------	----------	----------	----------	----------	-
Echinops_telfairi	----------	----------	----------	----------	----------	----------	----------	-
Sorex_araneus	----------	----------	----------	----------	----------	----------	----------	-
Canidae	----------	----------	----------	----------	----------	----------	----------	-
Otolemur_garnettii	----------	----------	----------	----------	----------	----------	----------	-
Gallus	----------	----------	----------	----------	----------	----------	----------	-
Callithrix_jacchus	----------	----------	----------	----------	----------	----------	----------	-
Crocodylus_porosus	----------	----------	----------	----------	----------	----------	----------	-
Erinaceus_europaeus	----------	----------	----------	----------	----------	----------	----------	-
rnd-6_family-7426-LTR-ERV1	----------	----------	----------	----------	----------	----------	----------	-
Esox_lucius	----------	----------	----------	----------	----------	----------	----------	-
Salmo_salar	----------	----------	----------	----------	----------	----------	----------	-
Didelphidae	----------	----------	----------	----------	----------	----------	----------	-
Latimeria_chalumnae	----------	----------	----------	----------	----------	----------	----------	-
Monodelphis_domestica	----------	----------	----------	----------	----------	----------	----------	-
Macropus_eugenii	----------	----------	----------	----------	----------	----------	----------	-
Alligator_mississippiensis	----------	----------	----------	----------	----------	----------	----------	-
Chrysemys_picta_bellii	----------	----------	----------	----------	----------	----------	----------	-
Danio_rerio	----------	----------	----------	----------	----------	----------	----------	-
Taeniopygia	----------	----------	----------	----------	----------	----------	----------	-
Dasypus_novemcinctus	----------	----------	----------	----------	----------	----------	----------	-
LTR_XT	----------	----------	----------	----------	----------	----------	----------	-
Haplorrhini	GAACTAATAG	CTCTCACAAG	AGCACTACTT	CTAGCGAAGG	GAAAGTCAGT	GAATATCTAT	ACTGACTCAA	4785
Catarrhini	GAGCTCATTG	CTTTAACTCG	GGCTCTAGAA	CTCAGTGAAG	GTAAGACTGT	AAACATCTAC	ACTGACTCTC	4269
Anolis_carolinensis	GAGTTGATTG	CTCTGACAAG	NGCACTNGAA	TTGNCANAAG	GAAAAACAGT	TGATATTTAT	ACTGACTCNA	838
Murine_leukemia_virus	GAACTGATCG	CACTCACCCA	AGCCCTGAAA	ATGGCAGAAG	GTAAGAAGCT	AAATGTTTAC	ACTGATAGCC	4229
Phascolarctos_cinereus	GAACTAATCG	CCTTGACACA	AGCATTACGC	TTGGCCGAAG	GAAAAAGCAT	CAACATCTAC	ACGGACAGCA	4125
Estrildidae	----------	----------	----------	----------	----------	----------	----------	-
Xenopus_Silurana_tropicalis	----------	----------	----------	----------	----------	----------	----------	-
I_XT	TANNTAATGN	ANANAAGGNA	TGCCTGTTAN	GAANAATGAN	GCACANNCAA	CTATTAAAGC	TGTGTGAGAG	4308
Takifugu_rubripes	----------	----------	----------	----------	----------	----------	----------	-
Bos_taurus	----------	----------	----------	----------	----------	----------	----------	-
Homo_sapiens	----------	----------	----------	----------	----------	----------	----------	-
Microcebus_murinus	----------	----------	----------	----------	----------	----------	----------	-
Myotis_lucifugus	----------	----------	----------	----------	----------	----------	----------	-
Equus_caballus	----------	----------	----------	----------	----------	----------	----------	-
Tupaia_belangeri	----------	----------	----------	----------	----------	----------	----------	-
Loxodonta_africana	----------	----------	----------	----------	----------	----------	----------	-
Murinae	----------	----------	----------	----------	----------	----------	----------	-
Canis_lupus_familiaris	----------	----------	----------	----------	----------	----------	----------	-
Sus_scrofa	----------	----------	----------	----------	----------	----------	----------	-
Choloepus_hoffmanni	----------	----------	----------	----------	----------	----------	----------	-
Vicugna_pacos	----------	----------	----------	----------	----------	----------	----------	-
Gavialis_gangeticus	----------	----------	----------	----------	----------	----------	----------	-
Mammalia	----------	----------	----------	----------	----------	----------	----------	-
Passeroidea	----------	----------	----------	----------	----------	----------	----------	-
Takifugu	----------	----------	----------	----------	----------	----------	----------	-
Pteropus_vampyrus	----------	----------	----------	----------	----------	----------	----------	-
Ornithorhynchus	----------	----------	----------	----------	----------	----------	----------	-
Oryctolagus_cuniculus	----------	----------	----------	----------	----------	----------	----------	-
Gallus_gallus	----------	----------	----------	----------	----------	----------	----------	-
Hominidae	TCCTTCTCTG	CCAACCCAAC	CCTGTATATC	AAACAATTTA	GGTACCTATG	CCAGGCTTAT	GACCTCACCT	1095
Metatheria	----------	----------	----------	----------	----------	----------	----------	-
Rodentia	----------	----------	----------	----------	----------	----------	----------	-
Monodelphis	----------	----------	----------	----------	----------	----------	----------	-
Consensus	----------	----------	----------	----------	----------	----------	----------	

Conservation

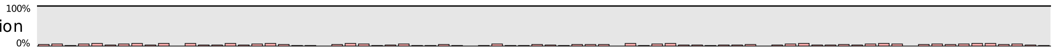

	4,840	4,860	4,880	4,900	
Dipodomys_ordii	---------- ----------	---------- ----------	---------- ----------	----------	-
Laurasiatheria	---------- ----------	---------- ----------	---------- ----------	----------	-
Eutheria	---------- ----------	---------- ----------	---------- ----------	----------	-
Tarsius_syrichta	---------- ----------	---------- ----------	---------- ----------	----------	-
Muridae	---------- ----------	---------- ----------	---------- ----------	----------	-
Gasterosteus_aculeatus	---------- ----------	---------- ----------	---------- ----------	----------	-
Felis_silvestris	---------- ----------	---------- ----------	---------- ----------	----------	-
Rattus	---------- ----------	---------- ----------	---------- ----------	----------	-
Papio_hamadryas	TTTTGACCAA GGCAAACGGA	CCCCTGTCCT CTCGTACATC	TTGGGTCCAG GGGGCAACAG	GAAGAAAGAT	2213
Pan_troglodytes	CTATCTCTCT GATATTCGAG	TTA--GGCAG GGGCCAGATC	TGGTTAGAAA TAGAAAGGAA	AACTTGCTCC	288
Ailuropoda_melanoleuca	---------- ----------	---------- ----------	---------- ----------	----------	-
Cavia_porcellus	---------- ----------	---------- ----------	---------- ----------	----------	-
Ictidomys_tridecemlineatus	---------- ----------	---------- ----------	---------- ----------	----------	-
Canis	---------- ----------	---------- ----------	---------- ----------	----------	-
Felis_catus	---------- ----------	---------- ----------	---------- ----------	----------	-
Ochotona_princeps	---------- ----------	---------- ----------	---------- ----------	----------	-
Cercopithecidae	---------- ----------	---------- ----------	---------- ----------	----------	-
Carnivora	---------- ----------	---------- ----------	---------- ----------	----------	-
Mus	---------- ----------	---------- ----------	---------- ----------	----------	-
Mus_musculus	---------- ----------	---------- ----------	---------- ----------	----------	-
Rattus_norvegicus	---------- ----------	---------- ----------	---------- ----------	----------	-
Tursiops_truncatus	---------- ----------	---------- ----------	---------- ----------	----------	-
Procavia_capensis	---------- ----------	---------- ----------	---------- ----------	----------	-
Primates	---------- ----------	---------- ----------	---------- ----------	----------	-
Echinops_telfairi	---------- ----------	---------- ----------	---------- ----------	----------	-
Sorex_araneus	---------- ----------	---------- ----------	---------- ----------	----------	-
Canidae	---------- ----------	---------- ----------	---------- ----------	----------	-
Otolemur_garnettii	---------- ----------	---------- ----------	---------- ----------	----------	-
Gallus	---------- ----------	---------- ----------	---------- ----------	----------	-
Callithrix_jacchus	---------- ----------	---------- ----------	---------- ----------	----------	-
Crocodylus_porosus	---------- ----------	---------- ----------	---------- ----------	----------	-
Erinaceus_europaeus	---------- ----------	---------- ----------	---------- ----------	----------	-
rnd-6_family-7426-LTR-ERV1	---------- ----------	---------- ----------	---------- ----------	----------	-
Esox_lucius	---------- ----------	---------- ----------	---------- ----------	----------	-
Salmo_salar	---------- ----------	---------- ----------	---------- ----------	----------	-
Didelphidae	---------- ----------	---------- ----------	---------- ----------	----------	-
Latimeria_chalumnae	---------- ----------	---------- ----------	---------- ----------	----------	-
Monodelphis_domestica	---------- ----------	---------- ----------	---------- ----------	----------	-
Macropus_eugenii	---------- ----------	---------- ----------	---------- ----------	----------	-
Alligator_mississippiensis	---------- ----------	---------- ----------	---------- ----------	----------	-
Chrysemys_picta_bellii	---------- ----------	---------- ----------	---------- ----------	----------	-
Danio_rerio	---------- ----------	---------- ----------	---------- ----------	----------	-
Taeniopygia	---------- ----------	---------- ----------	---------- ----------	----------	-
Dasypus_novemcinctus	---------- ----------	---------- ----------	---------- ----------	----------	-
LTR_XT	---------- ----------	---------- ----------	---------- ----------	----------	-
Haplorrhini	GATATGCTTT TGCCACCTTG	CATGCTCATG GAGCCATTTA	CAAAGAAAGA GGACTGTTAA	CTACTGAAGG	4855
Catarrhini	GATACGCCTT TCTAACCCTC	CAAGTGCATG GAGCATTATA	TAAGGAAAAG GGCCTGTTAA	ACTCTGGGGG	4339
Anolis_carolinensis	AATATGCCTT CANTACTCTN	CACGCNCACG GTGNNATTTA	GAAGGAAAGC GGACTGTTGA	CCTCTGCAGG	908
Murine_leukemia_virus	GCTATGCCTT CGCCACGGCC	CATGTCCATG GGGAAATATA	TAGGAGGCGG GGGTTGCTGA	CCTCAGAAGG	4299
Phascolarctos_cinereus	GGTACGCTTT TGCCACCGCT	CATGTTCACG GGGCTATATA	CAAACAAAGA GGACTGCTCA	CTTCCGCTGG	4195
Estrildidae	---------- ----------	---------- ----------	---------- ----------	----------	-
Xenopus_Silurana_tropicalis	---------- ----------	---------- ----------	---------- ----------	----------	-
I_XT	TATGTGTTCT CCAGATGGGG	TTTTCCACAA ATTTTAGAGT	GGGACAGNGG NACCTTATTT	ACTTGTCANG	4378
Takifugu_rubripes	---------- ----------	---------- ----------	---------- ----------	----------	-
Bos_taurus	---------- ----------	---------- ----------	---------- ----------	----------	-
Homo_sapiens	---------- ----------	---------- ----------	---------- ----------	----------	-
Microcebus_murinus	---------- ----------	---------- ----------	---------- ----------	----------	-
Myotis_lucifugus	---------- ----------	---------- ----------	---------- ----------	----------	-
Equus_caballus	---------- ----------	---------- ----------	---------- ----------	----------	-
Tupaia_belangeri	---------- ----------	---------- ----------	---------- ----------	----------	-
Loxodonta_africana	---------- ----------	---------- ----------	---------- ----------	----------	-
Murinae	---------- ----------	---------- ----------	---------- ----------	----------	-
Canis_lupus_familiaris	---------- ----------	---------- ----------	---------- ----------	----------	-
Sus_scrofa	---------- ----------	---------- ----------	---------- ----------	----------	-
Choloepus_hoffmanni	---------- ----------	---------- ----------	---------- ----------	----------	-
Vicugna_pacos	---------- ----------	---------- ----------	---------- ----------	----------	-
Gavialis_gangeticus	---------- ----------	---------- ----------	---------- ----------	----------	-
Mammalia	---------- ----------	---------- ----------	---------- ----------	----------	-
Passeroidea	---------- ----------	---------- ----------	---------- ----------	----------	-
Takifugu	---------- ----------	---------- ----------	---------- ----------	----------	-
Pteropus_vampyrus	---------- ----------	---------- ----------	---------- ----------	----------	-
Ornithorhynchus	---------- ----------	---------- ----------	---------- ----------	----------	-
Oryctolagus_cuniculus	---------- ----------	---------- ----------	---------- ----------	----------	-
Gallus_gallus	---------- ----------	---------- ----------	---------- ----------	----------	-
Hominidae	GGCGTGACCT ACATATTATC	CTAACATCCA CTCTGTCCCC	AGAGGAGAGG GAGCGAGTCC	AGGCGGTGGC	1165
Metatheria	---------- ----------	---------- ----------	---------- ----------	----------	-
Rodentia	---------- ----------	---------- ----------	---------- ----------	----------	-
Monodelphis	---------- ----------	---------- ----------	---------- ----------	----------	-
Consensus	---------- ----------	---------- ----------	---------- ----------	----------	

Conservation

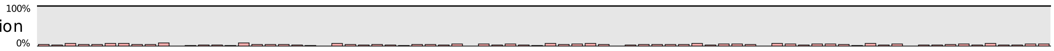

```
                                       4,920                4,940                4,960
                      Dipodomys_ordii ---------- ---------- ---------- ---------- ---------- ---------- ---------- -
                        Laurasiatheria ---------- ---------- ---------- ---------- ---------- ---------- ---------- -
                              Eutheria ---------- ---------- ---------- ---------- ---------- ---------- ---------- -
                      Tarsius_syrichta ---------- ---------- ---------- ---------- ---------- ---------- ---------- -
                              Muridae ---------- ---------- ---------- ---------- ---------- ---------- ---------- -
                Gasterosteus_aculeatus ---------- ---------- ---------- ---------- ---------- ---------- ---------- -
                      Felis_silvestris ---------- ---------- ---------- ---------- ---------- ---------- ---------- -
                                Rattus ---------- ---------- ---------- ---------- ---------- ---------- ---------- -
                      Papio_hamadryas GCACAAATGG ACTAACCGCC GGACAGTTAA CCTAGGGCAA GGAATGGTGA CACACTCCTT CTTGGTGGTA 2283
                       Pan_troglodytes GAATGGCCGC CTGTGTGGGT GGCCCGGGGA CCTTCGACTG CTCATCAGGG TAAGCAAGTT ACCGGGCCGG 358
                 Ailuropoda_melanoleuca ---------- ---------- ---------- ---------- ---------- ---------- ---------- -
                       Cavia_porcellus ---------- ---------- ---------- ---------- ---------- ---------- ---------- -
            Ictidomys_tridecemlineatus ---------- ---------- ---------- ---------- ---------- ---------- ---------- -
                                 Canis ---------- ---------- ---------- ---------- ---------- ---------- ---------- -
                           Felis_catus ---------- ---------- ---------- ---------- ---------- ---------- ---------- -
                      Ochotona_princeps ---------- ---------- ---------- ---------- ---------- ---------- ---------- -
                       Cercopithecidae ---------- ---------- ---------- ---------- ---------- ---------- ---------- -
                             Carnivora ---------- ---------- ---------- ---------- ---------- ---------- ---------- -
                                   Mus ---------- ---------- ---------- ---------- ---------- ---------- ---------- -
                           Mus_musculus ---------- ---------- ---------- ---------- ---------- ---------- ---------- -
                     Rattus_norvegicus ---------- ---------- ---------- ---------- ---------- ---------- ---------- -
                     Tursiops_truncatus ---------- ---------- ---------- ---------- ---------- ---------- ---------- -
                      Procavia_capensis ---------- ---------- ---------- ---------- ---------- ---------- ---------- -
                              Primates ---------- ---------- ---------- ---------- ---------- ---------- ---------- -
                      Echinops_telfairi ---------- ---------- ---------- ---------- ---------- ---------- ---------- -
                         Sorex_araneus ---------- ---------- ---------- ---------- ---------- ---------- ---------- -
                               Canidae ---------- ---------- ---------- ---------- ---------- ---------- ---------- -
                     Otolemur_garnettii ---------- ---------- ---------- ---------- ---------- ---------- ---------- -
                                Gallus ---------- ---------- ---------- ---------- ---------- ---------- ---------- -
                    Callithrix_jacchus ---------- ---------- ---------- ---------- ---------- ---------- ---------- -
                    Crocodylus_porosus ---------- ---------- ---------- ---------- ---------- ---------- ---------- -
                   Erinaceus_europaeus ---------- ---------- ---------- ---------- ---------- ---------- ---------- -
               rnd-6_family-7426-LTR-ERV1 ---------- ---------- ---------- ---------- ---------- ---------- ---------- -
                           Esox_lucius ---------- ---------- ---------- ---------- ---------- ---------- ---------- -
                           Salmo_salar ---------- ---------- ---------- ---------- ---------- ---------- ---------- -
                           Didelphidae ---------- ---------- ---------- ---------- ---------- ---------- ---------- -
                   Latimeria_chalumnae ---------- ---------- ---------- ---------- ---------- ---------- ---------- -
                  Monodelphis_domestica ---------- ---------- ---------- ---------- ---------- ---------- ---------- -
                       Macropus_eugenii ---------- ---------- ---------- ---------- ---------- ---------- ---------- -
             Alligator_mississippiensis ---------- ---------- ---------- ---------- ---------- ---------- ---------- -
                 Chrysemys_picta_bellii ---------- ---------- ---------- ---------- ---------- ---------- ---------- -
                           Danio_rerio ---------- ---------- ---------- ---------- ---------- ---------- ---------- -
                           Taeniopygia ---------- ---------- ---------- ---------- ---------- ---------- ---------- -
                   Dasypus_novemcinctus ---------- ---------- ---------- ---------- ---------- ---------- ---------- -
                                LTR_XT ---------- ---------- ---------- ---------- ---------- ---------- ---------- -
                           Haplorrhini AAAAGAAATA AAGAACAAAA AGGAAATAGA GCAGCTCCTA GAGGCTGTAT GGGCTCCAAA GGAAGTAGCA 4925
                            Catarrhini AAAGGACATA AAATATCAAC AAGAAATTCT ACAATTATTA GAGGCAGTGT GGAAACCTCA GAAGGCGGCA 4409
                    Anolis_carolinensis ACAGCAGATC AAANACCCAA NANAAATTTT GNANCTCTTA GATGNTNTTT GGATNCCNAA TGAGGTAGCA 978
                  Murine_leukemia_virus CAAGGAAATC AAAAACAAAA GCGAGATCCT AGCCTTGCTG AAAGCCCTCT TTCTGCCNAA GAGACTCAGT 4369
                 Phascolarctos_cinereus AAAAGACATC AAGAACAAAG AGGAGATTTT GGCCCTGCTA GAGGCCATCC ATCTCCCTAA GCGAGTCGCT 4265
                           Estrildidae ---------- ---------- ---------- ---------- ---------- ---------- ---------- -
             Xenopus_Silurana_tropicalis ---------- ---------- ---------- ---------- ---------- ---------- ---------- -
                                  I_XT NAANGCTAAA GACATATGCN ATNTTGGNNA TNACACTACG TACACATNGT GTNNCNTCCN NAATCTTNNG 4448
                      Takifugu_rubripes ---------- ---------- ---------- ---------- ---------- ---------- ---------- -
                             Bos_taurus ---------- ---------- ---------- ---------- ---------- ---------- ---------- -
                           Homo_sapiens ---------- ---------- ---------- ---------- ---------- ---------- ---------- -
                      Microcebus_murinus ---------- ---------- ---------- ---------- ---------- ---------- ---------- -
                      Myotis_lucifugus ---------- ---------- ---------- ---------- ---------- ---------- ---------- -
                         Equus_caballus ---------- ---------- ---------- ---------- ---------- ---------- ---------- -
                      Tupaia_belangeri ---------- ---------- ---------- ---------- ---------- ---------- ---------- -
                     Loxodonta_africana ---------- ---------- ---------- ---------- ---------- ---------- ---------- -
                               Murinae ---------- ---------- ---------- ---------- ---------- ---------- ---------- -
                 Canis_lupus_familiaris ---------- ---------- ---------- ---------- ---------- ---------- ---------- -
                             Sus_scrofa ---------- ---------- ---------- ---------- ---------- ---------- ---------- -
                    Choloepus_hoffmanni ---------- ---------- ---------- ---------- ---------- ---------- ---------- -
                           Vicugna_pacos ---------- ---------- ---------- ---------- ---------- ---------- ---------- -
                    Gavialis_gangeticus ---------- ---------- ---------- ---------- ---------- ---------- ---------- -
                              Mammalia ---------- ---------- ---------- ---------- ---------- ---------- ---------- -
                           Passeroidea ---------- ---------- ---------- ---------- ---------- ---------- ---------- -
                              Takifugu ---------- ---------- ---------- ---------- ---------- ---------- ---------- -
                      Pteropus_vampyrus ---------- ---------- ---------- ---------- ---------- ---------- ---------- -
                        Ornithorhynchus ---------- ---------- ---------- ---------- ---------- ---------- ---------- -
                  Oryctolagus_cuniculus ---------- ---------- ---------- ---------- ---------- ---------- ---------- -
                           Gallus_gallus ---------- ---------- ---------- ---------- ---------- ---------- ---------- -
                             Hominidae TAGGCAACAT GCCGACCAAA TTCATTTAAC TGACCCCGCC ATGCCTGTCG GAACCCTAGC AGTACCGGCA 1235
                            Metatheria ---------- ---------- ---------- ---------- ---------- ---------- ---------- -
                              Rodentia ---------- ---------- ---------- ---------- ---------- ---------- ---------- -
                            Monodelphis ---------- ---------- ---------- ---------- ---------- ---------- ---------- -
                             Consensus ---------- ---------- ---------- ---------- ---------- ---------- ----------
```

Conservation

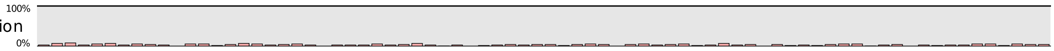

	4,980		5,000		5,020		5,040	
Dipodomys_ordii	----------	----------	----------	----------	----------	----------	----------	-
Laurasiatheria	----------	----------	----------	----------	----------	----------	----------	-
Eutheria	----------	----------	----------	----------	----------	----------	----------	-
Tarsius_syrichta	----------	----------	----------	----------	----------	----------	----------	-
Muridae	----------	----------	----------	----------	----------	----------	----------	-
Gasterosteus_aculeatus	----------	----------	----------	----------	----------	----------	----------	-
Felis_silvestris	----------	----------	----------	----------	----------	----------	----------	-
Rattus	----------	----------	----------	----------	----------	----------	----------	-
Papio_hamadryas	CCTGAATGTC	CGTACCCCCT	TCTGGGGCGA	GATCTCCTAA	CCAAACTCGG	AGCTCAGATC	CACTTCTCCG	2353
Pan_troglodytes	CCACTGACAT	CCTATATCCT	CATTGGAGGA	CCTGTTAGAC	CCCCCCTTGG	TGCCT---TC	CACCTGCCCT	425
Ailuropoda_melanoleuca	----------	----------	----------	----------	----------	----------	----------	-
Cavia_porcellus	----------	----------	----------	----------	----------	----------	----------	-
Ictidomys_tridecemlineatus	----------	----------	----------	----------	----------	----------	----------	-
Canis	----------	----------	----------	----------	----------	----------	----------	-
Felis_catus	----------	----------	----------	----------	----------	----------	----------	-
Ochotona_princeps	----------	----------	----------	----------	----------	----------	----------	-
Cercopithecidae	----------	----------	----------	----------	----------	----------	----------	-
Carnivora	----------	----------	----------	----------	----------	----------	----------	-
Mus	----------	----------	----------	----------	----------	----------	----------	-
Mus_musculus	----------	----------	----------	----------	----------	----------	----------	-
Rattus_norvegicus	----------	----------	----------	----------	----------	----------	----------	-
Tursiops_truncatus	----------	----------	----------	----------	----------	----------	----------	-
Procavia_capensis	----------	----------	----------	----------	----------	----------	----------	-
Primates	----------	----------	----------	----------	----------	----------	----------	-
Echinops_telfairi	----------	----------	----------	----------	----------	----------	----------	-
Sorex_araneus	----------	----------	----------	----------	----------	----------	----------	-
Canidae	----------	----------	----------	----------	----------	----------	----------	-
Otolemur_garnettii	----------	----------	----------	----------	----------	----------	----------	-
Gallus	----------	----------	----------	----------	----------	----------	----------	-
Callithrix_jacchus	----------	----------	----------	----------	----------	----------	----------	-
Crocodylus_porosus	----------	----------	----------	----------	----------	----------	----------	-
Erinaceus_europaeus	----------	----------	----------	----------	----------	----------	----------	-
rnd-6_family-7426-LTR-ERV1	----------	----------	----------	----------	----------	----------	----------	-
Esox_lucius	----------	----------	----------	----------	----------	----------	----------	-
Salmo_salar	----------	----------	----------	----------	----------	----------	----------	-
Didelphidae	----------	----------	----------	----------	----------	----------	----------	-
Latimeria_chalumnae	----------	----------	----------	----------	----------	----------	----------	-
Monodelphis_domestica	----------	----------	----------	----------	----------	----------	----------	-
Macropus_eugenii	----------	----------	----------	----------	----------	----------	----------	-
Alligator_mississippiensis	----------	----------	----------	----------	----------	----------	----------	-
Chrysemys_picta_bellii	----------	----------	----------	----------	----------	----------	----------	-
Danio_rerio	----------	----------	----------	----------	----------	----------	----------	-
Taeniopygia	----------	----------	----------	----------	----------	----------	----------	-
Dasypus_novemcinctus	----------	----------	----------	----------	----------	----------	----------	-
LTR_XT	----------	----------	----------	----------	----------	----------	----------	-
Haplorrhini	GTCATCCACT	GTAAAGGACA	TCAAACAGGA	GGAAGTGATG	AGGCTACAGG	AAACAGAAAA	GCAGACAAGG	4995
Catarrhini	GTCATGCACT	GCAGGGGACA	CCAGCGAGCC	TCCACCTCAG	TGGCCTTAGG	AAACTCTCGA	GCTGATTCAG	4479
Anolis_carolinensis	GTCATGCATT	GCAAAGGACA	GNAGTATGGA	GATGATCAGG	TAACCCACGG	AAATCGCCNN	GCNGATC---	1045
Murine_leukemia_virus	ATAATCCATT	GCCCGGGACA	TCAAAAAGGA	AACAGTGCCG	AAGCCAGGGG	CAACCGTATG	GCGGACCAAG	4439
Phascolarctos_cinereus	ATCATCCATT	GCCCCGGCCA	CCAAAGAGGG	ACTGACCCTG	TGGCTACCGG	AAACCGAAAG	GCCGATGAGG	4335
Estrildidae	----------	----------	----------	----------	----------	----------	----------	-
Xenopus_Silurana_tropicalis	----------	----------	----------	----------	----------	----------	----------	-
I_XT	CTATTGTCAA	AAGNNAANCG	CATCATAAAN	TCCAAANTGA	CANAACTGTN	NGTAGNNAAA	GGAAAGACTT	4518
Takifugu_rubripes	----------	----------	----------	----------	----------	----------	----------	-
Bos_taurus	----------	----------	----------	----------	----------	----------	----------	-
Homo_sapiens	----------	----------	----------	----------	----------	----------	----------	-
Microcebus_murinus	----------	----------	----------	----------	----------	----------	----------	-
Myotis_lucifugus	----------	----------	----------	----------	----------	----------	----------	-
Equus_caballus	----------	----------	----------	----------	----------	----------	----------	-
Tupaia_belangeri	----------	----------	----------	----------	----------	----------	----------	-
Loxodonta_africana	----------	----------	----------	----------	----------	----------	----------	-
Murinae	----------	----------	----------	----------	----------	----------	----------	-
Canis_lupus_familiaris	----------	----------	----------	----------	----------	----------	----------	-
Sus_scrofa	----------	----------	----------	----------	----------	----------	----------	-
Choloepus_hoffmanni	----------	----------	----------	----------	----------	----------	----------	-
Vicugna_pacos	----------	----------	----------	----------	----------	----------	----------	-
Gavialis_gangeticus	----------	----------	----------	----------	----------	----------	----------	-
Mammalia	----------	----------	----------	----------	----------	----------	----------	-
Passeroidea	----------	----------	----------	----------	----------	----------	----------	-
Takifugu	----------	----------	----------	----------	----------	----------	----------	-
Pteropus_vampyrus	----------	----------	----------	----------	----------	----------	----------	-
Ornithorhynchus	----------	----------	----------	----------	----------	----------	----------	-
Oryctolagus_cuniculus	----------	----------	----------	----------	----------	----------	----------	-
Gallus_gallus	----------	----------	----------	----------	----------	----------	----------	-
Hominidae	GCCGAGCCGG	ACTGGGATTA	CCAAGCTGGT	CAGACTGGCC	GTCGACGCCG	TGACCAAATG	GTTCAGTGCC	1305
Metatheria	----------	----------	----------	----------	----------	----------	----------	-
Rodentia	----------	----------	----------	----------	----------	----------	----------	-
Monodelphis	----------	----------	----------	----------	----------	----------	----------	-
Consensus	----------	----------	----------	----------	----------	----------	----------	

Conservation (100% – 0%)

5,060 5,080 5,100

Dipodomys_ordii	----------	----------	----------	----------	----------	----------	----------	-
Laurasiatheria	----------	----------	----------	----------	----------	----------	----------	-
Eutheria	----------	----------	----------	----------	----------	----------	----------	-
Tarsius_syrichta	----------	----------	----------	----------	----------	----------	----------	-
Muridae	----------	----------	----------	----------	----------	----------	----------	-
Gasterosteus_aculeatus	----------	----------	----------	----------	----------	----------	----------	-
Felis_silvestris	----------	----------	----------	----------	----------	----------	----------	-
Rattus	----------	----------	----------	----------	----------	----------	----------	-
Papio_hamadryas	AGGCAGGGGC	CCAGGTGTTA	GACCGAGATG	GCCAACCCAT	CCAAATTTTG	ACTGTGTCTC	TGCAAGATGA	2423
Pan_troglodytes	TAGCTCTCGC	CTAAAC--CA	AGCAACCAAC	GCCCCCCCAC	CTGTTCTCCG	AAGGGGACCC	--CTTCACAG	491
Ailuropoda_melanoleuca	----------	----------	----------	----------	----------	----------	----------	-
Cavia_porcellus	----------	----------	----------	----------	----------	----------	----------	-
Ictidomys_tridecemlineatus	----------	----------	----------	----------	----------	----------	----------	-
Canis	----------	----------	----------	----------	----------	----------	----------	-
Felis_catus	----------	----------	----------	----------	----------	----------	----------	-
Ochotona_princeps	----------	----------	----------	----------	----------	----------	----------	-
Cercopithecidae	----------	----------	----------	----------	----------	----------	----------	-
Carnivora	----------	----------	----------	----------	----------	----------	----------	-
Mus	----------	----------	----------	----------	----------	----------	----------	-
Mus_musculus	----------	----------	----------	----------	----------	----------	----------	-
Rattus_norvegicus	----------	----------	----------	----------	----------	----------	----------	-
Tursiops_truncatus	----------	----------	----------	----------	----------	----------	----------	-
Procavia_capensis	----------	----------	----------	----------	----------	----------	----------	-
Primates	----------	----------	----------	----------	----------	----------	----------	-
Echinops_telfairi	----------	----------	----------	----------	----------	----------	----------	-
Sorex_araneus	----------	----------	----------	----------	----------	----------	----------	-
Canidae	----------	----------	----------	----------	----------	----------	----------	-
Otolemur_garnettii	----------	----------	----------	----------	----------	----------	----------	-
Gallus	----------	----------	----------	----------	----------	----------	----------	-
Callithrix_jacchus	----------	----------	----------	----------	----------	----------	----------	-
Crocodylus_porosus	----------	----------	----------	----------	----------	----------	----------	-
Erinaceus_europaeus	----------	----------	----------	----------	----------	----------	----------	-
rnd-6_family-7426-LTR-ERV1	----------	----------	----------	----------	----------	----------	----------	-
Esox_lucius	----------	----------	----------	----------	----------	----------	----------	-
Salmo_salar	----------	----------	----------	----------	----------	----------	----------	-
Didelphidae	----------	----------	----------	----------	----------	----------	----------	-
Latimeria_chalumnae	----------	----------	----------	----------	----------	----------	----------	-
Monodelphis_domestica	----------	----------	----------	----------	----------	----------	----------	-
Macropus_eugenii	----------	----------	----------	----------	----------	----------	----------	-
Alligator_mississippiensis	----------	----------	----------	----------	----------	----------	----------	-
Chrysemys_picta_bellii	----------	----------	----------	----------	----------	----------	----------	-
Danio_rerio	----------	----------	----------	----------	----------	----------	----------	-
Taeniopygia	----------	----------	----------	----------	----------	----------	----------	-
Dasypus_novemcinctus	----------	----------	----------	----------	----------	----------	----------	-
LTR_XT	----------	----------	----------	----------	----------	----------	----------	-
Haplorrhini	AAGCAAAAAG	GGCTGCAATG	ACAGAAANAA	CAAAGAAAGA	AGAGACTTAT	GCCATGCCCT	TATTAGAGCC	5065
Catarrhini	AAGCTCGAAA	AGCAGCATCT	ACCCCT-TAC	CGGGCATCGG	TAGCAGCCCC	CTTACTCCCT	CA--AACGCC	4546
Anolis_carolinensis	AAGCT----A	GGCAGCAA--	--------AG	CGAACGTTCA	TGAGACAAGC	TCTA--CCTG	CAAGAAGAGT	1099
Murine_leukemia_virus	CGGCCCGAGA	GGCAGCCA--	--------CG	AGGACAGCTC	CAGAAACCTC	CACGCTCCTN	ATAGAGGACT	4499
Phascolarctos_cinereus	CCGCGAAACA	AGCTGCCC--	--------AG	TCAA-----C	CAGAA-----	-----TACTG	ACGGAG----	4376
Estrildidae	----------	----------	----------	----------	----------	----------	----------	-
Xenopus_Silurana_tropicalis	----------	----------	----------	----------	----------	----------	----------	-
I_XT	GGGCNGACAN	ACNNNCTGCN	GTTCTNATNA	CTANCAGNNA	TACCACCTNA	TCNNCTACCT	ACATNAATNN	4588
Takifugu_rubripes	----------	----------	----------	----------	----------	----------	----------	-
Bos_taurus	----------	----------	----------	----------	----------	----------	----------	-
Homo_sapiens	----------	----------	----------	----------	----------	----------	----------	-
Microcebus_murinus	----------	----------	----------	----------	----------	----------	----------	-
Myotis_lucifugus	----------	----------	----------	----------	----------	----------	----------	-
Equus_caballus	----------	----------	----------	----------	----------	----------	----------	-
Tupaia_belangeri	----------	----------	----------	----------	----------	----------	----------	-
Loxodonta_africana	----------	----------	----------	----------	----------	----------	----------	-
Murinae	----------	----------	----------	----------	----------	----------	----------	-
Canis_lupus_familiaris	----------	----------	----------	----------	----------	----------	----------	-
Sus_scrofa	----------	----------	----------	----------	----------	----------	----------	-
Choloepus_hoffmanni	----------	----------	----------	----------	----------	----------	----------	-
Vicugna_pacos	----------	----------	----------	----------	----------	----------	----------	-
Gavialis_gangeticus	----------	----------	----------	----------	----------	----------	----------	-
Mammalia	----------	----------	----------	----------	----------	----------	----------	-
Passeroidea	----------	----------	----------	----------	----------	----------	----------	-
Takifugu	----------	----------	----------	----------	----------	----------	----------	-
Pteropus_vampyrus	----------	----------	----------	----------	----------	----------	----------	-
Ornithorhynchus	----------	----------	----------	----------	----------	----------	----------	-
Oryctolagus_cuniculus	----------	----------	----------	----------	----------	----------	----------	-
Gallus_gallus	----------	----------	----------	----------	----------	----------	----------	-
Hominidae	TTCTGGCAGG	CATGCAGGCG	GCTTCCAATA	AGACGGTCAA	CTTTGACAAA	TTACGGGAGA	TTATTCAAGG	1375
Metatheria	----------	----------	----------	----------	----------	----------	----------	-
Rodentia	----------	----------	----------	----------	----------	----------	----------	-
Monodelphis	----------	----------	----------	----------	----------	----------	----------	-
Consensus	----------	----------	----------	----------	----------	----------	----------	

Conservation

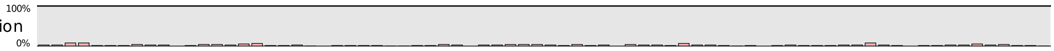

```
                                5,120                          5,140                          5,160                          5,180
                                    |                              |                              |                              |
Dipodomys_ordii              ---------- ---------- ---------- ---------- ---------- ---------- ---------- -
Laurasiatheria               ---------- ---------- ---------- ---------- ---------- ---------- ---------- -
Eutheria                     ---------- ---------- ---------- ---------- ---------- ---------- ---------- -
Tarsius_syrichta             ---------- ---------- ---------- ---------- ---------- ---------- ---------- -
Muridae                      ---------- ---------- ---------- ---------- ---------- ---------- ---------- -
Gasterosteus_aculeatus       ---------- ---------- ---------- ---------- ---------- ---------- ---------- -
Felis_silvestris             ---------- ---------- ---------- ---------- ---------- ---------- ---------- -
Rattus                       ---------- ---------- ---------- ---------- ---------- ---------- ---------- -
Papio_hamadryas              ACACCGGCTT TTTGACATCC CGGTCACCAC CAGCCTCCCT GATGTCTGGT TACAAGATTT TCCCCAAGCT 2493
Pan_troglodytes              ACCCCCTCCT ACCCCCGGCC CCCCCACCCC CCTGCTGCCG GAGGAGCAGC GGGACGCGCC CCGGCCCGCT 561
Ailuropoda_melanoleuca       ---------- ---------- ---------- ---------- ---------- ---------- ---------- -
Cavia_porcellus              ---------- ---------- ---------- ---------- ---------- ---------- ---------- -
Ictidomys_tridecemlineatus   ---------- ---------- ---------- ---------- ---------- ---------- ---------- -
Canis                        ---------- ---------- ---------- ---------- ---------- ---------- ---------- -
Felis_catus                  ---------- ---------- ---------- ---------- ---------- ---------- ---------- -
Ochotona_princeps            ---------- ---------- ---------- ---------- ---------- ---------- ---------- -
Cercopithecidae              ---------- ---------- ---------- ---------- ---------- ---------- ---------- -
Carnivora                    ---------- ---------- ---------- ---------- ---------- ---------- ---------- -
Mus                          ---------- ---------- ---------- ---------- ---------- ---------- ---------- -
Mus_musculus                 ---------- ---------- ---------- ---------- ---------- ---------- ---------- -
Rattus_norvegicus            ---------- ---------- ---------- ---------- ---------- ---------- ---------- -
Tursiops_truncatus           ---------- ---------- ---------- ---------- ---------- ---------- ---------- -
Procavia_capensis            ---------- ---------- ---------- ---------- ---------- ---------- ---------- -
Primates                     ---------- ---------- ---------- ---------- ---------- ---------- ---------- -
Echinops_telfairi            ---------- ---------- ---------- ---------- ---------- ---------- ---------- -
Sorex_araneus                ---------- ---------- ---------- ---------- ---------- ---------- ---------- -
Canidae                      ---------- ---------- ---------- ---------- ---------- ---------- ---------- -
Otolemur_garnettii           ---------- ---------- ---------- ---------- ---------- ---------- ---------- -
Gallus                       ---------- ---------- ---------- ---------- ---------- ---------- ---------- -
Callithrix_jacchus           ---------- ---------- ---------- ---------- ---------- ---------- ---------- -
Crocodylus_porosus           ---------- ---------- ---------- ---------- ---------- ---------- ---------- -
Erinaceus_europaeus          ---------- ---------- ---------- ---------- ---------- ---------- ---------- -
rnd-6_family-7426-LTR-ERV1   ---------- ---------- ---------- ---------- ---------- ---------- ---------- -
Esox_lucius                  ---------- ---------- ---------- ---------- ---------- ---------- ---------- -
Salmo_salar                  ---------- ---------- ---------- ---------- ---------- ---------- ---------- -
Didelphidae                  ---------- ---------- ---------- ---------- ---------- ---------- ---------- -
Latimeria_chalumnae          ---------- ---------- ---------- ---------- ---------- ---------- ---------- -
Monodelphis_domestica        ---------- ---------- ---------- ---------- ---------- ---------- ---------- -
Macropus_eugenii             ---------- ---------- ---------- ---------- ---------- ---------- ---------- -
Alligator_mississippiensis   ---------- ---------- ---------- ---------- ---------- ---------- ---------- -
Chrysemys_picta_bellii       ---------- ---------- ---------- ---------- ---------- ---------- ---------- -
Danio_rerio                  ---------- ---------- ---------- ---------- ---------- ---------- ---------- -
Taeniopygia                  ---------- ---------- ---------- ---------- ---------- ---------- ---------- -
Dasypus_novemcinctus         ---------- ---------- ---------- ---------- ---------- ---------- ---------- -
LTR_XT                       ---------- ---------- ---------- ---------- ---------- ---------- ---------- -
Haplorrhini                  TCCCCTTGCA GANACTCCTA ACTACTC--- -GTCCAGTGA GAAGGCGT-- --GGTTCGCG CAGGAAAACG 5127
Catarrhini                   TGACCTGGTA ------CCTA CCTATTCT-- -AAGGAAGAA AAAGACTT-- --CTTCCACG CAGAAGGGGG 4603
Anolis_carolinensis          GGGCC----- ---------- ---------- ---GAAGGGT GGAGGTTT-- --ATTCCTGG ATTCACCATG 1137
Murine_leukemia_virus        CGACCCCGTA TACGCCCGCC TATTTCCA-- CTACACCGAA ACAGATCTAA AAAGATTACG AGAACTGGGA 4567
Phascolarctos_cinereus       --ACCACGAA AAATCAAGAA CACTTCGAGC CTACACGGGG GAAGATC--- --AAGCCGAG AGAGCT---- 4435
Estrildidae                  ---------- ---------- ---------- ---------- ---------- ---------- ---------- -
Xenopus_Silurana_tropicalis  ---------- ---------- ---------- ---------- ---------- ---------- ---------- -
I_XT                         CTTTNGACTT CATNAAAGGA AGGCAAAAGT AAGGCTTCCA CACAAACCCA TATTAAGTGC ACNACTGAAG 4658
Takifugu_rubripes            ---------- ---------- ---------- ---------- ---------- ---------- ---------- -
Bos_taurus                   ---------- ---------- ---------- ---------- ---------- ---------- ---------- -
Homo_sapiens                 ---------- ---------- ---------- ---------- ---------- ---------- ---------- -
Microcebus_murinus           ---------- ---------- ---------- ---------- ---------- ---------- ---------- -
Myotis_lucifugus             ---------- ---------- ---------- ---------- ---------- ---------- ---------- -
Equus_caballus               ---------- ---------- ---------- ---------- ---------- ---------- ---------- -
Tupaia_belangeri             ---------- ---------- ---------- ---------- ---------- ---------- ---------- -
Loxodonta_africana           ---------- ---------- ---------- ---------- ---------- ---------- ---------- -
Murinae                      ---------- ---------- ---------- ---------- ---------- ---------- ---------- -
Canis_lupus_familiaris       ---------- ---------- ---------- ---------- ---------- ---------- ---------- -
Sus_scrofa                   ---------- ---------- ---------- ---------- ---------- ---------- ---------- -
Choloepus_hoffmanni          ---------- ---------- ---------- ---------- ---------- ---------- ---------- -
Vicugna_pacos                ---------- ---------- ---------- ---------- ---------- ---------- ---------- -
Gavialis_gangeticus          ---------- ---------- ---------- ---------- ---------- ---------- ---------- -
Mammalia                     ---------- ---------- ---------- ---------- ---------- ---------- ---------- -
Passeroidea                  ---------- ---------- ---------- ---------- ---------- ---------- ---------- -
Takifugu                     ---------- ---------- ---------- ---------- ---------- ---------- ---------- -
Pteropus_vampyrus            ---------- ---------- ---------- ---------- ---------- ---------- ---------- -
Ornithorhynchus              ---------- ---------- ---------- ---------- ---------- ---------- ---------- -
Oryctolagus_cuniculus        ---------- ---------- ---------- ---------- ---------- ---------- ---------- -
Gallus_gallus                ---------- ---------- ---------- ---------- ---------- ---------- ---------- -
Hominidae                    GTCTGACGAG AACCCAGCAG TTTTCCTTAA CCGCCTTACT GAGGCCCTCA TCCAGTATAC CCGCCTTGAT 1445
Metatheria                   ---------- ---------- ---------- ---------- ---------- ---------- ---------- -
Rodentia                     ---------- ---------- ---------- ---------- ---------- ---------- ---------- -
Monodelphis                  ---------- ---------- ---------- ---------- ---------- ---------- ---------- -
Consensus                    ---------- ---------- ---------- ---------- ---------- ---------- ----------
```

Conservation (100% / 0%)

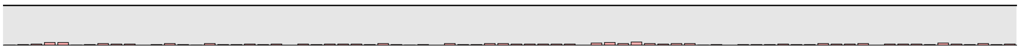

		5,200		5,220		5,240		
Dipodomys_ordii	----------	----------	----------	----------	----------	----------	----------	-
Laurasiatheria	----------	----------	----------	----------	----------	----------	----------	-
Eutheria	----------	----------	----------	----------	----------	----------	----------	-
Tarsius_syrichta	----------	----------	----------	----------	----------	----------	----------	-
Muridae	----------	----------	----------	----------	----------	----------	----------	-
Gasterosteus_aculeatus	----------	----------	----------	----------	----------	----------	----------	-
Felis_silvestris	----------	----------	----------	----------	----------	----------	----------	-
Rattus	----------	----------	----------	----------	----------	----------	----------	-
Papio_hamadryas	TGGGCGGAAA	CGGGAGGACT	TGGGCGGGCC	AAGTGTCAAG	CCCCAATCAT	AATTGATCTA	AAGCCCACA-	2562
Pan_troglodytes	AAACTTGAAC	CCGGGGGACC	--AGCCACTC	CGACGCCCTC	ACCGCCTCAC	GGGGTACGGA	ATGCCCCCGC	629
Ailuropoda_melanoleuca	----------	----------	----------	----------	----------	----------	----------	-
Cavia_porcellus	----------	----------	----------	----------	----------	----------	----------	-
Ictidomys_tridecemlineatus	----------	----------	----------	----------	----------	----------	----------	-
Canis	----------	----------	----------	----------	----------	----------	----------	-
Felis_catus	----------	----------	----------	----------	----------	----------	----------	-
Ochotona_princeps	----------	----------	----------	----------	----------	----------	----------	-
Cercopithecidae	----------	----------	----------	----------	----------	----------	----------	-
Carnivora	----------	----------	----------	----------	----------	----------	----------	-
Mus	----------	----------	----------	----------	----------	----------	----------	-
Mus_musculus	----------	----------	----------	----------	----------	----------	----------	-
Rattus_norvegicus	----------	----------	----------	----------	----------	----------	----------	-
Tursiops_truncatus	----------	----------	----------	----------	----------	----------	----------	-
Procavia_capensis	----------	----------	----------	----------	----------	----------	----------	-
Primates	----------	----------	----------	----------	----------	----------	----------	-
Echinops_telfairi	----------	----------	----------	----------	----------	----------	----------	-
Sorex_araneus	----------	----------	----------	----------	----------	----------	----------	-
Canidae	----------	----------	----------	----------	----------	----------	----------	-
Otolemur_garnettii	----------	----------	----------	----------	----------	----------	----------	-
Gallus	----------	----------	----------	----------	----------	----------	----------	-
Callithrix_jacchus	----------	----------	----------	----------	----------	----------	----------	-
Crocodylus_porosus	----------	----------	----------	----------	----------	----------	----------	-
Erinaceus_europaeus	----------	----------	----------	----------	----------	----------	----------	-
rnd-6_family-7426-LTR-ERV1	----------	----------	----------	----------	----------	----------	----------	-
Esox_lucius	----------	----------	----------	----------	----------	----------	----------	-
Salmo_salar	----------	----------	----------	----------	----------	----------	----------	-
Didelphidae	----------	----------	----------	----------	----------	----------	----------	-
Latimeria_chalumnae	----------	----------	----------	----------	----------	----------	----------	-
Monodelphis_domestica	----------	----------	----------	----------	----------	----------	----------	-
Macropus_eugenii	----------	----------	----------	----------	----------	----------	----------	-
Alligator_mississippiensis	----------	----------	----------	----------	----------	----------	----------	-
Chrysemys_picta_bellii	----------	----------	----------	----TTGCCC	GGAGGCGAAT	GCTGGAGGGA	GGAGGCCGCA	36
Danio_rerio	----------	----------	----------	----------	----------	----------	----------	-
Taeniopygia	----------	----------	----------	----------	----------	----------	----------	-
Dasypus_novemcinctus	----------	----------	----------	----------	----------	----------	----------	-
LTR_XT	----------	----------	----------	----------	----------	----------	----------	-
Haplorrhini	GGAGTTATCA	GAAAGGAGGC	TGGTGGA-AG	TTTTCAGATG	GGAGGCTTGC	CATTCCAGAA	GCCATTGCCC	5196
Catarrhini	GCAAGTAATA	-AAAGGAGGA	TGGATCA-GA	CTGCCAGATG	GGAGGGTAGC	TGTGCCGCAG	TTGCTGGGAG	4671
Anolis_carolinensis	GGTGACTTTA	GCCGCGATGT	TGTAAAA-AC	CTAGTGGATT	CGAATGCC--	----------	----------	1184
Murine_leukemia_virus	GCCGCCTATA	ATCAGATAAA	AGGATATTGG	GTCCTACAAG	GCAAGCCGGT	AATGCCCGAT	CAGTTCGCGT	4637
Phascolarctos_cinereus	--CACCCCTG	ACCAGGGAAG	GGAAT-----	-TCATTCAAC	G---------	---GCTGCAT	CAGCTCACGC	4485
Estrildidae	----------	----------	----------	----------	----------	----------	----------	-
Xenopus_Silurana_tropicalis	----------	----------	----------	----------	----------	----------	----------	-
I_XT	GNTGCAATTG	CAAAATCATG	ATNTTTGNAA	ATATNACAAN	GAAAGTTTGT	ATGCACNNTN	CCATATNCTT	4728
Takifugu_rubripes	----------	----------	----------	----------	----------	----------	----------	-
Bos_taurus	----------	----------	----------	----------	----------	----------	----------	-
Homo_sapiens	----------	----------	----------	----------	----------	----------	----------	-
Microcebus_murinus	----------	----------	----------	----------	----------	----------	----------	-
Myotis_lucifugus	----------	----------	----------	----------	----------	----------	----------	-
Equus_caballus	----------	----------	----------	----------	----------	----------	----------	-
Tupaia_belangeri	----------	----------	----------	----------	----------	----------	----------	-
Loxodonta_africana	----------	----------	----------	----------	----------	----------	----------	-
Murinae	----------	----------	----------	----------	----------	----------	----------	-
Canis_lupus_familiaris	----------	----------	----------	----------	----------	----------	----------	-
Sus_scrofa	----------	----------	----------	----------	----------	----------	----------	-
Choloepus_hoffmanni	----------	----------	----------	----------	----------	----------	----------	-
Vicugna_pacos	----------	----------	----------	----------	----------	----------	----------	-
Gavialis_gangeticus	----------	----------	----------	----------	----------	----------	----------	-
Mammalia	----------	----------	----------	----------	----------	----------	----------	-
Passeroidea	----------	----------	----------	----------	----------	----------	----------	-
Takifugu	----------	----------	----------	----------	----------	----------	----------	-
Pteropus_vampyrus	----------	----------	----------	----------	----------	----------	----------	-
Ornithorhynchus	----------	----------	----------	----------	----------	----------	----------	-
Oryctolagus_cuniculus	----------	----------	----------	----------	----------	----------	----------	-
Gallus_gallus	----------	----------	----------	----------	----------	----------	----------	-
Hominidae	CCCACCTCCC	CGGCAGGGGC	CACTGTCTTG	GCTACTCATT	TCATTTCCCA	ATCAGCGGGA	GATATTCGGA	1515
Metatheria	----------	----------	----------	----------	----------	----------	----------	-
Rodentia	----------	----------	----------	----------	----------	----------	----------	-
Monodelphis	----------	----------	----------	----------	----------	----------	----------	-
Consensus	----------	----------	----------	----------	----------	----------	----------	

Conservation

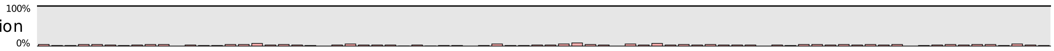

	5,260		5,280		5,300		5,320	
Dipodomys_ordii	----------	----------	----------	----------	----------	----------	----------	-
Laurasiatheria	----------	----------	----------	----------	----------	----------	----------	-
Eutheria	----------	----------	----------	----------	----------	----------	----------	-
Tarsius_syrichta	----------	----------	----------	----------	----------	----------	----------	-
Muridae	----------	----------	----------	----------	----------	----------	----------	-
Gasterosteus_aculeatus	----------	----------	----------	----------	----------	----------	----------	-
Felis_silvestris	----------	----------	----------	----------	----------	----------	----------	-
Rattus	----------	----------	----------	----------	----------	----------	----------	-
Papio_hamadryas	GCGGTGCCTG	TATCTATCAA	ACAATACCCC	ATGAGCCTAG	AAGCTCATAT	GGGCATTCGG	CAACACATTA	2632
Pan_troglodytes	ACAGGACCCA	GGTCCAGTAT	GGCCTTCTCC	ACCGATCTTA	AACTGGAAAC	CAGATCTCTT	TCAGACACCC	699
Ailuropoda_melanoleuca	----------	----------	----------	----------	----------	----------	----------	-
Cavia_porcellus	----------	----------	----------	----------	----------	----------	----------	-
Ictidomys_tridecemlineatus	----------	----------	----------	----------	----------	----------	----------	-
Canis	----------	----------	----------	----------	----------	----------	----------	-
Felis_catus	----------	----------	----------	----------	----------	----------	----------	-
Ochotona_princeps	----------	----------	----------	----------	----------	----------	----------	-
Cercopithecidae	----------	----------	----------	----------	----------	----------	----------	-
Carnivora	----------	----------	----------	----------	----------	----------	----------	-
Mus	----------	----------	----------	----------	----------	----------	----------	-
Mus_musculus	----------	----------	----------	----------	----------	----------	----------	-
Rattus_norvegicus	----------	----------	----------	----------	----------	----------	----------	-
Tursiops_truncatus	----------	----------	----------	----------	----------	----------	----------	-
Procavia_capensis	----------	----------	----------	----------	----------	----------	----------	-
Primates	----------	----------	----------	----------	----------	----------	----------	-
Echinops_telfairi	----------	----------	----------	----------	----------	----------	----------	-
Sorex_araneus	----------	----------	----------	----------	----------	----------	----------	-
Canidae	----------	----------	----------	----------	----------	----------	----------	-
Otolemur_garnettii	----------	----------	----------	----------	----------	----------	----------	-
Gallus	----------	----------	----------	----------	----------	----------	----------	-
Callithrix_jacchus	----------	----------	----------	----------	----------	----------	----------	-
Crocodylus_porosus	----------	----------	----------	----------	----------	----------	----------	-
Erinaceus_europaeus	----------	----------	----------	----------	----------	----------	----------	-
rnd-6_family-7426-LTR-ERV1	----------	----------	----------	----------	----------	----------	----------	-
Esox_lucius	----------	----------	----------	----------	----------	----------	----------	-
Salmo_salar	----------	----------	----------	----------	----------	----------	----------	-
Didelphidae	----------	----------	----------	----------	----------	----------	----------	-
Latimeria_chalumnae	----------	----------	----------	----------	----------	----------	----------	-
Monodelphis_domestica	----------	----------	----------	----------	----------	----------	----------	-
Macropus_eugenii	----------	----------	----------	----------	----------	----------	----------	-
Alligator_mississippiensis	----------	----------	----------	----------	----------	----------	----------	-
Chrysemys_picta_bellii	CGCCGTCTTC	GCCGGACTGT	TGCGGGACAA	GCCGGGATGA	ACGTGGGGAG	CTTGTAGGTG	AACAGTGGAT	106
Danio_rerio	----------	----------	----------	----------	----------	----------	----------	-
Taeniopygia	----------	----------	----------	----------	----------	----------	----------	-
Dasypus_novemcinctus	----------	----------	----------	----------	----------	----------	----------	-
LTR_XT	----------	----------	----------	----------	----------	----------	----------	-
Haplorrhini	CCCGATTTAT	AAAGCAGTTT	CACCAAGGAA	CACACATGGG	GAAGACAGCT	TTAGAGATTC	TCGT------	5260
Catarrhini	CCACAGTCGT	ATTGGCCATG	CACGAAACCA	CTCATCTAGG	NCAAGAGTCA	CTTGAAAAAT	TGTT------	4735
Anolis_carolinensis	----------	----------	----------	----------	----------	----------	----------	1184
Murine_leukemia_virus	TTGAACTATT	AGACTCCCTA	CACAGGCTCA	CCCACCTCAG	CCCTCAAAAG	ATGAAGGCAC	TCCTTGACAG	4707
Phascolarctos_cinereus	ACTTAGGGCC	AGACAAGCTT	CTCCAGCTTG	TAGGTCGCAC	CAGTTTTCAC	ATCCCGAATC	TCC-------	4548
Estrildidae	----------	----------	----------	----------	----------	----------	----------	-
Xenopus_Silurana_tropicalis	----------	----------	----------	----------	----------	----------	----------	-
I_XT	CNCNTTGTNN	GCAAAACATC	ACGCATCNCC	NATCTCANAT	TTCNAAAAGG	GGGGGATGGT	CNTCATNGAA	4798
Takifugu_rubripes	----------	----------	----------	----------	----------	----------	----------	-
Bos_taurus	----------	----------	----------	----------	----------	----------	----------	-
Homo_sapiens	----------	----------	----------	----------	----------	----------	----------	-
Microcebus_murinus	----------	----------	----------	----------	----------	----------	----------	-
Myotis_lucifugus	----------	----------	----------	----------	----------	----------	----------	-
Equus_caballus	----------	----------	----------	----------	----------	----------	----------	-
Tupaia_belangeri	----------	----------	----------	----------	----------	----------	----------	-
Loxodonta_africana	----------	----------	----------	----------	----------	----------	----------	-
Murinae	----------	----------	----------	----------	----------	----------	----------	-
Canis_lupus_familiaris	----------	----------	----------	----------	----------	----------	----------	-
Sus_scrofa	----------	----------	----------	----------	----------	----------	----------	-
Choloepus_hoffmanni	----------	----------	----------	----------	----------	----------	----------	-
Vicugna_pacos	----------	----------	----------	----------	----------	----------	----------	-
Gavialis_gangeticus	----------	----------	----------	----------	----------	----------	----------	-
Mammalia	----------	----------	----------	----------	----------	----------	----------	-
Passeroidea	----------	----------	----------	----------	----------	----------	----------	-
Takifugu	----------	----------	----------	----------	----------	----------	----------	-
Pteropus_vampyrus	----------	----------	----------	----------	----------	----------	----------	-
Ornithorhynchus	----------	----------	----------	----------	----------	----------	----------	-
Oryctolagus_cuniculus	----------	----------	----------	----------	----------	----------	----------	-
Gallus_gallus	----------	----------	----------	----------	----------	----------	----------	-
Hominidae	AAAAACTAAA	AAAGGCGGAG	GAAGGCCCTC	AAACCCCAAT	ACAGGACCTA	GTTAAAATGG	CCTTCAGGGT	1585
Metatheria	----------	----------	----------	----------	----------	----------	----------	-
Rodentia	----------	----------	----------	----------	----------	----------	----------	-
Monodelphis	----------	----------	----------	----------	----------	----------	----------	-
Consensus	----------	----------	----------	----------	----------	----------	----------	

Conservation 100% 0%

5,340 5,360 5,380

Dipodomys_ordii	----------	----------	----------	----------	----------	----------	----------	-
Laurasiatheria	----------	----------	----------	----------	----------	----------	----------	-
Eutheria	----------	----------	----------	----------	----------	----------	----------	-
Tarsius_syrichta	----------	----------	----------	----------	----------	----------	----------	-
Muridae	----------	----------	----------	----------	----------	----------	----------	-
Gasterosteus_aculeatus	----------	----------	----------	----------	----------	----------	----------	-
Felis_silvestris	----------	----------	----------	----------	----------	----------	----------	-
Rattus	----------	----------	----------	----------	----------	----------	----------	-
Papio_hamadryas	TCAAATTTCT	AGAACTTGGA	GTTTTGCGAC	CTTGTCGCTC	ACCCTGGAAT	ACTCCTCTTC	TGCCAGTAAA	2702
Pan_troglodytes	AGTAGCTTAT	AGATTCTGTC	ACCACCACCC	ACTTGGGATG	ATGTCACAGC	TCTCCTTTTT	TACCAC--GA	767
Ailuropoda_melanoleuca	----------	----------	----------	----------	----------	----------	----------	-
Cavia_porcellus	----------	----------	----------	----------	----------	----------	----------	-
Ictidomys_tridecemlineatus	----------	----------	----------	----------	----------	----------	----------	-
Canis	----------	----------	----------	----------	----------	----------	----------	-
Felis_catus	----------	----------	----------	----------	----------	----------	----------	-
Ochotona_princeps	----------	----------	----------	----------	----------	----------	----------	-
Cercopithecidae	----------	----------	----------	----------	----------	----------	----------	-
Carnivora	----------	----------	----------	----------	----------	----------	----------	-
Mus	----------	----------	----------	----------	----------	----------	----------	-
Mus_musculus	----------	----------	----------	----------	----------	----------	----------	-
Rattus_norvegicus	----------	----------	----------	----------	----------	----------	----------	-
Tursiops_truncatus	----------	----------	----------	----------	----------	----------	----------	-
Procavia_capensis	----------	----------	----------	----------	----------	----------	----------	-
Primates	----------	----------	----------	----------	----------	----------	----------	-
Echinops_telfairi	----------	----------	----------	----------	----------	----------	----------	-
Sorex_araneus	----------	----------	----------	----------	----------	----------	----------	-
Canidae	----------	----------	----------	----------	----------	----------	----------	-
Otolemur_garnettii	----------	----------	----------	----------	----------	----------	----------	-
Gallus	----------	----------	----------	----------	----------	----------	----------	-
Callithrix_jacchus	----------	----------	----------	----------	----------	----------	----------	-
Crocodylus_porosus	----------	----------	----------	----------	----------	----------	----------	-
Erinaceus_europaeus	----------	----------	----------	----------	----------	----------	----------	-
rnd-6_family-7426-LTR-ERV1	----------	----------	----------	----------	----------	----------	----------	-
Esox_lucius	----------	----------	----------	----------	----------	----------	----------	-
Salmo_salar	----------	----------	----------	----------	----------	----------	----------	-
Didelphidae	----------	----------	----------	----------	----------	----------	----------	-
Latimeria_chalumnae	----------	----------	----------	----------	----------	----------	----------	-
Monodelphis_domestica	----------	----------	----------	----------	----------	----------	----------	-
Macropus_eugenii	----------	----------	----------	----------	----------	----------	----------	-
Alligator_mississippiensis	----------	----------	----------	----------	----------	----------	----------	-
Chrysemys_picta_bellii	TCTTTTTACG	GGGACCAGTG	CCAGATGGAG	ACAGAGTACC	AGGAATCCAA	ACTCTGGACG	GAAGTGTTGT	176
Danio_rerio	----------	----------	----------	----------	----------	----------	----------	-
Taeniopygia	----------	----------	----------	----------	----------	----------	----------	-
Dasypus_novemcinctus	----------	----------	----------	----------	----------	----------	----------	-
LTR_XT	----------	----------	----------	----------	----------	----------	----------	-
Haplorrhini	---AGGGCGG	TATTTCTNTG	TGCCACGCCT	AACTGCCATC	ACTCGAGCCA	TCTGCGAGCA	GTGTGTTACT	5327
Catarrhini	---AGGCCGG	TACTTCTACG	TCTCACACTT	GCCAGCCCTT	GCCAAAGCAG	TAGCACAACG	GTGCGTTACT	4802
Anolis_carolinensis	----------	----------	----------	----------	----------	----------	----------	1184
Murine_leukemia_virus	AGAAGAAAGC	CCCTACTACA	TGTTAAACCG	GGACAGAACT	CTCCAGTACG	TGGCGGAATC	CTGCACAGCT	4777
Phascolarctos_cinereus	-------AGT	CTGTAGTGCG	TG--------	------AAAT	CACCAGCAAG	TGTCAG----	-------GTT	4586
Estrildidae	----------	----------	----------	----------	----------	----------	----------	-
Xenopus_Silurana_tropicalis	----------	----------	----------	----------	----------	----------	----------	-
I_XT	ACACTAANTA	TGGTTTGCTC	CTGGTATTNG	CGCTNGGANC	NANAGATNNC	TATANTAAAN	NNATNANGTN	4868
Takifugu_rubripes	----------	----------	----------	----------	----------	----------	----------	-
Bos_taurus	----------	----------	----------	----------	----------	----------	----------	-
Homo_sapiens	----------	----------	----------	----------	----------	----------	----------	-
Microcebus_murinus	----------	----------	----------	----------	----------	----------	----------	-
Myotis_lucifugus	----------	----------	----------	----------	----------	----------	----------	-
Equus_caballus	----------	----------	----------	----------	----------	----------	----------	-
Tupaia_belangeri	----------	----------	----------	----------	----------	----------	----------	-
Loxodonta_africana	----------	----------	----------	----------	----------	----------	----------	-
Murinae	----------	----------	----------	----------	----------	----------	----------	-
Canis_lupus_familiaris	----------	----------	----------	----------	----------	----------	----------	-
Sus_scrofa	----------	----------	----------	----------	----------	----------	----------	-
Choloepus_hoffmanni	----------	----------	----------	----------	----------	----------	----------	-
Vicugna_pacos	----------	----------	----------	----------	----------	----------	----------	-
Gavialis_gangeticus	----------	----------	----------	----------	----------	----------	----------	-
Mammalia	----------	----------	----------	----------	----------	----------	----------	-
Passeroidea	----------	----------	----------	----------	----------	----------	----------	-
Takifugu	----------	----------	----------	----------	----------	----------	----------	-
Pteropus_vampyrus	----------	----------	----------	----------	----------	----------	----------	-
Ornithorhynchus	----------	----------	----------	----------	----------	----------	----------	-
Oryctolagus_cuniculus	----------	----------	----------	----------	----------	----------	----------	-
Gallus_gallus	----------	----------	----------	----------	----------	----------	----------	-
Hominidae	CTATAATTCC	AGGGAGGAGA	CGGCTGAGGC	CCAAAGACAG	GCAAGGCTAA	AGCAGAAGGT	ACAGCTCCAG	1655
Metatheria	----------	----------	----------	----------	----------	----------	----------	-
Rodentia	----------	----------	----------	----------	----------	----------	----------	-
Monodelphis	----------	----------	----------	----------	----------	----------	----------	-
Consensus	----------	----------	----------	----------	----------	----------	----------	

Conservation

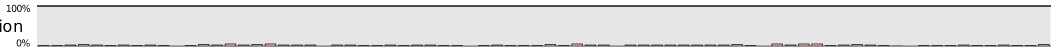

5,400 5,420 5,440 5,460

Dipodomys_ordii	----------	----------	----------	----------	----------	----------	----------	-
Laurasiatheria	----------	----------	----------	----------	----------	----------	----------	-
Eutheria	----------	----------	----------	----------	----------	----------	----------	-
Tarsius_syrichta	----------	----------	----------	----------	----------	----------	----------	-
Muridae	----------	----------	----------	----------	----------	----------	----------	-
Gasterosteus_aculeatus	----------	----------	----------	----------	----------	----------	----------	-
Felis_silvestris	----------	----------	----------	----------	----------	----------	----------	-
Rattus	----------	----------	----------	----------	----------	----------	----------	-
Papio_hamadryas	AAAGCCTGGT	ACCCAGGATT	ACAGGCCCGT	CCAAGATTTG	AGAGAAATTA	ATAAAAGAAC	TGTGGATATC	2772
Pan_troglodytes	AGAGGAAGGA	ATCTGAAGCT	GAACTGTCCC	GGGGGGACGG	CCCGACCCCC	ACCATAAGAA	TTTCCTCTAC	837
Ailuropoda_melanoleuca	----------	----------	----------	----------	----------	----------	----------	-
Cavia_porcellus	----------	----------	----------	----------	----------	----------	----------	-
Ictidomys_tridecemlineatus	----------	----------	----------	----------	----------	----------	----------	-
Canis	----------	----------	----------	----------	----------	----------	----------	-
Felis_catus	----------	----------	----------	----------	----------	----------	----------	-
Ochotona_princeps	----------	----------	----------	----------	----------	----------	----------	-
Cercopithecidae	----------	----------	----------	----------	----------	----------	----------	-
Carnivora	----------	----------	----------	----------	----------	----------	----------	-
Mus	----------	----------	----------	----------	----------	----------	----------	-
Mus_musculus	----------	----------	----------	----------	----------	----------	----------	-
Rattus_norvegicus	----------	----------	----------	----------	----------	----------	----------	-
Tursiops_truncatus	----------	----------	----------	----------	----------	----------	----------	-
Procavia_capensis	----------	----------	----------	----------	----------	----------	----------	-
Primates	----------	----------	----------	----------	----------	----------	----------	-
Echinops_telfairi	----------	----------	----------	----------	----------	----------	----------	-
Sorex_araneus	----------	----------	----------	----------	----------	----------	----------	-
Canidae	----------	----------	----------	----------	----------	----------	----------	-
Otolemur_garnettii	----------	----------	----------	----------	----------	----------	----------	-
Gallus	----------	----------	----------	----------	----------	----------	----------	-
Callithrix_jacchus	----------	----------	----------	----------	----------	----------	----------	-
Crocodylus_porosus	----------	----------	----------	----------	----------	----------	----------	-
Erinaceus_europaeus	----------	----------	----------	----------	----------	----------	----------	-
rnd-6_family-7426-LTR-ERV1	----------	----------	----------	----------	----------	----------	----------	-
Esox_lucius	----------	----------	----------	----------	----------	----------	----------	-
Salmo_salar	----------	----------	----------	----------	----------	----------	----------	-
Didelphidae	----------	----------	----------	----------	----------	----------	----------	-
Latimeria_chalumnae	----------	----------	----------	----------	----------	----------	----------	-
Monodelphis_domestica	----------	----------	----------	----------	----------	----------	----------	-
Macropus_eugenii	----------	----------	----------	----------	----------	----------	----------	-
Alligator_mississippiensis	----------	----------	----------	----------	----------	----------	----------	-
Chrysemys_picta_bellii	CTGAAAGAAA	AACGAGGCAC	TGAGAGCCAG	AGGATCTATT	AAACACCTCT	TTGTTTAGCA	AACCTTATCA	246
Danio_rerio	----------	----------	----------	----------	----------	----------	----------	-
Taeniopygia	----------	----------	----------	----------	----------	----------	----------	-
Dasypus_novemcinctus	----------	----------	----------	----------	----------	----------	----------	-
LTR_XT	----------	----------	----------	----------	----------	----------	----------	-
Haplorrhini	TGTGCCCAGA	ATAACCCAAG	GCAAGGGCCT	ACTCGGCCCC	CAGGAATTCA	GGAAACAGGA	GCAGTGCCAT	5397
Catarrhini	TGCCGACAGC	ACAATGCGAG	GCAAGGCCCC	ACTGTTCCGC	CCGGCATACA	AGCTTATGGA	GCGGCTCCTT	4872
Anolis_carolinensis	----------	----------	----------	----------	----------	----------	----------	1184
Murine_leukemia_virus	TGTGCTCAAG	TGAATGCTAG	TAAAG---CC	AAAATCGGGG	CAGGGGTACG	AGTACGCGGA	CATCGACCAG	4844
Phascolarctos_cinereus	TGTGCTGTAA	CTAACGCAGT	CACCA---CC	TACCGAGAGC	CTGGAAGGAG	GCAAAGGGGA	GATCGACCCG	4653
Estrildidae	----------	----------	----------	----------	----------	----------	----------	-
Xenopus_Silurana_tropicalis	----------	----------	----------	----------	----------	----------	----------	-
I_XT	CNGTAACAAC	ACANNTNCTA	ANNCNNACAA	AGCCAATTTA	NAACANACCT	AGTCAAAACN	TTTTATNGNT	4938
Takifugu_rubripes	----------	----------	----------	----------	----------	----------	----------	-
Bos_taurus	----------	----------	----------	----------	----------	----------	----------	-
Homo_sapiens	----------	----------	----------	----------	----------	----------	----------	-
Microcebus_murinus	----------	----------	----------	----------	----------	----------	----------	-
Myotis_lucifugus	----------	----------	----------	----------	----------	----------	----------	-
Equus_caballus	----------	----------	----------	----------	----------	----------	----------	-
Tupaia_belangeri	----------	----------	----------	----------	----------	----------	----------	-
Loxodonta_africana	----------	----------	----------	----------	----------	----------	----------	-
Murinae	----------	----------	----------	----------	----------	----------	----------	-
Canis_lupus_familiaris	----------	----------	----------	----------	----------	----------	----------	-
Sus_scrofa	----------	----------	----------	----------	----------	----------	----------	-
Choloepus_hoffmanni	----------	----------	----------	----------	----------	----------	----------	-
Vicugna_pacos	----------	----------	----------	----------	----------	----------	----------	-
Gavialis_gangeticus	----------	----------	----------	----------	----------	----------	----------	-
Mammalia	----------	----------	----------	----------	----------	----------	----------	-
Passeroidea	----------	----------	----------	----------	----------	----------	----------	-
Takifugu	----------	----------	----------	----------	----------	----------	----------	-
Pteropus_vampyrus	----------	----------	----------	----------	----------	----------	----------	-
Ornithorhynchus	----------	----------	----------	----------	----------	----------	----------	-
Oryctolagus_cuniculus	----------	----------	----------	----------	----------	----------	----------	-
Gallus_gallus	----------	----------	----------	----------	----------	----------	----------	-
Hominidae	ACCCAGGCCT	TGGTAGCTGC	CCCGCGGCTG	GCCGGCTCCG	GGAGCCAACC	GAAAGGGGGT	TCCGGCCACC	1725
Metatheria	----------	----------	----------	----------	----------	----------	----------	-
Rodentia	----------	----------	----------	----------	----------	----------	----------	-
Monodelphis	----------	----------	----------	----------	----------	----------	----------	-
Consensus	----------	----------	----------	----------	----------	----------	----------	

Conservation 100% 0%

		5,480		5,500		5,520		
Dipodomys_ordii	----------	----------	----------	----------	----------	----------	----------	-
Laurasiatheria	----------	----------	----------	----------	----------	----------	----------	-
Eutheria	----------	----------	----------	----------	----------	----------	----------	-
Tarsius_syrichta	----------	----------	----------	----------	----------	----------	----------	-
Muridae	----------	----------	----------	----------	----------	----------	----------	-
Gasterosteus_aculeatus	----------	----------	----------	----------	----------	----------	----------	-
Felis_silvestris	----------	----------	----------	----------	----------	----------	----------	-
Rattus	----------	----------	----------	----------	----------	----------	----------	-
Papio_hamadryas	CACCCTACGG	TCCCCAATCC	TTA---CAAC	TTGCTCAGTA	CCTTAAAACC	AGACTACAGC	T-----GGTA	2834
Pan_troglodytes	CGCCGGGTGG	GACTAAACAC	GCAAGGTAGG	GAGCTCATTA	TCGCCAGCTC	TAATGGCAGG	TCTCGAGCGC	907
Ailuropoda_melanoleuca	----------	----------	----------	----------	----------	----------	----------	-
Cavia_porcellus	----------	----------	----------	----------	----------	----------	----------	-
Ictidomys_tridecemlineatus	----------	----------	----------	----------	----------	----------	----------	-
Canis	----------	----------	----------	----------	----------	----------	----------	-
Felis_catus	----------	----------	----------	----------	----------	----------	----------	-
Ochotona_princeps	----------	----------	----------	----------	----------	----------	----------	-
Cercopithecidae	----------	----------	----------	----------	----------	----------	----------	-
Carnivora	----------	----------	----------	----------	----------	----------	----------	-
Mus	----------	----------	----------	----------	----------	----------	----------	-
Mus_musculus	----------	----------	----------	----------	----------	----------	----------	-
Rattus_norvegicus	----------	----------	----------	----------	----------	----------	----------	-
Tursiops_truncatus	----------	----------	----------	----------	----------	----------	----------	-
Procavia_capensis	----------	----------	----------	----------	----------	----------	----------	-
Primates	----------	----------	----------	----------	----------	----------	----------	-
Echinops_telfairi	----------	----------	----------	----------	----------	----------	----------	-
Sorex_araneus	----------	----------	----------	----------	----------	----------	----------	-
Canidae	----------	----------	----------	----------	----------	----------	----------	-
Otolemur_garnettii	----------	----------	----------	----------	----------	----------	----------	-
Gallus	----------	----------	----------	----------	----------	----------	----------	-
Callithrix_jacchus	----------	----------	----------	----------	----------	----------	----------	-
Crocodylus_porosus	----------	----------	----------	----------	----------	----------	----------	-
Erinaceus_europaeus	----------	----------	----------	----------	----------	----------	----------	-
rnd-6_family-7426-LTR-ERV1	----------	----------	----------	----------	----------	----------	----------	-
Esox_lucius	----------	----------	----------	----------	----------	----------	----------	-
Salmo_salar	----------	----------	----------	----------	----------	----------	----------	-
Didelphidae	----------	----------	----------	----------	----------	----------	----------	-
Latimeria_chalumnae	----------	----------	----------	----------	----------	----------	----------	-
Monodelphis_domestica	----------	----------	----------	----------	----------	----------	----------	-
Macropus_eugenii	----------	----------	----------	----------	----------	----------	----------	-
Alligator_mississippiensis	----------	----------	----------	----------	----------	----------	----------	-
Chrysemys_picta_bellii	TTATGAAAGA	TTTGATGATT	CTCGTCGGAC	GTCCCGCCCC	CCGTGTTGGG	TCCCCGGCCC	CACCCACAGG	316
Danio_rerio	----------	----------	----------	----------	----------	----------	----------	-
Taeniopygia	----------	----------	----------	----------	----------	----------	----------	-
Dasypus_novemcinctus	----------	----------	----------	----------	----------	----------	----------	-
LTR_XT	----------	----------	----------	----------	----------	----------	----------	-
Haplorrhini	GTGAAAACCT	GCTTGTAGAC	TTTACCGAAC	T---GCCTCA	GGCTGGAGGC	TATCGGTACA	TGTTAGTGTT	5464
Catarrhini	TTGAGGATCT	TCAGGTGGAT	TTCACAGAAA	T---GCCGAA	ATGTGGAGGT	AACAAGTATT	TGCTGGTTCT	4939
Anolis_carolinensis	----------	----------	----------	----------	----------	----------	----------	1184
Murine_leukemia_virus	GTACCCATTG	GGAAATCGAC	TTTACCGAAG	TTAAGCCAGG	GCTGTACGGG	TACAAGTACC	TCCTGGTGTT	4914
Phascolarctos_cinereus	GCGTGTATTG	GGAAGTAGAC	TTCACAGAGG	TTAAGCCTGG	CCGGTATGGT	AATAGATATT	TGCTAGTGTT	4723
Estrildidae	----------	----------	----------	----------	----------	----------	----------	-
Xenopus_Silurana_tropicalis	----------	----------	----------	----------	----------	----------	----------	-
I_XT	TNNNGAGGAT	TAANNTAGAT	TGTATNCANN	TANCCAAATG	TACTNCNTTA	TTTANTNGTA	NACANNAANA	5008
Takifugu_rubripes	----------	----------	----------	----------	----------	----------	----------	-
Bos_taurus	----------	----------	----------	----------	----------	----------	----------	-
Homo_sapiens	----------	----------	----------	----------	----------	----------	----------	-
Microcebus_murinus	----------	----------	----------	----------	----------	----------	----------	-
Myotis_lucifugus	----------	----------	----------	----------	----------	----------	----------	-
Equus_caballus	----------	----------	----------	----------	----------	----------	----------	-
Tupaia_belangeri	----------	----------	----------	----------	----------	----------	----------	-
Loxodonta_africana	----------	----------	----------	----------	----------	----------	----------	-
Murinae	----------	----------	----------	----------	----------	----------	----------	-
Canis_lupus_familiaris	----------	----------	----------	----------	----------	----------	----------	-
Sus_scrofa	----------	----------	----------	----------	----------	----------	----------	-
Choloepus_hoffmanni	----------	----------	----------	----------	----------	----------	----------	-
Vicugna_pacos	----------	----------	----------	----------	----------	----------	----------	-
Gavialis_gangeticus	----------	----------	----------	----------	----------	----------	----------	-
Mammalia	----------	----------	----------	----------	----------	----------	----------	-
Passeroidea	----------	----------	----------	----------	----------	----------	----------	-
Takifugu	----------	----------	----------	----------	----------	----------	----------	-
Pteropus_vampyrus	----------	----------	----------	----------	----------	----------	----------	-
Ornithorhynchus	----------	----------	----------	----------	----------	----------	----------	-
Oryctolagus_cuniculus	----------	----------	----------	----------	----------	----------	----------	-
Gallus_gallus	----------	----------	----------	----------	----------	----------	----------	-
Hominidae	GAGCGCCACC	TGGTGCCTGC	TTCAAGTGTG	GGAACGAAGG	CCACTGGGCC	TGACAATGCC	CGTACCCGAA	1795
Metatheria	----------	----------	----------	----------	----------	----------	----------	-
Rodentia	----------	----------	----------	----------	----------	----------	----------	-
Monodelphis	----------	----------	----------	----------	----------	----------	----------	-
Consensus	----------	----------	----------	----------	----------	----------	----------	

Conservation

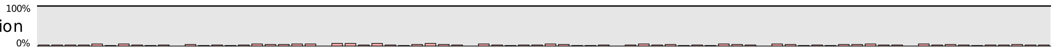

5,540 5,560 5,580 5,600

Dipodomys_ordii	----------	----------	----------	----------	----------	----------	----------	-
Laurasiatheria	----------	----------	----------	----------	----------	----------	----------	-
Eutheria	----------	----------	----------	----------	----------	----------	----------	-
Tarsius_syrichta	----------	----------	----------	----------	----------	----------	----------	-
Muridae	----------	----------	----------	----------	----------	----------	----------	-
Gasterosteus_aculeatus	----------	----------	----------	----------	----------	----------	----------	-
Felis_silvestris	----------	----------	----------	----------	----------	----------	----------	-
Rattus	----------	----------	----------	----------	----------	----------	----------	-
Papio_hamadryas	TACCGTACTG	GACTTAAAAG	ATGCCTTCTT	TTGTTTACCC	CTGGCCCCCC	AAAGCCAGGA	ACTATTTGCC	2904
Pan_troglodytes	CCCCACAATT	TGGCTAAAGT	ATCTATTCGC	AGGGAAAGAG	AAGCCCGCAC	CTCTTAGAAA	GATAATGGAA	977
Ailuropoda_melanoleuca	----------	----------	----------	----------	----------	----------	----------	-
Cavia_porcellus	----------	----------	----------	----------	----------	----------	----------	-
Ictidomys_tridecemlineatus	----------	----------	----------	----------	----------	----------	----------	-
Canis	----------	----------	----------	----------	----------	----------	----------	-
Felis_catus	----------	----------	----------	----------	----------	----------	----------	-
Ochotona_princeps	----------	----------	----------	----------	----------	----------	----------	-
Cercopithecidae	----------	----------	----------	----------	----------	----------	----------	-
Carnivora	----------	----------	----------	----------	----------	----------	----------	-
Mus	----------	----------	----------	----------	----------	----------	----------	-
Mus_musculus	----------	----------	----------	----------	----------	----------	----------	-
Rattus_norvegicus	----------	----------	----------	----------	----------	----------	----------	-
Tursiops_truncatus	----------	----------	----------	----------	----------	----------	----------	-
Procavia_capensis	----------	----------	----------	----------	----------	----------	----------	-
Primates	----------	----------	----------	----------	----------	----------	----------	-
Echinops_telfairi	----------	----------	----------	----------	----------	----------	----------	-
Sorex_araneus	----------	----------	----------	----------	----------	----------	----------	-
Canidae	----------	----------	----------	----------	----------	----------	----------	-
Otolemur_garnettii	----------	----------	----------	----------	----------	----------	----------	-
Gallus	----------	----------	----------	----------	----------	----------	----------	-
Callithrix_jacchus	----------	----------	----------	----------	----------	----------	----------	-
Crocodylus_porosus	----------	----------	----------	----------	----------	----------	----------	-
Erinaceus_europaeus	----------	----------	----------	----------	----------	----------	----------	-
rnd-6_family-7426-LTR-ERV1	----------	----------	----------	----------	----------	----------	----------	-
Esox_lucius	----------	----------	----------	----------	----------	----------	----------	-
Salmo_salar	----------	----------	----------	----------	----------	----------	----------	-
Didelphidae	----------	----------	----------	----------	----------	----------	----------	-
Latimeria_chalumnae	----------	----------	----------	----------	----------	----------	----------	-
Monodelphis_domestica	----------	----------	----------	----------	----------	----------	----------	-
Macropus_eugenii	----------	----------	----------	----------	----------	----------	----------	-
Alligator_mississippiensis	----------	----------	----------	----------	----------	----------	----------	-
Chrysemys_picta_bellii	CCTCCCCGGG	TCCTATACTG	AGTCCTCCAT	CGCCCTCGAT	CGTTCTTCAG	CTACTAGGCA	GGAATTCGCA	386
Danio_rerio	----------	----------	----------	----------	----------	----------	----------	-
Taeniopygia	----------	----------	----------	----------	----------	----------	----------	-
Dasypus_novemcinctus	----------	----------	----------	----------	----------	----------	----------	-
LTR_XT	----------	----------	----------	----------	----------	----------	----------	-
Haplorrhini	TGTCTGCACC	TTTTCAGGGT	GGGTCGAGGC	ATTCCCCACC	AGGACAGAAA	AGGCACGAGA	AGTAACCAGG	5534
Catarrhini	TGTGTGTACT	TACTCTGGGT	GGGTGGAGGC	TTATCCAACA	CGAACTGAAA	AGGCCTACGA	GGTAACCCGT	5009
Anolis_carolinensis	----------	----------	----------	----------	----------	----------	----------	1184
Murine_leukemia_virus	CGTGGACACC	TTCTCTGGCT	GGGTAGAAGC	CTTCCCAACT	AAACGTGAAA	CCGCCAAGGT	TGTAACCAAG	4984
Phascolarctos_cinereus	TATCGATACA	TTTTCTGGGT	GGGTAGAAGC	TTTCCCTACA	AAAACAGAGA	CAGCCCTGAC	CGTCTGCAAG	4793
Estrildidae	----------	----------	----------	----------	----------	----------	----------	-
Xenopus_Silurana_tropicalis	----------	----------	----------	----------	----------	----------	----------	-
I_XT	TATTTTGGAT	GGNTANAGGC	ATGNCCATTT	ACAAAAGCNA	TCGNACTAAT	NANNGCTAAG	GAATANTCNG	5078
Takifugu_rubripes	----------	----------	----------	----------	----------	----------	----------	-
Bos_taurus	----------	----------	----------	----------	----------	----------	----------	-
Homo_sapiens	----------	----------	----------	----------	----------	----------	----------	-
Microcebus_murinus	----------	----------	----------	----------	----------	----------	----------	-
Myotis_lucifugus	----------	----------	----------	----------	----------	----------	----------	-
Equus_caballus	----------	----------	----------	----------	----------	----------	----------	-
Tupaia_belangeri	----------	----------	----------	----------	----------	----------	----------	-
Loxodonta_africana	----------	----------	----------	----------	----------	----------	----------	-
Murinae	----------	----------	----------	----------	----------	----------	----------	-
Canis_lupus_familiaris	----------	----------	----------	----------	----------	----------	----------	-
Sus_scrofa	----------	----------	----------	----------	----------	----------	----------	-
Choloepus_hoffmanni	----------	----------	----------	----------	----------	----------	----------	-
Vicugna_pacos	----------	----------	----------	----------	----------	----------	----------	-
Gavialis_gangeticus	----------	----------	----------	----------	----------	----------	----------	-
Mammalia	----------	----------	----------	----------	----------	----------	----------	-
Passeroidea	----------	----------	----------	----------	----------	----------	----------	-
Takifugu	----------	----------	----------	----------	----------	----------	----------	-
Pteropus_vampyrus	----------	----------	----------	----------	----------	----------	----------	-
Ornithorhynchus	----------	----------	----------	----------	----------	----------	----------	-
Oryctolagus_cuniculus	----------	----------	----------	----------	----------	----------	----------	-
Gallus_gallus	----------	----------	----------	----------	----------	----------	----------	-
Hominidae	GGAACCGACC	CGACCATGCC	CTAACTGCCA	CCAGATGGGA	CATTGGAAGT	CTGAGTGCCC	CAGCGTCGGA	1865
Metatheria	----------	----------	----------	----------	----------	----------	----------	-
Rodentia	----------	----------	----------	----------	----------	----------	----------	-
Monodelphis	----------	----------	----------	----------	----------	----------	----------	-
Consensus	----------	----------	----------	----------	----------	----------	----------	

Conservation 100% 0%

	5,620		5,640		5,660			
Dipodomys_ordii	----------	----------	----------	----------	----------	----------	----------	-
Laurasiatheria	----------	----------	----------	----------	----------	----------	----------	-
Eutheria	----------	----------	----------	----------	----------	----------	----------	-
Tarsius_syrichta	----------	----------	----------	----------	----------	----------	----------	-
Muridae	----------	----------	----------	----------	----------	----------	--TTTCTGGA	8
Gasterosteus_aculeatus	----------	----------	----------	----------	----------	----------	----------	-
Felis_silvestris	----------	----------	----------	----------	----------	----------	----------	-
Rattus	----------	----------	----------	----------	----------	----------	----------	-
Papio_hamadryas	TTTGAGTGGA	AGGACCCTGA	GAGAGGAATC	TCAGGCCA--	ATTGACCTGG	ACTCGACTTC	CCCAGGGGTT	2972
Pan_troglodytes	GTTTAGAAGT	ACACCCTAGA	TCCAGAGGCT	CCAGGCAGGC	ACGTGAATGT	CTTTCGTAAA	TCAGGCAGCC	1047
Ailuropoda_melanoleuca	----------	----------	----------	----------	----------	----------	----------	-
Cavia_porcellus	----------	----------	----------	----------	----------	----------	----------	-
Ictidomys_tridecemlineatus	----------	----------	----------	----------	----------	----------	----------	-
Canis	----------	----------	----------	----------	----------	----------	----------	-
Felis_catus	----------	----------	----------	----------	----------	----------	----------	-
Ochotona_princeps	----------	----------	----------	----------	----------	----------	----------	-
Cercopithecidae	----------	----------	----------	----------	----------	----------	----------	-
Carnivora	----------	----------	----------	----------	----------	----------	----------	-
Mus	----------	----------	----------	----------	----------	----------	----------	-
Mus_musculus	----------	----------	----------	----------	----------	----------	----------	-
Rattus_norvegicus	----------	----------	----------	----------	----------	----------	----------	-
Tursiops_truncatus	----------	----------	----------	----------	----------	----------	----------	-
Procavia_capensis	----------	----------	----------	----------	----------	----------	----------	-
Primates	----------	----------	----------	----------	----------	----------	----------	-
Echinops_telfairi	----------	----------	----------	----------	----------	----------	----------	-
Sorex_araneus	----------	----------	----------	----------	----------	----------	----------	-
Canidae	----------	----------	----------	----------	----------	----------	----------	-
Otolemur_garnettii	----------	----------	----------	----------	----------	----------	----------	-
Gallus	----------	----------	----------	----------	----------	----------	----------	-
Callithrix_jacchus	----------	----------	----------	----------	----------	----------	----------	-
Crocodylus_porosus	----------	----------	----------	----------	----------	----------	----------	-
Erinaceus_europaeus	----------	----------	----------	----------	----------	----------	----------	-
rnd-6_family-7426-LTR-ERV1	----------	----------	----------	----------	----------	----------	----------	-
Esox_lucius	----------	----------	----------	----------	----------	----------	----------	-
Salmo_salar	----------	----------	----------	----------	----------	----------	----------	-
Didelphidae	----------	----------	----------	----------	----------	----------	----------	-
Latimeria_chalumnae	----------	----------	----------	----------	----------	----------	----------	-
Monodelphis_domestica	----------	----------	----------	----------	----------	----------	----------	-
Macropus_eugenii	----------	----------	----------	----------	----------	----------	----------	-
Alligator_mississippiensis	----------	----------	----------	----------	----------	----------	----------	-
Chrysemys_picta_bellii	GGTTTTCCTG	AGGCGGCACC	TTAGATTCAA	TCGAGACTGT	CGAGGAGCGA	AGGTAACTAG	CTGGGGAGCA	456
Danio_rerio	----------	----------	----------	----------	----------	----------	----------	-
Taeniopygia	----------	----------	----------	----------	----------	----------	----------	-
Dasypus_novemcinctus	----------	----------	----------	----------	----------	----------	----------	-
LTR_XT	----------	----------	----------	----------	----------	----------	----------	-
Haplorrhini	ATATTATTAA	AAGACATTAT	TCCTAGATTT	GGACTGCCTC	TAACTTTAGG	CTCGGACAAC	GGACCAGCTT	5604
Catarrhini	GTGCTTCTCC	GAGATCTTAT	TCCTAGGTTT	GGACTGCCCT	TACGAATCGG	CTCAGATAAC	GGGCCGGCGT	5079
Anolis_carolinensis	----------	----------	----------	----------	----------	----------	----------	1184
Murine_leukemia_virus	AAGCTATTAG	AAGAAATATT	CCCAAGATTC	GGGATGCCAC	AGGTATTGGG	TTCCGATAAT	GGGCCTGCCT	5054
Phascolarctos_cinereus	AAGATACTTG	AGGAAATCCT	ACCTCGTTTC	GGGATCCCTA	AGGTACTCGG	GTCCGACAAT	GGCCCTGCCT	4863
Estrildidae	----------	----------	----------	----------	----------	----------	----------	-
Xenopus_Silurana_tropicalis	----------	----------	----------	----------	----------	----------	----------	-
I_XT	NCGTTACNGA	NNACACTGAG	ANAGGTANCN	NTANTACAAN	AAAAGTANTT	GAANAAATNC	TGNAGNANTA	5148
Takifugu_rubripes	----------	----------	----------	----------	----------	----------	----------	-
Bos_taurus	----------	----------	----------	----------	----------	----------	----------	-
Homo_sapiens	----------	----------	----------	----------	----------	----------	----------	-
Microcebus_murinus	----------	----------	----------	----------	----------	----------	----------	-
Myotis_lucifugus	----------	----------	----------	----------	----------	----------	----------	-
Equus_caballus	----------	----------	----------	----------	----------	----------	----------	-
Tupaia_belangeri	----------	----------	----------	----------	----------	----------	----------	-
Loxodonta_africana	----------	----------	----------	----------	----------	----------	----------	-
Murinae	----------	----------	----------	----------	----------	----------	----------	-
Canis_lupus_familiaris	----------	----------	----------	----------	----------	----------	----------	-
Sus_scrofa	----------	----------	----------	----------	----------	----------	----------	-
Choloepus_hoffmanni	----------	----------	----------	----------	----------	----------	----------	-
Vicugna_pacos	----------	----------	----------	----------	----------	----------	----------	-
Gavialis_gangeticus	----------	----------	----------	----------	----------	----------	----------	-
Mammalia	----------	----------	----------	----------	----------	----------	----------	-
Passeroidea	----------	----------	----------	----------	----------	----------	----------	-
Takifugu	----------	----------	----------	----------	----------	----------	----------	-
Pteropus_vampyrus	----------	----------	----------	----------	----------	----------	----------	-
Ornithorhynchus	----------	----------	----------	----------	----------	----------	----------	-
Oryctolagus_cuniculus	----------	----------	----------	----------	----------	----------	----------	-
Gallus_gallus	----------	----------	----------	----------	----------	----------	----------	-
Hominidae	GCGTCCACAG	TGCCTCTACG	CTGTGAAAAC	TCCGAGACGA	CCGGTGGCGC	CTTCCAATTA	CTCAGCGTGG	1935
Metatheria	----------	----------	----------	----------	----------	----------	----------	-
Rodentia	----------	----------	----------	----------	----------	----------	----------	-
Monodelphis	----------	----------	----------	----------	----------	----------	----------	-
Consensus	----------	----------	----------	----------	----------	----------	----------	

Conservation

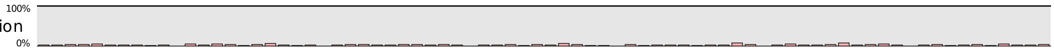

	5,680		5,700		5,720		5,740	
Dipodomys_ordii	----------	----------	----------	----------	----------	----------	----------	-
Laurasiatheria	----------	----------	----------	----------	----------	----------	----------	-
Eutheria	----------	----------	----------	----------	----------	----------	----------	-
Tarsius_syrichta	----------	----------	----------	----------	----------	----------	----------	-
Muridae	GGTCCCANTG	AGATNCCCAG	AGACCAGACT	TGGAGACACC	AGGGGTCTCA	TCTCCGGATT	GGGAAGAGCG	78
Gasterosteus_aculeatus	----------	----------	----------	----------	----------	----------	----------	-
Felis_silvestris	----------	----------	----------	----------	----------	----------	----------	-
Rattus	----------	----------	----------	----------	----------	----------	----------	-
Papio_hamadryas	CAAAAACTCT	CCCACTCTCT	TCGATGAGGC	TCTCCACAGG	GACCTCACCG	ACTTCCGGAC	CCAGCATCCA	3042
Pan_troglodytes	CAGATATTAA	GAGAAAACTC	CAGAAATTAG	AGACTTGAGG	AACGGATCAG	ACTCTTAGCC	CAGGTTTAAA	1117
Ailuropoda_melanoleuca	----------	----------	----------	----------	----------	----------	----------	-
Cavia_porcellus	----------	----------	----------	----------	----------	----------	----------	-
Ictidomys_tridecemlineatus	----------	----------	----------	----------	----------	----------	----------	-
Canis	----------	----------	----------	----------	----------	----------	----------	-
Felis_catus	----------	----------	----------	----------	----------	----------	----------	-
Ochotona_princeps	----------	----------	----------	----------	----------	----------	----------	-
Cercopithecidae	----------	----------	----------	----------	----------	----------	----------	-
Carnivora	----------	----------	----------	----------	----------	----------	----------	-
Mus	----------	----------	----------	----------	----------	----------	----------	-
Mus_musculus	----------	----------	----------	----------	----------	----------	----------	-
Rattus_norvegicus	----------	----------	----------	----------	----------	----------	----------	-
Tursiops_truncatus	----------	----------	----------	----------	----------	----------	----------	-
Procavia_capensis	----------	----------	----------	----------	----------	----------	----------	-
Primates	----------	----------	----------	----------	----------	----------	----------	-
Echinops_telfairi	----------	----------	----------	----------	----------	----------	----------	-
Sorex_araneus	----------	----------	----------	----------	----------	----------	----------	-
Canidae	----------	----------	----------	----------	----------	----------	----------	-
Otolemur_garnettii	----------	----------	----------	----------	----------	----------	----------	-
Gallus	----------	----------	----------	----------	----------	----------	----------	-
Callithrix_jacchus	----------	----------	----------	----------	----------	----------	----------	-
Crocodylus_porosus	----------	----------	----------	----------	----------	----------	----------	-
Erinaceus_europaeus	----------	----------	----------	----------	----------	----------	----------	-
rnd-6_family-7426-LTR-ERV1	----------	----------	----------	----------	----------	----------	----------	-
Esox_lucius	----------	----------	----------	----------	----------	----------	----------	-
Salmo_salar	----------	----------	----------	----------	----------	----------	----------	-
Didelphidae	----------	----------	----------	----------	----------	----------	----------	-
Latimeria_chalumnae	----------	----------	----------	----------	----------	----------	----------	-
Monodelphis_domestica	----------	----------	----------	----------	----------	----------	----------	-
Macropus_eugenii	----------	----------	----------	----------	----------	----------	----------	-
Alligator_mississippiensis	----------	----------	----------	----------	----------	----------	----------	-
Chrysemys_picta_bellii	GTTAGAGAAC	GCCCCCCGGT	GATCCGAAGT	ACCTTACGGC	CAACATGGCA	AAAACAGAAA	GAACCGGTCT	526
Danio_rerio	----------	----------	----------	----------	----------	----------	----------	-
Taeniopygia	----------	----------	----------	----------	----------	----------	----------	-
Dasypus_novemcinctus	----------	----------	----------	----------	----------	----------	----------	-
LTR_XT	----------	----------	----------	----------	----------	----------	----------	-
Haplorrhini	TTGTGGCAGA	AGTAGTACAG	CAACTAACTC	AGATGTTAAA	AATTAAATGG	AAACTGCATA	CAGCCTATCA	5674
Catarrhini	TTGTGGCTGA	CTTGGTACAG	AAGACAGCAA	AGGCATTAGG	AATCACTTGG	AAGCTACGTG	CCGCCTACCG	5149
Anolis_carolinensis	----------	----------	----------	----------	----------	----------	----------	1184
Murine_leukemia_virus	TCGTCTCCCA	GGTAAGTCAG	TCGGTGGCCG	ATTTACTGGG	GATCGATTGG	AAATTACATT	GTGCTTATAG	5124
Phascolarctos_cinereus	TCGTTGCTCA	GGTAAGCCAG	GGGTTGGCCA	CTCAACTGGG	GATTGATTGG	AAATTACATT	GTGCATATAG	4933
Estrildidae	----------	----------	----------	----------	----------	----------	----------	-
Xenopus_Silurana_tropicalis	----------	----------	----------	----------	----------	----------	----------	-
I_XT	ANATACACCA	ACAANAANAC	TGTANNGACC	NGANGTNCCA	AGCNAANTAG	AAGGAGCTAT	CGGAANTCAA	5218
Takifugu_rubripes	----------	----------	----------	----------	----------	----------	----------	-
Bos_taurus	----------	----------	----------	----------	----------	----------	----------	-
Homo_sapiens	----------	----------	----------	----------	----------	----------	----------	-
Microcebus_murinus	----------	----------	----------	----------	----------	----------	----------	-
Myotis_lucifugus	----------	----------	----------	----------	----------	----------	----------	-
Equus_caballus	----------	----------	----------	----------	----------	----------	----------	-
Tupaia_belangeri	----------	----------	----------	----------	----------	----------	----------	-
Loxodonta_africana	----------	----------	----------	----------	----------	----------	----------	-
Murinae	----------	----------	----------	----------	----------	----------	----------	-
Canis_lupus_familiaris	----------	----------	----------	----------	----------	----------	----------	-
Sus_scrofa	----------	----------	----------	----------	----------	----------	----------	-
Choloepus_hoffmanni	----------	----------	----------	----------	----------	----------	----------	-
Vicugna_pacos	----------	----------	----------	----------	----------	----------	----------	-
Gavialis_gangeticus	----------	----------	----------	----------	----------	----------	----------	-
Mammalia	----------	----------	----------	----------	----------	----------	----------	-
Passeroidea	----------	----------	----------	----------	----------	----------	----------	-
Takifugu	----------	----------	----------	----------	----------	----------	----------	-
Pteropus_vampyrus	----------	----------	----------	----------	----------	----------	----------	-
Ornithorhynchus	----------	----------	----------	----------	----------	----------	----------	-
Oryctolagus_cuniculus	----------	----------	----------	----------	----------	----------	----------	-
Gallus_gallus	----------	----------	----------	----------	----------	----------	----------	-
Hominidae	ACGACGACCG	AAGAGGCCCA	GACTCGGGAA	CCCCCCTCAC	TCTTGCCGAG	CCCAGGGTAA	CGCTCCAGGT	2005
Metatheria	----------	----------	----------	----------	----------	----------	----------	-
Rodentia	----------	----------	----------	----------	----------	----------	----------	-
Monodelphis	----------	----------	----------	----------	----------	----------	----------	-
Consensus	----------	----------	----------	----------	----------	----------	----------	

Conservation

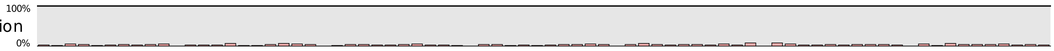

			5,760		5,780		5,800	
Dipodomys_ordii	----------	----------	----------	----------	----------	----------	----------	-
Laurasiatheria	----------	----------	----------	----------	----------	----------	----------	-
Eutheria	----------	----------	----------	----------	----------	----------	----------	-
Tarsius_syrichta	----------	----------	----------	----------	----------	----------	----------	-
Muridae	CAGTGGTCAA	GGGCCTCCTA	GGGGGTGCTC	AGCCTGAGAT	GTTCCAGGTC	CCCAGGGTTC	CCTTTGATTA	148
Gasterosteus_aculeatus	----------	----------	----------	----------	----------	----------	----------	-
Felis_silvestris	----------	----------	----------	----------	----------	----------	----------	-
Rattus	----------	----------	----------	----------	----------	----------	----------	-
Papio_hamadryas	GAAGTGACCC	TGCTCCAGTA	TGTAGATGAC	CTCCTCTTGG	CGGCCCCCAC	AAAGAAAGCC	TGCACGCAAG	3112
Pan_troglodytes	AAAGAGATAC	TCAGAGGAAA	AGCAATAGGC	ATAAAATGAC	CAATCCTGGC	AGCAGTGTAC	AGAAAGCTAA	1187
Ailuropoda_melanoleuca	----------	----------	----------	----------	----------	----------	----------	-
Cavia_porcellus	----------	----------	----------	----------	----------	----------	----------	-
Ictidomys_tridecemlineatus	----------	----------	----------	----------	----------	----------	----------	-
Canis	----------	----------	----------	----------	----------	----------	----------	-
Felis_catus	----------	----------	----------	----------	----------	----------	----------	-
Ochotona_princeps	----------	----------	----------	----------	----------	----------	----------	-
Cercopithecidae	----------	----------	----------	----------	----------	----------	----------	-
Carnivora	----------	----------	----------	----------	----------	----------	----------	-
Mus	----------	----------	----------	----------	----------	----------	----------	-
Mus_musculus	----------	----------	----------	----------	----------	----------	----------	-
Rattus_norvegicus	----------	----------	----------	----------	----------	----------	----------	-
Tursiops_truncatus	----------	----------	----------	----------	----------	----------	----------	-
Procavia_capensis	----------	----------	----------	----------	----------	----------	----------	-
Primates	----------	----------	----------	----------	----------	----------	----------	-
Echinops_telfairi	----------	----------	----------	----------	----------	----------	----------	-
Sorex_araneus	----------	----------	----------	----------	----------	----------	----------	-
Canidae	----------	----------	----------	----------	----------	----------	----------	-
Otolemur_garnettii	----------	----------	----------	----------	----------	----------	----------	-
Gallus	----------	----------	----------	----------	----------	----------	----------	-
Callithrix_jacchus	----------	----------	----------	----------	----------	----------	----------	-
Crocodylus_porosus	----------	----------	----------	----------	----------	----------	----------	-
Erinaceus_europaeus	----------	----------	----------	----------	----------	----------	----------	-
rnd-6_family-7426-LTR-ERV1	----------	----------	----------	----------	----------	----------	----------	-
Esox_lucius	----------	----------	----------	----------	----------	----------	----------	-
Salmo_salar	----------	----------	----------	----------	----------	----------	----------	-
Didelphidae	----------	----------	----------	----------	----------	----------	----------	-
Latimeria_chalumnae	----------	----------	----------	----------	----------	----------	----------	-
Monodelphis_domestica	----------	----------	----------	----------	----------	----------	----------	-
Macropus_eugenii	----------	----------	----------	----------	----------	----------	----------	-
Alligator_mississippiensis	----------	----------	----------	----------	----------	----------	----------	-
Chrysemys_picta_bellii	CGTGATGCAA	TCTTACATGG	TAGTTTAGAT	ATTAGATAGA	AAAAGATTTG	GAATGGAATT	ATGCAAAGCA	596
Danio_rerio	----------	----------	----------	----------	----------	----------	----------	-
Taeniopygia	----------	----------	----------	----------	----------	----------	----------	-
Dasypus_novemcinctus	----------	----------	----------	----------	----------	----------	----------	-
LTR_XT	----------	----------	----------	----------	----------	----------	----------	-
Haplorrhini	TCCACAAAGT	TCTGGAAAGG	TTGAAAGGAT	GAACCGGACA	CTGAAACAAC	TGTTGAAGAA	GTTTTGCCAA	5744
Catarrhini	ACCTCAGAGT	TCCGGAAAGG	TGGAGCGAAT	GAATCGGACT	ATCAAAAATA	GTTTAGGGAA	AGTATGTCAG	5219
Anolis_carolinensis	----------	----------	----------	----------	----------	----------	----------	1184
Murine_leukemia_virus	ACCCCAGAGT	TCAGGTCAGG	TAGAAAGAAT	GAATAGAACC	ATCAAGGAGA	CTTTAACCAA	ATTAACGCTT	5194
Phascolarctos_cinereus	ACCCCAGAGC	TCAGGTCAGG	TAGAAAGGAT	GAATAGGACA	ATTAAAGAGA	CCTTGACAAA	ATTGGCCTTA	5003
Estrildidae	----------	----------	----------	----------	----------	----------	----------	-
Xenopus_Silurana_tropicalis	----------	----------	----------	----------	----------	----------	----------	-
I_XT	AAANNTNNAG	TTNGTAANTN	CTGTANNNAN	ATNNAGTTAA	GTTAGGGTAC	AAATTTTNAN	CGNTGCATTG	5288
Takifugu_rubripes	----------	----------	----------	----------	----------	----------	----------	-
Bos_taurus	----------	----------	----------	----------	----------	----------	----------	-
Homo_sapiens	----------	----------	----------	----------	----------	----------	----------	-
Microcebus_murinus	----------	----------	----------	----------	----------	----------	----------	-
Myotis_lucifugus	----------	----------	----------	----------	----------	----------	----------	-
Equus_caballus	----------	----------	----------	----------	----------	----------	----------	-
Tupaia_belangeri	----------	----------	----------	----------	----------	----------	----------	-
Loxodonta_africana	----------	----------	----------	----------	----------	----------	----------	-
Murinae	----------	----------	----------	----------	----------	----------	----------	-
Canis_lupus_familiaris	----------	----------	----------	----------	----------	----------	----------	-
Sus_scrofa	----------	----------	----------	----------	----------	----------	----------	-
Choloepus_hoffmanni	----------	----------	----------	----------	----------	----------	----------	-
Vicugna_pacos	----------	----------	----------	----------	----------	----------	----------	-
Gavialis_gangeticus	----------	----------	----------	----------	----------	----------	----------	-
Mammalia	----------	----------	----------	----------	----------	----------	----------	-
Passeroidea	----------	----------	----------	----------	----------	----------	----------	-
Takifugu	----------	----------	----------	----------	----------	----------	----------	-
Pteropus_vampyrus	----------	----------	----------	----------	----------	----------	----------	-
Ornithorhynchus	----------	----------	----------	----------	----------	----------	----------	-
Oryctolagus_cuniculus	----------	----------	----------	----------	----------	----------	----------	-
Gallus_gallus	----------	----------	----------	----------	----------	----------	----------	-
Hominidae	AGCGGGTAAG	TCCATATCTT	TTCTCGTGCA	TATGGGGGCT	ACCTATTCTG	TTTTGCCTTC	CTTCGGCGGG	2075
Metatheria	----------	----------	----------	----------	----------	----------	----------	-
Rodentia	----------	----------	----------	----------	----------	----------	----------	-
Monodelphis	----------	----------	----------	----------	----------	----------	----------	-
Consensus	----------	----------	----------	----------	----------	----------	----------	

Conservation

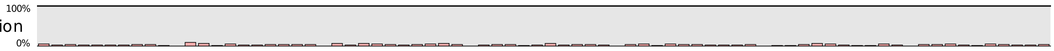

	5,820		5,840		5,860		5,880	
Dipodomys_ordii	----------	----------	----------	----------	----------	----------	----------	-
Laurasiatheria	----------	----------	----------	----------	----------	----------	----------	-
Eutheria	----------	----------	----------	----------	----------	----------	----------	-
Tarsius_syrichta	----------	----------	----------	----------	----------	----------	----------	-
Muridae	ATCGCCACCT	AGCGCTTCAG	AGAGGTCTGA	CACCTCCACC	CCAAAAGGAA	ACAAATTAGA	AAGCCGCCTG	218
Gasterosteus_aculeatus	----------	----------	----------	----------	----------	----------	----------	-
Felis_silvestris	----------	----------	----------	----------	----------	----------	----------	-
Rattus	----------	----------	----------	----------	----------	----------	----------	-
Papio_hamadryas	GTACTAGGCA	TCTACTCCAG	GAACTAGGTG	AGAAAGGATA	CCGGGCATCT	GCCAAGAAGG	CACAAATTTG	3182
Pan_troglodytes	AGACCAAGGG	CGGCAAAATG	AAAGACCAAT	GTGCCTATTG	AAGGGGCATG	GGTAAGAGAC	TGCCAAAACA	1257
Ailuropoda_melanoleuca	----------	----------	----------	----------	----------	----------	----------	-
Cavia_porcellus	----------	----------	----------	----------	----------	----------	----------	-
Ictidomys_tridecemlineatus	----------	----------	----------	----------	----------	----------	----------	-
Canis	----------	----------	----------	----------	----------	----------	----------	-
Felis_catus	----------	----------	----------	----------	----------	----------	----------	-
Ochotona_princeps	----------	----------	----------	----------	----------	----------	----------	-
Cercopithecidae	----------	----------	----------	----------	----------	----------	----------	-
Carnivora	----------	----------	----------	----------	----------	----------	----------	-
Mus	----------	----------	----------	----------	----------	----------	----------	-
Mus_musculus	----------	----------	----------	----------	----------	----------	----------	-
Rattus_norvegicus	----------	----------	----------	----------	----------	----------	----------	-
Tursiops_truncatus	----------	----------	----------	----------	----------	----------	----------	-
Procavia_capensis	----------	----------	----------	----------	----------	----------	----------	-
Primates	----------	----------	----------	----------	----------	----------	----------	-
Echinops_telfairi	----------	----------	----------	----------	----------	----------	----------	-
Sorex_araneus	----------	----------	----------	----------	----------	----------	----------	-
Canidae	----------	----------	----------	----------	----------	----------	----------	-
Otolemur_garnettii	----------	----------	----------	----------	----------	----------	----------	-
Gallus	----------	----------	----------	----------	----------	----------	----------	-
Callithrix_jacchus	----------	----------	----------	----------	----------	----------	----------	-
Crocodylus_porosus	----------	----------	----------	----------	----------	----------	----------	-
Erinaceus_europaeus	----------	----------	----------	----------	----------	----------	----------	-
rnd-6_family-7426-LTR-ERV1	----------	----------	----------	----------	----------	----------	----------	-
Esox_lucius	----------	----------	----------	----------	----------	----------	----------	-
Salmo_salar	----------	----------	----------	----------	----------	----------	----------	-
Didelphidae	----------	----------	----------	----------	----------	----------	----------	-
Latimeria_chalumnae	----------	----------	----------	----------	----------	----------	----------	-
Monodelphis_domestica	----------	----------	----------	----------	----------	----------	----------	-
Macropus_eugenii	----------	----------	----------	----------	----------	----------	----------	-
Alligator_mississippiensis	----------	----------	----------	----------	----------	----------	----------	-
Chrysemys_picta_bellii	CCTAGAGAGG	AAAAGAAAAC	AAAATATGGT	CACGGGAAGG	GAGGGGGGGA	GTGGGGGAGG	GGTAACAGTT	666
Danio_rerio	----------	----------	----------	----------	----------	----------	----------	-
Taeniopygia	----------	----------	----------	----------	----------	----------	----------	-
Dasypus_novemcinctus	----------	----------	----------	----------	----------	----------	----------	-
LTR_XT	----------	----------	----------	----------	----------	----------	----------	-
Haplorrhini	GAGACTCATT	TAAGG---TG	GGATCAGGTA	TTGCCCATGG	TCCTTCTCCG	AGTCAGGTGT	ACCCCTACTA	5811
Catarrhini	GAAACAGGAT	TAAAG---CG	GATACAGGCC	CTTCCTATGG	TATTGTTTAA	AATTAGATGC	ACTCCTTCTA	5286
Anolis_carolinensis	----------	----------	----------	----------	----------	----------	----------	1184
Murine_leukemia_virus	GCAGCTGGCA	CTAGAGACTG	GGTACTCCTA	CTCCCCCTGG	CCCTCTACCG	AGCCCGGAAT	ACTCCGGGCC	5264
Phascolarctos_cinereus	GAGACCGGTG	GAAAAGACTG	GGTGACCCTC	CTCCCTTTGG	CGCTACTTAG	GGCCCGGAAT	ACCCCTGGCC	5073
Estrildidae	----------	----------	----------	----------	----------	----------	----------	-
Xenopus_Silurana_tropicalis	----------	----------	----------	----------	----------	----------	----------	-
I_XT	TTANCTANGA	GACACACTCC	NATGGATCCC	NAGNANTTAT	CTNCTTNTGA	NTTACNGTTT	GGTANANCAA	5358
Takifugu_rubripes	----------	----------	----------	----------	----------	----------	----------	-
Bos_taurus	----------	----------	----------	----------	----------	----------	----------	-
Homo_sapiens	----------	----------	----------	----------	----------	----------	----------	-
Microcebus_murinus	----------	----------	----------	----------	----------	----------	----------	-
Myotis_lucifugus	----------	----------	----------	----------	----------	----------	----------	-
Equus_caballus	----------	----------	----------	----------	----------	----------	----------	-
Tupaia_belangeri	----------	----------	----------	----------	----------	----------	----------	-
Loxodonta_africana	----------	----------	----------	----------	----------	----------	----------	-
Murinae	----------	----------	----------	----------	----------	----------	----------	-
Canis_lupus_familiaris	----------	----------	----------	----------	----------	----------	----------	-
Sus_scrofa	----------	----------	----------	----------	----------	----------	----------	-
Choloepus_hoffmanni	----------	----------	----------	----------	----------	----------	----------	-
Vicugna_pacos	----------	----------	----------	----------	----------	----------	----------	-
Gavialis_gangeticus	----------	----------	----------	----------	----------	----------	----------	-
Mammalia	----------	----------	----------	----------	----------	----------	----------	-
Passeroidea	----------	----------	----------	----------	----------	----------	----------	-
Takifugu	----------	----------	----------	----------	----------	----------	----------	-
Pteropus_vampyrus	----------	----------	----------	----------	----------	----------	----------	-
Ornithorhynchus	----------	----------	----------	----------	----------	----------	----------	-
Oryctolagus_cuniculus	----------	----------	----------	----------	----------	----------	----------	-
Gallus_gallus	----------	----------	----------	----------	----------	----------	----------	-
Hominidae	CCCAGTTTCC	CGTCCCCGGT	CACGGTAGTG	GGGATTGACG	GTACCCCTTC	CACCCATCGT	CAGACCCCCC	2145
Metatheria	----------	----------	----------	----------	----------	----------	----------	-
Rodentia	----------	----------	----------	----------	----------	----------	----------	-
Monodelphis	----------	----------	----------	----------	----------	----------	----------	-
Consensus	----------	----------	----------	----------	----------	----------	----------	

Conservation (100% / 0%)

		5,900		5,920		5,940		
Dipodomys_ordii	----------	----------	----------	----------	----------	----------	----------	-
Laurasiatheria	----------	----------	----------	----------	----------	----------	----------	-
Eutheria	----------	----------	----------	----------	----------	----------	----------	-
Tarsius_syrichta	----------	----------	----------	----------	----------	----------	----------	-
Muridae	GTCTGTCACC	TCCGGAGGTA	AGTCCTGTTA	AGGCACCTTC	GTTTAGAAAA	GTAGGAGAGG	TCNGGTNGGG	288
Gasterosteus_aculeatus	----------	----------	----------	----------	----------	----------	----------	-
Felis_silvestris	----------	----------	----------	----------	----------	----------	----------	-
Rattus	----------	----------	----------	----------	----------	----------	----------	-
Papio_hamadryas	TCAGACCAAG	GTAACTTACC	TGGGGTACAT	ACTGAGTGAG	GGAAAA----	-AGGTGGCTC	ACCCCTGGGC	3247
Pan_troglodytes	GAGGACGGAG	CCAGCTCCCT	GTAGACAGAT	AGGGGCGGGT	TCGACCCCCC	CGACCTGGTA	ACTTGTGGGG	1327
Ailuropoda_melanoleuca	----------	----------	----------	----------	----------	----------	----------	-
Cavia_porcellus	----------	----------	----------	----------	----------	----------	----------	-
Ictidomys_tridecemlineatus	----------	----------	----------	----------	----------	----------	----------	-
Canis	----------	----------	----------	----------	----------	----------	----------	-
Felis_catus	----------	----------	----------	----------	----------	----------	----------	-
Ochotona_princeps	----------	----------	----------	----------	----------	----------	----------	-
Cercopithecidae	----------	----------	----------	----------	----------	----------	----------	-
Carnivora	----------	----------	----------	----------	----------	----------	----------	-
Mus	----------	----------	----------	----------	----------	----------	----------	-
Mus_musculus	----------	----------	----------	----------	----------	----------	----------	-
Rattus_norvegicus	----------	----------	----------	----------	----------	----------	----------	-
Tursiops_truncatus	----------	----------	----------	----------	----------	----------	----------	-
Procavia_capensis	----------	----------	----------	----------	----------	----------	----------	-
Primates	----------	----------	----------	----------	----------	----------	----------	-
Echinops_telfairi	----------	----------	----------	----------	----------	----------	----------	-
Sorex_araneus	----------	----------	----------	----------	----------	----------	----------	-
Canidae	----------	----------	----------	----------	----------	----------	----------	-
Otolemur_garnettii	----------	----------	----------	----------	----------	----------	----------	-
Gallus	----------	----------	----------	----------	----------	----------	----------	-
Callithrix_jacchus	----------	----------	----------	----------	----------	----------	----------	-
Crocodylus_porosus	----------	----------	----------	----------	----------	----------	----------	-
Erinaceus_europaeus	----------	----------	----------	----------	----------	----------	----------	-
rnd-6_family-7426-LTR-ERV1	----------	----------	----------	----------	----------	----------	----------	-
Esox_lucius	----------	----------	----------	----------	----------	----------	----------	-
Salmo_salar	----------	----------	----------	----------	----------	----------	----------	-
Didelphidae	----------	----------	----------	----------	----------	----------	----------	-
Latimeria_chalumnae	----------	----------	----------	----------	----------	----------	----------	-
Monodelphis_domestica	----------	----------	----------	----------	----------	----------	----------	-
Macropus_eugenii	----------	----------	----------	----------	----------	----------	----------	-
Alligator_mississippiensis	----------	----------	----------	----------	----------	----------	----------	-
Chrysemys_picta_bellii	GCATTGGGGA	GGGACATGGA	AAAATGAGTG	CCCAGGGAGC	GCAACAGGGG	CGGGGATCTG	CCCAACCATA	736
Danio_rerio	----------	----------	----------	----------	----------	----------	----------	-
Taeniopygia	----------	----------	----------	----------	----------	----------	----------	-
Dasypus_novemcinctus	----------	----------	----------	----------	----------	----------	----------	-
LTR_XT	----------	----------	----------	----------	----------	----------	----------	-
Haplorrhini	AATTAACCGG	GTATTCACCC	TATGAGATAG	TGTATGGCCG	ACCACCCCCA	CTCATA----	TCTCAGGTAA	5877
Catarrhini	AGAAAACAGG	ATACTCCCCT	TATGAAATAC	TGTATCATAG	GCCTCCTCCT	ATACTA----	CGGGAGCTTC	5352
Anolis_carolinensis	----------	----------	----------	----------	----------	----------	----------	1184
Murine_leukemia_virus	CCCA---TGG	ACTTACTCCG	TATGAAATTC	TGTATGGGGC	ACCCCCGCCC	CTTGTCAATT	TTCATGATCC	5331
Phascolarctos_cinereus	AGTT---TGG	CCTGACTCCT	TATGAGATTC	TTCATGGGGG	GCCGCCCCCT	GTACTTGCGT	CTGGGGAAGT	5140
Estrildidae	----------	----------	----------	----------	----------	----------	----------	-
Xenopus_Silurana_tropicalis	----------	----------	----------	----------	----------	----------	----------	-
I_XT	TATANACTAG	NTTGCTTNTT	NGACANTATT	NNTTGGAGGA	ACATGACTTC	TNNAACTGAC	TATGTTAACC	5428
Takifugu_rubripes	----------	----------	----------	----------	----------	----------	----------	-
Bos_taurus	----------	----------	----------	----------	----------	----------	----------	-
Homo_sapiens	----------	----------	----------	----------	----------	----------	----------	-
Microcebus_murinus	----------	----------	----------	----------	----------	----------	----------	-
Myotis_lucifugus	----------	----------	----------	----------	----------	----------	----------	-
Equus_caballus	----------	----------	----------	----------	----------	----------	----------	-
Tupaia_belangeri	----------	----------	----------	----------	----------	----------	----------	-
Loxodonta_africana	----------	----------	----------	----------	----------	----------	----------	-
Murinae	----------	----------	----------	----------	----------	----------	----------	-
Canis_lupus_familiaris	----------	----------	----------	----------	----------	----------	----------	-
Sus_scrofa	----------	----------	----------	----------	----------	----------	----------	-
Choloepus_hoffmanni	----------	----------	----------	----------	----------	----------	----------	-
Vicugna_pacos	----------	----------	----------	----------	----------	----------	----------	-
Gavialis_gangeticus	----------	----------	----------	----------	----------	----------	----------	-
Mammalia	----------	----------	----------	----------	----------	----------	----------	-
Passeroidea	----------	----------	----------	----------	----------	----------	----------	-
Takifugu	----------	----------	----------	----------	----------	----------	----------	-
Pteropus_vampyrus	----------	----------	----------	----------	----------	----------	----------	-
Ornithorhynchus	----------	----------	---GTTTACG	AAAAGGGTCT	TTTTGGGGAG	GGGGGCTGAC	CCTCGGGAAC	47
Oryctolagus_cuniculus	----------	----------	----------	----------	----------	----------	----------	-
Gallus_gallus	----------	----------	----------	----------	----------	----------	----------	-
Hominidae	CATTGTCTTG	CCGGCTGGAC	GACACTCTCA	TCTCCCATTC	CTTCCTCATT	ATCCCTTCCT	GTCCCGTCCC	2215
Metatheria	----------	----------	----------	----------	----------	----------	----------	-
Rodentia	----------	----------	----------	----------	----------	----------	----------	-
Monodelphis	----------	----------	----------	----------	----------	----------	----------	-
Consensus	----------	----------	----------	----------	----------	----------	----------	

Conservation

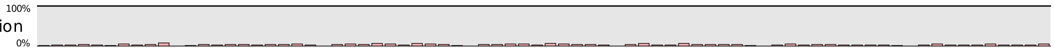

	5,960		5,980		6,000		6,020	
Dipodomys_ordii	----------	----------	----------	----------	----------	----------	----------	-
Laurasiatheria	----------	----------	----------	----------	----------	----------	----------	-
Eutheria	----------	----------	----------	----------	----------	----------	----------	-
Tarsius_syrichta	----------	----------	----------	----------	----------	----------	----------	-
Muridae	CCGCTGATCC	AGTGTCTGGG	ATCCATGTGA	GATGTCACTG	GATCCTTCTG	TCTGTTCAGG	TGTATGAATG	358
Gasterosteus_aculeatus	----------	----------	----------	----------	----------	-----TAAAG	GGTTTTATAA	15
Felis_silvestris	----------	----------	----------	----------	----------	----------	----------	-
Rattus	----------	----------	----------	----------	----------	----------	----------	-
Papio_hamadryas	GCATAGAGAC	TGTGGCTCGC	ATTCCACCGC	CCCGGAATCC	CAGAGAGGTG	CGTGAATTCC	TGGGAACTGC	3317
Pan_troglodytes	GTCCCCAGTC	AGTTCTGTGA	ACGGGCCAGC	AGTGTTTCTG	GAGGAAAAAA	AGTAAAAGAT	TTTTCCGCTT	1397
Ailuropoda_melanoleuca	----------	----------	----------	----------	----------	----------	----------	-
Cavia_porcellus	----------	----------	----------	----------	----------	----------	----------	-
Ictidomys_tridecemlineatus	----------	----------	----------	----------	----------	----------	----------	-
Canis	----------	----------	----------	----------	----------	----------	----------	-
Felis_catus	----------	----------	----------	----------	----------	----------	----------	-
Ochotona_princeps	----------	----------	----------	----------	----------	----------	----------	-
Cercopithecidae	----------	----------	----------	----------	----------	----------	----------	-
Carnivora	----------	----------	----------	----------	----------	----------	----------	-
Mus	----------	----------	----------	----------	----------	----------	----------	-
Mus_musculus	----------	----------	----------	----------	----------	----------	----------	-
Rattus_norvegicus	----------	----------	----------	----------	----------	----------	----------	-
Tursiops_truncatus	----------	----------	----------	----------	----------	----------	----------	-
Procavia_capensis	----------	----------	----------	----------	----------	----------	----------	-
Primates	----------	----------	----------	----------	----------	----------	----------	-
Echinops_telfairi	----------	----------	----------	----------	----------	----------	----------	-
Sorex_araneus	----------	----------	----------	----------	----------	----------	----------	-
Canidae	----------	----------	----------	----------	----------	----------	----------	-
Otolemur_garnettii	----------	----------	----------	----------	----------	----------	----------	-
Gallus	----------	----------	----------	----------	----------	----------	----------	-
Callithrix_jacchus	----------	----------	----------	----------	----------	----------	----------	-
Crocodylus_porosus	----------	----------	----------	----------	----------	----------	----------	-
Erinaceus_europaeus	----------	----------	----------	----------	----------	----------	----------	-
rnd-6_family-7426-LTR-ERV1	----------	----------	----------	----------	----------	----------	----------	-
Esox_lucius	----------	----------	----------	----------	----------	----------	----------	-
Salmo_salar	----------	----------	----------	----------	----------	----------	----------	-
Didelphidae	----------	----------	----------	----------	----------	----------	----------	-
Latimeria_chalumnae	----------	----------	----------	----------	----------	----------	----------	-
Monodelphis_domestica	----------	----------	----------	----------	----------	----------	----------	-
Macropus_eugenii	----------	----------	----------	----------	----------	----------	----------	-
Alligator_mississippiensis	----------	----------	----------	----------	----------	----------	----------	-
Chrysemys_picta_bellii	AAGGGGTGTT	GAATGATTTG	TGACTGGAGC	CCGCGTAACC	ACCCTTCGTC	CGGCAATCTA	CGGTGGGACA	806
Danio_rerio	----------	----------	----------	----------	----------	----------	----------	-
Taeniopygia	----------	----------	----------	----------	----------	----------	----------	-
Dasypus_novemcinctus	----------	----------	----------	----------	----------	----------	----------	-
LTR_XT	----------	----------	----------	----------	----------	----------	----------	-
Haplorrhini	AAGGAGATTT	AAAGGAAATT	GGAGAACTGA	CCCTAAGAAG	ACAAATGCAG	GCATTAGGTG	AGGTAATGCA	5947
Catarrhini	CAGGCACTCC	CCGAGAGTTA	GGTGAAATTG	AATTACAGCG	ACAGCTACAG	GCTTTAGGAA	AAATTACACA	5422
Anolis_carolinensis	----------	----------	----------	----------	----------	----------	----------	1184
Murine_leukemia_virus	TG-AAATGTC	AAAGTTAACT	AATAGTCCCT	CTCTCCAAGC	TCACTTACAG	GCCCTCCAAG	CAGTACAACG	5400
Phascolarctos_cinereus	CGTGGGTTCT	AATGGTGATT	TTTT-TCCCG	TTCTATTTAC	CCACTTGAAG	GCTTTAGAGG	TTGTGAGGAC	5209
Estrildidae	----------	----------	----------	----------	----------	----------	----------	-
Xenopus_Silurana_tropicalis	----------	----------	----------	----------	----------	----------	----------	-
I_XT	ANATNTATAN	GCAGCTNACT	AANCTANATG	GANAACNCTT	CTNCNTTTTA	ACANATACAG	AGACAGTATT	5498
Takifugu_rubripes	----------	----------	----------	----------	----------	----------	----------	-
Bos_taurus	----------	----------	----------	----------	----------	----------	----------	-
Homo_sapiens	----------	----------	----------	----------	----------	----------	----------	-
Microcebus_murinus	----------	----------	----------	----------	----------	----------	----------	-
Myotis_lucifugus	----------	----------	----------	----------	----------	----------	----------	-
Equus_caballus	----------	----------	----------	----------	----------	----------	----------	-
Tupaia_belangeri	----------	----------	----------	----------	----------	----------	----------	-
Loxodonta_africana	----------	----------	----------	----------	----------	----------	----------	-
Murinae	----------	----------	----------	----------	----------	----------	----------	-
Canis_lupus_familiaris	----------	----------	----------	----------	----------	----------	----------	-
Sus_scrofa	----------	----------	----------	----------	----------	----------	----------	-
Choloepus_hoffmanni	----------	----------	----------	----------	----------	----------	----------	-
Vicugna_pacos	----------	----------	----------	----------	----------	----------	----------	-
Gavialis_gangeticus	----------	----------	----------	----------	----------	----------	----------	-
Mammalia	----------	----------	----------	----------	----------	----------	----------	-
Passeroidea	----------	----------	----------	----------	----------	----------	----------	-
Takifugu	----------	----------	----------	----------	----------	----------	----------	-
Pteropus_vampyrus	----------	----------	----------	----------	----------	----------	----------	-
Ornithorhynchus	TTACCTCCCC	TGAACTTTGC	CCGCTTTGGG	TCCGGCTCCT	GCTGCCTCGC	CAATTTCAGT	GAGCGGTGGT	117
Oryctolagus_cuniculus	----------	----------	----------	----------	----------	----------	----------	-
Gallus_gallus	----------	----------	----------	----------	----------	----------	----------	-
Hominidae	GCTCTTCGGA	AGGGACTTGC	TGTCTAAGTT	AGGGGCCTCC	ATTCGGTTGC	GCCCCAGCCT	CCCCTCCAGT	2285
Metatheria	----------	----------	----------	----------	----------	----------	----------	-
Rodentia	----------	----------	----------	----------	----------	----------	----------	-
Monodelphis	----------	----------	----------	----------	----------	----------	----------	-
Consensus	----------	----------	----------	----------	----------	----------	----------	
Conservation (100% / 0%)								

		6,040		6,060		6,080		
Dipodomys_ordii	----------	----------	----------	----------	----------	----------	----------	-
Laurasiatheria	----------	----------	----------	----------	----------	----------	----------	-
Eutheria	----------	----------	----------	----------	----------	----------	----------	-
Tarsius_syrichta	----------	----------	----------	----------	----------	----------	----------	-
Muridae	TGTGGNGGCC	ACGCGTGGTT	GTCTGTTCTG	TGTCTCTCTA	TGTGCATGTG	TGTCTCTGTG	TGTCTTTCTA	428
Gasterosteus_aculeatus	GAAAATCGAT	TTGTGAAGGC	TGACAAGACG	GCCCCTTGCG	GGAACNNTAC	TGGTAAAATA	TGTTATANCA	85
Felis_silvestris	----------	----------	----------	----------	----------	----------	----------	-
Rattus	----------	----------	----------	----------	----------	----------	----------	-
Papio_hamadryas	TGGGTTCTGT	CGCTTGTGGA	TACCCGGTTT	TGCTGAATTG	GCCGCCCCCC	TTTACGCACT	CACCAAGGAA	3387
Pan_troglodytes	GGGATTAATT	CATCCTCGAC	TGACATAATG	GGTCTTACCC	ACTTCCGAAA	CCCCACTCCT	TAGGTGGTAG	1467
Ailuropoda_melanoleuca	----------	----------	----------	----------	----------	----------	----------	-
Cavia_porcellus	----------	----------	----------	----------	----------	----------	----------	-
Ictidomys_tridecemlineatus	----------	----------	----------	----------	----------	----------	----------	-
Canis	----------	----------	----------	----------	----------	----------	----------	-
Felis_catus	----------	----------	----------	----------	----------	----------	----------	-
Ochotona_princeps	----------	----------	----------	----------	----------	----------	----------	-
Cercopithecidae	----------	----------	----------	----------	----------	----------	----------	-
Carnivora	----------	----------	----------	----------	----------	----------	----------	-
Mus	----------	----------	----------	----------	----------	----------	----------	-
Mus_musculus	----------	----------	----------	----------	----------	----------	----------	-
Rattus_norvegicus	----------	----------	----------	----------	----------	----------	----------	-
Tursiops_truncatus	----------	----------	----------	----------	----------	----------	----------	-
Procavia_capensis	----------	----------	----------	----------	----------	----------	----------	-
Primates	----------	----------	----------	----------	----------	----------	----------	-
Echinops_telfairi	----------	----------	----------	----------	----------	----------	----------	-
Sorex_araneus	----------	----------	----------	----------	----------	----------	----------	-
Canidae	----------	----------	----------	----------	----------	----------	----------	-
Otolemur_garnettii	----------	----------	----------	----------	----------	----------	----------	-
Gallus	----------	----------	----------	----------	----------	----------	----------	-
Callithrix_jacchus	----------	----------	----------	----------	----------	----------	----------	-
Crocodylus_porosus	----------	----------	----------	----------	----------	----------	----------	-
Erinaceus_europaeus	----------	----------	----------	----------	----------	----------	----------	-
rnd-6_family-7426-LTR-ERV1	----------	----------	----------	----------	----------	----------	----------	-
Esox_lucius	----------	----------	----------	----------	----------	----------	----------	-
Salmo_salar	----------	----------	----------	----------	----------	----------	----------	-
Didelphidae	----------	----------	----------	----------	----------	----------	----------	-
Latimeria_chalumnae	----------	----------	----------	----------	----------	----------	----------	-
Monodelphis_domestica	----------	----------	----------	----------	----------	----------	----------	-
Macropus_eugenii	----------	----------	----------	----------	----------	----------	----------	-
Alligator_mississippiensis	----------	----------	----------	----------	----------	----------	----------	-
Chrysemys_picta_bellii	GGAAAACGTC	CGTTCCGTCT	GGGCCTTCAT	TTCCCCTTCC	TCTCTGGGAC	GGGACTGTTG	TTTAGGACCT	876
Danio_rerio	----------	----------	----------	----------	----------	----------	----------	-
Taeniopygia	----------	----------	----------	----------	----------	----------	----------	-
Dasypus_novemcinctus	----------	----------	----------	----------	----------	----------	----------	-
LTR_XT	----------	----------	----------	----------	----------	----------	----------	-
Haplorrhini	AGAAGTACAA	GGGTGGGTAA	GAGAAAGAAT	ACCTGTTAGC	CTTACAGATG	CAAT------	ACATCCCTTT	6011
Catarrhini	AACAATCTCA	ACTTGGGTAA	ATGAGAGGTG	TCCCATCAGC	TTATTCTCCC	CAGT------	TCACCCTTTC	5486
Anolis_carolinensis	----------	----------	----------	----------	----------	----------	----------	1184
Murine_leukemia_virus	AGAGGTCTGG	AAGCCGCTGG	CCGCTGCTTA	TCAGGACCAG	CTGGATCAGC	CAGTGATACC	ACACCCCTTC	5470
Phascolarctos_cinereus	TCAGATCTGG	GACCAGATAA	AAGAAGCCTA	C--AGGCCTG	GTACTGTGGC	CATT----CC	CCACCCGTTC	5273
Estrildidae	----------	----------	----------	----------	----------	----------	----------	-
Xenopus_Silurana_tropicalis	----------	----------	----------	----------	----------	----------	----------	-
I_XT	TGGANGCANN	ANCTTCAACC	AGGANTTGNG	TGNTGATAAA	GAGATTNGTG	NNGCAAGAGC	ATAGNTTAGA	5568
Takifugu_rubripes	----------	----------	----------	----------	----------	----------	----------	-
Bos_taurus	----------	----------	----------	----------	----------	----------	----------	-
Homo_sapiens	----------	----------	----------	----------	----------	----------	----------	-
Microcebus_murinus	----------	----------	----------	----------	----------	----------	----------	-
Myotis_lucifugus	----------	----------	----------	----------	----------	----------	----------	-
Equus_caballus	----------	----------	----------	----------	----------	----------	----------	-
Tupaia_belangeri	----------	----------	----------	----------	----------	----------	----------	-
Loxodonta_africana	----------	----------	----------	----------	----------	----------	----------	-
Murinae	----------	----------	----------	----------	----------	----------	----------	-
Canis_lupus_familiaris	----------	----------	----------	----------	----------	----------	----------	-
Sus_scrofa	----------	----------	----------	----------	----------	----------	----------	-
Choloepus_hoffmanni	----------	----------	----------	----------	----------	----------	----------	-
Vicugna_pacos	----------	----------	----------	----------	----------	----------	----------	-
Gavialis_gangeticus	----------	----------	----------	----------	----------	----------	----------	-
Mammalia	----------	----------	----------	----------	----------	----------	----------	-
Passeroidea	----------	----------	----------	----------	----------	----------	----------	-
Takifugu	----------	----------	----------	----------	----------	----------	----------	-
Pteropus_vampyrus	----------	----------	----------	----------	----------	----------	----------	-
Ornithorhynchus	GGTTGTCTCG	TTGAGAGTTT	CGTTCGAGAA	GAGGTTGAGA	GGGATACCTG	GCGGTTGGCC	CCCCGCGCCA	187
Oryctolagus_cuniculus	----------	----------	----------	----------	----------	----------	----------	-
Gallus_gallus	----------	----------	----------	----------	----------	----------	----------	-
Hominidae	GCAATCTCTT	TGCTTCCTCT	GCTGGCACTT	AGCGATGACA	CTCCTTCGCC	GATCCCATCG	CTCCCTGTGC	2355
Metatheria	----------	----------	----------	----------	----------	----------	----------	-
Rodentia	----------	----------	----------	----------	----------	----------	----------	-
Monodelphis	----------	----------	----------	----------	----------	----------	----------	-
Consensus	----------	----------	----------	----------	----------	----------	----------	

Conservation

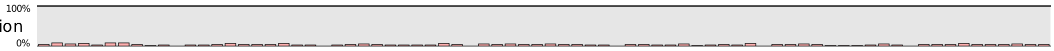

	6,100		6,120		6,140		6,160	
Dipodomys_ordii	----------	----------	----------	----------	----------	----------	----------	-
Laurasiatheria	----------	----------	----------	----------	----------	----------	----------	-
Eutheria	----------	----------	----------	----------	----------	----------	----------	-
Tarsius_syrichta	----------	----------	----------	----------	----------	----------	----------	-
Muridae	TGTGTTTCTG	TCANTTTTGT	TTCCCTGTAT	TGACCAGTGG	CTTTCTCTGG	TGTGAGAATC	CCCTAGCCTT	498
Gasterosteus_aculeatus	TTNTNTGGTG	CNAAACCCGT	GTCTCNAGTG	TCCTGTANTN	CCCTGGTGCA	AAGAGCANCA	TGATACAAAA	155
Felis_silvestris	----------	----------	----------	----------	----------	----------	----------	-
Rattus	----------	----------	----------	----------	----------	----------	----------	-
Papio_hamadryas	AGCACCCCTT	TCACCTGGCA	GACAGAGCAT	CAATTGGCTT	TTGAGGCACT	AAAAAAGGCA	CTCTTGTCTG	3457
Pan_troglodytes	AAAAGCCCAG	GACTGTTGAT	TACGCTTTAT	GGCAGAGAAC	CAGGGCCTTC	AATAAAAGC-	--CTACTCTC	1534
Ailuropoda_melanoleuca	----------	----------	----------	----------	----------	----------	----------	-
Cavia_porcellus	----------	----------	----------	----------	----------	----------	----------	-
Ictidomys_tridecemlineatus	----------	----------	----------	----------	----------	----------	----------	-
Canis	----------	----------	----------	----------	----------	----------	----------	-
Felis_catus	----------	----------	----------	----------	----------	----------	----------	-
Ochotona_princeps	----------	----------	----------	----------	----------	----------	----------	-
Cercopithecidae	----------	----------	----------	----------	----------	----------	----------	-
Carnivora	----------	----------	----------	----------	----------	----------	----------	-
Mus	----------	----------	----------	----------	----------	----------	----------	-
Mus_musculus	----------	----------	----------	----------	----------	----------	----------	-
Rattus_norvegicus	----------	----------	----------	----------	----------	----------	----------	-
Tursiops_truncatus	----------	----------	----------	----------	----------	----------	----------	-
Procavia_capensis	----------	----------	----------	----------	----------	----------	----------	-
Primates	----------	----------	----------	----------	----------	----------	----------	-
Echinops_telfairi	----------	----------	----------	----------	----------	----------	----------	-
Sorex_araneus	----------	----------	----------	----------	----------	----------	----------	-
Canidae	----------	----------	----------	----------	----------	----------	----------	-
Otolemur_garnettii	----------	----------	----------	----------	----------	----------	----------	-
Gallus	----------	----------	----------	----------	----------	----------	----------	-
Callithrix_jacchus	----------	----------	----------	----------	----------	----------	----------	-
Crocodylus_porosus	----------	----------	----------	----------	----------	----------	----------	-
Erinaceus_europaeus	----------	----------	----------	----------	----------	----------	----------	-
rnd-6_family-7426-LTR-ERV1	----------	----------	----------	----------	----------	----------	----------	-
Esox_lucius	----------	----------	----------	----------	----------	----------	----------	-
Salmo_salar	----------	----------	----------	----------	----------	----------	----------	-
Didelphidae	----------	----------	----------	----------	----------	----------	----------	-
Latimeria_chalumnae	----------	----------	----------	----------	----------	----------	----------	-
Monodelphis_domestica	----------	----------	----------	----------	----------	----------	----------	-
Macropus_eugenii	----------	----------	----------	----------	----------	----------	----------	-
Alligator_mississippiensis	----------	----------	----------	----------	----------	----------	----------	-
Chrysemys_picta_bellii	ATTCACCAGA	TGTACTTCCC	CGAGAATCTA	TGCCTTCATT	ACCCAGCACA	CCCCCATGGC	AGGTCTGGCA	946
Danio_rerio	----------	----------	----------	----------	----------	----------	----------	-
Taeniopygia	----------	----------	----------	----------	----------	----------	----------	-
Dasypus_novemcinctus	----------	----------	----------	----------	----------	----------	----------	-
LTR_XT	----------	----------	----------	----------	----------	----------	----------	-
Haplorrhini	CAACCTGGGG	ACTCTGTATG	GGTTAAACGC	TGGAATCCTA	CCACCCTCGG	GCCCTTATGG	GATGGCCCCC	6081
Catarrhini	TCTCCAGGTG	ATCGCGTGTG	GATCAAGGAC	TGGAACGTAG	CCCCTTTGCG	GCCACGGTGG	AAAGGACCTC	5556
Anolis_carolinensis	----------	----------	----------	----------	----------	----------	----------	1184
Murine_leukemia_virus	CGTGTCGGTG	ACGCCGTGTG	GGTACGCCGG	CACCAGACTA	AGAACTTAGA	ACCCCGCTGG	AAAGGACCCT	5540
Phascolarctos_cinereus	CAGGTCGGTG	ACCGAGTGCT	GGTTAGACGT	CATCGGTCCG	GCAGCCTTGA	GCCTCGGTGG	AAAGGCCCAT	5343
Estrildidae	----------	----------	----------	----------	----------	----------	----------	-
Xenopus_Silurana_tropicalis	----------	----------	----------	----------	----------	----------	----------	-
I_XT	CGGACTNTAT	CANNTTCNAG	TAAATACNGN	NNNNNNACCT	ATANTCATAG	AAAGGCCATT	NNTTNACTGN	5638
Takifugu_rubripes	----------	----------	----------	----------	----------	----------	----------	-
Bos_taurus	----------	----------	----------	----------	----------	----------	----------	-
Homo_sapiens	----------	----------	----------	----------	----------	----------	----------	-
Microcebus_murinus	----------	----------	----------	----------	----------	----------	----------	-
Myotis_lucifugus	----------	----------	----------	----------	----------	----------	----------	-
Equus_caballus	----------	----------	----------	----------	----------	----------	----------	-
Tupaia_belangeri	----------	----------	----------	----------	----------	----------	----------	-
Loxodonta_africana	----------	----------	----------	----------	----------	----------	----------	-
Murinae	----------	----------	----------	----------	----------	----------	----------	-
Canis_lupus_familiaris	----------	----------	----------	----------	----------	----------	----------	-
Sus_scrofa	----------	----------	----------	----------	----------	----------	----------	-
Choloepus_hoffmanni	----------	----------	----------	----------	----------	----------	----------	-
Vicugna_pacos	----------	----------	----------	----------	----------	----------	----------	-
Gavialis_gangeticus	----------	----------	----------	----------	----------	----------	----------	-
Mammalia	----------	----------	----------	----------	----------	----------	----------	-
Passeroidea	----------	----------	----------	----------	----------	----------	----------	-
Takifugu	----------	----------	----------	----------	----------	----------	----------	-
Pteropus_vampyrus	----------	----------	----------	----------	----------	----------	----------	-
Ornithorhynchus	GGCCCCTTGT	GGAAGGGGCT	GCCACGGGGA	TGGTCTCTTT	GTCTTTCTTC	TTGAGACCTC	ACCTGGGAAG	257
Oryctolagus_cuniculus	----------	----------	----------	----------	----------	----------	----------	-
Gallus_gallus	----------	----------	----------	----------	----------	----------	----------	-
Hominidae	CCGTTGATCC	AATAGTATGG	GACATCTCAA	CCCCCTCCAT	CGCCCGACAC	CATGCCCCAA	TAATGATCAA	2425
Metatheria	----------	----------	----------	----------	----------	----------	----------	-
Rodentia	----------	----------	CTCATNTTGC	TGCCGTGACT	CGGATAGGCT	CTCCTGGTGC	CTGTGCTCCC	50
Monodelphis	----------	----------	----------	----------	----------	----------	----------	-
Consensus	----------	----------	----------	----------	----------	----------	----------	

Conservation
100%
0%

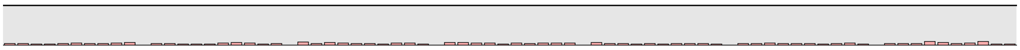

		6,180		6,200		6,220		
Dipodomys_ordii	----------	----------	----------	----------	----------	----------	----------	-
Laurasiatheria	----------	----------	----------	----------	----------	----------	----------	-
Eutheria	----------	----------	----------	----------	----------	----------	----------	-
Tarsius_syrichta	----------	----------	----------	----------	----------	----------	----------	-
Muridae	AAGACCTGTG	CCCCCTCCTG	TTGGGGACCT	GAAATCCTTT	TGNCTCTGCC	AGAGNAACCC	AGAAAACCTC	568
Gasterosteus_aculeatus	TGTTGCTNTG	CAGCCGTGTN	CTNAGAAGGG	AGGAGGTCNN	CTCNATTNAT	NGAAGNTTCA	AANCANNGGA	225
Felis_silvestris	----------	----------	----------	----------	----------	----------	----------	-
Rattus	----------	----------	----------	----------	----------	----------	----------	-
Papio_hamadryas	CCCCAGCCCT	TGGGTTACCG	GACACCTCAA	AGCCCTTTAC	CCTCTTCCTG	GACGAAAGGC	AAGGGATTGC	3527
Pan_troglodytes	GCGCCGCACT	AGGACTACCT	GACNTTACCA	AGCCCTTCCA	CTTATACGTG	GATGAGAATA	AGGGNNTCGC	1604
Ailuropoda_melanoleuca	----------	----------	----------	----------	----------	----------	----------	-
Cavia_porcellus	----------	----------	----------	----------	----------	----------	----------	-
Ictidomys_tridecemlineatus	----------	----------	----------	----------	----------	----------	----------	-
Canis	----------	----------	----------	----------	----------	----------	----------	-
Felis_catus	----------	----------	----------	----------	----------	----------	----------	-
Ochotona_princeps	----------	----------	----------	----------	----------	----------	----------	-
Cercopithecidae	----------	----------	----------	----------	----------	----------	----------	-
Carnivora	----------	----------	----------	----------	----------	----------	----------	-
Mus	----------	----------	----------	----------	----------	----------	----------	-
Mus_musculus	----------	----------	----------	----------	----------	----------	----------	-
Rattus_norvegicus	----------	----------	----------	----------	----------	----------	----------	-
Tursiops_truncatus	----------	----------	----------	----------	----------	----------	----------	-
Procavia_capensis	----------	----------	----------	----------	----------	----------	----------	-
Primates	----------	----------	----------	----------	----------	----------	----------	-
Echinops_telfairi	----------	----------	----------	----------	----------	----------	----------	-
Sorex_araneus	----------	----------	----------	----------	----------	----------	----------	-
Canidae	----------	----------	----------	----------	----------	----------	----------	-
Otolemur_garnettii	----------	----------	----------	----------	----------	----------	----------	-
Gallus	----------	----------	----------	----------	----------	----------	----------	-
Callithrix_jacchus	----------	----------	----------	----------	----------	----------	----------	-
Crocodylus_porosus	----------	----------	----------	----------	----------	----------	----------	-
Erinaceus_europaeus	----------	----------	----------	----------	----------	----------	----------	-
rnd-6_family-7426-LTR-ERV1	----------	----------	----------	----------	----------	----------	----------	-
Esox_lucius	----------	----------	----------	----------	----------	----------	----------	-
Salmo_salar	----------	----------	----------	----------	----------	----------	----------	-
Didelphidae	----------	----------	----------	----------	----------	----------	----------	-
Latimeria_chalumnae	----------	----------	----------	----------	----------	----------	----------	-
Monodelphis_domestica	----------	----------	----------	----------	----------	----------	----------	-
Macropus_eugenii	----------	----------	----------	----------	----------	----------	----------	-
Alligator_mississippiensis	----------	----------	----------	----------	----------	----------	----------	-
Chrysemys_picta_bellii	CGCCCAAAGC	CATCAAACCC	GTTATTTCAA	ACGCGTCATA	CCTAAGAAGC	GGGATGATGA	TTCTAAGAGG	1016
Danio_rerio	----------	----------	----------	----------	----------	----------	----------	-
Taeniopygia	----------	----------	----------	----------	----------	----------	----------	-
Dasypus_novemcinctus	----------	----------	----------	----------	----------	----------	----------	-
LTR_XT	----------	----------	----------	----------	----------	----------	----------	-
Haplorrhini	ATATTGTGAT	CATGTCTACC	CCTACCGCTG	TTAAAGTTGC	AGGTATTACA	CCTTGGATCC	ATCACAGCCG	6151
Catarrhini	AGACCGTCAT	CCTGACCACC	CCCACGGCTG	TAAAGGTAGA	AGGAATCCCA	GCCTGGATCC	ACCACAGCCG	5626
Anolis_carolinensis	----------	----------	----------	----------	----------	----------	----------	1184
Murine_leukemia_virus	ACACCGTCCT	GCTGACCACC	CCCACCGCTC	TCAAAGTAGA	CGGCATCTCT	GCGTGGATAC	ACGCCGCTCA	5610
Phascolarctos_cinereus	ACCTGGTGCT	ACTGACCACC	CCGACCGCGG	TGAAGGTTGA	CGGTATTGCT	GCCTGGGTCC	ATGCTTCTCA	5413
Estrildidae	----------	----------	----------	----------	----------	----------	----------	-
Xenopus_Silurana_tropicalis	----------	----------	----------	----------	----------	----------	----------	-
I_XT	NATAAGGTAT	TTCCNGAAAC	AATATGGCCG	AAANCCTNAN	AAGAGCANNA	ATTCCAGTGA	CAGANNCTGA	5708
Takifugu_rubripes	----------	----------	----------	----------	----------	----------	----------	-
Bos_taurus	----------	----------	----------	----------	----------	----------	----------	-
Homo_sapiens	----------	----------	----------	----------	----------	----------	----------	-
Microcebus_murinus	----------	----------	----------	----------	----------	----------	----------	-
Myotis_lucifugus	----------	----------	----------	----------	----------	----------	----------	-
Equus_caballus	----------	----------	----------	----------	----------	----------	----------	-
Tupaia_belangeri	----------	----------	----------	----------	----------	----------	----------	-
Loxodonta_africana	----------	----------	----------	----------	----------	----------	----------	-
Murinae	----------	----------	----------	----------	----------	----------	----------	-
Canis_lupus_familiaris	----------	----------	----------	----------	----------	----------	----------	-
Sus_scrofa	----------	----------	----------	----------	----------	----------	----------	-
Choloepus_hoffmanni	----------	----------	----------	----------	----------	----------	----------	-
Vicugna_pacos	----------	----------	----------	----------	----------	----------	----------	-
Gavialis_gangeticus	----------	----------	----------	----------	----------	----------	----------	-
Mammalia	----------	----------	----------	----------	----------	----------	----------	-
Passeroidea	----------	----------	----------	----------	----------	----------	----------	-
Takifugu	----------	----------	----------	----------	----------	----------	----------	-
Pteropus_vampyrus	----------	----------	----------	----------	----------	----------	----------	-
Ornithorhynchus	GGACCGAGAG	GGAGACCATT	CCTTTTTTCT	TACTTTAACC	CCTGATGTGG	ATTATGGCAA	GGCTCTTGTC	327
Oryctolagus_cuniculus	----------	----------	----------	----------	----------	----------	----------	-
Gallus_gallus	----------	----------	----------	----------	----------	----------	----------	-
Hominidae	ACTCAAGGAC	CCTACCAAAT	TTCCCTCGCG	GCCACAATTC	CCCATCTCAG	TTGAACACCG	CCAAGGGTTA	2495
Metatheria	----------	----------	----------	----------	----------	----------	----------	-
Rodentia	CCTTGGGGTC	TGAGCCGCAC	TAGGACCCTA	TCCCTCATCT	CCAGTCNACC	TGCAACAGAC	TCAC--CTTC	118
Monodelphis	----------	----------	----------	----------	----------	----------	----------	-
Consensus	----------	----------	----------	----------	----------	----------	----------	

Conservation

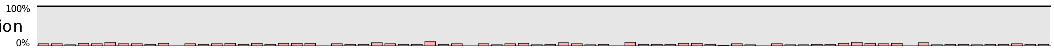

		6,240		6,260		6,280		6,300	
Dipodomys_ordii		----------	----------	----------	----------	----------	----------	----------	-
Laurasiatheria		----------	----------	----------	----------	----------	----------	----------	-
Eutheria		----------	----------	----------	----------	----------	----------	----------	-
Tarsius_syrichta		----------	----------	----------	----------	----------	----------	----------	-
Muridae		CACCTGTCCC	ACTCTGGGAG	CAGGAGCGCT	TGCTTGTTTT	GTTGCTTGAC	TCCTGCTCAC	GGTCAGGGTT	638
Gasterosteus_aculeatus		CTTCTGTNCA	TGNCCGCGGG	ATATNTTATT	NNGTANGNAA	GNTCNGGATN	NAAGTGTCNA	NTTNATGGCA	295
Felis_silvestris		----------	----------	----------	----------	----------	----------	----------	-
Rattus		----------	----------	----------	----------	----------	----------	----------	-
Papio_hamadryas		CAAAGGAGTC	CTGACCCAAA	AATTGGGGCC	TTGGAAAAGA	CCGGTAGCAT	ACCTGTCCAA	GAAGCTGGAC	3597
Pan_troglodytes		CAAGGCGGTA	ATAACTCAAA	ANTTAGGNCC	CTGGANGAGN	CCAGTTGCCT	ACCTGTCAAA	GAAACTNGAC	1674
Ailuropoda_melanoleuca		----------	----------	----------	----------	----------	----------	----------	-
Cavia_porcellus		----------	----------	----------	----------	----------	----------	----------	-
Ictidomys_tridecemlineatus		----------	----------	----------	----------	----------	----------	----------	-
Canis		----------	----------	----------	----------	----------	----------	----------	-
Felis_catus		----------	----------	----------	----------	----------	----------	----------	-
Ochotona_princeps		----------	----------	----------	----------	----------	----------	----------	-
Cercopithecidae		----------	----------	----------	----------	----------	----------	----------	-
Carnivora		----------	----------	----------	----------	----------	----------	----------	-
Mus		----------	----------	----------	----------	----------	----------	----------	-
Mus_musculus		----------	----------	----------	----------	----------	----------	----------	-
Rattus_norvegicus		----------	----------	----------	----------	----------	----------	----------	-
Tursiops_truncatus		----------	----------	----------	----------	----------	----------	----------	-
Procavia_capensis		----------	----------	----------	----------	----------	----------	----------	-
Primates		----------	----------	----------	----------	----------	----------	----------	-
Echinops_telfairi		----------	----------	----------	----------	----------	----------	----------	-
Sorex_araneus		----------	----------	----------	----------	----------	----------	----------	-
Canidae		----------	----------	----------	----------	----------	----------	----------	-
Otolemur_garnettii		----------	----------	----------	----------	----------	----------	----------	-
Gallus		----------	----------	----------	----------	----------	----------	----------	-
Callithrix_jacchus		----------	----------	----------	----------	----------	----------	----------	-
Crocodylus_porosus		----------	----------	----------	----------	----------	----------	----------	-
Erinaceus_europaeus		----------	----------	----------	----------	----------	----------	----------	-
rnd-6_family-7426-LTR-ERV1		----------	----------	----------	----------	----------	----------	----------	-
Esox_lucius		----------	----------	----------	----------	----------	----------	----------	-
Salmo_salar		----------	----------	----------	----------	----------	----------	----------	-
Didelphidae		----------	----------	----------	----------	----------	----------	----------	-
Latimeria_chalumnae		----------	----------	----------	----------	----------	----------	----------	-
Monodelphis_domestica		----------	----------	----------	----------	----------	----------	----------	-
Macropus_eugenii		----------	----------	----------	----------	----------	----------	----------	-
Alligator_mississippiensis		----------	----------	----------	----------	----------	----------	----------	-
Chrysemys_picta_bellii		TTCGACCAGT	CCTTGAACAC	CCATCTCCGT	ACAAAAGCCA	TGGACTATCG	GTGTCAGGAC	TGGCTAATGG	1086
Danio_rerio		----------	----------	----------	----------	----------	----------	----------	-
Taeniopygia		----------	----------	----------	----------	----------	----------	----------	-
Dasypus_novemcinctus		----------	----------	----------	----------	----------	----------	----------	-
LTR_XT		----------	----------	----------	----------	----------	----------	----------	-
Haplorrhini		ACTGAAACCT	GCAGCCTCAG	TTCAAGACCG	GTGGACGAGT	CAGCAAGATC	CAGATCATCC	AACTCGACTG	6221
Catarrhini		TGTGAAACCT	GCAGCC---G	CTGAAACCTG	GGAG------	--GCAAAACC	GAGCCCGGAC	AAC-------	5678
Anolis_carolinensis		----------	----------	----------	----------	----------	----------	----------	1184
Murine_leukemia_virus		CGTAAAGGCG	GCGACAACTC	CTCCGGCCGG	AACAGC----	ATGGAAGGTC	CAGCGTTCTC	AAA-------	5669
Phascolarctos_cinereus		TCTCAAACCC	GCGCCACCTG	GGGCACCCGA	TGAGTC----	CTGGGAACTG	GAAAAGACTG	ATC-------	5472
Estrildidae		----------	----------	----------	----------	----------	----------	----------	-
Xenopus_Silurana_tropicalis		----------	----------	----------	----------	----------	----------	----------	-
I_XT		ANNTGTCNTA	CACNCTTAAA	CTTACCATCA	AANTCAANAA	TATTNATATC	AACANAAAGA	TAACTAAGGA	5778
Takifugu_rubripes		----------	----------	----------	----------	----------	----------	----------	-
Bos_taurus		----------	----------	----------	----------	----------	----------	----------	-
Homo_sapiens		----------	----------	----------	----------	----------	----------	----------	-
Microcebus_murinus		----------	----------	----------	----------	----------	----------	----------	-
Myotis_lucifugus		----------	----------	----------	----------	----------	----------	----------	-
Equus_caballus		----------	----------	----------	----------	----------	----------	----------	-
Tupaia_belangeri		----------	----------	----------	----------	----------	----------	----------	-
Loxodonta_africana		----------	----------	----------	----------	----------	----------	----------	-
Murinae		----------	----------	----------	----------	----------	----------	----------	-
Canis_lupus_familiaris		----------	----------	----------	----------	----------	----------	----------	-
Sus_scrofa		----------	----------	----------	----------	----------	----------	----------	-
Choloepus_hoffmanni		----------	----------	----------	----------	----------	----------	----------	-
Vicugna_pacos		----------	----------	----------	----------	----------	----------	----------	-
Gavialis_gangeticus		----------	----------	----------	----------	----------	----------	----------	-
Mammalia		----------	----------	----------	----------	----------	----------	----------	-
Passeroidea		----------	----------	----------	----------	----------	----------	----------	-
Takifugu		----------	----------	----------	----------	----------	----------	----------	-
Pteropus_vampyrus		----------	----------	----------	----------	----------	----------	----------	-
Ornithorhynchus		CCTCCACCCC	AATTTTTTTA	AGACCCTGAG	GTATTAATTA	ATGTAAAAAT	TGGAAAATCC	CGCAGGAGAT	397
Oryctolagus_cuniculus		----------	----------	----------	----------	----------	----------	----------	-
Gallus_gallus		----------	----------	----------	----------	----------	----------	----------	-
Hominidae		AAACCTATCA	TCACCAGGCT	CTTGCAACAA	CACATCCTTA	TCCCGGTAAA	CTCCCGTTGC	AACACGCCCA	2565
Metatheria		----------	----------	----------	----------	----------	----------	----------	-
Rodentia		CGGCTCACCG	ATCGCTTGGC	ACCCCATCCA	C-CAGCGGCA	ATTAGGTAAG	CTTCCCCTTT	TGGTCGGTGT	187
Monodelphis		----------	----------	----------	----------	----------	----------	----------	-
Consensus		----------	----------	----------	----------	----------	----------	----------	

Conservation

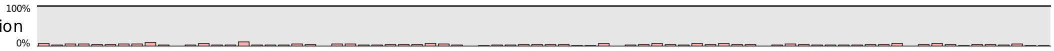

	6,320		6,340		6,360			
Dipodomys_ordii	----------	----------	----------	----------	----------	----------	----------	-
Laurasiatheria	----------	----------	----------	----------	----------	----------	----------	-
Eutheria	----------	----------	----------	----------	----------	----------	----------	-
Tarsius_syrichta	----------	----------	----------	----------	----------	----------	----------	-
Muridae	GGTCCCTGTG	TGGAGGTGGG	GAGGGGCTGG	GCCTCTCATC	AAGGCCCCCT	GCCATCTAAG	CGGAATGCAC	708
Gasterosteus_aculeatus	ATNAATNAGA	TTNNCAGCGG	CCACTGAGGA	NGATGATGTG	TGTGNNNGCT	GCCACNGATN	CACCGGGTTC	365
Felis_silvestris	----------	----------	----------	----------	----------	----------	----------	-
Rattus	----------	----------	----------	----------	----------	----------	----------	-
Papio_hamadryas	CCTGTGGCGG	CCGGCTGGCC	CCCATGTCTT	CGTATCATGG	CAGCCACCGC	TATGCTGGTC	AAAGACTCTG	3667
Pan_troglodytes	CCAGTAGCTG	CCGGGTGGCC	CCCTTGTCTC	CG-ATGATTG	CGGCCACGGC	TATGNTNGTC	AAAGACACTG	1743
Ailuropoda_melanoleuca	----------	----------	----------	----------	----------	----------	----------	-
Cavia_porcellus	----------	----------	----------	----------	----------	----------	----------	-
Ictidomys_tridecemlineatus	----------	----------	----------	----------	----------	----------	----------	-
Canis	----------	----------	----------	----------	----------	----------	----------	-
Felis_catus	----------	----------	----------	----------	----------	----------	----------	-
Ochotona_princeps	----------	----------	----------	----------	----------	----------	----------	-
Cercopithecidae	----------	----------	----------	----------	----------	----------	----------	-
Carnivora	----------	----------	----------	----------	----------	----------	----------	-
Mus	----------	----------	----------	----------	----------	----------	----------	-
Mus_musculus	----------	----------	----------	----------	----------	----------	----------	-
Rattus_norvegicus	----------	----------	----------	----------	----------	----------	----------	-
Tursiops_truncatus	----------	----------	----------	----------	----------	----------	----------	-
Procavia_capensis	----------	----------	----------	----------	----------	----------	----------	-
Primates	----------	----------	----------	----------	----------	----------	----------	-
Echinops_telfairi	----------	----------	----------	----------	----------	----------	----------	-
Sorex_araneus	----------	----------	----------	----------	----------	----------	----------	-
Canidae	----------	----------	----------	----------	----------	----------	----------	-
Otolemur_garnettii	----------	----------	----------	----------	----------	----------	----------	-
Gallus	----------	----------	----------	----------	----------	----------	----------	-
Callithrix_jacchus	----------	----------	----------	----------	----------	----------	----------	-
Crocodylus_porosus	----------	----------	----------	----------	----------	----------	----------	-
Erinaceus_europaeus	----------	----------	----------	----------	----------	----------	----------	-
rnd-6_family-7426-LTR-ERV1	----------	----------	----------	----------	----------	----------	----------	-
Esox_lucius	----------	----------	----------	----------	----------	----------	----------	-
Salmo_salar	----------	----------	----------	----------	----------	----------	----------	-
Didelphidae	----------	----------	----------	----------	----------	----------	----------	-
Latimeria_chalumnae	----------	----------	----------	----------	----------	----------	----------	-
Monodelphis_domestica	----------	----------	----------	----------	----------	----------	----------	-
Macropus_eugenii	----------	----------	----------	----------	----------	----------	----------	-
Alligator_mississippiensis	----------	----------	----------	----------	----------	----------	----------	-
Chrysemys_picta_bellii	TTAGCTCCTT	TCCAAACCTA	CATTGGCTCT	ATACCATTTC	GTTGATTTGC	TTTCTTCATC	CGTACCATCC	1156
Danio_rerio	----------	----------	----------	----------	----------	----------	----------	-
Taeniopygia	----------	----------	----------	----------	----------	----------	----------	-
Dasypus_novemcinctus	----------	----------	----------	----------	----------	----------	----------	-
LTR_XT	----------	----------	----------	----------	----------	----------	----------	-
Haplorrhini	ATCTTGCAGA	GGAACCAAGG	CGCAGCAGAA	AAAGACGACT	GCCCTGCTCC	GACCACACCG	GAGGCTGGTC	6291
Catarrhini	-CCCTGCAAA	GTGACT----	-----CTGAG	GAGGACGAC-	----------	----------	----------	5707
Anolis_carolinensis	----------	----------	----------	----------	----------	----------	----------	1184
Murine_leukemia_virus	ACCCCTTAAA	GATAAG-ATT	AACCCGTG--	GGGCCCCCTG	AT--------	----------	----------	5708
Phascolarctos_cinereus	ATCCTCTTAA	GTTGCGTGTT	CGGCGGCGCC	GAAATGAATC	AA--------	----------	----------	5514
Estrildidae	----------	----------	----------	----------	----------	----------	----------	-
Xenopus_Silurana_tropicalis	----------	----------	----------	----------	----------	----------	----------	-
I_XT	NGCATTACCN	NTCNTTCNGA	AATCAGGATN	ANAAAAANAN	ATCATTAAGT	AAGTTAGACN	AGAGTAGTAA	5848
Takifugu_rubripes	----------	----------	----------	----------	----------	----------	----------	-
Bos_taurus	----------	----------	----------	----------	----------	----------	----------	-
Homo_sapiens	----------	----------	----------	----------	----------	----------	----------	-
Microcebus_murinus	----------	----------	----------	----------	----------	----------	----------	-
Myotis_lucifugus	----------	----------	----------	----------	----------	----------	----------	-
Equus_caballus	----------	----------	----------	----------	----------	----------	----------	-
Tupaia_belangeri	----------	----------	----------	----------	----------	----------	----------	-
Loxodonta_africana	----------	----------	----------	----------	----------	----------	----------	-
Murinae	----------	----------	----------	----------	----------	----------	----------	-
Canis_lupus_familiaris	----------	----------	----------	----------	----------	----------	----------	-
Sus_scrofa	----------	----------	----------	----------	----------	----------	----------	-
Choloepus_hoffmanni	----------	----------	----------	----------	----------	----------	----------	-
Vicugna_pacos	----------	----------	----------	----------	----------	----------	----------	-
Gavialis_gangeticus	----------	----------	----------	----------	----------	----------	----------	-
Mammalia	----------	----------	----------	----------	----------	----------	----------	-
Passeroidea	----------	----------	----------	----------	----------	----------	----------	-
Takifugu	----------	----------	----------	----------	----------	----------	----------	-
Pteropus_vampyrus	----------	----------	----------	----------	----------	----------	----------	-
Ornithorhynchus	AAAAGCCCTT	ATAAACTATT	TAGCAGAGAT	GAGGAAAAAT	TTATAATATT	CCTTCGGAAG	AAAATCCCGA	467
Oryctolagus_cuniculus	----------	----------	----------	----------	----------	----------	----------	-
Gallus_gallus	----------	----------	----------	----------	----------	----------	----------	-
Hominidae	TTCTGCCCAT	CCGTAAGGCC	TCTGGCGCGT	ACCGTTTAGT	GCAAGATCTT	CGCATCATCA	ACGAGGCTNT	2635
Metatheria	----------	----------	----------	----------	----------	----------	----------	-
Rodentia	AAATGCTTCC	CTGAGTCCGA	GGAAGTGACT	GTTGAGCGTC	CGGCACCCCT	CTGGTACTCT	TGGAGGTGGA	257
Monodelphis	----------	----------	----------	----------	----------	----------	----------	-
Consensus	----------	----------	----------	----------	----------	----------	----------	

Conservation

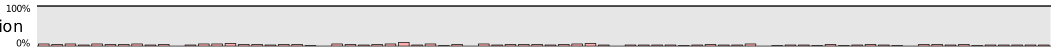

6,380 | 6,400 | 6,420 | 6,440

Sequence								
Dipodomys_ordii	----------	----------	----------	----------	----------	----------	----------	-
Laurasiatheria	----------	----------	----------	----------	----------	----------	----------	-
Eutheria	----------	----------	----------	----------	----------	----------	----------	-
Tarsius_syrichta	----------	----------	----------	----------	----------	----------	----------	-
Muridae	ACAGGGAGGG	GGAAATAGTT	CCCCCAGGTG	AGCATTCTCC	TCTCCTCTGC	GCCTGCTCTA	AGCAGCCCCA	778
Gasterosteus_aculeatus	NAATCTGANC	ATGATTNGTA	GCNACCGGAA	CTGGCACGGA	GANTGTCCTT	CACATGGTAN	AAAAACTCAG	435
Felis_silvestris	----------	----------	----------	----------	----------	----------	----------	-
Rattus	----------	----------	----------	----------	----------	----------	----------	-
Papio_hamadryas	CTAAGTTAAC	CCTTGGGCAG	CCACTGACTG	TTATTACTCC	ACATACTCTA	GAGGCCATAG	TGCGGCAGCC	3737
Pan_troglodytes	ATAAGCTTAN	CNTNGGGCAA	GAATTGCCGG	TCNTTACCCC	ACATGC-CTC	GAAGCTANAG	TCANACAGCC	1812
Ailuropoda_melanoleuca	----------	----------	----------	----------	----------	----------	----------	-
Cavia_porcellus	----------	----------	----------	----------	----------	----------	----------	-
Ictidomys_tridecemlineatus	----------	----------	----------	----------	----------	----------	----------	-
Canis	----------	----------	----------	----------	----------	----------	----------	-
Felis_catus	----------	----------	----------	----------	----------	----------	----------	-
Ochotona_princeps	----------	----------	----------	----------	----------	----------	----------	-
Cercopithecidae	----------	----------	----------	----------	----------	----------	----------	-
Carnivora	----------	----------	----------	----------	----------	----------	----------	-
Mus	----------	----------	----------	----------	----------	----------	----------	-
Mus_musculus	----------	----------	----------	----------	----------	----------	----------	-
Rattus_norvegicus	----------	----------	----------	----------	----------	----------	----------	-
Tursiops_truncatus	----------	----------	----------	----------	----------	----------	----------	-
Procavia_capensis	----------	----------	----------	----------	----------	----------	----------	-
Primates	----------	----------	----------	----------	----------	----------	----------	-
Echinops_telfairi	----------	----------	----------	----------	----------	----------	----------	-
Sorex_araneus	----------	----------	----------	----------	----------	----------	----------	-
Canidae	----------	----------	----------	----------	----------	----------	----------	-
Otolemur_garnettii	----------	----------	----------	----------	----------	----------	----------	-
Gallus	----------	----------	----------	----------	----------	----------	----------	-
Callithrix_jacchus	----------	----------	----------	----------	----------	----------	----------	-
Crocodylus_porosus	----------	----------	----------	----------	----------	----------	----------	-
Erinaceus_europaeus	----------	----------	----------	----------	----------	----------	----------	-
rnd-6_family-7426-LTR-ERV1	----------	----------	----------	----------	----------	----------	----------	-
Esox_lucius	----------	----------	----------	----------	----------	----------	----------	-
Salmo_salar	----------	----------	----------	----------	----------	----------	----------	-
Didelphidae	----------	----------	----------	----------	----------	----------	----------	-
Latimeria_chalumnae	----------	----------	----------	----------	----------	----------	----------	-
Monodelphis_domestica	----------	----------	----------	----------	----------	----------	----------	-
Macropus_eugenii	----------	----------	----------	----------	----------	----------	----------	-
Alligator_mississippiensis	----------	----------	----------	----------	----------	----------	----------	-
Chrysemys_picta_bellii	AGATTTCCTT	TAGGAAGCAG	CTGGACTTCC	AGGGTTAATC	CCCCCTTTCC	AGCCTGCCAG	AGACTGGAGA	1226
Danio_rerio	----------	----------	----------	----------	----------	----------	----------	-
Taeniopygia	----------	----------	----------	----------	----------	----------	----------	-
Dasypus_novemcinctus	----------	----------	----------	----------	----------	----------	----------	-
LTR_XT	----------	----------	----------	----------	----------	----------	----------	-
Haplorrhini	GGTCAACGCA	CGGCTGAAGC	TTGAGGAAAC	GTCAAGCCCT	GCTCTAGTCA	CACAACTGGA	AGCTGACTAG	6361
Catarrhini	----------	----------	----------	---AAGCCCT	GCTCCAGTCA	CAC--CCGGA	AGCTGACTGG	5742
Anolis_carolinensis	----------	----------	----------	----------	----------	----------	----------	1184
Murine_leukemia_virus	----------	----------	------AGTT	ATGGGGATCT	TGGTGAGGGC	AGGAGCCTCG	GTACAACGTG	5752
Phascolarctos_cinereus	----------	----------	------CAGC	ATAACAACCC	TCACCAACCC	ATGACTCTCA	CCTGGCAGGT	5558
Estrildidae	----------	----------	----------	----------	----------	----------	----------	-
Xenopus_Silurana_tropicalis	----------	----------	----------	----------	----------	----------	----------	-
I_XT	CANTAATCCA	CACTAGTCTG	ATAACCAACG	NTTGNAGATT	AAGGTNNCGT	ATCAATGGTG	GGCNTANTCT	5918
Takifugu_rubripes	----------	----------	----------	----------	----------	----------	----------	-
Bos_taurus	----------	----------	----------	----------	----------	----------	----------	-
Homo_sapiens	----------	----------	----------	----------	----------	----------	----------	-
Microcebus_murinus	----------	----------	----------	----------	----------	----------	----------	-
Myotis_lucifugus	----------	----------	----------	----------	----------	----------	----------	-
Equus_caballus	----------	----------	----------	----------	----------	----------	----------	-
Tupaia_belangeri	----------	----------	----------	----------	----------	----------	----------	-
Loxodonta_africana	----------	----------	----------	----------	----------	----------	----------	-
Murinae	----------	----------	----------	----------	----------	----------	----------	-
Canis_lupus_familiaris	----------	----------	----------	----------	----------	----------	----------	-
Sus_scrofa	----------	----------	----------	----------	----------	----------	----------	-
Choloepus_hoffmanni	----------	----------	----------	----------	----------	----------	----------	-
Vicugna_pacos	----------	----------	----------	----------	----------	----------	----------	-
Gavialis_gangeticus	----------	----------	----------	----------	----------	----------	----------	-
Mammalia	----------	----------	----------	----------	----------	----------	----------	-
Passeroidea	----------	----------	----------	----------	----------	----------	----------	-
Takifugu	----------	----------	----------	----------	----------	----------	----------	-
Pteropus_vampyrus	----------	----------	----------	----------	----------	----------	----------	-
Ornithorhynchus	ACGTCACTTC	GGAAATCTTA	TAGAAGAACG	GTATCTCTTC	CAGTTAGGAC	CAGACCCTAT	GGAGACCCTC	537
Oryctolagus_cuniculus	----------	----------	----------	----------	----------	----------	----------	-
Gallus_gallus	----------	----------	----------	----------	----------	----------	----------	-
Hominidae	NGTNCCNANN	NTTCCNGNAG	NGNNNANCNC	NTANNCNCNC	CNANCCCGCA	NNCNNNNGNN	NACNNCCCNN	2705
Metatheria	----------	----------	----------	----------	----------	----------	----------	-
Rodentia	GGTACCCCCA	GCCACGGT--	CTTTAAGGCT	ACGGCTGGCC	CTCTTCACCT	GACCCCTACC	CTCTCCCACC	325
Monodelphis	----------	----------	----------	----------	----------	----------	----------	-
Consensus	----------	----------	----------	----------	----------	----------	----------	

Conservation

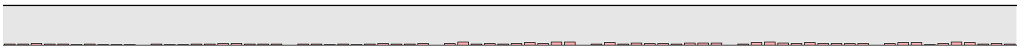

		6,460		6,480		6,500		
Dipodomys_ordii	----------	----------	----------	----------	----------	----------	----------	-
Laurasiatheria	----------	----------	----------	----------	----------	----------	----------	-
Eutheria	----------	----------	----------	----------	----------	----------	----------	-
Tarsius_syrichta	----------	----------	----------	----------	----------	----------	----------	-
Muridae	GGGAAGGGGA	AAAGTCCCCT	GGGCTGAGCC	CGAAGGAAAA	GAGAAAGTTC	CACTCCTTTC	TCTTTTATGA	848
Gasterosteus_aculeatus	CGTCTTCACN	GCTCGGAGAC	GCATTACAAC	NGANCGCTGA	GATACGNGNT	TGTATNGNAC	NTCCTGCTNG	505
Felis_silvestris	----------	----------	----------	----------	----------	----------	----------	-
Rattus	----------	----------	----------	----------	----------	----------	----------	-
Papio_hamadryas	CCCGGACCGG	TGGATAACCA	ACGCACGCCT	AACCCACTAC	CAGGCCCTCT	TACTGGACAC	GGACCGCGTC	3807
Pan_troglodytes	ACCTAAACGA	TNGATCACNA	ACGCCCGGCT	NACCCACTAC	CAAGCACTAN	TACTNAACCC	GCTCAGNATA	1882
Ailuropoda_melanoleuca	----------	----------	----------	----------	----------	----------	----------	-
Cavia_porcellus	----------	----------	----------	----------	----------	----------	----------	-
Ictidomys_tridecemlineatus	----------	----------	----------	----------	----------	----------	----------	-
Canis	----------	----------	----------	----------	----------	----------	----------	-
Felis_catus	----------	----------	----------	----------	----------	----------	----------	-
Ochotona_princeps	----------	----------	----------	----------	----------	----------	----------	-
Cercopithecidae	----------	----------	----------	----------	----------	----------	----------	-
Carnivora	----------	----------	----------	----------	----------	----------	----------	-
Mus	----------	----------	----------	-------GGG	GGGGTAATGT	TTTGTCTTGT	TTGTTGACGT	33
Mus_musculus	----------	----------	----------	----------	----------	----------	----------	-
Rattus_norvegicus	----------	----------	----------	----------	----------	----------	----------	-
Tursiops_truncatus	----------	----------	----------	----------	----------	----------	----------	-
Procavia_capensis	----------	----------	----------	----------	----------	----------	----------	-
Primates	----------	----------	----------	----------	----------	----------	----------	-
Echinops_telfairi	----------	----------	----------	----------	----------	----------	----------	-
Sorex_araneus	----------	----------	----------	----------	----------	----------	----------	-
Canidae	----------	----------	----------	----------	----------	----------	----------	-
Otolemur_garnettii	----------	----------	----------	----------	----------	----------	----------	-
Gallus	----------	----------	----------	----------	----------	----------	----------	-
Callithrix_jacchus	----------	----------	----------	----------	----------	----------	----------	-
Crocodylus_porosus	----------	----------	----------	----------	----------	----------	----------	-
Erinaceus_europaeus	----------	----------	----------	----------	----------	----------	----------	-
rnd-6_family-7426-LTR-ERV1	----------	----------	----------	----------	----------	----------	----------	-
Esox_lucius	----------	----------	----------	----------	----------	----------	----------	-
Salmo_salar	----------	----------	----------	----------	----------	----------	----------	-
Didelphidae	----------	----------	----------	----------	----------	----------	----------	-
Latimeria_chalumnae	----------	-----CCCCT	TTACACTAAG	AACGAATATC	TCCATCGATA	TGAGGAGTCT	CAGCAAAAAG	55
Monodelphis_domestica	----------	----------	----------	----------	----------	----------	----------	-
Macropus_eugenii	----------	----------	----------	----------	----------	----------	----------	-
Alligator_mississippiensis	----------	----------	----------	----------	----------	----------	----------	-
Chrysemys_picta_bellii	GGTTGCACTC	TTCAATATGT	AGATGATTTG	CCTTTCTTGA	GACTTGACTC	CTGATTGAGG	ACGGACCAAG	1296
Danio_rerio	----------	----------	----------	----------	----------	----------	----------	-
Taeniopygia	----------	----------	----------	----------	----------	----------	----------	-
Dasypus_novemcinctus	----------	----------	----------	----------	----------	----------	----------	-
LTR_XT	----------	----------	----------	----------	----------	----------	----------	-
Haplorrhini	TCTACGCATG	GCTGAAGCTT	GAGGAAACGT	CAAGCCCTGC	TCTAGTCACA	CAACCGGAAG	CTGACTAGTC	6431
Catarrhini	TCTACGCACG	GCCGAAGCAT	GAGGAGA---	----------	---------A	TCATCGTGGG	---ACTCATT	5787
Anolis_carolinensis	----------	----------	----------	----------	----------	----------	----------	1184
Murine_leukemia_virus	ACAGCCCTCA	CCAGGTCTTC	AATGTCACTT	GG--AGAGTT	ACCAACCTAA	TGACAGGACA	AACAGCTAAC	5820
Phascolarctos_cinereus	ACTGTCCCAG	ACGGGA----	AGTGTCGTTT	GGGAAAAGAA	AGCAGTCGAG	CCACCCTGGA	CGTGGTGGCC	5624
Estrildidae	----------	----------	----------	----------	----------	----------	----------	-
Xenopus_Silurana_tropicalis	----------	----------	----------	----------	----------	----------	----------	-
I_XT	TGNCGAATAN	GANTATATCA	TNANCAAGNA	AAAGNGACCT	TTACAAAACA	ATGACTCAAT	ATTTGCTNTT	5988
Takifugu_rubripes	----------	----------	----------	----------	----------	----------	----------	-
Bos_taurus	----------	----------	----------	----------	----------	----------	----------	-
Homo_sapiens	----------	----------	----------	----------	----------	----------	----------	-
Microcebus_murinus	----------	----------	----------	----------	----------	----------	----------	-
Myotis_lucifugus	----------	----------	----------	----------	----------	----------	----------	-
Equus_caballus	----------	----------	----------	----------	----------	----------	----------	-
Tupaia_belangeri	----------	----------	----------	----------	----------	----------	----------	-
Loxodonta_africana	----------	----------	----------	----------	----------	----------	----------	-
Murinae	----------	----------	----------	----------	----------	----------	----------	-
Canis_lupus_familiaris	----------	----------	----------	----------	----------	----------	----------	-
Sus_scrofa	----------	----------	----------	----------	----------	----------	----------	-
Choloepus_hoffmanni	----------	----------	----------	----------	----------	----------	----------	-
Vicugna_pacos	----------	----------	----------	----------	----------	----------	----------	-
Gavialis_gangeticus	----------	----------	----------	----------	----------	----------	----------	-
Mammalia	----------	----------	----------	----------	----------	----------	----------	-
Passeroidea	----------	----------	----------	----------	----------	----------	----------	-
Takifugu	----------	----------	----------	----------	----------	----------	----------	-
Pteropus_vampyrus	----------	----------	----------	----------	----------	----------	----------	-
Ornithorhynchus	TCCCTTGATG	TGTAAATATC	CAGATACTAA	TTTTCATGAA	AATTGAATAT	TAGATGAACC	AATGTGTCCT	607
Oryctolagus_cuniculus	----------	----------	----------	----------	----------	----------	----------	-
Gallus_gallus	----------	----------	----------	----------	----------	----------	----------	-
Hominidae	NTNNNNGNNN	NNGNCCTCNN	NNNNNNTNNN	NTCANNNTNC	NCCTCCACNN	NNANNNNTNC	NTNNNGTNCG	2775
Metatheria	----------	----------	----------	----------	----------	----------	----------	-
Rodentia	ACTGGAGCTG	CAATCCTGCA	GCTTCCTTAG	GCCCNACTGG	GCTATTGCAC	CCCTNCCTGC	CCTCTTCTCC	395
Monodelphis	----------	----------	----------	----------	----------	----------	----------	-
Consensus	----------	----------	----------	----------	----------	----------	----------	

Conservation

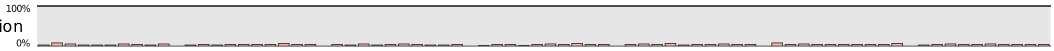

	6,520		6,540		6,560		6,580	
Dipodomys_ordii	----------	----------	----------	----------	----------	----------	----------	-
Laurasiatheria	----------	----------	----------	----------	----------	----------	----------	-
Eutheria	----------	----------	----------	----------	----------	----------	----------	-
Tarsius_syrichta	----------	----------	----------	----------	----------	----------	----------	-
Muridae	TTTATGTCTC	CTTATNAACC	AATGATCCGT	ATAATTGGAA	AACTCAAAAC	CCACCATTCT	CTGAAAAAAA	918
Gasterosteus_aculeatus	AAGCAATAAA	TAGAGCNNTA	NGNAGTTGGA	GTATTNGACA	GCAACTATTN	NATCGANAAA	NCANTCTTNT	575
Felis_silvestris	----------	----------	----------	----------	----------	----------	----------	-
Rattus	----------	----------	----------	----------	----------	----------	----------	-
Papio_hamadryas	CAGTTTGGCC	CTCCGGTCAC	CCTAAACCCT	GCTACGCTGC	TGCCGGTACC	AGAAAACCAA	CCAAGCCCAC	3877
Pan_troglodytes	ATTTTCNNCC	CACCAGTTAC	TTTAAANCCT	GCCGCNCTGC	TACCCNACCC	GGACANAGAT	GTGAGACNCC	1952
Ailuropoda_melanoleuca	----------	----------	----------	----------	----------	----------	----------	-
Cavia_porcellus	----------	----------	----------	----------	----------	----------	----------	-
Ictidomys_tridecemlineatus	----------	----------	----------	----------	----------	----------	----------	-
Canis	----------	----------	----------	----------	----------	----------	----------	-
Felis_catus	----------	----------	----------	----------	----------	----------	----------	-
Ochotona_princeps	----------	----------	----------	----------	----------	----------	----------	-
Cercopithecidae	----------	----------	-GCCGCTGGT	CTCGCGACCT	GGTCCCGACC	CGTCGGCGTC	TCTCTTAACC	49
Carnivora	----------	----------	----------	----------	----------	----------	----------	-
Mus	ATTGTTTTTA	GGTGAAGGTG	AGACCGAGGG	ACGGGGGCGG	CTGATACTGC	CCCTCGTTTT	GCAGGCTTTG	103
Mus_musculus	----------	----------	----------	----------	----------	----------	----------	-
Rattus_norvegicus	----------	----------	----------	----------	----------	----------	----------	-
Tursiops_truncatus	----------	----------	----------	----------	----------	----------	----------	-
Procavia_capensis	----------	----------	----------	----------	----------	----------	----------	-
Primates	----------	----------	----------	----------	----------	----------	----------	-
Echinops_telfairi	----------	----------	----------	----------	----------	----------	----------	-
Sorex_araneus	----------	----------	----------	----------	----------	----------	----------	-
Canidae	----------	----------	----------	----------	----------	----------	----------	-
Otolemur_garnettii	----------	----------	----------	----------	----------	----------	----------	-
Gallus	----------	----------	----------	----------	----------	----------	----------	-
Callithrix_jacchus	----------	----------	----------	----------	----------	----------	----------	-
Crocodylus_porosus	----------	----------	----------	----------	----------	----------	----------	-
Erinaceus_europaeus	----------	----------	----------	----------	----------	----------	----------	-
rnd-6_family-7426-LTR-ERV1	----------	----------	----------	----------	----------	----------	----------	-
Esox_lucius	----------	----------	----------	----------	----------	----------	----------	-
Salmo_salar	----------	----------	----------	----------	----------	----------	----------	-
Didelphidae	----------	----------	----------	----------	----------	----------	----------	-
Latimeria_chalumnae	ACAACCAAGC	ATCCGGCGAC	GGAACTTGTC	TCAATAAATC	CTCTTGAAAA	ATTAGCTCAT	GCCCATTTCA	125
Monodelphis_domestica	----------	----------	----------	----------	----------	----------	----------	-
Macropus_eugenii	----------	----------	----------	----------	----------	----------	----------	-
Alligator_mississippiensis	----------	----------	----------	----------	----------	----------	----------	-
Chrysemys_picta_bellii	CATCCGTCAT	ACTGGTTTCA	CATCAGGGGC	ACTTCAAGGA	AGCTATCATT	CCTACCGCAC	TCGTCGCTTT	1366
Danio_rerio	----------	----------	----------	----------	----------	----------	----------	-
Taeniopygia	----------	----------	----------	----------	----------	----------	----------	-
Dasypus_novemcinctus	----------	----------	----------	----------	----------	----------	----------	-
LTR_XT	----------	----------	----------	----------	----------	----------	----------	-
Haplorrhini	TACGCACGGC	CGAAGCCTGA	GGAAGCCAGC	GCTAGATAAG	TAAATGTGGA	TTGAATTTGC	AAGTGTAGTT	6501
Catarrhini	TTCCT-----	----------	----------	----------	----TATAAT	TTGGACTTGT	A----TAGTA	5814
Anolis_carolinensis	----------	----------	----------	----------	----------	----------	----------	1184
Murine_leukemia_virus	GCCACCTCCC	TCC----TGG	GGACGATGAC	AGACACCTTC	----CCTAAA	CTATATTTTG	AC---CTGTG	5879
Phascolarctos_cinereus	CTCTCTTGAG	CCTGATGTGT	GCGCGCTGGT	AGCCGGTCTC	GAGTCCTGGG	ACATCCCGGA	ACTCACTGCA	5694
Estrildidae	----------	----------	----------	----------	----------	----------	----------	-
Xenopus_Silurana_tropicalis	----------	----------	----------	----------	----------	----------	----------	-
I_XT	CTGGNTAAAG	NTTCNTATCA	TTNANNCCTT	TCCCTTTTCA	CTCCAGTTAA	ANGNNGGTTA	GNNTNAAATN	6058
Takifugu_rubripes	----------	----------	----------	----------	----------	----------	----------	-
Bos_taurus	----------	----------	----------	----------	----------	----------	----------	-
Homo_sapiens	----------	----------	----------	----------	----------	----------	----------	-
Microcebus_murinus	----------	----------	----------	----------	----------	----------	----------	-
Myotis_lucifugus	----------	----------	----------	----------	----------	----------	----------	-
Equus_caballus	----------	----------	----------	----------	----------	----------	----------	-
Tupaia_belangeri	----------	----------	----------	----------	----------	----------	----------	-
Loxodonta_africana	----------	----------	----------	----------	----------	----------	----------	-
Murinae	----------	----------	----------	----------	----------	----------	----------	-
Canis_lupus_familiaris	----------	----------	----------	----------	----------	----------	----------	-
Sus_scrofa	----------	----------	----------	----------	----------	----------	----------	-
Choloepus_hoffmanni	----------	----------	----------	----------	----------	----------	----------	-
Vicugna_pacos	----------	----------	----------	----------	----------	----------	----------	-
Gavialis_gangeticus	----------	----------	----------	----------	----------	----------	----------	-
Mammalia	----------	----------	----------	----------	----------	----------	----------	-
Passeroidea	----------	----------	----------	----------	----------	----------	----------	-
Takifugu	----------	----------	----------	----------	----------	----------	----------	-
Pteropus_vampyrus	----------	----------	----------	----------	----------	----------	----------	-
Ornithorhynchus	TGCTAATAGA	AGCAATGAAT	TTATCTTTCA	GCAAATTAAG	TTGGTATCAA	GAGTGACGGA	GAGCCTTTTT	677
Oryctolagus_cuniculus	----------	----------	----------	----------	----------	----------	----------	-
Gallus_gallus	----------	----------	----------	----------	----------	----------	----------	-
Hominidae	NTTNCNNNNN	GNNAGNNCNN	NNCNNCNNNN	NCTNCTCGCA	NNNNNNNTNG	NCCGNNNNNN	NGNNAGNNNN	2845
Metatheria	----------	----------	----------	----------	----------	----------	----------	-
Rodentia	CGTTCCCTCC	TTGGAGCTGC	CTGTAATCCC	ACAGCTTCTC	TAGGCCCTCG	TGGCTTTTGC	GACTCCATTC	465
Monodelphis	----------	----------	----------	----------	----------	----------	----------	-
Consensus	----------	----------	----------	----------	----------	----------	----------	

Conservation

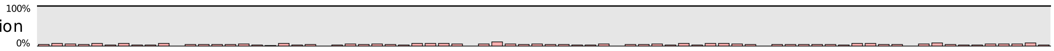

	6,600		6,620		6,640			
Dipodomys_ordii	----------	----------	----------	----------	----------	----------	----------	-
Laurasiatheria	----------	----------	----------	----------	----------	----------	----------	-
Eutheria	----------	----------	----------	----------	----------	----------	----------	-
Tarsius_syrichta	----------	----------	----------	----------	----------	----------	----------	-
Muridae	GCNAAGAAAT	AAACAAAANG	GACTTCCCCT	TCTGTCCAAC	ACTGACCAAG	GAGACTTTAA	ACANTTTTNG	988
Gasterosteus_aculeatus	CATNCACNNT	NNGANATAGA	CNGAATGAGA	AGCAAAATGG	TAGTAATGCA	TGAAAAACAA	AACAATGGGG	645
Felis_silvestris	----------	----------	----------	----------	----------	----------	----------	-
Rattus	----------	----------	----------	----------	----------	----------	----------	-
Papio_hamadryas	ACGATTGTCG	GCAAGTACTG	GCTGAGACCC	ATGGAACACG	AGAAGACCTT	AAAGACCAAG	AGCTCCCAGA	3947
Pan_troglodytes	ATGACTGNNN	AAAGNTGNNA	GCTCAGGCNC	ACGGANTTCG	AGAAGACCTG	CAGGACTGCG	AACTTCCTGA	2022
Ailuropoda_melanoleuca	----------	----------	----------	----------	----------	----------	----------	-
Cavia_porcellus	----------	----------	----------	----------	----------	----------	----------	-
Ictidomys_tridecemlineatus	----------	----------	----------	----------	----------	----------	----------	-
Canis	----------	----------	----------	----------	----------	----------	----------	-
Felis_catus	----------	----------	----------	----------	----------	----------	----------	-
Ochotona_princeps	----------	----------	----------	----------	----------	----------	----------	-
Cercopithecidae	CTATGATCCA	TAATGAGTGG	CCGAGGACTT	TTTACAATCG	GGGAATCTAT	TGGATCCTGC	CTCTACCAAC	119
Carnivora	----------	----------	----------	----------	----------	----------	----------	-
Mus	TCTGTTGTGT	TTTTTTTAAT	GGGAAGACGT	GCACCCTTAC	TGCCATTGAC	GATTAGCTAG	GCAAATTTAG	173
Mus_musculus	----------	----------	----------	----------	----------	----------	----------	-
Rattus_norvegicus	----------	----------	----------	----------	----------	----------	----------	-
Tursiops_truncatus	----------	----------	----------	----------	----------	----------	----------	-
Procavia_capensis	----------	----------	----------	----------	----------	----------	----------	-
Primates	----------	----------	----------	----------	----------	----------	----------	-
Echinops_telfairi	----------	----------	----------	----------	----------	----------	----------	-
Sorex_araneus	----------	----------	----------	----------	----------	----------	----------	-
Canidae	----------	----------	----------	----------	----------	----------	----------	-
Otolemur_garnettii	----------	----------	----------	----------	----------	----------	----------	-
Gallus	----------	----------	----------	----------	----------	----------	----------	-
Callithrix_jacchus	----------	----------	----------	----------	----------	----------	----------	-
Crocodylus_porosus	----------	----------	----------	----------	----------	----------	----------	-
Erinaceus_europaeus	----------	----------	----------	----------	----------	----------	----------	-
rnd-6_family-7426-LTR-ERV1	----------	----------	----------	----------	----------	----------	----------	-
Esox_lucius	----------	----------	----------	----------	----------	----------	----------	-
Salmo_salar	----------	----------	----------	----------	----------	----------	----------	-
Didelphidae	----------	----------	----------	----------	----------	----------	----------	-
Latimeria_chalumnae	GTATGCCCGA	CAATATAATG	GGGCTATAAA	GCGATAAAAT	CACGAGGCCA	GTTGCAAAAA	AAAAGTGGTA	195
Monodelphis_domestica	----------	----------	----------	----------	----------	----------	----------	-
Macropus_eugenii	----------	----------	----------	----------	----------	----------	----------	-
Alligator_mississippiensis	----------	----------	----------	----------	----------	----------	----------	-
Chrysemys_picta_bellii	CTGGGCATGG	CGGTTTGCAG	AATGGATCCA	GATTGAGGCT	AAACCTAGAA	TAAGGAGCGC	TGACCCCTTC	1436
Danio_rerio	----------	----------	----------	----------	----------	----------	----------	-
Taeniopygia	----------	----------	----------	----------	----------	----------	----------	-
Dasypus_novemcinctus	----------	----------	----------	----------	----------	----------	----------	-
LTR_XT	----------	----------	----------	----------	----------	----------	----------	-
Haplorrhini	ATACTATTGC	TTATACTGAT	TGTCTTGCTG	TCATGCTATC	TTTGCAATTG	CTATCAAACT	TGTTGCCCAG	6571
Catarrhini	AAA-----AC	TTCCACTGAT	TTTC------	----------	----------	----------	-----CCCGC	5838
Anolis_carolinensis	----------	----------	----------	----------	----------	----------	----------	1184
Murine_leukemia_virus	TGA-------	TTTAGTNGG-	-AGACNACTG	GG--------	---------A	TGA-----CC	CGGAACCCGA	5918
Phascolarctos_cinereus	TCGG---CCT	CTCAACAGGC	CAGGCCCCCT	GATTCA----	-----AACTA	TGAACATGCT	TATAATCAGA	5752
Estrildidae	----------	----------	----------	----------	----------	----------	----------	-
Xenopus_Silurana_tropicalis	----------	----------	----------	----------	----------	----------	----------	-
I_XT	CTATGNGNTT	TTNATNCTAG	CAGNATGGAA	ANTAGATACA	TAATACTNAC	NGNTGAAGNN	GTTAATCNGT	6128
Takifugu_rubripes	----------	----------	----------	----------	----------	----------	----------	-
Bos_taurus	----------	----------	----------	----------	----------	----------	----------	-
Homo_sapiens	----------	----------	----------	----------	----------	----------	----------	-
Microcebus_murinus	----------	----------	----------	----------	----------	----------	----------	-
Myotis_lucifugus	----------	----------	----------	----------	----------	----------	----------	-
Equus_caballus	----------	----------	----------	----------	----------	----------	----------	-
Tupaia_belangeri	----------	----------	----------	----------	----------	----------	----------	-
Loxodonta_africana	----------	----------	----------	----------	----------	----------	----------	-
Murinae	----------	----------	----------	----------	----------	----------	----------	-
Canis_lupus_familiaris	----------	----------	----------	----------	----------	----------	----------	-
Sus_scrofa	----------	----------	----------	----------	----------	----------	----------	-
Choloepus_hoffmanni	----------	----------	----------	----------	----------	----------	----------	-
Vicugna_pacos	----------	----------	----------	----------	----------	----------	----------	-
Gavialis_gangeticus	----------	----------	----------	----------	----------	----------	----------	-
Mammalia	----------	----------	----------	----------	----------	----------	----------	-
Passeroidea	----------	----------	----------	----------	----------	----------	----------	-
Takifugu	----------	----------	----------	----------	----------	----------	----------	-
Pteropus_vampyrus	----------	----------	----------	----------	----------	----------	----------	-
Ornithorhynchus	CTTGTTTCCT	CGTTAATATC	TACTTAAGTA	TGCAGTTGGT	TTTTAGTTCT	AGCCAAAACT	TTTACAATGG	747
Oryctolagus_cuniculus	----------	----------	----------	----------	----------	----------	----------	-
Gallus_gallus	----------	----------	----------	----------	----------	----------	----------	-
Hominidae	CCGAGACAGN	CCTNNNNNCT	TTNGNCNGNN	NCTNNCTAAA	GNCCNNNNNN	CATGCNCNNT	GNCCNNNNNN	2915
Metatheria	----------	----------	----------	----------	----------	----------	----------	-
Rodentia	CCNACCCTTT	CCCGTGGATG	AGACCCCATT	CCTGAGCTCG	GTTTTCGTTC	CTTTTGCACC	TTCTGGCCTC	535
Monodelphis	----------	----------	----------	----------	----------	----------	----------	-
Consensus	----------	----------	----------	----------	----------	----------	----------	

Conservation (100% / 0%)

6,660 6,680 6,700 6,720

Dipodomys_ordii	----------	----------	----------	----------	----------	----------	----------	-
Laurasiatheria	----------	----------	----------	----------	----------	----------	----------	-
Eutheria	----------	----------	----------	----------	----------	----------	----------	-
Tarsius_syrichta	----------	----------	----------	----------	----------	----------	----------	-
Muridae	CTAGGTCCTT	TTNAAAGGAT	TAAAACAAAT	GCTGCNAAAA	AGATTTCCTT	TGTCACCCAG	TCGGCCCCAG	1058
Gasterosteus_aculeatus	CTCACAGGCA	CTGCACGTCA	CAGAAGGTCG	GAATAGGGAG	GGAGCGAGAA	AACATGAACG	GGAAAACCGT	715
Felis_silvestris	----------	----------	----------	----------	----------	----------	----------	-
Rattus	----------	----------	----------	----------	----------	----------	----------	-
Papio_hamadryas	CGCGGATCAC	ACCTGGTACA	CAGACGGCAG	CAGTTACCTT	GACTCAGGTA	CTCGGAGGGC	GGGAGCAGCG	4017
Pan_troglodytes	CGCNNACCNC	AGTTGGTACA	CAGACGGTAG	CAGCTTCATG	GANTCAGGTA	CTGGGGACGC	TGGGGCGGCA	2092
Ailuropoda_melanoleuca	----------	----------	----------	----------	----------	----------	----------	-
Cavia_porcellus	----------	----------	----------	----------	----------	----------	----------	-
Ictidomys_tridecemlineatus	----------	----------	----------	----------	----------	----------	----------	-
Canis	----------	----------	----------	----------	----------	----------	----------	-
Felis_catus	----------	----------	----------	----------	----------	----------	----------	-
Ochotona_princeps	----------	----------	----------	----------	----------	----------	----------	-
Cercopithecidae	CTCCGACCCC	CCCCCCCCCG	CCCCCACCCC	GCCCGACTCC	TCGAGCCGTG	TTTTATGCAT	TTCACCATTC	189
Carnivora	----------	----------	----------	----------	----------	----------	----------	-
Mus	TAAAACTGGA	CTTTTGTCTC	ATGGCCTTTG	GTGGTGGCCC	AGGATTTTCT	CATGTAATTT	TTTTCAGGCG	243
Mus_musculus	----------	----------	----------	----------	----------	----------	----------	-
Rattus_norvegicus	----------	----------	----------	----------	----------	----------	----------	-
Tursiops_truncatus	----------	----------	----------	----------	----------	----------	----------	-
Procavia_capensis	----------	----------	----------	----------	----------	----------	----------	-
Primates	----------	----------	----------	----------	----------	----------	----------	-
Echinops_telfairi	----------	----------	----------	----------	----------	----------	----------	-
Sorex_araneus	----------	----------	----------	----------	----------	----------	----------	-
Canidae	----------	----------	----------	----------	----------	----------	----------	-
Otolemur_garnettii	----------	----------	----------	----------	----------	----------	----------	-
Gallus	----------	----------	----------	----------	----------	----------	----------	-
Callithrix_jacchus	----------	----------	----------	----------	----------	----------	----------	-
Crocodylus_porosus	----------	----------	----------	----------	----------	----------	----------	-
Erinaceus_europaeus	----------	----------	----------	----------	----------	----------	----------	-
rnd-6_family-7426-LTR-ERV1	----------	----------	----------	----------	----------	----------	----------	-
Esox_lucius	----------	----------	----------	----------	----------	----------	----------	-
Salmo_salar	----------	----------	----------	----------	----------	----------	----------	-
Didelphidae	----------	----------	----------	----------	----------	----------	----------	-
Latimeria_chalumnae	CAAGGTTTAA	ATTGTGAGAG	ATTGCTTTAT	ACATCAGAGA	ACTATGTATC	AGAGAAGATC	TCCTGATTCG	265
Monodelphis_domestica	----------	----------	----------	----------	----------	----------	----------	-
Macropus_eugenii	----------	----------	----------	----------	----------	----------	----------	-
Alligator_mississippiensis	----------	----------	----------	----------	----------	----------	----------	-
Chrysemys_picta_bellii	TGGTCCCCAG	AGCGACGCTT	TTAAAACTGG	CCCCGCCCTG	GGCTGCCGAC	TAAGCCTTCT	TCATGAAAAG	1506
Danio_rerio	----------	----------	----------	----------	----------	----------	----------	-
Taeniopygia	----------	----------	----------	----------	----------	----------	----------	-
Dasypus_novemcinctus	----------	----------	----------	----------	----------	----------	----------	-
LTR_XT	----------	----------	----------	----------	----------	----------	----------	-
Haplorrhini	GAGGATGCCC	GTGCATAGTA	TAAACTTGAT	CACACTAGTG	ACAATAATGC	TAACAGGCAT	GGGAGGAAAC	6641
Catarrhini	ATGGAGGACT	GCTCTCAGTG	TATAC---AT	CAGGTTACCG	A---------	-----GGTAG	GGCAACAAGT	5891
Anolis_carolinensis	----------	----------	----------	----------	----------	----------	----------	1184
Murine_leukemia_virus	TA--TTGGAG	AT-----GGT	TGCCGCTCTC	CCGGGGGAAG	A---------	--AAAAGGAC	AAGACTGTAT	5970
Phascolarctos_cinereus	TCACTTGGGG	AACCCTGGGA	TGTAGCTATC	CCCGGGCCAG	A---------	--ACAAGAAT	TGCTAGGTCC	5811
Estrildidae	----------	----------	----------	----------	----------	----------	----------	-
Xenopus_Silurana_tropicalis	----------	----------	----------	----------	----------	----------	----------	-
I_XT	TCNAGTTAGN	CNAAAGNNTA	GACCNTTAGT	AGGGATACCT	ATCNCTANAA	ATGCNCANAA	TNTTANAGGT	6198
Takifugu_rubripes	----------	----------	----------	----------	----------	----------	----------	-
Bos_taurus	----------	----------	----------	----------	----------	----------	----------	-
Homo_sapiens	----------	----------	----------	----------	----------	----------	----------	-
Microcebus_murinus	----------	----------	----------	----------	----------	----------	----------	-
Myotis_lucifugus	----------	----------	----------	----------	----------	----------	----------	-
Equus_caballus	----------	----------	----------	----------	----------	----------	----------	-
Tupaia_belangeri	----------	----------	----------	----------	----------	----------	----------	-
Loxodonta_africana	----------	----------	----------	----------	----------	----------	----------	-
Murinae	----------	----------	----------	----------	----------	----------	----------	-
Canis_lupus_familiaris	----------	----------	----------	----------	----------	----------	----------	-
Sus_scrofa	----------	----------	----------	----------	----------	----------	----------	-
Choloepus_hoffmanni	----------	----------	----------	----------	----------	----------	----------	-
Vicugna_pacos	----------	----------	----------	----------	----------	----------	----------	-
Gavialis_gangeticus	----------	----------	----------	----------	----------	----------	----------	-
Mammalia	----------	----------	----------	----------	----------	----------	----------	-
Passeroidea	----------	----------	----------	----------	----------	----------	----------	-
Takifugu	----------	----------	----------	----------	----------	----------	----------	-
Pteropus_vampyrus	----------	----------	----------	----------	----------	----------	----------	-
Ornithorhynchus	AAAATAGGAT	TTTTAGTACA	TAGGTCTGAT	AAGCCCTAGG	AAAACAATGG	GGTGAAGGAA	TTGCAGAATC	817
Oryctolagus_cuniculus	----------	----------	----------	----------	----------	----------	----------	-
Gallus_gallus	----------	----------	----------	----------	----------	----------	----------	-
Hominidae	ANNNTNCTNC	NNNANGNNNA	NGNCNNTCNN	NNTNNCCAGN	NNNNCCNNGT	NTNNNTCNCN	NNNNGANNNN	2985
Metatheria	----------	----------	----------	----------	----------	----------	----------	-
Rodentia	TCGAAAGCCC	TGGTACCAGG	CGCCGAGTCT	CCTAGGCGGT	TAGCCCTGAA	CCCCGTTGAG	TGTGGTGATG	605
Monodelphis	----------	----------	----------	----------	----------	----------	----------	-
Consensus	----------	----------	----------	----------	----------	----------	----------	

Conservation 100% 0%

6,740 6,760 6,780

Dipodomys_ordii	----------	----------	----------	----------	----------	----------	----------	-
Laurasiatheria	----------	----------	----------	----------	----------	----------	----------	-
Eutheria	----------	----------	----------	----------	----------	----------	----------	-
Tarsius_syrichta	----------	----------	----------	----------	----------	----------	----------	-
Muridae	ATATTAGAGA	AGAAGTTATA	AAAGTTAAAA	AAATTAAAAG	TATAAACATT	AAAGGATAAG	TAGGTCCCAG	1128
Gasterosteus_aculeatus	CCAACCGCGG	CACACCAGAA	GTTTCCCGAC	CCGTCGACGT	AGCAAACCCT	CATGCATAAG	TGACATGAAA	785
Felis_silvestris	----------	----------	----------	----------	----------	----------	----------	-
Rattus	----------	----------	----------	----------	----------	----------	----------	-
Papio_hamadryas	GTAGTAGACG	GACACAATAC	CATTTGGGCA	CAATCACTAC	CTCCTGGCAC	GTCTGCACAG	AAGGCTGAGT	4087
Pan_troglodytes	GTAATANAAG	NNNCNGCTGT	GATNTGGGCG	CAGTCTCTGC	CGCCGGGGAC	GTCGGCCCAG	AAGGCCGAAC	2162
Ailuropoda_melanoleuca	----------	----------	----------	----------	----------	----------	----------	-
Cavia_porcellus	----------	----------	----------	----------	----------	----------	----------	-
Ictidomys_tridecemlineatus	----------	----------	----------	----------	----------	----------	----------	-
Canis	----------	----------	----------	----------	----------	----------	----------	-
Felis_catus	----------	----------	----------	----------	----------	----------	----------	-
Ochotona_princeps	----------	----------	----------	----------	----------	----------	----------	-
Cercopithecidae	AACACTGGAG	ACATTTCCGA	GAAGTCGGCG	AGCCGGCCCC	CAGGTCCCCC	TGGGACTAAG	AGGAGGCCAT	259
Carnivora	----------	----------	----------	----------	----------	----------	----------	-
Mus	GCATCCGACA	CCTATAGTTG	GAGATGGCAC	CCCTGTCCGC	TTGCAAAAGA	AAACCGGCCC	GCCAATCTAT	313
Mus_musculus	----------	----------	----------	----------	----------	----------	----------	-
Rattus_norvegicus	----------	----------	----------	----------	----------	----------	----------	-
Tursiops_truncatus	----------	----------	----------	----------	----------	----------	----------	-
Procavia_capensis	----------	----------	----------	----------	----------	----------	----------	-
Primates	----------	----------	----------	----------	----------	----------	----------	-
Echinops_telfairi	----------	----------	----------	----------	----------	----------	----------	-
Sorex_araneus	----------	----------	----------	----------	----------	----------	----------	-
Canidae	----------	----------	----------	----------	----------	----------	----------	-
Otolemur_garnettii	----------	----------	----------	----------	----------	----------	----------	-
Gallus	----------	----------	----------	----------	----------	----------	----------	-
Callithrix_jacchus	----------	----------	----------	----------	----------	----------	----------	-
Crocodylus_porosus	----------	----------	----------	----------	----------	----------	----------	-
Erinaceus_europaeus	----------	----------	----------	----------	----------	----------	----------	-
rnd-6_family-7426-LTR-ERV1	----------	----------	----------	----------	----------	----------	----------	-
Esox_lucius	----------	----------	----------	----------	----------	----------	----------	-
Salmo_salar	----------	----------	----------	----------	----------	----------	----------	-
Didelphidae	----------	----------	----------	----------	----------	----------	----------	-
Latimeria_chalumnae	CTTGATTCAA	TTGCGACTAT	TCTATTACCC	TCGCGATCTC	TATACTGTGT	TCCAAAGTCT	TCTCTGCTTC	335
Monodelphis_domestica	----------	----------	----------	----------	----------	----------	----------	-
Macropus_eugenii	----------	----------	----------	----------	----------	----------	----------	-
Alligator_mississippiensis	----------	----------	----------	----------	----------	----------	----------	-
Chrysemys_picta_bellii	GGTGCCTGGG	AGTTACTCAG	TGGGCAACGT	CCGTGGCTAT	TTCTCTACTG	GACGTTGCAG	GTCCCTGCTG	1576
Danio_rerio	----------	----------	----------	----------	----------	----------	----------	-
Taeniopygia	----------	----------	----------	----------	----------	----------	----------	-
Dasypus_novemcinctus	----------	----------	----------	----------	----------	----------	----------	-
LTR_XT	----------	----------	----------	----------	----------	----------	----------	-
Haplorrhini	CAAGATAATT	GTCATCATTG	TATGATAGAA	GCTTGGTCTG	GTAAAGGTAT	AACTAAAACC	CTGTTATATC	6711
Catarrhini	TAAAACAATC	TTT-------	----------	----------	----------	----------	CTGTTCTAT-	5913
Anolis_carolinensis	----------	----------	----------	----------	----------	----------	----------	1184
Murine_leukemia_virus	GACTTCTATG	TTTGCCCC--	----GGTCAT	ACTGTACCAA	TAG------G	GTGTGGAGGG	CCGGGAGAGG	6028
Phascolarctos_cinereus	CAGTTCTACG	TGTGCCCCCG	GGATGGCCGT	TCCCTGTCCG	AGGCCCGGAG	GTGCGGAGGA	CTTGAATCTT	5881
Estrildidae	----------	----------	----------	----------	----------	----------	----------	-
Xenopus_Silurana_tropicalis	----------	----------	----------	----------	----------	----------	----------	-
I_XT	AATATACTAG	GAAGTNNCGN	TACATANAAA	CNATAAGTAT	CAGAATGCNA	TTAGTNCTTN	AGAAAAATAC	6268
Takifugu_rubripes	----------	----------	----------	----------	----------	----------	----------	-
Bos_taurus	----------	----------	----------	----------	----------	----------	----------	-
Homo_sapiens	----------	----------	----------	----------	----------	----------	----------	-
Microcebus_murinus	----------	----------	----------	----------	----------	----------	----------	-
Myotis_lucifugus	----------	----------	----------	----------	----------	----------	----------	-
Equus_caballus	----------	----------	----------	----------	----------	----------	----------	-
Tupaia_belangeri	----------	----------	----------	----------	----------	----------	----------	-
Loxodonta_africana	----------	----------	----------	----------	----------	----------	----------	-
Murinae	----------	----------	----------	----------	----------	----------	----------	-
Canis_lupus_familiaris	----------	----------	----------	----------	----------	----------	----------	-
Sus_scrofa	----------	----------	----------	----------	----------	----------	----------	-
Choloepus_hoffmanni	----------	----------	----------	----------	----------	----------	----------	-
Vicugna_pacos	----------	----------	----------	----------	----------	----------	----------	-
Gavialis_gangeticus	----------	----------	----------	----------	----------	----------	----------	-
Mammalia	----------	----------	----------	----------	----------	----------	----------	-
Passeroidea	----------	----------	----------	----------	----------	----------	----------	-
Takifugu	----------	----------	----------	----------	----------	----------	----------	-
Pteropus_vampyrus	----------	----------	----------	----------	----------	----------	----------	-
Ornithorhynchus	TTATCATTTG	ACCTTCCTCT	TTGAACCAAA	CTAATTAATG	TAAATGTTCC	AACTTTATAA	CCTTGGAAGT	887
Oryctolagus_cuniculus	----------	----------	----------	----------	----------	----------	----------	-
Gallus_gallus	----------	----------	----------	----------	----------	----------	----------	-
Hominidae	NNNANNNNCN	NTNANNNCTN	NGNNANNNNN	GNGTNTNNAG	TNNCCNNTCN	NANNNTNCAG	CTCTGNNCCN	3055
Metatheria	----------	----------	----------	----------	----------	----------	----------	-
Rodentia	ACCCACTGAA	TGGCTTGGTC	TCAGTCCTCC	CTGAGTCCTG	GGGACGCCCN	TGACTNCAGG	CTGCAGTG-A	674
Monodelphis	----------	----------	----------	----------	----------	----------	----------	-
Consensus	----------	----------	----------	----------	----------	----------	----------	

Conservation

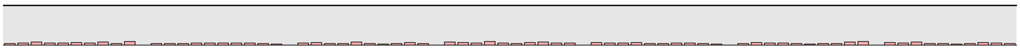

		6,800		6,820		6,840		6,860
Dipodomys_ordii	----------	----------	----------	----------	----------	----------	----------	-
Laurasiatheria	----------	----------	----------	----------	----------	----------	----------	-
Eutheria	----------	----------	----------	----------	----------	----------	----------	-
Tarsius_syrichta	----------	----------	----------	----------	----------	----------	----------	-
Muridae	TTGCTGGAGG	TAGCTCAAAG	GGTGTTTAAC	AACAGAGAAC	TGATTGGGAC	AAGACCTTTT	GCATAAACTG	1198
Gasterosteus_aculeatus	GCTTGAGAGA	AGGAGTTTTT	TGCGCAAGAC	AAGACTAAGA	GAACATTAAC	TGTAGAATAC	ACATTCCAAT	855
Felis_silvestris	----------	----------	----------	----------	----------	----------	----------	-
Rattus	----------	----------	----------	----------	----------	----------	----------	-
Papio_hamadryas	TAATAGCACT	AACCAAGGCC	CTAGAGCTGT	CCAAGGGAAA	GAAAGCTAAC	ATTTATACTG	ATAGCCGATA	4157
Pan_troglodytes	TNATAGCGCT	CACCAAGGCT	CTA-ACNTAG	GCGGGGAAAA	AGAATATAAC	A--------G	ACAGCGTATG	2223
Ailuropoda_melanoleuca	----------	----------	----------	----------	----------	----------	----------	-
Cavia_porcellus	----------	----------	----------	----------	----------	----------	----------	-
Ictidomys_tridecemlineatus	----------	----------	----------	----------	----------	----------	----------	-
Canis	----------	----------	----------	----------	----------	----------	----------	-
Felis_catus	----------	----------	----------	----------	----------	----------	----------	-
Ochotona_princeps	----------	----------	----------	----------	----------	----------	----------	-
Cercopithecidae	CAGCTTGCGG	TGGCCAATTA	ATATCAGGAA	CCGCTTAGTG	AGTGTACCCT	GACCGCCGAC	TGGTTTCACG	329
Carnivora	----------	----------	----------	----------	----------	----------	----------	-
Mus	CCGAATTGAC	TATGCCCACC	CCTTCCACCA	CCCAGACCCC	CCCCGCGGGG	CCCCCCGGAG	CTCGATCACG	383
Mus_musculus	----------	----------	----------	----------	----------	----------	----------	-
Rattus_norvegicus	----------	----------	----------	----------	----------	----------	----------	-
Tursiops_truncatus	----------	----------	----------	----------	----------	----------	----------	-
Procavia_capensis	----------	----------	----------	----------	----------	----------	----------	-
Primates	----------	----------	----------	----------	----------	----------	----------	-
Echinops_telfairi	----------	----------	----------	----------	----------	----------	----------	-
Sorex_araneus	----------	----------	----------	----------	----------	----------	----------	-
Canidae	----------	----------	----------	----------	----------	----------	----------	-
Otolemur_garnettii	----------	----------	----------	----------	----------	----------	----------	-
Gallus	----------	----------	----------	----------	----------	----------	----------	-
Callithrix_jacchus	----------	----------	----------	----------	----------	----------	----------	-
Crocodylus_porosus	----------	----------	----------	----------	----------	----------	----------	-
Erinaceus_europaeus	----------	----------	----------	----------	----------	----------	----------	-
rnd-6_family-7426-LTR-ERV1	----------	----------	----------	----------	----------	----------	----------	-
Esox_lucius	----------	----------	----------	----------	----------	----------	----------	-
Salmo_salar	----------	----------	----------	----------	----------	----------	----------	-
Didelphidae	----------	----------	----------	----------	----------	----------	----------	-
Latimeria_chalumnae	CTTGTTTATA	TATCACAGGC	GGTGAGCAGC	CTTTCTGTCT	GTCAATGGAA	CACTCTATCG	GTCACGGGAG	405
Monodelphis_domestica	----------	----------	----------	----------	----------	----------	----------	-
Macropus_eugenii	----------	----------	----------	----------	----------	----------	----------	-
Alligator_mississippiensis	----------	----------	----------	----------	----------	----------	----------	-
Chrysemys_picta_bellii	GGCGTGCGCC	GCCCTGTTTG	ACGAACTCTG	GCTTGGTTTC	CCAGTAGCCT	GCTACTAAGG	GCCCACGCTC	1646
Danio_rerio	----------	----------	----------	----------	----------	----------	----------	-
Taeniopygia	----------	----------	----------	----------	----------	----------	----------	-
Dasypus_novemcinctus	----------	----------	----------	----------	----------	----------	----------	-
LTR_XT	----------	----------	----------	----------	----------	----------	----------	-
Haplorrhini	AAACTTATTA	TGAGTGTACA	GG-AACTCCC	CTGGGGACAT	GTGTTTATAA	TCAAACCAGC	TACTCCGTCT	6780
Catarrhini	--AGTTACTA	TGAATGCCTA	GG-AACTTTA	AAAGGAACAT	GTTTATATAA	TGACACTCAG	TACAAGGTAT	5980
Anolis_carolinensis	----------	----------	----------	----------	----------	----------	----------	1184
Murine_leukemia_virus	GCTACTGCGG	CAAATGGGGA	TGTGAGACCA	CTGGACAGGC	ATACTGGAAG	CCATCATCAT	CATGGGACCT	6098
Phascolarctos_cinereus	TGTACTGTAA	AGAGTGGGGT	TGTGAGACCG	CGGGAACCGC	CTACTGGCAA	CCCCGGTCCT	CGTGGGACCT	5951
Estrildidae	----------	----------	----------	----------	----------	----------	----------	-
Xenopus_Silurana_tropicalis	----------	----------	----------	----------	----------	----------	----------	-
I_XT	TTGGAAAGTA	CAGGATTACT	AGATANNCCG	ACTATATGTG	TNNNACNNNN	GTTNNCTAAA	TCANACNATT	6338
Takifugu_rubripes	----------	----------	----------	----------	----------	----------	----------	-
Bos_taurus	----------	----------	----------	----------	----------	----------	----------	-
Homo_sapiens	----------	----------	----------	----------	----------	----------	----------	-
Microcebus_murinus	----------	----------	----------	----------	----------	----------	----------	-
Myotis_lucifugus	----------	----------	----------	----------	----------	----------	----------	-
Equus_caballus	----------	----------	----------	----------	----------	----------	----------	-
Tupaia_belangeri	----------	----------	----------	----------	----------	----------	----------	-
Loxodonta_africana	----------	----------	----------	----------	----------	----------	----------	-
Murinae	----------	----------	----------	----------	----------	----------	----------	-
Canis_lupus_familiaris	----------	----------	----------	----------	----------	----------	----------	-
Sus_scrofa	----------	----------	----------	----------	----------	----------	----------	-
Choloepus_hoffmanni	----------	----------	----------	----------	----------	----------	----------	-
Vicugna_pacos	----------	----------	----------	----------	----------	----------	----------	-
Gavialis_gangeticus	----------	----------	----------	----------	----------	----------	----------	-
Mammalia	----------	----------	----------	----------	----------	----------	----------	-
Passeroidea	----------	----------	----------	----------	----------	----------	----------	-
Takifugu	----------	----------	----------	----------	----------	----------	----------	-
Pteropus_vampyrus	----------	----------	----------	----------	----------	----------	----------	-
Ornithorhynchus	ACAGAGTTGA	TTGAAATTGT	ATACTTTTTT	AAATCAGTTT	CTTCAAGTTT	TTGATAGCAA	AATTTCACTT	957
Oryctolagus_cuniculus	----------	----------	----------	----------	----------	----------	----------	-
Gallus_gallus	----------	----------	----------	----------	----------	----------	----------	-
Hominidae	CGNNNNNNNN	NTGACCNNNG	NANNNCNCTN	ACNGCNNNCN	CNNANANCNN	NNCCANANAN	NGANNNNGNC	3125
Metatheria	----------	----------	----------	----------	----------	----------	----------	-
Rodentia	CATTCTNCTT	CCCCTCTCTC	ATCCATGGGA	ACGGTGTCTT	CCGTACCCTC	TCATTCCCCC	TTGGGATGCC	744
Monodelphis	----------	----------	----------	----------	----------	----------	----------	-
Consensus	----------	----------	----------	----------	----------	----------	----------	

Conservation

100%

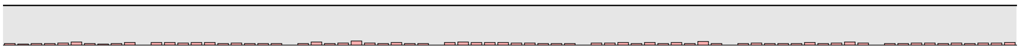

0%

6,880 6,900 6,920

Dipodomys_ordii	----------	----------	----------	----------	----------	----------	----------	-
Laurasiatheria	----------	----------	----------	----------	----------	----------	----------	-
Eutheria	----------	----------	----------	----------	----------	----------	----------	-
Tarsius_syrichta	----------	----------	----------	----------	----------	----------	----------	-
Muridae	AAAGCCACTG	TCACCTTCTC	TGATGATGAG	ACA-CAGTTA	GACTTACAGA	TANCCAGCAA	AATTTTGGTG	1267
Gasterosteus_aculeatus	GCCAAGATCT	AGGGTACTGA	TGCTAGTGGG	TCAACAGTTA	CAAACAAAAT	ATGTACGCGG	ACCAACTACC	925
Felis_silvestris	----------	----------	----------	----------	----------	----------	----------	-
Rattus	----------	----------	----------	----------	----------	----------	----------	-
Papio_hamadryas	TGCCTTTGCA	ACGGCTCATA	CTCATGGAAG	TATCTATGAA	AGAAGAGGTC	TCCTAACCTC	AGAAGGAAAG	4227
Pan_troglodytes	---CTTTGCA	ACGCCAAACA	T-----GGGG	CATTTACAGG	CGAGG-----	TACTGACCT-	-GAAGGAAAA	2278
Ailuropoda_melanoleuca	----------	----------	----------	----------	----------	----------	----------	-
Cavia_porcellus	----------	----------	----------	----------	----------	----------	----------	-
Ictidomys_tridecemlineatus	----------	----------	----------	----------	----------	----------	----------	-
Canis	----------	----------	----------	----------	----------	----------	----------	-
Felis_catus	----------	----------	----------	----------	----------	----------	----------	-
Ochotona_princeps	----------	----------	----------	----------	----------	----------	----------	-
Cercopithecidae	CGAATAAATA	AGAGTCAGGA	TTGCGTTAAA	AGACCGAGAA	AGCAACTGCA	GCGGGAACTG	TCTGAGGCAT	399
Carnivora	----------	----------	----------	----------	----------	----------	----------	-
Mus	TTCTGCGCCG	ACTCCTCAGT	ATGGCCTTTT	CTGATTAATG	AACATCACCC	TTTTCAAAAC	CCGTACGGTT	453
Mus_musculus	----------	----------	----------	----------	----------	----------	----------	-
Rattus_norvegicus	----------	----------	----------	----------	----------	----------	----------	-
Tursiops_truncatus	----------	----------	----------	----------	----------	----------	----------	-
Procavia_capensis	----------	----------	----------	----------	----------	----------	----------	-
Primates	----------	----------	----------	----------	----------	----------	----------	-
Echinops_telfairi	----------	----------	----------	----------	----------	----------	----------	-
Sorex_araneus	----------	----------	----------	----------	----------	----------	----------	-
Canidae	----------	----------	----------	----------	----------	----------	----------	-
Otolemur_garnettii	----------	----------	----------	----------	----------	----------	----------	-
Gallus	----------	----------	----------	----------	----------	----------	----------	-
Callithrix_jacchus	----------	----------	----------	----------	----------	----------	----------	-
Crocodylus_porosus	----------	----------	----------	----------	----------	----------	----------	-
Erinaceus_europaeus	----------	----------	----------	----------	----------	----------	----------	-
rnd-6_family-7426-LTR-ERV1	----------	----------	----------	----------	----------	----------	---------A	1
Esox_lucius	----------	----------	----------	----------	----------	----------	----------	-
Salmo_salar	----------	----------	----------	----------	----------	----------	----------	-
Didelphidae	----------	----------	----------	----------	----------	----------	----------	-
Latimeria_chalumnae	CGTGTTTTTA	TATCCCGTGC	CTCATTTGAA	GAAAATTTTC	AACTCGTCGT	CACCCCATCC	TAATTACTTT	475
Monodelphis_domestica	----------	----------	----------	----------	----------	----------	----------	-
Macropus_eugenii	----------	----------	----------	----------	----------	----------	----------	-
Alligator_mississippiensis	----------	----------	----------	----------	----------	----------	----------	-
Chrysemys_picta_bellii	GTCGTAAGAC	TTTCTATTAC	CTATGCCCCT	AACCGCACCT	GCTCCGCAGA	AGAACAGACT	GTTGGTATAT	1716
Danio_rerio	----------	----------	----------	----------	----------	----------	----------	-
Taeniopygia	----------	----------	----------	----------	----------	----------	----------	-
Dasypus_novemcinctus	----------	----------	----------	----------	----------	----------	----------	-
LTR_XT	----------	----------	----------	----------	----------	----------	----------	-
Haplorrhini	GTGACCCGGG	AAATGGGCAA	CCTC--AAGT	ATGTTATGAT	CC----AGGC	CTCCTAC-CC	TACGACTTCT	6843
Catarrhini	GTAGCCCAGG	AAACGACNGA	CCAG--ATGT	GTGTTATGAC	CCCTCTGAGC	CTCCCATGTC	CACAGTTTTT	6048
Anolis_carolinensis	----------	----------	----------	----------	----------	----------	----------	1184
Murine_leukemia_virus	AATTTCCCTT	AAGCGAGGAA	ACACTCCTAA	GGATCAGGGC	CCCTGTTATG	ATTCCTCGGT	CTCCAGTGGC	6168
Phascolarctos_cinereus	TAT-------	AA--------	------CTGT	AGGCCAGGGT	CAC-------	---------C	CTACAGGGAC	5984
Estrildidae	----------	----------	----------	----------	----------	----------	----------	-
Xenopus_Silurana_tropicalis	----------	----------	----------	----------	----------	----------	----------	-
I_XT	GTNANGACAG	ATTCNGTANT	ATNNCNANCT	ANNATNCATC	TNCGAGACAC	AACGATTGTT	AAAANGTAAA	6408
Takifugu_rubripes	----------	----------	----------	----------	----------	----------	----------	-
Bos_taurus	----------	----------	----------	----------	----------	----------	----------	-
Homo_sapiens	----------	----------	----------	----------	----------	----------	----------	-
Microcebus_murinus	----------	----------	----------	----------	----------	----------	----------	-
Myotis_lucifugus	----------	----------	----------	----------	----------	----------	----------	-
Equus_caballus	----------	----------	----------	----------	----------	----------	----------	-
Tupaia_belangeri	----------	----------	----------	----------	----------	----------	----------	-
Loxodonta_africana	----------	----------	----------	----------	----------	----------	----------	-
Murinae	----------	----------	----------	----------	----------	----------	----------	-
Canis_lupus_familiaris	----------	----------	----------	----------	----------	----------	----------	-
Sus_scrofa	----------	----------	----------	----------	----------	----------	----------	-
Choloepus_hoffmanni	----------	----------	----------	----------	----------	----------	----------	-
Vicugna_pacos	----------	----------	----------	----------	----------	----------	----------	-
Gavialis_gangeticus	----------	----------	----------	----------	----------	----------	----------	-
Mammalia	----------	----------	----------	----------	----------	----------	----------	-
Passeroidea	----------	----------	----------	----------	----------	----------	----------	-
Takifugu	----------	----------	----------	----------	----------	----------	----------	-
Pteropus_vampyrus	----------	----------	----------	----------	----------	----------	----------	-
Ornithorhynchus	CCTGAGCTTT	GACAAGCCTG	TTTATTCAGG	AAGTTGGGTA	CTGTCTTTAG	TGGAATATTC	ACAAGGCTGC	1027
Oryctolagus_cuniculus	----------	----------	----------	----------	----------	----------	----------	-
Gallus_gallus	----------	----------	----------	----------	----------	----------	----------	-
Hominidae	TCATTNNNGN	CCNCCNANTT	CCCNANNNCG	CNNANAANAN	CCNCTCCTTC	GTAGGGCTAG	TAGGGTNCNT	3195
Metatheria	----------	----------	----------	----------	----------	----------	----------	-
Rodentia	TCTTAGAAAA	CCTCCAGCCT	CTCCACCTGG	CACCCGACCT	GAAGGGCCCT	AAATTTATTC	ACTTTTGCAA	814
Monodelphis	----------	----------	----------	----------	----------	----------	----------	-
Consensus	----------	----------	----------	----------	----------	----------	----------	

Conservation

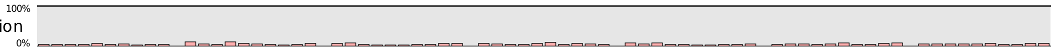

	6,940		6,960		6,980		7,000	
Dipodomys_ordii	----------	----------	----------	----------	----------	----------	----------	-
Laurasiatheria	----------	----------	----------	----------	----------	----------	----------	-
Eutheria	----------	----------	----------	----------	----------	----------	----------	-
Tarsius_syrichta	----------	----------	----------	----------	----------	----------	----------	-
Muridae	TCTCACCCTC	TGTCAGAAGA	AACNAACAAT	CGAGCCCCCA	GGACTGGCCC	AACACCAAGC	TCCTATAATT	1337
Gasterosteus_aculeatus	AGAGCCTACA	TGCAGGCTAC	CCCCCATAAT	TTGGAAATGA	GATGATATAA	AGCGAGGTGC	CAATTGGCCG	995
Felis_silvestris	----------	----------	----------	----------	----------	----------	----------	-
Rattus	----------	----------	----------	----------	----------	----------	----------	-
Papio_hamadryas	GAAATCAAGA	ACAAAGCTGA	AATAATTGCC	TTATTAAAGG	CCCTTTTTCT	TCCTCAAGAA	GTGGCTATAA	4297
Pan_troglodytes	GAA--TAAAA	ACAAGG----	---AATCCTA	GCCCTTAAAG	CCCTTG----	----CCAAAA	ATGCC---AT	2328
Ailuropoda_melanoleuca	----------	----------	----------	----------	----------	----------	----------	-
Cavia_porcellus	----------	----------	----------	----------	----------	----------	----------	-
Ictidomys_tridecemlineatus	----------	----------	----------	----------	----------	----------	----------	-
Canis	----------	----------	----------	----------	----------	----------	----------	-
Felis_catus	----------	----------	----------	----------	----------	----------	----------	-
Ochotona_princeps	----------	----------	----------	----------	----------	----------	----------	-
Cercopithecidae	GGGATGCCCA	ACCCCTGAGA	TGGCGTCGCC	CCCCGACCGG	TATGTGGGGC	TTCTTGAACG	GGCTCGTTGG	469
Carnivora	----------	----------	----------	----------	----------	----------	----------	-
Mus	GATTATGTTC	ACCACCACTG	GGATGATGCA	GCACTTTCAG	CTTAACTGAG	AGAGGAAGGA	TCTTGAGCGA	523
Mus_musculus	----------	----------	----------	----------	----------	----------	----------	-
Rattus_norvegicus	----------	----------	----------	----------	----------	----------	----------	-
Tursiops_truncatus	----------	----------	----------	----------	----------	----------	----------	-
Procavia_capensis	----------	----------	----------	----------	----------	----------	----------	-
Primates	----------	----------	----------	----------	----------	----------	----------	-
Echinops_telfairi	----------	----------	----------	----------	----------	----------	----------	-
Sorex_araneus	----------	----------	----------	----------	----------	----------	----------	-
Canidae	----------	----------	----------	----------	----------	----------	----------	-
Otolemur_garnettii	----------	----------	----------	----------	----------	----------	----------	-
Gallus	----------	----------	----------	----------	----------	----------	----------	-
Callithrix_jacchus	----------	----------	----------	----------	----------	----------	----------	-
Crocodylus_porosus	----------	----------	----------	----------	----------	----------	----------	-
Erinaceus_europaeus	----------	----------	----------	----------	----------	----------	----------	-
rnd-6_family-7426-LTR-ERV1	TGCTAAACGC	TTAATCGGGA	GGAGGTTTAN	TGATCCNTCT	GTACAGAGTG	ATATGAAGTT	ATGGCCGTTT	71
Esox_lucius	----------	----------	----------	----------	----------	----------	----------	-
Salmo_salar	----------	----------	----------	----------	----------	----------	----------	-
Didelphidae	----------	----------	----------	----------	----------	----------	----------	-
Latimeria_chalumnae	TTTAACGTTG	TGCTTTCTAA	CATCGTCCAA	TGTTTCAAAG	GCATTTGCTC	CCTTGCTCCG	ACGAAGCCTG	545
Monodelphis_domestica	----------	----------	----------	----------	----------	----------	----------	-
Macropus_eugenii	----------	----------	----------	----------	----------	----------	----------	-
Alligator_mississippiensis	----------	----------	----------	----------	----------	----------	----------	-
Chrysemys_picta_bellii	GCCCTCCCGC	CGATTAGATC	CCTCCCAATC	TGACTGTGTT	ACGATGGTTA	GGCAAGGCGG	TATGCTGTGT	1786
Danio_rerio	----------	----------	----------	----------	----------	----------	----------	-
Taeniopygia	----------	----------	----------	----------	----------	----------	----------	-
Dasypus_novemcinctus	----------	----------	----------	----------	----------	----------	----------	-
LTR_XT	----------	----------	----------	----------	----------	----------	----------	-
Haplorrhini	GGTTTGAAAT	TCAAATAGGG	AAACCTTTGT	TACCCTCATA	TGCC-AATCC	TAAAGACGTT	GGAACTGGGA	6912
Catarrhini	GAAATAAGAT	TAAGGACTGA	AGACTGGTGG	GGAC-TCATA	AAT--GATAC	AAGTAAAGTA	TTAGCCAGAA	6115
Anolis_carolinensis	----------	----------	----------	----------	----------	----------	----------	1184
Murine_leukemia_virus	GTCCAGGGTG	CCACACCGGG	GGGTCGATGC	AACCCCCTAG	TCTTAGAATT	CACTGACGCG	GGTAAAAAGG	6238
Phascolarctos_cinereus	ATGCGAG---	-CGCACCGG-	---TTGGTGC	AACCCACTCA	AGATAGAGTT	CACCGAGCCT	GGCAAACGGT	6046
Estrildidae	----------	----------	----------	----------	----------	----------	----------	-
Xenopus_Silurana_tropicalis	----------	----------	----------	----------	----------	----------	----------	-
I_XT	TACTGTGAAA	NTNTACATAG	CTTGTATGGG	AATGAAAAGT	GATNTGNTCC	ACGGTAATTN	AAATGANTNC	6478
Takifugu_rubripes	----------	----------	----------	----------	----------	----------	----------	-
Bos_taurus	----------	----------	----------	----------	----------	----------	----------	-
Homo_sapiens	----------	----------	----------	----------	----------	----------	----------	-
Microcebus_murinus	----------	----------	----------	----------	----------	----------	----------	-
Myotis_lucifugus	----------	----------	----------	----------	----------	----------	----------	-
Equus_caballus	----------	----------	----------	----------	----------	----------	----------	-
Tupaia_belangeri	----------	----------	----------	----------	----------	----------	----------	-
Loxodonta_africana	----------	----------	----------	----------	----------	----------	----------	-
Murinae	----------	----------	----------	----------	----------	----------	----------	-
Canis_lupus_familiaris	----------	----------	----------	----------	----------	----------	----------	-
Sus_scrofa	----------	----------	----------	----------	----------	----------	----------	-
Choloepus_hoffmanni	----------	----------	----------	----------	----------	----------	----------	-
Vicugna_pacos	----------	----------	----------	----------	----------	----------	----------	-
Gavialis_gangeticus	----------	----------	----------	----------	----------	----------	----------	-
Mammalia	----------	----------	----------	----------	----------	----------	----------	-
Passeroidea	----------	----------	----------	----------	----------	----------	----------	-
Takifugu	----------	----------	----------	----------	----------	----------	----------	-
Pteropus_vampyrus	----------	----------	----------	----------	----------	----------	----------	-
Ornithorhynchus	ATTTGCATTA	AGCATACTGT	AAGTTTGAAA	TGTCTTCCAC	AGAGCGTTTA	GTTTAAACAA	AGTTCATGTG	1097
Oryctolagus_cuniculus	----------	----------	----------	----------	----------	----------	----------	-
Gallus_gallus	----------	----------	----------	----------	----------	----------	----------	-
Hominidae	CCNGCNCNNN	NTCCCAANNT	TNNNNGTCTN	NNCNAAGNNN	NNNNACNANN	CGGCGANNGA	ANNNNCNNCN	3265
Metatheria	----------	----------	----------	----------	----------	----------	----------	-
Rodentia	TCAA-ATTTG	GCCTCAATAT	CCCTTAGATA	ATCAATCCAA	ATGGCCGCCT	AATGGCACCC	TAGATCCCAA	883
Monodelphis	----------	----------	----------	----------	----------	----------	----------	-
Consensus	----------	----------	----------	----------	----------	----------	----------	

Conservation

100%

0%

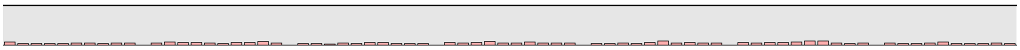

	7,020		7,040		7,060			
Dipodomys_ordii	----------	----------	----------	----------	----------	----------	----------	-
Laurasiatheria	----------	----------	----------	----------	----------	----------	----------	-
Eutheria	----------	----------	----------	----------	----------	----------	----------	-
Tarsius_syrichta	----------	----------	----------	----------	----------	----------	----------	-
Muridae	GTTCAACTAA	CCAGTTCAGC	TACCTCCATC	CAGATTAAAC	AATACCCAAT	GACCCTGGAA	ACAAAAGNGG	1407
Gasterosteus_aculeatus	TCCACCCGTA	TACAACCTGC	AAGCAGCCAT	GACAGGGGAA	ACCCCACGTT	TCAAATGTGA	GATGCGCAAA	1065
Felis_silvestris	----------	----------	----------	----------	----------	----------	----------	-
Rattus	----------	----------	----------	----------	----------	----------	----------	-
Papio_hamadryas	TTCACTGCCC	CGGGCATCAG	AAAGGACAGG	ATCCAGTCGC	AGTAGGAAAC	AGACAGGCTG	ACCGAGTGGC	4367
Pan_troglodytes	GTCATTGCCC	-GGGCATCAA	AATA------	------TCCA	TGCCAGGAAC	TCGCGACCAA	CGCAAGAAGT	2385
Ailuropoda_melanoleuca	----------	----------	----------	----------	----------	----------	----------	-
Cavia_porcellus	----------	----------	----------	----------	----------	----------	----------	-
Ictidomys_tridecemlineatus	----------	----------	----------	----------	----------	----------	----------	-
Canis	----------	----------	----------	----------	----------	----------	----------	-
Felis_catus	----------	----------	----------	----------	----------	----------	----------	-
Ochotona_princeps	----------	----------	----------	----------	----------	----------	----------	-
Cercopithecidae	CTCGGAGACG	ACCCTGTCCA	TCTTTTTCCG	CCCCCTTAGG	GGATCTCAAT	GGGCTCATTG	CCCTCTCTGA	539
Carnivora	----------	----------	----------	----------	----------	----------	----------	-
Mus	AAAAGTCCGT	GGCCACCATA	ATGGTTCCTC	CGCTGATAAT	ACAGTAGGGA	CGCTTAGGGC	TGTGGCGGTG	593
Mus_musculus	----------	----------	----------	----------	----------	----------	----------	-
Rattus_norvegicus	----------	----------	----------	----------	----------	----------	----------	-
Tursiops_truncatus	----------	----------	----------	----------	----------	----------	----------	-
Procavia_capensis	----------	----------	----------	----------	----------	----------	----------	-
Primates	----------	----------	----------	----------	----------	----------	----------	-
Echinops_telfairi	----------	----------	----------	----------	----------	----------	----------	-
Sorex_araneus	----------	----------	----------	----------	----------	----------	----------	-
Canidae	----------	----------	----------	----------	----------	----------	----------	-
Otolemur_garnettii	----------	----------	----------	----------	----------	----------	----------	-
Gallus	----------	----------	----------	----------	----------	----------	----------	-
Callithrix_jacchus	----------	----------	----------	----------	----------	----------	----------	-
Crocodylus_porosus	----------	----------	----------	----------	----------	----------	----------	-
Erinaceus_europaeus	----------	----------	----------	----------	----------	----------	----------	-
rnd-6_family-7426-LTR-ERV1	AAGGTTATTC	CCGGNCCTGG	TGACAAGCCN	ACGATTGTTG	TCAACTTCAA	GGGTGAGGAG	AAGCAGTTTN	141
Esox_lucius	----------	----------	----------	----------	----------	----------	----------	-
Salmo_salar	----------	----------	----------	----------	----------	----------	----------	-
Didelphidae	----------	----------	----------	----------	----------	----------	----------	-
Latimeria_chalumnae	AAGGTTAGCA	AAACATTTCA	GGCGATTGGA	GCTTTGTGCG	CTCAACAGGC	CGGTGGGGCC	AAGATCGTTT	615
Monodelphis_domestica	----------	----------	----------	----------	----------	----------	----------	-
Macropus_eugenii	----------	----------	----------	----------	----------	----------	----------	-
Alligator_mississippiensis	----------	----------	----------	----------	----------	----------	----------	-
Chrysemys_picta_bellii	CCTCCTGAAC	TGAAGCTGTT	TACCTCTGGA	CTCGCCATGC	GAATTGCCTA	CCGTGCCTGG	CAGAAGTTAA	1856
Danio_rerio	----------	----------	----------	----------	----------	----------	----------	-
Taeniopygia	----------	----------	----------	----------	----------	----------	----------	-
Dasypus_novemcinctus	----------	----------	----------	----------	----------	----------	----------	-
LTR_XT	----------	----------	----------	----------	----------	----------	----------	-
Haplorrhini	AACTCGTAAG	CAAAACACAG	GTATTCCCTT	ATTCACATAA	AGGATCTGTT	TCTATATATT	TTGATGCCTG	6982
Catarrhini	----CAGAAG	AAAA----AG	GGGTGCCCAA	ACGCATA---	---ATCT---	----TGAAAT	TTGATGCCTG	6164
Anolis_carolinensis	----------	----------	----------	----------	----------	----------	----------	1184
Murine_leukemia_virus	CCAGCTGGGA	TGCCCCCAAA	GTTTGGGGAC	TAAGACTCTA	TCGATCCACA	GGGGCCGACC	CGGTGACCCG	6308
Phascolarctos_cinereus	TCCG--AAAC	TGGCTACAGG	G---GCGAAC	CTGGGGTCTA	AGGTTCTATG	TGACTGGACA	CCCAGGCGTG	6111
Estrildidae	----------	----------	----------	----------	----------	----------	----------	-
Xenopus_Silurana_tropicalis	----------	----------	----------	----------	----------	----------	----------	-
I_XT	ACTGGNNNTG	NANAGTGAAT	NNCTCTTNTN	GCNACAGGTA	ATNNTNNACN	NATGAANNAN	NCNATGGCAT	6548
Takifugu_rubripes	----------	----------	----------	----------	----------	----------	----------	-
Bos_taurus	----------	----------	----------	----------	----------	----------	----------	-
Homo_sapiens	----------	----------	----------	----------	----------	----------	----------	-
Microcebus_murinus	----------	----------	----------	----------	----------	----------	----------	-
Myotis_lucifugus	----------	----------	----------	----------	----------	----------	----------	-
Equus_caballus	----------	----------	----------	----------	----------	----------	----------	-
Tupaia_belangeri	----------	----------	----------	----------	----------	----------	----------	-
Loxodonta_africana	----------	----------	----------	----------	----------	----------	----------	-
Murinae	----------	----------	----------	----------	----------	----------	----------	-
Canis_lupus_familiaris	----------	----------	----------	----------	----------	----------	----------	-
Sus_scrofa	----------	----------	----------	----------	----------	----------	----------	-
Choloepus_hoffmanni	----------	----------	----------	----------	----------	----------	----------	-
Vicugna_pacos	----------	----------	----------	----------	----------	----------	----------	-
Gavialis_gangeticus	----------	----------	----------	----------	----------	----------	----------	-
Mammalia	----------	----------	----------	----------	----------	----------	----------	-
Passeroidea	----------	----------	----------	----------	----------	----------	----------	-
Takifugu	----------	----------	----------	----------	----------	----------	----------	-
Pteropus_vampyrus	----------	----------	----------	----------	----------	----------	----------	-
Ornithorhynchus	GTGGTGTAGA	AAAATTCTTG	AATGCCTGGA	AATTCTTGAA	AGGCAAAGAT	GGTATCTTAA	CCGCAGTACT	1167
Oryctolagus_cuniculus	----------	----------	----------	----------	----------	----------	----------	-
Gallus_gallus	----------	----------	----------	----------	----------	----------	----------	-
Hominidae	GNNCNNNTGN	CTGNNCNCNN	NNTACCNTNG	NCNNCCANTN	NCGCNGGCNN	CNNNANNGCN	NACTCNCANC	3335
Metatheria	----------	----------	----------	----------	----------	----------	----------	-
Rodentia	TATTATTCGG	GACCTTTACA	AATACTGCGA	GCGATCGGGG	AAATGGAAAG	AGATTCCCCA	TTCCCTACGT	953
Monodelphis	----------	----------	----------	----------	----------	----------	----------	-
Consensus	----------	----------	----------	----------	----------	----------	----------	

Conservation
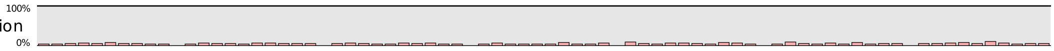

7,080 7,100 7,120 7,140

Dipodomys_ordii	----------	----------	----------	----------	----------	----------	----------	-
Laurasiatheria	----------	----------	----------	----------	----------	----------	----------	-
Eutheria	----------	----------	----------	----------	----------	----------	----------	-
Tarsius_syrichta	----------	----------	----------	----------	----------	----------	----------	-
Muridae	AAATCGNAGT	CTATATTGCC	TGGNTTAAAG	AGACAGGCAT	CCTGGTGCCC	TGCCAGTCAC	CCTGGAATAT	1477
Gasterosteus_aculeatus	AAAAACAACA	CGAGGACACC	TGCCTCAACC	AGACCAGAAT	AAACATACCT	TACTCATACC	CACTAGCGAG	1135
Felis_silvestris	----------	----------	----------	----------	----------	----------	----------	-
Rattus	----------	----------	----------	----------	----------	----------	----------	-
Papio_hamadryas	CAGGCAAGCC	GCCATGGCGG	AAGTACTGAC	CCTAGCCACA	GAACCTGACA	ACACCAGCCA	CATAACTATT	4437
Pan_troglodytes	---GCAAGCT	AC--------	----ATTGAC	CCAC----CT	GACCCGGCCC	CGACTTGCCA	TATCCCAATC	2436
Ailuropoda_melanoleuca	----------	----------	----------	----------	----------	----------	----------	-
Cavia_porcellus	----------	----------	----------	----------	----------	----------	----------	-
Ictidomys_tridecemlineatus	----------	----------	----------	----------	----------	----------	----------	-
Canis	----------	----------	----------	----------	----------	----------	----------	-
Felis_catus	----------	----------	----------	----------	----------	----------	----------	-
Ochotona_princeps	----------	----------	----------	----------	----------	----------	----------	-
Cercopithecidae	ACAGCGCTCC	AGGGGAACTG	CAACCCTTTA	ACCATCCATC	CGAGGTCATT	CTCTTCCTCC	TGAAACTCCT	609
Carnivora	----------	----------	----------	----------	----------	----------	----------	-
Mus	GCTGCGCCCA	CAATTGGCAG	GTAAGAGAGT	TCAGGGCGAC	CCTCTTTTGA	GTATGGAGCT	TATGGGTACC	663
Mus_musculus	----------	----------	----------	----------	----------	----------	----------	-
Rattus_norvegicus	----------	----------	----------	----------	----------	----------	----------	-
Tursiops_truncatus	----------	----------	----------	----------	----------	----------	----------	-
Procavia_capensis	----------	----------	----------	----------	----------	----------	----------	-
Primates	----------	----------	----------	----------	----------	----------	----------	-
Echinops_telfairi	----------	----------	----------	----------	----------	----------	----------	-
Sorex_araneus	----------	----------	----------	----------	----------	----------	----------	-
Canidae	----------	----------	----------	----------	----------	----------	----------	-
Otolemur_garnettii	----------	----------	----------	----------	----------	----------	----------	-
Gallus	----------	----------	----------	----------	----------	----------	----------	-
Callithrix_jacchus	----------	----------	----------	----------	----------	----------	----------	-
Crocodylus_porosus	----------	----------	----------	----------	----------	----------	----------	-
Erinaceus_europaeus	----------	----------	----------	----------	----------	----------	----------	-
rnd-6_family-7426-LTR-ERV1	CTGCTGAGGA	GATCTCATCN	ATGGTNTTAA	CNAAGATGAA	GGAAACCGCC	GAGGCTTTCC	TTGGGACAAC	211
Esox_lucius	----------	----------	----------	----------	----------	----------	----------	-
Salmo_salar	----------	----------	----------	----------	----------	----------	----------	-
Didelphidae	----------	----------	----------	----------	----------	----------	----------	-
Latimeria_chalumnae	ACCCATAGGA	GGTGTCCGGG	CAAAGCTTAC	CTGCGACACA	CCTAGCCCTG	CCACCCCTTC	GCCCTTCCAG	685
Monodelphis_domestica	----------	----------	----------	----------	----------	----------	----------	-
Macropus_eugenii	----------	----------	----------	----------	----------	----------	----------	-
Alligator_mississippiensis	----------	----------	----------	----------	----------	----------	----------	-
Chrysemys_picta_bellii	TTTACTGACT	CTATGCTTTT	GGGTTCATGT	AGCTTGGAAG	AAGGGTCTAC	ACCGGTCTCC	TAAGATGGAA	1926
Danio_rerio	----------	----------	----------	----------	----------	----------	----------	-
Taeniopygia	----------	----------	----------	----------	----------	----------	----------	-
Dasypus_novemcinctus	----------	----------	----------	----------	----------	----------	----------	-
LTR_XT	----------	----------	----------	----------	----------	----------	----------	-
Haplorrhini	--TCAGGCTG	CACACCTCAG	CAACCTAAAC	AATCTAGGAG	TAGTCTGCAA	GAACTTAGGA	CAAGAAAGAG	7050
Catarrhini	--T---GCTG	------TCAT	TAATAGCAAT	AAGTTAGGAA	GGGGATGTGG	CTCTTTTAGT	TGGGAAAAAG	6223
Anolis_carolinensis	----------	----------	----------	----------	----------	----------	----------	1184
Murine_leukemia_virus	GTTCTCTTTG	ACCCGCCAGG	TCCTCAATGT	AGGACCCCG-	-CGTCCCCAT	TGGGCCT-AA	TCCCGTGATC	6375
Phascolarctos_cinereus	----CAGTTG	ACCATACGAC	TAGTGATCAC	AAGTCCCCCA	CCGGTAGTAG	TGGGCCCCGA	TCCTGTCCTC	6177
Estrildidae	----------	----------	----------	----------	----------	----------	----------	-
Xenopus_Silurana_tropicalis	----------	----------	----------	----------	----------	----------	-ATTATTGGC	9
I_XT	TNAAACATAT	NNNTCTATGG	NCAGATAAAT	GGGNTGGTTT	CAGANTGNNA	NGNGTNTCAN	GGTACANNAT	6618
Takifugu_rubripes	----------	----------	----------	----------	----------	----------	----------	-
Bos_taurus	----------	----------	----------	----------	----------	----------	----------	-
Homo_sapiens	----------	----------	----------	----------	----------	----------	----------	-
Microcebus_murinus	----------	----------	----------	----------	----------	----------	----------	-
Myotis_lucifugus	----------	----------	----------	----------	----------	----------	----------	-
Equus_caballus	----------	----------	----------	----------	----------	----------	----------	-
Tupaia_belangeri	----------	----------	----------	----------	----------	----------	----------	-
Loxodonta_africana	----------	----------	----------	----------	----------	----------	----------	-
Murinae	----------	----------	----------	----------	----------	----------	----------	-
Canis_lupus_familiaris	----------	----------	----------	----------	----------	----------	----------	-
Sus_scrofa	----------	----------	----------	----------	----------	----------	----------	-
Choloepus_hoffmanni	----------	----------	----------	----------	----------	----------	----------	-
Vicugna_pacos	----------	----------	----------	----------	----------	----------	----------	-
Gavialis_gangeticus	----------	----------	----------	----------	----------	----------	----------	-
Mammalia	----------	----------	----------	----------	----------	----------	----------	-
Passeroidea	----------	----------	----------	----------	----------	----------	----------	-
Takifugu	----------	----------	----------	----------	----------	----------	----------	-
Pteropus_vampyrus	----------	----------	----------	----------	----------	----------	----------	-
Ornithorhynchus	TTGTAGTTGT	ACAAAAAGCT	GTTTGGGAAG	AAGGTTAATT	TCAGGGGGGT	AGCTTCGTAG	TGTTCAGCAA	1237
Oryctolagus_cuniculus	----------	----------	----------	----------	----------	----------	----------	-
Gallus_gallus	----------	----------	----------	----------	----------	----------	----------	-
Hominidae	TNCANTTTTA	TCCNNNNCGA	NCNNNCNNNN	NNNTNTNNNN	NNCNANNCAN	ATNNNCNNNA	NNCNANTTNN	3405
Metatheria	----------	----------	----------	----------	----------	----------	----------	-
Rodentia	CCAAGCTTTC	TCTTATCTCC	GCTCCAAACC	TTCTCTCTGT	TCTCCCACTC	AGCTCCTCCT	AGCTATGAAG	1023
Monodelphis	----------	----------	----------	----------	----------	----------	----------	-
Consensus	----------	----------	----------	----------	----------	----------	----------	

Conservation 100% 0%

		7,160		7,180		7,200		
Dipodomys_ordii	----------	----------	----------	----------	----------	----------	----------	-
Laurasiatheria	----------	----------	----------	----------	----------	----------	----------	-
Eutheria	----------	----------	----------	----------	----------	----------	----------	-
Tarsius_syrichta	----------	----------	----------	----------	----------	----------	----------	-
Muridae	GCCTCTCCTG	CCTGTGAAGA	AACTGGGACC	TCTGATTCTC	AGGTGANTCC	TCCAGAGAAA	CAGGTGTTCA	1547
Gasterosteus_aculeatus	GATAAACTCA	CCTCTGGAGT	TGCCTCAACC	GAACAGCCAT	TGTAAGCAGG	AAATGGCGTG	GCAGACCTAG	1205
Felis_silvestris	----------	----------	----------	----------	----------	----------	----------	-
Rattus	----------	----------	----------	----------	----------	----------	----------	-
Papio_hamadryas	GAACATACTT	ATACCTCCGA	AGACCAGGAA	GAAGCAAGAG	CCATAGGGGC	TACAGAAAAC	AAAGACACTA	4507
Pan_troglodytes	AGAAAGAC--	----------	----------	--AGTGTGAA	CC--------	---------C	CTAAACAAAA	2465
Ailuropoda_melanoleuca	----------	----------	----------	----------	----------	----------	----------	-
Cavia_porcellus	----------	----------	----------	----------	----------	----------	----------	-
Ictidomys_tridecemlineatus	----------	----------	----------	----------	----------	----------	----------	-
Canis	----------	----------	----------	----------	----------	----------	----------	-
Felis_catus	----------	----------	----------	----------	----------	----------	----------	-
Ochotona_princeps	----------	----------	----------	----------	----------	----------	----------	-
Cercopithecidae	TCCGTAAGTA	GCGTCAGATG	TAAATATCCG	TCCAACCTAA	TCTCTCCTAC	GTTGATAAGA	GCTTCTTTCC	679
Carnivora	----------	----------	----------	----------	----------	----------	----------	-
Mus	TTGACCTCTA	GGCGTATGTT	ATGGCAGTCG	CGAATTAGAA	AGTTACAGGT	GAGGTCAGAT	TAACTATTGT	733
Mus_musculus	----------	----------	----------	----------	----------	----------	----------	-
Rattus_norvegicus	----------	----------	----------	----------	----------	----------	----------	-
Tursiops_truncatus	----------	----------	----------	----------	----------	----------	----------	-
Procavia_capensis	----------	----------	----------	----------	----------	----------	----------	-
Primates	----------	----------	----------	----------	----------	----------	----------	-
Echinops_telfairi	----------	----------	----------	----------	----------	----------	----------	-
Sorex_araneus	----------	----------	----------	----------	----------	----------	----------	-
Canidae	----------	----------	----------	----------	----------	----------	----------	-
Otolemur_garnettii	----------	----------	----------	----------	----------	----------	----------	-
Gallus	----------	----------	----------	----------	----------	----------	----------	-
Callithrix_jacchus	----------	----------	----------	----------	----------	----------	----------	-
Crocodylus_porosus	----------	----------	----------	----------	----------	----------	----------	-
Erinaceus_europaeus	----------	----------	----------	----------	----------	----------	----------	-
rnd-6_family-7426-LTR-ERV1	TGTTAAGAAC	GCTGTGGTCA	CCGTTCCAGC	TTACTTCAAT	GACTCGCAAA	GNCAGGCAAC	TAAGGATGCT	281
Esox_lucius	----------	----------	----------	----------	----------	----------	----------	-
Salmo_salar	----------	----------	----------	----------	----------	----------	----------	-
Didelphidae	----------	----------	----------	----------	----------	----------	----------	-
Latimeria_chalumnae	CCGTGTGGCA	GTTGATTGCA	CATCCACAAA	CCTCGTACAC	GAACCTTCAC	ATGAATCTAA	CCCGATCAAA	755
Monodelphis_domestica	----------	----------	----------	----------	----------	----------	----------	-
Macropus_eugenii	----------	----------	----------	----------	----------	----------	----------	-
Alligator_mississippiensis	----------	----------	----------	----------	----------	----------	----------	-
Chrysemys_picta_bellii	TCTTGAGCGT	ACTCCCTCAG	CTAGCGTGTA	TGCAAGCCAC	AAGGATAGAT	GTCAGGGAAT	GCGCAGACGG	1996
Danio_rerio	----------	----------	----------	----------	----------	----------	----------	-
Taeniopygia	----------	----------	----------	----------	----------	----------	----------	-
Dasypus_novemcinctus	----------	----------	----------	----------	----------	----------	----------	-
LTR_XT	----------	----------	----------	----------	----------	----------	----------	-
Haplorrhini	TCAGCAGCAA	GGCTGCTAAG	ATCATAACAG	GAGAACCAGA	AGAAGAATGT	CCTGATTGTA	ACATTCA-AT	7119
Catarrhini	---GCTATAT	GACCG--AAA	ATAAGTACAT	TTGTCATGAA	TTAGGACTGT	GTGGAAATGA	ATGTGGATAC	6288
Anolis_carolinensis	----------	----------	----------	----------	----------	----------	----------	1184
Murine_leukemia_virus	ACTGACCAGC	TACCCCCATC	CCAA---CCC	GTGCAGATCA	TGCTCCCCAG	GCCTCCTCAT	CCTCCTCCTT	6442
Phascolarctos_cinereus	GCAGAACAAG	GACCTCCCAG	AAAAATTCCC	TTCCTCCCCA	GAGTGCCGGT	TCCTACCCTA	TCACCCCCAG	6247
Estrildidae	----------	----------	----------	----------	----------	----------	----------	-
Xenopus_Silurana_tropicalis	TAAGCATTGT	ATGATCATAA	AAGAACCATT	TTTTTAAAGA	TTATCTGAAA	GAATATCTAT	CTGATCTTTT	79
I_XT	TANNTNNGGT	AATCAGACAA	GAACTACCAN	TATATTCAAC	ANAGCTTATT	CANGGCTNCC	TATGGTAGCA	6688
Takifugu_rubripes	----------	----------	----------	----------	----------	----------	----------	-
Bos_taurus	----------	----------	----------	----------	----------	----------	----------	-
Homo_sapiens	----------	----------	----------	----------	----------	----------	----------	-
Microcebus_murinus	----------	----------	----------	----------	----------	----------	----------	-
Myotis_lucifugus	----------	----------	----------	----------	----------	----------	----------	-
Equus_caballus	----------	----------	----------	----------	----------	----------	----------	-
Tupaia_belangeri	----------	----------	----------	----------	----------	----------	----------	-
Loxodonta_africana	----------	----------	----------	----------	----------	----------	----------	-
Murinae	----------	----------	----------	----------	----------	----------	----------	-
Canis_lupus_familiaris	----------	----------	----------	----------	----------	----------	----------	-
Sus_scrofa	----------	----------	----------	----------	----------	----------	----------	-
Choloepus_hoffmanni	----------	----------	----------	----------	----------	----------	----------	-
Vicugna_pacos	----------	----------	----------	----------	----------	----------	----------	-
Gavialis_gangeticus	----------	----------	----------	----------	----------	----------	----------	-
Mammalia	----------	----------	----------	----------	----------	----------	----------	-
Passeroidea	----------	----------	----------	----------	----------	----------	----------	-
Takifugu	----------	----------	----------	----------	----------	----------	----------	-
Pteropus_vampyrus	----------	----------	----------	----------	----------	----------	----------	-
Ornithorhynchus	TCGTCTTTGC	GGTAGGTTCC	TGTTCCTGGA	GCGAGGTTTG	TCGCTGGATC	CCTGAATCGG	CTCTCATATA	1307
Oryctolagus_cuniculus	----------	----------	----------	----------	----------	----------	----------	-
Gallus_gallus	----------	----------	----------	----------	----------	----------	----------	-
Hominidae	TNCNNNNNTN	CTNGGNCNNC	CGNTAGGACN	CNNCNNNCNG	NTGNNGNCNN	NNNTTTNCNG	GCAGCNTGNN	3475
Metatheria	----------	----------	----------	----------	----------	----------	----------	-
Rodentia	CCCGAACCAG	ATGATTCCTC	CACTATTTTC	GATCCNGCTA	ATGAGCCTCC	CCCTTATCGG	CCTCAACCCA	1093
Monodelphis	----------	----------	----------	----------	----------	----------	----------	-
Consensus	----------	----------	----------	----------	----------	----------	----------	

Conservation 100% 0%

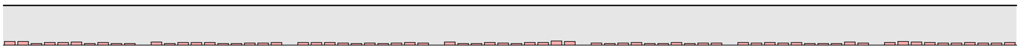

	7,220		7,240		7,260		7,280	
Dipodomys_ordii	----------	----------	----------	----------	----------	----------	----------	-
Laurasiatheria	----------	----------	----------	----------	----------	----------	----------	-
Eutheria	----------	----------	----------	----------	----------	----------	----------	-
Tarsius_syrichta	----------	----------	----------	----------	----------	----------	----------	-
Muridae	CCGTCTTGGA	CCTGAAAGAT	GTATTCTTCA	GCCTCCCATT	GGCAGAGGTG	AGTCAACCCA	TCTTTNCTTT	1617
Gasterosteus_aculeatus	CTAATAGTAC	AACTCCAAAC	CCTAAATGTC	CCAGACATGT	TACTGTTGAT	CAAGCTTTTC	CTACCGCGAG	1275
Felis_silvestris	----------	----------	----------	----------	----------	----------	----------	-
Rattus	----------	----------	----------	----------	----------	----------	----------	-
Papio_hamadryas	GAAACTGGGA	AAAAGAAGGG	AAAATAGTCC	TTCCCCAAAA	GGAAGCCCTG	GCAATGATCC	AGCAGATGCA	4577
Pan_troglodytes	GATGGTGGGA	AATGAAA---	---------C	TCCTCCAGAA	AAAAGC----	----------	----------	2499
Ailuropoda_melanoleuca	----------	----------	----------	----------	----------	----------	----------	-
Cavia_porcellus	----------	----------	----------	----------	----------	----------	----------	-
Ictidomys_tridecemlineatus	----------	----------	----------	----------	----------	----------	----------	-
Canis	----------	----------	----------	----------	----------	----------	----------	-
Felis_catus	----------	----------	----------	----------	----------	----------	----------	-
Ochotona_princeps	----------	----------	----------	----------	----------	----------	----------	-
Cercopithecidae	TCCAGCACTT	TGCTTTGGGA	CCGATCATAC	TGGACTCCCA	GGTTCACCTT	TCGAGCCTGA	CACCAACTTC	749
Carnivora	----------	----------	----------	----------	----------	----------	----------	-
Mus	AGAGCGAGAA	AGTGTCAAGG	GAACGAGAGA	AAGAGAAGAG	AGAGAGAAGA	AGGGAGAGAA	TTGACTAATT	803
Mus_musculus	----------	----------	----------	----------	----------	----------	----------	-
Rattus_norvegicus	----------	----------	----------	----------	----------	----------	----------	-
Tursiops_truncatus	----------	----------	----------	----------	----------	----------	----------	-
Procavia_capensis	----------	----------	----------	----------	----------	----------	----------	-
Primates	----------	----------	----------	----------	----------	----------	----------	-
Echinops_telfairi	----------	----------	----------	----------	----------	----------	----------	-
Sorex_araneus	----------	----------	----------	----------	----------	----------	----------	-
Canidae	----------	----------	----------	----------	----------	----------	----------	-
Otolemur_garnettii	----------	----------	----------	----------	----------	----------	----------	-
Gallus	----------	----------	----------	----------	----------	----------	----------	-
Callithrix_jacchus	----------	----------	----------	----------	----------	----------	----------	-
Crocodylus_porosus	----------	----------	----------	----------	----------	----------	----------	-
Erinaceus_europaeus	----------	----------	----------	----------	----------	----------	----------	-
rnd-6_family-7426-LTR-ERV1	GGCGTTATTG	CAGGTCTNAA	TGTNATGAGN	ATTATCAATG	AACCTACNGC	AGCTGCNATT	GCTTATGGTC	351
Esox_lucius	----------	----------	----------	----------	----------	----------	----------	-
Salmo_salar	----------	----------	----------	----------	----------	----------	----------	-
Didelphidae	----------	----------	----------	----------	----------	----------	----------	-
Latimeria_chalumnae	TTGATCAGAA	ACTTTAAATT	TTCATTCTCG	CCTGCTGGAG	GAATTTAAAT	TTTATCTAGT	CACTAAAAAC	825
Monodelphis_domestica	----------	----------	----------	----------	----------	----------	----------	-
Macropus_eugenii	----------	----------	----------	----------	----------	----------	----------	-
Alligator_mississippiensis	----------	----------	----------	----------	----------	----------	----------	-
Chrysemys_picta_bellii	CAAGCTGCTC	CCTACCCAGG	AGATCTGTGC	ATCCACAGTA	CTCCCAGACT	CCCAAAGAAA	CTCTGGCTGG	2066
Danio_rerio	----------	----------	----------	----------	----------	----------	----------	-
Taeniopygia	----------	----------	----------	----------	----------	----------	----------	-
Dasypus_novemcinctus	----------	----------	----------	----------	----------	----------	----------	-
LTR_XT	----------	----------	----------	----------	----------	----------	----------	-
Haplorrhini	GGACCACACA	TGAGTTCAGC	CAACGCCTTT	ACGCAGGGAG	AGTAGCTCTG	CTTGCCAGCC	AAGAAGCAAA	7189
Catarrhini	TGGTCTTGTG	TCATTTGGGC	CA-CTTGGAT	AAAAAATGAA	AAGGATCCAG	TCCACCTTCA	GAAAGGAAAA	6357
Anolis_carolinensis	----------	----------	----------	----------	----------	----------	----------	1184
Murine_leukemia_virus	CAGGCGCGGT	CTCTATGGTG	CCTGG-GGCT	CCCCCGCCTT	-----CTCAA	CAACCTGGGA	CGGGAGACAG	6506
Phascolarctos_cinereus	CGTCCCCTAT	CCCCACGGTA	CAGGCTAGCC	CCCCGGCCCC	TAGTACTCCA	TCTCCCACCA	CGGGCGACCG	6317
Estrildidae	----------	----------	----------	----------	----------	----------	----------	-
Xenopus_Silurana_tropicalis	TGACCCCTTT	AGAATATATA	ANAATGGTTG	TNCCTTATCA	GACAAAACCA	GAACAATAAT	AAT----GAC	145
l_XT	TNNNGATATT	GTACCATANA	AAGNGNTATA	NCGGTTATCA	GACAANATCT	GAACATCNNA	NACANAGNAA	6758
Takifugu_rubripes	----------	----------	----------	----------	-----TCCAT	CGACCTGACT	GTTCGTTGAG	25
Bos_taurus	----------	----------	----------	----------	----------	----------	----------	-
Homo_sapiens	----------	----------	----------	----------	----------	----------	----------	-
Microcebus_murinus	----------	----------	----------	----------	----------	----------	----------	-
Myotis_lucifugus	----------	----------	----------	----------	----------	----------	----------	-
Equus_caballus	----------	----------	----------	----------	----------	----------	----------	-
Tupaia_belangeri	----------	----------	----------	----------	----------	----------	----------	-
Loxodonta_africana	----------	----------	----------	----------	----------	----------	----------	-
Murinae	----------	----------	----------	----------	----------	----------	----------	-
Canis_lupus_familiaris	----------	----------	----------	----------	----------	----------	----------	-
Sus_scrofa	----------	----------	----------	----------	----------	----------	----------	-
Choloepus_hoffmanni	----------	----------	----------	----------	----------	----------	----------	-
Vicugna_pacos	----------	----------	----------	----------	----------	----------	----------	-
Gavialis_gangeticus	----------	----------	----------	----------	----------	----------	----------	-
Mammalia	----------	----------	----------	----------	----------	----------	----------	-
Passeroidea	----------	----------	----------	----------	----------	----------	----------	-
Takifugu	----------	----------	----------	----------	----------	----------	----------	-
Pteropus_vampyrus	----------	----------	----------	----------	----------	----------	----------	-
Ornithorhynchus	TGCTCTGAAG	CGGCCCCAGA	CCCTTGGCGA	GCCGCAGCTT	CATCCTTACT	TCGCTTAGGA	ACGCCCCTGC	1377
Oryctolagus_cuniculus	----------	----------	----------	----------	----------	----------	----------	-
Gallus_gallus	----------	----------	----------	----------	----------	----------	----------	-
Hominidae	NCNAGNNCNN	NNGGCTNGCN	NNCCTGCCTN	NNNGNCTNNN	CAGCNNCNNN	NGNNNTTAAT	CCNNNNNNNG	3545
Metatheria	----------	----------	----------	----------	----------	----------	----------	-
Rodentia	CAGCTCCTCT	CCCCCNCTCC	CTCCTGTCTC	CCTTTCTCCC	TCTTCAGCAA	CAGGCCGCCC	AAATCAACTC	1163
Monodelphis	----------	----------	----------	----TGTGAA	GATTAAATTT	AACTCTCCCC	TGATTGTNAA	36
Consensus	----------	----------	----------	----------	----------	----------	----------	

Conservation
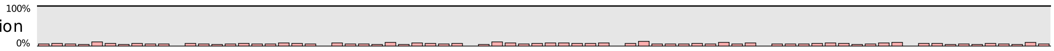

		7,300		7,320		7,340		
Dipodomys_ordii	----------	----------	----------	----------	----------	----------	----------	-
Laurasiatheria	----------	----------	----------	----------	----------	----------	----------	-
Eutheria	----------	----------	----------	----------	----------	----------	----------	-
Tarsius_syrichta	----------	----------	----------	----------	----------	----------	----------	-
Muridae	TAANTGAAAA	GACCCANCGG	GAGATTATAG	TGGGCAACAT	AACTGAACNA	NTTTGCCCCA	GTATTTAAAG	1687
Gasterosteus_aculeatus	TTTCATTATA	GAATTCTTAC	CGTCTCAGGG	TACAATCCCG	GTTAAGAGTT	AGAATTTGTC	TCCAGGCTTC	1345
Felis_silvestris	----------	----------	----------	----------	----------	----------	----------	-
Rattus	----------	----------	----------	----------	----------	----------	----------	-
Papio_hamadryas	TGCCTGGACA	CACTTGGGTA	ATCGAAAGCT	AAAATTGTTA	ATTGAAAAAA	CTGACTTTCT	AATCCCAAGG	4647
Pan_troglodytes	---------A	CAGTGTAACA	ATCCACAACC	---ACTGCCG	GGTGAACCTA	TCACCAATCA	AATCC-----	2552
Ailuropoda_melanoleuca	----------	----------	----------	----------	----------	----------	----------	-
Cavia_porcellus	----------	----------	----------	----------	----------	----------	----------	-
Ictidomys_tridecemlineatus	----------	----------	----------	----------	----------	----------	----------	-
Canis	----------	----------	----------	----------	----------	----------	----------	-
Felis_catus	----------	----------	----------	----------	----------	----------	----------	-
Ochotona_princeps	----------	----------	----------	----------	----------	----------	----------	-
Cercopithecidae	ATAGTGATGA	TTTCGATTGA	CCTTACTGGA	AGGTACGGCC	AAGCCATTGC	GTACTACTGG	TTAGTACGGA	819
Carnivora	----------	----------	----------	----------	----------	----------	----------	-
Mus	GGCGCAGTGA	GATCAGAGGG	ACCTGGACAG	CAGGAGCCTG	AAACCAGCTA	TGCAAAAAGA	TGGAGATGCC	873
Mus_musculus	----------	----------	----------	----------	----------	----------	----------	-
Rattus_norvegicus	----------	----------	----------	----------	----------	----------	----------	-
Tursiops_truncatus	----------	----------	----------	----------	----------	----------	----------	-
Procavia_capensis	----------	----------	----------	----------	----------	----------	----------	-
Primates	----------	----------	----------	----------	-----GAGGG	CGCCCGGAGT	CCACCCCAGC	25
Echinops_telfairi	----------	----------	----------	----------	----------	----------	----------	-
Sorex_araneus	----------	----------	----------	----------	----------	----------	----------	-
Canidae	----------	----------	----------	----------	----------	----------	----------	-
Otolemur_garnettii	----------	----------	----------	----------	----------	----------	----------	-
Gallus	----------	----------	----------	----------	----------	----------	----------	-
Callithrix_jacchus	----------	----------	----------	----------	----------	----------	----------	-
Crocodylus_porosus	----------	----------	----------	----------	----------	----------	----------	-
Erinaceus_europaeus	----------	----------	----------	----------	----------	----------	----------	-
rnd-6_family-7426-LTR-ERV1	TGGATAAAAA	GGCTACCAGT	GTAGGTGAGA	AGAACGTGCT	CATCTTCGAT	CTGGGTGGTG	GTACTTTCGA	421
Esox_lucius	----------	----------	----------	----------	----------	----------	----------	-
Salmo_salar	----------	----------	----------	----------	---TAATCAG	AGAAAAATTT	AACCTTTAAA	27
Didelphidae	----------	----------	----------	----------	----------	----------	----------	-
Latimeria_chalumnae	CTTGCTCCTC	CGTAGCCAAC	AGAATGGTGC	CGGCTAATAA	AATATTGGAC	CCTGACTGTG	CTGCTTGCCA	895
Monodelphis_domestica	----------	----------	----------	----------	----------	----------	----------	-
Macropus_eugenii	----------	----------	----------	----------	----------	----------	----------	-
Alligator_mississippiensis	----------	----------	----------	----------	----------	----------	----------	-
Chrysemys_picta_bellii	CAAGTCTTTT	TCCCAGGGTT	CCAAGCTATC	TGCACTCAAC	CAGGGCAAGA	GGTTTATAAA	ATTCCCGGTA	2136
Danio_rerio	----------	-----TTTAT	AATAATAGTT	AATTTTTATG	TTAAAGTTAT	ATATTAAAGA	TATTTTTAAT	55
Taeniopygia	----------	----------	----------	----------	----------	----------	----------	-
Dasypus_novemcinctus	----------	----------	----------	----------	----------	----------	----------	-
LTR_XT	----------	----------	----------	----------	----------	----------	----------	-
Haplorrhini	GATTGGT---	--TGTGCGAC	TAAAACATGC	AACCCCCTTA	ATCTGACCAT	ATTAAAGCCA	AACATGCCTT	7254
Catarrhini	AATGGCCCTT	CCTGTACTAA	GGGACAATGT	AACCCCTTAG	AGCTAGTAAT	AACCAATCCC	CTTGATCCTC	6427
Anolis_carolinensis	----------	----------	----------	----------	----------	----------	----------	1184
Murine_leukemia_virus	GCTGCTAAAC	CTGGTAGAAG	GAGCCTACCA	AGCAC--TCA	ACCTCACCAG	TCCCGACAAA	ACCCAAGAGT	6574
Phascolarctos_cinereus	GCTCTTTGGC	CTCGTACAAG	GGGCCTTTCT	GGCCT--TGA	ATGCCACCAA	CCCGGAGGCC	ACGGAGTCTT	6385
Estrildidae	----------	----------	----------	----------	----------	----------	----------	-
Xenopus_Silurana_tropicalis	ANTANAATAT	NAGTTCCAGN	GTTATTCACA	AANCAAATGG	AATTATTTAN	CATTAGTACA	ATNAATGTGT	215
l_XT	ATCAANTTAT	CCACATGANN	GNANTTNACN	AAAAAAAGNN	TTTNNTTTCC	ACCTCAGATN	AANAAGGTAT	6828
Takifugu_rubripes	GTCATGTGTC	AGGGGTGAGG	CGGGACAGNG	CAGNGTAAGG	TANAGNNNAG	--CTCAAGCC	AGNNGNTNCN	93
Bos_taurus	----------	----------	----------	----------	----------	----------	----------	-
Homo_sapiens	----------	----------	----------	----------	----------	----------	----------	-
Microcebus_murinus	----------	----------	----------	----------	----------	----------	----------	-
Myotis_lucifugus	----------	----------	----------	----------	----------	----------	----------	-
Equus_caballus	----------	----------	----------	----------	----------	----------	----------	-
Tupaia_belangeri	----------	----------	----------	----------	----------	----------	----------	-
Loxodonta_africana	----------	----------	----------	----------	----------	----------	----------	-
Murinae	----------	----------	----------	----------	----------	----------	----------	-
Canis_lupus_familiaris	----------	----------	----------	----------	----------	----------	----------	-
Sus_scrofa	----------	----------	----------	----------	----------	----------	----------	-
Choloepus_hoffmanni	----------	----------	----------	----------	----------	----------	----------	-
Vicugna_pacos	----------	----------	----------	----------	----------	----------	----------	-
Gavialis_gangeticus	----------	----------	----------	----------	----------	----------	----------	-
Mammalia	----------	----------	----------	----------	----------	----------	-----TGTAA	5
Passeroidea	----------	----------	----------	----------	----------	----------	----------	-
Takifugu	----------	----------	----------	----------	----------	----------	----------	-
Pteropus_vampyrus	----------	----------	----------	----------	----------	----------	----------	-
Ornithorhynchus	CCTTGGGCTC	CCGATAAAAA	CCTTCCCCTG	TTGTCATGAG	TGGGTGGCGT	GGGTCCTTGC	CAACTGGCCC	1447
Oryctolagus_cuniculus	----------	----------	----------	----------	----------	----------	----------	-
Gallus_gallus	----------	----------	----------	----------	----------	----------	----------	-
Hominidae	NCCNCNANCT	TAGTCTCTCN	NNCNCCNNCT	CNNNNTNNAC	NNCCNCCANC	NNNNNACNNN	NNTNCTCNCT	3615
Metatheria	----------	----------	----------	----------	----------	----------	----------	-
Rodentia	TCCCCGGCAC	CCACTTTCAG	CCCACCGGCA	GCCCCTCCTC	TGCTACTCCC	GCTCCGCCAT	AGGACCCCCT	1233
Monodelphis	NATGNAATTN	ANNAANNCNN	NNCTNNTNGN	NNAGNNNNAN	NTGNNNNNCC	TGCCCANTTT	TAGATTTANT	106
Consensus	----------	----------	----------	----------	----------	----------	----------	

Conservation

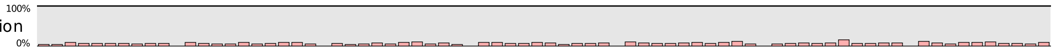

	7,360		7,380		7,400		7,420	
Dipodomys_ordii	----------	----------	----------	----------	----------	----------	----------	-
Laurasiatheria	----------	----------	----------	----------	----------	----------	----------	-
Eutheria	----------	----------	----------	----------	----------	----------	----------	-
Tarsius_syrichta	----------	----------	----------	----------	----------	-------GAC	AAGAAAGGTT	13
Muridae	ACACTAAATG	CCGACCTCCT	GTCTTTCCGT	CAAAGGTTTC	CTAGGANNAT	AGTTGCTTCT	GCAAGAGTTG	1757
Gasterosteus_aculeatus	AGTAGTGAGG	CAGCCCAAGG	TAAAGACCTT	TAGCGGGCAA	GGCAGTCAAG	TNCAGGTTGN	NTGACGANAN	1415
Felis_silvestris	----------	----------	----------	----------	----------	---------T	GAGA-GACCC	10
Rattus	----------	----------	----------	----------	----------	----------	----------	-
Papio_hamadryas	GCAAGTACAC	TCATAGAGCA	AGTGACATCT	GCCTGTAAGG	TCTGTCAGCA	GGTAAACGCT	GGGGCTACCC	4717
Pan_troglodytes	--AGCTGAAC	CCAGAGATGA	CGTTGCA---	--------AG	TCTGCCAGCA	---------C	AACGCTCCCC	2600
Ailuropoda_melanoleuca	----------	----------	----------	----------	----------	----------	----------	-
Cavia_porcellus	----------	----------	----------	----------	TGTTATAGAC	AGGTNATAGN	CAAATTTCAT	30
Ictidomys_tridecemlineatus	----------	----------	----------	----------	----------	----------	-ACAATTTAT	9
Canis	----------	----------	----------	----------	----------	----------	----------	-
Felis_catus	----------	----------	----------	----------	----------	----------	----------	-
Ochotona_princeps	----------	----------	----------	----------	----------	----------	----------	-
Cercopithecidae	TTCCCCCATT	TCCTGGCGGA	GGGTTAGTGG	ATCCCGTTCG	CGCTGCCAAG	CCCCTTATCA	GGCANCCAAG	889
Carnivora	----------	----------	----------	----------	----------	----------	----------	-
Mus	AAAAGGGTGG	ATACACCTAC	TAAAAGCTCA	GTTGCAAGAT	ACTATGGATA	CTGAGAGACG	TGGTCGAAGC	943
Mus_musculus	----------	----------	----------	----------	----------	----------	----------	-
Rattus_norvegicus	----------	----------	----------	----------	----------	----------	----------	-
Tursiops_truncatus	----------	----------	----------	-------CGC	AGAAATCAAT	CTTGGTGATT	AAGAACTCTA	33
Procavia_capensis	----------	----------	----------	----------	----------	----------	----------	-
Primates	CTGGCGGCCC	ATGAACAGAC	TTTACGCCAA	TTGCTTTGNC	CCGCCACGCC	CCCCTGTACC	CATAANAACC	95
Echinops_telfairi	----------	----------	----------	----------	----------	----------	----------	-
Sorex_araneus	----------	----------	----------	----------	----------	----------	----------	-
Canidae	----------	----------	----------	----------	----------	--TGATGTAG	GAACGAGTTA	18
Otolemur_garnettii	----------	----------	----------	----------	----------	----------	----------	-
Gallus	----------	----------	----------	--------TG	AAGNGTGANA	ATGGNCNNCA	GTAGNGNGAA	32
Callithrix_jacchus	----------	----------	----------	----------	----------	----------	----ATGTTG	6
Crocodylus_porosus	----------	----------	----------	----------	----------	----------	TTGCTTATCT	10
Erinaceus_europaeus	----------	----------	----------	----------	----------	----------	----------	-
rnd-6_family-7426-LTR-ERV1	TGTGTCTCTT	CTCACTATCG	AAGAGGGGAT	TTTTGAGGTC	AAAGCTACTG	CTGGNGATAC	TCATCTNGGA	491
Esox_lucius	----------	----------	----------	----------	----------	----------	----------	-
Salmo_salar	CTTTCGGATG	ATCATATGTA	GATGATATGT	TGAAGNTGTN	TAAATCAAAG	NAAAGATGTN	AAATNGACAC	97
Didelphidae	----------	----TGGGAA	GAGANTTNTT	CCAGACATGA	GAANTNACAG	NAAGGAACTG	NAAGATTCCC	56
Latimeria_chalumnae	TTCTCCTTTA	TTCATGATCA	AAAAATGTCT	TACAAAATTG	CCTATAGAAG	AAACCACCTT	AAAATTTTTA	965
Monodelphis_domestica	----------	----------	----------	----------	----------	----------	----------	-
Macropus_eugenii	----------	----------	----------	----------	----------	----------	----------	-
Alligator_mississippiensis	----------	----------	----------	----------	----------	----------	-CTTATCAAA	9
Chrysemys_picta_bellii	ACCTGCACCA	GCGAATGTTA	TTGCCAAAAG	AAACTGAGGT	AGTGCGCCCG	GCCCCCAGGC	CTGTGGCAAA	2206
Danio_rerio	AAAAAAATAT	TGTTAAATAT	TTTATAAACT	TTTGTATTTA	TATANGTNNN	NTTAGAAAAT	TTTCAATATA	125
Taeniopygia	----------	----------	----------	----------	----AATAAT	ATAAAATAAT	AATTATTTGA	26
Dasypus_novemcinctus	----------	----------	----------	----------	----------	----------	----------	-
LTR_XT	----------	----------	----------	----------	----------	----------	----------	-
Haplorrhini	TTTGGACTAA	AGGACACCAA	GGAGAGCTAA	GCTTTGATCG	AGAAGGAGCA	AATCTAGGTA	TTCCGTTAAT	7324
Catarrhini	GCTGGAAAAA	AGGGGAGCGT	G------TGA	CCTTAGGAAT	CGACGGGGCC	GGACTGGATC	CTCGAGTAAA	6491
Anolis_carolinensis	----------	----------	----------	----------	----------	----------	----------	1184
Murine_leukemia_virus	GCTGGCTGTG	TCTGGTATCG	GGA---CCCC	CCTACTACGA	AGGGGTTGCC	GTCCTAGGTA	CCTACTCCAA	6641
Phascolarctos_cinereus	GCTGGCTCTG	TCTAGCCCTC	GGC---CCTC	CTTATTACGA	AGGAATCGCA	ACTCCAGGG-	--CAAGTCAC	6449
Estrildidae	----------	----------	----------	----------	------TA-A	AAT-TAT---	TTACAGCAAA	19
Xenopus_Silurana_tropicalis	TCCCNTTGAA	AGTATNNANA	AGAATTNNTC	NATAGATGAA	TAAGNATA-N	NNT-TATAGG	TTATGATANA	283
I_XT	NTCATATCTN	NCTGCCCTGT	TNGAATTGNN	AATANNATAG	TCAANATATN	AAT-TAA---	TTACACTAGA	6894
Takifugu_rubripes	TATNCNGGAG	NGNAAAAGGA	AAGNTTCANC	CNTGCTNTNC	CCAATTGGCG	GANATCNANN	CTATGNNNNC	163
Bos_taurus	----------	----------	----------	----------	----------	----------	----------	-
Homo_sapiens	----------	----------	----------	----------	----------	----------	----------	-
Microcebus_murinus	----------	----------	----------	----------	----------	----------	----------	-
Myotis_lucifugus	----------	----------	----------	----------	----------	----------	----------	-
Equus_caballus	----------	----------	----------	----------	----------	----------	----------	-
Tupaia_belangeri	----------	----------	----------	----------	----------	----------	----------	-
Loxodonta_africana	----------	----------	----------	----------	----------	----------	----------	-
Murinae	----------	----------	----------	----------	----------	----------	----------	-
Canis_lupus_familiaris	----------	----------	----------	----------	----------	----------	----------	-
Sus_scrofa	----------	----------	----------	----------	----------	----------	----------	-
Choloepus_hoffmanni	----------	----------	----------	----------	----------	----------	----------	-
Vicugna_pacos	----------	----------	----------	----------	----------	----------	----------	-
Gavialis_gangeticus	----------	----------	----------	-----AACCA	TATNNTGACA	TNGATCCTAC	TGNAGCCTTA	35
Mammalia	TGGGATAATA	TTTGAAATAT	ATTAATGTGC	TTTTCCTTTT	CTTTGTGACC	TTCTCCCTTC	AGCGAGGCAT	75
Passeroidea	----------	----------	TGTTATGTGT	AATTTGGATA	ATTCGNGCNT	ATAGAATAAN	ATNTATGTAA	50
Takifugu	----------	----------	--TGAAGAGA	ATTGTGTTTA	TCTTGTTATC	ATGTGTTNTA	CCATATGT--	46
Pteropus_vampyrus	----------	----------	----------	----------	----------	----------	----------	-
Ornithorhynchus	TCGCCGGTGC	ATATATTCTC	ATGACCGTTA	TAGCAGTCCC	CTGTATCGTG	ATTGCAGCGC	GTCTTGATGC	1517
Oryctolagus_cuniculus	----------	----------	----------	----------	----------	----------	----------	-
Gallus_gallus	----------	----------	----------	----------	----------	----------	----------	-
Hominidae	CNCNNNNNTC	TNNCNNNNNT	NNNNCNCTGC	CNNNATNNNN	NTNNTTCNNN	NGCNNTTNGN	CNNNNNNNNN	3685
Metatheria	----------	----------	----------	----------	----------	-------TGT	GTGAATTTAA	13
Rodentia	TTACCTCCCC	GTACCCGGCC	TAACCCGGCC	ATNGTTCTCC	CTTTGCGGGA	AGTTGCCGGG	GTGGACGGCT	1303
Monodelphis	CNNNNANANT	GNANACNCCC	TACTTAAAAG	NTNNNNNNNG	NNNNCTGNNC	ATNNNTNGAN	TNNANCNNNA	176
Consensus	----------	----------	----------	----------	----------	----------	----------	

Conservation

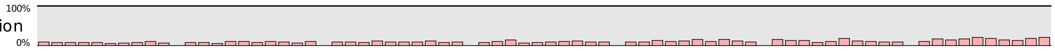

	7,440		7,460		7,480			
Dipodomys_ordii	----------	--------GG	TCTGAACTAG	AATNACTNCA	TTTTGAGCNT	NGNCTCCNTN	TTAGNGTTTN	52
Laurasiatheria	----------	----------	----------	----------	-------TGT	TAAATTAATT	A-AACAAGGA	22
Eutheria	----------	----TAAATC	TTCTGCTNAG	TAAAATCAAA	TNGAGCTNTT	TNGCCTAAAG	CTCCCTNCTT	56
Tarsius_syrichta	TNAAAGANTT	GNAATANCTT	GTTTGATAAG	TAACTTCATN	TTTTTAAAAA	TCGAC-AATC	TTATCTGTNT	82
Muridae	CAGACCTTGG	GCTACAGAAT	GTCGGCTAAG	AAAGCCCAAC	TTTATANATC	AGAGGCTACC	TATCTTGGCT	1827
Gasterosteus_aculeatus	GTNTNTTTCT	TNGGANGAAN	GCTNTCGGTG	ANGNNTNAAN	NCTNTCACCA	GAACACANGT	NTNTCAGTTT	1485
Felis_silvestris	CTGGCTTAGC	TTAATAAAAC	ACCCCCACCC	TATCTTCGGC	TTTGTAAACG	CCTCACTTGC	AG---AGCTT	77
Rattus	----------	----------	----------	----------	----------	AGAATNAACT	TCACTGAGAT	20
Papio_hamadryas	GAGTGCCAGC	AGGGAAACGG	ACTCGTGGTA	ACCGCCCGGG	AGTCTATTGG	GAAATAGACT	TCACTGAAGT	4787
Pan_troglodytes	CAGCGCAGGA	AAGGTGGGGA	CGCCG-----	----------	GATCTACTGG	GAAGTAGATT	TTACTGAAAT	2655
Ailuropoda_melanoleuca	----------	----------	----------	----------	----------	--------GT	ATGNNAAAGN	12
Cavia_porcellus	TCCAGTCAGA	NAGAGAATTG	ANTCTCGAGA	NAGACNNTCC	NACCTCAGCT	ATCAGNAAAG	AGNAGAAGGA	100
Ictidomys_tridecemlineatus	TAAAGTCAGA	ATTAAAATTT	ACACAAGTTG	TTAACAA--A	TTCTTTGTTT	ANNAGTAAAA	TATAGAA--A	75
Canis	----------	----------	----------	-----TGTGG	GNCCCNGNAN	ANTTAAGAAA	AATCCCC--T	33
Felis_catus	----------	----------	----------	----------	----------	---------C	AAGCTAGANT	11
Ochotona_princeps	----------	----------	----------	--GTCATGAA	AGTNTAAGGC	CATTGAGAAA	CNACTGCNTT	38
Cercopithecidae	GAACTACACC	CCTTANAGCG	GTCCGACCCA	GCATGAGTTG	GCTTTTGAGN	AGNTTCAAGA	CTTCCNNCTC	959
Carnivora	----------	----------	----------	----------	---------T	TG-ATGTGGA	GAAAAAAGGC	20
Mus	GAAAACTGTA	CAGATCCCAC	CCCACACGCA	GTAAGAGAGT	TCCTGGGAAN	TGCANGGTNT	TNNANNCTCT	1013
Mus_musculus	----------	------GATA	CTACTAAAGA	AAGTCTGAGC	TCNAGGACTC	CCTTAACCAA	ATC-AACNTT	53
Rattus_norvegicus	----------	----------	----------	----------	----------	--------TA	AGC-ACTGAT	11
Tursiops_truncatus	TTATAACTAT	TGTTANAAAG	CAAACACCCA	CCGACTGANT	CCATGGGATT	TAAANGCTGG	GCCTAACANT	103
Procavia_capensis	----------	----------	----------	----------	--AACTNAAT	TAAACCAANA	NCGAGATAAT	28
Primates	ANACTCCAAA	CCCAAGNNTA	ANACCAGCCG	CAGAAANGTN	CCATTAGNAA	TNTACGTTAA	TGCGTTCGTG	165
Echinops_telfairi	----------	----------	-----TTTAG	GAAGNCAAC-	--ATTGGCAC	CATGGNNGNN	TCC-GTTACT	41
Sorex_araneus	----------	----------	----TGTTAG	GNAGAGAGCC	AAACTGCCGC	CATGACAGGC	TNCAGNTAAT	46
Canidae	GGGGCAGNCG	GGGCTAGGCA	GGGAAAGNTA	NGGGAAAGGC	CNCNGAGTCA	GGCTCCNACC	CATCCCAAGA	88
Otolemur_garnettii	----------	----------	----------	----------	----------	----------	----------	-
Gallus	NGGACCCGCA	GNAACAAANG	GCAANNTATG	TGACNGATTA	GNNATAGGGG	CGTACAGAGN	NNNCTNAGGT	102
Callithrix_jacchus	TATCAGGGCN	GGAATAGCNA	GNCANCATCN	AGANAAGAGG	GTGCCAAATT	NCAGGCTCTN	TGTTTCCGGC	76
Crocodylus_porosus	CACGGNAATC	AAGAGCCCAA	AACTTCAGGT	CTTGCACTGA	TACCTGTCTT	GGAGAAGCNA	AGCCCTTAAC	80
Erinaceus_europaeus	--------TT	GTATGNNNNA	TGNTATGTGN	NCTTNCNTCT	TGGC-NNCCC	AGANACNNTG	TNAGATGTA-	60
rnd-6_family-7426-LTR-ERV1	GGTGAGGATT	TTGACAATAG	GATGGTGAAT	CGTTTCGTNA	NGGA-GTTTA	AGAGGAAGCA	TAAGAAGGAT	560
Esox_lucius	--------TG	TTG-TGGAAA	TTTT-TGAAC	TGATGTATAC	TTAC-TCATA	ANGTATNAAG	GGGCTACTAT	59
Salmo_salar	TAGAGCANTG	TTAATNTTAA	TNNCATGAAA	AAGNTCANAA	AGTT-TCACN	TAGNAAGNTA	NAGCTAGNNT	166
Didelphidae	CTGGAGACCT	NGGAGAAGAG	NTTCCNAAAN	GACTATGACA	GGAG-GGACA	AGACATCACA	GAACTGTNAT	125
Latimeria_chalumnae	TAAATTAAAT	TTGTGACAAA	TGTAATTAAT	TTTTGAAAAA	ATAA-AATCA	GCTCACAATA	AGTTTGGNNT	1034
Monodelphis_domestica	-----TGGAA	TCGT---AGA	TGGAA--AAT	ACTTCTAGGA	AAAG-ACTCC	AGTAAAGAAN	AGTTTATCC-	58
Macropus_eugenii	----TTGAAT	GCNATGNAGC	TCNTAANCAT	TTCTCNGNAA	TTNA-CANTA	GGTTTCNANA	NGCCNCCTT-	64
Alligator_mississippiensis	TGATTTTTAA	TCCAATTGGT	ACAAATA---	-----TTCTT	GTATTNATAG	NTTCTGGATG	GATAGAAGCC	71
Chrysemys_picta_bellii	TGATAT---A	TCACAAGGGT	ACANATACCT	GTTGGTTTTA	GTNGATATAT	TNACNGGATG	GCTCGAAGCC	2273
Danio_rerio	TTTNTACAAA	GTNAAAATGN	AATTTCAATA	TATTAAAATA	ATAANAAAAT	AT-GTAAAAA	TATATAAATT	194
Taeniopygia	TTGTTTAAAT	TAGCAAAAGC	AGTTTTAGTG	TGAGAAAA-A	AAANATNAAC	NG-ATAATTN	CNTAGAAGGA	94
Dasypus_novemcinctus	----------	-----GATAG	GGCTGTGCAT	GATTCAAATA	CCGAGTTGAT	GGCATTGAGT	AATATAGGCC	55
LTR_XT	----------	----------	----------	-------GTA	GTATATGTTA	GGTNTGCATG	AATNTNANN-	32
Haplorrhini	CATTATTAAA	AAGACTCAAC	GAGCTAAAGT	TCAAGTCAGT	CCAATGTCAC	AGTTCAGATT	TTTCAAATCC	7394
Catarrhini	TATCTT----	--GGTTCGAG	GAG---AAGT	TTA---CAAA	C-ACTCTCCT	GAGCCAG-TG	TTTCAAA-CT	6546
Anolis_carolinensis	----------	----------	----------	----------	----------	----------	----------	1184
Murine_leukemia_virus	CCATAC----	---CTCTGCC	CCGGCTAACT	GCTCCGTGGC	CTCCCAACAC	AAGCTGACCC	TGTCCGAAGT	6704
Phascolarctos_cinereus	TTATGC----	---CTCCACA	GATTCCCAAT	GTCGCTGGGG	AGGAAAAGGG	AAGCTCACCC	TCACTGAAGT	6512
Estrildidae	TT-TG-----	ATGCGTTAGG	ATGTTAGGTT	ATATATTTAG	-CANGAAACT	GTA----CTG	GTTTAGTGTG	78
Xenopus_Silurana_tropicalis	ATATA-----	ACATCTTATA	AGTTTTTNTT	AAATTNGTAN	ATGATAAATT	TTA----ATT	NANGAAGATG	344
I_XT	TAANGG----	ATGGTANAAT	ANNTTNAACT	ATAANTTCCA	NCAATANANT	NANTA-AANN	ATTCAAGNCN	6959
Takifugu_rubripes	NNNTTA----	CTATTTTGCT	GNCTNCTATA	GGGTTGCAAT	NTNTGCCATN	GTGTATGNTT	ACGGTGNGNN	229
Bos_taurus	-----AAAAT	GAAATATAGT	TGACCCAGGG	AACAGACCCT	CGTTAAATTC	GAAATCATCA	ACGGATATAT	65
Homo_sapiens	----------	---------T	AAAACCACTA	AATTAAAAAC	AATTAACCTC	TTTCCTAAAA	GATAGTA-AC	50
Microcebus_murinus	----------	----------	----------	----------	----TTACCA	GCAACAAACA	NGTCCCANAT	26
Myotis_lucifugus	----------	----------	----------	----------	------GGAG	ACAAAAAAGA	TATAGCAACC	24
Equus_caballus	----------	----------	-------ATG	A-ATTNCAAT	TGACTACCTA	AAAAATAAAA	TNTAATATTC	42
Tupaia_belangeri	----------	----------	---AAATATA	ATATACCCTT	TNACCAAGAC	AAGTCTANTA	ATTGACTCTT	47
Loxodonta_africana	----------	----------	----------	AGGACACGAT	GTTTCAGACN	GGNCANAACN	GGTCCCGNGC	40
Murinae	----------	-----TTAGA	CTGNNCACTN	ACTTTGGGTC	ANACTACTNN	AGNAATACAN	TNTAANANTC	55
Canis_lupus_familiaris	----------	----------	AATGACAAGG	ATGAGACTAA	AATAGAGNAG	GCATNCTAGC	TAACAGNNTC	50
Sus_scrofa	----------	----------	----------	--------AG	TAGAAGGACA	ACAATACAAG	TATCATCGCT	32
Choloepus_hoffmanni	----------	----------	----------	ATAATATAGT	GACTGAAAGA	GGAAATAGNA	AATAAAAGGG	40
Vicugna_pacos	----------	----------	----------	--GATATTAT	GANTTCTAGT	AGACCTTGAG	CATCAATACA	38
Gavialis_gangeticus	TANTTTNCNT	AAGTTGATCT	GTGTNATGN-	-ATTNTAATC	NGNACANATA	AGCCCTNACA	TTCTGTNTCT	103
Mammalia	CCATTGCCTC	GGGAGGATTA	GGATTGTTAC	AGCAGGAATG	CAAAGAGAAA	GAAGTTAATA	TTCAGCTTCT	145
Passeroidea	TATTTTATAT	TGTAAGAAAA	AANCNNGCA-	-AGTCGGAGC	CGGGAGTTGC	NT--CACNTA	TNTTGAAACC	116
Takifugu	TTCTGTTTTC	TGTTANANNT	ANTTTAGAA-	-GTTAGGGAT	NGTANAATCN	TTAGNAAGTG	TAATAAAGAT	114
Pteropus_vampyrus	----------	--------AA	AAGGCAAGA-	-GTTTGAATC	CGGCCACATC	C------ATG	TACNGNAGAT	44
Ornithorhynchus	CAGGACTTTC	TGGGCACCGG	GTTTACCGCC	CCGTCGAAGG	GCCACACAGG	CATTNTCCGN	TTCCCGTNTG	1587
Oryctolagus_cuniculus	----------	----------	----------	-CATAGGATG	CGCTCCTAAA	AGTTA----C	TTTAGCCAGA	35
Gallus_gallus	----------	----------	----------	----------	--AGATAGAG	GGTGAGGAGA	TTTAACCACA	28
Hominidae	NATATCNCNN	TNNNNNCCNN	NNNNNCTNTT	AACCCTGCNA	CACNTNNNNC	CNTNGNNNCC	GTCTNANCCC	3755
Metatheria	TATA----AA	TTGTACCCCT	CTTTCCTTTT	AGAAAGG-AA	AGTTTTGAAA	ATCCATTTCG	GTCTCAGCCC	78
Rodentia	TAGGGTA-CA	TGCTACATGT	CCCTTCCATT	AAGCGAA-CT	CTCTTAGAGA	AGAGANTGGG	GTCCTACACT	1371
Monodelphis	ANGGTGTAAA	NANNNNANTC	NNNNTTGNGT	GNGNNANGNN	TNTGCTANNN	ACGTNNNNNN	TNNTNGGTNN	246
Consensus	----------	----------	-------A--	-AAT-ANAAA	TTANTAAAAA	AGAAAAAATA	TATCAAANCT	

Conservation

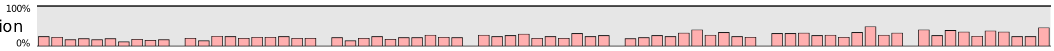

```
                                   7,500                      7,520                      7,540                      7,560
Dipodomys_ordii               GGNNTANNAA ------NCAT GCTGCTTCCA NTGANCCCAG CNTGTGTTGN AANTGAAAGA ACAACNCTGN 116
Laurasiatheria                GGCCATTAGA ------CTGA GGTGGTTCTA ATG-CCTTAG CAGCCTACGT AAGCAAACCA AAACCTAAGC 85
Eutheria                      ACCAGGCNGN -------CAT ANANANCTAA CTCCANCTCC TAA--GTCAC TAT---GTNA ACAACCAGAA 114
Tarsius_syrichta              ACATAACCTA -------AAA ATNACCCCCC TTACGTGTGA TGATTGTAAC CTTCCCGTTN NCACACAACC 145
Muridae                       ACCAGCTGAA GGGAGGCAAA AGGAGCCTGT CTNNNTGNNG GANTNNTNCN NNN---NTNN NNANNCCNAC 1894
Gasterosteus_aculeatus        ANNCTTCCAA A----TCCAC AAACCNNNAA ANAGATGNTN NNATTTTTGG GANTGANTGN NT-CTGTAGT 1550
Felis_silvestris              GTACTTCCAC T----ATTAA CAAGCAGGAT ATCTGCGGTT AGACAGTCGG GACCCCGGGA GAGCTGTA-C 142
Rattus                        NAAACCAGAA AAATATAGTC ACAAATATCT CCTAGTGCTT GTAGAAAC-- --CTTTTCAG GT--AGTANA 84
Papio_hamadryas               AAAACCTCAC TATGCTGGGT ATAAGTACTT ACTAGTGTTT GTAGATAC-- --CTTTTCAG GATGGGTAGA 4853
Pan_troglodytes               AAAGCCGAA- ------GGGT ATACTTATGT -------TTT GTAGATAC-- ---TTTTCAG GT---GGAGA 2703
Ailuropoda_melanoleuca        AAAAACTGAC TA----GANA ATGGACANAC AACAAGCCCN AGACGAGG-- --CTTTNTAG GA-----ACT 69
Cavia_porcellus               AACATTTAAC TCCTTAAAGT GGNCCCCTAG GCNATGAGAG NAAAGCC--- --AGAAGGAA NGTATCTGCT 165
Ictidomys_tridecemlineatus    TCCCTGTGGG TAACAAAACT G--CCCAGAA TCTATGTTCA ACTCNCC--- --C-ATGTCT GGTGNTCAAT 137
Canis                         GACTCCTGGA CAAGCAGAGC NNGNCTNNAC TCTGTANTCG NGCTGCA--- --CCCGGCNT CTNCNGCNNC 98
Felis_catus                   ANT--CTNGN CTNAGAGAGT CTCTAGAAAA CATAGTTAAA TTTCTCC--- ---GCTN--- CAATAAGAAA 70
Ochotona_princeps             GCTAGCTTGG CNGGAGGAGT NTGTGAGTGC TCCAGNCCTA NCCACCT--- ---ACGTGAC CNGTTGCAAG 102
Cercopithecidae               TCTGCCCCCG CNCTCGNACT CCCNCACNCC NCNAAGCCCT TNCANCTA-- --TNCNGGGA NGAGCGCAAG 1025
Carnivora                     AAAAGGA--- ------AATG TAAATAAAAT TAA--ATATC CTTATAAC-- --CTGCAGCC C---ATTGAC 72
Mus                           GGATACN--- ------TGNN TNANCNACGT TAGCGGNACC NNNNTAAC-- --CACTNACT N---AAGAAA 1067
Mus_musculus                  GAAAAAC--- -----CAACC CCCCCTCAGG C---TGACTA TCCCAGA--- ----ATAAAC A--CTCCGTA 103
Rattus_norvegicus             GAAAGAC--- -----TAGCA CCAN-TGGAC C---NGGACN CCCCNAA--- ----GAAAAC A--CCAGGTA 60
Tursiops_truncatus            TGNTAACCTA -----NAGTA CGCCCTNNTC T---CGGCCT CTTCTAG--- ----AGAATC AACCCTTGCG 158
Procavia_capensis             GAAANAT--- -----GAGNG CAATCGCGAG ----TGACTT CCTAANA--- ----NGNAAC A---TANTTN 76
Primates                      GAAAGACAAN AAAAATGGCA CGGCTAAAAG CTACTGAACG TCGGNAAG-- --CTACAGTT AGACTGGCCG 231
Echinops_telfairi             CA-------- -GGCAGGCCC NAGCTTCGGG TTTCTAAGCA GGGCACAC-- --TGGANNTT NTCNGGGGAA 98
Sorex_araneus                 NATAAAAATT AGCTAGGCCT AAGTTTCGGT TNCCAAACCT GAGCAGAC-- ---AGACTNC CGCAACNANG 111
Canidae                       AAACAGAGAT AANACAGTAA AAACAGAAAA AATAAACCTG NCTNCCNA-- --GCTCCAAN NTGTTAG--N 152
Otolemur_garnettii            ---------- ---------- ---------- GCCTCCACAG TCTGATTA-- --GATCCATA TGGCT----C 32
Gallus                        AGAGTGTGNT NGANCAGATG NGATNNCCAG NANNTNTGNA CAGACCAG-- --GGNTNGAA TAACCAGGTG 168
Callithrix_jacchus            GGACAGGGAA GACCANAGAA CACNTCTCCC TCCANCCNNT NGCCNC---- ---GGGAGNA GGGNGTTCAG 139
Crocodylus_porosus            TNATNACAGA GGCCCGGATN CAATTNA--- -CTGGACCCT TGANA----- ---GGGANGA GGCCTTTAAC 138
Erinaceus_europaeus           -------GNC AATNCCAAGT CATTTATTGC CTGACCNGNA NCCTN----- -----GTGCC CANTGNGGAA 113
rnd-6_family-7426-LTR-ERV1    ATCAGCGGGC AATCCTAGAG CNTTGAGGAG GTTGAGGACA GCNTGT---- ---GAGAGGG CNAAGAGGAC 623
Esox_lucius                   TTAAATCTGC -TGAGTGAAT GTAGGAGGGG ATCTGGTATT TG-------- --TCAGTGGA TGAAGTNAAT 118
Salmo_salar                   GGANACATGT -TAAGTANTT GGGNCAAGNN ATTATACANN AAGGACGAAA GCTTNNTNNA GAGAATAAGC 235
Didelphidae                   GGCTTCTGAC CTTTGGNGCT GANNGAGNNA GNCTTAAAGN NAANATCTTC CCTTGGCTCC TGNACTATTA 195
Latimeria_chalumnae           GGAAGCCTGA AANAATAGAA CTATAAAAAA CAGGNTTGTT AAGNTA---- --TGTATATA TATTGGTATT 1098
Monodelphis_domestica         ---AGACTGG AATTG----A CTATTAATCC ATCTTCTCTT TATGAA---- --CAAATAAA TAGAATGGAC 115
Macropus_eugenii              --AGGCCCAA GNTTCCATTC CCCAGAANTC CTGTGANTTC NANGAA---- --GTTCTGAA GNNACTCAAA 126
Alligator_mississippiensis    TTCCCCNGC- ----AGANAG -CANAGCC-- --TACTGTGG TAAAGAAA-- --NTTCTTAA TGAATTTATT 127
Chrysemys_picta_bellii        TTNCCATGN- ----CGGAAG -GCCANTGCA AGNACGGTTG NAATTCAN-- --NTGCTCAA GGACATNATG 2333
Danio_rerio                   TAAATTATA- ----TAAAAT -TAAATTATG GATGACTTTA AAATCAGA-- --GCATGAAN TGAGAATGAT 254
Taeniopygia                   ATAGAAGAA- ----AAAANA -GGNATTAGG TCTGNCNTGG AACATAAG-- --AAGAACTA TGAACATGAG 154
Dasypus_novemcinctus          TANAATCTA- ----TGAAN- ---ATNCAGA CATGCTCTNA GACTGAAG-- --GTGTGACA TTCTA--GNT 110
LTR_XT                        TGANTTNAA- ----GATCAT -TTNNTTATA TATATTAGAG AAAANAGA-- --ATNCATNT TGTTT--NCT 90
Haplorrhini                   TTCAATAAAC ATTTTAACCC -CAAGGAGCC AAAAGTTCAG ATTCCACC-- --AATGTCAG CCGAGAACCT 7459
Catarrhini                    TTCTATGA-- ----TGAACT -AAATGTGCC AGTACCAGAA ATTCCAGG-- --AA---AAA CAAGAAATTT 6602
Anolis_carolinensis           ---------- ---------- ---------- ---------- ---------- ---------- ---------- 1184
Murine_leukemia_virus         GAC------- ----CGGACA -GGGACTCTG CGTAGGAGCA GTTCCCAA-- --AA--CCCA TCAGGCCCTG 6756
Phascolarctos_cinereus        CTC------- ----TGGACT -CGGGTTATG TATAGGGAAA GTACCTCC-- --TA--CTCA CCAACATCTC 6564
Estrildidae                   TAC------- ------CTCT -GAGCTTAA- --TATAAAGA GTTC------ ---------- --TGAAGNTC 113
Xenopus_Silurana_tropicalis   GAN------- ------AAAT -ATANTCTA- --TAAGATAN ATTCCTTT-- --TT--ANTA NATGTGGATT 391
I_XT                          GTA------- ------NAAT -GNCTTNNAA GATNANAANA GGCCTATA-- --TT--ATCT ACNAANAGNT 7009
Takifugu_rubripes             TNA------- ------AANT -NATGNAAAN ATTTCGAAGN ANTGATGG-- --TT--TGTN CGNGGAGATN 279
Bos_taurus                    ATAANAATC- -------NGA -GACTTT-TC CC---TCNGT TATTTTAC-- --ATATAATC AAANCCCCTT 118
Homo_sapiens                  ATATTAACA- -------TAC -GACTTTATC CC---AATAA TAGCATAA-- --ACAAACCA AAAATATATT 104
Microcebus_murinus            AGANAAAAC- -------TGA -ACACCNNAC AC---TGCTT AAGAAT---- ---CANAAAT ATAT--CGTA 75
Myotis_lucifugus              AAAAGAAAT- -------AGA -GGCNCTATG CC---TTTGC CAGCAG---- ---CAAAGCC AAGTTACNTA 75
Equus_caballus                NTGCTAAAA- -------ACT -TAATCANAA AC---TTCTC CAAAAATA-- --AACCAATT NCTATNNGGA 96
Tupaia_belangeri              CTACTGCCT- -------TTC -AGCTNGATT NC---CNCTN ATGCNATA-- --AACTAAGG GCTATATATA 101
Loxodonta_africana            ATNCGTAGA- -------AAT -CAAT-GTGA CC---CATGT TTGACNTA-- --CCCCAA-- GATGCATGNA 91
Murinae                       CCANNAGCTT T---TCTNTN -NGCNTAATN TCA-TNTCNG NNTNAGNA-- --ANANAATG GANGCNTNGA 116
Canis_lupus_familiaris        NNTCTTTTAA A---ANAANA -TNATNTCAG GCTGGTATAT GAAGGAAG-- --GCGNAATC AGGAAGATCN 112
Sus_scrofa                    AGCCAATCTA G---CCAAAA -GATNAGCGT GCAGTTATAT GCATGCAA-- --CCCCAACT A--------- 85
Choloepus_hoffmanni           AGCAGTTAGG C---TTAAAG -GGCTGAGAT ATGAAAACGG ---AAAGT-- --CAATATAT AGA-----TT 94
Vicugna_pacos                 T---GATTNA N---TTCAGC -TGCTGGCCT CCTAAAATAG CACAAACN-- --CTGTCTCC AGAA---CTG 94
Gavialis_gangeticus           ---ATNTGCA TTNACAT--- --AGCAA-AA ACNNCNGTAA NNTGTATN-- --NNTNTGCC NAAAGGTTTT 160
Mammalia                      GACNTCCAAA ATAGGATTTT GCAGCAAGAN AATAGTNTTG AAAGCCAG-- --GCNTTGTC GAGAGANNAA 211
Passeroidea                   ACCGCTGACA ANAGGGAGTA -NGACCTNAT TTGCAAGCNA ATACACGT-- --GGCTNGNC CGGANATGN- 180
Takifugu                      AAGTCAGTTG ACCCTTCCT- -TGACATGAT TGTTGTTTAG ACAGTTGT-- --AA-TCAGN AGGAGACAG- 176
Pteropus_vampyrus             A-------CT ATCCTNACCC -TAACGTAAC CTTAGCCTCA TTATACAC-- --TCATTATA ATATTCAGG- 101
Ornithorhynchus               ACNCGTTACG AGCTCGCCTT GGTTGGTAAC CCCCCTGTCN CCGTGGAACG CTGCG-GTGN TTTAAACCCC 1656
Oryctolagus_cuniculus         AAAAGCTGCT CACCCACAAC AACC--TAGA CCCTCCTAGA CCACCAGGCT TANCA-GAGT NNCTNAACTT 102
Gallus_gallus                 AACACTCCCC CATTGGC--C AGTCAGGAAC AGATGTAGTG GAATGCAGTA GACTACAAGA ACTAAATAAA 96
Hominidae                     NNTNCTTANT NCNNNCNCNN NNTNGTNCCN NACNNNTNNN CTNNATCNNC AACTCCNNAC NNNNNCNCTN 3825
Metatheria                    CACCCTTTGA TTGGGCGCGC ATGCGTACTN TGGNNGGACG NNNGANGCCN --CGNGNNNN CNTGGNGNCN 146
Rodentia                      GCTAATTCCT CCACCTTTAT TAAAGAATTC CAATATATTA CCCAATCTTA CAACCTCACC TTCCATGACG 1441
Monodelphis                   CGNNNCNNAN TNNANNNGAN NNNNNCTNNG NANNNNNANA NCNNNGCNTN AACNNTNNTT NNTAGACGTA 316
Consensus                     AAAAAATAA- ------AAAT -GACNTAAAC CCTAGTATTA AAACAAA--- --CTATAAAA AGAANATATA
```

Conservation

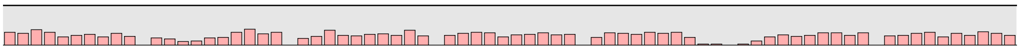

		7,580		7,600		7,620		
Dipodomys_ordii	NC---ACNGT	CANATCANGG	TTAANCCAGN	AACCCTNGTG	CTNGACNGGN	TGNGGAACCC	CANAGACNG-	182
Laurasiatheria	CT---GTAAA	TGCCTCAAGG	TTAAGAAATC	AAAACCTAAG	GACAACCAAT	CACAAACAGC	CAA--CTAG-	149
Eutheria	AC---CTAAC	TCAAAGTATT	CTCGTAACCA	ANAGCTACCT	CTCGAGCCAA	CCACAATCCT	GCGCTTTAA-	180
Tarsius_syrichta	ACATCCTTGC	TGCAAGAATC	CTGCTGACTG	TTTGCAACTC	CTGTAGCCA-	TGAATTCCCT	GTAAATTA--	212
Muridae	NC--CAANGA	NNNNGNNANA	GNNNNGANNN	NNCCNNNGNA	CNGNNGNNAN	NNCNGCNNCT	GNATACCAG-	1961
Gasterosteus_aculeatus	TN---ATTCC	NAACTANTCA	ACT-CANTCA	ACTTCTGTGN	NNNTTGNTAG	ATNAAAATCC	GAAANCNNAN	1616
Felis_silvestris	AG---GTTGC	TACATAAATA	AC--CGCTTT	GCTCTCGCTT	CTGTAACCTT	CTTACGCGCC	CAACCCTA--	205
Rattus	ANCNNTCCTC	ACAAAGCAAG	AGACGGCCTC	NGCAGTCACC	AAGAAGAAAN	TGGAAGAAAT	CTTCCCCAGN	154
Papio_hamadryas	AGCCTTTCCC	ACCCGGCAAG	AAACGGCACA	CATAGTAGCC	AAGAAGATCC	TAGAAGAAAT	CTTTCCTAGA	4923
Pan_troglodytes	AGC--TTCCC	ACC-----AG	AAACTGC-CA	TGTAG----C	AAGAAAAT-T	TGGAAGAA--	-TTTCCCAGG	2757
Ailuropoda_melanoleuca	ACCANNNTCC	ANCTAATCAC	AAATCATTCT	NGAGG----N	ATGAAATTNC	CANACATCAC	AACCGTTAGC	135
Cavia_porcellus	GGCNANCNNA	TAANGGTTCT	GTATTATGTC	AGGATC--AA	GCTNAG--CA	GAAANNAGGT	ACCNTACAAA	231
Ictidomys_tridecemlineatus	GACCACATAA	GAACC-TTCT	TAGTTAAGCA	CGTCGC--CT	CCCAGGGCCC	TAAACAAATC	ACTTTGAATG	204
Canis	GGCNACNNNA	CCTGCTTANN	NNTTAANGTC	CTAGCC--CG	CCCCAGANAA	CCANCAGAAC	ACACNCNAAG	166
Felis_catus	GCTACCTNCA	CTGANA--CT	CACACNCCAC	CTACNC--CT	GGGTGTGNGA	GACCAGGCNT	CN----CAGG	132
Ochotona_princeps	GGCAAGGGNC	NTGACN--CG	NNAAGCAAGC	CCCNGC--TG	CGGANGGCAG	TCCCTGGCGT	NTGAN-CAGA	167
Cercopithecidae	CGGGTNGCCA	ANGGCGTCCT	AGCNCAGCAC	NTAGGN--CC	NTGACAGAAG	NCTGTGGCCT	AACTCTCAAA	1093
Carnivora	AAATAC---T	TGAGACAGGC	AGAGTAT-AA	CATTCC--TC	CAGGAAGCTC	CCAACTGTCT	TAATGTTAAT	136
Mus	AANGGCAGTT	CCTGGACCAC	AGAACACCAG	AAAGCC--TT	TGAANNTNTC	ANANAGGCCC	TACTGNCGGC	1135
Mus_musculus	AAACTAATAT	TCCNCATAAC	CGTTTTGCGA	AAAGTC--CT	CAG--AACCN	GCTCANANAG	TCCTGAGACA	169
Rattus_norvegicus	CCGNNAAGCT	CNACAACCCT	CGAGTAACGA	GATGCC--CN	CAACTGACAT	AACCAAANTA	GTCTGACAAA	128
Tursiops_truncatus	GCNCNCTTNN	TTCCAGGAAC	AAGGTCNCGA	ANCAAA--AA	CTTA-GNGAC	AAAANAGCAA	TTTCAGGACA	225
Procavia_capensis	CCTGTCATTC	NCAATGTGNG	ATNCCACCTT	TACGAC--CC	ATACTTCCCC	AATAATGACC	NGCCGGGGNA	144
Primates	ACCAGCACTN	TCNANG-GTG	CAGTCATNGC	GGAGCC--CG	GCCGGTATAC	AGCTTAGCTT	CAGGGGAAGA	298
Echinops_telfairi	TGNGGCTANC	CACACCCGGG	ACAGGTGAGC	GANACA--NC	TGGACTGGCC	CACCTAGGC-	CAGGACCACT	165
Sorex_araneus	AAANAACATC	TNCNNNGATG	ACATGN-ACC	CACTCA--GG	TAANTTG--C	TANGAGGACA	TNGGTNCAAG	176
Canidae	GAGNATCNCC	CTNTAATNGC	TACTAAGTAA	N----CACAG	AAATCCCAGA	TGTAGAGAAA	TANNATGGAG	218
Otolemur_garnettii	AGACANCTCC	CCGNAAATAA	NCCCCA-CAA	C----CTNAN	CCCTTNCAAA	CAAACAGCTG	CTTTAC----	93
Gallus	GATNAAAGCC	NTGAAGATNN	AGATTTGAGA	CGCAGNNCGN	GAACANCTNG	CAAGAAGGTG	ATTGGCNGTA	238
Callithrix_jacchus	TGNCTAA--A	AGCTCTNTAA	CNANATCCTG	ATCAGGTAAC	CNAATCGAAG	ATGAAGGGCT	CCAGGGTAAN	207
Crocodylus_porosus	TCTTTNA--A	ACCAGCCNTA	TCACANGCGC	AT-GCNTTAT	TANTTCCTGA	TTATTTAGCC	TTC---TAGC	202
Erinaceus_europaeus	ACAATCA-CA	GCNGNTACAG	GTGNNNCNGA	TGCAGNTTTN	ACACGAGAGA	NTAAAGTGTA	GCGAATCAAA	182
rnd-6_family-7426-LTR-ERV1	NCTTTCATCC	ACCGCTCAGA	CNACCGTTGA	AATCGATTCG	TTGTATGAGG	GNATTGACTT	CTACTCNACC	693
Esox_lucius	TATANGTTTA	TNCNGAAACC	ATAAAATCTG	TTATAAATNA	TCAGTGTACA	CTG--TAGAT	-TGTCCAGG-	184
Salmo_salar	AAGCTATTCA	AACTGCAANG	AAANCNNTTA	CTATGAAAGA	ANTGNTNTCN	TTGATTGGTA	-TGGCAAGTT	304
Didelphidae	AGCATGNTTA	CGTAGCTAAA	NGTGNAGGTT	ATCTTAGNAN	TAAGGNTTAG	CTGAGAAGAA	-GNTTTATCT	264
Latimeria_chalumnae	AATTNANANA	AAGGAATGCN	ANNANTCCTT	ATGAGCAAAA	GNNTNATTGC	CAATNNAAAA	--GCATANCT	1166
Monodelphis_domestica	AGTGAATAAT	AAGT--TANA	AGGAGTCACT	GTTACATTGA	GATNTATCTA	CTATCTATCT	CTGCTGAAGT	183
Macropus_eugenii	NCTTCCCTGG	AATGNCCGCT	AAGTTGCCAA	ACAAGAATTC	NGCACTCCCT	NGNTNGANTN	CAACNCATCA	196
Alligator_mississippiensis	CCCAGATTTG	GNATGCCACA	NACNATAGAC	--ATTGATNG	AGGAACACAT	TTTACAGGAA	AAATAGTNCA	195
Chrysemys_picta_bellii	CCNCNCNNCG	GANTCCCCNN	GTGAATNTAA	--ACTGACCN	CGGAACACAC	TTCACANATC	AAGTTGTTCA	2401
Danio_rerio	TTTAAAAAAG	ATATAT-AAA	TGTGTGATAA	CAATTGAATA	AAAATGTCTT	ANTAAAAATG	AATTTCNGCT	323
Taeniopygia	TTCAACTATA	A-GTAG-ATA	GGCCNCAAGG	TAATTGAAGA	GNAA------	GGNAGATCTG	AAT-------	209
Dasypus_novemcinctus	TTCACCCAGA	AGGCAT-TTG	GGCGATATAA	-ATGTAAACT	TCANCTAATC	TGGGAAGCNG	CAG-------	171
LTR_XT	TTTATCTATC	TTNTNTCATA	TNTTTTAATA	TNGNAGNANT	TNAAATATGT	TANAGAATCC	AGNGCTA---	157
Haplorrhini	ATTCGCTCAG	CTAGCTGAAA	GTATTGCTAC	TAATCTTGGA	GTCACCTCAT	GTTATGTATG	TGGAGGTACC	7529
Catarrhini	GTTTTTGCAA	TTAGCCGAGC	GTGTAGCCCA	GTCTCTCAAT	GTCACTTCAT	GTTATGTATG	TGGAGGAACT	6672
Anolis_carolinensis	----------	----------	----------	----------	----------	----------	----------	1184
Murine_leukemia_virus	TGTAATACCA	C----CCAGA	AGACGAGCGA	CGGGTCCT--	ACTATCTGGC	CGCTCCCGCC	GGGACCATCT	6820
Phascolarctos_cinereus	TGCAACCTGA	CTATACCCTT	AAACGCCTCA	CATACTCACA	AGTACCTCCT	CCCCTCCAAC	CGCAGCTGGT	6634
Estrildidae	TCTNCCCAGA	TNAGGTAAAC	AAAA------	CAATTCTNCC	TGGAGACCAA	AGGACAAAAA	AAACAANCA-	176
Xenopus_Silurana_tropicalis	GATTTCANAT	NATNNCNAAT	ANAN------	AGACTTGTAN	AGNATATAAG	GAATCATATA	ANCCAATTAA	455
I_XT	NCANNTGGAG	TTATACANTC	AGANGTCAGA	ATANTTGTNC	CTAAATTGNG	GACTCANACT	GACCCANTTN	7079
Takifugu_rubripes	GC-ANANGNG	TANTACAAGG	ACAN------	GNGGTTGTCN	AATTNATCAA	GGCANTGCCT	GTTTNAAAAA	342
Bos_taurus	TTGCCAAATG	GCAGA-----	--NGCAGGAC	CCCCTAATGA	TCAAAATTGG	AAACNA--AA	AGAATAANAA	179
Homo_sapiens	CCCTTANACA	TTACT-----	--ATGANCAT	AGCTCTAC-A	TACAATTCTC	AAATCA--AA	AAACTAAAAA	164
Microcebus_murinus	GCT-------	CNCNG-----	--ACTCA-AG	TGGACTGACN	GGTACAACAC	CA---T--NG	CAANACATGG	125
Myotis_lucifugus	ACAATAATGG	CTAGG-----	--ACTAAGAA	TTCATTAAAG	CNCACCACCC	AAAACT--TA	CAAATCCAAA	136
Equus_caballus	--GAGAAAAG	NCTTT-----	---ATANGAN	TAANCT-TGA	TCAACAAACT	CCACAA---C	CACATACTAA	152
Tupaia_belangeri	TTNAGAAAAG	TCCANTACCC	TGAACAAAAG	CAGGCCATGA	GTAACTGAAT	ACATNA--TN	NATTTCCTGA	169
Loxodonta_africana	CTGNNGACAG	CCCCT-----	-ACNGGTAAT	CAAACC-NGA	NGCNCATGCT	AGATTA--NG	CATNCGCCAC	152
Murinae	TGGCCNANAG	NANAT---CG	AAACNTATTT	ACCNNNANAA	NANTGACTCC	ACNNTC--NN	GACNTACAAC	181
Canis_lupus_familiaris	TCNGCTGAAA	GNACTAAAAC	NNCNTGAGCT	TTTTACTAAA	NACCTNCTCC	ACAGGN--GA	AACCTGAGTT	180
Sus_scrofa	-CNGCAAAGG	AGCTTANAGA	GACATGAG--	--------GA	ACCCCCGAAC	ATTGCC--GA	ATCTGTTAAC	142
Choloepus_hoffmanni	ACTCCTAGTC	ACCTCCTAAT	---CTGATAT	T-------AA	AG-ACTAGCC	AAAGAT--GA	AATANCTGA-	150
Vicugna_pacos	ACNCCAAGCT	GATTTTAAAG	GNATTAAAAC	T-------GG	GGCGTTCTCC	ACAGNCTGAA	AATTACNGGC	157
Gavialis_gangeticus	TNTGANCCAN	TCANCATTTN	TTTTAAAAAA	TNAANAAGAG	AATNAGATGC	NGTCCANATA	AAACGT-GAT	229
Mammalia	GGANTTTCTA	AANNCTTTAG	CTCNCTTTAG	CATATCA-AG	AGCCAGGCCT	GGNNGACNNA	GNNCNTAGAG	280
Passeroidea	GTCGTTTCNC	GAGNTGATAC	GAGGAANNCN	CAGGNGATGA	TCANCNCGNT	AACTACCCAN	GACTG--AGA	248
Takifugu	TCAGN--CTG	GAAGTGTTAA	ACNCCCATCG	TAAAACGNGA	CAGCATAGTT	TACTGGTGTT	GATAA--GTT	242
Pteropus_vampyrus	CCTGTGCCTG	AAGTTCANGA	ATCCTAATTA	TGACTAACGA	ATTNAGGATC	AGCCCACGCA	CACTA--GA-	168
Ornithorhynchus	GCCNCNCTNC	TGGGCCCTGG	GNCGGTAGAT	CCGNNGGTTN	NGGCTGA--G	GNACCACATG	A-TTGTGTTC	1723
Oryctolagus_cuniculus	AGCTTAGGAA	TAG--CNTGG	GA-GGGATGT	TNCTCTATTT	TCACTCA--G	CAANCCGTTN	A-TTCCTGNA	166
Gallus_gallus	GTCACACCAC	CGATCCATGC	AGCNGTACCC	AACATCGCNT	CN-CTCA--N	NGGTANGTTA	AGTNGANAAN	163
Hominidae	CGNNCNNNGA	NNNNNNCTAA	TCCTGNNNGN	ACTCTGNNNG	NNGANGGCNN	CNNANNCCNN	ANCCNNNNCN	3895
Metatheria	NGNCAAGNNG	CNTGNCNCNT	GNCCNCNGNN	ANNCNNNNNG	NCCGGAANAC	NGGNCTNNNA	NNCGNACNNA	216
Rodentia	TTTATATGAT	CCTCACCAAC	AATCTTCTCC	CTGAGGAGCG	CAGGCGAGTC	TGGGANCAGG	CTAGGGCACA	1511
Monodelphis	ANNTTNNNNG	NAGNCNCNGG	AAGTNNCNNN	NANNNNAGNN	TCTNNNN-AN	NNNNNTTNNA	NCNTNNNGAG	385
Consensus	ACTATAATAA	TAATACAAAG	AAACTAACAA	NAAACCA-CA	CAGAAGACAC	AAAAAAGATA	AACTGCCAAA	
Conservation (100% / 0%)								

	7,640		7,660		7,680		7,700	
Dipodomys_ordii	-GATACANGN	GN----TGNC	CNTGCTNGCC	AATGANCAGT	CAATTAAACT	GNGTCTTTNC	CACCCACTAC	247
Laurasiatheria	-GCTTTCCCA	AA----TAAG	GCAACTGCTT	AAGCTATAGC	CAATCAAATA	ATTTCCTTGC	T--TTGCTTC	212
Eutheria	-C-CAATCAC	AA----NCAG	CCAACT---G	CTCCATCACT	NTTCAAGAAA	TTTCCTCTTN	ACATGTCCAA	241
Tarsius_syrichta	---TATGCTC	TN----TTAA	CCGA-----A	ATCTGATAGT	TTNCCAAGTA	ATTCNTTCCC	AC-TTTNTAN	269
Muridae	-AGNNTTCGG	AA----ATNN	CNNGNCCNGT	NTNCANNNNC	ANNNNNNAGG	GCNCTNANNC	CNNAANNTGG	2026
Gasterosteus_aculeatus	GGCNNATCTC	AAGGTCCCTG	CANNNTNCNA	AGCTTTCANN	GACTNAAGGA	NGCTTTGNCC	CATGCAGCGA	1686
Felis_silvestris	-GCCGTTCGC	AA-----CCC	CGGGCTCCAA	G--TTGCATC	AGCCAAAGGA	AACTTCATTT	----------	257
Rattus	TTTGGAGTGC	CCAAGGTAAT	CGA---AGAT	AAGGGCCCTA	GCTTCGTCGC	CCAGGTAAGC	CAGGGTGTGG	221
Papio_hamadryas	TTTGGACTTC	CCAAGGTAAT	TGGGTCAGAC	AACGGGCCGG	CCTTCGTTTC	CCAGGTAAGT	CAGGGGCTAG	4993
Pan_troglodytes	TTGG----TC	CCCAGAT---	-GGGTCAGAT	AATGGGCCG-	-CTTCGT--C	CTAGGTAAGT	CAGG---TTG	2812
Ailuropoda_melanoleuca	CAGAAGNAGT	ANAAANTCCC	TTNANCAGAC	ACATTGNAT-	-CNTTAGATA	CCGCTTAGAC	CTGAAGGAAA	203
Cavia_porcellus	NGNGTCGGG-	CACNCCAA--	-AGACNGNNN	AAGACCT--C	CGATCC-CCC	TGGTTACCCA	ACNNNTGA-C	293
Ictidomys_tridecemlineatus	TGTAACCCT-	CCTTGNAN--	-TGACTAATC	ACNGTNTGCC	CAACTTGTTC	CCGCCATGAA	TGTGCTAATC	270
Canis	CNGAGATCG-	GNNAGCTG--	-AAATGGCAT	TANGTCT--C	TAANTA----	CAGTGAGAGG	NCNGCTCA-A	225
Felis_catus	AACNCATTC-	CNGGACAA--	-AGCCGAANA	NGGGCNCCAA	CGTTTCCN-A	GNCTTATGGC	TA--AACANA	195
Ochotona_princeps	AACATAGTC-	CCCNA-----	--GTCACGAG	CANTATATGT	CGTCTTTATA	GTNTGNTGGC	AT--AGGACA	227
Cercopithecidae	AAAGCTAGA-	NCCAAT----	-GGNTGANGG	CTGGCCCCCT	TGTCTNCGNA	TNATCGNGGC	CACCNCNNTA	1157
Carnivora	----------	GCCTTGCT--	-AGAGGGAAA	AACAACCTTA	GCTTGACAA-	-TAGCNAGGC	CTCCAGNATC	191
Mus	CCAGGCNTTG	GCCCTGCC--	-AGACCTAAN	TAAANCTTTC	ACCTTATACG	TTGNTGAGAN	CTGNNGTNGC	1202
Mus_musculus	TCCCTAGCT-	-GGATGTA--	-ATACTTGAT	GACCTNTNAT	CATTCTCN--	-TG--ACGTT	TGAATAGACC	229
Rattus_norvegicus	GCGTTCCC--	---ATGCC--	-ACAGN-GGC	CTCCTTTCAA	GACCCAAA--	-TA--ATCCT	CATTGAGATC	184
Tursiops_truncatus	GCNGGGCCC-	-AAGAACC--	-CAGCAACCT	GACNCCNTGT	AACCNNAT--	-NA--ACAAC	CAANAGNAAC	285
Procavia_capensis	CCCCAANTC-	-TCAGGCT--	--CATCCCGT	NACCTGTTAC	TACCTANA--	-GACCATACC	CCCNCGCNAT	205
Primates	TCACCCTNC-	-CAACACC--	-ATCCCCTCT	TCCAGNTCCC	CATCCACCC-	-TGCGANACC	CACTTCCACC	361
Echinops_telfairi	CAANTAANC-	---CAATA--	-GNGTCAAAC	AGNAGCCGTC	GACCACNCTC	CCTGCNAACC	CCGGAAAATC	228
Sorex_araneus	CAATCATTC-	---TCAAA--	-GCNCCTAAG	CAATANCTCT	GTTCCCANT-	--TGAAAACN	CCCAGANGCC	236
Canidae	GAGANNNTAA	CCAGAGAGAA	GGGAANACNT	TGTTTGGCTC	ATGATATTTA	CAANGAACAT	CTTGTTTGTT	288
Otolemur_garnettii	AATAACNTAA	TCACAGCACA	CCAGCNATTN	CCTCCGGTCA	AGAATNCAAC	CACCCCCCAC	CTGAGCCAAT	163
Gallus	NNCAGGGTAA	TNTAAAACNG	NNNCGGGGNG	TNCCCNACNN	TCNGGANCAG	NAGTTCTTGC	AGNNAGGAAG	308
Callithrix_jacchus	NTTTACANAA	ACTCCTCCCA	GTGGTC-NAC	ANTAANAATG	GTAAGTNAAT	TTACTNAGGG	GGCTTTAAAC	276
Crocodylus_porosus	CTTGCTATAC	ATNAGAAGCA	AGGANT-AGC	CTCGGGAGTT	ATNCAGTACT	TNGGACCCAT	GGCGTGCAGT	271
Erinaceus_europaeus	AGCACGCCGC	GCAAANCTTG	ANGAGT---G	NATAANCNNC	TTTCNGAACC	CGGCAG-GCT	GGCT-AGAGT	247
rnd-6_family-7426-LTR-ERV1	ATCACNC-GT	GCNAGATTCG	AAGAGTTGAA	CATGGATCTG	TTTAGGAAGT	GTATGGANCC	AGTTGAGAAG	762
Esox_lucius	---AGNTGTN	AGTN-TCCTN	AAGGGTTGAN	AAGCNNACTC	AGTCGTGATG	TGTGTGAAAA	CANTNT-GTC	249
Salmo_salar	AC-AGTAGTG	CGTGATTCAT	NGGCTTTGAA	GAACNTATTT	ACCCACTTCN	TGAGCTTATA	TATGGTAGNA	373
Didelphidae	CTTTGGTCCT	GGTGGCCCGC	CAG----AAA	NAACNGACNT	AANTTCCTTC	TGTGGGTATA	GNCTAGNGAN	330
Latimeria_chalumnae	TTTTNCCTAN	GTTAAATAAT	T------ANT	GNCNNANCNT	ATTACACTGT	NTAGAACACN	NNNGGTTGAC	1230
Monodelphis_domestica	TTTTCCTGTA	ATTGAATTGT	TGN----ACT	GTGATTTGTT	TTTCCTGAGA	CAGGGGTAAC	CCTAATAGAT	249
Macropus_eugenii	NCATTCACGT	NGGANTTTNN	CCN----GGT	AATGGGTNTT	AAAATNGGAC	CTGGTTCNCA	NCTGAGAGGG	262
Alligator_mississippiensis	AGAAGTATNA	AAANCTCTCA	ACATAAAATA	AAAATTCCAT	ACCCCGTATC	ACCCANAAAG	CACTGGACAA	265
Chrysemys_picta_bellii	AAAGNTATCA	GGTGNCTTGC	ANATCCCACA	GANGCTGCAC	NGCCCATNCC	AACCACAGGC	CNNTGGAGTA	2471
Danio_rerio	NTGTATGGCT	AAATNAAANT	ATTGAAGACT	GAGAATGCAG	TAAAGCAATN	ATCCTATAAT	TATT-ACTTA	392
Taeniopygia	ATGCATGGCT	GGATG-----	--TGAAAACN	TTTNAAGTAN	AAAAACCA-G	GTCACTGAAG	CGAC-ACCCA	270
Dasypus_novemcinctus	ATNTNNCAAT	GGTTA-----	--TTCAAGCT	TACCTGGAAT	GTGGGGAATA	TGCTAACAAG	C-TT-ACCTG	232
LTR_XT	TTNNANGTNT	ATATGTTGCT	TTTGCCTAAT	GNAANGNTNN	GCNANNNA-A	GTCTGGAAAA	-----ATATA	221
Haplorrhini	AGTATAGGTG	ACCAATGGCC	CTGGGAGGCT	AGAGAACTAA	TGCC-ACAAG	ATAACTTTAC	CATACCGGAA	7598
Catarrhini	GTAATGGGAG	ATCAATGGCC	ATGGGAAGCC	CGAGAATTAG	TACCTACAGA	CCCAGTTCCT	GAT----GAA	6738
Anolis_carolinensis	----------	----------	----------	----------	----------	----------	----------	1184
Murine_leukemia_virus	GGGCTTGCAA	CACCGGGCTC	ACTCCCTGCC	TATCTACTAC	TGTACTCA-A	CCTCACCACC	GATTACTGTG	6889
Phascolarctos_cinereus	GGGCTTGTAA	TTCTGGCCTC	ACCCCCTGCC	TCTCTACATC	AGTCTTCA-A	CCAGTCTAAC	GATTTCTGTA	6703
Estrildidae	-TCAGACCCG	G--AA----A	AGTACAAACA	GGAGGAATAA	GGGGAGCG--	--AN---AAA	ACATGGAAGA	232
Xenopus_Silurana_tropicalis	GCCCAACACA	A--ACCCTTG	NGNTTNCATN	GAAATAATAG	GANACACG--	--ATTNANAA	ATGTTNAAAG	519
I_XT	ANCCCTNCAG	A--ANCCTTT	NTCATNCATG	GNAATAATA-	GGNACNTG--	--GNTACNAN	ANGTTTAAGG	7142
Takifugu_rubripes	AACNNGGCAA	AGTACACTTA	GCNTCTGATA	AGAATAGTAA	ANCATACATA	ACGGTGGAAA	ACGAGTAAAA	412
Bos_taurus	CAGTGCNCCC	AAAANCTGCA	AACC---GNC	ANCCTCCCAG	---GANC--A	AAAGAAC-CA	TCAAGCTNG-	239
Homo_sapiens	CAAT-TCCCN	AAGCTCAATA	TCCCTCAAAA	AACCTGGCAA	---AAACCTA	AATTCCC-CA	CCAACCAAA-	228
Microcebus_murinus	CCAAACTACA	AGGTTAAACA	CT-AACCCGG	GTCNTNTAAC	---TATTTGA	AAATCANAGA	ATCTTTNAA-	190
Myotis_lucifugus	GNTCACCACA	ACGNTAAACA	TTTGATTAAG	GTCCTAACNT	---GAGCCCT	--CCCACANA	CCNGCTCAAC	201
Equus_caballus	-------TTA	TACCTGTAAT	GA-------T	ANCCATAATN	---AAAGACA	-CAC-GCCCT	TNGCAATAAC	203
Tupaia_belangeri	ATCAACCCTA	AGCCTGTGAG	AAACTTTCTC	NCCAACAACA	---AAAAAAA	GCACTGCTTT	TAATCTTACC	236
Loxodonta_africana	----------	ACCCTCCAAT	AAGACN--TA	ACCNNCATTT	---NCAGAAG	ACGTGAC--G	TAGCCATATT	205
Murinae	NTATCTGACN	AAATTNCNTG	TAANACTANN	ACCTTTNCCT	---NAATGTT	GNGTCANTCA	TANNTGCTA-	247
Canis_lupus_familiaris	GTCNTAAAAC	CANCTCTACT	TCATTATAAA	ACTAANATTG	CCTCCTAANT	AGGTGAGAAT	NGAACTAATG	250
Sus_scrofa	CCCCTTNCGC	AAGTTTCATC	CCAT-ATGTG	A--AAGGCCC	GAAANTGNGG	GCACCAGGAT	CGNACAGGT-	208
Choloepus_hoffmanni	-AATTATNNA	AAAN--CACT	GNAC--TNTA	ATCCAGCTGC	---CATA-AA	CTATAAAAAT	AATNGAAAAA	211
Vicugna_pacos	TGATCACCAT	AGAT--AACT	GTCTGACTTA	ATCNAGNTAC	T--CATNTGA	ACATAAGAAT	CCANAAAATA	223
Gavialis_gangeticus	NCTGNGCTA-	GTTCAATANN	TAAATGATTT	GCNTGNAANA	TCTACTACNG	TCCGACTNTG	TAANGCTGAT	298
Mammalia	TTNATACCAT	ATCTAGGNAN	GAGNTNNTTC	GGATATGNCG	TNAGGNAAGN	CCCTGCCAAN	AGNGGAAGAG	350
Passeroidea	TCGCTGGAAC	NACCANGCNG	-ANGCATCNN	AATGCCGAGT	TCCGGTAACT	CGATNAGNGN	NATACATCGC	317
Takifugu	CCTCTGCAAG	AAGAATGTGA	-AC----TTT	GACCTGGCCA	TCTGGGAAGA	CAACGGAGAA	CAAGGACTGT	307
Pteropus_vampyrus	-----AAAAG	ANNTATNTCC	-AC-------	---AGAGCCA	ATAACAATCA	CAATGANNAC	ACTTGGCNAT	222
Ornithorhynchus	AGGTTGCTCA	AACCGTGGCN	ANGGCCCGGT	CCNATCTNAC	GGNCGNTCCT	CTCGAGANCC	CTGACGATNN	1793
Oryctolagus_cuniculus	NTATAGCCAA	GTTTCTCATC	GCAGCTCGAG	G-TATATCNT	TAACATNCCA	AATAGTGTAA	ATGANGA-CT	234
Gallus_gallus	TAGNAACATA	NCACTGCGTN	NTGGNTGNNG	CNNATGTATT	TTTCANTGNT	CC---NATTG	CTAAGGANTN	230
Hominidae	NNNNNNNNCN	GGCNNNNNAN	GNNNTNNNNN	CCCNNGANNA	NNCNGNNGAG	GNNG-CANCN	NNANNCCTTN	3964
Metatheria	NGNCNANTNN	NNCNNNCCAN	GCATATGANA	CCCNNANNNN	GNNANGTNAN	NNNGGTNATA	ANTTANNNNA	286
Rodentia	TGC-AGACGA	AATCCACCAA	ACTAACGCTG	CCCACCCAGT	CGGGGCCGAG	GCGGTCCCCG	ACCAGGACCC	1580
Monodelphis	TGN-AGTTNG	AANACTNNNN	NTTNTNCNTT	CTTNNNANTG	NANNNTNNAN	NAGAANNNNC	NNNCTNNNCC	454
Consensus	ATCTAACCAA	AACAAACA--	-AGACCAAAT	AACATGCCAT	TATCCANATA	CNATGAAAAC	CATTGNAAAN	

Conservation 100% 0%

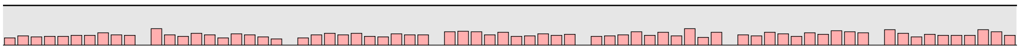

			7,720		7,740		7,760	
Dipodomys_ordii	TGTGCAAGCC	TGTCAATTTT	NNCCGCNGCA	ACNCCNTNCC	NTAAACGANT	CGCTTCAACC	GNTAGATTCN	317
Laurasiatheria	TGCCTCTTCT	CTATAAAAGT	CTTTCCCCTA	GCTCCTGTCG	GTGGAGCGCT	CCTAACCACT	TCCTGGTTTG	282
Eutheria	ATAAGGCAAC	T-GCCTCAGN	TNTAACCA--	-ATCAGGTGA	TTGCTT-TAT	CTTACTTCCA	CCTTCTCTTT	306
Tarsius_syrichta	TNAGTGCAAN	TTAANGTAAG	CCTTGTAC--	-CTCACCTCA	CAACTA-TGT	CGCCTACTTA	GCTG---TTT	332
Muridae	NNNGNGNNAN	N-ANNNNAGA	NNTTACANAA	GNGTACGTNN	NANNCAGCAN	NNTGTTNNGG	NCANNGANNN	2095
Gasterosteus_aculeatus	NTTTNGCTCT	NCCNGATNAT	AGCGAACCGT	TCAACCTAGN	TGTTGACGAT	NGANCCGGNN	GTNTTANTNN	1756
Felis_silvestris	CCCAAGCTTC	CCCGGGACGA	AATTACCCA-	-CAACC---C	TAACCACCAC	CGAGCAGAGA	GCCTACCACC	322
Rattus	CCT---AGAA	TATAGCGGTT	GATTGAAAAT	NANATTGTAC	TTACAGCCCG	CACAGTTCAG	GACAGGTAGA	288
Papio_hamadryas	CCA---GGAT	ACTGGGGATT	AATTGGAAAT	TGCATTGTGC	TTATAGACCC	CAGAGCTCAG	GACAGGTAGA	5060
Pan_troglodytes	CTC---A---	TCTGGG----	AATTGGAAAC	TACATTGCGC	T-ACAGGCCC	CAGAGTTCAG	G-CAGGTAGA	2870
Ailuropoda_melanoleuca	CTAT-AAANC	ANCANNGTNC	CAAACNAACC	TACCTTATCC	GAAAACATCN	TNTCTANCAN	ATTGCCTANA	272
Cavia_porcellus	CTNTCCAATC	ANCTNTAGCC	AAGTNC--TG	CACCGTTCCA	AATTAACCAA	TGTGATNTGC	TT--CTGTAA	359
Ictidomys_tridecemlineatus	NTGTCTGAAA	ATTGTTATTA	GATTTT--CC	CNCGGTNTTT	GGCAAATTNC	TAAGAAATGT	TAAGCTGTAA	338
Canis	CTATGCNACA	GGCTAAACTN	CAATTCAATN	TGCAACNAGA	ACCCGGACCT	NGNTGACTGT	NCTGCTTTCT	295
Felis_catus	GAAGTANAAA	ACA-TATAAA	AAATTCCTCC	CCCTTCC---	GATCGGCCCT	CAGNNCTCTT	GANACNTCAC	261
Ochotona_princeps	ACAACTANAG	ACA-TGCAAA	GAATAATGGC	NCTGTGNACA	GANTGGGCTG	NCATTACCAT	-AAACATGGT	295
Cercopithecidae	CTGGTCNAAG	ACNCTGTTAA	GCTTANCCTC	GGGCAANAAT	TAACGGTCNN	CACNCCACAC	-GCCCTGNNN	1226
Carnivora	TTGTAA--GT	CTTCTTTAGC	ATATGAAANT	CCTTTTGG--	-A----AACC	TCCCTTTTCC	TTACCTCCC-	251
Mus	CAGAAGAGNC	CTTANTCAGA	CTNTGGGACC	NTGNAAAN--	-GCCGTGGCC	TACCTATCCA	ANNNNTTGGA	1269
Mus_musculus	CAA------T	TATTGTAACT	GCGCCCAACT	TTTCCCAC--	-CCCACC---	CCAATCC---	TGAGTACCTA	286
Rattus_norvegicus	CA--------	---TGTAACC	ACACC-AGCT	TCTGAANT--	-CCCGCT-GC	TTAATCCCCC	TNCTCACCCT	238
Tursiops_truncatus	CNG--A--AN	AGCTATCNNA	GTNCTTCACT	GCTATNAT--	-CCCNAT-CA	CCCTNCCANC	TCTAAAANTT	347
Procavia_capensis	TTC--A--CC	CCCCCCCGNT	ATGCCTCACT	CNCCCACTAA	ACACTCN-CC	CCCCCTCNGT	ACTCTGNCTT	270
Primates	CNTCAA--TA	AAANCTCNGC	ATTCCCCATC	CTTNAAGCCN	ATGTGCGACC	TTNTTCCTCC	TGGACACCNG	429
Echinops_telfairi	CAG-----GA	ACTCTN---C	GGAACNACGN	CATACAGCGG	C--GGCCTGG	GTACGCCCCC	GCCTGCGTAA	288
Sorex_araneus	NGC-----AA	TCANANAATN	GTAACTACTT	CNNAAAANTG	CTCAGATGCT	GNATGANCAC	TTTTGCCATA	301
Canidae	ATTANGATAG	TT-NCNAGTC	-C--ACCNTG	TTTGTTCTAA	ATATCCACNC	NGATATTGAC	AAGAAATTTA	354
Otolemur_garnettii	CCAAATCACA	TT-ATTAGTA	AT--ACCCNA	CTTATAAAAA	AACTAACCAC	CAACA-----	AAGACATGTA	225
Gallus	ACTATNAGCC	TTCGCGAGNA	ATGGACAACG	AGNNTTCAAN	NATNGAGGAC	CANGAG----	GAGACCCCNA	374
Callithrix_jacchus	T----CCAGC	TTCTCCTNTN	CAATAGNGNT	TCCNNNTCAG	AG--TNACTT	NNTACTANTN	NANGGAAGCC	340
Crocodylus_porosus	T----GCTTA	CTNTTGTGTT	TNTCTGAACC	AGTTGNTAGA	NGATTGACTT	CCTGTTTCCG	CGCAGGTGCT	337
Erinaceus_europaeus	T----GT--A	ANAGT-CNNC	TAGNTTTCCG	NCGGTGTNAT	AANGTTCNGT	CCTGCCCTCT	NCNCNGN--A	308
rnd-6_family-7426-LTR-ERV1	T----GTTTG	AGGGA-TGCT	AAGGTGGACA	AANGCGGNGT	N-CATGAGGT	CGTTCTTGTT	GGTGGATCGA	826
Esox_lucius	CCNTGGTGTA	NNGNNTAGAA	CCAGT---GA	TAGAACAAAG	GGAATGTGTT	TTATTTGACA	TAGGACAGAT	316
Salmo_salar	NTATGGAAGT	GAATGAAGAT	ATTGTCTGGA	CNGNAGAGCC	AAAAANNGNN	CTGGNGAAAN	TTNAACAAGN	443
Didelphidae	TTCCCGATTT	CACTCTTCGT	CACCT-----	TAGCANACCT	AATATCCATT	TTCAATATCC	GNANANCTAA	395
Latimeria_chalumnae	CGCCC-TTCN	ANNTNNCNTT	NATNTNGCTN	NCNNACAGGN	TATAACCTTT	TGTAANNNNC	TAGTAANATG	1299
Monodelphis_domestica	TTTAAATTTG	AAGCCTTCGT	CACCTTGATN	TTTGA--GGC	TANCTCAGTT	TTTAACTTTA	TAG-AATCTA	316
Macropus_eugenii	------TTAG	GTNTCCCGNN	TAGATTGTAA	GCCT---GCC	TCCCAGCCAA	TGTGAATATN	GNNCAGTGNG	323
Alligator_mississippiensis	GTAGAAAGAA	TGAATGAGTN	TATTAAAAAN	TAAAATAGCA	AAAATGTGTG	ATAAAACTGN	TCTGAANTGG	335
Chrysemys_picta_bellii	GTGGAACGNA	NAAATGNANN	-ACTCAAGCN	NCANCTCTCN	AAGNTCTGTG	ANGANNCATN	TNTAAGGTGG	2540
Danio_rerio	GTA-ATGGAT	AANATAAATA	ANTTTATACT	AAAAACAAGTG	AATTTGAAAN	TGGTCATGTA	TGGAAAAGNG	461
Taeniopygia	GAA-AGAACA	NNCATGAGAA	ACTACGCATT	CTTAAAAGC-	--TNTGGAAC	TATTACAGGA	--GAAANGAA	334
Dasypus_novemcinctus	GAATGTGGGN	GATATGTGCA	ANC---TATN	GAGTNTTGTC	AAAACGTAAC	TGATACTCAG	ACAAAAACTT	299
LTR_XT	AGAAGTATAC	ANGATNNAAA	AGTATATTNG	NTCTACTGCC	TATACNGTTC	TTAAACNNTA	ANACANANAA	291
Haplorrhini	TTTGTTACAA	AGTTCAATGC	AAACCCA-AG	TGTCTGGCTA	TTAAAAACCC	CTATCATTGG	AAGATACTGC	7667
Catarrhini	TTCCCGGCTC	AAAAGAAT-C	ACCCTGATAA	TTTCTGGGTC	CTAAAANCCT	CAATTATTGG	ACAATATTGC	6807
Anolis_carolinensis	----------	----------	----------	----------	----------	----------	----------	1184
Murine_leukemia_virus	TCCTGGTTGA	GCTCTGGCCA	AAGGTGACCT	ACCACTCCCC	TGGTTATGT-	TTATGGCCAG	TTTGAGAGAA	6958
Phascolarctos_cinereus	TCCAGATCCA	GCT-TGTCCC	TCGC--ATCT	ACTATCACCC	AGACGTTACC	TTGCTACAGG	CCTATGAGTC	6770
Estrildidae	TGGACTACAA	-AANCAAAAG	CTGCAATNGG	AGCATGAACC	-CGGCTAAGC	AAATGGAGCA	AAACTTAGAA	300
Xenopus_Silurana_tropicalis	ACTGTTACAG	TANTTTANAA	GTGGAATTGC	ATTAAGCTTT	GTAGTTNATN	TTNTATCATG	ANTAGTACTT	589
I_XT	GTTNTTANAT	-ACTTNACAA	AAGNAATTGC	AANAGGCTGT	---GNTNATN	TTTAACANG	NNNNA-GTNG	7207
Takifugu_rubripes	ATGNCTAAGC	AGAAGGAGGA	GCCCGGGGAG	GGGTAANCCC	AGGCTCCAAA	TTGTATAAAA	GCTGG-ANAA	481
Bos_taurus	-TAATCTTTA	ANTATGACCA	TGTCACGCCT	CCCNTCAACC	ANCATAAANA	TTT-TCNTGA	-NATAACCAC	306
Homo_sapiens	-TCCTATTAA	NACTCCTCCC	TACCATGAAC	TCCTNCTACT	CCTACCATCC	CCC-CTCCCA	-ACCCTCCAG	295
Microcebus_murinus	TCAACACCAA	AAATGTTTAT	GCCCCCACTT	AGCGTAGTCC	GAACCCAAGA	NCN-AAAAGA	-AANAATCCC	258
Myotis_lucifugus	TCNCTAACGA	CAACAGACCC	AACTGGGCCT	ACCCTCTCTN	CCTCCCAACC	GCG-GAAATA	TAACCNTCCA	270
Equus_caballus	A--CCAACCT	GANTACCATA	AACCATAC-A	GTCCCCAACC	CAAAACTCCA	TAC-TTTCAG	CCACCATAA-	268
Tupaia_belangeri	TTCCNANNAT	AAACACTCNA	TNACACCCCN	NTCCCCATTC	CATNTCCTTT	TCC-CCCAGN	CCACTAAAAA	305
Loxodonta_africana	AGATCCCTAT	TGCGCCTGCG	TGCTAATTAA	TNNTGAATCT	CCATGNTGTA	ATC-TGCANG	ANCCCATTAA	274
Murinae	-TCATANCCC	ATGNNCAAGA	TTCTACTNAN	NTCTGCATNN	CTANCTNNCA	TCNATNTNAA	TGGAAANCCC	316
Canis_lupus_familiaris	TATAACACAT	GNCGATGAAT	GNACCAACAT	GTTATTAAAC	TNC--GTATC	CGTAATTNTN	CCAAGCGCCA	318
Sus_scrofa	TGCANGATAT	NNNGGTCAAC	GA-CCAGCAA	GGCCCCAAAC	AAGCAGGACC	NATAAATCAA	ACATGCNTCT	277
Choloepus_hoffmanni	CT-ANT-TAC	CTAGCTCTCT	---CGGAATT	GTTGAA--AC	CTCTGGAATN	CCCCCCNCCT	GTACACAAGT	274
Vicugna_pacos	TG-ACAGCAC	CAAACACCTN	GGCCNTAATC	ACTCANCTAC	TTCGGATACC	GCCCCCTCTC	GANTNTATAT	292
Gavialis_gangeticus	TNGTT-TGAA	CCNCTGNCTN	TCTTGCTTNT	ANGGNNT---	-AAAAGGTAT	CTTNNTCAAA	NGCTGN---A	360
Mammalia	NGGAAGTGAA	AGCCCGCCCC	TTCTGGCCCT	GGGGGATTGT	GGGAAGGAGA	AGGAGNNGGA	GGTAGGGC-A	419
Passeroidea	TTCCC--GNA	CACNGANTTG	TNNAACTCAG	CGCAGAGAAA	AGAGACNNN-	ATGAATATGT	GGNACTCTGA	384
Takifugu	NCC-----AG	CATCCAA---	TGGGACTGGA	AGAAGAAGAC	GAGGTCATCC	AAGAAGAAGG	ACTGGATTGG	369
Pteropus_vampyrus	CTNCC--TAA	GATC-ACCCA	TATAACCCCT	CAGTATGGAC	CAAATTTAT-	----------	AACANATTTA	278
Ornithorhynchus	GNTANTCANN	GATGGCTCCT	CTTTCCTGGA	TGAGGGGAAG	CGGTNCGCCG	GGGCTGCAGT	NGNAACTAAC	1863
Oryctolagus_cuniculus	GCAAGCCAAG	CACTTTGCCC	CGGTTCTG-A	CCCGCCCGCC	TGANTGACTG	ACNAGGTGTA	CCAAAACAGT	303
Gallus_gallus	CCAAGANNNG	NNTACATTCN	TCTGAGGNCA	GCTAGTGGNC	TTTTCANGNN	NNNCNNCAGG	GGTNNGTNCA	300
Hominidae	NGA-CNNNNT	CNCANAANGN	TNANCTCCNN	NCNCNNNCCN	NGGCNNNNNN	CC-TNNNNCN	GGNANNNCNN	4032
Metatheria	TCN-NGGANN	NNNNNNNNCT	TNGTCTGNNN	ANNNNNCNNG	NNNNNCNNNN	CNCNTNCGNT	NNTNNNNNNN	355
Rodentia	TGAATGGAAT	TACAATANCC	CAGGGGGCAT	CCTAGCTAGG	GATCAATTTA	TAACTTGCCT	CCTGGCCGGT	1650
Monodelphis	TNAGNCCCNG	NNNCNNNTNN	NGNNGTNNNT	ANNTNNCNNN	NNTTNNNNTG	NNNCNNGGTG	AGTGAAAAGN	524
Consensus	TTAA-GATAA	AACTTTAACA	AATTACANCT	TCTAACATCC	AAACTGAACC	CTAAATCCGA	GCTAAATTAA	

Conservation 100% 0%

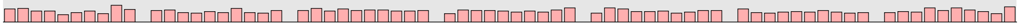

7,780 | 7,800 | 7,820 | 7,840

Dipodomys_ordii	C-GGTCTTCT	CA-AATCANC	CTGATAGNNT	NGTTTTAACA	G-TTTTCATG	GGNTGGATTG	TAACTCATTG	384
Laurasiatheria	G-TG-CTGCC	TG-ATTTGAA	TCGAT--TTT	TGCTCAAATA	AACTCTTAAA	ATTTTTAATA	TGCCTCAGT-	346
Eutheria	C-TGCCTATA	AA-AACTCCC	TGCTCACCTT	GGTCCAGTGG	AGCTCTCNGC	ACCTCTTCTA	GTTNTGAG--	372
Tarsius_syrichta	T-TGCCTATA	AA-AGCTCTC	TG-----CTA	GGTTNATTCG	GGGCTCNAGC	TGCTTCTGGA	GGCGTGAGC-	394
Muridae	N-NNNTNTNA	NNTANNTNTC	CANNNNATTN	TGNNCCNGNN	NNCANNNGAN	GNCNNNCNTG	NCNAANAGN-	2163
Gasterosteus_aculeatus	AGTGCTNNTN	CAGAANNGNA	GAGANN-GAA	NANGNAAATG	NTNGCTTATT	NCNNTGCNAA	ATTCGATCCT	1825
Felis_silvestris	A--GCCTGTA	TGCAAATGTA	---ACT-CAA	AATGGTATAA	AAGACCTGTA	ACCCCGTTTA	--TCGGCTCT	384
Rattus	GCACATAANT	ATATC--TTA	AAGAGNCATT	TACCAAATTA	AATGCTATGG	AGACTGGCTC	ATG-------	349
Papio_hamadryas	GAGAATGAAT	AGAACAATAA	AAGAGACCCT	TACTAAATTG	A---CCTTAG	AGACTGGCTT	AAAAGATTGG	5127
Pan_troglodytes	-AGGATGAAT	GGAC---TAA	AAGAGAC-CT	TAACAAATTG	A-----CTAG	AGACTGGCTA	A---------	2921
Ailuropoda_melanoleuca	GTNCCCTANT	TAATT--TTT	CANAAACTAT	CGCCCATCTN	AGCCCACCGT	NG--CAACNC	A---------	329
Cavia_porcellus	A----CTCGC	TTGCC--TTG	CGGTTTNCAC	CTTTAAAAAC	----CCTGCC	CCGCNTCCCC	TCGGGGCCCT	419
Ictidomys_tridecemlineatus	GACAGCNCCC	CTGCC--TTC	TCCATATANT	GTATTAAAAC	----TCTGCT	CCACCTGCGC	TNGGGGCTCT	402
Canis	G----CGNGT	CCCAT--GGG	CCACCNGCCN	CTATNAAGNN	----GCTGCA	CCTCTTAGCC	TCGGGNTCCA	355
Felis_catus	CANCCCCTCT	GAGCC--CNC	CNGTACTAAA	T--AAAACCT	-----CCNTT	NTACNAACCN	CCNCCGGTGC	322
Ochotona_princeps	GANGTAGTCT	GCAAC--CTA	TGAANTCGGC	C--TGTACGT	A--TGCGCCN	GCGCTAANGG	TCGGTGCTAN	359
Cercopithecidae	GACNTCCTCN	GACAG--CCN	CCTGNCCGAT	GGATGANCCA	C--GCCCGGC	TTACCCACTA	CAANGGCTAT	1292
Carnivora	CCAACTCCAA	AGTAT--ATA	ATCAGCCACT	CCTCACAAC-	----CCCGGT	GCAGCAGCTC	TTTCTGCCCA	314
Mus	CCCNNNTNNN	AGCGG--GTG	GCCCTCNTGC	CTCGANGNC-	----NTCGCC	GCTAN-GCTC	TGCTTGTNNG	1331
Mus_musculus	TAAAAACCCC	TAGNT--CTN	GCAACTCGGG	GTCGAACTC-	----CTCTAC	CCCCTGCGTG	GGATACGAGG	349
Rattus_norvegicus	NTAAAAACTC	TTGTT--TGT	CAAGGTCGGT	GCNTCAGTC-	----TTCTNC	GGGTCGTCTN	TGTCTCGACC	301
Tursiops_truncatus	TCATTTNCCG	TNCTN--CCC	CTTCTATGGA	GTCTCAGAT-	----CTTAGG	GCACNAGCCT	CCTATCTCTT	410
Procavia_capensis	CAAACCCCTC	TAGTC--CTA	CCNGCGTAAG	CAGGACAGT-	----CTTCGA	GAGCTGGTAT	CCNCCCTGTC	333
Primates	TCAANANCCC	GGGCT--TCA	AAAAGCCNTN	ATTTAGNTCT	AAAGCTGGNT	GGNCTNGANA	CCCCTCTNCG	497
Echinops_telfairi	GCCNTGTNTA	TA-AA--CTA	CTNGGCTTNT	NCCNTAGTC-	-----NTGGG	GCGCNGGCNC	AGGAGGGTGC	349
Sorex_araneus	GATGCCCGTA	CGTAA--CTC	TAAAACCTAT	ATAAACNTC-	-----TCGTG	ATTTGGAGNC	GGGGCCCTNN	363
Canidae	CCAAGTTCCC	TGGTNNGNTT	CCTATAAAAG	ACNG--ACCA	TA----NAAG	CCCTCAGTGG	CAACCCTGTC	418
Otolemur_garnettii	CC-----CCT	AGACAGACCC	CCCACGCGGG	NTCTCTACCC	TA----TAAA	ACCCTTCCTC	CCCCTCTGCN	286
Gallus	NTGCNC-NTG	ANGCNTGCAA	GNGGTNGAGG	GACTGTATGC	TAA---NAAG	NTCTCAGNAA	ATGTAATNAA	440
Callithrix_jacchus	CTCTCTGTCT	GNANTTGCNC	GGANTNCCTA	ANAAACCGGC	TT-----TA-	---CCGNANA	TCTCTNCNGG	401
Crocodylus_porosus	CAGGAAG-CA	GNGATGGTAA	ANGTCTCCTA	AAAACTTGTA	CT-----NGG	GTTCCGCCTT	GATGTGAGGG	401
Erinaceus_europaeus	CTTGGCCTCA	GACTCATTCT	CTGNATCTGN	CCCACCNNTA	CTGCAATTGT	NACNCCTGTA	GTTGGACCAN	378
rnd-6_family-7426-LTR-ERV1	CTAGAATTCC	TAAGGTNCAA	CAGCTNCTGC	AGGACTTTTT	CGACGGGAAG	GAGCCCCGCA	AGAGCGTCAA	896
Esox_lucius	G--GGCAGCA	GCTT--ACCA	CACCCCT-TT	TTCACTGTAA	TAAA------	-TACTGATTG	TGTCATGTAT	374
Salmo_salar	TCTAACATCT	NCTNCAACNT	NGGNCCT-TC	CTNAATANAN	TAAACCTGTT	TTCCTCTTNG	TNGCANAGAG	512
Didelphidae	ACTTATTGCA	GTNA-GATAA	TATTATT-CT	NTC--TTTNG	TNAAG-----	-TACNN-CAG	NGGGAGGTAA	454
Latimeria_chalumnae	TTTNAGGANT	ATTCACTCAC	AGNTAT--AA	NNTACCTTNN	NAATAGAANN	NNATGAACNN	TNTNNTTTCA	1367
Monodelphis_domestica	TTGTACATTT	TTTTCTTCAT	TANTTTTAAA	CTTAATTTGT	CCAT---TTT	TTATATNATT	TACCTTTTTA	383
Macropus_eugenii	GGNGGTNGGA	TNNTCCGCGT	TAGGGCT--A	TATAAGCTGT	GTTTC--TAN	CTCCGTCAGT	TGCCTCCCTA	389
Alligator_mississippiensis	CCACATGCTC	TGCCAATGGC	TTTTATGGNT	ATAAGAAACA	CCCCCTNGAG	AAAATNTGGA	CTGACCCCNT	405
Chrysemys_picta_bellii	CCTGATGCTT	TNCCCCTNGT	ANTACTCNAN	ATNCGTGCTA	TCCCCAAGGG	CAGGTTAGGG	CTTAGTCCCT	2610
Danio_rerio	AGNATTTATA	TTCAATATTA	GGAAANTAA-	ATTGAAATAA	NAATATAAAT	AAGANGNAAA	TTAAAATAAG	530
Taeniopygia	GGGGACNATN	T-ACAANGAC	TNAAAATAT-	GCATAAAAAG	TAGTACATAG	TATATGGAAA	ACATGTGAGG	402
Dasypus_novemcinctus	AAAGTAACTA	ATAAAANTGT	NAAAAGCACC	ATTTGACTCC	TCACCCCTAC	ATTCTCTGTA	TAAAAGGGAC	369
LTR_XT	NNAG-AGATA	NNAAANTATT	NTANAANTAN	ANNACATAGA	TGCTGTNGNT	CAATTGCNTT	ANNNTNTGAT	360
Haplorrhini	ATA---GCAC	GATGGGGAAA	AGACTTTCAA	ACTCAAGTAG	GAGATACAAC	TTGTTTAGAT	CAGCAATATT	7734
Catarrhini	ATA---GCTA	GAGAAGGAAA	AGAATTCACT	CACCCCGTAG	GACGACTTAG	TTGTCTGGGA	CAGAAACTGT	6874
Anolis_carolinensis	----------	----------	----------	----------	----------	----------	----------	1184
Murine_leukemia_virus	------AAAC	CAAATATAAA	AGAGAGCCGG	TGTCATTAAC	TCTGGCCCTG	CTGTTGGGAG	GACTTAC--T	7020
Phascolarctos_cinereus	CCCC-CACTC	TAGAAACAAG	AGAGAGCCTG	TCTCACTCAC	CCTGGCTGTC	CTTCTCGGAT	TAGGGGT--C	6837
Estrildidae	-------AAG	TGAGNAAGCT	CGNGAACGGT	TGTGCATTT-	TGTGNAAAAT	GTCTTGCATA	GAGAACACCT	362
Xenopus_Silurana_tropicalis	-------AGT	TAAGGATAAT	TGAANATTAT	TATTCNGANA	TNTNAGGAAN	ATGNGCTGC	AGGAAAAGTG	652
I_XT	-------TNT	TGNTGCNAAT	ANAACATTAT	TATNCAGAGN	TTNCNNGGAG	GGGCNGCTGA	AGGAAANGNG	7270
Takifugu_rubripes	-------TTC	TNGCCAGATT	TCAGACTTAG	CCCGGGCTAC	TTCTCATGCA	TGGTGAATTG	TGAATCGACA	544
Bos_taurus	TCACANATNT	TCTTCATCCC	TCCTCCGCCC	TNCAATTCCC	TTTTAN---A	TGCCTTTANA	ACTATTTNCC	373
Homo_sapiens	AAA-ATCACT	CTTTCATCAC	TCCCTTACCA	TTTN-TNCCC	CCTTTG---A	TCCCTTTTCA	CTTGCCTCTC	360
Microcebus_murinus	TTANTCACAA	GTTGTGTGTC	TCTNCTTCTT	TGAAAGCCCT	TTGCCNCTGA	AACTCTGGGA	TATGG-AATT	327
Myotis_lucifugus	TAAATTTTAA	TGTCTATGAG	GCCTCCCCCT	TGTTGGTCCA	TGGCTC---G	GACTTTGGCG	TGCATTGGCT	337
Equus_caballus	-------CCA	ATTAAATAAC	TCNTACCTAA	TTTTTTCTTT	GTTTGCTTTG	TGCTTTAAAA	ACCTATGCCT	331
Tupaia_belangeri	ACCTCTTTTC	ACTNAATAAC	AATCTGTGTT	CNCGTCTCCC	GNCGNCAGCG	TTTCTNTAAA	ANGAGCGGCT	375
Loxodonta_africana	TAATANACCT	TNTAAATAAA	AACTCNCCCA	NCCTTTCTCG	GGGAGC--CA	TTTTGGAAGC	CCCAGCCCCN	342
Murinae	NTNTCTGGAN	NTCTAANATN	GACTTGNNGC	GGGNTNCCNT	GGNCTCCACC	TNTCTCCTNN	NATGGCTGNC	386
Canis_lupus_familiaris	ATTACATNTN	NTTATGACTC	AATTCCCATN	CTCNCTNCCT	TTCCCCTTTA	AAACCCTGNA	TTNANNCTTC	388
Sus_scrofa	------TTGC	TGAATGAGCT	AATTTGTGTT	GCTAGTCTTC	CCTTATCTTC	TTGCCCTGTN	A-AACTCTTC	340
Choloepus_hoffmanni	----------	------GANC	AACTTGC-CT	TTTCTTCNTA	TGTACTTAAA	AAACCTTTGA	TCNCTTAANC	327
Vicugna_pacos	------TTTG	TNTATAAACT	AACGCCA-CC	CTCCCTCCCC	TGGGCTTACA	TGTCTCTCAG	NCA-TTAGCC	354
Gavialis_gangeticus	ATTNTGTCTG	NCNTCNGNNC	TCTNATTGGA	--TTNTATTN	TCTCCAATNG	ACNNCGACTG	CTNTNTNTNN	428
Mammalia	AGGAGGTCAG	ACGCCAGGGT	CCTCGCTTNT	CCTTCNCTTG	GGGCNGAACN	CCANNGGGAG	GGGGGCTNTA	489
Passeroidea	ATNGAAAGAA	AAGCCTGATC	CNNNAACTCN	GCCTCGGGCA	NAATAAACAG	TATAAAAACC	GCTTGNACNN	454
Takifugu	ACTGAATTAG	CATCAATAAT	TTNCATATGT	GTTTCTA-TA	AAAGACTGGG	TCAAGGGATA	GTTAGGTTGT	438
Pteropus_vampyrus	CC--AACNAG	NATGTATGAG	CTGCCCTTNC	CTGCCTA-CA	AGGTAAAGTC	TCAGATGAT-	GCTCGGTTCT	344
Ornithorhynchus	GCTNGCAGNC	TCTGGTNNGG	GGCNCTNCNT	CNNTCGTATA	NCNCTCNGGC	TGCTGA-GCT	NGTCGC-TCT	1931
Oryctolagus_cuniculus	GCTTACAAAC	CCCACTACAA	GCTGCTG-AT	NCGTGGTTTT	TCTCTCTAAA	AGCCCNCNCN	CATNGC-T--	369
Gallus_gallus	TTCTNTTACT	NATANTNTGG	TNNCANGTGN	CTTNGNAAAN	TNNAACAAAN	GCTCTNNNNT	CANNNTGTTN	370
Hominidae	NNNANCNNNN	NCANNNNNNN	NANNNNTNCN	NNNTCNCNNN	NCNCGNNNGN	NNNCTGNTNN	NNGTGC-NGN	4101
Metatheria	NNNNCNNNGT	ACNNCCCNNT	TGACTTNNCN	TNGNNNNNNC	TNTNTNNGCT	NNCNNNNNNN	NNNNNN-NGG	424
Rodentia	CTCCGCAAGG	CCGCCCTTAA	AGCGGTAAAT	TATGAAAAAA	TCCAAGAGGT	CATCCAGGAC	AAACAC-GAA	1719
Monodelphis	CNNNCNNNNN	TCNNNGNNNN	TNCNGAAGNG	NCTNGNNNCN	NGNGNGNCNT	NNCTCNNNNN	AGAGAC-TTC	593
Consensus	CCAGACTCTT	TATACATCAN	TAAANTCTAT	TTTTCAATCA	T---CCTGAG	TTCCTGGNTA	TNTCGCTTCT	

Conservation

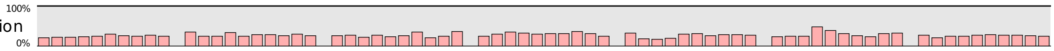

		7,860		7,880		7,900		
Dipodomys_ordii	GCCACTTT--	--CTGNTGTC	TNGTATGACT	NTGCAATCCT	TGTAAGTGTA	TNANAANCCT	GTTGGTCCTC	450
Laurasiatheria	-TTATCTT--	--TTAACACA	----------	----------	----------	----------	----------	361
Eutheria	--TGCTGC--	--CCGATT-C	ATGAATCGT-	TAATTTGCTN	AAATAAACTC	TTTNAAATTT	NTNTCTT---	431
Tarsius_syrichta	-CTGCTCT--	--GCGGCCGC	NAGCTTAAT-	AAAGGTACTT	ANAANATTAN	CTATGGCTCG	GTGTGTT---	455
Muridae	-NNNNNCN--	--NGNANTGC	TAGTCTNANG	NAANNNANNA	AANAANTNAA	CTCTNNNTCA	NNATCTNATA	2228
Gasterosteus_aculeatus	ATAGCANNAG	GACTACNANA	ATGCANNCGA	GNTGTGGCAG	CNGNTGNAAA	GGCNCTAANG	NNCTCTNCTN	1895
Felis_silvestris	CCCGCTTT--	--CTAACAC-	-----TGGGA	GCCCTGGT-G	CACCAGTAAA	GACTCTCTGC	CGAAGTCGGA	443
Rattus	-GACTCTCCT	AACCTCTTGC	NCTCTTCCGA	GCNCGGNATA	CCCTC--CCT	ATATAANCTC	NCTCCNTTTG	416
Papio_hamadryas	AGACGCCTCC	TATCTCTAGC	TCTATTAAGA	GCCCGGAATA	CGCCTAACCG	CTTTGGGCTC	ACTCCATATG	5197
Pan_troglodytes	------TTGG	TACCTCTCCT	GCTCTTTCGG	GCCCGTAATA	CCCTT-----	--ACTGGCTC	ACCCCTTACG	2978
Ailuropoda_melanoleuca	-----CNCGG	GTCCCNCCNN	NCTNTATAAA	NCTNGGTTTA	TAACN---TT	TCGACNCCTN	ACCGCTTCTN	391
Cavia_porcellus	----------	--CCGTATAC	GTTTGCTTGG	AGGACCC-CA	TGCGCACGGA	ATNANAA-AC	CCTTTTGT--	473
Ictidomys_tridecemlineatus	GTCTTGCTCT	CCCTTCTTGA	GNGNGCCCGG	AGCCCCCGCG	TGCTGGTGCC	CCAATAA-AC	CACCCTGCTG	471
Canis	AGTCC-CTGC	TCCGCTGTGT	CGGGNNCACT	TGGNCCCAAG	CTCGAGCTTG	TAAATAA-AC	CCTCGNGT--	421
Felis_catus	CCCTCNGCTT	TNCCGGCTGT	NTGCNNAGGA	CCAG---GTT	CCACTTCACC	CCGATAA-AC	GATCTTTTGT	388
Ochotona_princeps	NTTCTTGCCT	GTCGCTTGGG	CCCCGTCTGA	CTGGCCTGCT	TGNCCCGAGC	TTAACGG-TC	TGTCCTCTGC	428
Cercopithecidae	TNCTNNATCC	CTCTCNCNTG	ACTCTTNTGN	CCCC--NNTC	NCTCTAAACC	CTGCTTC-GC	TNTTGCCATC	1359
Carnivora	CGGGTCCTGT	CCCCGTGCTT	TAATAA--AA	TCACCTTTTT	GCACCAAAGA	CGTCTCA-AG	AATTCTTTCT	381
Mus	NGATNCTGGT	NNACNACTAN	AGNTCAGCAN	NTACTGAATA	ANNCCCCATG	CTNTTNACAG	CANTNTCTGG	1401
Mus_musculus	TCGGCCCCAG	NNTCCGCTGT	T-CGCAAAAA	AAGACCTCTT	GCNTTTTGCA	TCAAGGACGG	TTTCTCTTG-	417
Rattus_norvegicus	CGGNGGTACC	CCTAANNCGT	C-GGATTAAT	AAAACCTCTT	GCTTATTGCA	TCGAACACGG	TCTCCGGTG-	369
Tursiops_truncatus	NCGANCGGCC	GCTCATNTG-	--NAATTAAG	ACTATCTCTN	NTTTAANCTN	CAACTTTCNG	CTTCNGTTG-	476
Procavia_capensis	ACTCGGGGCC	GCTTGCTTGG	A-GCATCAAG	GCCCTGTTTC	CGCCNGTG-A	CTAATGTCTC	CGTAATTCG-	400
Primates	GAAGGCTGCC	GCTTTNCCGG	NCCGAATTAA	TACATACNCA	CNCNTNGAAA	CTAATGCCTT	AGCGCNTAAC	567
Echinops_telfairi	CCGTGG----	GTTGGCACTT	TAGAACCTGA	AACNTC-GCN	TAACCNTGAT	AAATANACAC	TTT---TTTC	411
Sorex_araneus	TCAAGACGCC	ACTGTGTCGG	CTGAGCCTAG	GACCCTAGCT	CGAGCTAGCA	ATAAAGTCCC	TTTGCTTTTG	433
Canidae	GGGACCCCTC	TCACTNCTGA	GAGCTTTC--	-TCTGTATCT	CTGCTTAATA	AACTTCTATC	GCTTTNCTCA	485
Otolemur_garnettii	AGGGGCTNTG	NTTCCTTTTG	NNACTTCT--	-AGTATCTGT	GTCNGGAATA	AAACTTTCCT	CTTACTATTG	353
Gallus	NAGGNATANG	AATTNCGNGA	AAATCATTGA	TTATGTANTA	GTTCGGCCTN	NATCTATAGC	ANGATTTNCA	510
Callithrix_jacchus	NCCNCAA---	------GNNC	AAGTTGNNNT	G----ANATA	NTCACCTCTC	CNTGGCCN--	---NCATCNT	453
Crocodylus_porosus	TCCCTCACTC	TGTTCCGACC	TTCTTGCAAC	G----AAATA	AAACTCTTGT	CTTGNCTA--	---ATGATTG	462
Erinaceus_europaeus	NCTC--TNGA	GCTGTAACAT	NCTGACCTGN	A----NCACA	CNGNNCTG--	----------	----------	420
rnd-6_family-7426-LTR-ERV1	CCCCGACGAG	GCAGTTGCTT	ATGGNGCTGC	T----GTNCA	AGCTGCNATT	CTCAGTGGTG	AGGGTAACGA	962
Esox_lucius	TNCAT-----	----TAGAGA	NTCCTCAGAC	G-ATTATATT	GTGTAAGCGT	TTGANGCGTC	TCTCTTATTT	434
Salmo_salar	TTGNCCTTG-	----TGANAT	CTGTTCTGAC	ACAGTATCAT	GGAGAATNAC	ATNGACNTNT	TGTTATATTC	577
Didelphidae	GTNTT-----	----TTGAGA	ATACNGATAC	AATTANTAAT	AANTCAAATT	CTTAAGAGCT	GCTGNCANCG	515
Latimeria_chalumnae	TTCACGCAGG	CAGTTGGGTN	CATNTAAANG	TATTTCANCG	GNANAANTTT	GTAGAACTGC	GNTNGAAANG	1437
Monodelphis_domestica	NTCAC-----	--------TA	ATTATAAAAT	T--TTAGATT	GGACAAATTT	GNTTTTAGAT	TTTAAGAAAG	438
Macropus_eugenii	CTCNTG----	-------ATT	CTTACAGAAG	A---------	GACGCCCNTT	CTCGCNAGAN	CNTAATAAAA	439
Alligator_mississippiensis	ATGAAGTGCT	GATTGGAAAG	CCATGTCNNT	TTGCTT---T	CCAGCAGTAC	CACNAC----	--ANNCCCAG	466
Chrysemys_picta_bellii	TNGAGATTAT	GATTGGAAGG	GCNTGGCCTN	TGAAGGG--T	ACACTNGCTC	TGGTAG----	--GGGAGTTG	2672
Danio_rerio	TTAAAATATA	ATTTTAAATT	ATATGTAAAA	AGAATAGATT	GTTATAGATG	TTTTNNTAAC	AGAANTNTTT	600
Taeniopygia	ATAGAGNGTN	-TGTTAANTC	ACANGGCNGG	GGCCCAGNGC	ATNTTGGATT	GGTCTC----	---GCTNGCT	464
Dasypus_novemcinctus	CCNAAAATTC	TATTCGGGGC	TCGGATTTTT	GGACAGGAGT	CCCCCGAGCC	CGGCCGGCCG	TTAATAAACC	439
LTR_XT	CATNATTTTG	NAGNCNTGNC	NTNANTATGT	CTGTNTGANT	ACTCNNATCA	NGTAAN----	--AAAAACTT	424
Haplorrhini	TCGAAGAGTC	TAAGAACAAG	ACACAGTGGA	GAAGCTTTAT	AGACAATTCC	TCTGTACCAG	ATTTAACCC	7804
Catarrhini	ATAATGGTAC	CACAAAAACA	GTCACTTGGT	GGAGTTCAAA	TCACA-----	-------CAG	AGAGGAATCC	6932
Anolis_carolinensis	----------	----------	----------	----------	----------	----------	----------	1184
Murine_leukemia_virus	ATGGGCGGCA	TAGCTGCAGG	AGTAGGAACA	GGGACTACAG	CCCTAGTGGC	CACCAAACAA	--TTCGAGCA	7088
Phascolarctos_cinereus	GCAGCAGGTA	TAGGTACCGG	CTCGACCGCC	CTAATAAAAG	GGCCCATAGA	CCTCCAACAA	GGTTTGACTA	6907
Estrildidae	TTGGGGGCAC	ANGCACTGCC	CAAGGTGGAC	CCAGTGNAGT	CTCTT-T---	----TAATAA	-GTCCAGCTA	423
Xenopus_Silurana_tropicalis	CTNNATCNAN	TAGCACTTNG	TTGAA-AGAT	NCAACNGGCT	CTGTTGT---	----CAANAA	CNTTCTGTGN	714
I_XT	CNAAACCANT	TANCTCCTGG	NTCAA-ANNT	ACNACAGCCT	CTTCTGT---	----NAGCAA	NCTTCTGTNT	7332
Takifugu_rubripes	ATAAANCATC	NTTGGGNNCG	TAAACCAGCT	GACCCTGACT	CATCATT---	----CGACTT	TGTNGCGGNA	607
Bos_taurus	CTCACTCANA	CCTCTCAGGG	AGATCGAGGT	TTTTGAGANG	TTGGCTTCCC	GTTTCTCCTC	NTTTGNCTGC	443
Homo_sapiens	GTGAACCCAC	TCTTTTTGAT	AG--------	-CTGTACAAG	TTCTCTTCTC	----TTANTA	ATTAGTATAC	417
Microcebus_murinus	CTAACCCTNG	CNAGCNNCAA	TAA--ACNCT	TCTTTTGATT	TANTCNATCC	----TCTGTT	GCTCGTTCTC	391
Myotis_lucifugus	CTGGTCNTCC	NCGCCTGCAA	T----CCTGT	ACTTACTAAA	TAGGACAATN	----TTTTTA	CTTCTTGCTC	399
Equus_caballus	TTTTCTCCCC	TATTCGGCTC	TTA----TGT	TAATAAAAAC	TTTTTTTTCC	CACAAATGTA	AT-----TCT	392
Tupaia_belangeri	GTGACCTCCC	CATCCCGGCT	GCA----CAG	TAATAAAGTG	TCTTTCNNTT	TCTAATCCTA	GTCTCTGTCT	441
Loxodonta_africana	GTTTNCTCCT	TTNCTTGGCC	AAC----TA-	-AATAAAGNT	NCCTTTCCCT	CTCTCTCGCC	GG-----CCT	401
Murinae	CNCACTCACT	NACTATCTTG	AC-----TGC	TNTTACANGN	CNATNAANTN	----TTTNTA	ATTGGCTANC	447
Canis_lupus_familiaris	CTTCCTGNGN	CCACNCGGGG	AG-----TCA	CTGCTTTNGT	NTGTATCTCC	CGTGTTTTCA	NTACTTAAGN	453
Sus_scrofa	CCTACTCCTC	CCCCTGGGCG	AC-----CAG	CTCNTCTAGG	CCCTAGCC--	--TGCTNTCA	CCTCCTNGGC	401
Choloepus_hoffmanni	NACTCCGGGA	TGCACATTTG	GG-----TTG	TCNCC-TGAA	TNTGCNCNGA	AGTAAATAAA	ATCTTTTGTT	391
Vicugna_pacos	TG--CCGTAA	CTCTCCTTTG	GC-----TGC	TTTCTATAAA	TCTGTTCCCT	TCTTCTACTC	CTCATNCTCT	417
Gavialis_gangeticus	AACGAATACC	AGCTATNT-T	NGNNANGCCT	GATNCCNTCC	CTA-------	----------	----------	470
Mammalia	GAAACAT-CC	AGATAGGTAT	GGGGGAACCC	GAGANCATCG	GGGCT--AGT	GGCNGCTTC	CCCNGGCATA	556
Passeroidea	GANGGTCGNT	GTNATAACAC	AGGGGAACCC	TATGCTGTNG	AANTCGGACC	TGTGTCNCAC	CCAGCGCCGA	524
Takifugu	CAGACTTCAT	GCCCTGNCA-	-ATTGACTCT	GTTATTGTAG	GGTTATGAAC	TGGCCCGGAG	CTCTGTAATA	506
Pteropus_vampyrus	TAGANCNTCN	CAGTTNCTNC	CGGTCTGCNT	CCGTCTGCGT	AAATA--ANC	CGCNTTCTTC	TCTTCAANCA	412
Ornithorhynchus	TANNCGGGCG	NTCCTTGGTT	GTNGGCTTNC	GCGCTAATGT	TTGCACGGAT	TCTCACNAAG	GCTTTGAANT	2001
Oryctolagus_cuniculus	-GCTCGG--G	GCTTCTGACT	GGATGCCTTT	GCCCTAAN--	----------	------CAAG	CCTTGGTAGC	418
Gallus_gallus	CACTACACNG	ATGATTTGAT	GTTAACATCT	GACTCAATG-	----AGGCAT	TAGAAGCAGT	ACTCATAATC	435
Hominidae	ANCNNGGNNN	NCTNNNNANN	NNNGGNNCNN	NANNCNNCAN	CNNNCNTCNT	NNCCNTAAAC	CCTTNNANNN	4171
Metatheria	NGAGCGGCAA	NCTCNNNNTN	GCNNGGCNNG	G---CCATNT	NACNNTNTNN	CNTNNTNAAT	NTCTNNANNT	491
Rodentia	AACCCATCTC	AATTCCTAGA	CCGCCTTACA	AAGGCCCTCT	TACAATATAC	CAATCTGGAT	CCTGAGACCC	1789
Monodelphis	NCNNGGNNNN	CNGCTNTGNN	GNNGNNNNNN	NAGNNCTAAC	NCNNNNTNCC	NNGNTTNANN	NNNGNNNNNG	663
Consensus	CTCACCTTCC	TATCTCNTGG	ATGTNTCTGT	GAACTAAATT	CNCTAATATC	CTTATTACAC	CTTTCTATTC	

Conservation (100% / 0%)

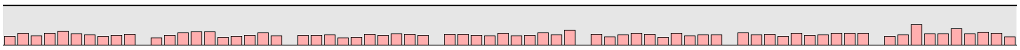

	7,920		7,940		7,960		7,980	
Dipodomys_ordii	TGCTCGGGGC	CCCCTTNCCG	TCATGGGNTT	NGCCTNTGAA	TACNCAAACN	TCCGAGCTCG	AGCTTGCANT	520
Laurasiatheria	----------	----------	----------	----------	----------	----------	----------	361
Eutheria	--GCCAAAGT	TTTTCTTTTA	ACA-------	----------	----------	----------	----------	452
Tarsius_syrichta	--GGCNGTCC	CTTTCTCGCA	CTCCAGTTAT	AACA------	----------	----------	----------	487
Muridae	NTNNCAGCNN	CNNTNNNACN	TTNNNNNNCT	NCNCTGAGGA	ACACCAGAAC	ACTGTCTAAT	GNCTGTGTGA	2298
Gasterosteus_aculeatus	ACATTGTGAN	GTNTNCNCCG	NTAAAGNTGC	TN--ANANCA	CACAGGGTNA	TGTCTACGNA	CAGANANAAT	1963
Felis_silvestris	GTGCCGTGTG	GTTCTTTGCG	CCAACTCTCA	TTCCATAAGG	CCTAGGAGCA	TAATTCCTAA	CA--------	505
Rattus	AGATTC----	---GGGGTCG	GCCCCCCTGT	CC-CCTGAGT	GGGATANGTG	TCTGACCCGN	ATGTCCTNGT	478
Papio_hamadryas	AAATCCTCTA	TGGAGGACCT	CCCCCTTTGT	CAACCTTACT	TAACTCCTTT	TCTCCCTCCA	ATTCTAAGAC	5267
Pan_troglodytes	AAATCAT---	TAGGAGACCT	CCCCCTGCTA	CTTAGATCCT	CTTAAACAAA	ACTGATTTTT	CTTACAAGCT	3045
Ailuropoda_melanoleuca	GGANT-----	---GACNTTT	NTTTTCNTTT	GACACTNTAT	TACAAATAAA	CTCTTCTCCT	TGATCTGGTC	453
Cavia_porcellus	CCTTCACACA	AGAGCTGNGT	CCTTGGGGTC	ATTCCTTG--	-----CGACG	CCGGCCTTAC	A---------	527
Ictidomys_tridecemlineatus	CATTTGCATC	AAGATTGTCT	TCTGTGGTCT	CTTCNTTNGA	G--GCCTNGC	NCGACCTTAC	A---------	530
Canis	-GTTTGCATC	GGTGTNGGCT	CCTTGGTGGT	TTCTCGGATT	C--GCNATCT	TGGGCACAAC	A---------	479
Felis_catus	CC-TTTNTTT	TGNCTCNGCA	GNCAGGTGGT	CTGTTT----	----------	----------	----------	423
Ochotona_princeps	CT-ANTGACA	TAGTTGGGTC	AATTAGCTTT	TTATTGAATA	T--AACTTTC	TGGGTTNGCT	GTCGTCCTCT	495
Cercopithecidae	CAGATCGACC	CCCCATGATC	TCCCGGCTGA	CTACCGACCG	TCGAGGAGCG	CAGGGGCGGC	CGTGATCCTCC	1429
Carnivora	TGGCCGTCGG	--CTCCGAAC	CTCACACATT	CCAA--ACAC	ATCA------	----------	----------	421
Mus	CAGCCCCTTG	ACCNTTNGAN	GNCNAACGCC	CGGATGACNC	ANTACCAGAG	CCTGTTGCTN	ACTGACNGAG	1471
Mus_musculus	NGTCTTTGGG	GTGCTCGCCG	AGTCCTGGGA	CTTGAGTGGG	GGTCTCCCCT	CCGGGCTTTC	A---------	478
Rattus_norvegicus	ATT-----GG	GTGTTCGG-G	ANTTCCCGGA	CTAGAGNGAC	AGATTCCG--	--GGTCTTAC	A---------	420
Tursiops_truncatus	TTTTCTTCGG	NAG--CGTCG	NGCAGCGGGN	CACGTTCGGC	AACA------	----------	----------	518
Procavia_capensis	GCTTCTAGGG	ACA--NGCGG	GATGNACTCT	CTNCTTCCGG	AACA------	----------	----------	442
Primates	ANTCTCTCGG	GTTCAAAAAC	CCCGGCTCCA	NCCCGTCAGC	ATGGTCCCAT	ACGGGGTNTG	AGGNNGGNGN	637
Echinops_telfairi	AAATNNCANG	CNTCGGTGTT	TGTTCTTCCT	CGNNCGNAGC	CNAAACCGAG	GNATGNCTAA	CA--------	473
Sorex_araneus	CATTACCATG	TGTGGGTCTT	TGTCTCTTCG	C-AACGGGGA	ACCCTCGGNC	CCGGNCCTAA	CA--------	494
Canidae	CTCTCTGTTG	TCCGCGAGAT	TCATTCTTCG	ACTCCGTGAG	AC-AAGAACC	AAGCTCTCCC	GTATCA----	550
Otolemur_garnettii	ATCTCTGTGG	TCCGTG-GAT	TCATTCTTCG	AATCCCCGAG	ACCAAGAACC	TGGGTACAAC	A---------	413
Gallus	TNCTGGNAGG	AGTGCNAGTC	NGNTGNNGCA	ATCCCNNTTG	CATCNGGCTN	TACGCGCGTC	GTAAATNAAA	580
Callithrix_jacchus	CATGCTTCGA	-ANTTTGAGA	NGCTNAGAAC	T-CTTCCGCG	AGGT------	----------	----------	495
Crocodylus_porosus	TGTGTCTCAG	TAGATTNCTT	TGNTGTCNGC	GGCCTCCATG	GANTCGAACN	GCGTATTGTG	CAACA-----	527
Erinaceus_europaeus	----------	----------	----------	----------	----------	----------	----------	420
rnd-6_family-7426-LTR-ERV1	GAAGGTTCAA	GACCTTTTGC	TTTTGGATGT	TACTCCTTTG	TCTCTCGGTC	TNGAAACCGC	CGGAGGTGTN	1032
Esox_lucius	G-CATAATAA	TTAATAAACT	TGTTAA--AT	TGTGC--CTA	GAGN----AT	TTTGTCTCTT	TACANCTAAT	495
Salmo_salar	A-CG-GATTG	TGAGGTGGTT	TGTTTA--AT	TGTGCTGCTG	AAGCTTGGAT	TTTGTTTCAT	GATTACTATT	643
Didelphidae	T-CATACCTT	GAGGGTTAGT	TGTTTATGAG	CGGNAATTNC	AATGTTTTTT	TTTGCGTTTT	TTNTACATGT	584
Latimeria_chalumnae	ACNGTNCNCN	GTANTANACA	CNNCCAANAC	TGNTG--TAN	AATGCCAANG	TCNTTCNTCT	NNNTCTAAAT	1505
Monodelphis_domestica	GGCATAAGTT	TTAATAGAAA	TTT---AAGC	TGTT------	AATNCTAAA-	--TCTTCCTT	ATCTTCATTT	496
Macropus_eugenii	TTTGTNTTTT	CTTTTTCTAC	CTT---GAGT	CGTC---TTC	ANTTTCAATT	TGAGTNAGTG	TTTTCTTNCC	503
Alligator_mississippiensis	CCTTTTAGAG	NTGATTAACC	TCT---GTGC	TCATATNTTN	AATCTN-TAC	TGGATCATTT	NAACTCGTTA	532
Chrysemys_picta_bellii	GATTTGAGGN	ATGGTTTCNT	NCTTCAGTAC	AAGTGCTCTC	TGNCAGCTGT	TCTTTCNTTT	CTNCACAG-G	2741
Danio_rerio	TTATTNAACT	AT-ATAANTA	TATTCATTTT	TAATTGAATA	AAATCNTTAA	ATAGGTANCT	AATATAAAGT	669
Taeniopygia	GCCAGNNGTC	TT-AATAAAA	TATCCATTNT	TTATNTAAAA	AGA--CTTNA	TGAGTNTATT	TGTATCAATT	531
Dasypus_novemcinctus	ATTTTTCCTT	CTCAANACNA	TTCTTGAGTC	CTGGCCTTTC	TATACGCCAA	TAATNGAATC	TCTCTCGCTT	509
LTR_XT	ANCTNCNATT	AAACTGACCT	ATCNTACAGT	TTTTTTTTAC	CAGTATNTAN	TNNNTNGCCN	TNNNAAGCAT	494
Haplorrhini	TCTCTCTCAG	TTTCCAGCGC	TAAATCAGTC	GTGGTATCAA	CTAGACGCTC	CAAATGTTTG	GAAAGCACCG	7874
Catarrhini	ATTTAGTAAA	TTCCCAAAGT	TGCAAACCGT	GTGGACCCAC	CCGGAGTCCC	ACCGGGACTG	GACAGCCCCC	7002
Anolis_carolinensis	----------	----------	----------	----------	----------	----------	----------	1184
Murine_leukemia_virus	GC--TCCAGG	CAGCCATACA	TACAGACCTT	GGGGCCTTAG	AAAAATCAGT	CAGTGCCCTA	GAAAAGTCTC	7156
Phascolarctos_cinereus	GCC-TCCAGA	TTGCCATGGA	TACAGACCTT	AGGGCCCTTC	AAGACTCCAT	AAGTAAACTA	GAGGATTCCT	6976
Estrildidae	GTTTTNATT-	TNCTTAACTC	AGNCTAGCCT	CTGTTNTTAG	TTAGTCTTAA	TAAGGCATCA	----------	482
Xenopus_Silurana_tropicalis	NTTNTCNTNA	TTATTCATNT	CATTATNCCT	TTCCTATNTG	TTANGNTNCN	GATGTANTTN	NTTTTTTAGA	784
l_XT	TNGNCNNACN	TTATNCATTN	CAGGATCCCA	TTCCTNTGTA	T-ATNNTANA	GATGNANTTG	CTGNGTTAGA	7401
Takifugu_rubripes	TCTCNGCGNT	CAATTCGTTC	GGCACTGGCA	TAGGTATCTN	TGACTTAAGG	AAAAGCATAA	GTTTATNAAN	677
Bos_taurus	CTTCCAAATA	AAGCCNTA--	CTTTTCTTTT	TTCCACAACT	CGGTGTCTTT	NTATTTTTTG	GCCTCAGCGG	511
Homo_sapiens	CTTT---ATA	AATCCTTTAC	TTTTTCTTTT	TTCTGC----	----------	----------	----------	450
Microcebus_murinus	TTTGTCTCGN	GGACTGACGA	NTTNCTGGCA	TTGAACA---	----------	----------	----------	428
Myotis_lucifugus	TTCGCTTAGT	GG--TGCACG	TNTTTCNGTC	TTGCA-----	----------	----------	----------	432
Equus_caballus	CTAGATTTTT	AG--CTAAAC	TTTTCGCTTC	NAGTA-AACA	GAGCCCTTTT	GTTTGGTTAC	A---------	450
Tupaia_belangeri	CTNGTTTTTT	GG--CTTTGT	TAATCCTCNT	TAAAT-ATCA	GN-TCTGGTN	GCAGTCTTGC	A---------	498
Loxodonta_africana	CTTTTTTATT	GG--CTAATA	NAGTCNCGCC	GAGNAGAACC	TGGGAGCTTC	TCCCGGTAAC	A---------	460
Murinae	NCTGGGNNGT	TGGCCGNNCC	TTTCTTGTTT	CGGACTGTNA	CN--------	----------	----------	489
Canis_lupus_familiaris	CCAAATAAAC	GCTTNCTTGT	TTTACTNTTC	NGCCTTGTTT	TNTCTTAACT	TGNTTCACAN	CAAGAGAACC	523
Sus_scrofa	CTGGCTTAAT	AAAGCTATCT	TTGTCTCTTT	CACCCAAGNC	TCTCC-GGCC	AGGT------	----------	454
Choloepus_hoffmanni	CCTCCACAAT	CGGGTNAGCT	GTTATTCCNT	--TTTAGGCA	AAACC----T	CNGCTAACA-	----------	444
Vicugna_pacos	CTCGAATAAT	TTTTTTTTGC	GTTGCGCAAG	AACNGAGGCA	NNTTT--GTT	TCGTCAACA-	----------	474
Gavialis_gangeticus	----------	----------	----------	----------	----------	----------	----------	470
Mammalia	GCNGGGGGAG	GCTGCAAGCC	TCTAGGAGAA	GCCCCGCATN	CGGCNGGCAC	NNGTCCAACA	TGGCGCGGGA	626
Passeroidea	TCCCNGGCTC	GGCACTGNCC	T-TTTGCTTG	TGGCTCNCTN	AGATAGAANC	TCGATCNCTA	TATAAAAATT	593
Takifugu	TTTGTATCTT	GAATTGGTTC	TATTTGCTAA	ATACATTTTG	AAATTGGCAT	T---TTTCTT	CTTGAAGTCT	573
Pteropus_vampyrus	TTCGTTTGTT	AAA----TCT	TTCTTTNTTT	TGATGTCCCG	GAGCACTTGT	T---TTACTG	CTCAACA---	472
Ornithorhynchus	TTGCCATGCG	ANNGGGGCCT	TGTGGC----	-GAACCCCGNG	GTTANTTNAC	TTCAGCAGGA	GNGCCNGTTG	2066
Oryctolagus_cuniculus	TTNTTTTAAA	AAAAATACTC	TCTGCC----	-TTNCNCGCC	TGTANTTTTT	TCTTGCGTGT	ANGACAAAAA	483
Gallus_gallus	TATTTACAGG	AAAGGATGGC	TATA------	--AACCCAAA	AGTAC--AAG	GCCAGGCTAT	CAGTTAAATT	495
Hominidae	NTTNCANGNG	CNNCNAGAGG	TNNNGNTCAT	NNNNNNNNAN	AGTNNCCANN	NCNCAANNNN	CCNNNNNGCN	4241
Metatheria	TCNAANNTTT	NNTNGAGNCT	NNTAANTNNT	TAGGTNANNN	ANNNNNCNNN	NNCCAGATCG	NANNNTNNTC	561
Rodentia	CAGATGGAAA	ACAACTCCTT	ATGACCTACT	TCTTCCAGAG	TTTCCCTGAT	ATTAGGGCCA	AACTTAAACG	1859
Monodelphis	NCTANTNAAT	NNNTNNGNGN	TTNNNCTAGN	TNNCNTACCC	TATCCTCTCT	CTGATTTTCT	TACTTCACTC	733
Consensus	CTTTTTTATG	TTACTTNACT	TTTTCCCTTT	TTCCCTTGTG	AATACC-AAT	TCGGTCTTT-	----------	

Conservation

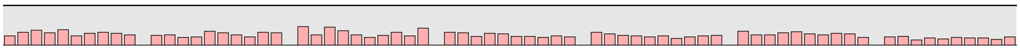

		8,000		8,020		8,040		
Dipodomys_ordii	ATCANCTCCC	GNGTCTGTGC	TTCGGTCCTG	GCCGGTCGGT	GGTCTTTCNG	GATAGAGTTN	NNTATNTGGG	590
Laurasiatheria	----------	----------	----------	----------	----------	----------	----------	361
Eutheria	----------	----------	----------	----------	----------	----------	----------	452
Tarsius_syrichta	----------	----------	----------	----------	----------	----------	----------	487
Muridae	CCCAGTACCA	GGCCCTACTC	CTGGNCCACC	CTCGAGTCTG	GTTTGAGAAA	CCCACCACCA	TCAATCCTGC	2368
Gasterosteus_aculeatus	CTNACANNTN	GNCNGCGCNN	NGACANNGTA	NGNNNACTNG	NCTNTTAGAC	ATGCCNAATG	TCACTNTAAN	2033
Felis_silvestris	----------	----------	----------	----------	----------	----------	----------	505
Rattus	TNA---ATAA	ACCTAACTCT	TGCTGATTAC	ATCGAGAGCG	GTCTC----T	CGTGAGTCNG	GGGC----CT	537
Papio_hamadryas	TGACCTACAG	GCCCGGCTAA	-AAGGACTAC	AAGCAGTACA	GGCCCAAATC	TGGGCCCCCT	TGGCAGAACT	5336
Pan_troglodytes	ACAGAAATCA	AAATTGGCAA	-GCTGAAGAC	TATAAGACCA	GCCCCCACGA	CACACCTACC	AGGGGAGACT	3114
Ailuropoda_melanoleuca	TGAATTCTT-	----------	----------	----------	----------	----------	----------	462
Cavia_porcellus	----------	----------	----------	----------	----------	----------	----------	527
Ictidomys_tridecemlineatus	----------	----------	----------	----------	----------	----------	----------	530
Canis	----------	----------	----------	----------	----------	----------	----------	479
Felis_catus	----------	----------	----------	----------	----------	----------	----------	423
Ochotona_princeps	GG---CTTGA	CAGGATCCTA	ACA-------	----------	----------	----------	----------	515
Cercopithecidae	GGACCCCAAG	CGATAGCCTC	GCTTGAAATT	AACGATAGCT	AGCCAACATT	GGGTTACCAA	GGATAAATTT	1499
Carnivora	----------	----------	----------	----------	----------	----------	----------	421
Mus	TGACTTTCGC	CCCGCTCCAA	CCTCCCTCTC	CAGCGAGCCA	ATGGGCTGGC	GAAGAACTGG	GGACTAGACC	1541
Mus_musculus	----------	----------	----------	----------	----------	----------	----------	478
Rattus_norvegicus	----------	----------	----------	----------	----------	----------	----------	420
Tursiops_truncatus	----------	----------	----------	----------	----------	----------	----------	518
Procavia_capensis	----------	----------	----------	----------	----------	----------	----------	442
Primates	CCGACCAACG	AGCCACCTGG	CCGTGGACTC	TTCA------	----------	----------	----------	671
Echinops_telfairi	----------	----------	----------	----------	----------	----------	----------	473
Sorex_araneus	----------	----------	----------	----------	----------	----------	----------	494
Canidae	----------	----------	----------	----------	----------	----------	----------	550
Otolemur_garnettii	----------	----------	----------	----------	----------	----------	----------	413
Gallus	ACATACCTGT	TTTTATNANT	NTTTGTTGTT	ATAGNGNTTG	ATTCCGCACN	TCA-------	----------	633
Callithrix_jacchus	----------	----------	----------	----------	----------	----------	----------	495
Crocodylus_porosus	----------	----------	----------	----------	----------	----------	----------	527
Erinaceus_europaeus	----------	----------	----------	----------	----------	----------	----------	420
rnd-6_family-7426-LTR-ERV1	ATGACCGCGT	TGATNCCNAG	AAACACNACC	ATCCCAACCA	AGAAAGAGCA	GGTNTTCTCN	ACCTACTCGG	1102
Esox_lucius	TTCTTAAAGG	GTTTTGATAG	ATGGAATTAC	CATCACA---	----------	----------	----------	532
Salmo_salar	CTCTTAA---	--TTTGCAAA	ACGGCACTAC	CTGCTGACAG	TGTTGAATCC	AATATAAGTA	CATTAACCGC	708
Didelphidae	CATAAAACCA	GTATTCTCTA	TTACCATAAC	TACCCTAACT	AATAAGNNTG	NATTCCAT--	----------	642
Latimeria_chalumnae	CGACAGCAG-	--ACGGAGAA	ATTGTTTTTT	CTCCTAAGCA	ACTAACAACT	GCGACAACCA	GATTTATTTT	1572
Monodelphis_domestica	TATAAAAAA-	--ATATAAAA	ATGATTAAGA	ACTTTCGGGG	ACTTAAGGCT	----------	----------	543
Macropus_eugenii	CACACA----	----------	----------	----------	----------	----------	----------	509
Alligator_mississippiensis	CATACNCATG	CCCGTTGCCT	ACCCCTACGA	CATA------	----------	----------	----------	566
Chrysemys_picta_bellii	TNNACCAAAG	GTTGCCANCC	TCTCCTGCCG	GANACCCCTG	TCCACTCCTT	GGAGCCAGGT	GACTCGGTTC	2811
Danio_rerio	TAATTTAAAA	AAAATCAAAT	TAATACAGAA	----------	----------	----------	----------	699
Taeniopygia	TATGACA---	----------	----------	----------	----------	----------	----------	538
Dasypus_novemcinctus	CAC-------	----------	----------	----------	----------	----------	----------	512
LTR_XT	ATAAATAAA-	----------	----------	----------	----------	----------	----------	503
Haplorrhini	GCAGGACTAT	ATTGGATCTG	TGGGACAAAA	GCCTACCAAC	TATT-GCCCG	AGAAGTGGAC	CGGAGCCTGT	7943
Catarrhini	ACTGGATTAT	ACTGGATATG	TGGGCATAGA	GCTTACGCCA	AATT-ACCTG	ACCGGTGGGC	AGGTAGTTGT	7071
Anolis_carolinensis	----------	----------	----------	----------	----------	----------	----------	1184
Murine_leukemia_virus	TGACCTCGTT	GTCTGAGGTG	GTCCTACAGA	ACCGGAGAGG	ATTAGATCTG	CTGTTCCTAA	AAGAAGGAGG	7226
Phascolarctos_cinereus	TAACCTCCCT	GTCTGAAGTA	GTGCTCCAGA	ATAGGAGAGG	CCTTGATCTG	CTATTTTTGA	AGGAAGGGGG	7046
Estrildidae	----------	----------	----------	----------	----------	----------	----------	482
Xenopus_Silurana_tropicalis	TGNAAAGNCT	GTAGCAATTA	TGCCTATA--	----------	----------	----------	----------	812
I_XT	TGNAAANCNT	GNAGGCAATT	CAANACTGAC	ACTNAACNNN	NAGAAGTGAN	CNNAANAGAN	TTCTNGNTNA	7471
Takifugu_rubripes	GTNNTNNGGC	ANAGGGAAAA	AGCCACTACA	----------	----------	----------	----------	707
Bos_taurus	CGGCAGATGA	CTTTCGGTAA	CA--------	----------	----------	----------	----------	533
Homo_sapiens	----------	----------	----------	----------	----------	----------	----------	450
Microcebus_murinus	----------	----------	----------	----------	----------	----------	----------	428
Myotis_lucifugus	----------	----------	----------	----------	----------	----------	----------	432
Equus_caballus	----------	----------	----------	----------	----------	----------	----------	450
Tupaia_belangeri	----------	----------	----------	----------	----------	----------	----------	498
Loxodonta_africana	----------	----------	----------	----------	----------	----------	----------	460
Murinae	----------	----------	----------	----------	----------	----------	----------	489
Canis_lupus_familiaris	CTGTTCGGTA	ACA-------	----------	----------	----------	----------	----------	536
Sus_scrofa	----------	----------	----------	----------	----------	----------	----------	454
Choloepus_hoffmanni	----------	----------	----------	----------	----------	----------	----------	444
Vicugna_pacos	----------	----------	----------	----------	----------	----------	----------	474
Gavialis_gangeticus	----------	----------	----------	----------	----------	----------	----------	470
Mammalia	GCAATAGTGC	AGCACTGGCG	GAGGGAGGTG	GCTAGATAGA	TGAGCCTGAG	GCAGCGCTCC	TGGCTCCCCA	696
Passeroidea	TATNTTTATT	AATTTTTAAT	TTGGCTNGAT	CAATTTTTAN	CTATAACA--	----------	----------	641
Takifugu	TCATTCAAAG	AACACGCGGA	TTGGCAGTGA	CACACGAGAG	GTATTGCGAT	CCAACA----	----------	629
Pteropus_vampyrus	----------	----------	----------	----------	----------	----------	----------	472
Ornithorhynchus	CCC--ACGGN	ACTATGCGGG	CTTTAGGCTG	CAGACCGCTG	CTGCGTATCC	AGTGACCACA	CGGGACTTAG	2134
Oryctolagus_cuniculus	CCCG-ACGTC	AC--------	----------	----------	----------	----------	----------	494
Gallus_gallus	CCTGGGTGTT	GTC-------	----------	----------	----------	----------	----------	508
Hominidae	CNAGGAAATN	NNCTANCNGA	CNCNACNNNT	NNGNCNCTNG	NNNTNANNTC	TGCCCCNGCC	NCGGAGCNNC	4311
Metatheria	CNANNACA--	----------	----------	----------	----------	----------	----------	569
Rodentia	TCTAGAGAGG	GGACCCCTGA	CTCCACAGGC	AGAAGTCTTA	GCGGTGGCCT	TTAAGGTATA	CCATGGGAGA	1929
Monodelphis	TTTCTATATT	TTGTAAATAA	AT-TGTAAAT	AAATCTCTTT	GGAATAAA--	TTAAATTCCT	GGCGACCCTA	800
Consensus	----------	----------	----------	----------	----------	----------	----------	

Conservation (100% / 0%)

	8,060		8,080		8,100		8,120	
Dipodomys_ordii	TACAACA---	----------	----------	----------	----------	----------	----------	597
Laurasiatheria	----------	----------	----------	----------	----------	----------	----------	361
Eutheria	----------	----------	----------	----------	----------	----------	----------	452
Tarsius_syrichta	----------	----------	----------	----------	----------	----------	----------	487
Muridae	TACCCTCTTG	CCTGATGACG	ACCCGCAGGT	GCTCATCCAT	GACTGTCACG	AGATGAAAGA	TAAAACCAGG	2438
Gasterosteus_aculeatus	ACNCTNAAGT	NTCCANANCN	CNGCATGATT	GCTANCGACT	NNNGNGGATG	ANGAGGANNA	NAANTGTTTG	2103
Felis_silvestris	----------	----------	----------	----------	----------	----------	----------	505
Rattus	GCGCCCTCCC	GAGACCT---	-GAGCGAGCG	TCCNCTCCAG	GTCGGNGGGT	CTTTCA----	----------	589
Papio_hamadryas	GTACCGGCCA	GGACATTCGC	AGACCAGCCA	CCCCTTCCAG	GTGGGGGACT	CCGTCTACGT	TAGACGACAC	5406
Pan_troglodytes	GGTCGGTTAA	CGACACC---	-GACTAGAC-	------CCGG	TGGAAGGACC	ACGTCTCCT-	--GACCACAC	3170
Ailuropoda_melanoleuca	----------	----------	----------	----------	----------	----------	----------	462
Cavia_porcellus	----------	----------	----------	----------	----------	----------	----------	527
Ictidomys_tridecemlineatus	----------	----------	----------	----------	----------	----------	----------	530
Canis	----------	----------	----------	----------	----------	----------	----------	479
Felis_catus	----------	----------	----------	----------	----------	----------	----------	423
Ochotona_princeps	----------	----------	----------	----------	----------	----------	----------	515
Cercopithecidae	GCTCCAATGC	ATTCATGGCA	CAGACCTCGG	AACGCGAGCG	GGCCCCCGCC	CCCATCAGAA	CTGGAACTCC	1569
Carnivora	----------	----------	----------	----------	----------	----------	----------	421
Mus	TTGCCGGTGG	TCACGAGGAG	AGCTTTGGTG	AGGAAAGCGG	GGCAGTGTGA	GAAGTATTGG	GCAGCTCGAG	1611
Mus_musculus	----------	----------	----------	----------	----------	----------	----------	478
Rattus_norvegicus	----------	----------	----------	----------	----------	----------	----------	420
Tursiops_truncatus	----------	----------	----------	----------	----------	----------	----------	518
Procavia_capensis	----------	----------	----------	----------	----------	----------	----------	442
Primates	----------	----------	----------	----------	----------	----------	----------	671
Echinops_telfairi	----------	----------	----------	----------	----------	----------	----------	473
Sorex_araneus	----------	----------	----------	----------	----------	----------	----------	494
Canidae	----------	----------	----------	----------	----------	----------	----------	550
Otolemur_garnettii	----------	----------	----------	----------	----------	----------	----------	413
Gallus	----------	----------	----------	----------	----------	----------	----------	633
Callithrix_jacchus	----------	----------	----------	----------	----------	----------	----------	495
Crocodylus_porosus	----------	----------	----------	----------	----------	----------	----------	527
Erinaceus_europaeus	----------	----------	----------	----------	----------	----------	----------	420
rnd-6_family-7426-LTR-ERV1	ACAACCAAAC	CGGCGTNTTG	ATCCAGGTNT	ACGAAGGTGA	AAGAGCGAGG	ACNAGGGACA	ACAANCTTGT	1172
Esox_lucius	----------	----------	----------	----------	----------	----------	----------	532
Salmo_salar	ACTTCTAACA	GACGTCATTG	GACAGGGTAC	TCATTAGTGA	ACGACAGATT	AATTGAGATG	GAAATTGCAA	778
Didelphidae	----------	----------	----------	----------	----------	----------	----------	642
Latimeria_chalumnae	ATAACCAAGT	TATTAACTTT	TTTTCAAACA	TGAGTATCAA	TATCAACATG	TCGAACTCTA	CAAGGCCAAT	1642
Monodelphis_domestica	----------	----------	----------	----------	----------	----------	----------	543
Macropus_eugenii	----------	----------	----------	----------	----------	----------	----------	509
Alligator_mississippiensis	----------	----------	----------	----------	----------	----------	----------	566
Chrysemys_picta_bellii	TCTCGCAGAG	CCTCCGAGGT	GGAAGGGCCC	ACGTCCTGCT	CCCACACGCG	AAGTAGGACC	CTGGATCATT	2881
Danio_rerio	----------	----------	----------	----------	----------	----------	----------	699
Taeniopygia	----------	----------	----------	----------	----------	----------	----------	538
Dasypus_novemcinctus	----------	----------	----------	----------	----------	----------	----------	512
LTR_XT	----------	----------	----------	----------	----------	----------	----------	503
Haplorrhini	GTGTTAGGGA	CAATAAGACC	GTCCTTCTTC	TTGCTCCCAC	TGAAACAAGG	GGAAGATTTA	AGTTACCCAG	8013
Catarrhini	GTTATTGGCA	CTATTAAACC	ATCTTTCTTC	CTACTGCCCA	TAAAAACAGG	CGAACTCCTG	GGCTTCCCTG	7141
Anolis_carolinensis	----------	----------	----------	----------	----------	----------	----------	1184
Murine_leukemia_virus	ATTATGTGCT	GCCCTAAAAG	AAGAATGCTG	TTTCTACGCG	GACCACACTG	GCGTAGTAAG	GGATAGCATG	7296
Phascolarctos_cinereus	CCTTTGTGCA	GCCCTAAAAG	AGGAATGCTG	TTTCTATGTT	GACCACTCAG	GCGCGGTGCG	AGACTCCATG	7116
Estrildidae	----------	----------	----------	----------	----------	----------	----------	482
Xenopus_Silurana_tropicalis	----------	----------	----------	----------	----------	----------	----------	812
I_XT	GAAAAAATAT	ATGTTAAGAT	TTTGCACAAC	ANGNAGGA--	----------	----------	----------	7509
Takifugu_rubripes	----------	----------	----------	----------	----------	----------	----------	707
Bos_taurus	----------	----------	----------	----------	----------	----------	----------	533
Homo_sapiens	----------	----------	----------	----------	----------	----------	----------	450
Microcebus_murinus	----------	----------	----------	----------	----------	----------	----------	428
Myotis_lucifugus	----------	----------	----------	----------	----------	----------	----------	432
Equus_caballus	----------	----------	----------	----------	----------	----------	----------	450
Tupaia_belangeri	----------	----------	----------	----------	----------	----------	----------	498
Loxodonta_africana	----------	----------	----------	----------	----------	----------	----------	460
Murinae	----------	----------	----------	----------	----------	----------	----------	489
Canis_lupus_familiaris	----------	----------	----------	----------	----------	----------	----------	536
Sus_scrofa	----------	----------	----------	----------	----------	----------	----------	454
Choloepus_hoffmanni	----------	----------	----------	----------	----------	----------	----------	444
Vicugna_pacos	----------	----------	----------	----------	----------	----------	----------	474
Gavialis_gangeticus	----------	----------	----------	----------	----------	----------	----------	470
Mammalia	TGGCCTGCGC	GTGGCNTGCA	GGGGATCCAG	AAGTTCCCNC	GTGCCCCGGT	GAGGGGACGC	GGAGGTGCTG	766
Passeroidea	----------	----------	----------	----------	----------	----------	----------	641
Takifugu	----------	----------	----------	----------	----------	----------	----------	629
Pteropus_vampyrus	----------	----------	----------	----------	----------	----------	----------	472
Ornithorhynchus	CCTGGGAACG	AGCGACACCC	GCTGGGGCTG	CTCGACCGTC	CGAACACTGG	GCTCTGAGCG	GGACCCGTGC	2204
Oryctolagus_cuniculus	----------	----------	----------	----------	----------	----------	----------	494
Gallus_gallus	----------	----------	----------	----------	----------	----------	----------	508
Hominidae	CGCNNTGTNN	CCNNNNGGNN	CACGCACCCC	TNCCNNNNNN	NCNNAGGNGA	CCNTCCANCT	CATNTCCAAC	4381
Metatheria	----------	----------	----------	----------	----------	----------	----------	569
Rodentia	GATGAAAAAG	CCTGAAAACA	AAAATACC--	-----TGATG	CTGGCTAAGG	CCTTTCAGCC	---GGCCATA	1989
Monodelphis	TTTTAAATAT	AATCCAGTCC	AACCTTTTTA	TAAATTAACC	CCTTTTCCCC	CCTTACA---	----------	857
Consensus	----------	----------	----------	----------	----------	----------	----------	

Conservation
100%
0%
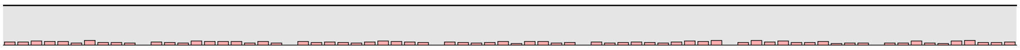

	8,140		8,160		8,180			
Dipodomys_ordii	----------	----------	----------	----------	----------	----------	----------	597
Laurasiatheria	----------	----------	----------	----------	----------	----------	----------	361
Eutheria	----------	----------	----------	----------	----------	----------	----------	452
Tarsius_syrichta	----------	----------	----------	----------	----------	----------	----------	487
Muridae	TACGTGGGAG	CTGCAGTAGT	TACTTTAACT	GAAATGATTT	GGGCCCAAGC	CAGGTACCTC	AGCCCAGAGA	2508
Gasterosteus_aculeatus	GCTGTCAAAG	CTGAGNNCAC	ACTTNGGACG	GACACAGNNG	ACACTCTACT	GCTTAACAAC	TACNTNGTNA	2173
Felis_silvestris	----------	----------	----------	----------	----------	----------	----------	505
Rattus	----------	----------	----------	----------	----------	----------	----------	589
Papio_hamadryas	CGTTCTCAGG	GACTGGAGCC	TCGGTGGAAA	GGACCCTACA	TTGTTCTCCT	GACCACACCC	ACAGCCATAA	5476
Pan_troglodytes	CCACCGCCTG	AAGTGAAGGT	TGCCTGGATC	CACACCCAC-	--GTAACAGT	GGACCCACCG	ATGGGCTCCC	3237
Ailuropoda_melanoleuca	----------	----------	----------	----------	----------	----------	----------	462
Cavia_porcellus	----------	----------	----------	----------	----------	----------	----------	527
Ictidomys_tridecemlineatus	----------	----------	----------	----------	----------	----------	----------	530
Canis	----------	----------	----------	----------	----------	----------	----------	479
Felis_catus	----------	----------	----------	----------	----------	----------	----------	423
Ochotona_princeps	----------	----------	----------	----------	----------	----------	----------	515
Cercopithecidae	AAGTTAATCA	CACTGATCAA	CTGCTGAACC	CCCGGGGCCG	GATGGAATGA	TTACATCCAT	TACTCTTTGT	1639
Carnivora	----------	----------	----------	----------	----------	----------	----------	421
Mus	GACTCGCCAA	AGCGACTTCT	AAAGCTTGGC	AGAGGAGCTA	ATTAACTGAC	AGCTAGCTTT	TGCCACGCCA	1681
Mus_musculus	----------	----------	----------	----------	----------	----------	----------	478
Rattus_norvegicus	----------	----------	----------	----------	----------	----------	----------	420
Tursiops_truncatus	----------	----------	----------	----------	----------	----------	----------	518
Procavia_capensis	----------	----------	----------	----------	----------	----------	----------	442
Primates	----------	----------	----------	----------	----------	----------	----------	671
Echinops_telfairi	----------	----------	----------	----------	----------	----------	----------	473
Sorex_araneus	----------	----------	----------	----------	----------	----------	----------	494
Canidae	----------	----------	----------	----------	----------	----------	----------	550
Otolemur_garnettii	----------	----------	----------	----------	----------	----------	----------	413
Gallus	----------	----------	----------	----------	----------	----------	----------	633
Callithrix_jacchus	----------	----------	----------	----------	----------	----------	----------	495
Crocodylus_porosus	----------	----------	----------	----------	----------	----------	----------	527
Erinaceus_europaeus	----------	----------	----------	----------	----------	----------	----------	420
rnd-6_family-7426-LTR-ERV1	TGGGCGAATT	TGAGCTCTCT	GGTATCCCTC	CTGCACCAAG	GGGNGTTCCT	CAAATCGANG	TCCGCTTCGA	1242
Esox_lucius	----------	----------	----------	----------	----------	----------	----------	532
Salmo_salar	ATTGTATCAG	AGGGGAAGGT	ATA-------	----------	----------	----------	----------	801
Didelphidae	----------	----------	----------	----------	----------	----------	----------	642
Latimeria_chalumnae	ATTTAGGGCA	TGTAAATCCT	AGTCTACTCG	CCCTCTATCT	GCAAAGGCTA	TAAGAAACTT	GGCATCAACA	1712
Monodelphis_domestica	----------	----------	----------	----------	----------	----------	----------	543
Macropus_eugenii	----------	----------	----------	----------	----------	----------	----------	509
Alligator_mississippiensis	----------	----------	----------	----------	----------	----------	----------	566
Chrysemys_picta_bellii	CTCAAAGCCC	CCCGCCACCA	GAACCCGAGT	TGCTTCTTCA	AAGAAGGCGA	ATGCTGTACT	CTCTACAGCA	2951
Danio_rerio	----------	----------	----------	----------	----------	----------	----------	699
Taeniopygia	----------	----------	----------	----------	----------	----------	----------	538
Dasypus_novemcinctus	----------	----------	----------	----------	----------	----------	----------	512
LTR_XT	----------	----------	----------	----------	----------	----------	----------	503
Haplorrhini	TCTATAACGA	AGAAAGAAAA	AGGACCAGAA	GAAACGTCTT	TACTCAGATA	AGTACCGTAG	AAAAGATAAA	8083
Catarrhini	TCTATG----	----------	----------	-------CTT	CCCGC-----	---------G	AAAAGAGAAG	7166
Anolis_carolinensis	----------	----------	----------	----------	----------	----------	----------	1184
Murine_leukemia_virus	GCTAAG----	----------	----------	----------	--CTAAGAGA	AAGGCTAAAC	CAGAGACAAA	7330
Phascolarctos_cinereus	AGGAGA----	----------	----------	----------	--CTCAAGGA	AAGGTTAGAT	AAGAGGCAGT	7150
Estrildidae	----------	----------	----------	----------	----------	----------	----------	482
Xenopus_Silurana_tropicalis	----------	----------	----------	----------	----------	----------	----------	812
I_XT	----------	----------	----------	----------	----------	----------	----------	7509
Takifugu_rubripes	----------	----------	----------	----------	----------	----------	----------	707
Bos_taurus	----------	----------	----------	----------	----------	----------	----------	533
Homo_sapiens	----------	----------	----------	----------	----------	----------	----------	450
Microcebus_murinus	----------	----------	----------	----------	----------	----------	----------	428
Myotis_lucifugus	----------	----------	----------	----------	----------	----------	----------	432
Equus_caballus	----------	----------	----------	----------	----------	----------	----------	450
Tupaia_belangeri	----------	----------	----------	----------	----------	----------	----------	498
Loxodonta_africana	----------	----------	----------	----------	----------	----------	----------	460
Murinae	----------	----------	----------	----------	----------	----------	----------	489
Canis_lupus_familiaris	----------	----------	----------	----------	----------	----------	----------	536
Sus_scrofa	----------	----------	----------	----------	----------	----------	----------	454
Choloepus_hoffmanni	----------	----------	----------	----------	----------	----------	----------	444
Vicugna_pacos	----------	----------	----------	----------	----------	----------	----------	474
Gavialis_gangeticus	----------	----------	----------	----------	----------	----------	----------	470
Mammalia	AGAGGGCCGG	TGGACCNGCA	GAGGCCTGGG	GTCNGGACGA	AGAGGCCGCN	GNGTGCGGGG	ACTTCGNGAC	836
Passeroidea	----------	----------	----------	----------	----------	----------	----------	641
Takifugu	----------	----------	----------	----------	----------	----------	----------	629
Pteropus_vampyrus	----------	----------	----------	----------	----------	----------	----------	472
Ornithorhynchus	CGCCGCTTAC	GGAGGAGGGT	CTGTTGCGAC	CAATAGAGGC	TACCGAGCGA	GGGTACTTCT	GGTCCCGGCC	2274
Oryctolagus_cuniculus	----------	----------	----------	----------	----------	----------	----------	494
Gallus_gallus	----------	----------	----------	----------	----------	----------	----------	508
Hominidae	TTAAAAGGNA	NGACNNNNNA	AGANGNNNNN	ANNNGGGNCG	ATANCCNNNT	NNCCCTNNCC	GANTCCCAGG	4451
Metatheria	----------	----------	----------	----------	----------	----------	----------	569
Rodentia	GCAACTGCCC	AGGCTCCCTG	---CCTCCCA	AAGCCCGAGA	ACCTCCAGGT	CCCTGCTTCA	AATGTGGTCA	2056
Monodelphis	----------	----------	----------	----------	----------	----------	----------	857
Consensus	----------	----------	----------	----------	----------	----------	----------	

Conservation
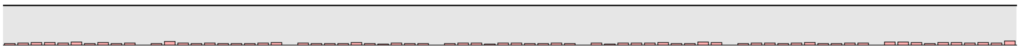

	8,200		8,220		8,240		8,260	
Dipodomys_ordii	----------	----------	----------	----------	----------	----------	----------	597
Laurasiatheria	----------	----------	----------	----------	----------	----------	----------	361
Eutheria	----------	----------	----------	----------	----------	----------	----------	452
Tarsius_syrichta	----------	----------	----------	----------	----------	----------	----------	487
Muridae	GCAGAACTGA	TTGATCTGAC	CCAGGCTCTC	AGATGGGGAA	AAGGAAAGTC	CACCACCATA	TACACGGACA	2578
Gasterosteus_aculeatus	TGGTTGTTGT	TTTANCCGTC	CAACGNNATC	CNNAGANTGG	TGATAAGCGT	GTAGNTAGNN	CTNTTGTTTC	2243
Felis_silvestris	----------	----------	----------	----------	----------	----------	----------	505
Rattus	----------	----------	----------	----------	----------	----------	----------	589
Papio_hamadryas	AGGTTGACGG	AATCGCCACG	TGGATTCACG	CATCCCACGC	CAAGGCTGCT	CCAGGGACGC	CTGGACCGAC	5546
Pan_troglodytes	GGGCCAACGA	CACGCGTACC	TAGA---ACC	CTTTCCCGCC	CGGAGCAAAC	TCCTCAACAT	CGTTATGCAA	3304
Ailuropoda_melanoleuca	----------	----------	----------	----------	----------	----------	----------	462
Cavia_porcellus	----------	----------	----------	----------	----------	----------	----------	527
Ictidomys_tridecemlineatus	----------	----------	----------	----------	----------	----------	----------	530
Canis	----------	----------	----------	----------	----------	----------	----------	479
Felis_catus	----------	----------	----------	----------	----------	----------	----------	423
Ochotona_princeps	----------	----------	----------	----------	----------	----------	----------	515
Cercopithecidae	GAACTTTCAG	GTGGAGCTCC	CACGAACGCA	TGGCAATTGA	ATCTCCAGTG	GTCCCATTCG	AAAGGCCGCT	1709
Carnivora	----------	----------	----------	----------	----------	----------	----------	421
Mus	TCAGCATTAG	ACAGGGCTTA	CTCGCGGAAG	AATAAAAATA	AAGAGAATTC	TTGAGCTCAT	GCCAGTGGAT	1751
Mus_musculus	----------	----------	----------	----------	----------	----------	----------	478
Rattus_norvegicus	----------	----------	----------	----------	----------	----------	----------	420
Tursiops_truncatus	----------	----------	----------	----------	----------	----------	----------	518
Procavia_capensis	----------	----------	----------	----------	----------	----------	----------	442
Primates	----------	----------	----------	----------	----------	----------	----------	671
Echinops_telfairi	----------	----------	----------	----------	----------	----------	----------	473
Sorex_araneus	----------	----------	----------	----------	----------	----------	----------	494
Canidae	----------	----------	----------	----------	----------	----------	----------	550
Otolemur_garnettii	----------	----------	----------	----------	----------	----------	----------	413
Gallus	----------	----------	----------	----------	----------	----------	----------	633
Callithrix_jacchus	----------	----------	----------	----------	----------	----------	----------	495
Crocodylus_porosus	----------	----------	----------	----------	----------	----------	----------	527
Erinaceus_europaeus	----------	----------	----------	----------	----------	----------	----------	420
rnd-6_family-7426-LTR-ERV1	CATTGACGCC	AATGGTATNT	TAAATGTNTC	CGCNGAGGAC	AAGACTACCG	GACAGAAGAA	CAAGATTACC	1312
Esox_lucius	----------	----------	----------	----------	----------	----------	----------	532
Salmo_salar	----------	----------	----------	----------	----------	----------	----------	801
Didelphidae	----------	----------	----------	----------	----------	----------	----------	642
Latimeria_chalumnae	GGCCCAGCCC	AAAGAATTCG	TAACAGCACG	CTGGCGATTT	TTACTCTGTA	TCAA------	----------	1766
Monodelphis_domestica	----------	----------	----------	----------	----------	----------	----------	543
Macropus_eugenii	----------	----------	----------	----------	----------	----------	----------	509
Alligator_mississippiensis	----------	----------	----------	----------	----------	----------	----------	566
Chrysemys_picta_bellii	GCATCCCGAA	ACCAGATTCT	ATCGGACTAA	CGGCTTTTGG	ATTCTTTCAT	TGTTAAGTAG	TGGGTTAGTT	3021
Danio_rerio	----------	----------	----------	----------	----------	----------	----------	699
Taeniopygia	----------	----------	----------	----------	----------	----------	----------	538
Dasypus_novemcinctus	----------	----------	----------	----------	----------	----------	----------	512
LTR_XT	----------	----------	----------	----------	----------	----------	----------	503
Haplorrhini	CACAAACATA	AAGAAGGACA	TTGAGATAGG	TAGCTGGAAA	GATAATGAAT	GGCCTCCTGA	AAGAATTATC	8153
Catarrhini	CATAGCTATA	----------	--------GG	AAATTGGAAA	GATGATGAAT	GGCCCCCTGA	GAGAATCATA	7218
Anolis_carolinensis	----------	----------	----------	----------	----------	----------	----------	1184
Murine_leukemia_virus	AATTGTTCGA	ATCAGGACAA	G-------GG	TGGTTTGAGG	GACTGTTTAA	CAGGTCCCCA	TGGTTCACGA	7393
Phascolarctos_cinereus	TAGAGCACCA	AAAGAATTTA	A-------GT	TGGTACGAGG	GATGGTTCAA	CCGTTCCCCC	TGGCTTACTA	7213
Estrildidae	----------	----------	----------	----------	----------	----------	----------	482
Xenopus_Silurana_tropicalis	----------	----------	----------	----------	----------	----------	----------	812
I_XT	----------	----------	----------	----------	----------	----------	----------	7509
Takifugu_rubripes	----------	----------	----------	----------	----------	----------	----------	707
Bos_taurus	----------	----------	----------	----------	----------	----------	----------	533
Homo_sapiens	----------	----------	----------	----------	----------	----------	----------	450
Microcebus_murinus	----------	----------	----------	----------	----------	----------	----------	428
Myotis_lucifugus	----------	----------	----------	----------	----------	----------	----------	432
Equus_caballus	----------	----------	----------	----------	----------	----------	----------	450
Tupaia_belangeri	----------	----------	----------	----------	----------	----------	----------	498
Loxodonta_africana	----------	----------	----------	----------	----------	----------	----------	460
Murinae	----------	----------	----------	----------	----------	----------	----------	489
Canis_lupus_familiaris	----------	----------	----------	----------	----------	----------	----------	536
Sus_scrofa	----------	----------	----------	----------	----------	----------	----------	454
Choloepus_hoffmanni	----------	----------	----------	----------	----------	----------	----------	444
Vicugna_pacos	----------	----------	----------	----------	----------	----------	----------	474
Gavialis_gangeticus	----------	----------	----------	----------	----------	----------	----------	470
Mammalia	CAGAGGCAAA	TGGCCGGGAC	CACGGACTCC	AGCGGTGGGT	GCCAGCACNA	NNCCCCAAAA	GGCCAGANGG	906
Passeroidea	----------	----------	----------	----------	----------	----------	----------	641
Takifugu	----------	----------	----------	----------	----------	----------	----------	629
Pteropus_vampyrus	----------	----------	----------	----------	----------	----------	----------	472
Ornithorhynchus	CCCATCTCCC	TTGCTGTGTT	TGTCATTGAC	ACAGTTCCCA	TTGGGCGGGC	CTTGCGCGGT	TCGTTACTGG	2344
Oryctolagus_cuniculus	----------	----------	----------	----------	----------	----------	----------	494
Gallus_gallus	----------	----------	----------	----------	----------	----------	----------	508
Hominidae	NTNANGCTNN	NNNTANCGNG	TNNGNNCANN	NCCNTNNTNN	TAGNCNCAAN	NNNNNNNTAN	NNNNTNTTNN	4521
Metatheria	----------	----------	----------	----------	----------	----------	----------	569
Rodentia	GGAGGGTCAC	TGGGCCCGGG	CCTGTCCNAA	CCCTCGTAAA	CCACCTNGAC	CATGTCCAAG	GTGCCATCAA	2126
Monodelphis	----------	----------	----------	----------	----------	----------	----------	857
Consensus	----------	----------	----------	----------	----------	----------	----------	

Conservation 100% 0%

	8,280		8,300		8,320			
Dipodomys_ordii	----------	----------	----------	----------	----------	----------	----------	597
Laurasiatheria	----------	----------	----------	----------	----------	----------	----------	361
Eutheria	----------	----------	----------	----------	----------	----------	----------	452
Tarsius_syrichta	----------	----------	----------	----------	----------	----------	----------	487
Muridae	GTCGCTATGC	TTTTACTACC	GGGCATGTCC	ACGGTATGAT	CTATAGAGAA	AGAGGGCTTC	TCACCGCTGG	2648
Gasterosteus_aculeatus	AGACTNANNG	GTTCTGTGTG	CAGATNCTNT	TCCNNGTCAC	CTCTCAGCCC	AGNCAGCAGA	ANTNNTCGCA	2313
Felis_silvestris	----------	----------	----------	----------	----------	----------	----------	505
Rattus	----------	----------	----------	----------	----------	----------	----------	589
Papio_hamadryas	GTCGTCCGGA	ACTTGGAGAC	TCCGCCGCTC	CGAGGACCCC	CTTAAGATAA	GACTCTCACG	CACTTGACTT	5616
Pan_troglodytes	TCTAATTGCC	CGGCGAAAAA	GAGCCCTAAC	CTGTGATGCT	TTATGAACAA	ACCCCTGGCA	CATAGCCGGA	3374
Ailuropoda_melanoleuca	----------	----------	----------	----------	----------	----------	----------	462
Cavia_porcellus	----------	----------	----------	----------	----------	----------	----------	527
Ictidomys_tridecemlineatus	----------	----------	----------	----------	----------	----------	----------	530
Canis	----------	----------	----------	----------	----------	----------	----------	479
Felis_catus	----------	----------	----------	----------	----------	----------	----------	423
Ochotona_princeps	----------	----------	----------	----------	----------	----------	----------	515
Cercopithecidae	TCTCATACAG	TGCTGAGGAA	TCACTACCCA	TCGGAGGTAG	AGAAGTAATA	CAATACTGAA	CGTGGCTCTC	1779
Carnivora	----------	----------	----------	----------	----------	----------	----------	421
Mus	TCTGCGGCAC	AAAGCGTGAG	GAATCAATGG	CAGGCAAAAG	GCAAGGGTGA	GCAAGCTGAA	AATAGATAGA	1821
Mus_musculus	----------	----------	----------	----------	----------	----------	----------	478
Rattus_norvegicus	----------	----------	----------	----------	----------	----------	----------	420
Tursiops_truncatus	----------	----------	----------	----------	----------	----------	----------	518
Procavia_capensis	----------	----------	----------	----------	----------	----------	----------	442
Primates	----------	----------	----------	----------	----------	----------	----------	671
Echinops_telfairi	----------	----------	----------	----------	----------	----------	----------	473
Sorex_araneus	----------	----------	----------	----------	----------	----------	----------	494
Canidae	----------	----------	----------	----------	----------	----------	----------	550
Otolemur_garnettii	----------	----------	----------	----------	----------	----------	----------	413
Gallus	----------	----------	----------	----------	----------	----------	----------	633
Callithrix_jacchus	----------	----------	----------	----------	----------	----------	----------	495
Crocodylus_porosus	----------	----------	----------	----------	----------	----------	----------	527
Erinaceus_europaeus	----------	----------	----------	----------	----------	----------	----------	420
rnd-6_family-7426-LTR-ERV1	ATTACGAACG	ACAAGGGTAG	GCTNTCGAAG	GAAGAGATTG	AGAGGATGGT	NCGGGAAGCT	GAGAAGTACA	1382
Esox_lucius	----------	----------	----------	----------	----------	----------	----------	532
Salmo_salar	----------	----------	----------	----------	----------	----------	----------	801
Didelphidae	----------	----------	----------	----------	----------	----------	----------	642
Latimeria_chalumnae	----------	----------	----------	----------	----------	----------	----------	1766
Monodelphis_domestica	----------	----------	----------	----------	----------	----------	----------	543
Macropus_eugenii	----------	----------	----------	----------	----------	----------	----------	509
Alligator_mississippiensis	----------	----------	----------	----------	----------	----------	----------	566
Chrysemys_picta_bellii	TTTTCAGGCA	TTACCCCACC	TCAGATACCA	AACTTAGCCC	AATTGCTGCC	TTACCCACTG	TGGATATAAC	3091
Danio_rerio	----------	----------	----------	----------	----------	----------	----------	699
Taeniopygia	----------	----------	----------	----------	----------	----------	----------	538
Dasypus_novemcinctus	----------	----------	----------	----------	----------	----------	----------	512
LTR_XT	----------	----------	----------	----------	----------	----------	----------	503
Haplorrhini	AAATACTATG	GGCCAGCTAC	GTGGGCCCAA	GATGGATCAT	GGGGCTACCG	CACCCCTA--	TTTACATGTT	8221
Catarrhini	CAATATTATG	GGCCTGCTAC	TTGGGCACAA	GACGGCTCGT	GGGGATACCG	GACCCCCA--	TTTACATGAT	7286
Anolis_carolinensis	----------	----------	----------	----------	----------	----------	----------	1184
Murine_leukemia_virus	CCCTGATATC	CACCATTATG	GGCCCTCTGA	TAGTACTTTT	ATTAATCCTA	CTCCTCGGAC	CCTGCATTCT	7463
Phascolarctos_cinereus	CTTTACTGTC	TGCCCTTGCT	GGTCCCCTGC	TACTCCTCCT	TCTGTTACTC	ACCCTCGGGC	CTTGTGTCAT	7283
Estrildidae	----------	----------	----------	----------	----------	----------	----------	482
Xenopus_Silurana_tropicalis	----------	----------	----------	----------	----------	----------	----------	812
I_XT	----------	----------	----------	----------	----------	----------	----------	7509
Takifugu_rubripes	----------	----------	----------	----------	----------	----------	----------	707
Bos_taurus	----------	----------	----------	----------	----------	----------	----------	533
Homo_sapiens	----------	----------	----------	----------	----------	----------	----------	450
Microcebus_murinus	----------	----------	----------	----------	----------	----------	----------	428
Myotis_lucifugus	----------	----------	----------	----------	----------	----------	----------	432
Equus_caballus	----------	----------	----------	----------	----------	----------	----------	450
Tupaia_belangeri	----------	----------	----------	----------	----------	----------	----------	498
Loxodonta_africana	----------	----------	----------	----------	----------	----------	----------	460
Murinae	----------	----------	----------	----------	----------	----------	----------	489
Canis_lupus_familiaris	----------	----------	----------	----------	----------	----------	----------	536
Sus_scrofa	----------	----------	----------	----------	----------	----------	----------	454
Choloepus_hoffmanni	----------	----------	----------	----------	----------	----------	----------	444
Vicugna_pacos	----------	----------	----------	----------	----------	----------	----------	474
Gavialis_gangeticus	----------	----------	----------	----------	----------	----------	----------	470
Mammalia	GACCAGTCGC	ACCTCAGCGG	NCACCAGTCC	AGGGACCAGA	CCAGACCAGC	CACTCCGCAG	CAGAGACCAG	976
Passeroidea	----------	----------	----------	----------	----------	----------	----------	641
Takifugu	----------	----------	----------	----------	----------	----------	----------	629
Pteropus_vampyrus	----------	----------	----------	----------	----------	----------	----------	472
Ornithorhynchus	TTTGCCCCTG	GCATCACCTG	CCGAGTACAG	CTGTCCCATC	TGCCGTGTTT	AATGCCCGCC	TCGGTCCCCG	2414
Oryctolagus_cuniculus	----------	----------	----------	----------	----------	----------	----------	494
Gallus_gallus	----------	----------	----------	----------	----------	----------	----------	508
Hominidae	NANCAATTTT	NNNNNGCCCN	GC-CNNNCAG	NNCCTCANTC	NCNAGNTANA	NNNANNNNNN	GCNNTCNNTN	4590
Metatheria	----------	----------	----------	----------	----------	----------	----------	569
Rodentia	GAGGGACATT	GGGNTGTTGA	CTGCCCCCAT	GTCCCTCACG	GCATGGGGAC	ATCAAGCCCA	GATCGCCCTC	2196
Monodelphis	----------	----------	----------	----------	----------	----------	----------	857
Consensus	----------	----------	----------	----------	----------	----------	----------	

Conservation

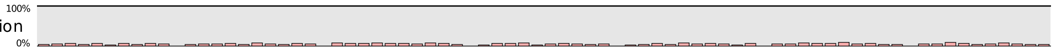

	8,340		8,360		8,380		8,400	
Dipodomys_ordii	----------	----------	----------	----------	----------	----------	----------	597
Laurasiatheria	----------	----------	----------	----------	----------	----------	----------	361
Eutheria	----------	----------	----------	----------	----------	----------	----------	452
Tarsius_syrichta	----------	----------	----------	----------	----------	----------	----------	487
Muridae	GAAAAAGGAC	ATTAAAAACA	AAGAGAAATC	TTGGCCCTCC	TAGAGGCTAT	TTGGCTTCCC	CTACAAATTG	2718
Gasterosteus_aculeatus	CTGACAGAAG	CNTNTCAACT	NGCNAAGGGA	AACGTAACTT	ACACTGATCT	AGCTGGGAGT	CATGGGGCGT	2383
Felis_silvestris	----------	----------	----------	----------	----------	----------	----------	505
Rattus	----------	----------	----------	----------	----------	----------	----------	589
Papio_hamadryas	CTTACTTGCC	CTTGTCCTCC	TACCCCGCGC	CGTACATAGC	AGTAACCCCT	CATATAGTCC	AAGATGGGAT	5686
Pan_troglodytes	ATTAGGCATG	CCTCCTCAGT	GAAGCAATTT	ACTATTCATC	CAAAACCAGT	GGAATAATAT	GCTATTGCAT	3444
Ailuropoda_melanoleuca	----------	----------	----------	----------	----------	----------	----------	462
Cavia_porcellus	----------	----------	----------	----------	----------	----------	----------	527
Ictidomys_tridecemlineatus	----------	----------	----------	----------	----------	----------	----------	530
Canis	----------	----------	----------	----------	----------	----------	----------	479
Felis_catus	----------	----------	----------	----------	----------	----------	----------	423
Ochotona_princeps	----------	----------	----------	----------	----------	----------	----------	515
Cercopithecidae	CTGCTCTGGC	CCCGGCTACC	TGATTTAGGG	CCCTCAACCC	CTATTGTCAC	AAGCCTACAC	TCAGGAGACG	1849
Carnivora	----------	----------	----------	----------	----------	----------	----------	421
Mus	AAGCATAGAA	AATCGAAATA	AGAAATTTCA	TAAGTTGGAA	TAAAACGTGT	CTAAAGCGCG	AAGCGGGAGC	1891
Mus_musculus	----------	----------	----------	----------	----------	----------	----------	478
Rattus_norvegicus	----------	----------	----------	----------	----------	----------	----------	420
Tursiops_truncatus	----------	----------	----------	----------	----------	----------	----------	518
Procavia_capensis	----------	----------	----------	----------	----------	----------	----------	442
Primates	----------	----------	----------	----------	----------	----------	----------	671
Echinops_telfairi	----------	----------	----------	----------	----------	----------	----------	473
Sorex_araneus	----------	----------	----------	----------	----------	----------	----------	494
Canidae	----------	----------	----------	----------	----------	----------	----------	550
Otolemur_garnettii	----------	----------	----------	----------	----------	----------	----------	413
Gallus	----------	----------	----------	----------	----------	----------	----------	633
Callithrix_jacchus	----------	----------	----------	----------	----------	----------	----------	495
Crocodylus_porosus	----------	----------	----------	----------	----------	----------	----------	527
Erinaceus_europaeus	----------	----------	----------	----------	----------	----------	----------	420
rnd-6_family-7426-LTR-ERV1	AGTCCGAGGA	TGAAGAGCGC	AAGAAGAAGG	TNGAGGCNAA	GAACGCTTTG	GAGAACTACG	CGTACAACAT	1452
Esox_lucius	----------	----------	----------	----------	----------	----------	----------	532
Salmo_salar	----------	----------	----------	----------	----------	----------	----------	801
Didelphidae	----------	----------	----------	----------	----------	----------	----------	642
Latimeria_chalumnae	----------	----------	----------	----------	----------	----------	----------	1766
Monodelphis_domestica	----------	----------	----------	----------	----------	----------	----------	543
Macropus_eugenii	----------	----------	----------	----------	----------	----------	----------	509
Alligator_mississippiensis	----------	----------	----------	----------	----------	----------	----------	566
Chrysemys_picta_bellii	CAACGCCTGG	AGGTCAAATT	TCATATGTTA	AACGATGAAA	CTGAGAATGT	AAAGCTGGAC	AATACTTGCA	3161
Danio_rerio	----------	----------	----------	----------	----------	----------	----------	699
Taeniopygia	----------	----------	----------	----------	----------	----------	----------	538
Dasypus_novemcinctus	----------	----------	----------	----------	----------	----------	----------	512
LTR_XT	----------	----------	----------	----------	----------	----------	----------	503
Haplorrhini	AAACCGAATT	ATAAGATTGC	AAGCAGTACT	AGAAATCATA	GTCAATGAAA	CAGCTCGAGC	CTTGGATCTG	8291
Catarrhini	CAACCGAATC	ATACGGTTAC	AAGCTGTCTT	AGAAATAATC	ACTAATAAAA	CCGGCAGAGC	CTTGACTATT	7356
Anolis_carolinensis	----------	----------	----------	----------	----------	----------	----------	1184
Murine_leukemia_virus	CAACCGCTTG	GTCCAGTTTG	TAAAAG-ACA	GAATTTCGGT	GGTGCAGGCC	CTGGTTCTGA	CCCAACAGTA	7532
Phascolarctos_cinereus	CAATAAGTTA	GTGCAATTCA	TCAATG-ATA	GGGTTAGTGC	AGTAAGGATT	CTGGTTCTCA	GGCACAAGTA	7352
Estrildidae	----------	----------	----------	----------	----------	----------	----------	482
Xenopus_Silurana_tropicalis	----------	----------	----------	----------	----------	----------	----------	812
I_XT	----------	----------	----------	----------	----------	----------	----------	7509
Takifugu_rubripes	----------	----------	----------	----------	----------	----------	----------	707
Bos_taurus	----------	----------	----------	----------	----------	----------	----------	533
Homo_sapiens	----------	----------	----------	----------	----------	----------	----------	450
Microcebus_murinus	----------	----------	----------	----------	----------	----------	----------	428
Myotis_lucifugus	----------	----------	----------	----------	----------	----------	----------	432
Equus_caballus	----------	----------	----------	----------	----------	----------	----------	450
Tupaia_belangeri	----------	----------	----------	----------	----------	----------	----------	498
Loxodonta_africana	----------	----------	----------	----------	----------	----------	----------	460
Murinae	----------	----------	----------	----------	----------	----------	----------	489
Canis_lupus_familiaris	----------	----------	----------	----------	----------	----------	----------	536
Sus_scrofa	----------	----------	----------	----------	----------	----------	----------	454
Choloepus_hoffmanni	----------	----------	----------	----------	----------	----------	----------	444
Vicugna_pacos	----------	----------	----------	----------	----------	----------	----------	474
Gavialis_gangeticus	----------	----------	----------	----------	----------	----------	----------	470
Mammalia	CGAGGATCCA	GAGGACACCG	CGTGGATCCG	AGGNCCCTTC	CCCCCNCCCN	CCACGAGGTC	ACGTAAGCCC	1046
Passeroidea	----------	----------	----------	----------	----------	----------	----------	641
Takifugu	----------	----------	----------	----------	----------	----------	----------	629
Pteropus_vampyrus	----------	----------	----------	----------	----------	----------	----------	472
Ornithorhynchus	TGGGGCCCTG	GGTTTACCCC	TTTTGAGGGC	TCCAATTGAT	TTTACCCTTC	CCGAGCTGAG	GGCTTCGCAT	2484
Oryctolagus_cuniculus	----------	----------	----------	----------	----------	----------	----------	494
Gallus_gallus	----------	----------	----------	----------	----------	----------	----------	508
Hominidae	CANCNNNCNN	CANACAAGGA	GGNCTTNNNN	GCCCNGGGCC	NCNTNNNCNN	CTNNNNNNNN	NTCNNNNANN	4660
Metatheria	----------	----------	----------	----------	----------	----------	----------	569
Rodentia	CAGCTGACCT	CCTAGGTCTG	GCCATGGACG	ACTGAGGGGG	CCCGGGCTCC	CTTGGCCTGA	CCACTGCCAT	2266
Monodelphis	----------	----------	----------	----------	----------	----------	----------	857
Consensus	----------	----------	----------	----------	----------	----------	----------	

Conservation

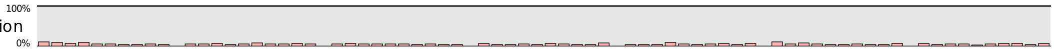

8,420 8,440 8,460

Dipodomys_ordii	----------	----------	----------	----------	----------	----------	----------	597
Laurasiatheria	----------	----------	----------	----------	----------	----------	----------	361
Eutheria	----------	----------	----------	----------	----------	----------	----------	452
Tarsius_syrichta	----------	----------	----------	----------	----------	----------	----------	487
Muridae	CTGTCCTCCA	CTGCCGAGAT	GGGCCGCTGA	CCTGGCTGCC	CGACAAACGG	CCCTAAGACC	AGTGGGGCCT	2788
Gasterosteus_aculeatus	GGAAGATTTC	TACGGG----	----------	----------	----------	----------	----------	2399
Felis_silvestris	----------	----------	----------	----------	----------	----------	----------	505
Rattus	----------	----------	----------	----------	----------	----------	----------	589
Papio_hamadryas	TCACAACAAA	GATAATCTTC	TTATACAACC	TAGTACTGGT	CTACGCGGGG	TTTGACGACC	CTCGCAAAGC	5756
Pan_troglodytes	GTAATGTAAA	CTATGTGGAA	ATGGCATTAT	TAACATCTAA	CCAATGGTGG	AGAGAGATCC	GATCCTCAAA	3514
Ailuropoda_melanoleuca	----------	----------	----------	----------	----------	----------	----------	462
Cavia_porcellus	----------	----------	----------	----------	----------	----------	----------	527
Ictidomys_tridecemlineatus	----------	----------	----------	----------	----------	----------	----------	530
Canis	----------	----------	----------	----------	----------	----------	----------	479
Felis_catus	----------	----------	----------	----------	----------	----------	----------	423
Ochotona_princeps	----------	----------	----------	----------	----------	----------	----------	515
Cercopithecidae	TGTACCCTCC	GGTGGAGGAC	CGTTCCCACC	CCACGCAATG	GTCCTGGCAC	CAAACCCCCC	CGCCTGCATA	1919
Carnivora	----------	----------	----------	----------	----------	----------	----------	421
Mus	CTGGGTACTG	GGAGATTACG	ATAACCGATA	TGGAAAATAT	TAGTTTATAG	ACACTTTCAG	GTGGGTAAGC	1961
Mus_musculus	----------	----------	----------	----------	----------	----------	----------	478
Rattus_norvegicus	----------	----------	----------	----------	----------	----------	----------	420
Tursiops_truncatus	----------	----------	----------	----------	----------	----------	----------	518
Procavia_capensis	----------	----------	----------	----------	----------	----------	----------	442
Primates	----------	----------	----------	----------	----------	----------	----------	671
Echinops_telfairi	----------	----------	----------	----------	----------	----------	----------	473
Sorex_araneus	----------	----------	----------	----------	----------	----------	----------	494
Canidae	----------	----------	----------	----------	----------	----------	----------	550
Otolemur_garnettii	----------	----------	----------	----------	----------	----------	----------	413
Gallus	----------	----------	----------	----------	----------	----------	----------	633
Callithrix_jacchus	----------	----------	----------	----------	----------	----------	----------	495
Crocodylus_porosus	----------	----------	----------	----------	----------	----------	----------	527
Erinaceus_europaeus	----------	----------	----------	----------	----------	----------	----------	420
rnd-6_family-7426-LTR-ERV1	GAGGAACACA	ATNAAGGACG	AGAAGATTGC	ATCCAAACTG	TCCGCTGATG	ANAAGGAGAA	GATTGAGGAC	1522
Esox_lucius	----------	----------	----------	----------	----------	----------	----------	532
Salmo_salar	----------	----------	----------	----------	----------	----------	----------	801
Didelphidae	----------	----------	----------	----------	----------	----------	----------	642
Latimeria_chalumnae	----------	----------	----------	----------	----------	----------	----------	1766
Monodelphis_domestica	----------	----------	----------	----------	----------	----------	----------	543
Macropus_eugenii	----------	----------	----------	----------	----------	----------	----------	509
Alligator_mississippiensis	----------	----------	----------	----------	----------	----------	----------	566
Chrysemys_picta_bellii	GTATCGTATT	TACACAATAT	GGTCTGCATT	TTAGCTGACT	CCTTATCCGT	GCTCCCCGAG	CGTAGAACAT	3231
Danio_rerio	----------	----------	----------	----------	----------	----------	----------	699
Taeniopygia	----------	----------	----------	----------	----------	----------	----------	538
Dasypus_novemcinctus	----------	----------	----------	----------	----------	----------	----------	512
LTR_XT	----------	----------	----------	----------	----------	----------	----------	503
Haplorrhini	TTAGCTATAC	AGGCCACTCA	AATGAGAGAT	GCTATATACC	AAAATAGGCT	AGCATTAGAC	TACCTCCTAG	8361
Catarrhini	CTGGCCCGGC	AAGAAACTCA	GATGAGAAAT	GCTATCTATC	AAAATAGATT	GGCTCTCGAC	TACTTGCTAG	7426
Anolis_carolinensis	----------	----------	----------	----------	----------	----------	----------	1184
Murine_leukemia_virus	TCACCAACTC	AAATCAATAG	ATCCAGAAGA	AGTGGAATCG	CGTGAATAAA	AGATTTTATT	CAGTTTCCAG	7602
Phascolarctos_cinereus	CCA--GACCC	TAGACAACGA	G----GATAA	CCTTTAATTC	TGC---TCTA	TGATTAGAGC	TACCCTCAAA	7413
Estrildidae	----------	----------	----------	----------	----------	----------	----------	482
Xenopus_Silurana_tropicalis	----------	----------	----------	----------	----------	----------	----------	812
I_XT	----------	----------	----------	----------	----------	----------	----------	7509
Takifugu_rubripes	----------	----------	----------	----------	----------	----------	----------	707
Bos_taurus	----------	----------	----------	----------	----------	----------	----------	533
Homo_sapiens	----------	----------	----------	----------	----------	----------	----------	450
Microcebus_murinus	----------	----------	----------	----------	----------	----------	----------	428
Myotis_lucifugus	----------	----------	----------	----------	----------	----------	----------	432
Equus_caballus	----------	----------	----------	----------	----------	----------	----------	450
Tupaia_belangeri	----------	----------	----------	----------	----------	----------	----------	498
Loxodonta_africana	----------	----------	----------	----------	----------	----------	----------	460
Murinae	----------	----------	----------	----------	----------	----------	----------	489
Canis_lupus_familiaris	----------	----------	----------	----------	----------	----------	----------	536
Sus_scrofa	----------	----------	----------	----------	----------	----------	----------	454
Choloepus_hoffmanni	----------	----------	----------	----------	----------	----------	----------	444
Vicugna_pacos	----------	----------	----------	----------	----------	----------	----------	474
Gavialis_gangeticus	----------	----------	----------	----------	----------	----------	----------	470
Mammalia	CCCCCCCATA	CACCCAGACG	CCATCTTGGN	GAGGAGCAGG	GGGAGGNGGA	GGAAATCTGA	AAGACTGAGC	1116
Passeroidea	----------	----------	----------	----------	----------	----------	----------	641
Takifugu	----------	----------	----------	----------	----------	----------	----------	629
Pteropus_vampyrus	----------	----------	----------	----------	----------	----------	----------	472
Ornithorhynchus	GCCACGGGTG	ATGAGGCTTC	CCGCAAGGAC	ACTGCCTGGT	TGTGTGTCTT	CTAACCTTTC	TGCTTGGTTC	2554
Oryctolagus_cuniculus	----------	----------	----------	----------	----------	----------	----------	494
Gallus_gallus	----------	----------	----------	----------	----------	----------	----------	508
Hominidae	NNNNNNCTNN	CANCNNNNNN	TNNNNNNCNT	NCNNNNGCAN	ANNCATNNNC	GNTANCTTCT	TACTNTNGTA	4730
Metatheria	----------	----------	----------	----------	----------	----------	----------	569
Rodentia	CTCCGACAGG	GAGCCTTGGG	TAGTCATCAC	GGTATCAGGG	CGATCCATCT	CCTTCCTTCT	GGACACCGGG	2336
Monodelphis	----------	----------	----------	----------	----------	----------	----------	857
Consensus	----------	----------	----------	----------	----------	----------	----------	

Conservation

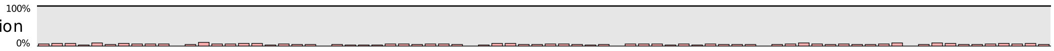

	8,480		8,500		8,520		8,540	
Dipodomys_ordii	----------	----------	----------	----------	----------	----------	----------	597
Laurasiatheria	----------	----------	----------	----------	----------	----------	----------	361
Eutheria	----------	----------	----------	----------	----------	----------	----------	452
Tarsius_syrichta	----------	----------	----------	----------	----------	----------	----------	487
Muridae	ATTGATATTC	TGATGACATA	CCCCGACCCG	GTGCTGCCTG	AGACTCCATG	GTAAAGAAGA	AATGGACAGC	2858
Gasterosteus_aculeatus	----------	----------	----------	----------	----------	----------	----------	2399
Felis_silvestris	----------	----------	----------	----------	----------	----------	----------	505
Rattus	----------	----------	----------	----------	----------	----------	----------	589
Papio_hamadryas	CATAGAACTA	GTACAAAAGC	GATATGGCCG	ACCATGCGAT	TGCAGCGGAG	GACAAGTGTC	CGAGCCCCCG	5826
Pan_troglodytes	GAGAGCCTCA	AACTGAGACC	TGGACGGACC	ATACGGCGAC	TCC--CCAAT	GCTAGCAGTA	CCATCTGGGG	3582
Ailuropoda_melanoleuca	----------	----------	----------	----------	----------	----------	----------	462
Cavia_porcellus	----------	----------	----------	----------	----------	----------	----------	527
Ictidomys_tridecemlineatus	----------	----------	----------	----------	----------	----------	----------	530
Canis	----------	----------	----------	----------	----------	----------	----------	479
Felis_catus	----------	----------	----------	----------	----------	----------	----------	423
Ochotona_princeps	----------	----------	----------	----------	----------	----------	----------	515
Cercopithecidae	CTCCCTAGGC	CAAGGTTGAC	ACCAAAGATG	AGTGCCAGGA	TTAGATTGTT	GTATCAGCCT	TTTACAGAAA	1989
Carnivora	----------	----------	----------	----------	----------	----------	----------	421
Mus	TTCCACCAGG	AACCAGTGTC	AAGAAGATTG	AAGAATCTCC	TTGGGATCCA	GTAATGTGAA	AGCCTGCTTG	2031
Mus_musculus	----------	----------	----------	----------	----------	----------	----------	478
Rattus_norvegicus	----------	----------	----------	----------	----------	----------	----------	420
Tursiops_truncatus	----------	----------	----------	----------	----------	----------	----------	518
Procavia_capensis	----------	----------	----------	----------	----------	----------	----------	442
Primates	----------	----------	----------	----------	----------	----------	----------	671
Echinops_telfairi	----------	----------	----------	----------	----------	----------	----------	473
Sorex_araneus	----------	----------	----------	----------	----------	----------	----------	494
Canidae	----------	----------	----------	----------	----------	----------	----------	550
Otolemur_garnettii	----------	----------	----------	----------	----------	----------	----------	413
Gallus	----------	----------	----------	----------	----------	----------	----------	633
Callithrix_jacchus	----------	----------	----------	----------	----------	----------	----------	495
Crocodylus_porosus	----------	----------	----------	----------	----------	----------	----------	527
Erinaceus_europaeus	----------	----------	----------	----------	----------	----------	----------	420
rnd-6_family-7426-LTR-ERV1	GCTATTGANG	AAGCTATCGA	GTGGCTNGAC	GGNAACCAAC	TCGCCGAGGC	TGATGAGTTT	GAAGACAAGN	1592
Esox_lucius	----------	----------	----------	----------	----------	----------	----------	532
Salmo_salar	----------	----------	----------	----------	----------	----------	----------	801
Didelphidae	----------	----------	----------	----------	----------	----------	----------	642
Latimeria_chalumnae	----------	----------	----------	----------	----------	----------	----------	1766
Monodelphis_domestica	----------	----------	----------	----------	----------	----------	----------	543
Macropus_eugenii	----------	----------	----------	----------	----------	----------	----------	509
Alligator_mississippiensis	----------	----------	----------	----------	----------	----------	----------	566
Chrysemys_picta_bellii	TTTGGTAAGC	TAAGTCCTTG	GGTACTGTTA	GCTCCCTCTC	GGTCTCCCAG	CCCCGAGTTT	ACCACCATAA	3301
Danio_rerio	----------	----------	----------	----------	----------	----------	----------	699
Taeniopygia	----------	----------	----------	----------	----------	----------	----------	538
Dasypus_novemcinctus	----------	----------	----------	----------	----------	----------	----------	512
LTR_XT	----------	----------	----------	----------	----------	----------	----------	503
Haplorrhini	CCTCAGAAGG	AGGAGTTTGT	GGCAAGCTTA	ATTTGACCAA	CTGTTGCTTA	CAAATCGATG	ATAATGGAAG	8431
Catarrhini	CAGCTGAAGG	AGGGGTCTGC	GGGAAATTTA	ACCTTACTAA	TTGCTGTCTA	CACATAGATG	ATCAAGGGCA	7496
Anolis_carolinensis	----------	----------	----------	----------	----------	----------	----------	1184
Murine_leukemia_virus	AAAGAGGGGG	GAA-------	----------	----------	----------	----------	----------	7615
Phascolarctos_cinereus	GAAAATGGGG	GAA-------	----------	----------	----------	----------	----------	7426
Estrildidae	----------	----------	----------	----------	----------	----------	----------	482
Xenopus_Silurana_tropicalis	----------	----------	----------	----------	----------	----------	----------	812
I_XT	----------	----------	----------	----------	----------	----------	----------	7509
Takifugu_rubripes	----------	----------	----------	----------	----------	----------	----------	707
Bos_taurus	----------	----------	----------	----------	----------	----------	----------	533
Homo_sapiens	----------	----------	----------	----------	----------	----------	----------	450
Microcebus_murinus	----------	----------	----------	----------	----------	----------	----------	428
Myotis_lucifugus	----------	----------	----------	----------	----------	----------	----------	432
Equus_caballus	----------	----------	----------	----------	----------	----------	----------	450
Tupaia_belangeri	----------	----------	----------	----------	----------	----------	----------	498
Loxodonta_africana	----------	----------	----------	----------	----------	----------	----------	460
Murinae	----------	----------	----------	----------	----------	----------	----------	489
Canis_lupus_familiaris	----------	----------	----------	----------	----------	----------	----------	536
Sus_scrofa	----------	----------	----------	----------	----------	----------	----------	454
Choloepus_hoffmanni	----------	----------	----------	----------	----------	----------	----------	444
Vicugna_pacos	----------	----------	----------	----------	----------	----------	----------	474
Gavialis_gangeticus	----------	----------	----------	----------	----------	----------	----------	470
Mammalia	ATTTACCCGA	AAGAGACTGA	GTNATCCAAA	AGAGACTATT	TAAACNGAAG	AGACTGTAGN	TANTNCTTAA	1186
Passeroidea	----------	----------	----------	----------	----------	----------	----------	641
Takifugu	----------	----------	----------	----------	----------	----------	----------	629
Pteropus_vampyrus	----------	----------	----------	----------	----------	----------	----------	472
Ornithorhynchus	CGCTCGGCTG	GATCATTCCA	GTAAAGCACT	GTTGTGGCGT	CCTGGGGCCC	GGAGGACCTG	CGCGTCCCTC	2624
Oryctolagus_cuniculus	----------	----------	----------	----------	----------	----------	----------	494
Gallus_gallus	----------	----------	----------	----------	----------	----------	----------	508
Hominidae	NANACNTTCN	CAGGNTNGAT	TNANGCCTNN	---CCNACNN	CNCNTNANAC	AGNAGANGTN	GNAGNNTCNN	4797
Metatheria	----------	----------	----------	----------	----------	----------	----------	569
Rodentia	GCCACCTACT	CAGTCCTGAC	GGAGTTTTGG	GGACCCACCT	CTCCTTGTTC	CCCTATTGTC	GGGGTAGGGG	2406
Monodelphis	----------	----------	----------	----------	----------	----------	----------	857
Consensus	----------	----------	----------	----------	----------	----------	----------	

Conservation (100% – 0%)

		8,560		8,580		8,600		
Dipodomys_ordii	----------	----------	----------	----------	----------	----------	----------	597
Laurasiatheria	----------	----------	----------	----------	----------	----------	----------	361
Eutheria	----------	----------	----------	----------	----------	----------	----------	452
Tarsius_syrichta	----------	----------	----------	----------	----------	----------	----------	487
Muridae	TCAAACCCAC	AGGTTGATGG	AGGACACCAG	AGGGAAAGAT	CATTCTCCCA	GAAACAACTG	GCAGGACTCT	2928
Gasterosteus_aculeatus	----------	----------	----------	----------	----------	----------	----------	2399
Felis_silvestris	----------	----------	----------	----------	----------	----------	----------	505
Rattus	----------	----------	----------	----------	----------	----------	----------	589
Papio_hamadryas	TCAGACAGGG	TCAGTCAAGT	GACTTGCTCA	GGCAAGACAG	CTTACTTAAT	GCCCGACCAA	AGATGGAAAT	5896
Pan_troglodytes	TCGGTAGGCC	CCGGTCATGA	TATCCAAGAA	CCCCGACAAA	GTCCTTTTTC	CGGTTAATTA	AAAAGATTCC	3652
Ailuropoda_melanoleuca	----------	----------	----------	----------	----------	----------	----------	462
Cavia_porcellus	----------	----------	----------	----------	----------	----------	----------	527
Ictidomys_tridecemlineatus	----------	----------	----------	----------	----------	----------	----------	530
Canis	----------	----------	----------	----------	----------	----------	----------	479
Felis_catus	----------	----------	----------	----------	----------	----------	----------	423
Ochotona_princeps	----------	----------	----------	----------	----------	----------	----------	515
Cercopithecidae	GTGTTGCCTC	CCAACTCGCA	ATCCCACAAA	ACCCCCGGAA	CGCTCACTGC	CCCAAACCCC	CCGGAATCCC	2059
Carnivora	----------	----------	----------	----------	----------	----------	----------	421
Mus	TCCCAGGTAA	GTCAGGATTG	CCACATGGGG	AAATTGGAAT	TAATTGTGCT	AACCCCAGAG	TCAGAGGTAG	2101
Mus_musculus	----------	----------	----------	----------	----------	----------	----------	478
Rattus_norvegicus	----------	----------	----------	----------	----------	----------	----------	420
Tursiops_truncatus	----------	----------	----------	----------	----------	----------	----------	518
Procavia_capensis	----------	----------	----------	----------	----------	----------	----------	442
Primates	----------	----------	----------	----------	----------	----------	----------	671
Echinops_telfairi	----------	----------	----------	----------	----------	----------	----------	473
Sorex_araneus	----------	----------	----------	----------	----------	----------	----------	494
Canidae	----------	----------	----------	----------	----------	----------	----------	550
Otolemur_garnettii	----------	----------	----------	----------	----------	----------	----------	413
Gallus	----------	----------	----------	----------	----------	----------	----------	633
Callithrix_jacchus	----------	----------	----------	----------	----------	----------	----------	495
Crocodylus_porosus	----------	----------	----------	----------	----------	----------	----------	527
Erinaceus_europaeus	----------	----------	----------	----------	----------	----------	----------	420
rnd-6_family-7426-LTR-ERV1	TGAAGGAGCT	NGAGGGNGTN	TGCAATCCGA	TCATCGCGAA	GATGTATCAA	GGTGGCGCTG	GTGGTGGTCT	1662
Esox_lucius	----------	----------	----------	----------	----------	----------	----------	532
Salmo_salar	----------	----------	----------	----------	----------	----------	----------	801
Didelphidae	----------	----------	----------	----------	----------	----------	----------	642
Latimeria_chalumnae	----------	----------	----------	----------	----------	----------	----------	1766
Monodelphis_domestica	----------	----------	----------	----------	----------	----------	----------	543
Macropus_eugenii	----------	----------	----------	----------	----------	----------	----------	509
Alligator_mississippiensis	----------	----------	----------	----------	----------	----------	----------	566
Chrysemys_picta_bellii	AGGAAAGGTT	TGGAGATCCC	CATTGACACA	TAAAGCTACA	CCTGGCCAAG	AAAGTAGTGG	CCGACGCAGG	3371
Danio_rerio	----------	----------	----------	----------	----------	----------	----------	699
Taeniopygia	----------	----------	----------	----------	----------	----------	----------	538
Dasypus_novemcinctus	----------	----------	----------	----------	----------	----------	----------	512
LTR_XT	----------	----------	----------	----------	----------	----------	----------	503
Haplorrhini	AGCTGTTATG	GAAATTACTG	CTAGAATGCG	GAAGTTAGCC	CATGTCCCAG	TCCAGACTTG	GTCCNGGTGG	8501
Catarrhini	AGTAGTTGAA	GACATAGTTA	GAGATATGAC	AAAACTGGCA	CACGTGCCCG	TGCAAGTGTG	GCATGGATTT	7566
Anolis_carolinensis	----------	----------	----------	----------	----------	----------	----------	1184
Murine_leukemia_virus	----------	----------	----------	----------	----------	----------	----------	7615
Phascolarctos_cinereus	----------	----------	----------	----------	----------	----------	----------	7426
Estrildidae	----------	----------	----------	----------	----------	----------	----------	482
Xenopus_Silurana_tropicalis	----------	----------	----------	----------	----------	----------	----------	812
I_XT	----------	----------	----------	----------	----------	----------	----------	7509
Takifugu_rubripes	----------	----------	----------	----------	----------	----------	----------	707
Bos_taurus	----------	----------	----------	----------	----------	----------	----------	533
Homo_sapiens	----------	----------	----------	----------	----------	----------	----------	450
Microcebus_murinus	----------	----------	----------	----------	----------	----------	----------	428
Myotis_lucifugus	----------	----------	----------	----------	----------	----------	----------	432
Equus_caballus	----------	----------	----------	----------	----------	----------	----------	450
Tupaia_belangeri	----------	----------	----------	----------	----------	----------	----------	498
Loxodonta_africana	----------	----------	----------	----------	----------	----------	----------	460
Murinae	----------	----------	----------	----------	----------	----------	----------	489
Canis_lupus_familiaris	----------	----------	----------	----------	----------	----------	----------	536
Sus_scrofa	----------	----------	----------	----------	----------	----------	----------	454
Choloepus_hoffmanni	----------	----------	----------	----------	----------	----------	----------	444
Vicugna_pacos	----------	----------	----------	----------	----------	----------	----------	474
Gavialis_gangeticus	----------	----------	----------	----------	----------	----------	----------	470
Mammalia	TTGGCAAGTT	TTAAAATTTN	NCCCTCNTTN	TCCCCNCTAC	CCAGCNGGGT	GGGGGCTCGT	GAGGAAGATT	1256
Passeroidea	----------	----------	----------	----------	----------	----------	----------	641
Takifugu	----------	----------	----------	----------	----------	----------	----------	629
Pteropus_vampyrus	----------	----------	----------	----------	----------	----------	----------	472
Ornithorhynchus	CCACCAGCTG	CGGTTGCTGC	CGAGGCCGGT	CACTTACCTC	ATCCCGTTGT	GGTGCGTCCT	CTGTGGTAAT	2694
Oryctolagus_cuniculus	----------	----------	----------	----------	----------	----------	----------	494
Gallus_gallus	----------	----------	----------	----------	----------	----------	----------	508
Hominidae	NCCTNNTNGA	ACANATNATC	CCNNGNTTTG	GACTTCCCCG	ATCCCTGCAA	TCAGACAACG	GCCCNGCNTT	4867
Metatheria	----------	----------	----------	----------	----------	----------	----------	569
Rodentia	GACAGCCTTA	CCAGCCTCAC	CAAACCCCAC	CACTTAGCTG	TATCCCCCTT	ACCCACTCCT	TCCTAGTGGT	2476
Monodelphis	----------	----------	----------	----------	----------	----------	----------	857
Consensus	----------	----------	----------	----------	----------	----------	----------	

Conservation

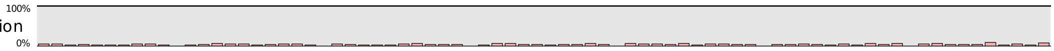

	8,620		8,640		8,660		8,680	
Dipodomys_ordii	----------	----------	----------	----------	----------	----------	----------	597
Laurasiatheria	----------	----------	----------	----------	----------	----------	----------	361
Eutheria	----------	----------	----------	----------	----------	----------	----------	452
Tarsius_syrichta	----------	----------	----------	----------	----------	----------	----------	487
Muridae	GGTAACTGAC	CTTCACCAGG	CTACTCATCT	GGGGTCCACA	AAACTTTCTG	AGTTGCTCAG	ACCAAGGTAT	2998
Gasterosteus_aculeatus	----------	----------	----------	----------	----------	----------	----------	2399
Felis_silvestris	----------	----------	----------	----------	----------	----------	----------	505
Rattus	----------	----------	----------	----------	----------	----------	----------	589
Papio_hamadryas	GTAAGTCAAT	TCCAAAAGAC	ACCTCCCCAA	GCGGGCCACT	CCAAGAGTGC	CCCTGTAATT	CTTACCAGTC	5966
Pan_troglodytes	ACCGGTGCAC	TC------GT	CCCTGCCCCT	GCTGGGGACA	CCACCTCCCA	AACCACAAAC	CTCTGCCCAT	3716
Ailuropoda_melanoleuca	----------	----------	----------	----------	----------	----------	----------	462
Cavia_porcellus	----------	----------	----------	----------	----------	----------	----------	527
Ictidomys_tridecemlineatus	----------	----------	----------	----------	----------	----------	----------	530
Canis	----------	----------	----------	----------	----------	----------	----------	479
Felis_catus	----------	----------	----------	----------	----------	----------	----------	423
Ochotona_princeps	----------	----------	----------	----------	----------	----------	----------	515
Cercopithecidae	CAACTCCCCC	TCTCCTCCCC	CGCGGTCTGT	TGGGGGGCCT	ACACAATCAT	CTGATATCTC	CTATACTCTC	2129
Carnivora	----------	----------	----------	----------	----------	----------	----------	421
Mus	AGATGAAAGA	CCTAAAGAAC	CTACAAATGC	TTGACCGGGA	GATGGCCTCT	CCTTGTTGGC	AAACCCGGTT	2171
Mus_musculus	----------	----------	----------	----------	----------	----------	----------	478
Rattus_norvegicus	----------	----------	----------	----------	----------	----------	----------	420
Tursiops_truncatus	----------	----------	----------	----------	----------	----------	----------	518
Procavia_capensis	----------	----------	----------	----------	----------	----------	----------	442
Primates	----------	----------	----------	----------	----------	----------	----------	671
Echinops_telfairi	----------	----------	----------	----------	----------	----------	----------	473
Sorex_araneus	----------	----------	----------	----------	----------	----------	----------	494
Canidae	----------	----------	----------	----------	----------	----------	----------	550
Otolemur_garnettii	----------	----------	----------	----------	----------	----------	----------	413
Gallus	----------	----------	----------	----------	----------	----------	----------	633
Callithrix_jacchus	----------	----------	----------	----------	----------	----------	----------	495
Crocodylus_porosus	----------	----------	----------	----------	----------	----------	----------	527
Erinaceus_europaeus	----------	----------	----------	----------	----------	----------	----------	420
rnd-6_family-7426-LTR-ERV1	ATGGANGAAG	AAGGTCCTGC	TCCTAGTGGT	----------	----------	----------	----------	1692
Esox_lucius	----------	----------	----------	----------	----------	----------	----------	532
Salmo_salar	----------	----------	----------	----------	----------	----------	----------	801
Didelphidae	----------	----------	----------	----------	----------	----------	----------	642
Latimeria_chalumnae	----------	----------	----------	----------	----------	----------	----------	1766
Monodelphis_domestica	----------	----------	----------	----------	----------	----------	----------	543
Macropus_eugenii	----------	----------	----------	----------	----------	----------	----------	509
Alligator_mississippiensis	----------	----------	----------	----------	----------	----------	----------	566
Chrysemys_picta_bellii	CTATGTTGCC	GGGGGTGTGC	CTATTGAAAG	AATGTTGTTT	TCCGACAGAT	TAAACACCAC	ACACCCAGAA	3441
Danio_rerio	----------	----------	----------	----------	----------	----------	----------	699
Taeniopygia	----------	----------	----------	----------	----------	----------	----------	538
Dasypus_novemcinctus	----------	----------	----------	----------	----------	----------	----------	512
LTR_XT	----------	----------	----------	----------	----------	----------	----------	503
Haplorrhini	AGCCCAAACT	CACTTTTTGG	AGGATGGTTC	TCATGGTTTG	GAGGCTTTAA	AACTTTGATA	ATTGGTTTTA	8571
Catarrhini	GATCCTGGGG	CCATGTTTGG	AAAATGGTTC	CCAGCGCTAG	GAGGATTTAA	AACTCTTATA	ATAGGAGTTA	7636
Anolis_carolinensis	----------	----------	----------	----------	----------	----------	----------	1184
Murine_leukemia_virus	----------	----------	----------	----------	----------	----------	----------	7615
Phascolarctos_cinereus	----------	----------	----------	----------	----------	----------	----------	7426
Estrildidae	----------	----------	----------	----------	----------	----------	----------	482
Xenopus_Silurana_tropicalis	----------	----------	----------	----------	----------	----------	----------	812
I_XT	----------	----------	----------	----------	----------	----------	----------	7509
Takifugu_rubripes	----------	----------	----------	----------	----------	----------	----------	707
Bos_taurus	----------	----------	----------	----------	----------	----------	----------	533
Homo_sapiens	----------	----------	----------	----------	----------	----------	----------	450
Microcebus_murinus	----------	----------	----------	----------	----------	----------	----------	428
Myotis_lucifugus	----------	----------	----------	----------	----------	----------	----------	432
Equus_caballus	----------	----------	----------	----------	----------	----------	----------	450
Tupaia_belangeri	----------	----------	----------	----------	----------	----------	----------	498
Loxodonta_africana	----------	----------	----------	----------	----------	----------	----------	460
Murinae	----------	----------	----------	----------	----------	----------	----------	489
Canis_lupus_familiaris	----------	----------	----------	----------	----------	----------	----------	536
Sus_scrofa	----------	----------	----------	----------	----------	----------	----------	454
Choloepus_hoffmanni	----------	----------	----------	----------	----------	----------	----------	444
Vicugna_pacos	----------	----------	----------	----------	----------	----------	----------	474
Gavialis_gangeticus	----------	----------	----------	----------	----------	----------	----------	470
Mammalia	AGATCAGTTA	TAGAAAATAA	AGAAGCTACA	TTTTCTTTGC	ACATCTGAGT	GTAGTGTGAG	TAAATTTGCG	1326
Passeroidea	----------	----------	----------	----------	----------	----------	----------	641
Takifugu	----------	----------	----------	----------	----------	----------	----------	629
Pteropus_vampyrus	----------	----------	----------	----------	----------	----------	----------	472
Ornithorhynchus	TTGTTTCCTG	TTGGTCCTCC	GCTGGGTGGT	TACAGTTCCG	TGCTCACTCG	GATAAACCGG	GCCTGGTGCT	2764
Oryctolagus_cuniculus	----------	----------	----------	----------	----------	----------	----------	494
Gallus_gallus	----------	----------	----------	----------	----------	----------	----------	508
Hominidae	NNTCTCNAAA	ATNNNNNANC	ANGTNNCNNN	NN--CNCTNN	GCGTNANNTG	GAAGCTNCAN	ANNCCNTACC	4935
Metatheria	----------	----------	----------	----------	----------	----------	----------	569
Rodentia	ACCAACCTGT	CCCATCCCCT	TGCTGGGAAG	GGACCTCCTA	GCCAAAGTGG	GAGCCTCTAT	TTCCTTTGCT	2546
Monodelphis	----------	----------	----------	----------	----------	----------	----------	857
Consensus	----------	----------	----------	----------	----------	----------	----------	

Conservation

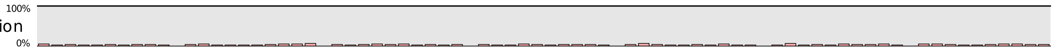

8,700 8,720 8,740

Dipodomys_ordii	----------	----------	----------	----------	----------	----------	----------	597
Laurasiatheria	----------	----------	----------	----------	----------	----------	----------	361
Eutheria	----------	----------	----------	----------	----------	----------	----------	452
Tarsius_syrichta	----------	----------	----------	----------	----------	----------	----------	487
Muridae	GTTATACCTA	GACTGGACAG	CCTAGCGCAG	GATGTCTCAG	CCAGATGCCA	GGTTTGTGCT	CAAGTGAATC	3068
Gasterosteus_aculeatus	----------	----------	----------	----------	----------	----------	----------	2399
Felis_silvestris	----------	----------	----------	----------	----------	----------	----------	505
Rattus	----------	----------	----------	----------	----------	----------	----------	589
Papio_hamadryas	CTCAGTACAC	AGTTCTTGTT	ATACCTCATA	CCAACAATGC	AGATCAGGCA	ATAAGACATA	TTATACGGCT	6036
Pan_troglodytes	CACGCTTCCT	AAACCCCAGC	AGAAAGCCTA	CTGTTGGATC	AGCCTCAACA	TAGAGCCCTA	TTACTCCAGC	3786
Ailuropoda_melanoleuca	----------	----------	----------	----------	----------	----------	----------	462
Cavia_porcellus	----------	----------	----------	----------	----------	----------	----------	527
Ictidomys_tridecemlineatus	----------	----------	----------	----------	----------	----------	----------	530
Canis	----------	----------	----------	----------	----------	----------	----------	479
Felis_catus	----------	----------	----------	----------	----------	----------	----------	423
Ochotona_princeps	----------	----------	----------	----------	----------	----------	----------	515
Cercopithecidae	AGTCAAACGG	GTGATTAAGG	GGTGCTAAGA	ATGGTTAATA	TCAGGTGTAA	CAAGTGGTCC	GTCCTTGCCC	2199
Carnivora	----------	----------	----------	----------	----------	----------	----------	421
Mus	ACCCTTGATT	TGGGGCCCCC	CTGCGGTAAT	GTGTCTCTCT	TTCCTAAAGC	TGATGTAGAA	CTGGGACATA	2241
Mus_musculus	----------	----------	----------	----------	----------	----------	----------	478
Rattus_norvegicus	----------	----------	----------	----------	----------	----------	----------	420
Tursiops_truncatus	----------	----------	----------	----------	----------	----------	----------	518
Procavia_capensis	----------	----------	----------	----------	----------	----------	----------	442
Primates	----------	----------	----------	----------	----------	----------	----------	671
Echinops_telfairi	----------	----------	----------	----------	----------	----------	----------	473
Sorex_araneus	----------	----------	----------	----------	----------	----------	----------	494
Canidae	----------	----------	----------	----------	----------	----------	----------	550
Otolemur_garnettii	----------	----------	----------	----------	----------	----------	----------	413
Gallus	----------	----------	----------	----------	----------	----------	----------	633
Callithrix_jacchus	----------	----------	----------	----------	----------	----------	----------	495
Crocodylus_porosus	----------	----------	----------	----------	----------	----------	----------	527
Erinaceus_europaeus	----------	----------	----------	----------	----------	----------	----------	420
rnd-6_family-7426-LTR-ERV1	----------	----------	----------	----------	----------	----------	----------	1692
Esox_lucius	----------	----------	----------	----------	----------	----------	----------	532
Salmo_salar	----------	----------	----------	----------	----------	----------	----------	801
Didelphidae	----------	----------	----------	----------	----------	----------	----------	642
Latimeria_chalumnae	----------	----------	----------	----------	----------	----------	----------	1766
Monodelphis_domestica	----------	----------	----------	----------	----------	----------	----------	543
Macropus_eugenii	----------	----------	----------	----------	----------	----------	----------	509
Alligator_mississippiensis	----------	----------	----------	----------	----------	----------	----------	566
Chrysemys_picta_bellii	GGCCATTTTG	TTGGTGTTCC	CGACTGGGGG	GTCTCTGGTT	TGTTGTTCTG	TCCTGTTAGG	TATTTCCCCC	3511
Danio_rerio	----------	----------	----------	----------	----------	----------	----------	699
Taeniopygia	----------	----------	----------	----------	----------	----------	----------	538
Dasypus_novemcinctus	----------	----------	----------	----------	----------	----------	----------	512
LTR_XT	----------	----------	----------	----------	----------	----------	----------	503
Haplorrhini	TAGCTATAAT	TGGAGGATGT	CTAATACTGC	CTTGTCTTTT	ACCTCTCCTC	ATCAGAAGCA	TCCAGTCTAC	8641
Catarrhini	TAATAGTAAT	AGGAACCTGC	TTACTGCTCC	CTTGTTTGCT	ACCTGTACTT	CTTCAAATGA	TAAAAAGCTT	7706
Anolis_carolinensis	----------	----------	----------	----------	----------	----------	----------	1184
Murine_leukemia_virus	----------	----------	----------	----------	----------	----------	----------	7615
Phascolarctos_cinereus	----------	----------	----------	----------	----------	----------	----------	7426
Estrildidae	----------	----------	----------	----------	----------	----------	----------	482
Xenopus_Silurana_tropicalis	----------	----------	----------	----------	----------	----------	----------	812
I_XT	----------	----------	----------	----------	----------	----------	----------	7509
Takifugu_rubripes	----------	----------	----------	----------	----------	----------	----------	707
Bos_taurus	----------	----------	----------	----------	----------	----------	----------	533
Homo_sapiens	----------	----------	----------	----------	----------	----------	----------	450
Microcebus_murinus	----------	----------	----------	----------	----------	----------	----------	428
Myotis_lucifugus	----------	----------	----------	----------	----------	----------	----------	432
Equus_caballus	----------	----------	----------	----------	----------	----------	----------	450
Tupaia_belangeri	----------	----------	----------	----------	----------	----------	----------	498
Loxodonta_africana	----------	----------	----------	----------	----------	----------	----------	460
Murinae	----------	----------	----------	----------	----------	----------	----------	489
Canis_lupus_familiaris	----------	----------	----------	----------	----------	----------	----------	536
Sus_scrofa	----------	----------	----------	----------	----------	----------	----------	454
Choloepus_hoffmanni	----------	----------	----------	----------	----------	----------	----------	444
Vicugna_pacos	----------	----------	----------	----------	----------	----------	----------	474
Gavialis_gangeticus	----------	----------	----------	----------	----------	----------	----------	470
Mammalia	ACCCCGCTAC	A---------	----------	----------	----------	----------	----------	1337
Passeroidea	----------	----------	----------	----------	----------	----------	----------	641
Takifugu	----------	----------	----------	----------	----------	----------	----------	629
Pteropus_vampyrus	----------	----------	----------	----------	----------	----------	----------	472
Ornithorhynchus	CGGGGCCCAG	GACCGGGGGA	C---------	----------	----------	----------	----------	2785
Oryctolagus_cuniculus	----------	----------	----------	----------	----------	----------	----------	494
Gallus_gallus	----------	----------	----------	----------	----------	----------	----------	508
Hominidae	NNCCNCANTC	NTCTGGN---	AAAGTNGAAN	GNGCNAACNG	CCTNNTCAAA	NAACACCTNA	NNAAANTNNC	5002
Metatheria	----------	----------	----------	----------	----------	----------	----------	569
Rodentia	CCCCCCATCC	GCTTGACCCC	AGGCTCGCCA	GCACCNCTTC	CCCTTCCCTC	CTCCTCCTCC	AAACCACACA	2616
Monodelphis	----------	----------	----------	----------	----------	----------	----------	857
Consensus	----------	----------	----------	----------	----------	----------	----------	

Conservation

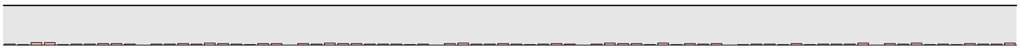

	8,760		8,780		8,800		8,820	
Dipodomys_ordii	----------	----------	----------	----------	----------	----------	----------	597
Laurasiatheria	----------	----------	----------	----------	----------	----------	----------	361
Eutheria	----------	----------	----------	----------	----------	----------	----------	452
Tarsius_syrichta	----------	----------	----------	----------	----------	----------	----------	487
Muridae	CAAAACAGAG	AGCCCTTACC	CAGGAAGGGG	TCCAAATGAG	AGACCAACAC	CCTGGCAAAC	TCTGGGAAAA	3138
Gasterosteus_aculeatus	----------	----------	----------	----------	----------	----------	----------	2399
Felis_silvestris	----------	----------	----------	----------	----------	----------	----------	505
Rattus	----------	----------	----------	----------	----------	----------	----------	589
Papio_hamadryas	ACTCTGCTAA	AAACACAAAC	TGGGGGCACC	AGTGATGTAC	AA-GTATTAG	GATCCACCAA	CAAACTTATA	6105
Pan_troglodytes	ATTCTGTGAG	GACAACAAAA	CTACAGCGTA	ACCTGTGCCC	GTCACAAAAA	AAATCCCATG	GATTCCCTCA	3856
Ailuropoda_melanoleuca	----------	----------	----------	----------	----------	----------	----------	462
Cavia_porcellus	----------	----------	----------	----------	----------	----------	----------	527
Ictidomys_tridecemlineatus	----------	----------	----------	----------	----------	----------	----------	530
Canis	----------	----------	----------	----------	----------	----------	----------	479
Felis_catus	----------	----------	----------	----------	----------	----------	----------	423
Ochotona_princeps	----------	----------	----------	----------	----------	----------	----------	515
Cercopithecidae	TTTTTTTGCC	TGTTTTAATT	AATGTCTACC	AAAAGATGTG	GGAA------	----------	----------	2243
Carnivora	----------	----------	----------	----------	----------	----------	----------	421
Mus	AAGACTACGG	GTGCCCAGTT	AGTGGGACCG	TCTGTGCACG	AGGGACGACC	GTGGAAGGCC	TATGTCTTGA	2311
Mus_musculus	----------	----------	----------	----------	----------	----------	----------	478
Rattus_norvegicus	----------	----------	----------	----------	----------	----------	----------	420
Tursiops_truncatus	----------	----------	----------	----------	----------	----------	----------	518
Procavia_capensis	----------	----------	----------	----------	----------	----------	----------	442
Primates	----------	----------	----------	----------	----------	----------	----------	671
Echinops_telfairi	----------	----------	----------	----------	----------	----------	----------	473
Sorex_araneus	----------	----------	----------	----------	----------	----------	----------	494
Canidae	----------	----------	----------	----------	----------	----------	----------	550
Otolemur_garnettii	----------	----------	----------	----------	----------	----------	----------	413
Gallus	----------	----------	----------	----------	----------	----------	----------	633
Callithrix_jacchus	----------	----------	----------	----------	----------	----------	----------	495
Crocodylus_porosus	----------	----------	----------	----------	----------	----------	----------	527
Erinaceus_europaeus	----------	----------	----------	----------	----------	----------	----------	420
rnd-6_family-7426-LTR-ERV1	----------	----------	----------	----------	----------	----------	----------	1692
Esox_lucius	----------	----------	----------	----------	----------	----------	----------	532
Salmo_salar	----------	----------	----------	----------	----------	----------	----------	801
Didelphidae	----------	----------	----------	----------	----------	----------	----------	642
Latimeria_chalumnae	----------	----------	----------	----------	----------	----------	----------	1766
Monodelphis_domestica	----------	----------	----------	----------	----------	----------	----------	543
Macropus_eugenii	----------	----------	----------	----------	----------	----------	----------	509
Alligator_mississippiensis	----------	----------	----------	----------	----------	----------	----------	566
Chrysemys_picta_bellii	TCCCAATTAT	TCATAATCCT	GCAGGAGTAA	ACATTTTGTC	TAGAAATCGC	AATGTGATGC	AAAGAGG---	3578
Danio_rerio	----------	----------	----------	----------	----------	----------	----------	699
Taeniopygia	----------	----------	----------	----------	----------	----------	----------	538
Dasypus_novemcinctus	----------	----------	----------	----------	----------	----------	----------	512
LTR_XT	----------	----------	----------	----------	----------	----------	----------	503
Haplorrhini	TATTGAAGCA	ATAGTGGACC	GAACAACTAC	CACCAAAATA	ATGGCACTAC	AAAAATACCA	ACCAGTCC-C	8710
Catarrhini	CATCGCTACC	TTAGTTCACC	AAAATGCT--	--TCAGCACA	AGTGTACTAT	ATGAATCACT	ATCGATCTGT	7772
Anolis_carolinensis	----------	----------	----------	----------	----------	----------	----------	1184
Murine_leukemia_virus	----------	----------	----------	----------	----------	----------	----------	7615
Phascolarctos_cinereus	----------	----------	----------	----------	----------	----------	----------	7426
Estrildidae	----------	----------	----------	----------	----------	----------	----------	482
Xenopus_Silurana_tropicalis	----------	----------	----------	----------	----------	----------	----------	812
I_XT	----------	----------	----------	----------	----------	----------	----------	7509
Takifugu_rubripes	----------	----------	----------	----------	----------	----------	----------	707
Bos_taurus	----------	----------	----------	----------	----------	----------	----------	533
Homo_sapiens	----------	----------	----------	----------	----------	----------	----------	450
Microcebus_murinus	----------	----------	----------	----------	----------	----------	----------	428
Myotis_lucifugus	----------	----------	----------	----------	----------	----------	----------	432
Equus_caballus	----------	----------	----------	----------	----------	----------	----------	450
Tupaia_belangeri	----------	----------	----------	----------	----------	----------	----------	498
Loxodonta_africana	----------	----------	----------	----------	----------	----------	----------	460
Murinae	----------	----------	----------	----------	----------	----------	----------	489
Canis_lupus_familiaris	----------	----------	----------	----------	----------	----------	----------	536
Sus_scrofa	----------	----------	----------	----------	----------	----------	----------	454
Choloepus_hoffmanni	----------	----------	----------	----------	----------	----------	----------	444
Vicugna_pacos	----------	----------	----------	----------	----------	----------	----------	474
Gavialis_gangeticus	----------	----------	----------	----------	----------	----------	----------	470
Mammalia	----------	----------	----------	----------	----------	----------	----------	1337
Passeroidea	----------	----------	----------	----------	----------	----------	----------	641
Takifugu	----------	----------	----------	----------	----------	----------	----------	629
Pteropus_vampyrus	----------	----------	----------	----------	----------	----------	----------	472
Ornithorhynchus	----------	----------	----------	----------	----------	----------	----------	2785
Oryctolagus_cuniculus	----------	----------	----------	----------	----------	----------	----------	494
Gallus_gallus	----------	----------	----------	----------	----------	----------	----------	508
Hominidae	TCTNGANACN	NNNCNNTCGT	GGGTNACCNT	NCTNCCNTNN	GCCCTNNCNC	GNCTCCGGGC	AGCNCCCNGN	5072
Metatheria	----------	----------	----------	----------	----------	----------	----------	569
Rodentia	ATCTGACACA	CCTTTTCCCT	TGCCAGCCTC	CTCCGGGGAT	CCCCAGGTAT	GGGACCCCAA	ACCCCTCTAT	2686
Monodelphis	----------	----------	----------	----------	----------	----------	----------	857
Consensus	----------	----------	----------	----------	----------	----------	----------	

Conservation (100% / 0%)

8,840 | 8,860 | 8,880

Dipodomys_ordii	----------	----------	----------	----------	----------	----------	----------	597
Laurasiatheria	----------	----------	----------	----------	----------	----------	----------	361
Eutheria	----------	----------	----------	----------	----------	----------	----------	452
Tarsius_syrichta	----------	----------	----------	----------	----------	----------	----------	487
Muridae	CTGACTTCAC	CAAGATCTGG	CCTGCCAGGG	GAGGATAAAA	GTACTTGCTA	GTTTTTATAG	ATACCTTTTC	3208
Gasterosteus_aculeatus	----------	----------	----------	----------	----------	----------	----------	2399
Felis_silvestris	----------	----------	----------	----------	----------	----------	----------	505
Rattus	----------	----------	----------	----------	----------	----------	----------	589
Papio_hamadryas	CAATCTCCCT	GTAATGGCAT	AAAAGGGCAG	TCTATTTGCT	GGAGCACTAC	AGCTCCTATC	CACGTCTCTG	6175
Pan_troglodytes	CCCTATGGGA	GGCTGGGAGG	CCTAGGTCAA	CCACAATGGC	TGAGATCAGG	ATCTTCCTAC	TACTCCCTCA	3926
Ailuropoda_melanoleuca	----------	----------	----------	----------	----------	----------	----------	462
Cavia_porcellus	----------	----------	----------	----------	----------	----------	----------	527
Ictidomys_tridecemlineatus	----------	----------	----------	----------	----------	----------	----------	530
Canis	----------	----------	----------	----------	----------	----------	----------	479
Felis_catus	----------	----------	----------	----------	----------	----------	----------	423
Ochotona_princeps	----------	----------	----------	----------	----------	----------	----------	515
Cercopithecidae	----------	----------	----------	----------	----------	----------	----------	2243
Carnivora	----------	----------	----------	----------	----------	----------	----------	421
Mus	CACGGAAGTG	AATCCTGGTC	CACGCTCCCA	CTAAGCCCAC	AGATTGACCT	TAGGCCTTCA	CACCTGGCTG	2381
Mus_musculus	----------	----------	----------	----------	----------	----------	----------	478
Rattus_norvegicus	----------	----------	----------	----------	----------	----------	----------	420
Tursiops_truncatus	----------	----------	----------	----------	----------	----------	----------	518
Procavia_capensis	----------	----------	----------	----------	----------	----------	----------	442
Primates	----------	----------	----------	----------	----------	----------	----------	671
Echinops_telfairi	----------	----------	----------	----------	----------	----------	----------	473
Sorex_araneus	----------	----------	----------	----------	----------	----------	----------	494
Canidae	----------	----------	----------	----------	----------	----------	----------	550
Otolemur_garnettii	----------	----------	----------	----------	----------	----------	----------	413
Gallus	----------	----------	----------	----------	----------	----------	----------	633
Callithrix_jacchus	----------	----------	----------	----------	----------	----------	----------	495
Crocodylus_porosus	----------	----------	----------	----------	----------	----------	----------	527
Erinaceus_europaeus	----------	----------	----------	----------	----------	----------	----------	420
rnd-6_family-7426-LTR-ERV1	----------	----------	----------	----------	----------	----------	----------	1692
Esox_lucius	----------	----------	----------	----------	----------	----------	----------	532
Salmo_salar	----------	----------	----------	----------	----------	----------	----------	801
Didelphidae	----------	----------	----------	----------	----------	----------	----------	642
Latimeria_chalumnae	----------	----------	----------	----------	----------	----------	----------	1766
Monodelphis_domestica	----------	----------	----------	----------	----------	----------	----------	543
Macropus_eugenii	----------	----------	----------	----------	----------	----------	----------	509
Alligator_mississippiensis	----------	----------	----------	----------	----------	----------	----------	566
Chrysemys_picta_bellii	----------	----------	----------	----------	----------	----------	----------	3578
Danio_rerio	----------	----------	----------	----------	----------	----------	----------	699
Taeniopygia	----------	----------	----------	----------	----------	----------	----------	538
Dasypus_novemcinctus	----------	----------	----------	----------	----------	----------	----------	512
LTR_XT	----------	----------	----------	----------	----------	----------	----------	503
Haplorrhini	CCAGGAAGAA	TATGTGCCTA	CACAGGAAGA	GATAAACGAT	TGTGGTGCTC	TTTATTAATC	TACATTTATG	8780
Catarrhini	CTTGCAAGAA	GACATGGGTA	GTGAGAATGA	AAGTGAGAAC	T------CCC	ACTATTGAGT	GAGATTC---	7833
Anolis_carolinensis	----------	----------	----------	----------	----------	----------	----------	1184
Murine_leukemia_virus	----------	----------	----------	----------	----------	----------	----------	7615
Phascolarctos_cinereus	----------	----------	----------	----------	----------	----------	----------	7426
Estrildidae	----------	----------	----------	----------	----------	----------	----------	482
Xenopus_Silurana_tropicalis	----------	----------	----------	----------	----------	----------	----------	812
I_XT	----------	----------	----------	----------	----------	----------	----------	7509
Takifugu_rubripes	----------	----------	----------	----------	----------	----------	----------	707
Bos_taurus	----------	----------	----------	----------	----------	----------	----------	533
Homo_sapiens	----------	----------	----------	----------	----------	----------	----------	450
Microcebus_murinus	----------	----------	----------	----------	----------	----------	----------	428
Myotis_lucifugus	----------	----------	----------	----------	----------	----------	----------	432
Equus_caballus	----------	----------	----------	----------	----------	----------	----------	450
Tupaia_belangeri	----------	----------	----------	----------	----------	----------	----------	498
Loxodonta_africana	----------	----------	----------	----------	----------	----------	----------	460
Murinae	----------	----------	----------	----------	----------	----------	----------	489
Canis_lupus_familiaris	----------	----------	----------	----------	----------	----------	----------	536
Sus_scrofa	----------	----------	----------	----------	----------	----------	----------	454
Choloepus_hoffmanni	----------	----------	----------	----------	----------	----------	----------	444
Vicugna_pacos	----------	----------	----------	----------	----------	----------	----------	474
Gavialis_gangeticus	----------	----------	----------	----------	----------	----------	----------	470
Mammalia	----------	----------	----------	----------	----------	----------	----------	1337
Passeroidea	----------	----------	----------	----------	----------	----------	----------	641
Takifugu	----------	----------	----------	----------	----------	----------	----------	629
Pteropus_vampyrus	----------	----------	----------	----------	----------	----------	----------	472
Ornithorhynchus	----------	----------	----------	----------	----------	----------	----------	2785
Oryctolagus_cuniculus	----------	----------	----------	----------	----------	----------	----------	494
Gallus_gallus	----------	----------	----------	----------	----------	----------	----------	508
Hominidae	NGCCCCACAG	GNCTNAGCCC	CTTNGAACTN	CTNTACGGNC	GCCCCTTCCT	NNTTCNNGNN	CTNCCNNNNA	5142
Metatheria	----------	----------	----------	----------	----------	----------	----------	569
Rodentia	AGCCAAACA-	---TCACCCC	CCTCTCATCA	TCCAGTGGAC	GGCCAGTCCT	GNCTCCTGGC	CCATGTCTCA	2752
Monodelphis	----------	----------	----------	----------	----------	----------	----------	857
Consensus	----------	----------	----------	----------	----------	----------	----------	

Conservation

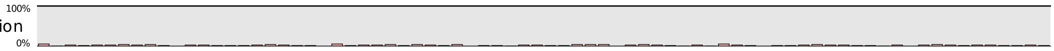

	8,900		8,920		8,940		8,960	
Dipodomys_ordii	----------	----------	----------	----------	----------	----------	----------	597
Laurasiatheria	----------	----------	----------	----------	----------	----------	----------	361
Eutheria	----------	----------	----------	----------	----------	----------	----------	452
Tarsius_syrichta	----------	----------	----------	----------	----------	----------	----------	487
Muridae	CAGGTGGGTA	GAAGCGTTCC	CCACCCGGAC	TGAAACTGCT	CAAATAGTAG	CTAAAAAGCT	CCTCCAGGAA	3278
Gasterosteus_aculeatus	----------	----------	----------	----------	----------	----------	----------	2399
Felis_silvestris	----------	----------	----------	----------	----------	----------	----------	505
Rattus	----------	----------	----------	----------	----------	----------	----------	589
Papio_hamadryas	ATGGAGGAGG	TCCATTAGAC	ACCACAAGAA	TTAAAAGTGT	TCAGAGAAAA	CTGGAAGAAA	TTCATAAAGC	6245
Pan_troglodytes	GTATCAAACG	GGGTGATATT	AAAGGGGTGT	CTAAGAATGT	TGTTTAATAT	CGGTGTAAGA	CCCAAACAGT	3996
Ailuropoda_melanoleuca	----------	----------	----------	----------	----------	----------	----------	462
Cavia_porcellus	----------	----------	----------	----------	----------	----------	----------	527
Ictidomys_tridecemlineatus	----------	----------	----------	----------	----------	----------	----------	530
Canis	----------	----------	----------	----------	----------	----------	----------	479
Felis_catus	----------	----------	----------	----------	----------	----------	----------	423
Ochotona_princeps	----------	----------	----------	----------	----------	----------	----------	515
Cercopithecidae	----------	----------	----------	----------	----------	----------	----------	2243
Carnivora	----------	----------	----------	----------	----------	----------	----------	421
Mus	AACTATGGTA	TCTTTCTGTT	ATAAACGAGA	GTTAGAAACG	AAGTCAAGTG	GTCTTTTCTC	CCTACTCTAT	2451
Mus_musculus	----------	----------	----------	----------	----------	----------	----------	478
Rattus_norvegicus	----------	----------	----------	----------	----------	----------	----------	420
Tursiops_truncatus	----------	----------	----------	----------	----------	----------	----------	518
Procavia_capensis	----------	----------	----------	----------	----------	----------	----------	442
Primates	----------	----------	----------	----------	----------	----------	----------	671
Echinops_telfairi	----------	----------	----------	----------	----------	----------	----------	473
Sorex_araneus	----------	----------	----------	----------	----------	----------	----------	494
Canidae	----------	----------	----------	----------	----------	----------	----------	550
Otolemur_garnettii	----------	----------	----------	----------	----------	----------	----------	413
Gallus	----------	----------	----------	----------	----------	----------	----------	633
Callithrix_jacchus	----------	----------	----------	----------	----------	----------	----------	495
Crocodylus_porosus	----------	----------	----------	----------	----------	----------	----------	527
Erinaceus_europaeus	----------	----------	----------	----------	----------	----------	----------	420
rnd-6_family-7426-LTR-ERV1	----------	----------	----------	----------	----------	----------	----------	1692
Esox_lucius	----------	----------	----------	----------	----------	----------	----------	532
Salmo_salar	----------	----------	----------	----------	----------	----------	----------	801
Didelphidae	----------	----------	----------	----------	----------	----------	----------	642
Latimeria_chalumnae	----------	----------	----------	----------	----------	----------	----------	1766
Monodelphis_domestica	----------	----------	----------	----------	----------	----------	----------	543
Macropus_eugenii	----------	----------	----------	----------	----------	----------	----------	509
Alligator_mississippiensis	----------	----------	----------	----------	----------	----------	----------	566
Chrysemys_picta_bellii	----------	----------	----------	----------	----------	----------	----------	3578
Danio_rerio	----------	----------	----------	----------	----------	----------	----------	699
Taeniopygia	----------	----------	----------	----------	----------	----------	----------	538
Dasypus_novemcinctus	----------	----------	----------	----------	----------	----------	----------	512
LTR_XT	----------	----------	----------	----------	----------	----------	----------	503
Haplorrhini	GCGAGCACCA	AAGGGGGGGA	A---------	----------	----------	----------	----------	8801
Catarrhini	-------TCA	AAGGCGGGGA	A---------	----------	----------	----------	----------	7847
Anolis_carolinensis	----------	----------	----------	----------	----------	----------	----------	1184
Murine_leukemia_virus	----------	----------	----------	----------	----------	----------	----------	7615
Phascolarctos_cinereus	----------	----------	----------	----------	----------	----------	----------	7426
Estrildidae	----------	----------	----------	----------	----------	----------	----------	482
Xenopus_Silurana_tropicalis	----------	----------	----------	----------	----------	----------	----------	812
I_XT	----------	----------	----------	----------	----------	----------	----------	7509
Takifugu_rubripes	----------	----------	----------	----------	----------	----------	----------	707
Bos_taurus	----------	----------	----------	----------	----------	----------	----------	533
Homo_sapiens	----------	----------	----------	----------	----------	----------	----------	450
Microcebus_murinus	----------	----------	----------	----------	----------	----------	----------	428
Myotis_lucifugus	----------	----------	----------	----------	----------	----------	----------	432
Equus_caballus	----------	----------	----------	----------	----------	----------	----------	450
Tupaia_belangeri	----------	----------	----------	----------	----------	----------	----------	498
Loxodonta_africana	----------	----------	----------	----------	----------	----------	----------	460
Murinae	----------	----------	----------	----------	----------	----------	----------	489
Canis_lupus_familiaris	----------	----------	----------	----------	----------	----------	----------	536
Sus_scrofa	----------	----------	----------	----------	----------	----------	----------	454
Choloepus_hoffmanni	----------	----------	----------	----------	----------	----------	----------	444
Vicugna_pacos	----------	----------	----------	----------	----------	----------	----------	474
Gavialis_gangeticus	----------	----------	----------	----------	----------	----------	----------	470
Mammalia	----------	----------	----------	----------	----------	----------	----------	1337
Passeroidea	----------	----------	----------	----------	----------	----------	----------	641
Takifugu	----------	----------	----------	----------	----------	----------	----------	629
Pteropus_vampyrus	----------	----------	----------	----------	----------	----------	----------	472
Ornithorhynchus	----------	----------	----------	----------	----------	----------	----------	2785
Oryctolagus_cuniculus	----------	----------	----------	----------	----------	----------	----------	494
Gallus_gallus	----------	----------	----------	----------	----------	----------	----------	508
Hominidae	NTNTTTCGCC	CCCNCCNCTN	GNNNCNTANC	TNCCNTANCT	NACCCTNNTN	NGNGANCTNC	TNNGNNANCA	5212
Metatheria	----------	----------	----------	----------	----------	----------	----------	569
Rodentia	CTATCA---C	CTCTCCCTGA	TCACCTNCTT	ACTCCTCTGT	CACACTATNT	TCGCACCTCC	TTGGAACTCA	2819
Monodelphis	----------	----------	----------	----------	----------	----------	----------	857
Consensus	----------	----------	----------	----------	----------	----------	----------	
Conservation (100% / 0%)								

8,980 9,000 9,020

Dipodomys_ordii	----------	----------	----------	----------	----------	----------	----------	597
Laurasiatheria	----------	----------	----------	----------	----------	----------	----------	361
Eutheria	----------	----------	----------	----------	----------	----------	----------	452
Tarsius_syrichta	----------	----------	----------	----------	----------	----------	----------	487
Muridae	CTGGTCCCCT	GATTTGGCCT	CCCCCTAGCA	ATGGGGTCCG	ACAATGGCCT	GGCTTTCATA	GCCAAAACCT	3348
Gasterosteus_aculeatus	----------	----------	----------	----------	----------	----------	----------	2399
Felis_silvestris	----------	----------	----------	----------	----------	----------	----------	505
Rattus	----------	----------	----------	----------	----------	----------	----------	589
Papio_hamadryas	CCTATATCCT	GAACTTCAGT	ATCACCCTTT	GGCCATACCT	AAGGTTAGAG	ATAACCTCAT	GGTCGATGCC	6315
Pan_troglodytes	AACA-AAGAA	GAACATTGAT	AACATCTGTT	TCTCTTCCCT	CCTGGCCCCT	GTTTTTTTAC	TGCCTGTTTT	4065
Ailuropoda_melanoleuca	----------	----------	----------	----------	----------	----------	----------	462
Cavia_porcellus	----------	----------	----------	----------	----------	----------	----------	527
Ictidomys_tridecemlineatus	----------	----------	----------	----------	----------	----------	----------	530
Canis	----------	----------	----------	----------	----------	----------	----------	479
Felis_catus	----------	----------	----------	----------	----------	----------	----------	423
Ochotona_princeps	----------	----------	----------	----------	----------	----------	----------	515
Cercopithecidae	----------	----------	----------	----------	----------	----------	----------	2243
Carnivora	----------	----------	----------	----------	----------	----------	----------	421
Mus	ATATAGCTTA	ACAAGACACG	GTAATAGAAG	AAACCCA---	----------	----------	----------	2488
Mus_musculus	----------	----------	----------	----------	----------	----------	----------	478
Rattus_norvegicus	----------	----------	----------	----------	----------	----------	----------	420
Tursiops_truncatus	----------	----------	----------	----------	----------	----------	----------	518
Procavia_capensis	----------	----------	----------	----------	----------	----------	----------	442
Primates	----------	----------	----------	----------	----------	----------	----------	671
Echinops_telfairi	----------	----------	----------	----------	----------	----------	----------	473
Sorex_araneus	----------	----------	----------	----------	----------	----------	----------	494
Canidae	----------	----------	----------	----------	----------	----------	----------	550
Otolemur_garnettii	----------	----------	----------	----------	----------	----------	----------	413
Gallus	----------	----------	----------	----------	----------	----------	----------	633
Callithrix_jacchus	----------	----------	----------	----------	----------	----------	----------	495
Crocodylus_porosus	----------	----------	----------	----------	----------	----------	----------	527
Erinaceus_europaeus	----------	----------	----------	----------	----------	----------	----------	420
rnd-6_family-7426-LTR-ERV1	----------	----------	----------	----------	----------	----------	----------	1692
Esox_lucius	----------	----------	----------	----------	----------	----------	----------	532
Salmo_salar	----------	----------	----------	----------	----------	----------	----------	801
Didelphidae	----------	----------	----------	----------	----------	----------	----------	642
Latimeria_chalumnae	----------	----------	----------	----------	----------	----------	----------	1766
Monodelphis_domestica	----------	----------	----------	----------	----------	----------	----------	543
Macropus_eugenii	----------	----------	----------	----------	----------	----------	----------	509
Alligator_mississippiensis	----------	----------	----------	----------	----------	----------	----------	566
Chrysemys_picta_bellii	----------	----------	----------	----------	----------	----------	----------	3578
Danio_rerio	----------	----------	----------	----------	----------	----------	----------	699
Taeniopygia	----------	----------	----------	----------	----------	----------	----------	538
Dasypus_novemcinctus	----------	----------	----------	----------	----------	----------	----------	512
LTR_XT	----------	----------	----------	----------	----------	----------	----------	503
Haplorrhini	----------	----------	----------	----------	----------	----------	----------	8801
Catarrhini	----------	----------	----------	----------	----------	----------	----------	7847
Anolis_carolinensis	----------	----------	----------	----------	----------	----------	----------	1184
Murine_leukemia_virus	----------	----------	----------	----------	----------	----------	----------	7615
Phascolarctos_cinereus	----------	----------	----------	----------	----------	----------	----------	7426
Estrildidae	----------	----------	----------	----------	----------	----------	----------	482
Xenopus_Silurana_tropicalis	----------	----------	----------	----------	----------	----------	----------	812
I_XT	----------	----------	----------	----------	----------	----------	----------	7509
Takifugu_rubripes	----------	----------	----------	----------	----------	----------	----------	707
Bos_taurus	----------	----------	----------	----------	----------	----------	----------	533
Homo_sapiens	----------	----------	----------	----------	----------	----------	----------	450
Microcebus_murinus	----------	----------	----------	----------	----------	----------	----------	428
Myotis_lucifugus	----------	----------	----------	----------	----------	----------	----------	432
Equus_caballus	----------	----------	----------	----------	----------	----------	----------	450
Tupaia_belangeri	----------	----------	----------	----------	----------	----------	----------	498
Loxodonta_africana	----------	----------	----------	----------	----------	----------	----------	460
Murinae	----------	----------	----------	----------	----------	----------	----------	489
Canis_lupus_familiaris	----------	----------	----------	----------	----------	----------	----------	536
Sus_scrofa	----------	----------	----------	----------	----------	----------	----------	454
Choloepus_hoffmanni	----------	----------	----------	----------	----------	----------	----------	444
Vicugna_pacos	----------	----------	----------	----------	----------	----------	----------	474
Gavialis_gangeticus	----------	----------	----------	----------	----------	----------	----------	470
Mammalia	----------	----------	----------	----------	----------	----------	----------	1337
Passeroidea	----------	----------	----------	----------	----------	----------	----------	641
Takifugu	----------	----------	----------	----------	----------	----------	----------	629
Pteropus_vampyrus	----------	----------	----------	----------	----------	----------	----------	472
Ornithorhynchus	----------	----------	----------	----------	----------	----------	----------	2785
Oryctolagus_cuniculus	----------	----------	----------	----------	----------	----------	----------	494
Gallus_gallus	----------	----------	----------	----------	----------	----------	----------	508
Hominidae	CGCNGACNNC	NGCCTNCCNN	ANCCNNNNCC	NNNCNNCCCN	GACNNNCCNG	NNNTNNTNNC	NCCAGGNGAT	5282
Metatheria	----------	----------	----------	----------	----------	----------	----------	569
Rodentia	CCCA--TCAC	TACTTGCCTC	AGCCTCATCC	TGACCCCCTT	CCCTCTTCAA	TCAACATCG-	-----GGGAC	2881
Monodelphis	----------	----------	----------	----------	----------	----------	----------	857
Consensus	----------	----------	----------	----------	----------	----------	----------	

Conservation 100% 0%

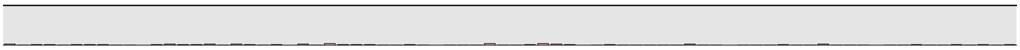

	9,040		9,060		9,080		9,100	
Dipodomys_ordii	----------	----------	----------	----------	----------	----------	----------	597
Laurasiatheria	----------	----------	----------	----------	----------	----------	----------	361
Eutheria	----------	----------	----------	----------	----------	----------	----------	452
Tarsius_syrichta	----------	----------	----------	----------	----------	----------	----------	487
Muridae	CTCAATTAAT	AGCTAAAGCT	TTAGACATAG	ATTGAAAACT	TCATTGTGCA	TATAGACCTC	AGAGTTCTGG	3418
Gasterosteus_aculeatus	----------	----------	----------	----------	----------	----------	----------	2399
Felis_silvestris	----------	----------	----------	----------	----------	----------	----------	505
Rattus	----------	----------	----------	----------	----------	----------	----------	589
Papio_hamadryas	CAGACTTTAA	ACATTCTCAA	TGCCACTTAC	AACTTACTCC	TAATGTCCAA	CACGAGCCTA	GTGGACGACT	6385
Pan_troglodytes	ACGTTTTTAA	AGAAGTTAAG	T--CAATGCC	TACTACCACG	TAACTAAGAG	ACCAAGATTG	CTAAAAAAAG	4133
Ailuropoda_melanoleuca	----------	----------	----------	----------	----------	----------	----------	462
Cavia_porcellus	----------	----------	----------	----------	----------	----------	----------	527
Ictidomys_tridecemlineatus	----------	----------	----------	----------	----------	----------	----------	530
Canis	----------	----------	----------	----------	----------	----------	----------	479
Felis_catus	----------	----------	----------	----------	----------	----------	----------	423
Ochotona_princeps	----------	----------	----------	----------	----------	----------	----------	515
Cercopithecidae	----------	----------	----------	----------	----------	----------	----------	2243
Carnivora	----------	----------	----------	----------	----------	----------	----------	421
Mus	----------	----------	----------	----------	----------	----------	----------	2488
Mus_musculus	----------	----------	----------	----------	----------	----------	----------	478
Rattus_norvegicus	----------	----------	----------	----------	----------	----------	----------	420
Tursiops_truncatus	----------	----------	----------	----------	----------	----------	----------	518
Procavia_capensis	----------	----------	----------	----------	----------	----------	----------	442
Primates	----------	----------	----------	----------	----------	----------	----------	671
Echinops_telfairi	----------	----------	----------	----------	----------	----------	----------	473
Sorex_araneus	----------	----------	----------	----------	----------	----------	----------	494
Canidae	----------	----------	----------	----------	----------	----------	----------	550
Otolemur_garnettii	----------	----------	----------	----------	----------	----------	----------	413
Gallus	----------	----------	----------	----------	----------	----------	----------	633
Callithrix_jacchus	----------	----------	----------	----------	----------	----------	----------	495
Crocodylus_porosus	----------	----------	----------	----------	----------	----------	----------	527
Erinaceus_europaeus	----------	----------	----------	----------	----------	----------	----------	420
rnd-6_family-7426-LTR-ERV1	----------	----------	----------	----------	----------	----------	----------	1692
Esox_lucius	----------	----------	----------	----------	----------	----------	----------	532
Salmo_salar	----------	----------	----------	----------	----------	----------	----------	801
Didelphidae	----------	----------	----------	----------	----------	----------	----------	642
Latimeria_chalumnae	----------	----------	----------	----------	----------	----------	----------	1766
Monodelphis_domestica	----------	----------	----------	----------	----------	----------	----------	543
Macropus_eugenii	----------	----------	----------	----------	----------	----------	----------	509
Alligator_mississippiensis	----------	----------	----------	----------	----------	----------	----------	566
Chrysemys_picta_bellii	----------	----------	----------	----------	----------	----------	----------	3578
Danio_rerio	----------	----------	----------	----------	----------	----------	----------	699
Taeniopygia	----------	----------	----------	----------	----------	----------	----------	538
Dasypus_novemcinctus	----------	----------	----------	----------	----------	----------	----------	512
LTR_XT	----------	----------	----------	----------	----------	----------	----------	503
Haplorrhini	----------	----------	----------	----------	----------	----------	----------	8801
Catarrhini	----------	----------	----------	----------	----------	----------	----------	7847
Anolis_carolinensis	----------	----------	----------	----------	----------	----------	----------	1184
Murine_leukemia_virus	----------	----------	----------	----------	----------	----------	----------	7615
Phascolarctos_cinereus	----------	----------	----------	----------	----------	----------	----------	7426
Estrildidae	----------	----------	----------	----------	----------	----------	----------	482
Xenopus_Silurana_tropicalis	----------	----------	----------	----------	----------	----------	----------	812
I_XT	----------	----------	----------	----------	----------	----------	----------	7509
Takifugu_rubripes	----------	----------	----------	----------	----------	----------	----------	707
Bos_taurus	----------	----------	----------	----------	----------	----------	----------	533
Homo_sapiens	----------	----------	----------	----------	----------	----------	----------	450
Microcebus_murinus	----------	----------	----------	----------	----------	----------	----------	428
Myotis_lucifugus	----------	----------	----------	----------	----------	----------	----------	432
Equus_caballus	----------	----------	----------	----------	----------	----------	----------	450
Tupaia_belangeri	----------	----------	----------	----------	----------	----------	----------	498
Loxodonta_africana	----------	----------	----------	----------	----------	----------	----------	460
Murinae	----------	----------	----------	----------	----------	----------	----------	489
Canis_lupus_familiaris	----------	----------	----------	----------	----------	----------	----------	536
Sus_scrofa	----------	----------	----------	----------	----------	----------	----------	454
Choloepus_hoffmanni	----------	----------	----------	----------	----------	----------	----------	444
Vicugna_pacos	----------	----------	----------	----------	----------	----------	----------	474
Gavialis_gangeticus	----------	----------	----------	----------	----------	----------	----------	470
Mammalia	----------	----------	----------	----------	----------	----------	----------	1337
Passeroidea	----------	----------	----------	----------	----------	----------	----------	641
Takifugu	----------	----------	----------	----------	----------	----------	----------	629
Pteropus_vampyrus	----------	----------	----------	----------	----------	----------	----------	472
Ornithorhynchus	----------	----------	----------	----------	----------	----------	----------	2785
Oryctolagus_cuniculus	----------	----------	----------	----------	----------	----------	----------	494
Gallus_gallus	----------	----------	----------	----------	----------	----------	----------	508
Hominidae	NNNGTNCTNN	TNAANGANCT	NCAGNCNANN	NNNNTNNCCC	CNCGGTGGNN	AGGCCCNTAN	ACNGTNATNC	5352
Metatheria	----------	----------	----------	----------	----------	----------	----------	569
Rodentia	TTGGTCCTCC	CCTCTCNTCC	--------TG	NTCACANCCC	CCTAATGGAC	AGGCCCCTTT	AGGGTCATCC	2943
Monodelphis	----------	----------	----------	----------	----------	----------	----------	857
Consensus	----------	----------	----------	----------	----------	----------	----------	

Conservation
100%
0%

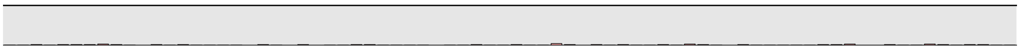

	9,120		9,140		9,160			
Dipodomys_ordii	----------	----------	----------	----------	----------	----------	----------	597
Laurasiatheria	----------	----------	----------	----------	----------	----------	----------	361
Eutheria	----------	----------	----------	----------	----------	----------	----------	452
Tarsius_syrichta	----------	----------	----------	----------	----------	----------	----------	487
Muridae	GCAGGTAAAA	AGAATAAACA	GGACATTAAA	AGAAACTCTA	ACAAAACTCT	CATTAGAGTC	CAGCGAAGGG	3488
Gasterosteus_aculeatus	----------	----------	----------	----------	----------	----------	----------	2399
Felis_silvestris	----------	----------	----------	----------	----------	----------	----------	505
Rattus	----------	----------	----------	----------	----------	----------	----------	589
Papio_hamadryas	GTTGGCTTTG	TTTAAAATTA	GGTCCCCCTA	CTCCCCTCGC	AATACCTAAC	TTCCTATTAT	CCTACGTGAC	6455
Pan_troglodytes	GGGGA-----	----------	----------	----------	----------	----------	----------	4138
Ailuropoda_melanoleuca	----------	----------	----------	----------	----------	----------	----------	462
Cavia_porcellus	----------	----------	----------	----------	----------	----------	----------	527
Ictidomys_tridecemlineatus	----------	----------	----------	----------	----------	----------	----------	530
Canis	----------	----------	----------	----------	----------	----------	----------	479
Felis_catus	----------	----------	----------	----------	----------	----------	----------	423
Ochotona_princeps	----------	----------	----------	----------	----------	----------	----------	515
Cercopithecidae	----------	----------	----------	----------	----------	----------	----------	2243
Carnivora	----------	----------	----------	----------	----------	----------	----------	421
Mus	----------	----------	----------	----------	----------	----------	----------	2488
Mus_musculus	----------	----------	----------	----------	----------	----------	----------	478
Rattus_norvegicus	----------	----------	----------	----------	----------	----------	----------	420
Tursiops_truncatus	----------	----------	----------	----------	----------	----------	----------	518
Procavia_capensis	----------	----------	----------	----------	----------	----------	----------	442
Primates	----------	----------	----------	----------	----------	----------	----------	671
Echinops_telfairi	----------	----------	----------	----------	----------	----------	----------	473
Sorex_araneus	----------	----------	----------	----------	----------	----------	----------	494
Canidae	----------	----------	----------	----------	----------	----------	----------	550
Otolemur_garnettii	----------	----------	----------	----------	----------	----------	----------	413
Gallus	----------	----------	----------	----------	----------	----------	----------	633
Callithrix_jacchus	----------	----------	----------	----------	----------	----------	----------	495
Crocodylus_porosus	----------	----------	----------	----------	----------	----------	----------	527
Erinaceus_europaeus	----------	----------	----------	----------	----------	----------	----------	420
rnd-6_family-7426-LTR-ERV1	----------	----------	----------	----------	----------	----------	----------	1692
Esox_lucius	----------	----------	----------	----------	----------	----------	----------	532
Salmo_salar	----------	----------	----------	----------	----------	----------	----------	801
Didelphidae	----------	----------	----------	----------	----------	----------	----------	642
Latimeria_chalumnae	----------	----------	----------	----------	----------	----------	----------	1766
Monodelphis_domestica	----------	----------	----------	----------	----------	----------	----------	543
Macropus_eugenii	----------	----------	----------	----------	----------	----------	----------	509
Alligator_mississippiensis	----------	----------	----------	----------	----------	----------	----------	566
Chrysemys_picta_bellii	----------	----------	----------	----------	----------	----------	----------	3578
Danio_rerio	----------	----------	----------	----------	----------	----------	----------	699
Taeniopygia	----------	----------	----------	----------	----------	----------	----------	538
Dasypus_novemcinctus	----------	----------	----------	----------	----------	----------	----------	512
LTR_XT	----------	----------	----------	----------	----------	----------	----------	503
Haplorrhini	----------	----------	----------	----------	----------	----------	----------	8801
Catarrhini	----------	----------	----------	----------	----------	----------	----------	7847
Anolis_carolinensis	----------	----------	----------	----------	----------	----------	----------	1184
Murine_leukemia_virus	----------	----------	----------	----------	----------	----------	----------	7615
Phascolarctos_cinereus	----------	----------	----------	----------	----------	----------	----------	7426
Estrildidae	----------	----------	----------	----------	----------	----------	----------	482
Xenopus_Silurana_tropicalis	----------	----------	----------	----------	----------	----------	----------	812
I_XT	----------	----------	----------	----------	----------	----------	----------	7509
Takifugu_rubripes	----------	----------	----------	----------	----------	----------	----------	707
Bos_taurus	----------	----------	----------	----------	----------	----------	----------	533
Homo_sapiens	----------	----------	----------	----------	----------	----------	----------	450
Microcebus_murinus	----------	----------	----------	----------	----------	----------	----------	428
Myotis_lucifugus	----------	----------	----------	----------	----------	----------	----------	432
Equus_caballus	----------	----------	----------	----------	----------	----------	----------	450
Tupaia_belangeri	----------	----------	----------	----------	----------	----------	----------	498
Loxodonta_africana	----------	----------	----------	----------	----------	----------	----------	460
Murinae	----------	----------	----------	----------	----------	----------	----------	489
Canis_lupus_familiaris	----------	----------	----------	----------	----------	----------	----------	536
Sus_scrofa	----------	----------	----------	----------	----------	----------	----------	454
Choloepus_hoffmanni	----------	----------	----------	----------	----------	----------	----------	444
Vicugna_pacos	----------	----------	----------	----------	----------	----------	----------	474
Gavialis_gangeticus	----------	----------	----------	----------	----------	----------	----------	470
Mammalia	----------	----------	----------	----------	----------	----------	----------	1337
Passeroidea	----------	----------	----------	----------	----------	----------	----------	641
Takifugu	----------	----------	----------	----------	----------	----------	----------	629
Pteropus_vampyrus	----------	----------	----------	----------	----------	----------	----------	472
Ornithorhynchus	----------	----------	----------	----------	----------	----------	----------	2785
Oryctolagus_cuniculus	----------	----------	----------	----------	----------	----------	----------	494
Gallus_gallus	----------	----------	----------	----------	----------	----------	----------	508
Hominidae	TNACNACNCC	GACGGCNGCT	AANNTNNTAG	GNCTNCCNTC	CTGGTANCAT	NTNTCNCANN	TNAAGANNGC	5422
Metatheria	----------	----------	----------	----------	----------	----------	----------	569
Rodentia	TTACTACCCC	AACTGCCGCT	AAGCTCGATG	GTTTCCCTCA	CTGGATTCAT	CTTTCTCATC	TCAAGCCCTT	3013
Monodelphis	----------	----------	----------	----------	----------	----------	----------	857
Consensus	----------	----------	----------	----------	----------	----------	----------	

Conservation (100% – 0%)

	9,180		9,200		9,220		9,240	
Dipodomys_ordii	----------	----------	----------	----------	----------	----------	----------	597
Laurasiatheria	----------	----------	----------	----------	----------	----------	----------	361
Eutheria	----------	----------	----------	----------	----------	----------	----------	452
Tarsius_syrichta	----------	----------	----------	----------	----------	----------	----------	487
Muridae	TGGATAGACC	TCCTTCCTTT	GCTTCGGGCC	GCTGCACCCC	ATATAGGGAG	GGATTTACCC	CTCCCTATGA	3558
Gasterosteus_aculeatus	----------	----------	----------	----------	----------	----------	----------	2399
Felis_silvestris	----------	----------	----------	----------	----------	----------	----------	505
Rattus	----------	----------	----------	----------	----------	----------	----------	589
Papio_hamadryas	TCGCTCCTCG	GATAATATCT	CTTGTTTAAT	AATTCCCCCC	CTTCTAGTTC	AACCGATGCA	GTTTTCCAAT	6525
Pan_troglodytes	----------	----------	----------	----------	----------	----------	----------	4138
Ailuropoda_melanoleuca	----------	----------	----------	----------	----------	----------	----------	462
Cavia_porcellus	----------	----------	----------	----------	----------	----------	----------	527
Ictidomys_tridecemlineatus	----------	----------	----------	----------	----------	----------	----------	530
Canis	----------	----------	----------	----------	----------	----------	----------	479
Felis_catus	----------	----------	----------	----------	----------	----------	----------	423
Ochotona_princeps	----------	----------	----------	----------	----------	----------	----------	515
Cercopithecidae	----------	----------	----------	----------	----------	----------	----------	2243
Carnivora	----------	----------	----------	----------	----------	----------	----------	421
Mus	----------	----------	----------	----------	----------	----------	----------	2488
Mus_musculus	----------	----------	----------	----------	----------	----------	----------	478
Rattus_norvegicus	----------	----------	----------	----------	----------	----------	----------	420
Tursiops_truncatus	----------	----------	----------	----------	----------	----------	----------	518
Procavia_capensis	----------	----------	----------	----------	----------	----------	----------	442
Primates	----------	----------	----------	----------	----------	----------	----------	671
Echinops_telfairi	----------	----------	----------	----------	----------	----------	----------	473
Sorex_araneus	----------	----------	----------	----------	----------	----------	----------	494
Canidae	----------	----------	----------	----------	----------	----------	----------	550
Otolemur_garnettii	----------	----------	----------	----------	----------	----------	----------	413
Gallus	----------	----------	----------	----------	----------	----------	----------	633
Callithrix_jacchus	----------	----------	----------	----------	----------	----------	----------	495
Crocodylus_porosus	----------	----------	----------	----------	----------	----------	----------	527
Erinaceus_europaeus	----------	----------	----------	----------	----------	----------	----------	420
rnd-6_family-7426-LTR-ERV1	----------	----------	----------	----------	----------	----------	----------	1692
Esox_lucius	----------	----------	----------	----------	----------	----------	----------	532
Salmo_salar	----------	----------	----------	----------	----------	----------	----------	801
Didelphidae	----------	----------	----------	----------	----------	----------	----------	642
Latimeria_chalumnae	----------	----------	----------	----------	----------	----------	----------	1766
Monodelphis_domestica	----------	----------	----------	----------	----------	----------	----------	543
Macropus_eugenii	----------	----------	----------	----------	----------	----------	----------	509
Alligator_mississippiensis	----------	----------	----------	----------	----------	----------	----------	566
Chrysemys_picta_bellii	----------	----------	----------	----------	----------	----------	----------	3578
Danio_rerio	----------	----------	----------	----------	----------	----------	----------	699
Taeniopygia	----------	----------	----------	----------	----------	----------	----------	538
Dasypus_novemcinctus	----------	----------	----------	----------	----------	----------	----------	512
LTR_XT	----------	----------	----------	----------	----------	----------	----------	503
Haplorrhini	----------	----------	----------	----------	----------	----------	----------	8801
Catarrhini	----------	----------	----------	----------	----------	----------	----------	7847
Anolis_carolinensis	----------	----------	----------	----------	----------	----------	----------	1184
Murine_leukemia_virus	----------	----------	----------	----------	----------	----------	----------	7615
Phascolarctos_cinereus	----------	----------	----------	----------	----------	----------	----------	7426
Estrildidae	----------	----------	----------	----------	----------	----------	----------	482
Xenopus_Silurana_tropicalis	----------	----------	----------	----------	----------	----------	----------	812
I_XT	----------	----------	----------	----------	----------	----------	----------	7509
Takifugu_rubripes	----------	----------	----------	----------	----------	----------	----------	707
Bos_taurus	----------	----------	----------	----------	----------	----------	----------	533
Homo_sapiens	----------	----------	----------	----------	----------	----------	----------	450
Microcebus_murinus	----------	----------	----------	----------	----------	----------	----------	428
Myotis_lucifugus	----------	----------	----------	----------	----------	----------	----------	432
Equus_caballus	----------	----------	----------	----------	----------	----------	----------	450
Tupaia_belangeri	----------	----------	----------	----------	----------	----------	----------	498
Loxodonta_africana	----------	----------	----------	----------	----------	----------	----------	460
Murinae	----------	----------	----------	----------	----------	----------	----------	489
Canis_lupus_familiaris	----------	----------	----------	----------	----------	----------	----------	536
Sus_scrofa	----------	----------	----------	----------	----------	----------	----------	454
Choloepus_hoffmanni	----------	----------	----------	----------	----------	----------	----------	444
Vicugna_pacos	----------	----------	----------	----------	----------	----------	----------	474
Gavialis_gangeticus	----------	----------	----------	----------	----------	----------	----------	470
Mammalia	----------	----------	----------	----------	----------	----------	----------	1337
Passeroidea	----------	----------	----------	----------	----------	----------	----------	641
Takifugu	----------	----------	----------	----------	----------	----------	----------	629
Pteropus_vampyrus	----------	----------	----------	----------	----------	----------	----------	472
Ornithorhynchus	----------	----------	----------	----------	----------	----------	----------	2785
Oryctolagus_cuniculus	----------	----------	----------	----------	----------	----------	----------	494
Gallus_gallus	----------	----------	----------	----------	----------	----------	----------	508
Hominidae	ACCNACNCAN	CANCAGGCCA	CTTGGACTGN	NNCNNNNNNC	TCNCCAACCA	ANCNGNAACT	CTNNNNATCA	5492
Metatheria	----------	----------	----------	----------	----------	----------	----------	569
Rodentia	TACNCCATCG	TCTCAAGATA	ATCCTCTTNN	TNNANNNAAC	NCNNNNNGNA	NNNNGCTCTC	TTAAGTNTCN	3083
Monodelphis	----------	----------	----------	----------	----------	----------	----------	857
Consensus	----------	----------	----------	----------	----------	----------	----------	

Conservation

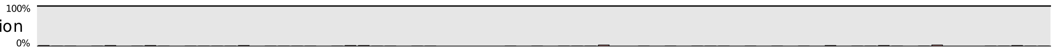

	9,260			9,280		9,300		
Dipodomys_ordii	----------	----------	----------	----------	----------	----------	----------	597
Laurasiatheria	----------	----------	----------	----------	----------	----------	----------	361
Eutheria	----------	----------	----------	----------	----------	----------	----------	452
Tarsius_syrichta	----------	----------	----------	----------	----------	----------	----------	487
Muridae	GATTATGTTT	GGAAGACCTC	CCCACTGCTT	CCCAGGCTCA	GAGACCAGCA	GTTGGCAGAA	CTCTCTAACC	3628
Gasterosteus_aculeatus	----------	----------	----------	----------	----------	----------	----------	2399
Felis_silvestris	----------	----------	----------	----------	----------	----------	----------	505
Rattus	----------	----------	----------	----------	----------	----------	----------	589
Papio_hamadryas	TCATCTTGCC	TCTTTTCCCC	CTCCTACAAC	AGTACAGAAG	AAATAGATCT	AGGCCATGTT	GCCTTCAGCA	6595
Pan_troglodytes	----------	----------	----------	----------	----------	----------	----------	4138
Ailuropoda_melanoleuca	----------	----------	----------	----------	----------	----------	----------	462
Cavia_porcellus	----------	----------	----------	----------	----------	----------	----------	527
Ictidomys_tridecemlineatus	----------	----------	----------	----------	----------	----------	----------	530
Canis	----------	----------	----------	----------	----------	----------	----------	479
Felis_catus	----------	----------	----------	----------	----------	----------	----------	423
Ochotona_princeps	----------	----------	----------	----------	----------	----------	----------	515
Cercopithecidae	----------	----------	----------	----------	----------	----------	----------	2243
Carnivora	----------	----------	----------	----------	----------	----------	----------	421
Mus	----------	----------	----------	----------	----------	----------	----------	2488
Mus_musculus	----------	----------	----------	----------	----------	----------	----------	478
Rattus_norvegicus	----------	----------	----------	----------	----------	----------	----------	420
Tursiops_truncatus	----------	----------	----------	----------	----------	----------	----------	518
Procavia_capensis	----------	----------	----------	----------	----------	----------	----------	442
Primates	----------	----------	----------	----------	----------	----------	----------	671
Echinops_telfairi	----------	----------	----------	----------	----------	----------	----------	473
Sorex_araneus	----------	----------	----------	----------	----------	----------	----------	494
Canidae	----------	----------	----------	----------	----------	----------	----------	550
Otolemur_garnettii	----------	----------	----------	----------	----------	----------	----------	413
Gallus	----------	----------	----------	----------	----------	----------	----------	633
Callithrix_jacchus	----------	----------	----------	----------	----------	----------	----------	495
Crocodylus_porosus	----------	----------	----------	----------	----------	----------	----------	527
Erinaceus_europaeus	----------	----------	----------	----------	----------	----------	----------	420
rnd-6_family-7426-LTR-ERV1	----------	----------	----------	----------	----------	----------	----------	1692
Esox_lucius	----------	----------	----------	----------	----------	----------	----------	532
Salmo_salar	----------	----------	----------	----------	----------	----------	----------	801
Didelphidae	----------	----------	----------	----------	----------	----------	----------	642
Latimeria_chalumnae	----------	----------	----------	----------	----------	----------	----------	1766
Monodelphis_domestica	----------	----------	----------	----------	----------	----------	----------	543
Macropus_eugenii	----------	----------	----------	----------	----------	----------	----------	509
Alligator_mississippiensis	----------	----------	----------	----------	----------	----------	----------	566
Chrysemys_picta_bellii	----------	----------	----------	----------	----------	----------	----------	3578
Danio_rerio	----------	----------	----------	----------	----------	----------	----------	699
Taeniopygia	----------	----------	----------	----------	----------	----------	----------	538
Dasypus_novemcinctus	----------	----------	----------	----------	----------	----------	----------	512
LTR_XT	----------	----------	----------	----------	----------	----------	----------	503
Haplorrhini	----------	----------	----------	----------	----------	----------	----------	8801
Catarrhini	----------	----------	----------	----------	----------	----------	----------	7847
Anolis_carolinensis	----------	----------	----------	----------	----------	----------	----------	1184
Murine_leukemia_virus	----------	----------	----------	----------	----------	----------	----------	7615
Phascolarctos_cinereus	----------	----------	----------	----------	----------	----------	----------	7426
Estrildidae	----------	----------	----------	----------	----------	----------	----------	482
Xenopus_Silurana_tropicalis	----------	----------	----------	----------	----------	----------	----------	812
I_XT	----------	----------	----------	----------	----------	----------	----------	7509
Takifugu_rubripes	----------	----------	----------	----------	----------	----------	----------	707
Bos_taurus	----------	----------	----------	----------	----------	----------	----------	533
Homo_sapiens	----------	----------	----------	----------	----------	----------	----------	450
Microcebus_murinus	----------	----------	----------	----------	----------	----------	----------	428
Myotis_lucifugus	----------	----------	----------	----------	----------	----------	----------	432
Equus_caballus	----------	----------	----------	----------	----------	----------	----------	450
Tupaia_belangeri	----------	----------	----------	----------	----------	----------	----------	498
Loxodonta_africana	----------	----------	----------	----------	----------	----------	----------	460
Murinae	----------	----------	----------	----------	----------	----------	----------	489
Canis_lupus_familiaris	----------	----------	----------	----------	----------	----------	----------	536
Sus_scrofa	----------	----------	----------	----------	----------	----------	----------	454
Choloepus_hoffmanni	----------	----------	----------	----------	----------	----------	----------	444
Vicugna_pacos	----------	----------	----------	----------	----------	----------	----------	474
Gavialis_gangeticus	----------	----------	----------	----------	----------	----------	----------	470
Mammalia	----------	----------	----------	----------	----------	----------	----------	1337
Passeroidea	----------	----------	----------	----------	----------	----------	----------	641
Takifugu	----------	----------	----------	----------	----------	----------	----------	629
Pteropus_vampyrus	----------	----------	----------	----------	----------	----------	----------	472
Ornithorhynchus	----------	----------	----------	----------	----------	----------	----------	2785
Oryctolagus_cuniculus	----------	----------	----------	----------	----------	----------	----------	494
Gallus_gallus	----------	----------	----------	----------	----------	----------	----------	508
Hominidae	NNNANNGCAN	NNANNNTCCN	CNNNANAGNC	TCCNACAATT	NNTNCNGNNN	CTNCTCGAGG	AAAGCTGGTC	5562
Metatheria	----------	----------	----------	----------	----------	----------	----------	569
Rodentia	GNAGANACNN	AGGTNGACNN	NNTNGANNNN	NNNTNCATNT	GNNG-AGGNN	NNAGCCGANG	NTNNNGGNCN	3152
Monodelphis	----------	----------	----------	----------	----------	----------	----------	857
Consensus	----------	----------	----------	----------	----------	----------	----------	

Conservation (100% – 0%)

	9,320		9,340		9,360		9,380	
Dipodomys_ordii	----------	----------	----------	----------	----------	----------	----------	597
Laurasiatheria	----------	----------	----------	----------	----------	----------	----------	361
Eutheria	----------	----------	----------	----------	----------	----------	----------	452
Tarsius_syrichta	----------	----------	----------	----------	----------	----------	----------	487
Muridae	ATTCCTTTCT	CAAGTCACTA	CAGGCCCTCC	AGTCCTCCAT	AAAGGCTATT	CATTGGACTA	TCTGAGAGAC	3698
Gasterosteus_aculeatus	----------	----------	----------	----------	----------	----------	----------	2399
Felis_silvestris	----------	----------	----------	----------	----------	----------	----------	505
Rattus	----------	----------	----------	----------	----------	----------	----------	589
Papio_hamadryas	ACTGTACCTC	CATAACCAAT	GTCACCGGTC	CCATATGCGC	TGTAAATGGT	TCGGTCTTTC	TCTGTGGCAA	6665
Pan_troglodytes	----------	----------	----------	----------	----------	----------	----------	4138
Ailuropoda_melanoleuca	----------	----------	----------	----------	----------	----------	----------	462
Cavia_porcellus	----------	----------	----------	----------	----------	----------	----------	527
Ictidomys_tridecemlineatus	----------	----------	----------	----------	----------	----------	----------	530
Canis	----------	----------	----------	----------	----------	----------	----------	479
Felis_catus	----------	----------	----------	----------	----------	----------	----------	423
Ochotona_princeps	----------	----------	----------	----------	----------	----------	----------	515
Cercopithecidae	----------	----------	----------	----------	----------	----------	----------	2243
Carnivora	----------	----------	----------	----------	----------	----------	----------	421
Mus	----------	----------	----------	----------	----------	----------	----------	2488
Mus_musculus	----------	----------	----------	----------	----------	----------	----------	478
Rattus_norvegicus	----------	----------	----------	----------	----------	----------	----------	420
Tursiops_truncatus	----------	----------	----------	----------	----------	----------	----------	518
Procavia_capensis	----------	----------	----------	----------	----------	----------	----------	442
Primates	----------	----------	----------	----------	----------	----------	----------	671
Echinops_telfairi	----------	----------	----------	----------	----------	----------	----------	473
Sorex_araneus	----------	----------	----------	----------	----------	----------	----------	494
Canidae	----------	----------	----------	----------	----------	----------	----------	550
Otolemur_garnettii	----------	----------	----------	----------	----------	----------	----------	413
Gallus	----------	----------	----------	----------	----------	----------	----------	633
Callithrix_jacchus	----------	----------	----------	----------	----------	----------	----------	495
Crocodylus_porosus	----------	----------	----------	----------	----------	----------	----------	527
Erinaceus_europaeus	----------	----------	----------	----------	----------	----------	----------	420
rnd-6_family-7426-LTR-ERV1	----------	----------	----------	----------	----------	----------	----------	1692
Esox_lucius	----------	----------	----------	----------	----------	----------	----------	532
Salmo_salar	----------	----------	----------	----------	----------	----------	----------	801
Didelphidae	----------	----------	----------	----------	----------	----------	----------	642
Latimeria_chalumnae	----------	----------	----------	----------	----------	----------	----------	1766
Monodelphis_domestica	----------	----------	----------	----------	----------	----------	----------	543
Macropus_eugenii	----------	----------	----------	----------	----------	----------	----------	509
Alligator_mississippiensis	----------	----------	----------	----------	----------	----------	----------	566
Chrysemys_picta_bellii	----------	----------	----------	----------	----------	----------	----------	3578
Danio_rerio	----------	----------	----------	----------	----------	----------	----------	699
Taeniopygia	----------	----------	----------	----------	----------	----------	----------	538
Dasypus_novemcinctus	----------	----------	----------	----------	----------	----------	----------	512
LTR_XT	----------	----------	----------	----------	----------	----------	----------	503
Haplorrhini	----------	----------	----------	----------	----------	----------	----------	8801
Catarrhini	----------	----------	----------	----------	----------	----------	----------	7847
Anolis_carolinensis	----------	----------	----------	----------	----------	----------	----------	1184
Murine_leukemia_virus	----------	----------	----------	----------	----------	----------	----------	7615
Phascolarctos_cinereus	----------	----------	----------	----------	----------	----------	----------	7426
Estrildidae	----------	----------	----------	----------	----------	----------	----------	482
Xenopus_Silurana_tropicalis	----------	----------	----------	----------	----------	----------	----------	812
I_XT	----------	----------	----------	----------	----------	----------	----------	7509
Takifugu_rubripes	----------	----------	----------	----------	----------	----------	----------	707
Bos_taurus	----------	----------	----------	----------	----------	----------	----------	533
Homo_sapiens	----------	----------	----------	----------	----------	----------	----------	450
Microcebus_murinus	----------	----------	----------	----------	----------	----------	----------	428
Myotis_lucifugus	----------	----------	----------	----------	----------	----------	----------	432
Equus_caballus	----------	----------	----------	----------	----------	----------	----------	450
Tupaia_belangeri	----------	----------	----------	----------	----------	----------	----------	498
Loxodonta_africana	----------	----------	----------	----------	----------	----------	----------	460
Murinae	----------	----------	----------	----------	----------	----------	----------	489
Canis_lupus_familiaris	----------	----------	----------	----------	----------	----------	----------	536
Sus_scrofa	----------	----------	----------	----------	----------	----------	----------	454
Choloepus_hoffmanni	----------	----------	----------	----------	----------	----------	----------	444
Vicugna_pacos	----------	----------	----------	----------	----------	----------	----------	474
Gavialis_gangeticus	----------	----------	----------	----------	----------	----------	----------	470
Mammalia	----------	----------	----------	----------	----------	----------	----------	1337
Passeroidea	----------	----------	----------	----------	----------	----------	----------	641
Takifugu	----------	----------	----------	----------	----------	----------	----------	629
Pteropus_vampyrus	----------	----------	----------	----------	----------	----------	----------	472
Ornithorhynchus	----------	----------	----------	----------	----------	----------	----------	2785
Oryctolagus_cuniculus	----------	----------	----------	----------	----------	----------	----------	494
Gallus_gallus	----------	----------	----------	----------	----------	----------	----------	508
Hominidae	ANNCCCNANN	NTNNCNANNA	NCNNNCNCNN	NNCTGAANNN	CNGTTGTCCT	ATATAGACGA	ACNGNTGTGG	5632
Metatheria	----------	----------	----------	----------	----------	----------	----------	569
Rodentia	AGNNNCTANN	TACNTTNNCT	TNNGCTNNCT	NTNGCNCGCC	NNNANCAGCN	NTNNNNTNNN	CNNNTNNNNN	3222
Monodelphis	----------	----------	----------	----------	----------	----------	----------	857
Consensus	----------	----------	----------	----------	----------	----------	----------	

Conservation (100% – 0%)

		9,400		9,420		9,440		
Dipodomys_ordii	----------	----------	----------	----------	----------	----------	----------	597
Laurasiatheria	----------	----------	----------	----------	----------	----------	----------	361
Eutheria	----------	----------	----------	----------	----------	----------	----------	452
Tarsius_syrichta	----------	----------	----------	----------	----------	----------	----------	487
Muridae	CCAGCCGACC	TCCGAGTCCA	CCACCAGTTT	CCCCTTGTTC	GAGTCCAGTG	ATACTGTCTG	GGTCAAGCAG	3768
Gasterosteus_aculeatus	----------	----------	----------	----------	----------	----------	----------	2399
Felis_silvestris	----------	----------	----------	----------	----------	----------	----------	505
Rattus	----------	----------	----------	----------	----------	----------	----------	589
Papio_hamadryas	TAACATGGCA	TACACTTATC	TACCCACGAA	CTGGACGGGG	CTTTGCGTCC	TAGCAACTCT	CCTCCCCGAC	6735
Pan_troglodytes	----------	----------	----------	----------	----------	----------	----------	4138
Ailuropoda_melanoleuca	----------	----------	----------	----------	----------	----------	----------	462
Cavia_porcellus	----------	----------	----------	----------	----------	----------	----------	527
Ictidomys_tridecemlineatus	----------	----------	----------	----------	----------	----------	----------	530
Canis	----------	----------	----------	----------	----------	----------	----------	479
Felis_catus	----------	----------	----------	----------	----------	----------	----------	423
Ochotona_princeps	----------	----------	----------	----------	----------	----------	----------	515
Cercopithecidae	----------	----------	----------	----------	----------	----------	----------	2243
Carnivora	----------	----------	----------	----------	----------	----------	----------	421
Mus	----------	----------	----------	----------	----------	----------	----------	2488
Mus_musculus	----------	----------	----------	----------	----------	----------	----------	478
Rattus_norvegicus	----------	----------	----------	----------	----------	----------	----------	420
Tursiops_truncatus	----------	----------	----------	----------	----------	----------	----------	518
Procavia_capensis	----------	----------	----------	----------	----------	----------	----------	442
Primates	----------	----------	----------	----------	----------	----------	----------	671
Echinops_telfairi	----------	----------	----------	----------	----------	----------	----------	473
Sorex_araneus	----------	----------	----------	----------	----------	----------	----------	494
Canidae	----------	----------	----------	----------	----------	----------	----------	550
Otolemur_garnettii	----------	----------	----------	----------	----------	----------	----------	413
Gallus	----------	----------	----------	----------	----------	----------	----------	633
Callithrix_jacchus	----------	----------	----------	----------	----------	----------	----------	495
Crocodylus_porosus	----------	----------	----------	----------	----------	----------	----------	527
Erinaceus_europaeus	----------	----------	----------	----------	----------	----------	----------	420
rnd-6_family-7426-LTR-ERV1	----------	----------	----------	----------	----------	----------	----------	1692
Esox_lucius	----------	----------	----------	----------	----------	----------	----------	532
Salmo_salar	----------	----------	----------	----------	----------	----------	----------	801
Didelphidae	----------	----------	----------	----------	----------	----------	----------	642
Latimeria_chalumnae	----------	----------	----------	----------	----------	----------	----------	1766
Monodelphis_domestica	----------	----------	----------	----------	----------	----------	----------	543
Macropus_eugenii	----------	----------	----------	----------	----------	----------	----------	509
Alligator_mississippiensis	----------	----------	----------	----------	----------	----------	----------	566
Chrysemys_picta_bellii	----------	----------	----------	----------	----------	----------	----------	3578
Danio_rerio	----------	----------	----------	----------	----------	----------	----------	699
Taeniopygia	----------	----------	----------	----------	----------	----------	----------	538
Dasypus_novemcinctus	----------	----------	----------	----------	----------	----------	----------	512
LTR_XT	----------	----------	----------	----------	----------	----------	----------	503
Haplorrhini	----------	----------	----------	----------	----------	----------	----------	8801
Catarrhini	----------	----------	----------	----------	----------	----------	----------	7847
Anolis_carolinensis	----------	----------	----------	----------	----------	----------	----------	1184
Murine_leukemia_virus	----------	----------	----------	----------	----------	----------	----------	7615
Phascolarctos_cinereus	----------	----------	----------	----------	----------	----------	----------	7426
Estrildidae	----------	----------	----------	----------	----------	----------	----------	482
Xenopus_Silurana_tropicalis	----------	----------	----------	----------	----------	----------	----------	812
I_XT	----------	----------	----------	----------	----------	----------	----------	7509
Takifugu_rubripes	----------	----------	----------	----------	----------	----------	----------	707
Bos_taurus	----------	----------	----------	----------	----------	----------	----------	533
Homo_sapiens	----------	----------	----------	----------	----------	----------	----------	450
Microcebus_murinus	----------	----------	----------	----------	----------	----------	----------	428
Myotis_lucifugus	----------	----------	----------	----------	----------	----------	----------	432
Equus_caballus	----------	----------	----------	----------	----------	----------	----------	450
Tupaia_belangeri	----------	----------	----------	----------	----------	----------	----------	498
Loxodonta_africana	----------	----------	----------	----------	----------	----------	----------	460
Murinae	----------	----------	----------	----------	----------	----------	----------	489
Canis_lupus_familiaris	----------	----------	----------	----------	----------	----------	----------	536
Sus_scrofa	----------	----------	----------	----------	----------	----------	----------	454
Choloepus_hoffmanni	----------	----------	----------	----------	----------	----------	----------	444
Vicugna_pacos	----------	----------	----------	----------	----------	----------	----------	474
Gavialis_gangeticus	----------	----------	----------	----------	----------	----------	----------	470
Mammalia	----------	----------	----------	----------	----------	----------	----------	1337
Passeroidea	----------	----------	----------	----------	----------	----------	----------	641
Takifugu	----------	----------	----------	----------	----------	----------	----------	629
Pteropus_vampyrus	----------	----------	----------	----------	----------	----------	----------	472
Ornithorhynchus	----------	----------	----------	----------	----------	----------	----------	2785
Oryctolagus_cuniculus	----------	----------	----------	----------	----------	----------	----------	494
Gallus_gallus	----------	----------	----------	----------	----------	----------	----------	508
Hominidae	CAAGGCTCCC	TCCAGANNTT	NNACCNNCAT	G----ANNNT	NGNNNNNNNN	GNNNNGTCAC	NNNTCTNNNC	5698
Metatheria	----------	----------	----------	----------	----------	----------	----------	569
Rodentia	TNTGANNTTC	AGNANACCTN	TNNNNAATNG	NTNAANCNNN	NNNNNCATNT	NNTNNNTNNG	NAGACNNCTN	3292
Monodelphis	----------	----------	----------	----------	----------	----------	----------	857
Consensus	----------	----------	----------	----------	----------	----------	----------	

Conservation (100% / 0%)

	9,460		9,480		9,500		9,520	
Dipodomys_ordii	----------	----------	----------	----------	----------	----------	----------	597
Laurasiatheria	----------	----------	----------	----------	----------	----------	----------	361
Eutheria	----------	----------	----------	----------	----------	----------	----------	452
Tarsius_syrichta	----------	----------	----------	----------	----------	----------	----------	487
Muridae	GTAGCCAAAG	GGGCGCTGGA	ACCAACTTGG	AAAGGACCCT	ACACGGTGAT	TCTCTCCACT	CCTACGGCTG	3838
Gasterosteus_aculeatus	----------	----------	----------	----------	----------	----------	----------	2399
Felis_silvestris	----------	----------	----------	----------	----------	----------	----------	505
Rattus	----------	----------	----------	----------	----------	----------	----------	589
Papio_hamadryas	ATTGACATCA	TTCCCGGAGA	TGAACCGGTC	CCCATCCCTG	CTATTGATCA	TTTTATATAT	AGACCTAAAC	6805
Pan_troglodytes	----------	----------	----------	----------	----------	----------	----------	4138
Ailuropoda_melanoleuca	----------	----------	----------	----------	----------	----------	----------	462
Cavia_porcellus	----------	----------	----------	----------	----------	----------	----------	527
Ictidomys_tridecemlineatus	----------	----------	----------	----------	----------	----------	----------	530
Canis	----------	----------	----------	----------	----------	----------	----------	479
Felis_catus	----------	----------	----------	----------	----------	----------	----------	423
Ochotona_princeps	----------	----------	----------	----------	----------	----------	----------	515
Cercopithecidae	----------	----------	----------	----------	----------	----------	----------	2243
Carnivora	----------	----------	----------	----------	----------	----------	----------	421
Mus	----------	----------	----------	----------	----------	----------	----------	2488
Mus_musculus	----------	----------	----------	----------	----------	----------	----------	478
Rattus_norvegicus	----------	----------	----------	----------	----------	----------	----------	420
Tursiops_truncatus	----------	----------	----------	----------	----------	----------	----------	518
Procavia_capensis	----------	----------	----------	----------	----------	----------	----------	442
Primates	----------	----------	----------	----------	----------	----------	----------	671
Echinops_telfairi	----------	----------	----------	----------	----------	----------	----------	473
Sorex_araneus	----------	----------	----------	----------	----------	----------	----------	494
Canidae	----------	----------	----------	----------	----------	----------	----------	550
Otolemur_garnettii	----------	----------	----------	----------	----------	----------	----------	413
Gallus	----------	----------	----------	----------	----------	----------	----------	633
Callithrix_jacchus	----------	----------	----------	----------	----------	----------	----------	495
Crocodylus_porosus	----------	----------	----------	----------	----------	----------	----------	527
Erinaceus_europaeus	----------	----------	----------	----------	----------	----------	----------	420
rnd-6_family-7426-LTR-ERV1	----------	----------	----------	----------	----------	----------	----------	1692
Esox_lucius	----------	----------	----------	----------	----------	----------	----------	532
Salmo_salar	----------	----------	----------	----------	----------	----------	----------	801
Didelphidae	----------	----------	----------	----------	----------	----------	----------	642
Latimeria_chalumnae	----------	----------	----------	----------	----------	----------	----------	1766
Monodelphis_domestica	----------	----------	----------	----------	----------	----------	----------	543
Macropus_eugenii	----------	----------	----------	----------	----------	----------	----------	509
Alligator_mississippiensis	----------	----------	----------	----------	----------	----------	----------	566
Chrysemys_picta_bellii	----------	----------	----------	----------	----------	----------	----------	3578
Danio_rerio	----------	----------	----------	----------	----------	----------	----------	699
Taeniopygia	----------	----------	----------	----------	----------	----------	----------	538
Dasypus_novemcinctus	----------	----------	----------	----------	----------	----------	----------	512
LTR_XT	----------	----------	----------	----------	----------	----------	----------	503
Haplorrhini	----------	----------	----------	----------	----------	----------	----------	8801
Catarrhini	----------	----------	----------	----------	----------	----------	----------	7847
Anolis_carolinensis	----------	----------	----------	----------	----------	----------	----------	1184
Murine_leukemia_virus	----------	----------	----------	----------	----------	----------	----------	7615
Phascolarctos_cinereus	----------	----------	----------	----------	----------	----------	----------	7426
Estrildidae	----------	----------	----------	----------	----------	----------	----------	482
Xenopus_Silurana_tropicalis	----------	----------	----------	----------	----------	----------	----------	812
I_XT	----------	----------	----------	----------	----------	----------	----------	7509
Takifugu_rubripes	----------	----------	----------	----------	----------	----------	----------	707
Bos_taurus	----------	----------	----------	----------	----------	----------	----------	533
Homo_sapiens	----------	----------	----------	----------	----------	----------	----------	450
Microcebus_murinus	----------	----------	----------	----------	----------	----------	----------	428
Myotis_lucifugus	----------	----------	----------	----------	----------	----------	----------	432
Equus_caballus	----------	----------	----------	----------	----------	----------	----------	450
Tupaia_belangeri	----------	----------	----------	----------	----------	----------	----------	498
Loxodonta_africana	----------	----------	----------	----------	----------	----------	----------	460
Murinae	----------	----------	----------	----------	----------	----------	----------	489
Canis_lupus_familiaris	----------	----------	----------	----------	----------	----------	----------	536
Sus_scrofa	----------	----------	----------	----------	----------	----------	----------	454
Choloepus_hoffmanni	----------	----------	----------	----------	----------	----------	----------	444
Vicugna_pacos	----------	----------	----------	----------	----------	----------	----------	474
Gavialis_gangeticus	----------	----------	----------	----------	----------	----------	----------	470
Mammalia	----------	----------	----------	----------	----------	----------	----------	1337
Passeroidea	----------	----------	----------	----------	----------	----------	----------	641
Takifugu	----------	----------	----------	----------	----------	----------	----------	629
Pteropus_vampyrus	----------	----------	----------	----------	----------	----------	----------	472
Ornithorhynchus	----------	----------	----------	----------	----------	----------	----------	2785
Oryctolagus_cuniculus	----------	----------	----------	----------	----------	----------	----------	494
Gallus_gallus	----------	----------	----------	----------	----------	----------	----------	508
Hominidae	TNNCACACNN	GNNNNNGTNG	ACNNNCNNCC	TCNCNGNNNN	CNNNNCNNNG	NNNNTTTTNN	CNTCACTGAG	5768
Metatheria	----------	----------	----------	----------	----------	----------	----------	569
Rodentia	NCNNNCANNN	NNCCAAAAGN	NNANANTNNN	NNCNGATANC	ANCTANNNCN	NNTNNNAAAN	CNNCNNNCAN	3362
Monodelphis	----------	----------	----------	----------	----------	----------	----------	857
Consensus	----------	----------	----------	----------	----------	----------	----------	

Conservation

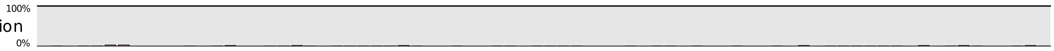

		9,540		9,560		9,580		
Dipodomys_ordii	----------	----------	----------	----------	----------	----------	----------	597
Laurasiatheria	----------	----------	----------	----------	----------	----------	----------	361
Eutheria	----------	----------	----------	----------	----------	----------	----------	452
Tarsius_syrichta	----------	----------	----------	----------	----------	----------	----------	487
Muridae	TGAAGGTAGC	TGGCATTACA	CCATGGATCC	ACCACACCCA	GGTCAAGAAA	GCAGAAGCCA	CAGATGCCGC	3908
Gasterosteus_aculeatus	----------	----------	----------	----------	----------	----------	----------	2399
Felis_silvestris	----------	----------	----------	----------	----------	----------	----------	505
Rattus	----------	----------	----------	----------	----------	----------	----------	589
Papio_hamadryas	GGGCCATACA	GTTTATTCCT	TTACTAGCAG	GGCTAGGGAT	CACCGCAGCC	TTCACAACAG	GAGCTACAGG	6875
Pan_troglodytes	----------	----------	----------	----------	----------	----------	----------	4138
Ailuropoda_melanoleuca	----------	----------	----------	----------	----------	----------	----------	462
Cavia_porcellus	----------	----------	----------	----------	----------	----------	----------	527
Ictidomys_tridecemlineatus	----------	----------	----------	----------	----------	----------	----------	530
Canis	----------	----------	----------	----------	----------	----------	----------	479
Felis_catus	----------	----------	----------	----------	----------	----------	----------	423
Ochotona_princeps	----------	----------	----------	----------	----------	----------	----------	515
Cercopithecidae	----------	----------	----------	----------	----------	----------	----------	2243
Carnivora	----------	----------	----------	----------	----------	----------	----------	421
Mus	----------	----------	----------	----------	----------	----------	----------	2488
Mus_musculus	----------	----------	----------	----------	----------	----------	----------	478
Rattus_norvegicus	----------	----------	----------	----------	----------	----------	----------	420
Tursiops_truncatus	----------	----------	----------	----------	----------	----------	----------	518
Procavia_capensis	----------	----------	----------	----------	----------	----------	----------	442
Primates	----------	----------	----------	----------	----------	----------	----------	671
Echinops_telfairi	----------	----------	----------	----------	----------	----------	----------	473
Sorex_araneus	----------	----------	----------	----------	----------	----------	----------	494
Canidae	----------	----------	----------	----------	----------	----------	----------	550
Otolemur_garnettii	----------	----------	----------	----------	----------	----------	----------	413
Gallus	----------	----------	----------	----------	----------	----------	----------	633
Callithrix_jacchus	----------	----------	----------	----------	----------	----------	----------	495
Crocodylus_porosus	----------	----------	----------	----------	----------	----------	----------	527
Erinaceus_europaeus	----------	----------	----------	----------	----------	----------	----------	420
rnd-6_family-7426-LTR-ERV1	----------	----------	----------	----------	----------	----------	----------	1692
Esox_lucius	----------	----------	----------	----------	----------	----------	----------	532
Salmo_salar	----------	----------	----------	----------	----------	----------	----------	801
Didelphidae	----------	----------	----------	----------	----------	----------	----------	642
Latimeria_chalumnae	----------	----------	----------	----------	----------	----------	----------	1766
Monodelphis_domestica	----------	----------	----------	----------	----------	----------	----------	543
Macropus_eugenii	----------	----------	----------	----------	----------	----------	----------	509
Alligator_mississippiensis	----------	----------	----------	----------	----------	----------	----------	566
Chrysemys_picta_bellii	----------	----------	----------	----------	----------	----------	----------	3578
Danio_rerio	----------	----------	----------	----------	----------	----------	----------	699
Taeniopygia	----------	----------	----------	----------	----------	----------	----------	538
Dasypus_novemcinctus	----------	----------	----------	----------	----------	----------	----------	512
LTR_XT	----------	----------	----------	----------	----------	----------	----------	503
Haplorrhini	----------	----------	----------	----------	----------	----------	----------	8801
Catarrhini	----------	----------	----------	----------	----------	----------	----------	7847
Anolis_carolinensis	----------	----------	----------	----------	----------	----------	----------	1184
Murine_leukemia_virus	----------	----------	----------	----------	----------	----------	----------	7615
Phascolarctos_cinereus	----------	----------	----------	----------	----------	----------	----------	7426
Estrildidae	----------	----------	----------	----------	----------	----------	----------	482
Xenopus_Silurana_tropicalis	----------	----------	----------	----------	----------	----------	----------	812
I_XT	----------	----------	----------	----------	----------	----------	----------	7509
Takifugu_rubripes	----------	----------	----------	----------	----------	----------	----------	707
Bos_taurus	----------	----------	----------	----------	----------	----------	----------	533
Homo_sapiens	----------	----------	----------	----------	----------	----------	----------	450
Microcebus_murinus	----------	----------	----------	----------	----------	----------	----------	428
Myotis_lucifugus	----------	----------	----------	----------	----------	----------	----------	432
Equus_caballus	----------	----------	----------	----------	----------	----------	----------	450
Tupaia_belangeri	----------	----------	----------	----------	----------	----------	----------	498
Loxodonta_africana	----------	----------	----------	----------	----------	----------	----------	460
Murinae	----------	----------	----------	----------	----------	----------	----------	489
Canis_lupus_familiaris	----------	----------	----------	----------	----------	----------	----------	536
Sus_scrofa	----------	----------	----------	----------	----------	----------	----------	454
Choloepus_hoffmanni	----------	----------	----------	----------	----------	----------	----------	444
Vicugna_pacos	----------	----------	----------	----------	----------	----------	----------	474
Gavialis_gangeticus	----------	----------	----------	----------	----------	----------	----------	470
Mammalia	----------	----------	----------	----------	----------	----------	----------	1337
Passeroidea	----------	----------	----------	----------	----------	----------	----------	641
Takifugu	----------	----------	----------	----------	----------	----------	----------	629
Pteropus_vampyrus	----------	----------	----------	----------	----------	----------	----------	472
Ornithorhynchus	----------	----------	----------	----------	----------	----------	----------	2785
Oryctolagus_cuniculus	----------	----------	----------	----------	----------	----------	----------	494
Gallus_gallus	----------	----------	----------	----------	----------	----------	----------	508
Hominidae	AATTGGAGTN	CNNNANANGN	NNTCTNCTCN	NGNCTNGTNG	CNANNNCANC	ATNNNCNNNN	NCNNNGTNNC	5838
Metatheria	----------	----------	----------	----------	----------	----------	----------	569
Rodentia	CNTNNNNTNN	CNNNCTCTNT	GATNNANCNA	NNNATNNNTN	TTNANGTTGN	CNNGAGANTT	ATGNAGGCTG	3432
Monodelphis	----------	----------	----------	----------	----------	----------	----------	857
Consensus	----------	----------	----------	----------	----------	----------	----------	

Conservation (100% / 0%)

	9,600		9,620		9,640		9,660	
Dipodomys_ordii	----------	----------	----------	----------	----------	----------	----------	597
Laurasiatheria	----------	----------	----------	----------	----------	----------	----------	361
Eutheria	----------	----------	----------	----------	----------	----------	----------	452
Tarsius_syrichta	----------	----------	----------	----------	----------	----------	----------	487
Muridae	TGCCAAATGG	ACTGTCTCTG	GACTATGGAC	CCATTGAAAT	TAAGGTTTCA	TTGGTCCCCA	GGCCCTTCCA	3978
Gasterosteus_aculeatus	----------	----------	----------	----------	----------	----------	----------	2399
Felis_silvestris	----------	----------	----------	----------	----------	----------	----------	505
Rattus	----------	----------	----------	----------	----------	----------	----------	589
Papio_hamadryas	CCTAGGTGTC	TCTGTGACCC	AATATACAAA	ATTATCTAAT	CAGCTAATTT	CTGATGTACA	AATCTTATCT	6945
Pan_troglodytes	----------	----------	----------	----------	----------	----------	----------	4138
Ailuropoda_melanoleuca	----------	----------	----------	----------	----------	----------	----------	462
Cavia_porcellus	----------	----------	----------	----------	----------	----------	----------	527
Ictidomys_tridecemlineatus	----------	----------	----------	----------	----------	----------	----------	530
Canis	----------	----------	----------	----------	----------	----------	----------	479
Felis_catus	----------	----------	----------	----------	----------	----------	----------	423
Ochotona_princeps	----------	----------	----------	----------	----------	----------	----------	515
Cercopithecidae	----------	----------	----------	----------	----------	----------	----------	2243
Carnivora	----------	----------	----------	----------	----------	----------	----------	421
Mus	----------	----------	----------	----------	----------	----------	----------	2488
Mus_musculus	----------	----------	----------	----------	----------	----------	----------	478
Rattus_norvegicus	----------	----------	----------	----------	----------	----------	----------	420
Tursiops_truncatus	----------	----------	----------	----------	----------	----------	----------	518
Procavia_capensis	----------	----------	----------	----------	----------	----------	----------	442
Primates	----------	----------	----------	----------	----------	----------	----------	671
Echinops_telfairi	----------	----------	----------	----------	----------	----------	----------	473
Sorex_araneus	----------	----------	----------	----------	----------	----------	----------	494
Canidae	----------	----------	----------	----------	----------	----------	----------	550
Otolemur_garnettii	----------	----------	----------	----------	----------	----------	----------	413
Gallus	----------	----------	----------	----------	----------	----------	----------	633
Callithrix_jacchus	----------	----------	----------	----------	----------	----------	----------	495
Crocodylus_porosus	----------	----------	----------	----------	----------	----------	----------	527
Erinaceus_europaeus	----------	----------	----------	----------	----------	----------	----------	420
rnd-6_family-7426-LTR-ERV1	----------	----------	----------	----------	----------	----------	----------	1692
Esox_lucius	----------	----------	----------	----------	----------	----------	----------	532
Salmo_salar	----------	----------	----------	----------	----------	----------	----------	801
Didelphidae	----------	----------	----------	----------	----------	----------	----------	642
Latimeria_chalumnae	----------	----------	----------	----------	----------	----------	----------	1766
Monodelphis_domestica	----------	----------	----------	----------	----------	----------	----------	543
Macropus_eugenii	----------	----------	----------	----------	----------	----------	----------	509
Alligator_mississippiensis	----------	----------	----------	----------	----------	----------	----------	566
Chrysemys_picta_bellii	----------	----------	----------	----------	----------	----------	----------	3578
Danio_rerio	----------	----------	----------	----------	----------	----------	----------	699
Taeniopygia	----------	----------	----------	----------	----------	----------	----------	538
Dasypus_novemcinctus	----------	----------	----------	----------	----------	----------	----------	512
LTR_XT	----------	----------	----------	----------	----------	----------	----------	503
Haplorrhini	----------	----------	----------	----------	----------	----------	----------	8801
Catarrhini	----------	----------	----------	----------	----------	----------	----------	7847
Anolis_carolinensis	----------	----------	----------	----------	----------	----------	----------	1184
Murine_leukemia_virus	----------	----------	----------	----------	----------	----------	----------	7615
Phascolarctos_cinereus	----------	----------	----------	----------	----------	----------	----------	7426
Estrildidae	----------	----------	----------	----------	----------	----------	----------	482
Xenopus_Silurana_tropicalis	----------	----------	----------	----------	----------	----------	----------	812
I_XT	----------	----------	----------	----------	----------	----------	----------	7509
Takifugu_rubripes	----------	----------	----------	----------	----------	----------	----------	707
Bos_taurus	----------	----------	----------	----------	----------	----------	----------	533
Homo_sapiens	----------	----------	----------	----------	----------	----------	----------	450
Microcebus_murinus	----------	----------	----------	----------	----------	----------	----------	428
Myotis_lucifugus	----------	----------	----------	----------	----------	----------	----------	432
Equus_caballus	----------	----------	----------	----------	----------	----------	----------	450
Tupaia_belangeri	----------	----------	----------	----------	----------	----------	----------	498
Loxodonta_africana	----------	----------	----------	----------	----------	----------	----------	460
Murinae	----------	----------	----------	----------	----------	----------	----------	489
Canis_lupus_familiaris	----------	----------	----------	----------	----------	----------	----------	536
Sus_scrofa	----------	----------	----------	----------	----------	----------	----------	454
Choloepus_hoffmanni	----------	----------	----------	----------	----------	----------	----------	444
Vicugna_pacos	----------	----------	----------	----------	----------	----------	----------	474
Gavialis_gangeticus	----------	----------	----------	----------	----------	----------	----------	470
Mammalia	----------	----------	----------	----------	----------	----------	----------	1337
Passeroidea	----------	----------	----------	----------	----------	----------	----------	641
Takifugu	----------	----------	----------	----------	----------	----------	----------	629
Pteropus_vampyrus	----------	----------	----------	----------	----------	----------	----------	472
Ornithorhynchus	----------	----------	----------	----------	----------	----------	----------	2785
Oryctolagus_cuniculus	----------	----------	----------	----------	----------	----------	----------	494
Gallus_gallus	----------	----------	----------	----------	----------	----------	----------	508
Hominidae	ANNCACNCAN	NNNNTNCCNN	GNTCTAAAAT	TCTNTTNNNN	NGNCNCNNNN	NGANNANNCC	CTGCACTNTG	5908
Metatheria	----------	----------	----------	----------	----------	----------	----------	569
Rodentia	CCNTTNTTNG	TNCTGTANNA	NANATNANGC	AANTTCANGA	ANNNGCTTCN	ACNTTNNAAN	NNNGAATNNG	3502
Monodelphis	----------	----------	----------	----------	----------	----------	----------	857
Consensus	----------	----------	----------	----------	----------	----------	----------	

Conservation
100%
0%

		9,680		9,700		9,720		
Dipodomys_ordii	----------	----------	----------	----------	----------	----------	----------	597
Laurasiatheria	----------	----------	----------	----------	----------	----------	----------	361
Eutheria	----------	----------	----------	----------	----------	----------	----------	452
Tarsius_syrichta	----------	----------	----------	----------	----------	----------	----------	487
Muridae	CTCCCTGCCC	CTCTACCTCC	TCCTGAGTCT	TATGTTTGGT	ATGAATACAG	GGTCATGGTC	CAACCCCCAC	4048
Gasterosteus_aculeatus	----------	----------	----------	----------	----------	----------	----------	2399
Felis_silvestris	----------	----------	----------	----------	----------	----------	----------	505
Rattus	----------	----------	----------	----------	----------	----------	----------	589
Papio_hamadryas	AGCACCATAC	AAGATCTGCA	AGATCAAGTA	GACTCATTAG	CCGAAGTGGT	TCTCCAGAAC	AGAAGGGGGC	7015
Pan_troglodytes	----------	----------	----------	----------	----------	----------	----------	4138
Ailuropoda_melanoleuca	----------	----------	----------	----------	----------	----------	----------	462
Cavia_porcellus	----------	----------	----------	----------	----------	----------	----------	527
Ictidomys_tridecemlineatus	----------	----------	----------	----------	----------	----------	----------	530
Canis	----------	----------	----------	----------	----------	----------	----------	479
Felis_catus	----------	----------	----------	----------	----------	----------	----------	423
Ochotona_princeps	----------	----------	----------	----------	----------	----------	----------	515
Cercopithecidae	----------	----------	----------	----------	----------	----------	----------	2243
Carnivora	----------	----------	----------	----------	----------	----------	----------	421
Mus	----------	----------	----------	----------	----------	----------	----------	2488
Mus_musculus	----------	----------	----------	----------	----------	----------	----------	478
Rattus_norvegicus	----------	----------	----------	----------	----------	----------	----------	420
Tursiops_truncatus	----------	----------	----------	----------	----------	----------	----------	518
Procavia_capensis	----------	----------	----------	----------	----------	----------	----------	442
Primates	----------	----------	----------	----------	----------	----------	----------	671
Echinops_telfairi	----------	----------	----------	----------	----------	----------	----------	473
Sorex_araneus	----------	----------	----------	----------	----------	----------	----------	494
Canidae	----------	----------	----------	----------	----------	----------	----------	550
Otolemur_garnettii	----------	----------	----------	----------	----------	----------	----------	413
Gallus	----------	----------	----------	----------	----------	----------	----------	633
Callithrix_jacchus	----------	----------	----------	----------	----------	----------	----------	495
Crocodylus_porosus	----------	----------	----------	----------	----------	----------	----------	527
Erinaceus_europaeus	----------	----------	----------	----------	----------	----------	----------	420
rnd-6_family-7426-LTR-ERV1	----------	----------	----------	----------	----------	----------	----------	1692
Esox_lucius	----------	----------	----------	----------	----------	----------	----------	532
Salmo_salar	----------	----------	----------	----------	----------	----------	----------	801
Didelphidae	----------	----------	----------	----------	----------	----------	----------	642
Latimeria_chalumnae	----------	----------	----------	----------	----------	----------	----------	1766
Monodelphis_domestica	----------	----------	----------	----------	----------	----------	----------	543
Macropus_eugenii	----------	----------	----------	----------	----------	----------	----------	509
Alligator_mississippiensis	----------	----------	----------	----------	----------	----------	----------	566
Chrysemys_picta_bellii	----------	----------	----------	----------	----------	----------	----------	3578
Danio_rerio	----------	----------	----------	----------	----------	----------	----------	699
Taeniopygia	----------	----------	----------	----------	----------	----------	----------	538
Dasypus_novemcinctus	----------	----------	----------	----------	----------	----------	----------	512
LTR_XT	----------	----------	----------	----------	----------	----------	----------	503
Haplorrhini	----------	----------	----------	----------	----------	----------	----------	8801
Catarrhini	----------	----------	----------	----------	----------	----------	----------	7847
Anolis_carolinensis	----------	----------	----------	----------	----------	----------	----------	1184
Murine_leukemia_virus	----------	----------	----------	----------	----------	----------	----------	7615
Phascolarctos_cinereus	----------	----------	----------	----------	----------	----------	----------	7426
Estrildidae	----------	----------	----------	----------	----------	----------	----------	482
Xenopus_Silurana_tropicalis	----------	----------	----------	----------	----------	----------	----------	812
I_XT	----------	----------	----------	----------	----------	----------	----------	7509
Takifugu_rubripes	----------	----------	----------	----------	----------	----------	----------	707
Bos_taurus	----------	----------	----------	----------	----------	----------	----------	533
Homo_sapiens	----------	----------	----------	----------	----------	----------	----------	450
Microcebus_murinus	----------	----------	----------	----------	----------	----------	----------	428
Myotis_lucifugus	----------	----------	----------	----------	----------	----------	----------	432
Equus_caballus	----------	----------	----------	----------	----------	----------	----------	450
Tupaia_belangeri	----------	----------	----------	----------	----------	----------	----------	498
Loxodonta_africana	----------	----------	----------	----------	----------	----------	----------	460
Murinae	----------	----------	----------	----------	----------	----------	----------	489
Canis_lupus_familiaris	----------	----------	----------	----------	----------	----------	----------	536
Sus_scrofa	----------	----------	----------	----------	----------	----------	----------	454
Choloepus_hoffmanni	----------	----------	----------	----------	----------	----------	----------	444
Vicugna_pacos	----------	----------	----------	----------	----------	----------	----------	474
Gavialis_gangeticus	----------	----------	----------	----------	----------	----------	----------	470
Mammalia	----------	----------	----------	----------	----------	----------	----------	1337
Passeroidea	----------	----------	----------	----------	----------	----------	----------	641
Takifugu	----------	----------	----------	----------	----------	----------	----------	629
Pteropus_vampyrus	----------	----------	----------	----------	----------	----------	----------	472
Ornithorhynchus	----------	----------	----------	----------	----------	----------	----------	2785
Oryctolagus_cuniculus	----------	----------	----------	----------	----------	----------	----------	494
Gallus_gallus	----------	----------	----------	----------	----------	----------	----------	508
Hominidae	CNTNNTGTNN	NATNNANGTN	ACNCCAANNN	CNATACCNNC	T-GGGTCAAA	GAAANTNNAN	NNNNNCNNTN	5977
Metatheria	----------	----------	----------	----------	----------	----------	----------	569
Rodentia	GGNTNANNTA	NATNNNNNTT	NNNGNNCCAT	GGNNNCNCAN	NTNGGNNNCN	GNANNNACTG	GTANNNNTTA	3572
Monodelphis	----------	----------	----------	----------	----------	----------	----------	857
Consensus	----------	----------	----------	----------	----------	----------	----------	

Conservation 100% 0%

9,740 9,760 9,780 9,800

Dipodomys_ordii	----------	----------	----------	----------	----------	----------	----------	597
Laurasiatheria	----------	----------	----------	----------	----------	----------	----------	361
Eutheria	----------	----------	----------	----------	----------	----------	----------	452
Tarsius_syrichta	----------	----------	----------	----------	----------	----------	----------	487
Muridae	NGCCCTCAGG	CCTGGACCTG	GAAACTGAAG	AAGACAGATA	CAGGGAAGGT	ATTAGCTAGC	GTGACTACAG	4118
Gasterosteus_aculeatus	----------	----------	----------	----------	----------	----------	----------	2399
Felis_silvestris	----------	----------	----------	----------	----------	----------	----------	505
Rattus	----------	----------	----------	----------	----------	----------	----------	589
Papio_hamadryas	TAGATCTACT	TACAGCAGAA	CAAGGAGGAA	TCTGTTTAGC	CCTGCAAGAA	AAATGCTGCT	TTTATGTTAA	7085
Pan_troglodytes	----------	----------	----------	----------	----------	----------	----------	4138
Ailuropoda_melanoleuca	----------	----------	----------	----------	----------	----------	----------	462
Cavia_porcellus	----------	----------	----------	----------	----------	----------	----------	527
Ictidomys_tridecemlineatus	----------	----------	----------	----------	----------	----------	----------	530
Canis	----------	----------	----------	----------	----------	----------	----------	479
Felis_catus	----------	----------	----------	----------	----------	----------	----------	423
Ochotona_princeps	----------	----------	----------	----------	----------	----------	----------	515
Cercopithecidae	----------	----------	----------	----------	----------	----------	----------	2243
Carnivora	----------	----------	----------	----------	----------	----------	----------	421
Mus	----------	----------	----------	----------	----------	----------	----------	2488
Mus_musculus	----------	----------	----------	----------	----------	----------	----------	478
Rattus_norvegicus	----------	----------	----------	----------	----------	----------	----------	420
Tursiops_truncatus	----------	----------	----------	----------	----------	----------	----------	518
Procavia_capensis	----------	----------	----------	----------	----------	----------	----------	442
Primates	----------	----------	----------	----------	----------	----------	----------	671
Echinops_telfairi	----------	----------	----------	----------	----------	----------	----------	473
Sorex_araneus	----------	----------	----------	----------	----------	----------	----------	494
Canidae	----------	----------	----------	----------	----------	----------	----------	550
Otolemur_garnettii	----------	----------	----------	----------	----------	----------	----------	413
Gallus	----------	----------	----------	----------	----------	----------	----------	633
Callithrix_jacchus	----------	----------	----------	----------	----------	----------	----------	495
Crocodylus_porosus	----------	----------	----------	----------	----------	----------	----------	527
Erinaceus_europaeus	----------	----------	----------	----------	----------	----------	----------	420
rnd-6_family-7426-LTR-ERV1	----------	----------	----------	----------	----------	----------	----------	1692
Esox_lucius	----------	----------	----------	----------	----------	----------	----------	532
Salmo_salar	----------	----------	----------	----------	----------	----------	----------	801
Didelphidae	----------	----------	----------	----------	----------	----------	----------	642
Latimeria_chalumnae	----------	----------	----------	----------	----------	----------	----------	1766
Monodelphis_domestica	----------	----------	----------	----------	----------	----------	----------	543
Macropus_eugenii	----------	----------	----------	----------	----------	----------	----------	509
Alligator_mississippiensis	----------	----------	----------	----------	----------	----------	----------	566
Chrysemys_picta_bellii	----------	----------	----------	----------	----------	----------	----------	3578
Danio_rerio	----------	----------	----------	----------	----------	----------	----------	699
Taeniopygia	----------	----------	----------	----------	----------	----------	----------	538
Dasypus_novemcinctus	----------	----------	----------	----------	----------	----------	----------	512
LTR_XT	----------	----------	----------	----------	----------	----------	----------	503
Haplorrhini	----------	----------	----------	----------	----------	----------	----------	8801
Catarrhini	----------	----------	----------	----------	----------	----------	----------	7847
Anolis_carolinensis	----------	----------	----------	----------	----------	----------	----------	1184
Murine_leukemia_virus	----------	----------	----------	----------	----------	----------	----------	7615
Phascolarctos_cinereus	----------	----------	----------	----------	----------	----------	----------	7426
Estrildidae	----------	----------	----------	----------	----------	----------	----------	482
Xenopus_Silurana_tropicalis	----------	----------	----------	----------	----------	----------	----------	812
I_XT	----------	----------	----------	----------	----------	----------	----------	7509
Takifugu_rubripes	----------	----------	----------	----------	----------	----------	----------	707
Bos_taurus	----------	----------	----------	----------	----------	----------	----------	533
Homo_sapiens	----------	----------	----------	----------	----------	----------	----------	450
Microcebus_murinus	----------	----------	----------	----------	----------	----------	----------	428
Myotis_lucifugus	----------	----------	----------	----------	----------	----------	----------	432
Equus_caballus	----------	----------	----------	----------	----------	----------	----------	450
Tupaia_belangeri	----------	----------	----------	----------	----------	----------	----------	498
Loxodonta_africana	----------	----------	----------	----------	----------	----------	----------	460
Murinae	----------	----------	----------	----------	----------	----------	----------	489
Canis_lupus_familiaris	----------	----------	----------	----------	----------	----------	----------	536
Sus_scrofa	----------	----------	----------	----------	----------	----------	----------	454
Choloepus_hoffmanni	----------	----------	----------	----------	----------	----------	----------	444
Vicugna_pacos	----------	----------	----------	----------	----------	----------	----------	474
Gavialis_gangeticus	----------	----------	----------	----------	----------	----------	----------	470
Mammalia	----------	----------	----------	----------	----------	----------	----------	1337
Passeroidea	----------	----------	----------	----------	----------	----------	----------	641
Takifugu	----------	----------	----------	----------	----------	----------	----------	629
Pteropus_vampyrus	----------	----------	----------	----------	----------	----------	----------	472
Ornithorhynchus	----------	----------	----------	----------	----------	----------	----------	2785
Oryctolagus_cuniculus	----------	----------	----------	----------	----------	----------	----------	494
Gallus_gallus	----------	----------	----------	----------	----------	----------	----------	508
Hominidae	GCACTGNNNN	AATANNNATG	NNNNNNNAAN	TNNNNNNNNN	ANNNNNNCNG	-ACCCANNGN	NNTNTGTCNN	6046
Metatheria	----------	----------	----------	----------	----------	----------	----------	569
Rodentia	NTNNNNNNNN	NATNANAGCN	GNNNNTNNNG	CNNNNNCAAA	ATACAGAGGG	TACACATCCC	ATTCTCTCAA	3642
Monodelphis	----------	----------	----------	----------	----------	----------	----------	857
Consensus	----------	----------	----------	----------	----------	----------	----------	

Conservation 100% 0%

9,820 9,840 9,860

Dipodomys_ordii	---------- ---------- ---------- ---------- ---------- ---------- ----------	597
Laurasiatheria	---------- ---------- ---------- ---------- ---------- ---------- ----------	361
Eutheria	---------- ---------- ---------- ---------- ---------- ---------- ----------	452
Tarsius_syrichta	---------- ---------- ---------- ---------- ---------- ---------- ----------	487
Muridae	ACCAGCAACC CATATTCATC TTGACCTTTG CTCACTGATG CCCACTTTAA GTGTCTGGTT GTATTGGAGA	4188
Gasterosteus_aculeatus	---------- ---------- ---------- ---------- ---------- ---------- ----------	2399
Felis_silvestris	---------- ---------- ---------- ---------- ---------- ---------- ----------	505
Rattus	---------- ---------- ---------- ---------- ---------- ---------- ----------	589
Papio_hamadryas	CAAGTCAGGG ATTGTGAGAG ACAAAATAAA AACCTTACAA GAAGAACTAG AAAGACGTAG AAAAGATCTA	7155
Pan_troglodytes	---------- ---------- ---------- ---------- ---------- ---------- ----------	4138
Ailuropoda_melanoleuca	---------- ---------- ---------- ---------- ---------- ---------- ----------	462
Cavia_porcellus	---------- ---------- ---------- ---------- ---------- ---------- ----------	527
Ictidomys_tridecemlineatus	---------- ---------- ---------- ---------- ---------- ---------- ----------	530
Canis	---------- ---------- ---------- ---------- ---------- ---------- ----------	479
Felis_catus	---------- ---------- ---------- ---------- ---------- ---------- ----------	423
Ochotona_princeps	---------- ---------- ---------- ---------- ---------- ---------- ----------	515
Cercopithecidae	---------- ---------- ---------- ---------- ---------- ---------- ----------	2243
Carnivora	---------- ---------- ---------- ---------- ---------- ---------- ----------	421
Mus	---------- ---------- ---------- ---------- ---------- ---------- ----------	2488
Mus_musculus	---------- ---------- ---------- ---------- ---------- ---------- ----------	478
Rattus_norvegicus	---------- ---------- ---------- ---------- ---------- ---------- ----------	420
Tursiops_truncatus	---------- ---------- ---------- ---------- ---------- ---------- ----------	518
Procavia_capensis	---------- ---------- ---------- ---------- ---------- ---------- ----------	442
Primates	---------- ---------- ---------- ---------- ---------- ---------- ----------	671
Echinops_telfairi	---------- ---------- ---------- ---------- ---------- ---------- ----------	473
Sorex_araneus	---------- ---------- ---------- ---------- ---------- ---------- ----------	494
Canidae	---------- ---------- ---------- ---------- ---------- ---------- ----------	550
Otolemur_garnettii	---------- ---------- ---------- ---------- ---------- ---------- ----------	413
Gallus	---------- ---------- ---------- ---------- ---------- ---------- ----------	633
Callithrix_jacchus	---------- ---------- ---------- ---------- ---------- ---------- ----------	495
Crocodylus_porosus	---------- ---------- ---------- ---------- ---------- ---------- ----------	527
Erinaceus_europaeus	---------- ---------- ---------- ---------- ---------- ---------- ----------	420
rnd-6_family-7426-LTR-ERV1	---------- ---------- ---------- ---------- ---------- ---------- ----------	1692
Esox_lucius	---------- ---------- ---------- ---------- ---------- ---------- ----------	532
Salmo_salar	---------- ---------- ---------- ---------- ---------- ---------- ----------	801
Didelphidae	---------- ---------- ---------- ---------- ---------- ---------- ----------	642
Latimeria_chalumnae	---------- ---------- ---------- ---------- ---------- ---------- ----------	1766
Monodelphis_domestica	---------- ---------- ---------- ---------- ---------- ---------- ----------	543
Macropus_eugenii	---------- ---------- ---------- ---------- ---------- ---------- ----------	509
Alligator_mississippiensis	---------- ---------- ---------- ---------- ---------- ---------- ----------	566
Chrysemys_picta_bellii	---------- ---------- ---------- ---------- ---------- ---------- ----------	3578
Danio_rerio	---------- ---------- ---------- ---------- ---------- ---------- ----------	699
Taeniopygia	---------- ---------- ---------- ---------- ---------- ---------- ----------	538
Dasypus_novemcinctus	---------- ---------- ---------- ---------- ---------- ---------- ----------	512
LTR_XT	---------- ---------- ---------- ---------- ---------- ---------- ----------	503
Haplorrhini	---------- ---------- ---------- ---------- ---------- ---------- ----------	8801
Catarrhini	---------- ---------- ---------- ---------- ---------- ---------- ----------	7847
Anolis_carolinensis	---------- ---------- ---------- ---------- ---------- ---------- ----------	1184
Murine_leukemia_virus	---------- ---------- ---------- ---------- ---------- ---------- ----------	7615
Phascolarctos_cinereus	---------- ---------- ---------- ---------- ---------- ---------- ----------	7426
Estrildidae	---------- ---------- ---------- ---------- ---------- ---------- ----------	482
Xenopus_Silurana_tropicalis	---------- ---------- ---------- ---------- ---------- ---------- ----------	812
I_XT	---------- ---------- ---------- ---------- ---------- ---------- ----------	7509
Takifugu_rubripes	---------- ---------- ---------- ---------- ---------- ---------- ----------	707
Bos_taurus	---------- ---------- ---------- ---------- ---------- ---------- ----------	533
Homo_sapiens	---------- ---------- ---------- ---------- ---------- ---------- ----------	450
Microcebus_murinus	---------- ---------- ---------- ---------- ---------- ---------- ----------	428
Myotis_lucifugus	---------- ---------- ---------- ---------- ---------- ---------- ----------	432
Equus_caballus	---------- ---------- ---------- ---------- ---------- ---------- ----------	450
Tupaia_belangeri	---------- ---------- ---------- ---------- ---------- ---------- ----------	498
Loxodonta_africana	---------- ---------- ---------- ---------- ---------- ---------- ----------	460
Murinae	---------- ---------- ---------- ---------- ---------- ---------- ----------	489
Canis_lupus_familiaris	---------- ---------- ---------- ---------- ---------- ---------- ----------	536
Sus_scrofa	---------- ---------- ---------- ---------- ---------- ---------- ----------	454
Choloepus_hoffmanni	---------- ---------- ---------- ---------- ---------- ---------- ----------	444
Vicugna_pacos	---------- ---------- ---------- ---------- ---------- ---------- ----------	474
Gavialis_gangeticus	---------- ---------- ---------- ---------- ---------- ---------- ----------	470
Mammalia	---------- ---------- ---------- ---------- ---------- ---------- ----------	1337
Passeroidea	---------- ---------- ---------- ---------- ---------- ---------- ----------	641
Takifugu	---------- ---------- ---------- ---------- ---------- ---------- ----------	629
Pteropus_vampyrus	---------- ---------- ---------- ---------- ---------- ---------- ----------	472
Ornithorhynchus	---------- ---------- ---------- ---------- ---------- ---------- ----------	2785
Oryctolagus_cuniculus	---------- ---------- ---------- ---------- ---------- ---------- ----------	494
Gallus_gallus	---------- ---------- ---------- ---------- ---------- ---------- ----------	508
Hominidae	TNNNTCNNNN GNNNNACNGG NCGNCTNTAN CGGATTTNNC CNNCNNANNN CTNNCCNNTN GGACCCCCGC	6116
Metatheria	---------- ---------- ---------- ---------- ---------- ---------- ----------	569
Rodentia	CCCCTAGGTA TAGACACGGT AGAAGAAGTG GTCCAACGCT CCTCAAAGGT CCTTACATCC CTAACATCAC	3712
Monodelphis	---------- ---------- ---------- ---------- ---------- ---------- ----------	857
Consensus	---------- ---------- ---------- ---------- ---------- ---------- ----------	

Conservation
100%
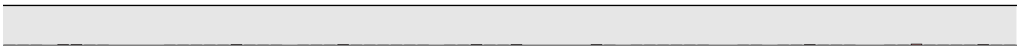
0%

	9,880		9,900		9,920		9,940	
Dipodomys_ordii	----------	----------	----------	----------	----------	----------	----------	597
Laurasiatheria	----------	----------	----------	----------	----------	----------	----------	361
Eutheria	----------	----------	----------	----------	----------	----------	----------	452
Tarsius_syrichta	----------	----------	----------	----------	----------	----------	----------	487
Muridae	AAGGAAAGAT	GTGGGAATTT	ATCTTTTGAG	ATAGGAATAC	AGAGTTTACA	ATTCTATGCA	TGCCCTGCAG	4258
Gasterosteus_aculeatus	----------	----------	----------	----------	----------	----------	----------	2399
Felis_silvestris	----------	----------	----------	----------	----------	----------	----------	505
Rattus	----------	----------	----------	----------	----------	----------	----------	589
Papio_hamadryas	GCTTCCAACC	CACTTTGGAC	TGGGCTTCAA	GGGCTCCTCC	CTTACCTCCT	GCCCTTTCTT	GGCCCTCTAC	7225
Pan_troglodytes	----------	----------	----------	----------	----------	----------	----------	4138
Ailuropoda_melanoleuca	----------	----------	----------	----------	----------	----------	----------	462
Cavia_porcellus	----------	----------	----------	----------	----------	----------	----------	527
Ictidomys_tridecemlineatus	----------	----------	----------	----------	----------	----------	----------	530
Canis	----------	----------	----------	----------	----------	----------	----------	479
Felis_catus	----------	----------	----------	----------	----------	----------	----------	423
Ochotona_princeps	----------	----------	----------	----------	----------	----------	----------	515
Cercopithecidae	----------	----------	----------	----------	----------	----------	----------	2243
Carnivora	----------	----------	----------	----------	----------	----------	----------	421
Mus	----------	----------	----------	----------	----------	----------	----------	2488
Mus_musculus	----------	----------	----------	----------	----------	----------	----------	478
Rattus_norvegicus	----------	----------	----------	----------	----------	----------	----------	420
Tursiops_truncatus	----------	----------	----------	----------	----------	----------	----------	518
Procavia_capensis	----------	----------	----------	----------	----------	----------	----------	442
Primates	----------	----------	----------	----------	----------	----------	----------	671
Echinops_telfairi	----------	----------	----------	----------	----------	----------	----------	473
Sorex_araneus	----------	----------	----------	----------	----------	----------	----------	494
Canidae	----------	----------	----------	----------	----------	----------	----------	550
Otolemur_garnettii	----------	----------	----------	----------	----------	----------	----------	413
Gallus	----------	----------	----------	----------	----------	----------	----------	633
Callithrix_jacchus	----------	----------	----------	----------	----------	----------	----------	495
Crocodylus_porosus	----------	----------	----------	----------	----------	----------	----------	527
Erinaceus_europaeus	----------	----------	----------	----------	----------	----------	----------	420
rnd-6_family-7426-LTR-ERV1	----------	----------	----------	----------	----------	----------	----------	1692
Esox_lucius	----------	----------	----------	----------	----------	----------	----------	532
Salmo_salar	----------	----------	----------	----------	----------	----------	----------	801
Didelphidae	----------	----------	----------	----------	----------	----------	----------	642
Latimeria_chalumnae	----------	----------	----------	----------	----------	----------	----------	1766
Monodelphis_domestica	----------	----------	----------	----------	----------	----------	----------	543
Macropus_eugenii	----------	----------	----------	----------	----------	----------	----------	509
Alligator_mississippiensis	----------	----------	----------	----------	----------	----------	----------	566
Chrysemys_picta_bellii	----------	----------	----------	----------	----------	----------	----------	3578
Danio_rerio	----------	----------	----------	----------	----------	----------	----------	699
Taeniopygia	----------	----------	----------	----------	----------	----------	----------	538
Dasypus_novemcinctus	----------	----------	----------	----------	----------	----------	----------	512
LTR_XT	----------	----------	----------	----------	----------	----------	----------	503
Haplorrhini	----------	----------	----------	----------	----------	----------	----------	8801
Catarrhini	----------	----------	----------	----------	----------	----------	----------	7847
Anolis_carolinensis	----------	----------	----------	----------	----------	----------	----------	1184
Murine_leukemia_virus	----------	----------	----------	----------	----------	----------	----------	7615
Phascolarctos_cinereus	----------	----------	----------	----------	----------	----------	----------	7426
Estrildidae	----------	----------	----------	----------	----------	----------	----------	482
Xenopus_Silurana_tropicalis	----------	----------	----------	----------	----------	----------	----------	812
I_XT	----------	----------	----------	----------	----------	----------	----------	7509
Takifugu_rubripes	----------	----------	----------	----------	----------	----------	----------	707
Bos_taurus	----------	----------	----------	----------	----------	----------	----------	533
Homo_sapiens	----------	----------	----------	----------	----------	----------	----------	450
Microcebus_murinus	----------	----------	----------	----------	----------	----------	----------	428
Myotis_lucifugus	----------	----------	----------	----------	----------	----------	----------	432
Equus_caballus	----------	----------	----------	----------	----------	----------	----------	450
Tupaia_belangeri	----------	----------	----------	----------	----------	----------	----------	498
Loxodonta_africana	----------	----------	----------	----------	----------	----------	----------	460
Murinae	----------	----------	----------	----------	----------	----------	----------	489
Canis_lupus_familiaris	----------	----------	----------	----------	----------	----------	----------	536
Sus_scrofa	----------	----------	----------	----------	----------	----------	----------	454
Choloepus_hoffmanni	----------	----------	----------	----------	----------	----------	----------	444
Vicugna_pacos	----------	----------	----------	----------	----------	----------	----------	474
Gavialis_gangeticus	----------	----------	----------	----------	----------	----------	----------	470
Mammalia	----------	----------	----------	----------	----------	----------	----------	1337
Passeroidea	----------	----------	----------	----------	----------	----------	----------	641
Takifugu	----------	----------	----------	----------	----------	----------	----------	629
Pteropus_vampyrus	----------	----------	----------	----------	----------	----------	----------	472
Ornithorhynchus	----------	----------	----------	----------	----------	----------	----------	2785
Oryctolagus_cuniculus	----------	----------	----------	----------	----------	----------	----------	494
Gallus_gallus	----------	----------	----------	----------	----------	----------	----------	508
Hominidae	NNNNCNNCNG	NTNTNNNTGN	NNNACTATAN	GAGNNNNNNN	CNNNNNTNTN	TCNANTNNNN	ANGANCNNCA	6186
Metatheria	----------	----------	----------	----------	----------	----------	----------	569
Rodentia	CCCTTTTAGA	CGATACCGAT	CTCCCTGTCA	TACCATCTCT	TGTTTCAGAC	CTTAACCTCT	GCATATCAGC	3782
Monodelphis	----------	----------	----------	----------	----------	----------	----------	857
Consensus	----------	----------	----------	----------	----------	----------	----------	

Conservation 100% 0%

		9,960		9,980		10,000		
Dipodomys_ordii	----------	----------	----------	----------	----------	----------	----------	597
Laurasiatheria	----------	----------	----------	----------	----------	----------	----------	361
Eutheria	----------	----------	----------	----------	----------	----------	----------	452
Tarsius_syrichta	----------	----------	----------	----------	----------	----------	----------	487
Muridae	CTGGGCCCTG	GTTGCCAGAA	ACAAGGGAGT	TTCCATTGCA	AAAACTGGGG	TTGTAAAACT	GAGGCTTCCT	4328
Gasterosteus_aculeatus	----------	----------	----------	----------	----------	----------	----------	2399
Felis_silvestris	----------	----------	----------	----------	----------	----------	----------	505
Rattus	----------	----------	----------	----------	----------	----------	----------	589
Papio_hamadryas	TTACCCTCCT	GCTCTTACTC	ACCATTGGGC	CGTGCATTTT	TAACCGTCTA	ACCGCTTTTA	TTAATGATAA	7295
Pan_troglodytes	----------	----------	----------	----------	----------	----------	----------	4138
Ailuropoda_melanoleuca	----------	----------	----------	----------	----------	----------	----------	462
Cavia_porcellus	----------	----------	----------	----------	----------	----------	----------	527
Ictidomys_tridecemlineatus	----------	----------	----------	----------	----------	----------	----------	530
Canis	----------	----------	----------	----------	----------	----------	----------	479
Felis_catus	----------	----------	----------	----------	----------	----------	----------	423
Ochotona_princeps	----------	----------	----------	----------	----------	----------	----------	515
Cercopithecidae	----------	----------	----------	----------	----------	----------	----------	2243
Carnivora	----------	----------	----------	----------	----------	----------	----------	421
Mus	----------	----------	----------	----------	----------	----------	----------	2488
Mus_musculus	----------	----------	----------	----------	----------	----------	----------	478
Rattus_norvegicus	----------	----------	----------	----------	----------	----------	----------	420
Tursiops_truncatus	----------	----------	----------	----------	----------	----------	----------	518
Procavia_capensis	----------	----------	----------	----------	----------	----------	----------	442
Primates	----------	----------	----------	----------	----------	----------	----------	671
Echinops_telfairi	----------	----------	----------	----------	----------	----------	----------	473
Sorex_araneus	----------	----------	----------	----------	----------	----------	----------	494
Canidae	----------	----------	----------	----------	----------	----------	----------	550
Otolemur_garnettii	----------	----------	----------	----------	----------	----------	----------	413
Gallus	----------	----------	----------	----------	----------	----------	----------	633
Callithrix_jacchus	----------	----------	----------	----------	----------	----------	----------	495
Crocodylus_porosus	----------	----------	----------	----------	----------	----------	----------	527
Erinaceus_europaeus	----------	----------	----------	----------	----------	----------	----------	420
rnd-6_family-7426-LTR-ERV1	----------	----------	----------	----------	----------	----------	----------	1692
Esox_lucius	----------	----------	----------	----------	----------	----------	----------	532
Salmo_salar	----------	----------	----------	----------	----------	----------	----------	801
Didelphidae	----------	----------	----------	----------	----------	----------	----------	642
Latimeria_chalumnae	----------	----------	----------	----------	----------	----------	----------	1766
Monodelphis_domestica	----------	----------	----------	----------	----------	----------	----------	543
Macropus_eugenii	----------	----------	----------	----------	----------	----------	----------	509
Alligator_mississippiensis	----------	----------	----------	----------	----------	----------	----------	566
Chrysemys_picta_bellii	----------	----------	----------	----------	----------	----------	----------	3578
Danio_rerio	----------	----------	----------	----------	----------	----------	----------	699
Taeniopygia	----------	----------	----------	----------	----------	----------	----------	538
Dasypus_novemcinctus	----------	----------	----------	----------	----------	----------	----------	512
LTR_XT	----------	----------	----------	----------	----------	----------	----------	503
Haplorrhini	----------	----------	----------	----------	----------	----------	----------	8801
Catarrhini	----------	----------	----------	----------	----------	----------	----------	7847
Anolis_carolinensis	----------	----------	----------	----------	----------	----------	----------	1184
Murine_leukemia_virus	----------	----------	----------	----------	----------	----------	----------	7615
Phascolarctos_cinereus	----------	----------	----------	----------	----------	----------	----------	7426
Estrildidae	----------	----------	----------	----------	----------	----------	----------	482
Xenopus_Silurana_tropicalis	----------	----------	----------	----------	----------	----------	----------	812
I_XT	----------	----------	----------	----------	----------	----------	----------	7509
Takifugu_rubripes	----------	----------	----------	----------	----------	----------	----------	707
Bos_taurus	----------	----------	----------	----------	----------	----------	----------	533
Homo_sapiens	----------	----------	----------	----------	----------	----------	----------	450
Microcebus_murinus	----------	----------	----------	----------	----------	----------	----------	428
Myotis_lucifugus	----------	----------	----------	----------	----------	----------	----------	432
Equus_caballus	----------	----------	----------	----------	----------	----------	----------	450
Tupaia_belangeri	----------	----------	----------	----------	----------	----------	----------	498
Loxodonta_africana	----------	----------	----------	----------	----------	----------	----------	460
Murinae	----------	----------	----------	----------	----------	----------	----------	489
Canis_lupus_familiaris	----------	----------	----------	----------	----------	----------	----------	536
Sus_scrofa	----------	----------	----------	----------	----------	----------	----------	454
Choloepus_hoffmanni	----------	----------	----------	----------	----------	----------	----------	444
Vicugna_pacos	----------	----------	----------	----------	----------	----------	----------	474
Gavialis_gangeticus	----------	----------	----------	----------	----------	----------	----------	470
Mammalia	----------	----------	----------	----------	----------	----------	----------	1337
Passeroidea	----------	----------	----------	----------	----------	----------	----------	641
Takifugu	----------	----------	----------	----------	----------	----------	----------	629
Pteropus_vampyrus	----------	----------	----------	----------	----------	----------	----------	472
Ornithorhynchus	----------	----------	----------	----------	----------	----------	----------	2785
Oryctolagus_cuniculus	----------	----------	----------	----------	----------	----------	----------	494
Gallus_gallus	----------	----------	----------	----------	----------	----------	----------	508
Hominidae	TTGCNNNNAN	CNTTAANACC	ANNNNNANNN	GGTNATCNNA	CTTAGCNNCN	TCNNTNCAGT	CAGCCNNNNN	6256
Metatheria	----------	----------	----------	----------	----------	----------	----------	569
Rodentia	TACTGAATAA	CGCTCTGCCT	GGCTGCACTT	TGGAGAATGT	TGGTTGTGTG	TCCCTCCTGG	-GTCAAACTC	3851
Monodelphis	----------	----------	----------	----------	----------	----------	----------	857
Consensus	----------	----------	----------	----------	----------	----------	----------	

Conservation (100% / 0%)

10,020 10,040 10,060 10,080

Dipodomys_ordii	----------	----------	----------	----------	----------	----------	----------	597
Laurasiatheria	----------	----------	----------	----------	----------	----------	----------	361
Eutheria	----------	----------	----------	----------	----------	----------	----------	452
Tarsius_syrichta	----------	----------	----------	----------	----------	----------	----------	487
Muridae	TAAAAACAGA	ATATACAGAA	AGCCCCTTCT	AAGAAATGCA	CAAGAAAAAT	GTAATCCAAT	GGCCATAAAA	4398
Gasterosteus_aculeatus	----------	----------	----------	----------	----------	----------	----------	2399
Felis_silvestris	----------	----------	----------	----------	----------	----------	----------	505
Rattus	----------	----------	----------	----------	----------	----------	----------	589
Papio_hamadryas	GTTAAACATA	ATACACGCTA	TGGTGCTAAC	CCAACAGTAT	CAGGTGCTCA	GAACCGATGA	AGAAGCTCAA	7365
Pan_troglodytes	----------	----------	----------	----------	----------	----------	----------	4138
Ailuropoda_melanoleuca	----------	----------	----------	----------	----------	----------	----------	462
Cavia_porcellus	----------	----------	----------	----------	----------	----------	----------	527
Ictidomys_tridecemlineatus	----------	----------	----------	----------	----------	----------	----------	530
Canis	----------	----------	----------	----------	----------	----------	----------	479
Felis_catus	----------	----------	----------	----------	----------	----------	----------	423
Ochotona_princeps	----------	----------	----------	----------	----------	----------	----------	515
Cercopithecidae	----------	----------	----------	----------	----------	----------	----------	2243
Carnivora	----------	----------	----------	----------	----------	----------	----------	421
Mus	----------	----------	----------	----------	----------	----------	----------	2488
Mus_musculus	----------	----------	----------	----------	----------	----------	----------	478
Rattus_norvegicus	----------	----------	----------	----------	----------	----------	----------	420
Tursiops_truncatus	----------	----------	----------	----------	----------	----------	----------	518
Procavia_capensis	----------	----------	----------	----------	----------	----------	----------	442
Primates	----------	----------	----------	----------	----------	----------	----------	671
Echinops_telfairi	----------	----------	----------	----------	----------	----------	----------	473
Sorex_araneus	----------	----------	----------	----------	----------	----------	----------	494
Canidae	----------	----------	----------	----------	----------	----------	----------	550
Otolemur_garnettii	----------	----------	----------	----------	----------	----------	----------	413
Gallus	----------	----------	----------	----------	----------	----------	----------	633
Callithrix_jacchus	----------	----------	----------	----------	----------	----------	----------	495
Crocodylus_porosus	----------	----------	----------	----------	----------	----------	----------	527
Erinaceus_europaeus	----------	----------	----------	----------	----------	----------	----------	420
rnd-6_family-7426-LTR-ERV1	----------	----------	----------	----------	----------	----------	----------	1692
Esox_lucius	----------	----------	----------	----------	----------	----------	----------	532
Salmo_salar	----------	----------	----------	----------	----------	----------	----------	801
Didelphidae	----------	----------	----------	----------	----------	----------	----------	642
Latimeria_chalumnae	----------	----------	----------	----------	----------	----------	----------	1766
Monodelphis_domestica	----------	----------	----------	----------	----------	----------	----------	543
Macropus_eugenii	----------	----------	----------	----------	----------	----------	----------	509
Alligator_mississippiensis	----------	----------	----------	----------	----------	----------	----------	566
Chrysemys_picta_bellii	----------	----------	----------	----------	----------	----------	----------	3578
Danio_rerio	----------	----------	----------	----------	----------	----------	----------	699
Taeniopygia	----------	----------	----------	----------	----------	----------	----------	538
Dasypus_novemcinctus	----------	----------	----------	----------	----------	----------	----------	512
LTR_XT	----------	----------	----------	----------	----------	----------	----------	503
Haplorrhini	----------	----------	----------	----------	----------	----------	----------	8801
Catarrhini	----------	----------	----------	----------	----------	----------	----------	7847
Anolis_carolinensis	----------	----------	----------	----------	----------	----------	----------	1184
Murine_leukemia_virus	----------	----------	----------	----------	----------	----------	----------	7615
Phascolarctos_cinereus	----------	----------	----------	----------	----------	----------	----------	7426
Estrildidae	----------	----------	----------	----------	----------	----------	----------	482
Xenopus_Silurana_tropicalis	----------	----------	----------	----------	----------	----------	----------	812
I_XT	----------	----------	----------	----------	----------	----------	----------	7509
Takifugu_rubripes	----------	----------	----------	----------	----------	----------	----------	707
Bos_taurus	----------	----------	----------	----------	----------	----------	----------	533
Homo_sapiens	----------	----------	----------	----------	----------	----------	----------	450
Microcebus_murinus	----------	----------	----------	----------	----------	----------	----------	428
Myotis_lucifugus	----------	----------	----------	----------	----------	----------	----------	432
Equus_caballus	----------	----------	----------	----------	----------	----------	----------	450
Tupaia_belangeri	----------	----------	----------	----------	----------	----------	----------	498
Loxodonta_africana	----------	----------	----------	----------	----------	----------	----------	460
Murinae	----------	----------	----------	----------	----------	----------	----------	489
Canis_lupus_familiaris	----------	----------	----------	----------	----------	----------	----------	536
Sus_scrofa	----------	----------	----------	----------	----------	----------	----------	454
Choloepus_hoffmanni	----------	----------	----------	----------	----------	----------	----------	444
Vicugna_pacos	----------	----------	----------	----------	----------	----------	----------	474
Gavialis_gangeticus	----------	----------	----------	----------	----------	----------	----------	470
Mammalia	----------	----------	----------	----------	----------	----------	----------	1337
Passeroidea	----------	----------	----------	----------	----------	----------	----------	641
Takifugu	----------	----------	----------	----------	----------	----------	----------	629
Pteropus_vampyrus	----------	----------	----------	----------	----------	----------	----------	472
Ornithorhynchus	----------	----------	----------	----------	----------	----------	----------	2785
Oryctolagus_cuniculus	----------	----------	----------	----------	----------	----------	----------	494
Gallus_gallus	----------	----------	----------	----------	----------	----------	----------	508
Hominidae	ANNGCTNNCA	NNGNCANCTN	CNACCGGCAG	CTGACNAGGC	NNAGNCNNNN	CCNTTNTCGT	GGNTAACNNT	6326
Metatheria	----------	----------	----------	----------	----------	----------	----------	569
Rodentia	ACGACCACCA	TTTGTGGCCT	CGCCCATTGG	CTTATTAGAC	TCTTTCACCT	CGCTGCTCGT	TAATTGCTCT	3921
Monodelphis	----------	----------	----------	----------	----------	----------	----------	857
Consensus	----------	----------	----------	----------	----------	----------	----------	

Conservation

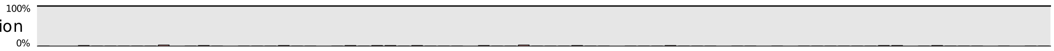

	10,100 10,120 10,140	
Dipodomys_ordii	---------- ---------- ---------- ---------- ---------- ---------- ----------	597
Laurasiatheria	---------- ---------- ---------- ---------- ---------- ---------- ----------	361
Eutheria	---------- ---------- ---------- ---------- ---------- ---------- ----------	452
Tarsius_syrichta	---------- ---------- ---------- ---------- ---------- ---------- ----------	487
Muridae	TTAAAAATCT GGAAAAAAAG ATTCTGGCCT TTGGGAAACA GGAAAAACCT GGGGAATCAG ACTCTATGCC	4468
Gasterosteus_aculeatus	---------- ---------- ---------- ---------- ---------- ---------- ----------	2399
Felis_silvestris	---------- ---------- ---------- ---------- ---------- ---------- ----------	505
Rattus	---------- ---------- ---------- ---------- ---------- ---------- ----------	589
Papio_hamadryas	GATTGAGCCT CTAAGACACA AGAAAAG--- ---------- ---------- ---------- ----------	7392
Pan_troglodytes	---------- ---------- ---------- ---------- ---------- ---------- ----------	4138
Ailuropoda_melanoleuca	---------- ---------- ---------- ---------- ---------- ---------- ----------	462
Cavia_porcellus	---------- ---------- ---------- ---------- ---------- ---------- ----------	527
Ictidomys_tridecemlineatus	---------- ---------- ---------- ---------- ---------- ---------- ----------	530
Canis	---------- ---------- ---------- ---------- ---------- ---------- ----------	479
Felis_catus	---------- ---------- ---------- ---------- ---------- ---------- ----------	423
Ochotona_princeps	---------- ---------- ---------- ---------- ---------- ---------- ----------	515
Cercopithecidae	---------- ---------- ---------- ---------- ---------- ---------- ----------	2243
Carnivora	---------- ---------- ---------- ---------- ---------- ---------- ----------	421
Mus	---------- ---------- ---------- ---------- ---------- ---------- ----------	2488
Mus_musculus	---------- ---------- ---------- ---------- ---------- ---------- ----------	478
Rattus_norvegicus	---------- ---------- ---------- ---------- ---------- ---------- ----------	420
Tursiops_truncatus	---------- ---------- ---------- ---------- ---------- ---------- ----------	518
Procavia_capensis	---------- ---------- ---------- ---------- ---------- ---------- ----------	442
Primates	---------- ---------- ---------- ---------- ---------- ---------- ----------	671
Echinops_telfairi	---------- ---------- ---------- ---------- ---------- ---------- ----------	473
Sorex_araneus	---------- ---------- ---------- ---------- ---------- ---------- ----------	494
Canidae	---------- ---------- ---------- ---------- ---------- ---------- ----------	550
Otolemur_garnettii	---------- ---------- ---------- ---------- ---------- ---------- ----------	413
Gallus	---------- ---------- ---------- ---------- ---------- ---------- ----------	633
Callithrix_jacchus	---------- ---------- ---------- ---------- ---------- ---------- ----------	495
Crocodylus_porosus	---------- ---------- ---------- ---------- ---------- ---------- ----------	527
Erinaceus_europaeus	---------- ---------- ---------- ---------- ---------- ---------- ----------	420
rnd-6_family-7426-LTR-ERV1	---------- ---------- ---------- ---------- ---------- ---------- ----------	1692
Esox_lucius	---------- ---------- ---------- ---------- ---------- ---------- ----------	532
Salmo_salar	---------- ---------- ---------- ---------- ---------- ---------- ----------	801
Didelphidae	---------- ---------- ---------- ---------- ---------- ---------- ----------	642
Latimeria_chalumnae	---------- ---------- ---------- ---------- ---------- ---------- ----------	1766
Monodelphis_domestica	---------- ---------- ---------- ---------- ---------- ---------- ----------	543
Macropus_eugenii	---------- ---------- ---------- ---------- ---------- ---------- ----------	509
Alligator_mississippiensis	---------- ---------- ---------- ---------- ---------- ---------- ----------	566
Chrysemys_picta_bellii	---------- ---------- ---------- ---------- ---------- ---------- ----------	3578
Danio_rerio	---------- ---------- ---------- ---------- ---------- ---------- ----------	699
Taeniopygia	---------- ---------- ---------- ---------- ---------- ---------- ----------	538
Dasypus_novemcinctus	---------- ---------- ---------- ---------- ---------- ---------- ----------	512
LTR_XT	---------- ---------- ---------- ---------- ---------- ---------- ----------	503
Haplorrhini	---------- ---------- ---------- ---------- ---------- ---------- ----------	8801
Catarrhini	---------- ---------- ---------- ---------- ---------- ---------- ----------	7847
Anolis_carolinensis	---------- ---------- ---------- ---------- ---------- ---------- ----------	1184
Murine_leukemia_virus	---------- ---------- ---------- ---------- ---------- ---------- ----------	7615
Phascolarctos_cinereus	---------- ---------- ---------- ---------- ---------- ---------- ----------	7426
Estrildidae	---------- ---------- ---------- ---------- ---------- ---------- ----------	482
Xenopus_Silurana_tropicalis	---------- ---------- ---------- ---------- ---------- ---------- ----------	812
I_XT	---------- ---------- ---------- ---------- ---------- ---------- ----------	7509
Takifugu_rubripes	---------- ---------- ---------- ---------- ---------- ---------- ----------	707
Bos_taurus	---------- ---------- ---------- ---------- ---------- ---------- ----------	533
Homo_sapiens	---------- ---------- ---------- ---------- ---------- ---------- ----------	450
Microcebus_murinus	---------- ---------- ---------- ---------- ---------- ---------- ----------	428
Myotis_lucifugus	---------- ---------- ---------- ---------- ---------- ---------- ----------	432
Equus_caballus	---------- ---------- ---------- ---------- ---------- ---------- ----------	450
Tupaia_belangeri	---------- ---------- ---------- ---------- ---------- ---------- ----------	498
Loxodonta_africana	---------- ---------- ---------- ---------- ---------- ---------- ----------	460
Murinae	---------- ---------- ---------- ---------- ---------- ---------- ----------	489
Canis_lupus_familiaris	---------- ---------- ---------- ---------- ---------- ---------- ----------	536
Sus_scrofa	---------- ---------- ---------- ---------- ---------- ---------- ----------	454
Choloepus_hoffmanni	---------- ---------- ---------- ---------- ---------- ---------- ----------	444
Vicugna_pacos	---------- ---------- ---------- ---------- ---------- ---------- ----------	474
Gavialis_gangeticus	---------- ---------- ---------- ---------- ---------- ---------- ----------	470
Mammalia	---------- ---------- ---------- ---------- ---------- ---------- ----------	1337
Passeroidea	---------- ---------- ---------- ---------- ---------- ---------- ----------	641
Takifugu	---------- ---------- ---------- ---------- ---------- ---------- ----------	629
Pteropus_vampyrus	---------- ---------- ---------- ---------- ---------- ---------- ----------	472
Ornithorhynchus	---------- ---------- ---------- ---------- ---------- ---------- ----------	2785
Oryctolagus_cuniculus	---------- ---------- ---------- ---------- ---------- ---------- ----------	494
Gallus_gallus	---------- ---------- ---------- ---------- ---------- ---------- ----------	508
Hominidae	ANTCNNNNAN GGNGCNCNNN TNNTNNANNN NNCAGGNNTA NNNAANCTCT CCCACTGNTT CCTNTGNNCN	6396
Metatheria	---------- ---------- ---------- ---------- ---------- ---------- ----------	569
Rodentia	AAGCCAAA-T ATTACNACCA CCNTATCTCT CCCACATCTC TCTCTTTGGT ATCGCAGACT GCTTTAACTC	3990
Monodelphis	---------- ---------- ---------- ---------- ---------- ---------- ----------	857
Consensus	---------- ---------- ---------- ---------- ---------- ---------- ----------	
Conservation (100% / 0%)		

	10,160 · 10,180 · 10,200 · 10,220	
Dipodomys_ordii	---------- ---------- ---------- ---------- ---------- ---------- ----------	597
Laurasiatheria	---------- ---------- ---------- ---------- ---------- ---------- ----------	361
Eutheria	---------- ---------- ---------- ---------- ---------- ---------- ----------	452
Tarsius_syrichta	---------- ---------- ---------- ---------- ---------- ---------- ----------	487
Muridae	ACAGGAAGGG ACCCTGGGGA TAGAGTTAAT CATTCCCAGC CAGAAATCAG CCAGGATTGC TGGTTATGCT	4538
Gasterosteus_aculeatus	---------- ---------- ---------- ---------- ---------- ---------- ----------	2399
Felis_silvestris	---------- ---------- ---------- ---------- ---------- ---------- ----------	505
Rattus	---------- ---------- ---------- ---------- ---------- ---------- ----------	589
Papio_hamadryas	---------- ---------- ---------- ---------- ---------- ---------- ----------	7392
Pan_troglodytes	---------- ---------- ---------- ---------- ---------- ---------- ----------	4138
Ailuropoda_melanoleuca	---------- ---------- ---------- ---------- ---------- ---------- ----------	462
Cavia_porcellus	---------- ---------- ---------- ---------- ---------- ---------- ----------	527
Ictidomys_tridecemlineatus	---------- ---------- ---------- ---------- ---------- ---------- ----------	530
Canis	---------- ---------- ---------- ---------- ---------- ---------- ----------	479
Felis_catus	---------- ---------- ---------- ---------- ---------- ---------- ----------	423
Ochotona_princeps	---------- ---------- ---------- ---------- ---------- ---------- ----------	515
Cercopithecidae	---------- ---------- ---------- ---------- ---------- ---------- ----------	2243
Carnivora	---------- ---------- ---------- ---------- ---------- ---------- ----------	421
Mus	---------- ---------- ---------- ---------- ---------- ---------- ----------	2488
Mus_musculus	---------- ---------- ---------- ---------- ---------- ---------- ----------	478
Rattus_norvegicus	---------- ---------- ---------- ---------- ---------- ---------- ----------	420
Tursiops_truncatus	---------- ---------- ---------- ---------- ---------- ---------- ----------	518
Procavia_capensis	---------- ---------- ---------- ---------- ---------- ---------- ----------	442
Primates	---------- ---------- ---------- ---------- ---------- ---------- ----------	671
Echinops_telfairi	---------- ---------- ---------- ---------- ---------- ---------- ----------	473
Sorex_araneus	---------- ---------- ---------- ---------- ---------- ---------- ----------	494
Canidae	---------- ---------- ---------- ---------- ---------- ---------- ----------	550
Otolemur_garnettii	---------- ---------- ---------- ---------- ---------- ---------- ----------	413
Gallus	---------- ---------- ---------- ---------- ---------- ---------- ----------	633
Callithrix_jacchus	---------- ---------- ---------- ---------- ---------- ---------- ----------	495
Crocodylus_porosus	---------- ---------- ---------- ---------- ---------- ---------- ----------	527
Erinaceus_europaeus	---------- ---------- ---------- ---------- ---------- ---------- ----------	420
rnd-6_family-7426-LTR-ERV1	---------- ---------- ---------- ---------- ---------- ---------- ----------	1692
Esox_lucius	---------- ---------- ---------- ---------- ---------- ---------- ----------	532
Salmo_salar	---------- ---------- ---------- ---------- ---------- ---------- ----------	801
Didelphidae	---------- ---------- ---------- ---------- ---------- ---------- ----------	642
Latimeria_chalumnae	---------- ---------- ---------- ---------- ---------- ---------- ----------	1766
Monodelphis_domestica	---------- ---------- ---------- ---------- ---------- ---------- ----------	543
Macropus_eugenii	---------- ---------- ---------- ---------- ---------- ---------- ----------	509
Alligator_mississippiensis	---------- ---------- ---------- ---------- ---------- ---------- ----------	566
Chrysemys_picta_bellii	---------- ---------- ---------- ---------- ---------- ---------- ----------	3578
Danio_rerio	---------- ---------- ---------- ---------- ---------- ---------- ----------	699
Taeniopygia	---------- ---------- ---------- ---------- ---------- ---------- ----------	538
Dasypus_novemcinctus	---------- ---------- ---------- ---------- ---------- ---------- ----------	512
LTR_XT	---------- ---------- ---------- ---------- ---------- ---------- ----------	503
Haplorrhini	---------- ---------- ---------- ---------- ---------- ---------- ----------	8801
Catarrhini	---------- ---------- ---------- ---------- ---------- ---------- ----------	7847
Anolis_carolinensis	---------- ---------- ---------- ---------- ---------- ---------- ----------	1184
Murine_leukemia_virus	---------- ---------- ---------- ---------- ---------- ---------- ----------	7615
Phascolarctos_cinereus	---------- ---------- ---------- ---------- ---------- ---------- ----------	7426
Estrildidae	---------- ---------- ---------- ---------- ---------- ---------- ----------	482
Xenopus_Silurana_tropicalis	---------- ---------- ---------- ---------- ---------- ---------- ----------	812
I_XT	---------- ---------- ---------- ---------- ---------- ---------- ----------	7509
Takifugu_rubripes	---------- ---------- ---------- ---------- ---------- ---------- ----------	707
Bos_taurus	---------- ---------- ---------- ---------- ---------- ---------- ----------	533
Homo_sapiens	---------- ---------- ---------- ---------- ---------- ---------- ----------	450
Microcebus_murinus	---------- ---------- ---------- ---------- ---------- ---------- ----------	428
Myotis_lucifugus	---------- ---------- ---------- ---------- ---------- ---------- ----------	432
Equus_caballus	---------- ---------- ---------- ---------- ---------- ---------- ----------	450
Tupaia_belangeri	---------- ---------- ---------- ---------- ---------- ---------- ----------	498
Loxodonta_africana	---------- ---------- ---------- ---------- ---------- ---------- ----------	460
Murinae	---------- ---------- ---------- ---------- ---------- ---------- ----------	489
Canis_lupus_familiaris	---------- ---------- ---------- ---------- ---------- ---------- ----------	536
Sus_scrofa	---------- ---------- ---------- ---------- ---------- ---------- ----------	454
Choloepus_hoffmanni	---------- ---------- ---------- ---------- ---------- ---------- ----------	444
Vicugna_pacos	---------- ---------- ---------- ---------- ---------- ---------- ----------	474
Gavialis_gangeticus	---------- ---------- ---------- ---------- ---------- ---------- ----------	470
Mammalia	---------- ---------- ---------- ---------- ---------- ---------- ----------	1337
Passeroidea	---------- ---------- ---------- ---------- ---------- ---------- ----------	641
Takifugu	---------- ---------- ---------- ---------- ---------- ---------- ----------	629
Pteropus_vampyrus	---------- ---------- ---------- ---------- ---------- ---------- ----------	472
Ornithorhynchus	---------- ---------- ---------- ---------- ---------- ---------- ----------	2785
Oryctolagus_cuniculus	---------- ---------- ---------- ---------- ---------- ---------- ----------	494
Gallus_gallus	---------- ---------- ---------- ---------- ---------- ---------- ----------	508
Hominidae	GCCCTCGGNN NACCNCCNNT AGTAGCNGTN CCNCTNCCNN NNNCNTTTAA NNNNNCNNGN AANNNNNCNN	6466
Metatheria	---------- ---------- ---------- ---------- ---------- ---------- ----------	569
Rodentia	CTCAGGGACA CACTTTGTGG GCACACTGGA TCCCNCAGAC TGCAATAATA CCNTAACCCT TAGCACTACC	4060
Monodelphis	---------- ---------- ---------- ---------- ---------- ---------- ----------	857
Consensus	---------- ---------- ---------- ---------- ---------- ---------- ----------	

Conservation (100% – 0%)

10,240 10,260 10,280

Dipodomys_ordii	---------- ---------- ---------- ---------- ---------- ---------- ----------	597
Laurasiatheria	---------- ---------- ---------- ---------- ---------- ---------- ----------	361
Eutheria	---------- ---------- ---------- ---------- ---------- ---------- ----------	452
Tarsius_syrichta	---------- ---------- ---------- ---------- ---------- ---------- ----------	487
Muridae	TAGACACCCG GCCCCCGTAC TACATAGGGA TAGGAACTAA CCAGACTGTC AGCCCATTGA CTGACAAAAC	4608
Gasterosteus_aculeatus	---------- ---------- ---------- ---------- ---------- ---------- ----------	2399
Felis_silvestris	---------- ---------- ---------- ---------- ---------- ---------- ----------	505
Rattus	---------- ---------- ---------- ---------- ---------- ---------- ----------	589
Papio_hamadryas	---------- ---------- ---------- ---------- ---------- ---------- ----------	7392
Pan_troglodytes	---------- ---------- ---------- ---------- ---------- ---------- ----------	4138
Ailuropoda_melanoleuca	---------- ---------- ---------- ---------- ---------- ---------- ----------	462
Cavia_porcellus	---------- ---------- ---------- ---------- ---------- ---------- ----------	527
Ictidomys_tridecemlineatus	---------- ---------- ---------- ---------- ---------- ---------- ----------	530
Canis	---------- ---------- ---------- ---------- ---------- ---------- ----------	479
Felis_catus	---------- ---------- ---------- ---------- ---------- ---------- ----------	423
Ochotona_princeps	---------- ---------- ---------- ---------- ---------- ---------- ----------	515
Cercopithecidae	---------- ---------- ---------- ---------- ---------- ---------- ----------	2243
Carnivora	---------- ---------- ---------- ---------- ---------- ---------- ----------	421
Mus	---------- ---------- ---------- ---------- ---------- ---------- ----------	2488
Mus_musculus	---------- ---------- ---------- ---------- ---------- ---------- ----------	478
Rattus_norvegicus	---------- ---------- ---------- ---------- ---------- ---------- ----------	420
Tursiops_truncatus	---------- ---------- ---------- ---------- ---------- ---------- ----------	518
Procavia_capensis	---------- ---------- ---------- ---------- ---------- ---------- ----------	442
Primates	---------- ---------- ---------- ---------- ---------- ---------- ----------	671
Echinops_telfairi	---------- ---------- ---------- ---------- ---------- ---------- ----------	473
Sorex_araneus	---------- ---------- ---------- ---------- ---------- ---------- ----------	494
Canidae	---------- ---------- ---------- ---------- ---------- ---------- ----------	550
Otolemur_garnettii	---------- ---------- ---------- ---------- ---------- ---------- ----------	413
Gallus	---------- ---------- ---------- ---------- ---------- ---------- ----------	633
Callithrix_jacchus	---------- ---------- ---------- ---------- ---------- ---------- ----------	495
Crocodylus_porosus	---------- ---------- ---------- ---------- ---------- ---------- ----------	527
Erinaceus_europaeus	---------- ---------- ---------- ---------- ---------- ---------- ----------	420
rnd-6_family-7426-LTR-ERV1	---------- ---------- ---------- ---------- ---------- ---------- ----------	1692
Esox_lucius	---------- ---------- ---------- ---------- ---------- ---------- ----------	532
Salmo_salar	---------- ---------- ---------- ---------- ---------- ---------- ----------	801
Didelphidae	---------- ---------- ---------- ---------- ---------- ---------- ----------	642
Latimeria_chalumnae	---------- ---------- ---------- ---------- ---------- ---------- ----------	1766
Monodelphis_domestica	---------- ---------- ---------- ---------- ---------- ---------- ----------	543
Macropus_eugenii	---------- ---------- ---------- ---------- ---------- ---------- ----------	509
Alligator_mississippiensis	---------- ---------- ---------- ---------- ---------- ---------- ----------	566
Chrysemys_picta_bellii	---------- ---------- ---------- ---------- ---------- ---------- ----------	3578
Danio_rerio	---------- ---------- ---------- ---------- ---------- ---------- ----------	699
Taeniopygia	---------- ---------- ---------- ---------- ---------- ---------- ----------	538
Dasypus_novemcinctus	---------- ---------- ---------- ---------- ---------- ---------- ----------	512
LTR_XT	---------- ---------- ---------- ---------- ---------- ---------- ----------	503
Haplorrhini	---------- ---------- ---------- ---------- ---------- ---------- ----------	8801
Catarrhini	---------- ---------- ---------- ---------- ---------- ---------- ----------	7847
Anolis_carolinensis	---------- ---------- ---------- ---------- ---------- ---------- ----------	1184
Murine_leukemia_virus	---------- ---------- ---------- ---------- ---------- ---------- ----------	7615
Phascolarctos_cinereus	---------- ---------- ---------- ---------- ---------- ---------- ----------	7426
Estrildidae	---------- ---------- ---------- ---------- ---------- ---------- ----------	482
Xenopus_Silurana_tropicalis	---------- ---------- ---------- ---------- ---------- ---------- ----------	812
I_XT	---------- ---------- ---------- ---------- ---------- ---------- ----------	7509
Takifugu_rubripes	---------- ---------- ---------- ---------- ---------- ---------- ----------	707
Bos_taurus	---------- ---------- ---------- ---------- ---------- ---------- ----------	533
Homo_sapiens	---------- ---------- ---------- ---------- ---------- ---------- ----------	450
Microcebus_murinus	---------- ---------- ---------- ---------- ---------- ---------- ----------	428
Myotis_lucifugus	---------- ---------- ---------- ---------- ---------- ---------- ----------	432
Equus_caballus	---------- ---------- ---------- ---------- ---------- ---------- ----------	450
Tupaia_belangeri	---------- ---------- ---------- ---------- ---------- ---------- ----------	498
Loxodonta_africana	---------- ---------- ---------- ---------- ---------- ---------- ----------	460
Murinae	---------- ---------- ---------- ---------- ---------- ---------- ----------	489
Canis_lupus_familiaris	---------- ---------- ---------- ---------- ---------- ---------- ----------	536
Sus_scrofa	---------- ---------- ---------- ---------- ---------- ---------- ----------	454
Choloepus_hoffmanni	---------- ---------- ---------- ---------- ---------- ---------- ----------	444
Vicugna_pacos	---------- ---------- ---------- ---------- ---------- ---------- ----------	474
Gavialis_gangeticus	---------- ---------- ---------- ---------- ---------- ---------- ----------	470
Mammalia	---------- ---------- ---------- ---------- ---------- ---------- ----------	1337
Passeroidea	---------- ---------- ---------- ---------- ---------- ---------- ----------	641
Takifugu	---------- ---------- ---------- ---------- ---------- ---------- ----------	629
Pteropus_vampyrus	---------- ---------- ---------- ---------- ---------- ---------- ----------	472
Ornithorhynchus	---------- ---------- ---------- ---------- ---------- ---------- ----------	2785
Oryctolagus_cuniculus	---------- ---------- ---------- ---------- ---------- ---------- ----------	494
Gallus_gallus	---------- ---------- ---------- ---------- ---------- ---------- ----------	508
Hominidae	CCCNNNNNNC NNNNGTCNNN NNNGNNCAGG TCCCNCTNTT CNNAGACCCN NNNNNNCNCN AGTTCCCNTT	6536
Metatheria	---------- ---------- ---------- ---------- ---------- ---------- ----------	569
Rodentia	ACCCAGCCCC TATGCCCGAA CGTTCACAAT GCCTCNATTC TCTGCGGCAC TCAAGTNTAC CACTGCCTAC	4130
Monodelphis	---------- ---------- ---------- ---------- ---------- ---------- ----------	857
Consensus	---------- ---------- ---------- ---------- ---------- ---------- ----------	

Conservation 100% 0%

	10,300		10,320		10,340		10,360	
Dipodomys_ordii	----------	----------	----------	----------	----------	----------	----------	597
Laurasiatheria	----------	----------	----------	----------	----------	----------	----------	361
Eutheria	----------	----------	----------	----------	----------	----------	----------	452
Tarsius_syrichta	----------	----------	----------	----------	----------	----------	----------	487
Muridae	TCACTGTGAC	TGGGGGCAGC	CCAAATTAAC	GTTAGAGGAC	TTACAAGGGG	CTAAAACTTG	TCTAATATCC	4678
Gasterosteus_aculeatus	----------	----------	----------	----------	----------	----------	----------	2399
Felis_silvestris	----------	----------	----------	----------	----------	----------	----------	505
Rattus	----------	----------	----------	----------	----------	----------	----------	589
Papio_hamadryas	----------	----------	----------	----------	----------	----------	----------	7392
Pan_troglodytes	----------	----------	----------	----------	----------	----------	----------	4138
Ailuropoda_melanoleuca	----------	----------	----------	----------	----------	----------	----------	462
Cavia_porcellus	----------	----------	----------	----------	----------	----------	----------	527
Ictidomys_tridecemlineatus	----------	----------	----------	----------	----------	----------	----------	530
Canis	----------	----------	----------	----------	----------	----------	----------	479
Felis_catus	----------	----------	----------	----------	----------	----------	----------	423
Ochotona_princeps	----------	----------	----------	----------	----------	----------	----------	515
Cercopithecidae	----------	----------	----------	----------	----------	----------	----------	2243
Carnivora	----------	----------	----------	----------	----------	----------	----------	421
Mus	----------	----------	----------	----------	----------	----------	----------	2488
Mus_musculus	----------	----------	----------	----------	----------	----------	----------	478
Rattus_norvegicus	----------	----------	----------	----------	----------	----------	----------	420
Tursiops_truncatus	----------	----------	----------	----------	----------	----------	----------	518
Procavia_capensis	----------	----------	----------	----------	----------	----------	----------	442
Primates	----------	----------	----------	----------	----------	----------	----------	671
Echinops_telfairi	----------	----------	----------	----------	----------	----------	----------	473
Sorex_araneus	----------	----------	----------	----------	----------	----------	----------	494
Canidae	----------	----------	----------	----------	----------	----------	----------	550
Otolemur_garnettii	----------	----------	----------	----------	----------	----------	----------	413
Gallus	----------	----------	----------	----------	----------	----------	----------	633
Callithrix_jacchus	----------	----------	----------	----------	----------	----------	----------	495
Crocodylus_porosus	----------	----------	----------	----------	----------	----------	----------	527
Erinaceus_europaeus	----------	----------	----------	----------	----------	----------	----------	420
rnd-6_family-7426-LTR-ERV1	----------	----------	----------	----------	----------	----------	----------	1692
Esox_lucius	----------	----------	----------	----------	----------	----------	----------	532
Salmo_salar	----------	----------	----------	----------	----------	----------	----------	801
Didelphidae	----------	----------	----------	----------	----------	----------	----------	642
Latimeria_chalumnae	----------	----------	----------	----------	----------	----------	----------	1766
Monodelphis_domestica	----------	----------	----------	----------	----------	----------	----------	543
Macropus_eugenii	----------	----------	----------	----------	----------	----------	----------	509
Alligator_mississippiensis	----------	----------	----------	----------	----------	----------	----------	566
Chrysemys_picta_bellii	----------	----------	----------	----------	----------	----------	----------	3578
Danio_rerio	----------	----------	----------	----------	----------	----------	----------	699
Taeniopygia	----------	----------	----------	----------	----------	----------	----------	538
Dasypus_novemcinctus	----------	----------	----------	----------	----------	----------	----------	512
LTR_XT	----------	----------	----------	----------	----------	----------	----------	503
Haplorrhini	----------	----------	----------	----------	----------	----------	----------	8801
Catarrhini	----------	----------	----------	----------	----------	----------	----------	7847
Anolis_carolinensis	----------	----------	----------	----------	----------	----------	----------	1184
Murine_leukemia_virus	----------	----------	----------	----------	----------	----------	----------	7615
Phascolarctos_cinereus	----------	----------	----------	----------	----------	----------	----------	7426
Estrildidae	----------	----------	----------	----------	----------	----------	----------	482
Xenopus_Silurana_tropicalis	----------	----------	----------	----------	----------	----------	----------	812
I_XT	----------	----------	----------	----------	----------	----------	----------	7509
Takifugu_rubripes	----------	----------	----------	----------	----------	----------	----------	707
Bos_taurus	----------	----------	----------	----------	----------	----------	----------	533
Homo_sapiens	----------	----------	----------	----------	----------	----------	----------	450
Microcebus_murinus	----------	----------	----------	----------	----------	----------	----------	428
Myotis_lucifugus	----------	----------	----------	----------	----------	----------	----------	432
Equus_caballus	----------	----------	----------	----------	----------	----------	----------	450
Tupaia_belangeri	----------	----------	----------	----------	----------	----------	----------	498
Loxodonta_africana	----------	----------	----------	----------	----------	----------	----------	460
Murinae	----------	----------	----------	----------	----------	----------	----------	489
Canis_lupus_familiaris	----------	----------	----------	----------	----------	----------	----------	536
Sus_scrofa	----------	----------	----------	----------	----------	----------	----------	454
Choloepus_hoffmanni	----------	----------	----------	----------	----------	----------	----------	444
Vicugna_pacos	----------	----------	----------	----------	----------	----------	----------	474
Gavialis_gangeticus	----------	----------	----------	----------	----------	----------	----------	470
Mammalia	----------	----------	----------	----------	----------	----------	----------	1337
Passeroidea	----------	----------	----------	----------	----------	----------	----------	641
Takifugu	----------	----------	----------	----------	----------	----------	----------	629
Pteropus_vampyrus	----------	----------	----------	----------	----------	----------	----------	472
Ornithorhynchus	----------	----------	----------	----------	----------	----------	----------	2785
Oryctolagus_cuniculus	----------	----------	----------	----------	----------	----------	----------	494
Gallus_gallus	----------	----------	----------	----------	----------	----------	----------	508
Hominidae	CTGNTACNCN	ACNCCNAANN	CNTCNTGGTG	NAACCAGACC	NATTCTGGNT	NCNTANCTAN	CNNCNCNGCA	6606
Metatheria	----------	----------	----------	----------	----------	----------	----------	569
Rodentia	CCGCCGGGTG	GTCTGGGACT	TGTGCCCTGG	TGTTCCTTTT	TCCAAAACTA	GGAGTGGTAC	CTGGTGAAGA	4200
Monodelphis	----------	----------	----------	----------	----------	----------	----------	857
Consensus	----------	----------	----------	----------	----------	----------	----------	

Conservation 100% 0%

		10,380		10,400		10,420		
Dipodomys_ordii	----------	----------	----------	----------	----------	----------	----------	597
Laurasiatheria	----------	----------	----------	----------	----------	----------	----------	361
Eutheria	----------	----------	----------	----------	----------	----------	----------	452
Tarsius_syrichta	----------	----------	----------	----------	----------	----------	----------	487
Muridae	AAAACCTTTA	ACCTAGGCAN	TTCCCCTTAT	TCTGATGCCT	GCTATTCTGC	CTTATGGGTT	AATTCATCTG	4748
Gasterosteus_aculeatus	----------	----------	----------	----------	----------	----------	----------	2399
Felis_silvestris	----------	----------	----------	----------	----------	----------	----------	505
Rattus	----------	----------	----------	----------	----------	----------	----------	589
Papio_hamadryas	----------	----------	----------	----------	----------	----------	----------	7392
Pan_troglodytes	----------	----------	----------	----------	----------	----------	----------	4138
Ailuropoda_melanoleuca	----------	----------	----------	----------	----------	----------	----------	462
Cavia_porcellus	----------	----------	----------	----------	----------	----------	----------	527
Ictidomys_tridecemlineatus	----------	----------	----------	----------	----------	----------	----------	530
Canis	----------	----------	----------	----------	----------	----------	----------	479
Felis_catus	----------	----------	----------	----------	----------	----------	----------	423
Ochotona_princeps	----------	----------	----------	----------	----------	----------	----------	515
Cercopithecidae	----------	----------	----------	----------	----------	----------	----------	2243
Carnivora	----------	----------	----------	----------	----------	----------	----------	421
Mus	----------	----------	----------	----------	----------	----------	----------	2488
Mus_musculus	----------	----------	----------	----------	----------	----------	----------	478
Rattus_norvegicus	----------	----------	----------	----------	----------	----------	----------	420
Tursiops_truncatus	----------	----------	----------	----------	----------	----------	----------	518
Procavia_capensis	----------	----------	----------	----------	----------	----------	----------	442
Primates	----------	----------	----------	----------	----------	----------	----------	671
Echinops_telfairi	----------	----------	----------	----------	----------	----------	----------	473
Sorex_araneus	----------	----------	----------	----------	----------	----------	----------	494
Canidae	----------	----------	----------	----------	----------	----------	----------	550
Otolemur_garnettii	----------	----------	----------	----------	----------	----------	----------	413
Gallus	----------	----------	----------	----------	----------	----------	----------	633
Callithrix_jacchus	----------	----------	----------	----------	----------	----------	----------	495
Crocodylus_porosus	----------	----------	----------	----------	----------	----------	----------	527
Erinaceus_europaeus	----------	----------	----------	----------	----------	----------	----------	420
rnd-6_family-7426-LTR-ERV1	----------	----------	----------	----------	----------	----------	----------	1692
Esox_lucius	----------	----------	----------	----------	----------	----------	----------	532
Salmo_salar	----------	----------	----------	----------	----------	----------	----------	801
Didelphidae	----------	----------	----------	----------	----------	----------	----------	642
Latimeria_chalumnae	----------	----------	----------	----------	----------	----------	----------	1766
Monodelphis_domestica	----------	----------	----------	----------	----------	----------	----------	543
Macropus_eugenii	----------	----------	----------	----------	----------	----------	----------	509
Alligator_mississippiensis	----------	----------	----------	----------	----------	----------	----------	566
Chrysemys_picta_bellii	----------	----------	----------	----------	----------	----------	----------	3578
Danio_rerio	----------	----------	----------	----------	----------	----------	----------	699
Taeniopygia	----------	----------	----------	----------	----------	----------	----------	538
Dasypus_novemcinctus	----------	----------	----------	----------	----------	----------	----------	512
LTR_XT	----------	----------	----------	----------	----------	----------	----------	503
Haplorrhini	----------	----------	----------	----------	----------	----------	----------	8801
Catarrhini	----------	----------	----------	----------	----------	----------	----------	7847
Anolis_carolinensis	----------	----------	----------	----------	----------	----------	----------	1184
Murine_leukemia_virus	----------	----------	----------	----------	----------	----------	----------	7615
Phascolarctos_cinereus	----------	----------	----------	----------	----------	----------	----------	7426
Estrildidae	----------	----------	----------	----------	----------	----------	----------	482
Xenopus_Silurana_tropicalis	----------	----------	----------	----------	----------	----------	----------	812
I_XT	----------	----------	----------	----------	----------	----------	----------	7509
Takifugu_rubripes	----------	----------	----------	----------	----------	----------	----------	707
Bos_taurus	----------	----------	----------	----------	----------	----------	----------	533
Homo_sapiens	----------	----------	----------	----------	----------	----------	----------	450
Microcebus_murinus	----------	----------	----------	----------	----------	----------	----------	428
Myotis_lucifugus	----------	----------	----------	----------	----------	----------	----------	432
Equus_caballus	----------	----------	----------	----------	----------	----------	----------	450
Tupaia_belangeri	----------	----------	----------	----------	----------	----------	----------	498
Loxodonta_africana	----------	----------	----------	----------	----------	----------	----------	460
Murinae	----------	----------	----------	----------	----------	----------	----------	489
Canis_lupus_familiaris	----------	----------	----------	----------	----------	----------	----------	536
Sus_scrofa	----------	----------	----------	----------	----------	----------	----------	454
Choloepus_hoffmanni	----------	----------	----------	----------	----------	----------	----------	444
Vicugna_pacos	----------	----------	----------	----------	----------	----------	----------	474
Gavialis_gangeticus	----------	----------	----------	----------	----------	----------	----------	470
Mammalia	----------	----------	----------	----------	----------	----------	----------	1337
Passeroidea	----------	----------	----------	----------	----------	----------	----------	641
Takifugu	----------	----------	----------	----------	----------	----------	----------	629
Pteropus_vampyrus	----------	----------	----------	----------	----------	----------	----------	472
Ornithorhynchus	----------	----------	----------	----------	----------	----------	----------	2785
Oryctolagus_cuniculus	----------	----------	----------	----------	----------	----------	----------	494
Gallus_gallus	----------	----------	----------	----------	----------	----------	----------	508
Hominidae	CCGNCNGGNG	GCTACTTCTG	GTGTAACTNC	ACNCTNACNA	AANNTCTTAA	NNNNNCNNNT	AANNANACCC	6676
Metatheria	----------	----------	----------	----------	----------	----------	----------	569
Rodentia	ACCCTTGCCA	GTTCCCATCA	CAGGGTTTAT	AGCGGCANGG	CATGACAAAT	GAGCGGTTCA	GATCCTACCC	4270
Monodelphis	----------	----------	----------	----------	----------	----------	----------	857
Consensus	----------	----------	----------	----------	----------	----------	----------	

100%
Conservation
0%

	10,440		10,460		10,480		10,500	
Dipodomys_ordii	----------	----------	----------	----------	----------	----------	----------	597
Laurasiatheria	----------	----------	----------	----------	----------	----------	----------	361
Eutheria	----------	----------	----------	----------	----------	----------	----------	452
Tarsius_syrichta	----------	----------	----------	----------	----------	----------	----------	487
Muridae	GATAACAGCA	ATATTACCGA	GCCCCTGAGA	CTACTTGGTG	GGCATGTACT	AATGGTTTGA	CTAAATGTGC	4818
Gasterosteus_aculeatus	----------	----------	----------	----------	----------	----------	----------	2399
Felis_silvestris	----------	----------	----------	----------	----------	----------	----------	505
Rattus	----------	----------	----------	----------	----------	----------	----------	589
Papio_hamadryas	----------	----------	----------	----------	----------	----------	----------	7392
Pan_troglodytes	----------	----------	----------	----------	----------	----------	----------	4138
Ailuropoda_melanoleuca	----------	----------	----------	----------	----------	----------	----------	462
Cavia_porcellus	----------	----------	----------	----------	----------	----------	----------	527
Ictidomys_tridecemlineatus	----------	----------	----------	----------	----------	----------	----------	530
Canis	----------	----------	----------	----------	----------	----------	----------	479
Felis_catus	----------	----------	----------	----------	----------	----------	----------	423
Ochotona_princeps	----------	----------	----------	----------	----------	----------	----------	515
Cercopithecidae	----------	----------	----------	----------	----------	----------	----------	2243
Carnivora	----------	----------	----------	----------	----------	----------	----------	421
Mus	----------	----------	----------	----------	----------	----------	----------	2488
Mus_musculus	----------	----------	----------	----------	----------	----------	----------	478
Rattus_norvegicus	----------	----------	----------	----------	----------	----------	----------	420
Tursiops_truncatus	----------	----------	----------	----------	----------	----------	----------	518
Procavia_capensis	----------	----------	----------	----------	----------	----------	----------	442
Primates	----------	----------	----------	----------	----------	----------	----------	671
Echinops_telfairi	----------	----------	----------	----------	----------	----------	----------	473
Sorex_araneus	----------	----------	----------	----------	----------	----------	----------	494
Canidae	----------	----------	----------	----------	----------	----------	----------	550
Otolemur_garnettii	----------	----------	----------	----------	----------	----------	----------	413
Gallus	----------	----------	----------	----------	----------	----------	----------	633
Callithrix_jacchus	----------	----------	----------	----------	----------	----------	----------	495
Crocodylus_porosus	----------	----------	----------	----------	----------	----------	----------	527
Erinaceus_europaeus	----------	----------	----------	----------	----------	----------	----------	420
rnd-6_family-7426-LTR-ERV1	----------	----------	----------	----------	----------	----------	----------	1692
Esox_lucius	----------	----------	----------	----------	----------	----------	----------	532
Salmo_salar	----------	----------	----------	----------	----------	----------	----------	801
Didelphidae	----------	----------	----------	----------	----------	----------	----------	642
Latimeria_chalumnae	----------	----------	----------	----------	----------	----------	----------	1766
Monodelphis_domestica	----------	----------	----------	----------	----------	----------	----------	543
Macropus_eugenii	----------	----------	----------	----------	----------	----------	----------	509
Alligator_mississippiensis	----------	----------	----------	----------	----------	----------	----------	566
Chrysemys_picta_bellii	----------	----------	----------	----------	----------	----------	----------	3578
Danio_rerio	----------	----------	----------	----------	----------	----------	----------	699
Taeniopygia	----------	----------	----------	----------	----------	----------	----------	538
Dasypus_novemcinctus	----------	----------	----------	----------	----------	----------	----------	512
LTR_XT	----------	----------	----------	----------	----------	----------	----------	503
Haplorrhini	----------	----------	----------	----------	----------	----------	----------	8801
Catarrhini	----------	----------	----------	----------	----------	----------	----------	7847
Anolis_carolinensis	----------	----------	----------	----------	----------	----------	----------	1184
Murine_leukemia_virus	----------	----------	----------	----------	----------	----------	----------	7615
Phascolarctos_cinereus	----------	----------	----------	----------	----------	----------	----------	7426
Estrildidae	----------	----------	----------	----------	----------	----------	----------	482
Xenopus_Silurana_tropicalis	----------	----------	----------	----------	----------	----------	----------	812
I_XT	----------	----------	----------	----------	----------	----------	----------	7509
Takifugu_rubripes	----------	----------	----------	----------	----------	----------	----------	707
Bos_taurus	----------	----------	----------	----------	----------	----------	----------	533
Homo_sapiens	----------	----------	----------	----------	----------	----------	----------	450
Microcebus_murinus	----------	----------	----------	----------	----------	----------	----------	428
Myotis_lucifugus	----------	----------	----------	----------	----------	----------	----------	432
Equus_caballus	----------	----------	----------	----------	----------	----------	----------	450
Tupaia_belangeri	----------	----------	----------	----------	----------	----------	----------	498
Loxodonta_africana	----------	----------	----------	----------	----------	----------	----------	460
Murinae	----------	----------	----------	----------	----------	----------	----------	489
Canis_lupus_familiaris	----------	----------	----------	----------	----------	----------	----------	536
Sus_scrofa	----------	----------	----------	----------	----------	----------	----------	454
Choloepus_hoffmanni	----------	----------	----------	----------	----------	----------	----------	444
Vicugna_pacos	----------	----------	----------	----------	----------	----------	----------	474
Gavialis_gangeticus	----------	----------	----------	----------	----------	----------	----------	470
Mammalia	----------	----------	----------	----------	----------	----------	----------	1337
Passeroidea	----------	----------	----------	----------	----------	----------	----------	641
Takifugu	----------	----------	----------	----------	----------	----------	----------	629
Pteropus_vampyrus	----------	----------	----------	----------	----------	----------	----------	472
Ornithorhynchus	----------	----------	----------	----------	----------	----------	----------	2785
Oryctolagus_cuniculus	----------	----------	----------	----------	----------	----------	----------	494
Gallus_gallus	----------	----------	----------	----------	----------	----------	----------	508
Hominidae	TTTCTAGAAA	CTTNTGCNTN	CCCNTCTCTC	TNNTNCCTNG	NCTNACNCTN	TANAGNNAGG	NNGAACTTAG	6746
Metatheria	----------	----------	----------	----------	----------	----------	----------	569
Rodentia	TTTCTTGCAG	TCCTGGGTAT	AACTGCT---	-GGTGTCGCC	ACTGGAACGG	CAG-GACTGA	CTACTTCCCT	4335
Monodelphis	----------	----------	----------	----------	----------	----------	----------	857
Consensus	----------	----------	----------	----------	----------	----------	----------	

Conservation

100%

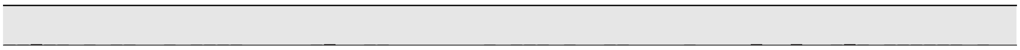

0%

	10,520		10,540		10,560			
Dipodomys_ordii	----------	----------	----------	----------	----------	----------	----------	597
Laurasiatheria	----------	----------	----------	----------	----------	----------	----------	361
Eutheria	----------	----------	----------	----------	----------	----------	----------	452
Tarsius_syrichta	----------	----------	----------	----------	----------	----------	----------	487
Muridae	CTCTTCTGAT	GCTTTCCAAA	CTAAGTAGAC	AGGTTGATCA	GGACCTAGAG	GTATTAAAAG	TTTCGTTTCT	4888
Gasterosteus_aculeatus	----------	----------	----------	----------	----------	----------	----------	2399
Felis_silvestris	----------	----------	----------	----------	----------	----------	----------	505
Rattus	----------	----------	----------	----------	----------	----------	----------	589
Papio_hamadryas	----------	----------	----------	----------	----------	----------	----------	7392
Pan_troglodytes	----------	----------	----------	----------	----------	----------	----------	4138
Ailuropoda_melanoleuca	----------	----------	----------	----------	----------	----------	----------	462
Cavia_porcellus	----------	----------	----------	----------	----------	----------	----------	527
Ictidomys_tridecemlineatus	----------	----------	----------	----------	----------	----------	----------	530
Canis	----------	----------	----------	----------	----------	----------	----------	479
Felis_catus	----------	----------	----------	----------	----------	----------	----------	423
Ochotona_princeps	----------	----------	----------	----------	----------	----------	----------	515
Cercopithecidae	----------	----------	----------	----------	----------	----------	----------	2243
Carnivora	----------	----------	----------	----------	----------	----------	----------	421
Mus	----------	----------	----------	----------	----------	----------	----------	2488
Mus_musculus	----------	----------	----------	----------	----------	----------	----------	478
Rattus_norvegicus	----------	----------	----------	----------	----------	----------	----------	420
Tursiops_truncatus	----------	----------	----------	----------	----------	----------	----------	518
Procavia_capensis	----------	----------	----------	----------	----------	----------	----------	442
Primates	----------	----------	----------	----------	----------	----------	----------	671
Echinops_telfairi	----------	----------	----------	----------	----------	----------	----------	473
Sorex_araneus	----------	----------	----------	----------	----------	----------	----------	494
Canidae	----------	----------	----------	----------	----------	----------	----------	550
Otolemur_garnettii	----------	----------	----------	----------	----------	----------	----------	413
Gallus	----------	----------	----------	----------	----------	----------	----------	633
Callithrix_jacchus	----------	----------	----------	----------	----------	----------	----------	495
Crocodylus_porosus	----------	----------	----------	----------	----------	----------	----------	527
Erinaceus_europaeus	----------	----------	----------	----------	----------	----------	----------	420
rnd-6_family-7426-LTR-ERV1	----------	----------	----------	----------	----------	----------	----------	1692
Esox_lucius	----------	----------	----------	----------	----------	----------	----------	532
Salmo_salar	----------	----------	----------	----------	----------	----------	----------	801
Didelphidae	----------	----------	----------	----------	----------	----------	----------	642
Latimeria_chalumnae	----------	----------	----------	----------	----------	----------	----------	1766
Monodelphis_domestica	----------	----------	----------	----------	----------	----------	----------	543
Macropus_eugenii	----------	----------	----------	----------	----------	----------	----------	509
Alligator_mississippiensis	----------	----------	----------	----------	----------	----------	----------	566
Chrysemys_picta_bellii	----------	----------	----------	----------	----------	----------	----------	3578
Danio_rerio	----------	----------	----------	----------	----------	----------	----------	699
Taeniopygia	----------	----------	----------	----------	----------	----------	----------	538
Dasypus_novemcinctus	----------	----------	----------	----------	----------	----------	----------	512
LTR_XT	----------	----------	----------	----------	----------	----------	----------	503
Haplorrhini	----------	----------	----------	----------	----------	----------	----------	8801
Catarrhini	----------	----------	----------	----------	----------	----------	----------	7847
Anolis_carolinensis	----------	----------	----------	----------	----------	----------	----------	1184
Murine_leukemia_virus	----------	----------	----------	----------	----------	----------	----------	7615
Phascolarctos_cinereus	----------	----------	----------	----------	----------	----------	----------	7426
Estrildidae	----------	----------	----------	----------	----------	----------	----------	482
Xenopus_Silurana_tropicalis	----------	----------	----------	----------	----------	----------	----------	812
I_XT	----------	----------	----------	----------	----------	----------	----------	7509
Takifugu_rubripes	----------	----------	----------	----------	----------	----------	----------	707
Bos_taurus	----------	----------	----------	----------	----------	----------	----------	533
Homo_sapiens	----------	----------	----------	----------	----------	----------	----------	450
Microcebus_murinus	----------	----------	----------	----------	----------	----------	----------	428
Myotis_lucifugus	----------	----------	----------	----------	----------	----------	----------	432
Equus_caballus	----------	----------	----------	----------	----------	----------	----------	450
Tupaia_belangeri	----------	----------	----------	----------	----------	----------	----------	498
Loxodonta_africana	----------	----------	----------	----------	----------	----------	----------	460
Murinae	----------	----------	----------	----------	----------	----------	----------	489
Canis_lupus_familiaris	----------	----------	----------	----------	----------	----------	----------	536
Sus_scrofa	----------	----------	----------	----------	----------	----------	----------	454
Choloepus_hoffmanni	----------	----------	----------	----------	----------	----------	----------	444
Vicugna_pacos	----------	----------	----------	----------	----------	----------	----------	474
Gavialis_gangeticus	----------	----------	----------	----------	----------	----------	----------	470
Mammalia	----------	----------	----------	----------	----------	----------	----------	1337
Passeroidea	----------	----------	----------	----------	----------	----------	----------	641
Takifugu	----------	----------	----------	----------	----------	----------	----------	629
Pteropus_vampyrus	----------	----------	----------	----------	----------	----------	----------	472
Ornithorhynchus	----------	----------	----------	----------	----------	----------	----------	2785
Oryctolagus_cuniculus	----------	----------	----------	----------	----------	----------	----------	494
Gallus_gallus	----------	----------	----------	----------	----------	----------	----------	508
Hominidae	CNNTNNNCTN	GNCTGGANCN	NGCCNANGCN	NCNNAATAAA	AGCAANCGGG	CNNTTTTCCN	ACCNNTNGTA	6816
Metatheria	----------	----------	----------	----------	----------	----------	----------	569
Rodentia	AGCTATTTAC	CATCGGTTTT	CCTCTCAGTT	CATCCAGGAT	CTCCAACGGG	TGGCTCAGAC	CATCCTCACC	4405
Monodelphis	----------	----------	----------	----------	----------	----------	----------	857
Consensus	----------	----------	----------	----------	----------	----------	----------	

Conservation

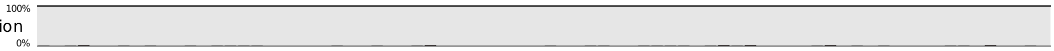

	10,580		10,600		10,620		10,640	
Dipodomys_ordii	----------	----------	----------	----------	----------	----------	----------	597
Laurasiatheria	----------	----------	----------	----------	----------	----------	----------	361
Eutheria	----------	----------	----------	----------	----------	----------	----------	452
Tarsius_syrichta	----------	----------	----------	----------	----------	----------	----------	487
Muridae	GAATTAAAAC	AACAGTTAGA	TTCCCTTGCA	GAAATGGTCC	TACAAAATTG	GAGAGGCTTA	GACTTACTTC	4958
Gasterosteus_aculeatus	----------	----------	----------	----------	----------	----------	----------	2399
Felis_silvestris	----------	----------	----------	----------	----------	----------	----------	505
Rattus	----------	----------	----------	----------	----------	----------	----------	589
Papio_hamadryas	----------	----------	----------	----------	----------	----------	----------	7392
Pan_troglodytes	----------	----------	----------	----------	----------	----------	----------	4138
Ailuropoda_melanoleuca	----------	----------	----------	----------	----------	----------	----------	462
Cavia_porcellus	----------	----------	----------	----------	----------	----------	----------	527
Ictidomys_tridecemlineatus	----------	----------	----------	----------	----------	----------	----------	530
Canis	----------	----------	----------	----------	----------	----------	----------	479
Felis_catus	----------	----------	----------	----------	----------	----------	----------	423
Ochotona_princeps	----------	----------	----------	----------	----------	----------	----------	515
Cercopithecidae	----------	----------	----------	----------	----------	----------	----------	2243
Carnivora	----------	----------	----------	----------	----------	----------	----------	421
Mus	----------	----------	----------	----------	----------	----------	----------	2488
Mus_musculus	----------	----------	----------	----------	----------	----------	----------	478
Rattus_norvegicus	----------	----------	----------	----------	----------	----------	----------	420
Tursiops_truncatus	----------	----------	----------	----------	----------	----------	----------	518
Procavia_capensis	----------	----------	----------	----------	----------	----------	----------	442
Primates	----------	----------	----------	----------	----------	----------	----------	671
Echinops_telfairi	----------	----------	----------	----------	----------	----------	----------	473
Sorex_araneus	----------	----------	----------	----------	----------	----------	----------	494
Canidae	----------	----------	----------	----------	----------	----------	----------	550
Otolemur_garnettii	----------	----------	----------	----------	----------	----------	----------	413
Gallus	----------	----------	----------	----------	----------	----------	----------	633
Callithrix_jacchus	----------	----------	----------	----------	----------	----------	----------	495
Crocodylus_porosus	----------	----------	----------	----------	----------	----------	----------	527
Erinaceus_europaeus	----------	----------	----------	----------	----------	----------	----------	420
rnd-6_family-7426-LTR-ERV1	----------	----------	----------	----------	----------	----------	----------	1692
Esox_lucius	----------	----------	----------	----------	----------	----------	----------	532
Salmo_salar	----------	----------	----------	----------	----------	----------	----------	801
Didelphidae	----------	----------	----------	----------	----------	----------	----------	642
Latimeria_chalumnae	----------	----------	----------	----------	----------	----------	----------	1766
Monodelphis_domestica	----------	----------	----------	----------	----------	----------	----------	543
Macropus_eugenii	----------	----------	----------	----------	----------	----------	----------	509
Alligator_mississippiensis	----------	----------	----------	----------	----------	----------	----------	566
Chrysemys_picta_bellii	----------	----------	----------	----------	----------	----------	----------	3578
Danio_rerio	----------	----------	----------	----------	----------	----------	----------	699
Taeniopygia	----------	----------	----------	----------	----------	----------	----------	538
Dasypus_novemcinctus	----------	----------	----------	----------	----------	----------	----------	512
LTR_XT	----------	----------	----------	----------	----------	----------	----------	503
Haplorrhini	----------	----------	----------	----------	----------	----------	----------	8801
Catarrhini	----------	----------	----------	----------	----------	----------	----------	7847
Anolis_carolinensis	----------	----------	----------	----------	----------	----------	----------	1184
Murine_leukemia_virus	----------	----------	----------	----------	----------	----------	----------	7615
Phascolarctos_cinereus	----------	----------	----------	----------	----------	----------	----------	7426
Estrildidae	----------	----------	----------	----------	----------	----------	----------	482
Xenopus_Silurana_tropicalis	----------	----------	----------	----------	----------	----------	----------	812
I_XT	----------	----------	----------	----------	----------	----------	----------	7509
Takifugu_rubripes	----------	----------	----------	----------	----------	----------	----------	707
Bos_taurus	----------	----------	----------	----------	----------	----------	----------	533
Homo_sapiens	----------	----------	----------	----------	----------	----------	----------	450
Microcebus_murinus	----------	----------	----------	----------	----------	----------	----------	428
Myotis_lucifugus	----------	----------	----------	----------	----------	----------	----------	432
Equus_caballus	----------	----------	----------	----------	----------	----------	----------	450
Tupaia_belangeri	----------	----------	----------	----------	----------	----------	----------	498
Loxodonta_africana	----------	----------	----------	----------	----------	----------	----------	460
Murinae	----------	----------	----------	----------	----------	----------	----------	489
Canis_lupus_familiaris	----------	----------	----------	----------	----------	----------	----------	536
Sus_scrofa	----------	----------	----------	----------	----------	----------	----------	454
Choloepus_hoffmanni	----------	----------	----------	----------	----------	----------	----------	444
Vicugna_pacos	----------	----------	----------	----------	----------	----------	----------	474
Gavialis_gangeticus	----------	----------	----------	----------	----------	----------	----------	470
Mammalia	----------	----------	----------	----------	----------	----------	----------	1337
Passeroidea	----------	----------	----------	----------	----------	----------	----------	641
Takifugu	----------	----------	----------	----------	----------	----------	----------	629
Pteropus_vampyrus	----------	----------	----------	----------	----------	----------	----------	472
Ornithorhynchus	----------	----------	----------	----------	----------	----------	----------	2785
Oryctolagus_cuniculus	----------	----------	----------	----------	----------	----------	----------	494
Gallus_gallus	----------	----------	----------	----------	----------	----------	----------	508
Hominidae	NTAGGNNTCT	CCTTNNCCTC	CTCNNTNGTN	GCNTCNGGGC	TNGGNANAGG	NGCNNTNNNN	CANTNNATNN	6886
Metatheria	----------	----------	----------	----------	----------	----------	----------	569
Rodentia	CTCCAGGGCC	AAATAGACTC	ACTGGCNGCC	GTGGTGCTAC	AAAATCGGCG	AGGATTAGAT	TTACTAACAG	4475
Monodelphis	----------	----------	----------	----------	----------	----------	----------	857
Consensus	----------	----------	----------	----------	----------	----------	----------	
Conservation (100% / 0%)								

		10,660		10,680		10,700		
Dipodomys_ordii	----------	----------	----------	----------	----------	----------	----------	597
Laurasiatheria	----------	----------	----------	----------	----------	----------	----------	361
Eutheria	----------	----------	----------	----------	----------	----------	----------	452
Tarsius_syrichta	----------	----------	----------	----------	----------	----------	----------	487
Muridae	TCATGAGATA	AAGGAAACTA	TGTATGGCTT	TGGGAGAAAC	CTGTTGTTTC	TATGCTAATT	GATCAGGAGT	5028
Gasterosteus_aculeatus	----------	----------	----------	----------	----------	----------	----------	2399
Felis_silvestris	----------	----------	----------	----------	----------	----------	----------	505
Rattus	----------	----------	----------	----------	----------	----------	----------	589
Papio_hamadryas	----------	----------	----------	----------	----------	----------	----------	7392
Pan_troglodytes	----------	----------	----------	----------	----------	----------	----------	4138
Ailuropoda_melanoleuca	----------	----------	----------	----------	----------	----------	----------	462
Cavia_porcellus	----------	----------	----------	----------	----------	----------	----------	527
Ictidomys_tridecemlineatus	----------	----------	----------	----------	----------	----------	----------	530
Canis	----------	----------	----------	----------	----------	----------	----------	479
Felis_catus	----------	----------	----------	----------	----------	----------	----------	423
Ochotona_princeps	----------	----------	----------	----------	----------	----------	----------	515
Cercopithecidae	----------	----------	----------	----------	----------	----------	----------	2243
Carnivora	----------	----------	----------	----------	----------	----------	----------	421
Mus	----------	----------	----------	----------	----------	----------	----------	2488
Mus_musculus	----------	----------	----------	----------	----------	----------	----------	478
Rattus_norvegicus	----------	----------	----------	----------	----------	----------	----------	420
Tursiops_truncatus	----------	----------	----------	----------	----------	----------	----------	518
Procavia_capensis	----------	----------	----------	----------	----------	----------	----------	442
Primates	----------	----------	----------	----------	----------	----------	----------	671
Echinops_telfairi	----------	----------	----------	----------	----------	----------	----------	473
Sorex_araneus	----------	----------	----------	----------	----------	----------	----------	494
Canidae	----------	----------	----------	----------	----------	----------	----------	550
Otolemur_garnettii	----------	----------	----------	----------	----------	----------	----------	413
Gallus	----------	----------	----------	----------	----------	----------	----------	633
Callithrix_jacchus	----------	----------	----------	----------	----------	----------	----------	495
Crocodylus_porosus	----------	----------	----------	----------	----------	----------	----------	527
Erinaceus_europaeus	----------	----------	----------	----------	----------	----------	----------	420
rnd-6_family-7426-LTR-ERV1	----------	----------	----------	----------	----------	----------	----------	1692
Esox_lucius	----------	----------	----------	----------	----------	----------	----------	532
Salmo_salar	----------	----------	----------	----------	----------	----------	----------	801
Didelphidae	----------	----------	----------	----------	----------	----------	----------	642
Latimeria_chalumnae	----------	----------	----------	----------	----------	----------	----------	1766
Monodelphis_domestica	----------	----------	----------	----------	----------	----------	----------	543
Macropus_eugenii	----------	----------	----------	----------	----------	----------	----------	509
Alligator_mississippiensis	----------	----------	----------	----------	----------	----------	----------	566
Chrysemys_picta_bellii	----------	----------	----------	----------	----------	----------	----------	3578
Danio_rerio	----------	----------	----------	----------	----------	----------	----------	699
Taeniopygia	----------	----------	----------	----------	----------	----------	----------	538
Dasypus_novemcinctus	----------	----------	----------	----------	----------	----------	----------	512
LTR_XT	----------	----------	----------	----------	----------	----------	----------	503
Haplorrhini	----------	----------	----------	----------	----------	----------	----------	8801
Catarrhini	----------	----------	----------	----------	----------	----------	----------	7847
Anolis_carolinensis	----------	----------	----------	----------	----------	----------	----------	1184
Murine_leukemia_virus	----------	----------	----------	----------	----------	----------	----------	7615
Phascolarctos_cinereus	----------	----------	----------	----------	----------	----------	----------	7426
Estrildidae	----------	----------	----------	----------	----------	----------	----------	482
Xenopus_Silurana_tropicalis	----------	----------	----------	----------	----------	----------	----------	812
I_XT	----------	----------	----------	----------	----------	----------	----------	7509
Takifugu_rubripes	----------	----------	----------	----------	----------	----------	----------	707
Bos_taurus	----------	----------	----------	----------	----------	----------	----------	533
Homo_sapiens	----------	----------	----------	----------	----------	----------	----------	450
Microcebus_murinus	----------	----------	----------	----------	----------	----------	----------	428
Myotis_lucifugus	----------	----------	----------	----------	----------	----------	----------	432
Equus_caballus	----------	----------	----------	----------	----------	----------	----------	450
Tupaia_belangeri	----------	----------	----------	----------	----------	----------	----------	498
Loxodonta_africana	----------	----------	----------	----------	----------	----------	----------	460
Murinae	----------	----------	----------	----------	----------	----------	----------	489
Canis_lupus_familiaris	----------	----------	----------	----------	----------	----------	----------	536
Sus_scrofa	----------	----------	----------	----------	----------	----------	----------	454
Choloepus_hoffmanni	----------	----------	----------	----------	----------	----------	----------	444
Vicugna_pacos	----------	----------	----------	----------	----------	----------	----------	474
Gavialis_gangeticus	----------	----------	----------	----------	----------	----------	----------	470
Mammalia	----------	----------	----------	----------	----------	----------	----------	1337
Passeroidea	----------	----------	----------	----------	----------	----------	----------	641
Takifugu	----------	----------	----------	----------	----------	----------	----------	629
Pteropus_vampyrus	----------	----------	----------	----------	----------	----------	----------	472
Ornithorhynchus	----------	----------	----------	----------	----------	----------	----------	2785
Oryctolagus_cuniculus	----------	----------	----------	----------	----------	----------	----------	494
Gallus_gallus	----------	----------	----------	----------	----------	----------	----------	508
Hominidae	NNNCNTCNCA	AGATCTNTCT	ANTNNNCTNC	AGNTGGCCAT	NGANGCNTCN	GCCGANTCCN	TNGCCTCNCT	6956
Metatheria	----------	----------	----------	----------	----------	----------	----------	569
Rodentia	CAGAGAGAGG	TGGTCTTTGT	CTCTTCCTCG	GGGAGGAATG	TTGCTTCTAT	GTCAACCAGT	CAGGTATAGT	4545
Monodelphis	----------	----------	----------	----------	----------	----------	----------	857
Consensus	----------	----------	----------	----------	----------	----------	----------	

Conservation (100% / 0%)

	10,720 · 10,740 · 10,760 · 10,780	
Dipodomys_ordii	---------- ---------- ---------- ---------- ---------- ---------- ----------	597
Laurasiatheria	---------- ---------- ---------- ---------- ---------- ---------- ----------	361
Eutheria	---------- ---------- ---------- ---------- ---------- ---------- ----------	452
Tarsius_syrichta	---------- ---------- ---------- ---------- ---------- ---------- ----------	487
Muridae	AATTAGAAAA AATCTTGCCA TGGTTGGGGA AAACTCAAAA AGAAAAAGAG ACAAAGACAT AAATCAGATA	5098
Gasterosteus_aculeatus	---------- ---------- ---------- ---------- ---------- ---------- ----------	2399
Felis_silvestris	---------- ---------- ---------- ---------- ---------- ---------- ----------	505
Rattus	---------- ---------- ---------- ---------- ---------- ---------- ----------	589
Papio_hamadryas	---------- ---------- ---------- ---------- ---------- ---------- ----------	7392
Pan_troglodytes	---------- ---------- ---------- ---------- ---------- ---------- ----------	4138
Ailuropoda_melanoleuca	---------- ---------- ---------- ---------- ---------- ---------- ----------	462
Cavia_porcellus	---------- ---------- ---------- ---------- ---------- ---------- ----------	527
Ictidomys_tridecemlineatus	---------- ---------- ---------- ---------- ---------- ---------- ----------	530
Canis	---------- ---------- ---------- ---------- ---------- ---------- ----------	479
Felis_catus	---------- ---------- ---------- ---------- ---------- ---------- ----------	423
Ochotona_princeps	---------- ---------- ---------- ---------- ---------- ---------- ----------	515
Cercopithecidae	---------- ---------- ---------- ---------- ---------- ---------- ----------	2243
Carnivora	---------- ---------- ---------- ---------- ---------- ---------- ----------	421
Mus	---------- ---------- ---------- ---------- ---------- ---------- ----------	2488
Mus_musculus	---------- ---------- ---------- ---------- ---------- ---------- ----------	478
Rattus_norvegicus	---------- ---------- ---------- ---------- ---------- ---------- ----------	420
Tursiops_truncatus	---------- ---------- ---------- ---------- ---------- ---------- ----------	518
Procavia_capensis	---------- ---------- ---------- ---------- ---------- ---------- ----------	442
Primates	---------- ---------- ---------- ---------- ---------- ---------- ----------	671
Echinops_telfairi	---------- ---------- ---------- ---------- ---------- ---------- ----------	473
Sorex_araneus	---------- ---------- ---------- ---------- ---------- ---------- ----------	494
Canidae	---------- ---------- ---------- ---------- ---------- ---------- ----------	550
Otolemur_garnettii	---------- ---------- ---------- ---------- ---------- ---------- ----------	413
Gallus	---------- ---------- ---------- ---------- ---------- ---------- ----------	633
Callithrix_jacchus	---------- ---------- ---------- ---------- ---------- ---------- ----------	495
Crocodylus_porosus	---------- ---------- ---------- ---------- ---------- ---------- ----------	527
Erinaceus_europaeus	---------- ---------- ---------- ---------- ---------- ---------- ----------	420
rnd-6_family-7426-LTR-ERV1	---------- ---------- ---------- ---------- ---------- ---------- ----------	1692
Esox_lucius	---------- ---------- ---------- ---------- ---------- ---------- ----------	532
Salmo_salar	---------- ---------- ---------- ---------- ---------- ---------- ----------	801
Didelphidae	---------- ---------- ---------- ---------- ---------- ---------- ----------	642
Latimeria_chalumnae	---------- ---------- ---------- ---------- ---------- ---------- ----------	1766
Monodelphis_domestica	---------- ---------- ---------- ---------- ---------- ---------- ----------	543
Macropus_eugenii	---------- ---------- ---------- ---------- ---------- ---------- ----------	509
Alligator_mississippiensis	---------- ---------- ---------- ---------- ---------- ---------- ----------	566
Chrysemys_picta_bellii	---------- ---------- ---------- ---------- ---------- ---------- ----------	3578
Danio_rerio	---------- ---------- ---------- ---------- ---------- ---------- ----------	699
Taeniopygia	---------- ---------- ---------- ---------- ---------- ---------- ----------	538
Dasypus_novemcinctus	---------- ---------- ---------- ---------- ---------- ---------- ----------	512
LTR_XT	---------- ---------- ---------- ---------- ---------- ---------- ----------	503
Haplorrhini	---------- ---------- ---------- ---------- ---------- ---------- ----------	8801
Catarrhini	---------- ---------- ---------- ---------- ---------- ---------- ----------	7847
Anolis_carolinensis	---------- ---------- ---------- ---------- ---------- ---------- ----------	1184
Murine_leukemia_virus	---------- ---------- ---------- ---------- ---------- ---------- ----------	7615
Phascolarctos_cinereus	---------- ---------- ---------- ---------- ---------- ---------- ----------	7426
Estrildidae	---------- ---------- ---------- ---------- ---------- ---------- ----------	482
Xenopus_Silurana_tropicalis	---------- ---------- ---------- ---------- ---------- ---------- ----------	812
I_XT	---------- ---------- ---------- ---------- ---------- ---------- ----------	7509
Takifugu_rubripes	---------- ---------- ---------- ---------- ---------- ---------- ----------	707
Bos_taurus	---------- ---------- ---------- ---------- ---------- ---------- ----------	533
Homo_sapiens	---------- ---------- ---------- ---------- ---------- ---------- ----------	450
Microcebus_murinus	---------- ---------- ---------- ---------- ---------- ---------- ----------	428
Myotis_lucifugus	---------- ---------- ---------- ---------- ---------- ---------- ----------	432
Equus_caballus	---------- ---------- ---------- ---------- ---------- ---------- ----------	450
Tupaia_belangeri	---------- ---------- ---------- ---------- ---------- ---------- ----------	498
Loxodonta_africana	---------- ---------- ---------- ---------- ---------- ---------- ----------	460
Murinae	---------- ---------- ---------- ---------- ---------- ---------- ----------	489
Canis_lupus_familiaris	---------- ---------- ---------- ---------- ---------- ---------- ----------	536
Sus_scrofa	---------- ---------- ---------- ---------- ---------- ---------- ----------	454
Choloepus_hoffmanni	---------- ---------- ---------- ---------- ---------- ---------- ----------	444
Vicugna_pacos	---------- ---------- ---------- ---------- ---------- ---------- ----------	474
Gavialis_gangeticus	---------- ---------- ---------- ---------- ---------- ---------- ----------	470
Mammalia	---------- ---------- ---------- ---------- ---------- ---------- ----------	1337
Passeroidea	---------- ---------- ---------- ---------- ---------- ---------- ----------	641
Takifugu	---------- ---------- ---------- ---------- ---------- ---------- ----------	629
Pteropus_vampyrus	---------- ---------- ---------- ---------- ---------- ---------- ----------	472
Ornithorhynchus	---------- ---------- ---------- ---------- ---------- ---------- ----------	2785
Oryctolagus_cuniculus	---------- ---------- ---------- ---------- ---------- ---------- ----------	494
Gallus_gallus	---------- ---------- ---------- ---------- ---------- ---------- ----------	508
Hominidae	ACAGNGACAG ATNACNTCNG TNGCNNAGGT NGCNNNNCAG AACNGGNGNG CCNTAGANNT NCTNACNGCN	7026
Metatheria	---------- ---------- ---------- ---------- ---------- ---------- ----------	569
Rodentia	A-AAAGATAA GATCCGACAA CTCCAGACAG ATCTCCGAAA ACGTNAAGAA ACAGCTTGAT GCCTCTGGCC	4614
Monodelphis	---------- ---------- ---------- ---------- ---------- ---------- ----------	857
Consensus	---------- ---------- ---------- ---------- ---------- ---------- ----------	
Conservation	100% / 0%	

	10,800		10,820		10,840			
Dipodomys_ordii	----------	----------	----------	----------	----------	----------	----------	597
Laurasiatheria	----------	----------	----------	----------	----------	----------	----------	361
Eutheria	----------	----------	----------	----------	----------	----------	----------	452
Tarsius_syrichta	----------	----------	----------	----------	----------	----------	----------	487
Muridae	ATTGGTACCA	GTCTCTGTTC	TCTTGGTCCC	CTGGCTAACT	ACTCTGCTAT	CAGCACTGGC	TGGGCCTTTA	5168
Gasterosteus_aculeatus	----------	----------	----------	----------	----------	----------	----------	2399
Felis_silvestris	----------	----------	----------	----------	----------	----------	----------	505
Rattus	----------	----------	----------	----------	----------	----------	----------	589
Papio_hamadryas	----------	----------	----------	----------	----------	----------	----------	7392
Pan_troglodytes	----------	----------	----------	----------	----------	----------	----------	4138
Ailuropoda_melanoleuca	----------	----------	----------	----------	----------	----------	----------	462
Cavia_porcellus	----------	----------	----------	----------	----------	----------	----------	527
Ictidomys_tridecemlineatus	----------	----------	----------	----------	----------	----------	----------	530
Canis	----------	----------	----------	----------	----------	----------	----------	479
Felis_catus	----------	----------	----------	----------	----------	----------	----------	423
Ochotona_princeps	----------	----------	----------	----------	----------	----------	----------	515
Cercopithecidae	----------	----------	----------	----------	----------	----------	----------	2243
Carnivora	----------	----------	----------	----------	----------	----------	----------	421
Mus	----------	----------	----------	----------	----------	----------	----------	2488
Mus_musculus	----------	----------	----------	----------	----------	----------	----------	478
Rattus_norvegicus	----------	----------	----------	----------	----------	----------	----------	420
Tursiops_truncatus	----------	----------	----------	----------	----------	----------	----------	518
Procavia_capensis	----------	----------	----------	----------	----------	----------	----------	442
Primates	----------	----------	----------	----------	----------	----------	----------	671
Echinops_telfairi	----------	----------	----------	----------	----------	----------	----------	473
Sorex_araneus	----------	----------	----------	----------	----------	----------	----------	494
Canidae	----------	----------	----------	----------	----------	----------	----------	550
Otolemur_garnettii	----------	----------	----------	----------	----------	----------	----------	413
Gallus	----------	----------	----------	----------	----------	----------	----------	633
Callithrix_jacchus	----------	----------	----------	----------	----------	----------	----------	495
Crocodylus_porosus	----------	----------	----------	----------	----------	----------	----------	527
Erinaceus_europaeus	----------	----------	----------	----------	----------	----------	----------	420
rnd-6_family-7426-LTR-ERV1	----------	----------	----------	----------	----------	----------	----------	1692
Esox_lucius	----------	----------	----------	----------	----------	----------	----------	532
Salmo_salar	----------	----------	----------	----------	----------	----------	----------	801
Didelphidae	----------	----------	----------	----------	----------	----------	----------	642
Latimeria_chalumnae	----------	----------	----------	----------	----------	----------	----------	1766
Monodelphis_domestica	----------	----------	----------	----------	----------	----------	----------	543
Macropus_eugenii	----------	----------	----------	----------	----------	----------	----------	509
Alligator_mississippiensis	----------	----------	----------	----------	----------	----------	----------	566
Chrysemys_picta_bellii	----------	----------	----------	----------	----------	----------	----------	3578
Danio_rerio	----------	----------	----------	----------	----------	----------	----------	699
Taeniopygia	----------	----------	----------	----------	----------	----------	----------	538
Dasypus_novemcinctus	----------	----------	----------	----------	----------	----------	----------	512
LTR_XT	----------	----------	----------	----------	----------	----------	----------	503
Haplorrhini	----------	----------	----------	----------	----------	----------	----------	8801
Catarrhini	----------	----------	----------	----------	----------	----------	----------	7847
Anolis_carolinensis	----------	----------	----------	----------	----------	----------	----------	1184
Murine_leukemia_virus	----------	----------	----------	----------	----------	----------	----------	7615
Phascolarctos_cinereus	----------	----------	----------	----------	----------	----------	----------	7426
Estrildidae	----------	----------	----------	----------	----------	----------	----------	482
Xenopus_Silurana_tropicalis	----------	----------	----------	----------	----------	----------	----------	812
I_XT	----------	----------	----------	----------	----------	----------	----------	7509
Takifugu_rubripes	----------	----------	----------	----------	----------	----------	----------	707
Bos_taurus	----------	----------	----------	----------	----------	----------	----------	533
Homo_sapiens	----------	----------	----------	----------	----------	----------	----------	450
Microcebus_murinus	----------	----------	----------	----------	----------	----------	----------	428
Myotis_lucifugus	----------	----------	----------	----------	----------	----------	----------	432
Equus_caballus	----------	----------	----------	----------	----------	----------	----------	450
Tupaia_belangeri	----------	----------	----------	----------	----------	----------	----------	498
Loxodonta_africana	----------	----------	----------	----------	----------	----------	----------	460
Murinae	----------	----------	----------	----------	----------	----------	----------	489
Canis_lupus_familiaris	----------	----------	----------	----------	----------	----------	----------	536
Sus_scrofa	----------	----------	----------	----------	----------	----------	----------	454
Choloepus_hoffmanni	----------	----------	----------	----------	----------	----------	----------	444
Vicugna_pacos	----------	----------	----------	----------	----------	----------	----------	474
Gavialis_gangeticus	----------	----------	----------	----------	----------	----------	----------	470
Mammalia	----------	----------	----------	----------	----------	----------	----------	1337
Passeroidea	----------	----------	----------	----------	----------	----------	----------	641
Takifugu	----------	----------	----------	----------	----------	----------	----------	629
Pteropus_vampyrus	----------	----------	----------	----------	----------	----------	----------	472
Ornithorhynchus	----------	----------	----------	----------	----------	----------	----------	2785
Oryctolagus_cuniculus	----------	----------	----------	----------	----------	----------	----------	494
Gallus_gallus	----------	----------	----------	----------	----------	----------	----------	508
Hominidae	GANAAAGGNG	GNACNTGNNT	NTTNNTNNNG	GAAGANTGCT	GNTATTANNT	CAANGANTCA	GGNNTAGTNG	7096
Metatheria	----------	----------	----------	----------	----------	----------	----------	569
Rodentia	AGTGGNTCAT	TGACAGTCCA	GTATGGAATT	GGATACTNCC	CTTCTTAACC	CCTTTCTCCT	CCTTTTCTTA	4684
Monodelphis	----------	----------	----------	----------	----------	----------	----------	857
Consensus	----------	----------	----------	----------	----------	----------	----------	

Conservation (100% / 0%)

		10,860		10,880		10,900		10,920	
Dipodomys_ordii	----------	----------	----------	----------	----------	----------	----------		597
Laurasiatheria	----------	----------	----------	----------	----------	----------	----------		361
Eutheria	----------	----------	----------	----------	----------	----------	----------		452
Tarsius_syrichta	----------	----------	----------	----------	----------	----------	----------		487
Muridae	ATTCTCATTC	TGATTGGATT	AACAGCGGGG	CCCTGCATCT	TAAACTATAT	GGCAATATGT	ATCAGTTCAG		5238
Gasterosteus_aculeatus	----------	----------	----------	----------	----------	----------	----------		2399
Felis_silvestris	----------	----------	----------	----------	----------	----------	----------		505
Rattus	----------	----------	----------	----------	----------	----------	----------		589
Papio_hamadryas	----------	----------	----------	----------	----------	----------	----------		7392
Pan_troglodytes	----------	----------	----------	----------	----------	----------	----------		4138
Ailuropoda_melanoleuca	----------	----------	----------	----------	----------	----------	----------		462
Cavia_porcellus	----------	----------	----------	----------	----------	----------	----------		527
Ictidomys_tridecemlineatus	----------	----------	----------	----------	----------	----------	----------		530
Canis	----------	----------	----------	----------	----------	----------	----------		479
Felis_catus	----------	----------	----------	----------	----------	----------	----------		423
Ochotona_princeps	----------	----------	----------	----------	----------	----------	----------		515
Cercopithecidae	----------	----------	----------	----------	----------	----------	----------		2243
Carnivora	----------	----------	----------	----------	----------	----------	----------		421
Mus	----------	----------	----------	----------	----------	----------	----------		2488
Mus_musculus	----------	----------	----------	----------	----------	----------	----------		478
Rattus_norvegicus	----------	----------	----------	----------	----------	----------	----------		420
Tursiops_truncatus	----------	----------	----------	----------	----------	----------	----------		518
Procavia_capensis	----------	----------	----------	----------	----------	----------	----------		442
Primates	----------	----------	----------	----------	----------	----------	----------		671
Echinops_telfairi	----------	----------	----------	----------	----------	----------	----------		473
Sorex_araneus	----------	----------	----------	----------	----------	----------	----------		494
Canidae	----------	----------	----------	----------	----------	----------	----------		550
Otolemur_garnettii	----------	----------	----------	----------	----------	----------	----------		413
Gallus	----------	----------	----------	----------	----------	----------	----------		633
Callithrix_jacchus	----------	----------	----------	----------	----------	----------	----------		495
Crocodylus_porosus	----------	----------	----------	----------	----------	----------	----------		527
Erinaceus_europaeus	----------	----------	----------	----------	----------	----------	----------		420
rnd-6_family-7426-LTR-ERV1	----------	----------	----------	----------	----------	----------	----------		1692
Esox_lucius	----------	----------	----------	----------	----------	----------	----------		532
Salmo_salar	----------	----------	----------	----------	----------	----------	----------		801
Didelphidae	----------	----------	----------	----------	----------	----------	----------		642
Latimeria_chalumnae	----------	----------	----------	----------	----------	----------	----------		1766
Monodelphis_domestica	----------	----------	----------	----------	----------	----------	----------		543
Macropus_eugenii	----------	----------	----------	----------	----------	----------	----------		509
Alligator_mississippiensis	----------	----------	----------	----------	----------	----------	----------		566
Chrysemys_picta_bellii	----------	----------	----------	----------	----------	----------	----------		3578
Danio_rerio	----------	----------	----------	----------	----------	----------	----------		699
Taeniopygia	----------	----------	----------	----------	----------	----------	----------		538
Dasypus_novemcinctus	----------	----------	----------	----------	----------	----------	----------		512
LTR_XT	----------	----------	----------	----------	----------	----------	----------		503
Haplorrhini	----------	----------	----------	----------	----------	----------	----------		8801
Catarrhini	----------	----------	----------	----------	----------	----------	----------		7847
Anolis_carolinensis	----------	----------	----------	----------	----------	----------	----------		1184
Murine_leukemia_virus	----------	----------	----------	----------	----------	----------	----------		7615
Phascolarctos_cinereus	----------	----------	----------	----------	----------	----------	----------		7426
Estrildidae	----------	----------	----------	----------	----------	----------	----------		482
Xenopus_Silurana_tropicalis	----------	----------	----------	----------	----------	----------	----------		812
I_XT	----------	----------	----------	----------	----------	----------	----------		7509
Takifugu_rubripes	----------	----------	----------	----------	----------	----------	----------		707
Bos_taurus	----------	----------	----------	----------	----------	----------	----------		533
Homo_sapiens	----------	----------	----------	----------	----------	----------	----------		450
Microcebus_murinus	----------	----------	----------	----------	----------	----------	----------		428
Myotis_lucifugus	----------	----------	----------	----------	----------	----------	----------		432
Equus_caballus	----------	----------	----------	----------	----------	----------	----------		450
Tupaia_belangeri	----------	----------	----------	----------	----------	----------	----------		498
Loxodonta_africana	----------	----------	----------	----------	----------	----------	----------		460
Murinae	----------	----------	----------	----------	----------	----------	----------		489
Canis_lupus_familiaris	----------	----------	----------	----------	----------	----------	----------		536
Sus_scrofa	----------	----------	----------	----------	----------	----------	----------		454
Choloepus_hoffmanni	----------	----------	----------	----------	----------	----------	----------		444
Vicugna_pacos	----------	----------	----------	----------	----------	----------	----------		474
Gavialis_gangeticus	----------	----------	----------	----------	----------	----------	----------		470
Mammalia	----------	----------	----------	----------	----------	----------	----------		1337
Passeroidea	----------	----------	----------	----------	----------	----------	----------		641
Takifugu	----------	----------	----------	----------	----------	----------	----------		629
Pteropus_vampyrus	----------	----------	----------	----------	----------	----------	----------		472
Ornithorhynchus	----------	----------	----------	----------	----------	----------	----------		2785
Oryctolagus_cuniculus	----------	----------	----------	----------	----------	----------	----------		494
Gallus_gallus	----------	----------	----------	----------	----------	----------	----------		508
Hominidae	ANACCANNNT	NCNNACNNTN	NANAAAAAAA	AGATCCNNGA	NGNNNTNNAN	CNNNCCTNCN	NNNCTCNCNN		7166
Metatheria	----------	----------	----------	----------	----------	----------	----------		569
Rodentia	ATCCTACTAA	TCGCACCCTG	CCTCATAAAC	---TTCTTGT	CTAGATTTCT	TCAACAACAG	ATCCAAAAGA		4751
Monodelphis	----------	----------	----------	----------	----------	----------	----------		857
Consensus	----------	----------	----------	----------	----------	----------	----------		

Conservation (100% / 0%)

10,940 10,960 10,980

Dipodomys_ordii	---------- ---------- ---------- ---------- ---------- ---------- ----------	597
Laurasiatheria	---------- ---------- ---------- ---------- ---------- ---------- ----------	361
Eutheria	---------- ---------- ---------- ---------- ---------- ---------- ----------	452
Tarsius_syrichta	---------- ---------- ---------- ---------- ---------- ---------- ----------	487
Muridae	TAAAATTAAT GGTCCTTGGA CCAACTACAC CTCTTTAGAG CAGGATGAGT CCACGATTTG ACTCATTCAC	5308
Gasterosteus_aculeatus	---------- ---------- ---------- ---------- ---------- ---------- ----------	2399
Felis_silvestris	---------- ---------- ---------- ---------- ---------- ---------- ----------	505
Rattus	---------- ---------- ---------- ---------- ---------- ---------- ----------	589
Papio_hamadryas	---------- ---------- ---------- ---------- ---------- ---------- ----------	7392
Pan_troglodytes	---------- ---------- ---------- ---------- ---------- ---------- ----------	4138
Ailuropoda_melanoleuca	---------- ---------- ---------- ---------- ---------- ---------- ----------	462
Cavia_porcellus	---------- ---------- ---------- ---------- ---------- ---------- ----------	527
Ictidomys_tridecemlineatus	---------- ---------- ---------- ---------- ---------- ---------- ----------	530
Canis	---------- ---------- ---------- ---------- ---------- ---------- ----------	479
Felis_catus	---------- ---------- ---------- ---------- ---------- ---------- ----------	423
Ochotona_princeps	---------- ---------- ---------- ---------- ---------- ---------- ----------	515
Cercopithecidae	---------- ---------- ---------- ---------- ---------- ---------- ----------	2243
Carnivora	---------- ---------- ---------- ---------- ---------- ---------- ----------	421
Mus	---------- ---------- ---------- ---------- ---------- ---------- ----------	2488
Mus_musculus	---------- ---------- ---------- ---------- ---------- ---------- ----------	478
Rattus_norvegicus	---------- ---------- ---------- ---------- ---------- ---------- ----------	420
Tursiops_truncatus	---------- ---------- ---------- ---------- ---------- ---------- ----------	518
Procavia_capensis	---------- ---------- ---------- ---------- ---------- ---------- ----------	442
Primates	---------- ---------- ---------- ---------- ---------- ---------- ----------	671
Echinops_telfairi	---------- ---------- ---------- ---------- ---------- ---------- ----------	473
Sorex_araneus	---------- ---------- ---------- ---------- ---------- ---------- ----------	494
Canidae	---------- ---------- ---------- ---------- ---------- ---------- ----------	550
Otolemur_garnettii	---------- ---------- ---------- ---------- ---------- ---------- ----------	413
Gallus	---------- ---------- ---------- ---------- ---------- ---------- ----------	633
Callithrix_jacchus	---------- ---------- ---------- ---------- ---------- ---------- ----------	495
Crocodylus_porosus	---------- ---------- ---------- ---------- ---------- ---------- ----------	527
Erinaceus_europaeus	---------- ---------- ---------- ---------- ---------- ---------- ----------	420
rnd-6_family-7426-LTR-ERV1	---------- ---------- ---------- ---------- ---------- ---------- ----------	1692
Esox_lucius	---------- ---------- ---------- ---------- ---------- ---------- ----------	532
Salmo_salar	---------- ---------- ---------- ---------- ---------- ---------- ----------	801
Didelphidae	---------- ---------- ---------- ---------- ---------- ---------- ----------	642
Latimeria_chalumnae	---------- ---------- ---------- ---------- ---------- ---------- ----------	1766
Monodelphis_domestica	---------- ---------- ---------- ---------- ---------- ---------- ----------	543
Macropus_eugenii	---------- ---------- ---------- ---------- ---------- ---------- ----------	509
Alligator_mississippiensis	---------- ---------- ---------- ---------- ---------- ---------- ----------	566
Chrysemys_picta_bellii	---------- ---------- ---------- ---------- ---------- ---------- ----------	3578
Danio_rerio	---------- ---------- ---------- ---------- ---------- ---------- ----------	699
Taeniopygia	---------- ---------- ---------- ---------- ---------- ---------- ----------	538
Dasypus_novemcinctus	---------- ---------- ---------- ---------- ---------- ---------- ----------	512
LTR_XT	---------- ---------- ---------- ---------- ---------- ---------- ----------	503
Haplorrhini	---------- ---------- ---------- ---------- ---------- ---------- ----------	8801
Catarrhini	---------- ---------- ---------- ---------- ---------- ---------- ----------	7847
Anolis_carolinensis	---------- ---------- ---------- ---------- ---------- ---------- ----------	1184
Murine_leukemia_virus	---------- ---------- ---------- ---------- ---------- ---------- ----------	7615
Phascolarctos_cinereus	---------- ---------- ---------- ---------- ---------- ---------- ----------	7426
Estrildidae	---------- ---------- ---------- ---------- ---------- ---------- ----------	482
Xenopus_Silurana_tropicalis	---------- ---------- ---------- ---------- ---------- ---------- ----------	812
I_XT	---------- ---------- ---------- ---------- ---------- ---------- ----------	7509
Takifugu_rubripes	---------- ---------- ---------- ---------- ---------- ---------- ----------	707
Bos_taurus	---------- ---------- ---------- ---------- ---------- ---------- ----------	533
Homo_sapiens	---------- ---------- ---------- ---------- ---------- ---------- ----------	450
Microcebus_murinus	---------- ---------- ---------- ---------- ---------- ---------- ----------	428
Myotis_lucifugus	---------- ---------- ---------- ---------- ---------- ---------- ----------	432
Equus_caballus	---------- ---------- ---------- ---------- ---------- ---------- ----------	450
Tupaia_belangeri	---------- ---------- ---------- ---------- ---------- ---------- ----------	498
Loxodonta_africana	---------- ---------- ---------- ---------- ---------- ---------- ----------	460
Murinae	---------- ---------- ---------- ---------- ---------- ---------- ----------	489
Canis_lupus_familiaris	---------- ---------- ---------- ---------- ---------- ---------- ----------	536
Sus_scrofa	---------- ---------- ---------- ---------- ---------- ---------- ----------	454
Choloepus_hoffmanni	---------- ---------- ---------- ---------- ---------- ---------- ----------	444
Vicugna_pacos	---------- ---------- ---------- ---------- ---------- ---------- ----------	474
Gavialis_gangeticus	---------- ---------- ---------- ---------- ---------- ---------- ----------	470
Mammalia	---------- ---------- ---------- ---------- ---------- ---------- ----------	1337
Passeroidea	---------- ---------- ---------- ---------- ---------- ---------- ----------	641
Takifugu	---------- ---------- ---------- ---------- ---------- ---------- ----------	629
Pteropus_vampyrus	---------- ---------- ---------- ---------- ---------- ---------- ----------	472
Ornithorhynchus	---------- ---------- ---------- ---------- ---------- ---------- ----------	2785
Oryctolagus_cuniculus	---------- ---------- ---------- ---------- ---------- ---------- ----------	494
Gallus_gallus	---------- ---------- ---------- ---------- ---------- ---------- ----------	508
Hominidae	CNNTGGNNNN NNNTGGNGGN NNTCNCCNNT NNNNNNNNNG ATNNTCCCTT TCNTNANCCC NATNNTAATN	7236
Metatheria	---------- ---------- ---------- ---------- ---------- ---------- ----------	569
Rodentia	TTTCTAACCA GACTTTAATC AGTTGCTATT ACAGGATTAC CAGCCCCTAG CTCTGAAGCC AGAGG----C	4817
Monodelphis	---------- ---------- ---------- ---------- ---------- ---------- ----------	857
Consensus	---------- ---------- ---------- ---------- ---------- ---------- ----------	

Conservation
100%
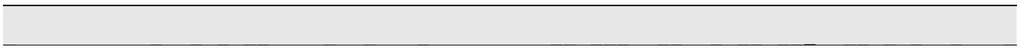
0%

11,000 11,020 11,040 11,060

Dipodomys_ordii	----------	----------	----------	----------	----------	----------	----------	597
Laurasiatheria	----------	----------	----------	----------	----------	----------	----------	361
Eutheria	----------	----------	----------	----------	----------	----------	----------	452
Tarsius_syrichta	----------	----------	----------	----------	----------	----------	----------	487
Muridae	TAAGACCAGT	GGGGAA----	----------	----------	----------	----------	----------	5324
Gasterosteus_aculeatus	----------	----------	----------	----------	----------	----------	----------	2399
Felis_silvestris	----------	----------	----------	----------	----------	----------	----------	505
Rattus	----------	----------	----------	----------	----------	----------	----------	589
Papio_hamadryas	----------	----------	----------	----------	----------	----------	----------	7392
Pan_troglodytes	----------	----------	----------	----------	----------	----------	----------	4138
Ailuropoda_melanoleuca	----------	----------	----------	----------	----------	----------	----------	462
Cavia_porcellus	----------	----------	----------	----------	----------	----------	----------	527
Ictidomys_tridecemlineatus	----------	----------	----------	----------	----------	----------	----------	530
Canis	----------	----------	----------	----------	----------	----------	----------	479
Felis_catus	----------	----------	----------	----------	----------	----------	----------	423
Ochotona_princeps	----------	----------	----------	----------	----------	----------	----------	515
Cercopithecidae	----------	----------	----------	----------	----------	----------	----------	2243
Carnivora	----------	----------	----------	----------	----------	----------	----------	421
Mus	----------	----------	----------	----------	----------	----------	----------	2488
Mus_musculus	----------	----------	----------	----------	----------	----------	----------	478
Rattus_norvegicus	----------	----------	----------	----------	----------	----------	----------	420
Tursiops_truncatus	----------	----------	----------	----------	----------	----------	----------	518
Procavia_capensis	----------	----------	----------	----------	----------	----------	----------	442
Primates	----------	----------	----------	----------	----------	----------	----------	671
Echinops_telfairi	----------	----------	----------	----------	----------	----------	----------	473
Sorex_araneus	----------	----------	----------	----------	----------	----------	----------	494
Canidae	----------	----------	----------	----------	----------	----------	----------	550
Otolemur_garnettii	----------	----------	----------	----------	----------	----------	----------	413
Gallus	----------	----------	----------	----------	----------	----------	----------	633
Callithrix_jacchus	----------	----------	----------	----------	----------	----------	----------	495
Crocodylus_porosus	----------	----------	----------	----------	----------	----------	----------	527
Erinaceus_europaeus	----------	----------	----------	----------	----------	----------	----------	420
rnd-6_family-7426-LTR-ERV1	----------	----------	----------	----------	----------	----------	----------	1692
Esox_lucius	----------	----------	----------	----------	----------	----------	----------	532
Salmo_salar	----------	----------	----------	----------	----------	----------	----------	801
Didelphidae	----------	----------	----------	----------	----------	----------	----------	642
Latimeria_chalumnae	----------	----------	----------	----------	----------	----------	----------	1766
Monodelphis_domestica	----------	----------	----------	----------	----------	----------	----------	543
Macropus_eugenii	----------	----------	----------	----------	----------	----------	----------	509
Alligator_mississippiensis	----------	----------	----------	----------	----------	----------	----------	566
Chrysemys_picta_bellii	----------	----------	----------	----------	----------	----------	----------	3578
Danio_rerio	----------	----------	----------	----------	----------	----------	----------	699
Taeniopygia	----------	----------	----------	----------	----------	----------	----------	538
Dasypus_novemcinctus	----------	----------	----------	----------	----------	----------	----------	512
LTR_XT	----------	----------	----------	----------	----------	----------	----------	503
Haplorrhini	----------	----------	----------	----------	----------	----------	----------	8801
Catarrhini	----------	----------	----------	----------	----------	----------	----------	7847
Anolis_carolinensis	----------	----------	----------	----------	----------	----------	----------	1184
Murine_leukemia_virus	----------	----------	----------	----------	----------	----------	----------	7615
Phascolarctos_cinereus	----------	----------	----------	----------	----------	----------	----------	7426
Estrildidae	----------	----------	----------	----------	----------	----------	----------	482
Xenopus_Silurana_tropicalis	----------	----------	----------	----------	----------	----------	----------	812
I_XT	----------	----------	----------	----------	----------	----------	----------	7509
Takifugu_rubripes	----------	----------	----------	----------	----------	----------	----------	707
Bos_taurus	----------	----------	----------	----------	----------	----------	----------	533
Homo_sapiens	----------	----------	----------	----------	----------	----------	----------	450
Microcebus_murinus	----------	----------	----------	----------	----------	----------	----------	428
Myotis_lucifugus	----------	----------	----------	----------	----------	----------	----------	432
Equus_caballus	----------	----------	----------	----------	----------	----------	----------	450
Tupaia_belangeri	----------	----------	----------	----------	----------	----------	----------	498
Loxodonta_africana	----------	----------	----------	----------	----------	----------	----------	460
Murinae	----------	----------	----------	----------	----------	----------	----------	489
Canis_lupus_familiaris	----------	----------	----------	----------	----------	----------	----------	536
Sus_scrofa	----------	----------	----------	----------	----------	----------	----------	454
Choloepus_hoffmanni	----------	----------	----------	----------	----------	----------	----------	444
Vicugna_pacos	----------	----------	----------	----------	----------	----------	----------	474
Gavialis_gangeticus	----------	----------	----------	----------	----------	----------	----------	470
Mammalia	----------	----------	----------	----------	----------	----------	----------	1337
Passeroidea	----------	----------	----------	----------	----------	----------	----------	641
Takifugu	----------	----------	----------	----------	----------	----------	----------	629
Pteropus_vampyrus	----------	----------	----------	----------	----------	----------	----------	472
Ornithorhynchus	----------	----------	----------	----------	----------	----------	----------	2785
Oryctolagus_cuniculus	----------	----------	----------	----------	----------	----------	----------	494
Gallus_gallus	----------	----------	----------	----------	----------	----------	----------	508
Hominidae	NTNTGNNTNN	TANTNNNNNT	NGCCCCNNNN	NTNNTNNAGT	TNNTCNNNNA	NCGNATNNNN	GAANTCNCCN	7306
Metatheria	----------	----------	----------	----------	----------	----------	----------	569
Rodentia	CTGCAACTCC	CGATTATACC	CTGATGGTGA	AGATCGAACT	TGCCCCAAGG	ACTTCGACCC	ACAAGCAGCA	4887
Monodelphis	----------	----------	----------	----------	----------	----------	----------	857
Consensus	----------	----------	----------	----------	----------	----------	----------	

Conservation 100% 0%

11,080 11,100 11,120

Dipodomys_ordii	----------	----------	----------	----------	----------	----------	----------	597
Laurasiatheria	----------	----------	----------	----------	----------	----------	----------	361
Eutheria	----------	----------	----------	----------	----------	----------	----------	452
Tarsius_syrichta	----------	----------	----------	----------	----------	----------	----------	487
Muridae	----------	----------	----------	----------	----------	----------	----------	5324
Gasterosteus_aculeatus	----------	----------	----------	----------	----------	----------	----------	2399
Felis_silvestris	----------	----------	----------	----------	----------	----------	----------	505
Rattus	----------	----------	----------	----------	----------	----------	----------	589
Papio_hamadryas	----------	----------	----------	----------	----------	----------	----------	7392
Pan_troglodytes	----------	----------	----------	----------	----------	----------	----------	4138
Ailuropoda_melanoleuca	----------	----------	----------	----------	----------	----------	----------	462
Cavia_porcellus	----------	----------	----------	----------	----------	----------	----------	527
Ictidomys_tridecemlineatus	----------	----------	----------	----------	----------	----------	----------	530
Canis	----------	----------	----------	----------	----------	----------	----------	479
Felis_catus	----------	----------	----------	----------	----------	----------	----------	423
Ochotona_princeps	----------	----------	----------	----------	----------	----------	----------	515
Cercopithecidae	----------	----------	----------	----------	----------	----------	----------	2243
Carnivora	----------	----------	----------	----------	----------	----------	----------	421
Mus	----------	----------	----------	----------	----------	----------	----------	2488
Mus_musculus	----------	----------	----------	----------	----------	----------	----------	478
Rattus_norvegicus	----------	----------	----------	----------	----------	----------	----------	420
Tursiops_truncatus	----------	----------	----------	----------	----------	----------	----------	518
Procavia_capensis	----------	----------	----------	----------	----------	----------	----------	442
Primates	----------	----------	----------	----------	----------	----------	----------	671
Echinops_telfairi	----------	----------	----------	----------	----------	----------	----------	473
Sorex_araneus	----------	----------	----------	----------	----------	----------	----------	494
Canidae	----------	----------	----------	----------	----------	----------	----------	550
Otolemur_garnettii	----------	----------	----------	----------	----------	----------	----------	413
Gallus	----------	----------	----------	----------	----------	----------	----------	633
Callithrix_jacchus	----------	----------	----------	----------	----------	----------	----------	495
Crocodylus_porosus	----------	----------	----------	----------	----------	----------	----------	527
Erinaceus_europaeus	----------	----------	----------	----------	----------	----------	----------	420
rnd-6_family-7426-LTR-ERV1	----------	----------	----------	----------	----------	----------	----------	1692
Esox_lucius	----------	----------	----------	----------	----------	----------	----------	532
Salmo_salar	----------	----------	----------	----------	----------	----------	----------	801
Didelphidae	----------	----------	----------	----------	----------	----------	----------	642
Latimeria_chalumnae	----------	----------	----------	----------	----------	----------	----------	1766
Monodelphis_domestica	----------	----------	----------	----------	----------	----------	----------	543
Macropus_eugenii	----------	----------	----------	----------	----------	----------	----------	509
Alligator_mississippiensis	----------	----------	----------	----------	----------	----------	----------	566
Chrysemys_picta_bellii	----------	----------	----------	----------	----------	----------	----------	3578
Danio_rerio	----------	----------	----------	----------	----------	----------	----------	699
Taeniopygia	----------	----------	----------	----------	----------	----------	----------	538
Dasypus_novemcinctus	----------	----------	----------	----------	----------	----------	----------	512
LTR_XT	----------	----------	----------	----------	----------	----------	----------	503
Haplorrhini	----------	----------	----------	----------	----------	----------	----------	8801
Catarrhini	----------	----------	----------	----------	----------	----------	----------	7847
Anolis_carolinensis	----------	----------	----------	----------	----------	----------	----------	1184
Murine_leukemia_virus	----------	----------	----------	----------	----------	----------	----------	7615
Phascolarctos_cinereus	----------	----------	----------	----------	----------	----------	----------	7426
Estrildidae	----------	----------	----------	----------	----------	----------	----------	482
Xenopus_Silurana_tropicalis	----------	----------	----------	----------	----------	----------	----------	812
I_XT	----------	----------	----------	----------	----------	----------	----------	7509
Takifugu_rubripes	----------	----------	----------	----------	----------	----------	----------	707
Bos_taurus	----------	----------	----------	----------	----------	----------	----------	533
Homo_sapiens	----------	----------	----------	----------	----------	----------	----------	450
Microcebus_murinus	----------	----------	----------	----------	----------	----------	----------	428
Myotis_lucifugus	----------	----------	----------	----------	----------	----------	----------	432
Equus_caballus	----------	----------	----------	----------	----------	----------	----------	450
Tupaia_belangeri	----------	----------	----------	----------	----------	----------	----------	498
Loxodonta_africana	----------	----------	----------	----------	----------	----------	----------	460
Murinae	----------	----------	----------	----------	----------	----------	----------	489
Canis_lupus_familiaris	----------	----------	----------	----------	----------	----------	----------	536
Sus_scrofa	----------	----------	----------	----------	----------	----------	----------	454
Choloepus_hoffmanni	----------	----------	----------	----------	----------	----------	----------	444
Vicugna_pacos	----------	----------	----------	----------	----------	----------	----------	474
Gavialis_gangeticus	----------	----------	----------	----------	----------	----------	----------	470
Mammalia	----------	----------	----------	----------	----------	----------	----------	1337
Passeroidea	----------	----------	----------	----------	----------	----------	----------	641
Takifugu	----------	----------	----------	----------	----------	----------	----------	629
Pteropus_vampyrus	----------	----------	----------	----------	----------	----------	----------	472
Ornithorhynchus	----------	----------	----------	----------	----------	----------	----------	2785
Oryctolagus_cuniculus	----------	----------	----------	----------	----------	----------	----------	494
Gallus_gallus	----------	----------	----------	----------	----------	----------	----------	508
Hominidae	GGGTNNCNNT	CAACCANATG	TTACTNCACC	CNTACNNCCN	NCTNCCNACC	TCNGATTTAG	NCCNCNNNCC	7376
Metatheria	----------	----------	----------	----------	----------	----------	----------	569
Rodentia	GGAAGTGGGA	CCTCTACGCC	CACATTCAGC	AGGANGTAGT	CTAATGATAA	CGTTGCCCCC	TTTCCCGGCC	4957
Monodelphis	----------	----------	----------	----------	----------	----------	----------	857
Consensus	----------	----------	----------	----------	----------	----------	----------	

Conservation 100% 0%

11,140 | 11,160 | 11,180 | 11,200

Dipodomys_ordii	----------	----------	----------	----------	----------	----------	----------	597
Laurasiatheria	----------	----------	----------	----------	----------	----------	----------	361
Eutheria	----------	----------	----------	----------	----------	----------	----------	452
Tarsius_syrichta	----------	----------	----------	----------	----------	----------	----------	487
Muridae	----------	----------	----------	----------	----------	----------	----------	5324
Gasterosteus_aculeatus	----------	----------	----------	----------	----------	----------	----------	2399
Felis_silvestris	----------	----------	----------	----------	----------	----------	----------	505
Rattus	----------	----------	----------	----------	----------	----------	----------	589
Papio_hamadryas	----------	----------	----------	----------	----------	----------	----------	7392
Pan_troglodytes	----------	----------	----------	----------	----------	----------	----------	4138
Ailuropoda_melanoleuca	----------	----------	----------	----------	----------	----------	----------	462
Cavia_porcellus	----------	----------	----------	----------	----------	----------	----------	527
Ictidomys_tridecemlineatus	----------	----------	----------	----------	----------	----------	----------	530
Canis	----------	----------	----------	----------	----------	----------	----------	479
Felis_catus	----------	----------	----------	----------	----------	----------	----------	423
Ochotona_princeps	----------	----------	----------	----------	----------	----------	----------	515
Cercopithecidae	----------	----------	----------	----------	----------	----------	----------	2243
Carnivora	----------	----------	----------	----------	----------	----------	----------	421
Mus	----------	----------	----------	----------	----------	----------	----------	2488
Mus_musculus	----------	----------	----------	----------	----------	----------	----------	478
Rattus_norvegicus	----------	----------	----------	----------	----------	----------	----------	420
Tursiops_truncatus	----------	----------	----------	----------	----------	----------	----------	518
Procavia_capensis	----------	----------	----------	----------	----------	----------	----------	442
Primates	----------	----------	----------	----------	----------	----------	----------	671
Echinops_telfairi	----------	----------	----------	----------	----------	----------	----------	473
Sorex_araneus	----------	----------	----------	----------	----------	----------	----------	494
Canidae	----------	----------	----------	----------	----------	----------	----------	550
Otolemur_garnettii	----------	----------	----------	----------	----------	----------	----------	413
Gallus	----------	----------	----------	----------	----------	----------	----------	633
Callithrix_jacchus	----------	----------	----------	----------	----------	----------	----------	495
Crocodylus_porosus	----------	----------	----------	----------	----------	----------	----------	527
Erinaceus_europaeus	----------	----------	----------	----------	----------	----------	----------	420
rnd-6_family-7426-LTR-ERV1	----------	----------	----------	----------	----------	----------	----------	1692
Esox_lucius	----------	----------	----------	----------	----------	----------	----------	532
Salmo_salar	----------	----------	----------	----------	----------	----------	----------	801
Didelphidae	----------	----------	----------	----------	----------	----------	----------	642
Latimeria_chalumnae	----------	----------	----------	----------	----------	----------	----------	1766
Monodelphis_domestica	----------	----------	----------	----------	----------	----------	----------	543
Macropus_eugenii	----------	----------	----------	----------	----------	----------	----------	509
Alligator_mississippiensis	----------	----------	----------	----------	----------	----------	----------	566
Chrysemys_picta_bellii	----------	----------	----------	----------	----------	----------	----------	3578
Danio_rerio	----------	----------	----------	----------	----------	----------	----------	699
Taeniopygia	----------	----------	----------	----------	----------	----------	----------	538
Dasypus_novemcinctus	----------	----------	----------	----------	----------	----------	----------	512
LTR_XT	----------	----------	----------	----------	----------	----------	----------	503
Haplorrhini	----------	----------	----------	----------	----------	----------	----------	8801
Catarrhini	----------	----------	----------	----------	----------	----------	----------	7847
Anolis_carolinensis	----------	----------	----------	----------	----------	----------	----------	1184
Murine_leukemia_virus	----------	----------	----------	----------	----------	----------	----------	7615
Phascolarctos_cinereus	----------	----------	----------	----------	----------	----------	----------	7426
Estrildidae	----------	----------	----------	----------	----------	----------	----------	482
Xenopus_Silurana_tropicalis	----------	----------	----------	----------	----------	----------	----------	812
I_XT	----------	----------	----------	----------	----------	----------	----------	7509
Takifugu_rubripes	----------	----------	----------	----------	----------	----------	----------	707
Bos_taurus	----------	----------	----------	----------	----------	----------	----------	533
Homo_sapiens	----------	----------	----------	----------	----------	----------	----------	450
Microcebus_murinus	----------	----------	----------	----------	----------	----------	----------	428
Myotis_lucifugus	----------	----------	----------	----------	----------	----------	----------	432
Equus_caballus	----------	----------	----------	----------	----------	----------	----------	450
Tupaia_belangeri	----------	----------	----------	----------	----------	----------	----------	498
Loxodonta_africana	----------	----------	----------	----------	----------	----------	----------	460
Murinae	----------	----------	----------	----------	----------	----------	----------	489
Canis_lupus_familiaris	----------	----------	----------	----------	----------	----------	----------	536
Sus_scrofa	----------	----------	----------	----------	----------	----------	----------	454
Choloepus_hoffmanni	----------	----------	----------	----------	----------	----------	----------	444
Vicugna_pacos	----------	----------	----------	----------	----------	----------	----------	474
Gavialis_gangeticus	----------	----------	----------	----------	----------	----------	----------	470
Mammalia	----------	----------	----------	----------	----------	----------	----------	1337
Passeroidea	----------	----------	----------	----------	----------	----------	----------	641
Takifugu	----------	----------	----------	----------	----------	----------	----------	629
Pteropus_vampyrus	----------	----------	----------	----------	----------	----------	----------	472
Ornithorhynchus	----------	----------	----------	----------	----------	----------	----------	2785
Oryctolagus_cuniculus	----------	----------	----------	----------	----------	----------	----------	494
Gallus_gallus	----------	----------	----------	----------	----------	----------	----------	508
Hominidae	CNACGACGCC	CNNNNNCAGC	AGGAAGNAGC	CAGANGATTN	CGTCGCCCCT	TTTNNNTAAC	CANNANGAGG	7446
Metatheria	----------	----------	----------	----------	----------	----------	----------	569
Rodentia	CCTGACTGTT	TATCAATAGT	AAACAAAAGG	GGGNGGGGGG	----------	----------	----------	4997
Monodelphis	----------	----------	----------	----------	----------	----------	----------	857
Consensus	----------	----------	----------	----------	----------	----------	----------	

Conservation 100% 0%

Dipodomys_ordii	------	597
Laurasiatheria	------	361
Eutheria	------	452
Tarsius_syrichta	------	487
Muridae	------	5324
Gasterosteus_aculeatus	------	2399
Felis_silvestris	------	505
Rattus	------	589
Papio_hamadryas	------	7392
Pan_troglodytes	------	4138
Ailuropoda_melanoleuca	------	462
Cavia_porcellus	------	527
Ictidomys_tridecemlineatus	------	530
Canis	------	479
Felis_catus	------	423
Ochotona_princeps	------	515
Cercopithecidae	------	2243
Carnivora	------	421
Mus	------	2488
Mus_musculus	------	478
Rattus_norvegicus	------	420
Tursiops_truncatus	------	518
Procavia_capensis	------	442
Primates	------	671
Echinops_telfairi	------	473
Sorex_araneus	------	494
Canidae	------	550
Otolemur_garnettii	------	413
Gallus	------	633
Callithrix_jacchus	------	495
Crocodylus_porosus	------	527
Erinaceus_europaeus	------	420
rnd-6_family-7426-LTR-ERV1	------	1692
Esox_lucius	------	532
Salmo_salar	------	801
Didelphidae	------	642
Latimeria_chalumnae	------	1766
Monodelphis_domestica	------	543
Macropus_eugenii	------	509
Alligator_mississippiensis	------	566
Chrysemys_picta_bellii	------	3578
Danio_rerio	------	699
Taeniopygia	------	538
Dasypus_novemcinctus	------	512
LTR_XT	------	503
Haplorrhini	------	8801
Catarrhini	------	7847
Anolis_carolinensis	------	1184
Murine_leukemia_virus	------	7615
Phascolarctos_cinereus	------	7426
Estrildidae	------	482
Xenopus_Silurana_tropicalis	------	812
I_XT	------	7509
Takifugu_rubripes	------	707
Bos_taurus	------	533
Homo_sapiens	------	450
Microcebus_murinus	------	428
Myotis_lucifugus	------	432
Equus_caballus	------	450
Tupaia_belangeri	------	498
Loxodonta_africana	------	460
Murinae	------	489
Canis_lupus_familiaris	------	536
Sus_scrofa	------	454
Choloepus_hoffmanni	------	444
Vicugna_pacos	------	474
Gavialis_gangeticus	------	470
Mammalia	------	1337
Passeroidea	------	641
Takifugu	------	629
Pteropus_vampyrus	------	472
Ornithorhynchus	------	2785
Oryctolagus_cuniculus	------	494
Gallus_gallus	------	508
Hominidae	TNGGAN	7452
Metatheria	------	569
Rodentia	------	4997
Monodelphis	------	857
Consensus	------	
Conservation (100% / 0%)		
