# Supplementary material for: A Deluge of Complex Repeats: The Solanum Genome
Source: PLoS One. 2015 Aug 4;10(8):e0133962. doi: 10.1371/journal.pone.0133962 (PMC4524691; doi:10.1371/journal.pone.0133962)
Supplement: S1 Table — (DOC) [file pone.0133962.s007.doc]

**RepeatModeler identified consensus sequence library**

***Solanum tuberosum***

>rnd-1_family-238#LTR/Gypsy ( RepeatScout Family Size = 393 Final Multiple Alignment Size (possibly truncated) = 100 Localized to 2361 out of 4218 contigs )

TGTAACAGCCCGTATTACGCATATGCTTGTTCTATTCTTGTGATACTTGACATAGCCTTATAGTATAGGTGAGGGACCATGTTTCTTATATGATTAGCGTTGGTTTAAACTAGCTCGGAAGGTGATGTGATGCCTTGCACGTTTCAGCATGTGTACTGCAAGTTATAACTCCGACGGAAGTATTTAAACTCGAGACGGTAAGGTGGTAATTGAGCTTAAAGTTGAAAGTTAAGTGCTAAGTTAAAGAAAAAGGGATTAATATTTAGAAAATGGAATAAAATAAGTGAGCTGCTTGCTTGACTTAACTAAGCTAGTAGGTGACAAGTAGGTGCATCACCTACTTTGACTTATTGACCAGTCCAAAGGACCAAGTAGGTGCATCACCTACTTGAGCTATATGGATCTAGTTAAAAAGGCCAAGGTTTATGGATCAGATTTGGGCCTATGCTAATGGACTCACTTGAGGCCCAAACTAAGTTAAATAAAACGAAAATGAAATTAAAGCCCAGCAGCCAAATCTGCCAAGGCCCAATATATTTAAGCCCAAGTGAAAGCAAGTAGGTGCATCACCTAGTTTGACCTTTTGAGCTGCTTGATGTACATAAGCAGGTGCATCACCTACTTGACCAATTAGTGGCTGAAAATGGACCAAAAAAAGGGAGGTTCTAGAGGATTATTCTTAAAGGTNAAGAAGGCTCNATATAAACCCAATATNGGCTGATCTTTTCACTCATTTTAACACACAAAATTCANAATTAACTCCAGCTCTCTCTCTCTCTTTCTCTCAAGGATTAAAACAGCAGCCTTAAAGTTCCTTCTAGGGTTCTTGAAGAAACCCTCATATCTGCAAACCAAGGTAAGAGAAACACTTATGGATTTAGCTTAATTCTCCCATGGAAGACTAGGAAAACATGTTATTCATGCTCAATTATAAGTACAAGGCAGAAGTTCCCACAAGCTACTGCTCACTTTCCTTAGTTATTAAGATACTTTTCTCTTCTTCTTTGGTTTAAGTGTGAAGGGAAGTTGAAGTTCTAGAGGAATTCAAGTTATAAGGAGAAGTTGCAGAAATTGAGGTAAGGTGACTGCTCCATTTTCTTCCTCCTTATGTATGAGTTTGAGTGAGGAATTATGGGTGTGCTGTCATAAAGAACTCAAGGGGGTGGTGAGTTCAATAATTGGTTGTATGATAAAGAATATTTGAGGCTGTTTTATGTGTATTGTTGTTGGTGCAGCTTGGGTAATCTTGGAAAAGGATATATAATGTATGTATTGGGCAGAGAAGGAGGGTGTGGTGATACACATTATTTTAAGGGCTGTATGGGCTCATTATCCGTCAAAGTTAGCTACTGTTTTACGAGGCTCACGGTGTGATAGCTGCTAATNTTAGTTTATCATGACTTATATTGTAGATTAAAACCTAAAAGGGGAGGCTGAATCGTGATAGTTGTATTGGTCAGAGGATAAGGTATGTAAGGCTATTCGATTCAATATTCTTCGGCATGAAATCTGATACTTGCGATTAATATCAATAAGTGAATTTCCTAAGTTCTATTCCTAGTAAAGGAAACATAGTGGCAGCGTACGATCTCCAAATAGCTAACAATATTTCCCCCTCTCCTTAGTGTATAGAATTACTTCATTATATGTTCACTACTCTATACTTGTGTAATTATCCTCCTATGTGCTGGTATAGAAATGGTATTTGCAAAAGCAAGTTTGGTGAATCCTCCTCTTAGTCAGTTGAAGTCAATTGTGTCATTTAAGCTCACAAGTAATGCATTGATGTTACATAGCTCCTTATGTCATTGTTACTGCCTTGAAGTATTATTTTCATGTCTTCTACTACTGGTCCGTAGTTCCGAATATNAAAAATGATTATGACCACCAAAACAACAATCTCAAAGATTAAAGAATCTAAGTGAAGCAATCTGTATAATTAAGGGTGGCAGCTCAGGGGCGAAAGCCTAGCATGGGCCGATCCCAATTGGTATAGAAGGGTGGCAGCTCAGGGGCGAAAGCCTAGCATGGGCC

>rnd-1_family-389#Unknown ( RepeatScout Family Size = 192 Final Multiple Alignment Size (possibly truncated) = 99 Localized to 2361 out of 4218 contigs )

TGATATAAAGCCGATGGGAAATGGTTCCGGCTAGTGATATGATATAAAGCCGATGGGAAATGGTTCCGGCTAGTGATATGATATAAAGCCGATGGGAAATGGTTCCGGCTAGTGATNTGATATAAAGCCGATGGGAAATGGTTCCGGCTAGTGATNTGATATAAAGCCGATGGGAAATGGTTCCGGCTAGTGATATTGTGCCGATGGGAAATGGTTCCGGCGAGAGATTGATTATTGTGACTTGATGCANTTTGATGCTTGTGNTTGACTTACTTGATANTTGTGATTGATGTACTTGTTACGTGTGATTGANCTACTTGACATTGAGATTGACTTATTTGACTGATTTCTGTGCAGGTTGTAGTTTGAGGAGGTTCGGTTGGGATGAAAGGAGTATTCGTTTTCTAGCTAGTTGCTTTGTTTAGTAGGTTGCTTGCTGGGTACCGTGTTGTTG

>rnd-1_family-426#LTR/Gypsy ( RepeatScout Family Size = 166 Final Multiple Alignment Size (possibly truncated) = 99 Localized to 2361 out of 4218 contigs )

GAGGAGCNNTAATCCTGTCGGAGATGGACGGCACCGAGTGGCCGAAACCGATCAACTCAGACGCCGTCAAGAGATACTACGTCCGACCTGAATTCTCAAGAATGAGATACGTAGGCGGCCTATGTCGGCCTCGGTCGTTCCCTTGTAATCCCTTTTTTAATATTTTATCCCTTTGTANTGAACTACGTTCGACCTGAATTCTCAAGAACGAGATACGTAGGCGGCCTATGTCGGCCTCGGTCGCCCTTTTATTTGAACTACGCNCGACCTGAATTCTCGTCTCGACGAGATACGTAGGCAACCCTCCTCGGGTTCGGTC

>rnd-1_family-239#LTR/Gypsy ( RepeatScout Family Size = 390 Final Multiple Alignment Size (possibly truncated) = 99 Localized to 2361 out of 4218 contigs )

TGTAACGACCCGGAAAACGANAGAGTGAAACTAGAGCCTCACATGTGAGTTTGGAGTCGAGAACTTAATGAAATGATTATATTTGAAACGACCCCAAAAGTGCCCGAAAATGTGCTTCGGAAATCACCTANAACTTGTAATTTANCCATATATCTCAAAATCCGTTNCGTGATTTCGGAAATCCGACTTCATATTCTTGTTCAGGGGGTCAAATTGAGTGGGAAATGGGTCTAACCCGAATTTTAGAGCAACCGTATCAAAATCCGAAATTTCCAAGCGAAGCCTTTTTCGAGGGTCNACTTTGGAGGGTCATATCTCCTAGCACACAAATTATTGGGNGGCCAATAACATATCCATGGAAAGCCCTTTGAGTTAGCTNCCCAACGCACTTCGTTTCACCTCATTCGGAGTTCGGACGAAGAAGTTATGCCCATTTTCGTAAAACCTGTCCGGCAGGAAAGGCAATTTCCAGTGAGCGTNTTTACTGTTCACCCGCCTCATTTTTTTTTCTAAGTGTTGGACCATTTTTTCCAAAGGGCATATGTTATTTCCTATATACCATTAGTTCAATTCCCATCTCTAAAACATTTCCCAAAACACCTCTCCCATACTTAGACACATTTTCTCTCAAGTTCTCTCAAGAACCCTAATCCAAAACCTTCCTCAAGATTGAAGAGACTCT

>rnd-1_family-69#LTR/Gypsy ( RepeatScout Family Size = 909 Final Multiple Alignment Size (possibly truncated) = 99 Localized to 2361 out of 4218 contigs )

GTTCTAACAACATCACTTCCTCGTTTTCTGAGGAAGATAGTGCGGGCGACATTCCAGTTCCCCCTGTATCGAGCCTGCACCAGTTGCTAAGGAGCCTAATCGATGGTGTGTGGATGGCCAGTACCAAATTTACAGGGATGCTTGTATGCTGAACGAGAATGATAAGCCAGCCAGATTGATCACCGAGGAGCACCGGGATCTCAAAGATAGCGTCCACACCGCTCCGATCATTGAGGGGCTATTCCATAAACACAGGTGTAAGTGGATGGAACGACCCCAAGGCACGTACAACGAGGAGATAGTCAAGGAGTTCTACGCCTCTTATGCCGCCACTGTTAGAGGGTCCATCTCTAAGGGGACCNATGGACCGCAGGTCAGCCTCCCTCGACGGACCGTNGGTACCCACCGTGGGTCGACAGTTCAGCTACTGCCCAAAAGTTTTTTAGATTTTTTTCGTTTTGGTTCGTTTCTTGTTCTAAAATTCATCCANGTGTTTTTCTTTGCTTGTTTTGGTACTGACTAAGCTCCCACAATTAATATGGACCCCAACAAAGCTCCGACCTACGCCGCAAAGGGTAANTCAAAATCGGTCGCTTCCTCGTGCGGTTTGGTCATTGCCGAGGNCGCATAGGACACGGACTACATTTCNCCCACCACCGGGACCTCCCCCACTGCTCCNCGCACTACGAGGAACCGAGCCCGGTNGGTGATTGCCGAGTTGGTCACTACCTCCTAGTCTGATGAGGGGGCCACAACGATCGGATCACCGGCTGGCTCTGAGNCCGCATCTGGCTCNGGCTCCGACTCCAATTCTAACGGCATCACCGCCTCGTCTTCTGAGGCNGATAGTGCGGGCGACATTCCAGTGACCCATNGTNTCGAGCCTGCACCAGTNGCTGAGGAGCCTAATCGATGGTGTGTGGAGGGCCAGTNCCAGNTNTACAGGTATGCCCGCATGCTGAACGGGAACGATAAGACAACCNGACTGGTCACCGAGGAGCGCCGGGTTCTCACAGGTAGCCCCTNCACCGCTCCGGTCATTGAGGGGCTATTCCGTAGACACGTGTGTCAGTGGANGGCACGGCCCCNAGACACGTACAACGAGGAGATAGTCAAAGAGTTCTACGCCTCTTATGCCGCCACTGTTAGAGGGTCAATCTCCAAGAGGGCAAAGCCCGCAGCTCAGCCTCCNTCGACGGACCGTAGGTACCCACCGTGGGTCGGCAGTTCAGCTACTGCCCAGTTTTTTTAANTTTTTGTTTTGGTTCGTTTCTTGTTCTAAAATTCTTCCGTGTGTTTTATTGGCTTGTNTTGGTACTAACTAAGCTCCCACATGTACAATGGCCCCTAAGCAAGCCCCCACTTATGCCGCGAAGGGCAAGTCGAAGTCTGTNGCNCCCTCCNTGCGGCTGATCGATGAGGATACGGATGGGGAGTACGTTCCACCTCCCACCAGGACATCCCCCACTGCTCCACGCACTACACGGAATCGANCCCGGCAGGTGANTTCCGACGTGGTCACTGCCTCCCAGTCTGATGAGGGGGCCACACCGATCGGNTCACCGGCTGGCTCTGAGTCCGCATCCGGCTCCGNCTCCGGCTCCAGCTCTAACGGCGCCGCCGCCTCATCCTCCGAGGCCACTAGTGCGGGGGACATTCCAGCGCCACCAAACACCGATCCTGCTCCAGTTGCTGAGGAGCCAAACCGATGGTGTGCGAGTGGCCAGTGGCAGATATNCAGAGACGCTCGCATGCTGACCGAGAAGGAGAAGACGGCTCGATTGGTCACCGAGGAGCACCGGGTACTCACATGNAGCCTTCATACCATGCCCGACATCCATCGGCTCTTCCAGAAACATAAGTGTGAGTGGATGGCCCGTAGCCCAGGGNCCTTCAGCGAGGAGATAGTTANGGAGTTCTACGCTTCCTACGCAGCCACCCTCCGAGGTTCGATCGACAGACGGGCCAAGCCCGCTNCCCANGCTTCACTCACGGACCGTAGGTGCCCACTTTAGGTGGTCACTGCTGATACTGCCCAAATTTTTTTAATTGTTTTTGTTTTGTTTCGTTTCTTGTTCTNAAAANCTTCCGNGTGTGTNATTTGCTTGTCTTGGTACTAACTAAAGCTCCCACATGTACCATGGCCCCCAACAAAGCTCCGACNTACGCCGCGAAGGGGAAGTCAAAATTCGTCGCTCCATCGCGATGCATGATCATTGANGAGGGCATGGAGGACACGGAGTACGTTCCNCCCACCACCAGGACCTCTCCCACTGGTCCNCGCACCACGAGGAACCGAGCACGGTNGGTGATTCCCGACGTCGTCACTGCCTCCCAGTCTGATGAGGGGGNCACACCAATCGGTTCACCGGCTGGCTCTGNGTCCGCNTCTGCCTCCGGCTCCGTCTTTGCATCCGGCTCTAACAACATCACTTCCTCGTTTTCTGAGGAAGATAGTGCGGGCGACATTCCAGTGCCCCCCTGTATCGAGCCTGCACCAGTTGCTGAGGAGTCTAATCGATGGTGTGTGGATGGCCAGTACCAAATTTACAGGGATGCTTGTATGCTTAACGAGAATGATAAGCCAGACAGATTGATCACCGAGGAGCAGCGGGATCTCAAAGATAGCGTCCACACCGCTCCGATCATTGAGGGGCTATTCCATAAACACAGGTTTAAGTGGATGGCACAACCCCAAGGCACGTACAACGAGGAGATAGTCAAGGAGTTCTACGCCTCTTATGCCGCCACTGTTAGAGGGTCCATNTCTACAGAGGGCCGATCCCGCGGCCCAGCGCTCCATCGACGGACCGTAGGTACCCACCGTGGGTCACCAGNCCAGNTACTGCCCGAAATTTTTTTGATTTTTTCGTCTAGGTCCGANTCTTGTTCTAAAATTCTTCCATGNGTTTTATTGNCTTGTTTTGGTACTAACTANTCTTCCACAGGTACCATGGCTCCGAAACAAGCTCNNGCGTACGCCACGAGAGGGCAAGTCNAAATCAGTCGCCCCNTCCCGACGCCTGTTGGATGAGGACACCGATCAGGAGTACGTTCCGCCNACCACCAGAGGCTCCCCTACTGTNCCCCGGACCACTAGGAGCCGGNCCCGGCAGGTGGTTTCTACTAGTGACGAGGTCGNAGGTCACCGTCCCCCAGTCTGAGGAGGGGGCCACACAGNCTGGAGAGTCGCAGTCCGGCTCGAGTCGCAGTCCGGCTCCAGTCCTAGCGGCANTNCCGCTTCTTCATTCGAGGCCGATAGCTCGGGNGACGTCCTGGTACCNCCTAGAGTTGAGCCGGCTCCCAGTGCCACNGAGCCGAACAGATGGTGTGTTGAGGGCCAGTACCAAATNTATGAGGATGGNACNGTTCTGAACGAGCACCGGAAGATGGCCAGGACCGTTACTGAGGAGCGTNGGGTNCTNACAGGNAGCCTCCACACCGCGCCCGCTATTGAGGAGCTATTCAGGAGGCACAAATGTGAGTGGATGGCACGGGGCCCGGGGAACTNTAGCGAGGAGATGACTAGAGAGTTNTATGCCTCTTACGCCGCCACCGTCCGNAACTCCATCTCCAAACGGGCCAAGCCCGTAGCTCAGCCTCCACTTCAGGCGACTNTAGGTACCCACCGTGGCTCGGTAGTTCGGCTACTGCCCAATTTTTTTTNTNAAAAAAATTNTTTTNGTTTTGTTCATTTCTTGTTCTTAAATTCTTCCATGTGTTTTATTGGCTTGTATTGGTACTAACTAAGCTCCCACAGGTACCATGGCCCTTNAAGCAACCCCCAACTTACGCCGCAAAGGGAAANTCAAAATCCGTCGCNNCCTCCTTCAAACTGATTGATGAGGACGAGGATGTGGAGTACATTCCGCCTCCACCAGNGACATCCCCCATTGCTCCACGCACTACACGGAATCGAAGCCGGTAGGTGAGAATNNCGNNCGACGTAGTCGCTGCCCACTAGTCTAATGAGGGGGTCACAACAATCGGCTCGNNNCACCGACTGGTTCTGAGTCAGCTTCTGGCTTCGGCTCCGACTCCGGCTCCGGCTCCGGCTCCAGCTCACAGCGGTAGCACCGCCTCGTCNNACGAGGCTGNTAGTGCGGGGGACACCCCAGTGCCACCCANTACCGAGCCTCCAGCGGTTGCTACGGAGCCAAATAGGTGGTGTGTGGAGGGCCAATNCNAGATATACACGGATGCTCGCATGNTGAATGAGCAAGANAAGATAGCCCGATTAGTCACCGAGGAGCGTCGGGTTCTCACAGGTAGCCTCCACACCGCTCCAGTCATTCACGANCTTTTCCGGAGGCACAGGTGCGAGTGGATGGCNCGTAGCCCAGGGTCNTACAGCGAGGAGATCGTGCGGGAGTTCTACGCTTCCTACGCGGCCACCCTCACGGGGTCCATCTCTAAGCGGGCCAAGCCCGCCGCTCAACCTCCACTCGA

>rnd-1_family-123#LTR/Gypsy ( RepeatScout Family Size = 651 Final Multiple Alignment Size (possibly truncated) = 99 Localized to 2361 out of 4218 contigs )

ATTNAAGAGGTAGTNAGACTNCATGGGGTTCCGGTCTCTATTATTTCAGATAGAGGTGCGCAATTTACTGCACANTTCTGGAAGTCNTTTCAGAAAGGTTTGGGTNCAAAGGTGAACTTAAGTACNGCCTTTCATCCTCAGACAGATGGTCAAGCAGAGCGCACNATTCAGACNTTAGAGGATATGTTGAGGGCTTGTGTGATTAGACTTCAAGGGTAGTTGGGATGATCACCTACCTCTCATTGAGTTCGCTTACAACAATAGTTACCACTCTAGCATCCAAATGGCTCCTTATGAAGCTCTNTATGGGAGAAGATGCAGATCTCCNATTGGATGGTTCGAAGTNGGTGAAGCNGGGTTGATAGGACCAGACTTAGTTCATCAAGCTATGGAAAAGTNAAAGTGATTCAAGAGAGGTTGAAAACGGCNCAGAGTCGNCAGAAATCCTACGCNGATGTTAGGAGAAGGGANTTAGAGTTCGAAGTAGATGATTGGGTNTNCTTGAAAGTNTCACCCATGAAGGGTGTTATGAGGTTTGGTAAGAAGGGGAAGCTTAGTCCCCGGTATATTGGNCCTTACAGGATTATCCGAAGGATTGGCAANGTAGCTTATGAGTTAGAGCTACCNCAAGAGCTAGCAGCGGTTCATCCGGTGTTTCACGTCTCNATGTTGAAGAAGTGCGTGGGCGATCCTTCTCGTATCGTNCCNACTGAAGATGTTGGGATCAAGGATAGCTTATCTTATGAGGAGATTCCCGTTCAGATTCTAGATCGNCAAGTTCGCAAGTTGAGGACNAAAGAAGTAGCNTCAGTCAAAGTNCTNTGGAGGAACCAATTCGTNGAGGAAGCTACTTGGGAAGCTGAGGAGGATATGAAGAAGAGATATCCNCATCTCTTTCAGNCCGAAGAGAAAGATGAAAATGTTGAGTTGAGGCGTTAACAAGTAAGTATAAGTCTATATGTTTAATGTGATTAATAGTAATACTAACTTAGGCTTGAACTTGTTTGGGCTGGATAACCATGCTAACATTCGAGGACGAATGTTTTAAGGGGGGAAGGATGTAACAGCCCGTATTATGCATATGCTTGTTCTATTCTTGTGATACTTGACATAGCCTTATAGTATAGGTGAGGGACCATGTTTCTTATATGATTAGCGTTGGTTTAAACTAGCTCGGAAGGTGATGTGATGCCTTGCACGTTTCAGCATGTGTACTGCAAGTTATAACTCCAACGGAAGTATTTAAACTCGAGACGGTAAGGTGGTAATTGAGCTTAAAGTTGAAAGTTAAGTGCTAAGTTAAAGAAAAAGAGATTAATATTTAGAAAATGGAATAAAATAAGTGAGCTGCTTGCTTGACTTAACTAAGCTAGTAGGTGACAAGTAGGTGCATCACCTACTTTGACTTATTGACCAGTCCAAAGGACCAAGTAGGTGCATCACCTACTTGAGCTATATGGATCTAGTTAAAAAGGCCAAGGTTTATGGATCAGATTTGGGCCTATGCTAATGGACTCACTTGAGGCCCAAACTAAGTTAAATAAAACGAAAATGAAATTAAAGCCCAGCAGCCAAATCTGCCAAGGCCCAATATATTTAAGCCCAAGTGAAAGCAAGTAGGTGCATCACCTAGTTTGACCTTTTGAGCTGCTTGATGTACATAAGCAGGTGCATCACCTACTTGACCAATTAGTGGCTGAAAATGGACCAAAAAAAGGGAGGTTCTAGAGGATTATTCTTNNATGGTCAAGAAGGCTCCATATAAACCCAATATGAGCTGATCTTTTCACTCATTTTAACACACAANATTCANAATTCACTCCAGCTCTCTCTCTCTCTCTCTTTCTCTTAA

>rnd-1_family-441#LTR/Gypsy ( RepeatScout Family Size = 158 Final Multiple Alignment Size (possibly truncated) = 99 Localized to 2361 out of 4218 contigs )

AAATGGAATAAAATAAGTGAGNTGCTTGCTTGACTTAACTAAGCTAGTAGGTGACAAGTAGGTGCATCACCTACTTTGACTTATTGACCAGCCCAAAGGACCAAGTAGGTGCATCACCTACTNAGACTCTTTGACCANGTTAANGGGCCAAGTAGGCGCATCACCTACTTGCATTAATCTGGCCCAAAGGACCAAGTAG

>rnd-1_family-110#LTR/Gypsy ( RepeatScout Family Size = 701 Final Multiple Alignment Size (possibly truncated) = 99 Localized to 2361 out of 4218 contigs )

TGAATGAATGTTGGGTTTGAGATGGATGCTCATCCGTGAGTTGAGGCGGGGTATCTAGTAGCAATCTCTTATCCCATAATAATGAACTAANGTAATGAATGTCAAGTACTCCGTGGGGAATAATGCTTAGCACCGAGTGGATATGAATATGAGATGGANGCTCTCNCGTTAGTTGAGGCGGGGTTTCTAGTAGCAATCTCTTGAGATGGANGCCCTCACGTTAGTTGAGGCGGGGTNTCTAGTAGCAATCTCCTTATCCCATAACTATGTGCCCACATAGGTTATTAGCTAGTGGATCCACCTAAGCTAAAAAGTAATGGTCCTACCTTAGGCAAGTAGGACACCCCTTATTCGGTGTGGGTGACACCGGATTCCATGTTAAGCTCACATGGTCTATGTCGGTTAAGGCTTATCCCCACATAAGAATGTTAATGAACTATGGCTTCTCAAGAATGCNCTACTTAGTGTGGGTGGTGGTATGGGACGTCATCCATGCATTGCACAAGTAGGCTTTGAGAGNGGTTATGGTGTGTTCCTTTATAATGTTAAATGAAATGAATGTGCTCTTATGATGANGCTAATGAAGAATGTTGATTTAATGTCTAATGAATGAAGGTGCTCTTAATGAAGCTAATGAAGNATGTTGACTTANATGCTTAAATGTGATGAT

>rnd-1_family-11#LTR/Gypsy ( RepeatScout Family Size = 1497 Final Multiple Alignment Size (possibly truncated) = 99 Localized to 2361 out of 4218 contigs )

AACCTTGAATTCATAATTCAAATTCAAGGTAGAGTTAAGAGTTAAGTCTTGAGAGTTCTTTCGAACATTTTGAGAAAGTCCCTTTGAGTCCTTTTGAGGAGTCTTCTACAACTTCTAATAACTTGTTTCAAGACTCGAGCAAGTGAGTATGAGAATGAGGAGAATGTATTCATGAGTCTACTTTATCATCACGAGATCCTTCATATCATGAACCATAACTCTTGAATTCATAATTCACATTCAAGAGAGAGTTAAGAGTAGAGTTCAAGAAAGTCTTTGAGTTCAATTGTGAATCCTTTGAGATCAACTATCGATTTGAGCTAAGTTTTGAGGAAGTAAGTATGAGAATGAGAAGAGTCGTATACATGAGTTCCATATTGTTATGTAGACCCTCGAGTCGAGTCGTTCATGCCCATAAATTCCGCATGAACTCCATAAGTTGAGTATCTTTGAGAGGAGTAGTATCTTCAAGTTCTAAGTCTTGAGTATTGAGTTCCTAACCCTTTTGAGATTATATTCCTAATCATGGGACTATTACATGTTACCCATGAAGTCCTTGAGTTGAGTTGTTCATGCCCATAATTCCGCATGAACCCTATAAGTCGAGCAGTCTTGAGATGAGAAGTATCTTTGAGTCCTTGAGTTGAGTAGTTCATGCCCATAATTCCGCATGAACCCTCTGAGTCGAGCATTCTTGAAATGAGTAGTATCATTGAAGTTCTTGAGTTCCGAGTATTGAGTTCTATCTATGGTTATTGAAAACCTTGCATTGAGTCGTTCACGTCCATAATTCGGCATGAACCATATTTTAAGAAGTCTTTTACAAACGTTTTAACTTTGTTTTAAGACTTAAGCTTTGAGTTGAGTAAAGAGTAAGAGTNAAGTTCTTTTTCAAAGAT

>rnd-1_family-41#LTR/Gypsy ( RepeatScout Family Size = 1139 Final Multiple Alignment Size (possibly truncated) = 99 Localized to 2361 out of 4218 contigs )

TTAAGCAAAGAGTAAAGAGTNGAGTTCATTTCTTCAAAAGTTATACGGGAACTAAGTATTCCCAAGAGTTTAAAATGTTTTCACATTTGAGCAAGAAAGGGAAACACCGATTTCCAAGAGAGCTTTTAAGCTAAGTTTTGAGTAATTATCTCAAACCAAAGAAAGAAGTTTTGTTTTAAAACATATGAGCTAAGTATATTTTGGGAGTAGTATTGAGCACCGATATGGGGACGCGAGTTCATANTAACTCAAGTCTCCATAAACCATGTAGCCATCATGGGTAGTAAAGGATCATACTTTTTAGATGACTCCTTAAGTGCTTTTTAGCATAGACTAGTGGATCCACTTAGTTAAGGCGTTCTATATGACGGCAAAGTATAGGACAGTTCTGGCAGCGTGGGCAAGACGTTGTATCACCACTTAGGCTCATAGTGGTGGTTGTCGGTTAGAGAAACTCCCACAGAAACTATATTACTTTATTATATGAGTAAAGTTGAGTTTGTTATTGCATTTCTTTAACGAACTAAGTTGCTTTCTACTGTTTTAAAAGCTTTCTATATATTGCATGTGTTTTATTGCTTTATATTGAGTTAAGTTATTCATGAGTTGAGTAAAGCCAAGGTAAGTGTTCCTTTCAGATTCCTTTCAAGCTTATGTTATGTTTAGCATTCCAACTCGCATACTCGTACATTCAATGTACTGATGCCAGTTGGCCTGCATCGTNTTATGATGCAGACGCAGGTAACCAGGATCAGCATCCAGCGCCTCGTTGATCCAGTTGAGCACTCAGAGTCAGTTGGTGAGCCTCCTTGCTTTCCGGAGGACTCCTTTTTACATTGCTTTCTAGTTTTCAGTTGTTAGGATGATCGGGGGTCTTGTCCCGACATCCCTCTTTGTTTTAGAGGCTTCATAGACAGNTAGNTNTAGTTCTTTAGTCTTATTCATTTCAGTTGTATACGTTTAAGACTTGAGTT

>rnd-1_family-16#LTR/Gypsy ( RepeatScout Family Size = 1425 Final Multiple Alignment Size (possibly truncated) = 99 Localized to 2361 out of 4218 contigs )

TTATGAATGATGATGATATATGTGAAAGGTTATTCTCACATACACACACACTCATGAATGAAGAATGAATGAAGTTTCTATGATTATGTTAATGATGATGTGTTGATTGAAAGGCTATTCTCAATTGTACNCTAATGAATGATAATGACATGTTGTGAAAGGACTTTCTCACAATATGGGGATTCTTAGNTGAAAGGCTATTCTCATCTTTGAATCTATGAGCTATCCAATGAATGAATGTTGGGTTTGAGATGGATGCTCATCCGTGAGTTGAGGCGGGGTATCTAGTAGCAATCTCTTATCCCATAATAATGAACTAAAGTAATGAATGTTAAGTACTCCGTGGGGAATAATGCTTAGCACCGAGTGGATATTGATATGAGATGGAAGCTCTCCCGTNAGTTGAGGCGGGGTTTCTAGNAGCAATCTCTTGAGATGGANGCCCTCCCGTGAGTTGAGGCGGGGTNTCTAGTAGCAATCTCCTTATCCCATAACTATGTGCCCACATAGGTTATTAGCTAGTGGATCCACCTAAGCTAAAAGTAATGGTCCTACCTTAGGCAAGTAGGACANCCCTTATTCGGTGTGGGTAACACCGGGATTCCATTATTAGCTCACATGGTCTATGTCGGTTAAGGCTTATCCCCACAATGAATGTTAATGAACTATGGCTTCTCAAGAATGCNCTACTTAGTGTGGGTGGTGGTATGGGACGCCATCCATGCATTGCACGAGTAGGCTTTGAGAGTGGTTATGGTGTGTTCCTTTAT

>rnd-1_family-6#LTR/Gypsy ( RepeatScout Family Size = 1646 Final Multiple Alignment Size (possibly truncated) = 99 Localized to 2361 out of 4218 contigs )

AATAGAGCCCAATCTTCTTCAGTTGCTCCACCAGACAGAGCTGCACCTAGAGGAGCTACTTCNGGTACTGGCGGAGGAGCAAACCGCCTNTATGCTATCACTAGTCGCCAAGAGCAAGAGGATTCTCCAGATGTTGTCACTGGTATGATCCAAGTCTTTACTTTTGATGTTTATGCTTTGCTAGACCCAGGAGCGAGTTTATCTTTTGTAACTCCTTATGTTGCGATGAATTTTGATGTTCTTCCTGAGAAACTTNGTGAACCCTTCNGTGTTTCTACACCTGTTGGTGAGTCTATTCTAGCAGAGGAGTCTATCGTGATTGTNCCGTTTCCGTCAATCACAAGAGCACCATGGCTGATTTAGTTGAGTTAGACATGGTAGATTTTGATGTCATTCTNGGTATGGACTGGCTTCATGCCTGTTATGCCTCAGTTGATTGTAGAACTCGAGTTGTCAAGTTCCAGTTTCCNAATGAGCCAGTCTTAGAGTGGAAGAGTAGTTCAGCAGTGCCTAAGGGTCGTTTCATTTCGTACCTTAAGGCGAGAAAGTTAGTTTCCAAGGGGTGTGTCTATCACTTAGTCCGAGTTAATGACTCTAGTGTTGAGATACCTCCTATTCAGTCAGTTCCAGTAGTAAAAGAGTTTCCAGAAGTCTTTCCAGATGATCTTCCCGGAGTCCCTCCTGAGAGAGAAATAGACTTCGGTATAGATATTCTTCCNGATACTCGTCCTATCTCTATTCCGCCATATAGAATGGCNCCAGCAGAGTTGAAAGAGTTNAAAGAGCAGTTGAAAGATCTCCTNGATAAGGGTTTTATTCGACCAAGTGTCTCACCTTGGGGCGCTCCGGTCTTATTTGTGAGGAAGAAAGATGGTTCCCTTAGGATGTGTATAGATTACCGTCAGTTGAACAAGGTTACCATCAAGAATAAGTATCCTCTTCCGAGAATTGATGATCTTTTCGATCAACTTCAGGGTGCCACTTGTTTCTCTAAGATAGACCTCAGATCNGGCTACCATCAGTTGAGAGTAAGGGAATGTGATATTCCAAAGACAGCNTTCAGGACCCGTTATGGTCATTATGAGTTTCTGGTCATGTCNTTCGGTTTGACCAATGCGCCTGCAGCGTTCATGGACCTTATGAATAGAGTATTCAAGCCTTATTTAGATATGTTTGTTATCGTNTTCATTGATGACATNCTAATCTATTCGAGGAATGAGGAAGATCATGCTAGTCATCTCAGAATAGTTCTCCAAACTCTNAAGGATAGAGAGTTGTATGCTAAGTTCTCTAAGTGTGAGTTTTGGCTTGAGTCTGTGGCATTCTTAGGCCACATTGTNTCCGGTGANGGAATTAGAGTTGATACTCAGAAAATNGAGGCAGTGCAGAATTGGCCTAGACCCACATCTCCAACTGATATTAGGAGTTTCTTGGGNTTGGCTGGCTATTATAGAAGGTTCGTAGAGGGGTTCTCATCTATTTCGTCCCCTTTGACCAAGTTGACTCAGAAAACAGTGAAGTTTCAATGGTCTGAAGCTTGTGAGAAAAGCTTTCAGGAATTGAAAAAGAGGTTGACTACTGCCCCAGTNTTGACCTTACCAGAAGGTACGCAAGGTTTTGTNGTGTATTGTGATGCGTCTAGAGTTGGTTTGGGTTGTGTGTTAATGCAGAATGGCAAGGTTATAGCTTATGCCTCCAGACAGTTGAAGGTTCACGAGAAGAATTACCCAACCCATGACCTAGAGTTGGCTGCCGTAGTATTCGCTTTGAAGATATGGCGTCATTATCTNTATGGTGTTCATGTNGATGTGTTCACCGATCACAAGAGTCTTCAGTATGTGTTCANTCAGAAAGAGCTTAATCTCAGACAAAGGAGGTGGTTAGAGTTACTCAAGGATTATGACATGAGTATTCTTTATCACCCAGGTAAGGCTAATGTTGTTGCTGATGCCTTGAGCAGGTTGTCTATGGGTAGTACCGCCCATGTTGAGGAAGAAAAGAGAGAGTTAGCGAAAGATGTGCACAGACTTGCACGNTTAGGAGTCCGACTAATGGATTCCACAGAAGGAGGAGTAGTGGTGATGAATGGGGCTGAATCATCATTAGTGTCAGAAGTGAAAGAGAAGCAAGACCAAGATCCTATTTTGCTTGAATTGAAGGCAAATGTTCATAAGCAAAAAGTANTGGCTTTTGAACAAGGGGGAGATGGTGTATTGAGGTATCAAGGTAGATTGTGTGTACCAANGGTGGATGAACTCCAAGAGAGGATCATGGAGGAAGCTCATAGCTCCAGATATTCCATCCATCCGGGTTCCACAAAGATGTATCGCGACTTGAGAGAAGTNTATTGGTGGAGTAGTATGAAGAAGGGCATTGCAGAGTTCGTTGCTAAGTGCCCGAATTGCCAACAAGTTAAAGTAGAGCACCAAAGGCCCGGTGGTATGGCTCAGAATATAGAACTTCCGGAATGGAAGTGGGAGATGATCAATATGGATTTCATCACAGGTTTGCCAAGGTCTCGCAGGCAGCATGATTCTATTTGGGTGATTGTCGATAGAATGACNAAATCAGCCCACTTTTTGCCGGTAAAGACTACCCATTCAGCAGAGGATTATGCCAAGTTGTATATTCAAGAGGTAGTNAGACTTCATGGAGTTCCGGTNTCCATTATTTCAGATAGAGGTGCGCAATTTACTGCACAGTTTTGGAAGTCNTTCCAGAAAGGCTTGGGTTCAAAGGTGAACTTGAGTACTGCTTTTCATCCTCAGACNGATGGNCAAGCAGAGCGCACTATTCAGACCTTAGAGGATATGTTGAGGGCNTGTGTGATCGATTTCAAAGGTAATTGGGATGATCACCTACCTCTCATTGAGTTTGCTTACAACAATAGTTACCACTCTAGCATCCAAATGGCTCCTTATGAAGCTCTTTATGGGAGAAGATGCAGATCTCCTATTGGATGGTTTGAAGTTGGTGAAGCTGGGTTGATAGGACCAGACTTAGTTCATCAAGCTATGGAGAAGGTGAAAGTNATTCAAGAGAGGTTGAAAACGGCGCAGAGTCGTCAGAAATCCTACACTGATGTTAGGAGAAGGGANTTAGAGTTCGAAGTAGATGATTGGGTNTACTTGAAAGTTTCACCCATGAAGGGTGTTATGAGGTTTGGTAAGAAGGGGAAGCTTAGTCCCCGGTATATTGGNCCTTACAGAATATCCAAGAGGATTGGCAATGTAGCTTATGAGTTGGAGCTACCGCAAGAGTTAGCAGCGGTTCATCCGGTATTTCACATCTCCATGTTGAAGAAGTGCATGGGCGATCCTTCATTNATCATACCAACTGAAGATATTGGGATCAAGGATAGCTTATCTTATGAGGAGATTCCNGTTCAGATTCTAGATCGCCAAGTTCGCAAGTTGAGAACNAAGGAGGTAGCATCAGTCAAGGTCCTTTGGAGGAATCAGTTTGTTGAGGAAGCTACTTGGGAAGCTGAGGAGGATATGAAGAAGAGATATCCACATCTCTTCGAATCCGGAGAAATTCCAGATCAAGGTACTAATCCTCTTCTTGGTACTCTTTAATTTATAAGTTGGCATGTTGTATTTGCATTGCTTGTTGGGTGTTTGAGCTGGATGTTAGATGTTACACCCTTAGCCTACTAAGAGTAATCTCATTCGAGGACGAATGTTCCCAAGGGGGAGATATTGTAACATCTCGCNANTTGAAAGAACTAGAAAGAGCTAGAATTGGAAAGAGTCATTTTTGGAAAGAATGAAAATCTGGAAAATTTGTCAAGTATGTTAAGTTTGAGTTTTNGGTCAACTTCAAACGACCATAACTCCTAGCTCAGGATGAGTTAGGTGTGCTNCAAGATATCGTAGGAAAGATCTTGGAATTATCTTTCCAACGCCGCCGAGTTTGCNCGATTTCGAGTTCGTATGAGTGAGATATGCCCGTTTGAAGTTGGGCTGTCTAGNTAAGGAAAGTCAANCCGGATTTTAGAAGGGTATTTTGGTCTTTTCCTTACCCAATTAATTTAATTCGTTTTTAGTAATTTANTTGGGGTNTAAACTGATTTGGTTCAGTTTACGCNATCGAAAATTACGCTAGGGTTTTGAGAGAAGAGAAAAGAAGAGAA

>rnd-1_family-26#Unknown ( RepeatScout Family Size = 1283 Final Multiple Alignment Size (possibly truncated) = 99 Localized to 2361 out of 4218 contigs )

TTACATATTCAGTACATTTTCTCGTACTGACGTCCCTCGCGGGGGACGCTGCATTTCATGCTGCAGGCACAGGTACTTCAGCTAGTAGACCTCCTCGGTAGGAGCACACGACACTCAGCTACTTTTGGTGAGCTCCAGGTTGATTCGGAGCTTTACTGAGTCCTTGGTAGATTCATTTTGGTATTGTATCGTAGTTAAGGGTAAGGCGGGGTCTGTCCCGACCTACTCTAAGGTTTTCTATCTTTTAGAGGCTTTGTAGACTTATGTATATGGTTCAGTTGCGTCTTAACAAGTTGTGGCCTCAACGGCCAAGTCTTGTATATAAGTTTTGGGTCTACTTATGTTGGTTCGTATCGCTGCATCCTATGTGAACTTGTCACAAGATTGTCATAGTTATATGCATAGTAAGCACAGGTTACATGTTGGTTCTCTCGGGCCTTTAGGGCATCGGGTGCCTGTCCGTCTTAATGGGATTTGAGGCGTGACAAAAGTGGTATCAGAGCAGGTCATCCTAGGGAGTCTACAAAGCCGTGTCTGTGTAGAGTCTTGCTTATGGATGTGTTGTGCACCACATTTATAAACAGGAGGCTACGGGACATGTAGGAGTTGTCTCTTTTCTTTCATTTCTTAGATCGTGCGATAGAACCAATGTTGTAGGCGAATCGTATCTAACTTCTAAATTGTTTTATTATAGCAAAGATGCCGGTTACGAGAAGGAGTAAAAGTACAGGTAAGCGAGTAGATGTGGCTGCCCAAGAAGGGACCAGCCAACCACCACCTAGTCAAGCTGTTCAAGGTGAGGCTCAAGATGAGTCTCTATCACAGACTTCCCCGACCCCTCCATCTCCAGAAGATCTAAGGAGGGAAGCTGCACCTCAAGAACCCCCTATTAATGCTACCGAGCAAGATTTGAGGAATGCGGTCCAATTATTGACTCGTATAGTTGCTGGCCACGGCCAAAGGCAAGAAGGTCCAGTTGCAGGTACTAGTGGTGTAGATAGAGCAGCTGGCACGCGGATACGTGATTTCCTTAATTTGGACCCTCCATCATTCACCGGGTCAGATCCTAATGAAGATCCACAAGACTTTATAGATCAGATCCAACGCACTTTCGATGTTATGCATGTAAGTGGTAAAGAAGCGCTCGAGCTAGCAGCATATAGATTAAAGGGTGTGGCTATATTGTGGTATGAAGCTTGGAAGCAATCCAGGGGAACAGATGCACCTTCAGCTACGTGGAAAGAGTTCAAAAAGGCATTCCTTGACCATTACCTGCCATTGGAGATCCGAGAGGCCCGTGCGGATCAGTTCTTAAATCTCCACCAAGGAAGTATGAGCGTGAGAGAGTACAGTCTCAAATTTAATTCCTTGGCCAGGTATGCTCCTAATGTAGTAGCTACTATGGGGGATAGAGTTCATCGATATGTGGATAGATTAGATTCATATCTGGTTAGAGACTGTACCATTGCTTCGTTGAATAAAGATATGGATATAGCAAGGATGCAAGCTTTTGCGCAAAAGTTGGAGGATCAAAGGCAAAGAAGAAGGACACAAGAGTCAGAAACAGGGCATTCTAAGAGGGCCAGATCCATGGGGCAGTTTACACCATCCCAAGGTGAGTTTAGGCCTCGGTTCTTTAACAGACCGCCTAGGCCATCATCTTCCTACTCTACAGCTAGCGCCCCACCTCGGTTTCAAGGGTCTAGGGGCAATCAGTTTGGGCAAAGAGGTGAAAGCCAAGGTTCACGAACAGCGGGGTACCAAGAGCAGGGAAGTACGAGCCAATCAAGACCTCCTCGACAGTCTTGTAAACAATGTGGAAGGAATCATTTAGGTGCATGTCGGTTTGGGACAAATGTTTGTTTTTGGTGTGGTACACCGGGTCATATGATGAGAAATTGCCCTCATAGGGGTGTGGGTGGTGTGGCACAACCTACTAGATCAGTTGTTGCATCCTCATCATCAGCACCTTCTTTAGGTAGGGGACAGACGCCTACTGGTCGTGGTAGAGGAGNTAGGGGAGCANCTAGTTCTAGCGGAGTTCAGAACCGCNCATATGCTCTCGNGGGTCGACAAGATTNAGAGGCNTCGCCGGATGTNGTCACNGGTACGTTGNCCGTCTTTTNACNTANTGTTTATGCNTTGNTAGATCCGGGGNCTACTCTGTCNTNTGTNACTCCATNTATTGCNGNGAAGTTCGATGTNANNCCAGAATTATTAGTGAAACCCTTCGNNGTNTCTACACCGGTCGGTGAGTCTATTATAGCTAGAAGGGTCTATCGNGATTGCACCGTNNCNGTCNNCGACCGNGNTACCTCGGCNGATCTCGTNGAGTTAGANATGGTAGATTTCGATGTCATTNTGGGTATGGATTGGTTAGCTTCTTGTTATGCCNCGGTCGATTGTNGAACTAGGATTGTTCGNTTCCAGTTTCCAGANGAGCCAGTCCTNGAATGGAAAGGTAGTATCGCAGCGCCTAAGGGTCGGTTTATTTCNTACCTTAAGGCGAGGAAGATGATTTCTAAGGGNTGTATCTATCATCTAGTTCGAGTTAANGATNTAGATGCGGAGCCACCGACTCTTCAGTCNATTCCGGTAGTNAATGAGTTTCCAGANGTATTTCCAGACGATCTTCCCGGNGTCCCTCCNGAANGGGAAATNGACTTCGGTATCGATNTNCTTCCAGATACNCAACCTATNTCTATTCCTCCNTATAGAATGGCTCCGGCNGAGCTAAAGGAGTTGAAGGAGCAGTTGAAAGACCTNCTAGATAAGGGCTTCATCAGGCCTAGTGTTTCNCCATGGGGCGCACCGGTNTTGTTCGTGAGGAAGAAAGATGGCTCNCTNAGAATGTGTATCGACTACCGGCAGTTGAATAAGGTNACNATCAAGAATAAGTATCCCCTCCCGAGGATTGATGACTTGTTCGATCAGCTNCAGGGTGCTAGNCGTTTCTCAAAGATNGACCTNAGATCGGGTTACCATCAGNTGAGAGTCAGGGANAGTGACATTCCGAAGACAGCTTTCAGAACNCGGTATGGTCATTNTGAGTTTTTGGTCATGTCNTTCGGNCTAACNAATGCNCCNGCAGCGTTCATGGACCTGATGAATAGGGTGTTCAAGCCGTATTTAGACNTGTTCGTNATCGTATTTATTGATGATATTCTGGTNTATTCAAGGAATGAAGAAGANCATGCNAATCATCTGNGAGTAGTTCTNCAAACTCTTCGAGATCGNGAGTTGTATGCNAAGTTCTCTAAGTGTGAGTTTTGGTTGGANTCTGTGGCNTTCTTAGGCCATATTGTGTCCGGTGAAGGAATTAGGGTTGATACNCAGAAGATAGAGGCAGTGAAGAATTGGCCCAGACCCACGNCTCCNACNGANATTNGNAGTTTCTTGGGNTTGGCNGGNTATTACAGAAGGTTCGTNGAGGGGTTCTCNTCCATTTCNTCNCCNTTGACNAAGTTGA

>rnd-1_family-103#Retroposon ( RepeatScout Family Size = 725 Final Multiple Alignment Size (possibly truncated) = 98 Localized to 2361 out of 4218 contigs )

TGTTATCTCCTGTATTCAGATTAGCAAACCAGAAATAATAAAAGATGAAAAGAAAAACANAAAGAACTATCCGAGTCCACAGAACCCACTGTGTGTCCTTAAGAAATTTAATCCCCTCAAGTACCCGAGGTTGCAGATTAATTCCTCCCAAGATAAAACGGATTAACCGTTAAAGAAGTAGCGGTACCTCAAACTTCGATAATTTCAACGAACTCAAAATGACAGCAACGAATCGCACACACAGACACGATCGATCGATTTTGTTTTGTAAGAAATGTATGCAGAAGAAGGGAAGAATTCGATGTTGAAAAATGAGAGAAAACCTCTCTATTTATAGCCAACAAAGGGTAAAGGTCACAACTCTTCGGAAAGAAGACAACCTTTCGGAAAGGTCACAACCCTTCGGAAAAGTCACAACCTTTCGAAAGGTCACAACCCTTCAGAAAAGTCACAACCTTTCATAAAGGTCGCAACCCTTCGNAAAAGTCGCAACTCTTCATAAAAGTCACAACTCTTCATTTCCCGTTCACACCTTTAAAACCCAACAAT

>rnd-1_family-399#DNA/CMC-EnSpm ( RepeatScout Family Size = 187 Final Multiple Alignment Size (possibly truncated) = 98 Localized to 2361 out of 4218 contigs )

TTTATCCAATAGGAAGACGCCACGTAATAAATAATAATAATAATAATTTAAAAATANAAGGCTGTTAGTTTTAAGGGTAATNNTGGACCAAAAAGGTNGACGTGAGGGGTATTTTTAGCCCAATAGGTGGACGAAGGGTATNTTTGAACCATTTCGAATAGTTCAAGGGTATTTTTGGCCCTTTTCCGT

>rnd-1_family-57#LTR/Gypsy ( RepeatScout Family Size = 1015 Final Multiple Alignment Size (possibly truncated) = 98 Localized to 2361 out of 4218 contigs )

TCTCACCTATTGAATCTATGAGCTATAGTATGAGTTATGTTGGATTGAGTGTCAAGACATTCTTCCATAAATATGAACTTAAGTCAAGACTTAATATGAACTCNATGGGAATTAATGCTTAGCACCGAGTGGATATGAATATGAGATGGAAGCTTTTACGCTAGTTGAGTCCGGCTTCCCAATGAAAGCTTTTACGCTAGTTGAGTCCGGCTTTCCAAATGGGTTCCTTCTACGCTAGTTGAGTCCGGGTTCCCAAAATGTGTATCTCATGAGATGGAAACCTTCTACGTTAGTTGAGTCCGGGTTTCTAGTAGCAATCTCCTTATCCCATAAACTATGTGCCCACATAGGTCTTTAGCTAGTGGATCCACCTAAGCTAANAGTNTAGTTCTACCTTAGGCAAGTAGGACAACCCCTTTTCGGTGTGGGGTAGACACCGGATTCCATGTNAGCTCACATGGTCTATGTCGGTTAAGGCTAAT

>rnd-1_family-155#LTR/Gypsy ( RepeatScout Family Size = 569 Final Multiple Alignment Size (possibly truncated) = 98 Localized to 2361 out of 4218 contigs )

ACTGAGTTGTTNTCTACTGTTTTAAANGCTTTNTATANACTGCATGTGTTTTATTGCTTTATATTGAGTTGAGTTATTCATGAGTTGAGTAAAGCCGAGGTAAGTGTTTCTTTCAGATTCCTTTCAAGCTTATGTCATGTTTAGCATTCCCACTCGCATGCTCGTACATTCAATGTACTGATGCCATTTGGCCTGCATCGTTTTATGATGCAGACACAGGTAACCAGGATCAGCATCCAGCGCCTCGTTGATCCAGTTGAGCACTCAGAGTCAGTTGGTGAGCCTCCTTGCTTTCCGGAGGATCCTTTTTATTGCTTT

>rnd-1_family-18#LTR/Gypsy ( RepeatScout Family Size = 1364 Final Multiple Alignment Size (possibly truncated) = 98 Localized to 2361 out of 4218 contigs )

TGTAACACCCCAGAANNTTTTTGAGCTAAGACTCGAACCATCCTTCGTTGTGAGTAGGATTTTACCGAGGAATTTAAAATTTCTTAAGTGTTAAGGTCACTAGATGTAGCACCTTGAGTTCCAAAAAGAACTAAAGAGAATTCGTTCAAGTCATTCCTAAGTTCTTTTAAGTTTTGGGTCAACTTCAAACGACCATAACTTTCAGTACANGATGAGTTAGGTGGCCCATAAGATATCAAATGAAAGGTCTTTGAATCNTCTTTCCAACGCCACCGAGTTTGCTAANTTTCGAGCTCGNATGAGGGAGATATGCCCGTTTGAAGTCGGGCTGTCCAGTTAAGGAAAGTTACCCGAAAATAGTGAGGGGTATTTTGGTCTTTTCCTTACCCAATCAGATTTAATNCGTTTTTAGTAAGGTTTTAGGGGTCTAANCCGATTAGGNTCAGTTTCATTAATCCCNAAATACGCTTAGGGTTTTAGAGAGAAGATTCAAGAGGAGAAAAAGTTCAAAGTTTCAAGCGTTCGTTCAAGAAATTCGAGATTTCGCCAAGAACCTAGTTCTTTGAGGTATGTAAGCTTCCATAGTGTTGGGTTCGTTCACCCACACGCCAATCATGAGTTTCTTTTGCGAATTTATCCATAAATATTGAGCGATTTAAGTTCTTGATGAGTTCTTGATAAGTGTTCTTGAAGTTTCCTTATCGATTAAGTTTTGATGTAAGATTCTACTTGGGTCAACTTCAAACGACCATATCTCTTAGAATATAAAGAGTTACGTGGCCCATAACCTATCAAATTAAAGGTATTTGAATCTNCTTTCCAACGCCACCAATTTCGCGTCAATCCAATTTTTGAGTAAAAAGTTATGACCATTTTAGTAACCTGATACAGTGCNGTNACGAATTAGCCGACGGGAAAACATTAAAAAGGGTCGTTTTTGTCTTTTTACCTCCAAGAAAACCCTAAACGAATTTCTTGACTAATNAAAGGCCCAAAACAATCAGATTTTCATCTCTTAATCCATCATTCTCTTCTCTCTCAAACCCTAAGGNAAAACCCTAAGGAAAAATCAAAGTAGAAGACTCCATTCAAGATTCTTCTTCAAGGTAAGTCCTTCTCCAAGAATTTCATTCTTTCCAACTCAAATTC

>rnd-1_family-381#Retroposon ( RepeatScout Family Size = 199 Final Multiple Alignment Size (possibly truncated) = 98 Localized to 2361 out of 4218 contigs )

AAAGTTCAGCCCTACCTTTTGCTTTACATATATCATTTTAAAGTTCCCCATTTAAATGTATCATAAGTAATTATTAAAATGGAAAATTAAAGGGAGAATGTATCATATTGGACAACAATTCAACTTATTATATATACTTGAAAGGGAAAAATATTACACAAAACTATTCAGTTTGTTTGGGTGTTTTTTNAAAATCAACATACCAGAGTGATGAAGTTCAATTTGAAACTACACTTCTATTCCAATCGCAGTGTCGAGAAACACATCTATTACTTCAAGAAAAGTATTTACAACTTGCAGTAAACTTGAGAATCTGTCCATACTTCATATTGAATATGAGCAATTAATTTATTGTGTAGTTATCTATGTCTTTGGGATTAAAAGANGCTCCAGTCTAGTCATATTCCTCTATAGGTCATCCGGCACATCCTTGAACCCATGTCAAAAGTTTATTAATTCTTGTCTTCTTTTCCTGGTTCACTTCGCACATCAAGGTGCGATAAAGTTTTTAAGGTCTTTATGTCCTTACAATGGCATACCAAATTTGAATTTTGTCTTCAAGAGATGGTAGTATAGATCTTTAAGGAATTTTTTTACTGTACCTTCAACAATTTCAGTAGAGAAAACTTGCGTGAATACTCAATTAACGTGTGTTGTTCAATCTGTACTAACAAATACTTATAACAATAACTTTGCAGAAACATAAACAACAAAGTTTAAANCATGTACTCAAAAACAACAATTAATAACATAAGCANAAATTAATGACTGTAAAAAACATACCAGGATCTGTAACAATAGAATGAAACAGAAAGGAAAAAATCGAGCCCACTGAATGCACAGTGTCCCCTTAAGGAAATTATTCCCCTCTAGTACCCGAGGTTTAANGGAATATATCCTCCCAGGATAGAACGATCTTANTCACCAGTGTATTGATACCNAAAACNATGGTGTCAGCGAGCCACTCAACGGCAGTAAA

>rnd-1_family-301#LTR/Gypsy ( RepeatScout Family Size = 282 Final Multiple Alignment Size (possibly truncated) = 98 Localized to 2361 out of 4218 contigs )

TTATCGGATTGTCGTGGTATCTTCTATTCTTGTGTGTTGATTATGGTCCTGCCTTGTTGTAGTATCGAGGGGGTTGTGAGTAAAAGTCGTTAGGCCACGTGAATTCAGCGAAAAAGGTATGTTAAGGCTATTCCCTACTTACGGCATGTTTCCTTAAAGCTTAGGAGCGATGTAATTGGGTTGTTATCCTTGNTATACTTGTAGCCGTNATTGTTGATTGTTGGCTGCTCGAGTNGATATAATCCTCCCTTGTCGGGATTCTTATTCAGTTAGTAGCGTCCCTATGTTGGACACGACTTAGAGGCTATTCGTATAGGTTGCTTATAACTAAATTGCTCGCTTTTAGCGATTGGATTGTTATTGTTGCCCTTGGGGCTATTGTGGTTAGCTGCAGGAATGTGATCTATGCCTAGGAGGGCTATGTGCTGCCTACGGGGCTATATTGATGCCTAAGAGGGCTATGNGTTGCCTACGGGGCTATATTGACGCCTAAGAGGGCTATGTGTTGCCTACGGGGCTATATTGACGCCTAAGAGGGCTATGT

>rnd-1_family-293#DNA/CMC-EnSpm ( RepeatScout Family Size = 296 Final Multiple Alignment Size (possibly truncated) = 98 Localized to 2361 out of 4218 contigs )

TAAGTTTGACTTTGGTCAACATTCTGAGTAAACGCGCTCGGATGAGAATTCCGTCAGCGCGGTTAGCTCCGGAATGTCGAGTTTGGTCTAGAACGACCCTTCGTTCGNNTCCCGAGGCTTCCGATCTCATTCCGAGCCCTTTTGTGGATTTTGGCTTAAAATGGCNTTTGGGNGTGGGACCCACTTTTTATCGAGACGACCTCGGATGGAAATTTCGACTGCGCCGTTGAGTCCGGAATATCGAATTTGGTAGGGTNGCATATCTCGTTTGCGTGCACGGGGTTCCGAACGAGTTCGGAGCACCCCGTCGGAGTTTTTAGTTTTTGGGAAATTGCAGATTTTCTGCAATAAATGCAGAAAAACCAGCAACATTTCCCAAACTTCAAACCCTCATAACTCTCTCATCTCTCAACCGATTTGGGCGATTCGAAAGGCAAAGTTGTGAGATTTTTCGAGGACAACGCGTTGGTGAGCTCGGATAGTGATTTGGGGTCCCCGTTTGAGGTAAATTTCGTAANATACCTGCTGCCACGACCCTTTTTGCGAGCTTGATTTTTGGAATTTTGACTTGATTATATCTCGGTCATTTTAGGTCCGATTTCGGTGATTCGAAGTCCCGCGCGTTGGGAATTGTCGTGGCTTCGTCGTAGAGTGTTCAAGGAGTGTTGGGAACCTCTCGTTTCTGGTAAATTTCGAGTTTAGGCTGCTGCATGTTTTCTTTTAAGTTGATATAANTGTGGTTTTCTTGCATGTTGGGTGTTGNTGCGTTTCGAGCCCCGAATTGTGTTCCATTTTGGGACACGAGTTCGGGGNGCTGTTTAGGACCTTTTGACGGAGTCAATTCCGGAATTTCGGCGCGGGTCCCACGATTCCCGTTTTGACTCCAAAATTGGTCCGTCTCCGTTTNTTGTGATTTTGGTGTCTAAACGACCGTANTAACGTTGTGATCCTATTTTTGATAGCGTGGCAGCGTTCCGAGGCCGTTCGGAAGGGAAAAGCTCCGGNGAAGTGATTTTGGAGCGCGCGCGATCGGCCTACAGGTAGGCTACGGCTTCCCTTCTCTTAGATTGAGCTCGAGTGTGTGAATGCATGTTGATTAGTTGGGATTTGGGGTGGGTAGTTATTGAATCNTGCNTAGGTGTTTAGAAATCATGTTTTAGGCCTATTTCGGGAATTATCGGGTAACTGTGAGCATGCTTTGTGTTATTAATGGACCCTCCTCGCTATGTGGAGTGCTTGCGTGCTTAATTATTGTTTATTTGAAGCATGTTTGGCCTTAGTCTAGGTTTAGACTAG

>rnd-1_family-542#Unknown ( RepeatScout Family Size = 112 Final Multiple Alignment Size (possibly truncated) = 98 Localized to 2361 out of 4218 contigs )

TGCACAACGCTATGTATCCCGCTATGTGGAATGTATCAAACCTAATGTATCCCGTGCACAACGCTATGTATCCCGCTNACATCATTTTTAAGGGATTTTTGTAAATAGAAAANTAGAAGGGATAGGATGTAATTTAGTNCTTACACTATGTGATTCCTGTAATTTATACAATTTAA

>rnd-1_family-526#LTR/Gypsy ( RepeatScout Family Size = 119 Final Multiple Alignment Size (possibly truncated) = 98 Localized to 2361 out of 4218 contigs )

AATCTTGATCCTAAACAAGGTATGTTTTTAGGAATGATGCAAATTGTTACTGCTCATAAATGGTATGTTAAATGTACCATATTGATTGATAATANTTTTTCTATAACTGATATTGCTATGATTGATAGTGGNGCCGATGTTAGTTGCATTCAAGAAGGTCTTGTACCTACTAAATATTTTGAAAAAACAACTCATATGGTTAAATCTGCTTCTGGACATGCTTTAGATATAAAGTATAAATTACCTAATACTCGTATTTGCCAAAATAAAGTTTGCATTCCNCATTTCTTTTTCTTGGTNAAAAATCAGTTATACCCTCCAATTATTCTNGGAACCCCTTTTATAAATGCTATTTATCCTTTTACTAGCATAAATGCTAAAGGNTTTTCTGCTACTTATGAAGACAGAGATATTAGTTATACTTTTATCACCGANCCTATTTCTCGNGATATTAATGCTTTGATTAATATGAAACAAAAGCATGTTGATTCTTTACAACTTGAGNTATTTAGTATGAATATATTCGATACTTTGAAATCTACTAAAGTACAGGAAAAATTAAATTAATTTCCGAACAAATNGCCATTGATATTTGTGCTGATCATCCTAGTGCTTTTTGGAATCGAAAAAAGCATATCGTNACTCTTCCATATGAAGATGATTTCTCTGAGGATGATATTCCTACCAAATCNCGTCCTTGTCAGATGAACGCCGAATTGGTNGAATTCTGCAAAAAGGAAATTGATAATTTGTTACAAAAGGGTTTGATAAAGCCTTCNAAATCACCNTGGTCTTGTACTGCNTTTTATGTTAATAACGCTGCTGAAAAAGAACGAGGTGTCCCCAGGTTGGTTATNAATTATAAACCTTTAAACAAATATTTAAAATGGATTAGGTATCCTATTCCNAATAAAAGAGATTTATTGNCNAGATTATATGATGCCAATATATTTTCAAAATTTGATTTAAAATCAGGATATTGGCAGATCCAAATATTTAAAGAGCATTCTTATAGAACGGCTTTTAATGTCCCGTTTGGACAATACGAATGGAATGTNATGCCNTTTGGNTTGAAAAATGCTCCNTCCGAATTTCAGAAAATNATGAATGATATTTTCAANCCATATTTGGATTTCATCATTGTTTATATTGATGATATTTTGGTNTACTCAAAAACTCTTGAAACGCATATTAAGCATCTAGACATTTTCAAGAAAATNGTTATNCAAAATGGTTTGGTNATTTCTAAACCAAAAATGAGTTTATTTCAAACNGANGTTAGATTTTTNGGNCATCATATTTGTCAAGGGAAAGTTACCCCTATTCAAAGATCTATTGANTTCGCATCAAAATTTCCTGATGTTATTACNGATAGGACNCAATTGCAGAGATTTTTGGGAAGTTTAAATTACGTNTCCCCCTTTTACAAAAATTTATCTCGNGATTTAGCCCCTTTATACGACAGGCTAAAAAAGGATCATAAANCGCCTTGGACTGATAGTCACACCGATCTGGTAAAGAATATTAAACTTCGNGTTNAATCTTTACCTTGTTTAACTCTTGCTAACCCTGCTTGGCAAAAGATTGTNGAGACGGATGCGTCTAATATTGGTTATGGNGGNATNTTGAAACAAATTAATCCNCATGATAAAAATGAATATCTNATTCGATTTCATTCNGGAAAATGGAGCGATGCCCAGAAGAAATATGCTACGGTGGCNCATGAAATGTTAACCATCGTTAAATGCGTNTTAAAATTTCAAGATGATTTATATAATCAAAAGTTTTTGATAAAAACTGATGCTCAATCTGTNAAATATATGTTTGATAAAGATTTTAAACATGATGCNTCNAAATTGATNTTCGCTAGGTGGCAGGCNCAGTTAGCCCCTTTTGATTTCGAAATCCATTATAAAAAGGGAAGTGATAACTCTCTTCCAGATTTCCTATCTAGAGAATATTTATCTTCATAATGAGTTTTCTTNCNCCCTTCTTTACTGANAAAACTTTGGTTACTATNTTGAAGGTAGTTCCTCTTAATAGAGATNTACAGGAATTNATTTTATGGAAAATTATAGATGATATGGTTAGAGAAGATTTCTCTATACATGATTTCGAAGAAATCGTCGATATCGACGACGAAAATTATTACAGTGATTACTATGATGCATTCGAATAAAACAAAATGTTATGTTTGCAGGATGGACCCTCCTTGGGTAACCAAGGGTAGAGGNAAAGGNAATAACACNCGNGGAAGGGGAAGATCATC

>rnd-1_family-295#LTR/Gypsy ( RepeatScout Family Size = 291 Final Multiple Alignment Size (possibly truncated) = 98 Localized to 2361 out of 4218 contigs )

AGTGATAGTTCTCTTGGTTACTTTAATGTGCTCTTAGATGGGTTTACCCTAAGTGGGGGATGATGAGTGAGTAGTGAGGTTGTTGATATCCTTATCTTTCCTTCTATTCCTATTCAAGCTTGAGACCAAGAGTTCCTCGTAGCTTGTTTCATGTTTTGGAGTCATGTGTGATTATGAGTTTCCATGATCTCCTTATGTTATGCATGTTCTTGANGAAATTATGTTATGCATGTTTTGAAGGATTCTTATTACATGAAAATGGAATTTTAGTGATAAAATTCATGTTTCTCATGTCGTTGTGCATCTTAGTATGGTGAATGCACTNTTGACTTCATGAGANGANTNTATGAGCATGCTTAGGTTNGGAGTTGAGAGATTCCTCCTTGAATGAATTGACCTTTGTGTAATANTTGCATATGAGCTCCATGATGCCATGTGTAGCATGTTNTGACTAGGAGATGGTAGTTCCTCCTTGAACATGACTAAACATGGTAATGTCATGCATGTTCTTGATAGTATGATGATTAAATGATAAGACTAGTAGATGATCTATANTCCTCATGTTGNGGTGCANCTAGTATGAGTTGCTTANTTGCCTCATGAGTNGAAATGTTGATCATGTCTATGTGTGCNTTTGAAAGNCATTGCATATTGGACCCTATGATGATATAAAAGGGGTAAATATCATG

>rnd-1_family-275#LTR/Gypsy ( RepeatScout Family Size = 324 Final Multiple Alignment Size (possibly truncated) = 98 Localized to 2361 out of 4218 contigs )

ATGTTTTTACACTAATGTNCATGATGATTCTTAAACTTGGTAAANTGCATGNTTTCAACTAAAATGTCCCTTTTAGCATGTTTTAAGGATTTTTATGCATGGCTATCATACTTAGTGCATTTTTGTACTAACCCATATTTTC

>rnd-1_family-364#LTR/Copia ( RepeatScout Family Size = 223 Final Multiple Alignment Size (possibly truncated) = 98 Localized to 2361 out of 4218 contigs )

CAGCATTCATATCATCCATTGAGCCNAAGAACGTCAAGGAAGCCTTNNAGGATGCAGATTGGGTNACNTCAATGCAAGAGGAACTTCATCAGTTTGAGAGAAGCAAAGTNTGGNACCTGGTCCCCAGACCTNTCGACAGAACCGTCATTGGNNCNAAATGGGTCTTCAAAANCAAACTNAANGCNGATGGNANCATCGANAGATACAAGGCNAGACTNGTNGTNAAGGGNTACANNCAANAGGAAGGNATTGATTACGATGAGACNTTTTCTCCNGTNGCNAGAATGGNNACNATNAGAATNNTNNTAGCNTTNGCNGCTCACATGGAATTTAAGCTNTATCAAATGGATGTCAAAANTGCCTTCCTNAATGGNGATCTGGAGGAAGAAGTNTACGTGGANCAACCNCAAGGTTTTGAAGATGCAGAACTGCCAAATTATGTGCTAAACTNAANAAGNCNCTGTATGGNCTNAAACAAGCNCCNAGAGCNTGGTATGAAAGNTTGNCNAAATTCCTGCTTGCAAATGGATTCAAAAGAGGTAAGATTGATAATACNTTGTTCCTAA

>rnd-1_family-34#LTR/Gypsy ( RepeatScout Family Size = 1192 Final Multiple Alignment Size (possibly truncated) = 98 Localized to 2361 out of 4218 contigs )

TAGTGTCGTCACGCAATCACCCCACTCCTCGTTGCTTGTAAGGTAATAGACTTTCCCCTCACTTTGAATTTCGGTTCATAGATTGAATATAGAAAGTCGGGAGTCGGGTCTTGACCTTGGGTAATTGTTAGATGAAATTGCATGATAAANTTGAAAGTTACAATGCCCTTGGAACGATAGATAGTGATTGTGTNGCCTTATTGTATGTGATCGTAAGTCTANATGTNAGTTTCGACTTAGGGTTCCACAACTAGTCTAAANTGTTTGGTAAAATGATCAGTCGGAATCCTAAGAATGACGAACTAGCATAATTTAATGTTGTAAATGCATGATNAGACTGTGNTAATGTTGAAAACTAAGGCCAAAAATGTATGATACCTGGCACAGATGGCATCCACGATACCCCGTCTACGGACCGTAGATCCGTCTACGGACCGTAGATNAGGTTCGTGANTGGATGCACATGAAAAATTTCACCAAGTGTGAAACCACGGATGTGGACTACGGTCCGTAGATCGATCTACGGACCGTTCTGCACATCCGTGGTTTCCATCTGCGACCNATATTGTNGGCCCCCTGATCCACGGAAGGGATCCACGGACCGTAGATCAGTCTACGGACCGTAGGTCTCCTCCGTGGATGACCACTGTAGCCTCTGTCTGAAATTTTCTTGGGACTTNGTTGNTGTTCGTTNNCAGTTCTAAAAGTTTTTTTTTGTTGCGTATGGTTAGTTTTGGCACTAATATCGCTCCCGCAGGTACGATGGCACCCAAGAAGCTGGTCACCTACTCAAAACGGGGCAAGTCAAAATCTGTTGCCCCTAGCTTCNGGTTAATCGATGAGGACACGGACACCGANANAGATCCAGCATACGTTCCTCCCAACACNAGGACTTCTCCCACTGCACCCCGAGCCACCAGAGGCACCCCCCGGAAGGTGNTTCCCGACGTAGTCACTGTCTCCCAGTCTGATGAGGAGCACACACTGATCGGGTCACCAACTGGGGCTGCTTCCAGTTCAGAGGGGTCTACGTCCGGNTCCGAGTCTGCCCATGCTTCGGGCTCCGAGTCCGCCCATGCTTCAGGNTCCGAGTCTGCCCATGCTNCGGGGTCCGGTGCCGAGTCAGCCACAGGGTCCGGCGAGAATGACCAAGCAGCCTCGTCTGATGAGGCNACTAGCTCGGAGTCCGTACCNGTACCACGGAACGACGACCCCGCTCCAGTGGCCGGCGAGCCGAATAGATGGTGCGTNGAGGGCCAATGGCAAATNTATCGGGATGCCAAGATGATNAATGACAAAGAGAAGATGGCCCGACTTATTACAGAGGAGCGCAGAGTCCTCACGGGGAGCTTGCACACCGTNCCCGACATCCACCGGCTNTTCAACCTTCACAAGTGTGACTGGATGGCTCGAGACCCAGGGACNTATAGCGAGGAGATCGTGCGGGAGTTCTACGCCTCCTATGCCGCCACTCTCCGNGGTTCGATTTCCAAGCGGTCAAAGCCCNTAGCCCAGGACCCTCTCACTTCCACTATGGTCCGGGGTTGTCCGGTGGACATATCACACGCCACCATCAGCCGNTTTCTCTATGGTCCTACCACGGGTCACTCTTGGTCNCTCAACACGGCGGAGTTTGACTACCGATGGGACATCGTGCGGAGCGGCGCTTTCCAGAGGAACGCCGAGCAGCGGGAGGCTGTTNTACTATGGTTGGCCAGGTACATTGCTGCAGATGGNGAGCGCGCGGAGTGGGTCGCCGCTCCGCGGTTGGGCATCCGGAAGGCCACATTGAATTTTGCGGCCAAGTTCTTCTGGCTGTTGGTGCGNAACAGAGTGTCGCCTACAAAGGCTGACAATNAAGTCACNTGGGACAGAGCGGTCATGGTTGCAGCATTGGTAGCAGGANTGGAGATCGACTTTGCCCGCATGCTGCTGGCAGAGATTCACGAGAGGGCNTTCAAGACCTCCACTACTTACCCTTCCCATGTCTGATTTTTCANTTGTGCAGGGACTCTGGAGTGCCGATCTGGCATTGTGACAGGTTGGTCCACCCTACAGGGACNTTGGACATCGGCCTCATTCGAGATGAGGCAAATGTGGCGGCACCTCGCAGAGAGCCCCAGGTTGAGGTACCTCCCTTGGGCGCCGATCTTGCAGACGCGGTGGGGCAGGCGCAGGGCGGTGACCCCATTATCCCAGACCACACCGACGCTGTCCCGGCCTCCTCTTCTCAGGCCGCTAGTNNGGCTCCTAGCTCNTCCCGGTCCACACCACCNTCAGGAGCTACCGTCGTCCCGTTGGCCAGAGTACAGAAGTTAGAGGCTCAGATGGCCACACTGCTGCATCACGTCCAGCCNTGGATGCAAAAGTCGATAGCCGAGTCCGAGGCNAGGATGGAGCGNAGGATGGAGGGGATGATGGACCGGAAGGTCCAGGCCGTTAATAAGCGCCTCGATGCCTTTGA

>rnd-1_family-443#Unknown ( RepeatScout Family Size = 156 Final Multiple Alignment Size (possibly truncated) = 98 Localized to 2361 out of 4218 contigs )

TCAATTACATGNTTTCCAATCGATTTCATATGAACCCATGCATTGATCATGATTTTGACTATGAACCCTAANTCGTATGAATTTATGAAGAAGCTATGAANCTATGAATGATGTTTTTATGAANTTATGCATGATTTATGAAAAGAAGAGTTTTATGATCTAGGTTGCTTTAAGATAAGCATCAATGAAATTGTGAAAGGATTTTCTCACATATTAAGGAATCATGATTTGGTGAAAGGTTTTCTCACATATGCNTGATTCAAGATTTAAGGAGCTACTCTATGATGTTTT

>rnd-1_family-391#Unknown ( RepeatScout Family Size = 191 Final Multiple Alignment Size (possibly truncated) = 98 Localized to 2361 out of 4218 contigs )

CCCAAGTGAGGACCACGGGGACCTTCACGGTCCGTGGTCCTCGNCACGGGCCGTGGTGGCGTCCGTGAAGCCTAGCCAGTAGCCCCCTAAATCCTGAGCCACCAGACCTCAGCCCAAGTGAGGACCACGGGGACCTTCACGGTCCGTGGTCCTTCACACGGGCCGTGGTA

>rnd-1_family-457#DNA/hAT-Tip100 ( RepeatScout Family Size = 145 Final Multiple Alignment Size (possibly truncated) = 98 Localized to 2361 out of 4218 contigs )

ATAATACATAAACATGACNCTTAACTTGGCCTCAGCTGACAANTAAGCACTCCAACTTTGAGTGTGCACATCTAGACACCTCAACT

>rnd-1_family-380#DNA/PIF-Harbinger ( RepeatScout Family Size = 200 Final Multiple Alignment Size (possibly truncated) = 98 Localized to 2361 out of 4218 contigs )

TTAGGGGTCGTTTGGTAGAGTGTATTAGNAAAAATAATGCATGCATTAGCTTTGTGTATTANTAGTACCTTGTTTGGTACGCTTTTTCANCCTATGTATAACTAATGCNTGCATTAGTTATACACTCTATTGTGTATTAAGGTGTGTATTACTAATACCATGGATTTCTAGGTATTAGTAATGCAANGGTTTTAATGCATGCATTAGCATGATTAAAGACNCAATTACCCCTCAAAACCTTTTTTACATCTTTTCCACCATAATTGTGGAGGGTATTTTTGTAAATAAATATTTTTATGCAATGCATGCTATTTTTAATACACCAAACCAAACAATGCATAAAAATAATCTATGTATAACTAATGCAAGCATAACTAATACANGCATTACTAATACACTCTATTTAGCATTATTTTTATACACTCTA

>rnd-1_family-49#LTR/Gypsy ( RepeatScout Family Size = 1091 Final Multiple Alignment Size (possibly truncated) = 98 Localized to 2361 out of 4218 contigs )

ATTTTCTTTATATTGCACTTGTTTTTAAACTGCTTTATATTGAAATGAGTTCAGTTATGTTGAGTTGAGTNGAGCCAAGGTAAGTGTTTCTTTCAGATTCCTTTCAAGCCTATGTTGTGTTTAGCATTCCAACTCGCATACTCGTACATTCAATGTACTGATGCCAGTTGGCCTGCATCNTATTATGATGCAGACGCAGGTAACCAGGATCGGCATCCAGCGCATCGTTGATCCAGTTGAGCAGTTCAGAGTCAGTTGGTGAGCCTCCTTGCATTCCGGAGGATCCCCCTTTTTATTGCTTTATCATTTTAGTTCGTTAGGATGTCGTGGGTCTGTCCCGACATCCATCTCAGTTGTTTTAGAGGCTTCATAGATAGNCAGTCAGATGTTAGTTCATTNGTCTTTCATTTTGTTATTGGCTTATGTTNAGACTTGAGTTGCCATTTTGGCTAAGTTGAATGTTTATTTTTAAAACATTCTGAGTTCATTTTGAGACAGTTGAGTTATNTTGCATTTGAATCGTTTTNNTTATGCNTANAGTCTTCCGCTGAGTAAGTAAGCCAGGCCAAGGGTTCGCTTGGGGCCAGCAATGGTTCTCGAGTGCCGGTCNCGCCCAGGGTGTAGGCTCGGGGCGTGACAAACTTGGTATCAGAGCACAGAGTTCAAGAGTCCTAGGGAGTCTATGAAGCCGTGTCTGTAGAGTCCTAGTTATCGGTGTGAAGCGCGCCACATCTATAATTAGGAGGCTGCAACATTTAGGAANTNTCTCACTTCTTTCACACTCATTTCGTGCGNTAGAGTTCGATCTCTAAAAAGTTTCCTTCTAATTCGTGCTTGCGCGTGTTTTT

>rnd-1_family-152#LTR/Gypsy ( RepeatScout Family Size = 579 Final Multiple Alignment Size (possibly truncated) = 98 Localized to 2361 out of 4218 contigs )

TGATGAGTCCGAGATTTCGACTCATTTAGGGCTTTGTTTTNATAGTTTTAGTGTCCTCAAATGCCTAATTTATGCTATAAACTGATGTTAAAGCGTTGATTTNCAGNNATGNAGGCTAAGGAGCAAGGCAAGGACACTACCGGGCAAAAAGGAGCGAAACAAGCTGAAGAAGTGAAGAAAGGCGAGCCTGGTGATCGCCAAGACCACTCGGCGAGTCGCCGAGTGGCTCTTTAGCTCGCCTAAAGTTCCAGCGTGCCAGCCCTGGGGAAGAACCAANTCGGCGACGGAAATGGGCAGTCGGCGA

>rnd-1_family-307#LTR/Copia ( RepeatScout Family Size = 272 Final Multiple Alignment Size (possibly truncated) = 98 Localized to 2361 out of 4218 contigs )

TGTTAAAGAATGACAAAAGTCCCACATCGGTGGTTAATGAGATGGGTGGACTCCTTATAAGGCTTGGGCAATCCTCCTCCCTTTGAGCTAGCTTTTGGGGTGTGAGTTAGGCCTAAGACCTAATTTAACATGGTATCAGAGCAGGGCCCGTCTCACCCGATGTTGGGGCCCCCAAAATCAAAATTGCCCACGCACCAGATGCTAAGCACTGGGCGTGAGGTGGGGTGTTAAAGAATGACAAAAGTCCCACATCGGTGGTTAATGAGATGGGTGGACTCCTTATAAGGCTTGGGCAATCCTCCTCCCTTTGAGCTAGCTTTTGGGGTGTGAGTTAGGCCTAAGACCTAATTTAACA

>rnd-1_family-222#LTR/Gypsy ( RepeatScout Family Size = 421 Final Multiple Alignment Size (possibly truncated) = 98 Localized to 2361 out of 4218 contigs )

ATCAAAATGGCGCCGCTGCCGGGGAGTGGTGTTGTTTGAGAATTCTTTTAGTNAAGTTGAATTTTGTTCTTNTTAGTCGTAGTTGAATCTACTTACTTTTAGTTTTGGTTTTTGTGTGTTGTTGAAACAGAAGANATGAGTGTNAACGGNAGCAATGGTAGCCAAGTGGGCCACCAAGACGACATCGGAAACCTCAACGATGTCAATGAGCCNAACGTCAATGACCCANATCNAATGGGTGGTGTTGGTGCCATTCGTTTGCCTCCGGCCGAAGGGAACGCGGTGTTCCACGTNACGAGCACNATGCTGCAGCTCTTGCAANTGAAGGGNCTNTTCGGNGGGTTGGCTCATGAGGATCCCCATGAGCATATTCGAAACTTCGTGGATGTNTGCGGNCCGTTCTCGTTCAAGAACATATCNCAAGAATCGGTCCGGCTNAGGTTGTTCCCATTTTCTCTGATGGGGGAAGCNAGCAAGTGGTTGGCCGAGTTGCCAAGGGACTCCATCACTTCGTGGGANGAGCTNATCACNGCATTTCANGTGAGATTTTTCCCTCCNTCGAAGATGATGACNCTNAGGGATAACATCCAAAGCTTCAAGCGNTTGGAGGGTGAGCCAATCCACGAGACNTGGCTGAGGTTTAAGAAGTTGGTGCTNCAATGCCCAACTCACGGNCTNCCNGACAACGTGTTGCTGCAATACTTCTACCGGAGTCTNGACTCGGTNAACAAAGGNGTNGCTGACCAACTTNTTCCGGGAGGNATAATGCAACAACCNTNCGAAGTAGCGTCTCAACTCCTTGATGGCATGACNAAGATNAACCGGGCGTGGTACACCCGNGAAGACCAAGTCTCTCCTCTCACTTTTAGANTGACGAAAGAGCAGATCGAGAAAGATCAAGAGAGGGACCAAAACATGGCCAAGATGATGACCCAAATGGAANTNCTACAAGAACGTCATGGA

>rnd-1_family-469#DNA ( RepeatScout Family Size = 141 Final Multiple Alignment Size (possibly truncated) = 98 Localized to 2361 out of 4218 contigs )

GGTATGAAGGAAATGTTTTCCAATTTTCTCATGTTTGGTTGGGTTAAATGTTTTGGAAAATGTTTTCCAAATCAACTCATTTTCCTCAAATTTAAGGAAAATGACTTCCCTTCAAAACTTAAGGAAAACATTTTCCAAAACTCTCTTCCAACTTCAAATTACAATTATTTTTTTGTTGAAAAAATCAATTTATTTTGTCCCTACCCTCAAACCAGCCCCCCAACCA

>rnd-1_family-115#LTR/Gypsy ( RepeatScout Family Size = 679 Final Multiple Alignment Size (possibly truncated) = 98 Localized to 2361 out of 4218 contigs )

TTTTGAGTAATTATCTCAAACCANAGAAAGAGTTTTGTTTTTAAACATATGAGCTGAGTATATTTTGGGAGTAGTATTGAGCACCGATATGGGGACGAGTTCATAATAACTCAAGTCTCCATAAACCATGTAGCCATCGTGGGTAGAAAGGATCATACTTTTTAGATGATTCCTTAATGCTTTTTAGCATAGACTAGTGGATCCACTTAGTTGAGGCGTTCTATACGACGGCAAAGTATAGGACAGTTCTGGCAGCGTGGGCGAGACGTTGTATCATCACTTAGGCTCATAGTGGTGGTTGTCGGTTAGAGAAACTCCCACAGAGTTATATTATATTTTTATATACAAATTGAGTTGTTATTGTATTTTTTCAATACATTGAGTTGTTATCTACTGTTTTAAAGCTTTCTATATATTGCATCTTTATTGTTGCTTTATCCTTGAGTATTGAGTTGAGTATTCATGAGTTGAGT

>rnd-1_family-22#LTR/Gypsy ( RepeatScout Family Size = 1313 Final Multiple Alignment Size (possibly truncated) = 98 Localized to 2361 out of 4218 contigs )

CCCTTCAAAACTCGATTTCGAAGCTNGCAANTTCTCCAAAANCTAGGGTTTTCAATCTAAGTCATGGGTTCTTCCTAAAAACGNTTTCAATTGTTGATTTATGTTGATTATGATGGATTATGGTTGAATTGATGGTTTATTGNTGATTTATGATGAAAATCCCCATGAACCCATGAATTCCCCATNTTNCTAAATTGTGAATTATGATGTGGGTTGATTGATCATAGAAGNATGACAATTGATTATGTCTATGTTGATTGAGTTATTGAATCATGATATTCCCNTATCTAATGTAGTAATTCTAGATGTTTGGGTTGANCGTGATTCATGGCCCTTGAAGGGCAAATTATGAGGGTTTGTACATGTTGATGAGAATTGTGTATGAGCTTATGATTCACTTAGAAAGTGAAGATCCTATGACTATTGTGTAATTGTGGTATTGTTGAAAGATCATTCTCCCCNTACCCTTACACATGACTATGCATGAANTGTCTATGATTATGATAATGTTGTTGTGTTGATTGAAAGGCTATTCTCATGAACACACTATGAACATGATGTGAAAGGTTTACTCACATATTGATGATTCTAAGGTTGAAAGGNCATTCTCACCTAATGAATCTATGAGCTATTATGATATCGTGTTGGATTGAGTGCTAGGGCATTCTTTCACGAACATGAACTTAAGTNAAGACTTAACATGAATTTAAAGGGAATTATGCTTAGCACCGAGTGGATATGAATATGAGATGGAGGCCTTCACGTTAGTAAGTCCGGGTTCCCAANGTAGTTATCTCATGAGATGGAAGCCCTCACGTTAGTAGTCCGGGTTTCTAGTAGCAATCTCCTTATCCCATAACTATGTGCCCACATAGGTCTTTAGCTAGTGGATCCACCTAAAAGCTAATACGTTAGTTCTACCTTAGGCAAGTAGGACACCTCTTTTCGGTGTGGGGGTANGACACCGGATTCCATGTAGCTCACATGGTCTATGTCGGTTAAGGCTATACTTCCCTACATGATAAGAATA

>rnd-1_family-555#Unknown ( RepeatScout Family Size = 107 Final Multiple Alignment Size (possibly truncated) = 98 Localized to 2361 out of 4218 contigs )

AGGTCAAGTCAAGTCTCCANTCTTCCAAGCTTTGTGGGCTTTGTAATCAAGGTATGATGGCTTATTCATCCAT

>rnd-1_family-134#LTR/Gypsy ( RepeatScout Family Size = 620 Final Multiple Alignment Size (possibly truncated) = 98 Localized to 2361 out of 4218 contigs )

AGAAAGAACTAAATTGGAAATAGCAAAATCTGAAATTTTGCAAGTTATGCTAAGTTATGAGTTTTGGGTCGACTTCAAACGACCATAACTCTCAGTACAGGATGAGTTAGGTGGNCCACAAGATATCGAATGAAAGGTCTTTGAATTATCTTTCCAACGCCACCGAGTTTGCTAAGTTTCGAGCTCGTATGAGGGAGATATGCCCGTTTGAAGTCGGGCTGTCCAGTTAAGGAAAGTTACCCGAAAATAGTGAGGGGTATTTTGGTCTTTTCCTTACCCAATCAGATTTAATTCGTTTTTAGTAATATTTTAGGGGTCTAATCCGATTNGGTTCAGTTTTATAATCCTAANATACGCCTAGGGTTTTAGTTGAGAGTTCAAGAAGAGAAAAGAAGAAGAGA

>rnd-1_family-28#LTR/Gypsy ( RepeatScout Family Size = 1262 Final Multiple Alignment Size (possibly truncated) = 98 Localized to 2361 out of 4218 contigs )

TTTTCCTTAGCTTTGAAGAATTGAAGNTTTTCACTTTTGAGAAATTGGAAGTGGGTCTTTGAGATTCTTCAANTTGGACCNTTGTAAAGACGAAATTAATCTTACCCAGTTGATGGAAACNCAATTTGGTAACGATTTTTATCTTTTTATGATGTCTAGCTAAAACCCCAATTCTTGGGGTGTGATTATGTGATTATGGGCTGATTTAGCTTATGGGTATTGCTAATTGTTAGTTTAAATGCTGTTTAGAAGTGATTTCAATCAGTAATTGTGGTTTAATTTAAGAATTGTAGTTGCAAATGCAGTTCTACCTTCGTGTTTTTGGCTTGCTCGAGAGAGAGGTTTTAAAACCAAGATTATTGATTGATGGTCTGTGGGTATTGGGTTGTCATGGGTTCAGCTCGAGAGAGTGAATCCTAAACCCTNTCCCACACATTCAGCTCGAGAGAGTGAATGGACTAAGGCGTAGGCTGTTCTTAATTTGCATGCTTGTTGGTGTTCGAGAGAAACCGACTTGATTCGGGGTAAATTGTTCGAGAGAAAGTTTACCTCCNCTATAGTCTAGCTTACTCACTAATNTNCAGCTATTTACTAATAGTTGCAATTACCCATTTGTCTATATTAGCCTATAATCCGATCACATCCCAAGAACCCGTCTCATTATTGTTATTTCGTATTGTTTGCGGCTGTTGTAGTTGATAATTAAAACCAAAACCCCCCATTTGACATTCGTGTCACCCCTTGATTTGAATTATGTTTTTATTCGATAATGTCTATTCCTATGACTGATTTGANCATATTCGTACTTTCCTATTGAATCTTAAACACGTACCGCTCCCTGTGGGATTCGACCCCAACTCATTTAGTTGGGTTNTATACTGATTAACGATCGTTGACGCTTAGAATTGGATGAAGTGTCCTTGAAACGTTAAATCAAAATGGCGCCGCTGCCGGGGAGTGGTGTTGTTTGAGATTCTTTTGGTNAAGTTGAATTTTGTTCTTNTTAGTCGTAGTTGAATCTACTTATTTTTAGTTTTGGTTTTTGTGTTGCTTGTTTAAACAGAGAAAATGAGCGTNAACGGAGCAACGGTAGCCAAGTGGGCCACCAAGACGACATCGGAAACCTCAACGATGTCAACGAGCCNAACGTCAATGACCCNNATCTAATGGGTGGTGTTGGTGCCATTCGTTTGCCTCCGGCCGAAGGGAACGCNGTGTTCCACATCACGAGCACCATGTTGCAGCTCTTGCAACTGAAAGGGTTGTTCGGTGGGTTGGCTCATGAGGATCCCCATGAGCATATTAGAAACTTCGTGGATGTNTGCGGACCGTTCTCNTTCAAGAACATATCNCAAGAGTCGGTCCGGTTGAGGTTGTTCCCGTTTTCTCTGATGGGAGAAGCGTGCAAGTGGCTGGCTGAGTTGCCANGNGANTCNATCACTTCGTGGGANGAGCTGGTCACCGCATTTCAAGTGCGATTTTTCCCTCCTTCGAAGATGATGACTCTTCGGGACAGCATCCAAAGCTTCAAGCGTTTGGAGGGCGAGCCAATCCACGAGACTTGGCTGNGATTTAAGAAGTTGGTGCTNCAATGCCCAACTCATGGNCTGCCCGATAACGTGTTGCTGCAATACTTTTACCGGAGCCTTGACTCGGTGAACAAAGGNGTNGCTGACCAACTTTCTCCCGGAGGTTTAATGCAACAACCNTACGCGGTAGCGNCCCAACTCCTNGATGGCATGACCAAGATCAACAGGGCGTGGTACACCCGCGAAGACCAAGTCTCCCCTCTCACNTTTAAANTGACNAAGGAGCAGATCGAAAAAGATCAAGAGAGGGACCAAAACATGGCCAAGATNATGACCCAGCTNGACATCTTGNCCAAAAATGTCATGGGNGCTGGTGCNAGGAGTGTCAATGTTGTGGGTGTCGGTGGTGCGAACCCCGANGAGGCCAAGTTTGAAGCGTTGTACAACGAGGAAGTGAACTTCCTAGCCAACCAAGGAGGNGGTTATCGTTCAAACTACCCGAGGCAGGGTGGTAACCAAGGTTGGAATAGAGATGAGGGCTGGAGAGATCGTGACAGAGAATGGNGNGATCGTAACGCCACTTGGAAGGANAGAGAGGGGGAGAAGGATAGGTACGTNCCTCCCCACGAGCGTCAAAAGCCCAAGGATTCNGAGGGTGGCCGNNCGGAGGATATGCTCTCNCGTATTCTCAACAAAGTTGAAGGGTCGGACAAAGTNTTGAAAGAAATGAAGGAAGATGTGTCGACCCTCAGCCAGACGGTCACCTCTCATTCAGTCTCNATCAAGCAGTTGGAGACCCAAATGGGTCANATCTCGTCTCATCTGAACCCGAGACAACAAGGGGGGTTGCCTAGTGATACTATGGCNAACCCCAAGAATGAGGCTTGAATGGGTACTTGCGTCGTGCCACGACGTTAACTAAGGCGCTNCTTGGGAGGCAACCCAAGTTTTTANTGCTTTNTGTTTTGATTTTGAATAACGGTGTGTTGATTGTGCAGGTTGAAAAGTGGAAAGTGTTTGAAAAGTGCAGGTTGGCGAGCCAAAAGTCCAGTCGGCGACTCGCCGAAGAGGTCGGCGAGCCCGACTTGGACCGCCGTTGGACTCA

>rnd-1_family-78#LTR/Gypsy ( RepeatScout Family Size = 844 Final Multiple Alignment Size (possibly truncated) = 98 Localized to 2361 out of 4218 contigs )

TTTCAAGCTTATGTCNTGTTTAGCATTCCAACTCGCATACTCGTACATTCAATGTACTGATGCCAGTTGGCCTGCATCGTNTTATGATGCAGACGCAGGTAACCAGGATCAGCATCCAGCGCCTCGTTGATCCAGTTGAGCACTCAGAGTCNGTTGGTGAGCCTCCTTGCNTTCCGGAGGACTCCTTTATTTGCTTTCTAGTNTTTCAGTTGTTAGGATGATCGGGGGTCTTGTCCCGACATCCCTCTTNGTTTTAGAGGCTTCATAGACAGTTAGTTAGTTCAGTTGTCTTTACATTNTCATTTCATATGTTAAGACTTGAGTTGCCATTTTGGCCAAGTTGAATGTTTACCTTTAAACGTTCTAAGTTATTTTATTAAACAGTTGAGTAAGTTCATTTGATTATTGTAAAATGCTTAGAGTCTTCCGCTGAGTAAGTAAGCCAGGCCAAGGGTTCGCTTGGGGCCAGCAATGGTTCTCGAGTGCCGGTCCCGCCCAGGGTGTAGGCTCGGG

>rnd-1_family-402#DNA ( RepeatScout Family Size = 183 Final Multiple Alignment Size (possibly truncated) = 98 Localized to 2361 out of 4218 contigs )

TTTAGGGGGGTAATTATTTCANTTTNAAATAGTTCAGGGGGGTAATAGGACCCCCGTAAAGTATGAGTGTGTAGTTGGGAATTCGGNTATAGGTTGGGGGGGCATTTGACCATTTTCTCTA

>rnd-1_family-225#LTR/Gypsy ( RepeatScout Family Size = 417 Final Multiple Alignment Size (possibly truncated) = 98 Localized to 2361 out of 4218 contigs )

GATGTTGTATATGTTCGGTATGTGCATGTGTTACTATGTTGTAGTTACTTGCGTATGTTNCACTTANTGGGATAGCCGCGTGATCCTACCAGTACACTGTGGTTGTGTACTGATACTGCACTTGCTCTTTCTTTGTTGAGTACAGGGCATCTTCAGGCGGCTATTGATAGACCTCGGCTAGGAGACCATGAGCGTGACCGAATTCAAGGGTGAGCCAGTTCTTCCGGGCTGCCATGGATCTTCCTTGTTTAAGTCCACTCTTTTCGGACTCAGACTATTTGTTTAAGGTGTTGTTTAGTTTCGGGGTTGTACCCCTTNTTCTTAGACTTGTCGTTAGTAGAGTTTTGGTACAATGACTTTCAGGTTCTAGGGGTTGTTCTTCCGCATTAGTT

>rnd-1_family-400#Unknown ( RepeatScout Family Size = 186 Final Multiple Alignment Size (possibly truncated) = 98 Localized to 2361 out of 4218 contigs )

AAATGCCCTTAAACTATGCGAAAGGAACAAAAATGCCCTCCGTTTATAGTTTGGTTCAAAAATGCCCTTGCCGTCAATACTTTGGTCCAAAAATGCCCTT

>rnd-1_family-361#LTR/Copia ( RepeatScout Family Size = 225 Final Multiple Alignment Size (possibly truncated) = 98 Localized to 2361 out of 4218 contigs )

TCAGAATCAGGTTCCACATTCCATACTGTNTCCTCAGTCACATCTCTATTCTATCCCCCCTCGTGTCTTTGGGAGCACATGCTTTGTTCATAACTTAGCCCCAGGGAAAGATAAATTAGCCCCTCGTGCCCTTAAATGTGTCTTTCTTGGTTACTCTCGAGTTCAAAAAGGGTATCGTTGCTATTCACATGATCTCCGTCGATACCTTATGTCCGCCGATGTTACATTCTTTGAGTCTCAACCTTACTATACATCTTCTGATCATCCTGATGTCTCTGAGGTCTTACCCATACCTCAGGTCTTACCTGTACCGACTTTTGAGGAATCTACGGTTACTTCTACATCTCCAGTTGTAGTGCCACCACTCCTAACTTATCATCGCGTCCGCGTCCAGCACTAGTCCCAGATGATTCATGTCATGCGCCGGACCCTGCTCCTACTGCGGACTTGCCTCCTCCTAGCCAACCGNTTGCNCTTCGAAAAGGTACACGATCCACTCGAAATACTAACCCACATANTNCNTTNTTAATTTTGTTTCCTATGATCGTTTATCCTCACCNCATTATGCCTTTGATTGCCTCTCTANACTCTATNTCCGTTCCTAAGACTACAGGTGAAGCACTTTCTCATCCTGGATGGAGGCAGGCTATGNTTGATGAGATGCGTGCTTTAGANGANAATCGTACTTGGGAGCTTGTCTNCCTTCCCGNAGGTAAANCTACCGTTGGNTGTAAATGGGTNTATAAAGTCAAAATCGGTCCAGATGGTNANGTNGANAGGTTCAAGGCTAGACTNGTNGCTAAAGGGTATACTCAGANANANGGGNTNGATTATNNTGANACTTTCTCTCCNGTNGCCAAAATGGCNACCGTNNGNNCTNTTATTTCTNTGGCTGCCGTTCGNCATTGGCCTNTNCATCAGNTGGATGTCAANAATGCNTTTCTNCATGGTGATCTNNANGAAGAAGTNTATATGGAGCAACCACCTGGTTTTGTTGCTCAGGGGGAGTCTAGTAGCCTTGTATGTCGATTGCGNAAGTCNCTNTATGGNCTGAAACAGNCTCCTCGAGCTTGGTTTGGNAAGTTCAGCGCGGTAGTTCAGGAGTTTGGCATGACTCGTAGTGGAGCTGATCACTCTGTGTTTTATCGACATTCTGCACCGAATCGGCGTATCTNTTTGGTTGTNTATGTTGATGATATTG

>rnd-1_family-247#Unknown ( RepeatScout Family Size = 366 Final Multiple Alignment Size (possibly truncated) = 98 Localized to 2361 out of 4218 contigs )

CCTTCACGAGCCACTTCACGGCTCGTGGTCATGACCACGGCCCGTGGGAAGGCTCGTGGGGTTGCCTTGGCCTCGTGGGAGTCGTGNAAAGTTGAGGGAGCTACTGCCTCAAGGACCACGGGCACCACCACGAGCCGTGGTGCCCTTGACGGNCCGTGAAGGTGGTCGTGAAGATCACTTGGGCGCGTGGGAGGCAAGGCTTCGAGGCCTAGCCACTGCCTCAAGGACCACGACCGTCACCACGACTCGTGGTCTTGACCACGGNTCGTGGGAGGTCTCGTGGTCATGCCTTGGGCCCTTGAGGGCTTGGCACGTTGAGGAAGGCTACTGCCCAAGGGACCACGGGCGGGACCACGGGCCGTGGTCCCCNCCACGGCTCGTGAAGGTGGGCGTGGTCCCTGACGGGGCCGCCTAAGTTGAGGGCTTTTGGGTAATTTCCTTTTAATTAATTATTAAGTGGGGTCGTTTTGGGTAGTTTTATAGCACTAGTATATATAGTTTTAAGTCTTAAACANTTCATTTACTCCATTATTCTCCAACACCCAAATCAAATCAAAAAGTTCCTCCTCTCAAAAATTCTCTCTCTAGAACTCGAAGAAGAAGAAGAA

>rnd-1_family-325#LTR/Gypsy ( RepeatScout Family Size = 256 Final Multiple Alignment Size (possibly truncated) = 98 Localized to 2361 out of 4218 contigs )

AAAAAAAAAAAAAAAATGATTGAAGCATTCTTGAGATGATGAGAATTACTTGTGAAATAATTGTTGAAGAGAGGGCTGAAAATAAAGAGAAGAAAATAATGGGTGATGAAGTCTAAAATTGCAAAGTGCTTAAGGAAGTGTAAGTCACTATTATATAGTTTTCCTACCCGTCCCCTAGCCTACATTACAACCCGTTAAAGTCCTATTTGATTCTATTCGAGCATGCTTAATTAGTGGAGATGTACATAATGGGCAAGCNTATGGTTCTTTGTGCATACATGTGAATTTTNTTTGTGAGTGTGAGAGTGTTCTTTGATGCGAAGTCCTTAATTTATATTCAATCTTTTGAATTGAGTGTGTGGACTATTTCTTCTTGTGAGGGCACTTGTTTCATGATAGATAGGTGATTTTATTAACTTTCTTTGATGAGAGTAGGTGAGCAAGCTTAAATTTGATGAGTTCAAAGTCCTTCCTTGAGGTTAGGAGGTTAGAGGTTTGTTGTTTATTTGGATGTCTTGCATAATGATTGAGAAGTGTACTTGATGTTTTGAAATTGGTATATGGCCTAATTGTTTGTATTAACCAAAGTGATGACATTCCTAGTNTGATTTTTTGAGATGAGCTTTAATGTTTGCTCGAGGACGAACAAAAGTTTAAGTGTGGGGTGTTGATGTGAGGCCAAAAATACATATTTTTAATCATTATTTGCCTCACATTTATTATTTATATTTGTCCTTTTTGAGCATGAATTTTATGGATTGTGCTAAATAGTGTATTTTATTTGTAGGATTAAATTGGTGGAAAAATGAAGAAGTTTGGAGCTAAAACGAATTAAAGATGGAAGGTTGAAGAAGGAAATCAAGCAGACAGCTTAATGGATTAAATTNAATAAAATAATATATTTATATATAAAA

>rnd-1_family-448#Unknown ( RepeatScout Family Size = 152 Final Multiple Alignment Size (possibly truncated) = 98 Localized to 2361 out of 4218 contigs )

AAAAAAAATATCTTGTTGAGTTGTCAAAAATTGAAGCACATGGTCATATTCATTTCTCTTTCACTTAGAACCTTTCAGCCCCCAAGGCCTAGCTCGAGTGGCAAAAGGTGGAGGATTTGTGGCTTAGGTCGCAGGTTCAAGCCCCGCACCATGCAAAGCGAAGCCCGGTATTTAAGTGGAGAAGGGTAGAGGGGCGGGCCCATTATCCACCGAGTTTAGAAGGCTGTGATTGGTCCAAAGGGCGGGTCACAGACGGATTTCTCGGTTATCAAAAAAAAAATATCTTGTTGAGTTGTCAAAAATTGAAGCACATGGTCATATTCATTTCTCTTTCACTTAGAACCTTTCAGCCCCCAA

>rnd-1_family-356#DNA/hAT-Ac ( RepeatScout Family Size = 226 Final Multiple Alignment Size (possibly truncated) = 98 Localized to 2361 out of 4218 contigs )

CCAGATACATGTATCTCGGGATACATGGGGTCAAAATTAGGTGTAATTTGTTCTAGATACACTGTATCCAAGTGGATTCGCATGTATCTGGGATACATAACAAATCTCGCCCCTCGCCTCCCTCCCATCTCGCTCGCCACTCTCCTATCTCGCTCGCGTATCTGGTATCCCAGATACATGCGAATCACACTAGATTTTTTGCTAGTGTTATTGAAACAACACCATTGTTTGCCATATTTGATTTGAAAATCTTCTCATTTTCTTGATGTTATAGAATTNTAAGTTTGAAAAGAAAAAAAAA

>rnd-1_family-289#DNA/hAT-Ac ( RepeatScout Family Size = 300 Final Multiple Alignment Size (possibly truncated) = 98 Localized to 2361 out of 4218 contigs )

TAGAGCCGTCAAAATGGGCTTAGCCCATGGGGCCGGCCCGACCCAACCCGTTATTGGGGTAGGGTTGGGATAGGATTTTTCAAGCCCATTTAAAACTAGGGCTTATAAGCCCGGCCCATATAAGCCCTTGGCCCTTGAGGCTTGATCGGGGCCGGGCCGGGGTCGGCCCATGGGCCAAAACTNTTTATTTAAGGGTAACTTACAANAATCTCACTAGTNTATGAGCTAATTACTTAGATACACGCTATGTTGTAATATTACGAATCTTACCAGATTTTGGTACGTCCAGATACATGTATCTCGGGATACATGGANTCAAAATTAGGTGTAATTTGTTCTAGATACACTGTATCCAAGTGGATTCGCATGTATCTGGGATACATAGACAAATCTCGCCCCTCGCCTCCCTC

>rnd-1_family-543#Unknown ( RepeatScout Family Size = 112 Final Multiple Alignment Size (possibly truncated) = 98 Localized to 2361 out of 4218 contigs )

TACTTAGCCCTAGTTCATCATCTAAGGCTTCTTTCTAGGGTTTAATCATATTTCGTTCGGTGCACGAAGGCCTACGCATAATCCTACCGTCGATTTACATATATTACGCAGTGAGCACGGATTTATAACTACTCAATTNATAAAACCTAGCCTACCTGGGCGCCAAGCAGCTGCACGGAGTTNTAGATAATTTTCTGGTTTCCGGGCTNCGTGACGGTGAATTTGGCCGGGAATCCGCAAGCTATCGCCATCCTCACGCTAGAAATCTCCTTCTCCTTCCTTCCCAAGCTTAGAGCAGCTTTTTGAGGGTTTCTAGGGTGNTTTACGNCTNATTTAGGTATTTTAGGAAGAAGGAGAAAATAAGATTTCGCGTNATTTACGTTCTGCCTCGCCTATCCCGCTCTGGCGCCAACTTACCCCGCCCTGGCGCCGCCGCCCTAGCGGCATCTGGCCCGCTCTGGCGGGACGGTGGCCCAGAATTTTTCCCGAATTTTTGCTTTTCCTAAATAACGACGGTTCGGGTCGTTACA

>rnd-1_family-266#LTR/Gypsy ( RepeatScout Family Size = 335 Final Multiple Alignment Size (possibly truncated) = 98 Localized to 2361 out of 4218 contigs )

TTCTCTCTCTAAAACCTCCATTGAAGAAGAAGAAGAAGTTGAAGCTAGGGTTCAAGAACTCAGCTTTTCTACTCAAATTCGTGAGGAAATCTTCAACTAAGGTATGGTAGTCTTCATCCNTGGNTAGCTTTCACCCANGGAGCCCTCAAANTNTGATTTCAAAGTTNGAAATTCCCCAAAACCTAGGGTTTTCANTCTAAGTCATGGGTTCTTCCTAAAAACGATTTCAATGGTTTAATTATGTTATATTATGATTGAATTGATGATTTATGGT

>rnd-1_family-226#Unknown ( RepeatScout Family Size = 417 Final Multiple Alignment Size (possibly truncated) = 98 Localized to 2361 out of 4218 contigs )

TTTTTAATACGGAAAAGGGTCAAAATTGCCCCTGAACTATGCGAAATAGACCACTTATACCCTTCGTTACATTTTGGGATCAAAAATGCCCCCGCCGTTATCCTAAGAGACCACAAATACCCTCAAGAGTTAACACCCCAAATTTTTAGTGACGTGGCAAGCCACGTGGGACTAATCCCTCCACCTAAGCGTTGCCAACTAGGATTCACTACAAAAGAACTNGACCTTTAGCGGCGACAAAGTCGCCACAAAATCCCAAAATATCGCCACTAAAGGTTTTTAACATTAAATTCTAATTTTGTGTTTTTTTCTTCATTGTTTTTAAAAAACCAAAAATGATATCGATTAAGTAGGAGTTTGAAGACACTGTATGTTTTATTTTTATGTCATATGTAAATGTATAAAATGTACAATGATGCATTATATAGTAGGAGATTTGAATCGAGAAGTAATTTTGATGATTTTTCGTAATAATATTGGATGTTTTTAATATTGATATTGCAAGTTATTTATTTTAGAAAATTAGGTCGCAACCGAAAATTAATTATCATATTTTTTTGTTGAAAAAATAGGTTTTACTAGCGAATGGTTGTTGGAGCCTTAGTCGCCACTAAAAATCACTCATAACCTTTAGTGGCGATATTTTGGGATTTTGTGGCGACTTTGTCGCCGCTAAAGGTCNAGTTCTTTTGTAGTGAATCCTAGTTGGCAACGCTTAGGTGGAGGGATTAGTCCCACGTGGCTTGCCACGTCACTAAAAATTTGGGGTGTTAACTCTTGAGGGTATTTGTGGTCTCTTAGGATAACGGCGGGGGTATTTTTGATCCCAAAATGTAACGAAGGGTATAAGTGGTCTATTTCGCATAGTTCAGGGGCAATTTTGACCCTTTTCCGT

>rnd-1_family-130#LTR/Gypsy ( RepeatScout Family Size = 640 Final Multiple Alignment Size (possibly truncated) = 98 Localized to 2361 out of 4218 contigs )

TGGATCGGGTGTCACGTTCCGACACACTANATTGGATCGGGTGCCACGTTCCGACGCATATATTGGATCGGGTGTCACGTTCCGACACACTAATTGGATCGGGTGCCACGTTCCGGCACACTAACAGTTTGGGTGTGGGTTCCATGAGAGGACCATTGACTTGTCATATCTACNTGTCATTGANAATGTGGAATTGTACGTTGCTCCTGAAATGATAATTGATATTGTATATGTTCGGTATATGCGTGTTTACTGTGTTGTAATTGCTTGCGTATGTTNCGCTTACTGGGATAGCCGCGTGATC

>rnd-1_family-137#LTR/Gypsy ( RepeatScout Family Size = 616 Final Multiple Alignment Size (possibly truncated) = 98 Localized to 2361 out of 4218 contigs )

TACGGCNATGCTCGTCGATGACATGGACATCTCTCGTCTTATGGTNCATGCTCAACAAATCGAGGAGGAGAAACTCAAGAGAAAGGTCTAGGGAGGCAAAGAGGGCTAGGACCGGTGATGGTAACTTCTCNCATGCNAGGTCCGATGGACATGGTCGTCCTAGGTTTCGACAAAGGTTTTCCGGNCAAGGTTCCTCCAATGCTCCTATTCACAAAGATGAGAGGGTGTCTAACCCTAAGCCTCAAGGAGGNAGTAGTGGGTCCCTATGGCCTACTTGCGCTAAGTGTGGAAGGAAGCATGAGGGTAAGTGTCTAGCCGGCACGGATGGTTGCTTTGGTTGTGGNAAGAGTGGCCACAAGATGAGGGATTGCCCGATGCTTAAGGCTAAGGGAAGAGAGGGTAAGCAAGCTGCCTCTAGTGGTTCGGATNCGGATGCTCCAAAGNAAGAATAGGTTTTATGCTCTCCAATCTAGAGATGATCAAGAGNGNTCTCCNNATGTCGCTACCGGTATGTTTTATGCTTTAGTAGATTTCCGGATTTCTTGTCTTTTGTGACTTGNTATGTTGGACTTTGAAGTTGTCTTTAGTATGAATGATGTTTAGAAGAGTTTGTGTAGGCTTACTCCCAAGGGGGAGATAGTGTGCCTATCTAGTAAGGTTATTGTGTGTTTATGTGTTGCCATGATTTCCTTATATGCATGTTCATGAAAATGCTTAGGTCTTGTTAGAAATGACTTATATGCATTGTTTTCCTCATGTTGTA

>rnd-1_family-63#LTR/Gypsy ( RepeatScout Family Size = 978 Final Multiple Alignment Size (possibly truncated) = 98 Localized to 2361 out of 4218 contigs )

GCATACTCGTACATTCAATGTACTGATGCCAGTTGGCCTGCATCTTATTATGATGCAGACGCAGGTAACCAGGATCAGCATCCAGCGCGCCGTTGATCCAGTTGAGCACTCCAGAGTCAGTGGTGAGCCTCCTTGCNTTCCGGAGGACTCTTTTATTTCGCTTTCCTAGTTTTGNTTTATTAGGATGTCGCGGGGTCTGTCCCGACATCCATCTCAGTATTTTAGAGGCTTCATAGACAGTCAGNCAGTTAGTTTTGAGTCTCTCATCTATGTNTTGCANATATTCTGTTTTGAGACTCGAGTTGCCNTTTTGGCCAAGATGTTATCATTTAAGTTATTNCATGAGTTTATTTCGTTGAGTTAAGTCTTCCGCTGAGTTAAGTAAGCCAGGCCAAGGGTTCGCTTGGGGCCAGCAATGGTCTTCGAGTGCCGGTCCCGCCCAGGGTGTAGGCTCGGGGCGTGACA

>rnd-1_family-89#Unknown ( RepeatScout Family Size = 800 Final Multiple Alignment Size (possibly truncated) = 98 Localized to 2361 out of 4218 contigs )

TTTCTTCCAATGTTGTGGGTTATGATGATTGTTGATCCAATTGAGTGGATTATGATTGTTTATGATNGAATTGATAGAATTACATGTTTTTAGGGTGAAATTACATGTACCCATGCCTATCCTATGATTNTAANCTAGAATTGATATAATTGGGTTGATGCCATGAAGGGAAACTTTATGGGTTTAGATNTATCCTTATAGGATTGATGAATTATGATTAGAATTGCTTGAGANTGAAGCATANATGATGATCATGAGATGTAGTGCTTTGAGAGTAAAGCTTATGATATGAATTGTTGATTATTGATGTTATTGTTGTGGAAGGGATACTTCCCACATGAATTGCTTAATTATGAATGTATGAATGTGAAAGGTTTTCTCACATGAATGATTCTAGGTTGAAAGGATTGCTCACCTAAAATGAATCTAGACTAAAAGACTATGAAATTGATGAGGCTAGAGGCGATTACCCTTGACCTCCATATGANAAGGATATGAGGCGATTACCCATACTCCTTAGAATTGAACAAGAGGCGANTCCCCACGTTCCTATTATGATGAATGGACATGGAGGCGAGTACCCACGNTCCTTATGAAATGACGTGGAGGCGAGTACCCGCGTCATATGATGAGATTGTGGAGGCGAGTACCCGCGATCTNAAAGTAAGTAAGGCAAGAGGCGAATACCCATTAGCCTTTATAAGTTAGGCAAAGAGGCGAATCCCCATGTCTTTAAAAGTAAGTATGAACTAAGTGAATTATGTTATGAAGAATGANTAGCTTGGATTGTTGCTNGATATGCCTTCCATGAGGTAAGCTTGACTAGAGGCGGATGACCCAAGTCTTGGCTAAGGATGAGTGAGGTTACTTAAGAAGTATTCCTTTCTTATTATGATGCTGATTTATGTGCATTGATTATGCTTATGNCTTATGTTGACTAACNCTTATGTTTATGTTGTTTATGCTAGCTATCATATTTAGTACATNNTTGTACTAACGCATACTCTTGCCTACATTCTTATCAAATGTAGGGTCCGGCGATATCGACTCTCATCCTCGTGGCTAGGTTCNTAGTAGATCAAGGAAGTGAAGATTTGGTGAGTCCTCATNGCATTCGAGGATTTGACCACCCATTTCTCTTATGTCTTTCTTTTATGTTTTGGACTATTGTATGGGCTGCGTCCCAAGATT

>rnd-1_family-132#LTR/Gypsy ( RepeatScout Family Size = 621 Final Multiple Alignment Size (possibly truncated) = 98 Localized to 2361 out of 4218 contigs )

CAAGTGAAGACCACGGGCGACTTCACGGGCCGTGGTCTTCACGACGGNCCGTGGTGGCGNTCGTGAAGTTGAGCCAGTGNTGGGCCTCGAGAAGCTGAAGAGCGGGATTGACCAAGTGAAGACCACGGACGACTTCACGGGCCGTGGTCCTCANNACGGNCCGTGGTGCGTGCCGTGAAGNCGANCCAGTGNGNAGGCNNCGAAGAGCCGCAAGACGTCGAAGCCCAAGTGAAGACCACGGGCGACTTCACGGNCCGTGGTCCTCGNCACGGGCCGTGGTAGCGCTCGTGGTGCATTTTCCAGTGNNCAGGCNTTGAAGACCCACTTTCGGGAAGTCCAAGTGAAGACCACGGAGGGCTTCACGGACCGTGAAGCCCTTGACGGGCCGTGGTAGCGGCCGTGAAAGCGGCCCGACAANTCAGTCCTACTTCGAGTAGGACTCNTTTTAAACATTCTATTTGAATTANTTTAGGTCGTTTTGATTATTATTCATTATCTTATATTGGACGACGAAGAACNGAGTTTCGATTATTTTTAGCTTTCGGTTTTTGAATCATCGATTATTGTTATCAGTTGGAAATTCGGATTCAGACTNTCGGATTATCAAAGTGATTGTTGATTTGCTAATTATTTTCGTAAGTCTTTCTCAATATGTTTTCATTCCAAATTNCTGNTCGTTTACGTGAAANCATGAGTGGCTAAACACCACGACTGGGGTTGTGGGATCTATGACGAAAATCTAAGTGCGATTTCGTAAAACGAGTAAAAGGCGGTTTGTGTTTGCAANNTGTAGTTCTTAGCTTTCGTTATCTTATTCTAATGAGTGCACGCATTAGACGAGCCTGTATCTGTCATTTGTTCGAGAGAAAAATANCAGTTAGGAAAAAGCTGAATTAAATTAACGAATTGGAGTTAAAGCTCGAGTTCGAGAGAAAAGANCTTTAACACCANATCATTCAAGCGAGGGTCAANGTTAGTAACACTCTAAGACCGAGAGGACNTGAGTGANAGTTATCTGATTAGGCTGAGAAGCGTTAGATANATTTAACTCGCAAGGATTATGTAAACANTGANTCGCCTGANACTCAAATTACGGGAGAACACTAGAACTAACGTCATGGGGAACACAATCCTGGTTTCNTTTAATTAATCGAATCAACCTTGNAACGNAATAGTCTGTTAGTCTTGAGCTTGGATTTTGACTGCAAGTTTTGGTTACTAGAACAATTCAACCAAACCCAATTTACTTTCCGCAATTATTTCTTTAGGAAATAATAACGACANTTGAATAAACGCGACAGTGAAACCATTTCTGTAAAGAAATTCTCTGTGGGATCGACCTCAACTCNCGTTGAGTTCTGTAAATTGACTTCGACCGTTTATACTTCTTAATTGAAGTGTAAGTTTGGACGTATCA

>rnd-1_family-333#LTR/Copia ( RepeatScout Family Size = 248 Final Multiple Alignment Size (possibly truncated) = 98 Localized to 2361 out of 4218 contigs )

TGTTAGAGTGGGTAAAAGTCCCACATCGGTTGGGGAATGAGACGGGTGGTCTCCTTATATGGACTTGGGCAATCCTCCCCTCGTGAGCTAGCTTTTGGGGTTGAGTTAGGCCCANGTGTCATATCTTTACA

>rnd-1_family-475#Unknown ( RepeatScout Family Size = 137 Final Multiple Alignment Size (possibly truncated) = 98 Localized to 2361 out of 4218 contigs )

AAAAAATTGTAGAAGCAAGGGGTGAGTACCAAACCACACGGTACTCAGCAAGTAAACCTCTAAACACAAGCTAAGGGGATAGAATACGGGTACTCCTTACACCCCAACCGAACCTCCACAACTACAACCTGCATAAAACCAGCCCAACCTAACAGTTCACAATTTACATAGCACACAGCTCAACAACAACACTCTAACAATTACATATCCTCAACAACAAGCTCAATATTCATCAATCACAAGTTCCACAAAAAGGCACTCACAAGATCAGCAACACACAAGATCAAGTTCACAATTTA

>rnd-1_family-549#Unknown ( RepeatScout Family Size = 110 Final Multiple Alignment Size (possibly truncated) = 98 Localized to 2361 out of 4218 contigs )

GAGATAAGTGTCAAAAACACACCTAAACTATCTTTTTTTTTAGTTTCATACCTAAACTATTGAGAGTGTGAGTTTCATACCTAAACTATCACTTATTAGTTTGAGAAACACACCTCTCTCTTTTATTACACTCTCATCATGGTGTGTGTAATACACTCTCTCTTTTATTTAAAAAATTGTCACATCACACTCCACATGGACAAAACATTCCACCTTGACAAAAATTAAATAAACTATTAGTATTAGTTAAAAATTAAAGATTAAA

>rnd-1_family-368#LTR/Copia ( RepeatScout Family Size = 218 Final Multiple Alignment Size (possibly truncated) = 98 Localized to 2361 out of 4218 contigs )

TGATGAAGCTGAATCTGATGCTGACGCGGAAGAAGAGATNACCAGAAGTCCGGATGGAAACATCAATCNTCTCATCCNCTGGACAATCTCATTTCCCCACTTGACTCTGGAATACAAACCAGATCCAAAACAAGAAATCTAGTTGCATATTCAGCATTCATATCATCCATTGAGCCAAAGAACGTCAAAGAAGCCTTACAGGATGCGGATTGGGTNACNNCAATGCAAGAGGAACTNCATCAGTTCGAGAGAAGCGAAGTNTGGNACCTGGTCCCCAGACCTNCCGACAGAACCGTCATTGGNNCNAAATGGGTNTTCAAAANCAAACTNAANGCNGATGGNACNATCGACAGATACAAGGCNAGACTNGTNGTNAAGGGNTACANNCAAGAGGAAGGNATTGATTACGATGAGACNTTTTCTCCNGTNGCNAGGATGGANACNATCAGAATNNTNNTAGCNTTNGCNGCTCACATGGAATTTAAGCTNTATCAAATGGATGTCAAAANTGCCTTCCTNAATGGNGATCTGGAGGAAGAAGTNTACGTGGAACAACCNCNAGGTTTTGAAGATGCAGAACTGCCAAATTATGTGCTAAATTGAANAAGNCNCTNTATGGNCTAAAACAAGCTCCNAGAGCNTGGTATGAAGCTTGNCAAAATTCCTTCTTGCAAATGGNTTCAAAAGAGGTAAAATTGACAATACCTTGTTCTTAAAATCCGAGGTAAGGAGTCGTTGATCGTNCGGGTNTATGTNGATGACATCATCTTTGGAGCCACTTCAGANTCGTTGTGCGAAGAGTTTGCCGATTTGATGAGTAGTGAGTTCGAAATGAGCATGATGGGGGAGCTCACCTTCTT

>rnd-1_family-365#LTR/Gypsy ( RepeatScout Family Size = 221 Final Multiple Alignment Size (possibly truncated) = 98 Localized to 2361 out of 4218 contigs )

TTCTCACTTACACACTTAGGCATAAATATGCTAGGAGATGAAAGGTTTTCTCACATAGAATGATTCTAGGTTGAAAGGCTTGCTCACCTAAAATGAATCAAAGCATGTTAGACTAGGATAGCTTGAAGTNGTGACATANGATCCCTTCATGNATAGCTTGACTTGGTAAGACAAGACCATGATTGGTAGCTTAGGATTGAGGATCTATACTCTCCTAAGGGTAGCTTGACTTAGCATCGTAGGATGCATTTCAANGGTAGCTTGAGTTAGGAGTAGTCATGGTAGCCTAGGATTGAGGACTCATACTCTCCTAAGGGTAGC

>rnd-1_family-438#LTR/Gypsy ( RepeatScout Family Size = 160 Final Multiple Alignment Size (possibly truncated) = 98 Localized to 2361 out of 4218 contigs )

AATTATACNGTGACCGAACAAGAGCTNCTTGCGGTGGTNTNCGCATTCGAGAAATTTNGATCNTACTTGCTNGGTACTAAGGTNATNGTGCATACTGACCATGCTGCATTGAGGTATTTGATGGCGAAGAAGGATGCAAAACCGAGGTTGATTAGATGGGTGTTGCTGCTGCAAGAGTTTGATTTTGAGGTNAAAGATAGAAAGGGNACCGAAAATCAAGTNGCCGATCACTTGTCCAGGTTAGAGGAAGAGGCTATGCTAAAGCTCGGAGATAGGGCTGAAATTAATGATGCNTTTCCNGATGAACAGGTATTGGCTGCTTCTCATGATCTGATTCCTTGGTNCGCAGACTTTGCTAATTATTTGGCGAGCGATATTGTGCCNCCGGATTTGACNTTTCACCAAAGGAAAAGGTTCATGCATGATGTGAAGAAGTNCTTTTGGGATGAACCNTACTTGTTTCGTAGTTGTGCTGACCGGATNATTCGNCGNTGTGTGCCCGAGGTTGAGATGNTGAGTATNCTCGAGGCNTGTCATTCATCGCCCGTTGGTGGGCATCATGGTGGTATTCGNACTGCNCATAAGATNCTNCAGTGTGGNTACTACTGGCCTACCATCCACCAAGATGCTCATGATTTCGCNAAGNNTTGTGATCGTTGCCAAAGAGAAGGAGGGATTTCGAGGAGGCANGAGCTNCCTNTGAATCCTATNTTGGAGGTNGAGTTGTTTGATGTNTGGGGNATCGATTTCATGGGTCCATT

>rnd-1_family-286#Unknown ( RepeatScout Family Size = 305 Final Multiple Alignment Size (possibly truncated) = 98 Localized to 2361 out of 4218 contigs )

TACTCCCTCCGTCCCAATTTATGTGACACTTTTCGGATTTCGAGATTCAAACAAGTCTATCTTTGACCGTAAATTTTTCATATATCTTTTAAATATTTTGAATTGTCAATTATTGTGACTTATAGTACTTTTTACGTAGTTTACAAATATATAAATTTCATTTCAAAAAATTGAAGATTTCATGCGCAAATTTCCGGTCAAACTTAAACTGTTTGACTCTCGAAAAACGAAAAGTGTCACATAAATTGGGACAGAGGGAGTA

>rnd-1_family-423#Unknown ( RepeatScout Family Size = 167 Final Multiple Alignment Size (possibly truncated) = 98 Localized to 2361 out of 4218 contigs )

GGCTTGGATTGACTATCTCCAAGGCTTGTGGTGAGTCCTCATGGTTCGAGGACGAGATGTTATTCATTTCTAG

>rnd-1_family-511#DNA/CMC-EnSpm ( RepeatScout Family Size = 126 Final Multiple Alignment Size (possibly truncated) = 98 Localized to 2361 out of 4218 contigs )

TTGACTTTGGTCAACATTCTTGGTAAACGCGCTCGGATGAGAATTCCGTCAGCGCGGTTAGCTCCGGAATGTCGAGTTTGGTCTAGAACGACCCTT

>rnd-1_family-344#LTR/Gypsy ( RepeatScout Family Size = 238 Final Multiple Alignment Size (possibly truncated) = 98 Localized to 2361 out of 4218 contigs )

TGAGGGTGAGCACCAGGANCCTCAGGCTGCANTTTCTGAGCCTGAGGATGACGAGCCGCTAGCAGCNCGGAGGGCNGAGCTGCGATCCAAGAGGNTGAATGATCCGTCTAGGATCAGGACTCCCCAGNCCACCACTACTCCTCCTCCAGCTCCAGCGCAGGCNGTGGTCCCGGCACCACCGGTACAGGGTCCTCCTCCCCGGTCTATGAACAGACTAAAGGCCGAGGGGTTGAGGACNATCNTNGAGGAGAAGCGGTTGTCCACGGATGGNGTGGTGGACAGGTACCCAGAGATGTGGGACGCCTNAAGTNCCACAAGTTCGAGATNTTCACCAGGCCCCGTGGCCCNTACATTCCNACNTGGGTCCGGGAGTTCTACGCTGCNTACGGNGCCTTGGTACCNCAGGGGAAGAAGAAGGCAGCTGCNTTCAAACCGGTNGACTACGTGGTNGTCCGGGGNAAGAAGGTGAAGTGCGACAGCGATGATATCAACGCNGTNTTGGGATGTCCCGACGATATTGANGACGACTGCCAGNACATGATCAGGACGAAGACNCTGGATGACNTGAAGGGNTGGTTGGCCCCNCTNATTTCTGACGNCACCCCGAGGTGGATNGAGGCGGGAGCNCCNATCGAGAAGAAGGACCTGAACGTAGCNGCNAGGTACTGGTTCGGNTTCATCAGCAGCACNATCATGCCGTCCCAGAACGAGTCNATCCTCCGCCACGCNAAGGCGGCCTGTCTNGGTTGCATCATNGACGGGACGAGGNTNAACTTGGGGNTGATCATTGNGCAGGAGATGGCCATGAGGGCCAAGCAGCGCCAGACNTCCCTTCCNTTCCCGGTGTTGATCACGGAGCTGTGCAGACGNGCTCGGGTNCCTCGAGATGCGAAGAAGGATGTGGAGGTGACTCCCACATCCTCTACCGACATCCGGCGNATCGAGGCCGAGTACNCGAAGGATGAGGCNGANAGGAAGAGGGCAGCCCCGGTGGACACNTCCCCGGNGGTTGACGTTGANNCGNTACCTGCAGAGGCANCTTTGCCTACTCCGGCCNCCGGGCCTTCAGGTACNTCTAGTNCCGCTCCTTCTGANACTCCNAGTTCTTCTGCTGCTCCCCTGCCTCCTAGATCTGCTGCT

>rnd-1_family-369#Unknown ( RepeatScout Family Size = 218 Final Multiple Alignment Size (possibly truncated) = 98 Localized to 2361 out of 4218 contigs )

TACTCCCTCCGTCCAACAATAGTTGTCCACTATACTAAAAATAGATGTCCAACAATACTTGTCCACTTTATGAAATCAATGGATAATTTTACACTTAGTTCCTAATTTACCCTTATCATTAATTATAGTCATTTCCCTATTACATTTTTCAAGACATTGTATTTATTATATTCAAAGGGTGATATAGTAAAATTACCCTTCTATTTATAGTTTCTTAAGAAGTGTGCNAAGTCAATAGTGGACAANTATTGTTGGACAGAGGGAGTAA

>rnd-1_family-458#LINE/L1 ( RepeatScout Family Size = 145 Final Multiple Alignment Size (possibly truncated) = 98 Localized to 2361 out of 4218 contigs )

TGTGGAGGTACTCNAATGAAGATCAAGCACTGTGGAGAAGAGTGATNAGNGCNAAATATGAGGAGGAGGATAGTTGGATGACCAAGGAGGTNACTACACCTTATGGNGTCGGTNTATGGAGATCCATCAGAANNTTATGGNATGAAGTCAAACTCAACTCCAAGATCAAAGTGGGNAATGGGAACAAAACNANNTTCTGGAAGGACGAATGGCATGAGNCAGGTAATCTGGAAGANCTNTTTCCNGACATNTNTAACCTAGTTCTCTCACAACAAAGGACNATNGCNGAAATGTGGACANCACAAGGNTGGAATNTNANCTTCAGAAGACATCTNAATGACTGGGAGGTNGNNAGAGTGGCNGAATTTCTCAACATCGTTGGAAACTTCAACGGACTNCAGGCTGANGAGGACGCATTATGGTGGAANGGNAACAGCAAAGGCGTNTTCAAAGTAAGTNCAGCTTACAGGNTGATGGANCAACCAAGTCAACAGATTNCTAACTGGCCNTGGAAGCANATNTGGAAAGTCAAGATNCCNCACAAGGTGGCNTGTTTCGCNTGGCTGNTGGCTAAGGAAGCAGCTCTGACNCAGGANAATCTNACGAAAAGGGGGATAACCTTGTGCTCNAGGTGCTTTTTNTGCGGAGAAACAGCGGAGACAGTCAACCATCTNTTTCTNCACTGCAAANTNACTGNNCAGCTGTGGAANATNTTCNTCAGTCTCAAAGGCATATCCTGGACNATGCCTAGGAGNATTACTGAGGCCCTANNNAGCTGGGAGGAGGCGGGAGTGCGAGCAAAAGACAGAAGAGATGGAGGATCGTCCCNGCTTGCATNTGGTGGACAATNTGGAAGGAGAGGAATNNNAGATGTTTTGAGAGCATAGAGAATAGTATGCAGAAAGTTAAANTGAACTGCATTTTGTTNTTATGTTTTTGGTGTAANCAANTATACTCNAATGANACTGTCTCTATNATTGATGTTTTAGACTCAATTTAGA

>rnd-1_family-144#LTR/Gypsy ( RepeatScout Family Size = 597 Final Multiple Alignment Size (possibly truncated) = 98 Localized to 2361 out of 4218 contigs )

ACGGACGCCTAGACGGCCCGTGGNGCTTCGCACGGNCCGTGAAGTGGTCCGTGCAAGTCACCCGTCAGATTTTGAGTTTTGGGTCAACTTCAAACGATCATATCTTTTAGCACAAAATGAATTAGGTGGCCCATGACCTATCAAATTAAAGGTCTTTGAGTCCTCTTTCCAACGCCACCGAGTTTGCCTAATTCCGAGCTCGGAGTAAAAAGTTATGCCCGTTTTAGTGAAGCC

>rnd-1_family-540#Unknown ( RepeatScout Family Size = 115 Final Multiple Alignment Size (possibly truncated) = 98 Localized to 2361 out of 4218 contigs )

TTTTAAAANTAAAATATTGTATTCACTCGCTTTNAAGCGCGTGTAATCACGCATTTTTCCAACTCAGCAATNTTAATGCCACATAAGCTCGGTCAATGGTCAGAGGGGTTTAAAATATTGTGTTTTANTGAGTTTAAGGGTTCAGTTGACAAAGTGATAAGTAGAAGGGTCCAGANTACAAACGGANNCAAGTACA

>rnd-1_family-256#LTR/Gypsy ( RepeatScout Family Size = 348 Final Multiple Alignment Size (possibly truncated) = 98 Localized to 2361 out of 4218 contigs )

TTTGAGTATCGGTTGTGATCAATACCGATGACTTGAATAGTGCTCATGATCGAACGAAATGAATGTGCGGCTACGATGAGGCCAAATGAAGTTGCATAGGCAATATGTGAACTTTTTGCATCTCGTTGTGACTAGCCGATTATCGAAGTGAGTCTCTTGCGATGAACATCGCACGCTAGCGAAAGTGTGGAGTCTTGTTTGATATTGGTGTCAATGCCTTGTGTGGTGAGCTTACCTTGAACCATGCGTGATGACACCTAGAATTTGCCCCGTTGGTCCGGTTGACTAAGTGATGCAANCTCTTGAAATGATCTTAGGCAACAATTTTGAAGAGTGAACCAATTTGACATTATACCTCTTTTGTGGCCTACCTTGTGAGTGTGTGAACCTCGCTTGAGCACCNTTTGAGCCTTAACCCTTTTCTTGGAAGAAACTTGATGCAATTGTGACCTTTCTTTATCCATTCACCCATAATCCTCATGAAGCGTGGTTTGTTTGAATAGCCAACTTAGGCCAAAAGCCTAAGTTGGGGGTGTGGTGAAAAGAGAAGGNAAATCAAGAAGTGCAAAGAA

>rnd-1_family-268#LTR/Gypsy ( RepeatScout Family Size = 332 Final Multiple Alignment Size (possibly truncated) = 98 Localized to 2361 out of 4218 contigs )

TAAGGGTGGCAGCTCAGGGGCGAAAGCCTAGCATGGGCCGATCCCAATTGGTATAGAAGGGTGGCAGCTCAGGGGCGAAAGCCTAGCATGGGCCGATCCCAATTTGTGTAATTATGAGGACGGCATCCCAGGGGTTAAAATGCCTAGCATGGGTCATCCTCTTCTACCACTGATCAGCTGGTCATTCGTATCACATGCCTTATAAAGATCATAACGCACAGAAAAGTAAAGAAAAGAATGTAACATACACGATCCCACAAGAGTNAACAAAGGTGGTCTTCTATTCTGATCTATGCACCATATATTGATACTTGGTTTCACTCGAGCTCTTATTTTACATATGTTATTGCCTTACATATTCAGTACATTCTTTCGTACTGACGTCCCTCGCGGGGGACGCTGCATTTCATGCTGCAGGCACANGTACTCCAGCTAGTAGACCTCCCCAGTAGGAGCACACGACACTCAGCTGCTNTTGGTGAGCTCCAGGTTGATTCGGGGCTTTACCGAGTCCTTGGTAGATTCATTTTGGTATTGTATCGTAGTTAAGGGTAAGGCGGGGTCTGTCCCGACCTACTCTAAGGTTTTCTATCTTTTAGAGGCTTTGTAGACTTATGTATATGGTTCGGTTGCGTCTTAACAAGTTGTGGCCTCAACGGCCAAGTCTTGTATATAAGTTTTGGGTCTACTTATGTCGGTTCGTATCGCTGCATCCTATGTGAACTTGTCACAAGATTGTCATAGTTATATGCATAGTAAGCGCAGGTTACATGTTGGTTCTCCCGGGCCNTTNAGGCATCGGGTGCCTGTCCGTCTTAATCGGATCTGAGGCGTGACA

>rnd-1_family-217#LTR/Gypsy ( RepeatScout Family Size = 435 Final Multiple Alignment Size (possibly truncated) = 98 Localized to 2361 out of 4218 contigs )

ATGACTAATGCGGAGNTTAGGNCGGCTTTTCAAATGTTGGCTCAAGCCGTGACGGCTCAAGCCAATAGGGATGTTGGGCCCCGGTGAACCCTAATGTGAGTACCGCGGCTTCNAGGNTGAGGGACTTCGCGAGGATGAACCCTCCNGNGTTTCNTGGTTCCAAAGTGGANGAAGATCCTCAAGAGTTCGTNGATGAGGTNTATAAGGTANTTGATGCTATGGGGGTGACTTCGGTAGAGAAGGCGGAGCTGGCCGCTTACCAATTGAAGGATGTNGCCCAAGTNTGGTACGNTCAATGGAAGGANAATAGGCCGGTAGGAGCGGGTCCCATAGANTGGGAGGTGTTCAAGNNAGCNTTCCTTGATAGGTTCTTTCCCCGNGAGATGAGGGAGGCNAAGGTGGAAGAGTTCATCAACCTTCGNCAAGGNAGTATGAGTGTGAAGGAGTATNCTTTGAAGTTCACCCAATTGTCCAAGTATGCTCCATCTTTGGTGNCGAATCCTAGGGATGAGATGAGT

>rnd-1_family-169#Unknown ( RepeatScout Family Size = 524 Final Multiple Alignment Size (possibly truncated) = 98 Localized to 2361 out of 4218 contigs )

AGGGAAAAGGGTCTGAAANATATTCGAACTTTGGCCGAAATTGCTGTNACGATACCAAACTTTGGGGAGGACCTTTTACCCCCTGCACTATTTAATAGTGTATTTTAAAGGTATATATGTGCCCACGTGGACATCATNANTATTGCATAATTATGCAATATTNATGATGTCCACGTGGGCACATATATACCTTTAAAATACACTATTAAATAGTGCAGGGGGTAAAAGGTCCTTCCCAAAGTTCGGTATCGTNACAGCAATTTCGGTCAAAGTTCGAATATNTTTCAGACCCTTTTCCCTTTAAA

>rnd-1_family-83#LTR/Gypsy ( RepeatScout Family Size = 830 Final Multiple Alignment Size (possibly truncated) = 98 Localized to 2361 out of 4218 contigs )

TTGAGTTGAGTATCCATGAGTTGAGTNGAGCCGAGGTAAGTGTTTCTTTCAGATTCCTTTCAAGCTTATGTCTTGTTTAGNATTCCCCTCGCATGCTCGTACATTCAATGTACTGACGCCATTTGGCCTGCATCNTTTTATGATGCAGATACAGGTAACCAGGATCAGCATCCAGCGCNTCGTTGATCCAGTTGAGCACTCAGAGTCGTTTGGTGAGCCTCCTTGCTTTCCGGAGGATCCCTTTTTCATGCTTTTAGTATTTCAGTTGTTAGGATGATCGNGGGTCTTGTCCCGACGTCCCTCTTAGTTTTAGAGGCTTCATAGACAGNTAGNTATAGTTCGTNAGTCTTTNCATTTCAGTTGTTTTGTTTGAGACTTGAGTTGCCACTTTGGCCAGTTGAATGTTTAATTTTAAAACATTCTAAGTTATTTTGAGCAGTTGAGTAAAGTTGCATTTGTTTTCTTTATTTAATGNTTAAAGTCTTCCGCTGAGTAAGTAAGCCAGGCCAAGGGTTCGCTTGGGGCCAGCAATGGTTCTCGAGTGCCGGTCNCGCCCAGGGTGTAGGCTCGGGGCGTGACAA

>rnd-1_family-302#Retroposon ( RepeatScout Family Size = 274 Final Multiple Alignment Size (possibly truncated) = 98 Localized to 2361 out of 4218 contigs )

AGAAGAACATAAATTAATTCACAAAACTAAAATCTGTAAAAACATACCAGAATCTGGAAAAACAGAATCAGAAAAAAATCGAGCCCACTGAATGCACAGTGTCCCCTTAAGGAAATTATTCCCCTCTAGTACCCGAGGTTTAATGGAATATATCCTCCCAGGATAGAACGATCTTANTCACCAGTGTATTGATACCCAAAACNATGGTGTCAGCGAGCCACTCAACGGCAGTAAAGTACACGAAGAATATTTGATTGTGCAGAAGAAGAAGAAGAAGTCAGAAATATTCGTAAGGAATAAGTCTGAGGAATCACTNTATTTATAGACAAGAGGAACTGGTTCCGAAAGGTTGCGACCTTTCGGAAACCACACGACCGTTCGTGAAAGTTCGCAACCTTTCAAACGGTCACGGCCGTTTCTGAAAGTCGCAACCTTTCAGAACAGTCACTTCCAACGGCGGGAAATTCAAATAAAACGGGAAAGAATTTAAATAAAACGGGTCGCGCGCGGATCCGAGTCGGGTCGGGTTAATTAACTAANTAATTGAAACGTTTCGGTTAATTAACTAATTAATTGAAACGTTTTGGCCATTTAATTTAATTTAATTAATTAAATAAATAATTA

>rnd-1_family-270#LTR/Gypsy ( RepeatScout Family Size = 330 Final Multiple Alignment Size (possibly truncated) = 98 Localized to 2361 out of 4218 contigs )

CACTCGGCGACTCGCCGAAAGTCTCCCTTGATCGCCCTTTCTGCGCCCCTAACCCCTAAAATGCACTGTAACTTTCGGCGGGCTAGTCCTNGCTCGCCGAATGNTGTCGGCGATTCGCCGAAAGGCCCTTCNCATCGCCGACATGCCATTTTTCTGGGAAATTTTTGAGCCAATCCTTTCGGCGAGCCCGATCTGGCTCGCCAAAGTGATTCGGCGACTCGCCGACCGGTTCGGCGAGTCTNTCTGCAACTTATTTTCTGCGTTTTTCTTGAATTGTTCTAACTTTTTTCGATGTGTTTTGCAGATATGGCTAGAACCAA

>rnd-1_family-190#LTR/Gypsy ( RepeatScout Family Size = 485 Final Multiple Alignment Size (possibly truncated) = 98 Localized to 2361 out of 4218 contigs )

GCTTTGTATGCTTGAGTTAGTCTTCCGCTTGTAGTCAGCCAGGATGAGGGTTCGCTTGGGGACCAGCAATGGTTCTCGAGTGCCGGCCACGTCCAGGGTGTAGGCTCGGGTCGTGACA

>rnd-1_family-163#Unknown ( RepeatScout Family Size = 542 Final Multiple Alignment Size (possibly truncated) = 98 Localized to 2361 out of 4218 contigs )

TGATGAGTGGTAGATTTGCACTCATTTGAGGCCTATTTAGTTCAACAATTAGTGTCCTCAATGCCTAATTATGTCCTATTCTAATGATATTTTGATGATTTGCAGCTATGAAGGCTAAAGGACAAGGCAAGGATGAATGTTTGAAAGAATGGCCAAAAACGCGAAAAGAAGTGCAAACTCCACATTGNTGATCGCCTAACAAGAAGGGCGGATCGCCCAANTGGCACGACCTCGCCCAAATTGACAGTGCACAACACTTGAATTAATCGAGGCAGGTCGGCGAACTCAGAGAGGAACGGCGATTCGCCGAATGCGAAAGGCGAACANGATGACCATCGCCGAAAGTGACAGTACTTGGAGAAAAANTGAAGGCTTAAAGGCGAACAAAGGTGGTANTCGGCGATTCGCCGAACGTGAAAGGCGGACTCGANCGAGTTCGCCGACTGAGGTTCAAACACCTGAAGCCTGGAGCAAGAGAAAGGCGAGCTGAAGAGCGANCGGCGATTCGCCGAACGAGTTCGGCGAGCCCGACTAACGTCGCCGAATNGGTCGCGCATTGAATTTTGGGCCAAATTTTGGGAAACTATAAATAGCCCAAAGGAGAGAGAAA

>rnd-1_family-104#LTR/Gypsy ( RepeatScout Family Size = 713 Final Multiple Alignment Size (possibly truncated) = 98 Localized to 2361 out of 4218 contigs )

CTTATACTTNTGTTCATGAAGTTTTACCAAAATGGCATAAAAGCATGACTTTCAAATAAATGTCCTTTTTAGCATGTTTTTGCATGGCTTCCATACTTAGTACTTAATTGTGCTAACCCCTTTCTTTCCCTTTTTACTAAAGTGTAGGGATTTGGAGATGTTGACGTTCCATGGCTTGGATAGGTTTCTAAGTGTCGAAGATGAAGACTTGGTGAGTCCTCATAGATTCGAGGACAATACCCANTATGTTCCTTTATGTCTTTTTTGTATTGTTTGAGTTNTGTACGGGCTNAGTCCCGAATTTTGTACTCTAAAGTATTAGATGGTTTGAGACATCATGTATAAAGTCTAGAAAGTCTTCCGCTTGCTATTTTTAATTAAATGTCTTCGATTGTAAAGAAAGTTTTAAATTCCTTATGGTTTTAGTGTCTATGCGATGAATGAATGCTAAGAGGCTTGTATNAGACCTCTTCGGGGTCGAGTACGCCGTGTTACGACTAGGGGGTGCTCCCGGGTCGTGACA

>rnd-1_family-170#SINE ( RepeatScout Family Size = 523 Final Multiple Alignment Size (possibly truncated) = 98 Localized to 2361 out of 4218 contigs )

GCCTTATGTTGTTTATTGCGTTTCGCTTCTCGCATTATTTTNTTGTTGTTATTGTCTCCTTTGTTGATTNTGCACTATTTTTCTTATTGGTTGTTATGCTTTATATATTTCTCTTCTTTTACTTGGATTTGTTGCACTTGAGCCGAGGGTCTNTCGGAAACAGCCTCTCTACCTCCACGAGGTAGTGGTAAGGTCTGCGTACACTCTACCCTCCCCAGACCCCACTTGTGGGATTNCACTGGGTATGTTGTTGTTGTTGTTG

>rnd-1_family-407#Unknown ( RepeatScout Family Size = 179 Final Multiple Alignment Size (possibly truncated) = 98 Localized to 2361 out of 4218 contigs )

CCAACTCCACTGCGCATCACTTTGTTATTTCAGGGGGTCCAAATTCTATATATACCAATATTAAATCAAAATTTTACNTGTATATATAGTGTAATTTTTCGACGAAGGGTGTCCGGNTGGACACCCTNGAGCCNACGTGGCTCCGCCCCTG

>rnd-1_family-128#LTR/Gypsy ( RepeatScout Family Size = 645 Final Multiple Alignment Size (possibly truncated) = 98 Localized to 2361 out of 4218 contigs )

TGATAATATTGTATATGTTCGGTATATGCATGTTATTATAATGTTGTGGTTACTTGCGTATGTTNCACTTANTGGGATAGCCGCGTGATCCTACCAGTACACTGTGGTTGTGTACTGATACTGCACTTGCTCTTTCTTTGTTGAGTACAGGGCATCTTCAGGCGGCTATTGACAGACCTCAGCTAGGNGACTATTGANCGTGACCGGATTCAAGGGTGAGCCAGTTCTTTCGGGCTGCCATGAGNTCTCTCTTGTTTAAGTCCACTCTTTTCGGACTCAGACTATCTTTAGTTAGTNTTATGTTTAGTTTCGGGGTTGTACCCCTTNTTCTTAGACTTGTCGATTAGTAGAGTTTTGGTACANTGACTTTCAGGTTCTAGGGGTTGTTCTTCCGCATTGTTTGTTTGGTTAGACTTTTATGGAGTTTATGGGAACTCCATTTTTNTAGCATTTAAACCTGCTTCCGTATCTTTAAATGCCTTAGTTTTCGGTTAAGTTGCTTAGTTTGGGTTGTAGTAATGGTTCTCCCACCGGAGGGTTAGTGTGGGTGCCAATCACGACGGTCTGGGTCGTGACAAAGTTGGTATCAGAGCCCTAGGTTCGTTGATCTCGNTGTACCAAAACGAGTCTAGTAGAGTCTTGCGGAACGGTACGGAGACGTCTGTACTTTTCTTCGAGAGGCTATAGGACTTTAGGAAATCTCTCTGTTTTCTCTTGTCTCCTTTCGTGCTATGACTTGATTCCAATTGGTATCTGGCGATNCAAATTGGTATCTAACCTCCTTCACTCTTCTGTCCGCAGATGGTTAACACTAGATTCAACGGCGTCAGGCCCGTAGCTCCCGTCAATGCTCCAGCTGAGGAATCCGCAGCGAGAGGTCGCGGTCGAGGCAGGGGTAGAGGAAGAGCTAGGGGTAGAGGCCGAGGAAGGGTAGCGCCTGCTAGGGATGGAGCNCCGGTTGAGAATGCTCCCAGGAATGAGGNCCCTCCTGCGCATCATGAAGAGATAGAGGAGAATGTNGAGGTTGAGGACGNNGAGGATGTTGGACAAGAGGAAGAGGTGCAGGCTGAGACTACAGGTATTCCTCCCTTAGACCCAGTGTTAGCTCAACAGATCATGTCGTTCTTGAAAGGGTTGGTTGGTCCTGGAGTGCTTCCCTCTGTTCAAGCAACTCAAGCTCCCGCCAATCCCCCTATTGCTANCACTGTNCCCAAGGTGGGTGGAACTGTAGGTAATGATGCTTTCTTCCGTCCTTTGTTGGGTCCTGTTATGACTGGTAATGAGCATGAGATGTTGACTAAGTTTTTGAAGCTGAAGCCNCCTGTGTTCCNTGGTTCTGAGAGTGAGGACGCCTATGAGTTCATCCTAGATTGCTATGAGAGGCTTCATAAGTTGGGNATTGTCCATCAGCATGGGGTTGAGTTCGTGACCTTTCAGCTTCAAGGTGAGGCTAAGCAGTGGTGGAGAGCTTATGTGGAATGCAGATCTTCAGCTTTACCTCCACTCACTTGGACCCAGTTTCATGCTCTGTTTCTGGAGAAGTATGTGCCTCGGACTTTGAGGGATCGCAAGAAGGATGAGTTCATGGCTTTGGAGCAAGGTGGTATGNCTGTGGCTGCTTATGAGGCCAAGTTCCATGCTTTGTCTAGATATGCTACNCAATTGGTGACTACTGAGGAAGAGAGGATCCGTCTATTTATTAAGGGACTAAATTCCGAGTTGCAGGTATTGTCTGTTCATATGACCTCTGCAGGGAGAAGCTTTAATGAGGTGACAGACTTTGTNAAGAAAGTGGAGGGGGTGAGGCGAGACGGTCAGGCTAAGGCATTGGCTAAGAAGGCCAAGAACTCGGGTAACTTTCAGGGTTCTTACTCCAGAGGTTCAGGNAGGCCGACGCTTGCAGCCCGGCCAATTCAGTCCGCTATGCCCGCCTCTACAGGTAGTTACTCGGGAACTCCACCTCATAATTTGATTCAGGATAGCCAGGGAGTCGCGCCTTCGGCGGGCGGCAGGCCATCTTTTGATCGTACTTGTTACAACTGTGGAGAACCTGGGCATATGAGGAGAGATTGTCCCCACCCGCGCGTGTTGGATTCCGCGCAGCAGCAGNCTAGAGCAGTGGTACCCGCGGGNAATGGTAATAATGGTAGAGGACGTCCACAAGGTGGGCGAGGAGGNAATCAGCGAGGCCGCGGAGGTAGAGGAAATGGTAA

>rnd-1_family-283#LTR/Gypsy ( RepeatScout Family Size = 312 Final Multiple Alignment Size (possibly truncated) = 98 Localized to 2361 out of 4218 contigs )

TGTAACGTCCCATATTTTGCATATACTAGTTTTATGTGAGTAGTGAGGGTTTGAAATAGGTTTGACAATATTCGAAAGAGGTTTGAGGCTAAATAGAGTAAGTAAAGTATAATTTGTCATAGTAATCAACCTACTTGCTTAAGCTACTGATTCGGATGTCTTACGTAATTAAGTTACTTACGTACATGTGTAGCTTACGTAGAAAGGATGACATAGGTTCTTAATGGTAGATTAACTGCTGACCTACTTAAATAAGTGGTAAGTAGGTGATGCACCTACTTAGGAAATATGTGGACTGCATTTTAAAAGAAAAGGGCAACTAGGTGATGCAGCTACTTAAAGAACTTGTGGCAGAAATTAAGGGAGTGATGTACCTGCTTACTTAAAGTGAGGGACTAGATATTTCTACACATTTCTTAATGTGAAAAACGTCCATAAAGGACCCTTAAGAGAATGAAGCTCATTTCATATATCTCATCACTTGTTCTACAGCAATTTACTCCCAAAGAGAAGCCCTAGAAGCTCCAAGAGTGCTGGCCTGCAAGTTCACCATTAAAAATCTGAAGAAAACCCTTTATTTTTTTCAAGTCCAAGTTGGTGTAACATCTCCCATCTTAAGGAATTCAAGNGGAGGAACTGATTTCACCTCAAGGTAAGGCTACTGCCATATGTTAATTTTTTCATCCCTCTTTTGTGCTTGAATCGGTTAGGAGATTTGGTATGTGTTAATGAGCTGAGAATTGTTCCTTCTACATGGTGTGAGATTGGGGAATGTTCTGGTTGATTGTGCATGTTGGGACAGCCATGGAGGGGAGTCCCCTTTAACTCGATTGTGAGCTCGTTTGAGCATCTTAAATGTAATGTTAGTTGGAACTCAAGGGACGGTGATCGGAAACCGTGAGTTCATATTGCTACTGCCACATTNTGATGTTTAGGGACTGTTTTGTGTATNGTAAAATAAGGGGGCATGTGCGGAGAAGTCATGGGAACTTGTTAAAGGATAAAACGTTGAAGGGTGTAGTGACGCACCACTTAATTGAGCGTTGCATGAGCTTGCTTCCTGCCACCCCTTTTGTATACTGTTTTCCANACGTTATGAAGCCCGAGGAGTTTCGGGGGACCTTATAAGAAGGTCGTATAGTATATGACGTTATTAGAGGAGAAGGGGTGGAANGAACACCATCGGTTNGATAAGTATGGGCTTACTGCTTGATTACTGTTTTGGTCNGATCAAGTAGCCCAAACTGTTGATTAGCTACTAATATTAGTTTTATNACTTATTTCATTGTAGATTAATAATCTGAAAGGAAGGAGGTTAANTCGTTGTACTATCCGAGTGGTACAGCAAGGTATGTAAGGCTATTCGATTCNATATTCTTCGGCATGAAATCCGATACTTGCGATTAATATCAACAAGTGAGCTTCCTAAGTTCTNTTCCTAGTAAAGGANACATAGTGGCAGCGTACGATCTCCAAATAGCTAACAATATTTCCCCCTCTCCTTAGTGTGTNGAATTACTTCANTATACGTGCGTAATTACCCTCCTATGTGCTGGTATAGAAATGGTATTTGCAAAAGCAAGTTTGGTGAATCCTCCTCTTAGTCAGTTGAAGTCAATTGTGTCATTTAAGCTCACAAGTAATGCATTGATGTTACATAGCTCCTTATGTCATTGTTACTGCCTTGAAGTATTATTTTCATGTCTTCTACTACTGGTCCGTAGTTCCGAATATCAAAAAATGATTATGACCACCAAAACAACAATCTCAAAGATTAAAGAATCTAAGTGAAGCGATCTGTATAATTAAGGGTGGCAGCCCAGGGGCGAAAGCCTAGCATGGGCCGATCCCAATTGGTATAGAAGGGTGGCAGCTCAGGGGCGAAAGCCTAGCATGGGCCGATCCCAATTT

>rnd-1_family-96#LTR/Gypsy ( RepeatScout Family Size = 760 Final Multiple Alignment Size (possibly truncated) = 98 Localized to 2361 out of 4218 contigs )

GGACCACGGCTCGTGGTCNTGACCACGGCTCGTGGGGNGCCTCGTGGNNATGCCTTGGGCTTGTGAGCTTCATGGCCCAAGTTCCTAGCTACTGCCCCAAGNCCACGGGCGGGACCACGGGTCGTGGTCCTCACCACGGCTCGTGAAGCCGCTCGTGGCGCCTGGGCGGGGTCGCTTAAGTGAGGGTCANTTNGGTNATTTCCTTTTTTAAGTCCAATTAAGTGGGGTCGTTTTGGGTANTTTTAGGGGTACTATATAAGTTGTTTTAAGTCNAAATTCACCCAATTTAAGTCATTATTCNCAAACACCCAAATCAAACCAAAAGTTCTCTTCCTCTCAATATTTCTCTCTCTAGAACTCGAAGAAGAAGAAGAAGAAATTGGAGCTAGGGTTCGAAGAATTCAAGGTTTCTTCCTCAAATTTCTTGGAGATTCTTAATCAAGGTATGGTAGCTTTTCATCCATGGGTAGCTTTCACCCATGGAGTCCCTTCAAAACTCGATTTCAAGNTTTCAATTTCCCCAAAANCTAGGGTTTCAATCTAAGTCATGGGTTCTCTTTCAAAACGTTTTCAATTGTTGATTTATGATTGATTATGATTGAATTGATGAATTATGGTTGAATTTATGATGAATT

>rnd-1_family-564#Unknown ( RepeatScout Family Size = 104 Final Multiple Alignment Size (possibly truncated) = 98 Localized to 2361 out of 4218 contigs )

ATGAGGACTGGTGATTTTATCTTAAGCCATAAAGGAGTGGATTGATACCGGAATGACAAGCTTATGGATAAAGAAAGTCCCANTGAGTATACTTGGGTGAAGGCAATTGAAGATTTTAGTAAGAAAGTGCGTATAAAATTGGGTTAGTCCTTTGAATTTGATGGGTATANCTGAGTTTGAGGTGTCGTGTCGATGAAAGGTTGACTTCGTGGTGATGGCGAGGGCTAACTGAGCGTAATGGTAATAAGAGTTCGTGATGGATTGTGATTGGGATGCAACTATATAATAAGATTGTTGGTTAAGCGAATTTTGTATAGTAATNACGACTTGCGAGTATCTAAGGTTGTGGTTTTCTANTATTTGGTGAGTAACGTGGTTGTATGGAAAGTTTTAAGAGATGGTGGGACACCGCTCGAATAGAATTTAAAGAGCTAGAGTCATAATGAAGAAAAATATAGTGGGATGTAGTACAGCCTTTAGAGAGAGGCGAGAGCAAGAATATTTTATTTGATTGCAAGGGCTAAAGAGGTATCTTCAGAGGGGTAGATAATTGGGTTCAGAGACGCGTGATTTCATTCGTTTTGCTNTGGCTATGGTAATAGTGTCACGGGTCCGTCAGATTGGTTAAAAACTATTGGAAAAGACTTGAGAAAGGAGTGTACAGACGTTAGCTCGAGTGTGATGATAAGTGCTTTGTTTTGTTTTTGAGTTTGGTAATGAGTACGGTTGAANTTTTAGCGAAGGATATNGTAAGATCGATGGAGATGCGATCAATGATAAGCGAAGATCCGTGAATTCATAAAAATCTTCAAGGTACGAGCTAATGTTGATAGATAAGGGGATTGTGTTATACGGTTGAANTTACAATTCAGATTTTTCATTGTAAGTCTACGTTCGTGGTGGTATATTTTGTCAAGGTATGAATTGGTAAGGTGAGAAATGATCGATCGTATCGGGTATTGAGCATTTGAAAGGACTTACGCNTGGNTTGAGTCGTAAAGTTTATTACTNTTGATTGTTAAAGTACCTAAGAAAGGTTTTCATTTAAAAGTGTTGGTTTAGAATAAGGTAAATGGTCGGAGTGTTTATGATGGTTGTTAGTATTGTGAGTATGATTCACTAGCTATGCTCNCGCGTGTTTTCACTTTTGTGGGTGGATCCCTAGTGGTGGATCGGGTGTACCGAATCCTGCGATGTTCTCTTGGTTGAGTGGGANGATTGGGAAGACTTGGTTATTTCGGATGACATANTTGGTGTTGTCTTGGAGTTGTTAAAGTATTTTAGTGGTTCTACACGTACCACCTTGCTTATG

>rnd-1_family-124#LTR/Gypsy ( RepeatScout Family Size = 649 Final Multiple Alignment Size (possibly truncated) = 98 Localized to 2361 out of 4218 contigs )

TGTAACGACCCTAAAAACGAACTAGTGAAACTAGAGCCTCACATGTGAGTTTGGAGTTGAGAACTTGATGAAATGATGAGAAATGACCGTGACGTCCGGAAACTAGTCGTTTGAACTAGTGAGTTGTAAGGGTCTTGTCGTTGAAGTTTGGAGTGTTGTCTAAGGGCTTAAGGGGTTGATTAGCTAGTTGATTAATCACCTTTATAAGCTAGCAAGACCTTAAGCTAGGTCATGAAATCATTTAGAACCGAGACCCTCAAAACAGTCCCCTAAAAACAGTTTTTAGTTTTAAGTTAGGAGTTAGGGTCAACTTCAAACGGTCATATCTTTTAGCACAAAATGAATTAGGTGGCCCATGACCTATCAAATTAAAGGTCTTTGAGTCCTCTTTCCAACGCCACCGAGTTTGCCNAATTCCGANCTCGGAGTAAAAAGTTATGCCCGTTTTAGTGAAGCCCTGTC

>rnd-1_family-58#LTR/Gypsy ( RepeatScout Family Size = 1010 Final Multiple Alignment Size (possibly truncated) = 98 Localized to 2361 out of 4218 contigs )

TTTTTGGAAAGAATAAGAAAATCTGGAAANTTTGTTTAAGTTAGGAAAAGTTGAGTTTTGGTCAACTTCAAACGGCCATAACTCCTAGCTCAGGATGAGTTAGGTGTACTTCCAGATATGGTNGGAAATNTCTTGGAATGATCTTTCCAACGCCGCCGAGTTTGCGCGATTCCGAGTTCGTATGAGTGAGATATGCCCTTCGGAAGTTGGGCTGTTGGATTAAGGAAAGTCCAATCCGGATTTTGGAAGGGTANTNTGGTCTTTTCCCTACCCAATTATTCTAATTCGTTTTTAGGAATTTAATTGGGGTAAAATCGGATTTTAGTCAGTTTTAGAAAAATTAATTTACGCTAGGGCTTGGAGAAGAGGAGAAAGGAGAAGAAAGNCAAGATTCGTCAAGATCGTCGAGNTTNGCTTGTGGATTTCGTCGGGGGTGATCCCTACGAGGTATGTGAGATCACATAGCGTTGGGTTAGTTCACCCACGCGCCAATCATGATTCAATTCAGCGAAATTTGTGTNATTTGAAAGTAAATCNTTGAGTTCTTGATGAAATTCGTTTGAATTCTTGTGGGTTGTGTTGTTGAAGTTTCTTGNGATTTGATTCGTGTTTCCGGGTGTNATTTCGAGTTGAATCTATCGTATATTGAGGGTATAAATGATTCTAAGTGTTTGGGGAAAGAACCATTGAAGTTTAGAGGGTTTAGAGNTGAAAAACGAAGAAGAAAAGTCGTCGAACACCTGGGGAAGGGGGTGGGGCGTCGCGCCAGCCAGNGCGCCCCAAAAGGGTCTCTGAAGTTTGGCCTCTGGGGCGCCGCGCCAGCCAGNGCGCCCCAACCCGGTCTCTGAAGTTTGAGGGCTGGCGCCCCGCGCCTCTCAGAGCGCCAGGGACGCCAGTTCTCCCCATTCNTTCCCCACCTTTTCGTACTTGTTCCTTAGCGACGTACCTATGTTTTCTAGTTGATTCCAACACTCTAAGGTACGTCTAAACATCATGAAATCATCCATAAACATGAGATCATGAACCTTGAATCCATAATCCAATTCAAGGAAAGTTAAGATCAAAGTCAAGAGAAGTTAAGAGTCAAGTCGAGAAGTTAAGAAGCAAGTCAAAGTAAAGTTTTCAAAAGTCTTTTCAAACGTTTTAACAA

>rnd-1_family-75#Unknown ( RepeatScout Family Size = 858 Final Multiple Alignment Size (possibly truncated) = 98 Localized to 2361 out of 4218 contigs )

TTTCGTCCCCNTTGACNAAGTTGACTCAGAAGACGGTNAAGTTTCAGTGGTCTGANGCTTGTGAGAAAAGCTTTCAGGAATTGAAAAANAGGTTGACTACCGCNCCAGTNTTGACCTTACCAGAGGGTACGGAAGGCTNTGTGGTGTATTGTGATGCNTCTAGAGTTGGNTTGGGTTGTGTGTTAATGCAGNATGGTAAGGTTATAGCTTATGCCTCNAGACAGCTGAAGGTNCATGAGAAGAACTACCCGACCCATGATCTAGAGTTGGCTGCNGTNGTGTTTGCTTTGAAGATNTGGCGNCACTATTTGTATGGTGTTCATGTNGACGTGTTCACCGATCACAAGAGCCTTCAATATGTGTTCANNCAGAAAGAGCTNAATCTCAGACAGAGGAGGTGGCTNGAGTTACTCAAGGATTATGACATGAGTATTCTTTATCACCCAGGTAAGGCTAATGTNGTNGCTGATGCCCTNAGCAGGTTGTCTATGGGTAGTACCGCCCATGTTGAGGAAGAAAAGAGAGAGCTAGCGAAAGATGTGCATAGACTTGCNCGCTTGGGAGTCCGACTNATGGATTCCACAGAAGGAGGAGTAGTGGTGACGAATGGGGCNGAATCATCATTAGTGGCGGAAGTGAAAGAGAAGCAAGACCAAGATCCTATTTTGCTTGAATTGAAGGCAAATGTTCANAAGCAAAAAGTATTAGCTTTCGANCAAGGGGGAGATGGNGTNCTTAGGTATCAAGGTNGATTGTGTGTNCCGAANGTGGATGGACTCCGAGAGAGGATTATGGAGGAAGCNCATAACTCCAGATATTCTATTCATCCGGGTTCCACGAAGATGTATCGCGATTTGAGAGAAGTCTATTGGTGGAATGGCATGAAGAAGGACATTGCGGAGTTTGTGGCTAAGTGTCCNAATTGCCAACAAGTNAAGGTAGAGCACCAAAGGCCCGGTGGTNTGGCTCAGGANATAGANNTTCCNNNNTGGAAGTGGGAGGCGATCAATATGGATTTCATCACAGGTTTACCTCGGTCTCGCAGGCAGCATGACTCTATTTGGGTGATTGTCGATAGAATGACNAAATCAGCCCACTTTTTGCCGGTNAAGACTACCCATTCGGCGGAAGATTATGCNAGGCTNTATATTCAAGAGATNGTNAGGCTNCACGGGGTTCCGGTNTCTATTATCTCAGATAGAGGTGCNCAATTTACCGCACANTTCTGGAAGTCNTTCCAGAAAGGTCTGGGTACGNAGGTNAACCTNAGTACNGCCTTTCATCCTCAGACGGATGGNCAGGCAGAGCGCACNATTCAGACNTTAGAGGATATGTTGAGGGCTTGCGTGATCGACTTCAAGGGTAGTTGGGATGATCACCTACCTCTTATTGAGTTCGCTTACAACAATAGTTACCATTCTAGCATCCAGATGGCNCCTTATGAGGCTCTNTATGGGAGAAGATGTAGATCTCCNATTGGATGGTTTGAAGTNGGTGAAGCNGGGTTGATAGGACCAGACTTAGTTCATCAAGCTATGGAGAAGGTGNAAGTCATTCGAGAGAGGTTGAAAACGGCNCAGAGTCGNCAGAAGTCCTACGCAGATGTTAGGAGAAGGGANTTAGAGTTCGAAGTNGATGATTGGGTNTNCTTGAAAGTNTCACCCATGAAGGGTGTTATGAGATTTGGTAAGAAGGGGAAGCTTAGTCCCCGATATATTGGNCCTTACAGGATATCCAAGAGGATTGGCAANGTGGCTTATGAGTTAGAGCTGCCGCAAGAGTTAGCAGCGGTTCATCCGGTNTTTCACGTCTCNATGTTGAAGAAGTGCGTGGGNGATCCTTCATCGATCGTNCCAACTGAAGATGTTGGGATCAAGGATAGCTTNTCTTATGAGGAGATTCCGGTTCAGATTCTAGATCGNCAAGTTCGNAAGTTGAGAACCAAAGAGGTAGCTTCGGTCAAAGTNTTNTGGAGGAATCAGTNCGTNGAGGAAGCTACTTGGGAAGCCGAGGAGGACATGAAGNNNAGATATCCTCATCTCTTT

>rnd-1_family-263#LTR/Gypsy ( RepeatScout Family Size = 338 Final Multiple Alignment Size (possibly truncated) = 98 Localized to 2361 out of 4218 contigs )

AAAAGGGGAGGCTGAATCGTGATAGTTGTATTGGTCAGAGGATAAGGTATGTAAGGCTATTCGATTCAATATTCTTCGGCATGAAATCTGATACTTGCGATTAATATCAATAAGTGAATTTCCTAAGTTCTATTCCTAGTAAAGGAAACATAGTGGCAGCGTACGATCTCCAAATAGCTAACAATATTTCCCCCTCTCCTTAGTGTATAGAATTACTTCATTATATGTTCACTACTCTATACTTGTGTAATTATCCTCCTATGTGCTGGTATAGAAATGGTATTTGCAAAAGCAAGTTTGGTGAATCCTCCTCTTAGTCAGTTGAAGTCAATTGTGTCATTTAAGCTCACAAGTAATGCATTGATGTTACATAGCTCCTTATGTCATTGTTACTGCCTTGAAGTATTATTTTCATGTCTTCTACTACTGGTCCGTAGTTCCGAATATCAAAAATGATTATGACCACCAAAACAACAATCTCAAAGATTAAAGAATCTAAGTGAAGCAATCTGTATAATTAAGGGTGGCAGCTCAGGGGCGAAAGCCTAGCATGGGCCGATCCCAATTGGTATAGAAGGGTGGCAGCTCAGGGGCGAAAGCCTAGCATGGGCCGATCCCAATTTGTGTAATTATGAGGACGGCATCCCAGGGGTTAAAATGCCTAGCATGGGTCATCCTCTTCTACCACTGATCAGCTGGTCATTCGTATCACATGCCTTATAAAGATCATAACGCACAGAAAAGTAAAGAAAAGAATGTAACATACACGATCCCACAAGAGTNAACAAAGGTGGTCTTCTATTCTGATCTATGCACCATATATTGATACTTGGTTTCATTCGAGCTCTTATTTTACATATGTTATTGCCTTACATATTCAGTACATTCTTTCGTACTGACGTCCCTCGCGGGGGACGCTGCATTTCATGCTGCAGGCACANGTACTCCAGCTAGTAGACCTCCCCAGTAGGAGCACACGACACTCAGCTGCTTTTGGTGAGCTCCAGGTTGATTCGGAGCTTTACCGAGTCCTTGGTAGATTCATTTTGGTATTGTATCGTAGTTAAGGGTAAGGCGGGGTCTGTCCCGACCTACTCTAAGGTTTTCTATCTTTTAGAGGCTTTGTAGACTTATGTATATGGTTCGGTTGCGTCTTAACAAGTTGTGGCCTCAACGGCCAAGTCTTGTATATAAGTTTTGGGTCTACTTATGTCGGTTCGTATCGCTGCATCCTATGTGAACTTGTCACAAGATTGTCATAGTTATATGCATAGTAAGCACAGGTTACATGTTGGTTCTCTCGGGCCTTTAGGGCATCGGGTGCCTGTCCGTCTTAATGGGATTTGAGGCGTGACAA

>rnd-1_family-180#LTR/Gypsy ( RepeatScout Family Size = 503 Final Multiple Alignment Size (possibly truncated) = 98 Localized to 2361 out of 4218 contigs )

ATGTTTTTATTCGTAAATGTCTATNCCTATGATTGACTTGANCATATTCGTACTTTNCTATTGANTCTCAAACACGTACCGCTCCCTGTGGGATTCGACCCCAACTCACTTAGTTGGGTTATATTACTGACTAACGATCGTTGACGCTTAGAATTGGATGAAGTGTCTTTGATACGTNAAATCA

>rnd-1_family-341#Unknown ( RepeatScout Family Size = 243 Final Multiple Alignment Size (possibly truncated) = 98 Localized to 2361 out of 4218 contigs )

ACATGACCAATTTTTATTGGTCAGTGCTGTCAAAAAGCTGCACCCACGCGCCTAACATNGGTGGAGTCACGGAGTGTGCCACGTAGGCCGAAAAGGGGTAGAAAATTATNTATAAAATAAGTTCGGGGGGGTAATAGGACCTTAGTANAGNTTAGGTGTGTCTCTGGGATTTCGGGCATAGGCTGGGGGGTACTTATGCATTTTCCC

>rnd-1_family-109#LTR/Gypsy ( RepeatScout Family Size = 702 Final Multiple Alignment Size (possibly truncated) = 98 Localized to 2361 out of 4218 contigs )

TCTTGAATTGGTAGGCTTAGGCATCCTCTTCTTGTGTAATGAATGTGTCTTGACTTGATGAATATGAATCGGTAGGCTTATGGCATTCCTTTCATATATGAGTAATGAATGTGTCTTGACTTGATGGATATGAATCGATAGGCTTATGGCATTCCTTTCATAAATGATCTAATGTAATGANGACTCTNGAATCGATAGGCTAATGGCATTCCTTTCATTAATGATGTCAATGNACCTTGAATCGGTAGGCCTATGGCATCCCTTTCATGAGTATAAGATGTAATGATGAACCTTGAATCGGTAGGCTTATGGCGCCCTTTCATGAGGANTCAATGAGCTATCCTAATAATGTACCTTGAATCGGTAGGCCTATGGCGCCCTTTCATGTGTAATAATGTACCTTGGGTCGGTAGGCCTAATGTTGGCGGCCCTCTCAAGTACAATAATGTTAATGTGAATGAANTACTCTATGGGAATGNAGGCTAAGCACCGAGTGGATATGGTAAGATGGAAGCTCTCCCNACGTTAGGCCGGGTTCCAATGAACATCTTCCTTATCCCATAACTATGTGCCCACATAGGATATTAGCTAGTGGAT

>rnd-1_family-29#LTR/Gypsy ( RepeatScout Family Size = 1249 Final Multiple Alignment Size (possibly truncated) = 98 Localized to 2361 out of 4218 contigs )

TTTTTTGTGGATGTTTGAGCTTGTGGCTCCTTTTATTTTAAATTGCAAAATGATTTTTGACCCTTCCGAAAAATTTTTGCCACCTCTTGTNTGTGTTGAATGTGGCTGTTCTTTTGCAAATTTGAGTATCGGTTTCGATCAATACCGATGACTTGAATAGTGCTCNTAATCGAACGAAAATGAATGTGCGGCTACGATGAGGCCAAATGAAGTTGCATAGACAATATGTGAACTCTTTGCATCTCGTTGTGACTAGCCGATTATCGAATCGAGTCTCTTGTGATGANCGTTGCACACTAGCGAAAGTGTGGAGTCTTGTTTGACNTTGGTGTCGATGCCTTGTGTGATGAGCTTACCGTGAACCATGCGTGATGACACCTAGAATTTTCCCCGTTGGTCCGGTCGACTAAGTGATGCGANCTCTTGANATGATCTTAGGCAACTTTGAAGAGTGTGAACCAATTTGACATATACCTCTTTTGTGGCCTACCTTGTGAGTGTGTGAACCTCTCTTGAACACCCCTTGAGCCTTACCCTTCTCTTGGAAGAAACTTGATGAAAACTNGACCTTTCTTNATCCATTACCCATAATCCTCATGANTTGTGGTTTGTTTGAATAGCCAACTTAGGCCAAAAGCCTAAGTTGGGGGTGTGGTGAAAAGAGAAGGTAAATCAAGAAGTGCAAGGAAAGTCCCCTTTAACCCATGGTTTTGAGAAAAATGGAACCCCTCCATACAAAAAAAAGAGAGAGAAGAGAAAGAAAAAGAAAAAGAATGAAAAAGAAAGTTGTGGAATAAAGTACAAAAGAAATGGGGTTTCCGAGCAATCCATGGAAAGTGAATAATGGGATGACTTATGAGCACTAAAGAGAATGATGAAGGAAAAGGAAAGGAAGTGTTGTGAGCACCACATTTCATGAGGATGAAAGCCACTGAGCCTAAATGACCATACCTTTGCACTCAGCCCCGTTACAAGCCTTGAAAAGACCTTTTTGATCTTGAGTGAGCTGAAACGAATGTCGATTGGAAAATANGGGCAAACCTATGGGTGAAANCATGCATTGTGTTCNTCTTTGTGAGTGTGAGCGTTGCATCTGATTCCGGAGCTTTAAATTGTTGAACCATTGTGTGTGAATATGGAATCATTCTTTGTGTGAGGGCATTTGAATACTTTTGTTGAGCTTGAACTTGCATTTGAAGCAAGTATTGCGAGCTTGAGAATCTTTGATAATGGTGAGTCACAACTTGAATCTTTGAGTGCACAATTGATCCTTGCATGAGTAAGTTGAGTCTTGTTGTGTGCATTCATGATTGAGTCTTGTGTAGCACTGTTTGAGACATCCTTTTTGAACTGCTGAACTTGAGTTTTACTTGAGGACAAGCAAAAGTTTAAGTTGGGGGTGTTGATGAGTCCACGAATTGGACTCATTTAGGGCTTTATTTTAATAGAANTAGTGTCCTCAAATGCTTATTTTGTCTCAATATCTGATGAAAACCCTTAAGTTTCAGGTATTTGAAGTTTGAGGGAAAGCATGGACACTANGGCGCAAAAAGGAACGAAAAGGCTGAAAAGAACGAAGAAATGAAGGCCTGAGGATCGCCGAGTCCACTTGGCGAGTCGCCGAAGGGNCNTACTTCGCCTTTTGTTCCAGTGCGCCGAGCCCTGAAGGAAAGGATCAAGTCGGCGANGAAAAGGAGCAGTCGGCGCGTCGCCGAGNAGTTCCGCGAAGCAGTACTATATCGCCCAATGACNCAGAACGCGANGATGCTGAAGGCNAGCGCAAGACGGCGATGAACTACACCAAAGGGCGGATCGCCGAGTTGATCGGCGATCCCGACTAACNNCGCCGAACGATCCGCCGCAGCACAAATTTCTGAAGACTATAAATACTAGTTAGAGTTNTAGTTTTTAGGAGTCGAACAATTATTATAATTTTCATATAGAATAGTACTTTTTAGTATTTTTTCTCTAAGTTTGAGAGGGTTTTGGAGACTTGAAGAAAGAGAGGGTTTCATCTTCGAGAANTTGGAAGTGGGTCTTCTNGATTCTTCATCCTTGGCCTGTATCAAGACTTAANTCTTCATACCCATTTGANTGCAANCTCATTTTGGTATAAATTTTTATCTCTTGATGTGTGGCTAAAAACCCCAATTCTTGGGGTGTGATTTAGCGAATATGGGTTGATATNATTGTTGGGTCTTGCTTGCTGATAGTTTAGATGTAGTTTAATGGTGATTTCGCCTAGTGGTTGTGGTTNAATTTAATGGGTTTGTAGTTGCAAATACAANNCCACCCATGTGTTTTCGGCTTGCTCGAGAGAGAGGTCGCGAAACCAAGACCACTAGATTGATGGCCTANGGAGTGGGTCGACATGAGGTTCAGCCCGAGAGGGTGAGCCCTAGTCCCATNTCCNNACACTCAGCTCGAGAGAGTGAGTGGGNTAAGGCGTAGGCTGGTCTTCATGCGGCAAATGGGTGTCCGAGAGGAACCCATTTGAAACGGGGTAAGTTGCTCGAGAGAGAACTTATCCCCATTAAAGCCTAGCCTAGTCACTATTATTCTACGAATTTCCTATCGAAAGCATGTACCCAACGATTTANCTTAACCCGTATTCCGGTCACATCCCAAGAATCCCNTCTCATTACTTAGATTTCTTGTTTATTTTGTTGTTTTTACTTACTTGTGACAAAACCCCCATTGATATTTGACACTTTTGTGTCCCCCTTTAATTTACNATGTTTTTANTCGTTAATGTCTTTAGCTACGACTAGTTAGAACTAAATTTTATTTTTCTATTAATTCTCAAAACCACTCCCTTGGGACACGACCCCAACCCTTGGTTGGGTTACTATACTATCGACGATCGTAGACACTCGCACCGTAGGTTAGTGTCGTTGGTCACGATAAGCATCA

>rnd-1_family-206#LTR/Gypsy ( RepeatScout Family Size = 457 Final Multiple Alignment Size (possibly truncated) = 98 Localized to 2361 out of 4218 contigs )

TTTTTGTCTTTTTACCTCCAAGAAAACCCTAAACGAATTTCTTGACTAATNAAAGGCCCAAAACAATCAGATTTTCATCTCTTAATCCATCATTCTCTTCTCTCTCAAACCCTAAGGCAAAACCCTAAGGAAAAATCAAAGTAGAAGACTCCATTCAAGATTCTTTCAATCTTTTTCAAGATTCTTCTTCAAGGTAAGTCTTTCTCCAAGAATTTCATTCTTTCCAACTCAAATTCCTCCATACAATTATGAATCACTAATGAAAATCATAATTCTTGGCCATAGAGATTTCAAGAAACTAATTCAAGAATCTCAAGAAAGGTTTTCTTGATCGTTCNCCTTCAAGCTAGGGTTTCAAGGCTTCTAATCAAGTTCTTCTTCAAGATTCTTCAAGAACCTTGTTCTTCCAGGTATGTAAGGCTATCATAGTGTTGGACTAGTTCGTCCTCACGCCCTACATCTACTTTCAAATCAGTAAAAGAGTAATCTAGGATTCTATCCCTAGATTTGAGTATTCTTGAGATGAGTTGATTTTGAGTTCTTGAGTTCNTGNTTCTTTTTGAAATTCTATTCCTAATTATTGAATTGCTATATGTTATGCATTGAGATTCTTGAGTTGATTCGTTCATGTCTATATTTCAGCATGAACCCTATNAATTGAGTTATAAATCTTGAGAGAGAGTNTTTTCGAGTGCCAACACTTGAGTTTTAAG

>rnd-1_family-44#LTR/Gypsy ( RepeatScout Family Size = 1113 Final Multiple Alignment Size (possibly truncated) = 98 Localized to 2361 out of 4218 contigs )

TGAAGTCAAAAACCCAATTCTAGCTAGGGTTTCCTTCTACATTGTGGGTAGGGTTTGGGTATGATTCCAATCGAGTGGATAATACCCGATTATGATTGANTTAGCATTGAATTATGATATTATGATGAATTTACGTGATTTCCATGGTTAACCCTAGTCTAAATTGATGAANNTGATATTTGTGGGTTTATGCCATTAGAGGCGAAATTGAAGGTTCATAGGTGATCTTCTNAAATCGATGTCATGTGTAGTAATTGTTCAAGATTAGGAGCATATAGACATGAATTGAACTAGACTNTANTGTATATGATGGATCNTGACTNGTAAAGCTNGAATTGAACCTTTATGATGAATTATGACTAGAATCGCCTAGTAATGAAGNATGTGATTAGAATGCCTTGAAATCTAGGTACGTATGATGATTGTAAGAAGTAAAGCTTTGAGAGTAAGGCTCGTAGTCATGAATGTTGATCACTAGGGCTTTGAGAGTAAAGCTAATATGATGAACTATGNATGTTAAGGCTTTGAGAGTAAAGTCTATATGAGAATAGAAGACTAATGGATAGAATTGTGGTGAAAGGCCTTTACCCACCTAATCTACCTTATGAATCNTNGATGGTATGAAGTGATAGAATCACTTGAGTCATGATGATATCCNTGTCTAATGTAGAATTCTAGACATGATGTTAGGCATGAATATGAACTTAACATGAAAGACTTCTCACATAGTAATGGTTCTAGGGTTGAAAGGGCTTCTCTTACCTAATTGAACCTAAGGCTATAGAGCCTTTATACCTAGGTCATGGATGGTGATAGTTGCTCACACATGGTCTAAAAGAGGTATTAGCATGGATTGGTTGTCAAGGACATCTTTCCATGAACTTGAGTCAAAGTAGAGACTTAATGATTATCTCGTGGGATTNATGCCTAGCACCGAGTGGATATTGAAATGAGATGGAAGTTCCCACCTAGTTGAGTCCGGGCTTCCATGGAGGCTCCCACCTAGTTGAGTCCGGGCTTCCAACATGGGATCTCTCACCTAGTNGAGTCCGGGTTTCCCAAAGTATGTCTCATGAGATGGAAGCCTTCACCTAGTNGAGTCCGGGTTTCTAGTAGCAATCTCCNTATCCCATAAACTACGTGCCCCCGTAGGATANTTAGCTAGTGGATCCACCTAAGCTAACAGACCGGTTCTACCTTAGGCAAGTAGACCAACCCTTTNCGGTGTGGGTAACACCGGGAGATCATGTNATAGCTCACATGGTCTCATGTCGGTTAAGGCTATATCCCAANTAAACAAGATTAGTAATGAACTAAGATTGTGC

>rnd-1_family-129#LTR/Gypsy ( RepeatScout Family Size = 643 Final Multiple Alignment Size (possibly truncated) = 98 Localized to 2361 out of 4218 contigs )

CGTTTTTGGGGGTTAGACATTATTGTANACTTTTAGTTTTTGGTATTTTTGGGAATTAACTTGCAAACTTTAGCAATTTAATTTCCGGANTTCGGTTTATACGATTTTCGCTTTGAANTTCAATTGAAGATTTCGGGTTTCATCTACTCTAATCGTAAGTTCATGAATTCTTCTNACCTAANTATGAATTGTGNNCTCTCAAGCATGAGTAGCTAAATCCACAACTAGGGTTGTGGGAACCATGAGCAATTAACAAAGTATGAATAATAATTAAGTAATTCTTGAATAGTGTTTATGCATGTATTGNTAATTCTTTCGTTTAGAAGTCTTTTTAACGGTGCGCAACGTTAGAACTCGCCTCGTTCCTACTTGCCGGACCAAGGAGGTAATNAATGAGAAAAGAATTAACAACAGAGATTTAGTGTGATGCTATCTAATAGTCTAATGTCGATTGGTGCGAGGGTGAAAACTAAGCCATACATCGACGTGATGTCTAATNTGAGGTAAAGGTAAGGGTTAGTAAANTATACACNCGTAGCCGGACCAAGGTGCGGGGTGAAATTCCCTANTTGGAGGACCAATCACTTAGGGATACCTAACTTACCGACTTTGCATGTAANACACTAGGAAAGGATTACTATTATTAGGATTACCGCGTTAAGAGCTTGTGGGGAACACGTACACCCTAGTTTCTCTCTCATATTGATAACTCAAAAGTTTGAATCTTGCTTACTTGTTACTTAACGACAATCAATTATTTGTTTCACAAACCCCCCCCCCCCTTTANTTACTTGTCTCGGAAATAGTTTGACTAAACGAATATAATCGTGAGTTAAGTTTAAGTCTAAACCATATTCCTCGTGGGATCGACCCCAACCTACAAGTTGGGTTCTTTACTTGATAACGATCGCTTATACTTCTTTAGGGAGGTGTAATTTGAGCGTATCA

>rnd-1_family-262#Unknown ( RepeatScout Family Size = 341 Final Multiple Alignment Size (possibly truncated) = 98 Localized to 2361 out of 4218 contigs )

TTCATCCATGGAATCCTTCCATCCATGGAGCCCCCTAGAATTCCCCAATTTGAAGAAGAATTCCCCAATTCNAGTTAGGGTTTCTTCCAATGTTGTGGGTTATGNTGAATGTTGATCCAATTGAGTGGATNATGATTGTTTATGATTGAATTGGTAGAATTACATGTTTTTAGGATGAAATTACATGTA

>rnd-1_family-13#LTR/Gypsy ( RepeatScout Family Size = 1448 Final Multiple Alignment Size (possibly truncated) = 98 Localized to 2361 out of 4218 contigs )

AATCATAACCTTGAATTCATAATTCAAATTCAAGGTAGAGTTAAGAGTTAAGTCTTGAGAGTTCTTTCGAACATTTTGAGAAAGTCCCTTTGAGTCCTTTTGAGGAGTCTTCTACAACTTCTAATAACTTGTTTCAAGACTCGAGCAAGTGAGTATGAGAATGAGGAGAATGTATTCATGAGTCTACTTTATCATCACGAGATCCTTCATATCATGAACCATAACTCTTGAATTCATAATTCACATTCAAGAGAGAGTTAAGAGTAGAGTTCAAGAAAGTCTTTGAGTTCAATTGTGAATCCTTTGAGATCAACTATCGATTTGAGCTAAGTTTTGAGGAAGTAAGTATGAGAATGAGAAGAGTCGTATACATGAGTTCCATATTGTTATGTAGACCCTCGAGTCGAGTCGTTCATGCCCATAAATTCCGCATGAACTCCATAAGTTGAGTATCTTTGAGAGGAGTAGTATCTTCAAGTTCTAAGTCTTGAGTATTGAGTTCCTAATCCCTTTTGAGAAGTATCTTTGAGTCCTTGAGTTGAGTAGTTCATGCTCATAATTCCGCATGAACCCTCTGAGTCGAGCATTCTTGAAATGAGTAGTATCATTGAAGTTCTTGAGTTCCAAGTATTGAGTTCTATCTATGGTTATTGAAAACCTTGCATTGAGTCGTTCACGTCCATAATTCGGCATGAACCATATTTTAAGAAGTCTTTTACAAACGTTTNAACTTTGTTTTAAGACTTAAGCTTTGAGTTGAGTAAAGAGTAAGAGTAAAGT

>rnd-1_family-84#LTR/Gypsy ( RepeatScout Family Size = 828 Final Multiple Alignment Size (possibly truncated) = 98 Localized to 2361 out of 4218 contigs )

TATGTGAAGGAATGATGTGAATGAGTTTTATGTGAAGCTATATGTGAAGGAATGATGTGAATGAGTTTTATGTGAAGCTATATGTGAAGGAATGATGTGAATGAGTTTTATGTGAAGCTATATGTGAAGGAATGATGTGAATGAGTTTTATGTGAAGCTATATGTGAAGGAATGATGTGAATGAGTTTTATGTGAAGCTATATGTGAAGGAATGATGTGAATGAGTTTTATGTGAAGCTATATGTGAAGGAATGATGTGAATGAGTTTTATGTGAAGCTATATGTGAAGGAATGATGTGAATGAGTTTTATGTGAAGCTATATGTGAAGGAATGATGTGAATGAGTTTTATGTGAAGCTATATGTGAAGGAATGATGTGAATGAGTTTTATGTGAAGCTATATGTGAAGGAATGATGTGAATGAGTTTTATGTGAAGCTATATGTGAAGGAATGATGTGAATGAGTTTTATGTGAAGCTATATGTGAAGGAATGATGTGAATGAGTTTTATGTGAAGCTATATGTGAAGGAATGATGTGAATGAGTTTTATGTGATGATCTTGTGATGATCCTATGTAAAGTGGATAGCGTTGTGAGTTAAAGTATGATTTCTCGTGATGTATATATGAAATGATGTTGGATTATGAAGTATCCTTAAATGAAAATGTTGTGACTTGGTTGGTAGACTAAAGTGTCCCTCCTTGTTACTTAAAGTCTTGAACGCATGAATGTGTGAAATGGGCAGAACTAAGAAATGGTGCCCTTTCATAAGTCTAGCTTTCCTAAGTAAATGTAAAACCTTGAAGTCGGTGGACCTNAGGGTGGTGGCCCTTCTCATGTGTGAAATGATGGACCTTGGATCGGTAGGCCTAAGGCACCCTTCTAGGGGGATTTAATGAGCTATCCTTATGAACCTTGATCGGCCATGACTAAGAAATGGGGCCTTTCTTGGGAAGAGGTACTATGAGTCGGTTGAACTAGAAAAGGAACCCTCTCTAAGTACATATGTGANTGAATACTCTATGGGAANCAAGGCTAAGCACCGAGTGGATGTGGTTAGGANGGTGGCCTNGTCGAGTATGAGGTTAGGCACAATGAACTTCCTTGGACATCCCCTAAACCATGTGCCTACATGGGTTGCTTACTAGTTCTACCTTNGGCAAGTAGAACACCTTCCACGGTGTGGGGTTTATGACACCGGATTCCATGTCTAGCTCGCATGGTCTATGTCGGTTAACGCCTATTCCCATCATGTGGGATGTGCACTCTAGTTACTTGATAGGTTCTACGAAGTGTAGGTAGTAGTATGGGATGCTATCTACACATNGCACGAGTAGGCTTTGAAGGTGCTANAGTGAGTTCCCTAAGTCTTAACGACTANTGTGTGAAAGTCCCCTTATGTGCNTGAATGTCTCCTAAATGGTTTNATGAAGAATGTTGGCCTATTGATGTTATGTTGTAAAGGAAATGTCCTTATCTAAGGTAATCTTGGGGAACACTTAAGTGTGCTTGAAGAGGTTGTATGGGCGGTCACTTCTTGTCTCACTCAAGTGTGTCTTAAGGTTATATTAGGTAGAGGTCCTATGGTAATGAAATGACATGTGTTCTTTTAAAGTTAAGCATGGGTTGTATATGGATNTATGTTGAGTATGTGTACGGTTGGCTATGGTCTTATATGTGAACGTTGACTT

>rnd-1_family-514#Unknown ( RepeatScout Family Size = 123 Final Multiple Alignment Size (possibly truncated) = 98 Localized to 2361 out of 4218 contigs )

TTATTATTTTTATGTTAAATCATTTTTTCTAAAAATAGTCAAAATATATTTTAGTCAAGTCGCCGGNCAACCGCGTGTTAGCGGACACTTCGAGTGCTTTAAAACCTTCTCGAAGTGTAAATATGAACCCCGAACCCTTTTTNGTATTTTCAANTGATTTTATCTGTTTAAATCTTTGAAAATTATAAGTTTTCTTAATTTCTTTAAAAAATTAAGTGGCGACTCTTTTCTAAGTATTTTTCTCAAATTGTTTTATACTTAGAACATTTCAAAAAGTGATTTTTCTAAAGATAGTAAAATTACGGCACAACA

>rnd-1_family-244#Unknown ( RepeatScout Family Size = 375 Final Multiple Alignment Size (possibly truncated) = 98 Localized to 2361 out of 4218 contigs )

TTAATGGTAAATATCCCACAAAATGAACTAAGTTTCTTTCAAAGGTTTTACGAAGCTTTCANTATGATTTTAAGATGCATTGACCATGTTCATGATTTATGATTCTTCTTTAAAGGTTTTACTATGTTTTAGCTTGGTCATGCATATCATGTTTTGATTATGTCCTCTTATGATCATGTTTCATGTTTTATGCATTTCCCTCATACTTAGTACATTCCATGTACTAATGCATACTTGTGCCTACATTGTTTCACTAATGTAGGG

>rnd-1_family-205#LTR/Gypsy ( RepeatScout Family Size = 461 Final Multiple Alignment Size (possibly truncated) = 98 Localized to 2361 out of 4218 contigs )

CTTCTGAGGTGACTCCGGGCACTGATGCCCAAGTCCAGAGTGNTACACCGGGCACTGATGCCCAGACAGATGGAGCGACTGCGTAGACAGGATCCCTTCTTTACCTCCCTCTCTGTCTTACTTTATTTTGACTTTTGGATATTATTTTGCTTGCATTTGAGGACAAATGNTTTTTATTTGTGGTGGGGTGAGGCCCACCTTTTGTGACCTTTTGTGACTATGGTTTTGTTTATATATTTGGGTTTTTGAGCTATAATTGTGTTTTACACTGTTTTGTGGATGTTTGAGCTTGTGGCTCCTTGTTTTNAATTGCAAATGATTTTTGGACCCTTCCGAAAAATTTTTGCCACCTCTTGTTTGTGTTTGAATGTGGCTGTTCTTTTGCAAATTTGAGTATCGGTTTCGATCAATACCGATGACTTGAATAGTGCTCNTAATCGAACGAAAATGAATGTGCGGCTACGATGAGGCCAAATGAAGTTGCATAGACAATATGTGAACTCTTTGCATCTCGTTGTGACTAGCCGATTATCGAATCGAGTCTCTTGTGATGAACGTTGCACACTAGCGAAAGTGTGGAGTCTTGTTTGACNTTGGTGTCGATGCCTTGTGTGATGAGCTTACCGTGAACCATGCGTGATGACACCTAGAATTTGCCCCGTTGGTCCGGTCGACTAAGTGATGCAATCTCTTGANATGATCTTAGGCAACTTCGAAGAGTGTGAANCAATTTGACATTATACCTCTTTTGTGGCCTACCTTGTGAGTGTGTGAACTCTGCTTGAACACCCTTTGAGCCTTACCCTTTTCTTGGAAGAAACTTGATGAAATCGTGACCTTTCTTTATCCATTACCCATAATCCTCATGANTTGTGGTTTGTTTGAATAGCCAACTTAGGCCAAAAGCCTAAGTTGGGGGTGTGGTGAAAAGAGAAGGNAAATCAAGAAGTGCAAGAAAGTCCCCTTTGACCCATGGTTTTGAGAAAAATGGAACCCCTCCATATAAAAAAAAAGAGAGAAAAAGAAGAAAAGAATGAAAAAGAA

>rnd-1_family-260#Unknown ( RepeatScout Family Size = 344 Final Multiple Alignment Size (possibly truncated) = 98 Localized to 2361 out of 4218 contigs )

TTTTTTTTTTTGGTTTCCCACCCGGTGTCCGGTACCCACATTGGAGCCCGACTAAATTCGGATTCGCGCCGGAAAGTCCCACATTGGGGGTAAAGCGCTCCCTAACAAAGGCGACTCCGTACCCAAGGGGACTCGAACCCGAGACCTCTTGGTTAAGGATGAAGGAGTACTTACCACTCCACCACAACCCTTGTTGGT

>rnd-1_family-213#LTR/Gypsy ( RepeatScout Family Size = 439 Final Multiple Alignment Size (possibly truncated) = 98 Localized to 2361 out of 4218 contigs )

GGGTCTTGTCCCGACATCCATCTTAGTATTAGAGGCTTCATAGACAGTTAGTCAGTTAGTTTTGAGTCTCTCATCTATGTTT

>rnd-1_family-355#Unknown ( RepeatScout Family Size = 227 Final Multiple Alignment Size (possibly truncated) = 98 Localized to 2361 out of 4218 contigs )

AATACAGAATTCGAAAGAAAATATACAAGTCTCAAATGACNTTGTTTCTAGAATAGAACAAGATCGTAAATAAGGGTAACGAGGGCCGTCTGGGATGACAAACAGCTACCTCNCAAGTATCTCCCGAAGCTCAATCTGAAAGAAAATANCAAGCCGAATCCGAGAGGGCCGTAACCTACANAAGTGTAGAAGCAAGGGGTGAGTACCAAACCACACGGTACTCAGCAAGTAAACCTCTAAACACAAGCTAAGGGGATAGAATACGAGTACTCCTTNCACCCCAACCGAACCTCCGCAACTACAACCTGCATAAAACCAGCCCAACCTAACAGTTCACAATCAACAGCCACAACCAACAGTATATCCTCAACAACCTCATATCAACAAATAAGATCAAGTAGATCAAATTTCACATAAGAAGGGCAAACNGATAAGATCCACACATCACAGACAAAGATGAAGTCAAATGCGATGCAAAATGCAATAACGATGTCANGTAGTNGTGATGCATGTCTGNCCTACGATACACATCCGTTGACGTAATCAGTCCGCGCTGAATACTCAAATCAGAACCCATGGGGGCCGCACATGGACCATGTATCGCCGGCCGGAGCCAGATCCCATCGGCATCCAAATCGCTAGCCGGAGCTAGTCTCAACTGTGTCTCATATCCATCCATCCTCATATCAGTGTCTCAGAACTCATGCAACAGATAGTTCACACATGGGATGAATAATGAATATGCACATGTCTCAATCACAACCATATCACGACCACATCATAGTATTACACATCATCTGTCTCAATCACAACCGTATCACGACCACATCATAGTATTACACATCATCTGTCTCAATCACAACCATATCACATGACTTATTCAACAAATTCAAGTCACATATAACACATTTAGCTCGTTTTATTCCCCATCTTTCATTTACAACAATTCCAATCAACAAATAAAACCCATCCTCAATCCCCATTACTCCCCAACACAATTAGGAAAGTAGTCATGCGTAGTCTAAGAGTTTAACAAAGTTTAGAAGCCACTTGCCTTAAAACACTCCAACAGTCACTCGAGACTTGAGCCTTNCCCTTCCGAGCACGNTCCGAATCACCGCAATCTATTCAAACAAGTAATCACAATAACAATTCGAATCCAACGACACCAANATCANGAAATTTAGGGAAAAAGGGCAAAACGGTCCAAAAGTCTCGTACCGGAAAACGATACCCAAATCTGGAAATTTTT

>rnd-1_family-254#Unknown ( RepeatScout Family Size = 351 Final Multiple Alignment Size (possibly truncated) = 98 Localized to 2361 out of 4218 contigs )

AAGGGTTGTTCGTGTGTTGTAATGGCGAGAAGTGGGGGTAGCGTACGTGATGTAAATGCTTAGGTGGATTTCACCTTGGAGAAAGGGATTCGTGTTTAGTGGTTAGNAAGCTTGACGTAATGTACTATGTGGCCATGGCTGGGTAATATGAAGAGGAAGTAAGGGTATGNCGTGAGCGGTTAGTCGGAATGCTNGTAGTTGTATTCGGGGACTCTTCTATGCGTTAGAATGGTTAGGCTATGATCAAGAACCTAGTAAGTAGGTTGACCGAGAGGGTGGTTAGTAGTAAGGGTACTAGTGTAATTCGGAGGTATTCCGGAGAGGGTAAAGGTGGAGGAGCGAGTGGTTTATTGCATGTTCTTGCTAGTTGATTTCTTATGTTATGCGGTGGGCGTCGGGTTGACCGATGATGCCTACCAGTACGTGTGGTTTGTACTGATACTACTCTTGCTATGCCTTTTGGCATAGTCTGGNTGCAGGATCGGTGATGTTTCATTAGGTGAAGACGAAGANAATCTCCAGCAGCTTCTACTTTTCCTTCCGCGTGGTGGGAGCTGTGTCTTTTATTTATTTTGTCCAGTTGTACTCTTAGTAGCTCTTGTACCTGTTTAGACTAGATCCTTGGGGGTGTTAATTACTTTACAAGTCTTAACTCGAATTGTTTAAAAGTTTTATTTATAAAAATCTTATGATTTCTTTATTCTCCGTTTTTCTTAATTTTCCGCATGTTTTAACTATTGGGATATGAGGGTTCTCCTACTTAGGTGTTAGAGTAGGTGCCCGCACGGCCCGGTGGGTTGGGTCGTGACAAATTGGTATCAGAGCCCAGGTTACCGGTCTCCCAGTACAAGAGCAAAATGTGGANTAGACTCCTGCGGATTGGTGTGTTGACGTCCACATCTAATCTTCAGGAAGCTANGAAGCATTCTAGGAAGTCGTTCCACTTNTTCTCTCCTTTCGTGCGATGTGTCGCTTGTGGTATGAGTTGAGTCCAATTGGTATCTCCCGATTCCAATTGGTATCTCAACGAAGTGAACTAGACGTCGTNGTCACGGCTGAAGGGACACAAGCAAGGTAAATTGGAACTGAGAAGGTTCCAATTGGTATCTGGCCCT

>rnd-1_family-348#DNA ( RepeatScout Family Size = 234 Final Multiple Alignment Size (possibly truncated) = 98 Localized to 2361 out of 4218 contigs )

TTATATTAGTGTATCTCGCGCATCAGATTAGTGTATCATGTATAAAATGTACATCAGATTAGTGTATCATGTATAAAATGTAATGTATCTTACTTAACGATTAATGTATCTCGCGCATCAGATTAATGTATCAGCGCTTATATTATTGTATCCGTTTGAGGGATTTCTGTAATTATAAACTTTTAAGGGATAGATTGTAATTTTGCCTTAAAAGTATGTGATTTCTGTAATTTGCCCTAAATTAAAT

>rnd-1_family-164#LTR/Gypsy ( RepeatScout Family Size = 541 Final Multiple Alignment Size (possibly truncated) = 98 Localized to 2361 out of 4218 contigs )

TTTCAATGGTTGATTTATGATGNATTATGATTGAATTGATGATTTAATGTTGATTTATGATGAAATTCCCCCANGAACCCATGATTCCCCCANATTNCTAAATTGTGANTTCGTGATGTGGGTTGATTGATTATGGAAGCATGTTAATNGATTATGTCTATATTGATTTAGTTATTGAATCATGTTATTATCCATGTCTAATGTAGTATTCTAGATGTTTGGGTTGAGANTGATTCATGGCCTTGAAGGGCAAATTATGAAGGTTNATGTATGATTATGATGNTTATGTATATGCNTGTGATTTCAATGAAAGTAATGTGATCATGNCTANAATGTAATTGTGTTATTATTGAAAGGTTATTCTCAATTATACACTTATGAATGATGATATATGTGAAAGGTTTCTTCAATGTAATGTTATATCTTGAATTGGTAGGCTTAGGGCATCCTCTTCTTGTATAAATGAANGTGATCTTGATTGATGAACCTTGAATCGGTAGGCTTAGGGCATCCTCTTCTTGTATAANTAATGTATCTTGACTTAATGAACNTTGAATCGGTAGGCCTAATGGTGGT

>rnd-1_family-296#LTR/Gypsy ( RepeatScout Family Size = 289 Final Multiple Alignment Size (possibly truncated) = 98 Localized to 2361 out of 4218 contigs )

GGTTAGAGTCGTAAAATGACTTCCCGTACTTAGAATGAAGGGTTTAGGGGTTAAACGTCAAGGTACGACCCCCCAAGGACCANCCAAGGGTCCTTGAGGAGGACCCCAAAACTGGCCCNAAAAGCTGTCAAAAACAGCTTGACCCACGGATGGGCACCCACGNGCCGTGGGTGGACCCACGCCCCGTAGGTGGGGTCGTGGGTCAAGGCTGGA

>rnd-1_family-94#LTR/Gypsy ( RepeatScout Family Size = 767 Final Multiple Alignment Size (possibly truncated) = 98 Localized to 2361 out of 4218 contigs )

CTCTTGTCACTTTGCATTGTCGTGTTGGTATCCTTTAAGACGTTAAAGACGCTTGTGTAACTCGCCCACTTGTACGGATTGTTTGATCACTTGGCACTCTTGTTGGGACCTTGTCCTTCTTGTGGCTGTGACTTTGTCACTATTGGCATGCCTCTTGATTCGGATCATTTGCCATCTTGACTCTGGATTTTTAGTCCGTCTTAAGAATGGAAGTTGTCCATTTTAAGTACGAACTAGAAAGCCATTTTTAATATGGTTCTTGGTTCTCTTGATTATTGGGCTTTGACCCTTCTTGATACAGGGCGTCGGCCCTTCTTGATACCTGCTTGTGCTTGGTGTGACGACATGATGTATATATTGGGCGAGCCTTGTTATTGAATCTCTTGGAAANTGGTGCTATGAGCGTTCTTGACACTTGGATCTTTAAATATCGAACTTGATACCCTTAATGTGCACTCTAGTTACTTGATAGGTTCTACAACGTGTAGGTAGTAGTATGGGATGCTATCTACACATGGCACGAGTAGGCTTTGAAGGTGCTAAAGTGAGTTCCCTAAGTCTAACGACTAATGTGTGAAAGTCCCCTAAATGCATGAATGTGTCCTAAATGANTTTAATGAAGAATGTTGACTTATTGTCTAAATGATATGTTGAATGAATAGCTTATATTAATGAAGTGAGTTACTTAGCATGTTACGTTGAAGCGTGATGGTTCTTGTCTATNGTAGCCTTGAGGAACACTTAGGTGTGTATGAAGAGGTNGTATGGGCNGCCACTTCATGTCTCGCTCAAGTGTGTCTTAGGGTNACTTTAGATGAAGGTCCTTGGATGATGAAATGANTTACTTGGTGAAATGTTGAGTATGTCTATAATGTGGTTAATGNCTTGTATGTTGGTTATGNCTTATATTATGCCTCTTATGTCAATGTTCATGAAGTTTTACCAAAATGGCATAAAGCATGACTTTCAACCAAATGTCCTTTTTAGCATGTTTTTGCATGGCTTCCATACTTAGTACTTAATTGTGCTAACCCCTTTCTTTCCTTTTGACTAAAGTGTAGGGATTTGGAGATGTTGACGTTCCATGGTTGATGGATAGGTTTCTAAGGTGCTTGAAGATGAAGACTTGGTGAGTCCTCATAGATTCGAGGACAATACCCAATATGTTCCTTTATGTCNTTTTAGTTTTGTTTAAGTTTTGTACGGGCTTAGTCCCGAATGTTGTACTCTAAAGTATTAGATGGTTTTGAGACATCGTGTATAAAGTCTAGAAAGTCTTCCGCTTGCTATTT

>rnd-1_family-315#LTR/Copia ( RepeatScout Family Size = 265 Final Multiple Alignment Size (possibly truncated) = 98 Localized to 2361 out of 4218 contigs )

TGGAAAGAGGCTGTCAATAGTGAGATCGAATCAATCTTAAGCAATCATACTTGGGANTTGGTTGATCTNCCTCCAGGGAANAAACCNTTGGGTTCNAAATGGGTCTTCAAAANGAAAATGAAAGCCGATGGNACTATTGACAAATATAAGGCTAGACTTGTTGCCAAAGGNTNTANNCAAAAAGAAGGNNTTGATTATTNTGANACNTTCTCNCCGGTNGCNAGGATNACATCCGTTCGGATGTTAATTGCNCTAGCTGCAGTATATGGTCTTGAAATNCATCAAATGGATGTNAAAACCGCNTTCTTAAATGGAGANCTGGAGGAAGAAATTTATATGGAACAACCCGAGGGTTTCGTAGTTCCNGGTAAAGAAAAGAAAGTGTGTAAACTCGNNAAGTCNCTNTATGGNCTNAAACAAGCNCCNAGACAATGGCATGNAAAGTTTGATCAAACCATGTTGTCAAATGGATTTAAGATCAACGAGTGTGATAANTGTGTTTACATTAAAGACGCTCGGATCAGGAAGTCATTGTTTGTCTGTATGTTGATGACATGTTGATAATGAGTAAGGATATTGCCGATATAAAAGCTACTAAGCGCATGCTCGCTAGCAAGTTCGATATGAAAGATCTNGGAGNNGCCGATTTGATCTTAGGAATAGAAAT

>rnd-1_family-241#SINE/tRNA ( RepeatScout Family Size = 377 Final Multiple Alignment Size (possibly truncated) = 98 Localized to 2361 out of 4218 contigs )

ACCCAAGGGTGTGGCCTAGTGGTCAATGAAGTGGGTTGAGAACCATGAGGTCTCAGGTTCAAATCCCAGCGGAGACAAAAACACTAGGTGATTTCTTCCCATCTGTCCTAGCCTTGGTGGACAGAGTTACCCGGTACCTGTTGCTGGTGGGAGGTGGCAGGTATCCCGTGGAATTAGTCGA

>rnd-1_family-202#LTR/Gypsy ( RepeatScout Family Size = 471 Final Multiple Alignment Size (possibly truncated) = 98 Localized to 2361 out of 4218 contigs )

TTGTAACACCCCGGAAATTCTATGACTTAAGTTAGAGCCTCACCTTATGAATAATGACTTAAAAATAGTTAAAATGATGTTTCTAAACCTAAATTAATGCAATTGACTCGGTTTGGAAGAGTTTGGAAGTCTAAACGTCAAGGGACGACCANGACGTCCGGAAACTAGNCNNTNACGTGTTCGGTGTGTTCTAGTATGTTGTTGGGCATGTTTNNGNGTGTCGTNTGGNGTCTANAATCAGTGGAAAGGTGACCCTAGTGTCTTAATANATGTTTTTAGGGTCAAAACGTTCGGATACGACTCCCCGGGGCCACCCTAAGGGTCCCCGAGGAGGACCCCAACCTTGGCCAAGCAAGCTGCCAAGGCAGCTTGCAAATTGCNCTTGGAGGGAGCGGCTCCGCGTCGCGGACTNGCTCCACCAAGGACAAAAAGTTCGGTTCGCGNCGCGGNCGCGTCGCGGACTCTACTGTCTCGAAATTTAATTCCAGANATCTTGNGGGAACACCTCCGCGTCGCGGACTTGTTCCCCGACGAAATNTGCGAAAATTCAAAACTCGAGTTTNACTTNNTTAAAGAGTCCAACTAAGTGAGGGGTATTTNGGGTACTTTAGGGGACGTGTATATAATGATTTTTAAGTCNACCTACCTCANGGTATTTTATCGCTCAAACACAAAACCCCAAAGATCAAAACCTAAAGTTCCTCCTTCTCTCTAACTCTCTCTCTCTTCTCAAGAACTCCATTGAAGAACATTGGAGCTTCAAGAAGAAGACCGACTCTCCNAGGTTTCAACTCGGATTTTCGTGGTTAATTCGTCAATAAGGTATGGTTGCTTATCACTCTTGGGATTCCTTTCCCCAAGAGGCCCCTTCAAGATGAATTTCAAAATCTCCAATTTCTAGGTTTCAATCCAAAAACGTGGGTCTTCTTTCTAAAGTGAAATTGATGTTATAAACTGACTTTGATTGATGTTATATGAACAATTATGGATGAATTTATGTTGTATTGGTGGTTTCTTGAAGAATTCCCCATGAGACCCATGTTTCTCCTTCTTTTCCTAATTCTAACCCTAGTTTGTGTTGGATATTGATTGAAGGCTATGAATTGAATGTGATGTTATGTAATTCATGTTTGTATGATGATTCTACCCTAGGTTATGTATAATTTCTATGAAATTAGGGTTGAGTCTTTGAATGAAGCCTTGAAGGCTATGAAGGGAATATGTCAATTGCTTATATTGATGTTGATTATGTTATTACTTCATGAATTACCTTGTATTATGTATTGGTTATGAATTGTTTGGTAATTGAAGTATGGATGTCCCACGAAGGGCAAGAAGGTATGAAGGTTGGTTCTTCTTTGAACTCAAGTATGAAAGGTGAATTACTTAGATTGTAATGATGCTATACCTAATGTGTTGTTGTGGTGTTGATGACCTAGTTTCCTCATCCTTAACCCTCTACACTTGAATTAGCTATGAAGTATGTATGTATGTTATGGTATGATTGCTATATGATTGAAAGGTAGTTCTCATGATTATAGTGTAATGTAAAGTGAAAGGTTTACTCACTTGCTAAGTGT

>rnd-1_family-195#Unknown ( RepeatScout Family Size = 482 Final Multiple Alignment Size (possibly truncated) = 98 Localized to 2361 out of 4218 contigs )

GGCTGGCGCGACGCGCCCCCGTAGCGCCAGGGGGGCTGGGGCGGCGCGCCAGCAGTGCGCCAGAAACGAGCCCCTGAAGTTTAGGGGCTGGCGCCCCGCGCCAGNAGCGCGCCNGGGTCGCCTGCCCCACCCGTTTTTCGTCGTTTAGCCCGTTTGAGTCCTTCTAAAGTGTACCNNCACTCCCTATTGATTCTAACTACTCTAAACGACTTCTAAACATCTAGAAATCATCCATAAACATGAATCATAACCTTGAATCCATAAATTCAAATTCAAGGTAGAGTTAAGAGCCAAGTCTNGAGAGTTCTTAGAGTCTTT

>rnd-1_family-493#Unknown ( RepeatScout Family Size = 130 Final Multiple Alignment Size (possibly truncated) = 98 Localized to 2361 out of 4218 contigs )

TAATACTTGTCATTTCACAAAATCAATGCATAAATNACANTATTCTTCCTATTTTACCCTTGAGAGTTATTAGCCTTGAAAATATGNATTTGACCAACATAGGAAAACTAAATAATTAATTCATGTTCATTGGTCAAATTAATTAATCAAAATAAATTAATTCATGTTCATTTATTGAAAACTTGACTTCAAGAGAACACTAAATAAGGGTANAATGGTAAACTTACNTAGTTTCTTAAGGNACGTGAAATCTAAAATGGTGACATATAAATAGGAACGGAGGGAGTAA

>rnd-1_family-527#LTR/Gypsy ( RepeatScout Family Size = 119 Final Multiple Alignment Size (possibly truncated) = 98 Localized to 2361 out of 4218 contigs )

AAAAATATTCGTTTCAATTAAGAATGTGGTTACACATCTTTTTTGAGACACTTAAAAGAAACTCAAGGATGCGGTGACGCACCTTGTCCAATAATATAATTAATTTTTATTAATTTAAANTAAGACTTGTAGATAAGATTATTAGGTGTGATGCGAAGAAGACAGTTATGTCTCTAAATGTCAAGACCGAGAATGCGGTTACGCATCTAGGTTGAGACCAAATAAAAGTTCAACAATAAAAAATAAGCGAACCATGGCCTGTAAACATAATATATCCAAAAATANTTTAAGATAAGTATAAGTCAATAAAAGCGACCGTGCTAGAACCACGGGACTCGAGGGATGCCTAATACCTTCCCCTCGGTCAACAGAATTCCTTACCCGAATTTCTGGTTCGCAGACCAAAATAAAGAGTCATTTCCTTTTGATTAGGGATTCAAAAAGGTGACTTGGAACACCATAACTCAATTCCAAGTGGCGACTCTGAAANAAAATNAAAATAATCCCTANTCAATACCGTCACTTAAATTGGAAAAACCCTTCC

>rnd-1_family-112#LTR/Gypsy ( RepeatScout Family Size = 695 Final Multiple Alignment Size (possibly truncated) = 98 Localized to 2361 out of 4218 contigs )

AAGTCTTCCGCTGAGTAAGTAAGCCAGGCCAAGGGTTCGCTTGGGGCCAGCAATGGTTCTCGAGTGCCGGTCCCGCCCAGGGTGTAGGCTCGGGGCGTGACAAACTTGGTATCAGAGCACAGAGTTCAAGAGTCCTAGGGAGTCTATGAAGCCGTGTCTGTAGAGTCCTAGTTATCGGTGTGAAGCGCGCCACATCTATAATTAGGAGGCTGCAACATTTAGGAAATNTCTCACTTCTTTCACACTCATTTCGTGCGTTAGAGTTTATCTCTAAAAAGTTTCTTTCTAATTCGTGCTTGCGCGT

>rnd-1_family-72#LTR/Gypsy ( RepeatScout Family Size = 893 Final Multiple Alignment Size (possibly truncated) = 98 Localized to 2361 out of 4218 contigs )

GCCTACTTGCGCCAAGTGTGGNAGGAGNCATTATGGTAAGTGCCTAGCCGGCANGGAGGGTTGCTATGGTTGTGGNGAGAGTGGNCACAAGATGAGAGATTGCCCGAAGGCTAAGGCTAAAGAAGGGAGGGTAAGCAAGTTGCCTCTAGTGGTCCGGATGNGGAGCCTCAAAAGAAGAATAGGTTTTATGCTCTCCAATCTAGAGAAGATCAAGAGTGATCTCCTCATGTCGCTNCCGGTATGTGTTTTATGCTTTAGTAGATTTCCGGATTTCTTGTCTTTTGTGACTTGNTATGTTGGACTTTGAAGTTGTCTTTAGTATGAATGATGTTTAGAAGAGTTTGTGTAGGCTTACTCCCAAGGGGGAGATAGTGTGCCTATCTAGTAAGGTTATTGTGTGTTTTATGTGTTGCCATGATTTCCTTATATGCATGTTCATGAAAATGCTTANGTCTTGNNAGAAATGACTTATATGCATTGTTTTCCTCATGTTGTAGTGCATTTGAGTATGAGTTGCNTATAGACTTCATGAGATGAATTATGAGCATGCCTTAGCTTGGAGTTGAGAGATTCCTCCTTGAATANATGCTTTTTGAGAGTAAATTGCATATAGGCTCCATGAGATTGTGTTGAGCATGTTTTAGCTTGGAGATGGTAGTTCCTCCTTGAANATGAGTAAATGTNGAATTTCATGCATATTGGGTTGTTAGATGTAGTTCCTACTTCCTTGTGTTGCATTGAGTATGTTTAGNTTGGAGTTGANGTTTCCTTCCTTGGTTGTGTGCATTTAGATGTAGTGCATGCATGATGGGCTGCTAGTCTTGAGTCTTTACTTTAAGAAGTGTCTAGAACGTCATTCGAGGACGAATGTTCCCAAGGGGGAGATANTGTAACACCCCGGAAACTAGTAGGCTAAACTAGAGAGTTCTAGAGCCTTT

>rnd-1_family-162#LTR/Gypsy ( RepeatScout Family Size = 543 Final Multiple Alignment Size (possibly truncated) = 98 Localized to 2361 out of 4218 contigs )

ATTGTAACATCCGGCAATTTGAAATAACTANGAAGAGGCTTAGAATTGGAAATAGTCATTTTTGGAAAGAATTTGAAAATCTGGAAATTTGGCTAAGTATGGGAAAAAGGTGAGTTTTTGGCCAACTTCGAGCGGTCATAACTCCTAGCTCAGGATGAGTTAGGNGTAGTTCCAGTTATGGTTGCGAAGCTCGTGGAATGATCTTTCCAACGCCGCCGAGTTTGCTCGATTCCGAGTTCGTATGAGCGAGTTATGCCCTTTGGAAGTTGGGCAGTTGGCAGGGAAATCCGTCCGGAAATTTTAAGGGCATTTTGGTCTTTTCCCTAGCCANTTCTTTTGGTTATATTGTTGTGTTAGGCTGATTTTTGGATCAGTTTGTCCCNTTTTAAAAGAGTGAGAGTTAGGGTTTGAGAGTGAAGAGGAGAAAGAGAAGA

>rnd-1_family-114#LTR/Gypsy ( RepeatScout Family Size = 680 Final Multiple Alignment Size (possibly truncated) = 98 Localized to 2361 out of 4218 contigs )

GTAACACCCCGGAAAATTTTTGAGCTAAGACTCGAACCATCCTTCGTTGTGAGTAGGATTTTACCGAGGAATTTAAAATTTCTTAAGTGTTAAGGTCACTAGATGTAGCACCTTGAGTTCCAAAAAGAACTAAAGAGAATTCATTCAAGTCATTCCTAAGTTCTTTTAAGTTTTGGGTCAACTTCAAACGACCATAACTCTTAGTACAGGATGAGTTAGGTGNNCCACAAGATATCAAATGAAAGGTCTTTGAATTATCTTTCCAACGCCACCGAGTTTGCTAAGTTTCGAGCTCGGATGAGTGAGATATGCCCTTTTGAAGTCAGGCTGTCCAGTTAAGGAAAGTTACCCGAAAATAGTGAGGGGTATTTTGGTCTTTTCCTTACCCAATCAGATTTAATTCGTTTTTAGTAAGGTTTTAGGGGTCTAATCCGATTAGGTTCAGTTTTATAATCCTAATATACGCCTAGGGTTTTAGTTGAGAGTTCAAGAAGAGAAAAGAAGAGAAAAGAGGAGA

>rnd-1_family-153#LTR/Gypsy ( RepeatScout Family Size = 575 Final Multiple Alignment Size (possibly truncated) = 98 Localized to 2361 out of 4218 contigs )

AGTGGTGGTTGTCGGTTAGAGAAACTCCCACAGCAGTAATTGCATGGTTCCTCAAGGGGTTCTGCTTAGTGTGAGTGGGGGTATGGGACTTCATTCATGCATTGCACAAGTAGACTTTGAGAGGGGGCATGGTATGTCTTTTTATGATATTATGATTATGAGTTGACGNCATGTTTTAAACAGTTTTCGCTCTTTATATTGCACTTGTTTTAAACTGCTTTATAATGA

>rnd-1_family-452#LTR/Copia ( RepeatScout Family Size = 150 Final Multiple Alignment Size (possibly truncated) = 98 Localized to 2361 out of 4218 contigs )

ACTTTTNCTCCCGTNGCCAAAATGGCNACCGTNNGANCNNTNATTGCTNTNGCTGCTTCCAAGAATTGGCCTNTNCATCAAATGGATGTCAANAATGCNTTCCTNCATGGTGATCTNNANGAAGAAGTNTATATGGAGCAACCACCTGGTTTTGTTGCTCAGGGGGAGTATGNGAAAGTNTGCGANTTGAAGAAGTCNTTGTATGGNCTGAAACAAGCTCCCGGGCTTGGTNTGGNAAGTTTAGCGAGGCNGTTCAAGATTTGGTTTCAAAATGAGCAAATNTGATCACTCAGTCTTCTATCGACAATCAACAGCTGGCATTGTCCTTCTTGTTGTNTATGTNGATGACATTGTTATCACAGGAAGTGATTATNCGCGGATCTCATCCCTCAAGTCTTTCCTGCATACTAGATTTCATACGAAGGACTTGGGNCNNTTACANTACTTTCTGGGNGTNGAAGT

>rnd-1_family-413#Unknown ( RepeatScout Family Size = 174 Final Multiple Alignment Size (possibly truncated) = 98 Localized to 2361 out of 4218 contigs )

TGTTGATCCAATTGAGTGGATTATGATTGTTTATGATTGAATTGATAGAATTACATGTTTTTAGGATGAANTTACATGTACCCATGCCTAACCCATGATTCTAGGGTGAAATTGATGTAATTGGGTTTATGCCATGAAGGGGAAATTTATGGGTT

>rnd-1_family-330#LTR/Gypsy ( RepeatScout Family Size = 252 Final Multiple Alignment Size (possibly truncated) = 98 Localized to 2361 out of 4218 contigs )

TTGAATCGGTAGGCTTAGGGCATCCTCTTCTTGTGTAATGAATGTATCTTGACTTAATGAACCTTGAATCGGTAGACCTAATGTTGGTAGCCCTTTCAT

>rnd-1_family-46#LTR/Gypsy ( RepeatScout Family Size = 1110 Final Multiple Alignment Size (possibly truncated) = 98 Localized to 2361 out of 4218 contigs )

AATGNATGAATGTGCCCTAAATGAAGCTAATGAAGAATGTTGACTTATATGCTTAAATGNAATGATGCATGCTATACTTGGATTGTATTATGAGTTACTTCCTTGTCATGACTTATTGAATATGAATGTGCCTTGTCTATGGTGGCTTCAAAGGAGTACTTAGTGTGAGTAGTAGTATGGGATGCTACTCGCACATTGCACAAGTATGACTTAGGGTTGTCTTAGGTGATGGTCCTTATGTGATGATATGNCTTATGNAATGTTTATGTCTCTTATGTATGAGTATTGTGTAAAGGTGGAAAGGATGAATGGTAAGTTCACTTTTGGTGACCTTAANGTGGTACCTTAGTGTGGATGGTGGTATGGGACGCTATCCATACATTGCACAAGGTATAGCTTGAGGGTTACTTGAGGTGAAGGCTTAA

>rnd-1_family-567#LTR/Gypsy ( RepeatScout Family Size = 103 Final Multiple Alignment Size (possibly truncated) = 98 Localized to 2361 out of 4218 contigs )

TTTATTTTTAAAAATTTACATTTGCTAATAACTCACAAAAATTTTCCAGTGAAAACTGGGGCAAAATNTTTTGTTTATTTCTTTTGTTTGTGATGGTTTTCAGGTTTCTTCAAGCGAAACATGGATTTGAAATTCAAGAACAAAGTTTCAACTCTTTTCAGAATGATCAATCCCATGATAGCCAGAATCAACTTCTTCAATGCATGTAGCAGCCTGACTTCCTGACATCTATTTCTATTCAGCTTCTGATCAAGAAAACATGCAGTCATTCAAGAACATTTTCAAATTGTCATTTTGAAGAGTGCCAAAAACTCACTCTAAGTGGCAAGTTAAGCATCGAATCCAATTCAAGATCAAGATACTCACCCAAAATCAAGGTGATATCTCAAAGTCAAGACTCAAGTGAAGACTCAAGAGAAGCTGCATAGATAGGAGCTTGTACTTTTATTTTCCTTTTGACTATTAGCTTTCATTTTTTTTATGTAAGGATAGGAGCCGCGACCTGGAGCCTCGATGGAACCTCACTTAACTTCCAACTCATCACCTCATCTCCTCGAACTACACGTGACCTGATTCTCNTATAACCCGGGATATGTAGGATGTCCAAAACCAGGGCTCGGTCGCACAAATTTTTCTTTCGAATAAGAGTCCGGTCAAAACTTGTCGCGTTGTCTACTTCTTTGTCTGAAAACTCTTCGTGTTTCCAGTCAAAGAGGGGCAAACTGTAGACACGTAATTTTTGACCGAACTTAAGGTTTTACACCATTTTTAGNGCTTAAATATTTTATAAAAATCTAAATTAAATATTTTGACTTTTATCTTATTTTANTAAGTTTATTTTAAAAGATTTGAAAACAACAAAAAA

>rnd-1_family-401#Unknown ( RepeatScout Family Size = 185 Final Multiple Alignment Size (possibly truncated) = 98 Localized to 2361 out of 4218 contigs )

AAGTGAGGTCGTTTTGCCTAAGCCTAGNACACCTATATAAGTNATTTTAACCCNTTAAACTCACCATTCAAGTCATTATTTCCAAAACCCCAAAAGAATCCCAAAGTTCTTCCTCTCAAATATTTCTCTCTCTAGAA

>rnd-1_family-534#Unknown ( RepeatScout Family Size = 117 Final Multiple Alignment Size (possibly truncated) = 98 Localized to 2361 out of 4218 contigs )

TATTGGGTTATTGGGTAAACCGATAACCCATTAAGACNATAGTAATTTACTACTTTACCCGTACATAAATATTAAATATTAATCTCAATACCTTACTAGTTACTGTCTTTGTCCTTTAGCCTTCAATTCACACTTCACAGTGACTTTGAGTTCACAACTTCACATTATACAAAACGTCAAAACCTAAAGTAAGAACCATATTCCTCTTAATTTCTCTTTGTGTTACACTTGTATAGTTCTTTTCATGTTAGTTATTATTGTTTCTATTTTATGAGCGTTTGTAAAGTTACATTATTGTCTTGTCGCGTCAAATTTATTGGAGAAGTCATATATTTGTTTTATAAGCATTTTCTTATTGGTTAAACCGAAAACCGAACCGTTAAGGACCAAAACCGATAAACCGAAAACCGATAAAAAA

>rnd-1_family-59#LTR/Gypsy ( RepeatScout Family Size = 1004 Final Multiple Alignment Size (possibly truncated) = 98 Localized to 2361 out of 4218 contigs )

AAGGGGGAGATGATAAGATGAGAAATCAAGTCAAGTGTTGAGAATTTCTTAAGGAGCTGGTTAGTAGGAGTCCATGGAGTTGTTAGAGTACTAAGCTTGAATGTGGTGTTGTTAAACATTAGGTGGTTCTTGATATGAGCCCAATGTGGTGGTAAAGAGTAATAGTTTTGGGATGAGATTCCCTATAGAGAGGTATAGAACCCGAACCTAAGGATTTGGGTTAAGCTTGAATATAGTAAGAGCATACCATTAGACTCAGACCTTAGTAAGAGTAACCCCATTCCATACCCAGTCCAGTCGTCATTCGAGGACGAATGATCCCAAGGGGGAGATATTGTAACACCCCAGAAANTTTTTGAGCTAAGACTCGAACCATCCTTCGTTGTGAGTAGGATTTTACCGAGGAATTTAAAATTTTCTTAAGTGTTAAGGTCACTAGATGTAGCACCTTGAGTTCCAAAAAGAACTAAAGAGAATTCATTCAAGTCATTCCTAAGTTCTTNTGAGTTTTGGGTCGACTTCAAACGACCATAACTCTTAGTACAGGATGAGTTAGGTGNNCCACAAGATACCGAATGAAAGGTCTTTGAATTATCTTTCCAACGCCACCGAGTTTGCTAAGTTTCGAGNTCGGATGAGTGAGATATGCCCTTTTGAAGTCGGGCTGTCCAGTTAAGGAAAGTTACCCGAAAATAGTGAGGGGTATTTTGGTCTTTTCCTTACCCAATCAGATTTAATTCGTTTTTAGTAAGGTTTTAGGGGTCTAATCCGATTAGGTTCAGTTTTATAATCCTAATATACGCCTAGGGTTTTAGTTGAGAGTTCAAGAAGAGAAAAGAAGAGAAAAGAGGAGAAGA

>rnd-1_family-10#Unknown ( RepeatScout Family Size = 1502 Final Multiple Alignment Size (possibly truncated) = 98 Localized to 2361 out of 4218 contigs )

AGTCTTCCGCTGAGTAAGTAAGCCAGGCCAAGGGTTCGCTTGGGGCCAGCAATGGTTCTCGAGTGCCGGTCNCGCCCAGGGTGTAGGCTCGGGGCGTGACAAACTTGGTATCAGAGCACAGAGTTCAAGAGTCCTAGGGAGTCTATGAAGCCGTGTCTGTAGAGTCCTAGTTATCGGTGTGAAGCGCGCCACATCTATAATTAGGAGGCTGCGACATTTAGGAANTATTCTCACTTCTTTCATACTCATTTCGTGCGNTAGAGTTTATCTCTAAAAAGTTTCCTTCTAATTCGTGCTTGCGCGTGTCTTTCAGATAATCATGCCTCCACGAAGAGCTGTCAGAGGTCGTCCTGCTAGGAGGAATGTTGAGGANCAAGGGGTACCCAATGCACCNGAAGTGCAACCCCAAGGAGAGGTCACCAATGCTGAGTTCCGNGANGCTATCCGGATGTTGAGTCAAGTTGTGACCAACCAGGTTGGGCAACAGAGAGGGGCTCGACAGGAAGTGGCTGATACTTCGAGGATCCGTGAGTTCTTGAGGATGAATCCTCCAAGCTTCACTGGTTCGAGCGCCACTGAGGATCCGGAGAACTTCGTTGAGGAGCTGCAGAAGGTNTTTGAGGTNATGCACGTTGCCGATGCTGAGCGAGTGGAACTAGCTGCATACCAACTGAAGGGTGTCGCTAGGACNTGGTTCGACCAGTGGAAGAAGAGTAGAGCTGAGGGTGCACCACTTGCGAGTTGGGCTTGTTTCGAGGAGGCCTTCTTGGGGCGTTTCTTTCCCCGNGAACTGAGAGAGGCNAAGGTACGAGAGTTCCTCACNCTTAAGCAGGANTCTNTGAGTGTTCATGAGTACGGNCTGAAGTTCACCCAACTNTCCCGCTATGCTCCGGAGATGGTTGCGGACATGAGGAGCAGGATGAGTTTGTTTGTTGCTGGGTTGTCTCGTCTGTCAAGCAAGGAGGGCAAGGCAGCTATGCTGATAGGGGACATGGACATAGCAAGGCTGATGGTCCATGTGCAGCAGGTTGAGGAAGANAAGCTGAGGGATAGAGAAGAGTTCAGAAATAAGAAGGCTAAGACAGGGAATGAGTCCGGGCAGCAGAAGAGTAATGCGAACCGGTCGTCCTTTCAACAGAAGCAAAAGGGACCTGCTCCATCATCTGCTAGTGCACCTGCACCAAGGAACAAAGGTGAGTACAATAGTCAGAATTCGCAGAACTTCAGAGCTAGACCTGCNCAGTCTCAGGGTAGTGTGGCACAAGGGGGTAATNGGGCTCCTGCATGTGCTAAGTGTGGTAGGAACCACCCAGGTACGTGTCGTGATGGCTCCACTGGTTGTTTCAAGTGTGGNCAGAANGGTCACTTCATGAGAGAGTGCCCTAAGAACAGGCAGGGTAGTGGNAATNGGGGCAATAGAGCCCAATCTTCTTCAGTTGCTCCACCAGACAGAGCTGCACCTAGAGGAGCTACTTCNGGTACTGGCGGAGGAGCAAACCGCCTNTATGCTATCACCAGTCGCCAAGAGCAAGAGAATTCNCCAGATGTTGTCACTGGTATGATCAAAGTCTTTACTTTTGATGTTTATGCTTTGCTAGATCCAGGAGCGAGTTTATCTTTTGTGACTCCTTATGTTGCNATGAATTTTGATGTTCTTCCTGAGNAACTTCGTGAGCCCTTCAGTGTTTCTACACCTGTTGGTGAGTCTATTCTAGCNGAGAGAGTCTATCGTGATTGTNCCATTTCCGTCAATCACAAGAGCACCATGGCTGATTTAGTTGAGTTAGACATGGTAGATTTTGATGTCATTCTAGGTATGGACTGGCTTCATGCCTGTTATGCCTCAGTTGATTGTAGAACTCGAGTTGTCAAGTTCCAGTTTCCTAATGAGCCAGTCNTAGAGTGGAGGAGTAGTTCAGCAGTGCCTAAGGGTCGTTTCATTTCGTACCTTAAGGCGAGAAAGTTAGTTTCCAAGGGGTGTGTCTATCACTTAGTCCGAGTTAATGACTCTAGTGTTGAGATACCTCCTATTCAGTCAGTTCCAGTAGTAAGAGAGTTTCCAGAAGTCTTTCCAGATGATCTTCCCGGAGTCCCTCCTGAGAGAGAAATAGACTTCGGTATAGATATTCTTCCNGATACTCGTCCTATNTCTATTCCGCCATATAGAATGGCACCAGCAGAGTTGAAAGAGTTNAAAGAGCAGTTGAAAGATCTCCTNGATAAGGGTTTTATTCGACCAAGTGTCTCACCTTGGGGCGCTCCGGTCTTATTTGTGAGGAAGAAAGATGGTTCCCTTAGGATGTGTATAGATTACCGTCAGTTGAACAAGGTTACCATCAAGAATAAGTATCCTCTTCCGAGAATTGATGATCTTTTCGATCAACTTCAGGGTGCCACTTGTTTCTCTAAGATAGACCTCAGATCNGGCTACCATCAGTTGAGAGTAAGGGAATGTGATATTCCAAAGACAGCATTCAGGACCCGTTATGGTCATTATGAGTTTCTGGTCATGTCGTTCGGTTTGACCAATGCGCCTGCAGCGTTCATGGACCTTATGAATAGAGTATTCAAGCCTTATTTAGATATGTTTGTTATCGTNTTCATTGATGACATACTAATCTATTCGAGGAATGAGGAAGATCATGCTAGCCATCTCAGAATAGTTCTCCAGACTCTGAAGGATAGGGAGTTGTATGCTAAGTTCTCTAAGTGTGAGTTTTGGCTTGAGTCTGTGGCATTCTTAGGCCACATTGTTTCCGGTGATGGAATTAGAGTTGATACTCAGAAAATNGAGGCAGTGCAGAATTGGCCTAGACCCACATCTCCAACTGATATTAGGAGTTTCTTGGGNTTGGCTGGCTATTATAGAAGGTTCGTAGAGGGGTTCTCGTCTATTTCGTCCCCTTTGACCAAGTTGACTCA

>rnd-1_family-135#LTR/Gypsy ( RepeatScout Family Size = 618 Final Multiple Alignment Size (possibly truncated) = 98 Localized to 2361 out of 4218 contigs )

TGTAACGACCCTAAAAACGAACTAGTGAAACTAGAGCCTCACATGTGAGTTTGGAGTTGAGAACTTGATGAAATGATGAGAAATGACCGTGACGTCCGGAAACTAGTCATTTGAACTAGTGAGTTGTAAGGGTCAACTTCAAACGGTCATATCTTTTAGCACAAAATGAATTAGGTGGCCCATGACCTATCAAATTAAAGGTCTTTGAGTCCTCTTTCCAACGCCACCGAGTTTGCCNAATTCCGANCTCGGAGTAAAAAGTTATGCCCGNNNTAGTGAAGCCCTGTC

>rnd-1_family-101#LTR/Gypsy ( RepeatScout Family Size = 737 Final Multiple Alignment Size (possibly truncated) = 98 Localized to 2361 out of 4218 contigs )

AAAGGGTCATACTTTTTAGATGATTCCTTAGTGCTTTTTAGCATAGACTAGTGGATCCACTTAGTAGTTAGGTTCTATACCCTCGGCAAGGTATAGGACGGCCCTGGCAGCGTGAGGCGAGACGTTGTATCATCACATAGCTCNTAGTGATGGTTGTCGGTTAGAGAAACTCCCACAGAGGTATTTTGTATTCTTATATACACAGAGTTTATTTGTATTTTTATATACANNCAGAGTTTATATTGTATCTTTGCATACANACNGAGTTACTATCGTATTTTTAAATACACAGAGTTGNTATCCATGTTTTAAAAGCTTTNCTTTATATTGCATTCGTATTNTTGCTTTATATTGAGTTGAGTTAAGTTGAGTTGAG

>rnd-1_family-552#LTR/Gypsy ( RepeatScout Family Size = 108 Final Multiple Alignment Size (possibly truncated) = 98 Localized to 2361 out of 4218 contigs )

AAATGAAGTAGGATAAACTAGAGCCTCACATGGGTTTTATGCGTTGATAACTTGTTAAAATGATGAAGAATAGCCTTTTNAGTCGGTTTTGAAGAGTTTGGAAGTCAAACGTCAAGGGACGACCANGACGTCCGGAAACTAGT

>rnd-1_family-218#LTR/Gypsy ( RepeatScout Family Size = 433 Final Multiple Alignment Size (possibly truncated) = 98 Localized to 2361 out of 4218 contigs )

TAGAAGGNTTGGGAGTTGATGAGAACCTTTCTTATGAAGAGGTTCCGGTTGAGATTTTAGACCGGCAAGTCAAGAAGTTGAGAAACAAGGAAGTAGCTTCCGTGAAGGTNTTATGGAGGAATCACTTAGTTGAGGGTGCTACTTGGGAGGCCGAGGCCGATATGAAGTCCCGATATCCTCATCTTTTTCCTTCTACTCCTATTCNAGCTTGAGGTAANNAGTTCCTCTTGAGTTTTNTTGTTTTGGGAGTCATGTGTGTTATATGTGTTTTCCATGATTTCCTTATGTTATGCATGTTCTTGAGAAATTCATGTTTAGTAANAAAATGAGTTTTCATGATTTATTTTCCTCATGTTGTTGTGCATTNAGTATGAGTTGCNTATTTGACTTCATGAGATGANTTGATGAACATGCTTAGTTATGCTTTTGAGAGTAATNGCATATTGGCTCCATGATGTTGTGTTGAGCATGCTTTTAGTTGGAGAAGGTAGTTCCTCCTATGAATATGAGAAATGNTGAGTTTCGTGCATANTGGGTTGTAGATGTAGTTCCTA

>rnd-1_family-208#LTR/Gypsy ( RepeatScout Family Size = 454 Final Multiple Alignment Size (possibly truncated) = 98 Localized to 2361 out of 4218 contigs )

TGCACTGTAACTTTCGGCGGACAAACCCTTGCTCGCCGAAAGTAGTCGGCGATTCGCCGAAAGGGCCTTCCCATCGCCGACTTGCCNTAATTTTTNAGCCAATCCCTTCGGCGAGCCCGATCTAGCTCGCCAAAGAGGTTCGGCGACTCGCCGACTGGATCGGCGAGTCTGTCTGCAATAAATTTTCTGTGCTTNTGTTTGAATTTTNTCCTAATTTNTCCCGATCTTTTGCAGATATGGTACGCACAAACCTAAACGCGCCACCCCAGAAAAAAGCACGGGGCATTACNATNAACGAAGGGGGATCAAATCCTCCAAAGAGGAGAAGGGAAGANCTCCCACCGGGAGATAAAGGCAAAAGGAAGAAGCACATAGCTAGAAAAGGAGTAGCTATTGANNCCCAGGCTGNTTTNTCTGAGCCNGAGGACGACCAGCCTTTGNNGTCCCGGCGGGATGAGATCCGGGCTAGATCTCAGCCCACACCCACCAGAGTTCCCTCAGCTGCCACCCCTCCGGCAGCAGACTCAGTGCCAGCTCCGGCACCTCCNGTGGCCCCAGCGCCACCNGTCGTTCCTCCTCCCAGGCTACTNAACAGATTAAAAGNCGANGGNTTGCGGACCATCCTNGAGGAGAAGNTATTGTCCACGGANGGCGTGGNGGGCAGGTACCCCGANGTGNGGGACACCCTCCGGTNCCACGGGTTCGAGCAGTTCACCAGGCCCCGAGGCCCNTACATTCCTNCNTGGGTCCGGGAGTTCTACGCNGCNTACGGNGACTTGGTNCCNAAGGGNAAGAAGAAGGCCAGCGCGTTCAGACCGGTGGAGTCNGTCATGGTCCGAGGNAAGGAAGTNGAGTGCGACAGCGANCATATCAACGCTGTATTNGGTAGACCGCTGCACTCTGCACTTCCCTACGAGGGNTTGCCTATCGCTCAATCCCTGGATGACCTGAAGGGTTGGCTGGCTCCCCTGATTTCTGACACCACCCCGAGGTGGATCGAGGCGGGAGCNCCNATCGAGAAGAAGGACCTGAACGTNGCCGCCCGGTACTGGTTCGGNTTCATCAGCAGCACCATCATGCCGTCCCAGAACGAGTCCATCCTCCGCCATCCNAAGGCGGCCTGCCTCGGNTCTATCATGTCCAGGAGG

>rnd-1_family-327#LTR/Gypsy ( RepeatScout Family Size = 254 Final Multiple Alignment Size (possibly truncated) = 98 Localized to 2361 out of 4218 contigs )

GGATCAAGGTCTATCTGGGAAATGCTGAGAGTGTGCAAGAAGTGATTGAGGCCTACNATCTTGATGAAGTCTGAGTAATCAAGGGGCCTGCGTCGTGCCGCGACGTTAAATCAAGCGCTGCTTGGGAGGCAACCCAAGATGTACAATCTAGCCAACNATAGGNTTCTCTTTTCCATTT

>rnd-1_family-497#Unknown ( RepeatScout Family Size = 129 Final Multiple Alignment Size (possibly truncated) = 98 Localized to 2361 out of 4218 contigs )

TTTCACCAATGAGTTCCAAANGCCTTGGGTAACGTTTTCAAGTAAAGATTTTCGGATTCAAACCTCATTTTCGGAATTGGGTTTTGAGTTGATTGACCCATTTTTCAAAGTAATTGATATGGGTGTTGTTANTTTCGAAATCTTATTGTGATTACTTANTTGAATAGATTGCGTNGNTTTGGAAGTCGATCGGAAGGGGAAGACTCAAGTCTCGGAGTGATTGTTCGATTGATTTGAGGCAAGTGATCTCTTAAACCTTGTTAAGTCTAGTAGAATTCGTGTATTTCCTTNTTTANTTATGTGTTGGGAGTAATGGGAATNAGGTGATGGGTTGACTTGTTCCACTTGATTAATCTTAATGAATAAAAAAGGGTTGAAATGAAAAGGCAATGTGTTGATCGTGATGNTTGTGAATTGCCTTGTTGTGACCCTTATTGATTGATTGATGAAATGCTTGATAGCATGANTGTGGACTTGAATTGCTTGAATTGTTGTCTCTTCCNTGTGGTGTGANATTGCTTATGTGCATTGATTGTTGNACACCGTGATGAGACATGTGATGATTATGCCGACG

>rnd-1_family-490#Unknown ( RepeatScout Family Size = 131 Final Multiple Alignment Size (possibly truncated) = 98 Localized to 2361 out of 4218 contigs )

GGGNAAAAGGGCGAGCTAAGACGGGCAAGGGGCGAATTTTTAAGTCGAAACTTTANGGGTCCTTAGTTCTTTTCCNAAGTTGAGTCCCCATTATTCTATTCCTTTCCTAGCAACTATAAGACCTTTTTAGACCCGAATTCNTTCAATTAAGTCTCTCCAAATAGAATCAAAAGAAAACCCACCTCCTCTCAAATATTTCTCTCTCTAGAAATCCTCCATTGAAGAAGAAGAAGAA

>rnd-1_family-415#LTR/Gypsy ( RepeatScout Family Size = 173 Final Multiple Alignment Size (possibly truncated) = 98 Localized to 2361 out of 4218 contigs )

GAAATTTTGAGCAACGTTTGACTTGGGAAAGGATTGTAGGCTTTCTTTGATCCGATTGTGCCTTTCAAAGCCTACCGAATGACATGTTATCCATAGAACTCCCATTTCGAGCCTGAAACCTTTTTATTTGGACAACCGCATCTTAGNCATATATCCTTTTGAGTGCATAAATGCACTATCCCTTGGTCCCACCTCCTTGAGCGCACTATCAATNATCTACACAATGCCAAAGATCTAAGTTGGGTAACAAAGTGNAAAGGCCAAATATGCGAAAGTCCAAAAAAAAAATGACAAAGAAAAGCTAGAACAGAAAAATGAAAAAAAAAACCTCCAACGAAAAGGAGAGAAAATGAGAAAAAAAAGGAAAATAAATGAGGAGAAGCTTTCAAGAAAACAAAGGTCGAATGCGAAAATGAAAAGGTTGAAAGCACTGTGCAANGTTCAAGGAGGAGAAAGTCACTANATNTACCCAAATGTATCCTACCCGNCCCCGAGCCTACGTTACAAGCCGATAAAGTCCTATTTGATCTCATTTTCGAGCGTNCTTAATTAGTGGAGATGTACATAAGGGCAAGCCTATGGTTCTCGTGCATGCATGTGAATTTNCTTCTGAGTGTGAGTGTCTTTGAANAAGTCCTACGATCTANATTCATTCTTATTGTGTGTGAANTAGGATTTCGTTTGTTGTGAGGGCACTTGNTNCATGATGGATATGAATGATTTTATTCCTTAATTTANGCAAGATGAACTGATCTTGTNAATACTTAAGTATGTTTGAGATACCATTTGAAACTATAGGGATTACTCAAAGTTGACCTCAAATTTGTTTGAAAGTAGTTGAAGGNGATTCATATGCATAATACTAATTGTTGGTACCAACTAAAGTCAAGACATCGTCGANCATGAACCGATTCNTTTCTCAAAAGATGTCTTTTGTAAAAAAAA

>rnd-1_family-73#LTR/Gypsy ( RepeatScout Family Size = 881 Final Multiple Alignment Size (possibly truncated) = 98 Localized to 2361 out of 4218 contigs )

TGTAACGACCCGGAAAACTAGTAGGTGAAACTAGAGCCTAACGNGTGTGTNTTTGAGTTTGNTGTGGGGTTTGANGTTGTAAAATGNTNTCCCGAGCTTAGGTTGAGGGGTTTAGGGGTTAAACGTCAAGGTACGACTCCCCAAGGACCAACCCAAGGGTCCTTGAGGAGGACCCTAAAGCCTAACCCCAAGGCTGCCCCAAGAAACCAAAGCTGCCAAAATGTGGTGACCTACGGATGGGACCTACGCCCCGTAGGTCCATCCACGCCCCGTGGATGGGCTCCGTAGGTCAAGGCCCCAAAATGNCGAGCCGCAGACCCAAACCACGGACCNNCAGNACGGGNCGTGGGCCCACCCACGGTCCGTGGGTCGGGGGTCGTGGGTCGCGCCTGNAAATCTGCCNAGGCTCGGCCAAGTGAGGGGTAANTTGGTAAATTCCTANNTTGGTTAGTTAAGTTAGGGGACGGTTTATTTTGATTNATTANAGTTATTTAAGGNTTTTTAAANCATTAAACACCCTTTACAATTCATTATTCCCAAAACCCAAAATAGAAACCACCCTCTCTCCAAAANATTCTCTCTCTAGAAACCTCCATTGGAGAAGAAGAAGTCAAGGTCTAGGGCTCAAGANTTCANCTTTTCCACTCAATTTCGTGGAGATTCTTCAATTAAGGTATGGTAGTCTTCATCCNTGGTTAGCTTTCATCCANGGAGCCCCTTCAAAACNTGATTTCAAAGTTTCCAAATTCCCCCAAAANCTAGGGTTTCAATCTAAGTCATGGGTTCNTTTCAAAACGTTTTCAAAGGTTGATTTATGACGAATTATGATTGAATTGNTGATTTAATGATGATTTATGATGAAATTCCCCCATGAACCCATGATTTCCCCAAATTCCTAAATTG

>rnd-1_family-54#LTR/Gypsy ( RepeatScout Family Size = 1031 Final Multiple Alignment Size (possibly truncated) = 98 Localized to 2361 out of 4218 contigs )

GCTCACATGGTCTATGTCGGTTAAGGCTATGTCTCCCGAANGNTANGAATACGAACTATGATAAGAACATGGACTCTAATCATGACTTCTTAAGGGGTACTACTTAGTGTGAGTGGGGGTATGGGACTTCACTTATGCATTGCACAAGTGGACTCTAAGAGGGGTTATGGTTAGGCTCTCTTATGTNATGAAGACTTATGGATTATGTATGTTAATATGAANGCATGTTAAGGTTGACTTTACTTATGACAAGATATGAAGTATGATTATGCACGTGAATTACACTTATGACTTCTTAATGGGTTTGCTTAGTATATGTGGGGGTATGGGACTCCACGTGTACATTGCACAAGTGGGCTTGTGAAGGGGTTGTGAGGGTGGTTTNCCTATGATATGAACTAAATGGGTTATGACTAATGATGTAGTAAATGAATGAGTCCTTATATGATGTTATGAAGTATGTTGGTCTTATGTCTAATGTTAAATGAAGATGTTATGACTTATTGAATATGAATGTGCCTNGTCTATGGTGGCTTCAAAGGAGTACTTAGTGTGAGTAGTANTATGGGATGCTACTCGCACATTGCACAAGTATGACTTAGGGGTTGTCTTAGGTGATGGTCCTTATGTGATGATATGACTTATGGTGTGCTTATGNCTCTATGAATATGTGTAGTTGGGTNGATGAAGGCTGAATGGTAAGTTCACCTTTGGTGACCTTAAGGTGGTACNTTGCGCCGAGTATTCCCTAAGGGNCGCTTGAGGNANACAGTTAAGATAAAGAGGAAGGTAATGTATTGTATGCGTTGAATGTGCCTAAAATGTTATAAGTGGNTCTATGTGCTATTATGAATGGTTTCCATGTGTATGATGATGTATGTGGCTTATATTGTGCCTCTTGTACTAAATGTTCATGAAGTTTCTCTAAAAAGGCATAATGCATGGTTTCCAACTAAAATGTCCCTTTTAAAAGCATGTTTTTGCATGGTTATCATACTTAGTACATTCTTGTGCTAACCCATNCTTCTTCATCTTTTTACACCAAGTGTAGGTNNCGGCGGCTAAAGGGCCTTTCCTAGAAGTGATGAGCTTGGNTTACTATTCCCAAGTTCGGGTGTGTCCTCANAGATTCGAGGACCTTTATGTTNAAGAGTTTCCTTAAGTTCAATGTACTAGATA

>rnd-1_family-211#LTR/Gypsy ( RepeatScout Family Size = 444 Final Multiple Alignment Size (possibly truncated) = 98 Localized to 2361 out of 4218 contigs )

TTTCATATTGCCTTTACTTTTCTTGCTCAGTCGGCCTATGATGCCTACTGGGTACCTGTTGTTTTGGTACTCATGCTACGCTCTGCATCTATTTCGTGATGCAGGTCCGAGCACCAGTNACCAGCGTTGATCGAGCTTGGAGCAGACTGATCCGGAGACGAGGGTGAGCACACGGCGTTCTGGACTATTTCAGTCTCCNTCTGTATATATAGACTNGTCTTTTGCCTTTCGAGACAGTCCNGTCTCTGTNGTCCACTTTTGGGACTTGTACTCATTTTGTTAGTAGCTCTGTACTNGTGACTTCCAGGTTCTGGGAGGGATCTTTATTTGTATATATGTTTTGGTTTGCTTCCGCCTGTTTATATTGTTATTTGCCTACTCTTGTTTAGTTTCTACCCTTANACCCATTACTTGTCGTTCCGGGTTACGGGTTGGCTTACCTACTGGTGGGTTATAGTAGGTGCCATCACGACTCGAGAAATCGGGTCGTGACAAGTTGGTATCAGAGCCCCAGGTTCGTCGGTCTCACTTGTACAGAGCTAACGTCTAGTAGAGTCCTGCGGATCGGTGCGGAGACGTCCGTAACTTATCTTCGGGAGGCTACAGGATGTCTTTAGGAAAATCTCCTCTTTTGTTTCGCTTTCGTGCAGCGTGTGTCTTATTGGTTTCTGGAAATCTCACTTGTTTCACTCTTTCGCAGGATGGCTCGCACGCGAGGTGGCGCGGCCAGGGGTGGCGCACCCGGGCCTGCAGGCAGAGCCCCAGCCTGGGGTAGACGCCGACCCCGAGGTCGAGGCAGGGCCGTAGCACCAGCCCCGGATAGGGAGGAGGAGCCTCAGGCAGCATATGTACCT

>rnd-1_family-159#LTR/Gypsy ( RepeatScout Family Size = 554 Final Multiple Alignment Size (possibly truncated) = 98 Localized to 2361 out of 4218 contigs )

TTGTAACATCCGGCAATTTGAAATAACTANGAAGAGGCTTAGAATTGGAAATAGTCATTTTTGGAAAGAATTTGAAAATCTGGAAATTTGGCTAAGTATGGGAAAAAGGTGAGTTTTTGGCCAACTTCGAGCGGTCATAACTCCTAGCTCAGGATGAGTTAGGNGTAGTTCCAGTTATGGTTGCGAAGCTCGTGGAATGATCTTTCCAACGCCGCCGAGTTTGCTCGATTCCGAGTTCGTATGAGCGAGTTATGCCCTTTGGAAGTTGGGCAGTTGGCAGGGAAATCCGTCCGGAAATTTTAAGGGCATTTTGGTCTTTTCCCTAGCCANTTCTTTTGGTTATATTGTTGTGTTAGGCTGATTTTTGGATCAGTTTGTCCCATTTTAAAAGAGTGAGAGTTAGGGTTTGAGAGTGAAGAGGAGAAAGAGAAGA

>rnd-1_family-326#LTR/Gypsy ( RepeatScout Family Size = 255 Final Multiple Alignment Size (possibly truncated) = 98 Localized to 2361 out of 4218 contigs )

GCACTTCGCCAACTGGTTCGGCGAAGCGATTCTACACCCAGAACTCCCCTGCTCTTGTTTGAATACTNTCTAACTTTTTCTCGAAGTCTTTTGCAGAAATGGCTAGACCGAAAGTCGCAGGGAGAGATATGCCACCCCGACACGTAAGAGCACGAGATTTCAAAAGAGACGAAAAGANAGCAGANCCGGCCAAANAGAGAAGNGAGAGCAAGAAAGCNAGCGCCAGCAGAAGAATCCCCGTCGACCCCACTATTCCTTCGTGGANACGCGGATTCTACACGGCTATACACTCCTTTTCGGCGGCTCATGACGTGGACAGAATGGTTGCAGCTAATCTCGCTGCAGAGGCTAAGGCAAAGGNAAACAATGAGGACCAAAATGACATCCGGGNACCGNTGCCCAAGTTCAGANTGATGCACCGGGCACTGATGCCCAGACAGATGGAGCGACTGCGTAGACAGGATCCCCTCTTTACCTCCCTCTCTGTCTTTCTTTATTTTNACTTTTGGATATTTTTTATTTGCATTTGAGGACAAATGTTTTTATTTGTGGTGGGGTGAGGCCCACCT

>rnd-1_family-353#LTR/Copia ( RepeatScout Family Size = 228 Final Multiple Alignment Size (possibly truncated) = 98 Localized to 2361 out of 4218 contigs )

TTGAGAAAGTTGATTCTCAGAACAGTGAGAGCCTAGTTGATGTTGATCCAGTTCCTTTGACTATTCCACCAGATGAAAATCTTCAAAATGATGAAAATCAAGTTGATATTGAAGATGGTGATCATATTCAGAATGACCAGTATGCCGTTGATGCTCCAGTGCAAGACGATGTGGTTGGTCAGCAACCAACTATTATTGATGCTCCAGAGAGTTCTCTCAGAAGATCTACTAGAGAGAAAATACCTTCATCTCGTTATTCTCCCAATGAGTATGTACTCTTGACTGACGGGGGAGAACCAGAGAGTCTTGATGAGGCCATGGAAAGCGAAGAANANGATAAATGGNTTGATGCTATGAANGANGAGATGAAGCTCTACATGATAATCATACCTGGGANTTGGTTGATNTTACCTAAAGGTAAAAAGGCCATTGGGTGCAAATGGGTCTTCAAGACGAAACACGAAGATGGTAACTAGTTGAAAGGTACAAGGCTAGACTNGTNGCNAAGGGNTACANNCAAAAAGAAGGNATTGATTATNATGANACNTTCTCNCCGGTNGCGAAGATGTCATACATCCGTGTGGTTCTAGCCTTGGCTGCAAGTCTGGACTTGGAGNTTCATCAAATGGATGTNAAAACTGCNTTTCTNNATGGNGANCTNGATGAAGAAGTTTATATGGAGCAACCCGAGGGTTTCGNAGTCAAGGGAAAAGAGAATNGTGTGCAAACTNAAGAAGTCCTTGTATGGNCTNAAACAAGCNCCCAGGCAATGGTACATGAAGTTTGATTCTTTCATGAGTCAGCATGGNTTCAAGAAGANTTCTTAGACCATTGTGTTTATATGCAAAAATTCTCTGACGGTGACTTTATTATCGTTTTGTTNTATGTTGATGACATGCTTGTTGCTGGTNATAATACTTGCAGGATTAAAGAGTTGAAGCAAGAGTTGAACAAGTCTTTCGANATGAAAGACTTGGGA

>rnd-1_family-350#LTR/Gypsy ( RepeatScout Family Size = 232 Final Multiple Alignment Size (possibly truncated) = 98 Localized to 2361 out of 4218 contigs )

CTAGTCCAGACTCGGACGAAATCTGAGTCAGGTGAGGTCTAGGGAAATCCGGTAACGAATGGGGGATCTAATTATGAATTTGATCACATGAGTAGATAGCTAAGACGTTAAGGAACCCGTACGACTTTACGGAATCGAATTCGGGCGAGTAGAACTCCCGAAACTGATCTTGGCGTGAANGGGTAGTTTTTGAACGTCANGGGTTGAAGGTGTGTTGTGAA

>rnd-1_family-4#LTR/Gypsy ( RepeatScout Family Size = 1685 Final Multiple Alignment Size (possibly truncated) = 98 Localized to 2361 out of 4218 contigs )

TAAGCCAGGCCAAGGGTTCGCTTGGGGCCAGCAATGGTTCTCGAGTGCCGGTCNCGCCCAGGGTGTAGGCTCGGGGCGTGACAAACTTGGTATCAGAGCACAGAGTTCAAGAGTCCTAGGGAGTCTATGAAGCCGTGTCTGTAGAGTCCTAGTTATCGGTGTGAAGCGCGCCACATCTATAATTAGGAGGCTGCAACATTTAGGAANTATCTCACTTCTTTCATACTCATTTCGTGCGNTAGAGTTTATCTCTATAAAAGTTTCTTTCTAATTCGTGCTTGCGCGTGTCTTTCAGATCATGCCTCCACGAAGAGCTGTCAGAGGTCGTCCTGCTAGGAGGAATGTTGACCCAGGAGCAAGGGGTACCCAATGCACCNGAAGTGCAACCCCAAGGAGAGGTCACCAATGCTGAGTTCCGNGANGCTATCCGGATGTTGAGTCAAGTTGTGACCAACCAGGTTGGGCAACAGAGAGGGGNTCGACAGGAAGTGGCTGATACTTCGAGGATCCGTGAGTTCTTGAGGATGAATCCTCCAAGCTTCACTGGTTCAAGCGCCACTGAGGATCCGGAGAACTTTGTTGAGGAGCTGCAGAAGGTNTTTGAGGTTATGCACGTTGCCGATGCTGAGCGAGTGGAACTAGCTGCATACCAACTGAAGGGTGTCGCTAGGACNTGGTTCGACCAGTGGAAGAAGAGTAGAGCTGAGGGTGCACCACTTGCGAGTTGGGCTTGTTTTGAGGAGGCCTTCTTGGGGCGTTTCTTTCCCCGNGAACTGAGAGAGGCNAAGGTACGAGAGTTCCTCACNCTTAAGCAGGANTCTNTGAGTGTTCATGAGTACGGNCTGAAGTTCACCCAACTNTCCCGCTATGCTCCGGAGATGGTTGCGGACATGAGGAGCAGGATGAGTTTGTTCGTTGCTGGGTTGTCTCGTCTGTCAAGCAAGGAGGGCAAGGCAGCTATGCTGATAGGGGACATGGACATAGCAAGGCTGATGGTCCATGTGCAGCAGGTTGAGGAAGANAAGCTGAGGGATAGGGAAGAGTTTAGAAATAAGAAGGCTAAGACACAGGGAATGAGTCCGGGCAGCAGAAGAGTAATGCGAACCGGTCGTCCTTCCAACAGAAGCAAAAGGGACCTGCTCCATCATCTGCTAGTGCACCTGCACCNAGGAACAAAGGTGAGTACAATAGTCAGAATTCGCAGAACTTCAGAGCTAGACCTGCNCAGTCTCAGGGTAGTGTGGCACAAGGGGGTAGTNGGGCTCCTGCATGTGCTAAGTGTGGTAGGAACCACCCAGGTACGTGTCGTGATGGCTCCACTGGTTGTTTCAAGTGTGGNCAGGANGGTCACTTCATGAAAGAGTGCCCTAAGAACAGGCAAGGTAGTGGNAATNGGGGCAATAGAGCCCAATCTTCNTCAGTTGCTCCACCAGACAGAGCTGCACCTAGAGGAGCTACTTCNGGTACNGGCGGAGGAGCAAACCGCCTNTATGCTATCACTAGTCGCCAAGAGCAAGAGAATTCNCCAGATGTTGTCACTGGTATGATCAAAGTCTTTACTTTTGATGTTTATGCTTTGCTAGATCCAGGAGCGAGTTTATCTTTTGTGACTCCTTATGTTGCGATGAATTTTGATGTTCTTCCTGAGNAACTTCGTGAGCCCTTCAGTGTTTCTACACCTGTTGGTGAGTCTATTCTAGCNGAGAGAGTCTATCGTGATTGTNCCATTTCCGTCAATCACAAGAGCACCATGGCTGATTTAGTTGAGTTAGACATGGTAGATTTTGATGTCATTCTAGGTATGGACTGGCTTCATGCCTGTTATGCCTCAGTTGATTGTAGAACTCGAGTTGTCAAGTTCCAGTTTCCTAATGAGCCAGTCATAGAGTGGAGGAGTAGTTCAGCAGTGCCTAAGGGTCGTTTCATTTCGTACCTTAAGGCGAGAAAGTTAGTTTCCAAGGGGTGTGTCTATCACTTAGTCCGAGTTAATGACTCTAGTGTTGAGATACCTCCTATTCAGTCAGTTCCAGTAGTAAGAGAGTTTCCAGAAGTCTTTCCAGATGATCTTCCCGGAGTCCCTCCTGAGAGAGAAATAGACTTCGGTATAGATATTCTTCCNGATACTCGTCCTATCTCTATTCCGCCATATAGAATGGCACCAGCAGAGTTGAAAGAGTTNAAAGAGCAGTTGAAAGATCTCCTNGATAAGGGTTTTATTCGACCAAGTGTCTCACCTTGGGGCGCTCCGGTCTTATTTGTGAGGAAGAAAGATGGTTCCCTTAGGATGTGTATAGATTACCGTCAGTTGAACAAGGTTACCATCAAGAATAAGTATCCTCTTCCGAGAATTGATGATCTTTTCGATCAACTTCAGGGTGCCACTTGTTTCTCTAAGATAGACCTCAGATCNGGCTACCATCAGTTGAGAGTAAGGGAATGTGATATTCCAAAGACAGCNTTCAGGACCCGTTATGGTCATTATGAGTTTCTGGTCATGTCGTTCGGTTTGACCAATGCGCCTGCAGCGTTCATGGACCTTATGAATAGAGTATTCAAGCCTTATTTAGATATGTTTGTTATCGTNTTCATTGATGACATACTAATCTATTCGAGGAATGAGGAAGATCATGCTAGTCATCTCAGAATAGTTCTCCAAACTCTNAAGGATAGAGAGTTGTATGCCAAGTTCTCTAAGTGTGAGTTTTGGCTTGAGTCTGTGGCATTCTTAGGCCACATTGTTTCCGGTGATGGAATTAGAGTTGATACTCAGAAAATNGAGGCAGTGCAGAATTGGCCTAGACCCACATCTCCAACTGATATTAGGAGTTTCTTGGGNTTGGCTGGCTATTATAGAAGGTTCGTAGAGGGGTTCTCATCTATTTCGTCCCCTTTGACCAAGTTGACTCAGAAAACAGTGAAGTTTCAATGGTCTGAAGCTTGTGAGAAAAGCTTTCAGGAATTGAAAAAGAGGTTGACTACTGCCCCAGTNTTGACCTTACCAGAAGGTACGCAAGGTTTTGTNGTGTATTGTGATGCGTCTAGAGTTGGTTTGGGTTGTGTGTTAATGCAGAATGGCAAGGTTATAGCTTATGCCTCCAGACAGTTGAAAGTTCACGAGAAGAATTACCCAACCCATGACCTAGAGTTGGCTGCCGTAGTATTCGCTTTGAAGATATGGCGTCATTATCTNTATGGTGTTCATGTNGATGTGTTCACCGATCACAAGAGTCTTCAGTATGTGTTCAGTCAGAAAGAGCTTAATCTCAGACAAAGGAGGTGGTTAGAATTACTCAAGGATTATGACATGAGTATTCTTTATCACCCAGGTAAGGCTAATGTTGTTGCTGATGCCTTGAGCAGGTTGTCTATGGGTAGTACCGCCCATGTTGAGGAAGANAAGAAAGAGTTAGCGAAAGATGTGCATAGACTTGCACGNTTGGGAGTCCGACTAATGGATTCCACAGAAGGAGGAGTAGTGGTGATGAATGGGGCTGAATCATCATTAGTGTCAGAAGTGAAAGAGAAGCAAGACCAAGATCCTATTTTGCTTGAATTGAAGGCAAATGTTCATAAGCAAAAAGTAATGGCTTTTGAACAAGGGGGAGATGGCGTATTGAGGTATCAAGGTAGATTGTGTGTACCAAGGGTGGATGAACTCCAAGAGAGGATCATGGAGGAAGCTCATAGCTCCAGATATTCCATCCATCCGGGTTCCACAAAGATGTATCGCGACTTGAGAGAAGTNTATTGGTGGAGTAGTATGAAGAAGGGCATTGCAGAGTTCGTTGCTAAGTGCCCGAATTGCCAACAAGTTAAAGTAGAGCACCAAAGGCCCGGTGGTATGGCTCAGAATATAGAACTTCCGGAATGGAAGTGGGAGATGATCAATATGGATTTCATCACAGGTTTGCCGAGGTCTCGCAGGCAGCATGATTCTATTTGGGTGATTGTA

>rnd-1_family-251#LTR/Gypsy ( RepeatScout Family Size = 359 Final Multiple Alignment Size (possibly truncated) = 98 Localized to 2361 out of 4218 contigs )

TTTTGGGGTTTTNNAACTTTGCTCTTGAAATTGACAAATTGAAGTTGAATTCGAAGTGGGTTTGCTTGTAATTGCTTAATTTCATGTTACCCATGGCTGCTTTCATGGAAGGTATAATTTCTTTACTTTTTGTGATGTGTGGCTAAAACCCCAATTCTNGGGGTGCGATTATGGGGTTGGGTGTTGATTTAATGTAGTGGGTATTGTGTTNTGCTCTGTTTTATTGTTGTTCATNCATGGATTTGGGCTAATTAGCATGGGGTTAATTTATGGGTTAAGGTTGCAAACTTAATTCCTACTTTCGATCTCCGATTGATGCT

>rnd-1_family-12#LTR/Gypsy ( RepeatScout Family Size = 1491 Final Multiple Alignment Size (possibly truncated) = 98 Localized to 2361 out of 4218 contigs )

ATGTTGATGATTGAAAGTGGGTTTTGAACCTCGAAAGGTGAATTATGGAATTATGCATGTTCTTATGAAATCTATGCAAATGTTTTAAGGATGCTTTGAGAGTAAAGTATCAATGATGATGTTGTTGTTGTGTTGTTGAAAGGATTTTCTCATAAACACATGAAAGCATGATTGTGAAAGGTTTTCTCACATAGTAAGGATTCTAAGGTTGAAAGGTCTTCTCACCTAATTGAATCAAAGATAAGGAGCTATCCTAATGAAACCTAGCTTGGATTGGTTATTGAATGATACCTTTCCATGGATGACATATGACTTAGCTTGGATTGGCAAGATGACGTGCCCTTCCATGGATAATAAGAACAAGTAAGAAATGACTATTCCGTGGGATTTATGCTTAGCACCGAGAGGATATTGAGATGGAAGCTCTCCCGTTAGTAGAGGCCGGGTTTCTAGTAGCAATCTCCTTATCCCNTAACTATGTGCCCCCATAGGATGATTGTCTTAGATAGACATTAGCTAGTGGATCCACTTNAGCTAGAAGTTCATGGTCCTTACCTTGGCAAGTAGGACANCCCTTTTCGGTGTGGGGTAGACACCGGATTCCATGTTATAGCTCACATGGTCTCTATGTCGGTTAAGGNTAAACTTCCCACAATAATGAACTAAGGTTACTTCAAGAAGTATCTCACATGTGTTTNAAGATGATGTTAGCTTGCATTGACCATGTNTTATGACTTATGTTTTCTAACCTTCTTATCTTATGNTTTAGCTTGGTCATTGCATGTCATGTAAAGTCCTTTTTAGCATGATTTCATAACTTTTATGCATTGCTATCATACTTGGTACATTCCNTGTACTAACGCATACTCTTGCCTACATTGTTTCACNAATGTAGGGTCCGACGCTCCTANTTCNCATNCTCGTGGCTAGTGATCTCNTNTNGAAGNTTGAAGATTGGTGAGTCCTCATGTTCCGAGGACCGAGACCTTTTATTGCTTTTTTGTTTTTATTTTCAGTTTTGTACGTGATGTATGGGTTACGTCCCGATCACTCTTGATGTANTAGATGGCTTTGAGACTATGTTAGACTTCCGCTTTTTTTTGTCAAACTCTTTTATGATGAAACTGTCTTTTAAATCTCTTNATTTCTATTATCTTGTATGATGTATGCTAAGTGGCTTGTGTAGGGCCTCTCGGGGTCCTATACGCCATGTTACGCCTAGGGTGTACTTTGGGTCGTGACAA

>rnd-1_family-324#Unknown ( RepeatScout Family Size = 257 Final Multiple Alignment Size (possibly truncated) = 98 Localized to 2361 out of 4218 contigs )

GGCTTTGATCATCAAGGTATGTGGGAATTCACCAATGGATTCCNTTCATCCATTGGGTCCCAAAGATTCTCAATTCTCCAATTCAATTTCACCCAATTTGATTAGGGTTTCATTCGATTCTTCATGGGTTCTATTTATTNTGATTCAATTGTTTGATTTCGATCTTATTATGCATGATTTGATTCAATTACATGNTTTTCAATTGATTTCATATGAACCCATGCATTATNCATGATTTTGACTATGAATT

>rnd-1_family-291#LTR/Gypsy ( RepeatScout Family Size = 299 Final Multiple Alignment Size (possibly truncated) = 98 Localized to 2361 out of 4218 contigs )

TTGTATTTTTATATACATCCAGAGTTTATATTGTATCTTTGCATACATACAGAGTTATTATTGTATTTTTAAATACACAGAGTTGNTATCCATGTTTTAAACAGCTTTTCTTTATATTGCACTTGTTTTTAACTGCTTTATATTGAAATGAGTTCAGTTGAGTTGAGTTGAGTT

>rnd-1_family-102#LTR/Gypsy ( RepeatScout Family Size = 728 Final Multiple Alignment Size (possibly truncated) = 98 Localized to 2361 out of 4218 contigs )

ATACTTGATAGATTGGAAACGTTCGGAGGCATTGAGGAAAGGAAAAGCATTGGAGAAGTAGCTTGCTTGACTTCGGTTCTTCGGTGGAGGTAGGTTATGGTTTATTCTATGTGATAGATAGACTCTTAATAGCGATTGATATTCATTGAGTGATATTGTGAANTTCTCTATGTACTTGGTTGTTGTGGTTTGGATAATTCGTGTGTTGTTTGTGATTGGTCTGAAATCCCGAGACCGCGAAATTCATAATCTTGAACCTTCTCTATCGAAGTGATGCCTTGAATAAAGAAGGCTTGATGAAATATTGTTAATGAAGTGAAAAGTGGTGATAATAAATGATAAATTAACGATATTATTGGATCGGGTGTCACGTTCCGACACGATATTATTGGATCGGGTGTCACGTTCCGACACGATATTATTGGATCGGGTGTCACGTTCCGACACGATATTATTGGATCGGGTGTCACGTTCCGACACGGTAACATTGGAT

>rnd-1_family-165#LTR/Gypsy ( RepeatScout Family Size = 537 Final Multiple Alignment Size (possibly truncated) = 98 Localized to 2361 out of 4218 contigs )

AAATAGTTAAAATGATGTTTCTAAACCTAAACTAATGCAATTGACTCGGTTTTAAAGAGTTTGGAAGTCTAAACGTCAAGGGACGACCAAGACGTTCGGAAACTAGTCTTTTACGTGTTGGTGTGTTCTAGTATGTTGTTCATGTTTTTGTGTGTCGTNTGGAGTCTAAAATCAGTGGAGGTGACCCTAGTGTCTTAATAAATGTTTTTAGGGTCAAAACGTTCGGATACGACTCCCCGGGGCCACCCTAAGGGTCCCCGAGGAGGACCCCAACCTTGGCCAAGCAAGCTGCCAAGGCAGCTTGCAAGTTGCGCTTGGAGGGAGCGGNTCCGCGTCGCGGACTNGCTCCACCAAGGAGCAAAAAGTTTGGTTCGCGNCGCGGNCGCGTCGCGGACTCTACTGTCTCGAAATTTAATTCCAAANATCTTGNGGGAACACCTCCGCGTCGCGGACTTGTTCCCCGACGAAATCTGCGGAAATTCAAAACTCGAGTTTGACTTGTTTAAAGAGTCCAACTAAGTGAGGGGTANTTNGGGTACTTTGGGGGACGATTATATAATGGTTTTTAAGTCAATTTACCTCACTTTTATCGCTCAAACGCAAAACCCCAAAGATCAAAACCTAAAGTTCCTCCTTCTCTCTACTCTCTCTCTCTTCTCAAGAACTCCATTGAAGAACATTGGAGCTTCAAGAAGAAGACCGACTCTCCAAGGTTTCAACTCGAATTTTCGTGGTTAATTCGTCAATAAGGTATGGTTGCTTATCACTCTTGGGATTCCTTTCACCCAAGAGGCCCCNTCAAGATGAATTTCAAAATCTCCAATTTCTAGGTTTCAATCCAAAAACGTGGGTCTTCTTTCTAAAGTGAAATTGATGTTATAAACTGACTTTGATTGATGTTATATGAACAATTATGGATGAATTTATGTTGTATTGGTGGTTTCTTGAAGAATTCCCCATGAGACCCATGTTTCTCCTTCTTTTCCTAATTCTAACCCTAGTTTGTGTTGGATATTGATTGAAGGCTATGAATTGAATGTGATGTTATGTAATTCATGTTTGTATGATGATTCTACCCTAGGTTATGTATAATTTCTATGAAATTAGGGTTGAGTCTTTGAATGAAGCCTTGAAGGCTATGAAGGGAATATGTCAATTGCTTATATTGATGTTGATTATGTTATTACTTCATGAATTACCTTGTATTATGTATTGGTTATGAATTGTTTGGTAATTGAAGTATGGATGTCCCACGAAGGGCAAGAAGGTATGAAGGTTGGTTCTTCTTTGAACTCAAGTATGAAAGGTGAATTACTTAGATTGTAATGATGCTATACCTAATGTGTTGTTGTGGTGTTGATGACCTAGTTTCCTCATCCTTAACCCTCTACACTTGAATTAGCTATGAAGTATGTATGTATGTTATGGTATGATTGCTATATGATTGAAAGGTAGTTCTCATGATTATAGTGTAATGTAAAGTGAAAGGTTTACTCACTTGCTAAGTGTTTCTAAAGTGAAAGGATTGTTACTCACTTATGAACACATATGAGCTATTATGGTAAGATGTCTACATAAGAGTCTAGTAAAGGCTAATGTGAACTTATGTGATGACTAAAGATGATTACAAAAGGGAATTAGATGCTTAGCACCGAAAGGGCATGTAAATGAGATGGGGGTCTCACGTTTAGTAAGTCC

>rnd-1_family-182#Unknown ( RepeatScout Family Size = 495 Final Multiple Alignment Size (possibly truncated) = 98 Localized to 2361 out of 4218 contigs )

AGTTAAGAAGTAAGTCNAAGCAAGTCTTTAAAGCTTTTCAAGAGTCTTTATTGAACGTTTTAACTTCGTTTTAAGGCTCAAGTTTCAAGTCAAGTAAAGAGTATAGAGTTTCAAGTTAAGTAAAGAGTCTCAAGTTTCAAGTTAAGTAAAGAGTATAGAGTTGAGTTCATTTCTCAAAAGTTATTAGGGAACTAAGTATTCCCAAAGAAGTTTATAAATGTTTTCACATTT

>rnd-1_family-38#Unknown ( RepeatScout Family Size = 1167 Final Multiple Alignment Size (possibly truncated) = 98 Localized to 2361 out of 4218 contigs )

CATTTCAAGGGTAGCTTGAGTTAGGTTAGTCATGGTAGCTTGGGATTGAGGACTCATACTCTCCTAAGGGTAGCTTAGGATTGAGAATCATACTCTCCTAAGGATAGCTTGCATTTGCATCGTAGTATGCTTGTAAGGGTAGCATGACTTAGTTGGTCTAGGACTATTTCATGGTAGCTAGGATGGGACACTTGTACCCTTCCTAGAATAGCTTGGACTTGCATGNTAGTGTGTCTTCCANGGTAGTTAGCTTAGCTTAACCTAGTTATGTTAGTAAGATGATAGCATGGTCTAGCCTAGTTAGGGNGTTATTCCTTGGTAGCCTTGAATTGAGAAATAGAGTTCATTCATGGGTTGTGACTTGCCTAGTACTAAATAGAGAAGTACCTTATAGTGAATAAAGGCAAATAGAAGAGGGACTAGTCCCCAACTAGNGAACATNAGTATGATGGCCTAAAGGAGTACTTAGTATGGGTAGTAGTATGGGATGCTATTCATGCATTGCACAAGTATGACTTAAGGTTACTTATGAAGTGTTCTCTTATATGATGTATGCTTAGCTTGGATTGTTGCTATAGTTGCCTTCCNNGATATGATTGACTAAAGGTGANAGTTCCCTTGTCTATGNGAAGTAAGCTAATAATGAGACCAAAGGAGGGTGAATATGACTANGATGACTTCAAGGTTATGCTTAGTGTGAAGGGTATTATGGGATGCCTTTCATGCATTGCACAAGTGTGCCTTGAGGTTACTTAGGAGTATGGT

>rnd-1_family-319#Unknown ( RepeatScout Family Size = 260 Final Multiple Alignment Size (possibly truncated) = 98 Localized to 2361 out of 4218 contigs )

TTTTTTTTTTTTGGTTTCCCACCCGGTGTCCGGAGCCCGCATTGGAGCTCCGACTAAATTCGGATCGCGCACTGCAGGGCCCATTCGGGGGTGGCGCTCCCAACAAGATTTTCTCCATACCCAGGGCTCGAACCCGAGACCTCTGGTTAAGGGTGAAGCAGTCCCACCACTGCACCACAACCCATGTTG

>rnd-1_family-481#Unknown ( RepeatScout Family Size = 135 Final Multiple Alignment Size (possibly truncated) = 98 Localized to 2361 out of 4218 contigs )

GGCGAAATGGTCTGGTGGACCCTTGTACTTGTATGNGTTTGTATTNTGAACCCTTCTACTTACTCATTTGTCAACTGAACCCTTAAACTCATTAAAACGCAATATTTTAAACCCCTTTTT

>rnd-1_family-303#LTR/Gypsy ( RepeatScout Family Size = 274 Final Multiple Alignment Size (possibly truncated) = 98 Localized to 2361 out of 4218 contigs )

AGCACTACTGAGGGTGCTACGATNGTTGATGCGGGCGCTACTGAGGGCGNCCCNANTGTTGNTCCAGCGGGCTCCGGGAAACCGGACCCACCCGCTTGTTGATGGTTCNTCGGCGCTATGCGCCACAGGTTTGCTTCACCCAATCCATTATCTTTTACTGTTTTTGTGTGCATTGGGGACAATCGCACCTATTTTTGTTGGGGGTGGGGTAAATGGATTGTGAGTGATAGGGTGAAGTCTGAGTAACCCAACTCATAATCCTCTCTTGGGGTTTTCTTGCCTGTGTTCTTTTCCCCCAAGAGACTGTTTAATTTCTGTTGAACCGGCATGTGTTAGGATCGTATGTGTAGAAGCATGAAAAAGAATGAAGCATGATGGCTTATGAAAAATGATACCCGTCCAAATTTTGTGATGTGTGCTAGAATTTGTAGGCTANGATGAGTTGAATGTGTTGGCTCTGAGTATGACATGATAATGAACCGACTTGACGGCATGACTTAAGCTAAAACTTGAACTGGGTAATCTGATAATCTGATAATGAAACTATGTGCCAAGAGTGTGTGAGAATAGTTGANTGTTCAAACAGTTTTTGTGCNAATCTAGAACTTGCCCGGTTAGTCCTGCTAAGACAATCTAGTCTAGAAGTTAGGAAGTGATCATAGGCCCTTGTTCTGATAAGTCCACAAATCGCCTAAAAGAAGGATCCAACCAAATGAAATAAATGCTCCCTTTGATCCAAATGCTTTGAGCCTAAAATTAGACCATTTCTTTCNAAACCCCTAACTCACCTTCCCATAATCTACTATGTTGGCCCCGGTCCCTCCTTGGACATGTGCACCTCGACTTAGGCCAAAAGCCTAAGTTGAGGGTGGCTAATGTAAAAAGTNACCTCGATCTTGGCCCTGGTCCGACATCGGGTATTGTGCACCTCAACTAATGGAAAATCCATAAGTTGAGGGTGGCTATGAAAGAGGAAACCGAAAGAAAAATGGGTATGAAAAGAGTTTNGTGTTGAAAAAGAGAGGAAAGGATGTGACTTGTTGAAAGCTAAAGAAAAGAATAAAATGCAAGAAAAAGAAGAAA

>rnd-1_family-362#LTR/Gypsy ( RepeatScout Family Size = 225 Final Multiple Alignment Size (possibly truncated) = 98 Localized to 2361 out of 4218 contigs )

TAGGTGATGGTTGTGATTCTATGATTGTGTGATGTATGTTTGTTCTTGCTTCATTATGATACGTGTATGTATGCGAATGTTGCATTATTGATCACACTTGTTGTAAATGAGATAGATGAATGATGAATGCCATGAATCATGACTTGATTGTATGATCNTGAGAATATACTTGTGATTGTGATTGTGG

>rnd-1_family-33#LTR/Gypsy ( RepeatScout Family Size = 1193 Final Multiple Alignment Size (possibly truncated) = 98 Localized to 2361 out of 4218 contigs )

TGATATACCGTGAGTTTACGGTATTTTTAATACGTTTCACTTACGAGTTGTGTGTGTCTAGGGCCTTTTTGTATTGGTTTTTATGTGTTTTTATCATGTTTTGCAGGAAAGACGTTCAGGCGCGGAAGTAAAGAGACAGCTGAAAGAAGTGCAGAAAAGGGCATCTACGGAGGTGATCTACGGACCGTAGGTCCATTGACGCTCCGTAGATGATGGCCGTAGATCGGCAGGCAAGGCATGGAAGGCAAATCAGGGAAATCTTGACCAAGTGTGGAACCACGGTGGCCATCGACGGNCCGTAGATCGATCTACGGACCGTACTGCTGATCCGTAGANTGATACNGNGAAGGGGTTCCAGTACCTGATTTTTGAAGATTCTAAGTGTGGAGCTACGGAAGGGGATCGACGGACCGTAGATCGATCTACGGTCCGTACTGCTGGTCCGTCGTTTGCTTCAGAGAAGCTGATTTTTGGAAGCTGAAATATTTTCTAAGTCCTGANCGACGGAAGGGACCTACGGACCGTAGGTCGATCGACGGTCCGTAGGTCAGTCTCGTGGATCGAAGACGCGTCCTGAAANATTATTTCCTTATTTCGTTTCCTTTTTATTTAGGACTTGTTTTCTATAAATAGGGCATGTAAACCTCGTTTTTGGGGGTTAGACNTTATTACTTTTATTCTAGTTTTTGACTTTGGAGATTAACTTGCAACTTTAGCAATTCTTGATTTCCTAAGTTCGTTTTGAAGATTTTGGCTTTGCAATTCAAGTTGAAATTCCGGGTTTATTATTCTCNTTACGTAAGTTCATGATTTCTTCATCTAAAANTATGAATTGTGTTCTTGCGATTATGGGTAACTAAACTCCACAACTAGGGTTGTGGGAACCATGAGCGATTAACAAAGTATGAATAGTAAATAAGCAATTCTTGAATAGTGTTTTGCATGCATTGATAATTCTTTCGTTTAGAAGTCTTTTTAACGGTGGCCAACGTTAGAACTCGCCTTGTTGCTACTTGCCGGACCAAGGAGGTAATCAACGAGAAAAGAATTATCAACATAGATTTAGTGTGATACTATCTAATAGTCTAGTGTCGATTGGTGCGAGGGTAATAACTAAGCCAAACATCGATGTGATGTCTAATATGAGGTAAAGGTAAGGGTTAGTAAATTATACACACGTAGCCGGACCAAGGTGCGGGGTGAAATTCTCTAGATGCCGGACCAAGGATTTAGAGATACCTAACTTATCACTTTGCATGTAATACACTAGGAAAGGATTGCTATTACTAGGATTACCGCGTTATGAGCTTGTGGGGAACACGTATACCCTAGTTACTTTCTCATCTTGATAACAACCAAAGTTTGAATCTTGCTTACTGATTACTTACTAANTTCTTACAATTTGTTTCACAAATCCCCCCCCCCCTTTAATTACTTGTTTTCGGAAATANTTTGACTAAACGAATATAATCGTCGGTTAAAGTTAAGTCTAAACCATATTCCTCGTGGGATCGACCCCAACCTACAAGTTGGGTTCTTTACTTGATAACGATCGCTTATGCTTCTTTAGGGAGGTGTAATTTGAGCGTATCAAATTTTGGCGCCGCTGCCGGGGAATACGGCTTTTAGATTAAGTTTAACTACATTATTGAACTTTAGTCAAGTATTTCCTAATTTTACTTTTTCTTTTGTTTTTGTCTCTCTACAGGTTCCTACTCTAGTGTATGCCAAGTACACGGAGTCGAGGCGAACCACTGACCCCCTACGATCCAGAGTTGAGCAGAACTTTGCGAAGAATGAATAATCAAGGGGTNCCAGTTAATCCNATCGGAGAGGATCTAGGTGATGGAGTTGAGTTGCAGCCTCCGAGGGTTGTTAATGNGNACGNTCAGGTTCANGGTGGNAATCTANTNGGAGATGCAGTGAGGGTGCAGGATCCGCCAGGACCGAGGCTGCGTGATAACTACAGGGTCGACTTCGATACGGTTGAATCNGANGGACCTATTGTTCTNCCTCCTCTACCTCCGGGGCATACATTTGTGGTGACCAGCAGTCTGATGCAGATGCTTACAGCGAGGGGGTTGTTTTCGGGTTGGCNTCGGAAGATCCGCATGCTCACATGGCCAAGTTGAGGAGTGTTTGCAAGAGTTGTGTGGGNCGACCGGACTTGGATATGGATGTCATNGGNTTGNGAGTNTTCCCTCTATCATTGACCGGCGATGCTGCNGTGTGGTTTNCTGAGCTTCCNTATAACTCGATCTATACATGGGACCAATTGNACGAGGTGTTCATGGCAAGGTACTTTCCGGTGTCCAAGAAGCTNAATCACAAAGATAAACTCAACAATTTCGTAGCACTACCTGGAGAGTCCGTGAGTAGTTCTTGGGACAGGTTCACTGCGTTCATAAGAAGTGTTCCGAATCACCGCATTGATGATGAGTCGCTGAAGGAGTATTTCTATNGAGGNCAGGATGACAACGGTAAAGAAGCTGTGCTTGATACTATTGCTGGAGGNTCATATGGTGAGTGCACTTTTGAGNAGATCGCTGAAAAACTGGAGAAGATCTCCCGNAACAATAAAGCATGGAGCACTAGGAAGTCGGATACTGGGAGAAGCACGTTTGCCGTTCAAGCTGCAACCAACCAATCCGCNGATGANATTCGTGAGGAGATGGCTCAGATGAGGACNGAGCTGGGGTTGGTATTGAAGCATGTGAGCGGAGGTGCAGAAAAGGTAAATGCTGTGAATTACTTGACTAGAACTCCACCACCAGTTGAGGAGTGTTACTATGAGGAGGATGCNTATNCGGTGAATGATCAGACGGGGGGTTTCCGANCCAACGCCCAAGGTTCCAACNCGGATAATTGGCGCCAAGGTCGGAACTATGGNAACTACAACCGAGAGGGTCANTATGTCCGGGATGGGAACTNCAATCGCGACAACAACTACAACCGGAACAACTATGGCAACAGAAACGATCGGGTTGGGCCTTATGTTCCNCCTCGAAATCGGGAATCTGGTACTAGGGAAGCTGGAGGTAGTATGNCGCGTATTGAGGATATGATGCAGAAGATGATGAGGAGGTTTGATGCGACTGATGAGAATGTGAAGGAGATGCGAAACGACTTGTCTGGNATTGGTCAAAAGGTTGATGCCCATGCAGTGTCGATAAAGCANCTTGAGCAGCAGATGACTCAGTTGTCCACTACAGTGAACCCACGTCAACCTGGCACNCTTCCTAGCAACACCATCCAGAATCCGAAGAATGATGGGCATTGTATGGCAGTCACTACTCGAGGGGGTAAGCAAACCATTGATCCACCTATGCCGTCTGNGGTGGAAGTTGAGGTTAGAAAAGANGATGATGNGATNGAGGTTANNGGAGAGTCCGAGAATGCGACAGAGAAGGAAGCGGAGATAACCCAAAAAGTTGTCCCCATGCCTAGACCTCCACCNCCNTTCCCACAGAGGTTAGTGAAGAAGACTGAAGAAGGNAAATACCGCAGGTTTATTACTATGTTGAAGCAACTTTCCATCAACGTTCCGTTGATAGAAGCTTTGGAGCAAATGCCTGGGTATGCGAAGTTTATGAAAGATNTGGTGACAAAGAAGAGGGCCGTGAGTTTTGAAGATGATGATAGGTTGCAACATTGTAGCGCTATTGCTACAAGGTCNCTTGTGCAGAAGAAGGAGGATCCTGGTGCTTTCACTATTCCNTGTACCATCGGGTTGTTGCACTTTGCNAAGGCGTTGTGTGATCTTGGTGCTAGCATCAACCTGATGCCATTGTCTATTTACAAGAAGTTGGGTCTAGGGGATCCAAAACCGACTGCGATGCGGTTACTCATGGCCGATAGAACCGTGAAGAGGCCCATTGGTGTGCTCCANGATGTGCTAGTGAAAGTGGAGTCGTTCATCTTTCCGGCGGATTTTGTGATNCTTGATTGTGAGGTTGATTTTGAGGTTCCCATCATCCTTGGGAGACCATTCCTTGCCACNGGGCGCGCNTTGGTCGATATGGAGAAGGGGCAGATGAAGTTCNGACTGAATAATGAAGAGGCAACTTTCAATATTTGTAGGTCCATGAAGCAGAGTGGCGAGCTCNAATCGGTATCTGCGGTAACTCACANNGTNGAGAGTGGNNCNGAAGTGCGNATTGAAGAGAGACTGGGTGTTGAGGCACTAGCAGCGGTTATGATGAATTTCGAGAGTGATGGTATTGAAGANTACGATGAGTTGGTNGCCGCACTCGATAGGTNCGAA

>rnd-1_family-372#LTR/Gypsy ( RepeatScout Family Size = 213 Final Multiple Alignment Size (possibly truncated) = 98 Localized to 2361 out of 4218 contigs )

TGTAACACCCCGGAAACTAGTAGGCTAAACTAGAGCTTGACGTGAGGGTTAGAGTCGTAAAATAACTTCCCGTACTTAGAATGAAGGGTTTAGGGTTAGAACGTCAAGGTACGACCCCCCAAGGACCAACCAAGGGTCCTTGAGGA

>rnd-1_family-424#LTR/Copia ( RepeatScout Family Size = 167 Final Multiple Alignment Size (possibly truncated) = 98 Localized to 2361 out of 4218 contigs )

CACTACAACAAAAACAACTTTTAGCGGCAATAAATATACACATTAATAAAGAGTGCTAAAGTCTTTACCGGCATTAGTTAATTGTCATTAGATCCAATGTCGCTATAGGCTTTAGGGACATTTACAAAGAGTGCTAATTGCCGCTAAAAATACATATTTAGCGGCAATTAAGCTATTGCCGTTAATTAATTGCCGCTAA

>rnd-1_family-178#LTR/Gypsy ( RepeatScout Family Size = 510 Final Multiple Alignment Size (possibly truncated) = 98 Localized to 2361 out of 4218 contigs )

TCGTCCGTGAAAGGNACTCGTCAGACTTTGAGTTTTGGGTCAACTTCAAACGATCATATCTTTCAGCACAAAATGAATTAGGTGGCCCATGACCTATCAAATTAAAGGTCTTTGAGTCCTCTTTCCAA

>rnd-1_family-453#LTR/Gypsy ( RepeatScout Family Size = 147 Final Multiple Alignment Size (possibly truncated) = 98 Localized to 2361 out of 4218 contigs )

TTCGAGCTAAAAGCGAGTTGTACTCTCTTCCCTAAGCCTTGTTTCAGCTGAGATCCTTATGAGGAAGTATGTTTAGAGCTTGGGTAGTATTTTCACCCGAAAAGGTAGCATGAGTTACGAGATTATGAGCAGTAGTAGTGAAAGNGCCCAGAGTTAGAGGAAAGTATGCAGAGGTTTAGTAATCTAAGAAAGGGAGATAGTGATTTGAGGAGCTATGTTATTATAGTCATGCACCCAGTAAGTTATTAATGAGGAGTTTTCCCTTATGGGTAGACTAAGAGTTGCTAGTCAAGATGAGATAGAGTATATATTTTTAACCAGAATAGAGTTGATGTCGATGTGCCATCGTGTTAGAGGAGACCAAATNCATGTGTTGAATAGAAGAGCAGAGTATTCATGAAGTGATAGATCTAAGCTAGGCTTATGATTTGTTTGAGAGGGTCATGATGTTCAGAAGGTTATAGAACTGATCAAGTAATGAGGTATAGTATAGTAAGCGAGCAATCATATTGATATTCGGGTTTAGTAATAAACATTAAGCTTGAATTTGAGATGAGTGTCGTGATCAAAAGAATGGCAACATGAGGGTGATGATGTTAGTCTTGAGATTTGATCTGGTAGACTTGACATAGAGTAGGTGAGGAATTGAGGTGTTAGCTGCAATAAATATGAGTGGTACAAGTGAGAGACCCTAACCTTTAGATAAGGGCAGTGAAGTGTGGCTAAGTCACAAGAGTAACAAGA

>rnd-1_family-558#LTR/Gypsy ( RepeatScout Family Size = 106 Final Multiple Alignment Size (possibly truncated) = 98 Localized to 2361 out of 4218 contigs )

TGTAACGACTCGGAAAATGATAGGTTGAACTAGAGCCTNAACGTGTGGGTTAGTGTGACCTTAGGTTGATGTTTAGGCTGTTTTAGAACTTAAATGGAGCAAGGTAGAAGCCTAGGGTGCAAGCAAGGGCCTAGGCTAGCAAGATAGAGTGAAAGAAANCTTGTTCTTTGAGGGGCCGTTTGGCGATGGGGGTGCTTGATCGCCCAAGGGGTTTGGCGAGTCGCCCNTGGGCCCTTGCGNTCGCCTAGTGGGCTGCCCCATTGGCCNAGNTGCTGGACCAAAGGGCGAGCTAGGTCGGGGCTCGCCAACCAGCTCGGCGCGTCGCCCATCGACCCCTCTAGATCGCCTAGTGGACTGCCCAATTCGTGAGCTCACTGGAAATTTNGGCGAGCCAAGGGTNCACTTGGCGAGTCGCCTAGT

>rnd-1_family-67#LTR/Gypsy ( RepeatScout Family Size = 941 Final Multiple Alignment Size (possibly truncated) = 98 Localized to 2361 out of 4218 contigs )

GAATNATCTTTCCAACGCCGCCGAGTTTGCGCGATTCCGAGTTCGTATGAGTGAGNTATGCCCNTTGGAAGTTGGGCTGTTCGAATAAGGAAAGTCCAATCCGGATTTTNGAAGGGTATTTTGGTCTTTTCCTTACCCAATTANTTTAATTCGTTTTTAGTAANTTAATTGGGGTCTAAACTGAATTAGTTCAGTTTACGCANTTGAAAATTACGCTAGGGCTTTGAGAGAAGAGAAAAGAGGAGGAAAGGAGAAGAAAAANCAAGANTTCGTCGAGTTCGTGAGGANTTGNTTGTGGATTTCGCCAAGGGTTTGATCCCTACGAGGTATGTGAGNCTCTCATAGCGTTGGGTTCGTTCACCCACGCGCCAANCATGTTTATTTCAGCGAAATTCGTCCTAGAAAGTTGAAAGTTGATGTTCTTGAATGGTGTTCTTGAATTGGCTTTCGTTCTTGAATTGGGNTGAGATTGAGGAGTTTCTTGAGGTTATTACGTCGANTTNTAGAGTTGTTTGAGTTAGATTCTTGTGTACATNTGCTGGGTATCTGAATCTAAAGAGAATTTGAGAAAAAGAACCGAGTTTAGGCGAATTGGGGCTGGAAAACGAAGAAGGAACAAGTCGGGCGAATCTGGGTACCGGGTCCGCGTCGCGGACCTGNTCCCCAGANTTGAAAATTTTCTCTTCGCGTCGCGGAGCGGTCACGGACCGTTCTGCCTCAAAAGTTATTTTCGCGACCAAATATTTAAATGCGTCTCCGCGTCGCGGACCCGCTCCCAGACAGTGATTTCTCGATCTTTTCTCATGTTTAGCTATCTAAAATCACTCCTAAACATCATGAGATCTTTCCTATCACAAATCACAATCCTTGAATCCATAATTCAATTCAAGGAAAGTTAAGAGTCAAGTCAAGAGAAGTTAAGAGTCAAAGTCAAGAG

>rnd-1_family-138#LTR/Gypsy ( RepeatScout Family Size = 615 Final Multiple Alignment Size (possibly truncated) = 98 Localized to 2361 out of 4218 contigs )

TGATGTGAGGCTAAAAATACATATTTTTAATCATTATTTCCTCACATTTATTATTTATATTTGTCCTTTTTGAGCATGAATTTTATGAATTGTGTTAAATGGTATATTTTATTTGTAGGATTAAATTGGTGGAAAAATGAAGAAGTTTGGAGCTAAAACGAATTAAAGATGGAAGGTTGAAGAAGGAAATCAAGCAGACAGCTTAATGGATTAAATTTAATAAAATAATATATTTATATATAAAAGGAAATGGCGGCTGCAAATTGTGGAATTTCAATTTTTACGCTTGCTTCTTGCTGCTGCCACGTGGCAGCAAGTGAANCGTGCAGAATTATGTTGAGGCGGCGGTATTACTGTTCACGCGGCGAAAAAGNAAAAGGAATCCAATTCCTTTTGGTTTTGGATTCCTAATTTAATTTGGACTCAATTATTTTATTTAGGGTTTCCTACATCTATAAATAGTGCATAGAAATAATTATTTGGGGGAGGCCAAGAACCAAGATACAAGACACAATATAAATTTTCTTCTAGCTTAGGAACTTTGGAAGAGCCGCCGTGGAGGCCGGAGAATTGTGGTTTATTCTTTCTTCTCCTTTATTATTTATTTGTATTCGTGATTAATAAAAGATAGTTTAGCTATTGTTCTTAACTTTTTATGATTAACGTAAGTTCATGATTTCTTCATCTAATAAATTATGAATTGTGTTCTTGCGATTATGGGTAACTAAACTCCACAACTAGGGTTGTGGGAACCATGAGCGATTAACAAAGTATGAATAGTAAATAAGCAATTCTTGAATAGTGTTGTTGCATGCATTGATAATTCTTTCGTTTAGAAGTCTTTTTAACGGTGCACAGCGTTAGAACTCGCCTTGTTGCTACTTGCCGGACCAAGGAGGTAATCAACAAGAAAAGAATTATCAACATAGATTTAGTGTGATACTATCTAATAGNCTAGTGTCGATTGGTGCGAAGTAATAACTAAGCCAAACATCGATTGTGATGTCTAATATGAGGTAAAGGTAAGGGTTAGTAAATTATACACACGTAGCCGGACCAAGGTGCGGGGTGAAATTCTCTAGATGCCGGACCAAGGATTTAGAGATACCTAACTTATCACTTTGCATGTAATACACTAGGAAAGGATTGCTATTACTAGGATTACCGCGTTATGAGCTTGTGGGGAACACGTATACCCTAGTTACTTTTACTCATCTTGATAACAACAAATACTTAGTTCGAANCTATCTTCTTATTGAATTCTTACAATTTGTTTCACAAATCCCCCCATTTTTATATAACGACTATCGAGTTAAATATTTGCTATTGACAATATTCTTCCATATTCTCTGTGGGATCGACCCCGACTCATAGTTGGGTAAATATATTGCATACGATCGTTTATATTTCTTTTCAAGAAGTATATTTGAACGTTATCAATAA

>rnd-1_family-221#LTR/Gypsy ( RepeatScout Family Size = 422 Final Multiple Alignment Size (possibly truncated) = 98 Localized to 2361 out of 4218 contigs )

GTATATTGGNCCTTACAGAATATCCAAGAGGATTGGCAATGTAGCTTATGAGTTGGAGCTACCGCAAGAGTTAGCAGCGGTTCATCCGGTATTTCACATCTCCATGTTGAAGAAGTGCATGGGCGATCCTTCACTNATCATACCAACTGAAGATATTGGTATCAAGGATAGCTTATCTTATGAGGAGATTCCCGTTCAGATTCTAGATCGCCAAGTTCGCAAGTTGAGAACNAAGGAGGTAGCATCAGTCAAAGTCCTTTGGAGGAACCAGTTTGTTGAGGAAGCTACTTGGGAAGCTGAGGAGGATATGAAGAAGAGATATCCACATCTCTTCGAATCCGGAGAAATTCCAGATCAAGGTACTAATTCTCTTCTTAGTACTCTTTAANTTATAAGTTGGCATGTTG

>rnd-1_family-140#LTR/Gypsy ( RepeatScout Family Size = 607 Final Multiple Alignment Size (possibly truncated) = 98 Localized to 2361 out of 4218 contigs )

TCCGCGTCGCGGACCNGCTCCACCAANGGCCAAAAAGTNGGTCCGCGTCGCGGACGCGCCGCGGACTCTACTGTCTCGAAATTTAATTTCCAGAAACTTGTGGGAACACCTCCGCGTCGCGGACTTGTTTCCCGACGAAATCTGCGAAAATAAAACTCAAGTTTGACTTGTTTAAAAGGTCCAACTAAGTGAGGGGTAGTTTGGGTACTTTGGGGAATTATTATATTCTAGTNNTTTTAGGTATTTTAAGGGTATTAAAACATTNAAANCNTGATTTAGANCTCATTCTTCCAAAATCAAAACCCCAGTTCTTTCTCTCTCTAAAGTTCTCTCNAGAACTCCATGGAAGACGAAGGTGGAGAGCTAGGGTTTCGATTGAGGGTTCCCTTCCGCCAAATTTCGTTGGGANTTCATCAATTAAGGTATGGTGACTCTTTATCTAGGACTCGTCTTCATCCTAGAGTCAATTTCAAAGGATTTTCAANGTTTTCAAAAAGTCACTAAAAACCTCGATTTCAATTCTATCATGGGTTCTTGCATGAAACGATTTCAAACGATNAGNTATGGATTAATTGATGTTTAATTGATGGTTTTAGGATGAAANACTCCATGAACCCNTGNCTTCTACGAAACCCTAAATTTGCTATGAAGTGGGTCTATTGATTATGAATTGAATGTTGATGGAATTATGCGTAGATTGANTTGGGTTATTGATATCTCTTGTGTTGTTTNATGTCTANTTATGATGAATTGTTGATAATGGATGAATTAAGGGTGGTAAGCCTTATGGGCGAAGTATTGGTGATTTTGGCTATGGCTTTGGATTATGGTAAGAAGGCTTGAACTCTCTTTATTGAATTGATTGTTGTTGGTAGGTTGGATCCAAAAGTGTTACATCTTATTCTTGTGTGGTATTATTGTGAATTGTAGCCGTGAAGGCATTGGTGAAGTTGTTGTATAATGTGGGAANATTGCATTGTTGTGAATTGAGGCCACGAAGGGCATTATATGAGTGTTATTACATTGTTGTATTATTATACACATATGAGGCTATATGTAAAGTCCTATATGAAGCTATATGTAAAGTTTGTGTGAAGCTATAATGTGAAGATGTTCTAATGGTATTAATGTGATATTGTATAAAGTGGTATTATGCTAATGTGAAAGCATGTTTCTCTTGAAATGGTGATATGTGGTGATGATGGACTATGATGAGTCTCTATAAGATGATGTTGTAGCTCATATGCTAGCCGCTTACCAATTGAAGGATGTGGCCCAAGTNTGGTATACTCAATGGAAGGNNAATAGGCCGGTAGAAGCGGGTCCTATAGANTGGGAGGNGTTTAAGGAAGCATTCCTTGATAGGTTCTTTCCCCGNGAGAAGAGGGAGTGCTAAGGTGGAGGAGTTTATCAACCTTAGGCAAGGTAGCATGAGTGTNNAAGAGTATTCTTTGAAATTTACCCNNTTGTCTAAGTATGCTCCATCT

>rnd-1_family-40#LTR/Gypsy ( RepeatScout Family Size = 1141 Final Multiple Alignment Size (possibly truncated) = 98 Localized to 2361 out of 4218 contigs )

TAATGATGATTCTAAGGTTGAAAGTNCATTCTCACCTAATTGAATCTATGAGCTATTTTATGAGTTGTGTTGAATGAAGGGTAAGGCATTCCTTCACGCATGTAAATNCAATTCAAGACTAAATATGTATAATTTGGGGAATAATGCTTAGCACCGAGTGGATATGGAAAATGAGATGGAGGCCCTCCCGTCAATACAATAGGCCGGGTCATCTAAATTAATCTCATGAGATGGAAGCTCCTCCGCCAATACAATAGGTCGGGTTTCTAGNAGCAATCTCCCTATCCCATAACTATGTGCCCACATAGGTCTTTAGCTAGTGGATCCACCTAAAAGCTAATACGTTTAGTTCTACCTTAGGCAAGTAGGACACCTCTTTTCGGTGTGGGGTTACACGACACCGGATTCCATGTAGCTCACATGGTCTATGTCGGTTAAGGCTATTTTCCCTATATGACAAGAANATGAATATGACATGAACTATACTTATGACTTCTTAANGAGTTTTACTTAGTATGAGTGGGGGTATGGGACTTCACTTATGCATTGCACAAGTAGACTTTGAAAGGGGTTATGGGATGGTTCTCTTATGTTATGATGANATATGAATGTATGCATGTATGTTGAATGGATTGTTGCTATGAATGACTTTCCTTATGTTCAAGTTAATGNACATGANTGTTTCCTTGTTTATGANTATTGGCTATGATGAGGTCTAAGGATGATATGTATGACTANGGTGGCCTCAAAGNGGTACTTAGTATGGTTGGTATTATGGGATGCCANCCATACATTGCACAAGTATNCCTTAGGGTTGCTNAGGTGATGGTTTTACTATG

>rnd-1_family-288#LTR/Gypsy ( RepeatScout Family Size = 301 Final Multiple Alignment Size (possibly truncated) = 98 Localized to 2361 out of 4218 contigs )

TATTTTATTACTTCGACATCAAAGGAAGAGTACATTGAAGATTATATTGCAAATATGAGACACAAGCTTTATTTGCTTTACGTCAAACCCGATGGAGTTGACGTCAAATGTTCAAGGAGAGCAATTAAGTGTCAACACGGTAAAAGACGCTATCTCTCCATTTGAATTGCATTTTTATGCTAATGAGTTTTATCTCAGTCTTTGTCGAGAGCATCCGAGAGGATGAATTCTCAAAACAAACCACAGGTGCGGACGACATCAAAAAGGATGCAGCACAACGTGGAACTTTTTCTTAGCAGCGAACTGGGACATAGTCCGAAGAGGAGTGCCAAACTCTTAGGACGCGAGCNGAGGAGCGACTTCCACCACAATCAAACCACACCCCTATCAGAAGGCATGTGGGTTTTCTTTAGGTTTATCGGTTTCAGTTTCGCATCCGGAAATTTTGGTCTCTACCGGAAGCAACTTTCCAATCCGAGTGACAATGCGCCAAGAGCTTTGGAAATTATGTCCGTGTAAGTTCTTAATTATGGGTGTGAAGCGCGCCACATTCATGGCTAAGAGGTTGCAAGCCTCTTTTTCATACTCGACTTACTTTGTCGCTCTTCCAGAACCGAAGGTTATCTTGCATAATAGATTTCCTTTTCAACCGATCGAGTCGAACTACAAGCAGCCTGATTCCCTAAGTTGAGGGATATGTAGGCGGGATCAATGTTGAAAACTCGGCTGTATTCCAACATTCGCTCTTAAATCTTATTCCCGAGCATTCCGGTCCCTTCATAATTCGATATCGGGATGGCTTTTGAATTCTTTCAAAATCGTGCGTCAAATCAAGCGTATCGAACTACAAGTGGCCTGAATTCTCATATAGCCTGAGATATGTAGGAAACCCANTTCCGGGGTTCGGCCATAATTCCTAAAGTCCGTACCAAAATCCCTTTTCCGAAAGATGAAGATGTGGTCGGTCAAAATTGGATTCGTCAATTTCATTTTCCTCGAATTTCTTTCATCAATCCAAGTCAAACGAGGGACAGTTGTTGACACCCAATTTTGGCCCTCCACGATATAAATTAATCATCGAGCTTCTTCAATTTCAAACGATTTGAAATAATTAGTTTTATAAAATAAAATTCAAAAAATAAAGTAAATAAGTTTTTAAGTAATTTTGTCACTTTTTATAATTTTTAAATAATATATATGTTTTATATAATTATGTATATAATTAGTATATTT

>rnd-1_family-27#LTR/Gypsy ( RepeatScout Family Size = 1268 Final Multiple Alignment Size (possibly truncated) = 98 Localized to 2361 out of 4218 contigs )

TTGATTATGTGCATTGATTATGCTTATGTCTTATGTTGACTAACGCTTATGTTTATGTTGATGTTTATGCNAGCTATCATACTTAGTACATTTTTGTACTAACGCATACTCTTGCCTACATTCTTATCAAATGTAGGGTCCGGCGATCTCGANTCTCATCCTCGTGGCTAGGNTCTTAGTAGAAGCAAGGNTTGAAGATTGGTGAGTCCTCATNGCATTCGAGGACGTGACCACCATTTCCTTATGTCTTTCATTTTATGTTTTGAACATGTTGTATGGGCTGCGTCCCAANTTATTCTATTGTATTAGATGGTTTGAGACAAGTGTGTTAGACTTCCGCTTCGTTTTATAAAAGACTTCGATTGTATTGAAAATGTTTTAAATTTCCGCATCGTTTCTATTATCTTATGTTATGATATGCTAAGTGGCTTGTATGAGGCCTTTCGGGGTCCTGTACGCCGTGTTACATCTAGGGGGTACCCCTGGGTCGTGACAAACTTGGTAATCAGAGCACAAGGTTTAGAATGGTTCTAGGATGTCTCATAAGCCACGTCTAGTAGAGTCTTGTTCATAAGTGTGAAGCGCGCCACATTTATGAATGAGAGGCTATNAGATGTTTAGGAAACTTCACTTCTTCNNTTACTCTAAGTCGTGCCATAGAGTTTAACTCTATAAGGTCTCTCTCCTAATCCTTACCCGATGGTCTTCAGGATATGACTACTCGAAGGGCTNATGCTAGAAGGAATGAGGGAGACAATGTGGANCAAGAAGCTCCTCCCCAAGCTCCTCAAGCTCCGATCGACCCTTTGGCCGAGNATGTGACNAATGCGGAGTTTAGGNCNGCTTTCCAAGTGTTGGCTCAAGCCGTGACGGCCCAAGCCAATAGGGAGGTTGTGGCTCCCGTGAACCCAAATGTGGGTACGGCGGCNTCAAGAGTGAGGGACTTCACNAGGATGAACCCTCCGGAGTTTCATGGCTCCAAAGTTGAGGAAGATCCTCAAGAGTTTATCGATGAGGTCTATAAGGTGCTNGCNATCATGGGAGTGACNCCGGTAGAAAAGGCGGAGTTGGCCGCTTATCAACTNAAGGGTGTTGCTCAAGTTTGGTNCAACCAATGGAAAGAGGNGAGGCCGGTAGANGCGGGTCCCGTAGANTGGGAAANGTTCAAGGNTGCTTTTCTTGATAGGTTCT

>rnd-1_family-35#LTR/Gypsy ( RepeatScout Family Size = 1185 Final Multiple Alignment Size (possibly truncated) = 98 Localized to 2361 out of 4218 contigs )

CCTTAAACTATGTGCCCCCATAGGTTGNTTAGCTAGTGGATCCACTTAAGCTAGAAGTTCATGGTCCTTACCTTAGGCAAGTAGGACAACCCTTTTCGGTGTGGGAGACACCGGGGGATCATGTTATAGCTCACATGGTCTCTATGTCGGTTAAGGCTAATTCCCACAAAAGTTAATAATGAACTAAGGTTACTTCAAGAAGTATCTCATATGTGTTCAAAGGTTTTAAAGATGATGTTAGCTTGCATTGACCATGTTTTATGACTTATGTTTTCTAACCCTCTTATATCATGTTTTAGCTTGGTCAATTGCATGTCATGAAATGAAATGTCCCTTTTAGCATGNTTTAAATGTTTTATGCATGGCTATCATACTTGGTACATTGTTTTGTACTAACGCATACTCTTGCCTACATTTTCCCCAAATGTAGGGTCCGACAGTCAGGGTTCTCAGTTTCGTGGCTAGNGGTTGACTTGCGAGATTTCCCGAGCTTTGGTGAGTCCTCATGCTTCGAGGACGGATTTTCATTGTTTCAGTTTCTTTCTTGTATTTCNCTTTTGGACATGATGTATGGGCTAGGCCCAATTTCATTCTATTGTANTAGATGGCTATGATGAGACAAGTGTTTAGACTTCCGCTTTNCTTTTATAAAAATTTTTGGACTTGAAATGTTGTTTTACTCTTATTGTTCTATTTCTTATGTTATGAANGCTAAGTGGCTTGTATGGGGTCCTTCGGGGTTCTATACGCCATGTTACATCTAGGGGGTACCCCTGGGTCGTGACA

>rnd-1_family-530#Unknown ( RepeatScout Family Size = 118 Final Multiple Alignment Size (possibly truncated) = 98 Localized to 2361 out of 4218 contigs )

AGTTGAGTATCATATTGAAGTTGAGTACCTTATTTGAGTCGAGTATCNTATCGTTGAGTTGAGTATCTTATCTCTGAGTTGAGTATCTTATCCTTGAGTACTTCTGAGTTGAGTAAGTTTGAGTAGTTTTGAGTATCCTTGAGTATTCCTTGAGTTGAGT

>rnd-1_family-331#LTR/Gypsy ( RepeatScout Family Size = 251 Final Multiple Alignment Size (possibly truncated) = 98 Localized to 2361 out of 4218 contigs )

AATAATGTACCTTGAATCGGTAGGCTTATGGCATCCCTTTCATGTGTTAAATAATGTACCTTGGGTCGGTAGGCCTATGGCACCCTTTCA

>rnd-1_family-494#LTR/Gypsy ( RepeatScout Family Size = 130 Final Multiple Alignment Size (possibly truncated) = 98 Localized to 2361 out of 4218 contigs )

CTACTTGANTCCAATTGGTATCTGGCGATNCAAATTGGTATCTNACCTCCTTCACTCTTCTGTCCGCAGATGGTTAACACTAGAGCAATAGAAGTGGNAACANCAACACCAGCAAGAGAAAATGCGTCCTTGGAAATAGGCATGTTTCCCCGATTGACTACAGGGCCTGTNATGACTGGTAACGAGCATGANATGTTGACTAAGTTCTTGAAGTTGAAGCCTCCTGTGTTTCATGGTTCTGAGAGTGAGGATGCTTATGAGTTCATCGTNGATTGCTATGAGAGGCTTCATAAGNTGGGNATTGTNCATCGGCATGGNGTTGAGTTTGTGACNTTCCAGCTTCAGGGNGAGGCTAAGCAGTGGTGGAGGGCTTATGTGGAGTGCCGACCAGTAAAGGCACTACCTATGACTT

>rnd-1_family-393#LTR/Gypsy ( RepeatScout Family Size = 189 Final Multiple Alignment Size (possibly truncated) = 98 Localized to 2361 out of 4218 contigs )

AAAAAGTTACCTCGATCTTGGCCCTGGTCCGACATCGGACATCGTGCACCTCAACTCATGCAAAATNCGTAAGTTGAGGGTGGCTATGAAAAAGGAAACCGAAAGAAAA

>rnd-1_family-358#LTR/Gypsy ( RepeatScout Family Size = 226 Final Multiple Alignment Size (possibly truncated) = 98 Localized to 2361 out of 4218 contigs )

AAGAGTCCGAGAATGCGACAGAGAAAGAAGCGGAGATATCTCAAAAAGTCGTCCCCATACCTAGACCTCCACCACCTTTTCCNCAAAGGTTNGNGAAGAAGANTGAAGATGGNAAGTATCGCAGGTTTATTACTATGTTGAAGCAGCTTTCNATCAACGTTCCNTTGGTGGAAGCNTTGGAGCAAATGCCTGGGTATGCNAAGTTCATGAAAGANTTGGTNACAAAGAAGAGGGCTGTGAGTTTTGAAGACGATGATAGATTGCANCATTGTAGCGCTATTGCTACAAGGTCNCTTGTGCAGAAGAAGGAAGATCCCGGNGCNTTCACNATTCCATGCACCATCGGGNTGCTNCANTTTGCNAAGGCNTTGTGTGATTTGGGNGCNAGCATCAACCTGATGCCATTGTCNATTTACAAGAAGTTGGGTTTAGGGGANCCAAAGCCNACNACGATGCGGCTNCTNATGGCCGATCGANCCGTGAAGAGGCCCGTTGGCGTACTCCATGACGTNCTNGTGAAAGTGGAGTCGTTCATCTTTCCGGCGGATTTTGTGATTCTTGATTGCGAGGTTGATTTTGAGGTTCCCATCATCCTNGGGAGACCATTCCTTGCCACCGGGCGNGCGTTGGTCGATGTGGAGAGNGGGNAGNTGAAGTTCCGANTGAACGATGAAGAGGTAACTTTCAATATTTGTAGGTCCATGAAGCAGAGTGGTGAGCTCCAAGCGGTATCTGCTATAACTTACGGAGTNGAGAGTGGGNCAGAAGTGCGAATTGAAGAGCGGCTGGGTGTTGAGGCACTAGCAGCGGTTATGATGAATTTCGATGGTGATGGTATTGAAGANTACGATGAGTTGGTAGCCGCACTCGATAGGTGCGAATNTCGTTCCAAACCAAAGAAGTTGGANNTNGACNTGAAGAATCGCGAGTCCCCACCCGCAAAACCATCTATTGAGGAGCCACCGAAACTGGAGCTNAAGGCTCTACCACCNCATCTGAGGTATGTATTCTTGGGNGNAGATAACACTTTGCCGGTNATCATTGCGGCNGATTTGAATGCGAGGCAAGTAGAGTGCTTGGTGACTGTGTTGAAGAGGTTCAAGCGAGCTATTGGGTGGACTATCGCNGACATTATTGGGATTCCTCCCGGCATTTGTNCTCACAAAATCCAACTCATGCCGGATCGCAAGCCNAGTATCGAGCACCAAAGGAGATTAAATCCGCCTATGCAAGAGGTGGTNAAAAAGGAGATCATCAAGTGGTTGGATGCGGGAGTNATCTACCCCATTGCGGATAGTAGNTGGGT

>rnd-1_family-505#LTR/Gypsy ( RepeatScout Family Size = 127 Final Multiple Alignment Size (possibly truncated) = 98 Localized to 2361 out of 4218 contigs )

TTTTTTTAATATTTATCCCTTTGTANTGAACTACGTCCGACCTGAATTCTCAAGAATGAGATACGTAGGCGGCCTATGTCGGCCTCGGTCACCCCTTTATCCCTTTTTAATATTTTT

>rnd-1_family-568#LTR/Gypsy ( RepeatScout Family Size = 103 Final Multiple Alignment Size (possibly truncated) = 98 Localized to 2361 out of 4218 contigs )

CTTGGCTCGCCAAGGCCACTCGGCGACTCGCCGAGTGGNCCTTCGGCTCGCCTAAAGTTCCAGTGAGCTCACGAATCGGGCAGTCCACTNGGCGATCGGAGGGGNCATTCGGCGACGCGCCGAGCGGGTCGGCGAGCCNCGACCTAGCTCGCCCTTTGGTCCAGCANCTAGGCCAATGGG

>rnd-1_family-502#LTR/Gypsy ( RepeatScout Family Size = 128 Final Multiple Alignment Size (possibly truncated) = 98 Localized to 2361 out of 4218 contigs )

TTTCGACAATTCGAGAATCAACACAAGCCGAATCTGGAAGAAACCGAGACTGTGAATCTNGGAGATGAGGAATGCGTCAAAGAAGTNAGAATCAGCGTCCATCTGACTGAAGCTCAAAGAAGAGACCTGGTTCGTTTGCTTAGGGAATACATCGACGTATTTGCCTGGTCTTACGAAGACATGCCAGGGTTGAGCACGAATATGGTGTCCCACAAGTTGCCGATCGATCCAGATTTCAGTCCAGTAAAGCAAAAGACTCGGAAGTTCAAGCCNGAGTTGAGTTTGAAGATCAAAGAGGAGATTACCAAGCAGATCGAGTCCCAAGTAGTGGAAGTGACGCAATATCCGACTTGGTTAGCNAATATTGTTCCGGTTGCCAAGAAAGACGGAAAGATCAGAATTTGTGTTGACTATAGAGATCTCAACAAAGCTAGCCCNAAAGATGATTTTCCNCTNCCAAACATTCACATNCTCATCGATAACTGTGCTAAGCATGAGATGCAGTCATTTGTGGATTGTTATGCGGGTTATCACCAGATCCTGATGGACGAAGAAGATGCAGAAAAGACNGCTTTCATCACGCCTTGGGGTGTNTATCGCTACCGAGTGATGCCNTTTGGTCTCAAGAATGCCGGTGCTACTTACATGAGGGCTATGACGACCATNTTTCACGATATGATTCACAAGGAGATCGAAGTGTACGTGGATGACGTCGTNATCAAGTCCCGCGAGAGTTCGGATCACTTGACTCACTTGAGGAAATTCTTTGATCGCTTGCGNAGATACAATCTGAAGTTGAATCCNGCNAAGTGTGCTTTCGGAGTGCCAGCTGGCAAGTTGTTGGGATTTATNGTCAGCAGGAGGGGCATTGAGCTTGACCCTTCCAAGATCAAGGCGATCCAAGAGTTACCTCCTCCGAAGACCAAGAAAGAGGTGATGAGTTTCTTGGGAAGGTTGAACTACATCAGCCGGTTCATAGCTCAATCCACCGTGGTGTGTGAGCCCATCTTTAAGTTATTGAAAAAAGATGCCTCGACCAAGTGGACCGGAGAATGTCAAANTGCTTTTGACGCCATCAAGAACTATTTGTCCAATCCGCCGGTGTTGGTCCCTCCGCGAGAAGGGAGTCCGTTGTTGCTATATTTGTCNGTCTCGGATAACGCATTNGGATGCGTGCTTGGTCAACATGATGAGACTGGGAAGAAGGAGCGAGCCATTTACTACTTGAGCAAGAAGTTCACTCCATACGAGGCNCGTTACACTCTNTTGGAGAGAACATGTTGCGCTTTGACTTGGATCGCTCAAAAGTTGAGGCATTATTTGTCTTCNTACACCACGTACCTTATTTCCAGGATGGACCCGTTGAAGTATATTTTCCAGAAAGCAATGCCGACCGGGAAGTTGGCNAAATGGCAAATGCTTTTGAGCGAGTTTGACATTGTGTACGTGACTCAGAAGGCGATAAAAGCACAAGCCTTGGCTGATCATCTCGCGGAGAATCCGGTTGACGAAGAGTACGAACCGCTCAAGACTTATTTTCCCGATGAAGAAGTNNCGTTTGTGGGTGAAGATATTTCTGAAGCGTACCCTGGTTGGAGAGTATTCTTTGATGGAGCGGCGAATCACCAAGGGAAAGGTATCGGAGCGGTCTTAGTGTCGGAATCCGGTCAGCACTATCCTATGGCGGCTAAACTCCGATTTNATTGCACGAACAACATGGCNGAGTACGAAGCTTGCATCCTCGGTCTGAAGATGGCCATCGACATGAACGTTCACGAGCTNCTGGTTATTGGAGATTCAGACTTGCTGATTCATCAAGTTCAAGGAGAATGGGCCGCGAAGAACCCAAAGATCACGCCGTACGTGCAGTATATACAGAAGTTGTGCAAGAGATTTCGCAAGATTGAGTTCAGGCACGCTCCCAGGACGCAAAATGAGTTGGCCGATGCTCTTGCCACCATCGCCTCAATGATTAAACATCCAGATACAAGTTATATCGATCCNNTGGATATAGAGGTAAAAGAGCAGCATGTCCATTGTTCGCATGTTGAAGCGGAACCGGATGGTTTGCCTTGGTATTTTGACATCAAGAAGTATTTAGAGNNCGAGAACTTATCCTGAGAATGCAACGTTCAACCAGAAGAAGTCGATACGCCGTATGGCCNTCAATTTCTTCGCGAGTGGGGAAATCCTTTATAGGAGGACTCCAGATTTAGGTCTTCTCAGATGCGTCGACGCTANTGAAGCTGCGAAGCTTCTGGAANAAATACATGCCGGAGTNTGTGGTACTCACATGAATGGGCTCACTTTGGCGAGGAAGATCCTCCGAGCCGGNTATTTTTGGATGACTATGGAGCGNGATTGCTGCAAGTTTGTGCAGAAGTGTCATAAATGTCAAGTGCATGGNGATTTGATTCGGGTNCCGCCTCACGAACTCAATGCTATGAGTTCACCTTGGCCGTTTGTNGCTTGGGGCATGGATGTCATTGGNCCGATAGAGCCAGCCGCCTCTAACGGACACNGATTCATTTTGGTTGCCATCGACTACTTCACCAAGTGGGTGGAAGCAGCTTCGTATAAGTCGGTGACCAAGAAGGTNGTNGCNGATTTTGTTCGCAACAATTTGATATGTCGGTTTGGAGTNCCAGAATCCATCATTACNGATAATGGAGCGAATCTCAACAGTCACTTGATGAGAGAAATNTGTGAACGATTCAAGATTACTCACCGAAATTCNACCGCTTATCGNCCNCAAATGAATGGAGCTGTNGAAGCCGCCAACAAGAACATCAAGAAGATTTTGAGGAAAATGGTCGACAATCGCAGAGGTTGGCACGAGANGCTGCCATATGCTTTGTTGGGTTACCGNACGACCGTCAGAACGTCAACTGGAGCAACTCCNTATTTGCTGGTNTATGGAACAGAAGCAGTNATACCTGCCGAAGTTGAAATACCTTCNTTGAGAATCATCCAAGAAGCTGAATTGAGCGATGCTGAATGGGTTCG

>rnd-1_family-320#LTR/Gypsy ( RepeatScout Family Size = 260 Final Multiple Alignment Size (possibly truncated) = 98 Localized to 2361 out of 4218 contigs )

GAGCCTTNCGTGAGAGTTTTGGAGTGTGGTGTGAGGGTTAAAATCGTAAAATGGCTTCCCGTACTTAGAATGAGGTGTTTAGGGGTTAAACGTCAAGGTACGACTCCCCAAGGACCACCCAAGGGGTCCTTGAGGAGGACCCCAAAACTAGCCCCAAAAGCTGTCAA

>rnd-1_family-491#LINE/L1 ( RepeatScout Family Size = 131 Final Multiple Alignment Size (possibly truncated) = 98 Localized to 2361 out of 4218 contigs )

AAGTGAAATGTTTCACTTGGCTGGTGGCNAGAAGAGCNTGCCTGACNCANGAAGTNCTGCAGAAGAAAGGAAGCAACTAGTCTCTAGGTGCTTTTTNTGCGACGAGACGGNGGAAACAAACAACCATCTNTTCCTNCACTGCAAGNTNACTGCNCAACTNTGGNANNTGTTCCTCAGCATCACAGGNATNAGCTGGACNATGCCAGAACATACNGCGGATCTNCTGAGNTGCTGGATCAGAAGAGGGGGNAGCAAGAGTCAGAAGAAGTGGTGGAGGATNATCCCANCNTGCATNTGGTGGACAGTNTGGAAGGAGAGGAACGGNAGATGTTTTGAAGACAGATCCAATTCCATNCAGAAAGTNAAATGGAATTGTNTNGTATCTTTACTTTTTTGGTGTAAACAANNNTGTATAGAGGATGTAGATCAGNTTGTAGATTTNNTAGGNNCTTTGTAATT

>rnd-1_family-172#LTR/Gypsy ( RepeatScout Family Size = 520 Final Multiple Alignment Size (possibly truncated) = 98 Localized to 2361 out of 4218 contigs )

GAGTTGACATCATGTTTTTAAACAGCTTTTCTTTATATTGCACTTGTTTTAAACTGCTTTATATTGAAATGAGTTCAGTTAAGTATTCATGAGTTGAGTAGAGCCAAGGTAAGTTTTCAGTTTCTTTCAGATTCCCTTTCAAGCCTATGTTGTGTTTAGCATTCCAACTCGCATACTCGTACATTCAATGTACTGATGCCAGTTGGCCTGCATCNTNTTATGATGCAGACGCAGGTAACCAGGATCGGCATCCGGCGCATCGTTGATCCAGT

>rnd-1_family-249#LTR/Gypsy ( RepeatScout Family Size = 361 Final Multiple Alignment Size (possibly truncated) = 98 Localized to 2361 out of 4218 contigs )

TTTGGTTTAAGTGTGAAGGGAAGTTGAAGTTCTAGAGGAATTCAAGTTATAAGGAGAAGTTGCAGAAATTGAGGTAAGGTGACTGCTCCATTTTCTTCCTCCTTATGTATGAGTTTGAGTGAGGAATTATGGGTGTGCTGTCATAAAGAACTCAAGGGGGTGGTGAGTTCAATAATTGGTTGTATGATAAAGAATATTTGAGGCTGTTTTATGTGTATTGTTGTTGGTGCAGCTTGGGTAATCTTGGAAAAGGATATATAATGTATGTATTGGGCAGAGAAGGAGGGTGTGGTGATACACATTATTTTAAGGGCTGTATGGGCTCATTATCCGTCAAAGTTAGCTACTGTTTTACGAGGCTCACGGTGTGATAGCTGCTAATNTTAGTTTATCATGACTTATATTGTAGATTAAAACCTAAAAGGGGAGGCTGAATCGTGATAGTTGTATTGGTCAGAGGATAAGGTATGTAAGGCTATTCGATTCAATATTCTTCGGCATGAAATCTGATACTTGCGATTAATATCAATAAGTGAATTTCCTAAGTTCTATTCCTAGTAAAGGAAACATAGTGGCAGCGTACGATCTCCAAATAGCTAACAATATTTCCCCCTCTCCTTAGTGTATAGAATTACTTCATTATATGTTCACTACTCTATACTTGTGTAATTATCCTCCTATGTGCTGGTATAGAAATGGTATTTGCAAAAGCAAGTTTGGTGAATCCTCCTCTTAGTCAGTTGAAGTCAATTGTGTCATTTAAGCTCACAAGTAATGCATTGATGTTACATAGCTCCTTATGTCATTGTTACTGCCTTGAAGTATTATTTTCATGTCTTCTACTACTGGTCCGTAGTTCCGAATATAAAAAATGATTATGACCACCAAAACAACAATCTCAAAGATTAAAGAATCTAAGTGAAGCGATCTGTATAATTAAGGGTGGCAGCTCAGGGGCGAAAGCCTAGCATGGGCCGATCCCAATTGGTATAGAAGGGTGGCAGCTCAGGGGCGAAAGCCTAGCATGGGCCGATCCCAATTTGTGTAATTATGAGGACCGCATCCCAGGGGTTAAAATGCCTAGCATGGGTCATCCTCTTCTACCACTGATCAGCTGGTCATTCGTATCACATGCCTTATAAAGATCATAACGCACAGAAAAGTAAAGAAAAGAATGTAACATACACGATCCCACAAGNGTNAACAAAGGTGGTCTCTAATCCTNATCTATGCACATATATTGATACTTGGTTTCACTCGAGCTCTCATTTTACATATGGTTGTATTATGCCTTACATATTCAGTACATTCTTTCGTACTGACGTCCCTCGCGGGGGACGCTGCATTTCATGCTGCAGGCACANGTACTCCAGCTAGTAGACCTCCCCAATAGAAGCACACGACACTCAGCGGCTGTTGGTGAGCTCCAGGTTGATTCGGGGCTTTGCCGAGTCTTTGGTAGATTCATTTTGGTA

>rnd-1_family-117#LTR/Gypsy ( RepeatScout Family Size = 667 Final Multiple Alignment Size (possibly truncated) = 98 Localized to 2361 out of 4218 contigs )

GAACTCTGAGTATTGAGACAAGTTTTTCCTTAGCTTTGAAGAATTGAAGNTTTCCACTTTTGAGAAATTGGAAGTGGGTCTTTGAGATTCTTCAANTTGGACCTTTGTAAAGACGAAATCAATCTTACCCAGTTGATGGAAACNCAATTTGGTAACGATTTTTATCTTTTTATGATGTCTAGCTAAAACCCCAATTCTTGGGGTGTGATTATGTGATTATGGGCTGATTTAGCTTATGGGTATTGCTAATTGNTAGTTTAAATGCTGTTTAGAAGTGATTTCAATCAGTAATTGTGGTTTAATTTAAAGGGTTGTAGTTGCAAATGCAGTTCTACCTTCGTGTTTTTGGCTTGCTCGAGAGAGAGGTTTTAAAACCAAGATTACTGATTGATGGTCTGTGGGTATTGGGTTGTCATGGGTTCAGCTCGAGAGAGTGAATCCTAAACCCTTTTCCACACATTCAGCTCGAGAGAGTGAATGGACTAAGGCGTAGGTTGTTCTTAATTTGCATGCTTGTTGGTGTTCGAGAGAAACCGACTTGATTCGGGGTAAATTGTTCGAGAGAAAGTTTACCCCCNCTANAGTCTAGCTTACTCACTAATTTACAGCTATTTACTANCAGTTGCAATTACCCGTTTGTCTACNTTAGCCTATAATCGAATCACATCCCAAGAACCCGTCTCATTATT

>rnd-1_family-52#LTR/Gypsy ( RepeatScout Family Size = 1063 Final Multiple Alignment Size (possibly truncated) = 98 Localized to 2361 out of 4218 contigs )

TGTAACACCCCAGAAATTTTTTGAGCTAAGACTCGAACCGTTCTTCGTAGTGAGTGAGATTTTACCGAGGAATTTAAAATTTCTTAAGTGTTAAGGTCACTAGATGTAGCACCTTGAGTTCCAAGAAGAACTAAAGAGAGTTCATTCAAGTCATTCCTAAGTTCTTCTTTAAGTTTTGGGTCAACTTCAAACGACCATAACTCTTAGTACANGATGAGTTAGGTGGNCCACAAGATATCAAATGAAAGGTCTTCGAGTCNTCTTTCCAACGCCACCGAGTTTGCTAAATTTCGAGCTCTGAGTAAAAAGTTATGACTGATTTACTAACGACGCGCAAACCTGGCAGCAGCTCCGCGATGGCTCCGCGTTACGGAGCCAATACGGACCTCACTGCCTGGTGAAAAATCATTCCGACTCCCAGCTCGTTTTATTTATTTCTTTAAGGGTATTTTAGTCCTAAAAACCCTTAGGAGGTCATCTAAATTNCCCTAAACGTGTAGAAAATCAGTTTTCNTAAATCAAACCCTCTCATTCACTCCAAAAATTCTACGCTCAAGAAATTCAAGAAACGCTAAGGCTAGGGTTCATCTTCCAAGATTTTCCTTCAAGTTCTTCAAGAAGATGGTTCTTTCAGGTATGTGAGCTTTCATAGCGTTGGGTTCGTTCACCCACGCGCCAATCATGTTNATTTCAGCGTTAAATTCGTTCTAGAAAGTTGAAAGTTTGATGTTCTTGANTTGTGTTCTTGAAATTGGCTTTCGTTCTTGAGTTNGGATGAGATTGAGGAGTTCTTGAGGNNATCGTGTCGATTTTAGAGTTGTTTTGAGTTAGATTCTTGTGTACATGTGCTGGGTATCTGAATCTAAAGAGAAATTGAGAAAAAGAATCGAATTTAGGCGAATTGGGGTTAGAAAACGAAGAAGGAAAAAGTCGGGCGAATCTGGCACCAGGTCCGCGTCGCGGACCTGCTTCCCCTTT

>rnd-1_family-444#DNA/hAT-Tip100 ( RepeatScout Family Size = 156 Final Multiple Alignment Size (possibly truncated) = 98 Localized to 2361 out of 4218 contigs )

ACGTGTATTATGCCATGTAGGACGCGCGTGTCTATTTGTTCAATTTTATACAAGTTNAAGTGTCTACTTGTGCACNCTCAAAGTTGGAGGNCATAGTTGTNANTTGAAGCCAAGTTAAGGGTCACGTTTATGTATTATGCC

>rnd-1_family-535#Unknown ( RepeatScout Family Size = 117 Final Multiple Alignment Size (possibly truncated) = 98 Localized to 2361 out of 4218 contigs )

CAGAGGCGTATCCAGGAGGGGTCACCGGGTTCACGTGAACCCATGCTCCCCTCCCGAGATCATATATAGTAGTGTTATATTTTTNAAAAATATTTAAATATAGATGTGTGAACCCACGCTCGAAGTATCATATAATGTAATACGATGATGATTGGGTGCACCTCTCTAAGTGAGGTTAGAAATTTGAATCTTATATCTATCTTGTTTTATTTTTCTTTCTAAAATTTTGTAANTGGTTTGGATAGATAAAAATTAATTTTGGACCTATAATTTTAAAATTTTAACGGTTTTCAGTAATAAGAACCTAAATGTCAAACCGATCAAATTTATATCTTAGATCCACCTTTTCGTAATAGTGCACCCATCATCTCGAAATCCTGGATACGCCTCTGC

>rnd-1_family-81#Unknown ( RepeatScout Family Size = 837 Final Multiple Alignment Size (possibly truncated) = 98 Localized to 2361 out of 4218 contigs )

TGTTTCTCNAAGATNGACCTCAGATCGGGCTATCATCAGTTGAGAGTNAGGGANNGTGATATTCCGAAGACGGCNTTCAGAACCCGGTATGGTCATTATGAGTTTCTGGTCATGTCNTTTGGNTTGACNAATGCNCCNGCGGCGTTCATGGACCTNATGAATAGGGTGTTCAAGCCNTATCTAGATATGTTCGTTATCGTNTTCATTGATGACATTCTGATCTATTCGAGGAATGAGGAAGATCATGCGAGTCATCTGAGAGTNGTTCTGCAAACTCTCAAAGATCGNNAGTTGTATGCNAAGTTCNCTAAGTGTGAGTTTTGGCTNAAGTCCGTGGCNTTCCTNGGCCATATTGTGTCCGGCGAGGGNATTAGGGTTGATNCTCAGAAGATNGAGGCGGTGAAGAATTGGCCTAGACCCACGTCTCCGACNGATATTAGGAGTTTCTTGGGTTTGGCCGGTTATTATAGAAGGTTCGTNGAGGGGTTCTCNTCCATTNCNTCNCCNTTGACNAAGTTGACTCAGAAGACGGCNAAGTTTCAGTGGTCCGANGCTTGTGAGAAAAGCTTTCAGGAATTGAAAAATAGGTTGACTACCGCCCCGGTNTTGACCTTACCGGAGGGTACGGANGGCTTTGTNGTGTATTGTGATGCNTCTAGAGTTGGNTTGGGTTGTGTGCTNATGCAGNATGGTAAGGTTATAGCTTATGCCTCNAGACAGCTNAAGGTNCATGAGAAGAATTATCCGACCCATGATCTNGAGTTGGCNGCGGTNGTGTTTGCTTTGAAGATNTGGNGNCACTATTTGTATGGTGTTCATGTNGATGTGTTCACCGATCACAAGAGCCTTCAATATGTGTTCANCCAGAAAGAGCTGAATCTCNGACAAAGGAGGTGGCTAGAGTTACTCAAGGATTATGACATGAGTATTCTTTACCACCCNGGTAAGGCTAATGTNGTNGCTGATGCCCTNAGCAGGTTGTCTATGGGTAGTGTNGCCCATGTTGAGGAAGANAAGAAAGAGTTAGCGAAAGATGTGCATAGACTNGCNCGCTTGGGAGTCCGACTNATGGATTCCACAGAAGGNGGNGTAGTGGTNACGAATGGGGCNGAATCATCNTTAGTGGCGGAAGTGAAAGAGAAGCAAGACCGNGATCCTATTTTGCTTGAATTGAAGGCAAATGTTCATAAGCAAAAAGTAATGGCTTTCGANCAAGGGGGAGATGGTGTGCTTAGGTATCAAGGTAGATTGTGTGTNCCGAANGTGGATGAACTCCGAGAGAGGATCNTGGAGGAAGCTCATAGCTCCAGATATTCTATTCATCCGGGNTCCACNAAGATGTACCGTGATTTGAGAGAAGTCTATTGGTGGAATGGCATGAAGAAGGACATTGCGGAGTTTGTGGCTAAGTGTCCGAATTGCCAACAAGTNAAGGTAGAGCACCAAAGGCCCGGNGGTNTGGCTCAGGATATAGAACTTCCNACNTGGAAGTGGGAGATGATCAATATGGACTTCATCGCGGGTTTGCCTCGNNCTCGCAGGCAGCATGATTCTATTTGGGTGATTGTCGATAGAATGACNAAATCAGCCCACTTTTTGCCGGTNAAGACTACCTATTCGGCGGAAGATTATGCCAAGTTGTACATTCAAGAGATAGTNAGGCTNCATGGGGTTCCNNTNTCTATTATTTCGGATAGAGGTGCNCAATTTACCGCACANTTTTGGAAGTCNTTCCAGAAAGGTCTGGGTACNAAGGTNAACCTNAGTACNGCCTTTCATCCTCAGACGGATGGNCAAGCGGAGCGCACCATTCAGACCTTAGAGGATATGTTGAGGGCTTGTGTGATCGATTTCAAGGGTAGTTGGGATGATCACCTACCTCTNATTGAGTTCGCCTACAACAATAGTTACCACTCTAGCATCCAGATGGCTCCTTATGAGGCTCTNTATGGGAGAAGATGTAGATCTCCNATTGGNTGGTTTGAAGTNGGTGAAGCNGNNTTGATAGGACCAGATTTAGTTCATCAAGCTATGGAGAAAGTNAAACTNATTCGAGAGAGGTTGAAAACGGCNCAGAGTCGNCAGAAGTCCTACGCCGATGTTAGGAGAAGGGANTTAGAGTTCGAAGTNGATGATTGGGTNTACTTGAAAGTNTCACCCATGAAGGGTGTNATGAGATTTGGTAAGAAGGGGAAGCTTAGTCCCCGGTATGTNGGNCCTTACAGAATATCCGAGAGGATTGGNAANGTNGCTTATGAGTTAGAGTTGCCANAAGAGTTAGCAGCGGTTCATCCGGTNTTTCACGTCTCNATGTTGAAGAAGTGCGTGGGCGATCCNTCATCNATCGTNCCNACTGAAGATGTTGGTATCGAGGATAGCTTNTCTTATGAGGAGGTTCCGGTTGAGATTCTAGATCGNCAAGTTCGNAAGTTGAGAAACAAAGAAGTAGCTTCNGTNAAAGTNTTNTGGAGGAATCAGTTCGTTGAGGGAGCTACTTGGGAAGCCGAGGAGGACATGAAGAAGAGATATCCNCATCTCTT

>rnd-1_family-281#DNA/TcMar-Stowaway ( RepeatScout Family Size = 317 Final Multiple Alignment Size (possibly truncated) = 98 Localized to 2361 out of 4218 contigs )

TTTTGAATCTTGTGGTCCTAAATTAAAGNTATGTCGAATGTACCAAAATGTCCTTTAATCTTGTGGTCTTAAACATGTCACGTGGAAAGTTGAAATTAAAGNGTTGCCAAAAAAGGAAA

>rnd-1_family-237#Unknown ( RepeatScout Family Size = 394 Final Multiple Alignment Size (possibly truncated) = 98 Localized to 2361 out of 4218 contigs )

TAAGGTATGGTGATCCTTCATCTAGGGTTAGCTTTCACCCTAGAGTCAATTTCAAAGAAGTTTCAAAGTTTTCAAGTTCACGAAAAACCCTAGGTTTTACTCAATCTCATGGGTTCTTNCATCAAACGNTTTCAAATGATAAATTATGATTGTATTGATGTTTAATTGATGATTTANGATGAAAATACTCTATGAACCCATGATTTCTCTCAATTCCTAAATTGTGCCTTATGAAGTGGGTTTGTTGATTATGAATGNATGTTGATGGAATTATGTCTAGATTGATTTGGGTTATTGAATTGTATCATTATCTATGCCTAATTATGGTGAATTGTTGGTGNGTTGANGATTTGTGATTATGGCCTTGAAAGGGCAAGTTATGACCTAATTNCATGTTGAAGTAGAATTGTGCTTGANCTGTTGATCCACTTGAAAGGTGGAGGTGTTTACTTACTATGTATATTGTGATCATGGCCTTGAAGGCATAGTGTGATGAATTGAAGTATTATCATGATGTTGTA

>rnd-1_family-547#DNA/hAT-Ac ( RepeatScout Family Size = 111 Final Multiple Alignment Size (possibly truncated) = 98 Localized to 2361 out of 4218 contigs )

AATTACTTAGATACACTCTAGTTTGCAATATTACGAATCTTACCAGATTTTGGTGCGTCCAGATACATGTATCT

>rnd-1_family-184#LTR/Gypsy ( RepeatScout Family Size = 494 Final Multiple Alignment Size (possibly truncated) = 98 Localized to 2361 out of 4218 contigs )

TTAAGTCTTCCGCTGAGTAAGTAAGCCAGGCCAAGGGTTCGCTTGGGGCCAGCAATGGTTCTCGAGTGCCGGTCCCGCCCAGGGTGTAGGCTCGGGGCGTGACA

>rnd-1_family-351#LINE/RTE-BovB ( RepeatScout Family Size = 232 Final Multiple Alignment Size (possibly truncated) = 98 Localized to 2361 out of 4218 contigs )

AAAGGGTTCAGGTTGAGCAGGACCAAAACGGAATATTTGGAGTGCAAGTTCAGTGACGCGACGCATGAGGCGGACGTGGAAGTGAGGCTTGACGCACAGGTCATCCCCAAGAGAGAAAGTTTCAAGTATCTTGGGTCTATAATCCAAGGAAATGGGGAGATCGACGATGATGTCACACATCGTATTGGNGCGGCGTGGATGAAATGGAGGCTCGCATCCGGAGTCTTGTGTGATAAGAAGGTGCCACCAAGACTTAAAGGTAAGTTCTACAGAGTGGTGGTTAGACCGACTTTGTTGTATGGGGCGGAGTGTTGGCCAGTCAAGAACTCNCACGTTCAGAAGATGAAAGTTGCGGAAATGAGGATGTTGAGATGGATGTGTGGGCATACTAGGAGAGATAAGATTAGGAATGAGGATATCCGGGACAAGGTGGGAGTGGCCTCCGTGGTGGACAAGATGAGGGAAGCGAGACTGAGATGGTTCGGACATGTGAAGAGGAGATGCGCGGACGCCCCAGTGAGGAGGTGCGAGAGGTTGGCTATAGTGGGTNCGAGGAGAGGTAGAGGTAGGCCGAAGAAGTATTGGGGAGAGGTGATTAGACAGGACATG

>rnd-1_family-145#LTR/Gypsy ( RepeatScout Family Size = 596 Final Multiple Alignment Size (possibly truncated) = 98 Localized to 2361 out of 4218 contigs )

ATGATGTATGCTTAACTTGGATTGTTGCTATAAGTTGCCTTCCTTGATATGATTGACTAAAGGTGAATGTTCCCTTGTCTATGAGAANTAAGCTAAGGATGAGATCAAAGGATGGTAAGTATGAATGATGGCCTAAAGTGATGCTTAGCGTGGGTGGGATTATGGGATCTTATCCATGCATTGCACAAGTATGNCTTGAGGTTACTTATGAAGTGTTTCTCTTATGNTATGATGANCTAAGTATTGACTATGCNTATGACTATGATGTTATCTCTTATGCTTACTAGTTATGATTATTGTCTNCTATGTTATAGCCTATGTCTTATTATGATGTCTCTTATGCTTATGATTATGTGTCTCTTAT

>rnd-1_family-179#LTR/Gypsy ( RepeatScout Family Size = 505 Final Multiple Alignment Size (possibly truncated) = 98 Localized to 2361 out of 4218 contigs )

TGGTGAAAGGCCTATACTCACCTAACCTACCTTATGAATGCTTGAAAGTATGAAGTGATAGAATCACTTGAACATGGATTGATTGTCAAGGACATCCTTCCATGAGACATGAGTGTTAAGCAAGGCTTAACAAGTAAATCTTGGGGTGTATGCTTGGCCCCAAGTGGATATTGACAATGAGATGGAGTGTCACACAAGATAGGGTCCGGGCTCCAAGTAGAAAGGAGAACCACACCTAGTTGATGTGAGGACTATCCAAGTTAAAGGGGGAACTCTTACGTAGTAGAGTGCGGGTTTTCCCAAAGTTAGGTCTCATGAGATGGAAGCCTTCACATAGTTGAGTTAAGGTTTCTAGTAGCAATCTCCGTATCCAATGAACTAAGACGTGCTTAAAGAAGTATCTCNTAGTGTTCAAAAGCATAAATGAACTAAGACATGCTTAANGAAGTACCTCATAGTGTTCAAGAGTATAAATGGACTTAGACATGCTTAAGGAAGTATCTCATAGGGTCCATGTTAATAATGAACTTAGACGTGCTNAAAGAAGTATCTCATAGGGTTCAACCTAATAATGAACTAAGATGTGCTTAAGGNAGTACCTCATAG

>rnd-1_family-347#DNA/CMC-EnSpm ( RepeatScout Family Size = 235 Final Multiple Alignment Size (possibly truncated) = 98 Localized to 2361 out of 4218 contigs )

GGGGAGGGAGGAGTGTAGAGAGTAGACGACTAACCTTTTTATAATTTTAAATTTACTATGGAATTCAAAATCAAGTCAATCATATGAGCTCATATATTAAGTAATTTCAAATTATTTTAACGGAGAAGGGCCTAAAATGCCCTTNAACTATTNGAATTGGTACAAAATTACCCTCCATCCACCTAATGAGTATGATCCGTTAGANATAAGGGTATTTTTGAGCCAAAAGGTTGACGTCAAGGGTATTTTNGGGCCGAAAGGTGGATGGAGGGTANTTTTGTACCANTTCGAATAGTTNAAGGGCATTTTAGGCCCTTTTCC

>rnd-1_family-408#Unknown ( RepeatScout Family Size = 179 Final Multiple Alignment Size (possibly truncated) = 98 Localized to 2361 out of 4218 contigs )

AAAGGTATGTTAAGGCNAAGCTACGTCCCTCATTTTAGCACGACTCCTGGGAAATTCCTAAANCGCCGAATGTGATATTTTGCTATTCTCTTGCTAGAAAGACCTTCCGTGAAAGGCATTGGGATCGCAGCTGAATTATTTTCGTGATTCCGTTATTTTGATATTCCACTCGTTCGGNTAAATAGGGTATTCGACGTCTNCACGTCATTTTGTCGGTTCCCTTGACTATANGTCCGCGCGTACGAATTCTTATTCTTCCTTGGGTGNNGTGACTTAAAATGTCACGCGAGACCCTTACTACTTGCGACCAGCCCTCTTTCCCCGCTAAGGTTNTTATTNAAAAAAATCTTTAAAGTAAACGTCGATCTCCAAAACTCTCAAATATAANTCGNATGTTTAAACTACTAGAGCACTTGGTTTTTTGAAATACTTCTTTTACAAATTACCNAGGAACCANGTATAGGAACTAAGTGAGTCACCTTAAGCTCGTTATGAACATCCTCAAGGTTAGGTTACCTTTCTAAAATCCGAGTTAAGTTTTCAAGCTTATTTGGAAGAACATATNAGGTTCCACGAGATANTTACATGTTATACGAAAGTGATTACGGGGTTATGAGAACAAGAGTACNAACGGGCCAAGATTGGCAACATGACNACGGTAACACATANGCGTTACGAATCCATGACATGACATGCTATGCATTCTACTCCCGAGCTATAATCGGGAGGTATGGGGCCTATTGCCTATATTTATACGTATGAGACACAGATGGCCACAAGTTGGCGAATATGATGCCGGTTCGCTACCGGAAGGCACCACCATTGCTAGCATTTGGTTGGGTATGTCATGCATTTGCATCTATATATATATATATATGTATTCACGACATACGATTATACGAGCATACCATGCATATTTAANTNGTAGGAGGCTAGATGTCAGCCATGGAGACTATTTGAGTTTCAGTTTCAGGTGGTATCTCTATTACCCTTTTACAGTAACTATTTATGATTCTACTTTTCGCCTTACATACTCAGTACATTTTTATTCGTACTGACGTCCCGTTGCCTGGGGACGCTGCATTTTTTTCGTGCGTGCAGGTACAGGCGGGGACTCTAGTNGACCCCCCCGCTAGGATTCAGGTTCANCTGTGAGTTGGTNAGCTCCATTTCNTCCTGGAGCTNCCAGAGTCGGATGTTTACTTTTGTTGTACAGTTTGGGTATAGCCGGGGCCTTGTCCCGACAGTGCTTATGTCACTCNTAGAGGCTATTGCGGACACAGTATTCTGGGTTTTCTTTCTGTATGTTTCAGTCTCCAGCCANATAGGCTTGGCATGTATATATATGTGTGTGTGTAAGTAGGTATGTCTTCGGTTGTGGCCTCGTCGGCTAGNTAGTACATCTTTATTTTTTTTCGGCTTGTCTACCATCGTTTGTATGGTTTATTCTCATTCAGGTCATGACCTCTGGGTCCAGGCTTCACTTTTTAGAGGTTGTGCTGCCCGATCTTTTTGCCTCACAAGTTTGGTTTAGCTAGTATGTTTCAGTGGGTAACTCGATGTATTAGGGTATCGAGTGCCAGTTACGCCCCCTATTTCGGGGCGTGACA

>rnd-1_family-322#DNA/TcMar-Stowaway ( RepeatScout Family Size = 259 Final Multiple Alignment Size (possibly truncated) = 98 Localized to 2361 out of 4218 contigs )

TACTCCCTCCGTCCACTTTTAATTGTCATGGTTTCCTTTTTTAGAGTCAAACTATAAGAACTTTGACTAACATTTTACGATGTATTTTTTCATCATATTGATATGCAAAAAATTGCAATTTATAGTACTTTTCGTATAGTTTTTGAATATCTAAATTTTTTGTTTAAAATATCGAATTAATGTAATCTAATTTAACTTTGAAAATTAGTCAAATTGACTTTCGAAAAGCGCAACATGACAANTAAAAGTGGACGGAGGGAGTA

>rnd-1_family-522#Unknown ( RepeatScout Family Size = 121 Final Multiple Alignment Size (possibly truncated) = 98 Localized to 2361 out of 4218 contigs )

AAATAGCAGAGATGTAAAATGACCCCCGCTGTGCAAAAATCGAACCGACCGATAAATCGAACCGAAAAAAGTGTTATTGGGTTAACGGGTTTTTAATGGTTTTATAAAAAAAATTATCGGGTTATCGGTTCGGTNTTGGTTTTTAATATTGGGTTATTGGGTAAACCGATAACCCATTAAGACGGTATAGTAATTTACTACTTTACCCNTACATAAATATTAATATCAATACCTTACTAGTTACTGCCTTTGCCCTTTAGCCTTCAATTCACATTACTGTCCTAGTTCTTTTATTTGTGTTGCATTATTGTNTTGTCGCGTCAAATTCGTTGGAGAAGTCATATATTTATTTTATAAGCATTTTCTTATTGGTTAAACCGAAAATCGAAA

>rnd-1_family-517#LTR/Gypsy ( RepeatScout Family Size = 122 Final Multiple Alignment Size (possibly truncated) = 98 Localized to 2361 out of 4218 contigs )

AAAAAGAAATNAAGGGCAAATATGGAGCAACAAAAAGGGTAAATAGTCATGAAGTGTGTACTAGGAGGAAAGAAGAGCTATAAAGAAGATTGTTGGCAAAATAATGACATGTGCATTAAGGAGGTGTAAGTCACTAAAANTACCTAAATGTNCCCTCCCTTACCCTGAGCCTACGTTACAACCCGANAAAGTCCTATANTGATTCCTCGAGAACTATTTGGAGAGTAGCGGTGACANTANGGGCAAGCCTATGGTNTTGAGCACTAATGTACGTGAATTTCATTTGTGAGCGTGAGTGTTGTTTGATACGAAGTCCTTAATTTATATTCAATCTTTTGAGTGAAAGGCTTTACTAAACACAAACGAGTGAGGGCGTTCATGNCGTGTGTAGGATTATCTAATTGATGTGTTCGTATGAGTTTGCGTTAGCCCGGAAAAATAATAAAAGAGTCATTCTTTGAGCATGTGTTGTAACTTGGTGTAAATGTTGTGAATTGTTGCATCCANTGTGAACTACAAAATCATGATTAAATATGTTTGGTTGATTTTAAGGCGGGTTGATAAGTCCAAGGGTTGTGAAAAAGAGTTGATAGTTTAGAGGTTGTTCGAGGACGAACAAAGCTCTAAGTGTGGGGTGGTGATCTTAGGCGTATTTATATGCCTAAGTTGTCGATATTTGCCCACGAATTGATTAGATTGANGAATGATCTTGAGTTATTAGCTTGATTTTTGGTTTAACTTGCGATGATTGCGCACTAACACACATGATTAGTTGTTTCAGGAACTCTCGAGGGAATTGGAGTCGGAACGAAGAAAGAAGGAGCAAAGANGCGCGAGAAAACGGCTAAGGCGAAAACCACCAGTGGCCGGCGCACTGGTTGGC

>rnd-1_family-0#LTR/Gypsy ( RepeatScout Family Size = 2217 Final Multiple Alignment Size (possibly truncated) = 98 Localized to 2361 out of 4218 contigs )

TACAATGTTGGTCTTATGTCTAATGTTAAATGATAGATGTTATGACTTATTGAATACGAATGTGCCTTGTCTANGGTGACTTCCGAGGAGTACTTAGTGCGAGTAGTAGTANGGGATGCTACTTGCACATTGCACAAGNGTGACTTAGGGTTGTCTTAGGTGATGGTCCTTACGTGATGATATGACTTATGGAATGCTTATGTCTNTATGAATATATGTGGNTGGANNGATGAAGGCTGAATGGTAAGTTCACTCTTGGTGACCTTAAGGTGGTACNTTGCCCAGTGTTCCCGAGGGTGGCNTGGGACGGGACGTTAAGATAAAGAGTGAGGGTTACTTGAAGTGNATGCTTCAATGTGAAGAAAATGTTATATGTGGNTTTATGNGCTATTGTGAAGTATATGACTTTGTATATGTTGGTTGTGACTTGTANGGTGCCTATTATGTTGATGTTCATGAGGTTTTATCAAAATGGCATGAAAGCATAACTTCCAACNAAATGTCCTTTTTAAGCATGTTTTGCATGGTCCTCATACTTAGTACGTGTGTTGTACTAACCCCTTTCTTCTATTTTTACTACAAGTGTAGGTTGCGGTAACTAGGAGGNGATTGTGCCTNGGAGTTGCTTGGATTGCTTCACTCTTCCAAGTCTTTGGTATGTCCTCAAGATTCGAGGACTATGTTTAGAAGTTTCTTAGTCATTGTAATAGACACTTAAGTTTCATTTCGTATTGTAAAGGGTCGTGTCCCTTAGTACGTTTTAGTCTTGTCTAAGATTGGCTANATGAGACTTAGTAGTTCCGCTTTCGTATTGATGTTTTAAATGTGGTGACTCTTGAAGTCTTATGGTTTTATTGAATAAAAGTTTTAAATTCTTAGCGATTTTTATGATCTATGCGATGAATGAATGCTAAGGCTTGTATAAGACCTCCGAGAGGTCGAATACGCATGTAACGGCTAGGGGGTGCTCTCGGGTCGTTACAATGTTGGTCTTATGCCTANTGTTAAATGAAGATGTTATGACTTGTTGAATNTGATGNGCCTAGTCTATGGTGGCTTCCTAGGAANACTTGGNGTGAGTAGTATTATGGGATGCTANTTGCACATTGCACTAGTGTGACTTGGGGGTTCTCTTAGGTGATGGTCCCTATGTCAAGACTATGTCTTATGGTGTGCTTATGACTCTATGAATATATGTGGTTGGATTGATGAAGGCTATATGTAAGTTCACCCTTGGTAACCTTAAGGTGATACATTGCACCAAGTATTCCCTAAGGGTTGCTTGAGGAAAGGAGTTAAGATAAAGAGGAAGGTAATGTATTGTATGCATTGAATGTGCCTAAAATGTTATAAGTGGTTCNATGTGCTATTATGAATGGTTTCCATGTATATGATGATGTACGTGGTTTATATTGTTCCTATTGTACTAAATGTTCTTGAAGTTTCTCTAAAAAGGCATGATGCATGGTTTCCAATTAAATGTTCCTTTTAAAAACATGTTTTGCATGGTCATCAGACTTAGTGCATTCTTGTACTAACACCATTCTTCTTCATCTTTTTACACCAAGTGTAGGTGGTGGTGGCTAAAGGGCCTTNCCTAGAAATAATGAGCTTGGCTTACTATTCCCAAGTTCGGGTGTGTCCTTAAAGATTCAAGGACCTTTATGTTCAAGAGTTTCCTTAAGTTCAATGTACTAGGTAGTAGTTTCTTTTGTATTGTAAGGGTTGTGTCCCTCTTGTGTTGTAGTCGTGTTTTAAGATGGCTACCTTGAGATGTAGTACTCCGATTTTGTATTNAAGGTTTAAATGAGGTGACTTTTAAGTCTTGTGTTATATTATGAAAAAGTTTTAATTTTTCCGCTATTTTTCTATGATAAATGCTATGACGAATGCTAAGGTCTTGTATAAGACCTCCGAGAGGTCGAGTACGCCGGTAACGACTAGGGGGTGCTCTCGGGTCGTTACAATGTTGGTCTTATGTCTAATGTTAAATGAAGATGTTATGACTTGTTGAATATGATGTGCCTAGTCTATGGTGGCTTCCTAGGAACACTTGGTGTGAGTAGTATTATGGGATGCTACTTGCACATTGCACTAGTGTGACTTGGGGGTTGTCTTAGGTGATGGTTCCTATGTGAAGACTATGTCTTATGGTGTGCTTATGACTCTATGAATATATGTGGTTGGATTGATGAAGGCTATATGTAAGTTCACCCTTGGTAACCTTAAGGTGATACATTGCACCAAGTATTCCCTAAGGGTTGCTTGAGGAAAGGAGTTAAGATAAAGAGGAAGGTAATGTATTGTATGCATTGAATGTGCCTAAAATGTTATAAGTGGCTCTATGTGCTATTATGAATGGTTTCCGTGTGTATGATGATGTACGTGGTTTATATTGTGCCTATTGTACTAAATGTTCTTGAAGTTTCTCTAAAAAGGCATGATGCATGGTTTCCAACTAAATGTCCCTTTTAAAAGCATGTTTTTGCATGGTTATCATACTTAGTGCATTCTTGTACTAACACCATTCTTCTTCATCTTTTTACACCAAGTGTAGGTGATGGTGGCTAAAGGGCCTTTCCTAGAAGTGATGAGCTTGGCTTACTATTCCCAAGTTCGGGTGTGTCCTTAAAGATTCAAGGACCTTTATGTTAAAGAGTTTCCTTAAGTTCNATGTACTAGATAGTAGTTTCTTTTGTATTGTAAGGGCTGTGTCCCTCTTGTGTTGTAGTCGTGTTTTAAGATGGCTACCTTGAGACGTAGTACTCCGATTTTGTATTAAAGGTTTAAATGAGGTGACTTTTAAGTCTTGTGTTATATTATGAAAAAGTTTTAAATTTTCCGCTATTTTTCTATGATAAATGCTATGACGAATGCTAAGGGCTTGTATAAGACCTCCGAGAGGTCGAATACGCCGGTAACGACTAGGGGGTGCTCTCGGGTCGTTACAACGTTGGTCTTATGTCTAATGTTAAATGAAGATGTTATGACTTGTTGAATATGATGTGCCTAGTCTATGGTGGCTTCCTAGGAACACTTGGTGTGAGTAGTATTATGGGATGCTACTTGCACATTGCACTAGTGTGACTCGGGGGTTGTCTTAGGTGATGGTTCCTATGTCAAGACTATGTCTTATGGTGTGCTTATGACTCTATGAATATATGTGGTTGGATTGATGAAGGCTATATGTAAGTTCACCCTTGGTAACCTTAAGGCGATACATTGCACCAAGTATTCCCTAAGGGTTGCTTGAGGAAAGGAGTTAAGATAAAGAGGAAGGTAATGTATTGTATGCATTGAATGTGCCTAAAATGTTATAAGTGGCTCTATGTGCTATTATGAATGGTTTCCATGTGTATGATGATGTATGTGGTTTATATTGTGCCTATTGTACTAAATGTTCTTGAAGTTTCTCTAAAAAGGCATGATGCATGGTTTCCAACTAAATGTCCTTTTAAAAGCATGTTTTGCATGGTTATCATACTTAGTGCATTCTTGTACTAACACCATTCTTCTTCATCTTTTTACACCAAGTGTAGGTGATGGTGACTAAAGGGTCTTTCCTAGAAGTGATGAGCTTGGCTTACTATTCCCAAGTTCGGGTGTGTCCTTAAAGATTCAAGGACCTTTATGTTAAAGAGTTTCCTTAAGTTCTATGTACTAGATAGTAGTTTCTTTTGTATTGTAAGGGTTGTGTCC

>rnd-1_family-175#LTR/Gypsy ( RepeatScout Family Size = 512 Final Multiple Alignment Size (possibly truncated) = 98 Localized to 2361 out of 4218 contigs )

TCTTTATCACCCAGGTAAGGCTAATGTTGTTGCTGATGCCTTGAGCAGGTTGTCTATGGGTAGTACCGCCCATGTTGAGGAAGAAAAGAAAGAGTTAGCGAAAGATGTGCACAGACTTGCACGCTTGGGAGTCCGACTNATGGATTCCACAGAAGGAGGAGTAGTGGTGACGAATGGGGCTGAATCATCATTAGTGTCGGAAGTGAAAGAGAAGCAAGACCAAGATCCTATTTTGCTTGAATTGAAGGCAAATGTTCATAAGCAAAAAGTATTAGCTTTTGAACAAGGGGGAGATGGCGTATTGAGGTATCAAGGTAGATTGTGTGTACCAATGGTGGATGAACTCCAAGAGAGGATCATGGAGGAAGCTCATAGCTCCAGATATTCCATCCATCCGGGTTCCACAAAGATGTATCGCGACTTGAGAGAAGTNTATTGGTGGAGTGGCATGAAGAAGGGCATTGCGGAGTTCGTTGCTAAGTGTCCGAATTGCCAACAAGTNAAAGTAGAGCACCAAAGGCCCGGTGGTNTGGCTCAGAATATAGAACTTCCGGAATGGAAGTGGGAGATGATCAATATGGATTTCGTCACAGGTTTGCCNCGGTCTCGCAGGCAGCATGATTCTATTTGGGTGATTGTCGATAGAATGACNAAATCAGCCCACTTTTTGCCGGTAAAGACTACCCATTCGGCAGAAGATTATGCNAAGTTGTATATTCAAGAGGTAGTNAGACTTCATGGAGTTCCGGTCTCCATTATTTCAGATAGAGGTGCGCAATTTACTGCACAGTTTTGGAAGTCNTTCCAGAAAGGTTTGGGTTCAAAGGTGAACTTAAGTACCGCCTTTCATCCTCAGACAGATGGTCAAGCAGAGCGCACTATTCAGACNTTAGAGGATATGTTGAGGGCTTGTGTGATCGATTTCAAAGGTAATTGGGATGATCACCTACCTCTCATTGAGTTCGCTTACAACAATAGTTACCACTCTAGCATCCAAATGGC

>rnd-1_family-390#LTR/Gypsy ( RepeatScout Family Size = 192 Final Multiple Alignment Size (possibly truncated) = 98 Localized to 2361 out of 4218 contigs )

TGTAACACCCCGACTTTTCGAAACGTCTAAATTAACTCGTATCTTCGTGGAAAGACAAGGAGGGTGAGTAATAAATTAAGAATGATGTGGTATGTCATATTTTAAGTGTTCAAGGGTCGTATCTCAAGTTTTGAAGTTAGGTAAGTGGCGAAATAAAAGTTGGCGAAAGTTATCGTAAGTTCCTTTTTAAAGATTTCTCTGAAATTTGGGTCAAATGTCTCGGACGTTTTCTCCCAATATATAAAGAGTTAGAAGGCCCATCACCCATTAAATCGAAGGCCTACGAGTCTAGTTTCCAACGCACAAAACCCCGTATCCANNCGACNTCAGAGTAGAGAGNTATGAGGGTTTTACTACGGACTGCCGGGGCAGAATCCGGGTCGGGTAAAAAATGTGGTATGTCGGCTTACTCAACGTTTTAAGCCATGAAAACATTTCATTTCACTTCCAAACCAGAAAGAAAGCTTAAGAGA

>rnd-1_family-345#LTR/Gypsy ( RepeatScout Family Size = 238 Final Multiple Alignment Size (possibly truncated) = 98 Localized to 2361 out of 4218 contigs )

CGATGNGGTTNCTNATGGCCGATCGANCNGTGAAGAGGCCCGTTGGCGTACTCTATGACGTNCTNGTGAAAGTGGAGTCGTTCATCTTTCCGGCGGATTTTGTGATTCTTGATTGTGAGGTTGATTTTGAGGTTCCCATCATCCTNGGGAGACCATTCCTNGCCACCGGGCGNGCNTTGGTCGATGTGGAGAAGGGGCAGATGAAGTTCCGACTGAATAATGAAGAGGTAACTTTCAATATTTGTAGGTCCATGAAGCAGAGTGGTGAGCTCCAAGCGGTATCTGCTATAACTTACGGAGTGGAGAGTGGGACAGAAGTGCGAATTGAAGAGNGGCTGGGTGTTGAGGCACTAGCAGCGGTTATGATGAATTTCGATAGTGATGGTATTGAAGANTACGATGAGTTGGTAGCCGCACTCGATAGGTGCGAATACCGGTCCAAACCAAAGAAGTTGGANNTAGACATGAAGAATCGCGAGTCCCCACCCGCAAAACCGTCTATTGAGGAGCCACCGAAACTGGAGCTNAAGGCTCTACCATCNCATCTGAGGTATGTATTCTTGGGNGNAGATAACACTTTGCCGGTNATCATTGCGGCAGATTTGAATGCGAGACAAGTAGAGCGCTTGGTGACTGTGTTGAAGAGGTTCAAGCGAGCTATTGGGTGGACTATTGCNGACATTATTGGGATTCCTCCCGGCATTTGCNCTCACAAAATCCAACTCATGCCGGATCGCAAGCCNAGTATCGAGCACCAAAGGAGACTAAATCCGCCTATGCAAGAGGTGGTGAAAAAGGAGATCATCAAGTGGTTGGATGCNGGAGTNATNTACCCCATTGCGGATAGTAGNTGGGTNNGTCCNGTGCAGTGTGTGCCNAAAAAGGNGGNATNACTGTGGTTCCNAATGAAAGAACGAGCTTGTTCCGACGAGGCCNGTGACCGGATGGAGAGTNTGCATGGACTACCGGAAGCTGAACGCGTGGACNGAAAAAGACCACTTCCCNATGCCNTTCATGGATCAGATGCTNGATNGGCTNGCAGGNAAGGGTTGGTATTGTTTTCTNGATGGGTATTCGGGCTACAATCAAATCTCTATNGCTCCGGAAGACCAAGAGAAGACCACCTTCACTTGCCCTTATGGGACTTTCGCNTTCAAACGGATGCCATTCGGGTTGTGTAATGCTCCGGCGACNTTCCAGCGNTGTATGA

>rnd-1_family-480#Unknown ( RepeatScout Family Size = 136 Final Multiple Alignment Size (possibly truncated) = 98 Localized to 2361 out of 4218 contigs )

TATCGTATCGTTTAAATTCATCGTTAGGTAACGACGAAAAGNCCCATTTTATGTAACGACCGATTTGGTGTGATCGCGTCGTTACCTTAATATTTTCTTCTCATTTTGTCTTTACTTATTATTTAATAATNNTATTTTATCCTTTACCCTACCTTTTCATAGTAGCTCTACCTCGTACCCTACTTTTCTTGTAGGTTTATCATTCAAATTATNGATGTGTGACATTATGTAACGACGGAGAACGATACAATCTATCCAAACATTGTATTCATTAAAACAATACAATACGATACAATACAATACGATACATTATGAAACA

>rnd-1_family-565#LTR/Copia ( RepeatScout Family Size = 104 Final Multiple Alignment Size (possibly truncated) = 98 Localized to 2361 out of 4218 contigs )

ATAAAACATGTCTTATCACCTCTTCAAATAAATGGGTGATTGATTCAGGTGCCACAAATCACATGACAGGTAATCCTAATATTTTTTCTAGCTTTCAATCACACAAAGCACCCTCTCCGGTTACCGTAGCTGATGGATCAACTTGTAACAGTGTTGGATCTGGGACTGTTAAACCAACCTCCTCTATTACCCTGTCATCTGTATTAAGTCTACCAAAGTTGGCCTTTAATTTGATTTNTGTTAGTAAACTTACCAGAGACCTTAATTGTTATATCTCGTTCTTTCCCGATCATTGTTTGTTTCGTGATCTTANGACGAATCAGGTTATTGGTAAAGGACATGTATCTGACGATCTCTACATCCTTGATGAATGGGAGCCTCGATTTGTTGCCTGCTCTAGTGTCGTGTCTCCATTTGAAGCACATTGTCGATTGGGACATCCTTCTCTACCTCTGTTAAAGAAGCTTTGTCCTCAGTTCCAGAATATTTCTTCATTGGANTGTGAGTCGTGTCGATTTGCGAAACATCATCGCANCTCATTAGGTCCAAGGGTTAATAAGCGGGCTGAGTCAGCTTTTGAGTTAGTTCATTCNGATGTTTGGGGACCATGTCCTGTCGTTTCCGAAACTGGGCATAAGTATTTTGTTACTTTCGTNGATGATTTTTCCCGAATGACTTGGATTTATTTNATGAAGAGTCGNTCTGAAGTGTTTACTCACTTTCGTGCCTTCTATGCTGAAGTCAAAACTCAATTCGATGCTTCGGTACGTATTCTAAGGAGTGATAATGCCGNAGAATATATGTCAGAATCGTTTCAATCGTACATGAGACAACACGGNATTNTTCATCAGACNTCNTGTGTCNATACACCCTCACAAAATGGNGTNGCTGAGAGGAAGAATAGACATCTNCTTGAGACGGCTNGAGCACTTTCGTTCCAANCGAAGGTTCCTAAACAGTTTTGGGCGGATGCAGTCTCTACNGCTTGTTTTCTGATTAATAGAATGCCATCTACTGTNCTTGCTGGTAATGTNCCTTACAGTGTTCTNTTTCCAAACAAGTCACTATTTCCGGTGGAACCTAAGGTGTTTGGAAGCACGTGTTATGTTCGAGACGTTCGACCATCTGTTACTAAGTTGGATCCNAAGGCNTTGAAGTGTGTTTTTCTGGGTTATTCTCGNCTTCAAAAGGGGTATCGGTGTTATTCTACTGAACTTGGCAGATATCTNGTGTCAACTGATGTGGTATTTTCNGAGACTACACCATTCTTCTATGCACCTCCCATTTCTACAAGTCAGGGGGAGGAAGATGAGTGGTTAGTCTATCAGGTTACCCGTGTTGTGACAAAANAATCAGAT

>rnd-1_family-185#LTR/Gypsy ( RepeatScout Family Size = 494 Final Multiple Alignment Size (possibly truncated) = 98 Localized to 2361 out of 4218 contigs )

CCTATTGGATGGTTTGAAGTTGGTGAAGCTGGGTTGATAGGACCAGACTTAGTTCATCAAGCTATGGAGAAGGTGAAAGTNATTCAAGAGAGGTTGAAAACGGCGCAGAGTCGTCAGAAATCCTACACTGATGTTAGGAGAAGGGAGTTAGAGTTCGAAGTAGATGATTGGGTNTACTTGAAAGTTTCACCCATGAAGGGTGTTATGAGGTTTGGTAAGAAGGGGAAGCTTAGTCCCCGGTATATTGGNCCTTACAGAATATCCAAGAGGATTGGCAATGTAGCTTATGAGTTGGAGCTACCGCAAGAGTTAGCAGCGGTTCATCCGGTATTTCACATCTCCATGTTGAAGAAGTGCATGGGCGATCCTTCACTGATCATACCAACTGAAGATATTGGGATCAAGGATAGCTTATCTTATGAGGAGATTCCCGTTCAGATTCTAGATCGCCAAGTTCGCAAGTTGAGAACNAAGGAGGTAGCATCAGTCAAAGTCCTTTGGAGGAACCAGTTTGTTGAGGAAGCTACTTGGGAAGCTGAGGAGGATATGAAGAAGAGATATCCACATCTCTTCGAATCCGGAGAAATTCCAGATCAAGGTACTAATTCTCTTCTTAGTACTCTTTAATTTATAAGTTGGCATGTTGTATTTGCGTTGCTTGTTG

>rnd-1_family-214#Unknown ( RepeatScout Family Size = 438 Final Multiple Alignment Size (possibly truncated) = 98 Localized to 2361 out of 4218 contigs )

TTTTGGGGTTTTTCAACTTTGCTCTTGAANTTGACAAATTGAAGTTGAATTCGAAGTGGGTTTGCTTGTAATTGCTTAATTTCACGTTACCCATGGCTGCTTTCATGNAAGGTATAATTTCTTTACTTTTTGTGATGNGTGGCTAAAACCCCAANATCTAGGGGTGCGATTATGGGGTTGGGTGTTGATTTAATGTAGTGGGTATTGTGTTNTGCTCTGTTTTATTGTTGTTCATNCATGGATTTGGGCTAATTAGCATGGGGTTAATTTATGGGTTAAGGTTGCAAACTTAATTCCTACTTTCGATCTCCGATTGATGCTCGAAAGAGATCGATTGGAGTGGGTAATTAGTTCGATACTTGCAATTTAGGTTTGATGTCTGTAGCTTGCTCGAAAATAGAGGTTATGGGTCGGATCTATTTGCCCTATTTCTGCATCGAAAGAGAGAATANAGTAAGGTATTGGGTTGTTTGGATTGGCACATAGATGAGACCGAAAGGAGATTCTATGAANNGATGCTATGAGATCGAAAGAAGATTAGCGTCATTGAAGATGACCTAGCCTATCCATGANTGTTCANCACTATNGAGCAACACAATTACCCCTATGTGACATCGATTTCCAATCCCCGCACCCCTAGATTGCTTNTTACTTCTTGAATTTCTTGNAATTTTAGTTTAATTGTGATAATTGATAACCAAATCCTTTTATTAAATTGGTGGAATCATCCACCCACTTGTTCTTACTACTACAAATGAATNAGTTTACANTCCAAATTTCTTGATAGAATTCTCTACGNTATACCATTCCCTGTGGGATTCGACCCCGACTCTTNGTTGGGTATTTTTATTGACGACGACCGCTTACTCTTTTAATTGGTTTTGAAGAGTAATTTGAGCGTTAT

>rnd-1_family-99#DNA/hAT-Tip100 ( RepeatScout Family Size = 751 Final Multiple Alignment Size (possibly truncated) = 98 Localized to 2361 out of 4218 contigs )

AGGCATAATACATAAACATGNCCCTTAACTTGGCTTCAGCTGACANCTATGCCCTCCAACTTTGGGTGTGCACAAGTAGACACTTAAACTTGTATAAAATTGAACAAATAGACACACGCGTCCTACGTGGCATNNTACACGTAGGACGCCACGTGGGACGCAAATTCCCGTGTATTATGCCACGTAGGACGCGTGTGTCTACTTGTTCAATTTTATACAAGTTTAAGTGTCTACTTGTGCACACCCAAAGTTGGAGGGCATAGTTGTCAGTTGAAGCCAAGTTAAGGGTCATGTTTATGTA

>rnd-1_family-468#LTR/Copia ( RepeatScout Family Size = 142 Final Multiple Alignment Size (possibly truncated) = 98 Localized to 2361 out of 4218 contigs )

TGAATCAGTATACCCAACATTAGGGGGAGAACANAAGCAGTTGAAAAANGAGATAGATTGGAATTCATTATCACTATCTCATTTAGATCCTCGAACAAATNAATGTGAGCAAGAGGTTCAAAAGATNATTCATTTGCAAAATATTGCAAATCAACTGCCAGACGCATTTACTGACCTTCCAAGGGTTACTAAATCGCATATTCCAGCNGCGAATGCTCCAGTTCGAGTTGATGTCCCGATAGGACAAATTGTTAAAGCAAATGAGTCTANANCACGCCTGAAGCGTGGTAGACCAATCGGTTCCAAGGATAAAANTCCTCGAAAAAGGAAAAGGAGCAAATGATCAAGATGATCATGATAATGGAGGCAAGTGCTCAAGAAGAGCNCCANGACATAACANTTCATGAAACCNCGGAGAAGGTTCAGGTACCTGAAAATGATGAGAATGAAGAGATCTCGATAAGTTATGTCTCGACGGGAAAAAGGTGGAATCGAAATAATATCGTGGTCGACGATATTTTTGCTTATAATGTTGCGCTCGAAATAATGCAACAAGATGAGGATCTTGAACCNAAATCTGTCAATGAATGTGGACAGAGAAATGATTGGCCAAAATGGAAGGANGCAATTCAAGCAGANTTGGCTTCACTTGAAAAACGTGANGTTTTTGGACCNATAGTCCGAACACCTGAAGGTATAANGTCAGTGGGGTACAAATGGATTNTTGTGCGAAAACGAAATGAAAAAGGTGAAATCGTAAGATATAAAGCGCGACTTGTGGCACAAGGNTTTTCGCAAAGGCCTGACATTGATTATGTGGAGACGTATTCTCCTGTGGTGGATGCAATGACCTTCAGGTATCTAATAAATCTGGCAGTNCATGAAAANCTTGAAATACGTCTAATGGATGTTGTCACAGCCTATTTATATGGCTCACTGGACCACGANATTTTTATGAAAATTCCTGAAGCATTCAAAGTGCCTGAAGCATATAAAAATTCNCGAGAAANTTGTTCAATAAAGCTTCAAAAATCNTTATACGGATTGAAACAATCAGGGCGNATGTGGTATAATCGCCTTAGCGAGTATTTGNTAAAGGAAGGGTATAAAAATGACCCNATTTGTCCTTGTATTTTTATNAAAAGGTCNGGATCTGAATTTGTTATAATAGTCGTNTATGTTGATGACTTGAATATCATTGGAACTCCTAAAGAGCTTTCAAAAGCNGTTGAGTGTTTGAAGAAAGAATTTGAAATGAAAGATCTTGGNAAGACAAAATTTTGTCTTGGCCTACAGATTGAGCATTTGACAAATGGAATATTTATCCATCAATCAACATACACNGAAAAGGTTTTGAAGCGATTTTACATGGATAAATCACA

>rnd-1_family-532#LTR/Gypsy ( RepeatScout Family Size = 118 Final Multiple Alignment Size (possibly truncated) = 98 Localized to 2361 out of 4218 contigs )

AGGTAATTAAATATTAGGTGTATCGTATTATATGAATACCATTAGAGTTATGNTAATTGGAGGAATTGAGACTTAACCCTAGGCTTGAAATTTAGGAANTTGATTATAGATTTGGGTGAGTTTTTGGGTCTAGGCTAGACATATAGTAATATGGGTGTTTATAGACTCGTTAACTTACGAATTATGCATATATACTTGATAGATTGGAAACGTTCGGAGGCATTGAGGAAAGGAAAAGCATTGGAGAAGTAGCTTGCTTGACTTCGGTTCTTCGGTGGAGGTAGGTTATGGTTTATTCTATGTGATAGATAGACTCTTAATAGTGATTGATATTCATTGAGTGATATTGTGAANTTCTCTATGTACTTGATTGTGTGGTTTGGATAGNTCGTGTGGTTTGTGATTGGTCTGAAATCCCGAGACCGCGAAATTCATAATCTTGAACCTTCTCTATCGAAGTGATGCCTTGAATAAAGAAGGCTTGATGAAATATTGTTAATGAAGTGAAAAGTGGTGATAATAAATGATAAATTA

>rnd-1_family-21#Unknown ( RepeatScout Family Size = 1322 Final Multiple Alignment Size (possibly truncated) = 98 Localized to 2361 out of 4218 contigs )

AAGAGGGACTAGTCCCCAACTAAGGAACATGAGTATGATGACCTAAAGGAGTACTTAGTATGGGTAGTAGTATGGGATGCTATTCATGCATTGCACAAGTATGACTTAAGGTTACTTATGAAGTGTTCTCTTATATGATGTATGCTTAGATTGGATTGTTGCTATAGTTGCCTTCCNNGATATGATTGACTAAAGGTGANAGTTCCCTTGTCTATGNGAAGTAAGCTAANAATGAGACCAAAGGANGGTAAATATGACTANGATGACTTCAAGGTTATGCTTAGTGTGAAGGGTATTATGGGATGCCTTTCATGCATTGCACAAGTGTGCCTTGAGGTTACTTAGGAGTATGGTNCCCTTATGGTANAAAAGCTTAGAGACTTGACCATGTATATAGACTATGATTAAGATGACCAAAAAGGGATACTTGGCTTGGGTGGTATTATGGGATGCTATTCATGCTTTGCACATGTATGCTTTTGAGGTTACTTGAGAATGGTNCTATTATGGTATAGATGACTAAGAGTCGAATTATGCTTATATGATGCTTATGTTGGAGACTTTGCTTATTATGATTATGATATTGTCTCTTATGCTTATGGTTTATGTTTCATGAAGTATTTCATATGATTATGGATTATGT

>rnd-1_family-418#LTR/Gypsy ( RepeatScout Family Size = 171 Final Multiple Alignment Size (possibly truncated) = 98 Localized to 2361 out of 4218 contigs )

AGTTTCAGTTTCAGGTGGTATCTCTATTACCCTTTTACAGTAACTATTTATGATTCTACTTTCCGCCTTNCATACTCNGTACATTNTTTCGTACTGACGCCCCATTGCCTGGGGGCGCTGCATTTTTTCGTGCGTGCAGGTCCNGGCAGGCGACTCTGATNGACCTCCCCGGTAGGATTCAGGTTCANCTGCGAGTTGGCNAGCCCCATTTCCTCCTGGAGCTGCCAGAGTTGGGAGGTTTGTTACTTTTGTTGTACATATTTTTATGGGTATGGCCGGGGCCCTGTCCCGACAGTGCTTATGCCACTCNTAGAGGCTATTGCGGACACAGTATTCTGGGTTTTCTTTTTGTATGTTTCAACCTCCAGCCAATAGNCTTGGCATGTATATATATGTGTGTGTGTAAGTAGGTATGTCTTCGGTTGTGGCCTCGTCGGCTAGATAGTACATCTTTATTTTTCGGCTTGTCTACCATTGTTTGTATGGTTTATTCTCNTTCAGGTCATGACCTTCNGGGTCCAGGCTTCACTTTTTAGAGGTTGTGCTGCCCGATCTTTTTGCCTCACAGTTTGGTTTAGCTAGTATGTTTCAGTGGGTAACTCGATGTATTAGGGTATCGAGTGCCAGTTACGCCCCCTATTTCGGGGCGTGACA

>rnd-1_family-183#LTR/Gypsy ( RepeatScout Family Size = 495 Final Multiple Alignment Size (possibly truncated) = 98 Localized to 2361 out of 4218 contigs )

TTGAGTAAAGTTTACTTTTGAAGTCTTTTATTCTTGAGTAAGTCTTCCGCTGAGAGTTAAGCCAGGCCAAGGGTTCGCTTGGGGCCAGCAATGGTTCTCGAGTGCCGGCCACGTCCAGGGTGTAGGCTCGGGGCGTGACA

>rnd-1_family-370#DNA/CMC-EnSpm ( RepeatScout Family Size = 217 Final Multiple Alignment Size (possibly truncated) = 98 Localized to 2361 out of 4218 contigs )

AAATCCCAGAAAAGCGACGGACAGNGTCGTTTNGTCCGTCGCTTTTCTGGGAAATTTATTTTTAAAAACCCAGAAAAGCGACGGACAGCGTCGCTTTGTCCGTCGCTTTTCTGGGTTTTAAAAAAAATTAAAA

>rnd-1_family-282#LTR/Gypsy ( RepeatScout Family Size = 317 Final Multiple Alignment Size (possibly truncated) = 98 Localized to 2361 out of 4218 contigs )

GAAAAGTTGAGTGTTCTTGATGTTCATGATTTGGGTTGGCTTGAAATTCTCACCTTAAGAGGTGTAGTGAGTTTGAGAAGTTTAGTCCCGGTTTGGTAGGCTCATATGAGATCTTGAGTGGTGTTGGTAAGGTTGCTTGTGAGCAAGAGTTGCTTAATGAGAACCTCTCTAATTTAGAAGTACCGGTTGGGATCTTAGACCGGCAAGTTAAGAAATAGAAGAGTAAGGAAGTTGCTTCCATGAAAGTGTTGAGGAGTGGTATTTTAGTAAGGGTTTTAGTTGAGGGTGCTACTTGGGAGGCCGAGGGCCAATGAGATGTCCCGATATCCTCATCTNTTTCCTTCTATTCCTCCTCTAGCTTGAGATGATAAGTTCCCCTTGACTCGTTTTATGATTTTGGAGTCNTGCGTGTTATGTGTTTCCATGATTTCCTTGTCCCATAGAGTTACTCTTGTTAACCTTGGTAGGGTTTGGATGCATGGTTGTTAGACCTCTATTGGTTTTAGAGCCCGGTAAGGTTATAATGAGCCTTGGTGATGCTTTGGGNTTGTTTGAAAGGAGTAGTATTTGAGGTTTTAGTAAGGGGCTTACTCCCAAGGGGGGGATGGTATGCTTGAATAGCAAGGTTGATTGAGAGTCCCGATATCCTCATCTCTTTCCTTCTACTCCTATTCNAGCTTGAGGCAAAGAGTTCCTCGTGGCTTGTTTTATGTTTTGGAGTCATGTGTGTTTTANGTGTTTCCATGATCTCCTTATGTTATGCATGTTCTTGAGAAATTCNTGTTTCGTATAAA

>rnd-1_family-9#LTR/Gypsy ( RepeatScout Family Size = 1509 Final Multiple Alignment Size (possibly truncated) = 98 Localized to 2361 out of 4218 contigs )

TGTAACACCCCGTATCCGAAACAGACCAAAAATGCAGATTTCGAGAAGTTGCAGGTGCAACCCACGGTCGCCATCCACGGACCGTAGGTCAGACCACGGCCCGTGCTGGTGGTCCGTGGTTCACCACTGCAACCCCTCCCCAAACCCAGCTCAGAAAATCGGCTAAGTCTCGACCCACGGACGGACCCACGGTCCGTAGGTCAGACCACGGTCCGTGGTCTGTGTCCGTGGATCGAGACCTCCTTTACCCAGCCTCTGACGCGAACNACGGTCGACCAGCACGGACCGTCGTTCGATCCACGGTCCGTAGGTCTGACCGTAGGTGAGGGTCAGCAGCCAGTTAGATGAAAATTGTTGATGGGTCAACTTCAGATGGTCATAACTCTTAGCACAAAAGGAATTATGTGTCCCATGACCTATGATATGATAGATAATTGAATCCTCTTTCCAACGCCACCGAGTTTGCTAAAATCCGACCTCCGAGTAAAAAGTTATGCTCGTTTTAGTGAAGCCCTGTCGGGCAGACTCGACCGACGACCCCAATCGACGGACCGTNGATCGACCNACGGCCCGTCGGTCCGGTCCGTCGGTCGCTCCGACAGCAGTTAAGTCAGGGGTCTTTTGGTCTTTTCCTATTTCGTTGGACCCCTAAACTACGTCGTTTAGACCCTAAACTACGTGGTTTTGGTCAGTTTNAGCCTAGAAACATAACTAGAACTTACCTAAGTCAAATCATTNATCAAAACTTAGAAAAATTAGAGCGNAAGAGGAGAAGAGAAGTCGAGAACCCTAGTTCAAGAACGCAGCAAGGTTCCATCAGTTCCAGCCCCGAAATCGAAAGATTTCTCCGTGGAATTCGTCACCAGGTATGTGGGATTTCACTAGTGGGTTCCTTTCGCCCATTAGGTCCCTAGAATTCAGTCAGATTCTTGATTCCCTTATTATGACTAGACCTAGGGTTTCTAGAATTTCAGCAGATTATCGTGANTTAGTTGTTAGAATGNTCCAAATCAGATTATCATGTTATTACTTAGTTTCTTGCATGAATTTCAGAACCCTAGCTATGTATTTCTTTAGTTCTTGAATTACACATGCTAGGTCAGATATTTCAGTTATTCAGATATACATGCCTCAGTTTTNAATGCGTCATTATCAGTATATTAATTGTTGCATTCTCAGTTTGCATGTTCAGTTTTGAGCTATCCAGTATNTTACAGAAATTCAGTCATAATGTGTTAATCACTTAATCCATTGGGAGTAGCNTAATACCGAGTTGGACTAGGGTTCAGCGCACCCNCATAGTCCCAGAACTACGNGCCACGTAGGTTGTAAGTCCCCTCTGTGGGCATCATTTTAGTGATCACGCCAGTCATGCCTCTATACCTCTGGCAGGGTATATTGGGTCCTCTCGATGGGGCGTATACATCGGACTCCACATTTAGCTCATGTGGTTTTATGTCGGTTATTAGTAGCTCCCACAGTTCAGTCAGACTCTATTGCATTGACCACGTTTCAGTACAGTCAGTATTCAGTCTCAGCATGTTATAAACTTGGTCATTGCATTCAGTTAGCTCAGTATTCAGTATTTTCAGTATCTATATCATGTTCAGATTATATCATTGCTTTATTGCTTTGTTCAGTTATATGTTATTTCAGCTTTACTCTATCCTGCATGCTCAGTACCTTTCAAGTACTGACGCATACTCTGCGCTACATCTTCTCGTGATGTAGGTTCAGGTCCTCAGCATCCAGATCACGCNTAGATCGGTTCCCGATCTCCAGTTCAGCAGCATCAGTGGTGAGTCCTCATTCTTCGAGGACGATAGTCATGTTTCTTTTCAGTATTTTTAGTCTTTAGTTTCAGTTTTGCTAGACTTAGCTGGGGCATGTCCCAGCATTTCTAGTCAGTTAGAGGCTTATTTCAGACATAGTTAGATTCAGCTTAGTATTCGAGTTTGATATTTTCTTTGTATTAAACTCATCAGTTTTCATATATTCAGTTATAGTATATGGGTATTCCCCATCATTTCANTTTATTTATGATTTAGCTTCCGCATCAGTTTATTTATTATCTTTAGTATGCTCATGATCATGCCAGCAGGGTTAGCTTGGGGTCACTCGTGATCCTAGGTCCCGTGTCCGCGTCTCGGGGGTAGCCTCGGGGCGTGACA

>rnd-1_family-336#LTR/Gypsy ( RepeatScout Family Size = 247 Final Multiple Alignment Size (possibly truncated) = 98 Localized to 2361 out of 4218 contigs )

AGGGGTATTTTGGTCTTTTCCTTACCCCTNCTAAGCCTAAACCACGACCGTTTTCACCCAATTAAGGGTCTAAACAGTGTTAAAATATTCTTTCACGTTCNTTAACACTTCAAAGACTTAGAGCAAGGNTTCAAGAGGAGAAAAGCTAGGGTTTCAAGCGTTCTTCGAGTTCGTCGATTTCGCCAAGAACCTTGTTCTTCCAGGTATGTAAGGCTATCATAGTGTTGGACTAGTTCGTCCTCACGCCCTACATCTACTTTC

>rnd-1_family-45#Unknown ( RepeatScout Family Size = 1112 Final Multiple Alignment Size (possibly truncated) = 98 Localized to 2361 out of 4218 contigs )

TTTTGGTTTAGTTGCTTGGTTTGGGTTGTAGTAATGGTTCTCCCACCGGAGGGTTAGTGTGGGTGCCAATCACGACGGTCTGGGTCGTGACAAAGTTGGTATCAGAGCCTAGGTTCGTTGATCTCGATGTACCAAAACGAGTCTAGTAGAGTCTTGCGGAACGGTACGGAGACGTCTGTACTTTTCTTCGAGAGGCTATAGGACTTTAGGAAATCTCTCTGTTTTCTCTTGTCTCCTTTCGTGCTATAACTTGATTCCAATTGGTATCTGGCGATTCAAATTGGTATCTAACCTCCTTCACTCTTCTGTCCGCAGATGGTTAACACTAGATTCAACGGCGTCAGGCCCGTAGCTCCCGTCAATGCTCCAGCTGAGGAATCCGCAGCGAGAGGTCGCGGTCGAGGCAGGGGTAGAGGAAGAGCNAGGGGTAGAGGCCGAGGAAGGGTAGCGCCCGCTAGGGATGGAGCNCCGGTTGAGAATGCTCCCAGGAATGAGGCCCCTCCTGCGCATCATGAAGAGATAGAGGAGAATGTNGAGGTTGAGGATGNNGAGGATGTTGGACAAGAGGAAGAGGTGCAGGCTGAGACTACAGGTATTCCTCCCTTAGACCCAGTGTTAGCTCAACAGATCATGTCGTTCTTGAAAGGGTTGGTTGGTCCTGGAGTGCTTCCCTCTGTTCAAGCAACTCAAGCTCCCGCCAATCCCCCTATTGCTANCACTGTNCCCAAGGTGGGTGGAACNGTAGGTANTGATGCTTTCTTCCGTCCTTTGTTGGGTCCTGTTATGACTGGTAATGAGCATGAGATGTTGACTAAGTTTTTGAAGCTGAAGCCGCCTGTGTTCCATGGTTCTGAGAGTGAGGACGCCTATGAGTTCATCCTAGATTGCTATGAGAGGCTTCATAAGTTGGGAATTGTCCATCAGCATGGGGTTGAGTTCGTGACCTTTCAGCTTCAAGGTGAGGCTAAGCAGTGGTGGAGAGCTTATGTGGAATGCAGATCTTCAGCTTTACCTCCACTCACTTGGACCCAGTTTCATGCTCTGTTTCTGGAGAAGTATGTGCCTCGGACTTTGAGGGATCGCAAGAAGGATGAGTTCATGGCTTTGGAGCAAGGTGGTATGNCTGTGGCTGCTTATGAGGCCAAGTTCCATGCTTTGTCTAGATATGCTACNCAATTGGTGACTACTGAGGAAGAGAGGATCCGTCTATTTATTAAGGGACTAAATTCCGAGTTGCAGGTATTGTCTGTTCATATGACCTCTGCAGGGAGAAGCTTTAATGAGGTGACAGACTTTGTGAAGAAAGTGGAGGGGGTGAGGCGAGACGGTCAGGCTAAGGCATTGGCTAAGAAGGCCAAGAACTCGGGTAACTTTCAGGGTTCTTACTCCAGAGGTTCAGGNAGGCCGACGCTTGCAGCCCGGCCAATTCAGTCCGCTATGCCCGCCTCTACAGGTAGTTACTCGGGAACTCCACCTCATAATTTGATTCAGGATAGCCAGGGAGTCGCGCCTTCGACGGGCGGCAGGCCATCTTTTGATCGTACTTGTTACAACTGTGGAGAACCTGGGCATATGAGGAGAGATTGTCCCCACCCGCGCGTGTTGGATTCCGCGCAGCAGCAGNCTAGAGCAGTGGTACCCGCGGGNAACGGTAATAATGGTAGAGGACGTCCACAAGGTGGGCGAGGAGGNAATCAGCGAGGCCGTGGAGGTAGGGGAAATGGTAACGCAGGCAGAGGTACAGCGCAGCCAGGCAGGGAAGTCGCCCGTCAAGATGACAGGGCTCAGTGTTATGCCTTTCCGGGCAAGANCGAGGCGGAGGCGTCTGACGCGGTGATCACATGTACTATTCTTGTCTGTGACCGGATGGCTAATGTGTTATTTGATCCGGGTTCTACTTATTCTTATGTGTCCGTGCGATTTGCCTCGGAATTTGATATGATTTGTGATATACTTGATGCCCCCATCCATGTTTCTACCCCAGTTGGAGAGTCTGTCATAGTCACCCATGTCTATCGTGCTTGTCCTATTTTGTTTATGGGTTTTCAGACTTGGGCTGATTTGGTGATTTTGGATATGACTGACTTTGATATAATCTTAGGCATGACTTGGTTGTCCCCCTATTATGTTGTGCTTAATTGTAATACTAAGTCTGTGACTCTAGAAATTCCGGGAAGGGAAAAGTTAGAGTGGGAAGGGGTGTACAAGCCTAAGCGAGCTAAGATCATATCCTCCATTCGGGCTAGGAAACTGGTAGGGCAGGGTTGTTTGGCTTATTTGGCTCATATTCGGGATGTTGAGGTTGAGTCTCCATCTATTGAGTCTATTCCTGTGGTGTCAGAATTTAGAGAAGTGTTTCCTACTGATTTGCCTGGTATGCCTCCGGATAGAGATATAGATTTCTGCATTGATTTGGAACCTGGTACTCGCCCCATTTCTATCCCTCCATATCGCATGGCTCCGGCAGAATTAAGAGAGCTTAAGGCTCAAATCCAAGAGCTTCTTGATAAAGGNTTCATTCGTCCTAGTGCTTCCCCGTGGGGTGCTCCAGTTTTGTTTGTTAAGAAAAAGGATGGTAGTATGAGGATGTGTATAGATTACCGGCAATTGAATAGGGTCACCATTCGAAACAAGTACCCNTTGCCTCGAATAGATGATCTTTTTGACCAATTGCAAGGTGCATCAGTTTTCTCTAAGATTGATCTAAGGTCTGGTTACCATCAGTTAAAGATTAGGCCTGAGGATGTGCCCAAGACGGCGTTTAGGACCCGCTATGGGCACTATGAGTTTCTAGTTATGTCGTTTGGNTTGACCAATGCGCCTGCAGCTTTCATGAGTTTGATGAATGGNGTGTTCAAGCCATTTCTTGATTCGTTCGTTATAGTCTTTATTGATGATATTTTGGTCTATTCGAAAAGTGAGGAAGAGCATGCCGACCATCTTCGTATTGTTCTGGGTGTTCTTGGGAAACAAAAGTTGTATGCNAAATTTTCTAAGTGTGAATTTTGGTTGACTTCGGTTGCATTTTTGGGGCATGTAGTTTCAAAAGAAGGGGTAATGGTAGATCCCCAAAAGATTGAGGCGGTTAAGAATTGGGTTCGGCCTAGCTCTGTGACGGAAGTTAGGAGTTTTGTGGGGCTCGCTAGCTACTATCGTCGATTTGTGAAGAACTTTGCTTCTATCGCCACNCACTTGACNAATTTGACCAAAAAGGAGGTACCNTTCGAATGGACTGAAAAATGTGAGGAGAGCTTTCAAAAGCTCAAGACTCTCTTGACCACCGCACCTATTCTAGCACTACCGGTGGAAGGTAAAGATTTTATTGTTTATTGTGATGCTTCACATTCNGGTTTGGGTGCTGTGTTGATGCAGGATAAGAATGTTATAGCTTATGCATCGCGNCAGTTGAAGGTGCATGAGAGGAATTATCCAACGCATGATTTGGAGTTGGCAGCGGTAGTGTTTGCTCTTAAGATCTGGCGGCATTACCTCTATGGTGTCAAGTGTGAGGTGTTTACTGATCATCGTAGTTTGCAACATGTGTTCACTCAAAAGGATCTGAATTTGAGACAGCGAAGGTGGATGGAGTTACTCAAGGATTATGATGTNACCATTCAATACCATCCGGGTAAGGCTAATGTGGTGGCAGACGCNTTGAGTCGAAAGGCGGTGAGTATGGGTAGTCTAGCTTGCTTGAGTGTANCCAAGCGACCTTTGGCTAAGGAAATTCAGACCTTGGAGTCTAAGTTCATGCAGTTGGGCATCTCAGAAAAAGGTGGGGTGTTAGCTAGCATTGAGGTNAGGGCCACGTTCATTGAGGAGATCAAGGCCAAACAGTTTGAGGATGAGAATTTGAATGAGCTTAGAAAGAAGACNGCGATTGGTAAGGCACAAGAGACCACTCTTGATGCGGAAGGTGTGCTCAGTTTTAAAGGAAGGATTTGTGTTCCTNGAGTNGATGACTTGATTCAGAAATTGTTGACAGAATCCCATGGTTCGCGATATTCTATTCATCCGGGTGTGACCAAGATGTACCGAGATTTGAAGCGAATTTATTGGTGGCCGGGCATGAAGAAAGATATAGCGGAGTTTGTGGCTAAGTGTCAAAATTGTCAACAAGTGAAGTATGAACATCAAAGGCCTGCAGGTTTGCTTCAGAGAATGCCAATTCCGGAATGGAAGTGGGAAAGGATAGCCATGGATTTCGTGGTTGGTCTTCCCAAGACTTTGGGGAAGTTTGATTCTATTTGGGTAGTGGTTGACAGATTGACTAAGTCAGCCCATTTTATTCCGGTAAGAATAGATTATAATGCTGAACAATTGGCTAAGGTTTATGTGAAAGAGATAGTGAGGTTGCACGGGGTGCCCCTTTCTATCATCTCAGACCGTGGTACGCAGTTCACATCCAAGTTTTGGAGGAAATTGCATGATGAATTGGGCACACAACTCACTTTT

>rnd-1_family-279#Unknown ( RepeatScout Family Size = 318 Final Multiple Alignment Size (possibly truncated) = 98 Localized to 2361 out of 4218 contigs )

TGGTGAGTCCTCATGTTCCGAGGACCGNGNCACCTTTATTGCTTTCTTATGTCTTTATTTTCGGACGTTGTACGTGGTGTACGGGTTACGTCCCGACCACTCCTTNTGATGTANTAGATGGCTTGTCGAGACTATGTTAGACTTCCGCTTTATGTCAAACTCTTTTATTGATATGAGATTCTCTACTT

>rnd-1_family-377#DNA ( RepeatScout Family Size = 210 Final Multiple Alignment Size (possibly truncated) = 98 Localized to 2361 out of 4218 contigs )

TTATAGGAAAAATTACAGAAATCCCACCTTTTAGTTTACTTATTACCATTATCCCCTATAAGTTTTACAAATCTCCAAAATCCCTCATTTTCGCGCATCAGATTAGTGTATCTCGCGCATCANATTANTGTATCTCGCGCATCAGATTAATGTATCANCGCTTATATTANTGTATCTCGCGCATCAGATTAATGTA

>rnd-1_family-304#LTR/Gypsy ( RepeatScout Family Size = 273 Final Multiple Alignment Size (possibly truncated) = 98 Localized to 2361 out of 4218 contigs )

TGTCTTGAATCGGTAGACCTAATGGTGGTGGCCCTTTCATGATGAGTCNATGAACTANCGTAATGATGAACCTTGAATCGGTAGACCTAATGGTGGTGGCCCTTTCATGTGGAATCGATGAACT

>rnd-1_family-536#DNA/Harbinger ( RepeatScout Family Size = 117 Final Multiple Alignment Size (possibly truncated) = 98 Localized to 2361 out of 4218 contigs )

ATATATATATATATATTTGTGTGGTTTTCTAATTATTTGTATTTTGAACAATTTATAAAATTGCATACATATTAAAACTACAACTTGCAATTTTTATGTGTAAATGTAGATGGTTTATTCAATTTTACCATTGTTTACCGTCTTTTCGATTTTAAAAGTAGATGGTTTATCTTTTTTAAATTGAAAATTGACATTTTGTGAGTGATCAATTTATTAAGATGACAATTATTTATCCTCAAATAATTCAAGTGAAAAAATAGCAAACATGACAACAAAATTGTTCTTCTCATAGCTAGTTAGAAGGCCATAAAAAAATAATATTGTCCGGCAATTATCTACCTTGACCCAATTACATAATAATTCAAGGGTATAATTGGAAAGAAGTTTTTTGTAGAGTTTTAATCCAAACACAAGATGAGGTGGGAAACAATGAACCAAACACTTGATAAAAATAATCCCTGCATTACTAATCCCTGCATTACTAATCCCTGCATTACTAATCCCTGCGTTACTAATCCCTGCATTA

>rnd-1_family-460#LTR/Copia ( RepeatScout Family Size = 144 Final Multiple Alignment Size (possibly truncated) = 98 Localized to 2361 out of 4218 contigs )

GGTGGATGCAATTACCTTCGGGTATCTAATAAATTTGGCAGTTCATGAAAANCTTGAAATGCGTCTAATGGACGTNGTCACAGCCTATTTATATGGCTCACTGGACCACGANATTTNTATGAAAATTCCTGAAGCATTCAAAGTGCCTGAAGCATATAAAAATTCTCGAGAAANTTGTTCAATAAAGCTTCAAAAATCCTTATACGGATTGAAACAATCAGGGCGNATGTGGTATAATCGCCTTAGCGAGTATTTGNTAAAGGAAGGGTATAAAAATGACCCNATTTGTCCTTGTATTTTTATNAAAAGGTCNGGATCTGAATTTGTTATAATAGTCGTNTATGTTGATGACTTGAATATCATTGGAACTCCTAAAGAGCTTTCAAAAGCNGTTGAGTGTTTGAAGAAAGAATTTGAAATGAAAGATCTTGGAAAGACAAAATTTTGTCTTGGCCTACAGATTGAACATTTGACAAATGGAATATTTATCCATCAATCAACATATACTGAAAAGGTTTTGAAGCGATTTTACATGGATAAANCACATCCATTGAGTACCCCGATGGTTGTGAGATCGCTTGACGTNAATAAAGATCCATTTCGACCTCAAGAAAAGGATGAAGAGCTTCTTGGTGATGAAACNCCATATCTTAGTGCAATTGGNGCACTAATGTATCTTGCTAATAATACTCGACCAGATATACGTTTTGCGGTAAGTTTATTGGCAAGATTCAGTTCNTCCCCAACACGAAGACATTGGAATGGNGTTAAACATATATTNAGATATCTTCGAGGGACCATTGATATGGGNTTATTTTATTCTAACGAATCCGAGTCAGAANTGATTGGTTATGCAGATGCAGGATATTTATCTGATCCGCATAAAGCTCGATCTCAAACGGGCTATTTATTTACATACGGAGGCACAACTATATCNTGGCGNTCAACGAAGCAAACATTAGTCGCCACTTCTTCAAATCATGCAGAGATAATAGCCATCCATGAAGCAAGTCGAGAATGTGTNTGGTTGAGATCAATGACNCATCATATTCAGGAAATATGTGGTTTTCTTTGGAAAAGGATATTCCAACCACANTGTACGAAGACAATGCTGCATGCATAGCTCAATTGAAGGGAGGATACATCAAAGGAGATAGAACAAAACATATTTCACCAAAATTCTTTTTCACACATGATCTTCAAAAGAATGGTGANATAGACGTTCAACAAATTCGTTCAAGTGATAATCTNGCAGATTTATTCACTAAGGCATTACCAACNTCAACNTTTGAGAAGNTGGTATACAAGATTGGAATGCGTCGTCTCCGAGATATCAAGTGATGTTTTCATCAGGGGGAGTAAAATACGCGTTGTACTCTTTTTTCCTTAACCAAGGTTTTGTCCCATTGGGTTTTCCTGGTAAGGTTTTTAACGAGGCAACAANCAANGCGTATTANAAGATATGTGTACTCTTTTTTCCTTCACTAGGATTTTTT

>rnd-1_family-382#LINE/RTE-BovB ( RepeatScout Family Size = 197 Final Multiple Alignment Size (possibly truncated) = 98 Localized to 2361 out of 4218 contigs )

TTCTACAGAGTGGTGGTTAGACCGACTTTGTTGTATGGGGCGGAGTGTTGGCCAGTCAAGAACTCNCACGTTCAGAAGATGAAAGTTGCGGAAATGAGGATGTTGAGATGGATGTGTGGGCATACTAGGAGAGATAAGATTAGGAATGAAGATATCCGGGACAAGGTGGGAGTGGCCTCCGTGGTGGACAAGATGAGGGAAGCGAGACTGAGATGGTTCGGACATGTGAAGAGGAGATGCGCGGACGCCCCAGTGAGGAGGTGCGAGAGGTTGGCTATAGTGGGTNCGAGGAGAGGTAGAGGTAGGCCGAAGAAGTATTGGGGAGAGGTGATTAGACAGGACATGGC

>rnd-1_family-434#LTR/Gypsy ( RepeatScout Family Size = 162 Final Multiple Alignment Size (possibly truncated) = 98 Localized to 2361 out of 4218 contigs )

ATTGTGTTATTATTGAAAGGNTATTCTCAATTAGACACTCATGAATGATGATGATAGAATGTGAAGGGTTTCTTCAATGTAATGTTATATCTTGAATTGGTAGGCTTAGGCATCCTTTTCTTGTATAAATGAANGTANCGTTGATTGATGAATCTTGAATTGGTAGGCTTAGGCATCCTCTTCTTGTATAATGAATGTATCTTGACTTAATGAATATTGAATCGGTAGGCCTATGGCATCCCTTTCATAAATGANTAATGAAT

>rnd-1_family-220#LTR/Gypsy ( RepeatScout Family Size = 425 Final Multiple Alignment Size (possibly truncated) = 98 Localized to 2361 out of 4218 contigs )

AGCCTAGTAAGAGTAATCTCATTCGAGGACGAATGTTCCCAAGGGGGAGATATTGTAACATCTCGCAANTTGAAANAACTA

>rnd-1_family-151#LTR/Gypsy ( RepeatScout Family Size = 582 Final Multiple Alignment Size (possibly truncated) = 98 Localized to 2361 out of 4218 contigs )

GCTTGTATGAGACCCTTCGGGGTCGAGTACGCCGTGTTACGTCTAGGGGGTACCCTCGGGTCGTGACAAACTTGGTATCAGAGCACAAGGTTTAGAATGGTTCTAGGATGTCTCATAAGCCACGTCTAGTAGAGTCTTGTTCATGAGTGTGAAGCGCGCCACATTTATGAACGAGAGGCTATAAGACGNTTAGGAAATTTCACTTCTTTCATTACTCTTAAGTCGTGCCNTAGAGTTTAACTCTATAAGGTCCCTCTCCTAATCCTTCTCTTGTGTCTNCAGGATATGANTACNCGAAGGGCTAACGCTAGAAGGAATGAG

>rnd-1_family-166#LTR/Gypsy ( RepeatScout Family Size = 534 Final Multiple Alignment Size (possibly truncated) = 98 Localized to 2361 out of 4218 contigs )

TTTTACAAGCTTTCTATATATTGCACGTGTTTTATTGCTTTATATTGAGTTNAGTTATTCATGAGTTGAGTAGAGCCAAGGTAAGTGTTCCTTTCAGATTCTTTTCAAGCCTATGTTGTGTTTAGCATTCCAACTCGCATACTCGTACATTCAATGTACTGATGCCAGTTGGCCTGCATCNTNTTATGATGCAGACGCAGGTAACCAGGATCAGCATCCAGCGCCTCGTTGATCCAGTTGAGCACTCAGAGTCAGTTGGTGAGCCTCCTTGCATTCCGGAGGACTCCTTTTATTTTGCTTTCAGTNTTTCATTATTAGGATGATCGGGGGTCTTGTCCCGACATCCATCTTTGTTTTAGAGGCTTCATAGACAGTCAGTCAGTTAGTTCATTAGTCTTTTCATTTCGTTTGTATACGTTTTAAGACTTGAGTTGCCATTTTGGCTAAGTTATTATTTCTT

>rnd-1_family-106#LTR/Gypsy ( RepeatScout Family Size = 708 Final Multiple Alignment Size (possibly truncated) = 98 Localized to 2361 out of 4218 contigs )

TATGAACGATTGCATGCCATGAATCCATNACTTGATTGTATGATTATGAGNATGTACATTATGGGTGTGATTGTGATTGTGGTTGTGTTGANTGGATCGGGTGTCACGTTCCGACACACTAACTTGGATCGGGTGTCACGTTCCGACGCATATATTGGATCGGGTGTCACGTTCCGACACATATAT

>rnd-1_family-507#Unknown ( RepeatScout Family Size = 127 Final Multiple Alignment Size (possibly truncated) = 98 Localized to 2361 out of 4218 contigs )

TTTTATTGAATAACATTTTTGGTTAATAATTTTAAAAATANTTATGAAATAATTATGACGTGNCATTATTATATNTGACGTGGATATGACGTGTCATTTATGTAAGNGAGAGTGAGCTACACACATGGTCGGAGGGGTTTAAAATATCGTGTTTTANTGAGTTTAAGGGTTCAGTTGACAAAGGGNTAAGTAGAAGGGTTCANAATACAAACNCATACAAGTACAAGGGTCCACCAGAC

>rnd-1_family-436#LTR/Gypsy ( RepeatScout Family Size = 161 Final Multiple Alignment Size (possibly truncated) = 98 Localized to 2361 out of 4218 contigs )

AAAGTCGTCAATACTTTTTAGGGTTCTTTTCTTCTTCTTGTTTTCTTAGAGATGAGTAGCTAAACGCCCTNGTTCTGGGGCTGTAGCTACGGATTGATTGTTGATTATTTGAGGTTTATGAATTAATTNTTGGTTGTCATTATAAGTTACTTTTATATTCTTGTTTTAGTATTTCGTGCTTGATCACCATGAGATAAATNTATTGTCTAATCTTGAACTCGGGAGAGGAGGGGTTAGATAGACCGGAGAATATAGAGAGCTCGATCCACCCATTAGATTGAGGTGATTTGTGTTTAGGAGTGACGCCCAGACGAACACCTTGCTTGGTTACGAAACAGGATGAAATATAAATGCTCGCCAGTTAGTCTATCTATCCCCGCTCAACGATGTAGTTAGATAGCTAACTGGGGTAGGCGACTAGAGGTCGGGAGACCNNGATCATACAATTAACCCTGTAAATCAGTAACTTGACAACTGAAATTAGTTCTAAGCCAANAATATGATACATGAGATTCANTGCATGCTGCAGCCCTGGAATTTTCCCAATCATTGTAAGTAATTTTATTATTTCGTTCNTGCATTCTTTTCTTTTGGCACTCTTAAATAAATAATTAGACTAGTATAATTTAGTAAAAATAGTTAATCGACAAGTCCTTTGGGTTCGATAATCCGGTTCTTTTAAGAGCCACTATATTACTTGCGCGACCACGTACACTTGCGTGTGCAATTGGGAGCAACAAGTTT

>rnd-1_family-149#LTR/Gypsy ( RepeatScout Family Size = 583 Final Multiple Alignment Size (possibly truncated) = 98 Localized to 2361 out of 4218 contigs )

ACTAACTTGGATCGGTTGCCACGTTCCGGCATAANTATGGGATCGGTTGCCACGTTCCGGCATAANCATTGGATCGGGTACCACGTTCCGGTATGCTAACNGTTTGGGTTTGGGTTCCATGAGAGGACCAATGACTTGACATAATTGTGTATCTTGAGAANTGTGAAATTGTNCGTTGTTCGTAAATGATGATGATATTGTATTTCCCCGGAGTTGTTATATGGTTATGAGGACTTATATNTGATTGTTCTTCTATGTCGAGTNAACTCGTTGCGTATCTCGTTGTTGAATGAATCGGGAAGGCCAAGGGTT

>rnd-1_family-433#SINE/tRNA ( RepeatScout Family Size = 163 Final Multiple Alignment Size (possibly truncated) = 98 Localized to 2361 out of 4218 contigs )

GTGGGAGGTGGCAGGTATCCCGTGGAATTAGTCGAGGTGCGCGCAAGCTGGCCCGGACACCACGGTTATNAAAAAA

>rnd-1_family-429#LTR/Gypsy ( RepeatScout Family Size = 165 Final Multiple Alignment Size (possibly truncated) = 98 Localized to 2361 out of 4218 contigs )

ATTTTAATGGTGAAGTTGCAGGCCAGNACTCTTGGAGCTTCTANGGCTTCTCCTTGGGAGTAANTTTCTGGAGAACAAGTGATGAAGGAGATATGTGAAATGAGCTTGAGTCCCTTAGAGGTCCCTTNTGGACGTTTTCACNTNANGAAATGTGTAGAAATAATCTGGTCCCTCACTTTAAGCAAGTAGGTGCATCACCTACTTTGACTTNTTGACCAGTCCAAAGNACTAAGTAGGTGCATCACCTACTTGCCCTTTTCNTTTAAAATGCAGTCCACATATTTCCTAAGTAGGTGCATCACCTACTTGCCCTTTTCACTTAAAATGCAGTCCACTAATTCTAAGTAGGTGCATCACCTACTTGCCCTTTTCACTTAAAATGCAGTCCACTAATTCTAAGTAGGTGCATCACCTACTTG

>rnd-1_family-553#LTR/Gypsy ( RepeatScout Family Size = 108 Final Multiple Alignment Size (possibly truncated) = 98 Localized to 2361 out of 4218 contigs )

ATAATCTTCCTGAACTACGTTCGACCTGATTCTCGCCTCGACGAGATACGTAGGCAGCCTTATTCTGGGGTTCGGTCCAACCAAAAAAAAATCTAGAAAGTCCTCATCACAAAAACTGGGGCAAAAATAA

>rnd-1_family-419#Unknown ( RepeatScout Family Size = 170 Final Multiple Alignment Size (possibly truncated) = 98 Localized to 2361 out of 4218 contigs )

GGGAAAAGGCTCAAATATGCCATCGAACTTTGAGAAAAGGCTCATTTATGTCATCCGTTAAAAGTTTGGCTCATCTATGCCATTTCCGTTTGAGAAAAGGCTCATCCATGCCATTATTTNTTAACGGCGGTTTTGCAAAACCATTTTTTACACGTGGCCAATTATAATTCGGCCACGTCATTATTTTTTTTATAATAAAAAA

>rnd-1_family-74#LTR/Gypsy ( RepeatScout Family Size = 864 Final Multiple Alignment Size (possibly truncated) = 98 Localized to 2361 out of 4218 contigs )

TTTTTAAGTTAGGGGCAGTTGGGTCTTTTCCTAATTANTTTANCCCNATACTACGTCGTTTTACCCNTGNCTAAGACCCCTATATAAGTATTTTAACCCCNAAATTACCCATTCAANTCATTCTCTCAAATTCCCAAAAGAAGANCAAGTTCCTCTCCAAAATATTTCTCTCTCTAGAAACTTGAAGAAGAAGATTCAANCTAGGGTTTCAAGTCAAGTCTCCATTCCTCCATTGAAGGTAAGCATTTGGCTTCGAGGTATGATAGTTTTCATCCATGGATTCCTTCCATCCATGGAGTTCCCAAATTCTCCTATTTTCAAAGNTAGAANTCCCCAATTGAGTTAGGGTTTTCTTCAATTGTCATGGGTTCTTTTNATTATTGATTTAAATGATTGAATTATGATCTCTTATGCATGAATTGATGAANTACTATGATTTTATGATGATTCCCCATGAACCCATGTAATCCCCATGTTTTCCAAGTTATGATCATATGATGTGGGTTTTGATTATGAAAGAAGAGTTTTATGAAATTAAGCATGNATTGATAGANTTACATGAATTTATGATGAAATCCATGTCTAACCCATGTNTTCTAGATGTTGATGATTGAAAGTGGGTTTTGAACCTCGAAAGGTAAATTATGGAATTATGCATGTTCTTATGAAATCTATGCAAATGTTTTAAGGATGCTTTGAGAGTGAAGTATCAATGATGATGTTGTTGTTGTGTTGTTGAAAGGATATTCTCATAAACACATAAAAGCATGATGTGAAAGGTTTTCTCACATAGTAAGGATTCTNAGGTTGAAAGGCTTTCTCACCTAATTGAATCAAAGATTAAGAGCTATCCTAATGAAACCTAGCTTGGATTGGTTATCGAANGATACCTTTCCATGGATAATGAGTCAAGTAAGAAAGAAACG

>rnd-1_family-143#LTR/Gypsy ( RepeatScout Family Size = 601 Final Multiple Alignment Size (possibly truncated) = 98 Localized to 2361 out of 4218 contigs )

AGTTTTCTCTAGTTTTCCTTAGCTTTGAAGAATTGAAGTTTTCACTTTTGAGAAATTGGAAGTGGGTCTTTGAGATTCTTCGANTTGGANCATTGTAAAGACGAAATCAATCTTACCCAGTTGATGGAAACTCAATTTGGTAACGATTTTTATCTTTTTATGATGTCTAGCTAAAACCCCAATTCTTGGGGTGTGATTATGTGATTATGGGCTGATTTAGCTTATGGGTATTGCTAATTGTTAGTTTAAATGCTGTTTAGAAGTGANTTCAATCATTAATTGTGGTTTAATTTAAGAATTGTAGTTGCAAATGCAGTTCTACCTTCGTGTTTTTGGCTTGCTCGAGAGAGAGGTTTTAAAACCAAGATTATTGATTGATGGTCTGTGGGTATTGGGTTGTCATGGGTTCAGCTCGAGAGAGTGAATCCTAAACCCNNTCCCACACATTCAGCTCGAGAGAGTGAATGGACTAAGGCGTGGGTTGTTCTTATTTTGCATGCTTGTTGATGTTCGAGAGAAATCGACTTGATTCGGGGTAAATTGTTCGAGAGAAA

>rnd-1_family-394#LTR/Gypsy ( RepeatScout Family Size = 189 Final Multiple Alignment Size (possibly truncated) = 98 Localized to 2361 out of 4218 contigs )

GCACGGGGNATNGTNATNAATGAGGGAGCAGCTGCTTCTAAAAAGGGNAAGAAGGNACCTCCGAAGGGAGGCAAGGGCAAGGGCAAGGCGCCCGTAGCTGAGATACCGGAGCACAACTCCGGCAGTGAGGGAGAGTCCNNTGATTCTCAGGCTGCATTCTCTGAGCCTGAGGATGACCAGCCNCTACAGGCCCGGAGAGCGGAGCTNCGCTCCAAGGCTNCGCACGATCCGTCTAGGATCCCGGCGCCTCAGACTCCTCCTCCTCCAGCTCCGGCACAGACNGTGGTCCCGGCGCCACCAGTACAGGGTCCTCCTCCCCGGTCGCTCAACAGACTAAAGGCCGAGGGNTTGAGGACCATCCTNGAGGAGAAGNGGTTGTCCACGGATGGCGTGGTGGACAGGTACCCNGANGTGTGGGACACCCTCGGGTNCCACGAGTTCGAGNTNTTCACCAGGCCCCGNGGCCCNTACATTCCTACNTGGGTCCGGGAGTTCTACGCNGCNTACGGNGACTTGGTGCCNAAGGGGAAGAAGAAGGCCAGCGCGTTCAGACCGGTNGANTCCGTCGTGGTCCGGGGNAAGGAAGTNAAGTGCGACAGCGATNATATCAA

>rnd-1_family-449#Unknown ( RepeatScout Family Size = 152 Final Multiple Alignment Size (possibly truncated) = 98 Localized to 2361 out of 4218 contigs )

GCTTGTACACGTGACAACCAGGTTTTGGGGTATTGTTTAGAATGAATAAGTTTTCCGCNTTTATCTTGTTCTTGCTTTATTTTTCCGCATTTCTTTCGTTTTCGTTGGGTTNAGGCTGACTTGTCTTGGTGGGATAAGACGAGTGCCATCACGTCCATTTTTGGGTCGTGACAG

>rnd-1_family-403#DNA ( RepeatScout Family Size = 183 Final Multiple Alignment Size (possibly truncated) = 98 Localized to 2361 out of 4218 contigs )

TCTCGCTCGCCTCTCTCCTCCCTCTCCCAATCTCGCTCGCCTCTCTCCTCCCTCTCCCAATCTCGCTCGCCATATATACAAATACATATGTATAATATACAATTATCTAACCGATATACATATACAATTCACCTCTCTCCCACTCTCTGCCCTCTCTCGCTCGCCTCTCTCCTCCCTCTCCCAATCTCGCTCGCCTCTCTCCTCCCTATAACATGTAGCTACGAATCGTAATTAGCAAACTATAGCTATGGAGCGTAATTAAGCTATTTTTGAGTGGCTATATGCGAAAGTTCCCCTATTATAAA

>rnd-1_family-512#Unknown ( RepeatScout Family Size = 125 Final Multiple Alignment Size (possibly truncated) = 98 Localized to 2361 out of 4218 contigs )

GGCAAATGACGTGGCATGCCAAGTCAAACAAGAAGCCAATAGGATCATGACATGTGTCAAAGATGACAAGCCCGCTCCATAAAGCCCATATGTCATGTCACTTAAATCTGATTGGCCGAAGGGAATCCTATTCCAATCACAACTCCTCTATTCTAAAACTATAAATAGGGGTCCTCATAATTCAGAAAGAGGACCGAGAATTCTAAACAAGAAGCTAGAGAAAGCTCGTGGATCAAANGCCGCAAATTTCTCTACAAGCTACAAGTTCAAGAATTCAAGATTCAAGTTCAAGAACGATCAAGATCAAGACCGTCGGATTCAAGATCAAGCTCGAAGCCCTTGAATTCAAGTAGAAGTCAAGATCAAGATAAAGTTCAAGTTCATCGGAGATTCAAGATCAAGCTCGAGAGCCCTTGAATTTATATTTGAAAAGGCGAATTCAGAGGAATCATAGAGATTGTAACACTCGCACTTTGAAATAATAAATACGATTGTTGCGATAATTTTCCGTTCTTGATTATTGTTTTCTCGACGCGAATTTTATTGTCTACA

>rnd-1_family-196#LTR/Gypsy ( RepeatScout Family Size = 482 Final Multiple Alignment Size (possibly truncated) = 98 Localized to 2361 out of 4218 contigs )

TGTAACACCTCGCTATTAGAAAGAGCTAAANTTAGAAAGAACTAAATTGGAAATAGCNAAATCTGAAATTTTGCAAGTTATGCTTAAGTTGTGAGTTTTGGGTCAACTTCAAACGACCATAACTTTCAGTACANGATGAGTTAGGT

>rnd-1_family-450#LTR/Gypsy ( RepeatScout Family Size = 151 Final Multiple Alignment Size (possibly truncated) = 98 Localized to 2361 out of 4218 contigs )

CATGTTTTAGTTGGAGAAGGTAGTTCCTCCTATGAACATGAGAAATGTTGAAGTTTCGTGCATAATGGGTTGTTAGATGTAGTTCCTACTTCCTTGTGTTGCATTGAGTATGTTTGAGNTGGAGTTGATGTTTCCTCCTTGATAAAGTTGCATAACGTGCTTTAATGAGATAGTTGCATGTTGTGTTTCTTTGGTGTTTGA

>rnd-1_family-108#LTR/Gypsy ( RepeatScout Family Size = 704 Final Multiple Alignment Size (possibly truncated) = 98 Localized to 2361 out of 4218 contigs )

TAGTGTTGGCTAGANATAGAATAGGGTCCGTGTCTAAAAAGTGTCCAGAAAATGTAAAAAGAATAGCCTAATGGTTGCTACGTGTGCAGGGACGAAANGNAAAATCTGCAGAAAATGGTCTGGTGCAGNGGTCTACGGACCCCATCGACGGTCCGTCGNTCGATCCACGGACCGTGGATGGCGTCCGTAGATCCCAGGTAAGTTTCTAAATTTTTCCAAGTGTNAGAGTGATCTACGGTCCCGATCTACGGACCGTAGGTCGACCCACGGACCGTAGACGGGGTCTCGTGGGTCCAAACCCTNAGGCTGCCTGACCCGACCCGGACCCCCCTATTTAAAAACCCCCATTCGTTTTAAACCATTCCCCTTCCCTCCATTACTCCCAAAACCGTTTCAACCATCCCATAACCCCCCTAAATCACTCCATAACTCCCCCATAATATCATCCCTTCCATAATTCCCCAACTTCCCTTCCATTTCAAATACAACTCATCCCATANTCCACAAAAAGGGTCCGGTGTTCGGTCAGTAACGAAGAGGCGACTCCTTGGTCTTGCTAAGCTTAGTGTCGTCACGCAATCACCCCACTCCTCGTTGCTTGTAAGGTAATAGACTTTCCCCTCACTTTGAATTTCGGTTCATNGATTGAATATAGAAAGTCGGGAGTCGGGTCTTGACCTTGGGTAATTGTTAGATGAAATTGCATGNTAAACTTGAAAGTTACAACGCCCTTGGGACGATAGATAGTGATTGTGTNGCCTTATTGTATGTGATCGTAAGTCTANATGTAAGTTTCGACTTAGGGTTCCACAGCTNGTCTNAATTGTTTGGTAAAATGATCAGTCGGAATCCTGAGAATGACGAACTAGCATAATTNAATGTTGTAAATGCATGATTAGACTGCGTTAATGTTGAAAACTAAGGCCAAAAATGTATGATACCT

>rnd-1_family-60#LTR/Gypsy ( RepeatScout Family Size = 997 Final Multiple Alignment Size (possibly truncated) = 98 Localized to 2361 out of 4218 contigs )

AAAAGAGGAGAAAAGAGGAGAGGATCAAAGCGTTCGTCGAGATTCTTGCGTNTTGTTGCGGATTTTCGCCATGGGTTTGATCCCTAAGAGGTATGTAAGCTTCCATAGTGTTGGGTTCGTTCACCCACACGCCAATCATGTTATTTTCAGCGAAATTTCGTTCTCGAAGTTGAAAGATTAGAGTTCTTGATGAGTTCTTGATAGGTGTTCTTGAAGTTCGTTCTAAATCTTGTTGTGTTGGGGTTTTGATGATTTCTTGAGATGAAAGTTAGTGNTTTGAGGGTTGTTTTGAGTAGATTAGAGTGTACTTATGTTGGGTAATCGAATCTAAGTGATTGGGGAAGAAACCGTTCGATTCTAGGCGAATTAGGGTGAGAAAACGAGCAAGGAAAATTCGTCGGATTTTCTGGGTTGGGGCCTGGCGCGNCGCGCCAGCCAGTGCGCCCCAAACNNNCCTCTGAAGTTTAGGGGCTGGCGCCCCGCGCCACTCAGTGCGCCAGGGTCGCCTGCCCCNNCCNGTTTTCCGTCGNTTGCCCCGTTTGAGTTCTTTTAAAGTGTACCTTCACTCCTTTTCGATTCTAACTACTCTAAACTACTTCTAAACACCTAGAAATCATTCATAACATGAATCATAACCTTGAATTCATAATTCAAATTCAAGGTAGAGTTAAGAGTTAAGTCTTGAGAGTTCTT

>rnd-1_family-191#LTR/Gypsy ( RepeatScout Family Size = 484 Final Multiple Alignment Size (possibly truncated) = 98 Localized to 2361 out of 4218 contigs )

AAGTCTTCCGCTGAGTAAGTAAGCCAGGCCAAGGGTTCGCTTGGGGCCAGCAATGGTTCTCGAGTGCCGGTCNCGCCCAGGGTGTAGGCTCGGGGCGTGACA

>rnd-1_family-354#LTR/Gypsy ( RepeatScout Family Size = 228 Final Multiple Alignment Size (possibly truncated) = 98 Localized to 2361 out of 4218 contigs )

AGGACCCTCCTGAATAATGGGACGTAGTATAGCAAGGACCCTCCTGGATAATGG

>rnd-1_family-299#Unknown ( RepeatScout Family Size = 284 Final Multiple Alignment Size (possibly truncated) = 98 Localized to 2361 out of 4218 contigs )

ATGTCTTTAGCTACGACTAGTTAGAACTAAATTTTATTTTTCTATTAATTCTCAAAACCGCTCCCTTGGGACACGACCCCAACCCTTGGTTGGGTTACTATATTATCGACGATCGTAGACACTCGTACCGTAGGTTAGTGTCGTTGGTCACGATAAGCATCA

>rnd-1_family-404#LTR/Gypsy ( RepeatScout Family Size = 183 Final Multiple Alignment Size (possibly truncated) = 98 Localized to 2361 out of 4218 contigs )

AAATCCAAATCATCCTAGTTCGAGAAATACGCCACTAACGGCCCTCGAATCATGGATAAATCTTAGGAGAGNAGAATCAAGGGAGGAACAGAATTGTACCCGCAATNTTNATCAATAAAATTATGTTTCTTCATATTTTATTTGTGATTGTAATTTATTTTCCGTCGCTTTAAAATTTGTTGCAAACAAATTGGCACGCCCAGTGGGACCAAATCTGCCCTTCATCTCTTCTCTCGTAAATCAAATCTGAAAATCTGAAGCCGCAAAGGTGGAAGAATGAGTACTTCAAGCCTCGTCTTTGAGTTTCATCGAGTCTACACTCCGACAAGCCTTGAAGCAGGGGGCATTTGTAGACATTGAAATTTTGTGGGACTCTACAAGACGTGTTCAATTCGAGTTGGGACTCTACAAGGTGTGTTCAATTCCAATTAGGATTCTTGGAGGCTACTCAACCCTAAACCCTAGTTCATGCCTATATAAAGGGTACTANATTCCCTTAAAAGGCATCTCGAANATTCCATAAA

>rnd-1_family-141#LTR/Gypsy ( RepeatScout Family Size = 607 Final Multiple Alignment Size (possibly truncated) = 98 Localized to 2361 out of 4218 contigs )

TTGTAAAGGGTTGTGACCCTTATTGATATTCGATTGTATTAGATGGTTATTGAGACAATGTCTAGACTTCCGTTTGNTTTTATGAAAAGACTTCGATTGTAAAAAGTTTTAAATTTCCGCATCATTTCTATTACTTATGTTTATGATATGCTAAGGGCTTGTATGAGACCCCTTCGGGGTCGAGTACGCCGTGTTACGTCTAGGGGGTACCCCTGGGTCGTGACA

>rnd-1_family-435#LTR/Copia ( RepeatScout Family Size = 162 Final Multiple Alignment Size (possibly truncated) = 98 Localized to 2361 out of 4218 contigs )

TGATAAGTGCTAATGCTCCTAGGTGTTTTGATGATCTCCTCACAAGTGCAGGGACCCGGTCCTCTGCAGAGTACGCTCGTCCAGNGTCAATATGNTGTACAGCTGTAAAGCTGTCCGCGTCAGGAGGTTGGTGAAGCCGCAGCGTACTNCACCAATCAGAAGCGACGAGGTTGTTTGTNCGNTGGNTAATAACTCTTCATGGTTGATATATTCAACATNACAAACAACAAATNAANTCATTCATTCAAAGAGAGACATTTCAAGCTTCAAAAACAGCAAGGGACCTGATAGANTGCTTCAATTGCTTCCTGTACCAGCTGTTACTTTGTTTGCTTGTAATCGTGCTTATATTGTAGGATTCGTTTCGCTTGTGATAGAAACGTTTCAANCTTGTGTGAATCATTGTAAGAACTAGGTTGAGGGTAACTTAGTGTCTTACTCTAAAGTCTATATTAATCGGTTAGGGTTAGTATAGTGGGCAGTGTTGTATCTGCTTNGGCTCGNCTAAGTAATAGGAGGTATTGCTTAGTGTGGAGATTAGNAGGCTAATCTCGTTGTTGTAAACTGGCTTTTACTTTTGCTTGAGAAGATTAGTGAAAGCAGTTTGAAAAGTCCTGCGAGACAGGTCGTGGTTTTACTCCCTTGAGCAAGGAGGTTTCCACGTAAAGTTGCTTGTCAATCTTTACTTTCAGCATTTGTTCTTTACTGTTTGTTACTGTAACTGTGTCGAGGACCTGGTCCCATCGACATAGGTGGACGCATACATTCCAACAA

>rnd-1_family-477#Unknown ( RepeatScout Family Size = 137 Final Multiple Alignment Size (possibly truncated) = 98 Localized to 2361 out of 4218 contigs )

AATAGGAAGATATCATGAAATTAATTATTTTTTCAATATTATTTTCAAGCGTATCGGGTCAAAATATGAATCATATGTGGTTTTATAGAAAAATAGAATTTTTAAAAATATCATTTTTATTATTTATTTGAGAAGGGTTTAAATTTGGACCAATCACATTAAGCAACATAATAAATAAATATTTAATAATTTTACTTTAAGGGAGCTGTTAAAAATAAGGGCAAATNTGAGCCAAAGGGTTGACGTCGAGGGTATTTTTGAGCCAAAAGGTNGACGTNAGGGGTATTTTNGGGCCGATAGGTGGATGAAGGGTANTTTTGTACCANTTCGAATAGTTNAAGGGCATTTTAGGCCCTTTTCCGTA

>rnd-1_family-111#LTR/Gypsy ( RepeatScout Family Size = 696 Final Multiple Alignment Size (possibly truncated) = 98 Localized to 2361 out of 4218 contigs )

AGCCCAAGCCATGACGGCCCAAGCCAATAGAGANGTTGNGNCTCGGGTGAACCCTAATGTGAGTACCGCGGCTTCNAGGTTGAGAGACTTCGCGAGGATGAACCCTCCNGNGTTTCNTGGCTCCAAAGTGGAAGAGGATCCTCAAGAGTTTNTGGATGAGGTTTATAAGGTANTTGATGCTATGGGGGTGACTTCGGTAGAGAAAGCGGAGCTAGCCGCTTACCAATTGAAGGATGTNGCCCAAGTNTGGTACGCTCAATGGAAGGACGATAGGCCGGTAGGAGCGGGTCCCATAGANTGGGAAGTGTTTAAGNAAGCATTCCTTGATAGGTTCTTTCCCCGNGAGATGAGGGAAGCTAAGGTGGAAGAGTTCATCAACCTTCGNCAAGGNAGTATGAGTGTNNAGGAGTATTCTTTGAAGTTCACCCAATTGTCCAAGTATGCTCCATCTNTGGTGGCGGATCCNAGGGATGAGATGAGTAGGTTTGTGACGGGTGTGTCCGACTTGGTTGAGGAAGAGTGTCGTACGGCAATGCTCCATGATGACATGGATATCTCNAGNCTTATGGTGTATGCTCAACAAATTGAGGAGTCGAAACTTAAGAAGAAGAATAGGGAGGTGAAGAGGGCTAGGACCGATGAGCAAGGTCAACCTAGGTTCAAGAAGAGGGCTCCNAACCAAGATTCTTCAAGCGCTCCTAAGGCTAACCAAGAGAAAGGTGGTGGATCTCAATTTTCTAAACCTACTTGCGCCACTTGTGGAAAGAGGCACCATGGGAAGTGCCTAGCCGGTACTAGTGGGTGCTATGGTTGTGGNAAGAGTGATCACCAAGTGAGAGATTGTCCTACTCTTACGGCTAAAGGAAGGGAGGCCAAGCAAGCTCCTCTTAGTGGCCCGGATCCTAATGCTCCAAAGAAGAATCGTTTCTATGCGCTTCGAGCTAACGAGGACAAAGGAGCTAATCCGGATGAAGGCGCCGGTAAGTNATAGTTCCTTATAGTCGTGAA

>rnd-1_family-445#LTR/Gypsy ( RepeatScout Family Size = 156 Final Multiple Alignment Size (possibly truncated) = 98 Localized to 2361 out of 4218 contigs )

TTCGGNGGGTTGGCTCATGAGGATCCCCATGAGCATATTAGAAACTTCGTGGATGTNTGCGGACCGTTCTCGTTCAAGAACATNTCNCAAGAGTCGGTCCGGTTGAGGTTGTTCCCGTTCTCTTTGATGGGNGAAGCGTGCAAGTGGTTGGCCGAGTTGCCAAGNGANTCNATCA

>rnd-1_family-194#LTR/Gypsy ( RepeatScout Family Size = 483 Final Multiple Alignment Size (possibly truncated) = 98 Localized to 2361 out of 4218 contigs )

TTTAAGTTATTGCATGAGTTATCTTTTGAGACTATTTCTTGTGTTTAAGTCTTCCGCTGAGTAAGTAAGCCAGGCCAAGGGTTCGCTTGGGGCCAGCAATGGTTCTCGAGTGCCAGTCCCGCCCAGGGTGTAGGCTCGGGGCGTGACA

>rnd-1_family-264#LTR/Gypsy ( RepeatScout Family Size = 336 Final Multiple Alignment Size (possibly truncated) = 98 Localized to 2361 out of 4218 contigs )

CCTGGGCAGTGACCTACGGANGGGACCCACGCCCCGTAGGTCCGTCCACGCCCCGTGGGTGAGGGGTCGTGGGTCAAGGCCCCGAAAACAGGCTCCAGAACCCCACCCACGGNTNACCAGCACGGNCCGTGGGTCCACCCACGGTCCGTGGTCGTGGGGTCGTGGGTGGGCGCCTGTAANCTGTTTCGGCGGTTAGTTAGGGGTANTTTGGGGATTTCTCCAATTAATTAGTTATTAAGTGGCGTCGTTTTATACTAATTTAGACCACCTATATAAGGATTTAAGACCCCTAAATTAANTCATCTTCNTCATTATTCCAAAACCCCAAATCAAAACCAACTTCTCCCCAAATATTCTCTCTCTAGAACTCCANTGAAGAAGAAGAA

>rnd-1_family-566#LTR/Gypsy ( RepeatScout Family Size = 104 Final Multiple Alignment Size (possibly truncated) = 98 Localized to 2361 out of 4218 contigs )

AGATCGCCTTAGTGAAACTTCTAGCATCGAAATGTGCATCCAAGGNATGNATTGGGGTCTTCGCTACATCTTGCAAGGAATAAAGCCNAAAACNTTCGAAGAGCTGGCNACTCGCGCNCATGACATGGAGTTGAGCATGACTTCGAGAGAAGATCAACGATCTCTGTCCGTGAACCTCGCGAAGATGAAGATATAGAAGAACTCCAAAGTGGGGGCAAGTCTGCGTCCGAAGATGACTTTGAAGAGTCAATGTATATCTAAACGCTCCTCGACGCACGAGCCTAAACTGCANGTCGCAAAGCTCCTCAAATTCGAGCTAAATCTTCAAGTCGAAATGCTCCTCGACGCACGAGCCTAAACTGTATGTCACAAAGCTCTTCAAATTCGAGCTAAATCTTCAAGTTGAAATGCTCCTCGAAACACGAGCCTAAACTGCATGTCAC

>rnd-1_family-181#LTR/Gypsy ( RepeatScout Family Size = 500 Final Multiple Alignment Size (possibly truncated) = 98 Localized to 2361 out of 4218 contigs )

GAGAAGAGAAGAGGAAAAGTCAAGATTCGTCGAGTTCTTGAGGATTTGCTTGTGGATTTCGCCGAGGGTTGATCCCTACGAGGTATGTGAGATCNCATAGCGTTGGGTTCGTTCACCCACGCGCCAANCATGTTTANTTCAGCGTGAATTCGTCCTAAAAAGATTGAAAGTTTGATGTTCTTGATNNGTGTTCTTGAATTGCTTTCGTTCTTGAATTTGGGTTGAGATTGAGGAGTTTCTTGAGATTANAGTGTCGNTTCCTTGAGTTGTTTGAGTTAGATTCTTGTGTACATGTNTTGGGTATCTGAATCTAAAGAGAATTTGAGAAAAAGAATCGAGTTTAGGCGANTTAGGGTCGGAAAACGAGAAGAAAAGTTCGTCGGATTCGCCTGGGGAGGGGCTGGCGCGNCGCGCCCCCGTAGCGCCAGGAAGGCTGGGGCGGCGCGCCAGCA

>rnd-1_family-17#LTR/Gypsy ( RepeatScout Family Size = 1421 Final Multiple Alignment Size (possibly truncated) = 98 Localized to 2361 out of 4218 contigs )

TACTTAGTGTGGGTGGGGGTATGGGACTTCATCCATGCATTGCACAAGTAGGCTTTGAGAGTGGTTATGGTGTGTTCCTCTTATGTCATGATGACTCATGTAATGAATGTGCTTGTGACTAGATGGTAATTGCATTTGATATGAAGGCTACTTGTAATGATGTATGCTTAGCTTGGATTGTTGCTATGAGTTGCTTTCCTTGATATGNTTGACTAAAGGTGAATGTTCCCTTGTCTATGAGAANTAAGCTAAGGATGAGATCAAAAGATGGTAAATATGAATGANGGCCTAAAGTGGCTTGCTTAGTATGGGTGGGATTATGGGGTCTTATCCATGCATTGCACAAGCATGNCTTGAGGTTACTTATGAAGTGNTTCTCTTATGTTATGNTGANCTAGATTGCATTGACTATGCCTATGNCTATGNTGNTATCTCTTATGCTTACTAGTTATGATTATTGTCTATTATGTTATAGCCTATGTCATATTATGATGTCTNTTATGCTTATGACTTATGTTTTTNCACTATCTTCANGATGATGCTTNAACTTGGTAAATTGCATGCNTTCAACTAAAATGTCCCTTTTAGCATGTTTTCAAGGTTTTTATGCATGGCTATCATACTTAGTGCATTTTTGTACTAACCCATATTTTCCTACATTTTCCCAAGTGTAGGGTCCGGTTCTCGAGGTGGTCNTCNTTTGGTTAAAGCTTGGATTCGACTATTCTCCTNGCTTGTGGTGAGTCCTCATGATTCGAGGACGGATGTTTCACTTTGTAGTTTCATTTCTTGTATTT

>rnd-1_family-87#LTR/Gypsy ( RepeatScout Family Size = 823 Final Multiple Alignment Size (possibly truncated) = 98 Localized to 2361 out of 4218 contigs )

ATGTGTTTCCATGATTTCCTTATGTTATGCATGTTCTTGAGAAATTCATGTTTAGNAAGAAAATGAGTTTTCATGATTTATTTTCCTCATGTTGTTGTGCATTTAGTATGAGTTGCCTATAGACTTCATGAGATGAATTGATGAACATGCTTTAGATATGCTTTTTGAGAGTGAATTGCATATNGGCTCCATGANATTGTGTTGAGCATGCTTTTAGCTTGGAGAAGGTAGTTCCTCCTATGAACATGAGAAATGTTGAAGTTTCATGCATANTGGGTTGTTAGATGTAGTTCCTACTTCCTTGTGNTGCATTGAGTATGTTTAGNTTGGAGTTGATGTTTCCTCCTTNGTTNTGTGCATTGTTTTGATGTTTCATGCATGATGGGCTGCTAGNCTTGAGTTTCACCCCTCTTATGATGTTTTGAGAACGTCCTAGCTTGGAGTCGATAGTTCCTCCTCGGTTGTATGCATTGCATTGTTAGGTTGGGTTGTTAGTTCCTCTCCTACTCTTTTAGAACCAGTTTAAATCTCATTCGAGGACGAATGGTCCCAAGGGGGAGATATTGTAACGCCCCGTAT

>rnd-1_family-25#LTR/Gypsy ( RepeatScout Family Size = 1288 Final Multiple Alignment Size (possibly truncated) = 98 Localized to 2361 out of 4218 contigs )

AAGAGGAGAAAAGAGGAGAGGATCAAGGCGTTCGTCGAGATTCTTGAGATTTGTTGCGGATTTTCGCCAAGGGTTTGATCCCTACGAGGTATGTGAGCTTCCATAGCGTTGGGTTCGTTCACCCACGCGCCAANCATGTTTATTTCAGCGTAAATTCGTTCTAGAAAGTTGAAAGTTTGATGTTCTTGANNTGTGTTCTTGAAATTGGCTTTCGTTCTTGAGTTGGGNTGAGATTGANGAGTTCTTGAGGAAATCGTGTCGATTTTAGAGTTGTGTTTGAGTTAGATTCTTGTGTACATATGCTGGGTATCTGAATCTAAAGAGAAATTGAGAAAAAGAATCGAGTTTAGGCGAATTGGGGTTAGAAAACGAAGAAGGAAAAAGTCGGGCGGATCTGGGCACCGGGTCCGCGTCGCGGACCTGCTCCCCCAGTGNACAAANTTTCTCTCCGCGTCGCGGAGAGGNCGCGGACCGCTCTGCCTCAAAATTANTTTCGCGACCAAATATTTAAATGCATCTCCGCGTCGCGGACTTGCTTCCAGACAGTGATTTCTTGATCNTTTCTCATGTTTAACTATCTAAAAACACTCCTAAACATCACGAGATCNTTCCTATCACGAATCACAACCTTGAATCCATAATTCAAATTCAAGGTAGAGTTAAGAGTCAAGTCTTGAGAGTTCTTTCGAACGTTTTGAGAAAGTCCTTTTGAGGAGTCTTCTACAACTTCTAATAACTTGTTTCAAGACTCGAGCAAGTGAGTATGAGAGTGAGGAGAATGTATTCATGAGTCTACTTTATCATCACGAGATCCTTCATATCATGAACCATAACTCTTGAATTCATAATTCACATTCAAGAGAGAGTTAAGAGTAGAGTTCAAGAAAGTCTTTGAGTTCAATTGTGAATCCTTTGAGATCAACTATCGATTTGAGCTAAGTTTTGAGGAAGTAAGTATGAGAATGAGAAGAGTCGTATACATGAGTTCCATATTGTTATGTAGACCCTCGAGTCGAGTCGTTCATGCCCATAAATTCCGCATGAACTCCATAAGTTGAGTATCTTTGAGAGGAGTAGTATCTTCAAGTTCTAAGTCTT

>rnd-1_family-537#SINE ( RepeatScout Family Size = 117 Final Multiple Alignment Size (possibly truncated) = 98 Localized to 2361 out of 4218 contigs )

CAGTGGCGGAGCCAGGATTTTCAATAAGGGGGTTCAAAATCTGAAGAAGTAAACACACGAAGTAGCCGAAGGGGGTTCGACATCTACTATATATACATAAAAAATTATTTTAACCATGTATAAATAGTATAATTTTCCGCCGAAGGGGGTTCGGATGAACCCCTAANNATANGGTGGCTCCGCCCCTG

>rnd-1_family-385#Retroposon ( RepeatScout Family Size = 196 Final Multiple Alignment Size (possibly truncated) = 98 Localized to 2361 out of 4218 contigs )

AAAACAGAATTACTGATTNCAATAAACTTTGCAGAAACACGAAGCAACCAAAGTTTAATGAACCAGTAAGAATAGATGAACATAAATTAATTGACAAAACTAAAATCTGTAAAAACATACCAGAATCTGNAAAAACAGAAAAGAAAAAAATCGAGCCCACTGAATGCACAGTGTCCCCTTAAGGAAATTATTCCCCTCTAGTACCCGAGGTTTAATGGAATATATCCTCCCAGGATAGAACGATCTTANTCACCAGTGTATTGATACCCAAAACNATGGTGTCAGCGAGCCACTCAACGGCAGTAAAGTACACGAATATTTAATTGTGCAGAAGAAGAAGAAGAAGTCAGAAAATTTCGTAAGGAATAAGTCTGAGGAATCACTNTATTTATAGNCAAGAGGAACTGGTTCCGAAAGGTTGCAACCTTTCAGAANCCACACGACCATTCATGAAAGTTTGCAACCTTTCAAACGGTCATGGCTGTTTCTGAAANGTTGCAACCTTTCAGAACAGTCACTTCCAACGGCGGGAAATTCAAATAAAACGGGAAAGAATTTAAATAAAACGGGTCGCGCG

>rnd-1_family-334#Unknown ( RepeatScout Family Size = 248 Final Multiple Alignment Size (possibly truncated) = 98 Localized to 2361 out of 4218 contigs )

TCATTTATGTCATCCGTTAAAAGTTTGGCTCATCTATGTCATTTCCGTTTGAGAAAAGGCTCATTCATGCCATTATTTGTTAACTGAAATGACATAGATGAGCCAAACTTTTAACGGATGACATANATGAGCCTTTTCTCAAAGTTCGATGACATATTTGAGCCTTTTCCCTTTTAAAAAATAATTTAATTAATGACGTGGCCGAATCATAATTGACCACGTGTAAAAAATGGTTTTGCAAAATCGAAACTATTTTCAGTTAACAAATAATGGCATGGATGAGCCTTTTCTCAAACGGAAATGACATAGATGAGCCAAACTTTTAACGGATGACATAAAT

>rnd-1_family-359#LTR/Gypsy ( RepeatScout Family Size = 226 Final Multiple Alignment Size (possibly truncated) = 98 Localized to 2361 out of 4218 contigs )

CCGGTACAGGGTCCTCCTCCCCGGTCGCTGAACAGACTAAAGGCCGAGGGGTTGAGGACCATCCTNGAGGAGAAGNGGTTGTCCACGGANGGCGTGGTGGACAGGTACCCNGANGTGTGGGACACCCTCCGGTNCCACGAGTTCGAGNNGTTCACCAGGCCCCGNGGCCCNTACATTCCTACNTGGGTCCGGGAGTTCTACGCNGCNTACGGNGACTTGGTNCCNAAGGGGAAGAAGAAGGCCAGCGCGTTCAGACCGGTNGAGTCCGTCGTGGTCCGGGGNAAGGAAGTNGAGTGCGACAGCGATNATATCAACGCTGTATTTGANAGAGCTACACGGTTTGAGCATGACTACGAGGGCNTGACCACGACGCAGACCCTGGATGACCTGAAGGGTTGGCTGGCTCCNCTNATTTCTGACGCCACCCCGAGGTGGATCGAGGCGGGAGCNCCNATCGAGAAGAAGGACCTGAACGTNGCCGCNNGGTACTGGTTCGGNTTCATCAGCAGCACCATCATGCCGTCCCAGAACGAGTCCATCCTCCGCCATCCNAAGGCGGCCTGCCTTGGNTCNATCATNGCCGGGAGGCGGNTCGACTTGGGGCTGATCATTGAGCAGGAGATGGCCATGAGGGCCAAGCAGNGNCAGACNTCCCTNCCATTCCCGGTGTTGATCACNGAGTTGTGCCGGCGNGCCGGAGTNCCTCGNGACGAGAAGAGGGATGTNGAGGTNACTCCCACATCCTCCACCGACATCCGGCGTATCGAGGCCGAGTACACGCGGGANGAGGCTGACAGGAGGAGAGCAGCTCCGGCGGATACNTCCCCGGAGGTCGACGTTGACTCGNTACCTGCAGAGGCANCTTCGCCTACTCCGGCCTCCGGGCCTTCAGGTACATCTGCTTCCGCTCCTTC

>rnd-1_family-261#LTR/Gypsy ( RepeatScout Family Size = 343 Final Multiple Alignment Size (possibly truncated) = 98 Localized to 2361 out of 4218 contigs )

TCATGCATCGCGGGAAAAAGATTCATGCATCGCGAGAGAAAGATTCATGCATCGCGAGAAAAGATTCATGCATCGCGAGAGAAGATTCATGCATCGCGAGAAAAGATTCATGCATCGCGAGAGAAGATTCATGCATCGCGGAAAGAGATTCATGCATCGCGAGAGAAGATTCATGCATCGCGAGAGAAGATTCATGCATCGCGAGAAAAGATTCATGCATCGCGGGAAAAGATTCATGCATCGCGAGAAAAGATTCATGCATCGCGAGAAAGGAATTCATGCATCGCGAGAGAAGGATTCATGCATCGCGAGAGAAAGANTCATGCATCGCAAGAGAAAGATTCGCGCATCACAAGAAGATCGACATCGACTGCATCATTTTCTTTGCAANAACGACATCAAGAGAACTATCAACATATTACATCAGCCTATTTTATTACTTCGACATCAAAGGAAGAGTACATTGAAGATTATATTGCAAATATGAGACACAAGCTTTATTTGCTTTACGTCAAACCCGATGGAGTTGACGTCAAATGTTCAAGGAGAGCAATTAAGTGTCAACACGGTAAAAGACGCTATCTCTCCATTTGAATTGCATTTTTATGCTAATGAGTTTTATCTCAGTCTTTGTCGAGAGCATCCGAGAGGATGAATTCTCAAAACAAACCACAGGTGCGGACGACATCAAAAAGGATGCAGCACAACGTGGAACTTTTTCTTAGCAGCGAACTGGGACATAGTCCGAAGAGGAGTGCCAAACTCTTAGGACGCGAGCNGAGGAGCGACTTCCACCACAATCAAACCACACCCCTATCAGAAGGCATGTGGGTTTTCTTTGGGTTTATCGGTTTCAGTTTCGCATCCGGAAAATTTTGGTCTCTACCGGAAGCAACTTTCCAATCCGAGTGACAATGCGCCAAGAGCTTTGGAAATTATGTCCGTGTAAGTTCTTAATTATGGGTGTGAAGCGCGCCACATTCATGGCTAAGAGGTTGCAAGCCTCTTTTTCATACTCGACTTACTTTGTCGCTCTTCCAGAACCGAAGGTTATCTTGCATAATAGATTTCCTTTTCAACCGATCGAGTCGAACTACAAGCAGCCTGATTCCCTAAGTTGAGGGATATGTAGGCGGGATCAATGTTGAAAACTCGGCTGTATTCCAACATTCGCTCTTAAATCTTATTCCCGAGCATTCCGGTCCCTTCATAATTCGATATCGGGATGGCTTTTGAATTCTTTCAAAATCGTGCGTCAAATCAAGCGTATCGAACTACAAGTGGCCTGAATTCTCATATAGCCTGAGATATGTAGGAAACCCANTTTCGGGGTTCGGCCATAATTCCTAAAGTCCGTACCAAATCCCTTTTCCGAAAGATGAAGATGTGGTCGGTCAAAATTGGATTCGTCAATTTCATTTNCCTCGAATTTCTTTCATCAATCCAAGTCAAACGAGGGACAGTTGTTGACACCCAATT

>rnd-1_family-133#LTR/Gypsy ( RepeatScout Family Size = 621 Final Multiple Alignment Size (possibly truncated) = 98 Localized to 2361 out of 4218 contigs )

TATGACTTCTTAATGAGTTTTACTTAGTGTGAGTGGGGGTATGGGACTTCACTTATACATTGCACAAGTAGACTTTGAAAGGGGTTATGGTGTGGTTTCTTATGCTATGANGANATATGAATGTATGCATGTATGTTGAATGGATTGTTGCTATGAGTGACTTTCCTTATGTTCAAGTTAATGAACATGAATGTTTCCTTGTCTATGANTATTGGCTATGATGAGGTCTAAGGATGATATGTATGACTATGGTGGCCTNAAAGTGGTACTTAGTATGGNTGGTATTATGGGATGCTANCCATGCATTGCACAAGTATANCTTAGGGTTGCTTANGTGATGGTTCTACTATGTTATGANGATNTATGTGATGATGCTATGGCTTGTGTTTTACATTGAAATGCATGTTGGT

>rnd-1_family-171#LTR/Gypsy ( RepeatScout Family Size = 523 Final Multiple Alignment Size (possibly truncated) = 98 Localized to 2361 out of 4218 contigs )

TGTAACATCCGGCAATTTGAAATAACTANGAAGAGGCTTAGAATTGGAAATAGTCATTTTTGGAAAGAATTCGAAAATCTGGAAATTTGGCTAAGTATGGGAAAAAGGTGAGTTTTTGGCCAACTTCGAGCGGTCATAACTCCTAGCTCAGGATGAGTTAGGNGTAGTTCCAGTTATGGTTGCGAAGCTCGTGGAATGATCTTTCCAACGCCGCCGAGTTTGCTCGATTCCGAGTTCGTATGAGCGAGTTATGCCCTTTGGAAGTT

>rnd-1_family-338#LTR/Gypsy ( RepeatScout Family Size = 245 Final Multiple Alignment Size (possibly truncated) = 98 Localized to 2361 out of 4218 contigs )

AAACTAGTAGGCTAAACTAGAGCTTGACGTGAGGGTTAGAGTCGTAAAATGACTTCCCGTACTTAGAATGAAGGGTTTAGGGTTAGAACGTCAAGGTACGACCCCCCAAGGACCAACCAAGGGTCCTTGAGGAGGACCCT

>rnd-1_family-1#LTR/Gypsy ( RepeatScout Family Size = 1733 Final Multiple Alignment Size (possibly truncated) = 98 Localized to 2361 out of 4218 contigs )

TCTTATTGTTCTATTTCTTATGTTATGANAGCTAAGTGGCTTGTATGGGGCCCTTCGGGGTTCTATACGCCATGTTACATCTAGGGGGTACCCCTGGGTCGTGACAAACTTGGTAATCAGAGCACAAGGTTTAGAATGGTCCTAGGATGTCTCATAAGCCACGTCTAGTAGAGTCTTGTTCATGAGTGTGAAGCGCGCCACATTTATGAATGAGAGGCTACAAGATGTTTAGGAAACTCCACTTCTTTCATTACTCTTAAGTCGTGCGATAGAGTTTAACTCTATAAGGTCTCTCTCCTAATCCTTATCCGATGTTCTTCAGGATATGGCTACTCGAAGGGCTTACGCTAGAAGGAATGCGAGGGAGAATGTGGANCAAGAAGCTCCTCCCCAAGCTCCTCAAGCTCCGGTCGATCCTTTGGCCGAGCAAGTGACTAATGCGGAGTTTAGGGCNGCTTTCCAAGTGTTGGCTCAAGCCGTGACGGCCCAAGCCAATAGGGAGGTTGTGGTCCCCGTGAACCCNAATGTGGGTACGACGGCTTCNAGAGTGAGGGACTTCACNAGGATGAACCCTCCGGAGTTTCATGGTTCCAAAGTTGAGGAAGATCCTCAAGAGTTTATCGATGAGGTNTATAAGGTNTTGATGATCATGGGAGTGACTCCGGTGGAAAAGGCGGAGTTGGCCGCTTATCAACTNAAGGGTGTTGCTCAAGTNTGGTACGACCAATGGAAGGANGAGAGGCCGGTAGANGCGGGTCCCGTNGATTGGGAAANGTTCAAGNNCGCTTTTCTTGATAGGTTCTTTCCCCTTGAGATGAGGGAGGCNAAGGTGCNNGAGTTCATCAACCTTCGTCAAGGAAGTATGAGTGTGAAGGAATATGCTCTGAAGTTCACNCAATTGTCCAAGTATGCTCCAACTATGGTNGCGGATTCNAGGGCTAGGATGAGTAAGTTTGTTTCGGGTGTGTCCGANNTGGTGGTNAAAGAATGTCGTACCGCNATGCTCATCGATGACATGGACATCTCTCGTCTCATGGTNCATGCCCAACAAATTGAGGAGGAGAAACTTAAGGAAAAGTCTAGGGAGGCGAAGAGGGCNAGGACCGGTGATGGNAACTTCTCNNATGCNAGGTCCGATGGACAAGGTCGNCCNAGGTTCCGGCAAAGGTTTTCCGGCCAAGGTTCCTCCAATGCTCCTCCAAGGTTCAACAAAGATAGGGTGTCTAACCCTAAGCCTCAAGGAGGNAATGGTGGTGGNTCTTCGTTGNCTAGGCCTACTTGTGCTAAGTGTGGNAGGAAGCACGANGGTAAGTGCCTAGCCGGCACGGATGGTTGCTTTGGTTGTGGNAAGAGTGGTCACAAGATGAGGGATTGCCCGATGCTTACGGCTAAAGGAAGAGAGGGCAAGCAAGCTCCTCCTAGTGGTTCGGGTTCCGATGCTCCNAAGCAAAACCGNTTCTATGCTCTTCAGACTCGAGGTGANCAAGAGAGTTCCCCGGATGTGGTTACCGGTATGTTGAAAGTTTTCCAACTTGATGTNTATGCTTTGCTNGATCCCGGTGCTACTTTGTCTTTTGTGACGCCNTNTGTGGCTATGAGGTTTGATGTNCTCCCCGATGTGTTGTTAGAACCTTTTTCTGTCTCTACTCCTGTTGGTGATTCTGTNGTGGCTAAGAGGGTCTATAGAAGNTGTCCCGTTTCCTTGTCCCATAGAGTTACTCTTGTTGATTTGGTAGAGCTTGATATGTTAGACTTTGATGTTATTCTTGGTATGGATTGGTTGCATTCNTGTTATGCTTCTATTGATTGTAGAACTCGNGTAGTCAAGTTTCAGTTTCCGAATGAGCCTATCCTAGAGTGGAAGGGGGGAAATTCTATGCCTAGGGGTCGGTTCGTTTCTTGTCTTAAAGCTAGAAAGATGATCTCTAAGGGTTGCATTTACCATCTTGTTAGGGTGAGGGATGTNGATTCCGAAACCCCTNCTCTTGAGTCGGTCCCCGTNGTNAATGAGTTTCCGGAAGTNTTCCCGATGATTTACCCGGTATTCCTCCCGAANGGGAAATAGACTTCGGTATTGACCTTCTCCCGGATACGCAACCTATCTCTATTCCTCCTTATNGAATGGCTCCGGCGGAACTNAAGGAGTTGAAAGAGCAATTGAAGGATTTGTTGGATAAGGGTTTCATCCGACCGAGTATCTCTCCATGGGGTGCTCCGGTNTTGTTTGTTAGAAAGAAAGATGGNTCNCTTCGNATGTGTATCGACTATCGNCAATTGAACAAGGTGACCATTAAGAATAAGTATCCTCTTCCGAGGATNGATGACTTGTTTGACCAACTTCAAGGGGCNAGTTACTTCTCTAAGATTGACCTTCGGTCGGGTTATCACCAATTGAGGGTGAGAGAGGNTGACATTCCGAAGACGGCTTTTCGAACTCGGTATGGTCATTATGAGTTTTTGGTNATGTCGTTTGGNTTGACTAATGCTCCGGCGGCGTTTATGGACTTGATGAATAGGGTGTTTAGACAATACCTTGACATGTTTGTGATCGTGTTTATCGATGATATTTTGATCTATTCGAGGAGTGAGGATGAGCATATNGATCATTTGAGGATTGTGTTGCAAGTCCTTAAGGACCAACAACTCTTTGCAAAGTTTAGCAAGTGTGAGTTTTGGTTAAGGTCCGTAGCTTTCCTTGGTCATATTGTGTCGAGCAAGGGTATTGAGGTAGATCCTAAGAAGACGGATGCGGTCAAGAGTTGGCCTAGACCTCTANCTCCTTCGGATATTAGAAGCTTCTTGGGTTTGGCCGGTTACTATAGAAGGTTTGTTGAGGGGTTTTCCTCCATTGCCTCTCCGTTGACGGCTTTGACTCAAAAGAAGGCTAAGTTCGTGTGGTCGGAAGCTTGTGAGAAGAGTTTCCAAGAGTTGAAAGATAGACTTACTTCCGCTCCGGTGTTGACCTTACCGGAAGGTACNGATGGNTTTGTGGTNTATTGTGACGCCTCTAGAGTTGGGTTGGGATGTGTGCTTATGCAAAATGGTAAGGTNATTGCCTATGCTTCAAGGCAACTTAAGGTNCATGAGAAGAATTATCCNACCCATGATCTCGAATTAGCGGCGGTNGTNTTTGCCTTAAAGATTTGGAGGCATTACTTGTATGGGGTNCATGTNGATGTNTTCACCGACCACAAGAGTCTNCAATATGTGTTTAGTCAAAAGGATTTGAATCTTCGCCAAAGAAGGTGGCTNGAGTTNTTGAAGGATTATGACATGAGTGTCCTTTATCACCCCGGCAAAGCNAATGTGGTGGCGGATGCTCTTAGTCGATTGTCCATGGGTAGTGTNGCTCATGTNGAGGATGANAAGAAAGAGTTGGTTCGNGATGTNCATAGGTTGGCCCGGTTGGGTGTTCGACTAGTGGATTCCACCAAGGGTGGTGTCATGGTCCATAATGGTTCCGAATCGTCCTTTGTGGCGGATGTGAAGGCTAAGCAAGGTCTTGATCCNATTTTGGTNGAGTTGAAAGAANCGGTGCTCAAGAAGTCCGTTGAGGCTTTCTCCCAAGGGGGAGATGGNGTGCTTAGGTACCAAGGTCGNTTATGTGTTCCGAATGTTGATGGCTTGAGGGAGCAAATTTTAGNAGAAGCCCATAGTTCTCGGTATTCTATTCACCCGGGAGCCACCAAGATGTACCGTGATTTACGGGAAGTCTATTGGTGGAATGGNATGAAGAAGGATATTGCGGGATTTGTGGCCAAGTGTCCNAATTGTCAACAAGTNAAGGTCGAGCATCAAAGGCCGGGAGGTTTGTCCCAAGATATCGGTATTCCTACTTGGAAGTGGGAAGATGTGAATATGGACTTTATTGTNGGTTTGCCTCGTACTCGGNGGCAACATGATTCNATTTGGGTCATNGTAGATCGGATGACGAAATCGGCCCACTTCATTCCCGTCAAGGTTTCNTATTCGGCGGAAGACTATGCCAAGTTGTACATNAGGGAGATGGTGAGGTTGCATGGGGTNCCTTTGTCCATTATTTCNGATCGNGGTACTCAATTTACTTCNCANTTTTGGAAGTCNTTCCAAAAGGGTCTTGGTACTAANGTTAAGCTTAGCACGACCTTTCACCCNCAAACCGATGGGCAAGCGGAGCGTACCATCCAAACNTTGGAGGATATGTTGAGGGCNTGTGTNATNGATTTCAAGGGTAATTGGGATGACCAT

>rnd-1_family-559#DNA ( RepeatScout Family Size = 106 Final Multiple Alignment Size (possibly truncated) = 98 Localized to 2361 out of 4218 contigs )

GAATTATACAAATCCGCGAATTATACAAACGAGACAGCTTAAACATGTAGCTACGAATCGTAATTAGCAAACTATAGCTATGGAGCGTAATTAAGTTATTTTTGAGTGGCTATNTGCGAAAGTT

>rnd-1_family-446#Unknown ( RepeatScout Family Size = 153 Final Multiple Alignment Size (possibly truncated) = 98 Localized to 2361 out of 4218 contigs )

GTTGATCCAATTGAGTGGATAATGGTTGNTTATGATGTAATTGCATGATTTTAGTATGATTATACATGTATTCTTGATTAACCTAAGATTCTAGGGTGAAATTGATATAGTTTGGTTTTATGCCATGAAAAGGGAAATTGAGGGCTTAGATGTGATCTTCTAAGATTGATGAATTNCTTNATGAATTGCTTNAGATTAGANCACCTAGACATGAATTGCTTAAGAGTAAAGCTTGTAGACATGAATTGATTAGATTAGGGCNTATATGATGGATCATGACTAGTAATGTTGAGATTGAGCCTATATGATGATATATGACTAGAATCGCTTAAGAATGAAGCATGTGATTAGANTGCCTTGAGANCTAGGTATATATGATGATCGTGNGAAGTANNGCTTTGAGAGTAAGGCTTGTAGTCATGAATGTTGATTNCTAGGGCTTTGAGGGTAAAAGCTAATATGATGAATTANGNATGTTAAGGCTTTGAGAGTGAAGCTTATATGATGATGGTAGACTATTAGATGGNATTGTTGTGGAAGGACTTTACCCACATGACCTACATTATGAGTGTTTGAATGCATGAAGTGATTGAATCACTTGAGTTATGATTATATCCATGTCTAAT

>rnd-1_family-430#Unknown ( RepeatScout Family Size = 165 Final Multiple Alignment Size (possibly truncated) = 98 Localized to 2361 out of 4218 contigs )

AGGCCTAANCCATCGGCGGCCCCTTAAAGTTGNCANCATATTTCACTTAGACACCTCAACTAGGCNTTGTTCATNTTAGACACCTCATGTCGGGTCCCGTTGTGTCATTTTGACACTTTTTCGACAATCAGCCAAAANTATAAGGTGTGTGTAATACACTCGCGGATGACGTGGCAAAGTGACGAATTAAATGACGACACGTGGCAT

>rnd-1_family-332#Unknown ( RepeatScout Family Size = 251 Final Multiple Alignment Size (possibly truncated) = 98 Localized to 2361 out of 4218 contigs )

AGGGAAAAGGGTCAAATATGCCCCTAAACTATTCGAAAAGGTCTANATATACCCTCCGTTTAAAGTTTGGCTCANNNATGCCCTTGCCGTCCAACTTTTGGTCCAAATATGCCCTTATGGGCGTTAGTTGGCCTGCTGGAAATATCCAACTCATTTTNCATTTCTTTAAATGCCAAATGGAATTGCCACGTCATTTTTACATTACCACGTGGCATTTATATTAAAAGGAAAGGGGTCAANTATGCCCCTAAACTATTCGAACCCATAAA

>rnd-1_family-2#LTR/Gypsy ( RepeatScout Family Size = 1727 Final Multiple Alignment Size (possibly truncated) = 98 Localized to 2361 out of 4218 contigs )

TGTTATGTAATTCATGTTTGTATGATGATTCTACCCTAGGTTATGTATAATTTCTATGAAATTAGGGTTGAGTCTTTGAATGAAGCCTTGAAGGCTATGAAGGGAATATGTCAATTGCTTATATTGATGTTGATTATGTTATTACTTCATGAATTACCTTGTATTATGTATTGGTTATGAATTGNTTGGTAATTGAAGTATGGATGTCCCACGAAGGGCAAGAAGGTATGAAGGTTGGTTCTTCTTTGAACTCAAGTATGAAAGGTGAATTACTTAGATTGTAATGATGCTATACCTAATGTGTTGTTGTGGTGTTGATGACCTAGTTTCCTCATCCTTAACCCTCTACACTTGAATTAGCTATGAAGTATGTATGTATGTTATGGTATGATTGCTATATGATTGAAAGGTAGTTCTCATGATTATAGTGTAATGTAAAGTGAAAGGTTTACTCACTTGCTAAGTGTTTCTAAAGTGAAAGGATTGTTACTCACTTATGAACACATATGAGCTATTATGGTAAGATGTCTACATAAGAGTCTAGTAAAGGCTAATGTGAACTTATGTGATGACTAAAGATGATTACAAAAGGGAATTAGATGCTTAGCACCGAAAGGGCATGTAAATGAGATGGGGGTCTCACGTTTAGTAAGTCCGGCTCCCCACATGGGTTTCCTCACGTTTAGCAAGTCCGGATTACCCACGGTATGTGTTGTCTCATGAGATGGAAACCCCCACGTTTAGTAAGTTAGGGTTTCTAGAAGCAATCTCCTTGACCCTTAACTATGTGCCCACATAGGACTCTAGCTTAGTGGATCCACCTAGATAGCTATGTACGAATGGTTACACCTTAGGCAAGTGTTAACCCTCTCTTTTCGGTGTGGGAGTAGAACACCGGATTCCATGTAGCTCTCATGGTCTATGTCGGTTATTGCAATATTTCCCTAATGTAAAAGTAAATGAAGGTATGAAAGGTGTGAACTCTTACTAGGGCTTTCTTAAGGGTTTCTACATAGTGTAAGGGAGGGTATGGGACTTCTCTTGCACATTGCACTTGTGGGATCCTAAGAGATGGTTGTAGTAGTGTTCTCTTATGATTATGATGATTATGACTTATGTATGTTGAAGCTATGTTATGTATGATCATTATGAATATGGTAAGTTATGATGAAATATGATCTTATGAATGTATGCATGAAGTAGGTTGCTTAATGTGATACTTGGCTTTGAATAGGGTTATGGGGTTCTATTCTTTGCATTGCACTAGTATTGCTTGGGTTGTCTACATGTTGGGATGATATTATGTTGGTTTGGACTATGATGCTTACTTTGTGTGCATATTGGATTTACTTGGCAAAAAGCATGTTTTGACTAAAATGTCCTTTTATGCATGTTTCAATGGTTTTTATGCATAGTAGCCATACTTAGTACGTGTGTTGTACTAACCCATATTTTCTCCCTTTTCCCAAACATTTAGGTTCGGGCCGTTGAGGATTCAAGCTTACTTCTCAAGACACTTGGAGTTGCTTCCCCAAGTTGGTGAGTCCTCAAGTCCGAGGACAAGACCCTATGATCTAGCTTCTATGTTTAGTTCTTTGCTTATGTTGAAAGACATTGTTGTACTTTCCTATTTTCGAGACTTCTTATTGATGTAAGGGCTAAGTCCCATTTTCGATACTTCTATGTCTAGATGGTGCATTGTGACGAAGTTAGATTCTATTTTTCATATATGAAGTGAAGTTGAACTTCCAAAGTAAAAGTTTTAAATTTTCCGCGATTTTATTCTATGATACATGTTATGATGAATGCTAAGGGCTTGTATGAGACCTCTTCGGGGTCGAATACGCCGTGTNACGNCTAGGGGGTGCCCTCGGGTCGTGACAAACTTGGTAATCAGAGCACAAGGTTTAGAATGATTCTAGGATGATGTCTCATAAGCCACGTCTAGTAGAGTCTTGTTCATGAGTGTGAAGCGCGCCACACTTATGAATGAGAGGCTATAAGATGTTTAGGAAATTTCACTTCTTTCGTTACTCTNTAGTCGTGCCNTAGAGTCTTAACTCTATAAAGTCCTTTTCTCCTAATCCTTCTCTTGTGCTTNTAGGATATGAATACAAGAAGGGCTAACGCNAGAAGGGNCGAGGAGGAGAATGTGAACGAGGGAGTTCCTCCCCAAGCTCCTCAAGNTCCTCAAGCTCCTATTGAAGAAGGGGCTATGACTAATGCGGAGNTNAGGTCGGCTNTCCAAACGTTGGCTCAAGCCGTGACGGCTCAAGCCAATAGGGATGCTAGGGCTCNNGTGAACCCCAATGCGAGTACGACGGCTTCAAGGGTGAGGGATTTCACGAGGATGAACCCTCCNACGTTCTATGGCTCCAAAGTGGANGAAGATCCTCAAGGGTTTATNGATGAGGTNTATAAGGTNCTTGATGCTATGGGAGTGACTTCGGNAGAGAAGGCGGAGTTAGCCGCCTACCAACTNAAGGATGTGGCTCAAGTNTGGTACGANCAATGGAAGGAGGAGAGGCCNGTAGGAGCGGGTCCNATAGATTGGGAAGCGTTCAAGNCGGCNTTTCTTGATAGGTTCTTCCCCCTAGAGNTGAGGGAGGNNAAGGTGCAAGAGTTCATCAACCTTCGNCAAGGAGGTATGAGTGTGAAGGAGTATGCCCTNAAGTTCACTCAATTGTCCAAGTATGCTCCAACTTTGGTGGCGGACTCTAGGGCTAAGATGAGTAAGTTTGTTACGGGTGTGTCCGATCTT

>rnd-1_family-51#LTR/Gypsy ( RepeatScout Family Size = 1070 Final Multiple Alignment Size (possibly truncated) = 98 Localized to 2361 out of 4218 contigs )

TTGATGTTTATGCATGGCTATCATACTTAGTACATTTTTGTACTAACGCATACTCTTGCCTACATTTTTCACCAAATGTAGGGTCCGGCGNTCGNGGTTCTCATNCTCGTGGCTAGGATCTTGGTTGAAGCGAGNTTTCGAGNTTGGTGAGTCCTCATGNTTCGAGGATTTGACCACCATTTCCTTATTTCTTTCATTTTACGTTTTGGACATGTTGTATGGGCTGTGNCCCAATTTCATTCTATTGTANTAGATGGCTAATTGAGACAATGTCTAGACTTCCGCTTCGTTTTATAAAGACTTTGATTGTNTTGAAAATGTTTTAAATTTCTCATCGTTTCTATTATCTTATGTTATGATATGCTAAGTGGCTTGTATGGGGCCTTTCGGGGTCCTATACGCCATGTTACATCTAGGGGGTACCCCTGGGTCGTGACAAACTTGGTAATCAGAGCACAAGGTTTAGAATGGTTCTAGGATGTCTCATAAGCCACGTCTAGTAGAGTCTTGTTCATGAGTGTGAAGCGCGCCACATTTATGAATGAGAGGCTACAAGATGTTTAGGAAACTTCACTTCTTTCATTACTCTAAGTCGTGCCATAGAGTTTAACTCTATAAGGTCTCTCTCCTAATCCTTATCCGATGGTCTTCAGGATATGACTACTCGAAGGGCTNACGCTAGAAGGAATGAGGGAGANAATGTGGANCAAGAAGCTCCTCCTCAAGCTCCTCAAGCTCCGGTCGATCC

>rnd-1_family-284#LTR/Gypsy ( RepeatScout Family Size = 307 Final Multiple Alignment Size (possibly truncated) = 98 Localized to 2361 out of 4218 contigs )

TAGAATTGAAAGAAGCGGTGCTTAAAAAGTCCGTNGAGGCTTTCTCCCAAGGGGGAGATGGTGTGCTTAGATANCAAGGTCGTTTGTGTGTTCCNAATGTTGATGACTTGAG

>rnd-1_family-97#Unknown ( RepeatScout Family Size = 755 Final Multiple Alignment Size (possibly truncated) = 98 Localized to 2361 out of 4218 contigs )

ATTATGATCGATTATGNTTGATTAAGTCTTCTAATGCATGATTCGATGTTAATACATGATATTTAGTTGAAATTACATTGACCCATGCNTAATTCATGATTTTCAACTATGANCCTATGATGAACCCATGAACTATGAATTATGAATTTTGAAGATATGCATGTTTTTATGAAATTATTGTAAATGATTTAGGATGCTTTGAAAGGAAAGTATCAATGNTATTGTGAAAGGNTTTCTCACATACTATNATGATCATGATTGTGAAAGGTATTTCTCACATATGGATTCATATTGGTGAAAGGTTTTCTCACNTATGTTGAATCACGTTTAAGGAGCTACTCTATGTTTTGATCTTGAAAGCGTGGATTGGTATTGATCGTTGCCTTTCCNATGTTATGATGATATTTATGANTATGNCTATTCCGTTGGGATTTANACTTAGCACCGAGAGGACTTTGAGGTGGAGGCTCAAATAGGGTAGTCTCTAGTAGCAACCTCTAGTCCCAAACTACGTGCCCCCGTAGGATTGTCTTAAATGACCTAGCTAGTGGATCCACTTTAGCAAATTATGATATGTAGGGTTCTACCTTGGCAAGTAGANTCCCCTTCTTTCGGTGTGGGAGANACACCGGATTCCATGTTATAGCTCGCATGGTCTATTGTCGGTTAAAGCTAATATCCCACAAAATGAACTAAGTTTCCTTCAAAAGNTTTTATGATCCTTTATGTTGATGCATTGACCATGTTCATGATTTATGTATTCTTCTTTAAAGNTTTTNAATGTTTTAGCTTGGTCATGCATATCAATTTTTTTGTCCTTTATGCATCATTCCTATGTTTATGCATTTCCCTCATACTTAGTACATTCCATGTACTAATGCATACTTGTGCCTACATTGTTTCACTAATGTAGGGTCCGACGCTCCTACTTCCCACGCTCGTGGCTAGTTGATT

>rnd-1_family-105#LTR/Gypsy ( RepeatScout Family Size = 712 Final Multiple Alignment Size (possibly truncated) = 98 Localized to 2361 out of 4218 contigs )

TTTTATGCATGGCTATCATACTTAGTGCATTTTTGTGCTAACCCATATTTTCCTACATTTTTCCACAAGTGTAGGGTCCGGTTCTCGAGGTGATCTTCNTTTGGTTAAAGCTTGGATCGACTATTCTCCNAGCTTGTGGTGAGTCCTCATGGTTCGAGGACGGATGAGTCTCTTGTAGTTTCCTTTCATGTATTTCATTTTCGGACTATGTTGTANGGGCTGTGNCCCTATTTTGATTCGAGATTGTANTAGATGGCTAATTGAGACAANGTCTAGACTTCCGCTTGCTTTTATGAAAGACTTCGATTGTAAAAAGTTTTAAATTTCCGCATCATTTCTATTATCTTATGTTATGATATGCTAAGTGGCTTGTATGAGAC

>rnd-1_family-92#LTR/Gypsy ( RepeatScout Family Size = 772 Final Multiple Alignment Size (possibly truncated) = 98 Localized to 2361 out of 4218 contigs )

TTGGGTTGTAGTAATGGTTCTCCCACCGGAGGGTTAGTGTGGGTGCCAATCACGACGGTCTGGGTCGTGACAAAGTTGGTATCAGAGCCCTAGGTTCGTTGATCTCGNTGTACCAAAACGAGTCTAGTAGAGTCTTGCGGAACGGTACGGAGACGTCTGTACTTTTCTTCGAGAGGCTATAGGACTTTAGGAAANCTCTCTTCTTTCTNCTCCTTCGTGCTATGACTTGANTCCAATTGGTATCTGGCGATNCAAATTGGTATCTNACCTCCTTCACTCTACGGTCCGAACTAGAGCAACAAAAGTGGTAACACCATCACTAGCAAGAGAGGATGTGTATAGACTACCGACAGTTGAATAGGGTCACCATTCGGAACAAGTATCCATTGCCTCGGATAGATGACCTTTTTGACCAATTGCAAGGTGCATCGGTTTTCTCTAAGATTGATTTAAGGTCTGGTTACCATCAGTTAAAGATTGAGGCGGTTAAGAATTGGGTTCGACCAAGTTCTATGATTGAAATTAGCAGTTTTGTGGGGCTCGCTAGCTACTATTGTCGCTTGTGGTATGAGTTGAGTCCAATTGGTATCTCCCGATTCCAATTGGTATCTCAACGAAGTGAACTAGACGTCGTAGTCACGACTGAAGGGACACAAGCAAGTTAATTGGAACTGAGAAGGTTCCAATTGGTATCTGGCCCTAGGGGTCGTATAAGGCATGGATGATCAGTAATGGTCGTCTATGGAACGAACTAATTAAAGAATTTGGACTTGTCTGCGAGTTGTAACTGCTTGAGTGTCGTGAAACTTGTTGTGAAAAGAAAGTCATCACGAGATTAGGTGCAAGGGTCTAGATAAATTACGATGTGGTTCGTAAGCGAGAAATCTGGACGTGCGTTGCGACCAACTGATAACCGTGTGAGAGTCTGGGTGTATCCTGGGGATATGTGTGTGGTAGTGTTGAAACGATGTAAAAGGATTCTAAAGTGCGTACTTCGTTGGGTTGACCGGGGGG

>rnd-1_family-387#LTR/Gypsy ( RepeatScout Family Size = 194 Final Multiple Alignment Size (possibly truncated) = 98 Localized to 2361 out of 4218 contigs )

TACGGTCCGTAGGTCNAACCACGGACCGTNCTGGTCGACCGTGGNTNGCGTCAGAGACCGTAAAGATTTGGGCCTCGATCCACGGACACAGACCACGGACCGTGGTCCGACCTACGGACCGTGGGTCTGTCCGTGG

>rnd-1_family-478#SINE ( RepeatScout Family Size = 137 Final Multiple Alignment Size (possibly truncated) = 98 Localized to 2361 out of 4218 contigs )

CTTACCGAGGACATGACCCTAGATAGGAAGGTATGGAGGTCGCGGATTAGGGTAGAAGGTTAGTAGGTAGTCGAGCGTTGTCTAGTT

>rnd-1_family-373#LTR/Gypsy ( RepeatScout Family Size = 212 Final Multiple Alignment Size (possibly truncated) = 98 Localized to 2361 out of 4218 contigs )

AAATCAAAACCTTCCCTCCCAAAAAGTTCTCTCTAGAAACTCCATTGAAGANCAAGGTCGAGATCGAGCTAGGGTTGAGGGTTTCAAAGTGTTTCTTCTTCAATTTCGTGGGGTTCTTCAAGTATAAGGTATGGTGACTCTTCATCCTTGATTANCTTTCATTCAAGGAGTCAATTTCAAAGATGATTTCAAAGTTTTCAAAGTCACNAAAAACCTAGNTTTCAATCTAACTCATGGGTTCTTGCATNAAACGTTTTCAAATGATGANTTATGATTATATTGATGTTTAATTGATGGTTTAAGATGAAATACTCCATGAACCCATGATTTCTCCAAATTCCTAAATTTGGCCT

>rnd-1_family-346#Unknown ( RepeatScout Family Size = 238 Final Multiple Alignment Size (possibly truncated) = 98 Localized to 2361 out of 4218 contigs )

AATCAACTCAAAAATACCCCCAAAACCTGGTTGTCACGTGTACAAGCCTCTAANGTATTACAATNGATTCGAAAGAAAAACACAAGTCTCAAATGACGTTGTTTCTAGAATAGAACAAGATCATAAATAGGAGTAAGAAGGTCCGCTGAGATGACAAACAACTACCTCACAAATCTCCACAAGAAGCCTCGGANAAGAAGAGAAGAAAGTATCACGAAGATCCGGGCTCGTAACCTACAAAAATGTAGAAGCAAGGGGTGAGTACCAAACCACACGGTACTCAGCAAGTAAACCTCTAAACACAAGCTAAGGGGATAGAATACGGGTACTCCTTACACCCCAACCGAACCTCCACAACTACAACCTGCATAAAACCAGCCCAACCTAACAGTTCACAATTTACATAGCACACAGCTCAACAACAACACTCTAACAATTACATATCCTCAACAACAAGCTCAATATTCATCAATCACAAGTTCCACAGAAAGGCACTCACAAGATCAGCAACACACAAGATCAAGTTCACCAATGATAAAGATGTGCAATGCAATGAAATGCAATGTCAAGTATAGTGATGCATGTCTGACCTAACGATACACACCCGCTGTCTCTCAGTCCGGGACCCATGGGGGACATATCTGTCCATGCATCCATCGCGGCGCGCGACACGACCCTCGATAATAGNATCCGTCGCGGCGCGCGACACGTCCCTCGAAATATAGTATCCGTCGCGGCGCGCGACACGTCCCTCGAAATGGTACATCCTCTTTAATTTCTTATTCTTTCTCAATTCACATAACACTTGTCACAACCATGTCTCAGAACAATGCAAATGACATGTTCACACAAATAA

>rnd-1_family-519#Unknown ( RepeatScout Family Size = 122 Final Multiple Alignment Size (possibly truncated) = 98 Localized to 2361 out of 4218 contigs )

TGGACCCTTGTACTTGTATNNGTTTGTATTNTGAACCCTTCTACTTANCNNTTTGTCAACTGAACCCTTAAACTCATTAAAACGCAATATTTTAAACCCCTTTTTTACCACTCAACTGTGTGTGTATCACAAACGCCTGCCACGTAATTTTAAAAATAATTATAAAATGACACGTATGATTATAAAATGACACGTGTATATATAGTCACTCCATGTCAT

>rnd-1_family-243#LTR/Gypsy ( RepeatScout Family Size = 376 Final Multiple Alignment Size (possibly truncated) = 98 Localized to 2361 out of 4218 contigs )

ATCGTCAAAGTACCCTTGGCACGTATTCCCCTTATATAAATCAAAATGATCCTAGATTACACATGGTACATATGTTTATATGTTGGATATACAATCTGCCCATATCTTGCCAACTTGCTTCAATTGTCTATTCCATCATATATGTCATCTTTTCACATGTTCATATGTTCCCTTTATTGGTTATTACTCCCATTGTGTTAGACTATAATGTTACCTCACAAGATCCCTACTTGCTCTATTGTACATTTTATATGTACTTACTTGGAATATGTATCCGTCCTTAAGTTGAATCTGCCCACGTTCTCCCTTGGTTCCACGTTCATGTATACCTTGTATTTGATATTAGTTAGTGAGTATTTGGCTATCATATAGAGTTCAAATCTCTTGGCTGAATGTTAAATGTCTTCACTTGATATGTGCTTCAATTTGAGATGTCAAAATGCTTACTGAGTCATATAAGCTCCTAAGTATTACACGTTGGTGTCACATTTGCTTCCCATGTCATTGTTATTATCGTAAGTCCATATTCATATGTCTTCTACTACTGGTCCATAGTTCCAAATCTCAACAATGATTATGACCACCAAAACAACAATCTCAAAAGAGTTATGATCATCAAAAGAACAATCTCAAAAGAGTTATGAACATCAAAACAACAATCTCAAAGATTAAAGAATCTAAGTGAAGCAATCTGTATAATTATGGGTGGCAGCCCAGGGGCAAATGCCTAGCATGGGTCGATCCCAATCGGTATAGAAGGGTGGCAGCTCAGGGGCGAAAGCCTAGCATGGGCCGATCCCAATTGGTATAGAAGGGTGGCAGCTCAGGGGCGAAAGCCTAGCATGGGCCGATCCCAATTTGTGTAATTATGAGGACAGCATCCCAGGGGTTAAAATGCCTAGCATGGGTCATCCTCTTCTACCACTGATCAGCTGGTCATTCGTATCACATGCCTTATAAAGATCATAACGCACAGAAAAGTAAAGAAAAGAATGTAACATACACGATCCCACAAGNGTAAGCAAAGGTGGTCTCTAATCCTTATCTATTGGTACTTGGTTTCACTCGAGCTCTCATTTTACATATGGTTGTATTATTGCCTTACATATTCAGTACATTCTTTCGTACTGACGTCCCTCGTNGGGGACGCTGCATTTCATGCTGCAGGCACANGTACTCAGCTAGTAGACCTCCCCAATAGGAGCACACGACACTCAGCGGCTGTTGGTGAGCTCCAGGTTGATTCGGGGCTTTGCCGAGTCTTTACTATGCGTANTTCATAGTGGTACAGTCATGGATTCCAGTAGAGGCTTTGTAGACATTGTAAAGCTATCATCTGTATTCATAGTTTCACTTGTCACATGTATCGGTTCAGCTAGTCGACATGTGTTGTATATGAAGCTTCTCATTAGTAAATCTATCTGCATAGTAGTTTACTTTCTCATCATAGGTCACTTGTAAAGCATGTGTTCATATGGTACTTGTCCGCCTCACCTAGGGT

>rnd-1_family-176#LTR/Gypsy ( RepeatScout Family Size = 512 Final Multiple Alignment Size (possibly truncated) = 98 Localized to 2361 out of 4218 contigs )

TGTTAAGACTTGAGTTGCCATTTTGGCCAAGTTGAATGTTTACTTTTAAACATTCTNAGTTATTTTATTAGACAGTTGAGTTAAGTGCATTTGATCATTGTAATAATGCTTAGAGTCTTCCGCTGAGTAAGTAAGCCAGGCCAAGGGTTCGCTTGGGGCCAGCAATGGTTCTCGAGTGCCGGCCNCGCCCAGGGTGTAGGCTCGGGG

>rnd-1_family-442#DNA ( RepeatScout Family Size = 158 Final Multiple Alignment Size (possibly truncated) = 98 Localized to 2361 out of 4218 contigs )

GAGAAAATGGTCAAATGCCCCCCCAACCTATANCCGAATTCCCAACTACACACTCATACTTCACGGGGGTCCTATTACCCCCCTGAACTATTTTAAANTGGAATTATTACCCCCCTAAACGCTGACNTGGCAAAGNGAGTGTATNTCACTCTCTTTGAGAGAGTGAAGACGAAAAANNAATATTAAATTTTTATTTTTAATATTTTTTTAAATATATTTTTNTTTTTTNTTNTTTTTTTTTTTTATTTTCT

>rnd-1_family-36#LTR/Gypsy ( RepeatScout Family Size = 1182 Final Multiple Alignment Size (possibly truncated) = 98 Localized to 2361 out of 4218 contigs )

TGCCTAGAAGGGCTATGAGTTGCCTACGGGGCTATATTGACGCCTAAGAAGGCTATGTGCTTCCTACAGGGCCATGTTGTTGCCTAAGAGGGCTATGAGTTGTCTATAAGCTATGTTGATGCCTAAGAGAGCTATGTGCTGCCTACGGGGCTATATTAATGCCTAAGAGGGCTATGTGACTGCCTACGGGGCTAGGGGACTACCGATAGGGGTATATAGGCTGATTGGCACCTTCCGGGCTTATGGGGGCCTGAGTAGGTGGTCTTGTGTGTTGTTTGTACCTGCCGAGCTTATGGGGGCTTGGTTAGGTTGTTGTTTTATTATTTTTGATAAGTTTAGATTTAGGAGCGGGTCAGTACGCTTATCTTATCCTTGATTTATTTATCGGATTATTCCGGTATATCAGGATACCTCCTCATAGTCTATTGCCTTTCATACTCTGTACATTATTTTGTACTGACGCCCCATTGCCTTGGGGGCGCTGCATTCATGCATGCAGGTCCCGACAGACGACCGAGTAGACCTCCTCAGCAGCAGGATTGACTTCTATCCGGTTGGCTAGCCCCTTTCCTCCGGAGCTGCCAGAGTTGGGAGGTTTGTTACCTTTTGTTGTATATATCTTTATGGGTAGGCTGGGGCCCTGTCCCGCCAATCTTTACTACTCTTAGAGGCTTGCAGACTAGTGTGTGGGGTGTATAGCTACGTTATGGCCATGCTGGCCTAGNTTGTTGGTTTGCTGAAGCTTGTGAGCTGTTTGATGTATTGTATTGATATATACCTGCTTTTAGTTTTGGTATAGATATCATGGTGGCCTCGACGGCCCAATCAGGACTTCTGTGCTAGTTGGCTATTTATTTATATGATCTCCTGGGAGTAGCTGTTTCTTTTATAGATCNGTTGACAGGGGCCTTGCCGGCCGAGGGCTTAGTTTTAAGCCGAGATATACTTGTTTGTTTTCTTGATTGCGTGTGGCTGCCATGCGCTAGTAGCAAATGGTTCAGCAGGGTCGGCTCGGGCCTGAGTTTTGGCATTAGGAGCCAGTTGCGCCCCTCGAGTTTGGGGCGTGACAAACTTGGTATCAGAGCAACGTCGTATCCTAGGGAGTCTGCAAGCCGTGTCTAGTAGAGTCTTGTTTATAAATGTGTTGTGCACCACATTCGATAAGCAAGAAGCTACCGGGCATGTTAGAATGACTATCCTTCTTTTGCTCACCTCGTGCTATAGAGCGGTGTCTTAAGGGTTGAGGTTTCTGGCTTATAATTGATACTTGTATGCAGATAGAGATGGCTGACACCAGACAGACCACAACTAGCCAGGGGCTAGATACAACTGCAGGCGAGGGTACCGGCAAGGTACCACAGGTGGAGGTAGCCCATTGTGAGACCCAGGGGGAGACATCTTCTCAGCCCCCTATAGCTTCTCCACCGCTTCAGGAGCCTCCGAGGGCTACAGGACTTCCGGCTCCACCAGCAGTACCACCTCTAGTTCCTCCTATTCCATCTGATCAGGATTTCAAGAGTGCAGTATGTATGTTAGCACAGTTAGTAGCTGCACAGCGCCAGCCAGTTGCGCCAGATGTTGCCGGACCTTCTGAAGGGCCCGGAAGTTCGAGAGTTCGTGAATTTCTTGCCTTGAATCCTCCACAGTTTACAGGGACAGATCGCAGAGAGGACCCTCAACACTTTGTTGATCAGCTTCATAGGATCTTCAGAGTCATGCATGCCTCAGCGACTGAGTCAGTTGAGTTAGCAGCATTTCGATTGCGCGATGTTGCAGTATTGTGGTACGAAAGTTGGGAGAGATCTAGGGGAAAAGATGCATCTCTACCCACTTGGGATAGCTTTACAGAAGCTTTCATAGATCACTATTTGCCTCGAGAGATTAGGGATGGCCGGGTGGACCAGTTTCTGAACCTCCGACAGGGCGGCATGAGTGTCCGAGAGTACGGGCTGAGATTTGATTCATTGGCTAGATATGCACCAGCATTTGTTGATACGATGCATGACAGGGTGCGTAGATTTGTGGGAGGGCTCGACTCAGACTATATCGACGCTTGTTCTACCGCTGCACTAAATGATAATATGGATATCTCGCGAATTCAGGCCTTTGCACAAGGCATAGAGGATCGCCGACATCTACAGTATATGAGTGAGAGGGTCGAGAGAGAGAGACAGAAGAGGGCTAGGCCCGCTGGTTCGCAGGGAGATTTTCAGGGTGGTCCCAGACCCCGATATTCTATTAGGCCACCTAGACCTCCACCACAGCAGTTCCAGGGTAGTAGATTTGATCGTCAGGGACAGTCAGGCCCAGGTGAGGGTTCACGAGCATCAGGCTCNCAGCAGCAGAGGGGTTCAGGCCAGGCTAGGACAGCCCCGCCGCGTTGCGCTACTTGCGGTAGGATGCATTTCGGGAGGTGCAGACAGGGTTCCACAGGTTGTTATTCTTGTGGACAGGAGGGGCATGGGTGGAGAAACTGCCCNACCATAGGTCAGGGTGGTATAGGTCAGTCGACCGGGTCAGCAGTAGGTTCCTCCTCATCAGCACAGTCTACAGGGCGTGGACCCCAGACTTCAGCANGTAGAGGTAGAGGCGGAGGCAGAGAGGGAGCATCTAGTTCTGGTAGTGGACAGAACCGTACGTATTCACTAGCCGGCCGACAGGACTCAGAGTCATCACCAGATGTTGTGACAGGTACATTGATTGTTTGCTCCCATTGTGTTTATGCTTTGATAGATCCTGGATCTACTCTGTCTTATGTTACTCCATTTATTGCGGGGAAGTTATGTATAGTAGCAGAGTCATTAGATCGACCCTTTATTGTATCTACACCAGTTGGTGAGTCTATTGTTGCCAGGAGAGTCTATCGAGGTTGCACGGTAGAAATTATCGACCGTCAGACCTCTGTAGACCTCGTAGAGTTAGAGATGGTCGATTTCGACGTCATTATGGGTATGGACTGGTTAGCGTCTTGTTATGCTAATGTTGAGTGCCGGACAAAGATTGTTAGATTCCACTTTCCAGGAGAGGCAGTTCGGGAATGGAAAGGCGATACAGCAACGCCGAAAGGTAGGTTTATTTCCTATCTTAAGGCGAGGAGGATGATTACTAAGGGTTGTATTTATCATCTTGTTCGCGTTCATGATATAGATGCAGAACCACCGACTCTTCAGTCTATT

>rnd-1_family-556#Unknown ( RepeatScout Family Size = 107 Final Multiple Alignment Size (possibly truncated) = 98 Localized to 2361 out of 4218 contigs )

GTTTGAGCGAGCCGATTTTTCNGAAAACGCGTTTTGCTCATTCTCATACGAGTAGAAATGTGACGAAAAAATTAAAGGGAAATTCGACCGAAGCGAATGAGTTTTTCGAATAGATCTCGAAAAAATACGGGGAATCAAAAAAAAATTGCGTCAAAAGGACGTTCGAGTCGAAAGTTACGATCATTCGAAAATTCAGCATCTTAAAGGTCGAAGGTAGCCGGGGAAAAAGGAAATTCAAACGGCCATAACTCGGGCCTCGGACGTCCGTTTCAGTCGATTTTTTTTTTGAACCTGCGTATTTTTTCGATATCTACGCAACCGAACCGTCGTAANGCGGTTCGAGCGAGCCGATTTTTCGGAAAACGCCTTTTACTCATTGAAATACGACGAAAAAGTAAAAAGGAAATTCAATGAGAGGGGATGAGTTTTTCAAATAGATCTCGCAAAAATACGAGGAATCAAAAAAAAATCGCGTCGAAAGGACGTTCGAGTCGGAAGTTACGACCGTTTGAAAAATTCCCAAAGATCGAAAGGCGGCCGTGAATTTAGGAAACTTCAAACGGCCGTAACTCGAGCTTCGGACGTCCGTTTCAGGCGATTTTTTTTTTGAACCCGCGTATTTTTTCGATATCTACGCAACCAAACTGCCGTAAGGCGGTTTGAGCGAGCCGATTTTTCGAAAAACGCCTTTTGCTCATTCTTAGGCGAGTAGAAATAAGATGAGAAAAGAAAAAGGAAATTCAAACGGCCATAACTCGAGCTTCGGACGTCCGTTTCAGGCGATTTTTTTTTGAACCCGCGTATTTTTTCGATATCTACGCAACCGAANCGCCGTGANGCGGTTTGAGCGAGCCGATTTTTCCGAAAACGCCTTTTGCTCATTCTTATACGAGTAGAAATATGACGAAAAAATTAAAAGGAAATTCGACCGAAGCGGATGAGTTTTTCGAATAGATCTCGAAAAAATACGAGGANTCAAAAAAAAGATTGCGTCAAACGGACGTTCGAGTCGAAAGTTACGATCATTCGAAAATTCAGCATCTTAAAGGTCGAAGGTGGCCGNGGAAAAGGGAAACTTCAAACGGCCATAACTCGGGCTTCGGGCGTCCGTTTCAGGCGATTTTTTTTTTGAACCCGCGTATTTTTTCGATATCTACGCAACCAAACCGTCGTAAACCGGTTTGAGCGAGCCGATTTTTCGGAAAACGCCTTTTACTCATTGAAATACGACGAAAAAGTAAAAAGGAAATTCAA

>rnd-1_family-233#LTR/Gypsy ( RepeatScout Family Size = 399 Final Multiple Alignment Size (possibly truncated) = 98 Localized to 2361 out of 4218 contigs )

AAGGTGGGACTATTTCAAGAAGGCANGAGCTGCCTCTGAATCCTATNTTGGAGGTNGAGTTGTTTGATGTNTGGGGNATCGATTTCATGGGTCCATTTGTGAGTTCGTATGGGAANAAGTATATTTTGGTGCGGTTGATTATGTNTCNAAATGGGTGGAAGCNGTCGCGCTTCCNAACAATGAAGGNAAAAGTGTCGCCGCATTCCTGAAAAAGAACATCTTCNCNAGATTTGGCACNCCNAGGGCGATTATCAGCGATGGAGGTTCCCACTTTTGTAATAAGTTGTTCAAGGCGCTNCTTGAGAAATATGGNGTNCGNCACAANGTAGCNACNCCTTACCATCCGCAGACNAGTGGNCAAGTCGAAGTGTCGAACAGAGAGATCAAACAGATCTTGGCAAAGACNGTGAATGCCAATAGAACNGATTGGTCNAGGAAGCTNGATGATGCTCTATGGGCATATCGCACCGCNTNCAAGACNCCCATAGGTATGTCTCCNTACCAACTNGTATNTGGGAAGTCNTGTCATTTGCCGGTNGAGCTAGAGCATAAAGCNNTGTGGGCGNTGAAGAAGCTAAATTTGGATTGGGGCGCCGCGTCGAATCAAAGAGTGAATCAGNTGAATGAGCTNGATGAGTTTCGNCTNAAAGCNTATGAAAGTTCAGCCTTGTACAAAGAGAAGATGAAGAGGTGNCATGATCAAANAATCGAAAAGCGNGAATTCGCGGTNGGTGATCTGGTGCTNCTATTCAACTCNAGGTTGCGNTTGTTTCCCGGNAAGCTCAAGTCCAAGTGGACCGGGCCATTCANAGTNACGCGAGTGTTCCCACATGGAGCGGTTGAGCTCGAGAACAAGGANGGANCGAGGTTCANGGTGAACGGGCAAAGGNTNAAGNTCTATTTGGGAAATGCCGAGAGTGTGCAAGAAGTGATTGAGGCCTACNATCTTGATGAA

>rnd-1_family-200#LTR/Gypsy ( RepeatScout Family Size = 474 Final Multiple Alignment Size (possibly truncated) = 98 Localized to 2361 out of 4218 contigs )

TGAAATAAAGTTGTGAAAGTTGCAAAAGAAATGGGGTNTCCGATANTCCATGGAAAGTGAATAATGGGGTGAATTNTGAGCACGAATGAAAATGATGAAGAAAAGGAAAGAAAGTGATGTTCANACCACGCTTCGTGAGGGTAGAAGCCACTGAGCCTAAATGACCATACCTTTACACTCAGCCCCGTTACAAGCCTTGAAAAGACCTTTGTGATCTTGAGTGAGCTGAAACGAATGTTGATTGGAAAATANGGGCAAACCTATGGGTGAAGTCATGCATGTGTTCATCTTTGTGAGTGTGAGAGTTGTATNTGATTCCGGAACTNTAAATTGTTGAACNATTTGTGTGAATATGGAATCATTTTTGTGTGAGGGCATTTGAATACCTTTGTTGAGCTTGAACTTGCATTTGAAGCAAGTATTGTGAGCTTGAGANTCTTTGATAATGGCGAGTCACAACTTGAATCTTTGAGTGCACGATTGATCGTTGCATGAGTAAGTTGAGTCTTGTTGTGTGCATTCATGATTGAGTCTCGTGTAGCACTGTTTGAGACATCCTTNTTGAACTGCTGANCTTGAGTTTTACTTGAGGACAAGCAAAAGTTTAAGTTGGGGGTGTTGATGAGTCCGAGATTTGGACTCATTTAGGGCTTTATTTNNATAGATTTAGTGTCCTCAAATGCCTATTTTGTGCCAATAACTGATGTNAAATCCTTGATTTTCAGGTATNTGGATTGAGGAACAAGGCAAGGA

>rnd-1_family-285#Unknown ( RepeatScout Family Size = 307 Final Multiple Alignment Size (possibly truncated) = 98 Localized to 2361 out of 4218 contigs )

GGGAAAATGCATAAGTACCCCCCAGCCTATGCCCGAAATCCCAGAGACACACCTAAACTATACTAAGGTCCTATTACCCCCGAACTTATTTTATATATAATTTTCTACCCCTTTTCGGCCTACGTGGCACTATCTCGTGGGCCCAGCGCGCGTTGACAATTTTTTCAAGGATAGTGCCACGTAGGCCGAAAAGGGGTAGAAAATTATATANAAAATAAGTTCGGGGGGGTAATAGGACCTTAGTANAGNTTAGGTGTGTCTCTGGGATTTCGGGCATAGGCTGGGGGGGTACTTATGCATTTTCCCT

>rnd-1_family-167#Unknown ( RepeatScout Family Size = 534 Final Multiple Alignment Size (possibly truncated) = 98 Localized to 2361 out of 4218 contigs )

TTCCCCAATTTGAAGAAGAATTCCCCAATTCNAGTTAGGGTTTCTTCCAATGTTGTGGGTTATGATGAATGTTGATCCAATTGAGTGGATTATGATTGTTTATGATTGAATTGGTAGAATTACATGTTTTTAGGATGAAATTACATGTACCCATGCCTAACCCATGATTCTAGGATGAAATTGATGTAANTGGGTTTGATGCCATGAAGGGAAACTTTATGGGTTAGATGTATCCTTNTAGGATTGATGAATTATGANTAGAATTGCTTGAGATTGAAGCATATATGATGAATATGATNTGAATTGCTTGAGANTAAAGCATATATGATGATCATGAGATGTAGTGCTTTGAGAGTAAAGCTTATGNTATGAATTGTTGATTATTGATGTTATTGTTGTGGAAGGGATACTTCCCACATGAATTGCTAANTATGAATGTATGAATGTGAAAGGTTTTCTCACATGAATGATTCTAGGTTGAAAGGATTGCTCACCTAAAATGAATCTAGACTAAAATGCTTAATGA

>rnd-1_family-461#DNA/CMC-EnSpm ( RepeatScout Family Size = 143 Final Multiple Alignment Size (possibly truncated) = 98 Localized to 2361 out of 4218 contigs )

CGGAAAAGGGTCAAAAATACCCTTAAACTATNCGAAATAGNTCAAATATACCCTTCGTCTATATTTCGGCTCAAAAATACCCTTNCCGTCAAACTATTGGNTCAAAAATACCCTTCTNATTAACGGAAGTTGTTAAATGCCATGTGGATGCCACATTGCACGCCA

>rnd-1_family-274#Unknown ( RepeatScout Family Size = 326 Final Multiple Alignment Size (possibly truncated) = 98 Localized to 2361 out of 4218 contigs )

CCTTGACGGCTCGTGGAGCCTCNCGTGAAGAGGTTCGGTCAGCTNTAAGTTCAGGGGCNNTTGGGTCTTTTCCNAATTANTTTAACTCAATACTATGTCGTTTTGCCCATGCCTAGGACCCCTATATAAGTGATTTTAACCCCAAAATTCACCCATTCAANTCATTCTCTCAAATTCCCAAAAGAAATCGAGTTCTTCCTCTCAAATATTTCTCTCTCTAGAAACTTGAAGAAGAAG

>rnd-1_family-472#DNA/hAT-Ac ( RepeatScout Family Size = 139 Final Multiple Alignment Size (possibly truncated) = 98 Localized to 2361 out of 4218 contigs )

AACTTACATAAATCTCACTAGTTTAGGAGCTAATTACTTAGATACACTCTAGTTTGCAATATTACGAATCTTACCAGATTTTGGTGCGTCCAGATACGTCCAGATACATGTATCTCGGGATACATGGGGTCAAAATTAGGTGTAATTTGTTCTAGATACACTGTATCCAAGTGGATTCGCATGTATCTGAGATACATAACAAATCTCGCTCGCCTCCCTCCCATCTCT

>rnd-1_family-86#Unknown ( RepeatScout Family Size = 826 Final Multiple Alignment Size (possibly truncated) = 98 Localized to 2361 out of 4218 contigs )

GAAGAAGAAGAGAGGGTCAAGCTAGGTCGAAATTCCTCCATTGTTNCAAGCTTTTGTTAGGGCTTTGAATCAAGGTATGTTAGCTTATTCATCCATGGGTTCTTCCACCCATGGAGCCCCCTAGATTTCCCCCAATTGTGAAGTCAAAAACCCCCAATTCTAGCTAGGGTTTCTTCCTACATTGTGGGTAGGGTTTGGGTATGATTCCAATCGAGTGGATANTACCCGNTTATGATTGANTTAGCATTGAATCATGATATTATGATGAATTTACGTGNTTTCCATGGTTAACCCTAGTCTAATTGATGAANTTGATGTTTGTGGGTTTATGCCATNAGAGGCGAAATTGAAGGTTCATAGGTGATCTTCTNAAATCGATGNTATGTGTAGTAATTGTTCAAGATTAGGAGCATATAGACATGAATTGAACTAGACTANAGTATATATGATGGATCNTGACTNGTAAAGNTTGAGATTGAACCTTTATGATGAATTATGACTAGAATCGCCTAANAATGAAGNATGTGATTAGAATGCCTTGAAATCTAGGTATGTATGATGATTGTAAGAAGTAAAGCTTTGAGAGTAAGGCTCGTAGTCATGAATGTTGATCGCTAGGGCTTTGAGAGTAAAGCTAATATGATGAACTATGNATGTTAAGGCTTTGAGAGTAAAGTCTANATGAGAATAGAAGNCTAATAGGTAGAATTGTGGTGAAAGGCCTNTACCCACCTAATCTACCTTATGAATGCTTGAATGTATGAAGTGATAGAATCACTTGAGCATGGATTGATTGTCAAGGACATCCTTCCATGAGACATGAGTGTTAAGCAAGGCTTAACAAGTAAATCTTGGGGTGTATGCTTGGCCCCAAGTGGATATTGACAATGAGATGGAGTGTCACACAAGATAGGGTCCGGGCTCCAAGTAGAAAGGAGAACCACACCTAGTTGATGTGAGGACTATCCAAGTTAAAGGGGGAACTCTTACGTAGTAGAGTGTGGGTTTTCCCAAAGTTAGGTCTCATGAGATGGGAGCCTTCACATAGTTGAGTTAAGGTTCCTAGTAGCAATCTCCGTATCCAATGAACTAAGACATGCTTGAAGAAGTATCTCNTAGTGTTCAAAAGCATTAATGAACTAAGACNTACTTAA

>rnd-1_family-68#LTR/Gypsy ( RepeatScout Family Size = 912 Final Multiple Alignment Size (possibly truncated) = 98 Localized to 2361 out of 4218 contigs )

CGATCGCATCGGCGAAGGANGACCATGTCGCCGAANGTACTGATGGAGATCAAGACGAGCGCTGATNGGCGAGCTAAAAGGCNAAAGGGCGCATCGCGGAATGAATCGGCGATCCCGATCTAGATCGCCTAAAGTTACAGTGGTTCGAATAAGAAGATGCGAAGAAAGAAGGCGATGGANAAGGACGATCGGCGAGTCGCCGACCGGTCGGCGAAGCCCGACTTNCCTCGCCGATTGGCCCCAAAAAGCCACACCTCGGANTNGTATAAATTGATTTTTGGAAGAGAGAGAAGGCATTCAGAAATTGTGAGGAACGAAACATAGAGAGCAAAAACACTGCTNTTGTTGATTTTAAGCGATTTTAGCAAGATTTTTCAATTGGGTTTTGTAATAGAGAAGATTTTAGTTCATTTTAACTTTCGAATTTCATTTACCCAGCTATGGANAACGTAGTTGGTANCGATTTTTATCTTTTTATGATGTTTGGCTAAAACCCCAATCTTGGGGGTGTGATCATGTGATTAATTGTTGNATTTAGCTTATGGGTGTTGTTAATTACTAATTTAAGTGTTAATTTGAAGTGGGTTTNAATTATTATTGTGGTTTAATTTATGAATTGTAGTTGCAAATGCAATTCTAGTTTAGTGTTCTNNGCTTGCTCGAGAGAGAGGTNTGAAGCCAAGGTATTGATTGATGGCATTCGTAGTNATTGGGTCGTCGCGGNTTCAGCTCGAGAGAGTGAATCCTAAACTCAATCCTACGTATCTAGCTCGAGAGAGTGGATTTGATGAGGTTNTGGGCTGTTCATCTTATGGCTAGTGTCGAGGTTCGAGAGAACTCGCTTGANTCGNGGTAGTTGTTCGAGAGAAAATTGCCTCACCTATAGTCTAGCCTACCCACTAAGATTTAGCATTGTTAGTAATGATNTACACCCATTNAGTCGAATTCGGCAATTGGTTAGTCGATCCCCCAAGNATTCTCTCNCTATTTGATTGTTCCTTGTTGATTGTTGAAGTGTGTTGGTAACAACANCCTGGAATTTGANNTTTAAACTCTTAAGTGTNGTGTTGTTCAGCTGCAAAGGTCGCCGTCGTACTTTCATATTCGAATTCGAAAGAAAACTACTCCCTGTGGGANTCGACCCCAACTCATTTAGTTGGGTTATATACTGACTAGCGATCGTTGACACTTAGAATTGGATGAAGTGTCCTTGAAACGTTAATCAA

>rnd-1_family-79#LTR/Gypsy ( RepeatScout Family Size = 839 Final Multiple Alignment Size (possibly truncated) = 98 Localized to 2361 out of 4218 contigs )

TGTTTTAACAGTTTTACAAGCTTTATATATTGCACGTGTCTTATTGCTTTATATATTGAGTTCAGTTATTCATGAGTTGAGCAGAGCCAAGGTAAGTGTTCTTTCGTACTCCTTTCAAGCTTANGTTGTGTTTAGCNTTCCAACTCGCATACTCGTACATTCAATGTACTGATGCCAGTTGGCCTGCATCTTATTATGATGCAGACGCAGGTAACCAGGATCAGCATCCGGCGCGCCGTTGATCCAGCTGAGCACTCCAGAGTCAGTGGTGAGCCTCCTTGCATTCCGGAGGACTCNNTTATTTGTTCTTTTAGTTTTGTTTTATTAGGATGTTGCGGGGTCTGTCCCGACATCCATCTCAGTATTTTAGAGGCTTCATAGACAGTCAGTCAGTTAGTATTGAGTCTCTCATCTATGTATATATGTAAATATTCTATTTTGAGACTCGAGTTGCCTTTTTGGCCAGATTTTATCAGTTNGGTTGTTTTATAACCTTTCATTGCATTGAGTTATTCTGTTGAGTTAAGTCTTCCGCTGAGTTAAGNAAGCCAGGCCAAGGGTTCGCTTGGGGCCAGCAATGGTCTTCGAGTGCCGGTCCCGCCCAGGGTGTAGGCTCGGGGCGTGACA

>rnd-1_family-19#LTR/Gypsy ( RepeatScout Family Size = 1340 Final Multiple Alignment Size (possibly truncated) = 98 Localized to 2361 out of 4218 contigs )

TTGAGTTCTGAGTACCGAGTTTCCATATTGCTATTAAGATCCTTGAGTTGAGTCGTTCATGTCCATAATTCCGCATGAACCCTATGANTTGAGTTATAAATTTTGAGAAGCTTTTTAAAGCCAACACTTGAGTTCTTAAACTGAGTTTTGAGAAAAGAGTTTCTAAAACATGATTAGTATGACAATCGAGTATGCCATCAATGTATGAGCAGTATGGTATCTAGATATACCATCAATGTTTATGCAGTATGACTTCCCGAGTCATCAATGATCATATTACAATATGATTTTGATATGTTTTTGAGAAAGAGTTTTCGAGTATGAATTGAGTAAGAGTATAAGGGAACTAAGTATTCCCAAAGGTATCAATTTTTACATTGANAAAAGAGAAACTTCGATTTCTAAATGAGTTATGAGTTCAAAGATATTTCAAGAGCAGTTACCTCTTTTAAGAGGATANTTTGAGCAATTATCTCAAACTAGAGGAAGAGTTATGTTTTTAAACATATGAGCATGAGTATATATTATTGGGAGTAGTATTGAGCACCGATATGGGGACGAGTTCAGACAACTCACANTCCTCATAAACCATGTATGCCAACATGGGTAAAGGATCATACTTTTTAGATGATTCCTTATTGCTTTTAAGCATAGCTTAGTGGATCCACTTAGTTGAGGTGTTCTATACCCCGGCAAGGTATAGGACAGTTCTGGCAGCGTGGGCGAGACGTTGTATCATCACGTAGCTCATAGTGATGGTTGTCGGTTAGAGAATCTCCCAAATAGAGTNAAAGTGTATTTTTATATATCACTTATTATGTTGAGTTCAGAGATGAGNAAGTATTTTGTAAAGCTTTAAATGATTTCACTCCTTTATTTCTTTACATTCAGTTGAGTATATTATTATCTATTGCAGTCCTTTCGTTCAGTTATTTGTTATTCAGCTATATTACATACTCGTACATTCAATGTACTGATGTCATTCGACCTGCATCTTTTNATGATGCAGATACAGGTNTTTAGGATCATCAACAGGCGCTCCGTTGAGATCACTTCATCCGCTCGTCAGCTTTGGGTGAGACCTCCTTGCTTTCGGAGGACTCCANTTTTAT

>rnd-1_family-550#LTR/Gypsy ( RepeatScout Family Size = 110 Final Multiple Alignment Size (possibly truncated) = 98 Localized to 2361 out of 4218 contigs )

TGAGCTAAATCTTCAAGTTGAAATGCTCCTCGACGCACGAGCCTAAACTGCATGTCACAAAGCTCTTCAAATTCGAGCTAAATCTTCAAGTTGAAATGCTCCTCGANGCACGAGCCTAAACTGCATGTCA

>rnd-1_family-257#Unknown ( RepeatScout Family Size = 348 Final Multiple Alignment Size (possibly truncated) = 98 Localized to 2361 out of 4218 contigs )

CTTCACGAGCCGTCGAGGGCACCACGGGCCGTGGTGNCGCCCGTGGTCCCTTGACCAGTGGCTNCCTCGACTTTGCAACGCCCCCATCAAGCTAAGGCANCNCCACGAGCCTTCCCACGGNCCGTGGTCNTGACCACGAGCCGTGAAGTGGCTCGTGAAGGGGAGCCTTGACTTGGCACCGTTTTGGGGCGCACTGCCTCGCCACCACGGGCGGCACCACGANCCGTGGTGGCCACCACGGGTCGTGAAG

>rnd-1_family-386#LTR/Copia ( RepeatScout Family Size = 195 Final Multiple Alignment Size (possibly truncated) = 98 Localized to 2361 out of 4218 contigs )

AGAATTGGCCTTTNCATCAANTGGATGTCAANAATGCNTTCCTNCATGGTGATCTNNANGAAGAAGTNTATATGGAGCAACCACCTGGTTTTGTTGCTCAGGGGGAGTATGGGAAAGTNTGTCGATTGAANAAGTCNTTGTATGGNCTNAAACAGAGCCCCCGGGCNTGGTTTGGNAAGTTCAGCGAGGTAGTTCAAGAGTTTGGTTTGAAAATGAGCAAATGTGATCACTCNNTNTTCTATCGACAATCAACCGCTGGCATTGTNNTTCTTGTTGTNTATGTNGATGACATTGTTATCACAGGAAGTGATTATGCACGGATCTCATCCCTCAAGTCTTTCCTGCATACGAGATTTCANACGAAGGACTTGGGNCAGCTGAANTACTTTCTGGGNATNGAAGTAAATCGAAGCATGAAGGGAATTCTTNTNTCTCAGAGAAAGTATGCTCTNGANCTGCTTGCAGAGACTGGAAAGTTGGCAGCAAAACCCTGCAATACTCCAATGGNTCCNAATGTGCATCTTACGAAAGATGATGGNGANCCTCTTGATGATCCAGAGAGATACAGGAGGTTAGTTGGGAAGTTAAATTACCTCACTGTGACTCGNCCNGATATCGCTTTTGCNGTAAGTGTTGTTAGTCAGTTCATG

>rnd-1_family-272#LTR/Gypsy ( RepeatScout Family Size = 328 Final Multiple Alignment Size (possibly truncated) = 98 Localized to 2361 out of 4218 contigs )

TATTGAGATCCTTGAGTTGAGTCGTTCATGTCCATAATTCCGCATGAACCCTATGATTTGAGTTATAAATCTTGAGAAGCTTTTTAAAGCCAACACTTGAGTTTTAAACTGAGTTTTTGAGAAAGTAAAGATGAGTTTTGAGAAAGAGTTTCTAAAACATGATTAGTATGACAATCGAGTATGCCATCAATGTNTAAGCAGTATGGTATCTAGATATACCATCAATGTTTATGCAGTATGACTTCCCGAGTCATCAATGATCATATTANAATATGATTTTGATATGTTTTTGAGAAAGAGTTTTCAAGTATGAAT

>rnd-1_family-409#LTR/Gypsy ( RepeatScout Family Size = 179 Final Multiple Alignment Size (possibly truncated) = 98 Localized to 2361 out of 4218 contigs )

AGTTTTAGTTTTGTTTTGCTTGTTTGAANACAGAGGAAATGAGCGTGACGGAAGCATATGGTAGCCAAACGGACCACCCTCCTTCGAAGNTGATGANTCTTAGGGATAACATCCTAAGTTTCAAGCGNTTGGAGGGTGAGCCAATCCATGAGACGTGGCTGAGGTTTAAGACGCTGNTGCTGCAATGCCCAACTCATGGAATNCCNGATAANGTGCTGCTGGAATGCTTCTATAGGGGTCTTGGTCCCGNGAACAGAGGAGTGGCTGACCAACTTTCTCCGGGCGGTNTGATGCGACAACCCTATGNAATAGCAGCCCAACTCCTTGATCGCATGGCCAAGACCAACAAGGAGGCCGAGAAGGACCAAGAATGGGACGCACTA

>rnd-1_family-265#Unknown ( RepeatScout Family Size = 336 Final Multiple Alignment Size (possibly truncated) = 98 Localized to 2361 out of 4218 contigs )

CCCGAACTATCGTAAATGGTATGCAGATACCCTCCGTCATACTTTTGGGACATTGGTGCCCCTGCCGTCCAAAAACTAGAGCATATATACCCTTTATACTAACGGACATACACGTGTCATAATCTTATCCACCGATCCGACATTCGGATCGACGGATAAGATTGCGCCACGTGTCCCTATTTAGTCTTCCGTTAGAGTGAAGGGCATATATGCTCTAGTTTTTGGACGGCAGGGGCACCAATGTCCCAAAAGTATGACGGAGGGTATCTGCATACCATTTACGATAGT

>rnd-1_family-82#Retroposon ( RepeatScout Family Size = 833 Final Multiple Alignment Size (possibly truncated) = 98 Localized to 2361 out of 4218 contigs )

ACAAATACTTATAACTTTGCAGAAACATAAACAACAAAGTTTAAAACATGTACTCAAAAACAACAATTAATAACATAAGCANAAATTAATGACTGTAAAAAACATACCAGGATCTGTAACAATAGAATGAAACAGAAAGGAAAAAATCGAGCCCACTGAATGCACAGTGTCCCCTTAAGGAAATTATTCCCCTCTAGTACCCGAGGTTTAANGGAATATATCCTCCCAGGATAGAACGATCTTATTCACCAGTGTATTGATACAAAAACAATGGTGTCAGCGAGCCACTCAACGGGAGCAAAGTACACGAATATTTAATTGTGCAGAAGAAGAAGAAGATTCAGAATTTTCGTTGTTTTAAAATGAGAGGAAATCCCTCTATTTATAGACAACAAAGGGTAGTGTGAACAAATGTTTATTGTGCCTTATCGGAAAGGTCACAACCCTTCGGAAAAGTCGCAACCCTTCGGAAAGGTCACAACCTTTCATAAAAGTCGCAACTTTTCATAAAAGTCGCAACTCTTCATAAAAGTCGCAACTTTTCATAAAAGTCGCAACTTTTCATAAAAGT

>rnd-1_family-70#LTR/Gypsy ( RepeatScout Family Size = 905 Final Multiple Alignment Size (possibly truncated) = 98 Localized to 2361 out of 4218 contigs )

TCCAGCCCCGAAATCGAAGGATTTCTCCGTGGATTTCGTCACCAGGTATGTGGGATTTCACTAGTGGGTTCCTTTCACCCACTAGGTCCCTAGAATTCAGTCAGATTCTTGATTCTCCATATCTTGTTTAGACCTAGGGTTTCTAGAACCTTAGANATTCGTTGTGATTTAGCTGTTATAGTGTTCCNAATCGGATTATCATGTTTCTTGGAAANTTTGCTTAGTTCCTTGCATGAAACTCAGAACCCTAGTTGTGTAAATTCTCTTAGTTCATGAATTACACTTGCTAGGTCAGTTAAACAGATANNCATGCTTCAGATTTCGAATGTCACATTATCAGTATATAAATGTTGCATTCTCAGTTNGCATGTCAGTATTTTGAGCTATTCAGTATTTCAGCATTTCAGTTATATTGTGTCATATACATATTCAGTTGGGAGTAGACTTAGCACCGAGTTGGACTAGGGTTCAGCGTACCCTCATAGTCCCAGAACTACGTGCCCCCGTAGGTTTATAAGTCCCCTCTGTTGGGCATCAGATTTAGTGATCACGCCAGTCATGCCTTTATACCCCTGGCAAGGTATATTGGGTCCTCTCGATGGGGCGTATACATCGGACTCCACGTTTAGCTCACGTGGTTTTATGTCGGTTAATAGTAGCTCCCACAGTCAGACTCTTAGTGCATTTGACCAGGTTTTCAGTATATACTTCAGTATTCAGTCTCAGCATGTTATNTTCATGATTAGTACTTGGTCATTGCATTCAGTTTAGCTTTTCAGTTATGTTTCAGTATTTATATTCTTGCTCAGATTATATCATTGCTCAGTTATGCTTTGTTCAGCTTATATATATATTTGTTCAGCTTAGTATCTATATCCTGCATGCTCAGTACATTCCAAGTACTGACGCATACTCTGCGCTACATCCTTTCATGATGTAGGTTCAGGTACTCAGCATCCAGATCACGCTTAGATCGATTCTCGGTCCGCATTCAGCAGCTTCAGTGGTGAGTCCTCATACTTCGAGGACAATAGACATGCTTTTCATTTTAGTCTTTATTTTCAGTTTTCAGTTTTGTTAGAGTTAGCTGGGGGCATGTCCCAGCAACTCAACTCAGTTAGAGGCTTTT

>rnd-1_family-234#LTR/Gypsy ( RepeatScout Family Size = 397 Final Multiple Alignment Size (possibly truncated) = 98 Localized to 2361 out of 4218 contigs )

CAAGTTGAAGTGTCGAACAGAGAGATCAAGCAGATCTTGGCGAAGACNGTGAATGCCAATAGAACNGATTGGTCNAGGAAGCTTGATGATGCTCTATGGGCNTATCGCACCGCNTNCAAGACNCCCATNGGTATGTCTCCNTACCAACTNGTATNTGGGAAGTCTTGTCATTTGCCGGTNGAGCTAGAGCACAAGGCNNTGTGGGCGNTGAAGAAGCTAAATTTGGATTGGGGCGCCGCATCGAATCAAAGAGTGAATGACCTGAATGAGCTNGATGAGTTTCGNCTNAGAGCNTATGAAAGTTCAGCCTTGTACAAGGAGAAGATGAAGAAGTGNCATGATCGAAGAATCGAAAAGCGAGAATTCGNNGTNGGTGATCTGGTGCTNCTATTCAACTCNAGGTTGCGNCTGTTTCCGGGNAAGCTCAAGTCCAAGTGGACCGGGCCGTTCANAGTNACGCGAGTGTTCCCACATGGAGCGGTTGAGCTCGAAAATAAGGANGGAACGAGGTTCAAGGTGAACGGGCAAAGGNTGAAGNTCTATTTGGGGAATGCCGAGAGTGTGCAAGAAGTGATTGAGGCGTNNTATCTTGATGAAGTCTGAGTAATCAAGANGCCCGCGTCGTGCCGCGACGTTAAATCAAGCGCTGCTTGGGAGGCAACCCAAG

>rnd-1_family-277#LTR/Gypsy ( RepeatScout Family Size = 320 Final Multiple Alignment Size (possibly truncated) = 98 Localized to 2361 out of 4218 contigs )

TTTTTTATTTTTATTATTATCATATTTNTTTTACTCTTCTTGAGATGTCATCTGAAAAAGTATGTAGTGATAAGTGTTCTTGCAATTCTTGCNCTTCTGATATTATGGATTCTTATAAGCTAATTTTTTCTCAACAAGATAAGTTGTATGGGGACATGGAAGATTTATTGATACTNTGCTATAAATCAATATNTAAGCGCATGATTGATGAAGATACCTNTGAATTGGAATCTCTATNANTTAGATTTTGAGTTGAGTGCAAAGATTAAATCGTATGACACCCAACAATTTAGTAATAATATGTGTGAGGAAGAAAAAATACTTATAAGTCAACTTGAAGAGCTAAAAGTTGAGGGCCAATTGCTTGATCATACTGTTATTGATGTTGTCATTGTTGAGAAGAGTGCACTAGAGTTATGTAATGAAGTAGATAATATTATTTTTAAAAACTCTAGTATGTGTAAATATGAGGGTGTAAAAAATAATACAATTCATAAGTTAGGGCGCATTGGACCTCATTCCAATCATTTTTCTACATTATGTTTAGATGATGAGAAATTAATCGAGCCATCTGAACCAATGGANGAGTGTATGGATGAGGAACAAGGCGCCTACATTCTAGAGTTTNTTGGTCCAAGATCGGAGAATGACATTCCTCACNTAGGGNCCAAGAAGTGCANGATGCGACATCTATTGCTTGGNNCCTTTATCTTTATACCACCGCCCCTTGAGCGNAGTAGAAAAATTGATGCAAAGTTAGGGGTGCAATTCATANGTTCAAAGTGGAAGGAAAAGTGGTGAANTTGNTTGCGTCGTGCCGCGACGTTAAATCAAGCGCTTCTTGGGAGGCAACCCAATTAGTAGTGTAACTTCTTATTT

>rnd-1_family-395#Unknown ( RepeatScout Family Size = 188 Final Multiple Alignment Size (possibly truncated) = 98 Localized to 2361 out of 4218 contigs )

GTCCGTTAGTGTGAAGGGTATATATGCTCTAGTTTTTGGACGGCAGGGGCACCAATGTCCCAAAAGTATGACGGAGGGTATCTGCGTACCATTTACGATAGTTCGGGGGTATATTTGTCCTTTTTCCC

>rnd-1_family-314#LTR/Gypsy ( RepeatScout Family Size = 266 Final Multiple Alignment Size (possibly truncated) = 98 Localized to 2361 out of 4218 contigs )

GAACTGATGTTGACCAAGTGAAGACCACGGNCGACTTCACGGGCCGTGGTCCTCACGACGGNCCGTGGTGGCGCTCGTGGAGNTGAGGCAGTGTTGGGCCTCGAGAAGCTGAAGAGCAGNATTGACCAAGTGAAGACCACGGGGACCTTCACGGNCCGTGGTCCTCANCACGGNCCGTGGTGCGTGCCGTGAAGNCGANCCAGTGGNAGGCANCGAAGAGTCGAAAGACCTCGAAGCCCAAGTGAAGACCACGGGCGACTTCACGGNCCGTGGTCCTCGNCACGGGCCGTGGTAGCGCTCGTGGTGCATTNTCCAGTGNCCAAGCTTGAAGACCCACTTTCGGGAAGCTCCAAGTGAAGACCACGGAGGGCTTCACGGACCGTGAAGCCCTTGACGGGCCGTGGCGGTGGCCGTGAAGCNGCCCGACAGTTCAGTCCTACTTCGAGTAGGACTCTTTTTAAACATTCTATTTGAATTAATTTAGGTCGTTTTTNGTTTTTATCTCGTCTTATATTGGACGACGAAGAACNAAGTTTCGAATATTCTTAGCTTTCGGTTTTTGANTATTGAATATCGTTATCAGTTGGAAGTTCGGATTCGGATTNTCGGANTATCAAAGTGNTTGTCGATTTGCTTATTTTCGTAAGTACTTTCTCAACATGTTTTCATTNCATATTNCTGATCGTTTACGTGAAATCATGAGTGGCTAAATACCACGACTGGGGTTGTGGGATCTATGACGAAANTCTAAGTGCGATTTCGTAAAACGAGTAAAAGGCGGTCTGTGTTTACAACCTGTAGTTCTTAGCTTTCGTTATTCAT

>rnd-1_family-258#Unknown ( RepeatScout Family Size = 347 Final Multiple Alignment Size (possibly truncated) = 98 Localized to 2361 out of 4218 contigs )

TTTTTTTGCCATCTGATTCGGTATACAATTAGCCATTTTCGGTATACAATTAGCCATTTTATACATTTCGGTATAAAATGCGTTTGTGTTTGTATAAAGCGAGAGAAAAGTGTATATACAAATACAAATACATATATTTTCGTCCTATACACTTATAATTATACAAATACAGATCTTATTATACAAATTACAATGTATAAATGAATTTATACAAAACTGAACAATTTGTATAAAATTGGATGTNTCTAGCGAATTATACAAATCAAAAGCTCCATAGCAAACATAAAATTTGCTATGGAGCGCAATTATGCAAACTATAGCTATAACATACAAATATGATTTTTATGTTTGCTATATGTGAAAGTTG

>rnd-1_family-482#DNA/Harbinger ( RepeatScout Family Size = 135 Final Multiple Alignment Size (possibly truncated) = 98 Localized to 2361 out of 4218 contigs )

GACCCGTTTGGCCATAGATTTCCCAAGTAATATTTGGGAAAAAATTTGGCAAATAGTGTTTGTCCATACAATTTGCCATTATTTGGCAAATATTTTTGGCAAATATCCCAAATTCCCAAATACTAGTTTTTTCTAGTATTTGGGCCAAATCTCATTATTTGGGATCTTTTAAAAATTGAAATTTTACACCAAACTTTTATCTTTTACAAAAACACCCTCTATAGTAGTTGTTTGCGTTGTATTACATAATTTTTTACGTGAACACCAAAGTAGTGATGAAATATTCAGTGAATATGAAAGTGATGATATGGTTGTTGATGAAAATGACGAACAATCGGCTCAGGGTAACAAAGTCATGTGCTTCGTCTACTTCACGATGTATGGAATGATGCTTGTTGCACTCACTCCGAACTACCACATTGCTCCAGTGTCATGGACACTACTTGTTATTTGTTGCAACGAAGATCCAATTGACTTGTAATACAAACTTATGGTTAGTTTTGATAGTTTTTAAAACTTATGGGTATAAATCATATTTTTCTAAAAAAGTGAAATATGTTTCCCAAATACTATGGCCAAACACATGGTGAAATTTCACCCAAATTTTCACCCAAATAATATTTGCCAAAAATATTTGGAAA

>rnd-1_family-177#Unknown ( RepeatScout Family Size = 512 Final Multiple Alignment Size (possibly truncated) = 98 Localized to 2361 out of 4218 contigs )

TTAGGGGATAAGGGTCTGAAAAATACCCCAACTTTGGTCGGATTTGCTGTTGCGATACTAAACTTTCATGAGGACCTATTACCTCCCTAGACTATTTAATACCGTATTTTAAACATATATATTTGCCCACGTGGACATAAAAAATAATGCAAAATTATAAATAGTAATGTGTCCACGTGGGCACATATATACCTTTAAAATACACTATTAAATAGTTCAGGGGGTAAAAAATACGGTATTAAATAGTCTAGGGAGGTAATAGGTCCTCATGAAAGTTTAGTATCGCAACAGCAAATCCGACCAAAGTTGGGGTATTTTTCAGACCCTTATCCC

>rnd-1_family-122#LTR/Gypsy ( RepeatScout Family Size = 657 Final Multiple Alignment Size (possibly truncated) = 98 Localized to 2361 out of 4218 contigs )

TTTTTTCGGTTTGTCTACCATTGTTTGTATGGTTATTCTCNTTCAGGTCATGACCTCTGGGTCCAGGCTTCACTTTTTAGAGGTTGTGCTGCCCAATCTTTTTGCCTCACAAGTTTGGTTTAGCTAGTATGTTTCAGTGGGTAACTCGATGTATTAGGGCATCGGGTGCCAGTTACGCCCCTTGATTTCGGGGCGTGACAAACTTGGTATCAGAGCAGGTCGTATCCTAGGGAGTCTACAAGCCGTGTCTAGTAGAGTCTTGTTTATGGATGTGTTGTGCACCACATTTATAANCAAGAGGCTACGGGGCATGTNGGAATGGTTACCTTTCTTTCANTTCTTAGATCGTGCTATAGAGCGAAGGTATAAGAACGTTTGAAATCTAAATCGTGCTTTATGTTTCTTATCTAGACAGAGCTACACTCGCTCCGGATACGGCACATCGAGAGATAGGTACGGATCCAAGGGAAGGTACTAGCCGTTCCCCACCGGGTCAAAGAGATAGATTTCCCTCGGAGGCACAGGGTGGATCTCCAGTACCCCTAGTACCAGCCTCCCCGGCACCTGTTGAAGCCNCGAGGAGATGCAGTACCCCCGGCCTCACCAGTTCCATTGGTACCTGAGGAAGCTACGGACACAGGACCTCCAGTACCTATTGTTCCTCCACCAGAGATCTCTGGGGAGCAGGGNNTGAGGAANGCAGTCCAATTGTTGACTAGTATGGTTGCTGGCCACGGCCAGCGACAAGNGGCAGTTGCAGGTANTAGNGGTGCAGATAGAGCGGCCGGNAAGTTCGAGGGTNCGTGANTTTCTTGACTTGGACCCTCCATCGTTTACAGGGNCAGATCNTAGTGAGGACCCNCAAGACTTTATNGATCAGATTCATAGGATCTTCGGAGTCATGCATGCCTCAGCGACNGAGNCGGTCGAGTTAGCAGCATTTCGATTACGCGATGTNGCNGTATTGTGGTACGAAGCTTGGGAGAGATCTAGGGGAACAGATGCATCTCCACCCACNTGGGAANAGCTTCACAGAGGCTTTCNTNGACCACTATTTGCCNCGGGAGATTNGGGANGCCCGNGCGGACCAGTTTCTGAACCTCCGACAAGGNAGCATGAGCGTGNGAGAGTACGGNCTCAGATTTGATTCNTTGGCTAGATATGCACCAGCATGTTGTNGATACGATGCGNGATAGGGTNCGTCGATNTGTGGGANGGNTTGACTCAGACTATTATCGGAGNTTGTTCTACCGCTGCTCGCTAAATGATGATATGGATATCTCGCGGATNCAGGCCTTCGCGCAAGGCATAGAGGATCGCCAGACGATCTACGGTACACGAGNGAGGGTCGAGAGAGAGACAGNNNAAGAGGGCTAGGCCCGCCGGTTCACAGGGAGATTTTCAGGGTGGTCCCAGGCCCCGATATTCTANTAGGCCACCTAGACCTCCACCACAGCAGTTCCAGGGTAGTAGATTTGATCGTCAGGGACAGTCAGGCCCAGGTGAGGGTTCACGAGCATCAGGCTCGTCAGCAGAGGGGTTCAGGCCAGGCTAGGACANCCCCGCCGCGTTGCGCTACTTGCGGTAGGACGCATTTCGGGAGGTGCAGACAGGGTTCCACAGGTTGTTATTCTTGTGGACAGGAGGGGCATGGGTGGAGAAACTGCCCGACCATAGGTCAGGGTGGTATAGGTCAGTCGACCAGGATCAGCAGTAGGTTCCTCCTCATCAGCGCAGTCTACAGGGCGTGGACCCCAGNCTACTCAGGGTCGNGGTAGAGGCGGAGGTAGAGAGGGAGCATCTAGTTCTAGTGGTGGTCAGAATCGCACATATGCACTAGCCGGCCGACAGGACTCAGAGGCATCGCCGGATGTTGTNACAGGTACATTGACCGTCTNTTCNCATNNTGTTTATGCNTTGATAGATCCTGGNTCTACNCTGTCNTATGTTACTCCATTTATTGCTGGGAAGTTATGCANAGGAACACCAGAGTCATTAGNNCGACCNTTTGNNGTGTCTACACCGGTTGGTGAGTCTATTATAGCTAGTC

>rnd-1_family-321#LTR/Gypsy ( RepeatScout Family Size = 260 Final Multiple Alignment Size (possibly truncated) = 98 Localized to 2361 out of 4218 contigs )

TGTAACACCCCGACTTTTCGAAACGTCTAAATTAACTCGTATCTTCGTGGAAAGACAAGGAGGGTGAGTAATAAATTAAGAATGATGTGGTATGTCATATTTTAAGTGTTCAAGGGTTGTATCTCAAGTTTTGAAGTTAGGTAAGTGGCAAAATAAAAGTTGGCGAAAGTTATCGTAAGTTCCTTTTTAAAGATTTCTCTGAAATTTGGGTCAAATGTCTCGGACGTTTTCTCCCAATATATAAAGAGTTAGAGGGCCTACGACCCATTAAATCGAAGGCCTACGAGTCTAGTTTNCAACGCACAAAACCCCGTATCAAACCGACATCGGAGTAAAGAGTTATGAGGGTTTTACTAGGGACTGCCGGGGCAGAATCCGGGTCGGGTAAAAAATGTGGTATGTCGGCTTACTCAACGTTTTAAGCCATGAAAACATTTCATTTCACTTCCAAACCAGAGAGAAAGCTTAAGAGAGGGTTCCTATGGAGTTCTTGCATGATGAAGGTTAGTTTTTGACGATTTCTACCATTAAACGTTGCCCCCGTGCCTAGAAACGTGATCTCTACACGTGGCAATCGTTTTTCCCTTCTATTAGCTGCGTTTGGAGCTAGTTTTTGAAGATATAAATTTGTAGTTATTGGTGTTTCTTCAAGGTTTACACTTGGTTAACTAAGGTAATCATCTCGGGACTCTTTTATGATTGTTTTTATGAATTTCTACGGACGTTTCGACAAGTTGGGGCAATATGGCAAATCTGCCGATTTAAGATTAATTATGAACTGTTTATAGGTGCGTACCAGCTGTGTATATATAGTGTTTGCGAAGCTTAGGACGTAAGGAACAACTTTCGTGAAGGAACTACGGTCTTTCGAGCGTTCATTGGAAAGGTATGTTAAGGCTATTC

>rnd-1_family-375#Unknown ( RepeatScout Family Size = 211 Final Multiple Alignment Size (possibly truncated) = 98 Localized to 2361 out of 4218 contigs )

GTCCCTGGCGCTCTGAGAGGCGCGGGGCGCCAGCCCTCAAACTTCAGAGGGCCTGCTGGGGCGCTCTGGCNGGCGCGNCGCCCCAAGGGGGAAANTTCAGAGCCCCTTTTGGGGCGCG

>rnd-1_family-392#LTR/Gypsy ( RepeatScout Family Size = 191 Final Multiple Alignment Size (possibly truncated) = 98 Localized to 2361 out of 4218 contigs )

AAAGAAAGGGTGAAAAGCCAAGGCAAAAAGGCGATGGAANGGANCAANCGGCGGATCGCCGANCGGNTCGGCGAGCCCGACTTACTTCGCCGAGTGGCTCCGNAGCTNAAATTTTGGGAACGTATAAATNCAATTTTGAAAAGAAGAGAAGGAGTTCCCACATTGTCCAGTTCTGGGTTTTTGATCCTNAACATTCT

>rnd-1_family-192#LTR/Gypsy ( RepeatScout Family Size = 484 Final Multiple Alignment Size (possibly truncated) = 98 Localized to 2361 out of 4218 contigs )

ATTATANCCCAAGACCCCAAATCAAACCCAACTACTTTCCTCTCCAAAATTTTCTCTCTAGAAACCTCCATTGAAGAAGAAGAAGAAGGTCGAGCTAGGGCTCGAAGAGCAGGCTTTTCTNCTCAAATTCGTGNGGAATTCTTCACCTAAGGTATGGTAGTCTTCATCCATGGGTAGCTTTCACCCANGGAGCCCCTTCAAAACTCGATTTCAAAGCTAGAAATCCCCAAAAGCTAGGGTTTCGACTCCAAGTCATGGGTTCCTTTCAAAAACGTTTTCAATGGTTGAATTATGTTNTATTATGATTGAATTGATGATTTTATATGGTTTTATGNTGAATTCCCCAAGAATCCATGAATTCCCCAATCCTAGTTATGGTTCTTCTTTACATTGTGGGTGTTGTTGTANGTTGATCCAATTGAGTGGATGTGAGTTGNTTATGATGTAATTGCATGATTTTAGTATGATTNTACATGTATTCTTGATTAACCTAAGATTCTAGGGTGAAATTGATATAGTTTAGGGTTATGCCATGAAAGAGGCAAATTGAGGGCTTAGATGTGATCTTCTAAGATTGATGAACTNCTTTATGAATTGCCTNAGATTAGANCACCTAGACATGAATTGCTTAAGAGTAAAGCTTGTAGACATGAATTGACTNGATTAGGGCTTATATGATGAATCATGGTTGAGTAGTATTGAGATTGAG

>rnd-1_family-250#LTR/Gypsy ( RepeatScout Family Size = 360 Final Multiple Alignment Size (possibly truncated) = 98 Localized to 2361 out of 4218 contigs )

GAAAAGTGGAGAAAGTTGGAAATGATGAGTCGTTGTGTAATGTCAAGGAGGGCGGAAAGTCACTAAGAAGTACCCAAATGTACCATACCTGACCCTGAGCCTACGTTACAACCTAAGAAAGTCCTNTAGTGATCTCTAGCCAAATGGAGCTGAGTCNANGTTGATTGAAAATAAGGGCAAGCCTATGGTGTGAGCACTNACTGTGCATGAGGTATTCTTTGTGAGCGTGAGCGTTGNATCTTTGTCCCTTGTCTTGTTTATATGTGTGTGAATATGGGAATCTTCTTTNTTGTGAGGGCATTTAAGTTGAAAAAGTGGTGATTTTGAAAATTCCTAATTGAGTCAAGTGAGCATGTGAAAGAGTTAATGAGTTGAGTCATTTCTTGAGTTTGAGTTGTTGTCACTTAAATGATTCTTGGTTTTTGAAAATGTTTCATGAGAATGAGAGGATTTCTTTTGTGAAAAGTTTGCATGTGGACTATTTGTTAGTACCAACCGAAGCCATTACCTTTGTGCTTAATTGAGAAAGGGAAATTGATTTGTGGTGTGTCTTGAAATAAGAGCCTCTTGTGTTTTTGATTTTCTTTACTCGAGGACAAACAAAGTTTCTAAGTTGGGGGTGTTGATGAGTGGTGGATTTCCACTCATTTGAGGCTCTTTTCGTAGTTATTTTGGCTTGCTTTAGTTTAGAATTGGTGCTTTTAATGTGGTTTTCTTCATATTTGCANGAAAATGAAATGGAGAGTTGATTTAGGATATTTGAAGAAACCAAGTGAACTTGAGGCAAAAAGAGGTCAAGAGCTGAAAAT

>rnd-1_family-118#LTR/Gypsy ( RepeatScout Family Size = 667 Final Multiple Alignment Size (possibly truncated) = 98 Localized to 2361 out of 4218 contigs )

CTCCTCAAGCTCCGGTCGATCCTTTGGCCGAGCAAGTGACTAATGCGGAGTTTAGGGCTGCTTTTCAAGTGTTGGCTCAAGCCGTGACGGCCCAAGCCAATAGGGAGGTTGTGGCTCCCGTGAACCCAAATGTGGGTACGACGGCGNCNAGAGTGAGGGACTTCACTAGGATGAATCCTCCGGAGTTTCATGGTTCCAAGGTTGAGGAAGATCCTCAAGAGTTCATTGATGAGGTNTATAAGGTGTTGGCGATCATGGGAGTGACGCCGGTGGAAAAGGCGGAATTGGCCGCTTATCAACTNAAGGGTGTTGCTCAAGTTTGGTTCAACCAATGGAAGGAAGAGAGGNCGGTNGATGCGGGTCCTCTNGATTGGGAAAAGTTCAAGGNTGCTTTTCTTGATAGGTTCTTTCCCCTTGAGATGAGGGAGGCNAAGGTGCTTGAGTTCATTAACCTTCGTCAAGGAAATATGAGTGTGAAGGAGTATGCTTTGAAGTTCACNCAATTGTCTAAGTATGCTCCNACTATGGTTGCCGATCCTAGGGCAAGGATGAGTAAGTTCGTTTCGGGTGTGTCCGAAATGGTGGTCAAAGAATGTCGTACCGCCATGCTCGTTAATGANATGGACATTTCTCGTCTNATGGTNCATGCCCAACAAATTGAAGAGGAGAAACTTAAGGAAAGGTCTAGGGAGGCAAAGAGGGCNAGGACCGGTGATGGTGACTTCTCNCATTCAAGGTCCGATGGACATGGTCGTCCTAGGTTTCGACAAAGGTTTTCCGGTCAAGGTTCCTCCAATGCTCCTCCTAAGTTCAACAAAGATAGGGTGTCTAACCCTAAGCCTCAAGGAGGAATGGTAGTGGATCTTCATTGCCTACTTGTGCTAAGTGTGGAAGGAAGCACGAGGGTAAGTGCCTAGCCGGTNCNGATGGTTGCTTTGGTTGTGGNAAGAGCGGATCACAAGATGAGGGATTGTCCTTCGGTTGCTAAGAATGAAGGAGATAATCGTCGNAGGGCTCAACCCTACCCTTCATCCGGTCCNAGTGGT

>rnd-1_family-551#DNA/PIF-Harbinger ( RepeatScout Family Size = 110 Final Multiple Alignment Size (possibly truncated) = 98 Localized to 2361 out of 4218 contigs )

AGGGGTCGTTTGGTAGGGCGTATTAGGAGAAATAATNCATGCATTATGTATGGTATTATTTAGTACCNTGTTTGGTAGGAATTTGGGCCTATGTATAACTAATGCATGCATTAGTTATACACCCTACATGCGTATTATAGGGTGTATTACTAATACCNTCNATTTGNAGGTATTAGTAATACATNGGATNTAATACCATGGGATTAGTCTATGTAAAGACAAAANTATCCCTCAAATCATTTTAATTATTTTATTTATTTTCTTGATTTTATGTTTTATATTTGTACTAGTAAATTTATTTAAATAAATTAGCTTACAAATTATTTAGAGAGAGTTGTTTGTTACTAAATAAATCCAATACAAATCAATACAATATTTGAAATGTGAGTTGTTTAACATTTACTAATTAAA

>rnd-1_family-308#LTR/Copia ( RepeatScout Family Size = 272 Final Multiple Alignment Size (possibly truncated) = 98 Localized to 2361 out of 4218 contigs )

CTTTTGGAAAGAGGCTGTCAATAGTGAGATCGANTCAATCTTAAGCAATCATACTTGGGAGTTGGTTGATCTTCCTCCAGGAAATAAACCTTTGGGTTCAAAGTGGATCTTCAAAAGGAAAATGAAAGTCGATGGAACTATTGACAAATATAAGGCNAGACTTGTTGTCAAAGGCTTTAGACAAAAAGAAGGTCTTGATTATTTTGATACATACTCGCCAGTAACNAGGATTACATCNATTCGGATGTTAATTGCGCTAGCTGCGGTATATGATCTTGAAATCCATCAAATGGATGTGAAAACCGCCTTCTTAAATGGAGANTTGGAGGAAGAAATTTACATGGAACAACCCGAGGGNTTCGTAGTTCCTGGTAAAGAAAAGAAAGTGTGCAAACTTGTTAAGTCACTTTATGGACTAAAACAAGCACCCAAACAATGGCATGCGAAGTTTGACCAAACCATGTTGGCAAATGGATTTAAGATNAATGAGTGTGATAAATGTGTTTACATTAAAGACGTTNCGAATCACGAGGTCATTGTTTGTCTATATGTTGATGACATGTTGATCATNAGTAAAGACATTGCCGATATAAATGCTACTAAGCGTATGCTNGCTAGTAAGTTCGATATGAAAGACCTNGGAGTNGCCGATTTGATCTTNGGAATNAAAATTCACAAAACTCCTNANGGTCTAGCATTGTCTCAATCTCATTATATTGAAAAAGTACTTGANAAGTTCAAGTATTTGGATTTCAANANTGTCGAGACTCCAATTGATGTGAGCTTTGCATTTCNNAAGAATGAAGGCGAAAGTGACTCTCAATTGGANTATGCTAGAGTATTGGGAAGTTTGATGTATGTCACGAATTGTACGCGACCAGATATAGCATGTGCTATTAGTAAACTGAGTCGGTTCACGAGTAATCCCAATCAAACTCATTGGATGGCAATGAAAAGAGTTTTGGGGTATTTAAAACATACTCAAAACTATGCTTTGCATTATAACAAATATCCNGCNGTNNTTGAAGGATATAGTGATGCAAATTGGATCACCGGGTCAAATGAAGTAAAATCCACGAGTGGATATGTNTTTACTCTTGGTGGAGGAGCAGTCTCTTGGAAATCGTCCAAACAGACATGTATCGCTCGCTCTACAATGGAATCTGAATTTATCGCTTTAGATAAGGCCGGTGAAGAAGCTGAATGACTCCGGAATTTCTTAGAAGATATTCCGTTTTGGCCCAAACCNNTGGCACCTATATGCATACATTGTGATAGTCAAGCTGCAATAGGTAGGGCATGGAGCGTTATGTATAACGGAAAGTCTCGTCATATACGACGNAGACATAATACCGTNAGANAACTACTCTCTAGTGGAATTATCACAATTGACTATGTAAAGTCAAAGGATAATGTGTCGGATCCACTTACAAAAGGCCTAACTAGAGAGGNAGTTGAAAGATCATCNAAGGGAATGGGNTTACGGCCTAGGACAAGTCATCGTGGCGGTAACTCTACCTAGNAGACTGGAGATCCCAAGAGCTAGGTTCAAGGAGATCAAACAAAGTCGTGACTGACGGTTCGACATTGTCAAA

>rnd-1_family-116#LTR/Gypsy ( RepeatScout Family Size = 678 Final Multiple Alignment Size (possibly truncated) = 98 Localized to 2361 out of 4218 contigs )

TGTNGCCTTGTCTTGTGTAGCCTTAAGGATCACTTGGGTGTGGTATGGAGAGGTAGTATGGGCTGCCNCACCATGCCTTACTCAGGTGNGNCTTGGGGTTACATTTGAGGTGAGTGTCTTAGTGTNATGATGTGACTTATGTAATGTTTATGTCTCTTATGTATGATATTGTGTAAAGGTGGAAAGGATGAATGGTAAGTTCACTTTTGGTGACCTTAAGGTGGTGCCTTAGTGTGGGTGGTGGTATGGGACGCTATCCATACATTGCACAAGGTATAGCTTGAGGGTTACTTGAGGTGAAGGCTCAAGGTGAAGGTAAAGGTTTATATGTTGATTATGTTCAAATGTGAAGTATATGACTTGTATGTTGGTTGTGACTTGTATTATGCCTCTTATGCTAATGTTCATGAAGTTTTACNAAAATGGCATAAAGCATGACTTTCAACAAAATGTCCCTTTTAAGCATGTTTTTGCATGGTCATCATACTTAGTACATTTNTGTGTGCTAACCCATATTTCTTCTATTTTTACTACAAGTGTAGGTTCCGGCAAGTGATTGNTTCTGCTAGTTTGAAGTGCTTGGATTGANTTTCTTCCAAGACNTTGGTATGTCCTCATAGATTCGAGGACAAGACTTTTATGTTTCGTTTCTTCATGTAATAGACACTTANTAGTTTCNTTTCGATTGTAAAGGGCCGTGTCCCTACTTTGTTNAGGTCTTGTCTAAGATGGCCATGTGAGACTTAGACTTCCGCTGCGTNTTTTAAAGATTTTTTCAAATGTTGATGACTTTTGAAGTCTTATGGTTTTATTGAATAAAAGTTTTAATTCCGTANTGATTTCTATGATCTATGCGATGAACGAATGCTAGAGGCTTGTATAAG

>rnd-1_family-30#LTR/Gypsy ( RepeatScout Family Size = 1247 Final Multiple Alignment Size (possibly truncated) = 98 Localized to 2361 out of 4218 contigs )

AAGCAAGACCAAGACCCTATTTTGCTTGAATTGAAGGCAAATGTTCATAAGCAAAAAGTANTGGCTTTTGAACAAGGGGGAGATGGTGTATTGAGGTATCAAGGTAGATTGTGTGTACCAANGGTGGATGAACTCCAAGAGAGGATCATGGAGGAAGCTCATAGCTCCAGATATTCCATCCATCCGGGTTCCACAAAGATGTATCGTGACTTGAGAGAAGTNTATTGGTGGAGTAGTATGAAGAAGGGCATTGCAGAGTTCGTTGCTAAGTGTCCGAATTGCCAACAAGTTAAAGTAGAGCACCAAAGGCCCGGTGGTNTGGCTCAGAATATAGAACTTCCGGAATGGAAGTGGGAGATGATCAATATGGATTTCATCACAGGTTTGCCAAGGTCTCGCAGGCAGCATGATTCTATTTGGGTGATTGTCGATAGAATGACNAAATCAGCCCACTTTTTGCCGGTAAAGACTACCCATTCAGCAGAAGATTATGCCAAGTTGTATATTCAAGAGGTAGTNAGACTTCATGGAGTTCCGGTCTCCATTATTTCAGATAGAGGTGCGCAATTTACCGCACAGTTTTGGAAGTCTTTCCAGAAAGGCTTGGGTTCGAAGGTGAACTTGAGTACTGCCTTTCATCCTCAGACAGATGGNCAAGCAGAGCGCACTATTCAGACCTTAGAAGATATGTTGAGGGCNTGTGTGATCGATTTCAAAGGTAATTGGGATGATCACCTACCTCTCATTGAGTTTGCTTACAACAATAGTTACCACTCTAGCATCCAAATGGCTCCTTATGAAGCTCTTTATGGGAGAAGATGCAGATCTCCTATTGGATGGTTTGAAGTTGGTGAAGCTGGGTTGATAGGACCAGACTTAGTTCATCAAGCTATGGAGAAGGTGAAAGTGATTCAAGAGAGGTTGAAAACGGCGCAGAGTCGTCAGAAATCCTACACTGATGTTAGGAGAAGGGAGTTAGAGTTCGAAGTAGATGATTGGGTNTACTTGAAAGTTTCACCCATGAAGGGTGTTATGAGGTTTGGTAAGAAGGGGAAGCTTAGTCCCCGGTATATTGGNCCTTACAGAATATCCAAGAGGATTGGCAATGTAGCTTATGAGTTGGAGCTACCGCAAGAGTTAGCAGCGGTTCATCCGGTATTTCACATCTCCATGTTGAAGAAGTGCATGGGCGATCCTTCACTGATCATACCAACTGAAGATATTGGGATCAAGGATAGCTTATCTTATGAGGAGATTCCCGTTCAGATTCTAGATCGCCAAGTTCGCAAGTTGAGAACNAAAGAGGTAGCATCAGTCAAAGTCCTTTGGAGGAACCAGTTTGTTGAGGAAGCTACTTGGGAAGCTGAGGAGGATATGAAGAAGAGATATCCACATCTCTTCGAATCCGGAGAAATTCCAGATCAAGGTACTAATTCTCTTCTTAGTACTCTTTAAATTATGAGTTGGCATGTTGTTGTTTGCATTTGCTTGTTGGGTGTTTGAGCTGAATGTTAGATGTTACACCCTTAGCCTAGTAAGAGTAATCTCATTCGAGGACGAATGTTCCCAAGGGGGAGATATTGTAACATCTCGCAACTTGAAANAACTAGAAAGAAGCTAAGAATTGGAAATAGTCATTTTTGGAAAGAATAAGAAAATCTGGAAAATTGTTTAAGTTAGGTAAAGTGAGTTTTTGGTCAACTTCAAACGACCATAACTCCTAGCTCAGGATGAGTTAGGTGTANTTCCAGATATGGTAGGAAATATCTTGGAATNATCTTTCCAACGCCGCCGAGTTTGCGCGATTCCGAGTTCGTATGAGTGAGATATGCCCNTTGGAAGTTGGGCTGTTCGAATAAGGAAAGTCCAATCCGGATTTTGGAAGGGTANTTTGGTCTTTTCCTTACCCAATTANTTTAATTCGTTTTTAGGAATTTAATTGGGNTAAANTCAGATTTTAGTCAGTTTTAGAAAAGTTAAGTTACGCTAGGGCTTGGAGAAGAGGAGAAAAGAGGAGAAAGGAGAAGAAAGCCAAGATTCGTCAAGTTCGTCGAGNTTAGCTTGTGGATTTCGTCGGGGGTGATCCCTACAAGGTATGTGAGATCACATAGCGTTGGGTTAGTTCACCCACGCGCCAA

>rnd-1_family-462#LTR/Gypsy ( RepeatScout Family Size = 143 Final Multiple Alignment Size (possibly truncated) = 98 Localized to 2361 out of 4218 contigs )

TGTGAGACCCCGTGGTTCTTTCGCATAATATGCGTGGCGTAGCATGGCATGGTTATATGAGGCGTATATGACTTGGTATCGTATCATTGGAGANTAAGATTGCAAGTGGGTGGCAAAAATCAAGGAACTAAACTAAAGTTNTGGGTCAAAGGANCCCCTCAAACAAAGTTCGNGATNAAAGAAATGGCTCTTGTAACACCCCGACTTTTCGAAACGTCTAAATTAACTCGTACCTTCGTGGAAAGACAAGGAGGGTGAGTAATAAATTAAGAATGATGTGTTATGTGATATTTTAAGTGTTCAAGGGTCGTATCTCAAGTTTTGAAGTTAGGTAAGTGGCGAAATAAAAGTCGGCGAAAGTTATCGTAAGTTCCTTTTTAAAGATTTCTCTGAAATTTGGGTCAAATGTCTCGGANGTTTTCTCCCAATNTATAAAGAGTTACGGGGCCCACGACCTATNAAATCGAAGGTCTACGAGTCTAGTTTCCAACGCACNAAACCGTTTGTTCATACGACTTCAGAATAGAGAGATATTCGCGTTTTCGCGAGACTGCGCAAGCAGGCACGTGAGGTGGGGCCCTAGCCCCTANGTCGGTGGAACTTTATAANATATCATCTTCCTTCGTTTTNTCTCATTTTTTCGACCAAAATAAGAATAAAACAGTCCTAAATCCTCTCTAAAGGTTCCCCTATCATCCAAGCAAAGATCTACAAGATTTCAACGTGGTTTCACCCTCGGAACGTTCATAGAGATTGTGTTACTTCTTTCCTCCTCGTTGGTTGTGATTTGAAGGATTCTTCGTGGTGAAGGAGTCTCGTTTTTAGGTGGTGTTGCTGCTGTTTGGAGGTAATAATTCTCACCCTTCATTTTATTGAGTGAGTCCAAGTTATAGCTATGCTATATAAGCAAGAACACGAATAGACGGCTGGAAAAATCGTATTTGGATAGTTACGTGATGTTGGGCTGTTTTGAAGTATTTTCTGATGTTGTTGTTGCTGGAATTTTCGGAAATGCTTTGTNTGTGTGTGGTTCTTGTTGATATGAGTCTGGAAGAGAAGAAGAAGTATAGATGGGTANTTAATTGTAAGATAAATTAGTGTAACGTTGGTTCTATTATTGCATTGCAATTTTCAAATTGTTTCTTCTTGGTTCAACGTCTTGCGTTTGAGGAGACTGTTTGTTATTGACGAGACATGCTAAAATACTATAAGCTCCATCAAGTTTAAGTTAAGGTTCTGCCCTTTTANTCTCTTATTGCTTATGAAGAGAAAGACGTGATTCCAAACGTAATTATTGGACGAACTCACAGGATGGTGATCGGAAGCGGTGAGTTCGAGTTATTATGGTTATATTGAACAATTATAGGACTGTATTACATATATTTTTTCTGGCTTTAATGGTTTGAAGACTCGTGGTTGGGATTTGGTTGTGGTTATTTTGGGGTTGGAAAAGAGGGGTGTGGCGATACGCCGAGTTTTTATTGTTGTGCAGGCATACTCTATTTGGTTGTTGTTGACTATTGCGTAGGCCTCCACTTGTTAATAGCTGCTGCAAAAATGTATGTTACGTCATTAATTTGGGGTTGTAATTTGGAGAAGAAAAGGTAATGAAGCATACATTTAAAAAGGGTCCGGCGAGCTAATCACTCTGCTAGTAAGCTCTAAATTGCTACTTTGACCCTGACGTGTTAATATTCTGCCTGTAATGGTTGTGTGACATGATTTTCTGTAGATTACATACTGTAAGGAAGGGGTACAATCCATATTGATACAACAAGACCCAGAATTGAAAAGGTATGTTAAGGCTAAGCTACTGTCCCTCATTTTAGCACCACTCCTGGGAAATTCCTAAAACGCCGAATGTGATATTTTGCTATTCTCTTGCTAGAAAGACCTTCGGTGAAAGGCATTGGGATCGCAGCTGGATTATTTTCGTGACTCCGTTATTTTGATATTCCACTCGTTCGGNTAAATAGGGTATTCGACGTCTNCACGTCATTTTGTCGGTTCCCTTGACTATATGTCCGCGCGTACGAATTCTTATTCTTCCTTGGGTGNTGTGACTTAAAAATGTCACGCGAGACCCNTACTAACTTGCGACCAGCCCTCCTTTCTCCGCTAANGTTATATTTTTGAAAAATCTTTAAAGTAGCACGTCGATCTCCAAAACTCTCAAATATAANTCANATGTTTAAACTACTAGAGCACTTGGTTTTCT

>rnd-1_family-498#Unknown ( RepeatScout Family Size = 129 Final Multiple Alignment Size (possibly truncated) = 98 Localized to 2361 out of 4218 contigs )

TTTTCAAATAAACCATATGATTTTTCCAGCATTTGGCATGAAAGTAATAATCATAAAAGTAACAATCATTTTTCTTGCAAGTTTAGACACTAGACACATTCAAAGGAAAAAGTGTGAAAGTAACAATCGTTTAACCATTAACAATAAAAGTAGTAAATTACTATAGTCTTAATGGGTTATCGGTTTACCCAATAACCCAATATTAAAAACCGANACCGAACCGATAACCCGATAATTTTTTTTATAAAACCATTAAAAANCCGTTAACCCAATAACCCAATANCAATAAACCAATAACATTTTTTTCGGTTCGGTTTATCGGTCGGTTCGATTTTTGCACACCCCTA

>rnd-1_family-280#Unknown ( RepeatScout Family Size = 318 Final Multiple Alignment Size (possibly truncated) = 98 Localized to 2361 out of 4218 contigs )

GGGAAAATTGTATATAATAGCAAACTATTAATTCAAATTAAATGCTATAAATATAGTTTGATTTAATTGTACCCCGTAGCAAACTGTTGCTATTTCGCCTCTCTCCCGGTGAATCTCGCTCGCCACTCTCGTTCTCTCCCTCGCCTCTCTCGCTTTTTATACAAACGCAAATGTATAAAATGCGTTTGTGTTTGTATAAAGCGAGAGAAAATTGTATATATACATATATTTTCGTTCCCCTCTCTCCCCTCTCCCAGATCTCGCTCGCCACTCTCCCAGATCTCGCTCGCCA

>rnd-1_family-76#LTR/Gypsy ( RepeatScout Family Size = 858 Final Multiple Alignment Size (possibly truncated) = 98 Localized to 2361 out of 4218 contigs )

TGACTTATGAAATGTTTATGTCTCTTATGTATGAGTATTGTGTAAAGGTGGAAAGGATGAATGGTAAGTTCACTTTTGGTGACCTTAATGTGGTGCCTTAGTGTGGATGGTGGTATGGGACGCTATCCATACATTGCACAAGGTATAGCTTGAGGGTTACTTGAGGTGAAGGCTTAANGTGAAGGTAAAGGTTTATATGTTGATTATGTTCTAATGTGATGANATGACTTGTATGTTGGTTATGCTTGTATATTATGCCTCTTATACTAATGTTCATGANGTTTTATCAAAATGGCATAAAGCATGGCTTTCAAACAAAATGTCCNTTTTAAGCATGTTTTTGCATGGCCATCATACTTAGTACATTNNTTGTGCTAACCCATATTTCTTCATTTTTACTACAAGTGTAGGTTCCGGCAAGTGAGTTGNTTCTCTCTAGTTGAAGGCTTGGATTTGAGNTTCTCCAAGACTGTGGTATGTCCTCATAGATCGAGGACAAGCGTTTAGATGTTTCATTTTCTTTCATGTAATAGACTATTGTTAGACTTTTCGATTGTAAAGGGCCGTGNCCCTACTTATGTTTCAGAGTTGTTTTTAGATGGCCATGTGAGACTTAGACTTCCGCTGTGTTTTNAAAGGTTTTAAATGAGTAATTTTGATTGTATGGNTTTAAATAAAGTTTTAATTCCGCACTATTTCTATTATCTAATGCGATGAATGCTATGAGGCTTGTATGAGACCCCTTCGGGGTCGAGTACGCCGTGTTACGNCCGGGGGGTGCTCTCGGGTCGTGACA

>rnd-1_family-148#LTR/Gypsy ( RepeatScout Family Size = 590 Final Multiple Alignment Size (possibly truncated) = 98 Localized to 2361 out of 4218 contigs )

TTCCATGCTCTTTGATTCCATTACATGCCTTCAAGGTGTTTGACAAAATGTCCAACTATGGAGAATTGATGAATCGATGCTTTAAAGCTTGAGTTATGAGATGTATGGAAATCCAGAGATGTGTTGATGACAAACTAAGCTTGTGTATATGTTCATTCCATACGTGAGATTGCATTAGAGCCTAATGGGAATTTCCTATATAAAGTGTTGCATGACCATGCCCGGTCCGGGGGAAAAGAACCGGACAACCATGTGAGAGGTTTATATCTCACCACCTAGGATGCTTGGGGTGTGACCAACATCAACCATGTGAGAGGTTTATATCTCACCACCTAGGATGCTTGGGGTGTGACCAACATCAACCATGTGAGAGGTTTATATCTCACCACCTAGGATGCTTGGGGTGTGACCAACATCAACCATGTGAGAGGTTTATATCTCACCACCTAGGATGCTTGGGGTGTGACCAACATCAACCATGTGAGAGGTTTATATCTCACCACCTAGGATGCTTGGGGTGTGACCAACATCAACCATGTGAGAGGTTTATATCTCACCACCTAGGATGCTTGGGGTGTGACCAACATCAACCATGTGAGAGGTTTATATCTCACCACCTAGGATGCTTGGGGTGTGACCAACATCAACCATGTGAGAGGTTTATATCTCACCACCTAGGATGCTTGGGGTGTGACCAACATCAACCATGTGAGAGGTTTATATCTCACCACCTAGGATGCTTGGGGTGTGACCAACATCAACCATGTGAGAGGTTTATATCTCACCACCTAGGATGCTTGGGGTGTGACCAACATCAACCATGTGAGAGGTTTATATCTCACCACCTAGGATGCTTGGGGTGTGACCAACATCAACCATGTGAGAGGTTTATATCTCACCACCTAGGATGCTTGGGGTGTGACCAACATCAACCATGTGAGGGGTTTATACCTTGCCACCAGGGTGTCCGGGTGTGAACGGCATCCCCCATCCTAAGTTGGGGTTATAGATTGGTTGGATCATGCATACACATATGATATCTACTATTAGTATAGAGTTACTTAAACATGTTTTGACTTCACTTTGATCATGCATCCTCATATTGTTATTCATAATGCCTATGAAACTATTTCTTGTATTTCGATTGTGTCCTTGTCTATTGTTGCGTTGCACCCCGCATACTTAGTACATTCAAACGTACTAACGCATACTCTATGCCTACATGATGTCACCATGTAGGGACCGGAGCNNCGCTTGACCCTCCTCTTCTTCGCGTGGCTAGTACGATNCTTATCCGGGTTGTTGGTGAGTCCTCNATGATCCGGGGGCGNTGCTTATGNTTCCTNCTGCTATGTNTTGAGACTTTNNCTTNTAGTTGTGTTTTGACTATGAGGGTGAGCCCGGTAGTNTGTCNTGGCCCCGTCCTANGTACGTATGTTTAGAGGTGTGCGTTGGACAAGTTGTGTNTTGTGNNTGAGCTTGACTCTTGTTCTATGATGTCATTTTGAAATGGTTTGCCCTTAGTCCCTATTTCCCGTTAATTAATGTTGAATGTACTTCCGCGACCGATGAAGTGTGATGACAAGTTTGAGAGGCTTGTTTGGGGTTCCTTCGGGTTCCTCATTCGCCATGTCACGTCTAGGCCCTAGGCTTGGGTCGTGACA

>rnd-1_family-23#LTR/Gypsy ( RepeatScout Family Size = 1309 Final Multiple Alignment Size (possibly truncated) = 98 Localized to 2361 out of 4218 contigs )

GTTATCTCATGAGATGGAAACCTCCTACGTCAGTTAGTCCGGGTTTCTAGNAGCAATCTCCCTATCCCATAACTATGTGCCCACATAGGTCTTTAGCTAGTGGATCCACCTAAAAGCTAATATGTTAGTTCTACCTTAGGCAAGTAGGACACCTCTTTTCGGTGTGGGGGCANGACACCGGATTCCATGTAGCTCACATGGTCTATGTCGGTTAAGGCTATTTTCCCTACATGATAAGAATATGAACTATGACAAGAACATGAACATGAACTATACTTATGACTTCTTAAGGAGTTCTACTTAGTATGAGTGGGGGTATGGGACTTCACTTATGCATTGCACAAGTAGACTTTGAGAGGGGTTATGGTGTGGTTCTCTTATGTTATGANGACNTATGAATTTATGCATGTATGATATGGATNGTTGCTATGANTGACTTTCCTTGTTACAAGTTATGAACATGAATGTTTCCTTATTTATGANTATTGGCTATGGATGAGGTCAAGGTATGATATGTATGATTATGGTGGCCTAAAAGTGGTACTTAGTATNNTTGGTAGGGTTATGGGATCTTACCTATGCATTGCACAAGTATGGCTTAGGGTTGCTTATGTGNTGGTTTGACTTATGTTTCTTCATAAATGTGCATNATGGCTTTTAAACTTGATAAATGCATGGTTTCAACTAAATGTCCCTTTTACGCATGTTTTAAGGATTTTGTGCATGGCTTCCATACTTAGTACATTTTTGTGCTAACCCATATTTTCTACATTTT

>rnd-1_family-297#Unknown ( RepeatScout Family Size = 288 Final Multiple Alignment Size (possibly truncated) = 98 Localized to 2361 out of 4218 contigs )

CTAGGGTTCTTCAAGTTCAAGTCTCCTTTCTTCATCATTGCTTAGGGCTTCGATCATCAAGGTATGTAGGGANTTCATCAATGGGTTCCATTCACCCATTGAGTCCCCAAAGATTCTTCAATTCTCCAATTCAATTTCCCCAAATTGATTAGGTTTTCATTCAATTCCTCATGGGTTCTATTTGTATGGATTCAATTGTTTGATTTCGATCTTATTATGCATGATTTGATTCAATTACATGNTTTTCAATTGATTTCATATGAACCCATGCATTGNTCATGAATTTTGACTATGAACCCTAACTTACATGAATTTATGAAGAAGCTATGAATCTATGAATGATGTTTTTATGAA

>rnd-1_family-186#LTR/Gypsy ( RepeatScout Family Size = 494 Final Multiple Alignment Size (possibly truncated) = 98 Localized to 2361 out of 4218 contigs )

CTCGTCTTTATATAGTAGAGGCTTCATAGATAGACAGTTTTGGAGAGTCTTTCAGTTTCAGATGTTTTATTTAAAACTTATGTTTCATACTCAGTATATTCAGCGAAGTTTTGAGATTCAGTAAACTGTTTTTAAACGTTTCTTTCAGTTTTANGCTTATGTATGCTTGAGTTAGTCTTCCGCTTGTAGTCAGCCAGGATGAGGGTTCGCTTGGGGACCAGCAATGGTTCTCGAGTGCCGGCCACGTCCAGGGTGTAGGCTCGGGTCGTGACA

>rnd-1_family-228#LTR/Gypsy ( RepeatScout Family Size = 414 Final Multiple Alignment Size (possibly truncated) = 98 Localized to 2361 out of 4218 contigs )

TTTTAAGTCATTAAACACCCNTTTACACCTCATAATTCCCAAACCCAAAATAANAACCAACTCCTCTCCAAATAATTCTCTCTCTAAAACCTCCATTGAAGAAGAAGAAGAGGTCGAGGTCTAGGGTTCGAGAANTCAACTTTTCCACTCAATTTCGTGAGGAATCTTCACTAAGGTATGGTAGCTTTTCATCCNTGGGTAGCTTTCACCCANGGAGCCCCTTCAAAACGTGATTTCAAAGCTTCCAA

>rnd-1_family-292#LTR/Gypsy ( RepeatScout Family Size = 297 Final Multiple Alignment Size (possibly truncated) = 98 Localized to 2361 out of 4218 contigs )

AAAACCTAGGGTTTTACTCAATCTCATGGGTTCTTTCATCAAACGTTTTCAAACGGTTTCTAATGACGAATTATGATTGNATTGATGTTTAATTGATGATTTATGATGAAAATACTCTATGAACCCATGATTTCTCTCAATTCCTAAATTGTGCCTTATGAAGTGGGTTTGTTGATTATGAANGCATGTTGATGGATTATGTCTANATTGATTTAGATTATTGAATTGTATCATTATCTATGCCTAATTATGGTGAATTGTTGGTGTGTTGATGANTTGTGATTCATGGCCTTGAAGGGCAAGTTATGACCNAATTACATGTTGAAGTAGAATTGTGCTTGAGCTGTTGATCCACTTGAAAGGTGGAGGTGTTTACTTACTATGTATATTGTGATCATGGCCTTGAAGGCATAGTGTGACGAATTGAAGTATTATCATGATGTTGTATGTATGATCGTGATGTGAAGNATNTGCGTGTGATTTCAATGATATAATGATGATCATGTNTAGAATGTAATTGTGTT

>rnd-1_family-278#LTR/Gypsy ( RepeatScout Family Size = 320 Final Multiple Alignment Size (possibly truncated) = 98 Localized to 2361 out of 4218 contigs )

TCAGACGGTACTCCGAGGTGGNTNGAGGNNGGAGCNCCNATCGAGAAGAAGGACCTGAACGTAGCNGCGAGGTACTGGTTCGGCTTCATCAGCAGCACNATCATGCCATCCCAGAACGAGTCNATCCTCCGCCACGCNAAGGCGGCCTGTCTNGGTTGCATCATNGACGAGACGAGGNTNAACTTGGGGATGATCATTGCGCAGGAGATGGCCATGAGGGCCAAGCAGCGCCAGACNTCNCTTCCNTTCCCGGTGTTGATCACCGAGTTGTGCAGACGGGCTCGGGTNCCTCGAGATGCGAAGAAGGATGTGGAAGTGACTCCCACATCCTCTACCGACATCCGGNGGATCGAGGCCGAGTACTCGAAGGACGAGGCGGAGAAGAAGAAGGCAGCCCCGGTGGACACNTCCCCGGTNGTNGACGCTGANNCGNTACCTGCAGAGGCANCTTTGCCTACTCCGGCCCCTGGGCCTTCAGGTACNTCTAGTGCCGCTCCTTCTGACCCCCTAGTTCCTCTGCTGCTNCACTGCCTCCCAGACCTGCTGCTGTTGCTGCTTCCCGGCCTCCGCTCACCCAGGCTNCGTTACTCCGGATGGGGCAGCTNGCCCATTCTGCCGATCGTCGNGCCGCCAGACTNGAGGCCTCCGTTCCGGGCATGATTCAGACNGCCCTCGCTGATGCTGTGACACCNCTGAGCGCTACCATTGATGCCCTCGCGGCNAGGATAGCGGTGTGTGAGCGCGGCCAAGGGGCCACCGAGGAGGTGACGGCTTTAAAGGCCGCCATTGCTGAGCTGAGGAAAGATGTGGACCANCTGAAGTCCACCGATATGTCCATGATCTTCGGGACGGTGGAGATCCCAGACGTGCCNGAGATGCCTCCGGCTACCACCGGAGATGGGGNTAGAGTNGAGNAGACAGCCGATCCCGAGTCNGAGGCGGAGACTGACGAGGAGATGCTTGAGGNGGCTGAGGAAGCTTCGTATGAGGGCCTTACCGAGACTGAGGAGGCCATGGTAGATGCGGCTGTGCAGGCTTCCTTGG

>rnd-1_family-242#Unknown ( RepeatScout Family Size = 377 Final Multiple Alignment Size (possibly truncated) = 98 Localized to 2361 out of 4218 contigs )

TTGGGAAAAGGCACAAGTACCCCCCTAGACTATGACCGAAATCCCAGAGACACACCTTAACTAAACTAAGGTCCTATTACCCCCCGAACTCATTTTTTTTGTAATTTTGTACACCTTTTCGGCTTACGTGGCATCCAAATATCTCCCACGCGCCTCAANCGCGTGGAGTCACGGGGTGTGCCACGTAAGCCGAAAAGGTGTACAAAATTACANAAAAAATGAGTTCGGGGGGGTAATAGGACCTTAGTTTAGTTAAGGTGTGTCTCTGGGATTTCGGTCATAGTCTAGGGGGTACTTGTGCCTTTTCCCTAAT

>rnd-1_family-246#LTR/Gypsy ( RepeatScout Family Size = 368 Final Multiple Alignment Size (possibly truncated) = 98 Localized to 2361 out of 4218 contigs )

GAACACCTCCGCGTCGCGGACTTGTTTCCCGACGAAATCTGCGAAAATAAAACTCAAGTTTGACTTGTTTAAAAGGTCCAACTAAGTGAGGGGTAGTTTGGGTACTTTGGGGAATTATTATAAACGGTTATTCTACCTATTTTAGGGTATTTTAAGTNGGTTAAAACCCCAAAACCTAGAATTAGACCTCATTCTTCAAATCGACCCTTCCCTCTCAAAAAGTTCTCTCTAGAACTCCATTGAAGANCNNGATGAAGCTCAAGCTAGGGGATGGGTTTTCAACTGATTTCTTCTTCAATTTCGTGGGNNTTCAATAATTAAGGTATGGTGACTCTTNATCCTTGANTAACCTTTCATTCAAGGAGCCCAATCAAAGATGTTTCAAAAGTTTTCCAAAGACCAAAANCCCCAATTTTCAATCTAAGCATGGGTTCTTGCATCAAACGATTTTCAAATGATTCTATTGATGAATTAATGTTGNATTGATGTTTTTAAGATGATTTTNAATGAAATTCTCCATGAACCCATGATCTCCAAATTCCTAAAATTGGCCTATGAAGTGGGTTTNNTGATTATGATCTTATTGATGGAATTATGTTTAGATTGGNTTGTATTGTTGATTTNTATNATTATCTATGCTTATTTATGGTGAATTGTTGGTGTATTGATGANTTGAGGATTATGGCCTTATGGGCAAAGTTATGGGTTGATCCCTTCGTTTATAGAATTGTNCTTGGATTATGTTATGTTATTGAATTACGTTGTTGATTCCTATTGCTATCTATGTCAATTGATTATGAATTGTTGAGGATTGATCCATGGTGATCATGGCTTGGAGAATTGGTAAGTTGACCTAACTTCTAGTCTATAGTATGAGAATTGATGTTGTGTGGTTTGATCCACTTATGGTGGAGGTGTTTACTTCTTGTCTATCTATGCATGTTGAGATCATGGCCTTGAAGGCAATAGATGTGAAGTGAATTGATGATTATGATGCATATGTTTATGAAGTTGTGCTATGAAGGTTATGTATGAAATCCTCAATGCTAGCATGATGTTTAAAGTGTGTTGATGATGAGGAGTATAATGTGAATGATAATGTTAGGGATAAGGTGTGGTCTACATGATTGATTATGTTAGGTACTATTGTGGAATGACCCCTACCTATATGACCCCTATATGAATGTGTGATCATATACATGTTAGGAGTACCTATTCAATGTTGAATGTGTCCTTGTCTCCTATGATGAAGTGTGGGTGTGTGGTCTAGAATCCCTAAAGGCTTTATATGTGAATTGCTCATGATTTGGGGACATTGTGNACGTGTTGTGATCCCTTGAAGGGAAGTGCACTCGATGTCAATGTTGTTGCTATTATGTCACTACGAGGACCAATATGCACACACACACTCTACATGCATAGCTATGAATATGATGTGTTATGGTGTCTATTGAAAGGCTAACTCTCATATGCACCCTTACATATTATTATGCATGCAAATGTGTATGATTAGCAAGGTATGAATATGTGTTGTATAAAGGTGTTTATGTGAAAGGCTTTCTCACATATGTA

>rnd-1_family-187#DNA/TcMar-Stowaway ( RepeatScout Family Size = 494 Final Multiple Alignment Size (possibly truncated) = 98 Localized to 2361 out of 4218 contigs )

TATTACTCCCTCCGTCCCATTTTATGTGAGGTAGTTTGACTCGGCACGGAGTTTAAGAAAGAAAGGAAGACTTTTAAAACTTGTGGTCCAAAATGAATGATAGAAATTTGTGTGGCTGTAAATCATTTCATTAAGGGTAAAATAGACATTTTATAGTTAAATTGTTACTTAATATAGAAATGTGTCATTCTTTTTGGGACTGACTAAAAAGGAAAGTAAGTCATATAAATTGGGACAGAGGGAGTAT

>rnd-1_family-209#LTR/Gypsy ( RepeatScout Family Size = 445 Final Multiple Alignment Size (possibly truncated) = 98 Localized to 2361 out of 4218 contigs )

TTTCGTACNTGTTCCTTAGCGATGTACCTATGTTTTCTAGTTGATTCCAACACTCTAAGGTACGTCTAAACATCACGAAATCATCCATAAACATGAGATCATGAACCTTGAATCCATAATCCAATTCAAGGAAAGTTAGGATCAAAGTCAAGAGAAGTTAAGAGTCAAGTCNAGAAGTTAAGAAGCAAGTCAAAGCAAAGTTTTTAAAGTTTTCAAGAGTCTTTAACGAACGTTTTAACTT

>rnd-1_family-454#Unknown ( RepeatScout Family Size = 147 Final Multiple Alignment Size (possibly truncated) = 98 Localized to 2361 out of 4218 contigs )

TAGGGAAAAGGGTCAAANATACCCCTAAACTATTCGAAAAGGTCTAAATATACCCTCCGTTTATANTTTGGCTCAAANATGCCCTCGCCGTCNAACTTTTGGTCCAAATATGCCCTTATGGGCGTTAGTTGGCCCGCTGGAAATATCCAACTCATTTTNCATTTCTTTAAATGCCAAATGGAATTGCCA

>rnd-1_family-312#Unknown ( RepeatScout Family Size = 267 Final Multiple Alignment Size (possibly truncated) = 98 Localized to 2361 out of 4218 contigs )

TTTATATACGGAAAAGGGTCAAAAATACCCCTAAACTATNCGAAATAGNTCANATATACCCTCGTTATATTTTGGGCTCAAAAATACCCTTGCCGTCATACTATTGGNCCAAAAATACCCT

>rnd-1_family-421#DNA/TcMar-Stowaway ( RepeatScout Family Size = 169 Final Multiple Alignment Size (possibly truncated) = 98 Localized to 2361 out of 4218 contigs )

GGGTAAAATGGTAAACTCACTATGTCAATCATTGTTTTCTTAATAGGTGTGTCAATTCAAAAGTGGACAAGTAATTAGGGACGGAGGGAGTA

>rnd-1_family-487#Unknown ( RepeatScout Family Size = 133 Final Multiple Alignment Size (possibly truncated) = 98 Localized to 2361 out of 4218 contigs )

TCAGTTCGAAGATTTGAATATNNACACTATCACTTCTGATAATGTGATAGAACTTCTAAAAGAAGTTACCGATAATAATCTTCGTGAAAAAATTATTCAATTGGCTGCTAGTAATAATGCTAGCTCTTCAAANATTCCTGAAAAANAGGNNAANGATGAGTTTGANTATTCTGCTCCTTACTCTTTGTCTGAAGTTNATAATAGACTCTCTTCTAAACAAACCATTGTTACTCGAGATNCTTCTTTTGATGATTTAAAAGGNGAAATTGAAAATTTGAAAAANGAGATTAAATCTCTTAAACAAAATCAAATGATTTGTGATCATCGTCTTACTCAGATTGAGNCTGCTAATAACAAAGGTAAAAATATTGTTGAAGAATCTACTGCTGAAGAAAATACTCTTGCNAAACCTTTTAATCTTGATCCTAAACAAGGTATGTTTTTAGGAATGATGCAAATTGTTACTGCTCATAAATGGTATGTTAAATGTACCATATTGATTGATAATAGTTTTTCTATAACTGATATTGCTATGATTGATAGTGGAGCCGATGTTAGTTGCATTCAAGAAGGTCTTGTACCTACTAAATATTTTGAAAAAACAACTCATATGGTTAAATCTGCTTCTGGACATGCTTTAGATATAAAGTATAAATTACCTAATACTCGTATTTGCCAAAATAAAGTTTGCATTCCGCATTTCTTTTTCTTGGTNAAAAATCAGTTATACCCTCCAATTATNCTTGGAACCCCTTTTATAAATGCTATTTATCCTTTTACTAGCATAAATGCTAAAGGNTTTTCTGCTACTTATGAAGATCGAGATATTAGTTATACTTTTATCACCGANCCTATTTCTCGNGATATTAATGCTTTGATTAATATGAAACAAAAGCATGTTGATTCTTTACAACTCGAANTATTTAGTATGAATATATTCGATACTTTGAAATCTACCAAAGTACAGGAAAAGATTAAATTAATTTCCGAACAAATNGCCATTGATATTTGTGCTGATCATCCTAGTGCTTTTTGGAATCGAAAAAAGCATATTGTNACTCTTCCATATGAAGATGATTTCTCTGAGGATAATATTCCTACCAAATCNCGTCCTTGTCAGATGAACGCCGAATTGGTNGAATTCTGCAAAAAGGAAATTGATAATTTGTTACAAAAGGGTTTGATAAAGCCTTCNAAATCACCTTGGTCTTGTACTGCNTTTTATGTTAATAACGCTGCTGAAAAAGAACGAGGTGTCCCCAGGTTGGTTATCAATTATAAACCTTTAAACAAATATTTAAAATGGATTAGGTATCCTATTCCNAATAAAAGAGATTTATTGNCNAGATTATATGATGCCAATATATTTTCAAAATTTGATTTAAAATCAGGATATTGGCAGATCCAAATATTTAAAGAGCATTCTTATAGAACGGCTTTTAATGTCCCGTTTGGACAATACGAATGGAATGTNATGCCNTTTGGTTTGAAAAATGCTCCNTCTGAATTTCAGAAAATCATGAATGATATTTTCAACCCATATTTGGATTTCATCATTGTTTATATTGATGATATTTTGGTNTACTCAAAAACTCTTGAAACGCATATTAAGCATCTAGACATTTTCAAGAAAATCGTTATNCAAAATGGTTTGGTNATNTCTAAACCAAAAATGAGTTTATTTCAAACNGANGTTAGATTTTTAGGNCATCNTATTTGTCAAGGGAAAGTTACCCCTATTCANAGATCTATTGANTTCGCATCAAAATTTCCTGATGTTATTACNGATAGGACNCAATTGCAGAGATTTTTGGGAAGTTTAAATTACGTTTCCCCCTTTTACAAAAATTTATCTCGAGATTTAGCCCCNTTATACGACAGGCTAAAAAAGGATCATAAANCGCCTTGGACTGATAGTCNCACCGATCTGGTAAAGAATATTAAANTNCGNGTTAAATCTTTACCTTGTTTAACTCTTGCTAACCCTGCTTGGCAAAAGATTGTNGAGACGGATGCGTCTAATATTGGNTATGGAGGNATATTGAAACAAATTAATCCNCATGATAAAAATGAATATCTNATTCGATTTCATTCNGGAAAATGGAGCGATGCCCAGAAAAAATATGCTACGGTGGCNCATGAAATGTTAACCATCGTTAAATGCGTNTTAAAATTTCAAGATGATTTATATAATCAAAAGTTTTTGATAAAAACTGATGCTCAATCTGTNAAATATATGTTTGATAAAGATTTTAAACATGATGCNTCNAAATTAATNTTCGCTAGGTGGCAGGCNCAGTTAGCCCCTTTTGATTTCGAAATCCATTATAAAAAGGGAAGTGATAACTCTCTCCCAGATTT

>rnd-1_family-77#LTR/Gypsy ( RepeatScout Family Size = 857 Final Multiple Alignment Size (possibly truncated) = 98 Localized to 2361 out of 4218 contigs )

ATGAGTTAGGTGGCCCATAAGATATCAAATGAAAGGTCTTTGAATCCTCTTTCCAACGCCACCGAGTTTGCTAANTTTCGAGCTCGTATGAGGGAGATATGCCCGTTTGAAGTCGGGCTGTCCAGTTAAGGAAAGTTACCCGAANTNATGAGGGGTATTTTGGTCTTTTCCTTACCCAATCAGATTTAANCGTTTTTAGTAATGTTTTAGGGGTCTAAACTGATTTGGTTCAGTTTTCACAATCCTAAAATACGCTTAGGGTTTTTAGAGGAGTTCAAGAAGAAGAAAAGAGGAGAAAAGGNAAAGCGNTCAAGGTTCTTGCGAATCGAGGATTTGTTTCGCCAAATCGAGGTATGTAAGCTTTCATAGTGTTGGGTTCGTTCACCCACACGCCAATCATG

>rnd-1_family-459#DNA ( RepeatScout Family Size = 145 Final Multiple Alignment Size (possibly truncated) = 98 Localized to 2361 out of 4218 contigs )

AGGGTGTGTTTGGTATGAAGGAAATGTTTTCCAATTTTCTCATGTTTGGTTGGGTTAAATGTTTTGGAAAATGTTTTCCAAATCAACTCATTTTCCTCAAATTTAAGGAAAATGACTTCCCTTCAAAACTTAAGGAAAACATTTTCCAAAACTCTCTTCCAACTTCAAATTACAATTATTTTTTTGTTGAAAAAATCAATTTATTTTGTCCCTACCCTCAAA

>rnd-1_family-554#Unknown ( RepeatScout Family Size = 108 Final Multiple Alignment Size (possibly truncated) = 98 Localized to 2361 out of 4218 contigs )

CCTCTGCCTCCCGGCCCCCAGATCACTCAGGCCATGATCCTGAAGATGGGGCACCTAGCCCATTCTGCCGATGTGCGNGCTNCCCGGCTNGAGAGGTCCGTTCCGGGGATGATTGAGAGGG

>rnd-1_family-125#LTR/Gypsy ( RepeatScout Family Size = 649 Final Multiple Alignment Size (possibly truncated) = 98 Localized to 2361 out of 4218 contigs )

GACAAGAGGCGATTACCCATGTCNCGTAATGATAAGGACAAGAGGCGATTACCCATGTTCCTTTAAGAGCTAAGACGAATANGATTAATGACTATTCCGTGGGATTTATGCTTAGCACCGAGTGGACAAGTGGCGATTACCCGTGTCCCATAACTATGTGCCACCATAGGATTGTCTAGATAGACATTAGCTAGTGGATCCACNTAAGCTAGAAGTTCATGGTCCTTACCTTGGCAAGTAGGACANCCCTTTTCGGTGTGGGGTAGACACCGGATTCCATGTTATAGCTCACATGGTCTCTATGTCGGTTAAGGCTACTTCCCACAA

>rnd-1_family-318#LTR/Gypsy ( RepeatScout Family Size = 263 Final Multiple Alignment Size (possibly truncated) = 98 Localized to 2361 out of 4218 contigs )

GAGATGGTTGCNGACATGAGGAGCAGGATGAGTTTGTTCGTTGCTGGGTTGTCTCGTCTGTCAAGTAAGGAAGGCAAGGCAGCNATGCTGATAGGGGACATGGACATAGCAAGGCTGATGGTCCATGTGCAGCAGGTTGAGGAAGANAAGCTGAGGGATAGAGAAGAGTTTAGGAACAAGAGGGCTAAGACATCAGGGAATGAGTCCGGGCAGCAGAAGAGTAATGCGAACCGGTCNTCCTTCCAACAGAAGCAAAAGGGACCTGCTCCATCATCTGCTAGTGCACCTGCACCNAGGAACAAAGGTGAGTACAATAGTCAGAATT

>rnd-1_family-575#Unknown ( RepeatScout Family Size = 101 Final Multiple Alignment Size (possibly truncated) = 98 Localized to 2361 out of 4218 contigs )

AATTCATGTCTANGTGCTCTAATCTCGAGCAATTCATNANGTANNTCATCAATCCTAGAAGATCACATCTAAACCCTCAATTTCCCCTTTCATGGCATAA

>rnd-1_family-576#Unknown ( RepeatScout Family Size = 101 Final Multiple Alignment Size (possibly truncated) = 98 Localized to 2361 out of 4218 contigs )

TACCGGAAAAACCCGAAAAAACCCGAAAAAACCCGAGGTTGAAAAACCCGAGTTTTATTGGTTTGGTTTGGTTTATAAATTTAAAAATCCGACACAAATGGTTTGGTTTGATATTTGAAAAACCCGAACCAACCCGGCCATGTACACCCCTAAT

>rnd-1_family-188#LTR/Gypsy ( RepeatScout Family Size = 492 Final Multiple Alignment Size (possibly truncated) = 98 Localized to 2361 out of 4218 contigs )

TGAGTTGGAGAAGGNAGTTCCTCCAATGAAATGAGAAATGTGAAGTTTTGATGCATAATGAGTTGGTAGATGTAGTTCCTACTTCCTTGTGTTGCATTGAGTCTTNTTGATGTGGAGTTGATGTTTCCTCCTATGTTTGTGCATTTATATTGATGTTGCATGCATGATGGGCTGCTAGTCTTGAGTCGTTATCCCTTAANGTGTTTAAAGCGTCATTCGAGGACGAATGTTCCCAAGNGGGGGATANTGTAACGACCCCGAAAATGAAGTAGGNTAAACTAGAGCCTCACGTGAGT

>rnd-1_family-31#LTR/Gypsy ( RepeatScout Family Size = 1239 Final Multiple Alignment Size (possibly truncated) = 98 Localized to 2361 out of 4218 contigs )

AGTTGAGTAAAGAGTAAAGAGTAAAGNTTGAAGTTCATTTCTTCAAAAGTATATGGGGACTATGTATTCCCAAAGAGTTTAAAATGTTTTCACATTTAAACAAGAAAGGAAACTTCGATTTCCAAAGAGCCTTCGGGCTAGTTTTCAGAAAAGAGTAANCGCTTTCNNAATNAAGCAAGAGAGGAAACTTTGATTTCCAAGATAGCCTTTGAGCTAAGTTTTTGAGCAATNATCTCAAATCACAGAAAGAAGTATGTTTTTAAACATAAGAGCTAGTATCATATTTTGGGAGTAGTATTGAGCACCGATATGGGGGAGAGTTCAGACAACTCACAGCCCCCATAAACCATGTAGCCATCATGGGTAGAAAAGGGTCATACTTTTTAGATGATTCCTTAGTGCTTTTTAGCATAGACTAGTGGATCCACTTAGTAGTTCAGGTTCTATACCCTCGGCAAGGTATAGGACGGCCCTGGCAGCGTGAGGCAAAACGTTGTATCATCACNATAGCTCTTANGTGATGGTTGTCGGTTAGAGAAACTCCCACAGAAGTAATTGTATTCTTATATACACAGTTTATTTGTATTTTTACATACATTTCAGAGTTATATTGTATCTTTGCATACACACAGAGTTGACATCGTGTTTTAAACAACTTTTCTTTATATTGCACTTGTTTTAAACTGCTTTATATTGAAATGAGTTCAGTTATGTTGAGTTGAGTTGAGCCAGGTAAGTTCTTCAGTTCCTTTCAAGCCTATGTCGTGTTTAGCATTCCAACTCGCATACTCGTACATTCAATGTACTGATGCCAGTTGGCCTGCATCTTATTATGATGCAGACACAGGTAACCAGGATCGGCATCCAGCGCATCGTTGATCCAGTTGAGCACTTCAGAGTCAGTTGGTGAGCCTCCTTGCATTCCGGAGGACCCTTTTTATTGCTTTCAGTTTTATTATTAGTTCATTAGGATGTCGTGGGGCTTGTCCCGACATCCATCTCAGTTGTTTTAGAGGCTTCATAGACAGTCAGTCAGTTAGTTAGTTCATTTGTCATTACTTTGTTATTGGCTTATGTT

>rnd-1_family-560#DNA/Harbinger ( RepeatScout Family Size = 106 Final Multiple Alignment Size (possibly truncated) = 98 Localized to 2361 out of 4218 contigs )

TAAAAAACTTCTCTTTCGGCTACATATGAAAATGTTTAAAGCAATTTAATATGAAAAAGGACAAAATTGGTGATGTATGTTTGATAATCACTACTTCCAATATGTTTGATTTTAGCACGAAATTAATGCACTGAAAATGTGTAATTGCACACAAGTGAAACAAGGCATGAACCATTATGATATTGACACACAAGTGAAAATTGTCATAGCTTGTGCAGTATTACATAATTNTTTGCGNGAACACCAAAGTAGTGATGAAATATTTATGGTNTATGAACGTGANGATATAGTTGCGGATGANATTGACCAACAAACGGCTCAAAGTAACAATGTTGGTTCGTCTTCTCGGTCACATGATCGAGAAATGCAAGTTCAACGTGAGGAGATTGTCCGTACTATGTGGGAGGATTATATTAAAGACTAGTACACTACAATTTATGTTCAATTTTTTTATTGAATTAAAGTTTGATCAATTAAAGTTAGATGAAACAATTACTTAAGTGGAGAACAAATAGCTTTGTAATAATTTATTAT

>rnd-1_family-290#LTR/Gypsy ( RepeatScout Family Size = 300 Final Multiple Alignment Size (possibly truncated) = 98 Localized to 2361 out of 4218 contigs )

TGGGTCCTTAGTTCTTTTCCAAAGTTGAGCCCNCGTTATTCTATTCCTTTCCTAGCAACTATAAGACCTTTTTAGACCGAATTCTTTCATTTAAGTCACTCCAATTAGAATTAAAAAAGAAACCCACCTCCTCTCAAATATTTCTCTCTCTAGAAACCTCCATCGAAGAAGAAGAAGAAGTTGGAGCTAGGGTTTGGGATTCAAGGTGTTTCTTCNTCAAATTTCTTGGGGAATCTTAATAAAGGTATGGTGACCCTTCATCCTTGGTTAGCTTTCATTCAAGGAGCCCATTCAAAAGGNTTTCAAAGTTTTCAAAGACCAAAAATCCTAGTTTTCANTCTAAGCATGGGTTCTTNCGTCAAACGTTTTCAAAGTCATTATATTGNTGAATTGATGTTGTATTGATGATTTTAAGATGATTTTATATGAAANTTCTCCATGAACCCATGTTTTCNCTAAAA

>rnd-1_family-316#Unknown ( RepeatScout Family Size = 265 Final Multiple Alignment Size (possibly truncated) = 98 Localized to 2361 out of 4218 contigs )

TAGCAGGACCCTCCTACATAATGGGATTTAGATTAGCAGGACCCTCCTACATAATGGGA

>rnd-1_family-463#LTR/Gypsy ( RepeatScout Family Size = 143 Final Multiple Alignment Size (possibly truncated) = 98 Localized to 2361 out of 4218 contigs )

TTAAGGTATGGTGATCCTTNATCCTTGATTANCTTTCATTCAAGGAGTCAATTCAAAGAGTTTCAAAGTTTTCAAAAACACAAAAACCCTAATTTTTACTCAATCTCATGGGTTCTTGCATCAAACGATTTCAAATGATGANTTATGATTATATTGATGTTTAATTGATGGTTTAAGATGAAAATACTCCATGAACCCATGATTTCTCTCAATTCCTAAATTGTGCCTTATGAAGTGGGTTTGTTGATTATGAATGAATGTTGATGGAATTATGTNTAGATTGATTTGGGTTGTTGANTTGTATCATTATCTATGCCTAATTATGGTGAATTGTTGGTGAATTGAGGATTTGTGATTATGGCCTTGAANGGGCA

>rnd-1_family-267#LTR/Gypsy ( RepeatScout Family Size = 334 Final Multiple Alignment Size (possibly truncated) = 98 Localized to 2361 out of 4218 contigs )

ATCCCTTCATGGATAGCTTGACTTGGTAAGACAAGACCATTCATGGTAGCTTGAGTTAGTAGAACTCATGGATAGCCTAGGATTGAGAAATCGTACTCTCCTAANGGTTAGCTTGAACTAGCATNATAGGATGCTTNCATGGTAGCNTGACTTGGTNAGGCCANACCATTTCATGGTAGCTTGGATTGAGCAATNGTACCCTTCCTTGGGTAGCTTGGACTTGCATGATAGTGTGCCTTCCATGGTAGCTTGGTCTAGCATAATAAGGTGCTNTTCCTTGGTAGCCTTGAATTGGTAAATNGTAACCATTCATGGGTAATGACTTGTCTTGGACTAGTAAAGAAGTACTCTTCCATGAATAGTAGGAATAGCTAAGGAGGGACTAGTCCCCAATCAAAGTAGCATGNTTATGATGAAACAAGGTTACTTCAAGGAAGTATCGTAAGTATGNTTTAATATGAGATTATGTTATGATGATATATTAGCTTGGATTGTTGCTTGAGTTGCCTTCCATGATANGTATGACTAGAGGCGGATGACCCAAGTCTTGACTAAGGATAAGTTGAGGTTACTTGAGAAGTATTCCTCTTNTATTATGATGATTTATGTGCATTGATTATGCTTATGACTTATGTTGTCTAACTCTTATGTTTATGGTTTATGTCTTATGTTGACTAATGCTTATGTTAT

>rnd-1_family-273#LTR/Gypsy ( RepeatScout Family Size = 328 Final Multiple Alignment Size (possibly truncated) = 98 Localized to 2361 out of 4218 contigs )

CAAGGTTTNAAGCTCAAGANTTCATCTTTCTCACTCAATTTCGTGGAGATTCTTCAATNAAGGTATGGTAGTCTTCATCCNTGGNTAGNTTTCATCCANGGAGCCCCTTCAAAANNTGATTTCAAAGTTTCCAAANTCCCCCAAAAGCTAGGGTTTCAATCTAAGTCATGGGTTCNNTTCCAAAACGTTTTCAANGGTTTATTTATGATGAATTATGATTGAATTGNTGANTTAATGTTGATTTATGATGAAATTCCCCCATGAACCCATGATTCCCCCAAATTCCTAAATTGTGATTTGGGATTGATTGATTATGGAAGCATGTGAATTGATTATGTCTATATTGATTTAGTTTATTGAATCATGTTATTACCCATGTCTAATTGTAGTAATTCTAGATGTGTTGGTGAATTGTGATTCATGGCCTTGAAGGGCAAATTATGAAGGATTGATGTATGATTATGATGTTATGTATATGCGTGTGATCTCAATGAAAGT

>rnd-1_family-95#LTR/Gypsy ( RepeatScout Family Size = 764 Final Multiple Alignment Size (possibly truncated) = 98 Localized to 2361 out of 4218 contigs )

ATTATTATAATTTTCATATAGAATAGTACTTTTTGATATTTTTGCTCTAAGTTTGAGAGGGTTTTGAAGACTTGAAGATNNAAAGGGTTTCATCTCCAAGAATTTGGANTTGGGTCTCTTGGATTCTTCATTCTTGGCCTGTATCAAGACTTAAATCTTCATACCCATTTGAATGCAANCTCATTTTGGTATAAATTTCTATCTCTTNTACATGTGTGGCTAAAAACCCCAATTCTTGGGGTGTGATTTAGCGAATATGGGTTAAGATAATTGTTGGGTCTTGCTTGCTGATAGTTTANTCGTAGTTTAANTGTGATTTCGTTTAGTGGTTGTGGNTGAATTTAATGAGTTTGTAGTTGCAAATACAANTTCACCTATGTGTTTTCGGCTTGCTCGAGAGAGAGGTCGTAAAACTAAGACCACTAGATTGATGGCCGGTGNGAGTGGGTCGACATGAGGTTCAGCTCGAGAGAGTGAACCCTAGTCCCATGTCCACACACTCAGCTCGAGAGAGTGAGTGGGNTAAGGCGTAGGCTGGTCTTCATGCGGCAAGTGGGTGTCCGAGAGGAACCCATTTGAAACGGGGTAAGTTGCTCGAGAGAGAGCTTATCCCCCTTAAAGTCTAGCCTAGTCACAATTATTCTACGAATTTNCTATCGAAAGCATGTACCCAATAATTTAGCTTAACCCGTATTCCNGTCACATCCCAAGAATCCCCTCTCATTACTTAGAAATCTTCGTTTATTTTGCTGTTTTTACTTACTNGTGACAAACCCCCCATTGNTAATTGACACTCTTGTGTCCCCCTTTAATTTACNATGTTTTTANTCGTTAATGTCTTTAGCTACGACTAGTTAGAACTAAATTTTATTTTTCTATTAATTCTCAAAACCACTCCCTTGGGACTCGACCCCAACCCTTGGTTGGGTTACTATACTATTGTACGATCGTAGACACTCGCATCGGAAGTTGTGTCTTGATTACGCAAACATCAAAATGGCGCCGCTGCCGGGGAGTGGTGTTATTTGAGAATTTTTAGTTNAGTAGAATTTTTGTTCTTCTTAGTCGTAGTTTACTAACTTAATTTTTAGTTTTGTTTTGCTT

>rnd-1_family-197#LTR/Gypsy ( RepeatScout Family Size = 479 Final Multiple Alignment Size (possibly truncated) = 98 Localized to 2361 out of 4218 contigs )

GCCTAGTACGGGGGGTAGTAGGCCCGTATAACCGTATCGAGGGGTTGGTACCTTGGTTGCCTAGTACGGGGGGTAGTAGGCCCGTATAACCGTATCGAGGGGTTGGTACCTTGGTTGCCTAGTACGGGGGGTAGTAGGCCCGTATAACCGTATCGAGGGGTTGGTACCTTGGTTGCCTAGTACGGGGGGTAGTAGGCCCGTATAACCGTATCGAGGGGTTGGTACCTTGGTTGCCTAGTACGGGGGGTAGTAGGCCCGTATAACCGTATCGAGGGGTTGGTACCTTGGTTGCCTAGTACGGGGGGTAGTAGGCCCGTATAACCGTATCGAGGGGTTGGTACCTTGGTTGCCTAGTACGGGGGGTAGTAGGCCCGTATAACCGTATCGAGGGGTTTGTACCTTGGTCGCCTAGCTCGGGGGGTGGTAGGCCCGGGTAACCACATCGAGGGGTTTGTACCTCTTTCGCCTCTCCGAGGGGGTGGTAGGCCTTGGAAATACTATATTGATGGTCACTCACCACACATGTACTACGTCCCACTAAATATGATTTTTATGTAAGCGTCATTATATATATCATTTCATTAATGTGCTATCTATCGGGTATGATTATGCATTTTTCCTATGATGTCCTTATCTATTGTGCATTGCGTTGCACCCCGCATACTTAGTACATTCAAACGTACTAACGCATACTCTATGCCTACATGATGTCACCATGTAGGGACCGGAGCANCGCTTGACCNTCCTCCTCCGCGTGGCTAGTACGATTCTCTATCGAGGTTATTGGTGAGTCCTCCATGATCCGGGGACGTTGCTTATGATCCTNTTGCTATGTATAAGACTTTTNCTTTTAGTTATGTTTTGACTATGAGGGTGAGCCCGGTAGTTTGTCTTGGCCCCCGCTAAGTACCCATGTTTAGAGGTGGCGTTGGACAAGTTGTGTATTATGCTTGAGCTTGACTCATCTATGGTATTTATGTATCATTCCTTTTTTTTTTTCTCTTGCTCCCTTAGTCCCGATTTCCCCCTTGATTATGCTTATCGCTTATGGTCATTTTAATTGCTTCCGCGACCAAGGATGTGTAATGATACGCTATGAGGCTTGTTTGGGGTTCCTTCGGGTTCCCCATTCGCCGGTCACGTCTAGGCCCTAGGCTTGGGTCGTGACA

>rnd-1_family-127#LTR/Gypsy ( RepeatScout Family Size = 646 Final Multiple Alignment Size (possibly truncated) = 98 Localized to 2361 out of 4218 contigs )

ATCGGGTGTCGCGTTCCGACACACTAACTTGGATCGGNTGCCACGTTCCGGCGCANATATNGGATCGGGTGTCACGTTCCGACACACATATTGGATCGGGTACCACGTTCCGGTACGCTAACAGTTTGGGTGTGGGTTCCATGAGAGGACCATTGACTTGTCATATCTGCGTATCGTTGAGAATGTGGAATTGTACGTTGCTCCTGAAATGATAACTGATTTGTGCATTATGTTCGTATATGCGTGGTTTACTGTGTTGTAAGTTANTT

>rnd-1_family-405#Unknown ( RepeatScout Family Size = 181 Final Multiple Alignment Size (possibly truncated) = 98 Localized to 2361 out of 4218 contigs )

AAAAACCCGAAAAAACCCGAGGTCGAAAAACCCGAGTTTTATTGGTTTGGTTTGGTTTATAAATTTAAAAATCCGACATAAATGGTTTGGTTTGATATTTGACAAAACCCGAACCAACCCGGCCATGTACACCCCTA

>rnd-1_family-71#LTR/Gypsy ( RepeatScout Family Size = 899 Final Multiple Alignment Size (possibly truncated) = 98 Localized to 2361 out of 4218 contigs )

ACTGAGGATCATGNTCGNCATTTGAGGATNGTNCTCCAGACNTTGAGAGAAGAGAAGTTGTATGCCAAGTTCTCNAAGTGTGAGTTTTGGCTTGANTCTGTGGCATTCTTGGGACACGTGGTGTCCGAGGAGGGTATTAGGGTNGATCCNNCGAAGATTGAGGCAGTTCGGGATTGGNCCAGACCACATCTCCGACTGAGATTCGGAGTTTCGTGGGNCTNGCTGGCTATTATAGACGATTTGTNGAGGGNTTCTCNNCTATTGCATCTCCTTTGACCAGGTTGACTCGANAGAGTGTGAGNTTCCAGTGGTCTGANGAGTGTGAGGAGAGCTTTCAAAAGCTCAAGACNTTGTTGACTACNGCNCCTGTTTTGACTTTACCCGAGGAGGGTGNAGACTTTATCGTNTATTGTGATGCTTCTGGAGTCGGNTTGGGTGGTGTNTTGATGCAGAANGGNAAGGTNATAGCTTATGCCTCNAGACAGTTGAAGGTCCATGAGAAGAACTACCCNACCCATGATTTAGAGTTGGCGGCCGTAGTGTTTGCGCTTAAGATATGGCGNCATTACCTGTATGGNGTTCATTGTGAGGTNTTCACTGATCATCGGAGTCTTCAGTACGTNTTCANTCAGAGGGATCTNAATTTGAGGCAGCGNAGGTGGNTNGAGTTACTNAAGGACTACGACGTGACTATTCTNTACCATCCGGGNAAGGCCAATGTGGTGGCCGATGCTTTGAGTAGGAAGACTTTTAGCATGGGNAGTCTTCCTTTAATTGTGTCTATCTTTCTTTTATCTATCTTTTTTATTTCTGTTTTATTTTTATTTTTATCTTCATATTTTACATCAGGGCTGACTTTCTCCGCCAGGTCGTATGTTTTCAGAGCAGCCAAAGAGGTGGCTTCCTTCTAGTCCATTGTGAACACCGCCAATGAGGCAAAGTTGATGGTTAGGGAGAAGTTCGGAGATCCCAAGAGGGTCCCGCTCTTCAGGTTAGTTTTTTGGTGCCTCACTTGAGGCAGAGGTTGCGCATANAGGTAGTGGCTCTCTTCAGCGTTTCGGGACGGTTCATTCATCTATGCTAGCAGTNGAGAGTGGGTAGTTATCCCGAAGATCTTATGGTTCTAGCCGAGGTGGCTATGGTAGCTCATCTTGNTTCGCAATAGAAACTTTTCGTGCTGGGGTCGTGTTATGGTTGTGGAGATCCGGGTNATATGATNCGGTAGTGTCCTCTACAGACTCGCTCAGGCCCCAGCGTACTTATTCAGCTGCTCCGGCTAGAGATTCAGCGCCTCCGACCAGGGGTCNAGGCAGAGGTCAGTCCGGTAGAGGCGGTAGGACTTCTGGTAGGGGTGCTATAGCTCCGTAGAGTAGAGGTCGGGGTGCTACTCAGACTGNAGGTGGTCGAGAGGGNCAGTGTTATGCTTTTCCGGGCAGGNCCGAGGCNGAGGCGTCTGACGCNGTGATCACANGTACTATTCTTGTCTGCGACCGGATGGCTAATGTGTTATTTGATCCGGGTTCTACTTNTTCNTATGTGTCCGTNTANTTTGCCNCNGGATTTGATATGATNTGTGATATACTTGATGCCCCTATCCATGTTTCTACCCCAGTNGGNGAGTCCTTAGTCGTGGACCNATGTNTATCGNGCTTGTCCTGTTTTGTTTATGGGTTNTGANACTTGGGTTGATTTGGTCATTTTGGATATGGTTGACTTTGATATNATCTTAGGCATTGACTTGGTTNTCCCCCTATCATGCTGTNCTTGATTGTNATGCTAAGNCTGTGACTCTAGCGATNCCGGGTGANCCAAGAGTTAGAGTGGGGAGGGGTGCTACNAGTTCCTANCCAACTAAGGTCATNTCCTCCATTCGGGCTCGGAGATTGGTAGGGCAGGGTTGTTTGGCTTATTTGGCTCATATTCGGGATGTTGGTGTTGAGNCNCCTTCCATTGAGTCTATTCCTGTGGTTCGAGAGTTTNNAGANGTGTTTCCTACTGATCTNCCTGGTATNCCTCCGGATAGAGATATNGATTTTTGCATTGACTTGGAGCCGGGTACTCGNCCCATTTCTATTCCTCCNTATCGCATGGCTCCGGCAGAGTTGAGAGAGCTNAAGGCTCAANTNCAGGACTTCTTGAGTAAGGGNTTTATTCGTCCTAGTGCTTCCCCGTGGGGTGCTCCNGTNTTGTTTGTNAAGAAGAAGGATGGTAGTATGAGGATGTGTATNGATTACCGGCAGTTGAATAGGGTCACCATTCGAGAACAAGTATCCNTTGCCTCGNATNGATGATCTNTTTGACCAGCTGCAAGGTGCATCNGTNTTCTCTAAGATTGATCTGAGGTCCGGNTACCATCAGTTGAAGATTAGGGCATGGGATGTNCCCAAGACGGCGTTTAGGACCCGCTATGGGCACTATGAGTTTCTAGTGATGTCNTTTGGGTTGACTAATGCNCCTGCAGCNTTCATGGGTTTGATGAACGGGGTGTTCAAGCCGTNCCTNGATTCNTTTGTNATAGTCTTCATTGATGACATTTTGGTGTACT

>rnd-1_family-412#LTR/Gypsy ( RepeatScout Family Size = 175 Final Multiple Alignment Size (possibly truncated) = 98 Localized to 2361 out of 4218 contigs )

TTATGATTCAAGTTTTGATATATTGAGATACGTAATTAAATATTAGGTGTATTGTATTATATGAATACCATTAGATTTATGATATTTGGAGAAATTGAGACTTAACCCTAGGCTTGAAATTTGGAAAATATGAGAGTTGACATGTTAATTGGCATCAAGATTTAGGAACTTGATTATAGATTTGGGTGAGTTTTTGGGTCTAGGCTAGACATATAGTAATATGGGTGTTGTTAGTTTCGAAATCTTATTGTGATTATTTATTTGAATAGATTGCGTTGATTTGGAAGCTCAACGAAAGGGGAAGGCTCAAGTCCCGGAGTGATTGTTCGATTATTTGAGGCAAGTGGATTTCTAAACCCTTGTTAAGTGTATGGAATTCGTGTATTTCCTTGTAGTATGTGTTGGGGGTAATGAGACTTGGTGATGGGTTGACTTGTCCACATTGANTAATTCTAATGATGAAAAAGGGGTAATAAAAGGCAACGTGATTAATTGTTGGTGTGTGATGTGTTGAGAATNGTTT

>rnd-1_family-573#LTR/Gypsy ( RepeatScout Family Size = 102 Final Multiple Alignment Size (possibly truncated) = 98 Localized to 2361 out of 4218 contigs )

CCCTTTCATTCTACAAGGAAACATTGAGTTCANATCAACAGATGCCGGAAGACGCAGCAAGAAGACCTTAGCTCAACGCGATTCAACCTTTCTGAGTCACTAGCCTCAAAATAAAACGAGCAAATTCCTGCTTGTTCAAAATCAGTCCCCATTATTCGTGGGTTAGCGTGTCCTCAAACGAAGCAACTCTTATGTGTTTATNTGCCTTNTATGTGATATTTTGCATTATTTATCCTGAACTAATACTAACANATTTGCCTTTGAGTTGTTTTTCTCTACAGGTANTTAGAACTGATCTGTGTTGATAAGCTGGCCGANCATCCTTATTTCACAAGATCTAAAGGTCCTAAAGATTCCTTCCCCGATCGAAGTTCGCGAAAGGGAAAAGCAGCAATGGGTGATAACAACGAAGANACCGGCCTNACTGATGTCGTCGTGGCTCAGCCCGCCGTCGCCGATCAGAATGAATTGATCGCGCAGTTGATGCAACAGATCGCTGAGATGAGGGTCGAGATGCAGAGAAAGCAAGATTTGCCTCCTCCGGTTTTCGCTGTCAACGCTCNANCAGACGGAAGGCCTCCACTCCACTTTCCTCCTCCGAACGCGGAACANGCTCAAAACCCGCCCTCNAGTCCTGCTCGNAATCCCTCCATCATCGACCTAACCACCCAAAATCCCCATTACGCCTCCGCCTCTTACCAAACACCACCCCCTCCTCAAAATACCAACCCCCAAGCACCACTCCCTCCCCNNAATGCNAACCACCAAACTGGCCTACCCCCTCAAAACCAAAACGTTAACAACCCGCANACTTCCCTCCATCACCAAAACCAACATACCAACCCCCAGACTTTCCCCCAAAACTACCAAGCCCCTCAAAATGCCCAGAGTCCCTCCATCGCTCCACCCCTACCTCAAAAAGCCACTTTCCAAATCCCGGTCCCCAACGAGCATGACGCCNATGGTTCNGAGCTCGACCACTATGAGGAGAGGGAGAGAGAGTGGAGGTCGAAGGAAGAGACCGCCAAGATAGATATGAAGGAGGAGATTAGAAAAGCCATGAAGGAGTTGNACTGCATTCCTGAGGTCGCCGGGTTGAGTTACGAAGACTTGTGCATCCATCCGAATTTGGACCTCCCGGAAGGGTTCAAGGCGCCAAAGTTCGACATTTTCGGAGGAACGGGGAACCCCTTAGCGCACCTGAGGGCCTACTGTGACCAGCTCGTGGGAGTTGGGAGAGACGAAGCTTTGTTAATGCGGCTTTTTAGCCGAAGTCTGAGCGGAGAAGCNCTGGAATGGTTTACGTCTCANGAAACGAGACAGTGGCCTAGCTGGAACGCTCTGGCTAAGGACTTCGTCGAACGATTCGCCTACAATGTNGAGATCGTTCCTGATCGNTACTCNTTGGAGAAGATGAAGCAGAAGTCGACCGAAAGTTACCGAGAATNCGCGTACAGATGGAGAAAGGAGGCCGCTAGAGTTCGACCTCCTATGTCTGAGAAGGAAATCGTCGAAGTGTTTGTGCGGGTTCAAGAGCCCGAGTATTATGACAGGATNATGTCGCTCGTTGGAGCAAAATTTGCCGAGATAGTCAAGGTAGGTGAGACTATCGAAGATGGATTNAGAACCGGGAAGATTGCCCGTGTTGCTGCCTCGCCCGGATCTTCGGGNTTGTTGAAGAAGAAAAGAGAGGACGTGTCTTCTATCTCTTATGAGGGGAAGAAGACCCCAAGGAAATCCTCATCATACCAAGGTCGTTCTCGACCTTCGCAGAGTTCGTACCCGGCTTGTTATGCACAGGCTGATTACCAAAATACCCCTCCCCCCAGTTACCAAAATACCCCTCCTCCCAGTTACCAAAATACTCCCCCTCCCGNTTACCAAACTCCCCCTCCTATCTACCAAACTCCCCCTCCAGTTTACCAAACTCCATCTCATCACTACCGAAACGCCGCCCCCAACTGCGCNAACGTTCAGNCAAATTACCAAACNCCTCCCCAACGTACCAAACCCCACCTCCACATTACCAAAATACCCCCCCGAACTACCAAGCTCCACAACCAAATTACCAAACCAACTCATATCCCAGATATCAAGCCCCCCGTCCGAACGCTCCAAATTATCGTCAAATGCCTCCNCCTCAACAAGGCAATTACGATCCCCCCCGTCCNAGATTTGAGAAGAAGCCTGCTAGAATTTTCACTCCGCTNATTGAAAGCCGAACAAAGCTGTTCGAGCGATTGACCGCGGCAGGATATATTCATCCAGTGGGGCCCAA

>rnd-1_family-259#LTR/Gypsy ( RepeatScout Family Size = 345 Final Multiple Alignment Size (possibly truncated) = 98 Localized to 2361 out of 4218 contigs )

TATGGTTAGTTTTGGCACTAATATCGCTTCCGCAGGTACGATGGCACCCAAGAAGCTGGTCACCTACTCAAAACGGGGCAAGTCAAAATCTGTTGCCCCTAGCTTCAGGTTAATCGATGAGGACACGGACACGGAAANAGATCCAGCATACGTTCCTCCCAACACAAGGACTTCTCNCACTGCACCCCGAGGCACCAGAGGCACCCCCCGGAAGGTGNTTCCCGACGTAGTCACTGTCTCCCAGTCTGATGAGGAGCACACACTGATCGGGTCACCAACTGGGGCTGCTTCCAGTTCAGCTACTGGGTCTGCGTCCGGNTCCGAGTCTGCCCATGCTTCGGGCTCCGAGTCCGCCCATGCTTCAGGGTCCGAGTCTGCCCATGCTTCGGGGTCCGGTGCCGAGTCAGCCACAGGGTCCGGCGAGAATGACCAAGCGGCCTCGTCTGATGAGGCNACTAGCTCGGAGTCCGTACCNGTACCACGGAACGACGACCCCGCTCCAGTGGCCGGCGAGCCAAATAGATGGTGCGTNGAGGGCCAATGGCAAATNTATCGGGATGCCAAGATGATNAATGACAAAGAGAAGATGGCCCGACTTATTACAGAGGAGCGCAGAGTCCTCACGGGGAGCTTGCACACCGTCCCNGACATCCACCGGCTNTTTAACCTTCACAAGTGTGACTGGATGGCTCGAGACCCAGGGACGTATAGCGAGGAGATTGTGCGGGAGTTCTACGCCTCCTATGCCGCCACTCTCCGNGGTTCGATTTCCAAGCGGTCAAAGCCCNTAGCCCAAGACCCTCTCACTTCCACTATGGTCCGGGGTTGTCCGGTGGACATATCACCTGCCACCATCAGCCGNTTTCTCTATGGTCCTACCACGGGTCACTCTTGGTCACTNAACACNGCGGAGTTTGACTACCGATGGGACATCGTGCGGAGCGGCGCTTTCCAGAGGAACGCCGAGCAGCGGGAGGCTGTTNTACTATGGTTGGCCAGGTACATTGCTGCAGATGGCGAGCGTGCGGAGTGGGTCGCCGCTCCGCGGTTGGGCATCCGGAAGGCCACATTAAATTTTGTGGCCAAGTTCTTCTGGCTGTTGGTGCGNAACAGGGTGTCGCCTACAAAAGCTGACAATCAACTCACTTGGGACAGAGCGGTCATGGTTGCAGCATTGGTAGCAGGAGTGGAGATCGACTTTGCCCGCATGCTGCTGGCAGAGATTCACGAGAGGGCNTTCAAGACCTCCACTACTTACCCCTTCCCATGTCTGATTTTTCANTTGTGCAGGGACTCTGGAGTGCCGATCTGGCATTGTGACAAGTTGGTCCACCCTACGGGGACNTTGGACATNGGCCTNATTCGAGATGAGGCAAATGTGGCGGCACCTCGCAGAGAGCCCCAGGTTGAGGTACCTCCCTTGGGCGCNGATCTTGCAGACACGGTGGGGCAGGCGCAGGGCGGTGACCCCATTATCCCAGACCACACCGATACTGTCCCGGCCTCCTCTTCTCAGGCNGCTAGTANGGCTCCTAGCTCNTCCCGGTCCACACCACCGTTAGGAGCTACTGTCGTCCCGTTGGCCAGAGTACAGAAGTTAGAGGCTCAGATGGCCACACTGCTGCATCACGTCCAGCCNTGGATGCAAAAGTCGATAGCCGAGTCCGAGGCCAGAATGGAGCGNAGGATGGAGGGNATGATGGACCGGAAGGTCCAGGCCGTTAATAAGCGCCTCGATGCCTTCGAGTTGCGAGTCCTCGAGCGACCAGCCCCGACCACAGATCTATCCGCTTTNCGGGCCGAGTTAGCCAGTCTCCGGGCCGACGTTGATGCCATTCTTGCCGCACCTNCAGTTGAGCCTCAGGCTGCACCTACTGCACTGGCTGATGATACGGTGCTGGATGCTCTATTCAGTGGGACCGCCGAGGAGGGGCCCGCGCCNACACGTGCCAAAGGCAAGNGGCACCGTTCTAGTCGCACTGAGGAGGAGAAAGCTCANAAGAGACAGCGTCGGCAGGAGAAGGAGGCTAGGAGGGCTTCGATTGTAGACGAAGAGTTGCGCCAGCAGAGGGTGCGTGAGAGNGTTGCGGGGGCATCGAGCTCTGCCCCAGTTGCAGAGGTCCNGCCTGTTGTGAGGGACGTTGTGAGCACCACTGATGGTGCGGTGAGAGTGATNGAGAGCACTACTGAGGGTGCTACGATTGNTGATGTGGGCACTACTGAGGGTGNCCCNACTGTTGTTCCAGCGGGCTCCGGGAAACCGGACCCACCCGCTTGTTGATGATTCGTCGGCGCTNTGCGCCACAGGTTTGCTTCACCCAATCCATTNTCTTTTACTGTTTTTGTATGCATTGGGGACAATCGCATCTATTTTTGTTGGGGGTGGGGTAAATGGATTGTGAGTGATAGGGTGAAGTCTGAGTAACCCAACTCATGAATCCTCTCTTGGGGTTTTCTTGCCTGTGTTCTTTTCCCCCAAGAGACTGTTTAATTTCTGTTGAACCGGCATGTGTTAGGATCGTATGTGTAGAAGCATGANAAAGAATGAAGCATGATGGCTTATGAAAAATGATACCCGTCCAAATTTTGTGATGTGTGCTAGAATTTGTAGGCTANGATAAGTTGAATGTGTTGGCTCTGAGTATGACATGATAATGAACCAACTTGATGGCATGACTTAAGCTGAAACTTGAACTGGGTAATCTGATAATCTGATAATGAAACTATGTGCCAAGAGTGTGTGAGAATAGTTGANTGTTCAAACAGTTTTTGTGCNAANCTAGAACTTGCCCGGTTAGTCCTGCTAAGACAATTCANTCTAGAGGTTAGGAAGTGATCATAGGCCCTTGTTCTGATAA

>rnd-1_family-120#LTR/Gypsy ( RepeatScout Family Size = 662 Final Multiple Alignment Size (possibly truncated) = 98 Localized to 2361 out of 4218 contigs )

TCAACGACTTTTAGTAAATAATAATCACCCATTNAGCGAAATTGGCGTTCAATTGATCACACTCCCAAGGACCCCTCTCTACTTGNTTACTCCCGGTTGTTCGTAGTTGTTAATTGNTCACTCAAATCCCCCCATTTGACATTAATTGTCAACCCCTTGTNATTTACTTTATTGTTTCGAAAATGTCTACNTCTACAAACGATTAGAACACTATTTTACTTCGTAATCGAATTCTTTACCGTATCACTCCCTGTGGGATCGACCCCAACTCTTCGTTGGGTTTATACTTGNTAGCGACCGCCTACACTTAGAATCGGATGAAGTGTAGTTGAGCGTTATCAAAAATGGCGCCGCTGCCGGGGAGTGGTGTTTAGAATTCTTTTATTGAAGTTTAGTTGTGANCTTATTTTGTTGTAGTTGAATCGACTTATTTTTGTNTTTTGTTGGTTGCTNTTGAACAGGTGAAGAGATGAGCGTNAACGGGAGCAATGGTAGCCAACTTGGNCACCAAGACGACATCGGNAACTTGAACGATGTCAATGAAGATCAATTGGGCGGTGTNGGTGCCATTCGNTTGCCTCCGGCCGAGGGGAACGCGGTGTTCCACGTNACGAGCACNATGTTGCAGCTCTTGCAANTGAAGGGNCTNTTCGGNGGGCTGGCTCATGAGGATCCCCATGAGCATATTCGGAACTTCGTGGATGTNTGCGGNCCGTTCTCNTTCAAGAACATNTCNCAAGAGTCGGTCCGNCTNAGGTTGTTCCCNTTCTCTTTGATGGGNGAAGCGANCAAGTGGTTGGCCGAGTTGCCAAGGGANTCNATCACTTCNTGGGANGAGCTNACCGACGCATTTCANGTGAGATTTTTCCCTCCCTCGAAGATGATGACNCTNAGGGATAACATCCAAAGCTTCAAGCGNTTGGAGGGTGAGCCAATCCACGAGACGTGGCTGAGGTTTAAGAAGTTGGTGCTNCAATGCCCAACTCACGGNCTNCCNGACAACGTGTTGCTGCAATACTTCTACCGGAGCCTTGACTCGGTNAACAAAGGNGTNGCTGACCAACTTNCTCCGGGAGGNATAATGCAACAACCNTACGANGTAGCGTCTACTCTTCTTGATGGCATGACCAAGATNAACCGGGCGTGGTACACTCGNGAAGACCAAGTCTCTCCTCTCACNTTTAGGNTGACAAAGGAGCAGATCGAGAAGGACCAAGAGAGGGACCAAAACATGGCCAAGATGATGACCCAACTGGAATTTTGGCNAAAAATGTCATGGGGAGTGTTGGAAAACAAAGAGGCGAAAAAAGTGTCTAGACTNGAGGAGGGTTCTTNCCCGGGCTNTTTAAAGTCGGGCGGGAATCAAGGTTGGAACACGCNTAGNGAGGATGGACGGAGAAGTTACAATCGAGATTGGGCTGACCAAAATAACTTTTGGAGGAGAGAAGACGACNATGATGCGTGTTACATGCACATGGGTGATAGCCCAAGATCTAGGGACAGTTCTGGAAGCTTTCGGGTGGATGATTTGTTATCCCGCATTCTAAATAAGGTNGAAGGATCCGACGAAATGCTTAAAGAAATGAAGGCTGACTTCTCATCCTTGAATAATAAAGTAAACTC

>rnd-1_family-100#LTR/Gypsy ( RepeatScout Family Size = 746 Final Multiple Alignment Size (possibly truncated) = 98 Localized to 2361 out of 4218 contigs )

GAATCGGTAGACCTAATGTTGGTAGCCCTTTCATAAATGANTAATGAAGTATTCTAGGCTATGTCTTGAACCGGTAGACCTAATGGTGGTAGCCCTTCATGATGAGTCTAGGAACTAATGTAATGATGAACCTTGAATCGGTAGACCTAATGGCGGTGGCCCTTTCATGTGTGAATGATGAACCTTGAATCGGTAGACCTAATGGCGGTGGCCCTTTCNTGAGGATTCAATGAGCTATCCTAATAATGTACCTTGAATCGGTAGGCCTAATGGCGGCGGCCCTTTCATGTGTAATAATGTACCTTGGGTCGGTAGGCCTAATGNCGGTAGCCCTCTCAAGTACAATAATGTTAATGTGAATGAACTACTCTATGGGAATGNAGGCTAAGCACCGAGTGGATATGGTAAGATGGAAGCTCTCCCAACGTTAGGTCGGGTTTCAATGAACATCTTCCTTATCCCATAACTATGTGCCCACATAGGATATTAGCTAGTGGATCCACCTAAGCTAAAATGTTACCGGTCCTACCTTAGGCAAGTAGACCACCTCTTTACGGTGTGG

>rnd-1_family-236#Unknown ( RepeatScout Family Size = 395 Final Multiple Alignment Size (possibly truncated) = 98 Localized to 2361 out of 4218 contigs )

TTTTAAAGGGCTTTTGGGTCTTTTCCTAATTGTTTTAACCCAATACTATGTCGTTTTACCTTCTACCCTAAGCCCTATATAAGNNTTTTAACCCCNAAATCACCCANTCTNAATTCATTCTCCTAAATTCCCAAAANCCGACCAAGTTCATCCTCTCAAATTCTCTCAAGTCTAAGGAAGAAGAAGAAGAGCTAGGGTTTCAATTCAAGTCTCCAATTCNCCATTGNTTTTGGGCTTTTGGCATCAAGGTATGNTAGTATTCACCNATGGAGTCCTTTCCTCCATNGGGTCCCCAAAGTTCTCCAATTTC

>rnd-1_family-455#Unknown ( RepeatScout Family Size = 147 Final Multiple Alignment Size (possibly truncated) = 98 Localized to 2361 out of 4218 contigs )

GTCAATATGGGCTAGGCCCGTTGGGCCGGCCCGGCCTAACCCGTGATTTGATAGGGTTGGGCTACGATTTTTGGAGCCCATTTAAGAAAAGGGCTTTTTAGCCCGGCCCGAATAAGCCTGCCGATTTGTGGGGCTTGAGAAATATGGATAGGGCCGGCCCGTGGGCCAAATAAAAATAAAATAGAGTATTAAAAATAACATAGAGTCTAAACTCAAAATCTGTTTAAAGTTCGAAATATTACAATTTAAATACAAATTATGTTTATAAAATATGTACTACATAAAATAAAAATTCCCTAAAATTTCTAGGGAATCACTACATAGGCCGTAGCCTATGGAATATTGTCATAGTTTTTGTGTTTTATTATGATCATTGGAAAACAATTAGGTTAATATTTCTATTTAGCTATTTATGCTTTTACTTGAAATCAAACTTAATAAATATTATAAAGATAATTTTATTTGTGGATTTGATTAACAAGTAGTAACACTAACACCATGTAATATTGTCTTGTCGATATTTATGATAATATTTTTAAAATTATAATTTAATTTAAAAAATTATAAAAAATATACTTTTAAAAAAAGAGGGCTGGCCCGGCAAAGCCCGTAGCCCACGTACTTGTGGGCTGGGCCGACCATTTTCTGGCCCACACCAAAAATGGGCTAGCCCGGCCTGGCCCGTCAAATTTCAAAGCCTGTATGGGTTAGCCCGGATGGGGTGGGCCAGCCCATATTGA

>rnd-1_family-88#Retroposon ( RepeatScout Family Size = 807 Final Multiple Alignment Size (possibly truncated) = 98 Localized to 2361 out of 4218 contigs )

AACAATTAATAACATAAGCANAAATTAATGACTGTAAAAAACATACCAGAATCTGTAACAATAGAATGAAACAGAAAGGAAAAAATCGAGCCCACTGAATGCACAGTGTCCCCTTAAGGAAATTATTCCCCTCTAGTACCCGAGGTTTAATGGAATATATCCTCCCAGGATAGAACGATCTTANTCACCAGTGTATTGATACCCAAAACGATGGTGTCAGCGAGCCACTCAACGGNAGTAAAGTACACGAATATTTAATTGTGCAGAAGAAGAAGAAGAGTTCAAAATTTCGTTGTTTTAAAATGAGAGGAAATCCCTCTATTTATAGACAACAAAGGGTAGTGTGAACAAATGTTTATTGTGCCTTATCGGAAAGGTCACAACCCTTCGGAAAAGTCGCAACCCTTCGGAAAGGTCACAACCTTTCATAAAAGTCGCAACTTTTCATAAAAGTCGCAACTCTTCATAAAAGTCGCAACTTTTCATAAAAGTCGCAACTTTTCATAAAAGTCGCAACTCTTCAT

>rnd-1_family-43#LTR/Gypsy ( RepeatScout Family Size = 1132 Final Multiple Alignment Size (possibly truncated) = 98 Localized to 2361 out of 4218 contigs )

TTAGGCCGGGTTTCAATGAACATCTTCCTTATCCCATAACTATGTGCCCACATAGGTTATTAGCTAGTGGATCCACCTAAGCTAAATGTTACCGGTCCTACCTTAGGCAAGTAGACCACCTCTTTNCGGTGTGGGGTATGACACCGGATTCCATGNTTAGCTCNCATGGTCTATGTCGGTTAACGCTTATTCCCATCATGTGAGATGTGCACTATGGTTTCTCGAGAAGTTCTACGAAGTGTGGGTGGTAGTATGGGATGCCATCCATGCATTGCACGAGTAGGCTTTGAGAGGGCTATGGTGAGTTCTCTAATGTCTTAATGACTAATGAATGAATGTGCTCTNAATGAAGCTAATGAAGAATGTTGACTTAAATGCTTAATGTAATGATGCATGCTNTACTTGGATTGTATTATGAGTTACTTCCTTGTCATGACTTATGAAATGTGTATGTTCCTTATGTATGAGTATTGNCTAAAGATGAAATGGAAGAATGGTAAGTTCACTTTTGGTGGCCTTAANGTGGTACTTAGTGTGGATGGTGGTATGGGACGCTATTCATACATTGCACAAGTATAGCTTAGGGTTACTTGAGGTGAAGGTTTAATGTGATGTAATGACTTATATGTTGATTATGCTTGTATCTTATGCCTCTTATACTAANGTTCATGATGTTTTCTCAAATTGGCATAANGCATGGTTTTCAACCAAAATGTCCTTTTTAAGCATGGTTTTTGCATGGCTATCATACTTAGTACATT

>rnd-1_family-464#LTR/Copia ( RepeatScout Family Size = 143 Final Multiple Alignment Size (possibly truncated) = 98 Localized to 2361 out of 4218 contigs )

CAGGTCGTGGTTTTACTCCCTTGAGCAAGGAGGTTTCCACGTAAAGTTGTTGTGCAATCTTTACTTTCAGCATTTGTTTACTTTCTGTTATTGTAACTGTGTCGAGGACCTGGTCCCATCGACGACAGTGGACGCATACATTCCAACAAGTGGTATCAGAGCTTGACTTTTCTATCTGGTTAACACCAAGAAAAGGAAAGATCCTGAAGGAATGGCTGCTCCACCGAATNTGGAGGAAGGNCAGTCTTCTACCAGACCACCTCGTTTCAATGGNCAATNCTATGGNTGGTGGAAGANTCGNATGCACGACTACATCAACGCTGAGGACACTGAGCTGTGGGACGTNATTCTTGATGGACCNTACATTCCNACNAAGGANGTGAAGGATGGAGAGCTCACNANNGTCGTTCCCAAAACCAGAAAAGAGTACGATGAGGCGGACAGGAAGAAGATNGAGAAGAACTACAAAGCNAAGAAGNTNCTAGTGTGTGGNATCGGNGCNGATGAGTACAACCGAATCTCTGCNTGTGAGACNGCCAAGGAAATNTGGGACTGTCTNNAAACAGCCCACGAAGGAACNNAGCGAGTGAAGGAGTCTAAGGTNGATATGCTNACNACTCAGTACGAGAACTTCANCATGAAGGAAGGTGAAACCATTCACGAGATGAACACAAGATTCACCTCCATNACCAACGAGCTAAGATGCCTTGGTGAGCCTATTCCCNCGAGCAAACAAGTTCGNAAGATTCTCGAAGTNCTTCCCAAGTCNTGGGAAAGCAAAGTGGATGCCATNACNGAAGCAAAAGATCTGAAGACGCTTNCCATGGATGAACTGATTGGGAACCTTCAGACCTACGAGNTGAACAAGAAACAGGGGACAANCGTGAAGGAGGGAAAGAAGGAGAAGTCCGTNGCTCTGAAAACGTCTCAAAGTGATGCGACTGAGGAGGAGGATGAGATGGCNTATNTCACTAGAAGGTTTCAGAAGATCGTNAAGAAGCATGGAGGCTTTCAAAAGAAGGCCTCGACCAGCAGAGCTGCAAATGCAAATGACCTTTGTCACAAGTGTGGNAAGCCTGGTCACTTCATGAGGGACTGCCCNAGCCAGAAGCAGGAAACTCANGACTTCAGACCTCGCAGGAGGGACCTGGTCCCAGACCATGCCAAGAGGAAGGCTCATGCTGATCANNTGGTGAAGAAAGCCTTTGCTGTNTGGGGAAACGCNTCAAGTGAGTCAGAAGAAGATGCAGAANGTCCCGAGGATGTCTCGATGATGGCCGTTGAAGATGATGAAAACGTNTTCGACTCCATTTTCTCCCTNATGGCAAAATCTGACGATGAAGAGGATCNAGACGAGGTAACTCTTTTTGATCTCAAANATGATCTAGATACTCTTCCCGTTAAAAGATTGAGAAAACTTGTTGCTCTGCTNATTGACTCCGTTGATGAACTAACCACTGAAAACTTGACGNTGAATGAAAAATTGAGTTTGTGCGAGGATGAGAACTCAGCTCTCANNTCTCAAGTNTCTGAAATGAGTGTNAGGATAGGTATTCTAGAGACTGANAATCTAGAACCCGAGGAGGANCCTGGTACCTCNGAGGGTGGAAAGAGAAAGCTCAGCANCTTTGAGGTNGAACTGGAAGAGAAGCTTAAGACCTCTGAGTCTAAGCTAGTTGCCTCCCTNGAAAGAAATTCTCAGCTGGAGAAAGGACCTGGTANGGTCAAAGAGGAGCTGAACCACTCTCTNAAATGGACCGACTCCTCTAAGATACTCTCTAACNTANCTAGTCAAAGTTNCAACGGNAGGAAAGGGTTAGGNTGTAGACCGATAGANCCTCCCTACAATCCTCACAGCAAATATGTGTCTGTNTCTGACAATCTGTTGTGTACTCACTGTGGNCGNAATGGTCATCTGAAAGAGAAGTGTGAGACTCTGANAAGAGCAAAAGAAAGGCANGNGAAATTTGTCAGATC

>rnd-1_family-255#LTR/Copia ( RepeatScout Family Size = 349 Final Multiple Alignment Size (possibly truncated) = 98 Localized to 2361 out of 4218 contigs )

AAAGACAAAGGNAAGAGTCAAGCAAACATCGCGGAAGAGATGGAAGATGCAGACGACCTCTGTGCAATGATCTCGGAGTGCAACTTAGTTGGAAATCCCAAGGAGTGGTTTCTCGACTCNGGTGCCACTCGACATGTTTGCTCCGCGAAAGAAGCCTTTGCAACGTACACTCCTGCTGAGTTCAGACGAAGATTTGTTCATGGGGAACACAGCAACAGCAAGGATTGCAGGAACTGGGAAAGTNATGTTGAAGATGACATCCGGCAAGGTGTTGACTCTGAACAACGTTCCGCACGTTCCTACNATTAGGAAGAATTTAGTTTCTGTTGCACTGCTCGTTAAGAACGGGTTTAAGTGTGTCCTNGTTAGTGACAAAGTTGTAATAAGTAAGAATGAGATGTNCTTAGGAAAGGGCTACCTCACTGAGGGCCTCTTCAAACTGAATGTAATGGTTGTTGACAGTATNAATAAGAATTCTGCTTCTNCTTACTTGCTTGAGTCAAACGATTTGTGGCATGANCGTTTGGGACATGTCAATTACAAAACCTTGCGAAAACTGATTAACTTAGAAGTTTTGCCTAACTTCGAGTGCGATAAATCGAAATGTCAAGTTTGTGTTGAATCTAAGTATGCTAAGCATCCTTATAAATCTGTTGAAAGGAATTCNAATCCCTTAGANTTGATTCACACTGACATTTGCGATATGAAGTCAACACCATCTCGTGGTGGGAAAAAGTATTTCATAACTTTTATTGACGATTGCACTAGATATTGTTATGTCTATTTGCTGAATGGTAAGGATGAAGCAATNGANGCGTTTAGGCAATATAAAANTGAAGTTGAAAATCAGTTGGATAGAAAGATAAAAATGATTAGAAGTGATAGGGGTGGAGAATATGAATCTCCTTTTGCAGAAATATGTTTGGAAAATGGAATTATCCATCAAACTACTGCTCCCTACTCACCTCAATCTAATGGAATNGCGGAAAGGAAAAATCGAACTTTGAAGGAAATGATGAATGCCTTGCTTATAAGTTCAGGTTTACCGCAAAACTTGTGGGGGAAGCTATCCTTACGGCAAATCGAATACTCAATAGNGTGCCTCGTAGCAAGACACAATCTATTCCATATGAGNNATGGAAAGGAAGGAAACCCAACTTGAAATATTTCAAAGTGTGGGGGTGTCTAGCCAAAGTCCAGGTTCCTATACCTAAGAGGGTNAAAATNGGACCTAAGACTGTGGATTGTGTATTTATTGGATATGCCGCGAACAGTAAGGCCTGTCGATTTTTGGTTCATAAGTCCGANCATCCGGATATTCATGANAATACGGTAATTGAGTCAGATAACGCTGAATTCTTTGAACATATCTATCCGTATAAAACTAGACGTGAGTCGTCAAGTGAAGGGTCTAAACGACCNCGGGAAGAACCAAAGGAGAATGTACCTAATGAAGAGAGTCCGAGGCGTAGTAAACGTCAANGGAAANCTACTTCNTTCGGACCTGATTTTGTAACGTTCCTTCTTGAAAATGAGCCTCAAACATTCAAAGAAGCTATGTCTTCTATNGACTCAACCTTTTGGAAAGAGGCTGTCAATAGTGAGATCGAATCAATCTTAAGCAATCATACTTGGGAGTTGGTTGATCTTCCTCCAGGAAATAAACCTTTGGGTTCAAAGTGGATCTTCAAAAGGAAAATGAAAGNCGATGGAACTATTGACAAATATAAGGCNAGACTTGTTGTCAAAGGCTTTAGACAAAAAGAAGGTCTTGATTATTTTGATACATACTCGCCAGTAACNAGGATTACATCNATTCGGATGTTAATTGCNCTAGCTGCGGTATATGATCTTGAAATTCATCAAATGGATGTGAAAACNGCCTTCTTAAATGGAGAGTTGGAGGAAGAAATTTACATGGAACAACCCGAGGGNTTTGTGGTTCCTGGTAAAGAAAAGAAAGTGTGCAAACTTGTTAAGTCACTTTATGGACTAAAACAAGCACCCAAACAATGACATGCGAAGTTTGACCAAACCATGTTGGCAAATGGATTTAAGATCAATGAGTGTGATAAATGTGTTTACATTAAAGACGCTCCGAATCACGANGTCATTGTTTGTCTATATGTTGATGACATGTTGATAATGAGCAAAGACATTGCCGATATAAANGCTACTAAGCGTATGCTNGCTAGCAAGTTCGATATGAAAGACCTNGGAGTTGCCGATTTGATCTTAGGAATCAGGATTCACAAAAC

>rnd-1_family-465#Unknown ( RepeatScout Family Size = 143 Final Multiple Alignment Size (possibly truncated) = 98 Localized to 2361 out of 4218 contigs )

TTTTAAGTTTATTATTTTATTCTTTTAATTTNAATATTTTTAAAATTTAAATATGNTGTTCACTCGCCTTAANACGCGTGTAGTCACGCACTCTTGCCAACTCAGCNATATTAATGCCACATAAGCTCGGTCAATGGTCAGAGGGGTTTAAAATATTGTGTTTTANTGAGTTTAAGGGTTCAGTTGACAAAGNGNTAAGTAGAAGGGTTCANAATACAAACGNATACAAGTACAAGGGTCCACCAGACCATTTCGCC

>rnd-1_family-396#LTR/Gypsy ( RepeatScout Family Size = 188 Final Multiple Alignment Size (possibly truncated) = 98 Localized to 2361 out of 4218 contigs )

TTTCTCTGAAATTTGGGTCAAATGTCTCGGANGTTTTCTCCCAATCTATAAAGAGTTATGGGGCCCGCGACCTATNAAATCGAAGGTCTACGAGTCTAGTTTCCAACGCATCAAACCGTTCGTTCATACGACTTCAGAATAGAGAGATATTCGCGTTTTCGCGAGACTGCGCAAGCAGGCACGTGAGGTGGGTCCCCTAGGTCGGTGGAACTTTATATATATCNTCTTCTTCGACTTTTTCTTCATTTTTTCTTCATTTTTNAGCTAAGAACCAGAAAAATCAGANAGAAACCCACTTCTAGGATCAAGCGAAGTATAGATTTTCTCGACTTAAGTCCTTGACGAAAGTTGTTCTACGCGTTGGGCTCGTCATCGTGGTATAATTTGTTTTATCGATTTCGTAGCATATCGCACTTTGGTGTTGGGACAGCAAGGGTTTGGACATATTTGAAGGTTTTGAGGCGTATTACGAACTTAAGATCGAGGTAAGACCCTTCTTTCATCGACTCTAGCTTTNATAAGCCGCAAATAGCAATTTGCGACGGAAATACATATCTTTTATCGNATTGTCGTGGTATCTTCTATTCTTGTGTGTTGATTATGGTCCTGCCTTGTTGTAGTATCGAGGGGGTTGTGAGTAAAAGTCGTTAGGCCACGTGAATTCAGCGAAAAAGGTATGTTAAGGCTATTCCCTACTTACGGCATGTTTCCTTAAAGCTTAGGAGCAATGTAATTGGGTTGTTATCCTTGTTATACTTGTAGCCGTTATTGTTGATTGTTGGCTGCTC

>rnd-1_family-157#LTR/Gypsy ( RepeatScout Family Size = 562 Final Multiple Alignment Size (possibly truncated) = 98 Localized to 2361 out of 4218 contigs )

GTTTTCACTTTTGAGAAATTGGAAGTGGGTCTTTGAGATTCTTCGANTTGGAGCATTGTAAAGACGAAATCAATCTTACCCAGTTGATGGANAATCGATTTGGTAACGGTTTTTATCTTTTTATGATGTNTGGCTAAAACCCCAATTCTTGGGGTGTGATCATGTGATTATGGGCTGAATTAGCTTATGGGTATTGTTAATTACTAGTTTAAATGCTGTTTTGAAGTGGGTTTAATCGNTAGTTGTGGTTTAATTTATGAATTGTAGTTGCAAATGCAGTTCTANCTTNGTGTTCTTGGCTTGCTCGAGAGAGAGGTTTTAGAACCAAGGTTATCGATTGATGATTTGTGGGTATTGGGTTGNCGTGGGTTCAGCTCGAGAGAGTGAATCCTAAACCCAATCCTACCCATCTAGCTCGAGAGA

>rnd-1_family-150#LTR/Gypsy ( RepeatScout Family Size = 583 Final Multiple Alignment Size (possibly truncated) = 98 Localized to 2361 out of 4218 contigs )

AGGGTCAACTTCAAACGGTCATATCTTTTAGCACAAAACGAATTAGGTGGCCCATGACCTATCAAATTAAAGGTCTTTGAGTCCTCTTTCCAACGCCACCGAGTTTGCCNAATTCCGANCTCGGAGTAAAAAGTTATGCCCGTTTTAGTGAAGCCCTGTC

>rnd-1_family-3#LTR/Gypsy ( RepeatScout Family Size = 1720 Final Multiple Alignment Size (possibly truncated) = 98 Localized to 2361 out of 4218 contigs )

CAGATGGCCCCTTATGAGGCATTGTATGGGCGTAGATGTAGATCTCCAGTTGGTTGGTTTGAAGTAGGTGAAGCAGCCTTGATAGGGCCAGATTCGGTCCATGATGCTATGGAGAAAGTGCAACTCATTAGAGATAGACTTAAGACAGCCCAGAGTCGCCAGAAGTCTTATGCAGATGTAAGGAGAAGGGAACTAGAGTTCCAAGTTGATGATTGGGTTTTCCTGAAAGTGTCACCTATGAAGGGGGTGATGAGATTTGGCAAGAAAGGGAAGCTCAGTCCCAGATATGTAGGCCCTTACAGGATCTTGAAAAGGATTGGTAAAGTGGCTTATGAGTTAGAGTTGCCAGCAGAACTAGCAGCAGTGCATCCGGTCTTTCACATTTCGCTNTTGAAGAAGTGTGTGGGTGATCCAGCATCCATAGTGCCATTAGAGAGTGTGGCTGTGAAAGATAGTCTCNCTTATGAGGATGTACCAGTTGAGATTCTCGATCGTCAGGTTAGAAGGTTGAGAAACAAAGAAGTCGCTTCAGTCAAGGTTTTGTGGAGGAGTCAGTCCGTAGAGGGAGCTACTTGGGAAGCAGAAGCAGCCATGAAGGCCAAGTATCCTCACCTCTTTCCTTCCGATTCCACTCCAGCTTGAGGTAATAGTTCCTCTTCAGTTTTTCAGTCATTCATGCGTAAATTCAGTCTTAGNATCATGTTCCTTCAGTTTGTACTTGCATTTTCAGCGTATTTGCATGTTCTTGGAACTCAGTTCAGTCAGAAATTCAGTTCTCAGTGTTTAGTGGTAGGGATTGCAGCTCTCTCCCTCCATTTCAGCTAGTTTAGTCTTCATTCGAGGACGAATGTTCCCAAGGGGGAGATAATGTAACACCCCGTATCCGAAACAGACCAAAAATGCAGATTTCGAGAAGTTGCAGGTGCAACCCACGGTCGCCACCCACGGACCGTAGGTCGGACCACGGCCCGTGCTGGTGGTCCGTGGTTCGCCACTGCAACCCCTCCCAGACCCAGCTCAGAAAATTGGCTAAGTCTCGACCCACGGCCGGACCCACGGTCCGTAGGTCAGACCACGGTCCGTGGTCCGTGTCCGTGGATCGAGACCTCCTTTACCCAGCCTCTGACACGAACTACGGTCGACCAGCACGGACCGTCGTTCGATCCACGGTCCGTAGGTCTGACCGTAGATGAGGGTCAGCAGCCAGTTAGATGAAAATTATTGATGGGTCAACTTCAGATGGTCATAACTCTTAGCACAAAATGAACTCAAGGGTGCAGTCCATCAACAGAGAGTTGAGGTTTTCTCCCAAGGGGGAGATGGTGTGCTTCGCTACCAGGGTCGATTATGTGTTCCTAATGTGGGCGAGTTGAGACAGCANATTCTTGCAGAAGCCCATAACTCCAGATATTCTATTCATCCAGGCGCCACTAAGATGTACCGCGATCTGCGGGAAGTCTNTTGGTGGAACGGTATGAAAAGGGATATAGCAGATTTTGTGGCTAAGTGCCCCAATTGCCAGCAAGTCAAGGTAGAACATCAGAAACCAGGAGGTATGACTCAAGAGATCGACATTCCTACTTGGAAGTGGGAAGTGATCAACATGGACTTCATCACAGGTTTACCTCGTACTCGCAGACAACATGACTCCATTTGGGTGATAGTTGATAGAGTTACTAAGTCTTCTCGCTTTTTGGCGGTCAAGACTACAGATTCGGCGGAGGACTACGCCAAGCTTTACATTAATGAGATNGTCAGGTTGCATGGGGTTCCTTTGTCTATCATCTCAGATAGAGGTCCTCAGTTTACCTCTCATTTCTGGAAGTCGTTTCAGAAAGGTCTTGGTACTCAGGTTAATCTTAGCACAACATTTCATCCACAGACGGATGGTCAGGCAGAGCGTACCATTCAGACCTTAGAGGACATGTTGAGAGCTTGCGTGATCGACTTCAAGGGTAGTTGGGATGATCACCTTCCTCTTATAGAGTTCGCCTACAATAATAGCTACCATTCCAGCATTCAGATGGCCCCTTATGAGGCATTGTATGGGCGTAGATGTAGATCTCCGGTTGGTTGGTTTGAAGTAGGTGAAGCAGCCTTGATAGGGCCAGATTCGGTTCATGATGCTATGGAGAAAGTGCAACTCATTAGAGATAGACTTAAGACAGCCCAGAGTCGCCAGAAGTCCTATGCAGATGTAAGGAGAAGGGAACTAGAGTTCCAAGTCGATGATTGGGTTTTCCTGAAAGTGTCACCTATGAAGGGGGTGATGAGATTTGGCAAGAAAGGGAAGCTCAGTCCCAGATATGTAGGCCCTTACAGGATCTTGAAAAGGGTTGGTAAAGTGGCTTATGAGTTAGAGTTGCCAGCAGAACTAGCAGCAGTGCATCCGGTCTTTCA

>rnd-1_family-32#LTR/Gypsy ( RepeatScout Family Size = 1204 Final Multiple Alignment Size (possibly truncated) = 98 Localized to 2361 out of 4218 contigs )

AAAGCTAAAAGAAAAAANAAAATAAAAGTAAAAAGAGCTACAAAGTCTAAAGTCACATCCGTGGTTGAAGAAAATCAAGGGAAAGAAGGTAAAACGGTAGGATGNCAAAAATAGGGCAAAAAGAGGTTGAAAGCCTAGTGTAATGTCAAGGAGGGCAGAAAGTCACTAAGAAGTACCCAAATGTACCACACCTGACCCTGAGCCTACGTTACAAGCCAAGAAAGTCCTATAGTGATCCTAGAGTCTAGTTTGGAGAGTCTAAGCAGTGAAAATAAGGGCAAGCCTATGGTATTGAGCACTAACTGTACTTGAAGTTACTTCTGAGCGTGAGTGTTGAATGAATCCTTGTACCACGAATGTCATTCATTGTTGNGAAAAGGGGATTTCGTTTGAAGTAAGGGCACTAGTTACATTGTCGGAAAGTTGGTACCTTGGTGATAGTGAAACGGTGTATGTTGTGTGTCGGTTGGTCGATCTTTGTCATGNTTTGATCCGCACGAATTAGCTTGTCGAGTCTAAGAGAACGAATTTGCACCCGAANAGTGAGTCGGGGTAGAGAGCCATGTGATTGCTTGTGCTTGAAATGCTTGCTTCTATACAAGTGTCGTTTAGAGTCTTAGTGCGTCGCTTGAGGACAAACAACGAATTTAAGTTGAGGGTGTTGATATACCGTGAGTTTACGGTATTTCTAATACATTTCACTTACGAGTTGTGTGTGTCTAGGGCCTTTTTGTATTGGTTTTTATGTGTTTTTATCATGTTTTGCAGGAAAGACGTTCAGGCGCGGAAGTANAGAGACAGCTGAAAGAAATGCAGAAAAGGGCATCTACGGAGGTGATCTACGGACCGTAGGTCCATTCACGCTCCGTAGGTGATGGCCGTAGATCGGCAGGCAAGGCATGGAGGACAAATCAGGGAAATCTGACCAAGTGTGGAACCACGGTGNCCATTGACGGTCCGTAGATTGATCTACGGACCGTACTGTTGATCCGTAGANTGATACTGCGAGGGTTCCAGTACCTGATTTTTGAAGATTCTAAGTGTGGAGCTACGGAGGGGATCGACGGACCGTAGATCGATCTACGGTCCGTACTGCNGGTCCGTCGTTTGCTTCAGAGAAGCTGATTTTTGGAAGCTGAAATATTTTCTAAGTCCTGACCGACGGAAGGGACTTACGGACCGTAGATCCATCGACGGTCCGTAAGTCAGTCTCGTGGATCGAAGACGCGTGCCTGAACAAACTTTCCTTATTTCGTTTCCCTTTTAATTTAGGACTTGTTTTCTATAAATAGGGCATGTAAACCTCGTTTTTGGGGGTTAGACNTTATTACTTTAGTTCTAGTTCGTGACTTTGGAGATTAACTTGCAACCCTAGCAATTCTTGATTTCCTAAGTTCGTTTTGAAGATTTTGGCTTTGCAATTCAAGTTGAANTTCTGGGTTTATTATTCTCNTTACGTAAGTTCATGATTTCTTCATCTAAAANTATGAATTGTGTTCTTGCGATTATGGGTAACTAAACTCCACAACTAGGGTTGTGGGAACCATGAGCGATTAACAAAGTATGAATAGTAAATAAGCAATTCTTGAATAGTGTTTTGCATGCATTGATAATTCTTTCGTTTAGAAGTCTTTTTAACGAGTGCACGCGTTAGAACTCGCCTTGTTGCTACTTGCCGGACCAAGGAGGTAATCAACAAGAAAAGAATTATCAACATAGATTTAGTGTGATACTATCTAATAGNCTAGTGTCGATTGGTGCGAAGTAATAACTAAGCCAAACATCGATTGTGATGTCTAATATGAGGTAAAGGTAAGGGTTAGTAAATTATACACACGTAGCCGGACCAAGGTGCGGGGTGAAATTCTCTAGATGCCGGACCAAGGATTTAGAGATACCTAACTTATCACTTTGCATGTAATACACTAGGAAAGGATTGCTATTACTAGGATTACCGCGTTATGAGCTTGTGGGGAACACGTACACCCTAGTTANTTCTCTCATCTTGATAACAACCAAAGTTGANTTTTGCTTACTGATTACTTACTNAATTCTTACAATTTGTTTCACA

>rnd-1_family-570#DNA/CMC-EnSpm ( RepeatScout Family Size = 103 Final Multiple Alignment Size (possibly truncated) = 98 Localized to 2361 out of 4218 contigs )

CACTACTAAAAAACTGCCGAAAAACGACGGCCAAAAGCGACGGACTGCGTCGCTTTTTCGGTCAAAATCGACGGAAAAGCGACGCAGTCACTTCCGTCGCTTTTTAGGTCGTCGCTTTTCGAAAAGCGACGGACTGCGTCGCTAAAAAGCGACGGACTGCGTCGCTTTT

>rnd-1_family-229#LTR/Gypsy ( RepeatScout Family Size = 410 Final Multiple Alignment Size (possibly truncated) = 98 Localized to 2361 out of 4218 contigs )

TTGTCAAGGTATGAATTGGCAAGGTGAGAAATGATCGATTGTATCCGGTATTGAGCATTTGAAAGGACTTACGCCTGGGTTGAGTCGTAAAGTTTATTACTNTTGATTGTTAAAGTACCTAAGAAAGGTTTCATTTAAAAGTGTTGGTTTANAATAAGGTAAATGGTCGGAGTGTTTATGATGGTTGTTAGTATTGTGAGTATGATTCACTAGCTACGCTCNCGCGTGTTTTCACTTTTGTGGGTGGATCCCTAGTGGTGGATCGGGTGTACCGATCCTGCGATGTTCTCTTGGTTGAGTGGGATGATTGGGAAGACTTGGTTATTTCGGATGACATANTTGGTGTTGTCTTGGAGTTGTTAAAGTATTTTAGTGGTTCTACACGTACCACCTTGCTTATGAGAATTTATAGAAGAGCTACCATGCGTATTTACGGTTGTCCGACAATCTTAATATTCATCTAGTCGTCGAGGTCATGTATCGATTATGTGTGGTTGAATGTGGGGATCATTGGGTCCAATTCTACCGTTCTTAAGAGGTAATCTACAATTACTAAATCTAGCATTTCGATAGTGTTGTTAGAGGGTTTGTATGCTTGGACGTATCCCTCGATGAGTATCTGATGTCTAAAGATCAAATTTTTATAACTTAAGCTCGTGGAAANTTATGTTGTGAGATGGAGATGTTGATAAGAACTATGATTGTTTGGGTATTATGAGTGTGTGTTGGTTGGTTCAGGTTCACTTCTAATTGTAGCGAGTATTGATTTAGAATTTTATCATGGTTATGTGAGAAAGGATTCGTCCTGATGGTTGTGTCAAGGTCCTTGANAACATGTTGCAGACGTGTGTACGATCGACTTTGGTGGTTATTGGGAATCGTTTTGTCGTTAGTTGAGTTTACATACAACAATAGTTATCACTCGAGTATTGAGATGGCTCCATTTGAGGCATTGTATGGGAGGANGTGTAGGTNTCCAATTGGTTGGTTTGAGGTTGGAGATGTGAAACCCTTGGGG

>rnd-1_family-374#LTR/Gypsy ( RepeatScout Family Size = 212 Final Multiple Alignment Size (possibly truncated) = 98 Localized to 2361 out of 4218 contigs )

AAGAAGAAGAAAGGAGCAATTGGAGCTAGGGTTTGGAGGATTCAAGGTGTTTCTTCCTCAAATTTCTTGGGGATTCTTAATNAAGGTATGGTGGCTCTTCATCCTTGGTTAGCTTTCATCCAAGGAGCCNNTTCAAAAATGATTTCAA

>rnd-1_family-427#LINE/L1 ( RepeatScout Family Size = 166 Final Multiple Alignment Size (possibly truncated) = 98 Localized to 2361 out of 4218 contigs )

TGGACTCAACAGGGGTGGAATCTNACCTTTAGAAGGCTNCTNAATGACTGGGAGGTGGANAGAGTGGCCGANTTNCTAGGGATGCTAGGCGGAGTGACCATCAACGCAAATGCCACAGACAGGATGTTATGGAAACACAACAANGATGGGGNNTTCTCAGTNAACAGTGCTTACAAAAGAGGNCTCAGGCGACGGCAGGGAGACCGACGCACTTCTGGAAGNANATNTGGAAGGGCGANATCCCNACCAAAGTGAAATGTTTCACTTGGCTAGTGGCNAGGAGAGCNTGCCTGACNCANGAAGTACTNCAGAAGAAGGGNAGNCAACTGGTCCCTAGGTGCTTTTTNTGCGACGAGACAGGNGAAACNAACAACCATCTNTTCCTNCACTGCAAGNTNACTGCNCAANTNTGGAANNTGTTCCTNAGCATCTCAAATCCAGCTGGACNATGCCAGAACGTACNNCAGANCTNCTGAGNTGCTGGATNAGGAGAGGAGGNAGCAAGAGNCAGAAGAGATGGTGGAGGATNATCCCANCNTGCATNTGGTGGACAGTNTGGAAGGAGAGGAACGGNAGATGTTTTGAAGATAGATCCAATTCCATNCAGAAGGTTAAATGGAATTGTATAGTATCTTTACTTTTTTGGTGTAAANAGNANTGTATAGAAGATGTAGATCAGATTGTAGATTTNNTAGGANCTTTGTAATTGACACTTTTT

>rnd-1_family-66#Unknown ( RepeatScout Family Size = 973 Final Multiple Alignment Size (possibly truncated) = 98 Localized to 2361 out of 4218 contigs )

ATGACAAGANTATGAATATAACATGAACTATACTTATGACTTCTTAATGAGTTTTACTTAGTGTGAGTGGGGGTATGGGACTTCACTTATACATTGCACAAGTAGACTTTGAAAGGGGTTATGGTGTGGTTCTCTTATGCTATGAAGANATATGAATGTATGCATGTATGTTGAATGGATTGTTGCTATGAGTGACTTTCCTTATGTTCAAGTTAATGAACATGAATGTTTCCTTGTCTATGANTATTGGCTATGATGAGGTCTAAGGATGATATGTATGACTATGGTGGCCTAAAAGTGGTACTTAGTATGGNTGGTATTATGGGATCCTANCTATGCATTGCACAAGTATAGCTTAGGGTTGCTTATGTGATGGTTCTACTATGNTATGATGATCT

>rnd-1_family-24#LTR/Gypsy ( RepeatScout Family Size = 1303 Final Multiple Alignment Size (possibly truncated) = 98 Localized to 2361 out of 4218 contigs )

CTATGAGTGTTGGCTATGGATGAGGTCAAGGTATGTTATGCATGATCATGGTGGCCTAAAAGTGATACTTAGTGTAGATAGGATTATGGGATCCTATCTATGCATTGCACAAGTATAGCTTAGGGTTGCTTATGTGATAGTTCTATTATGATGAGATGATCTTGGTATNTGACTATGCATGTGAACTATGTCTTGTGAAGATTGGNATNTGCATTATGNTTATGTTGGAGACTATGCTTGTGATTATGATGATATCCTTTATGCTATGATGAATTGTGCTGAGTTGTCTTATGAGTTGCGTTCTCCAACTATTTCAAAGATGATGTTCAAAANTGGCATATAGCATGGTTTCAAACGAAATGTCCCTTTTATGCATGTTTTAAGGATTTTTATGCATGGCTTCCATACTTAGTACATTTTTGTGCTAACCCATATTTCTACATTTTCTACAAGTGTAGGGTTTGGAGATATCGGGTTCTACCCTCGTGGCTAGGTTTCTAGTAGATCAAGAAGACGAAGATTGGTGAGTCCTCATAGCTTCGAGGACGAANCCACTATTTCCTTTATGTCTTTCATTTATGTTTTAGACTTTTGTANGGGCTGTGTCCCTAAGATTGTATTCAAGAGTTTTATTCAAGTATTAGATGGTTTGAGACAAAGTGTCGTAGACTTCCGCTTGTTTTATGAAANGACTTCGATTGTAAAGAAAGTTTTAAATTTTCCGCATTTTTTCCTATGTTCTTATGTCATGATATGCTAAGGGCTTGTATGAGACCCCTTCGGGGTCGAGTACGCCGTGTTACGTCTAGGGGGTACCCCCGGGTCGTGACA

>rnd-1_family-131#LTR/Gypsy ( RepeatScout Family Size = 628 Final Multiple Alignment Size (possibly truncated) = 98 Localized to 2361 out of 4218 contigs )

GAAAATTCATGCTTAAGTTGTATTTTGAGTTTTCATGAATTTTGTTTCTCATGGTTGAAATTGCACCCATAGATGTTGCGTGATGTTGTCTTCATGAGAGTTGTAATGAGCATGCTTGATGTTGTCTTGTTAAAGAAATGTCTTGTTGATTGCTTAATATATATGTTGATGCTCATTGAATATGGAGAAGGTAGTTCCTCCTATACTTATGTGAAGTAAAGAGTTTTGAGCATAGTGATGTTGTGGATTAAATTCCTATATGATATGATGATATGATGTTGTTGATGTGACTGGTTGTTGTGCTGCTAGTTTGAGTCNTTGTCTCCCTAGAAAGAAGTCCAAGTGNCATTCGGGGACGAATGTTCCTAAGGGGGGGATATTGTAACACCCCGGAAATTCTATGACTTAAGTTAGAGCCTCACCTTATGAATAATGACTTAAAAATAGTTAAAATGATGTTTCTAAACCTAAATTAATGCAATTGACTTGGTTTGGAAGAGTTTGGAAGTCTAAACGTCAAGGGACGACCANGACGTCCGGAAACTAGNCNTTNACGTGTTCTGGTGTGTTCTAGNGTNTTGTTCANGTTTTGGNGTGTCGTNTGGNGTCTAAAATCTAGTAGGAGGTGACCCTAGTGTCTTAATANATGTTTTTAGGGTCAAAACGTTCGGGTACGACTCCCCGGGGCCACCCTAAGGGGTCCCCGAGGAGGACCCCAACCTTGGCCAAGCAAGCTGCCAAGGCAGCTTGCAAGTTGCNCTTGGAGGGAGCGGNTCCGCGTCGCGGACTNGCTCCACCAAGGAGCAAAAAGTTTGGTCCGCGTCGCGGNCGCGTCGCGGACTCTACTGTCTCGAAATTTAATTCCAAANANCTTGNGGGAACACCTCCGCGTCGCGGACTTGTTCCCCGACGAAATCTGCGAAAATAAAACTCGAGTTTGACTTGTTTAAAAAGTCCAACTAAGTGAGGGGTANTTTGGGTACTTTGGGGGACGTGTATATAATGATTTTTAAGTCAATTTA

>rnd-1_family-160#LTR/Gypsy ( RepeatScout Family Size = 549 Final Multiple Alignment Size (possibly truncated) = 98 Localized to 2361 out of 4218 contigs )

TTTGTGTTCCTAGAGTNGGCGACTTGANNNAGNAGATTCTTGACGGAGGCCCATGGTTCNCGATATTCTATTCATCCGGGCGCCACCAAGATGTACCGCGATTTGAGGCGAGTCTATTGGTGGAGTGGCATGAAGAGGGATATNGCGGANTTTGTGGCTAAGTGCCCGAATTGCCAACAAGTNAAGTACGAGCATCAGAGGCCNGGAGGTNTGCTTCAGAGAATGCCNATTCCNGNNTGGAAGTGGGAAGNGATCGCCATGGACTTCGTCGTNGGTTTNCCCGGNACTTCGGGGAAGTTTGATTCTATTTGGGTNATNGTTGATAGANTGACTAAGTCAGCCCACTTTATTCCGGTAAGAATAGATTATAACGGCGGAAGATTAGCCAAGNTNTACATTAANGAGATAGTGAGGTTGCATGGGGTNCCTNTNTCTATCATCTCAGATCGNGGTNCNCAGTTCACNTCNCANTTTTGGAGGNCATTNCANGANGGNTTGGGTACTCGAGTNAATCTTAGTACGACCTTTCATCCNCAGACGGACGGNCAGNCGGAGCGNACNATTCAAGCGTTAGAGGACATGTTGAGGGCNTGTGTGATCGACTTCGGAGGGCATTGGGATGANCACCTNCCTTTGTGCGAGTTCGCCTACAACAATAGTTACCACTCCAGCATTNANATGGCCCCNTTTGAGGCNCTGTATGGGAGGNGATGTAGATCTCCGGTTGGNTGGTTTGAGGCTGGAGATGTGAAGCCTTTAGGNNCAGATTTAGTTNANGANGCTNTGGAGAAGGTGAGGNTCATTCGAGATAGGCTNNNGACGGCCCAGAGTCGNCAGAAGNNNTATGCAGATCGTAAGGNAAGGGACCTGGAGTTTCAGGTTGGTGATCGGGTTTTTCTGAAGGTGTCACCCATGAAGGGNGTGATGAGATTTGGCAAGAAGGGNAAGCTTAGTCCCAGATACGTNGGNCCTTNCGAGATTCTCGAATGTGTAGGGGGCTTATGAGTTGGNNTTGCCACCNGANTTATCGGCGGTTCATCCGGTNTTTCACGTNTCCATGTTGAAGAAGTATCATGGTGACGGGGATNACGTCATAGTGTGACTCAATTGTGTTAGACGAAGACCTTCGNTATGAGGAGGANCCGGTTGCGATTCTTGATCGNGANGTCCGAAAGTTGAGGACCAAGGAGATTGCTTCCGTGAAGGTTCANTGGAGGCATCGTCCAGTTGAGGANGCTACTTGGGAGACCGAGNAGGACATGCGAGACAAGTATCCCCAGTTGTTCGACGATTCAGGTACTACTCCACTCTTACTTTAGC

>rnd-1_family-495#LTR/Gypsy ( RepeatScout Family Size = 130 Final Multiple Alignment Size (possibly truncated) = 98 Localized to 2361 out of 4218 contigs )

AACTTTTTCTCGAAGTTTTGCAGANATGGCTAGACCGAAAGTTGCAGGAAGAGACATGCCACCCCGACANACAAGAGCGCGAAATTTCAAAAGAGACGAAAAGGNAGCAAATCCTCCCAAAANGGANAATGAGGGCAAGAAGCCCAGTTCCGGCAGGAGGACNANTCCCCGAGACCCCACTATTCCTTCGTGGGCNCGNGGATTCTACGCAGCTATGCACGCCTTTTTGGCGGCTCATGACTTGGACAATCCTAGTGGGTCNGGTACCGCTGNTCCTTCTGAGGTGACTCCGGGCACTGATGCCCAAGTCCAGACTGATGCACCGGGCACTGATGCCCAGACAGATGGAG

>rnd-1_family-210#LTR/Gypsy ( RepeatScout Family Size = 445 Final Multiple Alignment Size (possibly truncated) = 98 Localized to 2361 out of 4218 contigs )

TTAGTTCATTAGGATGTCGTGGGGCTTGTCCCGACATCCATCTCAGTTGTTTTAGAGGCTTCATAGACAGTCAGATGTTAGTTCTTTAGTCTTTTCATTNTCATTATCTTATGTTAAGACTTGAGTTTGCCTTNTTGGCCAAGTTGAATGTTCCTTTATAACATTCTAGTTTATTACAT

>rnd-1_family-93#LTR/Gypsy ( RepeatScout Family Size = 770 Final Multiple Alignment Size (possibly truncated) = 98 Localized to 2361 out of 4218 contigs )

ATGATTTATCTCCTATGCTCATTAACTATGTCTTATGTTGACTTATGTTTATACGATGCTTATGTTGGAGATTATGCTATGACTATGNTGATTATGTTATGACTATGTTATTGTCTCTTATGCTTATGTCTTATGTTGACTAACCATTTTGANATGCTCTTATGATGTTATGCTAGCTATCATACTTAGTACATTTTGTACTAACGCATACTTTGCCTACATTCTAATCAAATGTAGGGTTCGGAGACTTGAGTTCCATTCTCGAGGCTAGGTTTCTAAGGAGCAAGGAGTTGAAGATTTGGTGAGTCCTCATAGCTTCGAGGACAAAACCCACATTTCCTTTATGTCTTTTATTTCATGTTTAGACTATTGTATGGGCTGCGTCCCAAGAGTTTATTCAAAAGTTGTATTAGATGGTTTGAGA

>rnd-1_family-363#LTR/Gypsy ( RepeatScout Family Size = 225 Final Multiple Alignment Size (possibly truncated) = 98 Localized to 2361 out of 4218 contigs )

AAAACTTAGGAACTTGATTATAGATTTGGGTGAGTTTTTGGGTCTAGGCTAGACNTATAGTAATATGGGTGTTGTTAGTTTCGAAATCTTATTGTGATTATTTATTTGAATAGATTGCGTTGATTTGGAAGCTCAACGAAAGGGGAAGGCTCAAGTCCCGGAGTGATTGTTCGATTATTTGAGGCAAGTGGATTTCTAAACCCTTGTTAAGTGTATGGAATTCGTGTATTTCCTTGTAGTATGTGTTTGGGAGTAATGAGACTTGGTGATGGGTTGACTTGTCCACATTGANTAATTCTAATGATGAAAAAGGGGTAATAAAAGGCAACGTGATTAATTGTTGTGTGATGTGTTGAGAATGGTTTGAAAGGCTTGTTGATTCATTGTTGATGTTGTATCACGATTGTGTTGTTGTGAATTGTGCATTGTTATGAAAATGGTCATCTCCTCATTATTTGTGTGAACATGTCATTTGCATTGGTTCTGAGACATGGTTGTGACAAGTGTTATGTGAATTGAGGAAGAATAAGAAATTAAAGAGGATGTACCATTTCGAGGGACGTGTCGCGCGCCGCGACGGATACTATATTTCGAGGGACGTATCGCGCGCCGCGATGGATNCTATTATCGAGGGNCGTGTCGCGCGCCGCGATGGTT

>rnd-1_family-158#LTR/Gypsy ( RepeatScout Family Size = 559 Final Multiple Alignment Size (possibly truncated) = 98 Localized to 2361 out of 4218 contigs )

TGGAAATAGTCATTTTTGGAAAGAATGAAAATCTGGAAAATTTGTTAAGTTAGGAAAAGTTGAGTTTTTGGTCAACTTCAAACGGCCATAACTCCTAGCTCAGGATGAGTTAGGTGTACTTCCAGATATGGTNGGAAANCTCTTGGAATGATCTTTCCAACGCCGCCGAGTTTGCGCGATTCCGAGTTCGTATGAGTGAGTTATGCCCTTTGGAAGTTGGGCTGTTNGANTAAGGAAAGTCCAATCCGGATTTTNGAAGGGTANTTTAGTCTTTTCCTTACCCAATTATTTTAATTCGTTTTTAGGAGTTTAATTGGGGTCAAATCAGANTTTAGTCAGTTTAGAAAANTTGAAATTCACGCTAGGGCTTGGAGAAAAGAGAAAAGAGGAGAAAGGA

>rnd-1_family-533#LTR/Gypsy ( RepeatScout Family Size = 118 Final Multiple Alignment Size (possibly truncated) = 98 Localized to 2361 out of 4218 contigs )

TTAGCTACAGTACCCGGTTNGAGTCCGTGGTCCAGCTTACACGTGGTGCATTCCCGATTCCTCACTAAAGCTTCAGTTTAGTCCTGATTACTCTCATCTGTAGGTTGAGGTTTTGGAGGTTGGAGANATTCCGATTAAAGTCCGGGACGTAATGAGATCTTGGAATATAATTGGGNNAATGGTTCAGTTTAAAAAGTCCGGAAATTGGTAATATTGTGGTAGACCCTCTAGCTCCATTATTTTACTTCAACTGCCGGTGTACCCGTCGGGCTTATGGGGGTCCGTTCGGGTTTTTATTTAAACTTGCGCACGAGTGTACCTTCTGGGCTTATGGGGGCCCGGTGNAGTTAGTTTAGATTACTTNAGTTAGTTCTTTGTACGCTCGTGTGCATTCCTTTTTANATTTCCGTTCTTTGACCTCGNTGTGTCTNGATTCTATCNTTTCNTATTGCCTTTACCTTTCTTGCTCAGTCGGCCTATGATGCCTACTGGGTACCTGTTGTTTTGGTACTCATGCTACGCTCTGCATCTATTT

>rnd-1_family-240#LTR/Gypsy ( RepeatScout Family Size = 384 Final Multiple Alignment Size (possibly truncated) = 98 Localized to 2361 out of 4218 contigs )

CCTCCTCCCAGGCTACTNAACAGACTAAAGGNCGAGGGNTTGNGGACCATCCTNGAGGAGAAGNTGTTGTCCACGGANGGCGTGGNGGGCAGGTACCCCGATGTGNGGGACACCCTCCGGTNCCACGAGTTCGAGCAGTTCACCAGGCCCCGAGGCCCNTACATTCCTNCNTGGGTCCGGGAGTTCTACGCNGCNTACGGNGACTTGGTNCCNAAGGGNAAGAAGAAGGCCAGCGCGTTCAGACCGGTGGAGTCGGTCGTGGTCCGGGGNAAGGAAGTNGAGTGCGACAGCGANCATATCAACGCTGTATTNGGTAGACCGCTGCACTCTGCACTTCCCTACGAGGGNTTGCCTATCGCTCAGTCCCTGGATGACCTGAAGGGTTGGCTGGCTCCNCTGATTTCTGACACCACCCCGAGGTGGATCGAGGCGGGAGCNCCNATCGAGAAGAAGGACCTGAACGTNGCCGCCCGGTACTGGTTCGGNTTCATCAGCAGCACCATCATGCCATCCCAGAACGAGTCCATCCTCCGCCATCCNAAGGCGGCCTGCCTTGGNTCNATCATNGCCAGGAGGCGCATCGACTTGGGACTGATNATTGAGCAGGAGATGGCCATGAGGGCCAAGCAGAGNCAGACNTCCCTNCCATTCCCGGTNTTGATCACNGAGTTGTGCCGGCGTGCCGGAGTNCCTCGNGACGAGACGAGGGATATNGAGGTCACCCCCNCNTCCTCCACCGACATCCGGCGTATNGAGGCCGAGTACACGCGGGANGAGGCCGACAGGAGGAGAGCAGCTCCGGCGGATACNTCCCCGGAGGTCGATGTTGACTCGNTACCTGCAGAGGCACCTTCGCCTACTCCGGCCTCCGGGCCTTCAGGTACATCCGCTCCTTCCTCTTCTTCACAGGCTCCAGGTGCTTCTNCCTCCTCCCAGCCCGCCAGGATTACTCAGGCCATGATCCTGAAGATGGGGCACCTGGCCCATTCAGCCGATGTGAGGGCTACCCGACTGGAGAGGTCCGTCCCGTGGATGATCGAGAGTGCCATCCTAGCTGCACTGACCCCCCTCCGGACTTCTATTGACGCTCTGACNGCGAGAGTCGCGGCTTGTGAGAGCAGACAGGGGGAGACCTCCGAGGTGACGGCTTTGAAAGCCGAGGTNGCGGATTTGAGGAAGGACGTAGACTATCTGAAGTCTACCGACTTCACTTCACTGATGCGGGGTGCAGATGACGNGGATGCTCCCGAGACCTCGGAGATTCCTCCGGCTACCACCGGAGACGTGCAGAGGGATGACGCGGCAGNTGANGAGTCGGATGCGGAGACCGACGAGGAGCAGATAGNGGTACATGAGGAGGAGATATACGGAGACCTGCCAGATCTGGAGATATTCAGAGACTTGCCAG

>rnd-1_family-53#LTR/Gypsy ( RepeatScout Family Size = 1045 Final Multiple Alignment Size (possibly truncated) = 98 Localized to 2361 out of 4218 contigs )

TTGTGGTTTGTTGAATGGATCGGTTGCCACGTTCCGGCATAAACTTGGATCGGTTGCCACGTTCCGGCATAAATATGGGATCGGTTGCCACGTTCCGGCATAAATATGGGATCGGTTGCCACGTTCCGGCATAAACATTGGATCGGGTACCACGTTCCGGTACGCTAACAGTTTGGGTTCGGGTTCCATGAGAGGACCATTGAATTGGGTTCCGTGAGAGGACCATTGAATTGTGTTGTGTATCTACTTGTGATGATTACGTTCATATCTAATGATGATTGATATTGGAAGACTGTATGTTGCTCTAAATTGATAACCAATGTGTATTATTGAGAACGTGGAATTGTACGTTGNTCCTGAAATGATGACTGATATTGTATATGTTCGGTATATGCATGTTTTATANTGTTGTGGTTACTTGCGTATGTTNCACTTANTGGGATAGCCGCGTGATCCTACCAGTACACTGTGGTTGTGTACTGATACTGCACTTGCTCTTTCTTTGTTGAGTACAGGGCATCTTCAGGCGGCTATTGATAGACCTCGGCTAGGNGACTANTGANCGTGACCGGATTCAAGGGTGAGCCAGTTCTTTCAGGCTGCCATGGATCTCTCTTGTTTAAGTCCACTCTTTTCGGACTCAGACTATTTGTTTAAGGTGTTTGTTTAGTTTCGGGGTTGTACCCCTTGTTCTTAGACTTGTCGTTAGTAGAGTTTTGGTACANTGACTTTCAGGTTCTAGGGGTTGTTCTTCCGCATTAGTTTGGTTAGTTGTTTGTTTAAACCTTTGGAGTTTATGGGAACTCCATTTTTATTGTCATTTAAACCTGCTTCCGTATCTTTAAATGCCTTAGTTTTTCGTTAAGTTGCTTAGTTTGGGTTGTAGTAATGGTTCTCCCACCGGAGGGTTAGTGTGGGTGCCAATCACGACGGTC

>rnd-1_family-189#LTR/Gypsy ( RepeatScout Family Size = 488 Final Multiple Alignment Size (possibly truncated) = 98 Localized to 2361 out of 4218 contigs )

ATTTGACCCCAAAAGTGCCCGAAAATGTGCTTCGGAAGTCACCTGNAACTTGTAATTTAGCCATATATCNCAAAATCCGTTTCGTTTTTCGGAAATCCGACTTCATATTCTTGTTCAGGGGGTCAAATTGAGTGGGAAATGGGTCTAACCCGAATTTTAGAGCAACCGTATCAAAATCCGAAATTTCCAAGCGAAGCCTTTTTCGAGGGTCTACTTTGGAGGGTCATATCTCCTAGCACACAAATTATTGGGTGGCCAATAACATATCCATGGAAAGCCCTTTGAGTTAGCTACCTAACGCACTTCGTTTCACCTCATTCGGAGTTCGGACGAAGAAGTTATGCCCATTTTCGTAAAACCTGTCCGGCAGGAAAGGCAATTTCCAGTGAGCGTNTTTACTGTTCACCCGCCTCATTTTTTTTTCTAAGTGTTGGACCATTTTTCCAAAGGGCATATGTTATTTCCTATATACCATTAGTTCAATTCCCATCTCTAAAACATTTCCCAAAACACCTCTCCCATACTTAGACACATTTTCTCTCAAGTTCTCTCAAGAACCCTAATCCAAAACCTTCCTCAAGATTGAAGAGACTCTTGCTCCAAGTTCCAAGCTTCCATTGAAGACACTCTCAAGACCTTCATAAAAACTCAAGGTATGTGGTGTTGAACTCATGGGTCCTTCCACCCATAGTGCCTAGACTCTATTCTACTCACTAATTCATGGTTTAAGTTAAGATTCATAAATTAGTCTTGTTCTTTGTAATAATTTCCTGCACATTGAATTTTAAACATGAAAAGTGATTGTTTTGAGCATGTTGTGACCCTAGAACTTGAACCTAAATTTGGAATGGATGTGGAGATTTTCATGTGGTATTGTGGGTGTTAAAAAGGTGTTGTTGTAGGTTGTTCACTGGTTTGGCTGCATAATTACTACCCTATTTTCCATGCTCTTTGATTCCATTACATGCCTTCAAGGTGTTTGACAAAATGTCCAACTATGGAGAATTGATGAATCGATGCTTTAAAGCTTGAGTTATGAGATGTATGGAAATCCAGAGATGTGTTGATGACAAACTAAGCTTGTGTATATGTTCATTCCATACGTGAGATTGCATTAGAGCCTAATGGGAATTTCCTATATAAAGTGTTGCATGACCATGCCCGGTCCGGGGGAAAAGAACCGGACAACCATGTGAGAGGTTTATATCTCACCACCTAGGATGCTTGGGGTGTGACCAACATCAACCATGTGAGAGGTTTATATCTCACCACCTAGGATGCTTGGGGTGTGACCAACATCAACCATGTGAGAGGTTTATATCTCACCACCTAGGATGCTTGGGGTGTGACCAACATCAACCATGTGAGAGGTTTATATCTCACCACCTAGGATGCTTGGGGTGTGACCAACATCAACCATGTGAGAGGTTTATATCTCACCACCTAGGATGCTTGGGGTGTGACCAACATCAACCATGTGAGAGGTTTATATCTCACCACCTAGGATGCTTGGGGTGTGACCAACATCAACCATGTGAGAGGTTTATATCTCACCACCTAGGATGCTTGGGGTGTGACCAACATCAACCATGTGAGAGGTTTATATCTCACCACCTAGGATGCTTGGGGTGTGACCAACATCAACCATGTGAGAGGTTTATATCTCACCACCTAGGATGCTTGGGGTGTGACCAACATCAACCATGTGAGAGGTTTATATCTCACCACCTAGGATGCTTGGGGTGTGACCAACATCAACCATGTGAGAGGTTTATATCTCACCACCTAGGATGCTTGGGGTGTGACCAACATCAACCATGTGAGAGGTTTATATCTCGCCACCTAGGATGCTTGGGGTGTGACCAACATCAACCATGTGAGGGGTTTATACCTCGCCACCA

>rnd-1_family-136#Unknown ( RepeatScout Family Size = 617 Final Multiple Alignment Size (possibly truncated) = 98 Localized to 2361 out of 4218 contigs )

TGGTTTAATTTAATGGGTTTGTAGTTGCAAATACAAAACCACCCATGTGTTTTCGGCTTGCCCGAGAGGGAGGTCGCGAAACCAAGACCGCTAGACTGATGGCCTAAGGAGTGGGTCGACATGAGGTTCAGCCCGAGAGGGTGAGCCCTAGTCCCATATCCTAACACTCAGCTCGAGAGAGTGAGTGGGGTAAGGCGTAGGCTGGTCTTCATGCGGCAAATGGGTGTCCGAGAGGAACGCATTTGAAACGGGGTAAGTTGCCCGAGAGGGAACTTATTTCCATCTAAAGCTTAGCCTAGTCACTATTATCTTGCAAATTTCCTATCGAAAGCATGTACCCAACGATTTATCTCAACTTGTATTGCGGTCACACCCCAAGAACTTTTCCCCATACTTGATTTTCTTGTTATTTTTGCTGTTTTTACTACTTGTGACAAAACCCCCAATTGATATTTGACACTTTCGTGTCACCCCCTTTAATTTACNATGTTTTTANTCGTTAATGTCTTTAGCTACGACTAGTTAGAACTAAATTTTATTTTTCTATTAATTCTCAAAACCACTCCCTTGGGACACGACCCCAACCCTTGGTTGGGTTACTATATTATCGACGATCGTAGACACTCGTACCGTAGGTTAGTGTCGTTGGTCACGATAAGCATCA

>rnd-1_family-484#Unknown ( RepeatScout Family Size = 135 Final Multiple Alignment Size (possibly truncated) = 98 Localized to 2361 out of 4218 contigs )

GTTAATATCTCAGATGGTCACTCAACTATGAACTNTTTTCTCAGAAAGTCACTCAACTTTGATTTTTAACTCAAAAGTCACTCAACTATGAATTNTTTTCTCAGAAAGTCACTCAACTATNAATNTTTTACTTAAAAAGTCACTCAACCTATTTAATTTTAATTCAAA

>rnd-1_family-456#LINE/L1 ( RepeatScout Family Size = 146 Final Multiple Alignment Size (possibly truncated) = 98 Localized to 2361 out of 4218 contigs )

CAGCTTACAGGNTGATGGACCAACCAAGTCAACAGATTCCTAACTGGCCNTGGAAGCNNATNTGGAAAGTCAAGATNCCNCACAAGGTGGCATGTTTCGCNTGGCTGNTGGCTAAGGAAGCAGCTCTGACNCAGGANAATCTNACGAAAAGGGGGATAACCTTGTGCTCTAGGTGCTTTCTNTGCGGAGAAACAGCGGAGACAATCAACCATCTNTTTCTNCACTGCAAANTNACTGNNCAACTGTGGAANATNTTCCTCAGTCTCAAAGGCATATCCTGGACNATGCCTAGGAGNATTACTGAGGCCCTAANNNGCTGGGAGGAGGCGGGAGTGCGAGCAAAAGACAGAAGAGATGGAGGATCGTCCCAGCTTGCATNTGGTGGACAATNTGGAAGGAGAGGAATNNNAGATGTTTTGAGAGCATAGAGAATAGTATGCAGAAAGTTAAANTGAACTGCATTTTGTTNTTATGTTTTTGGTGTAANCAANTATACTCTAATGANACTGTCTCTATNATTGATGTTTTAGACTCAATTTAGANNTAGAATAGTGGAGTTTTTGGAGTTCATCTGTAAATATGGTTTCAGTACAACCCATGTACTGTTGTGTTATAATACAAAGTTACCANTTTCAAAAAAAA

>rnd-1_family-451#Retroposon ( RepeatScout Family Size = 151 Final Multiple Alignment Size (possibly truncated) = 98 Localized to 2361 out of 4218 contigs )

AATTTTGGAAAAAGATATTCAGAAGCATATATCTACATCACAAGAGAAATTGTTAACAAAAACAAATGAATTTAGACTACATCATGTTAACTTCATATATTGGAACATTACATACTAATGTATATATTAAGCAAAAAAATAATTATTATATCATCAAAATTATTCCCCTCNAGTACCCGAGGTTACGGAATATATCCTCCCAGGATAGAACGATCTTACTCACCAGTGTATCGGTACCTCAAACTCCGGTGNCAGCGAACCACTCAACGACAGTAAATCACACGAAGAATNTTAATTGTGCAGAAGAAGAAGAGTAGAA

>rnd-1_family-91#LTR/Gypsy ( RepeatScout Family Size = 782 Final Multiple Alignment Size (possibly truncated) = 98 Localized to 2361 out of 4218 contigs )

TGAATTTTATTCAGTTTTTAAGCTTNTGTATGCTTGAGTTAGTCTTCCGCTTGTAGTCAGCCAGGACGAGGGTTCGCTTGGGGACCAGCAATGGTTCTCGAGTGCCGGCCACGTCCAGGGTGTAGGCTCGGGTCGTGACAAACTTGGTATCAGAGCACAGAGTTCAAGTGTCCTAGGGTGTCTATGAAGCCGTGTCAAGTAGGATCTTATTTATGGTTGTGAGGGCCCCACTTCTATAAATGAGGGACTACAGACATTTAAGAAAAGTTTCCCTTCTTTCATACTCTNAATCGTGCAATAGAGCTATGTCATAGAGACTTATTCANACTCGTGCGTTTCTCAGATTCATTCGACTACCCCTCATACTAGAGGTGGTTGAAAAGCAAAGTCAGTTCTTTATCGATGAGTTGCTCTTAGCAAAAGTCTTGAGGAAGTCATGAGACTCCAGAGGGGCTTGAGAGAAGCCCGTGAGTGAGTTAAGTGTTGAGTTTCAGAGGAATGCACTCATATTCATATGAATCGTGCGTTCGAGCTAAAAGCGAGNTGTACTCTCTTCCCTAAGCCTTGTTTCAGCTGAGATCCTTATGAGGAAGTATGTTTAGAGCTTGGGTAGTATTTTCACCCGAAAAGGTAGCATGAGTTACGAGATCATGAGCAGTAGTAGTGAAAGTGCCCAGAGTTAGAGGAAAGTATGCAGAGGTTTAGTAATCTAAGAAAGGGAGATAGTGNTNNGGCGAGCTATGTTATTATAGTCATGCACCCAGTAAGTTATTGATGAGGAGTTTTCCCTTATGGGTAGACTAAGAAGTGATGAGTTGTGAAGATTCCATGATAGGAGGTAGAGTGTGTTTTAGCTAGAAAGGAGTTGGTGNCGAAGTGTCATCGTGTAGGNAATGACCAAGTGTANGTGTTGAATAGAAGAGCAGAGTATTCATGAAGTGATAGATCTAAGCTAGGCTTATGATTTGTTTGAGAGGGTCATGATGTTCAGAAGGTTATAGAACTGATCAAGTAATGAGGTATAGTATAGTAGGCGAGCAATCGTATTGATATTCGGGTTTAGTAATAAACATTAAGCTTGAATTTGAGATGAGTGTCATGATCAAAAGAATGGCAACATGAGGGTGATGATGTTAGTCTTGAGATTTGATCTAGNAGACTTGACATAGAGTAGGTGAGGAATTGAGATGTTAGCTGCAATAAATATGAGTGGTACAAGTGAGAGACCCTAACCTTTAGATAAGGGCAGTGAAGTGTGGCTAAGTCACAAGAGTAACAAGAGCATTAAGAGGTGTACCGAGAGGGTATGTAATCAGANGAGGTTA

>rnd-1_family-90#LTR/Gypsy ( RepeatScout Family Size = 783 Final Multiple Alignment Size (possibly truncated) = 98 Localized to 2361 out of 4218 contigs )

TTTTCCTTAGCTTTGAAGAATTGAAGNTTTTCACTTTTGAGAAATTGGAAGTGGGTCTTTGAGATTCTTCGANTTGGANCNTTGTAAAGACGAAATCAATCTTACCCAGTTGATGGAAACNCAATTTGGTAACGATTTTTATCTTTTTATGATGTCTAGCTAAAACCCCAATTCTTGGGGTGTGATTATGTGATTATGGGCTGATTTAGCTTATGGGTATTGCTAATTGTTAGTTTAAATGCTGTTTAGAAGTGANTTCAATCANTAATTGTGGTTTAATTTAAGAATTGTAGTTGCAAATGCAGTTCTACCTTCGTGTTTTTGGCTTGCTCGAGAGAGAGGTTTTAAAACCAAGATTATTGATTGATGGTCTGTGGGTATTGGGTTGTCATGGGTTCAGCTCGAGAGAGTGAATCCTAAACCCNNTCCCACACATTCAGCTCGAGAGAGTGAATGGACTAAGGCGTGGGTTGTTCTTATTTTGCATGCTTGTTGATGTTCGAGAGAAATCGACTTGATTCGGGGTAAATTGTTCGAGAGAAAGTTTACCTCCNCTATAGTCTAGCTTACTCACTAATNTNCAGCTATTTACTAATAGTTGCAATTACCCATTTGTCTACATTAGCCTATAATCGAATCACATCCCAAGAACCCGTCTCATTATTGTTATTTCGTGTTGTTTGTCGCTGTTTGTAGTTGATAATTAAAACCAAAACCCCCTTATTTGACATTCGTGTCACCCCTTAATTTGAATTATGTCTTTATTCGATAATNTCTATTCCTATGACTGATTTGANCATATTCGTACTTCCCTATTGAATCTTAAACACGTACCGCTCCCTGTGGGATTCGACCCCAACTCATTTAGTTGGGTTATATACTGATTAACGATCGTTGACGCTTAGAATTGGATGAAGTGTCCTTGATAACGTTAT

>rnd-1_family-411#LTR/Gypsy ( RepeatScout Family Size = 177 Final Multiple Alignment Size (possibly truncated) = 98 Localized to 2361 out of 4218 contigs )

GAGCCCCGAATTGTGTTCCATTTTGGAACACGAGTTCGGGGAGCTGTTTAGGACCTTTTGACGGAGTCAATTTCGGAATTTCCGGCGCGGGTCCCACGATTCCCGTTTTGACTCCAAAATTGGTCCGTCTCCGTTTNTTGTGATTTTGGTGTCTAAACGACCGTANTAACGTTGTGATTCTATTTTTGATAGCGTGGCAGCGTTCGGAGGCCGTTCGGAAAGGAAAAGCTCCGGAGNAGTGATTTTGGAGCGCGCGCGATCGGCCTACAGGTAGGCTACGGCTTCCCTCTCTTAGATTGAGCTCGAGCGTGTGAATGCATGTTGATTAGTTNGGATTTGGGGTGGGTAGTTATTGAATCATGCATAGGTGTTNAGAAATCATGTTTTAGGCCTATTTCGGGAATTATCGGGTAACTGTGAGCATGCTTTGTGTTATTAACGGACCCTCCTCGCTATGTGGAGTGCTTGCATGCTTAATTATTGTTATTTGAAGCATGTTNGGCCTTAGTCTAGGCTTAGACTAGTGCTTGCCTTAGACNTACGTGATTCGGATCCGATAGGCCTTAGTTTTGCCCCGACGTCGCTCGGNCGGCTTAGNTCCTTGTAGACTGGTGTAGCAGACTTGAGTCTGATAGTTTGGGCCTTAGCTAGGCGATACTCTTNCTCCGACTATAGTTATCCTTATTTTCCCCGATGTTACGGCTTCACGAGTTACGTCGGCGACACTAGTTCTACTTCGCGATTTGAATTTGATTTTCGATTCGGTTCCAAGGACTTACATTGATTGGCTAAGTGTGGACGGCGTTCCACGGACATTTATGAGTATGGATCGATTGGGACTCTTTCAGCAGCTACATTGGCACTTATATAGAGCATCCGGTTTAAGGTCCGGCCTCTATCGCCCAGATACTTATATAGAGCATCCGGTTTAAGGTCCGGCCTCTATCGCCCAGATACTTATATAGAGCATCCGGTTTAAGGTCCGGCCTCT

>rnd-1_family-340#LTR/Gypsy ( RepeatScout Family Size = 245 Final Multiple Alignment Size (possibly truncated) = 98 Localized to 2361 out of 4218 contigs )

AAATCAAAACCCTTCCCTCCCAAAAAGTTCTCTCTAGAAACTCCATTGGAGANCAAGGTCAAGTTCGAGCTAGGGTTGGGGATTTCAAGGTGTTTCTTCTTCAATTTTCGTGGGGATTCAATAATTAAGGTATGGTAGTCTTCATCCTTGACTAANTTTCCATTCAAGGAGCCAATTCAAA

>rnd-1_family-447#Unknown ( RepeatScout Family Size = 153 Final Multiple Alignment Size (possibly truncated) = 98 Localized to 2361 out of 4218 contigs )

CTTGAGCTTGAGAGAGAATTGGAGAGGATGAATTTGGATGGAATTTGGGAATTTGAGNGAATGAGAGAGTTGGGGTAGTTTTAGGGGTAAAANGGTAGTTATAGGGCTTAGAANTAGGGTCAAAACGACGTAGTATAGGNATTAAACGNATAGGAAAAGACCCAAATACCCTTAAAAATAACTGT

>rnd-1_family-431#Unknown ( RepeatScout Family Size = 165 Final Multiple Alignment Size (possibly truncated) = 98 Localized to 2361 out of 4218 contigs )

ATAATAGGGAAAAGGCTCAAATATGTCATCGAACTTTGAGAAAAGGCTCATTTATGTCATCCGTTAAAAGTTTGGCTCATCTATGCCATTTCCGTTTGAGAAAAGGCTCATCCATGCCATTATTTNTTAAC

>rnd-1_family-544#LTR/Gypsy ( RepeatScout Family Size = 112 Final Multiple Alignment Size (possibly truncated) = 98 Localized to 2361 out of 4218 contigs )

ATATCGATTTTGGTCTAGAACGACCCTTCGTTCGCTTCCCGAGGCTTTCGAGGCCCNTTGTGGATTTTGGCTTAAAATGANNTTGGGTGTGGGACCCACTTTTTATCGAGACGACCTCCGATGGAAATTTCGACTGCGCCGTTGAGTCCGGAATATCGAATTCGGTANGGTTGCATATCTCGTTTGCGTGCACGGGGTTCCGAACGAGTTCGGAGCACCCCGTCGGAGTTTTAAGTTTGAAGAGTTGTTGAATNCTAACGCGGTGGTGACGTGGCAGAGCCACGCGACGCCAGGTGGCAGCGTTGTTGAAAATTTCCAGATTTAAGGACGATTTTCGTCCATTTTTTTCCCTCATCTTCAAACATAGATATCTCNTCCATTTCAAGCCCGATTTNGGTGATTCAAAAGGCTAACTTGTGAGATTTTTCGAGGAGAACGCGTTGGTGAGCTCGGAAACGCGAGGGGAGCCTTCGTTTGAGGTAAGNAATCGAATTTACCTGCTGCCACGGCCCTTTTTCGAGCTCGATTTTGGGAATTTTGAGACTTGATNTCTCGGCCATTTTAGGTCCGATTTCGGTGATTCTTGAGGCTANCTTGTAGTGGTTTTTCGCGGGGANCGTCGTGGTGTAGTGTTTTCGGGTTGGAAGGGTCTCGTTTCTCCAGAAATTGGTGATCAAGGGGCTGC

>rnd-1_family-198#Unknown ( RepeatScout Family Size = 479 Final Multiple Alignment Size (possibly truncated) = 98 Localized to 2361 out of 4218 contigs )

TGTCTACGGGTCCCATCTACGGACCGTAGAAGCTTCTACGGGCCGTAGATGGCAACCGTAGGAATGGGGAAAATGATTTTCTAAAATCAAGTCCCAGTACCCTTTTTACGGTTCACCAGGACGGTCCGTCGTTCNATGTACGAGTCGTAGATAGGTCTCGTAGGTGAGTCCCAGACTTAGTGAAATTTTAAGGGTTAAAGTTGGGTTCTACGGNTGGGGTCTACGGACCGTAGGAAGACCTACGGNCCGTAGANGGGTTCCGTCGAAAGTTGTCCAAAATTATGGTCTCCGAACTTTCCTACGGTTGACCAGTACGGTCCGTAGAAAGTCCTACGGACCGTAGGTCCGAACCGTAGACGGNGTTCGACAGTTATTTTNAAGGGCTTTTGGGTCTTTTCC

>rnd-1_family-107#LTR/Gypsy ( RepeatScout Family Size = 706 Final Multiple Alignment Size (possibly truncated) = 98 Localized to 2361 out of 4218 contigs )

ACAAGAACATGAATTATGAACTATGGTTNCTCAAGAAGCTCTACTTAGTATGGGTGGGGGTATGGGACCTCATTCATGCATTGCACAAGTAGGCTTTGAGGGTANTTATGGTATGTTTCTCTTATGNTATGATGATCTATG

>rnd-1_family-416#LTR/Gypsy ( RepeatScout Family Size = 173 Final Multiple Alignment Size (possibly truncated) = 98 Localized to 2361 out of 4218 contigs )

TGTAACACCCCGTATTNTGCGTGTGCTAGTTCTATTCATATGACGCTTGACATAGCCTTGTGGTATAGGTGANGGACCGTGTTTCCTATGCGACTAGTGTCGGTTTAAACTAGCTCGGAAGGTGATGTGATGCCTTGCACGTTTCAGCATGTGTACCGCAAGTTAACTCCGACGGAAGTATTTAAACTCGAGATNGTAAGGTGGTANTTGAGCTTAAAGTTGAAAGTTAAGTGCTAAGTTAAAGNAAAAGGGATTAANATTTAGAAAATGGAATAAAATAAGTGAGNTGCTTGCTTGACTTAACTAAGCTAGTAGGTGACAAGTAGGTGCATGCACCTACTTNGACTTATTGACCGGTCCAAAGGGCCAAGTAGGTGCATGCACCTACTT

>rnd-1_family-207#LTR/Gypsy ( RepeatScout Family Size = 455 Final Multiple Alignment Size (possibly truncated) = 98 Localized to 2361 out of 4218 contigs )

TTTGACTTATGTTTCTACACTAATGTGCATGATGGCTCTTAAACTTGATAAATGCATGNTTTCAACTAAAATGTCCCTTTTAGCATGTTTTAAGGATTTTATGCATGGCTTCCATACTTAGTACATTTTTGTGCTAACCCATATTTTCTACATTTTTCTATAAGTGTAGGTTCCGGTCGTTGAGGCGTACTTCTTCCTTCGANCGAAGCTTGGATCGACTATTCTCCANGCTTGTGGTGAGTCCTCATGGTTCGAGGACGGAAGTCGTTCGTTTCTAGTTTCTTNTACTTTTCCNTTTGAGACTATGTTGTAAAGGGCTGTGACCCTATTTGTACTCNATTGTANTAGATGGTTATTGAGACAAGTCTAGACTTCCGTTTNCTTTTATGAAAAGACTTCGATTGTAAAAGTTTTAA

>rnd-1_family-437#DNA/Harbinger ( RepeatScout Family Size = 161 Final Multiple Alignment Size (possibly truncated) = 98 Localized to 2361 out of 4218 contigs )

TAAGTAGGCGTTTGGCCATGCGATACCATATCACGATATGGNATCGTGAGATGGAATCAGCGTTTGGACATGCGATTTCACGCTGATTCCATCTCACGATTCCATATCATGAGAGTGATNCCATATTCTCCAAAAACCATGATATGGGATTAATGTGATTCCATATCATGATTTGAGATATTTTAATACAAAAATTGATCCACGAGTTTATATTTTGTTAAAACAACCCCACATTTATATCTACTAACCATTTATTTCATATGTAAATAAAATTTATAATCACATCATTACTTTTTAAAATTTATTATTCTCACCGACATAAAATTTATTATTACTTTAAATTTGGTGAATATAAATGATAAAGTAGAAATTGATTTGGTGAATATAAATAAGAACGGAGGGAGTAATACTTTTTTGATTTTTCATAATAAAAA

>rnd-1_family-500#LTR/Gypsy ( RepeatScout Family Size = 129 Final Multiple Alignment Size (possibly truncated) = 98 Localized to 2361 out of 4218 contigs )

GGTATAGTATAGTAGGCGAGCAATCATATTGATATTCGGGTTTAGTAATAAACATTAAGCTTGAATTTGAGATGAGTGTCGTGATCAAAAGAATGGCAACATGAGGGTGATGATGTTAGTCTTGAGATTTGATCTAGTAGACTTGACATAGAGTAGGTGAGGAATTGAGGTGTTAGCTGCAATAAATATGAGTGGTACAAGTGAGAGACCCTAACCTTTAGATAAGGGCAGTGAAGTGTGGCTAAGTCACAAGAGTAACAAGAGCATTAAGAGGTGTACCGAGANGGTATGTAATCAGANGAGGTTAAGAGAAACGCCTTAGAAAGAGGTCTTAGTACCTGCTTATGGAGAGTTCGGTAGCCATGAGATAAGCTTCTCCCGAGTGTTAGAGTAAAAGAGGATGTAGACCAGTTTAGAGATGATGGAATAGGTCCATAGCTTTCAAGTGTCAGCTTTAGACCTAAGGGGGAGATGATAAGATGAGAAATCAAGTCAAGTGTTGAGAATAAGATAGTAATGAGTTCCGAAGAGTATGGGAGATATCTTTTCGGAGGTCCTTTTAGTAATGGAGTGATGTTAGAAAGAGGAGGTTGATGTTCTTATCGTGATAGTGATGGTGTGATAATTCCGTCTAAGGTTATAAATGAGGAGGAAGAGGAGGATGAGGTTTTACTATGAGAAATAG

>rnd-1_family-432#Unknown ( RepeatScout Family Size = 165 Final Multiple Alignment Size (possibly truncated) = 98 Localized to 2361 out of 4218 contigs )

CCTCCTACATAATGAGATTTAGATTAGCAGGACCCTCCTACATAATGGGATT

>rnd-1_family-309#LTR/Copia ( RepeatScout Family Size = 270 Final Multiple Alignment Size (possibly truncated) = 98 Localized to 2361 out of 4218 contigs )

CCCAATTCGTTGTTCACCGATGTTGGGCCCCCATTTAGAAGTGTCCACGCTCCAGTTGAGGCCTGGGCGTGAGGNGGAGTGTTAAGAAGTCCCACATCGGTNGAGGGATGGGATGGTCTCCTTATATGGACTTGGGCAATCCTCCCCTCATGAGCTAGCTTTTGGGGTTGAGTTAGGCCCAAGTGTCATATCTTTAC

>rnd-1_family-523#Unknown ( RepeatScout Family Size = 121 Final Multiple Alignment Size (possibly truncated) = 98 Localized to 2361 out of 4218 contigs )

AAAATGGTTTTGCAAAACCGCAGTTAACAAATAATGGCATGGATGAGCCTTTTCTCAAACGGAAATGGCATAGATGAGCCAAACTTTTAACGGATGACATAAATGAGCCTTTTCTCAAAGTTCGATGACATATTTGA

>rnd-1_family-352#LTR/Gypsy ( RepeatScout Family Size = 229 Final Multiple Alignment Size (possibly truncated) = 98 Localized to 2361 out of 4218 contigs )

ATTATGCCCGTTGAGGATATTCGTATCGCTGAGGACTTATCTTATGAGGAGGTNCCGGTNGCNATTTTAGATCGGCAAGTTAGNAAGTTGAGGACTAAAGAAGTAGCTTCNGTNAAAGTGTTNTGGAGGAACAACAACGTCGAGGAAGTNACTTGGGAAGCCGAGGAGGANATGAGGAAGAAGTACCCNCANCTNTTTACGACTTAAGGTGAGTCGCGTTTCGATNATTGGCAATTATTTCTTCACAACGACTTAGTTAGATTTTAAGTAGCTCGAAGGTGNAATTTATATGTCTTCTTACGAGATAGCTATGCGAAGCTTNGTAGTTAAAATTTTCAGAATTTGACTTNAGGTTTGCGACCGTAACAAATATAGACTCGCAAATAAAGCACTGTGAGAAAAAAGAAAATACAACCAAGGAATTGTTCGGATCCATTCGCGGGCGAATGTTCATAAGGGGGGGAGAATGTGAGACCCCGTGGTTCTTTCGCATAATATACGTGGCGTAGTATGGCATGGTTATATGAGGTGTATATGACTTGGTGTATCGTACGTTGGAGANTAAGATTGCAAGTGGGTGGCAATATCAAGGAACTAAACTAAAGTTTTAGGTCAAGGGACGCCCTCAAACAAAGTTCGNGTTAAAAAATGGCTCTTGTAACACCCCGACTTTTCGAAACGTCTAAATTAACTCGTACCTTCGTGGAAAGACAAAGAGTGTGTAATAAATTAAGAATGATGTGTTATGTGATATTTTAAGTGTTCAAGGGTCGTATCTCAAGTTTTGAAGTTAAGTAAGTGGCGAAATAAAA

>rnd-1_family-367#Unknown ( RepeatScout Family Size = 219 Final Multiple Alignment Size (possibly truncated) = 98 Localized to 2361 out of 4218 contigs )

TTGTGAAAGGTTTTCTCACATAAAGGATTCTAAGGTTGAAAGGTTTTCTCACCTAAGTTGAATCAAGATTTAGGAGCTATCCTAATGAAACCTAGCTTGGATTGGTTACTTGAT

>rnd-1_family-55#LTR/Gypsy ( RepeatScout Family Size = 1026 Final Multiple Alignment Size (possibly truncated) = 98 Localized to 2361 out of 4218 contigs )

ATTTTTACATTNAAAAAAGAGAAACTTCGATTTCTAAATGAGTTCATGAGTTCAGAGATATTTCAGAGCAGTTACCTCTTTTAAGAGGATATTTTGAGCAATTATCTCAAANCGAGGAAGAGTATGTTTTTAAACATATGAGCATGAGTATATATTATTGGGAGTAGTATTGAGCACCGATATGGGGACGAGTTCAGACAACTCANAGTCCTCATAAACCATGTATGCCAACATGGGTAAAGGGTCATACTTTTTAGATGATTCCTTATTGCTTTTAAGCATAGCTTAGTGGATCCACTTAGTTGAGGTGTTCTATACCCCGGCAAGGTATAGGACAGTTCTGGCAGCGTGGGCGAGACGTTGTATCATCACGTAGCTCATAGTGATGGTTGTCGGTTAGAGAATCTCCCAAATAGAGTTAAAAGTGTATTTTTATTATATCACTTATTATGTTGAGTTCAGAGATGAGTAAGTATTTTGTAAAGCTTTAAATGATTTCACTCCTTTATTTCTTTACATTCAGTTGAGTNTATTNTTATCTATTGCAGTCCTTTCGTTCAGTTATTTGTTATTCAGCTATATTACATACTCGTACATTCAATGTACTGATGTCATTCGACCTGCATCTTTTNATGATGCAGATACAGGTGTTCAGGATCATCAACAAGCGTCTGTTGAGATCACTTCGTCTGCTCTCCAGCTTTTGAGTGAGACCTCCTTGCTTTCGGAGGACTCCANTTTTATGAGTTGTTAGTATTAGTCGTTTTGAGGNGTCGTGGGTCTTGTCCCGACACCCATCTTTATATAGTAGAGGCTTCATAGATAGACAGTTTTGAAGAGTCTTTCA

>rnd-1_family-50#LTR/Gypsy ( RepeatScout Family Size = 1073 Final Multiple Alignment Size (possibly truncated) = 98 Localized to 2361 out of 4218 contigs )

TGAGTTGAGTAAGTTTGAGTAGTTTTGAGTATCCTTGAGTATTCCTTGAGTTGAGTGAGTTGAGAAGAGGTAAGTATGTTTCCTTTTTATCAAGTTCAAGCTTATGTTTATGCTTTAGAATTCCCCTTACATGCTCGTACATTCCACGTACTGAGCCATTTGGCCTGCATCNTTTCATGATGCAGACACAGGTATTCAGGATCATCAACAGGAGCTTCGTTGATACCACATGGAGTTCGAGTTAGCTATGGTGAGCCTCCTTGCTTCCGGAGGATTTCATTTACTTTTCAGTTATATCAGTTGTTAGGATGTCGTGGGTCTTGTCCCGACTTCCATCTTTGTCAGTTAGAGGCTTCATAGATAGACAGTANAGTTTGAGAAGTCTGTATTATTCATTTATTTTATTAAATGTTTTAAAGACTTAAGTTGCCTATTTTATGGCGAGTTGAATATATTATTTTTATTCNGAGTTNTTCATTTGAGTTAAAGTTTTGTAAAGTTGAGTTTANTGAATTTTATTCAGTTTTAAGCTTTTGTATGCTTGAGTTAGTCTTCCGCTTGTAGTCAGCCAGGATGAGGGTTCGCTTGGGGACCAGCAATGGTTCTCGAGTGCCGGCCACGTCCAGGGTGTAGGCTCGGGTCGTGACAAACTTGGTATCAGAGCACAGAGTTCAAGTGTCCTAGGGTGTCTATGAAGCCGTGTCAAGTAGGATCTTGTTTATGGTTGTGAGGGCCCCACTTCTATAAATGAGGGACTACAGACATTTAAGAAAAGTTTCCCTTCTTTCATACTCTTAATCGTGCAATAGAGCTATGTCATAGAGACTTATTCANACTCGTGCGTTTCTCAGATTCATTCGACTACCCCTCATACTAGAGGTGGTTGAAAAGNAAAGTCAGTTCTTTATCGATGAGTTGCTCTTAGCAAAAGTCTTGAGGAAGTCATGAGACTCCAGAGGGGCTTGAGAGAAGCCCGTGAGTGAGTTAAGTGTTGAGTTTCAGAGGAATGCACTCATATTCATATGAATCGTGCGTTCGAGCTAAAAGCGAGTTGTACTCTCTTCCCTAAGCCTTGTTTCAGCTGAGATCCTTATGAGGAAGTATGTTTAGAGCTTGGGTAGTATTTTCACCCGAAAAGGTAGCATGAGTTACGAGATCATGAGCAGTAGTGAAAGAGCCCAGAGTTAGAGGAAAGTATGCAGAGGTTTAGTAATCTAAGAAAGGGAGATAGTGTTNTGAAGAGCTATGTTATTATAGTCATGCACCCAGTAAGTTATTAATGAGGAGTTTTCCCTTATGGGTAGACTAAGAAGTGATGAGTTGTGAAGATTCCATCATAGGAGGTAGAGTGTG

>rnd-1_family-20#LTR/Gypsy ( RepeatScout Family Size = 1332 Final Multiple Alignment Size (possibly truncated) = 98 Localized to 2361 out of 4218 contigs )

TCCCAAGGGGGAGATGGTGTGCTTCGNTACCAAGGTCGATTATGTGTTCCNAATGTNGATGACTTGAGGGAGCAAATTCTNGCAGAAGCCCATAGTTCNCGGTATTCTATTCATCCGGGAGCCACCAAGATGTACCGTGATCTACGGGAAGTCTATTGGTGGAATGGNATGAAGAGGGATATNGCGGGNTTTGTGGCTAAGTGTCCNAATTGTCAACAAGTNAAGGTCGAGCATCAGAAACCGGGAGGTTTGTCCCAAGANATCGACATTCCTACTTGGAAGTGGGAAGTGATGAACATGGACTTCATCGCGGGTTTGCCTCGTACTCGNAGACAACATGACTCNATTTGGGTNATNGTNGATAGGATGACNAAGTCNGCTCACTTTNTNCCCGTCAAGGCTNCNNATTCGGCGGAGGACTACGCCAAGTTGTACATNAANGAGATAGTGAGGTTGCATGGGGTTCCTTTGTCTATCATCTCGGATNGAGGTACTCAATTTACCTCTCATTTTTGGAAGTCATTCCAAAAGGGTCTTGGTACTNAGGTTAANCTTAGCACGACNTTTCATCCNCAAACGGATGGTCAAGCGGAGCGTACCATTCAGACCTTAGAGGACATGTTGAGAGCTTGTGTGATCGATTTCAAGGGTAGTTGGGATGATCACCTNCCNCTNATNGAGTTCGCCTACAATAATAGCTACCACTCNAGCATTGANATGGCCCCNTTTGAGGCATTGTATGGTAGGAGATGTAGATCTCCGGTTGGTTGGTTTGAAGTAGGTGAAGTNGCCTTGATAGGNCCCGANTCGGTTCATGANGCTATGGAGAAAGTTCGACTCATTAGAGANAGGCTGAAGACGGCCCAAAGTCGNCAAAAGTCTTATGCCGATGTNAGAAGAAGGGANCTAGAGTTCGAAGTTGATGATTGGGTTTACTTGAAAGTCTCACCTATGAAGGGTGTGATGAGGTTTGGCAAGAAAGGGAAGCTTAGTCCCCGGTATGTAGGCCCNTACCAGATCTTGAAACGTGTTGGTAAAGTTGCTTATGAGTTAGANTTGCCAAATGAGTTGGCANCGGTGCATCCGGTNTTTCACGTTTCNNTGTTGAAGAAGTGTGTTGGTGATCCGGCATCCATAGTNCCNTTAGAGGGTGTGGGNGTNGANGANAGTCTTTCTTATGAGGAGGTNCCGGTTGAGATTCTNGACCGNCAAGTTAAGAGGTTGAGAAACAAGGAAGTCGCTTCCGTNAAGGTTTTGTGGAGGAATCANTTAGTTGAGGGTGCTACTTGGGAGGCCGAGGCCGATATGAAGTCCCGGTATCCTCATCTCTTTCCTTCCGATTCCACTCCAGCTTGAGGTAATAGTTCCTCTTNAGTTTTTCAGTCATTCATGCGTAAATTCAGTCTTAGTATCATGTTCCTTCAGTTTGTACTTGCATTTTCAGCGTATTTGCATGTTCTTGGAACTCAGTTCAGTCAGAAATCAGTTTCCAGTGTTTAGTGGTAGGGATTGCAGCTCTTTCCCTCCANTTCAGCTAGTTTAGTCTTCATTCGAGGACGAATGTTCCCAAG

>rnd-1_family-287#LTR/Gypsy ( RepeatScout Family Size = 303 Final Multiple Alignment Size (possibly truncated) = 98 Localized to 2361 out of 4218 contigs )

TATCCTGAGAATGCAACGTTCAACCAGAAGAAGTCGATACGCCGTATGGCCCTCAATTTCTTCGCGAGCGGGGAAATCCTTTATAGGAGGACTCCAGATTTAGGTCTTCTCAGATGTGTCGACGCTAGTGAAGCTGCGAAGCTTCTGGAACAGATACATGCCGGAGTNTGTGGTACTCACATGAATGGGCTCACTTTGGCGAGGAAGATCCTCCGAGCCGGNTATTTTTGGATGACTATGGAGCGCGATTGTTGCAAGTTTGTGCAGAAGTGTCATAAATGTCAAGTGCATGGNGATTTGATTCGAGTNCCGCCTCACGAACTCAATGCTATGAGTTCACCTTGGCCGTTTGTAGCTTGGGGCATGGATGTCATTGGTCCGATAGAGCCGGCCGCCTCTAACGGACACAGATTCATTTTGGTTGCCATCGACTACTTCACCAAGTGGGTGGAAGCAGCTTCGTATAAGTCGGTGACCAAGAAGGTNGTGGCTGATTTTGTTCGCAACAATTTGATATGTAGGTTTGGAGTACCAGAATCCATCATTACNGATAATGGAGCGAATCTCAACAGCCACTTGATGAGGGAAATNTGTGAACGATTCAAGATTACTCACCGAAATTCNACCGCTTATCGNCCCCAAATGAATGGAGCTGTNGAAGCCGCCAACAAGAACATCAAGAAGATTTTGAGGAAAATGGTCGACAATCGCAGACGTTGGCACGAGANGCTGCCGTATGCTTTGTTGGGTTACCGNACGACCGTCAGAACGTCAATTGGAGCNACTCCNTATTTGCTGGTNTATGGAACAGAAGCAGTNATACCTGCCGAAGTTGAAATACCTTCATTGAGAATCATCCAAGAAGCTGAATTGAGCGATGCTGAATGGGTTCGCAAGCGGATTGATCAGTTAACGTTGATCGATGAGAAGAGAATGGTTGCTGTTTGTCATGGNCAACTNTATCGACAGAGAATGATTCGTGCTTTCCACAAGAAAGTAAGAGCCAGGATATTTGAAATCGGNCAGTTGGTTCTCAAACGCATTTTCCCTCATCAGGATGAGTACAAAGGGAAATTTGCACCAAATTGGCAAGGACCCTACATGGTTCGCAAAGTGTTATCTGGAGGTGCCTTGGTCCTGTCGGAGATGGACGGCACCGAATGGCCGAAACCGATCAACTCAGATGCCGTCAAGAGATACTACGTGTGAAGNTTCAGTTTGTATTTCCGTCATTTCCTTGTAATCGTTCTATTTGCTTGTAATCGCTTTGTTTAGAATTTTATCCCTTTGTAATGAACTACGTCTGACCTGAATTCTCAAGAATGAGATACGTAGGCGGCCTATGTCGGCCTCGGTCACCCCTTTATCCCTTTTTAATATTTCCTTTGTATTTGAACTACGTTCGACCTGAATTCTCAAGAATGAGATACGTAGGCGGCCTATGTCGGCCTCGGTCGGTTTCTTTGTAAATTTCCTATTTTTGTTAATCCTCGAGAGGGGAACTACGTTTGACCTGATTCCTGCCTCAACGGGATACGTAGGCGCCACAACGGCTCGGTCATATCTCCCGTAAGATTTCTATTTTTCTTTACAATAGAAACTGGGACAGAATTTTTGAGAGGGACTCAAAAATTCTCAAGAAGAAGATCTCTCCAACAAAGTCGAGACTGAAGTTACTTTGAGATTTGCAACTGGGACAGAATTTTTGAGAGGATCTCAAAAATTCCGTGATTCGCTCACCAACGGTTCGAGATAAGCCAAGACAGTTAACTGGGACAGAAATTTTGAGGATGGCCTCAAAATTTCAACAGGGTTCATCGGCAGTTTAGAGCAACTTTGAATACTTCCCAACAAGTAATCAGATAGACCTCGAGACATCAACTCCAAATGACGTTCATTAAGCTTGACGTGACATGACACTTGGCAGTGACCTTTTCCAAATATTATTTTTGAAAATCTATTTTTCCTTAAAAACTTTTCCTTCATGTTTCAATTATTTTTGCATAAAAATGTTTTGTCTTATCTATCAAGAAGCGGGAGATCGGAGAAATCAACCGGAGATAGCAAGACAAGGAGCAGACGACCGAAGAGATCGACACAGATCAGTTCGTTTTTAAAACTAACAATTTTTCTGTGGATGCAGGTCTATAAGTTTGCCTCAAGATCAGCATATTCCTCCATTCAATGAATATGGAGAAGTCAAAAGGGCGAGGAGCTCCCCAAAATTGACAATTTTTCTGTGGATGCAGGAAATATTTTATGCTTCTTATANCAAAGANTTGGGAATATGAGTAACATTATTTCATTCAATTCCCAACAAGGCGAGGTCCATAACGAACATGCAATTATGTTCGGAGATGGAACAAACGAAAACCNCGAAGAGAGAAATNTTATTATTTTCCAGCAACTAAAGATACCTGGAGGACGTTTATCTGCATTATATTGTCGGATATGATAATATTACTACCCGGAGAGTCATTTATATCGTATTGTCGAATGAGACGATACCACTACTCCGAGGGTCATTTTTATCGTATTGTCGATTGAGACGATACCGCCACCCNGA

>rnd-1_family-425#DNA/Harbinger ( RepeatScout Family Size = 167 Final Multiple Alignment Size (possibly truncated) = 98 Localized to 2361 out of 4218 contigs )

ACTGAAACAAGGCATGAACCATTATGATATTGACACACAAGTGAAAATTGTCATAGCTTGTGCAGTGTTACATAATTATTTGCGAGAATACCAAAGGAGTGATGAAATATTTATGGTCTATGAGCGTGAAAATATAGTTGCGGATGATATTGACCAACAAACGGCTCAAAGTAACAATGTTGGTTCGTCTTCTCGGTCACATGATCGAGAAATGCAAGTTCAACGTGAGGAGATTGTCCGTACTATGTGGGAGGATTATATTAAAGACTAGTACACTACAATTTATGTTCAATTTTTTTTATTGAATTAAAGTTNGATGAAACAATTACTTAAGTGGAGAACAAATAGCTTTGTAATAATTTATTATGCGATTTTATAAATTTTTGTTTATTTGGTAAGATTGAATAAACAATTGGGGCTATTTTAATAGTTTTACAACTTATGAGATTTTTATGTTTATGAGAAAATATACAACACAAAAATTTCANATCGCATGTCCAAACAAAACTTCAATTTCATCTCATGATTCCATATCATGATATCATATCATGATTCCATA

>rnd-1_family-64#LTR/Gypsy ( RepeatScout Family Size = 978 Final Multiple Alignment Size (possibly truncated) = 98 Localized to 2361 out of 4218 contigs )

GGGGCTGGGGCGCCGCGCCAGCAGTGCGCCANAAACGAGCCTCTGTAGTTTAGGGGCTGGCGCCCCGCGCCACTCAGTGCGCCAGGGTCGCCTNCCCCCACCCTTTTTCCGTCGTTCGTTCCGTCTGAGTTCTTTCGAANCGTACCTNCGCTCCCTNCTGATTCTAACTACTCTAAACTACGTCTAAACATCGAGAGATCATTCATAACATGAATCATAACCCTTGAATTCATAATTCAAATTCAAGGTAGAGTTAAGAGTCAAGTCTCGAGAGTTCTTTCGAGTCATTTTGAGAAGTCTTTTACAATCTTTTAAAACTTGTTTTAAGACTTGAGCACTTGAGTTTGAGGANGAGNAGAGTTGAGTTTCATTTTTTAAAATGAATATATGGGAACTAAGTATTCCCAAGAGTAAATGTTTTTACATTTAAGATGAGAGGAAACACCGATTTCCAAANGAGTTCATGAGNAGTTTTGAGTACCATCTCTTTTAAGAGAANCTTTTGAGTAATAATCTCAAAGTCGAGGAAGAGGANATGTTTTTAAACATATGAGCTGAGTTATATTTTGGGAGTAGTATTGAGCACCGATATGGGGACGAGTTCAGACAACTCANAGTCCCCATAAACCATGTAGCCAACGTGGGTAGAAAGGGTCATACTTTTTAGATGATTCCTTATTGCTTTTTAAGCATAGACTAGTGGATCCACTTAGTTGAGGANGTTCTATACCCCGGCAAGGTATAGGACAGTTCTGGCAGCGTGGGCGAGACGTTGTATCATCACATAGCTCATAGTGATGGTTGTCGGTTAGAGAATCTCCCAAATAGAGTTATTTTGTATTTTTACATACAAACTGAGTTATTATTGTATTTTTNAATACATTGAGTTGTTATCTACTGTTTTAA

>rnd-1_family-168#LTR/Gypsy ( RepeatScout Family Size = 528 Final Multiple Alignment Size (possibly truncated) = 98 Localized to 2361 out of 4218 contigs )

TGTTAGTTCTTTAGTCTTTTCATTTTCATTATCTTATGTTGAGACTCGAGTTTGCCTTNNTGGCCAGTTGAATGTTCCTTTATAACATTCCAGTTTATTNCATNGANTTATNTTTGTTGAGTTAAGTCTTCCGCTGAGTTAAGTAAGCCAGGCCAAGGGTTCGCTTGGGGCCAGCAATGGTCCTCGAGTGCCGGTCCCGCCCAGGGTGTAGGCTCGGGGCGTGACA

>rnd-1_family-15#LTR/Gypsy ( RepeatScout Family Size = 1434 Final Multiple Alignment Size (possibly truncated) = 98 Localized to 2361 out of 4218 contigs )

TTTCTATCTCTTGATGTGTGGCTAAAACCCCAATTCTTGGGGTGTGATTTAGCGAATATGGGTTGANATTATTGTTGGGTCTTGCTTGCTGATGGTTAGATGTAGTTTAATGGTGATTTCGTCTAGTGGTTGTGGTTNAATTTAATGGGTTTGTAGTTGCAAATACAANTCCACCCATGTGTTTTCGGCTTGCTCGAGAGAGAGGTCGCGAAACCAAGACCACTAGATTGATGGCCTGNGGAGTGGGTCGACATGAGGTTCAGCTCGAGAGGGTGAGCCCTAGTCCCATATCCTACACACTCAGCTCGAGAGAGTGAGTGGGNTAAGGCGTAGGCTGGTCTTCATGCGGCAAATGGGTGTCCGAGAGGAACCCATTTGAAACGGGGTAAGTTGCTCGAGAGAGAACTTATCCCCACTAAAGCCTAGCCTAGTCACTATTATCTCTACGAATTTCCTATCGAAAGCATGTACCCAACGATTTANCTTAACCCGTATTCCGGTCACATCCCAAGAATCCCCTCTCCATACTTGATTTTCTTGTTAATTTTTGCTGTTTTTGCTTACTTGTGACAAAACCCCCNATTGATANTTGACACTTTCGTGTCACCCTTTAATTTACNATGTTTTTANTCGTTAATGTCTTTAGCTACGACTAGTTAGAACTAAATTTTATTTTTCTATTAATTCTCAAAACCGCTCCCTTGGGACTCGACCCCAACCCTTGGTTGGGTTACTATACTATCGTACGATCGTAGACACTCGNATCGGAAGTTGTGTCGTTGATTACGCNAACATCAAAATGGCGCCGCTGCCGGGGAGTGGTGTTATTTGAGAATTTTTAGTTAAGTAGAATTTTGTTCTTCTTAGTCGTAGTTTACTAACTTAATTTTTAGTTTTGTTTTGCTTGTTTGTGTATCGAAACAGAGGAAATGAGCGTGAACGGTNACTACGGGNACCAAACGGATCACCCCCNTTNGAAGTNATNGAATCTCAGGGATAACATCCTAACNTTCAAGCAANTGGAGGGTGAGACGATCCACGAGNCGTGGCCGAGGTTTAAAGCACTGNTGCNGCAATGCCCAACTCATGGAATNCCCGACAAGNCGCTNCTGGANTGCTTCTATAGGGGTCTTGGTCCCGAAAATAGAAGCGTGGCCGACCAACTCNTTCCGGGCGGTATGNTACAACAGCCCTATGCGATAGCAGCCCAACTCCTTGATCGCATGGCTGAGACCAACAAGGAGGCAGAAAAGGACCACGAGTTGGCCGCACTGNTGACTCAGNTGGACGNCTTGGCCAAAAAGGTCGTGGAGTTGGAAGCGCAGTNCAAGAAGAAGGATAAGTACGTCCCCCCTCGCGAGCGTAAAAGNCGAAGGANCATGAGGGTGGACGNATCGAGGANACGCTCTCACTCATTCTCCACAAGGTCGANGAGCAAGATAGAGTGTTGGANGAGATGAAGGAGAACGTCGAAATGCTGAATCAGACGACNACCTCTCATTCCATGNCTATCCAGCTGCTGGAGACCCAAATGGGTCATGTGTTGTCTCGTCTCNACCCGNAACGAAAAGAGGGGTCGCCTAGTGACACTATGGCNAACCCCAAGAATGAAGCTTGAGTGNNGACTTGCGTCGTGCCACGACGTTAACTAAGGCGCTNCTTGGGAGGCAACCCAAGACCCTTTTATCGCTTTATTTTTGTTTTAGAAATAATGGTGTGTTGATTGTGCAGGTCGAAGTGTGGAAAGTGTTTGAAAAGTGCAGGTTGGCGAGCAAAAGGTCCAGTCGGCGAATCGCCGAAGAGGTCGGCGAGCCCGACCTGGACCGCCGTTGGACTCAAGACAACTTTAAATTGGAGTCTGTAAAACTCGGCGAGCCCAGGAANTNATCGGCGAATCGCCGACCAGGTCGGCGATCNCGATNTAGNCCGCCGTTTGGACCCCCANATTAACTGGAGGTCCTGTAAAACTCGGCGAGGTAAGTGACCACTCGGCGCGTCGCCGAGTGGNTCGGCGAGCGCGACTAACGTCGCCGAATGGGCGAGAACTGGACGGTTTTTAAAGGCCAAAGGTTTAAAATTTCAAACTCTTTCACTTTCCCTCGCTTTCGAACTTCNCAAACTCACACCCGCNTAGTATTTTCNATATTTTTGCATTTTTANCGTATTTTAGTCGTCGATTTGTGCTCGGAGTTGGATTACNTTGCCGCCTAGCNTTTCAAACTTTTTAATCGGCATCAAAGGTTGGTTTTCCGAACCCCGAATTTATNTTTAGNAATTTGTACTCATTTGCAATTGATNTTATCTTANTGACGTGTGCTGGGCCTCTCTGATAGGATTATAATGTTTGCGTGCCTGTTTTAATTTGAAAATCTTGCCCGTAATGCCTATANTGTTGAATTCCCGTAATTTTGTGCCTTAAAATTGATTAAATNTTGTGGGGTTGTGCTTGCGTGTTGTTGGGTCTAGTCGGGTGAAGTCTGAGTAACCCGTATTTGTGCCAAATGACGTAATTTGCCCAATGATGGAGCGGNTGACGTCATTCTTAAGGGCAAAAGTCGTCAAATGGCCAAATTTGGCAAAACCGGGAGAAGTCTGAGTATCCCGGGGCGATGGGTGTTATGGGTCTTGNTTTAATTTGATTGTTTTCGGGGGTTGCGGGGTTGAATTCGGGAAGTTGGGTTGAAAGTAGGCACGTTGGGTCGTTTGGCGAGCTGGGTCGAGCTCGCCGAACCGCTCGGCGGTTCGCCGAANGTCCCCATCTCGCCTTTAATTTCATGCTNNATGTGNTGTTTGGTTCTGTAACTTTCGGCGAGAAGCCTGAGGTCGCCGAANGCACTCGGCGACTCGCCGAAAGTCTTCTTGATCGCCCTTTGTCTGCGCCC

>rnd-1_family-85#LTR/Gypsy ( RepeatScout Family Size = 828 Final Multiple Alignment Size (possibly truncated) = 98 Localized to 2361 out of 4218 contigs )

AATTCAAGGGAAGTCAAGAGTCAAGTCAAGAGAAGTTCTTAGAGTTTTCCAAAAGTCTTTTACAAACGTTTTAACTTTGTTTTAAGACTTAAGTTTCGAGTTGAGTAAAGAGTAAAGATTGAAGTTCATTTCTTCAAAAGAGTATATGGGGACTATGTATTCCCAAAGAGTTTAAAATGTTTTCACATTTAAACAAGAAAGGAAACTTCGATTTCCAAAAGAGCCTTCGGGCTAGTTTTCAGAAAAGAGTAAATGCTTTCACAATTAAGCAAGAGAGGAAACNTCGATTTCCAAGANAGCCTTTGAGCTAAGTTTTGAGCAATTATCTCAAACCACAGAAAGAAGTTATGTTTTTAAACATAAGAGCTAGTATCATATTTTGGGAGTAGTATTGAGCACCGATATGGGGGAGAGTTCAGACAACTCACAGCCCCCATAAACCATGTAGCCATCATGGGTAGAAAAGGGTCATACTTTTTAGATGATTCCTTAGTGCTTTTTAGCATAGACTAGTGGATCCACTTAGTAGTCAGGTTCTATACCCTCGGCAAGGTATAGGACGGCCCTGGCAGCGTGAGGCAAAACGTTGTATCATCACNATAGCTCTTAAGTGATGGTTGTCGGTTAGAGAAACTCCCACAGAAGTATTTTGTATTCTTATATACAT

>rnd-1_family-232#Unknown ( RepeatScout Family Size = 404 Final Multiple Alignment Size (possibly truncated) = 98 Localized to 2361 out of 4218 contigs )

ACTGCCTCAAGCTCCACGAGCGGGACCACGGCCCGTGGTCNTGACCACGGCTCGTGGAGGNGCTCGTGAAGATGCCTTGGGCNTGTGAGCCTAAGGCCACCAAGTGCCTAGCTACTGCCCAAGGACCACGAGCGGGACCACGGCTCGTGGTCTTGACCACGGCTCGTGGGGGGGCTCGTG

>rnd-1_family-528#DNA/hAT-Ac ( RepeatScout Family Size = 119 Final Multiple Alignment Size (possibly truncated) = 98 Localized to 2361 out of 4218 contigs )

TTTGGTTTGGTTTGGTTTTAGATTTTTGAAAACCGATAGTATTTGGTTTGGTTNTGGTTTTATTCGAAAAAAACCGAAGAAATAACCGAACCGAACCGATAAATTATATACATANATTTTATAATTATTTATATATTATTCATAAATAAAATATAAATATTTTGTAAATATTTTNATTAACTTAAGTCTTTAACTTTACCATTTTCTCAAGCCCAACACTTAAATTTTAGCCCAACTATTACAATCCTAAGTCCAAGTCCATCAAAATCCATTACTTTAGTCTTTACCTACCCTACCTCACATAAGATAGTCACACTTTCTCTTAATTGAATCACTTTGTTCGTGTAATAATAACTCTAGTATTGCTTAATTGCTTAATTGAACCGACAATAGCTTTCCTGTGGATTGTTCATGTACTAGGTGTTTGTGTCTATCGAGATACGTATGTCCTCAAAAAATTTATTACTTTCAAACCGAAAAAACCAGAAAAACCGAACCAAACCGA

>rnd-1_family-525#DNA/hAT-Tip100 ( RepeatScout Family Size = 120 Final Multiple Alignment Size (possibly truncated) = 98 Localized to 2361 out of 4218 contigs )

TGGCATAATACATAAATNTGACCCTTAACTTGGCTTCAANTNACAACTATGNCCTCCAACTTTGANTGTGCACAAGTAGACACTTAAACTTGTATAAAATTGAACAAATAGACACGCGCGTCCTACATGGCATAATACGCGTAGGACGCCACGTAT

>rnd-1_family-496#LTR/Copia ( RepeatScout Family Size = 130 Final Multiple Alignment Size (possibly truncated) = 98 Localized to 2361 out of 4218 contigs )

AACTGTGCTGAGGACCTGGTCCCATCGGACTAAGTGGACGCATACATTCCAACAAGTGGTATCAGAGCTTGACTTTTCAGTTTGGTTAACACCAAGAAAAGAAAAGATCCTGAAGGAATGGCTGCTCCACCNAATNTGGAGGAAGGNCAGTCNTCTACCAGACCACCTCGTTTCAATGGNCAATACTATGGATGGTGGAAGACNCGAATGCACGACTACATCAACGCTGAGGACACTGAGCTGTGGGACGTGATTCTTGATGGACCNTACATTCCCACNAAGGANGTGAAGGATGGAGAGCTCACCACGACGGTTGTCAAAACCAGAAAAGAGTACGATGAGGCGGACAGGAAGAAGATNGAGAAGAACTACAAAGCNAAGAAGNTNCTAGTGTGTGGNATCGGNGCNGATGAGTACAACCGAATCTCTGCNTGTGAGACNGCCAAAGAAATNTGGGACTGTCTNCAAACAGCCCACGAAGGAACNACGCAAGTGAAGGAGTCTAAGGTNGATATGCTTACNACTCAGTACGAGAACTTCANCATGAAGGAAGGTGAAACCATTCACGAGATGAACACAAGATTCACCTCCATNACCAACGAGCTGAGATGCCTTGGTGAGCCTATTACCTCGAGCAAACAAGTTCGAAAGATTCTCGAAGTNCTTCCCAAGTCNTGGGAAAGCAAAGTGAATGCCATNACNGAAGCAAAGGATCTGAAGACGCTTNCCATGGATGAACTGATTGGAAACCTTCAGACCTACGAGNTGAACAAGAAACAGGGGACAANCGTGAAGGAGGGAAAGAAGGAGAAGTCCGTNGCNCTGAAAACGTCTCAAAACGATGCGACTGAGGAGGAGGATGAGATGGCNTATGTCACTAGAAGGTTTCAGAAGATCGTNAAGAAGCATGGAGGCTTTCGAAAGAAAGCCTCTACCAGCAGAGCTGCAAATGCAAATGACCTNTGTCACAAGTGTGGNAAGCCTGGTCACTTCATGAGGGACTGCCCCAGCCAGAAGCAGGAAACTCANGACTTCAGACCTCGCAGAAGGGACCTGGTCCCAGACCATGCCAAGAGGAAGGCTCATGCTGATCANNTGGTGAAGAAAGCTTTTGCTGTNTGGGGAAACGCNTCAAGTGAGTCAGAAGAAGANGCAGAANGTCNCGAGGATGTCTCGATGATGGCCGTTGAAGATGATGAAACTGTTTTCGACTCCATTTTCTCNCTNATGGCAAAATCTGACGATGAAGAGGATCNAGACGAGGTAACTCTTTTTNATCTCAAANATGATCTAGATACTCTTCCCGTTAAAAGATTGAGAAAACTTGTTGCCGTACTNATTGACTCCGTTGATGAACTAACCACTGAAAACTTGACGATAAATGAAAAATTGAGTTTGTGCGAGGATGAGAACTCAGCTCTCATNTCTCAAGTNTCTGAAATGAGTGTNAGGATAGGTATTCTAGAGACTGANAGTNTAGAACCCGAGGAGGANCCTGGTACCTCNGAGGGTGGAAAGAGAAAGCTCAGCANCTTTGAGGTNGAGCTAGAAGANAAGCTNAANACCGCTGAGTCTAAGCTAGTTGCCTCCCTNGAAAGAAACTCTCAGCTGGAGAGGGACCTGGTNNGGTCAAAGAAGAGCTGAACCACTCCCTNAAATGGACCGACTCCTCTAAGATACTCTCTAACTTAGCCAATCAGAAGTTCAACGGCAGGAAAGGGTTAGGTTGTAGACAGATAGANCCTCCCTACAATCCTCACAGCAAATATGTGTCTGTNTCTGACAATCTGTTGTGTACTCACTGTGGNCGNAATGGTCATCTGAAAGAGGANTGTGAGACTTTGAAAAGAGCAAAAGAAAGGCATGCGAANTTTCTCAGATCGAGAAAAGACGNTGANAAAAAGGAAAGGGTACCTGGTCCCGGTCACCGTTTNAGCAAGAACANTCTGCCCTCATGGACAAGAAGNTTTCTTATTANGCCTTTTGACAGCTTACTGGGAACTCCGTCTGAAGTGGGTTCCCAAGTCTAACAAGTGATTCTTGTGCAGAAGAGAGGAAGCAGTCAGTGCTGGTNCATGGACAGTGGATGCTCAAGGCACATGACTGGAAACACTNNAAACTTCCTCTCTCTCGAGGCACNCCAAGGTGGNGGTGTCTCNTTTGGCGGTGGAAAGAAGGGTTNTATTCTTGGNATTGGCAGGATNGGAAGATCNGTNGANCACTCTATNGANAATGTNCACTATGTGAATGGTTTGAAATACAATCTCTTAAGTGTTTCNCAAATNTGCGACAAGGGAAACGAAGTCAAGTTCNTGTCNGATAAATGCGTGGTCACAAACTGTGCGACTAANGGAGTTGTNATGTCTAAGTAAAGAATATGTACGTTGCNGATCTTGACTCTATTGAAGGGGACGATCTNTCATGCCTNAGTGCTCAAAGTGACGATGCNAATCTATGGCATAGGCGACTNGGTCATGTGAGTNCTTCTTTACTGAACAAACTTGTCGCAGGGGACCTGGTCCGCGGATTGCCAAAACTGAAGTTTTCAGACGACAAAGTTTGTGATGCNTGTGCNAAAGGGAAACAA

>rnd-1_family-305#Unknown ( RepeatScout Family Size = 273 Final Multiple Alignment Size (possibly truncated) = 98 Localized to 2361 out of 4218 contigs )

TGTCACGACCCAAAAATGGACGTGATGGCACTCGTCTTATCCCACCAAGACAAGTCAGCCTAAAACTCAACCATTACAATAAAATGCGGAAAACTTATAATCAACTTAAATATACCCCAAAACCTGGTTGTCACGTGTACAAGCCTCTAATGTATTACAATNGATTCGAAAGAAAAATACAAGTCTCAAATGACNTTGTTTCTAGAATAGAACAAGATCATAAATAGGAGTAAGAAGGTCCGCCGAGATGACAAGCAACTACCTCACAAATCTCCACAAGAAGCCTCGGANAAGGAGAAGAGAAAGTATCACGAAGATCCGGGCTCGTAACCTACANAAATGTAGAAGCAAGGGGTGAGTACCAAACCACACGGTACTCAGCAAGTAAACCTCTAAACACAAGCTAAGGGGATAGAATACGGGTACTCCTTACACCCCAACCGAACCTCCACAACTACAACCTGCATAAAACCAGCCCAACCTAACAGTTCACAATTTACATAGCACACAGCTCAACAACAACACTCTAACAATCACATATCCTCAACAACAAGCTCAATATTCATCAATCACAACTTCCACAA

>rnd-1_family-119#LTR/Gypsy ( RepeatScout Family Size = 664 Final Multiple Alignment Size (possibly truncated) = 98 Localized to 2361 out of 4218 contigs )

TATGACTTGTATGTTGGTTGTGACTTGTATTATGCCTCTTATACTNATGTTCATGAAGTTTTACNAAAATGGCATAAAGCATGGCTTCCAACAAAATGTCCCTTTTAAAGCATGTTTTTGCATGGTCATCATACTTAGTACATTTNTGTGTGCTAACCCATATTTCTCTTATTTTTACTACAAGTGTAGGTTCCGGCAAGTGATTGNTTCTNCTAGTTGAAGTGCTTGGATTGANTTTCTTCCAAGACTTGGTATGTCCTCATAGATTCGAGGACAAGACTTTTATGTTTCGTTTCTTCATGTAATAGACATTTAGTAGTTTCTTTTCGATTGTAAAGGGCCGTGTCCCTATTTTGTTNNAGTCTTGTCTAAGATGGCCACGTGAGACTTAGTCTTCCGCTGCGTNTTAAAGGTTTAAATGANGGTGACTTTTGAAGTCTTATGGTTTTAAATAAAAGTTTTAATTCCGTACTATTTCTATGATCTATGCGATGAACGAATGCTAAGAGGCTTGTATAAGACCTCCGAGAGGTCGAGTACGCCGTGTAACGACTAGGGGGTGCTCTCGGGTCGTTACA

>rnd-1_family-62#LTR/Gypsy ( RepeatScout Family Size = 983 Final Multiple Alignment Size (possibly truncated) = 98 Localized to 2361 out of 4218 contigs )

TCATGATTATAGTGTAATGTAAAGTGAAAGGTTTACTCACTTGCTAAGTGTTTCTAAGGTGAAAGGATCGTTACTCACTTATGAACACATATGAGCTATTATGGTAAGATGTCTACATAAGAGTCTAGTAAAGGCTAATGTGAACTTATGTGATGACTAAAGATGATTACAAAAGGGAATTAGATGCTTAGCACCGAGNGGGCATGNAAATGAGATGGGGGTCTCACGTTTAGTAAGTCCGGCTTCCCACATGGGTTCCTTCACGTTTAGCAAGTCCGGGTTCCCAANGTATGTGTTGTCTCATGAGATGGAAACCTCCACGTTTAGTAAGTCCGGGTTTCTAGNAGCAATCTCCTTATCCCATAACTATGTGCCCACATAGGTCTTTAGCTAGTGGATCCACCTAGATAGCTATTACGTATGGTTCTACCTTAGGCAAGTAGGACACTCTCTTTTCGGTGTGGGGGTAGAACACCGGATTCCATGTAGCTCACATGGTCTATGTCGGTTANGGCNATATTTCCCTAATGTAAAAGTAAATGAACTATGATAAGAACGTGAACTNTAACTATGACTTTCTTAAGGGGTTCTACTTAGTGTGAGTGGGGGTATGGGACTTCACTTACGCATTGCACAAGTGGACTCTAAGAGGGGTTATGGTAGTGTTCTCTTATGNTATGANGACTTATGANTTATGTATGTTGAAGC
[truncated: 2,779,957 more chars]
