# Supplementary material for: A Deluge of Complex Repeats: The Solanum Genome
Source: PLoS One. 2015 Aug 4;10(8):e0133962. doi: 10.1371/journal.pone.0133962 (PMC4524691; doi:10.1371/journal.pone.0133962)
Supplement: S4 Table — Coverage of different repeat super-families was calculated as the percentage of nucleotides represented by repetitive elements out of total nucleotides for the given chromosome. (DOC) [file pone.0133962.s010.doc]

**Supplementary Table 4: Chromosome wise density of Repeat Families**

| ***Solanum tuberosum*** | | | | | | | | | | | | | |
| --- | --- | --- | --- | --- | --- | --- | --- | --- | --- | --- | --- | --- | --- |
| **Repeat Family** | **chr 0** | **chr 1** | **chr 2** | **chr 3** | **chr 4** | **chr 5** | **chr 6** | **chr 7** | **chr 8** | **chr 9** | **chr 10** | **chr 11** | **chr 12** |
| **DNA/CMC-EnSpm** | 0.28 | 0.31 | 0.27 | 0.36 | 0.36 | 0.38 | 0.37 | 0.34 | 0.41 | 0.40 | 0.44 | 0.36 | 0.40 |
| **DNA/Harbinger** | 0.13 | 0.43 | 0.54 | 0.51 | 0.40 | 0.36 | 0.52 | 0.40 | 0.40 | 0.37 | 0.35 | 0.44 | 0.29 |
| **DNA/hAT-Ac** | 0.14 | 0.29 | 0.32 | 0.35 | 0.33 | 0.32 | 0.40 | 0.33 | 0.40 | 0.34 | 0.31 | 0.30 | 0.37 |
| **DNA/hAT-Tag1** | 0.01 | 0.04 | 0.05 | 0.04 | 0.04 | 0.03 | 0.04 | 0.04 | 0.04 | 0.04 | 0.02 | 0.06 | 0.05 |
| **DNA/hAT-Tip100** | 0.15 | 0.30 | 0.31 | 0.37 | 0.31 | 0.32 | 0.34 | 0.28 | 0.30 | 0.27 | 0.31 | 0.38 | 0.35 |
| **DNA/MULE-MuDR** | 0.11 | 0.22 | 0.22 | 0.25 | 0.18 | 0.19 | 0.22 | 0.21 | 0.22 | 0.16 | 0.18 | 0.21 | 0.16 |
| **DNA/PIF-Harbinger** | 0.05 | 0.20 | 0.25 | 0.26 | 0.20 | 0.19 | 0.22 | 0.19 | 0.21 | 0.19 | 0.17 | 0.24 | 0.16 |
| **DNA/TcMar-Pogo** | 0.01 | 0.08 | 0.09 | 0.06 | 0.06 | 0.05 | 0.09 | 0.05 | 0.07 | 0.06 | 0.08 | 0.08 | 0.06 |
| **DNA/TcMar-Stowaway** | 0.17 | 0.55 | 0.71 | 0.66 | 0.53 | 0.56 | 0.64 | 0.56 | 0.59 | 0.54 | 0.50 | 0.64 | 0.51 |
| **DNA** | 0.27 | 0.66 | 0.88 | 0.76 | 0.62 | 0.61 | 0.77 | 0.66 | 0.71 | 0.65 | 0.58 | 0.64 | 0.55 |
| **LINE/L1** | 1.35 | 1.23 | 1.38 | 1.34 | 1.30 | 1.20 | 1.43 | 1.38 | 1.44 | 1.35 | 1.17 | 1.76 | 1.21 |
| **LINE/RTE-BovB** | 0.11 | 0.47 | 0.49 | 0.53 | 0.57 | 0.63 | 0.49 | 0.61 | 0.52 | 0.57 | 0.52 | 0.78 | 0.57 |
| **LTR/Caulimovirus** | 0.15 | 0.11 | 0.10 | 0.10 | 0.09 | 0.12 | 0.13 | 0.11 | 0.12 | 0.09 | 0.11 | 0.10 | 0.10 |
| **LTR/Copia** | 2.07 | 3.69 | 3.39 | 3.30 | 4.16 | 3.53 | 3.70 | 3.74 | 3.89 | 3.97 | 4.00 | 3.41 | 3.84 |
| **LTR/Gypsy** | 22.29 | 27.16 | 18.58 | 23.63 | 28.55 | 30.27 | 25.94 | 27.89 | 27.01 | 30.09 | 32.73 | 24.53 | 32.47 |
| **RC/Helitron** | 0.16 | 0.07 | 0.05 | 0.06 | 0.07 | 0.10 | 0.13 | 0.08 | 0.07 | 0.09 | 0.03 | 0.26 | 0.07 |
| **Retroposon** | 0.19 | 0.39 | 0.28 | 0.26 | 0.38 | 0.32 | 0.35 | 0.26 | 0.27 | 0.34 | 0.34 | 0.34 | 0.40 |
| **Satellite** | 0.93 | 0.10 | 0.66 | 0.01 | 0.05 | 0.04 | 0.05 | 0.44 | 0.39 | 0.04 | 0.21 | 0.04 | 0.61 |
| **SINE/tRNA** | 0.02 | 0.12 | 0.13 | 0.15 | 0.13 | 0.15 | 0.14 | 0.15 | 0.13 | 0.13 | 0.13 | 0.19 | 0.14 |
| **SINE** | 0.01 | 0.06 | 0.05 | 0.07 | 0.07 | 0.08 | 0.06 | 0.07 | 0.07 | 0.07 | 0.06 | 0.07 | 0.06 |
| ***Solanum lycopersicum*** | | | | | | | | | | | | | |
| **Repeat Family** | **chr 0** | **chr 1** | **chr 2** | **chr 3** | **chr 4** | **chr 5** | **chr 6** | **chr 7** | **chr 8** | **chr 9** | **chr 10** | **chr 11** | **chr 12** |
| **DNA/CMC-EnSpm** | 0.52 | 0.72 | 0.70 | 0.68 | 0.61 | 0.71 | 0.68 | 0.69 | 0.80 | 0.66 | 0.72 | 0.65 | 0.70 |
| **DNA/Harbinger** | 0.28 | 0.47 | 0.58 | 0.49 | 0.35 | 0.37 | 0.51 | 0.37 | 0.40 | 0.35 | 0.32 | 0.40 | 0.37 |
| **DNA/hAT-Ac** | 0.34 | 0.55 | 0.85 | 0.72 | 0.60 | 0.69 | 0.79 | 0.60 | 0.58 | 0.57 | 0.65 | 0.91 | 0.55 |
| **DNA/hAT-Tag1** | 0.05 | 0.09 | 0.15 | 0.11 | 0.13 | 0.06 | 0.12 | 0.10 | 0.08 | 0.09 | 0.06 | 0.14 | 0.13 |
| **DNA/hAT-Tip100** | 0.15 | 0.29 | 0.33 | 0.29 | 0.29 | 0.27 | 0.33 | 0.23 | 0.25 | 0.23 | 0.30 | 0.27 | 0.29 |
| **DNA/MULE-MuDR** | 0.45 | 0.73 | 0.93 | 0.72 | 0.88 | 0.68 | 0.96 | 0.69 | 0.77 | 0.75 | 0.77 | 0.87 | 0.85 |
| **DNA/PIF-Harbinger** | 0.05 | 0.12 | 0.14 | 0.13 | 0.12 | 0.10 | 0.17 | 0.12 | 0.12 | 0.09 | 0.09 | 0.12 | 0.09 |
| **DNA/TcMar-Pogo** | 0.07 | 0.13 | 0.18 | 0.15 | 0.12 | 0.16 | 0.16 | 0.13 | 0.18 | 0.12 | 0.11 | 0.13 | 0.10 |
| **DNA/TcMar-Stowaway** | 0.20 | 0.66 | 0.73 | 0.57 | 0.51 | 0.38 | 0.67 | 0.45 | 0.47 | 0.40 | 0.42 | 0.46 | 0.42 |
| **DNA** | 0.50 | 1.14 | 1.41 | 1.20 | 1.04 | 0.96 | 1.37 | 0.95 | 1.05 | 1.06 | 0.98 | 1.06 | 0.94 |
| **LINE/L1** | 2.01 | 0.86 | 0.91 | 1.35 | 1.12 | 0.79 | 0.98 | 0.86 | 1.08 | 1.91 | 1.13 | 1.29 | 0.79 |
| **LINE/RTE-BovB** | 0.09 | 0.40 | 0.41 | 0.40 | 0.49 | 0.43 | 0.49 | 0.43 | 0.37 | 0.43 | 0.39 | 0.52 | 0.45 |
| **LTR/Caulimovirus** | 0.20 | 0.07 | 0.08 | 0.08 | 0.07 | 0.11 | 0.17 | 0.09 | 0.13 | 0.10 | 0.07 | 0.17 | 0.08 |
| **LTR/Copia** | 4.93 | 7.07 | 4.70 | 5.22 | 6.90 | 6.10 | 5.54 | 6.24 | 5.40 | 6.07 | 7.22 | 5.94 | 6.46 |
| **LTR/ERV1** | 0.00 | 0.01 | 0.00 | 0.01 | 0.00 | 0.00 | 0.01 | 0.00 | 0.00 | 0.00 | 0.00 | 0.01 | 0.00 |
| **LTR/Gypsy** | 50.16 | 35.59 | 29.75 | 36.91 | 36.79 | 42.71 | 32.83 | 43.40 | 43.53 | 43.12 | 40.57 | 38.55 | 44.21 |
| **RC/Helitron** | 0.08 | 0.10 | 0.10 | 0.07 | 0.12 | 0.09 | 0.09 | 0.11 | 0.09 | 0.07 | 0.08 | 0.09 | 0.06 |
| **Retroposon** | 0.30 | 0.68 | 0.24 | 0.24 | 0.57 | 0.35 | 0.30 | 0.27 | 0.30 | 0.37 | 0.41 | 0.38 | 0.41 |
| **Satellite** | 0.04 | 0.05 | 0.02 | 0.17 | 0.02 | 0.02 | 0.01 | 0.08 | 0.06 | 0.14 | 0.02 | 0.03 | 0.03 |
| **SINE/tRNA** | 0.02 | 0.14 | 0.12 | 0.12 | 0.13 | 0.12 | 0.16 | 0.12 | 0.11 | 0.11 | 0.11 | 0.15 | 0.11 |
| **SINE** | 0.03 | 0.06 | 0.07 | 0.07 | 0.07 | 0.06 | 0.07 | 0.06 | 0.06 | 0.07 | 0.06 | 0.05 | 0.07 |
